# Supplementary material for: Aromatic ring-opening metathesis
Source: Nature. 2024 Dec 11;638(8051):697–703. doi: 10.1038/s41586-024-08472-z (PMC11839454; doi:10.1038/s41586-024-08472-z)
Supplement: Supplementary file 1 — Supplementary Figs. 1–18, Tables 1–16, methods, computational studies, NMR spectra, high-performance liquid chromatography data, X-ray data, electron diffraction data and references. [file 41586_2024_8472_MOESM1_ESM.pdf]

nature portfolio

https://doi.org/10.1038/s41586-024-08472-z

Supplementary information

Aromatic ring-opening metathesis

In the format provided by the
authors and unedited

Supplementary Information

Aromatic Ring-Opening Metathesis

Valeriia Hutskalova, Christof Sparr*

Department of Chemistry, University of Basel

Email: christof.sparr@unibas.ch

Supplementary Methods

Table of Contents

General Information 1

Preparation of Substrates 2

Aromatic Ring-Opening Metathesis 57

Aromatic Ring Opening (ArROM) – Ring-Closing Metathesis (RCM) Cascade: Tetraphene 58
ArROM with Twofold RCM: Napthalene 59
ArROM with Twofold RCM: Benzofused Five-Membered Heterocycles 60
Bidirectional Twofold ArROM – RCM to Polycyclic Aromatic Hydrocarbons (PAHs) 62
Equilibration of Indoles by ArROM – RCM 65
ArROM – Twofold RCM for *N*-Aryl Indoles 77
Atroposelective Aromatic Ring-Opening Metathesis – RCM Cascade 84
Atroposelective Aromatic Ring-Opening Metathesis – Twofold RCM 95
Atroposelective Indole Ring-Opening Metathesis 103

Determination of the Rotational Barriers 108

Computational Studies 111

Gibbs Free Energy Differences of the Selected Pairs of Starting Materials and Products 111
Comparison of Metathesis Catalysts 113
Visualization of Noncovalent interactions (NCI) in DFT Computed Metallacycles 115

Cartesian Coordinates for All Optimized Geometries 117

NMR Spectra 170

HPLC Data 322

X-Ray Data (Dr. Alessandro Prescimone) 348

Microcrystal Electron Diffraction (Dr. Alessandro Prescimone, Eldico) 349

Supplementary References 350

General Information

All reaction solvents and reagents were obtained from commercial suppliers and used without further purification unless stated otherwise. Solvents for extractions and chromatography were technical grade. Syringes were used to transfer air and moisture sensitive liquids and solutions. Analytical thin layer chromatography (Merck silica gel 60 F254 plates) was utilized for monitoring reactions and visualized by UV light (254 nm and 350 nm). Flash Chromatography was performed with SiliCycle silica gel 60 (230-400 Mesh) or otherwise stated stationary columns. Concentration *in vacuo* was performed by rotary evaporation to ~10 mbar at 40°C and drying at ~ 10^{-2} mbar at room temperature.

All metathesis reactions were performed using degassed and dried solvents in an argon-filled glovebox. Commercially available dry solvents were charged with molecular sieves (3Å) (predried at 180°C (~ 10^{-2} mbar) for 24 h before use) and were degassed using the freeze–pump–thaw method. Solvent flasks were then transferred to an argon-filled glovebox and used within seven days of storage over molecular sieves.[1]

^{1}H-NMR spectra were recorded on Bruker DPX 400 MHz or Bruker DRX 500 MHz spectrometers at 298 K in the indicated deuterated solvent supplied by *Cambridge Isotope Laboratories*. Chemical shifts (δ) are quoted in parts per million (ppm) and referenced to the residual solvent peak (δ = 7.26 ppm for $CDCl_3$ and 2.50 ppm for $(CD_3)_2SO$). The multiplicities are reported in Hz as: s = singlet, br = broad singlet, d = doublet, t = triplet, q = quartet, m = multiplet, dm = doublet of multiplets, and ddm = doublet of doublet of multiplets. ^{13}C- and 2D-NMR spectra were recorded with ^{1}H-decoupling on Bruker DRX 500 MHz spectrometers at 298 K in the indicated deuterated solvent supplied by *Cambridge Isotope Laboratories*. Chemical shifts (δ) are quoted in parts per million (ppm) and referenced to the residual solvent peak (δ = 77.16 ppm for $CDCl_3$ and 39.52 ppm for $(CD_3)_2SO$).

Melting points were measured on a Büchi B-565 melting point apparatus and are uncorrected. IR spectroscopy was measured on an ATR Varian Scimitar 800 FT-IR spectrometer and reported in cm^{-1}. The intensities of the bands are reported as: w = weak, m = medium, s = strong. High-resolution mass spectrometry (HR-ESI) was recorded by Dr. Michael Pfeffer at the University of Basel on a *Bruker MaXis 4G QTOF* ESI mass spectrometer. Optical rotations were measured at 296 K on a Jasco P-2000 digital polarimeter with a path length of 10.0 cm, using the 589.3 nm sodium D-line and concentrations are reported in g/100 mL. UV/Vis spectra were measured in acetonitrile solution on a Jasco V-770 spectrometer with a 10 mm sample cell at 20°C.

Preparation of Substrates

4-(2-Vinylphenyl)tetraphene (1):

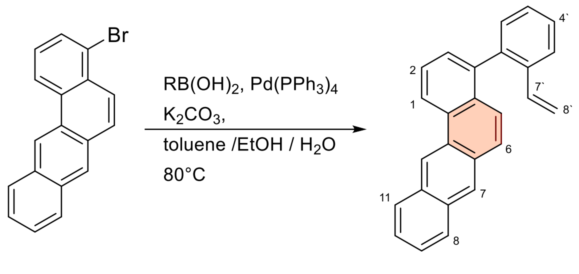

1

4-Bromotetraphene (92.2 mg, 300 µmol, 1.00 eq.), (2-vinylphenyl)boronic acid (49.3 mg, 333 µmol, 1.11 eq.), $Pd(PPh_3)_4$ (17.3 mg, 15.0 µmol, 5 mol%), and K_2CO_3 (124 mg, 900 µmol, 3.00 eq.) were weighed into a 10 mL crimp cap vial. The vial was sealed, evacuated and backfilled with argon before a degassed mixture of toluene, H_2O, ethanol (2 : 2 : 1) (3.3 mL, 0.11 mol·L^{-1}) was added. The resulting mixture was stirred at 80°C for 18 h, then cooled to room temperature, filtered through a thin layer of silica gel and Na_2SO_4 using EtOAc (20 mL). The solvents were removed under reduced pressure and the residue was purified by the silica gel chromatography (cyclohexane/EtOAc 100:0→80:20) to give the desired product 14-(2-vinylphenyl)tetraphene as a white solid (85.5 mg, 259 µmol, 86%, m.p. 169.6 – 170.0°C): R_f 0.24 (cyclohexane/CH_2Cl_2 9:1); ν_{max}(neat): 3052w, 3010w, 2916w, 1931w, 1829w, 1624w, 1548w, 1262w, 1210w, 1158w, 1075w, 993m, 913m, 872m, 838w, 755s; ^{1}H NMR (500 MHz, $CDCl_3$) δ = 9.25 (1H, s, C12*H*), 8.91 (1H, d, ^3J 8.4 Hz, C1*H*), 8.34 (1H, s, C7*H*), 8.19 – 8.13 (1H, m, C11*H*), 8.04 (1H, dd, ^3J 6.3, ^4J 2.6 Hz, C8*H*), 7.80 – 7.76 (1H, m, C3`*H*), 7.73 (1H, dd, $^3J$ 8.4, $^3J$ 7.2 Hz, C2*H*), 7.68 (1H, d, $^3J$ 9.3 Hz, C6*H*), 7.61 – 7.53 (2H, m, C9*H*, C10*H*), 7.50 – 7.44 (2H, m, C3*H*, C4`*H*), 7.40 (1H, td, ^3J 7.4, ^4J 1.4 Hz, C5`*H*), 7.33 (1H, dd, $^3J$ 7.4, $^4J$ 1.1 Hz, C6`*H*), 7.31 (1H, d, ^3J 9.3 Hz, C5*H*), 6.39 (1H, dd, ^3J 17.5, ^3J 11.0 Hz, C7`*H*), 5.69 (1H, dd, $^3J$ 17.5, $^2J$ 1.2 Hz, C8`*H*), 5.03 (1H, dd, ^3J 11.0, ^2J 1.2 Hz, C8`*H*); $^{13}$C NMR (126 MHz, $CDCl_3$) δ = 139.71 (*C1`*), 139.65 (*C4*), 136.9 (*C2`*), 135.3 (*C7`*), 132.21 (*C11a*), 132.17 (*C7a*), 131.1 (*C6`*), 130.8 (*C12b*), 130.6 (*C4a*), 130.5 (*C6a*), 129.1 (*C12a*), 129.0 (*C3*), 128.7 (*C11*), 128.0 (*C4`*), 127.9 (C8), 127.7 (*C5`*), 127.5 (*C6*), 126.9 (*C7*), 126.3 (*C2*), 126.0 (*C9*), 125.9 (*C10*), 125.0 (*C3`*), 125.0 (*C5*), 122.6 (*C1*), 122.0 (*C12*), 114.9 (*C8`*); ESI-MS: m/z calcd. for $C_{26}H_{18}Ag$ 437.0454 found 437.0450 [M+Ag^+].

1-Vinyl-4-(2-vinylphenyl)naphthalene (3):

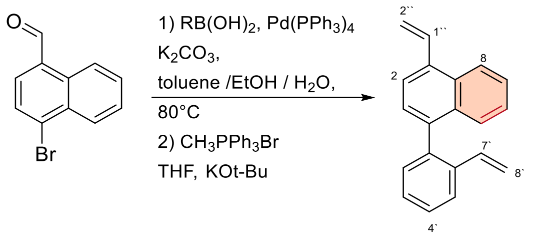

3

Step 1: 4-Bromo-1-naphthaldehyde (188 mg, 800 µmol, 1.00 eq.), (2-vinylphenyl)boronic acid (131 mg, 888 µmol, 1.11 eq.), $Pd(PPh_3)_4$ (46.2 mg, 40.0 µmol, 5 mol%), and K_2CO_3 (332 mg, 2.40 mmol, 3.00 eq.) were weighed into a 10 mL crimp cap vial. The vial was sealed, evacuated and backfilled with argon before a degassed mixture of toluene, H_2O, ethanol (2 : 2 : 1) (6.7 mL, 0.12 mol·L^{-1}) was added. The resulting mixture was stirred at 80°C for 18 h, then cooled to room temperature, filtered through a thin layer of silica gel and Na_2SO_4 using EtOAc (20 mL). The solvents were removed under reduced pressure and the residue was purified by the silica gel chromatography (cyclohexane/EtOAc 100:0→80:20) to give the desired 4-(2-vinylphenyl)-1-naphthaldehyde as orange oil (160 mg, 619 µmol, 77%): R_f 0.68 (cyclohexane/EtOAc 5:1).

Step 2: To a suspension of methyltriphenylphosphonium-bromide (310 mg, 867 µmol, 1.40 eq.) in THF (6.2 mL) at 0°C was added dry KO*t*-Bu (111 mg, 991 µmol, 1.60 eq.) under argon. After stirring the resulting suspension for 50 min at 0°C, a solution of 4-(2-vinylphenyl)-1-naphthaldehyde (160 mg, 619 µmol, 1.00 eq.) in THF (6.2 mL) was added dropwise. The reaction mixture was allowed to warm up to room temperature and stirred for 18 h. Water was then added and the mixture was extracted with EtOAc (3×30 mL). The combined organic layer was dried over anhydrous Na_2SO_4, filtered and the solvent was removed under reduced pressure. The residue was purified by silica gel column chromatography (cyclohexane/EtOAc 100:0→80:20) to give the desired product 1-vinyl-4-(2-vinylphenyl)naphthalene **3** as yellow oil (97.0 mg, 378 µmol, 61%): R_f 0.78 (cyclohexane/EtOAc 5:1); ν_{max}(neat): 3061w, 3023w, 2924w, 1829w, 1716w, 1688w, 1626w, 1578w, 1512w, 1424w, 1378w,, 1264w, 1056w, 989w, 915m, 847m, 764m; ^{1}H NMR (500 MHz, $CDCl_3$) δ = 8.18 (1H, d, ^3J 8.5 Hz, C8*H*), 7.79 – 7.73 (1H. m,C3`*H*), 7.71 – 7.64 (1H, m, C2*H*), 7.58 – 7.48 (3H, m, C1``*H*, C5*H*, 7*H*), 7.46 – 7.42 (1H, m,C4`*H*), 7.40 – 7.34 (2H, m, C6*H*, C5`*H*), 7.33 (1H, d, $^3J$ 7.3 Hz, C3*H*), 7.27 (1HM ddd, $^3J$ 7.5, $^4J$ 1.4, $^5J$ 0.4 Hz, C6`*H*), 6.33 (1H, dd, $^3J$ 17.5, $^3J$ 11.0 Hz, C7`*H*), 5.85 (1H, dd, $^3J$ 17.3, $^2J$ 1.5 Hz, C2``*H*), 5.66 (1H, dd, ^3J 17.5, ^2J 1.2 Hz, C8`*H*), 5.52 (1H, dd, ^3J 10.9, ^2J 1.5 Hz, C2``*H*), 5.00 (1H, dd, $^3J$ 11.0, $^2J$ 1.2 Hz, C8`*H*); $^{13}$C NMR (126 MHz, $CDCl_3$) δ = 139.5 (*C1`*), 138.9 (*C4*), 136.9 (*C2`*), 135.42 (*C1*), 135.37 (*C7`*), 134.6 (*C1``*), 132.5 (*C4a*), 131.2 (*C8a*), 131.1 (*C6`*), 128.0 (*C4`*), 127.6 (*C5`*), 127.5 (*C3*), 127.2 (*C5*), 126.1 (*C7*), 126.0 (*C6*), 124.9 (*C3`*), 124.1 (*C8*), 123.2 (*C2*), 117.3 (*C2``*), 114.6 (*C8`*); ESI-MS: m/z calcd. for $C_{20}H_{16}Ag$ 363.0297 found 363.0304 [M+Ag$^+$].

Note: 1-Vinyl-4-(2-vinylphenyl)naphthalene **3** was used directly in the next step due to its polymerization upon storage at 4°C.

1-Methyl-7-vinyl-4-(2-vinylphenyl)-1*H*-indole (5a):

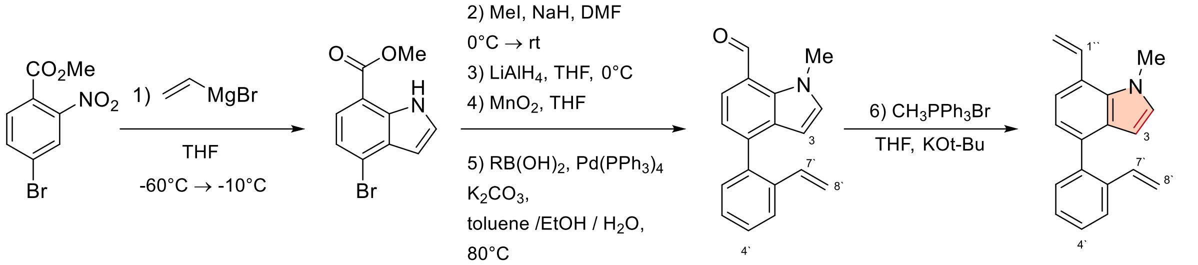

5a

Step 1: According to a modified literature procedure:[2] Methyl 4-bromo-2-nitrobenzoate (1.66 g, 6.40 mmol, 1.00 eq.) was dissolved in dry THF (9.0 mL) and the resulting solution was cooled to –60°C under argon atmosphere. Vinylmagnesium bromide solution in THF (17.9 mmol, 17.9 mL, 1.0 mol·L^{-1}, 2.80 eq.) was added dropwise at –60°C. The reaction mixture was stirred at –60°C for 1 h, then gradually warmed up to –10°C. The reaction progress was checked by ^{1}H-NMR. Upon the reaction completion, it was quenched with saturated aqueous NH_4Cl solution and extracted with EtOAc (3×50 mL). The combined organic layer was dried over anhydrous Na_2SO_4, filtered and the solvent was removed under reduced pressure. The residue was purified by flash silica gel column chromatography (cyclohexane/EtOAc 100:0→50:50) to give the desired methyl 4-bromo-1*H*-indole-7-carboxylate as a white solid (550 mg, 2.16 mmol, 34%). The spectral data were in accordance with those previously reported in the literature.[2]

Step 2: 4-Bromo-1*H*-indole-7-carboxylate (550 mg, 2.16 mmol, 1.00 eq.) was dissolved in dry DMF (8.0 mL). NaH (60% dispersion in mineral oil, 173 mg, 4.33 mmol, 2.00 eq.) was added portionwise at 0°C and the resulting solution was stirred at 0°C for 5 minutes before methyl iodide MeI (461 mg, 200 µL, 3.25 mmol, 1.50 eq.) was added dropwise at this temperature. The reaction mixture was allowed to warm up to room temperature, then quenched with MeOH, diluted with water and extracted with EtOAc (3×60 mL). The combined organic layer was washed with brine (3×100 mL), dried over anhydrous Na_2SO_4, filtered and the solvent was removed under reduced pressure. The residue was purified by flash silica gel column chromatography (cyclohexane/EtOAc 100:0→50:50) to give the desired methyl 4-bromo-1-methyl-1*H*-indole-7-carboxylate as a white solid (466 mg, 1.74 mmol, 80%).

Steps 3 and 4: To a solution of 4-bromo-1-methyl-1*H*-indole-7-carboxylate (466 mg, 1.74 mmol, 1.00 eq.) in dry THF (9.0 mL) was added $LiAlH_4$ (79.2 mg, 2.09 mmol, 1.20 eq.) portionwise at 0°C under argon. The reaction mixture was stirred for 2 h allowing to warm up to room temperature (the reaction progress was controlled by ^{1}H-NMR), cooled to 0°C, quenched with H_2O (0.25 mL) and 10% NaOH solution (0.16 mL). The resulting mixture was diluted with EtOAc and filtered through a thin silica gel layer. Solvents were removed under reduced pressure and the crude product was used in the next step without any additional purification. The obtained (4-bromo-1*H*-indol-7-yl)methanol was dissolved in THF (20 mL) and MnO_2 (1.81 g, 20.9 mmol, 12.0 eq.) was added portionwise. The reaction mixture was stirred for 18 hours at rt, filtered through celite (rinsing with EtOAc) and concentrated under reduced pressure. The residue was purified by flash silica gel column chromatography (cyclohexane/EtOAc 100:0→60:40) to give the title compound as a white solid (287 mg, 1.21 mmol, 69% over two steps): R_f 0.32 (cyclohexane/EtOAc 4:1).

Step 5: 4-Bromo-1-methyl-1*H*-indole-7-carbaldehyde (187 mg, 785 µmol, 1.00 eq.), (2-vinylphenyl)boronic acid (129 mg, 872 µmol, 1.11 eq.), $Pd(PPh_3)_4$ (45.4 mg, 39.3 µmol, 5 mol%), and K_2CO_3 (326 mg, 2.36 mmol, 3.00 eq.) were weighed into a 20 mL crimp cap vial. The vial was sealed, evacuated and backfilled with argon before a degassed mixture of toluene, H_2O, ethanol (2 : 2 : 1) (6.5 mL, 0.12 mol·L^{-1}) was added. The resulting mixture was stirred at 80°C for 18 h, then cooled to room temperature, filtered through a thin layer of silica gel and Na_2SO_4 using EtOAc (20 mL). The solvents were removed under reduced pressure and the residue was purified by the silica gel chromatography (cyclohexane/EtOAc 100:0→60:40) to give the desired product 1-methyl-4-(2-vinylphenyl)-1*H*-indole-7-carbaldehyde as yellow oil (160 mg, 612 µmol, 78%): R_f 0.40 (cyclohexane/EtOAc 5:1); ν_{max}(neat): 3059w, 2917w, 2850w, 2737w, 1886s, 1579s, 1520m, 1475w, 1394w, 1342w, 1317w, 1220m

1086m, 1032m, 791m; ^{1}H NMR (500 MHz, $CDCl_3$) δ = 10.26 (1H, s, C*H*O), 7.76 (1H, d, ^3J 7.4 Hz, C6*H*), 7.75 – 7.70 (1H, m, C3`*H*), 7.45 – 7.40 (1H, m, C4`*H*), 7.39 – 7.33 (2H, m, C5`*H*, C6`*H*), 7.14 (1H, d, ^3J 7.4 Hz, C5*H*), 7.06 (1H, d, ^3J 3.2 Hz, C2*H*), 6.51 (1H, dd, ^3J 17.5, ^3J 11.0 Hz, C7`*H*), 6.28 (1H, d, $^3J$ 3.2 Hz, C3*H*), 5.71 (1H, dd, $^3J$ 17.5, $^2J$ 1.2 Hz, C8`*H*), 5.10 (1H, dd, ^3J 11.0, ^2J 1.2 Hz, C8`*H*), 4.18 (3H, s, N–C*H*$_3$); $^{13}$C NMR (126 MHz, $CDCl_3$) δ = 190.9 (*C*HO), 141.0 (*C4*), 138.8 (*C1`*), 136.1 (*C2`*), 135.5 (*C7`*), 133.8 (*C7a*), 132.6 (*C2*), 131.3 (*C3a*), 130.4 (*C6*), 130.3 (*C6`*), 128.2 (*C4`*), 127.6 (*C5`*), 125.4 (*C3`*), 122.2 (*C7*) 120.8 (*C5*), 114.8 (*C8`*), 102.7 (*C3*), 39.3 (N–*C*H$_3$); ESI-MS: m/z calcd. for $C_{18}H_{16}NO$ 262.1126 found 262.1225 [M+H$^+$].

Step 6: To a suspension of methyltriphenylphosphonium-bromide (329 mg, 920 µmol, 2.00 eq.) in THF (4.6 mL) at 0°C was added dry KO*t*-Bu (119 mg, 1.06 mmol, 2.30 eq.) under argon. After stirring the resulting suspension for 50 min at 0°C, a solution of 1-methyl-4-(2-vinylphenyl)-1*H*-indole-7-carbaldehyde (120 mg, 460 µmol, 1.00 eq.) in THF (4.6 mL) was added dropwise. The reaction mixture was allowed to warm up to room temperature and stirred for 18 h. Water was then added and the mixture was extracted with EtOAc (3×30 mL). The combined organic layer was dried over anhydrous Na_2SO_4, filtered and the solvent was removed under reduced pressure. The residue was purified by neutral alumina column chromatography (cyclohexane/EtOAc 100:0→70:30) to give the desired product 1-methyl-7-vinyl-4-(2-vinylphenyl)-1*H*-indole **5a** as yellow oil (80.0 mg, 308 µmol, 67%): R_f 0.70 (cyclohexane/EtOAc 5:1); ν_{max}(neat):3089w, 3060w, 2974w, 2918w, 1824w, 1702w, 1623w, 1593w, 1520w, 1472m, 1396w, 1290s, 1087m, 988m, 906s, 823s; ^{1}H NMR (500 MHz, CD_2Cl_2) δ = 7.72 (1H, d, ^3J 7.8 Hz, C3`*H*), 7.65 – 7.55 (1H, m, C1``*H*), 7.40 – 7.35 (1H, m, C4`*H*), 7.35 – 7.31 (2H, m, C5`*H*, C6`*H*), 7.27 (1H, d, $^3J$ 7.4 Hz, C6*H*), 6.98 (1H, d, $^3J$ 3.2 Hz, C2*H*), 6.92 (1H, d, $^3J$ 7.4 Hz, C5*H*), 6.56 (1H, dd, $^3J$ 17.6, $^3J$ 11.0 Hz, C7`*H*), 6.12 (1H, d, $^3J$ 3.2 Hz, C3*H*), 5.75 – 5.63 (2H, m, C8`*H*, C2``*H*), 5.39 (1H, dd, ^3J 10.8, ^2J 1.6 Hz, C2``*H*), 5.06 (1H, dd, $^2J$ 11.0, $^3J$ 1.3 Hz, C8`*H*), 4.04 (3H, s, N–C*H*$_3$); $^{13}$C NMR (126 MHz, $CD_2Cl_2$) δ = 140.2 (*C1`*), 136.4 (*C2`*), 136.3 (*C7`*), 134.9 (*C1``*), 134.0 (*C7a*), 133.5 (*C4*), 131.4 (*C2*), 130.9 (*C6`*), 129.8 (*C3a*), 127.8 (*C4`*), 127.7 (*C5`*), 125.4 (*C3`*), 121.5 (*C5*), 120.9 (*C6*), 116.4 (*C2``*), 114.0 (*C8`*), 101.1 (*C3*), 37.7 (N–*C*H$_3$); ESI-MS: m/z calcd. for $C_{19}H_{17}AgN$ 366.0406 found 366.0408 [M+Ag$^+$].

Note: Decomposition of 1-methyl-7-vinyl-4-(2-vinylphenyl)-1*H*-indole **5a** was observed during silica gel chromatography, therefore neutral alumina has to be used for purification.

7-Vinyl-4-(2-vinylphenyl)benzofuran (5b):

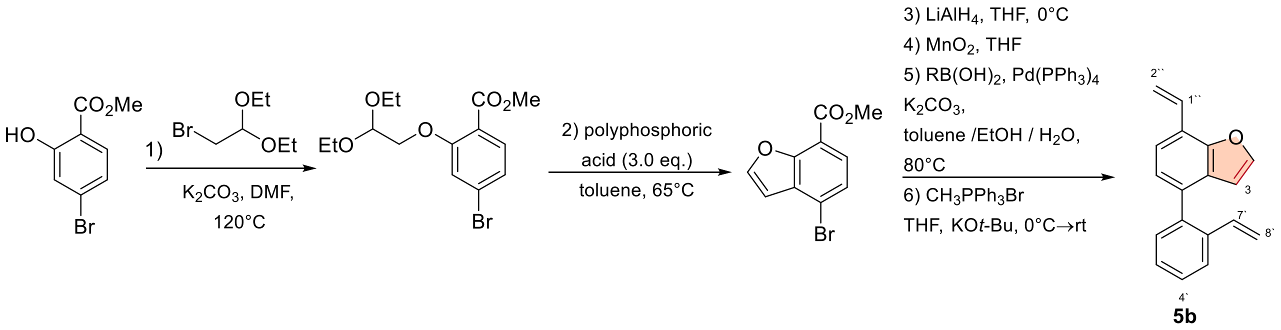

Steps 1 and 2: A mixture of methyl 4-bromo-2-hydroxybenzoate (809 mg, 3.50 mmol, 1.00 eq.), 2-bromo-1,1-diethoxyethane (1.04 g, 5.25 mmol, 1.50 eq.), dry K_2CO_3 (1.21 g, 8.75 mmol, 2.50 eq.) in dry DMF (7.0 mL) was stirred at 120°C for 18 h under argon. The reaction mixture was then cooled down to room temperature, diluted with water, extracted with EtOAc (3×50 mL). The combined organic layer was then washed with brine (3×100 mL), dried over anhydrous Na_2SO_4, filtered and the solvent was removed under reduced pressure. The obtained residue (1.57 g) was then introduced to the next step without additional purification. According to a modified literature procedure:[3] Crude methyl 4-bromo-2-(2,2-diethoxyethoxy)benzoate (1.57 g) in 8.8 mL of toluene was added to the mixture of polyphosphoric acid (4.59 g, 13.6 mmol) in 38 mL of toluene that was stirred at 65°C for 30 min. The obtained reaction mixture was stirred at 65°C for 4 h, then cooled down to room temperature, diluted with water and extracted with EtOAc (3×100 mL). The combined organic layer was washed with sodium bicarbonate solution, brine, dried over anhydrous Na_2SO_4, filtered and the solvent was removed under reduced pressure. The residue was purified by silica gel column chromatography (cyclohexane/EtOAc 100:0→70:30) to give methyl 4-bromobenzofuran-7-carboxylate as yellow solid (240 mg, 941 µmol, 27% over two steps): R_f 0.40 (cyclohexane/EtOAc 4:1).

Steps 3 and 4: To a solution of 4-bromobenzofuran-7-carboxylate (267 mg, 1.05 mmol, 1.00 eq.) in dry THF (6.6 mL) was added $LiAlH_4$ (47.7 mg, 1.26 mmol, 1.20 eq.) portionwise at 0°C under argon. The reaction mixture was stirred for 2 h allowing to warm up (the reaction progress was controlled by ^{1}H-NMR), cooled to 0°C, quenched with H_2O (0.10 mL) and 10% NaOH solution (0.15 mL). The resulting mixture was dilted with EtOAc and filtered through a thin silica gel layer. Solvents were removed under reduced pressure and the crude product was used in the next step without any additional purification. The obtained (4-bromobenzofuran-7-yl)methanol was dissolved in THF (12 mL) and MnO_2 (1.09 g, 12.6 mmol, 12.0 eq.) was added portionwise. The reaction mixture was stirred for 18 h at rt, filtered through celite (rinsing with EtOAc) and concenctrated under reduced pressure. The residue was purified by flash silica gel column chromatography (cyclohexane/EtOAc 100:0→60:40) to give the title compound as a white solid (73.0 mg, 324 µmol, 31% over two steps): R_f 0.46 (cyclohexane/EtOAc 4:1).

Step 5: 4-Bromobenzofuran-7-carbaldehyde (74.3 mg, 330 µmol, 1.00 eq.), (2-vinylphenyl)boronic acid (54.2 mg, 366 µmol, 1.11 eq.), $Pd(PPh_3)_4$ (19.1 mg, 16.5 µmol, 5 mol%), and K_2CO_3 (137 mg, 99.0 µmol, 3.00 eq.) were weighed into a 10 mL crimp cap vial. The vial was sealed, evacuated and backfilled with argon before a degassed mixture of toluene, H_2O, ethanol (2 : 2 : 1) (2.8 mL, 0.12 mol·L^{-1}) was added. The resulting mixture was stirred at 80°C for 18 h, then cooled to room temperature, filtered through a thin layer of silica gel and Na_2SO_4 using EtOAc (20 mL). The solvents were removed under reduced pressure and the residue was purified by the silica gel chromatography (cyclohexane/EtOAc 100:0→60:40) to give the desired product 4-(2-vinylphenyl)benzofuran-7-carbaldehyde **S1** as yellow oil (56.0 mg, 226 µmol, 68%): R_f 0.40 (cyclohexane/EtOAc 5:1); ν_{max}(neat): 3149w, 3060w, 2920w, 2850w, 2730w, 1688s, 1608s, 1538m, 1474w, 1410w, 1375m, 1252s, 1204m, 1135m, 1024m, 916w, 828w, 791w, 741w; ^{1}H NMR (500 MHz, $CDCl_3$) δ = 10.5 (1H, s, C*H*O), 7.87 (1H, d, ^3J 7.7 Hz, C6*H*), 7.77 (1H, d, ^3J 2.2 Hz, C2*H*), 7.76 – 7.70 (1H, m, C3`*H*), 7.48 – 7.42 (1H, m, C4`*H*), 7.39 (1H, td, ^3J 7.4, ^4J 1.3 Hz, C5`*H*), 7.36 – 7.31 (2H, m, C6`*H*, C5*H*), 6.64 (1H, d, ^3J 2.2 Hz, C3*H*), 6.51 (1H, dd, ^3J 17.5, ^3J 11.0 Hz, C7`*H*), 5.74 (1H, dd, $^3J$ 17.5, $^2J$ 1.1 Hz, C8`*H*), 5.17 (1H, dd, ^3J 11.0, ^2J 1.1 Hz, C8`*H*); ^{13}C NMR

(126 MHz, $CDCl_3$) δ = 188.8 (*C*HO), 153.8 (*C7a*), 146.5 (*C2*), 141.2 (*C4*), 137.6 (*C1`*), 136.1 (*C2`*), 135.2 (*C7`*), 130.2 (*C6`*), 129.1 (*C3a*), 128.7 (*C4`*), 127.9 (*C5`*), 126.3 (*C6*), 125.9 (*C3`*), 124.8 (*C5*), 120.4 (*C7*), 115.6 (*C8`*), 106.7 (*C3*); ESI-MS: m/z calcd. for $C_{17}H_{12}NaO_2$ 271.0730 found 271.0734 [$M+Na^+$].

Step 6: To a suspension of methyltriphenylphosphonium-bromide (161 mg, 451 µmol, 2.00 eq.) in THF (2.3 mL) at 0°C was added dry KO*t*-Bu (58.2 mg, 519 µmol, 2.30 eq.) under argon. After stirring the resulting suspension for 50 min at 0°C, a solution of 4-(2-vinylphenyl)benzofuran-7-carbaldehyde **S1** (56 mg, 226 µmol, 1.00 eq.) in THF (2.3 mL) was added dropwise. The reaction mixture was allowed to warm up to room temperature and stirred for 18 h. Water was then added and the mixture was extracted with EtOAc (3×30 mL). The combined organic layer was dried over anhydrous Na_2SO_4, filtered and the solvent was removed under reduced pressure. The residue was purified by neutral alumina column chromatography (cyclohexane/EtOAc 100:0→70:30) to give the desired product 7-vinyl-4-(2-vinylphenyl)benzofuran **5b** as yellow oil (44.0 mg, 179 µmol, 79%): R_f 0.70 (cyclohexane/EtOAc 5:1); ^{1}H NMR (500 MHz, CD_2Cl_2) δ = 7.78 – 7.71 (1H, m, C3`*H*), 7.70 (1H, d, *J* 2.2 Hz, C2*H*), 7.46 – 7.29 (4H, m, C6*H*, C4`*H*, C5`*H*, C6`*H*), 7.14 (1H, d, ^3J 7.3 Hz, C5*H*), 7.04 (1H, dd, ^3J 17.8, ^3J 11.3 Hz, C1``*H*), 6.68 – 6.52 (2H, m, C3*H*, C7`*H*), 6.26 (1H, dd, $^3J$ 17.8, $^2J$ 1.3 Hz, C2``*H*), 5.73 (1H, dd, ^3J 17.5, ^2J 1.2 Hz, C8`*H*), 5.54 (1H, dd, ^3J 11.3, ^2J 1.3 Hz, C2``*H*), 5.12 (1H, dd, $^3J$ 11.0, $^2J$ 1.2 Hz, C8`*H*); $^{13}$C NMR (126 MHz, $CD_2Cl_2$) δ = 152.6 (*C7a*), 145.5 (*C2*), 138.9 (*C1`*), 136.4 (*C2`*), 136.0 (*C7`*), 134.0 (*C4*), 131.8 (*C1``*), 130.8 (*C6`*), 128.2 (*C3a*), 128.0 (*C5`*), 125.8 (*C3`*), 124.9 (*C5*), 123.2 (*C6*), 121.7 (*C7*), 117.5 (*C2``*), 114.8 (*C8`*), 106.9 (*C3*); ESI-MS: m/z calcd. for $C_8H_{15}O$ 247.1117 found 247.1115 [$M+H^+$].

5,5'-(2,5-Divinyl-1,4-phenylene)bis(9,11-dimethylphenanthro[3,4-*d*][1,3]dioxole) (7a):

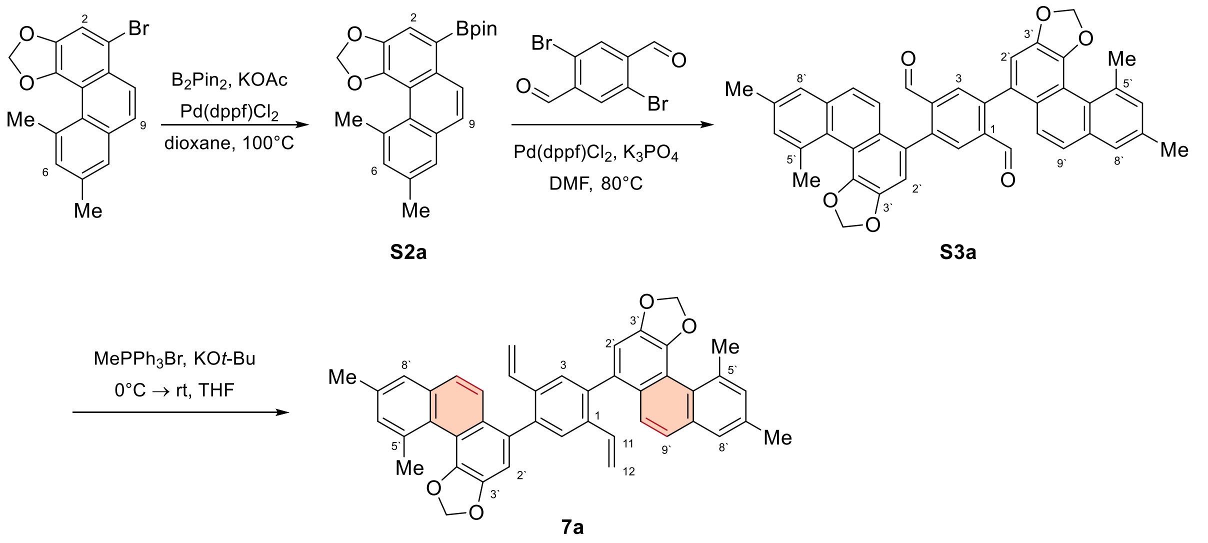

Step 1: 5-Bromo-9,11-dimethylphenanthro[3,4-d][1,3]dioxole (180 mg, 547 µmol, 1.00 eq.), bis(pinacolato)diboron (153 mg, 600 µmol, 1.10 eq.), dry KOAc (161 mg, 1.64 mmol, 3.00 eq.), Pd(dppf)$_2$Cl$_2$ (40 mg, 54.7 µmol, 10 mol%) were weighed in a 10 mL crimp cap vial. The vial was sealed, evacuated and backfilled with argon before dioxane (3.4 mL) was added. The reaction mixture was stirred at 100°C for 18 h under argon, cooled down to room temperature, filtered though a thin silica gel layer (using EtOAc to wash the filter bed). The solvents were removed under reduced pressure and crude product was purified by silica gel column chromatography (cyclohexane/EtOAc 100:0→60:40) to give the desired product **S2a** as a colorless oil (120 mg, 319 µmol, 58%): R$_f$ 0.67 (cyclohexane/EtOAc 5:1); ν$_{max}$(neat): 2977w, 2932w, 2874w, 1609w, 1528w, 1477w, 1446w, 1387s, 1343m, 1316w, 1272s, 1141s, 1103w, 1043m, 945w, 859m, 740w, 694w; ^{1}H NMR (500 MHz, CDCl$_3$) δ = 8.50 (1H, d, ^3J 9.1 Hz, C10*H*), 7.75 (1H, s, C2*H*), 7.45 – 7.34 (3H, m, C8*H*, C9*H*), 7.27 (1H, s, C6*H*), 6.12 (2H, s, OC*H*$_2$O), 2.66 (3H, s, C5-C*H*$_3$), 2.49 (3H, s, C7–C*H*$_3$), 1.42 (12H, s, 4×C*H*$_3$, BPin); ^{13}C NMR (126 MHz, CDCl$_3$) δ = 145.8 (*C4*), 144.6 (*C3*), 136.33 (*C5*), 136.26 (*C7*), 135.4 (*C10a*), 133.5 (*C8a*), 131.2 (*C6*), 126.7 (*C10*), 125.9 (*C9*), 125.5 (*C4b*), 124.9 (*C8*), 116.5 (*C2*), 116.4 (*C4a*), 100.3 (O*C*H$_2$O), 83.9 (–*C*(CH$_3$)$_2$–, BPin), 25.1 (–C(*C*H$_3$)$_2$–, BPin), 23.8 (C5–*C*H$_3$), 21.3 (C7–*C*H$_3$); ESI-MS: m/z calcd. for $C_{23}H_{26}BO_4$ 377.1923 found 377.1913 [M+H$^+$].

Step 2: 2-(9,11-Dimethylphenanthro[3,4-d][1,3]dioxol-5-yl)-4,4,5,5-tetramethyl-1,3,2-dioxaborolane **S2a** (155 mg, 411 µmol, 3.00 eq.), 2,5-dibromoterephthalaldehyde (40.0 mg, 137 µmol, 1.00 eq.), K$_3$PO$_4$ (116 mg, 548 µmol, 4.00 eq.), Pd(dppf)$_2$Cl$_2$ (15.8 mg, 13.7 µmol, 10 mol%) were weighed in a 10 mL crimp cap vial. The vial was sealed, evacuated and backfilled with argon before dry DMF (2.2 mL) was added. The reaction mixture was stirred at 100°C for 18 h under argon, cooled down to room temperature and diluted with water. Extraction with EtOAc (3×15 mL) was performed and the combined organic layer was washed with brine (3×20 mL), dried over anhydrous Na$_2$SO$_4$, filtered and the solvent was removed under reduced pressure. The residue was purified by flash silica gel column chromatography (cyclohexane/EtOAc 100:0→70:30) to give the desired product **S3a** as an orange solid (32.0 mg, 50.7 µmol, 37%, m.p. 250 – 252°C (decomposition)): R$_f$ 0.35 (cyclohexane/EtOAc 5:1); ν$_{max}$(neat): 2919m, 2852m, 1690s, 1590m, 1443s, 1392s, 1271s, 1202w, 1150w, 1102m, 1041m, 943w, 857m, 802w, 735w, 619w; ^{1}H NMR (500 MHz, CDCl$_3$) δ = 9.82 (1H, s, C*H*O), 9.82 (1H, s, C*H*O), 8.19 (2H, s, C3*H*, C6*H*), 7.45 – 7.33 (6H, m, C6`*H*, C8`*H*, C9`*H*), 7.32 (1H, s, *C2`*), 7.26 (1H, s, C2`*H*), 7.22 (1H, d, $^3J$ 9.1 Hz, C10`*H*), 7.14 (1H, d, ^3J 9.1 Hz, C10`*H*), 6.36 – 6.20 (4H, m, OC*H*$_2$O), 2.77 (6H, s, C5`–C*H*$_3$), 2.53 (3H, s, C7`–C*H*$_3$), 2.52 (3H, s, C7`–C*H*$_3$); ^{13}C NMR (126 MHz, CDCl$_3$) δ = 191.6 (*C*HO), 145.1 (*C3`*), 143.93 (*C4`*), 143.91 (*C4`*), 143.8 (*C1*, *C4*), 138.28 (*C2*, *C5*), 138.25 (*C2*, *C5*), 137.16 (*C7`*), 137.15 (*C7`*), 136.82 (*C5`*), 136.80 (*C5`*), 133.64 (*C8a`*), 133.62 (*C8a`*), 132.17 (*C6`*), 132.15 (*C6`*), 131.22 (*C3*, *C6*), 131.20 (*C3*, *C6*), 128.7 (*C10a`*), 128.6 (*C10a`*), 127.6 (*C1`*), 126.9 (*C9`*), 126.8 (*C9`*), 125.30 (*C8`*), 125.27 (*C8`*), 125.20 (*C4b`*), 125.18

(*C4b`*), 123.5 (*C10`*), 123.4 (*C10`*), 116.7 (*C4a`*), 111.2 (*C2`*), 111.1 (*C2`*), 100.8 (O*C*H_2O), 24.0 (C5`–*C*$H_3$), 21.3 (C7`–*C*H_3).

Step 3: To a suspension of methyltriphenylphosphonium-bromide (78.8 mg, 221 µmol, 4.50 eq.) in THF (1.0 mL) at 0°C was added dry KO*t*-Bu (28.6 mg, 255 µmol, 5.20 eq.) under argon. The resulting suspension was stirred for 40 min at 0°C and a solution of 2,5-bis(9,11-dimethylphenanthro[3,4-*d*][1,3]dioxol-5-yl)terephthalaldehyde **S3a** (30.9 mg, 49.0 µmol, 1.00 eq.) in THF (1.0 mL) was added dropwise. The reaction mixture was allowed to warm up to room temperature and stirred for 18 h. Water was then added and the mixture was extracted with EtOAc (3×15 mL). The combined organic layer was dried over anhydrous Na_2SO_4, filtered and the solvent was removed under reduced pressure. The residue was purified by flash silica gel column chromatography (cyclohexane/EtOAc 100:0→70:30) to give the desired product **7a** as a white solid (9.00 mg, 14.4 µmol, 29%, m.p. 250 – 253°C (decomposition)): R_f 0.59 (cyclohexane/EtOAc 5:1); ν_{max}(neat): 3080w, 2924w, 2867w, 1735w, 1694w, 1590m, 1476m, 1449s, 1426s, 1270s, 1205w, 1099m, 1042s, 990w, 945m, 909m, 856m, 804m; ^{1}H NMR (500 MHz, $CDCl_3$) δ = 7.69 (1H, s, C3*H*, C6*H*), 7.68 (1H, s, C3*H*, C6*H*), 7.41 (2H, d, ^3J 8.6 Hz, C8`*H*), 7.37 – 7.26 (7H, m, 1×C2`*H*, C6`*H*, C9`*H*, C10`*H*), 7.22 (1H, s, C2`*H*), 6.45 (2H, dd, ^3J 17.5, ^3J 11.1 Hz, C11*H*), 6.28 – 6.16 (4H, m, OCH_2O), 5.64 (2H, d, ^3J17.5 Hz, C12*H*), 5.02 (2H, d, ^3J 11.1 Hz, C12*H*), 2.78 (6H, s, C5`–C$H_3$), 2.52 (3H, s, C7`–CH_3), 2.51 (3H, s, C7`–C$H_3$); $^{13}$C NMR (126 MHz, $CDCl_3$) δ 144.9 (*C3`*), 142.8 (*C4`*), 139.4 (*C2*, *C5*), 136.7 (*C5`*, *C7`*), 136.4 (*C1*, *C4*), 136.3 (*C1*, *C4*), 134.8 (*C11*), 134.7 (*C11*), 133.84 (*C8a`*), 133.8 (*C8a`*), 132.38 (*C1`*), 132.36 (*C1`*), 131.63 (*C4b`*), 131.6 (*C4b`*), 128.2 (*C10a`*), 127.9 (*C3*, *C6*), 125.6 (*C6`*/*C9`*/*C10`*), 125.5 (*C6`*/*C9`*/*C10`*), 125.4 (*C6`*/*C9`*/*C10`*), 125.11 (*C8`*), 125.08 (*C8`*), 124.89, 124.86, 116.52 (*C4a`*), 116.50 (*C4a`*), 115.1 (*C12*), 115.0 (*C12*), 110.9 (*C2`*), 100.4 (O*C*H_2O), 24.0 (C5`–*C*$H_3$), 21.3 (C7`–*C*H_3); ESI-MS: m/z calcd. for $C_{44}H_{35}O_4$ 627.2530 found 627.2517 [M+H$^+$].

8,8'-(2,5-Divinyl-1,4-phenylene)bis(2,4-dimethoxy-5-methylphenanthrene) (7b):

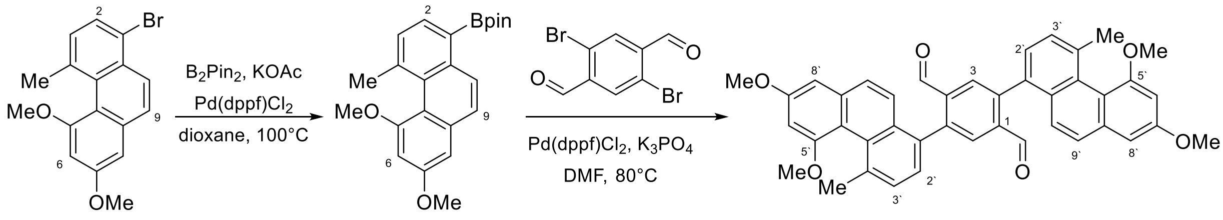

S2b **S3b**

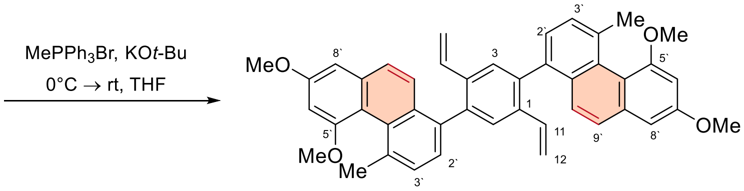

7b

Step 1: 1-Bromo-5,7-dimethoxy-4-methylphenanthrene (435 mg, 1.31 mmol, 1.00 eq.), bis(pinacolato)diboron (400 mg, 1.58 mmol, 1.20eq.), dry KOAc (387 mg, 3.94 mmol, 3.00 eq.), Pd(dppf)$_2$Cl$_2$ (96.1 mg, 13.1 µmol, 10 mol%) were weighed in a 10 mL crimp cap vial. The vial was sealed, evacuated and backfilled with argon before dry dioxane (7.2 mL) was added. The reaction mixture was stirred at 100°C for 18 h under argon, cooled down to room temperature, filtered though a thin silica gel layer (using EtOAc to wash the filter bed). The solvents were removed under reduced pressure and crude product was purified by silica gel column chromatography (cyclohexane/EtOAc 100:0→60:40) to give the desired product **S2b** as a colorless oil (300 mg, 793 µmol, 60%): R$_f$ 0.47 (cyclohexane/EtOAc 5:1); ν_{max}(neat): 2977w, 2933w, 2823w, 1602s, 1526m, 1453m, 1341s, 1286s, 1236w, 1218m, 1146s, 1065s, 1021w, 963w, 908w, 838w, 731w; ^{1}H NMR (500 MHz, CDCl$_3$) δ = 8.54 (1H, d, ^3J 8.9 Hz, C10*H*), 7.97 (1H, d, ^3J 7.2 Hz, C2*H*), 7.50 (1H, d, ^3J 8.9 Hz, C9*H*), 7.40 (1H, d, ^3J 7.2 Hz, C3*H*), 6.86 (1H, d, ^4J 2.4 Hz, C8*H*), 6.64 (1H, d, ^4J 2.4 Hz, C6*H*), 3.97 (3H, s, C7–OC*H*$_3$), 3.90 (3H, s, C5–OC*H*$_3$), 2.54 (3H, s, C4–C*H*$_3$), 1.41 (12H, s, 4×C*H*$_3$, Bpin); ^{13}C NMR (126 MHz, CDCl$_3$) δ = 159.0 (*C7*), 158.2 (*C5*), 140.2 (*C4*), 136.8 (*C10a*), 135.5 (*C8a*), 134.0 (*C2*), 128.9 (*C4a*), 128.4 (*C10*), 128.0 (*C3*), 126.0 (*C9*), 115.9 (*C4b*), 99.9 (*C8*), 98.0 (*C6*), 83.7 (–*C*(CH$_3$)$_2$–, BPin), 55.6 (C7–O*C*H$_3$), 55.1 (C5–O*C*H$_3$), 25.1 (–C(*C*H$_3$)$_2$–, BPin), 24.4 (C4–*C*H$_3$); ESI-MS: m/z calcd. for $C_{23}H_{28}BO_4$ 379.2079 found 379.2080 [M+H$^+$].

Step 2: 2-(5,7-Dimethoxy-4-methylphenanthren-1-yl)-4,4,5,5-tetramethyl-1,3,2-dioxaborolane **S2b** (96.0 mg, 254 µmol, 3.00 eq.), 2,5-dibromoterephthalaldehyde (24.7 mg, 84.6 µmol, 1.00 eq.), K$_3$PO$_4$ (71.8 mg, 338 µmol, 4.00 eq.), Pd(dppf)$_2$Cl$_2$ (9.78 mg, 8.46 µmol, 10 mol%) were weighed in a 10 mL crimp cap vial. The vial was sealed, evacuated and backfilled with argon before dry DMF (1.5 mL) was added. The reaction mixture was stirred at 100°C for 18 h under argon, cooled down to room temperature and diluted with water. Extraction with EtOAc (3×15 mL) was performed and the combined organic layer was washed with brine (3×20 mL), dried over anhydrous Na$_2$SO$_4$, filtered and the solvent was removed under reduced pressure. The residue was purified by flash silica gel column chromatography (cyclohexane/EtOAc 100:0→70:30) to give the desired product **S3b** as yellow oil (15.0 mg, 23.6 µmol, 28%, m.p. 250 – 252°C): R$_f$ 0.38 (cyclohexane/EtOAc 5:1); ν_{max}(neat): 2934w, 2855w, 2746w, 1689s, 1603w, 1527w, 1453m, 1400m, 1359m, 1310m, 1275m, 1160s, 1121m, 1058w, 943w, 832w, 792w; ^{1}H NMR (500 MHz, CDCl$_3$) δ = 9.81 (1H, s, C*H*O), 9.81 (1H, s, C*H*O), 8.19 (2H, s, C3*H*, C6*H*), 7.61 – 7.53 (2H, m, C3`*H*), 7.51 – 7.38 (5H, m, C9`*H*, C10`*H*, C2`*H*), 7.31 (1H, d, ^3J 8.9 Hz, C10`*H*), 6.88 (1H, d, $^4J$ 2.4 Hz, C8`*H*), 6.86 (1H, d, ^4J 2.4 Hz, C8`*H*), 6.78 – 6.72 (2H, m, C6`*H*), 4.00 (6H, s, C5`–OC*H*$_3$), 3.99 (3H, s, C7`–OC*H*$_3$), 3.98 (3H, s, C7`–OC*H*$_3$), 2.65 (6H, s, C4`–C*H*$_3$); ^{13}C NMR (126 MHz, CDCl$_3$) δ = 192.0 (*C*HO), 159.6 (*C7`*), 158.3 (*C5`*), 144.1 (*C2*, *C5*), 138.2 (*C1*, *C4*), 138.1 (*C1*, *C4*), 137.8 (*C4`*), 135.6 (*C8a`*), 131.3 (*C10a`*), 131.15 (*C3*, *C6*), 131.13 (*C3*, *C6*), 130.8 (*C1`*), 129.6 (*C4a`*), 128.64 (*C3`*), 128.62 (*C3`*), 127.6 (*C2`*), 127.5 (*C2`*), 127.1 (*C9`*), 127.0 (*C9`*), 125.24 (*C10`*), 125.2 (*C10`*), 115.60 (*C4b*), 115.57 (*C4b*), 100.21 (*C8`*),

100.18 (*C8`*), 98.7 (*C6`*), 55.7 (C7`–O*C*$H_3$), 55.2 (C5`–O*C*H_3), 24.3 (C4`–*C*H_3); ESI-MS: m/z calcd. for $C_{42}H_{35}O_6$ 635.2428 found 635.2426 [M+H^+].

Step 3: To a suspension of methyltriphenylphosphonium-bromide (98.0 mg, 274 µmol, 4.50 eq.) in THF (1.0 mL) at 0°C was added dry KO*t*-Bu (35.6 mg, 317 µmol, 5.20 eq.) under argon. The resulting suspension was stirred for 40 min at 0°C and a solution of 2,5-bis(5,7-dimethoxy-4-methylphenanthren-1-yl)terephthalaldehyde **S3b** (38.7 mg, 61.0 µmol, 1.00 eq.) in THF (1.0 mL) was added dropwise. The reaction mixture was allowed to warm up to room temperature and stirred for 18 h. Water was then added and the mixture was extracted with EtOAc (3×20 mL). The combined organic layer was dried over anhydrous Na_2SO_4, filtered and the solvent was removed under reduced pressure. The residue was purified by flash silica gel column chromatography (cyclohexane/EtOAc 100:0→70:30) to give the desired product **7b** as a white solid (26.0 mg, 41.2 µmol, 68%, m.p. 146.7 – 147°C): ν_{max}(neat): 2952w, 2930w, 2823w, 1603s, 1527w, 1453m, 1358w, 1308w, 1273w, 1160s, 1056w, 987w, 832w; ^{1}H NMR (500 MHz, $CDCl_3$) δ = 7.69 (1H, s, C3*H*/C6*H*), 7.68 (1H, s, C3*H*/C6*H*), 7.55 – 7.50 (2H, m, C3`*H*), 7.49 – 7.37 (6H, m, C2`*H*, C9`*H*, C10`*H*), 6.88 (1H, d, ^3J 2.4 Hz, C8`*H*), 6.86 (1H, d, $^3J$ 2.4 Hz, C8`*H*), 6.76 – 6.62 (2H, m, C6`*H*), 6.45 (2H, dd, $^3J$ 17.5, $^3J$ 11.1 Hz, C11*H*), 5.59 (2H, dd, $^3J$ 17.5, $^2J$ 0.9 Hz, C12*H*), 5.23 – 4.79 (2H, m, C12*H*), 3.99 (6H, s, C7`–*OCH₃*), 3.98 (3H, s, C5`–OC*H*₃), 3.97 (3H, s, C5`–OC*H*₃), 2.64 (6H, s, C4`–C*H*₃); $^{13}$C NMR (126 MHz, $CDCl_3$) δ = 159.2 (*C5`*), 158.3 (*C7`*), 139.5 (*C2*, *C5*), 136.2 (*C1*, *C4*, *C4`*), 136.1(*C1*, *C4*, *C4`*), 135.7 (*C8a`*), 135.22 (*C1`*), 135.19 (*C1`*), 135.13 (*C11*), 135.09 (*C11*), 131.2 (*C10a`*), 131.1 (*C10a`*), 129.2 (*C4a`*), 128.67 (*C3`*), 128.65 (*C3`*), 127.84 (*C3*/*C4*), 127.83 (*C3*/*C4*), 127.01 (*C2`*), 126.96 (*C2`*), 126.64 (*C10`*), 126.6 (*C10`*), 125.9 (*C9`*), 125.8 (*C9`*), 115.79 (*C4b`*), 115.76 (*C4b`*), 114.54 (*C12*), 114.49 (*C12*), 100.11 (*C8`*), 100.07 (*C8`*), 98.2 (*C6`*), 55.7 (C5`–O*C*$H_3$), 55.1 (C7`–O*C*H_3), 24.3 (C4`–*C*H_3); ESI-MS: m/z calcd. for $C_{44}H_{39}O_4$ 631.2843 found 631.2837 [M+H^+].

8,8'-(4,6-Divinyl-1,3-phenylene)bis(2,4-dimethoxy-5-methylphenanthrene) (7c):

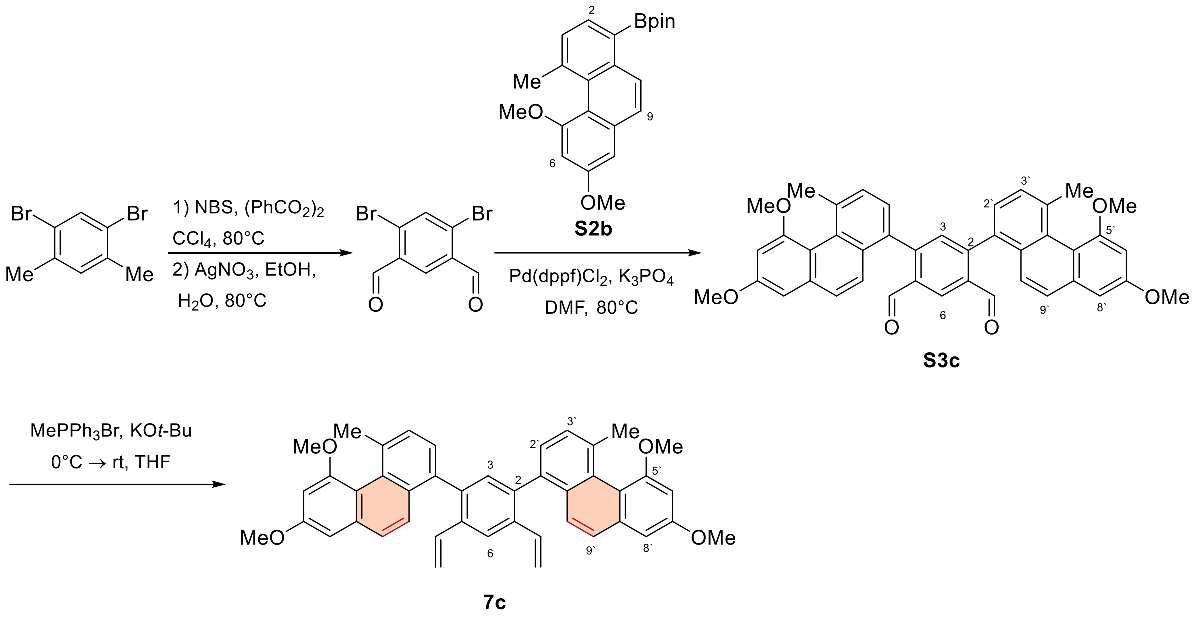

Step 1: According to a modified literature procedure:[4] *N*-bromosuccinimide (NBS) (730 mg, 4.10 mmol, 4.10 eq.) and benzoyl peroxide (7.27 mg, 30.0 µmol, 3 mol%) were added to a solution of 1,5-dibromo-2,4-dimethylbenzene (264 mg, 1.00 mmol, 1.00 eq.) in 1.0 mL CCl_4 under argon. The reaction mixture was stirred at reflux for 18 h, cooled down to room temperature, filtered off and rinsed with CCl_4 (1.0 mL). The solvent was then removed under reduced pressure and the obtained crude product was used for the next step without any additional purification. Crude 1,5-dibromo-2,4-bis(dibromomethyl)benzene was dissolved in EtOH (20 mL) and a solution of $AgNO_3$(713 mg, 4.20 mmol) in water (8.0 mL) was added. The resulting mixture was refluxed under argon for 18 h. EtOH was removed under reduced pressure and extraction with dichloromethane was performed (3×20 mL). The combined organic layer was dried over anhydrous Na_2SO_4, filtered and the solvent was removed under reduced pressure. The residue was purified by flash silica gel column chromatography (cyclohexane/EtOAc 100:0→70:30) to give the desired product 4,6-dibromoisophthalaldehyde as a white solid (140 mg, 480 µmol, 48% over two steps). The spectral data were in accordance with those previously reported in the literature.[5]

Step 2: 2-(5,7-Dimethoxy-4-methylphenanthren-1-yl)-4,4,5,5-tetramethyl-1,3,2-dioxaborolane (136 mg, 360 µmol, 3.00 eq.), 4,6-dibromoisophthalaldehyde (35.0 mg, 120 µmol, 1.00 eq.), K_3PO_4 (102 mg, 338 µmol, 4.00 eq.), Pd(dppf)$_2$Cl$_2$ (8.78 mg, 12.0 µmol, 10 mol%) were weighed in a 10 mL crimp cap vial. The vial was sealed, evacuated and backfilled with argon before dry DMF (2.0 mL) was added. The reaction mixture was stirred at 100°C for 18 h under argon, cooled down to room temperature and diluted with water. Extraction with EtOAc (3×15 mL) was performed and the combined organic layer was washed with brine (3×120 mL), dried over anhydrous Na_2SO_4, filtered and the solvent was removed under reduced pressure. The residue was purified by flash silica gel column chromatography (cyclohexane/EtOAc 100:0→70:30) to give the desired product **S3c** as a yellow solid (60.0 mg, 94.5 µmol, 79%, m.p. 174.8 – 176.0°C): R_f 0.15 (cyclohexane/EtOAc 5:1); ν_{max}(neat): 2992w, 2941w, 2833w, 1696m, 1603m, 1527w, 1454w, 1426w, 1359w, 1307w, 1220w, 1161w, 1116w, 1054w, 906m, 730m; ^{1}H NMR (500 MHz, $CDCl_3$) δ = 9.83 (1H, s, C*H*O), 9.81 (1H, s, C*H*O), 8.86 – 8.79 (1H, m, C6*H*), 7.68 – 7.67 (1H, m, C3*H*), 7.51 (2H, d, ^3J 7.6 Hz, C3`*H*), 7.48 – 7.38 (5H, m, C2`*H*, C9`*H*, 1×C10`*H*), 7.35 – 7.31 (1H, m, C10`*H*), 6.91 – 6.78 (2H, m, C8`*H*), 6.76 – 6.68 (2H, m, C6`*H*), 3.97 (12H, s, 4×OC*H*$_3$), 2.61 (6H, s, C4`–C*H*$_3$); ^{13}C NMR (126 MHz, $CDCl_3$) δ = 191.4 (*C*HO), 159.6 (*C7`*), 158.3 (*C5`*), 149.1 (*C2, C4*), 149.0 (*C2, C4*), 138.0 (*C4`*), 136.7 (*C3*), 136.5 (*C3*), 135.6 (*C8a`*), 135.5 (*C8a`*), 134.81 (*C1, C5*), 134.8 (*C1, C5*), 130.91 (*C10a`*), 130.86 (*C10a`*), 130.81 (*C1`*), 130.77 (*C1`*), 129.60 (*C4a`*), 129.56 (*C4a`*), 128.53 (*C3`*), 128.50, (*C3`*) 127.2 (*C9`*), 127.1 (*C2`*), 126.9 (*C6*), 126.8 (*C6*), 125.0 (*C10`*), 115.47 (*C4b`*), 100.2 (*C8`*), 98.7 (*C6`*), 55.7 (C5`–O*C*H$_3$/C7`–O*C*H$_3$), 55.2 (C5`–O*C*H$_3$/C7`–O*C*H$_3$), 24.3 (C4`–*C*H$_3$); ESI-MS: m/z calcd. for $C_{42}H_{35}O_6$ 635.2428 found 635.2424 [M+H$^+$].

Step 3: To a suspension of methyltriphenylphosphonium-bromide (135 mg, 378 µmol, 4.50 eq.) in THF (1.0 mL) at 0°C was added dry KO*t*-Bu (49.0 mg, 437 µmol, 5.20 eq.) under argon. The resulting suspension was stirred for 40 min at 0°C and a solution of 4,6-bis(5,7-dimethoxy-4-methylphenanthren-1-yl)isophthalaldehyde **S3c** (53.3 mg, 84.0 µmol, 1.00 eq.) in THF (1.0 mL) was added dropwise. The reaction mixture was allowed to warm up to room temperature and stirred for 18 h. Water was then added and the mixture was extracted with EtOAc (3×20 mL). The combined organic layer was dried over anhydrous Na_2SO_4, filtered and the solvent was removed under reduced pressure. The residue was purified by flash silica gel column chromatography (cyclohexane/EtOAc 100:0→70:30) to give the desired product **7c** as a beige solid (41.0 mg, 65.0 µmol, 77%, m.p. 166 – 167°C): R_f 0.61 (cyclohexane/EtOAc 5:1); ν_{max}(neat): 3085w, 3005w, 2932w, 2855w, 1735w, 1602m, 1526m, 1453m, 1423m, 1357m, 1308m, 1274m, 1216m, 1159s, 1117m, 1058w, 986w, 911w, 832w; ^{1}H NMR (500 MHz, $CDCl_3$) δ = 8.12 – 8.05 (1H, m, C6*H*), 7.49 – 7.33 (8H, m, C2`*H*, C3`*H*, C9`*H*, C10`*H*), 7.32 – 7.28 (1H, m, C3*H*), 6.84 (^3J 2.4 Hz), 6.82 (^3J 2.4 Hz) (1H, C8`*H*), 6.70 – 6.64 (2H, m, C6`*H*), 6.59 – 6.45 (2H, m, C11*H*), 5.83 – 5.74 (2H, m, C12*H*), 5.24 – 4.96 (2H, m, C12*H*), 3.962 (3H, s, C7`–OC$H_3$), 3.956 (3H, s, C7`–OCH_3), 3.950 (6H, s, C5`–OC$H_3$), 2.58 (6H, s, C4`–CH_3); ^{13}C NMR (126 MHz, $CDCl_3$) δ = 159.2 (*C7`*), 158.3 (*C5`*), 139.5 (*C2, C4*), 139.3 (*C2, C4*), 136.5 (*C1, C5*), 136.4 (*C1, C5*), 136.2 (*C4`*), 136.1 (*C4`*), 135.7 (*C8a`*), 135.6 (*C11*), 134.94 (*C1`*), 134.88 (*C1`*), 134.79 (*C3`*), 131.0 (*C10a`*), 129.22 (*C4a`*), 129.19 (*C4a`*), 128.64 (*C3`*), 128.58 (*C3`*), 127.0 (*C2`*), 126.6 (*C2`*), 126.5 (*C10`*), 125.7 (*C9`*), 115.8 (*C4b`*), 114.7 (*C12*), 100.0 (*C8`*), 98.2 (*C6`*), 55.7 (C7`–O*C*$H_3$), 55.1 (C5`–O*C*H_3), 24.2 (C4`–*C*H_3); ESI-MS: m/z calcd. for $C_{44}H_{39}O_4$ 631.2843 found 631.2836 [M+H$^+$].

5,5'-(3,7-Divinylnaphthalene-2,6-diyl)bis(9,11-dimethylphenanthro[3,4-*d*][1,3]dioxole) (7d):

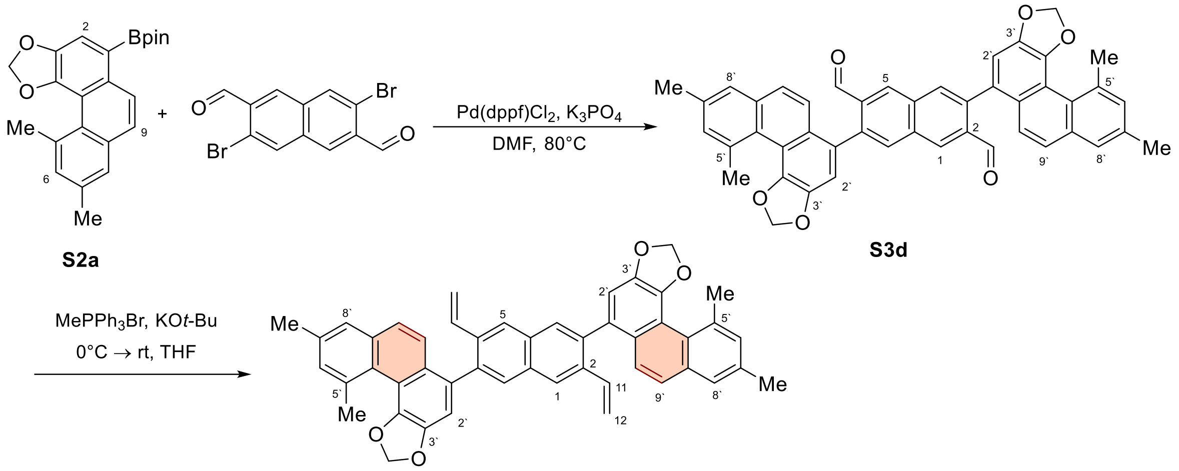

7d

Step 1: 2-(9,11-Dimethylphenanthro[3,4-*d*][1,3]dioxol-5-yl)-4,4,5,5-tetramethyl-1,3,2-dioxaborolane **S2a** (69.0 mg,183 µmol, 2.80 eq.), 3,7-dibromonaphthalene-2,6-dicarbaldehyde (22.0 mg, 65.5 µmol, 1.00 eq.), K_3PO_4 (55.6 mg, 262 µmol, 4.00 eq.), Pd(dppf)$_2$Cl$_2$ (4.79 mg, 6.55 µmol, 10 mol%) were weighed in a 10 mL crimp cap vial. The vial was sealed, evacuated and backfilled with argon before dry DMF (2.2 mL) was added. The reaction mixture was stirred at 100°C for 18 h under argon, cooled down to room temperature and diluted with water. Extraction with EtOAc (3×15 mL) was performed and the combined organic layer was washed with brine (3×20 mL), dried over anhydrous Na_2SO_4, filtered and the solvent was removed under reduced pressure. The residue was purified by flash silica gel column chromatography (cyclohexane/EtOAc 100:0→70:30) to give the desired product **S3d** as an orange solid (8.00 mg, 11.8 µmol, 18%, m.p. 185.5 – 190.0°C): R_f 0.32 (cyclohexane/EtOAc 5:1); ν_{max}(neat): 2967w, 2920w, 2860w, 1691m, 1588m, 1447m, 1397w, 1269m, 1226w, 1068m, 1042m, 943w, 904w, 857w, 755s; ^{1}H NMR (500 MHz, $CDCl_3$) δ = 9.84 (2H, s, C*H*O), 8.67 (2H, s, C1*H*, C5*H*), 8.19 (2H, s, C4*H*, C8*H*), 7.39 (2H, s, C8`*H*), 7.36 (2H, s, C6`*H*), 7.30 (2H, s, C2`*H*), 7.30 – 7.26 (2H, m, C9`*H*), 7.13 (d, ^3J 9.1 Hz), 7.11 (d, ^3J 9.1 Hz) (C10`*H*), 6.33 – 6.19 (4H, m, OC*H*$_2$O), 2.78 (6H, s, C5`–C*H*$_3$), 2.51 (6H, s, C7`–C*H*$_3$); $^{13}$C NMR (126 MHz, $CDCl_3$) δ = 192.0 (*C*HO), 145.0 (*C3`*), 143.6 (*C4`*), 140.6 (*C3*, *C7*), 137.1 (*C7`*), 136.8 (*C5`*), 135.5 (*C2*, *C6*), 134.5 (*C4a*, *C8a*), 133.7 (*C8a`*), 133.2 (*C4*, *C8*), 132.1 (*C6`*), 129.0 (*C1*, *C5*), 128.8 (*C10a*), 128.6 (*C1`*), 126.6 (*C9`*), 125.3 (*C8`*), 125.2 (*C4b`*) 123.63 (*C10`*), 123.6 (*C10`*), 116.6 (*C4a`*), 111.2 (*C2`*), 100.7 (O*C*H$_2$O), 24.0 (C5`–*C*H$_3$), 21.3 (C7`–*C*H$_3$); ESI-MS: m/z calcd. for $C_{46}H_{33}O_6$ 681.2272 found 681.2262 [M+H$^+$].

Step 2: To a suspension of methyltriphenylphosphonium-bromide (36.7 mg,103 µmol, 4.50 eq.) in THF (1.0 mL) at 0°C was added dry KO*t*-Bu (13.3 mg, 119 µmol, 5.20 eq.) under argon. The resulting suspension was stirred for 40 min at 0°C and a solution of 3,7-bis(9,11-dimethylphenanthro[3,4-*d*][1,3]dioxol-5-yl)naphthalene-2,6-dicarbaldehyde **S3d** (14.4 mg, 21.2 µmol, 1.00 eq.) in THF (1.0 mL) was added dropwise. The reaction mixture was allowed to warm up to room temperature and stirred for 18 h. Water was then added and the mixture was extracted with EtOAc (3×20 mL). The combined organic layer was dried over anhydrous Na_2SO_4, filtered and the solvent was removed under reduced pressure. The residue was purified by flash silica gel column chromatography (cyclohexane/EtOAc 100:0→70:30) to give the desired product **7d** as yellow oil (11.0 mg, 16.3 µmol, 77%): R_f 0.50 (cyclohexane/EtOAc 5:1); ν_{max}(neat): 3085w, 3005w, 2932w, 2855w, 1735w, 1602s, 1526w, 1453m, 1423m, 1357m, 1308m, 1216s, 1159s, 1058m, 986w, 911w, 832m; ^{1}H NMR (500 MHz, $CDCl_3$) δ = 8.15 – 8.08 (2H, s, C1*H*, C5*H*), 7.88 (2H, s, C4*H*, C8*H*), 7.37 (2H, s, C8`*H*), 7.33 (2H, s, C6`*H*), 7.25 – 7.20 (4H, m, C2`*H*, C9`*H*), 7.20 – 7.12 (2H, m, C10`*H*), 6.51 (2H, dd, $^3J$ 17.3, $^3J$ 11.0 Hz, C11*H*), 6.28 – 6.15 (4H, m, OC*H*$_2$O), 5.76 (2H, dd, $^3J$ 17.3, $^2J$ 1.0 Hz, C12*H*), 5.08 (2H, dd, $^3J$ 11.1, $^2J$ 1.0 Hz, C12*H*), 2.78 (6H, s, C5`–C*H*$_3$), 2.50 (6H, s, C7`–C*H*$_3$); $^{13}$C NMR (126 MHz, $CDCl_3$) δ = 144.8 (*C3`*), 142.8 (*C4`*), 138.9 (*C3*, *C7*), 136.7 (*C5`/C7`*), 136.50 (*C5`/C7`*), 136.46 (*C2*, *C6*), 135.6 (*C11*), 133.8 (*C8a`*), 132.51 (*C4a*, *C8a*), 132.48 (*C4a*, *C8a*), 131.6 (*C6`*), 130.2 (*C4*, *C8*), 128.3 (*C10a`*), 125.5 (*C9`*), 125.4 (*C4b`*), 125.1 (*C8`*), 124.9 (*C10`*), 124.8 (*C10`*), 123.84 (*C1`*), 123.83 (*C1*, *C5*), 116.5 (*C4a`*), 115.7 (*C12*), 115.6 (*C12*), 111.02 (*C2`*), 110.99 (*C2`*), 100.4 (O*C*H$_2$O), 24.0 (C5`–*C*H$_3$), 21.3 (C7`–*C*H$_3$); ESI-MS: m/z calcd. for $C_{48}H_{37}O_4$ 677.2686 found 677.2671 [M+H$^+$].

General Procedure A for the synthesis of 1-(2-vinylphenyl)-indoles 9a-i:

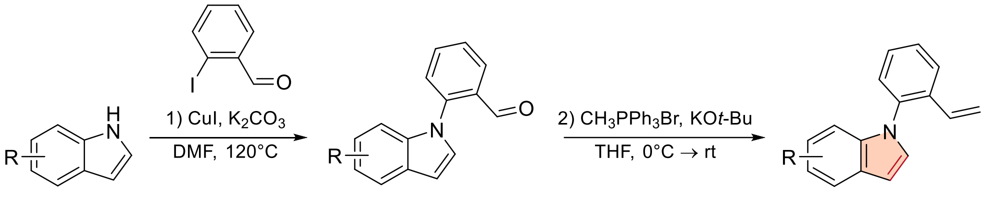

S4a-h **9a-h**

Step 1: A crimp cap vial was charged with the corresponding indole (1.00 eq.), 2-iodobenzaldehyde (1.00 eq.), K_2CO_3 (2.00 eq.) and CuI (0.10 eq.). The vial was sealed, evacuated and backfilled with argon before DMF (0.50 mol·L^{-1}) was added. The resulting mixture was stirred at 120°C for 18 h, then cooled to room temperature, diluted with water, and extracted with EtOAc (×3). The combined organic layer was washed with brine (×3), dried over anhydrous Na_2SO_4, filtered and the solvent was removed under reduced pressure. The residue was purified by flash silica gel column chromatography (cyclohexane/EtOAc 100:0→70:30) to give the desired aldehyde **S4a-h**.

Step 2: To a suspension of methyltriphenylphosphonium-bromide (1.50 eq. or 2.00 eq.) in THF (0.10 mol·L^{-1} with regard to aldehyde **S4a-h**) at 0°C was added dry KO*t*-Bu (1.70 eq. or 2.30 eq.) under argon. After stirring the resulting suspension for 50 min at 0°C, a solution of the corresponding aldehyde **S2a-h** (1.00 eq.) in THF (0.10 mol·L^{-1}) was added dropwise. The reaction mixture was allowed to warm up to room temperature and stirred for 18 h. Water was then added and the mixture was extracted with EtOAc (×3). The combined organic layer was dried over anhydrous Na_2SO_4, filtered and the solvent was removed under reduced pressure. The residue was purified by flash silica gel column chromatography (cyclohexane/EtOAc 100:0→70:30) to give the desired product **9a-h**.

5-Methoxy-1-(2-vinylphenyl)-1*H*-indole (9a):

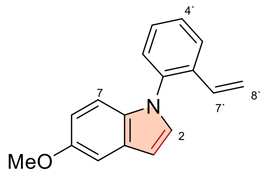

9a

Step 1 of the general procedure **A** was performed with 5-methoxy-1*H*-indole (125 mg, 850 µmol), 2-iodobenzaldehyde (197 mg, 850 µmol), K_2CO_3 (235 mg, 1.70 mmol) and CuI (16.2 mg, 85.0 µmol) to give 2-(5-methoxy-1*H*-indol-1-yl)benzaldehyde **S4a** as yellow oil (110 mg, 438 µmol, 52%): R_f 0.50 (cyclohexane/EtOAc 5:1). The obtained aldehyde **S4a** (106 mg, 420 µmol) was subjected to the step 2 utilizing methyltriphenylphosphonium-bromide (300 mg, 0.84 mmol, 2.00 eq.) and KO*t*-Bu (108 mg, 966 µmol, 2.30 eq.) to give **9a** as a yellow oil (70.0 mg, 281 µmol, 67%): R_f 0.55 (cyclohexane/EtOAc 5:1); ν_{max}(neat): 3067w, 2994w, 2945w, 2830w, 1701w, 1623w, 1576w, 1489s, 1487s, 1444m, 1344w, 1253s, 1220m, 1159s, 1116w, 1032m, 992w, 918w, 834w, 804m, 753m, 718m; ^{1}H NMR (500 MHz, $CDCl_3$) δ = 7.74 (1H, dd, ^3J 7.7, ^4J 1.7 Hz, C3`*H*), 7.46 – 7.41 (1H,m, C4`*H*), 7.39 (1H, td, ^3J 7.5, ^4J 1.7 Hz, C5`*H*), 7.35 – 7.30 (1H, m, C6`*H*), 7.16 (1H, d, ^3J 3.1 Hz, C2*H*), 7.15 (1H, d, ^4J.2.3 Hz, C4*H*), 7.02 – 6.95 (1H,m, C7*H*), 6.83 (1H, dd, ^3J 8.9, ^4J 2.3 Hz, C6*H*), 6.60 (1H, dd, ^3J 3.1, ^4J 0.8 Hz, C3*H*), 6.32 (1H, dd, ^3J 17.6, ^3J 11.0 Hz, C7`*H*), 5.72 (1H, dd, $^3J$ 17.6, $^2J$ 1.1 Hz, C8`*H*), 5.18 (1H, dd, ^3J 11.0, ^2J 1.1 Hz, C8`*H*), 3.87 (3H, s, OC*H*$_3$); $^{13}$C NMR (126 MHz, $CDCl_3$) δ = 154.6 (*C5*), 137.4 (*C1`*), 135.2 (*C2`*), 132.9 (*C7a*), 132.4 (*C7`*), 130.0 (*C2*), 129.0 (*C3a*), 128.7 (*C5`*), 128.2 (*C4`*, *C6`*), 126.4 (*C3`*), 116.3 (*C8`*), 112.5 (*C6*), 111.8 (*C7*), 102.6 (*C3*), 102.5 (*C4*), 56.0 (O*C*H$_3$); ESI-MS: m/z calcd. for $C_{17}H_{16}NO$ 250.1226 found 250.1217 [M+H$^+$].

5-Methyl-1-(2-vinylphenyl)-1*H*-indole (9b):

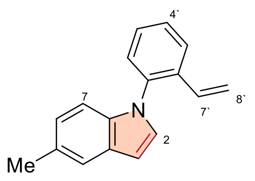

9b

Step 1 of the general procedure **A** was performed with 5-methyl-1*H*-indole (111 mg, 850 µmol), 2-iodobenzaldehyde (197 mg, 850 µmol), K_2CO_3 (235 mg, 1.70 mmol) and CuI (16.2 mg, 85.0 µmol) to give 2-(5-methyl-1*H*-indol-1-yl)benzaldehyde **S4b** as yellow oil (115 mg, 489 µmol, 58%): R_f 0.59 (cyclohexane/EtOAc 5:1). The obtained aldehyde **S4b** (113 mg, 480 µmol) was subjected to the step 2 utilizing methyltriphenylphosphonium-bromide (343 mg, 960 µmol, 2.00 eq.) and KO*t*-Bu (124 mg, 1.10 mmol, 2.30 eq.) to give **9b** as yellow oil (80.0 mg, 343 µmol, 71%): R_f 0.60 (cyclohexane/EtOAc 6:1); ν_{max}(neat): 3093w, 3015w, 2916w, 2855w, 1490s, 1469m, 1366w, 1332m, 1295w, 1222m, 1160m, 1118w, 1061w, 992w, 916w, 795w, 760m, 718m; ^{1}H NMR (500 MHz, $CDCl_3$) δ = 7.78 – 7.69 (1H, m, C3\`*H*), 7.51 – 7.47 (1H, m, C4*H*), 7.45 – 7.41 (1H, m, C4\`*H*), 7.39 (td, ^3J 7.5, ^4J 1.7 Hz, C5\`*H*), 7.35 – 7.30 (1H, m, C6\`*H*), 7.15 (1H, d, ^3J 3.2 Hz, C2*H*), 7.01 – 6.97 (2H, m, C6*H*, C7*H*), 6.60 (1H, d, ^3J 3.2 Hz, C3*H*), 6.32 (1H, dd, ^3J 17.5, ^3J 11.0 Hz, C7\`*H*), 5.73 (1H, dd, $^3J$ 17.5, $^2J$ 1.1 Hz, C8\`*H*), 5.18 (1H, dd, ^3J 11.0, ^2J 1.1 Hz, C8\`*H*), 2.47 (3H, d, $^4J$ 0.8 Hz, C5–C*H*$_3$); $^{13}$C NMR (126 MHz, $CDCl_3$) δ = 137.4 (*C1\`*), 136.0 (*C7a*), 135.2 (*C2\`*), 132.4 (*C7\`*), 129.6 (*C2*), 129.5 (*C5*), 128.8 (*C3a*), 128.7 (*C5\`*), 128.3 (*C6\`*), 128.2 (*C4\`*), 126.4 (*C3\`*), 123.9 (*C6*), 120.6 (*C4*), 116.2 (*C8\`*), 110.7 (*C7*), 102.5 (*C3*), 21.5 (C5–*C*H$_3$); ESI-MS: m/z calcd. for $C_{17}H_{16}N$ 234.1277 found 234.1277 [M+H$^+$].

7-Fluoro-1-(2-vinylphenyl)-1*H*-indole (9c):

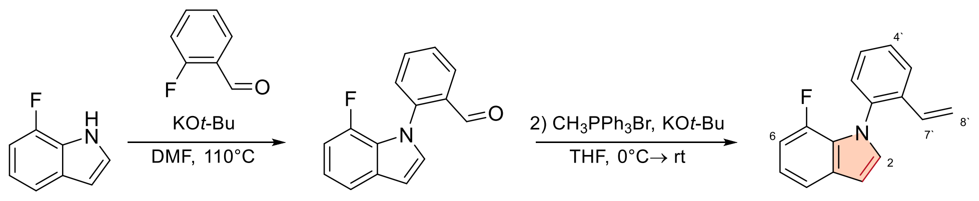

S4c **9c**

Step 1: A crimp cap vial was charged with 7-fluoro-1*H*-indole (115 mg, 850 µmol, 1.00 eq.), KO*t*-Bu (143 mg, 1.28 mmol, 1.50 eq.), and 3.7 mL of dry DMF under argon. The obtained mixture was stirred at 110°C while 2-fluorobenzaldehyde (160 mg, 1.29 mmol, 1.52 eq.) was added dropwise over 10 minutes. The reaction mixture was stirred at 110°C for 16 h, then cooled to room temperature, diluted with water, and extracted with EtOAc (3×20 mL). The combined organic layer was washed with brine (3×80 mL), dried over anhydrous Na_2SO_4, filtered and the solvent was removed under reduced pressure. The residue was purified by flash silica gel column chromatography (cyclohexane/EtOAc 100:0→70:30) to give the desired aldehyde **S4c** as a yellow oil (68.2 mg, 285 µmol, 34%): R_f 0.66 (cyclohexane/EtOAc 5:1).

Step 2: 2-(7-Fluoro-1*H*-indol-1-yl)benzaldehyde (66.0 mg, 276 µmol) **S4c** was subjected to the step 2 according of the general procedure **A** utilizing methyltriphenylphosphonium-bromide (138 mg, 386 µmol, 1.40 eq.) and KO*t*-Bu (49.5 mg, 441 µmol, 1.60 eq.) to give **9c** as a transparent oil (32.0 mg, 135 µmol, 49%): R_f 0.69 (cyclohexane/EtOAc 9:1); ν_{max}(neat): 3109w, 3074w, 2858w, 2758w, 1696s, 1599m, 1595m, 1491s, 1459w, 1398w, 1340w, 1277w, 1241m, 1160w, 1048w, 955w, 824w, 768m, 719m; ^{1}H NMR (500 MHz, $CDCl_3$) δ = 7.72 – 7.67 (1H, m, C3\`*H*), 7.48 – 7.40 (2H, m, C4\`*H*, C4*H*), 7.39 – 7.33 (2H, m, C5\`*H*, C6\`*H*), 7.11 (1H, d, ^3J 3.1 Hz, C2*H*), 7.05 (1H, td, ^3J 7.9, ^4J 4.4 Hz, C5*H*), 6.85 (1H, ddd, ^3J 12.2, ^3J 7.8, ^4J 0.7 Hz, C6*H*), 6.70 – 6.63 (1H, m,

C3*H*), 6.31 (1H, dd, ^3J 17.5, ^3J 11.1 Hz, C7`*H*), 5.69 (1H, dd, $^3J$ 17.5, $^2J$ 1.1 Hz, C8`*H*), 5.18 (1H, dd, ^3J 11.1, ^2J 1.1 Hz, C8`*H*); $^{13}$C NMR (126 MHz, $CDCl_3$) δ = 150.2 (d, $^1J_{CF}$ 246.8 Hz, *C7*), 138.2 (*C1`*), 135.5 (*C2`*), 132.61 (d, $^2J_{CF}$ 4.7 Hz, *C7a*), 131.9 (*C7`*), 131.0 (*C2*), 128.6 (*C4`*), 128.3 (*C6`*), 128.1 (*C5`*), 125.4 (*C3a*), 125.8 (*C3`*), 120.5 (d, $^3J_{CF}$ 6.4 Hz, *C5*), 116.8 (d, $^4J_{CF}$ 28.6 Hz, *C4*), 116.6 (*C8`*), 108.1 (d, $^2J_{CF}$ 18.9 Hz, *C6*), 103.6 (*C3*); ^{19}F NMR (376 MHz, $CDCl_3$) δ –132.28; ESI-MS: m/z calcd. for $C_{16}H_{13}FN$ 238.1027 found 238.1023 [$M+H^+$].

5-Fluoro-1-(2-vinylphenyl)-*1H*-indole (9d):

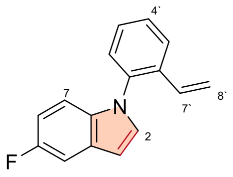

9d

Step 1 of the general procedure **A** was performed with 5-fluoro-1*H*-indole (176 mg, 1.30 mmol), 2-iodobenzaldehyde (302 mg, 1.30 mmol), K_2CO_3 (359 mg, 2.60 mmol) and CuI (24.8 mg, 130 µmol) to give 2-(5-fluoro-1*H*-indol-1-yl)benzaldehyde **S4d** as yellow oil (165 mg, 690 µmol, 53%): R_f 0.45 (cyclohexane/EtOAc 5:1). The obtained aldehyde **S4d** (165 mg, 690 µmol) was subjected to the step 2 utilizing methyltriphenylphosphonium-bromide (370 mg, 1.03 mmol, 1.50 eq.) and KO*t*-Bu (132 mg, 1.17 mmol, 1.70 eq.) to give **9d** as a transparent oil (100 mg, 421 µmol, 61%): R_f 0.63 (cyclohexane/EtOAc 9:1); ν_{max}(neat): 3068w, 2963w, 1624w, 1578w, 1490s, 1473s, 1448w, 1335w, 1260s, 1136m, 1093s, 1018s, 960w, 917w, 860s, 799s; ^{1}H NMR (500 MHz, $CDCl_3$) δ = 7.75 (1H, dd, ^3J 7.8, ^4J 1.6 Hz, C3`*H*), 7.49 – 7.43 (1H, m, C4`*H*), 7.40 (1H, td, ^3J 7.6, ^4J 1.6 Hz, C5`*H*), 7.35 – 7.29 (2H, m, C6`*H*, C4*H*), 7.22 (1H, d, ^3J 3.2 Hz, C2*H*), 6.99 (1H, dd, ^3J 8.9, $^3J_{HF}$ 4.5 Hz, C7*H*), 6.91 (1H, td, ^3J 9.1, ^4J 2.5 Hz, C6*H*), 6.63 (1H, dd, ^3J 3.2, ^4J 0.7 Hz, C3*H*), 6.26 (1H, dd, ^3J 17.5, ^3J 11.0 Hz, C7`*H*), 5.73 (1H, dd, $^3J$ 17.5, $^2J$ 1.0 Hz, C8`*H*), 5.19 (1H, dd, ^3J 11.0, ^2J 1.0 Hz, C8`*H*); $^{13}$C NMR (126 MHz, $CDCl_3$) δ = 158.3 (d, $^1J_{CF}$ 234.8 Hz, *C5*), 137.0 (*C1`*), 135.3 (*C2`*), 134.2 (*C7a*), 132.1 (*C7`*), 131.0 (*C2*), 128.8 (*C5`*, *C3a*), 128.5 (*C4`*), 128.3 (*C6`*), 126.5 (*C3`*), 116.6 (*C8`*), 111.7 (d, $^3J_{CF}$ 9.7 Hz, *C7*), 110.7 (d, $^2J_{CF}$ 26.3 Hz, *C6*), 105.7 (d, $^2J_{CF}$ 23.5 Hz, *C4*), 102.9 (d, $^4J_{CF}$ 4.7 Hz, *C3*); ^{19}F NMR (376 MHz, $CDCl_3$) δ = –124.69; ESI-MS: m/z calcd. for $C_{16}H_{12}AgFN$ 343.9999 found 343.9993 [$M+Ag^+$].

6-Fluoro-1-(2-vinylphenyl)-1*H*-indole (9e):

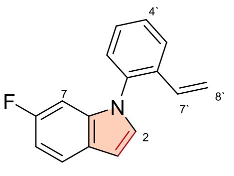

9e

Step 1 of the general procedure **A** was performed with 6-fluoro-1*H*-indole (176 mg, 1.30 mmol), 2-iodobenzaldehyde (302 mg, 1.30 mmol), K_2CO_3 (359 mg, 2.60 mmol) and CuI (24.8 mg, 130 µmol) to give 2-(6-fluoro-1*H*-indol-1-yl)benzaldehyde **S4e** as yellow oil (197 mg, 823 µmol, 63%): R_f 0.59 (cyclohexane/EtOAc 5:1). The obtained aldehyde **S4e** (196 mg, 820 µmol) was subjected to the step 2 utilizing methyltriphenylphosphonium-bromide (586 mg, 1.64 mmol, 2.00 eq.) and KO*t*-Bu (212 mg, 1.89 mmol, 2.30 eq.) to give **9e** as a white solid (155 mg, 653 µmol, 80%, m.p. 62.5 – 63.1 °C): R_f 0.81 (cyclohexane/EtOAc 9:1); ν_{max}(neat): 3067w, 2921w, 1619m, 1485s, 1465m, 1342m, 1290w, 1214s, 1177m, 1119w, 991w, 933m, 834w, 768m, 716m, 630s; ^{1}H NMR (500 MHz, $CDCl_3$) δ = 7.76 (1H, dd, ^3J 7.8, ^4J 1.6 Hz, C3`*H*), 7.59 (1H, dd, $^3J$ 8.6, $^4J$ 5.3 Hz, C4*H*), 7.49 – 7.43 (1H, m, C4`*H*), 7.41 (1H, td, ^3J 7.6, ^4J 1.6 Hz, C5`*H*), 7.34 – 7.30 (1H,

m, C6`*H*), 7.17 (1H, d, $^{3}J$ 3.2 Hz, C2*H*), 6.92 (1H, ddd, $^{3}J$ 9.5, $^{3}J$ 8.7, $^{4}J$ 2.3 Hz, C5*H*), 6.76 (1H, dd, $^{3}J$ 9.8, $^{4}J$ 2.3 Hz, C7*H*), 6.65 (1H, dd, $^{3}J$ 3.2, $^{4}J$ 0.9 Hz, C3*H*), 6.28 (1H, dd, $^{3}J$ 17.5, $^{3}J$ 11.0 Hz, C7`*H*), 5.74 (1H, dd, ^{3}J 17.5, ^{2}J 1.0 Hz, C8`*H*), 5.21 (1H, dd, $^{3}J$ 11.0, $^{2}J$ 1.0 Hz, C8`*H*); ^{13}C NMR (126 MHz, $CDCl_3$) δ = 160.3 (d, $^{1}J_{CF}$ 238.0 Hz, *C6*), 137.6 (d, $^{3}J_{CF}$ 12.1 Hz, *C7a*), 136.8 (*C1`*), 135.2 (*C2`*), 132.1 (*C7`*), 129.9 (d, $^{5}J_{CF}$ 3.7 Hz, *C2*), 128.8 (*C5`*), 128.6 (*C4`*), 128.1 (*C6`*), 126.5 (*C3`*), 124.9 (*C3a*), 121.7 (d, $^{3}J_{CF}$ 10.0 Hz, *C4*), 116.7 (*C8`*), 109.0 (d, $^{2}J_{CF}$ 24.6 Hz, *C5*), 103.0 (*C3*), 97.4 (d, $^{2}J_{CF}$ 26.7 Hz, *C7*); ^{19}F NMR (376 MHz, $CDCl_3$) δ = –120.42; ESI-MS: m/z calcd. for $C_{16}H_{13}FN$ 238.1027 found 237.1021 [M+H^{+}].

5-Chloro-1-(2-vinylphenyl)-1*H*-indole (9f):

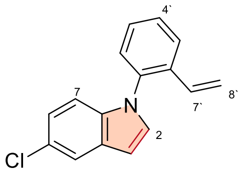

9f

Step 1 of the general procedure **A** was performed with 5-chloro-1*H*-indole (129 mg, 850 µmol), 2-iodobenzaldehyde (197 mg, 850 µmol), K_2CO_3 (235 mg, 1.70 mmol) and CuI (16.2 mg, 85.0 µmol) to give 2-(5-chloro-1*H*-indol-1-yl)benzaldehyde **S4f** as yellow oil (170 mg, 665 µmol, 78%): R_f 0.48 (cyclohexane/EtOAc 5:1). The obtained aldehyde **S4f** (161 mg, 630 µmol) was subjected to the step 2 utilizing methyltriphenylphosphonium-bromide (450 mg, 1.26 mmol, 2.00 eq.) and KO*t*-Bu (163 mg, 1.45 mmol, 2.30 eq.) to give **9f** as yellow oil (90.0 mg, 355 µmol, 56%): R_f 0.67 (cyclohexane/EtOAc 6:1); ν_{max}(neat): 3066w, 2968w, 2929w, 2855w, 1600w, 1570w, 1487s, 1462s, 1368w, 1327w, 1286w, 1229w, 1149w, 1064m, 1019w, 955w, 916w, 869w, 797m, 753s, 719s; ^{1}H NMR (500 MHz, $CDCl_3$) δ = 7.75 (1H, dd, ^{3}J 7.8, ^{4}J 1.6 Hz, C3`*H*), 7.69 – 7.58 (1H, m, C4*H*), 7.49 – 7.44 (1H, m, C4`*H*), 7.40 (1H, td, ^{3}J 7.6, ^{4}J 1.6 Hz, C5`*H*), 7.31 (1H, dd, $^{3}J$ 7.6, $^{4}J$ 1.3 Hz, C6`*H*), 7.21 (1H, d, ^{3}J 3.2 Hz, C2*H*), 7.16 – 7.05 (1H, m, C6*H*), 7.02 – 6.95 (1H, m, C7*H*), 6.62 (1H, dd, ^{3}J 3.2, ^{5}J 0.8 Hz, C3*H*), 6.24 (1H, dd, ^{3}J 17.5, ^{3}J 11.0 Hz, C7`*H*), 5.73 (1H, dd, $^{3}J$ 17.5, $^{2}J$ 1.0 Hz, C8`*H*), 5.19 (1H. dd, ^{3}J 11.0, ^{2}J 1.0 Hz, C8`*H*); $^{13}$C NMR (126 MHz, $CDCl_3$) δ = 136.8 (*C1`*), 136.0 (*C7a*), 135.3 (*C2`*), 132.0 (*C7`*), 130.7 (*C2*), 129.5 (*C3a*), 128.8 (*C5`*), 128.7 (*C4`*), 128.2 (*C6`*), 126.5 (*C3`*), 126.0 (*C5*), 122.6 (*C6*), 120.3 (*C4*), 116.7 (*C8`*), 112.0 (*C7*), 102.6 (*C3*); ESI-MS: m/z calcd. for $C_{16}H_{12}ClN$ 254.0731 found 254.0727 [M+Ag^{+}].

6-Chloro-1-(2-vinylphenyl)-1*H*-indole (9g):

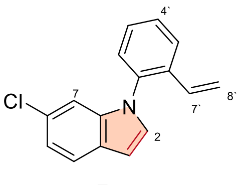

7g

Step 1 of the general procedure **A** was performed with 6-chloro-1*H*-indole (129 mg, 850 µmol), 2-iodobenzaldehyde (197 mg, 850 µmol), K_2CO_3 (235 mg, 1.70 mmol) and CuI (16.2 mg, 85.0 µmol) to give 2-(6-chloro-1*H*-indol-1-yl)benzaldehyde **S4g** as yellow oil (122 mg, 477 µmol, 56%): R_f 0.36 (cyclohexane/EtOAc 9:1). The obtained aldehyde **S4g** (120 mg, 470 µmol) was subjected to the step 2 utilizing methyltriphenylphosphonium-bromide (336 mg, 940 µmol, 2.00 eq.) and KO*t*-Bu (121 mg, 1.08 mmol, 2.30 eq.) to give **9g** as a transparent oil (55.0 mg, 217 mmol, 46%): R_f 0.71 (cyclohexane/EtOAc 5:1);

ν_{max}(neat): 3067w, 2986w, 2921w, 2851w, 1844w, 1701w, 1600w, 1566w, 1506m, 1462s, 1336m, 1234w, 1142w, 1060w, 991w, 918w, 849w, 807w, 768w, 718w; ^{1}H NMR (500 MHz, $CDCl_3$) δ = 7.76 (1H, dd, ^{3}J 7.8, ^{4}J 1.5 Hz, C3\`*H*), 7.59 (1H, d, $^{3}J$ 8.4 Hz, C4*H*), 7.47 (1H, td, $^{3}J$ 7.8, $^{4}J$ 1.2 Hz, C4\`*H*), 7.41 (1H, td, ^{3}J 7.8, ^{4}J 1.5 Hz, C5\`*H*), 7.32 (1H, dd, $^{3}J$ 7.7, $^{4}J$ 1.2 Hz, C6\`*H*), 7.17 (1H, d, ^{3}J 3.2 Hz, C2*H*), 7.12 (1H, dd, ^{3}J 8.4, ^{4}J 1.9 Hz, C5*H*), 7.09 – 7.05 (1H, m, C7*H*), 6.65 (1H, dd, ^{3}J 3.2, ^{4}J 0.8 Hz, C3*H*), 6.26 (1H, dd, ^{3}J 17.5, ^{3}J 11.0 Hz, C7\`*H*), 5.73 (1H, dd, $^{3}J$ 17.5, $^{2}J$ 1.0 Hz, C8\`*H*), 5.21 (1H, dd, ^{3}J 11.0, ^{2}J 1.0 Hz, C8\`*H*); $^{13}$C NMR (126 MHz, $CDCl_3$) δ = 137.9 (*C7a*), 136.6 (*C1\`*), 135.3 (*C2\`*), 131.9 (*C7\`*), 130.2 (*C2*), 128.8 (*C5\`*), 128.7 (*C4\`*), 128.4 (*C6*), 128.2 (*C6\`*), 127.0 (*C3a*), 126.6 (*C3\`*), 121.8 (*C4*), 121.0 (*C5*), 116.9 (*C8\`*), 110.9 (*C7*), 103.1 (*C3*); ESI-MS: m/z calcd. for $C_{16}H_{12}ClN$ 253.0653 found 253.0653 [$M^{+\cdot}$].

5-(Trifluoromethyl)-1-(2-vinylphenyl)-1*H*-indole (9h):

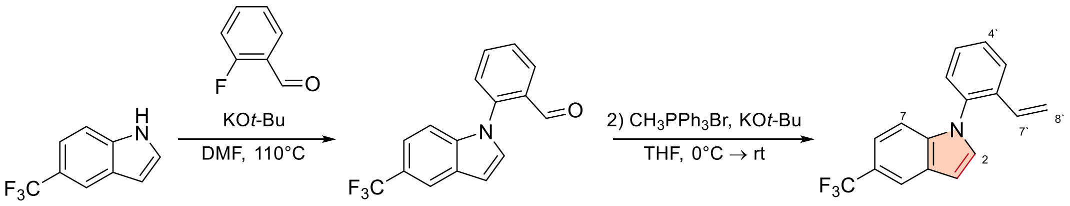

Step 1: A crimp cap vial was charged with 5-trifluoromethyl-1*H*-indole (174 mg, 940 µmol, 1.00 eq.), KO*t*-Bu (158 mg, 1.41 mmol, 1.50 eq.), and 4.7 mL of dry DMF under argon. The obtained mixture was stirred at 110°C while 2-fluorobenzaldehyde (177 mg, 1.43 mmol, 1.52 eq.) was added dropwise over 10 minutes. The reaction mixture was stirred at 110°C for 16 h, then cooled to room temperature, diluted with water, and extracted with EtOAc (3×20 mL). The combined organic layer was washed with brine (3×80 mL), dried over anhydrous Na_2SO_4, filtered and the solvent was removed under reduced pressure. The residue was purified by flash silica gel column chromatography (cyclohexane/EtOAc 100:0→70:30) to give the desired aldehyde **S4h** as a yellow oil (155 mg, 536 µmol, 57%): R_f 0.33 (cyclohexane/EtOAc 5:1).

Step 2:

2-(5-(Trifluoromethyl)-1*H*-indol-1-yl)benzaldehyde **S4h** (153 mg, 530 µmol) was subjected to the step 2 according of the general procedure **A** utilizing methyltriphenylphosphonium-bromide (379 mg, 1.06 mmol, 2.00 eq.) and KO*t*-Bu (137 mg, 2.20 mmol, 2.30 eq.) to give **9h** as a transparent oil (87.0 mg, 303 µmol, 57%): R_f 0.65 (cyclohexane/EtOAc 6:1); ν_{max}(neat): 3093w, 2988w, 2901w, 1627w, 1495m, 1456w, 1341s, 1318m, 1235wm 1165w, 1115s, 1054m, 918w, 810w, 767w, 729w; ^{1}H NMR (500 MHz, $CDCl_3$) δ = 8.07 – 7.89 (1H, m, C4*H*), 7.85 – 7.68 (1H, m, C3\`*H*), 7.51 – 7.45 (1H, m, C4\`*H*), 7.45 – 7.36 (2H, m, C5\`*H*, C6*H*), 7.35 – 7.31 (1H, m, C6\`*H*), 7.29 (1H, d, ^{3}J 3.2 Hz, C2*H*), 7.14 (1H, d, ^{3}J 8.7 Hz, C7*H*), 6.77 (1H, d, ^{3}J 3.2 Hz, C3*H*), 6.22 (1H, dd, ^{3}J 17.5, ^{3}J 11.0 Hz, C7\`*H*), 5.74 (1H, d, $^{3}J$ 17.5 Hz, C8\`*H*), 5.20 (d, ^{3}J 11.0 Hz, C8\`*H*); $^{13}$C NMR (126 MHz, $CDCl_3$) δ = 138.8 (*C7a*), 136.5 (*C1\`*), 135.3 (*C2\`*), 131.8 (*C7\`*), 131.2 (*C2*), 128.90 (*C4\`*), 128.86 (*C5\`*), 128.2 (*C6\`*), 127.8 (*C3a*), 126.6 (*C3\`*), 125.5 (q, $^{1}J_{CF}$ 271.5 Hz, *C*F_3), 122.7 (q, $^{2}J_{CF}$ 32.0 Hz, *C5*), 119.1 (q, $^{3}J_{CF}$ 2.6 Hz, C6), 118.79 (q, $^{3}J_{CF}$ 4.3 Hz, *C4*), 117.0 (*C8\`*), 111.3 (*C7*), 103.8 (*C3*); ^{19}F NMR (376 MHz, $CDCl_3$) δ = –60.31; ESI-MS: m/z calcd. for $C_{17}H_{13}F_3N$ 288.0994 found 288.0987 [$M+H^{+}$].

Methyl 1-(2-vinylphenyl)-1*H*-indole-5-carboxylate (9i):

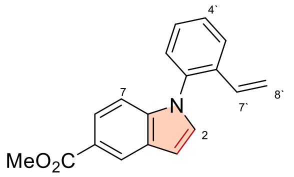

9i

Step 1 of the general procedure **A** was performed with methyl 1*H*-indole-5-carboxylate (149 mg, 850 µmol), 2-iodobenzaldehyde (197 mg, 850 µmol), K_2CO_3 (235 mg, 1.70 mmol) and CuI (16.2 mg, 85.0 µmol) to give methyl 1-(2-formylphenyl)-1*H*-indole-5-carboxylate **S4i** as yellow oil (200 mg, 716 µmol, 84%): R_f 0.33 (cyclohexane/EtOAc 5:1). The obtained aldehyde **S4i** (185 mg, 662 µmol) was subjected to the step 2 utilizing methyltriphenylphosphonium-bromide (331 mg, 927 µmol, 1.40 eq.) and KO*t*-Bu (119 mg, 1.06 mmol, 1.60 eq.) to give **9i** as yellow oil (150 mg, 541 µmol, 82%): R_f 0.61 (cyclohexane/EtOAc 5:1); ν_{max}(neat): 3382w, 2949w, 1709s, 1612m, 1492m, 1445m, 1333m, 1267s, 1229m, 1195m, 1123w, 1086w, 987w, 912w, 753s; ^{1}H NMR (500 MHz, $CDCl_3$) δ = 8.46 (1H, d, ^4J 1.3 Hz, C4*H*), 7.87 (1H, dd, ^3J 8.7, ^4J 1.6 Hz, C6*H*), 7.76 (1H, dd, ^3J 7.8, ^4J 1.3 Hz, C3`*H*), 7.55 – 7.45 (1H, m, C4`*H*), 7.42 (1H, td, ^3J 7.6, ^4J 1.3 Hz, C5`*H*), 7.33 (1H, dd, $^3J$ 7.8, $^4J$ 1.3 Hz, C6`*H*), 7.25 (1H, d, ^3J 3.2 Hz, C2*H*), 7.08 (1H, d, ^3J 8.7 Hz, C7*H*), 6.77 (1H, d, ^3J 3.2 Hz, C3*H*), 6.23 (1H, dd, ^3J 17.5, ^3J 11.0 Hz, C7`*H*), 5.83 – 5.65 (1H, m, C8`*H*), 5.19 (1H, dd, ^3J 11.0, ^2J 0.8 Hz, C8`*H*), 3.94 (3H, s, $CO_2CH_3$); $^{13}$C NMR (126 MHz, $CDCl_3$) δ = 168.2 ($CO_2CH_3$), 139.9 (*C7a*), 136.6 (*C1`*), 135.3 (*C2`*), 131.9 (*C7`*), 130.9 (*C2*), 128.9 (*C5`*), 128.8 (*C4`*), 128.2 (*C6`*), 128.1 (*C3a*), 126.6 (*C3`*), 124.1 (*C4*), 123.7 (*C6*), 122.4 (*C5*), 117.0 (*C8`*), 110.7 (*C7*), 104.3 (*C3*), 52.0 (OCH_3); ESI-MS: m/z calcd. for $C_{88}H_{77}O_8$ 1261.5613 found 1261.5632 [2M+H$^+$];); ESI-MS: m/z calcd. for $C_{18}H_{16}NO_2$ 278.1176 found 278.1177 [M+H$^+$].

1,1'-Bis(2-vinylphenyl)-1*H*,1'H-4,4'-biindole (11):

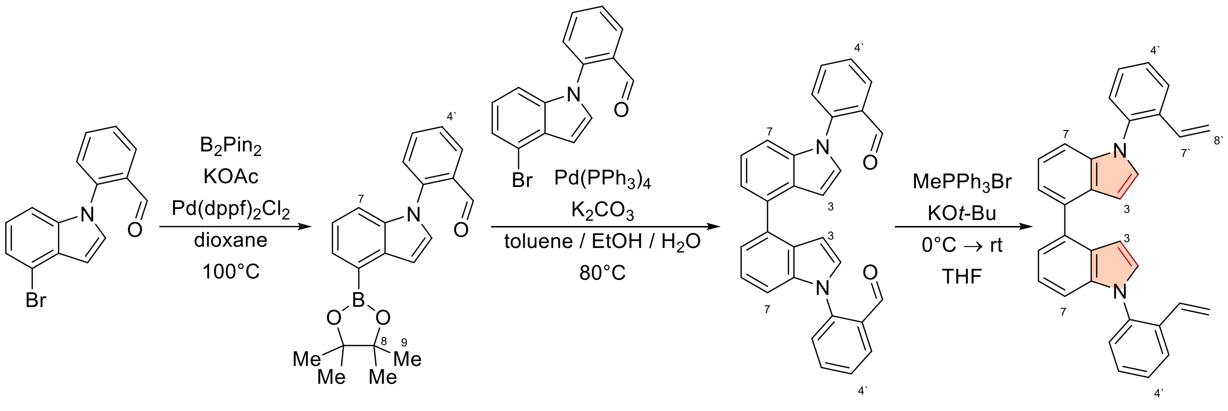

S5 **S6** **11**

Step 1: According to the modified literature procedure:[6] 2-(4-Bromo-1*H*-indol-1-yl)benzaldehyde (540 mg, 1.80 mmol, 1.00 eq.), bis(pinacolato)diboron (549 mg, 2.16 mmol, 1.20eq.), dry KOAc (530 mg, 5.40 mmol, 3.00 eq.), $Pd(dppf)_2Cl_2\cdot CH_2Cl_2$ (132 mg, 180 µmol, 10 mol%) were weighed in a 20 mL crimp cap vial. The vial was sealed, evacuated and backfilled with argon before dioxane (10 mL) was added. The reaction mixture was stirred at 100°C for 18 h under argon, cooled down to room temperature, filtered though a thin silica gel layer (using EtOAc to wash the filter bed). The solvents were removed under reduced pressure and crude product was purified by silica gel column chromatography (cyclohexane/EtOAc 100:0→60:40) to give the desired product **S5** as yellow oil (420 mg, 1.21 mmol, 67%): R_f 0.58 (cyclohexane/EtOAc 5:1); ν_{max}(neat): 3081w, 3058w, 2922w, 2851w, 2751w, 1696s, 1598m, 1513m, 1489m, 1420m, 1268w, 1194w, 994w, 912w, 754s; ^{1}H NMR (500 MHz,

$CDCl_3$) δ = 9.65 (1H, s, C*H*O), 8.10 (1H, dd, ^3J 7.8, ^4J 1.6 Hz, C3`*H*), 7.75 (1H, td, $^3J$ 7.7, $^4J$ 1.6 Hz, C5`*H*), 7.71 (1H, dd, ^3J 6.9, ^4J 0.9 Hz, C5*H*), 7.57 (1H, t, ^3J 7.6 Hz, C4`*H*), 7.48 (1H, dd, $^3J$ 7.7, $^4J$ 1.0 Hz, C6`*H*), 7.34 (1H, d, ^3J 3.2 Hz, C2*H*), 7.32 – 7.27 (2H, m, C3*H*, C7*H*), 7.25 – 7.20 (1H, m, C6*H*), 1.42 (12H, s, 4×C*H*3); ^{13}C NMR (126 MHz, $CDCl_3$) δ = 189.9 (*C*HO), 142.3 (*C1`*), 137.9 (*C7a*), 135.2 (*C5`*), 133.6 (*C3a*), 132.3 (*C2`*), 130.3 (*C2*), 129.0 (*C5*, *C4*), 128.7 (*C3`*), 128.4 (*C4`*), 128.3 (*C6`*), 122.7 (*C6*), 113.1 (*C7*), 106.5 (*C3*), 83.8 (*C8*), 25.2 (*C9*); ESI-MS: m/z calcd. for $C_{21}H_{22}BNNaO_3$ 370.1589 found 370.1591 [M+Na$^+$].

Step 2: 2-(4-Bromo-1*H*-indol-1-yl)benzaldehyde (66.0 mg, 220 µmol, 1.00 eq.), 2-(4-(4,4,5,5-tetramethyl-1,3,2-dioxaborolan-2-yl)-1*H*-indol-1-yl)benzaldehyde (**S5**) (84.8 mg, 244 µmol, 1.11 eq.), $Pd(PPh_3)_4$ (12.7 mg, 11.0 µmol, 5 mol%), and K_2CO_3 (91.2 mg, 660 µmol, 3.00 eq.) were weighed in a 10 mL crimp cap vial. The vial was sealed, evacuated and backfilled with argon before a degassed mixture of toluene, H_2O, ethanol (2 : 2 : 1) (2 mL, 0.11 mol·L^{-1}) was added. The resulting mixture was stirred at 80°C for 18 h, then cooled to room temperature, filtered through a thin layer of silica gel and Na_2SO_4 using EtOAc (15-20 mL). The solvents were removed under reduced pressure and the residue was purified by the silica gel chromatography (cyclohexane/EtOAc 100:0→70:30) to give the desired product 2,2'-(1*H*,1'*H*-[4,4'-biindole]-1,1'-diyl)dibenzaldehyde **S6** as yellow oil (82.0 mg, 186 µmol, 85%): R_f 0.42 (cyclohexane/EtOAc 5:1); ν_{max}(neat): 3061w, 2919w, 2857w, 2755w, 1693s, 1598m, 1487m, 1411m, 1295w, 1184w, 884w, 755; ^{1}H NMR (500 MHz, $CDCl_3$) δ = 9.77 (2H, d, ^4J 0.8 Hz, C*H*O), 8.15 (2H, dd, ^3J 7.8, ^4J 1.4 Hz, C3`*H*), 7.80 (2H, td, $^3J$ 7.6, $^4J$ 1.4 Hz, C5`*H*), 7.66 – 7.55 (4H, m, C4`*H*, C6`*H*), 7.48 (2H, dd, ^3J 7.3, ^4J 0.9 Hz, C5*H*), 7.37 (2H, dd, ^3J 8.2, ^3J 7.3 Hz, C6*H*), 7.33 (2H, d, ^3J 3.3 Hz, C2*H*), 7.27 – 7.22 (2H, m, C7*H*), 6.83 (2H, dd, ^3J 3.3, ^5J 0.9 Hz, C3*H*); ^{13}C NMR (126 MHz, $CDCl_3$) δ = 190.0 (*C*HO), 142.3 (*C1`*), 139.0 (*C7a*), 135.3 (*C5`*), 134.0 (*C4*), 132.3 (*C2`*), 129.8 (*C2*), 128.7 (*C3`*), 128.5 (*C4`*/*C6`*), 128.4 (*C4`*/*C6`*), 127.8 (*C3a*), 123.4 (*C6*), 122.0 (*C5*), 109.4 (*C7*), 104.9 (*C3*); ESI-MS: m/z calcd. for $C_{30}H_{20}KN_2O_2$ 479.1156 found 479.1155 [M+K$^+$].

Step 3: To a suspension of methyltriphenylphosphonium-bromide (264 mg, 740 µmol, 4.00 eq.) in THF (1.9 mL) at 0°C was added dry KO*t*-Bu (95.5 mg, 851 µmol, 4.60 eq.) under argon. After stirring the resulting suspension for 50 min at 0°C, a solution of 2,2'-(1*H*,1'*H*-[4,4'-biindole]-1,1'-diyl)dibenzaldehyde **S6** (81.5 mg, 185 µmol, 1.00 eq.) in THF (1.9 mL) was added dropwise. The reaction mixture was allowed to warm up to room temperature and stirred for 18 h. Water was then added and the mixture was extracted with EtOAc (3×30 mL). The combined organic layer was dried over anhydrous Na_2SO_4, filtered and the solvent was removed under reduced pressure. The residue was purified by flash silica gel column chromatography (cyclohexane/EtOAc 100:0→70:30) to give the desired product 1,1'-bis(2-vinylphenyl)-1*H*,1'*H*-4,4'-biindole **11** as yellow oil (66.0 mg, 151 µmol, 82%): R_f 0.57 (cyclohexane/EtOAc 5:1); ν_{max}(neat):.3062w, 2924w, 2837w, 1836w, 1709w, 1627w, 1597w, 1508w, 1488m, 1293m, 1181m, 1052w, 992w, 909m, 751s; ^{1}H NMR (500 MHz, $CDCl_3$) δ = 7.82 – 7.76 (2H, m, C3`*H*), 7.49 – 7.39 (8H, m, C5*H*, C4`*H*, C5`*H*, C6`*H*), 7.30 (2H, dd, ^3J 8.2, ^3J 7.3 Hz, C6*H*), 7.22 (2H, d, ^3J 3.2 Hz, C2*H*), 7.16 – 7.10 (2H, m, C7*H*), 6.79 (2H, dd, ^3J 3.2, ^5J 0.8 Hz, C3*H*), 6.42 (2H, dd, ^3J 17.5, ^3J 11.0 Hz, C7`*H*), 5.77 (2H, dd, $^3J$ 17.5, $^2J$ 1.0 Hz, C8`*H*), 5.23 (2H, dd, ^3J 11.0, ^2J 1.0 Hz, C8`*H*); $^{13}$C NMR (126 MHz, $CDCl_3$) δ = 138.1 (*C7a*), 137.5 (*C1`*), 135.3 (*C2`*), 133.9 (*C4*), 132.5 (*C7`*), 129.3 (*C2*), 128.7 (*C5`*), 128.5 (*C4`*), 128.3 (*C6`*), 127.6 (*C3a*), 126.4 (*C3`*), 122.4 (*C6*), 121.2 (*C5*), 116.3 (*C8`*), 110.0 (*C7*), 103.5 (*C3*); ESI-MS: m/z calcd. for $C_{32}H_{25}N_2$ 437.2012 found 437.2000 [M+H$^+$].

General Procedure B for the Synthesis of S8a-h:

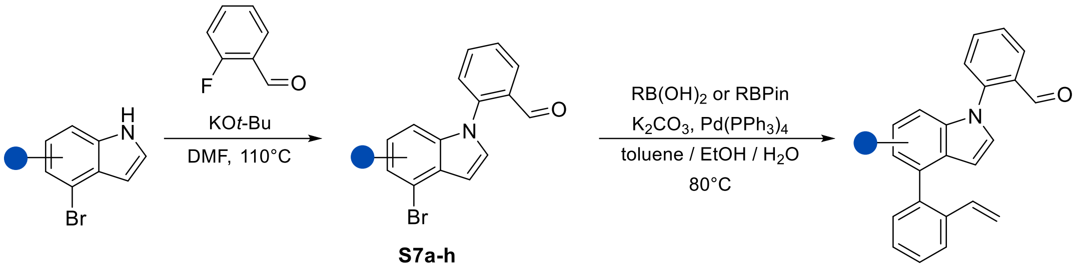

S7a-h

S8a-h

Step 1: A crimp cap vial was charged with the corresponding indole (1.00 eq.), KO*t*-Bu (1.50 eq.), and dry DMF (0.23 mol·L^{-1}) under argon. The obtained mixture was heated to 110°C and 2-fluorobenzaldehyde (1.52 eq.) was added dropwise over 10 minutes. The resulting solution was stirred at 110°C until the reaction was completed (the progress was monitored by ^{1}H-NMR). The mixture was cooled to room temperature, diluted with water, and extracted with EtOAc (×3). The combined organic layer was washed with brine (×3), dried over anhydrous Na_2SO_4, filtered and the solvent was removed under reduced pressure. The residue was purified by flash silica gel column chromatography (cyclohexane/EtOAc 100:0→70:30) to give the desired aldehyde **S7a-h**.

Step 2: Aldehyde **S7a-h** (1.00 eq.), the corresponding boronic acid or ester (1.11 eq.), Pd(PPh$_3$)$_4$ (5 mol%), and K_2CO_3 (3.00 eq.) were weighed into a 10 or 20 mL crimp cap vial. The vial was sealed, evacuated and backfilled with argon before a degassed mixture of toluene, H_2O, ethanol (2 : 2 : 1) (0.12 mol·L^{-1}) was added. The resulting mixture was stirred at 80°C for 18 h, then cooled to room temperature, filtered through a thin layer of silica gel and Na_2SO_4 using EtOAc (15-20 mL). The solvents were removed under reduced pressure and the residue was purified by the silica gel chromatography (cyclohexane/EtOAc 100:0→80:20) to give the desired product **S8a-h**.

2-(4-(2-Vinylphenyl)-1*H*-indol-1-yl)benzaldehyde (S8a):

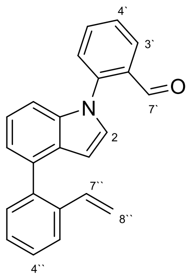

S8a

Step 1 of the general procedure **B** was performed with 4-bromo-1*H*-indole (471 mg, 2.40 mmol), 2-fluorobenzaldehyde (453 mg, 3.65 mmol), KO*t*-Bu (404 mg, 3.60 mmol) over 2 h to give 2-(4-bromo-1*H*-indol-1-yl)benzaldehyde **S7a** as yellow oil (630 mg, 2.10 mmol, 88%): R_f 0.50 (cyclohexane/EtOAc 5:1). The obtained aldehyde **S7a** (163 mg, 543 µmol) was subjected to the step 2 utilizing (2-vinylphenyl)boronic acid (89.2 mg, 603 µmol) to give **S8a** as yellow oil (160 mg, 495 µmol, 91%): R_f 0.57 (cyclohexane/EtOAc 5:1); ν_{max}(neat): 3055w, 2966w, 2851w, 2752w, 1688s, 1595s, 1485s, 1418s, 1262s, 1169s, 1101m, 1020m, 904w, 822w, 751s; ^{1}H NMR (500 MHz, CDCl$_3$) δ = 9.72 (1H, s, C*H*O), 8.13 (1H, dd, ^3J 7.8, ^4J 1.4 Hz, C3`*H*), 7.84 – 7.75 (2H, m, C5`*H*, C3``*H*), 7.63 – 7.59 (1H, m, C4`*H*), 7.56 (1H. d, $^3J$ 7.9 Hz, C6`*H*), 7.46 – 7.36 (3H, m, C4``*H*, C5``*H*, C6``*H*), 7.31 – 7.27 (2H, m, C2*H*, C6*H*), 7.20 (1H, dt, ^3J 8.4, ^4J 0.9 Hz, C7*H*), 7.11 (1H, dd, ^3J 7.1, ^4J 0.9 Hz, C5*H*), 6.67 (1H, dd, ^3J 17.5, ^3J 11.0 Hz, C7``*H*), 6.51 (1H, d, $^3J$ 3.2, C3*H*), 5.75 (1H, dd, $^3J$ 17.5, $^2J$ 1.2 Hz, C8``*H*), 5.13 (1H, dd, ^3J 11.0, ^2J 1.2 Hz, C8``*H*); $^{13}$C NMR (126 MHz, CDCl$_3$) δ = 189.9 (*C7*`), 142.3 (*C2*`), 139.5 (*C1*``), 138.4 (*C7a*), 136.2 (*C2*``), 136.0 (*C7*``), 135.3 (*C5*`), 134.1 (*C4*), 132.2 (*C1*`), 130.8 (*C6*``), 129.9 (*C2*), 128.7 (*C3*`), 128.49 (*C4*`), 128.47 (*C3a*), 128.35 (*C6*`), 127.7 (*C4*``), 127.6 (*C5*``), 125.4 (*C3*``), 123.1

(*C6*), 122.7 (*C5*), 114.3 (*C8``*), 109.3 (*C7*), 104.5 (*C3*); ESI-MS: m/z calcd. for $C_{23}H_{17}AgNO$ 430.0356 found 430.0348 [M+Ag$^+$].

2-(4-(5-Chloro-2-vinylphenyl)-1*H*-indol-1-yl)benzaldehyde (S8b):

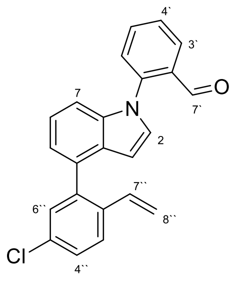

S8b

Step 1 of the general procedure **B** was performed with 4-bromo-1*H*-indole (471 mg, 2.40 mmol), 2-fluorobenzaldehyde (453 mg, 3.65 mmol), KO*t*-Bu (404 mg, 3.60 mmol) over 2 h to give 2-(4-bromo-1*H*-indol-1-yl)benzaldehyde **S7b** as yellow oil (630 mg, 2.10 mmol, 88%): R_f 0.50 (cyclohexane/EtOAc 5:1). The obtained aldehyde **S7b** (137 mg, 455 µmol) was subjected to the step 2 utilizing 2-(5-chloro-2-vinylphenyl)-4,4,5,5-tetramethyl-1,3,2-dioxaborolane (134 mg, 505 µmol) to give **S8b** as yellow oil (150 mg, 419 µmol, 92%): R_f 0.48 (cyclohexane/EtOAc 5:1); ν_{max}(neat): 3064w, 2855w, 2760w, 1695s, 1598s, 1512m, 1489s, 1395m, 1299w, 1193w, 1109w, 1058w, 913m, 825m, 757s; ^{1}H NMR (500 MHz, $CDCl_3$) δ = 9.70 (1H, s, C*H*O), 8.13 (1H, dd, ^3J 7.8, ^4J 1.4 Hz, C3`*H*), 7.86 – 7.74 (1H, m, C5`*H*), 7.68 (1H, d, ^3J 8.5 Hz, C3``*H*), 7.61 (1H, tt, $^3J$ 7.8, $^4J$ 1.0 Hz, C4`*H*), 7.55 (1H, d, $^3J$ 7.8 Hz, C6`*H*), 7.44 (1H, d, $^4J$ 2.2 Hz, C6``*H*), 7.38 (1H, ddd, ^3J 8.4, ^4J 2.3, ^5J 0.6 Hz, C4``*H*), 7.30 (1H, d, $^3J$ 2.9 Hz, C2*H*), 7.27 (1H, dd, $^3J$ 8.3, $^3J$ 7.1 Hz, C6*H*), 7.20 (1H, dt, $^3J$ 8.3, $^4J$ 0.9 Hz, C7*H*), 7.08 (1H, dd, $^3J$ 7.1, $^4J$ 0.9 Hz, C5*H*), 6.59 (1H, dd, $^3J$ 17.5, $^3J$ 11.0 Hz, C7``*H*), 6.50 (1H, dd, ^3J 2.9, ^5J 0.9 Hz, C3*H*), 5.72 (1H, dd, ^3J 17.5, ^2J 1.1 Hz, C8``*H*), 5.15 (1H, dd, $^3J$ 11.0, $^2J$ 1.1 Hz, C8``*H*); ^{13}C NMR (126 MHz, $CDCl_3$) δ = 189.8 (*C*HO), 142.1 (*C1`*), 141.0 (*C1``*), 138.4 (*C7a*), 135.3 (*C5`*), 134.9 (*C7``*), 134.8 (*C2``*), 133.2 (*C5``*), 132.6 (*C4*), 132.3 (*C2`*), 130.6 (*C6``*), 130.2 (*C2*), 128.8 (*C3`*), 128.6 (*C4`*), 128.4 (*C6`*), 128.2 (*C7a*), 127.8 (*C4``*), 126.8 (*C3``*), 123.1 (*C6*), 122.5 (*C5*), 114.8 (*C8``*), 109.8 (*C7*), 104.2 (*C3*); ESI-MS: m/z calcd. for $C_{23}H_{16}AgClNO$ 463.9966 found 463.9965 [M+Ag$^+$].

2-(4-(5-Methoxy-2-vinylphenyl)-1*H*-indol-1-yl)benzaldehyde (S8c):

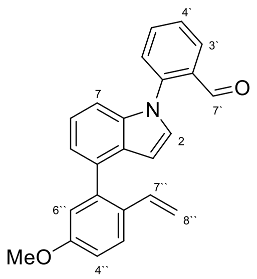

S8c

Step 1 of the general procedure **B** was performed with 4-bromo-1*H*-indole (471 mg, 2.40 mmol), 2-fluorobenzaldehyde (453 mg, 3.65 mmol), KO*t*-Bu (404 mg, 3.60 mmol) over 2 h to give 2-(4-bromo-1*H*-indol-1-yl)benzaldehyde **S7c** as yellow oil (630 mg, 2.10 mmol, 88%): R_f 0.50 (cyclohexane/EtOAc 5:1). The obtained aldehyde **S7c** (100 mg, 334 µmol) was subjected to the step 2 utilizing 2-(5-methoxy-2-vinylphenyl)-4,4,5,5-tetramethyl-1,3,2-dioxaborolane (96.4 mg, 371 µmol) to give **S8c** as yellow oil (100 mg, 283 µmol, 85%): R_f 0.28 (cyclohexane/EtOAc 5:1); ν_{max}(neat): 3060w, 2958w, 2853w, 2758w, 1695s, 1600s, 1516m, 1487s, 1423m, 1296m, 1229m, 1166m, 1043w, 908m, 758m, 732m; ^{1}H NMR (500 MHz, $CDCl_3$) δ = 9.72 (1H, s, C*H*O), 8.13 (1H, dd, ^3J 7.8, ^4J 1.5 Hz, C3`*H*), 7.78 (1H, td, $^3J$ 7.7, $^4J$ 1.5 Hz, C5`*H*), 7.75 – 7.68 (1H, m, C3``*H*), 7.60 (1H, t, $^3J$ 7.6 Hz, C4`*H*), 7.55 (1H, d, $^3J$ 7.9 Hz, C6`*H*), 7.31 – 7.26 (2H, m, C2*H*, C6*H*), 7.20 (1H, d, $^3J$ 8.3 Hz, C7*H*), 7.17 – 7.07 (1H, m, C5*H*), 7.00 – 6.94 (2H, m, C4``*H*, C6``*H*), 6.58 (1H, dd, $^3J$ 17.5, $^3J$ 11.0 Hz, C7``*H*),

6.53 (1H, d, ^3J 3.2 Hz, C3*H*), 5.62 (1H, dd, ^3J 17.5, ^2J 1.1 Hz, C8``*H*), 5.01 (1H, dd, $^3J$ 11.0, $^2J$ 1.1 Hz, C8``*H*), 3.85 (3H, s, OCH_3); ^{13}C NMR (126 MHz, $CDCl_3$) δ = 189.9 (*C*HO), 159.1 (*C5``*), 142.2 (*C1`*), 140.8 (*C1``*), 138.4 (*C7a*), 135.32 (*C5`*), 135.29 (*C7``*), 134.1 (*C4*), 132.2 (*C2`*), 130.0 (*C2*), 129.1 (*C2``*), 128.7 (*C3`*), 128.5 (*C4`*), 128.38 (*C3a*), 128.35 (*C6`*), 126.6 (*C3``*), 123.1 (*C6*), 122.5 (*C5*), 115.2 (*C6``*), 114.2 (*C4``*), 112.2 (*C8``*), 109.4 (*C7*), 104.5 (*C3*), 55.6 (OCH_3); ESI-MS: m/z calcd. for $C_{24}H_{19}KNO_2$ 392.1047 found 392.1048 [M+K$^+$].

2-(6-Chloro-4-(2-vinylphenyl)-1*H*-indol-1-yl)benzaldehyde (S8d):

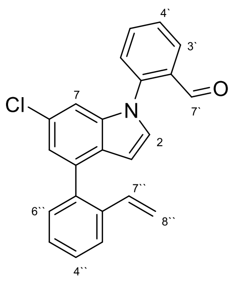

S8d

Step 1 of the general procedure **B** was performed with 4-bromo-6-chloro-1*H*-indole (237 mg, 1.03 mmol), 2-fluorobenzaldehyde (194 mg, 1.56 mmol), KO*t*-Bu (173 mg, 1.54 mmol) over 18 h to give 2-(4-bromo-6-chloro-1*H*-indol-1-yl)benzaldehyde **S7d** as yellow oil (182 mg, 544 µmol, 53%): R_f 0.38 (cyclohexane/EtOAc 5:1). The obtained aldehyde **S7d** (182 mg, 544 µmol) was subjected to the step 2 utilizing (2-vinylphenyl)boronic acid (89.3 mg, 604 µmol) to give **S8d** as yellow oil (192 mg, 537 µmol, 99%): R_f 0.39 (cyclohexane/EtOAc 5:1); ν_{max}(neat): 3066w, 2843w, 2752w, 1691s, 1597s, 1507m, 1460m, 1412s, 1126w, 992w, 911s; ^{1}H NMR (500 MHz, $CDCl_3$) δ = 9.69 (1H, s, C*H*O), 8.13 (1H, dd, ^3J 7.8, ^4J 1.4 Hz, C3`*H*), 7.82 – 7.78 (1H, m, C5`*H*), 7.77 – 7.74 (1H, m, C3``*H*), 7.67 – 7.60 (1H, m, C4`*H*), 7.54 (1H, d, $^3J$ 7.8 Hz, C6`*H*), 7.45 – 7.35 (3H, m C4``*H*, C5``*H*, C6``*H*), 7.25 (1H, d, ^3J 3.3 Hz, C2*H*), 7.17 (1H, dd, ^4J 1.7, ^5J 0.9 Hz, C7*H*), 7.11 (1H, d, ^4J 1.7 Hz, C5*H*), 6.63 (1H, dd, ^3J 17.5, ^3J 11.0 Hz, C7``*H*), 6.46 (1H, dd, $^4J$ 3.3, $^5J$ 0.9 Hz, C3*H*), 5.77 (1H, dd, $^3J$ 17.5, $^2J$ 1.2 Hz, C8``*H*), 5.17 (1H, dd, ^3J 11.0, ^2J 1.2 Hz, C8``*H*); $^{13}$C NMR (126 MHz, $CDCl_3$) δ = 189.5 (*C*HO), 141.5 (*C1`*), 138.6 (*C7a*), 138.2 (*C1``*), 136.2 (*C2``*), 135.5 (*C7``*), 135.4 (*C5`*), 135.3 (*C6*), 132.3 (*C2`*), 130.62 (*C6``*), 130.60 (*C2*), 129.03 (*C3`*), 129.01 (*C4*), 128.99 (*C4`*), 128.4 (*C6`*), 128.2 (*C4``*), 127.8 (*C5``*), 127.1 (*C3a*), 125.5 (*C3``*), 123.0 (*C6*), 114.9 (*C8``*), 109.3 (*C7*), 104.6 (*C3*); ESI-MS: m/z calcd. for $C_{23}H_{16}AgClNO$ 463.9966 found 463.9966 [M+Ag$^+$].

2-(6-(Trifluoromethyl)-4-(2-vinylphenyl)-1*H*-indol-1-yl)benzaldehyde (S8e):

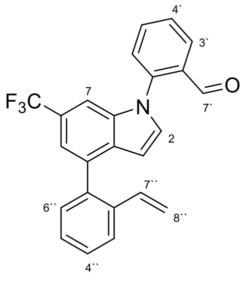

S8e

Step 1 of the general procedure **B** was performed with 4-bromo-6-(trifluoromethyl)-1*H*-indole (248 mg, 940 µmol), 2-fluorobenzaldehyde (177 mg, 1.43 mmol), KO*t*-Bu (158 mg, 1.41 mmol) over 18 h to give 2-(4-bromo-6-(trifluoromethyl)-1*H*-indol-1-yl)benzaldehyde **S7e** as yellow oil (176 mg, 478 µmol, 51%): R_f 0.30 (cyclohexane/EtOAc 5:1). The obtained aldehyde **S7e** (176 mg, 478 µmol) was subjected to the step 2 utilizing (2-vinylphenyl)boronic acid (78.5 mg, 531 µmol) to give **S8e** as yellow oil (156 mg, 399 µmol, 83%): R_f 0.36 (cyclohexane/EtOAc 5:1); ν_{max}(neat): 3061w, 2927w, 2852w, 2756w, 1696m, 1598m, 1505m, 1420m, 1368m, 1306m, 1253m, 1174s, 1114s,

992w, 917m, 865w, 765s; ^{1}H NMR (500 MHz, $CDCl_3$) δ = 9.67 (1H, s, C*H*O), 8.16 (1H, dd, ^{3}J 7.8, ^{4}J 1.4 Hz, C3`*H*), 7.83 (1H, td, $^{3}J$ 7.7, $^{4}J$ 1.4 Hz, C5`*H*), 7.80 – 7.76 (1H, m, C3``*H*), 7.71 – 7.63 (1H, m, C4`*H*), 7.57 (1H, d, $^{3}J$ 7.9 Hz, C6`*H*), 7.49 – 7.32 (6H, m, C2*H*, C4``*H*, C5``*H*, C6``*H*, C5*H*, C7*H*), 6.59 (1H, dd, ^{3}J 17.5, ^{3}J 11.0 Hz, C7``*H*), 6.55 (1H, dd, $^{3}J$ 3.2, $^{5}J$ 0.8 Hz, C3*H*), 5.76 (1H, dd, $^{3}J$ 17.5, $^{2}J$ 1.1 Hz, C8``*H*), 5.16 (1H, dd, ^{3}J 11.0, ^{2}J 1.1 Hz, C8``*H*); $^{13}$C NMR (126 MHz, $CDCl_3$) δ = 189.2 (*C*HO), 141.2 (*C1`*), 138.2 (*C1``*), 137.4 (*C7a*), 136.3 (*C2``*), 135.5 (*C5`*), 135.4 (*C7``*), 134.9 (*C4*), 132.6 (*C2`*), 132.4 (*C2*), 130.8 (*C7a*), 130.7 (*C6``*), 129.3 (*C4`*, *C3`*), 128.5 (*C6`*), 128.3 (*C4``*), 127.8 (*C5``*), 125.6 (*C3``*), 119.1 (q, $^{3}J_{CF}$ 3.2 Hz, *C5*), 115.1 (*C8``*), 106.7 (q, $^{3}J_{CF}$ 3.9 Hz, *C7*), 104.6 (*C3*); ^{19}F NMR (376 MHz, $CDCl_3$) δ –60.63; ESI-MS: m/z calcd. for $C_{24}H_{16}AgF_3NO$ 498.0229 found 498.0224 [M+Ag^{+}].

2-(7-Fluoro-4-(2-vinylphenyl)-1*H*-indol-1-yl)benzaldehyde (S8f):

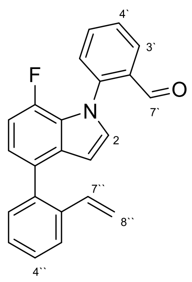

S8f

Step 1 of the general procedure **B** was performed with 4-bromo-7-fluoro-1*H*-indole (161 mg, 750 µmol), 2-fluorobenzaldehyde (141 mg, 1.14 mmol), KO*t*-Bu (126 mg, 1.13 mmol) over 3 h to give 2-(4-bromo-7-fluoro-1*H*-indol-1-yl)benzaldehyde **S7f** as yellow solid (143 mg, 449 µmol, 60%): R_f 0.45 (cyclohexane/EtOAc 5:1). The obtained aldehyde **S7f** (127 mg, 400 µmol) was subjected to the step 2 utilizing (2-vinylphenyl)boronic acid (65.7 mg, 444 µmol) to give **S8f** as yellow oil (130 mg, 381 µmol, 95%): R_f 0.45 (cyclohexane/EtOAc 5:1); ν_{max}(neat): 3046w, 2851w, 1691m, 1597m, 1500m, 1404w, 1239w, 1191w, 1105w, 994w, 905w, 821w, 769w; ^{1}H NMR (500 MHz, $CDCl_3$) δ = 9.82 (1H, s, C*H*O), 8.08 (1H, dd, ^{3}J 7.8, ^{4}J 1.5 Hz, C3`*H*), 7.79 – 7.67 (2H, m, C5`*H*, C3``*H*), 7.65 – 7.58 (1H, m, C4`*H*), 7.53 (1H, d, $^{3}J$ 7.6 Hz, C6`*H*), 7.49 – 7.32 (3H, m, C4``*H*, C5``*H*, C6``*H*), 7.19 (1H, d, ^{3}J 3.2 Hz, C2*H*), 7.03 – 6.91 (2H, m, C5*H*, C6*H*), 6.63 (1H, dd, ^{3}J 17.5, ^{3}J 11.0 Hz, C7``*H*), 6.49 (1H, d, $^{3}J$ 3.2, C3*H*), 5.74 (1H, dd, $^{3}J$ 17.5, $^{2}J$ 1.2 Hz, C8``*H*), 5.14 (1H, dd, ^{3}J 11.0, ^{2}J 1.2 Hz, C8``*H*); $^{13}$C NMR (126 MHz, $CDCl_3$) δ = 189.5 (*C*HO), 149.41 (d, $^{1}J_{CF}$ 246.6 Hz, *C7*), 142.8 (*C1`*), 136.4 (*C2``*), 135.8 (*C7``*), 134.5 (*C5`*), 132.5 (*C2`*), 132.2 ($^{3}J_{CF}$ 3.8 Hz, *C3a*), 138.8 (*C1``*), 131.5 (*C2*), 130.9 (*C6``*), 130.1 (*C4*), 128.7 (*C4`*, *C6`*), 128.3 (*C3`*), 127.8 (*C4``*), 127.7 (*C5``*), 125.6 (d, $^{2}J_{CF}$ 7.7 Hz, *C7a*), 125.4 (*C3``*), 122.7 (d, $^{3}J_{CF}$ 6.3 Hz, *C5*), 114.5 (*C8``*), 108.6 (d, $^{2}J_{CF}$ 17.9 Hz, *C6*), 105.3 (*C3*); ESI-MS: m/z calcd. for $C_{23}H_{17}FNO$ 342.1289 found 342.1286 [M+H^{+}].

2-(3-Methyl-4-(2-vinylphenyl)-1*H*-indol-1-yl)benzaldehyde (S8g):

Step 1 of the general procedure **B** was performed with 4-bromo-3-methyl-1*H*-indole (237 mg, 1.13 mmol), 2-fluorobenzaldehyde (213 mg, 1.71 mmol), KO*t*-Bu (190 mg, 1.69 mmol) over 18 h to give 2-(4-bromo-3-methyl-1*H*-indol-1-yl)benzaldehyde **S7g** as yellow oil (223 mg, 710 µmol, 63%): R_f 0.48 (cyclohexane/EtOAc 5:1). The obtained aldehyde **S7g** (360 mg, 1.15 mmol) was subjected to the step

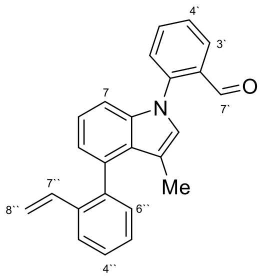

S8g

2 utilizing (2-vinylphenyl)boronic acid (188 mg, 1.27 mmol) to give **S8g** as yellow solid (95.0 mg, 282 µmol, 25%, m.p. 70.0 – 70.6°C) after NP-prep-HPLC (from hexane/EtOAc 98:2 to 80:20, 18 mL/min, 40°C): R_f 0.60 (cyclohexane/EtOAc 5:1); ν_{max}(neat): 3058w, 2943w, 2922w, 2857w, 2750w, 1829w, 1692s, 1598s, 1488s, 1457m, 1422s, 1300w, 1263w, 1160w, 1096w, 1049w, 1019w, 909m, 752s; ^{1}H NMR (500 MHz, $CDCl_3$) δ = 9.74 (1H, s, C*H*O), 8.11 (1H, dd, ^3J 7.8, ^4J 1.5 Hz, C3`*H*), 7.75 (1H, td, $^3J$ 7.7, $^4J$ 1.5 Hz, C5`*H*), 7.71 (1H, d, ^3J 7.7 Hz, C3``*H*), 7.60 – 7.54 (1H, m, C4`*H*), 7.53 (1H, d, $^3J$ 7.7 Hz, C6`*H*), 7.44 – 7.38 (1H, m, C4``*H*), 7.36 – 7.32 (2H, m, C5``*H*, C6``*H*), 7.22 (1H, dd, ^3J 8.3, ^3J 6.9 Hz, C6*H*), 7.17 (1H, dd, ^3J 8.3, ^4J 1.2 Hz, C7*H*), 7.02 (1H, q, ^4J 1.1 Hz, C2*H*), 6.97 (1H, dd, ^3J 6.9, ^4J 1.2 Hz, C5*H*), 6.51 (1H, dd, ^3J 17.4, ^3J 11.0 Hz, C7``*H*), 5.70 (1H, dd, $^3J$ 17.6, $^2J$ 1.2 Hz, C8``*H*), 5.08 (1H, dd, ^3J 11.0, ^2J 1.2 Hz, C8``*H*), 1.74 (3H, d, $^4J$ 1.1 Hz, C3–C$H_3$); $^{13}$C NMR (126 MHz, $CDCl_3$) δ = 190.2 (*C*HO), 142.5 (*C1`*), 140.0 (*C1``*), 139.0 (*C7a*), 136.8 (*C2``*), 135.6 (*C7``*), 135.2 (*C5`*), 134.7 (*C4*), 132.1 (*C2`*), 130.8 (*C6``*), 128.6 (*C3`*), 128.4 (*C2*), 128.2 (*C6`*), 128.1 (*C4`*), 127.8 (*C4``*), 127.3 (*C3a*), 127.0 (*C5``*), 124.3 (*C3``*), 122.8 (*C6*), 122.2 (*C5*), 114.6 (*C8``*), 114.3 (*C3*), 109.3 (*C7*), 11.4 (C3–*C*H_3); ESI-MS: m/z calcd. for $C_{24}H_{19}AgNO$ 444.0512 found 444.0507 [M+Ag$^+$].

2-(4-(2-Vinylphenyl)-1*H*-pyrrolo[2,3-c]pyridin-1-yl)benzaldehyde (S8h):

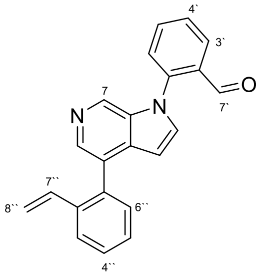

S8h

Step 1 of the general procedure **B** was performed with 4-bromo-1*H*-pyrrolo[2,3-c]pyridine (256 mg, 1.30 mmol), 2-fluorobenzaldehyde (245 mg, 1.98 mmol), KO*t*-Bu (219 mg, 1.95 mmol) over 18 h to give 2-(4-bromo-1*H*-pyrrolo[2,3-c]pyridin-1-yl)benzaldehyde **S7h** as yellow oil (260 mg, 863 µmol, 66%). The obtained aldehyde **S7h** (102 mg, 340 µmol) was subjected to the step 2 utilizing (2-vinylphenyl)boronic acid (55.8 mg, 377 µmol) to give **S8h** as yellow oil (96.0 mg, 296 µmol, 87%): R_f 0.41 (cyclohexane/EtOAc 1:1); ν_{max}(neat): 3012w, 2853w, 2751w, 1696m, 1598m, 1500m, 1425m, 1308w, 1262w, 1218w, 1172w, 1080w, 995w, 877w, 751s; ^{1}H NMR (500 MHz, $CDCl_3$) δ = 9.75 (1H, s, C*H*O), 8.61 (1H, s, C7*H*), 8.29 (1H, s, C5*H*), 8.16 (1H, dd, ^3J 7.8, ^4J 1.6 Hz, C3`*H*), 7.83 (1H, td, $^3J$ 7.7, $^4J$ 1.6 Hz, C5`*H*), 7.78 (1H, d, ^3J 7.8 Hz, C3``*H*), 7.68 (1H, t, $^3J$ 7.8 Hz, C4`*H*), 7.60 (1H, d, $^3J$ 7.7 Hz, C6`*H*), 7.49 – 7.38 (4H, m, C2*H*, C4``*H*, C5``*H*, C6``*H*), 6.64 (1H, dd, ^3J 17.5, ^3J 11.0 Hz, C7``*H*), 6.58 – 6.56 (1H, m, C3*H*), 5.77 (1H, dd, $^3J$ 17.5, $^2J$ 1.0 Hz, C8``*H*), 5.18 (1H, dd, ^3J 11.0, ^2J 1.0 Hz, C8``*H*); $^{13}$C NMR (126 MHz, $CDCl_3$) δ = 189.0 (*C*HO), 140.8 (*C1`*), 136.7 (*C2``*), 136.1 (*C1``*), 135.48 (*C7``*), 135.45 (*C5`*), 135.0 (*C7a*), 133.6 (*C3a*), 133.4 (*C2*), 132.6 (*C7*), 132.2 (*C2`*), 130.9 (*C6``*), 129.5 (*C3`*), 129.3 (*C4`*), 128.8 (*C4*), 128.4 (*C6`*), 128.3 (*C4``*), 127.9 (*C5``*), 125.7 (*C3``*), 115.2 (*C8``*), 103.9 (*C3*); ESI-MS: m/z calcd. for $C_{22}H_{17}N_2O$ 325.1335 found 325.1341 [M+H$^+$].

2-(2-Methyl-4-(2-vinylphenyl)-1*H*-indol-1-yl)benzaldehyde (S8i):

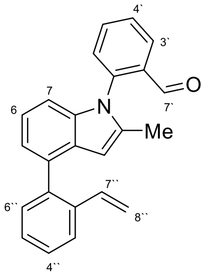

S8i

Step 1 of the general procedure **B** was performed with 4-bromo-2-methyl-1*H*-indole (290 mg, 1.38 mmol), 2-fluorobenzaldehyde (260 mg, 2.10 mmol), KO*t*-Bu (232 mg, 2.07 mmol) over 18 h to give 2-(4-bromo-2-methyl-1*H*-indol-1-yl)benzaldehyde **S7i** as yellow oil (230 mg, 732 µmol, 53%): R_f 0.50 (cyclohexane/EtOAc 5:1). The obtained aldehyde **S7i** (229 mg, 730 µmol) was subjected to the step 2 utilizing (2-vinylphenyl)boronic acid (120 mg, 810 µmol) to give **S8i** as yellow oil (211 mg, 625 µmol, 86%): R_f 0.50 (cyclohexane/EtOAc 5:1); ν_{max}(neat): 3058w, 2920w, 2852w, 2752w, 1695s, 1598m, 1555w, 1488m, 1422m, 1321w, 1266m, 1233w, 1193w, 1162w, 1085w, 1054w, 998^{1}H NMR (500 MHz, $CDCl_3$) δ = 9.47 (1H, s, C*H*O), 8.15 (1H, dd, ^3J 7.8, ^4J 1.4 Hz, C3`*H*), 7.81 (1H, td, $^3J$ 7.6, $^4J$ 1.7 Hz, C5`*H*), 7.76 (1H, dd, ^3J 7.6, ^4J 1.6 Hz, C3``*H*), 7.70 – 7.61 (1H, m, C4`*H*), 7.46 (1H, d, $^3J$ 7.8 Hz, C6`*H*), 7.45 – 7.34 (3H, m, C4``*H*, C5``*H*, C6``*H*), 7.14 (1H, dd, ^3J 8.1, ^3J 7.3 Hz, C6*H*), 7.04 (1H, dd, ^3J 7.3, ^4J 0.9 Hz, C5*H*), 6.89 (1H, dt, ^3J 8.1, ^4J 0.9 Hz, C7*H*), 6.69 (1H, dd, ^3J 17.5, ^3J 11.0 Hz, C7``*H*), 6.30 – 6.23 (1H, m, C3*H*), 5.74 (1H, dd, $^3J$ 17.5, $^2J$ 1.3 Hz, C8``*H*), 5.13 (1H, dd, ^3J 11.0, ^2J 1.3 Hz, C8``*H*), 2.20 (3H, d, $^4J$ 1.0 Hz, C2–C$H_3$); $^{13}$C NMR (126 MHz, $CDCl_3$) δ = 190.0 (*C*HO), 140.9 (*C1`*), 139.9 (*C7a*), 139.8 (*C1``*), 138.0 (*C2*), 136.2 (*C2``*), 136.0 (*C7``*), 135.4 (*C5`*), 134.0 (*C2`*), 132.7 (*C4*), 130.8 (*C6``*), 130.2 (*C6`*), 129.3 (*C4`*), 128.6 (*C3`*), 127.9 (*C3a*), 127.58 (*C5``*), 127.55 (*C4``*), 125.3 (*C3``*), 122.5 (*C5*), 121.8 (*C6*), 114.1 (*C8``*), 109.0 (*C7*), 102.3 (*C3*); 13.3 (*C*H_3); ESI-MS: m/z calcd. for $C_{24}H_{20}NO$ 338.1539 found 338.1538 [M+H$^+$].

2-(7-Methyl-4-(2-vinylphenyl)-1*H*-indol-1-yl)benzaldehyde (S8j):

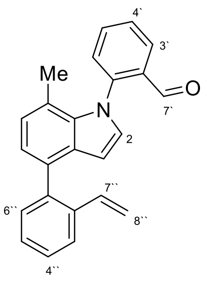

S8j

Step 1 of the general procedure **B** was performed with 4-bromo-7-methyl-1*H*-indole (158 mg, 750 µmol), 2-fluorobenzaldehyde (141 mg, 1.14 mmol), KO*t*-Bu (126 mg, 1.13 mmol) over 18 h to give 2-(4-bromo-7-methyl-1*H*-indol-1-yl)benzaldehyde **S7j** as yellow oil (75.0 mg, 239 µmol, 32%): R_f 0.40 (cyclohexane/EtOAc 5:1). The obtained aldehyde **S7j** (67.5 mg, 215 µmol) was subjected to the step 2 utilizing (2-vinylphenyl)boronic acid (35.3 mg, 239 µmol) to give **S8j** as yellow oil (68.0 mg, 202 µmol, 94%): R_f 0.51 (cyclohexane/EtOAc 5:1); ν_{max}(neat): 3059w, 3021w, 2974w, 2922w, 2858w, 2752w, 1696s, 1597s, 1486m, 1457m, 1401m, 1268w, 1191w, 1151w, 1110w, 1039w, 994w, 903w, 821m, 772s; ^{1}H NMR (500 MHz, $CDCl_3$) δ 9.52 (1H, s, C*H*O), 8.06 (1H, dd, ^3J 7.8, ^4J 1.5 Hz, C3`*H*), 7.79 – 7.70 (2H, m, C3``*H*, C5`*H*), 7.62 (1H, t, $^3J$ 7.6 Hz, C4`*H*), 7.54 (1H, d, $^3J$ 7.9 Hz, C6`*H*), 7.46 – 7.34 (3H, m, C4``*H*, C5``*H*, C6``*H*), 7.13 (1H, d, ^3J 3.3 Hz, C2*H*), 7.00 (2H, s, C5*H*, C6*H*), 6.67 (1H, dd, ^3J 17.5, ^3J 11.0 Hz, C7``*H*), 6.45 (1H, d, $^3J$ 3.3 Hz, C3*H*), 5.73 (1H, dd, $^3J$ 17.5, $^2J$ 1.2 Hz, C8``*H*), 5.12 (1H, dd, ^3J 11.0, ^2J 1.2 Hz, C8``*H*), 1.96 (3H, s, C7–C$H_3$); $^{13}$C NMR (126 MHz, $CDCl_3$) δ = 189.9 (*C*HO), 144.3 (*C1`*), 139.7 (*C1``*), 137.2 (*C7a*), 136.3 (*C2``*), 136.1 (*C7``*), 134.4 (*C5`*), 134.2 (*C2`*), 132.2 (*C4*), 131.3 (*C2*), 130.9 (*C6``*), 130.0 (*C6`*), 129.2 (*C4`*), 128.9 (*C3a*), 127.60 (*C3`*/*C4``*), 127.56 (*C3`*/*C4``*),

127.54 (*C5``*), 125.4 (*C6*), 125.3 (*C3``*), 122.6 (*C5*), 120.5 (*C7*), 114.1 (*C8``*), 104.2 (*C3*), 19.2 (C7–*C*H3); ESI-MS: m/z calcd. for $C_{24}H_{20}NO$ 338.1539 found 338.1531 [M+H$^+$].

2-(7-Chloro-4-(2-vinylphenyl)-1*H*-indol-1-yl)benzaldehyde (S8k):

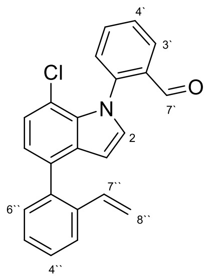

S8k

Step 1 of the general procedure **B** was performed with 4-bromo-7-chloro-1*H*-indole (254 mg, 1.10 mmol), 2-fluorobenzaldehyde (208 mg, 1.67 mmol), KO*t*-Bu (185 mg, 1.65 mmol) over 18 h to give 2-(4-bromo-7-chloro-1*H*-indol-1-yl)benzaldehyde **S7k** (110 mg, 329 µmol, 30%): R$_f$ 0.57 (cyclohexane/EtOAc 5:1). The obtained aldehyde **S7k** (136 mg, 406 µmol) was subjected to the step 2 utilizing utilizing (2-vinylphenyl)boronic acid (66.8 mg, 451 µmol) to give **S8k** as yellow oil (120 mg, 335 µmol, 83%): R$_f$ 0.44 (cyclohexane/EtOAc 5:1); ν_{max}(neat): 2964w, 2924w, 2855w, 2753w, 1696s, 1598m, 1492m, 1399m, 1336w, 1261w, 1193w, 1164m, 1101m, 1038w, 822w; ^{1}H NMR (500 MHz, CDCl$_3$) δ = 9.60 (1H, s, C*H*O), 8.05 (1H, dd, ^3J 7.7, ^4J 1.5 Hz, C3`*H*), 7.85 – 7.67 (2H, m, C5`*H*, C3``*H*), 7.63 (1H, t, $^3J$ 7.7 Hz, C4`*H*), 7.52 (1H, d, $^3J$ 7.1 Hz, C6`*H*), 7.22 (1H, d, $^3J$ 7.8 Hz, C6*H*), 7.17 (1H, d, $^3J$3.2 Hz, C2*H*), 7.01 (1H, d, $^3J$ 7.8 Hz, C5*H*), 6.62 (1H, dd, $^3J$ 17.5, $^3J$ 11.0 Hz, C7``*H*), 6.47 (1H, d, ^3J 3.2 Hz, C3*H*), 5.74 (1H, dd, ^3J 17.5, ^2J 1.1 Hz, C8``*H*), 5.15 (1H, d, $^3J$ 11.0 Hz, C8``*H*); ^{13}C NMR (126 MHz, CDCl$_3$) δ = 189.5 (*C*HO), 142.8 (*C1`*), 138.6 (*C1``*), 136.1 (*C2``*), 135.7 (*C7``*), 134.2 (*C5`*), 134.0 (*C2`*), 133.1 (*C7a,*), 132.2 (*C2*), 130.8 (*C4*, *C6``*), 130.1 (*C6`*), 129.3 (*C4`*), 128.0 (*C4``*), 127.7 (*C5``*), 127.6 (*C3`*), 125.5 (*C3``*), 124.1 (*C6*), 123.2 (*C5*), 116.3 (*C7*), 114.6 (*C8``*), 104.5 (*C3*); ESI-MS: m/z calcd. for $C_{23}H_{16}ClNO$ 358.0993 found 358.0988 [M+H$^+$].

2-(7-Chloro-4-(5-chloro-2-vinylphenyl)-1*H*-indol-1-yl)benzaldehyde (S8l):

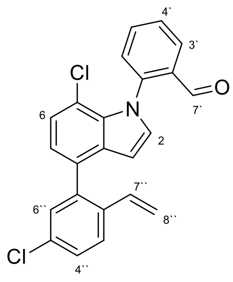

S8l

Step 1 of the general procedure **B** was performed with 4-bromo-7-chloro-1*H*-indole (254 mg, 1.10 mmol), 2-fluorobenzaldehyde (208 mg, 1.67 mmol), KO*t*-Bu (185 mg, 1.65 mmol) over 18 h to give 2-(4-bromo-7-chloro-1*H*-indol-1-yl)benzaldehyde **S7k** (110 mg, 329 µmol, 30%): R$_f$ 0.57 (cyclohexane/EtOAc 5:1). The obtained aldehyde **S7k** (145 mg, 433 µmol) was subjected to the step 2 utilizing 2-(5-chloro-2-vinylphenyl)-4,4,5,5-tetramethyl-1,3,2-dioxaborolane (127 mg, 481 µmol) to give **S8l** as yellow oil (80.0 mg, 204 µmol, 47%) after NP-prep-HPLC (from hexane/EtOAc 98:2 to 80:20, 18 mL/min, 40°C): R$_f$ 0.57 (cyclohexane/EtOAc 5:1); ν_{max}(neat): 3067w, 2848w, 2754w, 1696w, 1597w, 1492w, 1403w. 1264w, 1194w, 1166w, 1101w, 990w, 912w, 823w; ^{1}H NMR (500 MHz, CDCl$_3$) δ = 9.60 (1H, s, C*H*O), 8.06 (1H, dd, ^3J 7.8, ^4J 1.5 Hz, C3`*H*), 7.72 (1H, td, $^3J$ 7.6, $^4J$ 1.5 Hz, C5`*H*), 7.67 (1H, d, ^3J 8.3 Hz, C3``*H*), 7.66 – 7.61 (1H, m, C4`*H*), 7.52 (1H, d, $^3J$ 7.6 Hz, C6`*H*), 7.42 – 7.37 (2H, m, C4``*H*, C6``*H*), 7.23 (1H, d, $^3J$ 7.8 Hz, C6*H*), 7.20 (1H, d, $^3J$ 3.3 Hz, C2*H*), 6.99 (1H, d, $^3J$ 7.8 Hz, C5*H*), 6.54 (1H, dd$^3J$ 17.5, $^3J$ 11.0 Hz, C7``*H*), 6.46 (1H, d, ^3J 3.3 Hz, C3*H*), 5.72 (1H, dd, ^3J 17.5, ^2J 1.0 Hz, C8``*H*), 5.16 (1H, dd, $^3J$ 11.0, $^2J$ 1.0 Hz, C8``*H*); ^{13}C NMR (126 MHz, CDCl$_3$)

δ = 189.4 (*C*HO), 142.6 (*C1`*), 140.1 (*C1``*), 134.9 (*C2``*), 134.7 (*C7``*), 134.2 (*C5`*), 134.1 (*C2`*), 133.6 (*C7a*), 133.3 (*C5``*), 132.4 (*C2*), 131.6 (*C4*), 130.6 (*C6``*), 130.5 (*C3a*), 130.1 (*C6`*), 129.4 (*C4`*), 128.1 (*C4``*), 127.8 (*C3`*), 126.8 (*C3``*), 124.2 (*C6*), 123.0 (*C5*), 116.8 (*C7*), 115.2 (*C8``*), 104.2 (*C3*); ESI-MS: m/z calcd. for $C_{23}H_{16}Cl_2NO$ 392.0603 found 392.0600 [M+H$^+$].

General Procedure C for the synthesis of 13a-i, 19a-e:

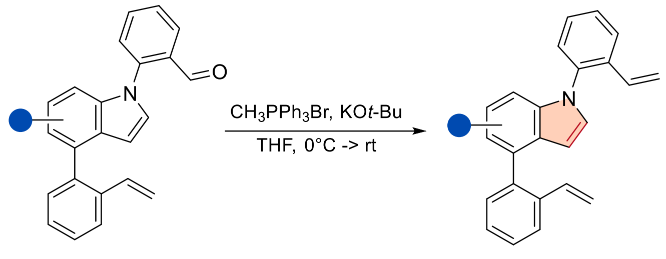

S8a-l **13a-h 19a-e**

To a suspension of methyltriphenylphosphonium-bromide (1.40 eq. or 2.00 eq.) in THF (0.10 mol·L^{-1} with regard to aldehyde **S8a-l**) at 0°C was added dry KO*t*-Bu (1.60 eq. or 2.30 eq.) under argon. After stirring the resulting suspension for 50 min at 0°C, a solution of the corresponding aldehyde **S8a-l** (1.00 eq.) in THF (0.10 mol·L^{-1}) was added dropwise. The reaction mixture was allowed to warm up to room temperature and stirred for 18 h. Water was then added and the mixture was extracted with EtOAc (×3). The combined organic layer was dried over anhydrous Na_2SO_4, filtered and the solvent was removed under reduced pressure. The residue was purified by flash silica gel column chromatography (cyclohexane/EtOAc 100:0→70:30) to give the desired product.

1,4-Bis(2-vinylphenyl)-1*H*-indole (13a):

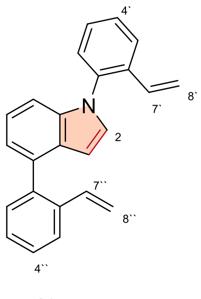

13a

Prepared according to the general procedure **C** using 2-(4-(2-vinylphenyl)-1*H*-indol-1-yl)benzaldehyde **S8a** (158 mg, 490 µmol, 1.00 eq.), methyltriphenylphosphonium-bromide (245 mg, 686 µmol, 1.40 eq.) and KO*t*-Bu (88.0 mg, 784 µmol, 1.60 eq.) to give **13a** as colorless oil (70.0 mg, 218 µmol, 44%): R_f 0.71 (cyclohexane/EtOAc 5:1); ν_{max} (neat): 3058w, 3024w, 2925w, 1830w, 1709w, 1597m, 1512m, 1490s, 1473m, 1421s, 1299m, 1170m, 1056w, 922m, 907m, 752s; ^{1}H NMR (500 MHz, $CDCl_3$) δ = 7.80 – 7.73 (2H, m, C3`*H*, C3``*H*), 7.49 – 7.34 (6H, m, C4`*H*, C5`*H*, C6`*H*, C4``*H*, C5``*H*, C6``*H*), 7.22 (1H, dd, ^3J 8.3, ^3J 7.1 Hz, C6*H*), 7.17 (1H, d, ^3J 3.2 Hz, C2*H*), 7.10 (1H, dd, ^3J 8.3, ^4J 0.9 Hz, C7*H*), 7.06 (1H, dd, ^3J 7.1, ^4J 0.9 Hz, C5*H*), 6.72 (1H, dd, ^3J 17.5, ^3J 11.0 Hz, C7``*H*), 6.43 (1H, dd, $^3J$ 3.2, $^5J$ 0.8 Hz, C3*H*), 6.37 (1H, dd, $^3J$ 17.5, $^3J$ 11.0 Hz, C7`*H*), 5.75 (1H, dd, $^3J$ 17.5, $^2J$ 1.0 Hz, C8`*H*), 5.74 (1H, dd, $^3J$ 17.5, $^2J$ 1.3 Hz, C8``*H*), 5.22 (1H, dd, ^3J 11.0, ^2J 1.0 Hz, C8`*H*), 5.12 (1H, dd, ^3J 11.0, ^2J 1.3 Hz, C8``*H*); $^{13}$C NMR (126 MHz, $CDCl_3$) δ = 140.0 (*C1``*), 137.5 (*C7a*), 137.3 (*C1`*), 136.20 (*C2``*), 136.18 (*C7``*), 135.2 (*C2`*), 133.5 (*C4*), 132.4 (*C7`*), 130.9 (*C6``*), 129.6 (*C2*), 128.7 (*C5`*), 128.38 (*C4`/C4``*), 128.36 (*C4`/C4``*), 128.2 (*C3a*), 127.6 (*C5``*), 127.5 (*C6`*), 126.4 (*C3`*), 125.3 (*C3``*), 122.1 (*C6*), 121.9 (*C5*), 116.4 (*C8`*), 114.0

(*C8``*), 110.1 (*C7*), 103.0 (*C3*); ESI-MS: m/z calcd. for $C_{24}H_{20}N$ 322.1590 found 322.1586 [M+H$^+$].

4-(5-Chloro-2-vinylphenyl)-1-(2-vinylphenyl)-1*H*-indole (13b):

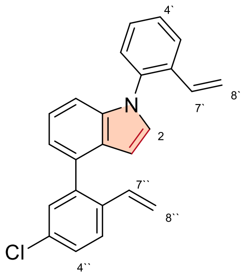

13b

Prepared according to the general procedure **C** using 2-(4-(5-chloro-2-vinylphenyl)-1*H*-indol-1-yl)benzaldehyde **S8b** (147 mg, 410 µmol, 1.00 eq.), methyltriphenylphosphonium-bromide (205 mg, 574 µmol, 1.40 eq.) and KO*t*-Bu (73.6 mg, 656 µmol, 1.60 eq.) to give **13b** as yellow oil (77.0 mg, 216 µmol, 53%): R_f 0.64 (cyclohexane/EtOAc 5:1); ν_{max} (neat): 3086w, 3062w, 2986w, 1898w, 1836w, 1702w, 1630m, 1590m, 1512s, 1491s, 1425s, 1298m, 1267w, 1171m, 1100w, 991w, 913s, 825m, 755s; ^{1}H NMR (500 MHz, $CDCl_3$) δ = 7.78 (1H, dd, ^3J 7.7, ^4J 1.6 Hz, C3`*H*), 7.68 (1H, d, ^3J 8.5 Hz, C3``*H*), 7.50 – 7.33 (5H, m, C4`*H*, C5`*H*, C6`*H*, C4``*H*, C6``*H*), 7.21 (1H, dd, $^3J$ 8.2, $^3J$ 7.1 Hz, C6*H*), 7.19 (1H, d, $^3J$ 3.2 Hz, C2*H*), 7.11 (1H, dt, $^3J$ 8.2, $^4J$ 0.9 Hz, C7*H*), 7.03 (1H, dd, $^3J$ 7.1, $^4J$ 0.9 Hz, C5*H*), 6.64 (1H, dd, $^3J$ 17.5, $^3J$ 11.0 Hz, C7``*H*), 6.42 (1H, dd, ^3J 3.2, ^5J 0.9 Hz, C3*H*), 6.34 (1H, dd, ^3J 17.5, ^3J 11.0 Hz, C7`*H*), 5.75 (1H, dd, $^3J$ 17.5, $^2J$ 1.0 Hz, C8`*H*), 5.22 (1H, dd, ^3J 11.0, ^2J 1.0 Hz, C8`*H*), 5.72 (11H, dd, ^3J 17.5, ^2J 1.1 Hz, C8``*H*), 5.14 (1H, dd, $^3J$ 11.0, $^2J$ 1.1 Hz, C8``*H*); ^{13}C NMR (126 MHz, $CDCl_3$) δ = 141.5 (*C1``*), 137.5 (*C7a*), 137.1 (*C1`*), 135.3 (*C2`*), 135.1 (*C7``*), 134.8 (*C2``*), 133.1 (*C5``*), 132.3 (*C7`*), 132.1 (*C4*), 130.7 (*C6``*), 129.9 (*C2*), 128.7 (*C5`*), 128.5 (*C4`*), 128.4 (*C6`*), 127.9 (*C3a*), 127.6 (*C4``*), 126.7 (*C3``*), 126.4 (*C3`*), 122.1 (*C6*), 121.8 (*C5*), 116.5 (*C8`*), 114.5 (*C8``*), 110.6 (*C7*), 102.7 (*C3*); ESI-MS: m/z calcd. for $C_{24}H_{18}AgClN$ 462.0173 found 462.0177 [M+Ag$^+$].

4-(5-Methoxy-2-vinylphenyl)-1-(2-vinylphenyl)-1*H*-indole (13c):

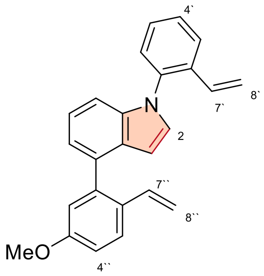

13c

Prepared according to the general procedure **C** using 2-(4-(5-methoxy-2-vinylphenyl)-1*H*-indol-1-yl)benzaldehyde **S8c** (110 mg, 311 µmol, 1.00 eq.), methyltriphenylphosphonium-bromide (167 mg, 467 µmol, 1.50 eq.) and KO*t*-Bu (59.4 mg, 529 µmol, 1.70 eq.) to give **13c** as colorless oil (60.0 mg, 171 µmol, 55%): R_f 0.57 (cyclohexane/EtOAc 6:1); ν_{max} (neat): 3074w, 2960w, 2832w, 1601m, 1566w, 1488s, 1426m, 1296m, 1229m, 1166m, 1045w, 994w, 920w; ^{1}H NMR (500 MHz, $CDCl_3$) δ = 7.78 (1H, dd, ^3J 7.7, ^4J 1.6 Hz, C3`*H*), 7.70 (1H, d, ^3J 8.7 Hz, C3``*H*), 7.49 – 7.36 (3H, m, C4`*H*, C5`*H*, C6`*H*), 7.22 (1H, dd, $^3J$ 8.2, $^3J$ 7.2 Hz, C6*H*), 7.18 (1H, d, $^3J$ 3.2 Hz, C2*H*), 7.10 (1H, dt, $^3J$ 8.2, $^4J$ 0.9 Hz, C7*H*), 7.06 (1H, dd, $^3J$ 7.2, $^4J$ 0.9 Hz, C5*H*), 7.01 (1H, d, $^4J$ 2.7 Hz, C6``*H*), 6.97 (1H, dd, ^3J 8.7, ^4J 2.7 Hz, C4``*H*), 6.63 (1H, dd, $^3J$ 17.5, $^3J$ 11.0 Hz, C7``*H*), 6.45 (1H, dd, ^3J 3.2, ^5J 0.8 Hz, C3*H*), 6.37 (1H, dd, ^3J 17.5, ^3J 11.0 Hz, C7`*H*), 5.76 (1H, dd, $^3J$ 17.5, $^2J$ 1.0 Hz, C8`*H*), 5.61 (1H, dd, ^3J 17.5, 1.3 Hz, C8``*H*), 5.22 (1H, dd, $^3J$ 11.0, $^2J$ 1.0 Hz, C8`*H*), 5.01 (1H, dd, $^3J$ 11.0, $^2J$ 1.3 Hz, C8``*H*), 3.85 (3H, s, OCH_3); ^{13}C NMR (126 MHz, $CDCl_3$) δ = 158.9 (*C5``*), 141.1 (*C1``*), 137.3 (*C7a*), 137.1 (*C1`*), 135.4 (*C7``*), 135.1 (*C2`*), 133.4

(*C4*), 132.2 (*C7`*), 129.5 (*C2*), 129.0 (*C2``*), 128.6 (*C5`*), 128.2 (*C4`/C6`*), 127.9 (*C3a*), 126.4 (*C3``/C3`*), 126.3 (*C3``/C3`*), 121.9 (*C6*), 121.6 (*C5*), 116.3 (*C8`*), 115.1 (*C6``*), 113.9 (*C4``*), 111.7 (*C8``*), 110.0 (*C7*), 102.8 (*C3*), 55.4 (O*C*H$_3$); ESI-MS: m/z calcd. for $C_{25}H_{21}AgNO$ 458.0669 found 458.0674 [M+Ag$^+$].

6-Chloro-1,4-bis(2-vinylphenyl)-1*H*-indole (13d):

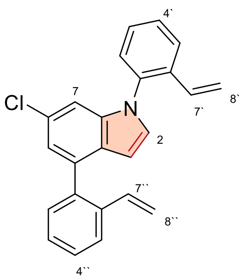

13d

Prepared according to the general procedure **C** using 2-(6-chloro-4-(2-vinylphenyl)-1*H*-indol-1-yl)benzaldehyde **S8d** (190 mg, 531 µmol, 1.00 eq.), methyltriphenylphosphonium-bromide (379 mg, 1.06 mmol, 2.00 eq.) and KO*t*-Bu (137 mg, 1.22 mmol, 2.30 eq.) to give **13d** as yellow oil (120 mg, 337 µmol, 64%): R$_f$ 0.75 (cyclohexane/EtOAc 5:1); ν$_{max}$ (neat): 3063w, 3019w, 2925w, 1832w, 1722w, 1592w, 1505m, 1458m, 1415m, 1322m, 1215w, 1166m, 1076w, 991w, 912s, 855w, 755s; ^{1}H NMR (500 MHz, CDCl$_3$) δ = 7.81 – 7.73 (2H, m, C3`*H*, C3``*H*), 7.51 – 7.46 (1H, m, C4`*H*), 7.45 – 7.35 (5H, m, C5`*H*, C6`*H*, C4``*H*, C5``*H*, C6``*H*), 7.15 (1H, d, ^3J 3.2 Hz, C2*H*), 7.08 (1H, dd, ^3J 1.8, ^5J 0.9 Hz, C7*H*), 7.05 (1H, d, ^3J 1.8 Hz, C5*H*), 6.68 (1H, dd, ^3J 17.5, ^3J 11.0 Hz, C7``*H*), 6.38 (1H, dd, $^3J$ 3.2, $^5J$ 0.9 Hz, C3*H*), 6.32 (1H, dd, $^3J$ 17.5, $^3J$ 11.0 Hz, C7`*H*), 5.76 (1H, dd, $^3J$ 17.5, $^2J$ 1.0 Hz, C8`*H*), 5.75 (1H, dd, $^3J$ 17.5, $^2J$ 1.2 Hz, C8``*H*), 5.25 (1H, dd, ^3J 11.1, ^2J 1.0 Hz, C8`*H*) 5.16 (1H, dd, ^3J 11.0, ^2J 1.2 Hz, C8``*H*); $^{13}$C NMR (126 MHz, CDCl$_3$) δ = 138.6 (*C1``*), 137.7 (*C7a*), 136.7 (*C1`*), 136.2 (*C2``*), 135.7 (*C7``*), 135.3 (*C2`*), 134.7 (*C4*), 132.0 (*C7`*), 130.7 (*C6``*), 130.2 (*C2*), 128.84 (*C4`/C5``*), 128.77 (*C4`/C5`*), 128.3 (*C6`*), 128.1 (*C6*), 128.0 (*C4``*), 127.7 (*C5``*), 126.8 (*C3a*), 126.6 (*C3`*), 125.4 (*C3``*), 122.3 (*C5*), 116.9 (*C8``*), 114.6 (*C8`*), 110.0 (C7), 103.1 (*C3*); ESI-MS: m/z calcd. for $C_{24}H_{18}AgClN$ 462.0173 found 462.0165 [M+Ag$^+$].

6-(Trifluoromethyl)-1,4-bis(2-vinylphenyl)-1*H*-indole (13e):

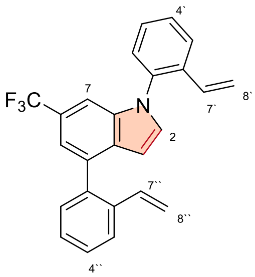

13e

Prepared according to the general procedure **C** using 2-(6-(trifluoromethyl)-4-(2-vinylphenyl)-1*H*-indol-1-yl)benzaldehyde **S8e** (150 mg, 383 µmol, 1.00 eq.), methyltriphenylphosphonium-bromide (274 mg, 766 µmol, 2.00 eq.) and KO*t*-Bu (98.9 mg, 881 µmol, 2.30 eq.) to give **13e** as a white solid (130 mg, 334 µmol, 87%, m.p. 85.2 – 86.2°C): R$_f$ 0.69 (cyclohexane/EtOAc 5:1); ν$_{max}$ (neat): 3065w, 3017w, 2926w, 1826w, 1734w, 1627w, 1599w, 1503m, 1422s, 1369s, 1335m, 1305m, 1225s, 1175s, 1113s, 991m, 917s, 867m, 759s; ^{1}H NMR (500 MHz, CDCl$_3$) δ = 7.83 – 7.72 (2H, m, C3`*H*, C3``*H*), 7.51 (1H, td, $^3J$ 7.4, $^4J$ 0.9 Hz, C4`*H*), 7.47 – 7.40 (3H, m, C5`*H*, C4``*H*, C6``*H*), 7.40 – 7.28 (5H, m, C2*H*, C5*H*, C7*H*, C6`*H*, C5``*H*), 6.64 (1H, dd, ^3J 17.5, ^3J 11.0 Hz, C7``*H*), 6.47 (1H, d, $^3J$ 3.2,C3*H*), 6.29 (1H, dd, $^3J$ 17.4, $^3J$ 11.0 Hz, C7`*H*), 5.77 (1H, dd, $^3J$ 17.4, $^4J$ 1.0 Hz, C8`*H*), 5.76 (1H, dd, $^3J$ 17.5, $^4J$ 1.2 Hz, C8``*H*), 5.24 (1H, d, ^3J 11.0 Hz, C8`*H*), 5.16 (1H, dd, ^3J 11.0, ^4J 1.2 Hz, C8``*H*); $^{13}$C NMR (126 MHz, CDCl$_3$) δ = 138.6 (*C1``*), 136.5 (*C1`*), 136.4 (*C7a*), 136.3 (*C2``*), 135.6 (*C7``*),

135.3 (*C2*`), 134.3 (*C4*), 132.1 (*C2*), 131.9 (*C7*`), 130.8 (*C4*``), 130.5 (*C3a*), 128.98 (*C6*``/*C6*`), 128.94 (*C5*`), 128.3 (*C4*`), 128.1 (*C6*``/*C6*`), 127.7 (*C5*``), 126.7 (*C3*`), 125.5 (*C3*``), 125.2 (q, $^{1}J_{CF}$ 271.8 Hz, *C*F$_3$), 124.4 (q, $^{2}J_{CF}$ 32.1 Hz, *C6*) 118.4 (q, $^{3}J_{CF}$ 3.4 Hz, *C5*), 117.1 (*C8*`), 114.7 (*C8*``), 107.7 (q, $^{3}J_{CF}$ 4.3 Hz, *C7*), 103.3 (*C3*); ^{19}F NMR (376 MHz, CDCl$_3$) δ –60.45; ESI-MS: m/z calcd. for $C_{25}H_{18}AgF_3N$ 496.0437 found 496.0437 [M+Ag$^+$].

7-Fluoro-1,4-bis(2-vinylphenyl)-1*H*-indole (13f):

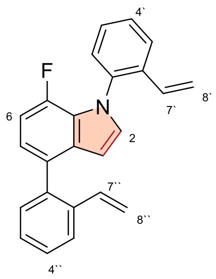

13f

Prepared according to the general procedure **C** using 2-(7-fluoro-4-(2-vinylphenyl)-1*H*-indol-1-yl)benzaldehyde **S8f** (119 mg, 350 µmol, 1.00 eq.), methyltriphenylphosphonium-bromide (175 mg, 490 µmol, 1.40 eq.) and KO*t*-Bu (62.8 mg, 560 µmol, 1.60 eq.) to give **13f** as yellow oil (50.0 mg, 147 µmol, 42%): R$_f$ 0.75 (cyclohexane/EtOAc 5:1); ν_{max} (neat): 3062w, 3017w, 1831w, 1717w, 1625w, 1580w, 1500s, 1402m, 1296m, 1240s, 1185m, 1110w, 991w, 907s ^{1}H NMR (500 MHz, CDCl$_3$) δ 7.78 – 7.74 (1H, m, C3``*H*), 7.72 (1H, d, $^{3}J$ 7.7 Hz, C3`*H*), 7.49 – 7.34 (6H, m, C4`*H*, C5`*H*, C6`*H*, C4``*H*, C5``*H*, C6``*H*), 7.09 (1H, d, ^{3}J 3.2 Hz, C2*H*), 7.00 – 6.86 (2H, m, C5*H*, C6*H*), 6.67 (1H, dd, ^{3}J 17.5, ^{3}J 11.0 Hz, C7``*H*), 6.41 (1H, d, $^{3}J$ 3.0 Hz, C3*H*), 6.40 – 6.33 (1H, m, C7`*H*), 5.73 (1H, dd, $^{3}J$ 17.5, $^{2}J$ 1.1 Hz, C8``*H*) , 5.72 (1H, dd, ^{3}J 17.5, ^{3}J 1.0 Hz, C8`*H*), 5.22 (1H, d, $^{3}J$ 11.1 Hz, C8`*H*), 5.14 (1H, dd, ^{3}J 11.0, ^{2}J 1.1 Hz, C8``*H*); $^{13}$C NMR (126 MHz, CDCl$_3$) δ = 149.6 (d, $^{1}J_{CF}$ 247.0 Hz, *C7*), 139.2 (*C1*``), 138.2 (*C1*`), 136.3 (*C2*``), 136.0 (*C7*``), 135.4 (*C2*`), 132.00 (d, $^{3}J_{CF}$ 4.3 Hz, *C3a*), 132.0 (*C7*`), 131.1 (*C2*), 131.0 (*C6*``), 129.5 (d, $^{4}J_{CF}$ Hz, *C4*), 128.6 (*C4*`), 128.4 (*C4*``), 128.3 (*C6*`), 128.2 (*C5*`), 127.6 (*C5*``), 125.8 (*C3*`), 125.4 (*C3*``), 124.8 (*C7a*), 122.0 (d, $^{3}J_{CF}$ 6.4 Hz, *C5*), 116.6 (*C8*`), 114.2 (*C8*``), 107.9 (d, $^{2}J_{CF}$ 17.8 Hz, *C6*), 103.8 (*C3*); ESI-MS: m/z calcd. for $C_{24}H_{19}FN$ 340.1496 found 340.1498 [M+H$^+$].

3-Methyl-1,4-bis(2-vinylphenyl)-1*H*-indole (13g):

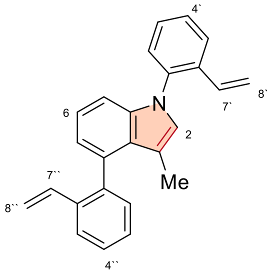

13g

Prepared according to the general procedure **C** using 2-(3-methyl-4-(2-vinylphenyl)-1*H*-indol-1-yl)benzaldehyde **S8g** (87.7 mg, 260 µmol, 1.00 eq.), methyltriphenylphosphonium-bromide (186 mg, 520 µmol, 2.00 eq.) and KO*t*-Bu (67.1 mg, 598 µmol, 2.30 eq.) to give **13g** as a white solid (60.0 mg, 179 µmol, 69%, m.p. 119.4 – 120.9°C): R$_f$ 0.77 (cyclohexane/EtOAc 6:1); ν_{max} (neat): 3068w, 2945w, 1628w, 1595w, 1573w, 1489s, 1453m, 1429s, 1307m, 1172w, 1049w, 993m, 914s; ^{1}H NMR (500 MHz, CDCl$_3$) δ = 7.76 (1H, dd, ^{3}J 7.6, ^{4}J 1.7 Hz, C3`*H*), 7.73 – 7.67 (1H, m, C3``*H*), 7.47 – 7.28 (6H, m, C4`*H*, C5`*H*, C6`*H*, C4``*H*, C5``*H*, C6``*H*), 7.16 (1H, dd, ^{3}J 8.2, ^{3}J 7.1 Hz, C6*H*), 7.06 (1H, dd, ^{3}J 8.2, ^{4}J 1.0 Hz, C7*H*), 6.93 – 6.87 (2H, m, C2*H*, C5*H*), 6.55 (1H, d, ^{3}J 17.6, ^{3}J 11.0 Hz, C7``*H*), 6.41 (1H, dd, $^{3}J$ 17.1, $^{3}J$ 11.1 Hz, C7`*H*), 5.76 (1H, dd, $^{3}J$ 17.1, $^{2}J$ 1.1 Hz, C8`*H*), 5.70 (1H, dd, $^{3}J$ 17.6, $^{2}J$ 1.3 Hz, C8``*H*), 5.22 (1H, d, ^{3}J 11.1 Hz, C8`*H*), 5.07 (1H, dd, ^{3}J 11.0, ^{2}J 1.3 Hz, C8``*H*), 1.74 (3H, d, $^{4}J$ 1.1 Hz, C*H*$_3$); $^{13}$C NMR (126 MHz, CDCl$_3$) δ = 140.5 (*C1*``), 138.0 (*C7a*), 137.4 (*C1*`), 136.8 (*C2*``), 135.8 (*C7*``), 135.1 (*C2*`), 134.2 (*C4*), 132.6 (*C7*`), 131.0 (*C6*``), 128.7 (*C5*`),

128.4 (*C4`/C6`*), 128.1 (*C4`/C6`*), 128.0 (*C2*), 127.5 (*C4``*), 126.9 (*C5``/C3a*), 126.3 (*C3`*), 124.2 (*C3``*), 121.8 (*C6*), 121.4 (*C5*), 116.1 (*C8`*), 114.0 (*C8``*), 112.9 (*C3*), 110.1 (*C7*), 11.5 (*C*H_3); ESI-MS: m/z calcd. for $C_{25}H_{21}N$ 335.1669 found 335.1662 [$M^{+\cdot}$].

1,4-Bis(2-vinylphenyl)-1*H*-pyrrolo[2,3-c]pyridine (13h):

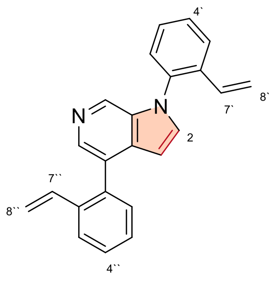

13h

Prepared according to the general procedure **C** using 2-(4-(2-vinylphenyl)-1*H*-pyrrolo[2,3-c]pyridin-1-yl)benzaldehyde **S8h** (90.8 mg, 280 µmol, 1.00 eq.), methyltriphenylphosphonium-bromide (200 mg, 560 µmol, 2.00 eq.) and KO*t*-Bu (72.3 mg, 644 µmol, 2.30 eq.) to give **13h** as a transparent oil (65.0 mg, 202 µmol, 72%): R_f 0.47 (cyclohexane/EtOAc 1:1); ν_{max} (neat): 3060w, 3013w, 1832w, 1694w, 1598w, 1499m, 1451w, 1424s, 1303w, 1172m, 1118w, 1082w, 991w, 914w, 879w; ^{1}H NMR (500 MHz, $CDCl_3$) δ = 8.49 (1H, d, ^4J 0.7 Hz, C7*H*), 8.23 (1H, s, C5*H*), 7.81 – 7.75 (2H, m, C3`*H*, C3``*H*), 7.55 – 7.47 (1H, m, C4`*H*), 7.47 – 7.38 (5H, m, C5`*H*, C6`*H*, C4``*H*, C5``*H*, C6``*H*), 7.32 (1H, d, ^3J 3.1 Hz, C2*H*), 6.68 (1H, dd, ^3J 17.5, ^3J 11.0 Hz, C7``*H*), 6.48 (1H, dd, $^3J$ 3.1, $^5J$ 0.8 Hz, C3*H*), 6.33 (1H, dd, $^3J$ 17.5, $^3J$ 11.0 Hz, C7`*H*), 5.89 – 5.69 (2H, m, C8`*H*, C8``*H*), 5.27 (1H, dd, ^3J 11.0, ^5J 0.9 Hz, C8`*H*), 5.17 (1H, dd, ^3J 11.0, ^2J 1.2 Hz, C8``*H*); $^{13}$C NMR (126 MHz, $CDCl_3$) δ = 140.1 (*C5*), 136.6 (*C2``*), 136.5 (*C1``*), 136.2 (*C1`*), 135.7 (*C7``*), 135.2 (*C2`*), 134.1 (*C7a*), 133.4 (*C7*), 133.2 (*C3a*), 133.1 (*C2*), 131.8 (*C7`*), 131.0 (*C6``*) 129.0 (*C4`*), 128.9 (*C5`*), 128.4 (*C4*), 128.2 (*C4``*), 128.1 (*C6`*), 127.8 (*C5``*), 126.8 (*C3`/C3``*), 125.6 (*C3`/C3``*), 117.4 (*C8`*), 114.9 (*C8``*), 102.5 (*C3*); ESI-MS: m/z calcd. for $C_{23}H_{19}N_2$ 323.1543 found 323.1549 [$M+H^+$].

4-(2-Vinylnaphthalen-1-yl)-1-(2-vinylphenyl)-1*H*-indole (13i):

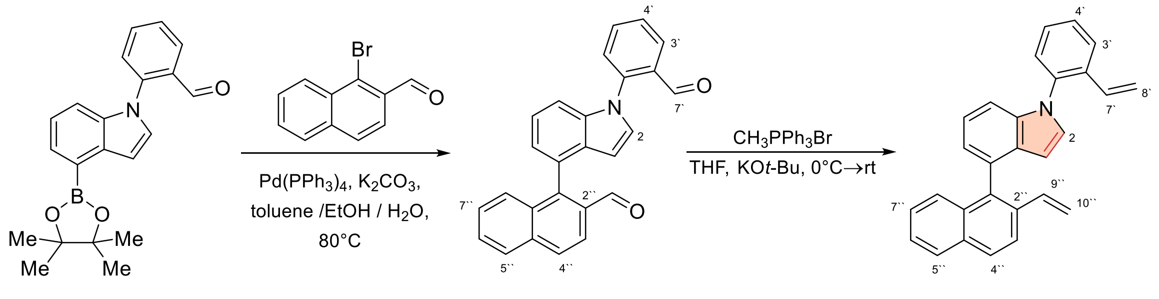

Step 1: 1-Bromo-2-naphthaldehyde (63.5 mg, 270 µmol, 1.00 eq.), 2-(4-(4,4,5,5-tetramethyl-1,3,2-dioxaborolan-2-yl)-1*H*-indol-1-yl)benzaldehyde (**S5**) (105 mg, 302 µmol, 1.12 eq.), $Pd(PPh_3)_4$ (15.6 mg, 13.5 µmol, 5 mol%), and K_2CO_3 (112 mg, 810 µmol, 3.00 eq.) were weighed in a 10 mL crimp cap vial. The vial was sealed, evacuated and backfilled with argon before a degassed mixture of toluene, H_2O, ethanol (2 : 2 : 1) (2.5 mL, 0.11 mol·L^{-1}) was added. The resulting mixture was stirred at 80°C for 18 h, then cooled to room temperature, filtered through a thin layer of silica gel and Na_2SO_4 using EtOAc (15-20 mL). The solvents were removed under reduced pressure

and the residue was purified by the silica gel chromatography (cyclohexane/EtOAc 100:0→60:40) to give the desired product 1-(1-(2-formylphenyl)-1*H*-indol-4-yl)-2-naphthaldehyde **S8m** as yellow oil (97.0 mg, 258 µmol, 96%): R_f 0.29 (cyclohexane/EtOAc 5:1); ν_{max} (neat): 3057w, 2853w, 2753w, 1691s, 1597m, 1488m, 1424w, 1248w, 1219w, 958w, 760w; ^{1}H NMR (500 MHz, $CDCl_3$) δ = 9.84 (1H, s, C2``–C*H*O), 9.74 (1H, s, C2`–C*H*O), 8.18 – 8.12 (2H, m, C3``*H*, C3`*H*), 8.01 (1H, d, ^3J 8.4 Hz, C4``*H*), 7.97 (1H, d, $^3J$ 8.2 Hz, C5``*H*), 7.82 (1H, td, ^3J 7.7, ^4J 1.6 Hz, C5`*H*), 7.72 – 7.53 (4H, m, C4`*H*, C6`*H*, C6``*H*, C8``*H*), 7.48 – 7.41 (2H, m, C7``*H*), 7.41 – 7.30 (2H, m, C6*H*), 7.31 – 7.19 (3H, m, C2*H*, C5*H*, C7*H*), 6.29 – 6.12 (1H, m, C3*H*); $^{13}$C NMR (126 MHz, $CDCl_3$) δ = 193.1 (C2``–*C*HO), 189.6 (C2`–*C*HO), 145.2 (*C1``*), 141.8 (*C1`*), 138.2 (*C7a*), 136.4 (*C4a``*), 135.4 (*C5`*), 132.6 (*C2``*), 132.3 (*C2`*), 131.7 (*C8a``*), 130.7 (*C2*), 130.2 (*C3a*), 129.0 (*C3`*), 128.9 (*C6``*), 128.8 (*C4`*), 128.7 (*C4``*), 128.4 (*C6`*), 128.2 (*C4*/*C5``*), 128.0 (*C8``*), 127.0 (*C7``*), 124.2 (*C5*), 123.0 (*C6*), 122.3 (*C3``*), 110.5 (*C7*), 103.9 (*C3*); ESI-MS: m/z calcd. for $C_{28}H_{18}NO_2$ 376.1332 found 376.1337 [M+H$^+$].

Step 2: To a suspension of methyltriphenylphosphonium-bromide (372 mg, 1.04 mmol, 4.00 eq.) in THF (2.6 mL) at 0°C was added dry KO*t*-Bu (134 mg, 1.20 mmol, 4.60 eq.) under argon. The resulting suspension was stirred for 40 min at 0°C and a solution of 1-(1-(2-formylphenyl)-1*H*-indol-4-yl)-2-naphthaldehyde **S8m** (97.6 mg, 260 µmol, 1.00 eq.) in THF (2.6 mL) was added dropwise. The reaction mixture was allowed to warm up to room temperature and stirred for 18 h. Water was then added and the mixture was extracted with EtOAc (3×30 mL). The combined organic layer was dried over anhydrous Na_2SO_4, filtered and the solvent was removed under reduced pressure. The residue was purified by flash silica gel column chromatography (cyclohexane/EtOAc 100:0→70:30) to give the desired product **13i** as a white solid (80.0 mg, 215 µmol, 83%, m.p. 75.0 – 76.0°C): R_f 0.71 (cyclohexane/EtOAc 5:1); ν_{max} (neat): 3082w, 3061w, 1631w, 1598w, 1512m, 1490s, 1422s, 14294w, 1214w, 1171w, 1028w, 992w, 914w, 823; ^{1}H NMR (500 MHz, $CDCl_3$) δ = 7.90 – 7.84 (2H, m, C3``*H*, C4``*H*), 7.79 (1H, d, ^3J 7.4 Hz, C3`*H*), 7.49 – 7.40 (5H, m, C4`*H*, C5`*H*, C6`*H*, C5``*H*, C7``*H*), 7.34 – 7.27 (3H, m, C6*H*, C6``*H*, C8``*H*), 7.20 (1H, dt, ^3J 8.3, ^4J 0.9 Hz, C7*H*), 7.11 (1H, d, ^3J 3.2 Hz, C2*H*), 7.07 (1H, dd, ^3J 7.1, ^4J 0.9 Hz, C5*H*), 6.63 (1H, dd, ^3J 17.6, ^3J 11.0 Hz, C9``*H*), 6.41 (1H, dd, $^3J$ 17.5, $^3J$ 11.0 Hz, C7`*H*), 6.09 (1H, dd, $^3J$ 3.2, $^5J$ 0.8 Hz, C3*H*), 5.82 (1H, dd, $^3J$ 17.6, $^2J$ 1.0 Hz, C10``*H*), 5.76 (1H, dd, ^3J 17.5, ^2J 1.0 Hz, C8`*H*), 5.24 (1H, dd, $^3J$ 11.0, $^2J$ 1.0 Hz, C8`*H*), 5.15 (1H, dd, ^3J 11.0, ^2J 1.0 Hz, C10``*H*); $^{13}$C NMR (126 MHz, $CDCl_3$) δ = 137.4 (*C7a*), 137.3 (*C1`*), 136.9 (*C1``*), 136.0 (*C9``*), 135.2 (*C2`*), 133.3 (*C2``*/*C4a``*), 133.2 (*C2``*/*C4a``*), 132.4 (*C7`*), 131.1 (*C4*), 129.7 (*C2*), 129.3 (*C3a*), 128.7 (*C5`*), 128.4 (*C8a``*), 128.3 (*C4`*/*C6`*), 127.9 (*C3``*), 127.8 (*C8``*), 127.5 (*C5``*), 126.5 (*C3`*), 126.1 (*C6``*), 125.8 (*C7``*), 122.72 (*C5*), 122.65 (*C4``*), 122.2 (*C6*), 116.4 (*C8`*), 114.2 (*C10``*), 110.3 (*C7*), 102.9 (*C3*); ESI-MS: m/z calcd. for $C_{28}H_{22}N$ 372.1747 found 372.1741 [M+H$^+$].

2-Methyl-1,4-bis(2-vinylphenyl)-1*H*-indole (19a):

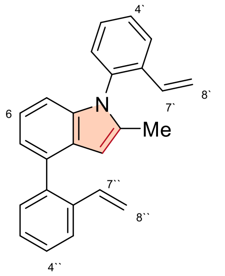

19a

Prepared according to the general procedure **C** using 2-(2-methyl-4-(2-vinylphenyl)-1*H*-indol-1-yl)benzaldehyde **S8i** (58.0 mg, 172 µmol, 1.00 eq.), methyltriphenylphosphonium-bromide (123 mg, 344 µmol, 2.00 eq.) and KO*t*-Bu (44.4 mg, 396 mmol, 2.30 eq.) to give **19a** as yellow oil (40.4 mg, 120 µmol, 70%, m.p. 150.8 – 152.4°C): R_f 0.71 (cyclohexane/EtOAc 5:1); ν_{max} (neat): 3058w, 3018w, 2918w, 2846w, 1826w, 1624w, 1602w, 1553m, 1488m, 1455m, 1422m, 1385m, 1323m, 1261w, 1162w, 1084w, 994w, 910w, 768s; ^{1}H NMR (500 MHz, $CDCl_3$) δ = 7.79 (1H, dd, ^3J 7.8, ^4J 1.3 Hz, C3`*H*), 7.78 – 7.73 (1H, m, C3``*H*), 7.53 – 7.46 (2H, m, C4`*H*, C6``*H*), 7.43 (1H, td, ^3J 7.6, ^4J 1.3 Hz, C5`*H*), 7.41 – 7.33 (2H, m, C4``*H*, C5``*H*), 7.30 (1H, d, ^3J 7.6 Hz, C6`*H*), 7.15 – 7.07 (1H, m, C6*H*), 7.00 (1H, dd, ^3J 7.2, ^4J 0.7 Hz, C5*H*), 6.84 (1H, d, ^3J 8.1 Hz, C7*H*), 6.75 (1H, dd, ^3J 17.6, ^3J 11.0 Hz, C7``*H*), 6.18 (1H, s, C3*H*), 6.14 (1H, dd, $^3J$ 17.6, $^3J$ 11.1 Hz, C7`*H*), 5.73 (1H, dd, $^3J$ 17.6, $^2J$ 1.2 Hz, C8``*H*), 5.69 (1H, d, ^3J 17.6 Hz, C8`*H*), 5.16 (1H, d, $^3J$ 11.1 Hz, C8`*H*), 5.12 (1H, dd, ^3J 11.0, ^2J 1.2 Hz, C8``*H*), 2.12 (3H, s, C2–CH_3); ^{13}C NMR (126 MHz, $CDCl_3$) δ =

140.3 (*C1``*), 138.5 (*C7a*), 138.1 (*C2*), 136.8 (*C2`*), 136.3 (*C7``*), 136.1 (*C2``*), 135.8 (*C1`*), 132.2 (*C4*), 131.9 (*C7`*), 130.9 (*C6``*), 129.8 (*C6`*), 129.0 (*C4`*), 128.8 (*C5`*), 127.8 (*C3a*), 127.5 (*C5``*), 127.3 (*C4``*), 126.2 (*C3`*), 125.2 (*C3``*), 121.8 (*C5*), 120.9 (*C6*), 116.5 (*C8`*), 113.8 (*C8``*), 109.4 (*C7*), 100.9 (*C3*), 13.2 (C2–CH_3); ESI-MS: m/z calcd. for $C_{25}H_{22}N$ 336.1747 found 336.1745 [M+H^+].

7-Methoxy-1,4-bis(2-vinylphenyl)-1*H*-indole (19b):

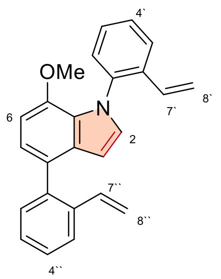

19b

Prepared according to the general procedure **C** using 2-(7-methoxy-4-(2-vinylphenyl)-1*H*-indol-1-yl)benzaldehyde (42.4 mg, 120 µmol, 1.00 eq.), methyltriphenylphosphonium-bromide (85.7 mg, 240 µmol, 2.00 eq.) and KO*t*-Bu (31.0 mg, 276 µmol, 2.30 eq.) to give **19b** as colorless oil (32.0 mg, 91.1 µmol, 76%): R_f 0.75 (cyclohexane/EtOAc 6:1); ν_{max} (neat): 3069w, 3031w, 1624w, 1600w, 1574w, 1528w, 1495s, 1457m, 1428m, 1297m, 1251s, 1176w, 1094m, 1051w, 993w, 914m; ^{1}H NMR (500 MHz, $CDCl_3$) δ = 7.74 (1H, dd, ^3J 7.4, ^4J 1.7 Hz, C6``*H*), 7.69 – 7.65 (1H, m, C3`*H*), 7.46 (1H, dd, $^3J$ 7.1, $^4J$ 1.8 Hz, C3``*H*), 7.42 – 7.29 (5H, m, C4`*H*, C5`*H*, C6`*H*, C4``*H*, C5``*H*), 7.00 (1H, d, ^3J 3.1 Hz, C2*H*), 6.95 (1H, d, ^3J 7.9 Hz, C5*H*), 6.74 (1H, dd, ^3J 17.6, ^3J 11.0 Hz, C7``*H*), 6.67 (1H, d, $^3J$ 7.9 Hz, C6*H*), 6.40 – 6.29 (2H, m, C3*H*, C7`*H*), 5.72 (1H, dd, 3*J* 17.6, 2*J* 1.3 Hz, C8``*H*), 5.67 (1H, dd, 3*J* 17.6, 2*J* 1.1 Hz, C8`*H*), 5.19 – 5.06 (2H, m, C8`*H*, C8``*H*), 3.61 (3H, s, OC*H*3); 13C NMR (126 MHz, CDCl3) δ = 147.0 (*C7*), 139.9 (*C4*), 139.6 (*C1`*), 136.40 (*C7``*), 136.37 (*C2``*), 135.6 (*C2`*), 132.6 (*C7`*), 131.1 (*C3``*), 130.5 (*C2*), 130.3 (*C3a*), 128.6 (*C6`*), 127.9 (*C4`*), 127.52 (*C4``*), 127.48 (*C5`*), 127.2 (*C5``*), 126.8 (*C7a*), 126.4 (*C1``*), 125.3 (*C6``*), 125.0 (*C3`*), 122.3 (*C5*), 115.5 (*C8`*), 113.7 (*C8``*), 103.5 (*C6*), 103.2 (*C3*), 55.8 (OCH_3); ESI-MS: m/z calcd. for $C_{25}H_{22}NO$ 352.1696 found 352.1698 [M+H^+].

7-Methyl-1,4-bis(2-vinylphenyl)-1*H*-indole (19c):

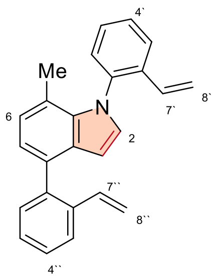

19c

Prepared according to the general procedure **C** using 2-(7-methyl-4-(2-vinylphenyl)-1*H*-indol-1-yl)benzaldehyde **S8j** (59.0 mg, 175 µmol, 1.00 eq.), methyltriphenylphosphonium-bromide (87.4 mg, 245 µmol, 1.40 eq.) and KO*t*-Bu (31.4 mg, 280 µmol, 1.60 eq.) to give **19c** as colorless oil (34.0 mg, 101 µmol, 58%): R_f 0.68 (cyclohexane/EtOAc 5:1); ν_{max} (neat): 3060w, 3021w, 2924m, 2850w, 1813w, 1703w, 1627w, 1598w, 1487s, 1453s, 1412w, 1380w, 1317m, 1266m, 1185w, 1150w, 1114w, 1039w, 991m, 903s, 871m, 760s; ^{1}H NMR (500 MHz, $CDCl_3$) δ = 7.78 – 7.74 (1H, m, C3``*H*), 7.71 (1H, d, $^3J$ 7.8 Hz, C3`*H*), 7.50 – 7.43 (2H, m, C4`*H*, C6``*H*), 7.41 – 7.31 (4H, m, C5`*H*, C6`*H*, C4``*H*, C5``*H*), 7.02 (1H, d, ^3J 3.2 Hz, C2*H*), 6.95 (2H, s, C5*H*, C6*H*), 6.72 (1H, dd, ^3J 17.6, ^3J 11.0 Hz, C7``*H*), 6.38 (1H, d, $^3J$ 3.2 Hz, C3*H*), 6.23 (1H, dd, $^3J$ 17.5, $^3J$ 11.1 Hz, C7`*H*), 5.73 (1H, dd, $^3J$ 17.6, $^2J$ 1.2 Hz, C8``*H*), 5.71 (1H, dd, ^3J 17.5, ^2J 1.0 Hz, C8`*H*), 5.18 (1H, d, $^3J$ 11.1 Hz, C8`*H*), 5.11 (1H, dd, ^3J 11.0, ^2J 1.2 Hz, C8``*H*), 1.96 (3H, s, C7–C*H*3); $^{13}$C NMR (126 MHz, $CDCl_3$) δ = 140.1 (*C1``*), 139.3 (*C1`*),

136.9 (*C2`*), 136.28 (*C7``*), 136.25 (*C2``*), 136.1 (*C7a*), 131.8 (*C7`*), 131.6 (*C4*), 131.0 (*C6``*), 130.4 (*C2*), 129.8 (*C4``*), 128.9 (*C4`*), 128.6 (*C3a*), 127.9 (C*5`*), 127.5 (*C5``*), 127.3 (*C6`*), 125.27 (*C3`*), 125.2 (*C3``*), 124.4 (*C6*), 121.9 (*C5*), 121.1 (*C7*), 116.7 (*C8`*), 113.8 (*C8``*), 102.9 (*C3*), 18.6 (C7–*C*H3); ESI-MS: m/z calcd. for $C_{25}H_{21}AgN$ 442.0719 found 442.0714 $[M+Ag^+]$.

7-Chloro-1,4-bis(2-vinylphenyl)-1*H*-indole (19d):

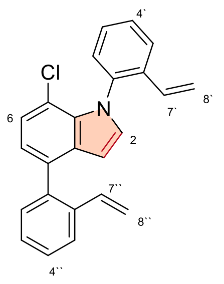

19d

Prepared according to the general procedure **C** using 2-(7-chloro-4-(2-vinylphenyl)-1*H*-indol-1-yl)benzaldehyde **S8k** (119 mg, 333 µmol, 1.00 eq.), methyltriphenylphosphonium-bromide (238 mg, 665 µmol, 2.00 eq.) and KO*t*-Bu (85.8 mg, 765 µmol, 2.30 eq.) to give **19d** as yellow oil (36.0 mg, 101 µmol, 30%): R_f 0.83 (cyclohexane/EtOAc 6:1); ν_{max} (neat): 3064w, 3026w, 2983w, 1844w, 1736w, 1627w, 1594w, 1492s, 1476s, 1413w, 1335w, 1262m, 1165s, 1107w, 991w, 914m; ^{1}H NMR (500 MHz, $CDCl_3$) δ = 7.79 – 7.73 (1H, m, C3``*H*), 7.69 (1H, d, $^3J$ 8.0 Hz, C3`*H*), 7.49 – 7.33 (6H, m, C4`*H*, C5`*H*, C6`*H*, C4``*H*, C5``*H*, C6``*H*), 7.18 (1H, d, ^3J 7.7 Hz, C6*H*), 7.06 (1H, d, ^3J 3.2 Hz, C2*H*), 6.96 (1H, d, ^3J 7.7 Hz, C5*H*), 6.66 (1H, dd, ^3J 17.5, ^3J 11.0 Hz, C7``*H*), 6.40 (1H, d, $^3J$ 3.2 Hz, C3*H*), 6.28 – 6.14 (1H, dd, m, C7`*H*), 5.73 (1H, dd, $^3J$ 17.5, $^2J$ 1.2 Hz, C8``*H*), 5.69 (1H, dd, ^3J 17.5, ^2J 1.0 Hz, C8`*H*), 5.18 (1H, d, $^3J$ 11.1 Hz, C8`*H*), 5.14 (1H, dd, ^3J 11.0, ^2J 1.2 Hz, C8``*H*); $^{13}$C NMR (126 MHz, $CDCl_3$) δ = 139.0 (*C1``*), 138.1 (*C1`*), 136.9 (*C2`*), 136.3 (*C2``*), 135.9 (*C7``*), 132.7 (*C4*), 132.6 (*C7a*), 131.8 (*C7`*), 131.7 (*C2*), 130.8 (*C6``*/*C4``*), 130.6 (*C3a*), 129.7 (*C4`*), 129.0 (*C6`*), 127.81 (*C5`*), 127.76 (*C6``*/*C4``*), 127.6 (*C5``*), 125.38 (*C3``*), 125.32 (*C3`*), 123.5 (*C6*), 122.5 (*C5*), 116.7 (*C8`*), 116.6 (*C7*), 114.4 (*C8``*), 103.3 (*C3*); ESI-MS: m/z calcd. for $C_{24}H_{18}AgClN$ 462.0173 found 462.0174 $[M+Ag^+]$.

7-Chloro-4-(5-chloro-2-vinylphenyl)-1-(2-vinylphenyl)-1*H*-indole (19e):

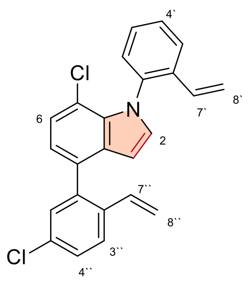

19e

Prepared according to the general procedure **C** using 2-(7-chloro-4-(5-chloro-2-vinylphenyl)-1*H*-indol-1-yl)benzaldehyde **S8l** (75.0 mg, 191 µmol, 1.00 eq.), methyltriphenylphosphonium-bromide (137 mg, 382 µmol, 2.00 eq.) and KO*t*-Bu (49.3 mg, 440 µmol, 2.30 eq.) to give **19e** as a colorless oil (62.0 mg, 159 µmol, 83%): R_f 0.60 (cyclohexane/EtOAc 5:1); ν_{max} (neat): 3085w, 3013w, 2925w, 2855w, 1842w, 1716w, 1591w, 1492s, 1411m, 1333w, 1262m, 1218w, 1165m, 1098m, 988m, 911s, 824s, 763s, 727s; ^{1}H NMR (500 MHz, $CDCl_3$) δ = 7.69 (1H, d, ^3J 7.9 Hz, C3`*H*), 7.67 (1H, d, ^3J 8.6 Hz, C3``*H*), 7.51 – 7.45 (1H, m, C4`*H*), 7.42 (1H, d, $^3J$ 2.1 Hz, C6``*H*), 7.40 – 7.33 (3H, m, C5`*H*, C6`*H*, C4``*H*), 7.18 (1H, d, $^3J$ 7.8 Hz, C6*H*), 7.08 (1H, d, $^3J$ 3.2 Hz, C2*H*), 6.94 (1H, d, $^3J$ 7.8 Hz, C5*H*), 6.58 (1H, dd, $^3J$ 17.5, $^3J$ 11.0 Hz, C7``*H*), 6.39 (1H, d, ^3J 3.2 Hz, C3*H*), 6.30 – 6.05 (1H, m, C7`*H*), 5.71 (1H, dd, ^3J 17.5, ^2J 0.9 Hz, C8``*H*), 5.69 (1H, dd, $^3J$ 17.5, $^2J$ 1.1 Hz, C8`*H*), 5.18 (1H, d, $^3J$ 11.1 Hz, C8`*H*), 5.15 (1H, dd, $^3J$ 11.0, $^2J$ 0.9 Hz, C8``*H*); ^{13}C NMR (126 MHz, $CDCl_3$) δ = 140.5 (*C1``*), 137.9

(*C1`*), 136.9 (*C2`*), 134.86 (*C2``*), 134.85 (*C7``*), 133.2 (*C5``*), 132.7 (*C7a*), 131.9 (*C2*), 131.7 (*C7`*), 131.1 (*C4*), 130.6 (*C6``*), 130.4 (*C3a*), 129.6 (*C6`*), 129.1 (*C4`*), 127.9 (*C4``*), 127.8 (*C5`*), 126.8 (*C3``*), 125.4 (*C3`*), 123.5 (*C6*), 122.4 (*C5*), 117.1 (*C7*), 116.8 (*C8`*), 114.9 (*C8``*), 103.0 (*C3*); ESI-MS: m/z calcd. for $C_{24}H_{17}AgCl_2N$ 495.9783 found 495.9779 [$M^{+\cdot}$].

Synthesis of S9a-o

General Procedure D:

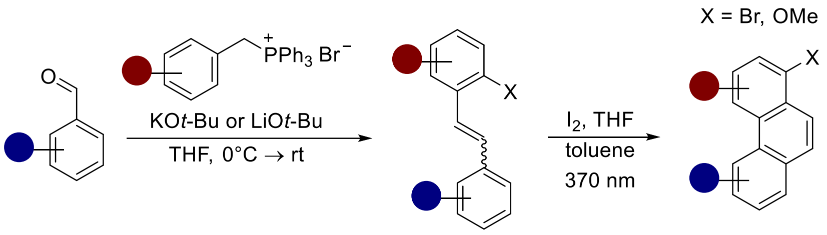

S9a-o

Step 1: According to the modified literature procedure[7]: A mixture of the corresponding phosphorous ylide (1.20 eq.) in THF (0.10 mol·L^{-1} with regard to aldehyde) was cooled to 0°C under argon and dry KO*t*-Bu or LiO*t*-Bu (2.00 eq.) was added portionwise. After stirring the resulting suspension for 60 min at 0°C, a solution of the corresponding aldehyde (1.00 eq.) in THF (0.10 mol·L^{-1}) was added dropwise. The reaction mixture was allowed to warm up to room temperature and stirred for 18 h. Water was then added and the mixture was extracted with EtOAc (×3). The combined organic layer was dried over anhydrous Na_2SO_4, filtered and the solvent was removed under reduced pressure. The residue was purified by flash silica gel column chromatography (cyclohexane/EtOAc 100:0→70:30) to give the desired alkene.

Step 2: According to the modified literature procedure[8]: The corresponding alkene (a mixture of *E* and *Z*-isomers) (1.00 eq.) and I_2 (1.50 eq.) were weighed in the flask which was sealed, evacuated and backfilled with argon before toluene (7.0 mmol·L^{-1}) and THF (30 eq.) were added. The flask was then transfered to the photoreactor utilizing Kessil 370 nm lamp (unless otherwise noted) as a light source and water for cooling. (The set-up was wrapped with alumnium foil for safety reasons.) The resulting solution was stirred upon irradiation (100% intensity) under argon and the reaction progress was monitored via ^{1}H-NMR. Upon the reaction completion, an aqueous saturated solution of $Na_2S_2O_3$ was added (1:1 volume) and the obtained mixture was stirred for 15 minutes and extracted with EtOAc (×3). The combined organic layer was dried over anhydrous Na_2SO_4, filtered and the solvent was removed under reduced pressure. The residue was purified by flash silica gel column chromatography (cyclohexane/EtOAc 100:0→70:30) to give the desired phenanthrene derivative.

1-Bromo-5,7-dimethoxy-4-methylphenanthrene (S9a):

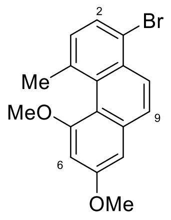

S9a

Step 1 of the general procedure **D** was performed using (2-bromo-5-methylbenzyl)triphenylphosphonium bromide (1.39 g, 2.64 mmol, 1.20 eq.), 3,5-dimethoxybenzaldehyde (366 mg, 2.20 mmol, 1.00 eq.), and KO*t*-Bu (444 mg, 3.96 mmol, 1.80 eq.) to give (*E*, *Z*)-1-bromo-2-(3,5-dimethoxystyryl)-4-methylbenzene as a transparet oil (450 mg, 1.35 mmol, 61%). The obtained alkene (500 mg, 1.50 mmol) was subjected to the step 2 according to the general procedure **D** over 72 h utilizing Kessil-370-Gen2 as a light source to obtain the product **S9a** as white solid (370 mg, 1.12 mmol, 75%) after NP-prep-HPLC (from hexane/EtOAc 98:2 to 80:20, 18 mL/min, 40°C): R_f 0.49 (cyclohexane/EtOAc 6:1); ν_{max} (neat): 299w, 2931w, 2853w, 1601s, 1564s, 1519w, 1452s, 1356s, 1308m, 1282w, 1234w, 1219m, 1160s, 1110m, 1060s, 1024w, 936w, 833m; ^{1}H NMR (500 MHz, $CDCl_3$) δ = 8.04 (1H, d, ^3J 9.0 Hz, C10*H*), 7.66 (1H, d, ^3J 7.9 Hz, C2*H*), 7.55 (1H, d, ^3J 9.0 Hz, C9*H*), 7.27 – 7.22 (1H, m, C3*H*), 6.87 (1H, d, ^4J 2.4 Hz, C8*H*), 6.68 (1H, d, ^4J 2.4 Hz, C6*H*), 3.97 (3H, s, C7–OC*H*$_3$), 3.92 (3H, s, C5–OC*H*$_3$), 2.49 (3H, s, C4–C*H*$_3$); ^{13}C NMR (126 MHz, $CDCl_3$) δ = 159.6 (*C7*), 158.2 (*C5*), 136.3 (*C4*), 135.9 (*C8a*), 131.1 (*C4a*), 130.7 (*C10a*), 129.5 (*C3*), 128.9 (*C2*), 127.3 (*C9*), 126.8 (*C10*), 119.4 (*C1*), 115.4 (*C4b*), 100.1 (*C8*), 98.6 (*C6*), 55.7 (C7–O*C*H$_3$), 55.2 (C5–O*C*H$_3$), 23.9 (C4–*C*H$_3$); ESI-MS: m/z calcd. for $C_{17}H_{16}BrO_2$ 331.0328 found 331.0328 [M+H$^+$].

5-Bromo-9,11-dimethylphenanthro[3,4-d][1,3]dioxole (S9b):

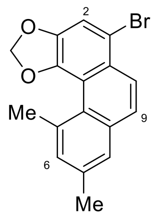

S9b

Step 1 of the general procedure **D** was performed using ((6-bromobenzo[d][1,3]dioxol-5-yl)methyl)triphenylphosphonium bromide (2.20 g, 3.96 mmol, 1.20 eq.), 3,5-dimethylbenzaldehyde (443 mg, 3.30 mmol, 1.00 eq.), and LiO*t*-Bu (528 mg, 6.60 mmol, 2.00 eq.) to give (*E*, *Z*)-5-bromo-6-(3,5-dimethylstyryl)benzo[d]-[1,3]dioxol as a beige solid (912 mg, 2.75 mmol, 83%). The obtained alkene (795 mg, 2.40 mmol) was subjected to the step 2 according to the general procedure **D** over 72 h to obtain the product **S9b** as a beige solid (500 mg, 1.52 mmol, 63%, m.p. 109.2 – 109.7°C): R_f 0.40 (cyclohexane/CH_2Cl_2 5:1); ν_{max}(neat): 3070w, 3010w, 2958w, 2895m, 2784w, 1721w, 1680w, 1592m, 1434s, 1361m, 1265s, 1149m, 1098s, 1040s, 941s, 897m, 846s, 752w, 708m, 648m; ^{1}H NMR (500 MHz, $CDCl_3$) δ 7.91 (1H, d, ^3J 9.1 Hz, C10*H*), 7.49 (1H, s, C2*H*), 7.44 (d, ^3J 9.2 Hz, C9*H*), 7.41 (1H, s, C8*H*), 7.30 (1H, s, C6*H*), 6.13 (2H, s, OC*H*$_2$O), 2.65 (3H, s, C5–C*H*$_3$), 2.50 (3H, s, C7–C*H*$_3$); ^{13}C NMR (126 MHz, $CDCl_3$) δ = 145.4 (*C3*), 143.0 (*C4*), 137.1 (*C7*), 136.6 (*C5*), 133.8 (*C8a*), 132.0 (*C6*), 127.5 (*C10a*), 127.0 (*C9*), 125.2 (*C8*), 125.14 (*C10*), 125.06 (*C4b*), 117.6 (*C4a*), 114.2 (*C1*), 113.2 (*C2*), 100.8 (O*C*H$_2$O), 23.8 (C5–*C*H$_3$), 21.2 (C7–*C*H$_3$); ESI-MS: m/z calcd. for $C_{34}H_{26}AgBr_2O_4$ 762.9243 found 762.9257 [2M+Ag$^+$].

1-Bromo-5,7-dimethoxyphenanthrene (S9c):

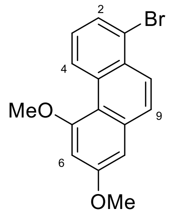

S9c

Step 1 of the general procedure **D** was performed using (2-bromobenzyl)triphenylphosphonium bromide (1.35 g, 2.64 mmol, 1.20 eq.), 3,5-dimethoxybenzaldehyde (366 mg, 2.20 mmol, 1.00 eq.), and KO*t*-Bu (444 mg, 3.96 mmol, 1.80 eq.) to give (*E*, *Z*)-1-(2-bromostyryl)-3,5-dimethoxybenzene as a beige solid (350 mg, 1.10 mmol, 50%, m.p. 121.4 – 122.0°C). The obtained alkene (112 mg, 350 µmol) was subjected to the step 2 according to the general procedure **D** over 48 h utilizing SOLIS-365C as a light source to obtain the product **S9c** as a beige solid (95.0 mg, 300 µmol, 86%): R_f 0.53 (cyclohexane/CH_2Cl_2 5:1); ν_{max}(neat): 3137w, 2996w, 2963w, 2931m, 2851w, 1728w, 1678w, 1612s, 1568s, 1454s, 1358s, 1302s, 1266m, 1199m, 1160s, 1067s, 1024m, 944m, 908w, 838m, 756w; ^{1}H NMR (500 MHz, $CDCl_3$) δ = 9.58 (1H, dt, ^3J 8.7, ^4J 0.9 Hz, C4*H*), 8.24 (1H, dd, ^3J 9.1, ^5J 0.6 Hz, C10*H*), 7.81 (1H, dd, ^3J 7.6, ^4J 0.9 Hz, C2*H*), 7.73 (1H, d, ^3J 9.1 Hz, C9*H*), 7.42 (1H, dd, ^3J 8.7, ^3J 7.6 Hz, C3*H*), 6.93 (1H, d, ^4J 2.5 Hz, C8*H*), 6.81 (1H, d, ^4J 2.5 Hz, C6*H*), 4.10 (3H, s, C5–OC*H*3), 3.97 (3H, s, C7–OC*H*3); ^{13}C NMR (126 MHz, $CDCl_3$) δ = 160.0 (*C5*), 158.8 (*C7*), 135.7 (*C8a*), 132.5 (*C4a*), 130.1 (*C10a*), 129.5 (*C2*), 128.6 (*C9*), 127.5 (*C4*), 126.9 (*C10*, *C3*), 123.2 (*C1*), 115.5 (*C4b*), 101.5 (*C8*), 100.1 (*C6*), 56.0 (C5–O*C*H3), 55.6 (C7–O*C*H3); ESI-MS: m/z calcd. for $C_{16}H_{14}BrO_2$ 317.0172 found 317.0169 [M+H$^+$].

1-Bromo-4,5,7-trimethoxyphenanthrene (S9d):

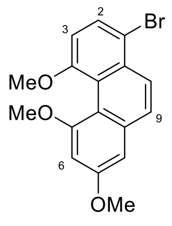

S9d

Step 1 of the general procedure **D** was performed using (2-bromo-5-methoxylbenzyl)triphenylphosphonium bromide (700 mg, 1.29 mmol, 1.20 eq.), 3,5-dimethoxybenzaldehyde (179 mg, 1.08 mmol, 1.00 eq.), and LiO*t*-Bu (172 mg, 2.15 mmol, 2.00 eq.) to give (*E*, *Z*)-1-bromo-2-(3,5-dimethoxystyryl)-4-methoxybenzene as a transparet oil (350 mg, 1.00 mmol, 93%). The obtained alkene (279 mg, 800 µmol) was subjected to the step 2 according to the general procedure **D** over 24 h to obtain the product **S9d** as yellow oil (255 mg, 734 µmol, 92%): R_f 0.26 (cyclohexane/EtOAc 5:1); ν_{max}(neat): 3068w, 2934w, 2832w, 1601s, 1519m, 1460m, 1423m, 1356m, 1323s, 1250s, 1218m, 1159s, 1071s, 1005w, 939w, 834m; ^{1}H NMR (500 MHz, $CDCl_3$) δ = 7.99 (1H, d, ^3J 9.0 Hz, C10*H*), 7.68 (1H, d, ^3J 8.5 Hz, C2*H*), 7.60 (1H, d, ^3J 9.0 Hz, C9*H*), 6.93 (1H, d, ^3J 8.5 Hz, C3*H*), 6.86 (1H, d, ^4J 2.4 Hz, C8*H*), 6.74 (1H, d, ^4J 2.4 Hz, C6*H*), 3.99 (3H, s, C4–OC*H*3), 3.98 (3H, s, C5–OC*H*3), 3.96 (3H, s, C7–OC*H*3); ^{13}C NMR (126 MHz, $CDCl_3$) δ = 159.4 (*C7*), 159.1 (*C5*), 156.9 (*C4*), 135.4 (*C8a*), 131.6 (*C10a*), 129.3 (*C2*), 128.3 (*C9*), 126.1 (*C10*), 121.7 (*C4a*), 113.9 (*C4b*), 113.1 (*C1*), 108.9 (*C3*), 100.1 (*C8*), 99.6 (*C6*), 56.0 (C4–O*C*H3/C5–O*C*H3), 55.99 (C4–O*C*H3/C5–O*C*H3), 55.7 (C7–O*C*H3); ESI-MS: m/z calcd. for $C_{34}H_{30}AgBr_2O_6$ 798.9455 found 798.9458 [2M+Ag$^+$].

1-Bromo-5-methoxy-4,8-dimethylphenanthrene (S9e):

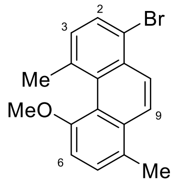

S9e

Step 1 of the general procedure **D** was performed using (2-bromo-5-methylbenzyl)triphenylphosphonium bromide (1.10 g, 2.10 mmol, 1.20 eq.), 5-methoxy-2-methylbenzaldehyde (263 mg, 1.75 mmol, 1.00 eq.), and KO*t*-Bu (353 mg, 3.15 mmol, 1.80 eq.) to give (*E*, *Z*)-1-bromo-2-(5-methoxy-2-methylstyryl)-4-methylbenzene as a transparet oil (350 mg, 1.10 mmol, 63%). The obtained alkene (251 mg, 790 µmol) was subjected to the step 2 according to general procedure **D** over 192 h to obtain the product **S9e** as yellow oil (115 mg, 365 µmol, 46%): R_f 0.67 (cyclohexane/CH_2Cl_2 5:1); ν_{max}(neat): 3081w, 2981w, 2953w, 2927m, 2828w, 1591w, 1563m, 1448s, 1377w, 1299w, 1260s, 1223m, 1167w, 1101s, 1025w, 940w, 867w, 811s; ^{1}H NMR (500 MHz, $CDCl_3$) δ = 8.09 (1H, d, ^3J 9.2 Hz, C10*H*), 7.82 (1H, d, ^3J 9.2 Hz, C9*H*), 7.72 (1H, d, ^3J 7.9 Hz, C2*H*), 7.40 (1H, dd, ^3J 8.0, ^5J 0.8 Hz, C7*H*), 7.27 (d, ^3J 7.9 Hz, C3*H*), 6.95 (1H, d, ^3J 8.0 Hz, C6*H*), 3.91 (3H, s, OCH_3), 2.68 (3H, s, C8–CH_3), 2.50 (3H, s, C4–CH_3); ^{13}C NMR (126 MHz, $CDCl_3$) δ 155.5 (*C5*), 137.2 (*C4*), 133.4 (*C8a*), 131.3 (*C10a*), 131.0 (*C4a*), 129.6 (*C2*), 129.2 (*C3*), 128.7 (*C7*), 126.1 (*C10*), 125.7 (*C8*), 123.6 (*C9*), 120.6 (*C4b*), 119.2 (*C1*), 107.2 (*C6*), 55.1 (O*C*H_3), 23.8 (C4–*C*H_3), 19.2 (C8–*C*H_3); ESI-MS: m/z calcd. for $C_{17}H_{15}BrO$ 314.0301 found 314.0301 [$M^{+\bullet}$].

1-Bromo-4-methoxy-5,8-dimethylphenanthrene (S9f):

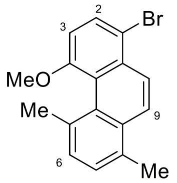

S9f

Step 1 of the general procedure **D** was performed using (2-bromo-5-methoxylbenzyl)triphenylphosphonium bromide (1.30 g, 2.40 mmol, 1.20 eq.), 2,5-dimethylbenzaldehyde (268 mg, 2.00 mmol, 1.00 eq.), and LiO*t*-Bu (320 mg, 4.00 mmol, 2.00 eq.) to give (*E*, *Z*)-1-bromo-2-(2,5-dimethylstyryl)-4-methoxybenzene as a transparet oil (600 mg, 1.89 mmol, 95%). The obtained alkene (381 mg, 1.20 mmol) was subjected to the step 2 according to the general procedure **D** over 192 h to obtain the product **S9f** as a beige solid (75.0 mg, 238 µmol, 20%, m.p. 94.1 – 94.5°C) after NP-prep-HPLC (from hexane/EtOAc 98:2 to 80:20, 18 mL/min, 40°C): R_f 0.57 (cyclohexane/EtOAc 5:1); ν_{max}(neat): 2964w, 2928w, 2862w, 2830w, 1581m, 1520m, 1450s, 1416s, 1378w, 1307s, 1257s, 1098s, 1028m, 975w, 939w, 817s, 712w, 644m; ^{1}H NMR (500 MHz, $CDCl_3$) δ = 8.00 (1H, d, ^3J 9.2 Hz, C10*H*), 7.92 (1H, d, ^3J 9.2 Hz, C9*H*), 7.77 (1H, d, ^3J 8.5 Hz, C2*H*), 7.36 (2H, s, C6*H*, C7*H*), 6.89 (1H, d, ^3J 8.5 Hz, C3*H*), 3.92 (3H, s, OCH_3), 2.72 (3H, s, C8–CH_3), 2.51 (3H, s, C5–CH_3); ^{13}C NMR (126 MHz, $CDCl_3$) δ = 156.5 (*C4*), 135.4 (*C5*), 132.7 (*C10a*), 131.9 (*C8a*), 130.7 (*C8*), 130.4 (*C2*), 128.9 (*C6*), 128.4 (*C4b*), 127.8 (*C7*), 125.2 (*C9*), 124.2 (*C10*), 123.0 (*C4a*), 113.0 (*C1*), 107.7 (*C3*), 55.3 (C4–O*C*H_3), 23.6 (C5–*C*H_3), 19.6 (C8–*C*H_3); ESI-MS: m/z calcd. for $C_{17}H_{15}BrO$ 314.0301 found 314.0297 [$M^{+\bullet}$].

1,8-Dibromo-4,5-dimethoxyphenanthrene (S9g):

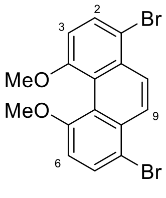

S9g

Step 1 of the general procedure **D** was performed using (2-bromo-5-methoxylbenzyl)triphenylphosphonium bromide (1.30 g, 2.40 mmol, 1.20 eq.), 2-bromo-5-methoxybenzaldehyde (430 mg, 2.00 mmol, 1.00 eq.), and LiO*t*-Bu (320 mg, 4.00 mmol, 2.00 eq.) to give (*E*, *Z*)-1,2-bis(2-bromo-5-methoxyphenyl)ethene as a white solid (754 mg, 1.89 mmol, 95%). The obtained alkene (450 mg, 1.13 mmol) was subjected to the step 2 according to the general procedure **D** over 24 h to obtain the product **S9g** as a white solid (440 mg, 1.11 mmol, 98%, m.p. 90.9 – 88.6°C): R_f 0.28 (cyclohexane/EtOAc 5:1); ν_{max}(neat): 2992w, 2931w, 2832w, 1588wm 1514m, 1443w, 1416m, 1311s, 1240s, 1108w, 1081s, 977w, 907m; ^{1}H NMR (500 MHz, $CDCl_3$) δ = 8.09 (2H, s, C9*H*, C10*H*), 7.78 (2H, d, ^3J 8.5 Hz, C2*H*, C7*H*), 6.97 (2H, d, ^3J 8.5 Hz, C3*H*, C6*H*), 4.00 (6H, s, OC*H*$_3$); ^{13}C NMR (126 MHz, $CDCl_3$) δ = 157.5 (*C4*, *C5*), 132.5 (*C8a*, *C10a*), 130.9 (*C2*, *C7*), 127.1 (*C9*, *C10*), 120.9 (*C4a*, *C4b*), 112.8 (*C1*, *C8*), 109.1 (*C3*, *C6*), 56.1 (C4–O*C*H$_3$, C5–O*C*H$_3$); ESI-MS: m/z calcd. for $C_{16}H_{13}Br_2O_2$ 394.9277 found 394.9720 [M+H$^+$].

1-Methoxy-4,5,7-trimethylphenanthrene (S9h):

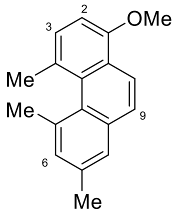

S9h

Step 1 of the general procedure **D** was performed using (3,5-dimethylbenzyl)triphenylphosphonium bromide (997 mg, 2.16 mmol, 1.20 eq.), 2-bromo-5-methoxybenzaldehyde (270 mg, 1.80 mmol, 1.00 eq.), and KO*t*-Bu (364 mg, 3.24 mmol, 1.80 eq.) to give (*E*, *Z*)-2-(3,5-dimethylstyryl)-1-methoxy-4-methylbenzen as a beige solid (380 mg, 1.51 mmol, 84%, m.p. 88.6 – 89.5°C). The obtained alkene (177 mg, 700 µmol) was subjected to the step 2 according to the general procedure **D** over 48 h to obtain the product **S9h** as a white solid (140 mg, 559 µmol, 80%): R_f 0.69 (cyclohexane/EtOAc 5:1); ν_{max}(neat): 2995w, 2949w, 2829w, 1828w, 1588m, 1524w, 1453s, 1376w, 1289s, 1256s, 1165w, 1024s, 907w, 854w, 802w; ^{1}H NMR (500 MHz, $CDCl_3$) δ = 8.04 (1H, d, ^3J 8.9 Hz, C10*H*), 7.49 (1H, d, ^3J 8.9 Hz, C9*H*), 7.47 (1H, s, C8*H*), 7.35 (1H, d, ^3J 8.1 Hz, C3*H*), 7.26 (1H, s, C6*H*), 6.99 (1H, d, ^3J 8.1 Hz, C2*H*), 4.04 (3H, s, OC*H*$_3$), 2.60 (3H, s, C5–C*H*$_3$), 2.56 (3H, s, C4–C*H*$_3$), 2.55 (3H, s, C7–C*H*$_3$); ^{13}C NMR (126 MHz, $CDCl_3$) δ = 153.2 (*C1*), 135.9 (*C5*), 135.6 (*C7*), 134.4 (*C8a*), 131.7 (*C4a*), 130.1 (*C6*), 128.5 (*C4b*), 128.4 (*C3*), 127.6 (*C4*), 125.7 (*C9*), 124.6 (*C8*), 124.3 (*C10a*), 119.9 (*C10*), 106.2 (*C2*), 56.1 (O*C*H$_3$), 22.7 (C5–*C*H$_3$), 22.2 (C4–*C*H$_3$), 21.3 (C7–*C*H$_3$); ESI-MS: m/z calcd. for $C_{18}H_{18}AgO$ 357.0403 found 357.0395 [M+Ag$^+$].

4,5,7-Trimethylphenanthren-1-yl trifluoromethanesulfonate (S9j):

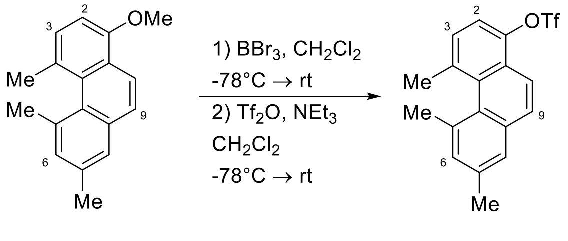

Step 1: According to a modified literature procedure[9]: 1-Methoxy-4,5,7-trimethylphenanthrene **S9h** (125 mg, 500 µmol, 1.00 eq.) was dissolved in dry CH_2Cl_2 (13 mL) and the resulting solution was cooled to –78°C under argon. A solution of BBr_3 in CH_2Cl_2 (1.0 mL, 1.00 mmol, 2.00 eq.,1.0 mol·L^{-1}) and a reaction mixture was left stirring for 18 h. Then water was added and the resulting mixture was extracted with CH_2Cl_2 (3×20 mL). The combined organic layer was dried over anhydrous Na_2SO_4, filtered and the solvent was removed under reduced pressure. The residue was purified by silica gel column chromatography (cyclohexane/EtOAc 100:0→60:40) to give the desired product 4,5,7-trimethylphenanthren-1-ol **S7i** as yellow oil (58.0 mg, 245 µmol, 49%): R_f 0.47 (cyclohexane/EtOAc 3:1); ν_{max}(neat): 3501w, 3394w, 2995w, 2924w, 1596m, 1528w, 1450s, 1387w, 1331w, 1291s, 1215w, 1151w, 1079w, 871w, 803w, 710w; ^{1}H NMR (500 MHz, $CDCl_3$) δ = 7.92 (1H, d, ^3J 8.9 Hz, C10*H*), 7.48 (1H, d, ^3J 8.9 Hz, C9*H*), 7.46 (1H, s, C8*H*), 7.27 – 7.24 (2H, m, C3*H*, C6*H*), 6.93 (1H, d, ^3J 7.9 Hz, C2*H*), 5.09 (1H, s, O*H*), 2.58 (3H, s, C5–C*H*$_3$), 2.54 (6H, s, C4–C*H*$_3$, C7–C*H*$_3$); ^{13}C NMR (126 MHz, $CDCl_3$) δ = 149.0 (*C1*), 136.0 (*C5*), 135.8 (*C7*), 134.3 (*C8a*), 131.9 (*C4a*), 130.2 (*C6*), 128.6 (*C4b*), 128.5 (*C3*), 128.1 (*C4*), 125.9 (*C9*), 124.7 (*C8*), 122.9 (*C10a*), 119.4 (*C10*), 110.9 (*C2*), 22.7 (C5–*C*H$_3$), 22.3 (C4–*C*H$_3$), 21.3 (C7–*C*H$_3$); ESI-MS: m/z calcd. for $C_{17}H_{16}NaO$ 259.1093 found 259.1088 [M+Na$^+$].

Step 2: According to a modified literature procedure[10]: A solution of 4,5,7-trimethylphenanthren-1-ol (78.0 mg, 330 µmol, 1.00 eq.) and NEt_3 (67.0 mg, 660 µmol, 2.00 eq.) in dry CH_2Cl_2 (3.0 mL) was cooled to –78°C. Trifluoromethanesulfonic anhydride (158 mg, 561 µmol, 1.70 eq.) was added dropwise and the reaction mixture for stirred for 18 h. A saturated aqueous solution of NH_4Cl was then added and the mixture was extracted with CH_2Cl_2 (3×20 mL). The combined organic layer was dried over anhydrous Na_2SO_4, filtered and the solvent was removed under reduced pressure. The residue was purified by silica gel column chromatography (cyclohexane/EtOAc 100:0→80:20) to give the desired product 4,5,7-trimethylphenanthren-1-yl trifluoromethanesulfonate **S9j** as yellow oil (106 mg, 289 µmol, 87%): R_f 0.75 (cyclohexane/EtOAc 6:1); ν_{max}(neat): 2992w, 2923w, 1861w, 1738w, 1657w, 1612w, 1418s, 1208s, 1139s, 1052m, 960w, 890s, 828s; ^{1}H NMR (500 MHz, $CDCl_3$) δ = 7.72 (1H, d, ^3J 8.9 Hz, C10*H*), 7.60 (1H, d, ^3J 8.9 Hz, C9*H*), 7.48 (1H, s, C8*H*), 7.43 – 7.40 (2H, m, C2*H*, C3*H*), 7.31 (1H, s, C6*H*), 2.60 (3H, s, C4–C*H*$_3$), 2.55 (3H, s, C7–C*H*$_3$), 2.53 (3H, s, C5–C*H*$_3$); ^{13}C NMR (126 MHz, $CDCl_3$) δ = 143.4 (*C1*), 136.9 (*C5*), 136.1 (*C4*), 136.0 (*C7*), 133.9 (*C8a*), 132.6 (*C4a*), 131.3 (*C6*), 129.0 (*C9*), 128.0 (*C3*), 127.9 (*C4b*), 126.2 (*C10a*), 125.2 (*C8*), 118.0 (*C10*), 117.7 (*C2*), 22.9 (C4–*C*H$_3$), 22.7 (C7–*C*H$_3$), 21.3 (C5–*C*H$_3$); ^{19}F NMR (376 MHz, $CDCl_3$) δ –73.34; ESI-MS: m/z calcd. for $C_{18}H_{15}F_3NaO_3S$ 391.0586 found 391.0580 [M+Na$^+$].

5,8-Dibromo-11-methoxyphenanthro[3,4-d][1,3]dioxole (S9k):

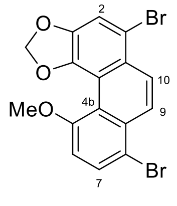

S9k

Step 1 of the general procedure **D** was performed using ((6-bromo-benzo[d][1,3]dioxol-5-yl)methyl)triphenylphosphonium bromide (1.00 g, 1.80 mmol, 1.20 eq.), 2-bromo-5-methoxybenzaldehyde (323 mg, 1.50 mmol, 1.00 eq.), and LiO*t*-Bu (240 mg, 3.00 mmol, 2.00 eq.) to give (*E*, *Z*)-5-bromo-6-(2-bromo-5-methoxy-styryl)benzo[d][1,3]dioxole as a transparent oil (465 mg, 1.13 mmol, 75%). The obtained alkene (350 mg, 850 µmol) was subjected to the step 2 according to the general procedure **D** over 200 h to obtain the

product **S9k** as a white solid (130 mg, 317 µmol, 37%, m.p. 125.3 – 126.0°C): R_f 0.50 (cyclohexane/EtOAc 4:1); ν_{max} (neat): 2999w, 2962w, 2902w. 2837w, 1863w, 1721w, 1676w, 1573m, 1430s, 1350w, 1273s, 1165w, 1105m, 1049s, 997w, 940m, 845m, 792m; ^{1}H NMR (500 MHz, $CDCl_3$) δ = 8.10 (1H, d, ^3J 9.5 Hz, C10*H*), 7.92 (1H, d, ^3J 9.5 Hz, C9*H*), 7.80 (1H, d, ^3J 8.5 Hz, C7*H*), 7.53 (1H, s, C2*H*), 6.97 (1H, d, ^3J 8.5 Hz, C6*H*), 6.16 (2H, s, OCH_2O), 4.01 (3H, s, OCH_3); ^{13}C NMR (126 MHz, $CDCl_3$) δ = 157.1 (*C5*), 146.3 (*C3*), 144.3 (*C4*), 132.8 (*C8a*), 132.0 (*C7*), 128.0 (*C10*), 126.9 (*C10a*), 124.7 (*C9*), 119.6 (*C4b*), 115.4 (*C4a*), 114.3 (*C1*), 114.0 (*C2*), 113.0 (*C8*), 109.5 (*C6*), 101.2 (O*C*H_2O), 56.1 (O*C*H_3); ESI-MS: m/z calcd. for $C_{16}H_{10}Br_2NaO_3$ 430.8889 found 430.8887 [M+Na$^+$].

1,8-Dibromo-4-methoxy-5-methylphenanthrene (S9l):

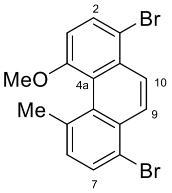

S9l

Step 1 of the general procedure **D** was performed using (2-bromo-5-methylbenzyl)triphenylphosphonium bromide (947 mg, 1.80 mmol, 1.20 eq.), 2-bromo-5-methoxybenzaldehyde (323 mg, 1.50 mmol, 1.00 eq.), and LiO*t*-Bu (240 mg, 3.00 mmol, 2.00 eq.) to give (*E*, *Z*)-1-bromo-2-(2-bromo-5-methoxystyryl)-4-methylbenzene as a transparent oil (435 mg, 1.14 mmol, 76%). The obtained alkene (275 mg, 720 µmol) was subjected to the step 2 according to the general procedure **D** over 48 h utilizing Kessil-370-Gen2 as a light source to obtain the product **S9l** as a beige solid (150 mg, 395 µmol, 55%, m.p. 94.3 – 95.6°C): R_f 0.63 (cyclohexane/EtOAc 5:1); ν_{max} (neat): 2967w, 2929w, 2851w, 1587w, 1558w, 1446m, 1417s, 1300s, 1259s, 1231s, 1098s, 1022m, 980w, 934w, 811m; ^{1}H NMR (500 MHz, $CDCl_3$) δ = 8.15 (1H, d, ^3J 9.3 Hz, C9*H*), 8.05 (1H, d, ^3J 9.3 Hz, C10*H*), 7.80 (1H, d, ^3J 8.5 Hz, C2*H*), 7.75 (1H, d, ^3J 7.9 Hz, C7*H*), 7.29 (1H, d, ^3J 7.9 Hz, C6*H*), 6.92 (1H, d, ^3J 8.5 Hz, C3*H*), 3.93 (3H, s, OCH_3), 2.48 (3H, s, C5–CH_3); ^{13}C NMR (126 MHz, $CDCl_3$) δ = 156.4 (*C4*), 137.3 (*C5*), 133.0 (*C10a*), 131.5 (*C8a*), 131.3 (*C2*), 130.4 (*C4b*), 130.3 (*C7*), 129.7 (*C6*), 127.8 (*C9*), 126.0 (*C10*), 122.4 (*C4a*), 119.3 (*C8*), 113.1 (*C1*), 108.3 (*C3*), 55.4 (O*C*H_3), 23.7 (C7–*C*H_3); ESI-MS: m/z calcd. for $C_{16}H_{13}Br_2O$ 378.9328 found 378.9318 [M+H$^+$].

1,8-Dimethoxy-4,5-dimethylphenanthrene (S9m):

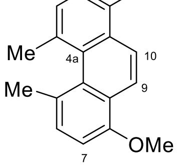

S9m

Step 1 of the general procedure **D** was performed using (2-methoxy-5-methylbenzyl)triphenylphosphonium bromide (1.72 g, 3.60 mmol, 1.20 eq.), 2-methoxy-5-methylbenzaldehyde (451 mg, 3.00 mmol, 1.00 eq.), and KO*t*-Bu (606 mg, 5.40 mmol, 1.80 eq.) to give (*E*, *Z*)-1,2-bis(5-methoxy-2-methylphenyl)ethene as a transparent oil (760 mg, 2.83 mmol, 94%). The obtained alkene (322 mg, 120 mmol) was subjected to the step 2 according to the general procedure **D** over 72 h to obtain the product **S9m** as a white solid (110 mg, 413 µmol, 34%, m.p. 155.2 – 157.3°C): R_f 0.54 (cyclohexane/EtOAc 5:1); ν_{max} (neat): 3006w, 2935w, 2842w, 1580s, 1526w, 1493m, 1451m, 1427s, 1378w, 1326w, 1291m, 1245s, 1214m, 1142s, 1079w, 1028s, 911w, 801s; ^{1}H NMR (500 MHz, $CDCl_3$) δ = 8.03 (2H, s, C9*H*, C10*H*), 7.30

(2H, d, ^3J 8.1 Hz, C3*H*, C6*H*), 6.95 (2H, d, ^3J 8.1 Hz, C2*H*, C7*H*), 4.01 (6H, s, 2 × OCH_3), 2.52 (6H, s, 2 × CH_3); ^{13}C NMR (126 MHz, $CDCl_3$) δ = 153.1 (*C1*, *C8*), 131.5 (*C4a*, *C4*b), 128.2 (*C3*, *C6*), 127.9 (*C4*, *C5*), 124.9 (*C8a*, *C10a*), 119.2 (*C9*, *C10*), 106.4 (*C2*, *C7*), 56.0 (O*C*H_3), 22.1 (*C*H_3); ESI-MS: m/z calcd. for $C_{18}H_{18}AgO_2$ 373.0352 found 3763.0348 [M+Ag$^+$].

4,5-Dimethylphenanthrene-1,8-diyl bis(trifluoromethanesulfonate) (S9o):

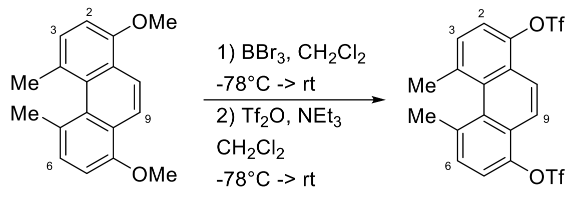

Step 1: According to a modified literature procedure[9]: 1,8-Dimethoxy-4,5-dimethylphenanthrene (394 mg, 1.48 mmol, 1.00 eq.) was dissolved in dry CH_2Cl_2 (37 mL) and the resulting solution was cooled to –78°C under argon. A solution of BBr_3 in CH_2Cl_2 (5.9 mL, 5.92mmol, 4.00 eq.,1.0 mol·L^{-1}) and a reaction mixture was left stirring for 18 h. Then water was added and the resulting mixture was extracted with CH_2Cl_2 (3×20 mL). The combined organic layer was dried over anhydrous Na_2SO_4, filtered and the solvent was removed under reduced pressure. The residue was purified by silica gel column chromatography (cyclohexane/EtOAc 100:0→60:40) to give the desired product 4,5-dimethylphenanthrene-1,8-diol **S9n** as a white solid (112 mg, 470 µmol, 32%, m.p. 205.5 – 206.0°C (decomposition)): R_f 0.33 (cyclohexane/EtOAc 5:2); ν_{max} (neat): 3216w, 2929w, 1847w, 1707w, 1590m, 1498w, 1446w, 1400m, 1337m, 1291m, 1243s, 1155m, 1121s, 972m, 854s, 806s; ^{1}H NMR (500 MHz, $CDCl_3$) δ = 7.94 (2H, s, C9*H*, C10*H*), 7.24 (2H, d, ^3J 7.8 Hz, C3*H*, C6*H*), 6.93 (1H, d, ^3J 7.8 Hz, C2*H*, C7*H*), 5.12 (2H, s, O*H*), 2.51 (6H, s, OCH_2); ^{13}C NMR (126 MHz, $CDCl_3$) δ = 148.9 (*C1*, *C8*), 131.7 (*C4a*, *C4b*), 128.6 (*C4*, *C5*), 128.5 (*C3*, *C6*), 123.4 (*C8a*, *C10a*), 118.9 (*C9*, *C10*), 111.3 (*C2*, *C7*), 22.2 (CH_3); ESI-MS: m/z calcd. for $C_{16}H_{13}O_2$ 237.0921 found 237.0925 [M–H$^+$].

Step 2: According to a modified literature procedure[10]: A solution of 4,5-dimethylphenanthrene-1,8-diol (62.0 mg, 260 µmol, 1.00 eq.) and NEt_3 (78.9 mg, 780 µmol, 3.00 eq.) in dry CH_2Cl_2 (2.0 mL) was cooled to –78°C. Trifluoromethanesulfonic anhydride (183 mg, 650 µmol, 2.50 eq.) was added dropwise and the reaction mixture for stirred for 18 h. A saturated aqueous solution of NH_4Cl was then added and the mixture was extracted with CH_2Cl_2 (3×20 mL). The combined organic layer was dried over anhydrous Na_2SO_4, filtered and the solvent was removed under reduced pressure. The residue was purified by silica gel column chromatography (cyclohexane/EtOAc 100:0→80:20) to give the desired product 4,5-dimethylphenanthrene-1,8-diyl bis(trifluoromethanesulfonate **S9o** as a white solid (91.0 mg, 181 µmol, 70%, m.p. 98.3 – 99.0°C): R_f 0.45 (cyclohexane/EtOAc 5:1); ν_{max} (neat): 2973m, 2920w, 1593w, 1420s, 1210s, 1137s, 1086s, 966m, 883m, 813m, 652w; ^{1}H NMR (500 MHz, $CDCl_3$) δ = 7.93 (2H, s, C9*H*, C10*H*), 7.60 – 7.46 (4H, m, C2*H*, C3*H*, C6*H*, C7*H*), 2.61 (6H, s, C4–CH_3, C5–CH_3); ^{13}C NMR (126 MHz, $CDCl_3$) δ = 143.3 (*C1*, *C8*), 136.8 (*C4*, *C5*), 131.7 (*C4a*, *C4b*), 129.3 (*C3*, *C6*), 126.5 (*C10a*, *C8a*), 120.7 (*C9*, *C10*), 119.3 (*C2*, *C7*), 22.8 (C4–CH_3, C5–CH_3); ^{19}F NMR (376 MHz, $CDCl_3$) δ = –73.19; ESI-MS: m/z calcd. for $C_{18}H_{12}F_6KO_6S_2$ 540.9611 found 540.9609 [M+K$^+$].

General Procedure E for the Synthesis of 15a-k, 17a-i:

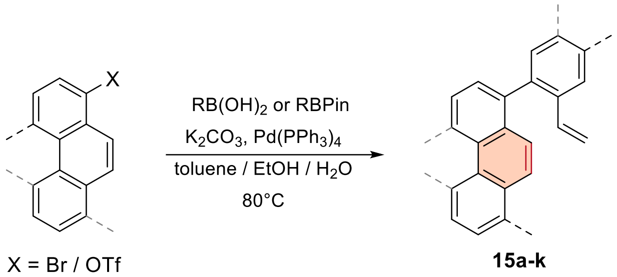

The bromide/triflate **S9** (1.00 eq.), the corresponding boronic acid or ester (1.11 eq.), $Pd(PPh_3)_4$ (5 mol%), and K_2CO_3 (3.00 eq.) were weighed into a 10 or 20 mL crimp cap vial. The vial was sealed, evacuated and backfilled with argon before a degassed mixture of toluene, H_2O, ethanol (2 : 2 : 1) (0.12 mol·L^{-1}) was added. The resulting mixture was stirred at 80°C for 18 h, then cooled to room temperature, filtered through a thin layer of silica gel and Na_2SO_4 using EtOAc (15-20 mL). The solvents were removed under reduced pressure and the residue was purified by the silica gel chromatography (cyclohexane/EtOAc 100:0→80:20) to give the desired product.

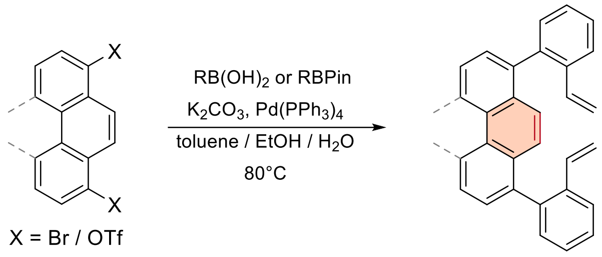

The bromide/triflate **S9** (1.00 eq.), the corresponding boronic acid or ester (2.80 eq.), $Pd(PPh_3)_4$ (5 mol%), and K_2CO_3 (4.00 eq.) were weighed into a 10 or 20 mL crimp cap vial. The vial was sealed, evacuated and backfilled with argon before a degassed mixture of toluene, H_2O, ethanol (2 : 2 : 1) (0.12 mol·L^{-1}) was added. The resulting mixture was stirred at 80°C for 18 h, then cooled to room temperature, filtered through a thin layer of silica gel and Na_2SO_4 using EtOAc (15-20 mL). The solvents were removed under reduced pressure and the residue was purified by the silica gel chromatography (cyclohexane/EtOAc 100:0→80:20) to give the desired product.

5,7-Dimethoxy-4-methyl-1-(2-vinylphenyl)phenanthrene (15a):

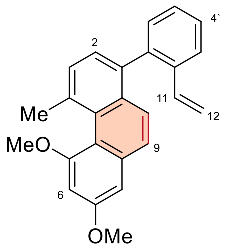

15a

Prepared according to the general procedure **E** using 1-bromo-5,7-dimethoxy-4-methylphenanthrene **S9a** (66.2 mg, 200 µmol, 1.00 eq.) and (2-vinylphenyl)boronic acid (32.8 mg, 222 µmol, 1.11 eq.) to give **15a** as yellow oil (56.0 mg, 158 µmol, 79%): R_f 0.63 (cyclohexane/EtOAc 6:1); ν_{max} (neat): 3049w, 2996w, 2960w, 2856w, 1603s, 1526m, 1453m, 1424m, 1356m, 1274m, 1216m, 1159s, 1109m, 1057m, 988w, 908m, 832m, 730m; ^{1}H NMR (500 MHz, $CDCl_3$) δ = 7.74 (1H, dd, ^{3}J 7.9, ^{4}J 1.2 Hz, C3\`*H*), 7.46 (1H, $^{3}J$ 7.0 Hz, C3*H*), 7.44 – 7.39 (1H, m, C4\`*H*), 7.38 – 7.31 (2H, m, C5\`*H*, C9*H*), 7.31 – 7.26 (2H, m, C6\`*H*, C2*H*), 7.23 (1H, d, ^{3}J 8.9 Hz, C10*H*), 6.83 (1H, d, ^{4}J 2.4 Hz, C8*H*), 6.70 (1H, d, ^{4}J 2.4 Hz, C6*H*), 6.41 (1H, dd, ^{3}J 17.5, ^{3}J 11.0 Hz, C11*H*), 5.66 (1H, dd, ^{3}J 17.5, ^{2}J 1.2 Hz, C12*H*), 4.98 (1H, dd, ^{3}J 11.0, ^{2}J 1.2 Hz, C12*H*), 3.97 (3H, s, C5–OCH_3), 3.96 (3H, s, C7–OCH_3), 2.60 (3H, s, C4–CH_3); ^{13}C NMR (126 MHz, $CDCl_3$) δ = 159.2 (*C7*), 158.2 (*C5*), 140.2 (*C1\`*), 137.2 (*C2\`*), 136.0 (*C4*), 135.7 (*C8a*, *C11*), 135.4 (*C1*), 131.7 (*C6\`*), 131.0 (*C10a*), 129.2 (*C4a*), 128.6 (*C3*), 127.7 (*C4\`*), 127.6 (*C5\`*), 126.9 (*C2*), 126.5 (*C10*), 125.7 (*C9*), 124.8

(*C3`*), 115.7 (*C4b*), 114.4 (*C12*), 100.0 (*C8*), 98.1 (*C6*), 55.6 (C7–O*C*H_3), 55.1 (C5–O*C*H_3), 24.2 (C4–*C*H_3); ESI-MS: m/z calcd. for $C_{25}H_{22}AgO_2$ 461.0665 found 461.0673 [M+Ag^+].

9,11-Dimethyl-5-(2-vinylphenyl)phenanthro[3,4-d][1,3]dioxole (15b):

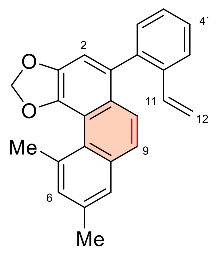

15b

Prepared according to the general procedure **E** using 5-bromo-9,11-dimethylphenanthro[3,4-d][1,3]dioxole **S9b** (165 mg, 500 µmol, 1.00 eq.) and (2-vinylphenyl)boronic acid (82.1 mg, 555 µmol, 1.11 eq.) to give **15b** as a beige solid (150 mg, 426 µmol, 85%, m.p. 140.7 – 142.0°C): R_f 0.64 (cyclohexane/EtOAc 7:1); ν_{max} (neat): 3055w, 2975w, 2872w, 2769w, 1747w, 1622w, 1588m, 1435s, 1382s, 1263s, 1103s, 1038s, 998m, 942m, 866m; ^{1}H NMR (500 MHz, $CDCl_3$) δ = 7.73 (1H, d, ^3J 7.8 Hz, C3`*H*), 7.43 (1H, t, $^3J$ 7.6 Hz, C4`*H*), 7.39 – 7.33 (2H, m, C5`*H*, C8*H*), 7.32 – 7.27 (2H, m, C6`*H*, C6*H*), 7.22 (1H, d, ^3J 9.1 Hz, C9*H*), 7.12 – 7.06 (2H, m, C10*H*, C2*H*), 6.41 (1H, dd, ^3J 17.5, ^3J 11.0 Hz, C11*H*), 6.17 (2H, dd, ^2J 9.5, ^5J 1.4 Hz, OCH_2O), 5.67 (1H, d, ^3J 17.5 Hz, C12*H*), 5.03 (1H, d, ^3J 11.0 Hz, C12*H*), 2.74 (3H, s, C5–CH_3), 2.49 (3H, s, C7–CH_3); ^{13}C NMR (126 MHz, $CDCl_3$) δ = 144.8 (*C3*), 142.6 (*C4*), 139.8 (*C1`*), 137.2 (*C2`*), 136.61 (*C7*), 136.58 (*C5*), 135.4 (*C11*), 133.7 (*C10a*), 132.7 (*C1*), 131.55 (*C6*), 131.53 (*C6`*), 128.01 (*C4b*), 127.96 (*C4`*), 127.7 (*C5`*), 125.38 (*C8a*), 125.32 (*C9*), 125.0 (*C8, C3`*), 124.8 (*C10*), 116.4 (*C4a*), 114.8 (*C12*), 110.8 (*C2*), 100.3 (O*C*H_2O), 24.0 (C5–*C*H_3), 21.2 (C7–*C*H_3); ESI-MS: m/z calcd. for $C_{25}H_{20}AgO_2$ 459.0509 found 459.0507 [M+Ag^+].

5,7-Dimethoxy-1-(2-vinylphenyl)phenanthrene (15c):

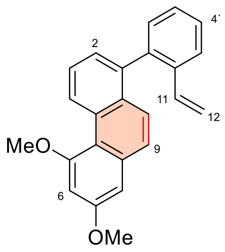

15c

Prepared according to the general procedure **E** using 1-bromo-5,7-dimethoxyphenanthrene **S9c** (79.3 mg, 250 µmol, 1.00 eq.) and (2-vinylphenyl)boronic acid (41.1 mg, 278 µmol, 1.11 eq.) to give **15c** as a beige solid (70.0 mg, 206 µmol, 82%, m.p. 180.0 – 181.0°C): R_f 0.51 (cyclohexane/EtOAc 5:1); ν_{max} (neat): 3054w, 3003w, 2938w, 2835w, 1834w, 1735w, 1609s, 1528m, 1453s, 1311s, 1199s, 1157s, 1103m, 1060m, 954m, 914m, 864m, 807m, 764m; ^{1}H NMR (500 MHz, $CDCl_3$) δ = 9.63 (1H, d, ^3J 8.7 Hz, C4*H*), 7.76 (1H, d, ^3J 7.8 Hz, C3`*H*), 7.64 (1H, dd, $^3J$ 8.7, $^3J$ 7.1 Hz, C3*H*), 7.50 (1H, d, $^3J$ 9.1 Hz, C9*H*), 7.47 – 7.42 (1H, m, C4`*H*), 7.41 – 7.35 (3H, m, C2*H*, C10*H*, C5`*H*), 7.31 (1H, dd, $^3J$ 7.5, $^4J$ 1.4 Hz, C6`*H*), 6.87 (1H, d, ^4J 2.5 Hz, C8*H*), 6.81 (1H, d, ^4J 2.5 Hz, C6*H*), 6.32 (1H, dd, ^3J 17.5, ^3J 11.0 Hz, C11*H*), 5.65 (1H, dd, ^3J 17.5, ^2J 1.1 Hz, C12*H*), 4.97 (1H, dd, ^3J 11.0, ^2J 1.1 Hz, C12*H*), 4.14 (3H, s, C5–OCH_3), 3.95 (3H, s, C7–OCH_3); ^{13}C NMR (126 MHz, $CDCl_3$) δ = 160.2 (*C5*), 158.5 (*C7*), 140.5 (*C1`*), 138.8 (*C1*), 137.0 (*C2`*), 135.4 (*C11*), 131.2 (*C6`*), 130.7 (*C4a*), 130.4 (*C10a*), 127.8 (*C4`*), 127.4 (*C5`*), 127.3 (*C4*), 127.0 (*C9*), 126.9 (*C2*), 126.3 (*C10*), 125.8 (*C3*), 124.8 (*C3`*), 115.9 (*C4b*), 114.5 (*C12*), 101.4 (*C8*), 99.7 (*C6*), 56.0 (C5–O*C*H_3), 55.6 (C7–O*C*H_3); ESI-MS: m/z calcd. for $C_{24}H_{20}AgO_2$ 447.0509 found 447.0503 [M+Ag^+].

4,5,7-Trimethoxy-1-(2-vinylphenyl)phenanthrene (15d):

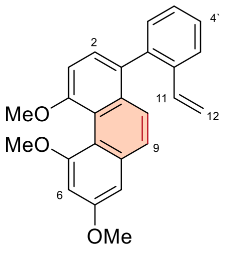

15d

Prepared according to the general procedure **E** using 1-bromo-4,5,7-trimethoxyphenanthrene **S9d** (86.8 mg, 250 µmol, 1.00 eq.) and (2-vinylphenyl)boronic acid (41.1 mg, 278 µmol, 1.11 eq.) to give **15d** as yellow solid (65.6 mg, 177 µmol, 71%, m.p. 86.0 – 86.5°C): R_f 0.55 (cyclohexane/EtOAc 5:1); ν_{max} (neat): 3085w, 2988w, 2933w, 2830w, 1600m, 1525m, 1452m, 1424m, 1353w, 1320w, 1243m, 1215m, 1156s, 1064s, 986w, 912w, 835w, 758w; ^{1}H NMR (500 MHz, $CDCl_3$) δ = 7.77 – 7.70 (1H, m, C3`*H*), 7.44 – 7.40 (1H, m, C4`*H*), 7.38 (1H, d, ^3J 9.1 Hz, C9*H*), 7.37 – 7.33 (1H, m, C5`*H*), 7.32 – 7.28 (2H, m, C2*H*, C6`*H*), 7.20 (1H, d, ^3J 9.1 Hz, C10*H*), 7.12 (1H, d, ^3J 8.1 Hz, C3*H*), 6.81 (1H, d, ^4J 2.4 Hz, C8*H*), 6.75 (1H, d, ^4J 2.4 Hz, C6*H*), 6.42 (1H, dd, ^3J 17.5, ^3J 11.0 Hz, C11*H*), 5.67 (1H, d, ^2J 1.3 Hz, C12*H*), 4.99 (1H, dd, ^3J 11.0, ^2J 1.2 Hz, C12*H*), 4.07 (3H, s, C4–OCH_3), 4.04 (3H, s, C5–OCH_3), 3.94 (3H, s, C7–OCH_3); ^{13}C NMR (126 MHz, $CDCl_3$) δ = 159.13 (*C5*), 159.05 (*C7*), 156.8 (*C4*), 139.9 (*C1`*), 137.4 (*C2`*), 135.7 (*C11*), 135.1 (*C8a*), 132.1 (*C10a*), 131.8 (*C6`*), 130.4 (*C1*), 127.7 (*C2*), 127.60 (*C4`*), 127.57 (*C5`*), 126.8 (*C9*), 125.8 (*C10*), 124.8 (*C3`*), 119.6 (*C4a*), 114.3 (*C12*), 114.2 (*C4b*), 107.7 (*C3*), 100.0 (*C8*), 99.1 (*C6*), 55.96 (C5–OCH_3), 55.91 (C4–OCH_3), 55.7 (C7–OCH_3); ESI-MS: m/z calcd. for $C_{25}H_{22}AgO_3$ 477.0614 found 477.0608 [M+Ag^+].

4-Methoxy-1,5-dimethyl-8-(2-vinylphenyl)phenanthrene (15e):

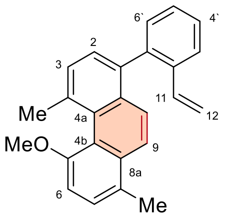

15e

Prepared according to the general procedure **E** using 1-bromo-5-methoxy-4,8-dimethylphenanthrene **S9e** (107 mg, 340 µmol, 1.00 eq.) and (2-vinylphenyl)boronic acid (55.8 mg, 278 µmol, 1.11 eq.) to give **15e** as a white solid (88.0 mg, 260 µmol, 77%, m.p. 80.5 – 81.5°C): R_f 0.64 (cyclohexane/EtOAc 7:1); ν_{max} (neat): 3057w, 2949w, 2929w, 2831w, 2730w, 1577m, 1527m, 1450m, 1427s, 1376w, 1327m, 1258s, 1219m, 1164w, 1098s, 1021w, 980w, 912w, 815s, 759s; ^{1}H NMR (500 MHz, $CDCl_3$) δ = 7.75 (1H, d, ^3J 6.8 Hz, C3`*H*), 7.59 (1H, d, $^3J$ 9.2 Hz, C9*H*), 7.48 (1H, d, $^3J$ 7.4 Hz, C3*H*), 7.42 (1H, dd, $^3J$ 7.6, $^4J$ 1.3 Hz, C4`*H*), 7.39 – 7.32 (3H, m, C2*H*, C7*H*, C5`*H*), 7.32 – 7.27 (2H, m, C10*H*, C6`*H*), 6.96 (1H, d, ^3J 8.0 Hz, C6*H*), 6.42 (1H, dd, ^3J 17.5, ^3J 11.0 Hz, C11*H*), 5.67 (1H, dd, ^3J 17.5, ^2J 1.2 Hz, C12*H*), 4.99 (1H, dd, ^3J 11.0, ^2J 1.1 Hz, C12*H*), 3.97 (3H, s, C5–OCH_3), 2.62 (3H, s, C8–CH_3), 2.61 (3H, C4–CH_3); ^{13}C NMR (126 MHz, $CDCl_3$) δ = 155.5 (*C5*), 140.0 (*C1`*), 137.2 (*C2`*), 137.0 (*C4*), 135.6 (*C11*), 135.1 (*C1*), 133.2 (*C8a*), 131.7 (*C6`*), 131.6 (*C10a*), 129.1 (*C4a*), 128.3 (*C3*), 128.1 (*C7*), 127.8 (*C4`*), 127.6 (*C5`*), 127.5 (*C2*), 125.7 (*C10*), 125.5 (*C8*), 124.8 (*C3`*), 121.9 (*C9*), 120.9 (*C4b*), 114.4 (*C12*), 106.6 (*C6*), 55.0 (OCH_3), 24.0 (C4–CH_3), 19.2 (C8–CH_3); ESI-MS: m/z calcd. for $C_{25}H_{22}O$ 338.1665 found 338.1659 [M^+].

4-Methoxy-5,8-dimethyl-1-(2-vinylphenyl)phenanthrene (15f):

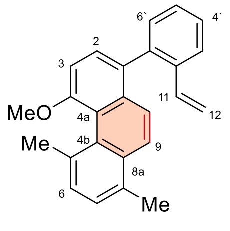

15f

Prepared according to the general procedure **E** using 1-bromo-4-methoxy-5,8-dimethylphenanthrene **S9f** (59.9 mg, 190 µmol, 1.00 eq.) and (2-vinylphenyl)boronic acid (31.2 mg, 211 µmol, 1.11 eq.) to give **15f** as a transparent oil (52.0 mg, 154 µmol, 81%): R_f 0.77 (cyclohexane/EtOAc 5:1); ν_{max} (neat): 3060w, 3017w, 2956w, 2928w, 2863w, 2831w, 1681w, 1578m, 1526m, 1453s, 1381w, 1305s, 1252s, 1219m, 1099s, 1026w, 993w, 908m, 820m, 761m, 729s, 630s; ^{1}H NMR (500 MHz, $CDCl_3$) δ = 7.78 – 7.73 (1H, M, C3`*H*), 7.69 (1H, d, $^3J$ 9.2 Hz, C9*H*), 7.48 – 7.41 (1H, m, C4`*H*), 7.39 (1H, d, ^3J 8.0 Hz, C2*H*), 7.37 – 7.29 (4H, m, C6*H*, C7*H*, C5`*H*, C6`*H*), 7.21 (1H, d, ^3J 9.2 Hz, C10*H*), 7.07 (1H, d, ^3J 8.0 Hz, C3*H*), 6.43 (1H, dd, ^3J 17.6, ^3J 11.0 Hz, C11*H*), 5.67 (1H, dd, ^3J 17.6, ^2J 1.2 Hz, C12*H*), 5.01 (1H, dd, ^3J 11.0, ^2J 1.2 Hz, C12*H*), 3.99 (3H, s, OCH_3), 2.65 (3H, s, C8–CH_3), 2.59 (3H, s, C5–CH_3); ^{13}C NMR (126 MHz, $CDCl_3$) δ = 156.5 (*C4*), 139.8 (*C1`*), 137.4 (*C2`*), 135.6 (*C11*), 135.4 (*C5*), 133.1 (*C10a*), 131.9 (*C6`*), 131.7 (*C8a*), 130.5 (*C8*), 130.2 (*C1*), 128.8 (*C4b*), 128.59 (*C2*/*C6*), 128.52 (*C2*/*C6*), 127.8 (*C4`*), 127.6 (*C5`*), 127.3 (*C7*), 124.9 (*C3`*), 123.9 (*C10*), 123.6 (*C9*), 121.0 (*C4a*), 114.5 (*C12*), 106.4 (*C3*), 55.0 (O*C*H_3), 23.8 (C5–*C*H_3), 19.6 (C8–*C*H_3); ESI-MS: m/z calcd. for $C_{25}H_{22}AgO$ 445.0716 found 445.0720 [M+Ag^+].

1-(5-Chloro-2-vinylphenyl)-5,7-dimethoxy-4-methylphenanthrene (15g):

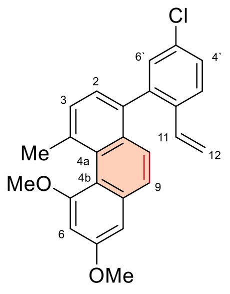

15g

Prepared according to the general procedure **E** using 1-bromo-5,7-dimethoxy-4-methylphenanthrene **S9a** (77.8 mg, 235 µmol, 1.00 eq.) and 2-(5-chloro-2-vinylphenyl)-4,4,5,5-tetramethyl-1,3,2-dioxaborolane (71.5 mg, 270 µmol, 1.11 eq.) to give **15g** as a beige solid (70.0 mg, 180 µmol, 77%): R_f 0.52. (cyclohexane/EtOAc 6:1); ν_{max} (neat): 3007w, 2948w, 1600s, 1527w, 1453m, 1424w, 1358w, 1310w, 1272w, 1218m, 1162s, 1116m, 1058w, 1024w, 988w, 914w, 829m, 736w; ^{1}H NMR (500 MHz, $CDCl_3$) δ = 7.66 (1H, d, ^3J 8.5 Hz, C3`*H*), 7.46 (1H, d, $^3J$ 7.4 Hz, C3*H*), 7.38 (1H, dd, $^3J$ 8.5, $^4J$ 2.3 Hz, C4`*H*), 7.36 (1H, d, ^3J 9.0 Hz, C9*H*), 7.30 (1H, d, ^4J 2.3 Hz, C6`*H*), 7.26 – 7.24 (1H, m, C2*H*), 7.19 (1H, d, ^3J 9.0 Hz, C10*H*), 6.83 (1H, d, ^4J 2.4 Hz, C8*H*), 6.70 (1H, d, ^4J 2.4 Hz, C6*H*), 6.33 (1H, dd, ^3J 17.5, ^3J 11.0 Hz, C11*H*), 5.63 (1H, dd, ^3J 17.5, ^2J 1.1 Hz, C12*H*), 5.00 (1H, dd, ^3J 11.0, ^2J 1.1 Hz, C12*H*), 3.98 (3H, s, C5–OCH_3), 3.96 (3H, s, C7–OCH_3), 2.60 (3H, s, C4–CH_3); ^{13}C NMR (126 MHz, $CDCl_3$) δ =159.3 (*C7*), 158.2 (*C5*), 141.7 (*C1`*), 136.6 (*C4*), 135.8 (*C2`*), 135.6 (*C8a*), 134.6 (*C11*), 133.9 (*C1*), 133.1 (*C5`*), 131.4 (*C6`*), 130.7 (*C10a*), 129.2 (*C4a*), 128.6 (*C3*), 127.9 (*C4`*), 126.6 (*C2*), 126.2 (*C3`*), 126.03 (*C9*/*C10*), 126.01 (*C9*/*C10*), 115.7 (*C4b*), 115.0 (*C12*), 100.1 (*C8*), 98.3 (*C6*), 55.7 (C7–O*C*H_3), 55.1 (C5–O*C*H_3), 24.2 (C4–*C*H_3); ESI-MS: m/z calcd. for $C_{25}H_{21}AgClO_2$ 495.0276 found 495.0276 [M+Ag^+].

1-(5-Chloro-2-vinylphenyl)-4,5-dimethoxyphenanthrene (15h):

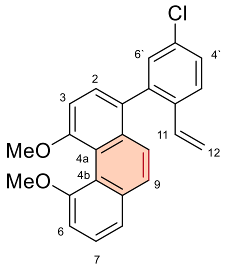

15h

Prepared according to the general procedure **E** using 1,8-dibromo-4,5-dimethoxyphenanthrene **S9g** (110 mg, 278 µmol, 1.00 eq.) and 2-(5-chloro-2-vinylphenyl)-4,4,5,5-tetramethyl-1,3,2-dioxaborolane (69.9 mg, 264 µmol, 0.95 eq.) but at 120°C over 18 h to give **15h** as a transparent oil (36.0 mg, 96.0 µmol, 36%) after after NP-prep-HPLC (from hexane/EtOAc 98:2 to 80:20, 18 mL/min, 35°C): R_f 0.48 (cyclohexane/EtOAc 5:1); ν_{max}(neat):3054w, 2994w, 2933w, 2832w, 1590w, 1524s, 1453s, 1433m, 1314s, 1249s, 1108m, 1064s, 983w, 908m, 862w, 818s, 731s, 632s; ^{1}H NMR (500 MHz, $CDCl_3$) δ = 7.66 (1H, d, ^3J 8.5 Hz, C3`*H*), 7.52 (1H, t, $^3J$ 7.8 Hz, C7*H*), 7.47 (1H, d, $^3J$ 9.0 Hz, C9*H*), 7.42 – 7.36 (2H, m, C8*H*, C4`*H*), 7.34 (1H, d, ^3J 8.1 Hz, C2*H*), 7.30 (1H, d, ^3J 2.2 Hz, C6`*H*), 7.17 (1H, d, ^3J 9.0 Hz, C10*H*), 7.15 – 7.11 (2H, m, C3*H*, C6*H*), 6.34 (1H, dd, ^3J 17.5, ^3J 11.0 Hz, C11*H*), 5.63 (1H, dd, ^3J 17.5, ^2J 1.1 Hz, C12*H*), 5.00 (1H, dd, ^3J 11.0, ^2J 1.1 Hz, C12*H*), 4.09 (3H, s, C4–OC*H*3), 4.08 (3H, s, C5–OC*H*3); ^{13}C NMR (126 MHz, $CDCl_3$) δ = 158.0 (*C5*), 157.6 (*C4*), 141.4 (*C1`*), 136.0 (*C2`*), 134.6 (*C11*), 134.4 (*C8a*), 133.2 (*C5`*), 132.8 (*C10a*), 131.6 (*C6`*), 128.8 (*C1*), 128.4 (*C2*), 127.9 (*C4`*), 127.3 (*C9*), 127.0 (*C7*), 126.2 (*C3`*), 124.7 (*C10*), 119.6 (*C8*), 119.3 (*C4a*/*C4b*), 119.2(*C4a*/*C4b*), 115.0 (*C12*), 108.4 (*C6*), 107.5 (*C3*), 55.96 (C4–O*C*H3/C5–O*C*H3), 55.94 (C4–O*C*H3/C5–O*C*H3); ESI-MS: m/z calcd. for $C_{24}H_{19}ClNaO_2$ 397.0966 found 397.0959 [M+Na$^+$].

4,5,7-Trimethyl-1-(2-vinylphenyl)phenanthrene (15i):

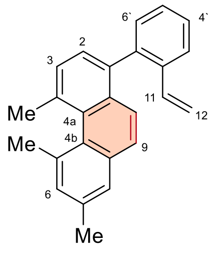

15i

Prepared according to the general procedure **E** using 4,5,7-trimethylphenanthren-1-yl trifluoromethanesulfonate **S9j** (99.5 mg, 270 µmol, 1.00 eq.) and (2-vinylphenyl)boronic acid (47.9 mg, 324 µmol, 1.20 eq.) **15i** as a white solid (70.0 mg, 217 µmol, 80%, m.p. 73.3 – 74.2°C): R_f 0.77 (cyclohexane/EtOAc 5:1); ν_{max}(neat): 3055w, 3017w, 2922w, 2850w, 1604w, 1445m, 1375w, 1304w, 1173w, 1028w, 990w, 909m, 855m, 829m, 756s, 720s, 646w; ^{1}H NMR (500 MHz, $CDCl_3$) δ = 7.84 – 7.67 (1H, m, C3`*H*), 7.49 – 7.26 (8H, m, C2*H*, C3*H*, C6*H*, C8*H*, C9*H*, C4`*H*, C5`*H*, C6`*H*), 7.20 – 7.05 (1H, m, C10*H*), 6.54 – 6.31 (1H, m, C11*H*), 5.71 (d, ^3J 17.5 Hz, C12*H*), 5.64 (d, ^3J 17.5 Hz, C12*H*), 5.09 (d, ^3J 11.0 Hz, C12*H*), 4.93 (d, ^3J 11.1 Hz, C12*H*), 2.67 – 2.60 (6H, m, C4–C*H*3, C5–C*H*3), 2.52 (3H, s, C7–C*H*3); ^{13}C NMR (126 MHz, $CDCl_3$) δ = 140.1 (*C1`*), 140.0 (*C1`*), 137.1 (*C2`*), 136.0 (*C5*), 135.8 (*C7*), 135.7 (*C11*), 135.5 (*C11*), 135.35 (*C1*), 135.26 (*C1*), 135.2 (*C4*), 133.9 (*C8a*), 133.86 (*C8a*), 132.3 (*C10a*), 131.8 (*C6`*), 131.7 (*C6`*), 130.9 (*C4a*), 130.7 (*C4a*), 130.3 (*C6*), 128.8 (*C4b*), 128.5 (*C4a*), 127.9 (*C3*), 127.8 (*C5`*), 127.7 (*C4`*), 127.65 (*C3*), 127.4 (*C5`*), 127.3 (*C2*), 126.3 (*C9*), 125.1 (*C3`*), 124.7 (*C11*), 124.6 (*C10*, *C8*), 114.5 (*C12*), 22.9 (C4–*C*H3), 22.8 (C5–*C*H3), 22.7 (C7–*C*H3); ESI-MS: m/z calcd. for $C_{25}H_{22}Ag$ 429.0767 found 429.0768 [M+Ag$^+$].

1-Bromo-4,5-dimethoxy-8-(2-vinylphenyl)phenanthrene (15j):

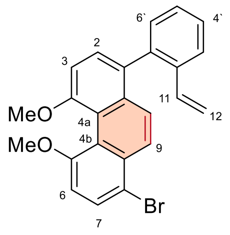

15j

Prepared according to the general procedure **E** using 1,8-dibromo-4,5-dimethoxyphenanthrene **S9g** (131 mg, 330 µmol, 1.00 eq.) and (2-vinylphenyl)boronic acid (44.9 mg, 304 µmol, 0.92 eq.) over 7 h to give **15j** as a white solid (48.0 mg, 114 µmol, 38%, m.p. 84.1 – 85.2°C): after NP-prep-HPLC (from hexane/EtOAc 98:2 to 80:20, 18 mL/min, 40°C) after NP-prep-HPLC (from hexane/EtOAc 98:2 to 80:20, 18 mL/min, 40°C): R_f 0.33 (cyclohexane/EtOAc 5:1); ν_{max} (neat): 3004w, 2932w, 2839w, 2753w, 2721w, 2668w, 2622w, 1582w, 1580w, 1522m, 1451w, 1421m, 1313s, 1265m, 1238m, 1109w, 1068s, 983w, 908m, 809m, 731s, 663w; ^{1}H NMR (500 MHz, $CDCl_3$) δ = 7.87 (1H, d, ^3J 9.2 Hz, C9*H*), 7.81 – 7.74 (2H, m, C3`*H*, C7*H*), 7.46 – 7.40 (2H, m, C2*H*, C4`*H*), 7.36 (1H, td, ^3J 7.4, ^4J 1.2 Hz, C5`*H*), 7.32 (1H, d, $^3J$ 9.2 Hz, C10*H*), 7.28 (1H, dd, $^3J$ 7.4, $^4J$ 1.4 Hz, C6`*H*), 7.16 (1H, d, ^3J 8.1 Hz, C3*H*), 6.98 (1H, d, ^3J 8.5 Hz, C6*H*), 6.40 (1H, dd, ^3J 17.5, ^3J 11.0 Hz, C11*H*), 5.67 (1H, dd, ^3J 17.5, ^2J 1.1 Hz, C12*H*), 5.00 (1H, dd, ^3J 11.0, ^2J 1.1 Hz, C12*H*), 4.08 (3H, s, C4–OC*H*$_3$), 4.06 (3H, s, C5–OC*H*$_3$); ^{13}C NMR (126 MHz, $CDCl_3$) δ = 157.5 (*C5*), 157.4 (*C4*), 139.4 (*C1`*), 137.4 (*C2`*), 135.6 (*C11*), 132.9 (*C10a*), 132.3 (*C8a*), 131.8 (*C6`*), 130.4 (*C7*), 130.3 (*C1*), 129.1 (*C2*), 127.9 (*C4`*), 127.7 (*C5`*), 126.8 (*C10*), 125.6 (*C9*), 125.0 (*C3`*), 121.2 (*C4b*), 118.9 (*C4a*), 114.6 (*C12*), 112.8 (*C8*), 108.7 (*C6*), 107.8 (*C3*), 56.1 (C5–O*C*H$_3$), 56.0 (C4–O*C*H$_3$); ESI-MS: m/z calcd. for $C_{24}H_{19}BrAgO_2$ 524.9614 found 524.9608 [M+Ag$^+$].

1-Bromo-8-(5-chloro-2-vinylphenyl)-4,5-dimethoxyphenanthrene (15k):

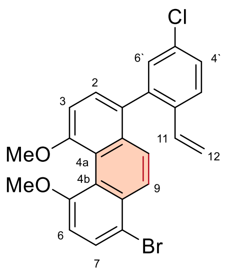

15k

Prepared according to the general procedure **E** using 1,8-dibromo-4,5-dimethoxyphenanthrene **S9g** (102 mg, 258 µmol, 1.00 eq.) and 2-(5-chloro-2-vinylphenyl)-4,4,5,5-tetramethyl-1,3,2-dioxaborolane (64.8 mg, 245 µmol, 0.95 eq.) but at 75°C over 7 h to give **15k** as a transparent oil (37.0 mg, 81.5 µmol, 32%) after NP-prep-HPLC (from hexane/EtOAc 98:2 to 80:20, 18 mL/min, 40°C): R_f 0.43 (cyclohexane/EtOAc 5:1); ν_{max}(neat): 3063w, 2949w, 2932w, 2836w, 1742w, 1721w, 1669w, 1585w, 1522m, 1461w, 1428m, 1313s, 1249s, 1235s, 1106m, 1067s, 1018m, 985w, 907m, 807s, 729s; ^{1}H NMR (500 MHz, $CDCl_3$) δ = 7.90 (1H, d, ^3J 9.2 Hz, C9*H*), 7.76 (1H, d, ^3J 8.5 Hz, C7*H*), 7.66 (1H, d, ^3J 8.5 Hz, C3`*H*), 7.42 – 7.36 (2H, m, C2*H*, C4`*H*), 7.29 (1H, s, C6`*H*), 7.29–7.27 (1H, m, C10*H*), 7.15 (1H, d, ^3J 8.1 Hz, C3*H*), 6.98 (1H, d, ^3J 8.5 Hz, C6*H*), 6.32 (1H, dd, ^3J 17.5, ^3J 11.0 Hz, C11*H*), 5.64 (1H, dd, ^3J 17.5, ^2J 1.0 Hz, C12*H*), 5.02 (1H, dd, ^3J 11.0, ^2J 1.0 Hz, C12*H*), 4.07 (3H, s, C4–OC*H*$_3$), 4.06 (3H, s, C5–OC*H*$_3$); ^{13}C NMR (126 MHz, $CDCl_3$) δ = 157.7 (*C4*), 157.5 (*C5*), 140.9 (*C1`*), 136.0 (*C2`*), 134.5 (*C11*), 133.2 (*C5`*), 132.7 (*C10a*), 132.3 (*C8a*), 131.5 (*C6`*), 130.5 (*C7*), 129.0 (*C2*), 128.8 (*C1*), 128.1 (*C4`*), 126.34 (*C3`*), 126.31 (*C10*), 125.9 (*C9*), 121.2 (*C4b*), 119.0 (*C4a*), 115.2 (*C12*), 112.9 (*C4*), 108.8 (*C6*), 107.8 (*C3*), 56.1 (C5–O*C*H$_3$), 56.0 (C4–O*C*H$_3$); ESI-MS: m/z calcd. for $C_{24}H_{19}BrClO_2$ 453.0251 found 453.0240 [M+H$^+$].

4,5-Dimethoxy-1,8-bis(2-vinylphenyl)phenanthrene (17a):

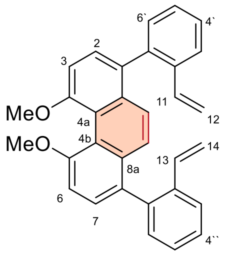

17a

Prepared according to the general procedure **E** using 1,8-dibromo-4,5-dimethoxyphenanthrene **S9g** (131 mg, 330 µmol, 1.00 eq.) and (2-vinylphenyl)boronic acid (137 mg, 924 µmol, 2.80 eq.) to give **17a** as a white solid (120 mg, 271 µmol, 82%, m.p. 95.0 – 95.7°C): R_f 0.61 (cyclohexane/EtOAc 6:1); ν_{max}(neat): 3057w, 3006w, 2930w, 2830w, 1624w, 1571m, 1524m, 1425m, 1310s, 1258s, 1076s, 982m, 910m, 810s, 758s, 702w; ^{1}H NMR (500 MHz, $CDCl_3$) δ = 7.74 – 7.63 (2H, m, C3`*H*, C3``*H*), 7.42 – 7.34 (4H, m, C2*H*, C7*H*, C4`*H*, C4``*H*), 7.34 – 7.22 (4H, m, C5`*H*, C6`*H*, C5``*H*, C6``*H*), 7.17 (2H, dd, ^{3}J 8.1, ^{4}J 1.3 Hz, C3*H*, C6*H*), 7.14 – 7.06 (2H, m, C9*H*, C10*H*), 6.53 – 6.30 (2H, m, C11*H*, C13*H*), 5.74 – 5.55 (2H, m, C12*H*, C14*H*), 5.14 – 4.91 (2H, m, C12*H*, C14*H*), 4.13 (6H, s, C4–OCH_3, C5–OCH_3); ^{13}C NMR (126 MHz, $CDCl_3$) δ = 157.43 (*C4*, *C5*), 157.39 (*C4*, *C5*), 139.7 (*C1`*, *C1``*), 139.6 (*C1`*, *C1``*), 137.29 (*C2`*, *C2``*), 137.28 (*C2`*, *C2``*), 135.7 (*C11*, *C13*), 135.6 (*C11*, *C13*), 132.8 (*C8a*, *C10a*), 131.9 (*C6`*, *C6``*), 131.8 (*C 6`*, *C6``*), 130.2 (*C1*, *C8*), 130.1 (*C1*, *C8*), 128.7 (*C2*, *C7*), (*C2*, *C7*), 127.70 (*C4`*, *C4``*), 127.67 (*C4`*, *C4``*), 127.54 (*C5`*, *C5``*), 127.47 (*C5`*, *C5``*), 125.18 (*C9*, *C10*), 125.15 (*C9*, *C10*), 124.9 (*C3`*, *C3``*), 124.8 (*C3`*, *C3``*), 119.2 (*C4a*, *C4b*), 119.1 (*C4a*, *C4b*), 114.4 (*C12*, *C14*), 114.3 (*C12*, *C14*), 107.41 (*C3*, *C6*), 107.37 (*C3*, *C6*), 55.9 (C4–O*C*H_3, C5–O*C*H_3); ESI-MS: m/z calcd. for $C_{32}H_{26}Ag$ 517.1080 found 517.1084 [M+Ag^+].

11-Methoxy-5,8-bis(2-vinylphenyl)phenanthro[3,4-d][1,3]dioxole (17b):

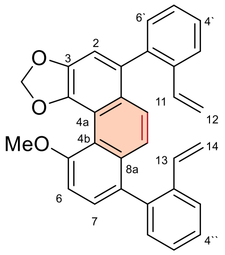

17b

Prepared according to the general procedure **E** using 5,8-dibromo-11-methoxyphenanthro[3,4-d][1,3]dioxole **S9k** (80.0 mg, 195 µmol, 1.00 eq.) and (2-vinylphenyl)boronic acid (80.8 mg, 546 µmol, 2.80 eq.) to give **17b** as a yellow oil (75.5 mg, 165 µmol, 85%): R_f 0.61 (cyclohexane/EtOAc 6:1); ν_{max}(neat): 3061w, 3012w, 2932w, 2876w, 1582m, 1538m, 1434s, 1366m, 1291s, 1219w, 1160w, 1071s, 995w, 949w, 908s, 864w, 813m, 633s; ^{1}H NMR (500 MHz, $CDCl_3$) δ 7.72 – 7.63 (2H, m, C3`*H*, C3``*H*), 7.47 – 7.34 (3H, m, C7*H*, C4`*H*, C4``*H*), 7.33 – 7.27 (2H, m, C5`*H*, C5``*H*), 7.25 – 7.18 (2H, m, C6`*H*, C6``*H*), 7.18 – 7.14 (1H, m, C6*H*), 7.13 – 7.11 (1H, m, C2*H*), 7.09 – 7.03 (1H, m, C10*H*), 6.98 – 6.88 (1H, m, C9*H*), 6.50 – 6.29 (2H, m, C11*H*, C13*H*), 6.27 – 6.14 (2H, m, OCH_2O), 5.73 – 5.52 (2H, m, C12*H*, C14*H*), 5.05 – 4.91 (2H, m, C12*H*, C14*H*), 4.13 (3H, s, OCH_3); ^{13}C NMR (126 MHz, $CDCl_3$) δ = 157.12 (*C5*), 157.08 (*C5*), 145.32 (*C3*), 145.28 (*C3*), 143.81 (*C4*), 143.78 (*C4*), 139.6 (*C1``*/*C1`*), 137.23 (*C2`*/*C2``*), 137.20 (*C2`*/*C2``*), 137.16 (*C2`*/*C2``*), 137.15 (*C2`*/*C2``*), 135.60 (*C11*/*C13*), 135.5 (*C11*/*C13*), 135.4 (*C11*/*C13*), 135.3 (*C11*/*C13*), 133.09 (*C8a*), 133.07 (*C8a*), 132.61 (*C1*), 132.59 (*C1*), 131.7 (*C6`*/*C6``*), 131.6 (*C6`*/*C6``*), 131.53 (*C6`*/*C6``*), 131.48 (*C6`*/*C6``*), 130.5 (*C8*), 130.4 (*C8*), 129.6 (*C7*), 127.9 (*C4`*/*C5`*), 127.74 (*C4`*/*C5`*), 127.71(*C4`*/*C5`*), 127.6, 127.5 (*C4`*/*C5`*), 127.4 (*C10a*), 126.10 (*C10*), 126.06 (*C10*), 124.93 (*C3`*/*C3``*), 124.92 (*C3`*/*C3``*), 124.86 (*C3`*/*C3``*), 124.80 (*C3`*/*C3``*), 122.6 (*C9*), 117.92 (*C4b*), 117.87 (*C4b*), 114.7 (*C12, C14*), 114.5 (*C4a*), 111.3 (*C2*), 111.2

(*C2*), 107.8 (*C6*), 100.6 (O*C*H$_2$O), 56.0 (O*C*H$_3$); ESI-MS: m/z calcd. for $C_{32}H_{24}AgO_3$ 563.0771 found 563.0771 [M+Ag$^+$].

4-Methoxy-5-methyl-1,8-bis(2-vinylphenyl)phenanthrene (17c):

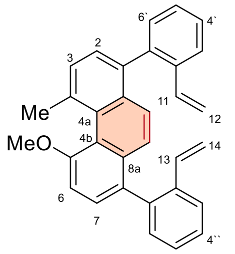

17c

Prepared according to the general procedure **E** using 1,8-dibromo-4-methoxy-5-methylphenanthrene **S9l** (76.0 mg, 200 µmol, 1.00 eq.) and (2-vinylphenyl)boronic acid (82.9 mg, 560 µmol, 2.80 eq.) to give **17c** as a white solid (65.2 mg, 153 µmol, 76%, m.p. 89.9 – 90.7°C): R_f 0.69 (cyclohexane/EtOAc 13:2); ν_{max}(neat): 3060w, 3006w, 2953w, 2929w, 2834w, 1630w, 1576w, 1527w, 1448m, 1426m, 1298m, 1259m, 1225m, 1165w, 1098s, 1022w, 989w, 910s, 820s; ^{1}H NMR (500 MHz, $CDCl_3$) δ = 7.73 – 7.67 (2H, m, C3`*H*, C3``*H*), 7.51 (1H, dd, $^3J$ 7.5, $^4J$ 2.3 Hz, C3*H*), 7.43 – 7.25 (8H, m, C2*H*, C7*H*, C4`*H*, C5`*H*, C6`*H*, C4``*H*, C5``*H*, C6``*H*), 7.18 – 7.09 (2H, m, C9*H*, C6*H*), 7.08 – 7.02 (1H, m, C10*H*), 6.53 – 6.33 (2H, m, C11*H*, C13*H*), 5.70 – 5.58 (2H, m, C12*H*, C14*H*), 5.07 – 4.92 (2H, m, C12*H*, C14*H*), 4.05 (3H, s, OC*H*$_3$), 2.68 (3H, s, C4–C*H*$_3$); ^{13}C NMR (126 MHz, $CDCl_3$) δ = 156.5 (*C5*), 156.4 (*C5*), 139.90 (*C1`*), 139.87 (*C1`*), 139.65 (*C1``*), 139.6 (*C1``*), 137.3 (*C2``*), 137.1 (*C4*), 137.0 (*C2`*), 135.7 (*C11*/*C13*), 135.6 (*C11*/*C13*), 135.1 (*C1*), 133.2 (*C8a*), 131.9 (*C10a*), 131.8 (*C10a*), 131.7 (*C6`*/*C6``*), 131.6 (*C6`*/*C6``*), 130.4 (*C8*), 130.3 (*C8*), 128.9 (*C4a*), 128.8 (*C7*), 128.4 (*C3*), 128.3 (*C3*), 127.8 (*C2*/*C4`*/*C5`*/*C4``*/*C5``*), 127.7 (*C2*/*C4`*/*C5`*/*C4``*/*C5``*), 127.6 (*C2*/*C4`*/*C5`*/*C4``*/*C5``*), 127.5 (*C2*/*C4`*/*C5`*/*C4``*/*C5``*), 125.8 (*C10*), 124.9 (*C3`*/*C3``*), 124.84, 124.7 (*C3`*/*C3``*), 124.06 (*C9*), 124.01 (*C9*), 120.8 (*C4b*), 120.7 (*C4b*), 114.5 (*C12*/*C14*), 114.4 (*C12*/*C14*), 106.5 (*C6*), 106.4 (*C6*), 55.1 (O*C*H$_3$), 24.2 (C4–*C*H$_3$); ESI-MS: m/z calcd. for $C_{32}H_{26}AgO$ 533.1029 found 533.1033 [M+Ag$^+$].

4,5-Dimethyl-1,8-bis(2-vinylphenyl)phenanthrene (17d):

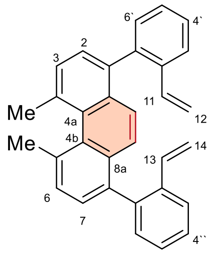

17d

Prepared according to the general procedure **E** using 4,5-dimethylphenanthrene-1,8-diyl bis(trifluoromethanesulfonate) **S9o** (60.3 mg, 120 µmol, 1.00 eq.) and (2-vinylphenyl)boronic acid (49.7 mg, 336 µmol, 2.80 eq.) to give **17d** as a white solid (39.0 mg, 95.0 µmol, 72%, m.p. 154.5 – 156.0°C): R_f 0.79 (cyclohexane/EtOAc 5:1); ν_{max}(neat): 3017w, 2955w, 2903w, 1431w, 1260w, 1182w, 1072w, 991m, 909s, 819s, 756s; ^{1}H NMR (500 MHz, $CDCl_3$) δ 7.81 – 7.57 (2H, m, C3`*H*, C3``*H*), 7.53 – 7.42 (2H, m, C3*H*, C6*H*), 7.40 – 7.15 (m, 8H), 7.11 – 6.95 (2H, m, C9*H*, C10*H*), 6.55 – 6.22 (2H, m, C11*H*, C13*H*), 5.78 – 5.51 (2H, m, C12*H*, C14*H*), 5.19 – 4.81 (2H, m, C12*H*, C14*H*), 2.74 – 2.66 (m, 6H, s, C4–C*H*$_3$, C5–C*H*$_3$); ^{13}C NMR (126 MHz, $CDCl_3$) δ = 139.9, 139.7, 139.6, 137.1, 137.0, 135.8, 135.64, 135.60, 135.5, 135.4, 135.3, 135.23, 135.20, 135.1, 132.3, 131.9, 131.9, 131.8, 131.64, 131.62, 131.60, 131.1, 130.8, 130.7, 130.6, 128.2, 128.0, 128.0, 127.8, 127.8, 127.8, 127.75, 127.70, 127.6, 127.5, 127.4, 127.3, 125.1, 125.0, 124.72, 124.66, 124.62, 124.5, 124.4, 114.51, 114.49, 22.98, 22.95, 22.91, 22.86; ESI-MS: m/z calcd. for $C_{32}H_{26}Ag$ 517.1080 found 517.1084 [M+Ag$^+$].

1-(5-Chloro-2-vinylphenyl)-4,5-dimethoxy-8-(2-vinylphenyl)phenanthrene (17e):

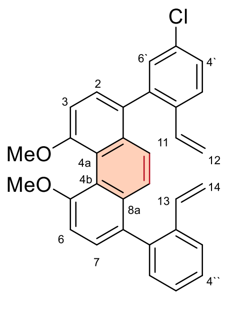

17e

Prepared according to the general procedure **E** using 1-bromo-4,5-dimethoxy-8-(2-vinylphenyl)phenanthrene **15j** (39.5 mg, 95.0 µmol, 1.00 eq.) and 2-(5-chloro-2-vinylphenyl)-4,4,5,5-tetramethyl-1,3,2-dioxaborolane (27.4 mg, 104 µmol, 1.09 eq.) to give **17e** as a transparent oil (23.9 mg, 50.1 µmol, 53%): R_f 0.56 (cyclohexane/EtOAc 5:1); ν_{max}(neat): 3090w, 2932w, 2829w, 1587w, 1527w, 1453w, 1431w, 1314m, 1261m, 1078m, 968w, 909w, 811w, 733w, 632w; ^{1}H NMR (500 MHz, $CDCl_3$) δ = 7.73 – 7.67 (1H, m, C3``*H*), 7.64 – 7.58 (1H, m, C3`*H*), 7.43 – 7.22 (7H, m, C2*H*, C7*H*, C4`*H*, C6`*H*, C4``*H*, C5``*H*, C6``*H*), 7.21 – 7.15 (2H, m, C3*H*, C6*H*), 7.14 – 7.03 (2H, m, C9*H*, C10*H*), 6.52 – 6.23 (2H, m, C11*H*, C13*H*), 5.71 – 5.52 (2H, m, C12*H*, C14*H*), 5.05 – 4.93 (2H, m, C12*H*, C14*H*), 4.13 (6H, s, C4–OC*H₃*, C5–OC*H₃*); ^{13}C NMR (126 MHz, $CDCl_3$) δ = 157.73 (*C4*), 157.70 (*C4*), 157.42 (*C5*), 157.37 (*C5*), 141.3 (*C1`*), 141.2 (*C1`*), 139.6 (*C1``*), 139.5 (*C1``*), 137.30 (*C2``*), 137.26 (*C1``*), 135.9 (*C2`*), 135.7 (*C13*), 135.5 (*C13*), 134.7 (*C11*), 134.5 (*C11*), 133.1 (*C5`*), 133.0 (*C5`*), 132.8 (*C8a*), 132.6 (*C10a*), 131.9 (*C6``*), 131.8 (*C6``*), 131.6 (*C6`*), 131.5 (*C6`*), 130.23 (*C8*), 130.16 (*C8*), 128.8 (*C2*/*C7*), 128.7 (*C2*/*C7*), 128.64 (*C2*/*C7*), 128.55 (*C1*), 128.4 (*C1*), 127.9 (*C4``*/*C5``*), 127.8 (*C4``*/*C5``*), 127.75 (*C4`*), 127.73 (*C4`*), 127.6 (*C4``*/*C5``*), 127.5 (*C4``*/*C5``*), 126.2 (*C3`*), 126.1 (*C3`*), 125.53 (*C9*), 125.51 (*C9*), 124.9 (*C3``*), 124.8 (*C3``*), 124.75 (*C10*), 124.70 (*C10*), 119.3 (*C4a*/*C4b*), 119.2 (*C4a*/*C4b*), 119.13 (*C4a*/*C4b*), 119.09 (*C4a*/*C4b*), 114.9 (*C12*/*C14*), 114.5 (*C12*/*C14*), 114.4 (*C12*/*C14*), 107.6 (*C6*), 107.5 (*C6*), 107.40 (*C3*), 107.36 (*C3*), 55.9 (C4–O*C*H₃, C5–O*C*H₃); ESI-MS: m/z calcd. for $C_{32}H_{25}AgClO_2$ 583.0589 found 583.0590 [M+Ag$^+$].

1,8-Bis(5-fluoro-2-vinylphenyl)-4,5-dimethoxyphenanthrene (17f):

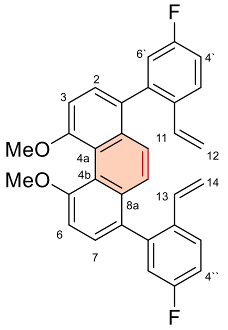

17f

Prepared according to the general procedure **E** using 1,8-dibromo-4,5-dimethoxyphenanthrene **S9g** (69.3 mg, 175 µmol, 1.00 eq.) and 2-(5-fluoro-2-vinylphenyl)-4,4,5,5-tetramethyl-1,3,2-dioxaborolane (122 mg, 480 µmol, 2.80 eq.) to give **17f** as yellow solid (73.0 mg, 153 µmol, 87%, m.p. 64.5 – 66.0°C): R_f 0.54 (cyclohexane/EtOAc 5:1); ν_{max}(neat): 3059w, 2981w, 2938w, 2835w, 1573m, 1527m, 1483s, 1431m, 1314s, 1262s, 1214m, 1143m, 1076s, 984w, 909w, 814m, 775m, 700w; ^{1}H NMR (500 MHz, $CDCl_3$) δ = 7.69 – 7.59 (2H, m, C3`*H*, C3``*H*), 7.39 – 7.34 (2H, m, C2*H*, C7*H*), 7.20 – 7.15 (2H, m, C3*H*, C6*H*), 7.12 – 7.04 (4H, m, C9*H*, C10*H*, C4`*H*, C4``*H*), 7.02 – 6.93 (2H, m, C6`*H*, C6``*H*), 6.42 – 6.17 (2H, m, C11*H*, C13*H*), 5.62 – 5.46 (2H, m, C12*H*, C14*H*), 5.02 – 4.85 (2H, m, C12*H*, C14*H*), 4.13 (6H, s, C4–OC*H₃*, C5–OC*H₃*); $^{13}$C NMR (126 MHz, $CDCl_3$) δ = 162.05 (d, $^1J_{CF}$ 248.0 Hz, *C5`*, *C5``*), 157.68 (*C4*, *C5*), 157.65 (*C4*, *C5*), 141.5 (*C1`*, *C1``*), 134.7 (*C11*, *C13*), 134.5 (*C11*, *C13*), 133.66 (*C2`*, *C2``*), 133.63 (*C2`*, *C2``*), 132.50 (*C8a*, *C10a*), 132.49 (*C8a*, *C10a*), 129.0 (*C1*, *C8*) 128.6 (*C2*, *C7*), 128.5 (*C2*, *C7*), 126.7 (*C3`*, *C3``*), 126.6 (*C3`*, *C3``*), 126.5 (*C3`*, *C3``*), 125.1 (*C9*, *C10*), 125.0 (*C9*, *C10*), 119.21 (*C4a*, *C4b*), 119.17 (*C4a*, *C4b*), 118.4 (*C12*, *C14*), 118.3 (*C12*, *C14*),

118.21 (*C6`*, *C6``*), 118.16 (*C6`*, *C6``*), 114.9 (*C4`*, *C4``*), 114.8 (*C4`*, *C4``*), 114.2 (*C4`*, *C4``*), 114.1 (*C4`*, *C4``*), 107.51 (*C3*, *C6*), 107.47 (*C3*, *C6*), 56.0 (C4–OCH_3, C5–OCH_3); ESI-MS: m/z calcd. for $C_{32}H_{24}AgF_2O_2$ 585.0790 found 585.0797 [$M+Ag^+$].

4-Methoxy-1,8-bis(5-methoxy-2-vinylphenyl)-5-methylphenanthrene (17g):

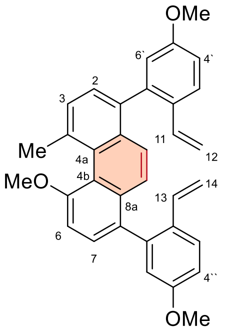

17g

Prepared according to the general procedure **E** using 1,8-dibromo-4-methoxy-5-methylphenanthrene **S9l** (41.8 mg, 110 µmol, 1.00 eq.) and 2-(5-methoxy-2-vinylphenyl)-4,4,5,5-tetramethyl-1,3,2-dioxaborolane (80.1 mg, 308 µmol, 2.80 eq.) to give **17g** as a white solid (45.0 mg, 92.5 µmol, 84%, m.p. 89.2 – 90.5°C): R_f 0.50 (cyclohexane/EtOAc 5:1); ν_{max}(neat): 3081w, 2963w, 2930w, 2833w, 2729w, 1686w, 1602m, 1576m, 1527m, 1456m, 1326m, 1257m, 1219m, 1166w, 1098s, 1053w, 1021w, 980w, 907w, 816s, 757s; ^{1}H NMR (500 MHz, $CDCl_3$) δ = 7.70 – 7.59 (2H, m, C3`*H*, C3``*H*), 7.55 – 7.48 (1H, m, C3*H*), 7.44 – 7.39 (1H, m, C7*H*), 7.38 – 7.31 (1H, m, C2*H*), 7.22 – 7.14 (1H, m, C10*H*), 7.13 – 7.05 (2H, m, C9*H*, C6*H*), 6.99 – 6.90 (2H, m, C4`*H*, C4``*H*), 6.86 – 6.75 (2H, m, C6`*H*, C6``*H*), 6.44 – 6.33 (1H, m, C13*H*), 6.29 (1H, dd, $^3J$ 17.5, $^3J$ 11.0 Hz, C11*H*), 5.59 – 5.42 (2H, m, C12*H*, C14*H*), 4.94 – 4.73 (2H, m, C12*H*, C14*H*), 4.05 (3H, s, C5–O$CH_3$), 3.80 (3H, s, C5`–O$CH_3$, C5``–OCH_3), 3.78 (3H, s, C5`–OCH_3, C5``–O$CH_3$), 3.78 (3H, s, C4`–O$CH_3$, C4``–OCH_3), 3.77 (3H, s, C5`–OCH_3, C5``–O$CH_3$), 2.67 (3H, s, C4–$CH_3$); $^{13}$C NMR (126 MHz, $CDCl_3$) δ = 159.0 (*C5`/C5``*), 158.92 (*C5`/C5``*), 158.86 (*C5`/C5``*), 156.5 (*C5*), 156.4 (*C5*), 141.2 (*C1`*), 140.95 (*C1``*), 140.92 (*C1``*), 137.11 (*C4*), 137.07 (*C4*), 135.14 (*C1*), 135.07 (*C1*), 135.0 (*C13*), 134.9 (*C11*), 133.18 (*C8a*), 133.16 (*C8a*), 131.6 (*C10a*), 130.33 (*C8*), 130.26 (*C8*), 130.18 (*C2``*), 130.00 (*C2`*), 129.97 (*C2`*), 128.9 (*C4a*), 128.80 (*C4a*), 128.76 (*C7*), 128.6 (*C7*), 128.4 (*C3*), 128.3 (*C3*), 127.6 (*C2*), 127.5 (*C2*), 126.1 (*C3`/C3``*), 126.0 (*C3`/C3``*), 125.9 (*C3`/C3``*), 125.91 (*C10*), 125.88 (*C10*), 124.13 (*C9*), 124.08 (*C9*), 120.7 (*C4b*), 116.3 (*C6`/C6``*), 116.2 (*C6`/C6``*), 116.1 (*C6`/C6``*), 116.0 (*C6`/C6``*), 114.23 (*C4`/C4``*), 114.19 (*C4`/C4``*), 112.31 (*C12/C14*), 112.29 (*C12/C14*), 112.28 (*C12/C14*), 112.26 (*C12/C14*), 55.5 (C4`–OCH_3, C4``–OCH_3), 55.1 (C5–OCH_3), 24.2 (C4–CH_3); ESI-MS: m/z calcd. for $C_{34}H_{30}AgO_3$ 593.1240 found 593.1231 [$M+Ag^+$].

4,5-Dimethoxy-1,8-bis(5-(trifluoromethyl)-2-vinylphenyl)phenanthrene (17h):

Prepared according to the general procedure **E** using 1,8-dibromo-4,5-dimethoxyphenanthrene **S9g** (35.6 mg, 90.0 µmol, 1.00 eq.) and 4,4,5,5-tetramethyl-2-(5-(trifluoromethyl)-2-vinylphenyl)-1,3,2-dioxaborolane (72.4 mg, 243 µmol, 2.70 eq.) to give **17h** as a transparent oil (36.9 mg, 63.8 µmol, 71%):R_f 0.59 (cyclohexane/EtOAc 5:1); ν_{max}(neat): 2935w, 2852w, 1614w, 1571w, 1527m, 1456w, 1408w, 1335s, 1257s, 1166ss, 1119s, 1077s, 1020w, 985w, 909w, 811w, 734w, 653w; ^{1}H NMR (500 MHz, $CDCl_3$) δ = 7.81

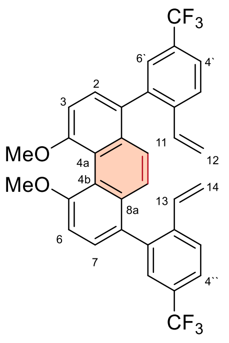

17h

– 7.75 (2H, m, C3`*H*, C3``*H*), 7.65 – 7.59 (2H, m, C4`*H*, C4``*H*), 7.56 – 7.49 (2H, m, C6`*H*, C6``*H*), 7.41 – 7.35 (2H, m, C2*H*, C7*H*), 7.22 – 7.15 (2H, m, C3*H*, C6*H*), 7.10 – 7.01 (2H, m, C9*H*, C10*H*), 6.57 – 6.28 (2H, m, C11*H*, C13*H*), 5.76 – 5.66 (2H, m, C12*H*, C14*H*), 5.15 – 5.05 (2H, m, C12*H*, C14*H*), 4.14 (3H, s, C4–OC*H*$_3$/C5–OC*H*$_3$), 4.14 (3H, s, C4–OC*H*$_3$/C5–OC*H*$_3$); ^{13}C NMR (126 MHz, $CDCl_3$) δ = 157.89 (*C4*, *C5*), 157.85 (*C4*, *C5*), 140.8 (*C2`*, *C2``*), 140.0 (*C1`*, *C1``*), 134.7 (*C11*, *C13*), 134.5 (*C11*, *C13*), 132.51 (*C8a*, *C10a*), 132.48 (*C8a*, *C10a*), 128.8 (*C2*, *C7*), 128.72 (*C2*, *C7*), 128.66 (*C6`*, *C6``*), 128.59 (*C6`*, *C6``*), 129.53 (q, $^2J_{CF}$ 31.6 Hz, *C5`*, *C5``*), 125.4 (*C3`*, *C3``*), 125.3 (*C3`*, *C3``*), 125.04 (*C9*, *C10*), 125.02 (*C9*, *C10*), 124.6 (*C4`*, *C4``*), 124.5 (*C4`*, *C4``*), 119.30 (*C4a*, *C4b*), 119.28 (*C4a*, *C4b*), 117.0 (*C12*, *C14*), 116.9 (*C12*, *C14*), 107.7 (*C3*, *C6*), 107.6 (*C3*, *C6*), 56.0 (C4–O*C*H$_3$, C5–O*C*H$_3$); ^{19}F NMR (376 MHz, $CDCl_3$) δ = –62.39, –62.42; ESI-MS: m/z calcd. for $C_{34}H_{24}AgF_6O_2$ 685.0726 found 685.0719 [M+Ag$^+$].

1,8-Bis(5-chloro-2-vinylphenyl)-4,5-dimethoxyphenanthrene (17i):

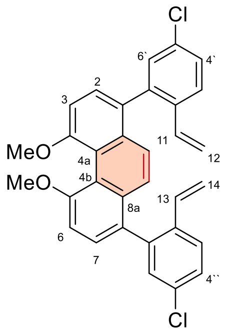

17i

Prepared according to the general procedure **E** using 1,8-dibromo-4,5-dimethoxyphenanthrene **S9g** (71.3 mg, 180.0 µmol, 1.00 eq.) and 2-(5-chloro-2-vinylphenyl)-4,4,5,5-tetramethyl-1,3,2-dioxaborolane (129 mg, 486 µmol, 2.70 eq.) to give **17i** as a white solid (40.0 mg, 78.2 µmol, 78%, m.p. 116.7 – 117.5°C): R_f 0.67 (cyclohexane/EtOAc 5:1); ν_{max}(neat): 2992w, 2929w, 1587w, 1527m, 1470w, 1431w, 1314w, 1262w, 1235w, 1078s, 985w, 907s, 812m, 713s, 632s; ^{1}H NMR (500 MHz, $CDCl_3$) δ = 7.64 – 7.59 (2H, m, C3*H*, C3`H), 7.39 – 7.32 (4H, m, C2*H*, C7*H*, C4`*H*, C4``*H*), 7.29 – 7.24 (2H, m, C6`*H*, C6``*H*), 7.19 – 7.14 (2H, m, C3*H*, C6*H*), 7.12 – 7.07 (2H, m, C9*H*, C10*H*), 6.49 – 6.14 (2H, m, C11*H*, C13*H*), 5.76 – 5.52 (2H, m, C12*H*, C14*H*), 5.08 – 4.89 (2H, m, C12*H*, C14*H*), 4.13 (6H, s, C4–OC*H*$_3$, C5–OC*H*$_3$); ^{13}C NMR (126 MHz, $CDCl_3$) δ = 157.74 (*C4*, *C5*), 157.70 (*C4*, *C5*), 141.2 (*C1`*, *C1``*), 141.1 (*C1`*, *C1``*), 135.95 (*C2`*, *C2``*), 135.89 (*C2`*, *C2``*), 134.7 (*C11*, *C13*), 134.5 (*C11*, *C13*), 133.1 (*C5`*, *C5``*), 132.5 (*C10a*), 131.6 (*C6`*, *C6``*), 131.5(*C6`*, *C6``*), 128.8 (*C1*, *C8*), 128.7 (*C1*, *C8*), 128.5 (*C2*, *C7*), 127.9 (*C4`*, *C4``*), 126.3 (*C3`*, *C3``*), 126.2 (*C3`*, *C3``*), 125.10 (*C9*, *C10*), 125.07 (*C9*, *C10*), 119.22 (*C4a*, *C4b*), 119.18 (*C4a*, *C4b*), 115.1 (*C12*, *C14*), 114.9 (*C12*, *C14*), 107.6 (*C3*, *C6*), 107.5 (*C3*, *C6*), 56.0 (C4–O*C*H$_3$, C5–O*C*H$_3$); ESI-MS: m/z calcd. for $C_{32}H_{24}AgCl_2O_2$ 617.0199 found 617.0197 [M+Ag$^+$].

Aromatic Ring-Opening Metathesis

Optimization Studies

Technique for inert conditions: All reactions involving Mo- and Ru-catalysts were performed using degassed and dried solvents in an argon-filled glovebox. Commercially available dry solvents were loaded with molecular sieves and were degassed using the freeze–pump–thaw method. Solvent flasks were then transferred to a nitrogen-filled glovebox and used within seven days of storage over molecular sieves.[1] Optimization studies were performed in 1 or 10 mL crimp cap vials closed in a glovebox. If temperature alteration was necessary, the vials were taken out of the glovebox and heated.

General procedure F for method optimization (7.50 µmol scale):

The catalyst was weighed into a 2 mL crimp cap vial. A solution of the corresponding substrate (7.50 µmol, 1.00 eq.) in the indicated solvent was then added. The reaction was stirred for the specified time at the indicated temperature, cooled down to room temperature, diluted with EtOAc and filtered through a thin layer of silica gel. Solvents were then removed under reduced pressure and the residue was analyzed by ^{1}H-NMR using durene (1,2,4,5-tetramethylbenzene) as an internal standard.

General procedure G for method optimization (70.0 µmol scale):

The catalyst was weighed into a 10 mL crimp cap vial. A solution of the corresponding substrate (70.0 µmol) in the indicated solvent was then added. The reaction was stirred for the specified time at the indicated,temperature cooled down to room temperature, diluted with EtOAc and filtered through a thin layer of silica gel. Solvents were then removed under reduced pressure and the residue was analyzed by ^{1}H-NMR. The obtained crude product was purified by silica gel column chromatography (cyclohexane/EtOAc 100:0→80:20) to give the desired product. The isolated yield was reported.

General optimization procedure H for atroposelective aromatic ring-opening metathesis with *in sit*u catalyst formation:

The Mo-precatalyst and the indicated ligand were weighed into a 2 mL crimp cap vial before the specified solvent was added to reach the desired concentration. The obtained solution was stirred at room temperature for 1 h (in an argon-filled glovebox) and then transfered to the corresponding substrate. The resuting reaction mixture was stirred at the specidied temperature for a period as stated. The solution was then diluted with EtOAc and filtered through a thin layer of silica gel. Solvents were removed and the obtained crude was analyzed by ^{1}H-NMR using durene (1,2,4,5-tetramethylbenzene) as an internal standard. The e.r. of the product was determined by HPLC on a chiral stationary phase (as specified).

Aromatic Ring Opening (ArROM) – Ring-Closing Metathesis (RCM) Cascade: Tetraphene

Optimization Studies (15.0 µmol scale): Performed accroding to the general procedure **F** utilizing 4-(2-vinylphenyl)tetraphene (4.96 mg, 15.0 µmol) and toluene (0.50 mL, 0.03 mol·L^{-1}) with the reaction time of 18 h.

Supplementary Table 1. Reaction conditions optimization for tetraphene ring-opening metathesis

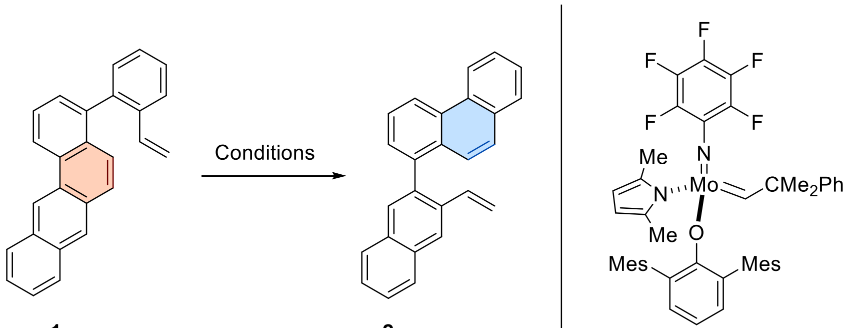

1 **2** **C1**

Entry ^{a}	Catalyst (mol%)	Solvent	Temp. [°C]	Conversion^{b} [%]	Yield^{b} [%]
1	**C1** (10)	Toluene	50	>95	73
2	**C1** (10)	Toluene	65	>95	80
3	**C1** (20)	Toluene	65	>95	49
4	**C1** (20)	Toluene	85	>95	41

^{a}Reactions were performed on 15.0 µmol scale of **1** for 18 h. ^{b}Conversion and yield were determined by ^{1}H-NMR with durene as an internal standard.

1-(3-Vinylnaphthalen-2-yl)phenanthrene (2):

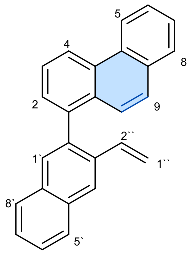

2

Prepared according to the general procedure **G** using 4-(2-vinylphenyl)tetraphene (23.1 mg, 70.0 µmol) and **C1** (5.83 mg, 7.00 µmol, 10 mol%) to give the title compound as a white solid (18.9 mg, 57.2 µmol, 82%, m.p. 92.8 – 94.0°C): R$_{f}$ 0.77 (cyclohexane/EtOAc 5:1); ν_{max} (neat): 3052w, 3010w, 2923w, 2860w, 1931w, 1798w, 1692w, 1591w, 1492w, 1450w, 1413w, 1262m, 1160w, 1090w, 1033w, 988w, 906s, 807s; ^{1}H NMR (500 MHz, $CDCl_3$) δ = 8.80 (1H, d, ^{3}J 7.2 Hz, C4*H*), 8.78 (1H, d, ^{3}J 6.0 Hz, C5*H*), 8.19 (1H, s, C4`*H*), 7.99 – 7.93 (1H, m, C5`*H*), 7.86 (1H, dd, ^{3}J 7.8, ^{4}J 1.3 Hz, C8*H*), 7.83 (1H, d, ^{3}J 7.9 Hz, C8`*H*), 7.81 (1H, s, C1`*H*), 7.73 (1H, dd, ^{3}J 8.4, ^{3}J 7.2 Hz, C3*H*), 7.69 (1H, ddd, ^{3}J 8.4, ^{3}J 7.0, ^{4}J 1.3 Hz, C6*H*), 7.64 – 7.58 (2H, m, C7*H*, C9*H*), 7.57 – 7.48 (3H, m, C2*H*, C6`*H*, C7`*H*), 7.40 (1H, d, ^{3}J 9.1 Hz, C10*H*), 6.49 – 6.37 (1H, m C2``*H*), 5.77 (1H, dd, $^{3}J$ 17.4, $^{2}J$ 1.2 Hz, C1``*H*), 5.04 (dd, ^{3}J 11.0, ^{2}J 1.2 Hz, C1``*H*); $^{13}$C NMR (126 MHz, $CDCl_3$) δ = 139.5 (*C1*), 138.3 (*C2`*), 135.8 (*C3`*), 135.6 (*C2``*), 133.2 (*C4`a*), 132.9 (*C8a`*), 131.9 (*C8a*), 131.0 (*C10a*), 130.48 (*C4b*), 130.45 (*C4a*), 129.7 (*C1`*), 128.7 (*C8*), 128.6 (*C2*), 128.0 (*C5`*), 127.8 (*C8`*), 127.1 (*C9*), 126.88 (*C6*), 126.84 (*C7*), 126.4 (*C7` / C6`*), 126.0 (*C3*), 125.1 (*C10*), 124.0 (*C4`*), 123.0 (*C5*), 122.5 (*C4*), 115.5 (*C1``*); ESI-MS: m/z calcd. for $C_{26}H_{18}Ag$ 437.0454 found 437.0453 [M+Ag^{+}].

ArROM with Twofold RCM: Napthalene

Optimization Studies (7.50 µmol scale): Performed accroding to the general procedure **F** utilizing 1-vinyl-4-(2-vinylphenyl)naphthalene (1.92 mg, 7.50 µmol) and toluene (0.25 mL, 0.03 mol·L^{-1}) with 18 h reaction time.
Optimization Studies (70.0 µmol scale): Performed accroding to the general procedure **G** utilizing 1-vinyl-4-(2-vinylphenyl)naphthalene (17.9 mg, 70.0 µmol) and toluene (2.3 mL, 0.03 mol·L^{-1}) with the reaction time of 18 h.

Supplementary Table 2. Reaction conditions optimization for napthalene ring-opening metathesis

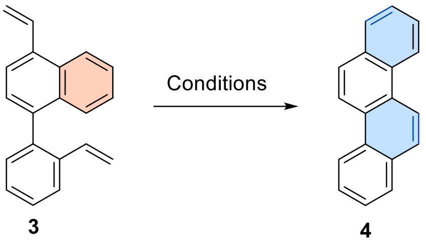

Entry	Catalyst (mol%)	Solvent	Temp. [°C]	Conversion[b] [%]	Yield[b] [%]
1[a]	**C1** (20)	Toluene	65	5	3
2[a]	**C1** (20)	Toluene	85	32	25
3[a]	**C1** (10)	Toluene	120	30	25
4[a]	**C1** (20)	Toluene	120	63	48
5[a]	**C4** (20)	Toluene	65	No conv.	–
6[a]	**C4** (20)	Toluene	85	No conv.	–
7[a]	**C3** (20)	Toluene	85	9	4
8[a]	**C5** (20)	Toluene	85	No conv.	–
9[a]	**C2** (20)	Toluene	85	53	46
10[a]	**C2** (30)	Toluene	85	69	41
11[a]	**C2** (20)	Toluene	120	85	70
12[c]	**C1** (20)	Toluene	120	71	51[d]
13[c]	**C2** (10)	Toluene	120	83	74[d]
14[c]	**C2** (20)	Toluene	120	92	64[d]

[a]Reactions were performed on 7.50 µmol scale of **3** for 18 h. [b]Conversion and yield were determined by ^{1}H-NMR with durene as an internal standard. [c]Reactions were performed on 70.0 µmol scale. [d]Isolated yield.

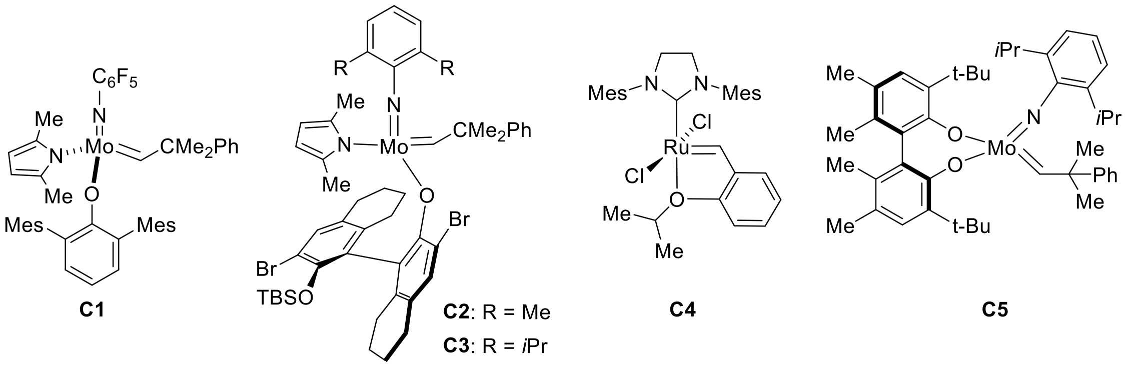

Chrysene (4):

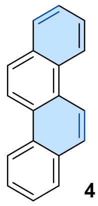

Prepared according to the general procedure **G** using 1-vinyl-4-(2-vinylphenyl)naphthalene (17.9 mg, 70.0 µmol) and **C2** (7.05 mg, 7.00 µmol, 10 mol%) to give the title compound as a white solid (11.8 mg, 51.7 µmol, 74%). The spectral data were in accordance with those previously reported in the literature.[11]

ArROM with Twofold RCM: Benzofused Five-Membered Heterocycles

Optimization Studies: Performed accroding to the general procedure **F** utilizing 1-methyl-7-vinyl-4-(2-vinylphenyl)-1*H*-indole **5a** (1.95 mg, 7.50 µmol) and toluene (0.25 mL, 0.03 mol·L^{-1}) with the reaction time of 18 h.

Supplementary Table 3. Reaction conditions optimization for ArROM with twofold RCM of indole

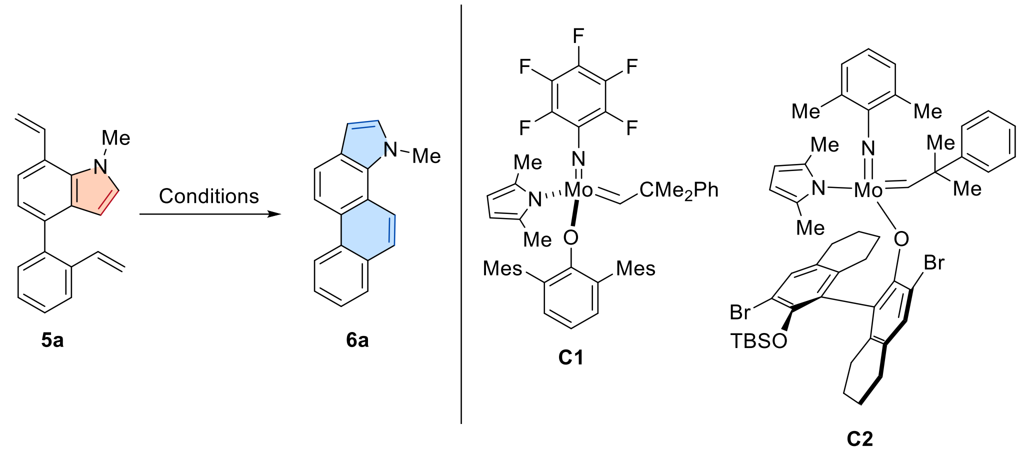

Entry[a]	Catalyst (mol%)	Solvent	Temp. [°C]	Conversion[b] [%]	Yield[b] [%]
1	**C1** (10)	Toluene	65	>95	92
2	**C1** (20)	Toluene	85	>95	98
3	**C2** (10)	Toluene	65	>95	98

[a]Reactions were performed on 7.50 µmol scale of **5a** for 18 h. [b]Conversion and yield were determined by ^{1}H-NMR with durene as an internal standard.

General procedure I for ArROM with twofold RCM for benzofused five-membered heterocycles:

The catalyst **C2** (7.05 mg, 7.00 µmol, 10 mol%) was weighed into a 10 mL crimp cap vial. A solution of the indole/benzofuran derivative (70.0 µmol, 1.00 eq.) in toluene (2.3 mL, 0.03 mol·L^{-1}) was then added. The reaction was stirred for 18 h at the indicated temperature, cooled down to room temperature, diluted with EtOAc and filtered through a thin layer of silica gel. Solvents were then removed under reduced pressure. The obtained crude product was purified by silica gel column chromatography (cyclohexane/EtOAc 100:0→80:20) to give the desired product.

3-Methyl-3*H*-naphtho[1,2-g]indole (6a):

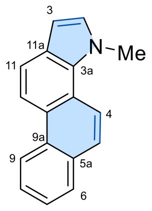

6a

Prepared according to the general procedure **I** using 1-methyl-7-vinyl-4-(2-vinylphenyl)-1*H*-indole **5a** (18.2 mg, 70.0 µmol) to give the title compound **6a** as a white solid (15.9 mg, 68.7 µmol, 98%, m.p. 140.1 – 140.8°C): R_f 0.44 (cyclohexane/EtOAc 5:1); ν_{max} (neat): 3054w, 2924w, 2855w, 1928w, 1755w, 1689w, 1524w, 1487w, 1426w, 1306m, 1266w, 1220w, 1123m, 1036w, 898w, 861w, 810m; ^{1}H NMR (500 MHz, $CDCl_3$) δ = 8.70 (1H, d, ^{3}J 8.4 Hz, C9*H*), 8.50 (1H, d, ^{3}J 9.2 Hz, C4*H*), 8.40 (1H, d, ^{3}J 8.8 Hz, C10*H*), 7.85 (1H, d, ^{3}J 6.9 Hz,

C6*H*), 7.82 (1H, d, ^3J 8.8 Hz, C11*H*), 7.77 (1H, d, ^3J 9.2 Hz, C5*H*), 7.59 (1H, ddd, ^3J 8.4, ^3J 6.9, ^4J 1.4 Hz, C8*H*), 7.53 – 7.44 (1H, m, C7*H*), 7.06 (1H, d, ^3J 3.0 Hz, C2*H*), 6.59 (1H, d, ^3J 3.0 Hz, C3*H*), 4.30 (3H, s, N–CH_3); ^{13}C NMR (126 MHz, $CDCl_3$) δ = 131.7 (*C3a*), 131.5 (*C9a*), 130.6 (*C5a*, *C2*), 128.5 (*C6*), 127.3 (*C11a*), 126.8 (*C9b*), 126.6 (*C8*), 126.0 (*C5*), 125.5 (*C7*), 123.3 (*C9*), 121.0 (*C11*), 120.8 (*C9b*), 120.3 (*C4*), 115.4 (*C10*), 101.8 (*C3*), 39.0 (N–CH_3); ESI-MS: m/z calcd. for $C_{17}H_{13}AgN$ 338.0093 found 338.0087 [M+Ag$^+$].

Phenanthro[1,2-b]furan (6b):

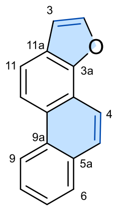

6b

Prepared according to the general procedure **I** using 7-vinyl-4-(2-vinylphenyl)benzofuran **5b** (17.2 mg, 70.0 µmol) to give the title compound **6b** as a white solid (14.1 mg, 64.6 µmol, 92%, m.p. 112.0 – 112.5°C): R_f 0.47 (cyclohexane/EtOAc 5:1); ν_{max} (neat): 3055w, 2923w, 2848w, 1920w, 1690w, 1599m, 1524w, 1489w, 1375w, 1307m, 1124m, 1037m, 941w, 862m, 811s; ^{1}H NMR (500 MHz, $CDCl_3$) δ = 8.73 (1H, d, ^3J 8.3 Hz, C9*H*), 8.56 (1H, d, ^3J 8.6 Hz, C10*H*), 8.31 (1H, d, ^3J 8.9 Hz, C4*H*), 7.95 (1H, d, ^3J 7.9 Hz, C6*H*), 7.90 (1H, d, ^3J 8.9 Hz, C5*H*), 7.85 (1H, d, ^3J 8.6 Hz, C11*H*), 7.82 (1H, d, ^3J 2.1 Hz, C2*H*), 7.68 (1H, ddd, ^3J 8.3, ^3J 7.0, ^4J 1.4 Hz, C8*H*), 7.60 (1H, ddd, ^3J 8.0, ^3J 7.0, ^4J 1.1 Hz, C7*H*), 6.97 (1H, d, ^3J 2.1 Hz, C3*H*); ^{13}C NMR (126 MHz, $CDCl_3$) δ = 151.7 (*C3a*), 144.9 (*C2*), 131.8 (*C5a*), 130.8 (*C9a*), 129.1 (*C6*), 127.7 (*C5*), 127.6 (*C9b*), 127.1 (*C8*), 126.3 (*C7*), 124.3 (*C11a*), 123.1 (*C9*), 119.9 (*C11*), 119.2 (*C3b*), 119.1 (*C4*), 118.1 (*C10*), 107.5 (*C3*); ESI-MS: m/z calcd. for $C_{32}H_{20}AgO_2$ 543.0509 found 543.0515 [2M+Ag$^+$].

Bidirectional Twofold ArROM – RCM to Polycyclic Aromatic Hydrocarbons (PAHs)

Optimization Studies (3.00 µmol scale): Performed accroding to the general procedure **F** utilizing 5,5'-(2,5-divinyl-1,4-phenylene)bis(9,11-dimethylphenanthro[3,4-d][1,3]dioxole) **7a** (1.88 mg, 3.00 µmol) or 8,8'-(2,5-divinyl-1,4-phenylene)bis(2,4-dimethoxy-5-methylphenanthrene) **7b** (1.89 mg, 3.00 µmol) and toluene (0.15 mL).

Supplementary Table 4. Reaction conditions optimization: biderctional twofold ArROM – RCM to PAHs

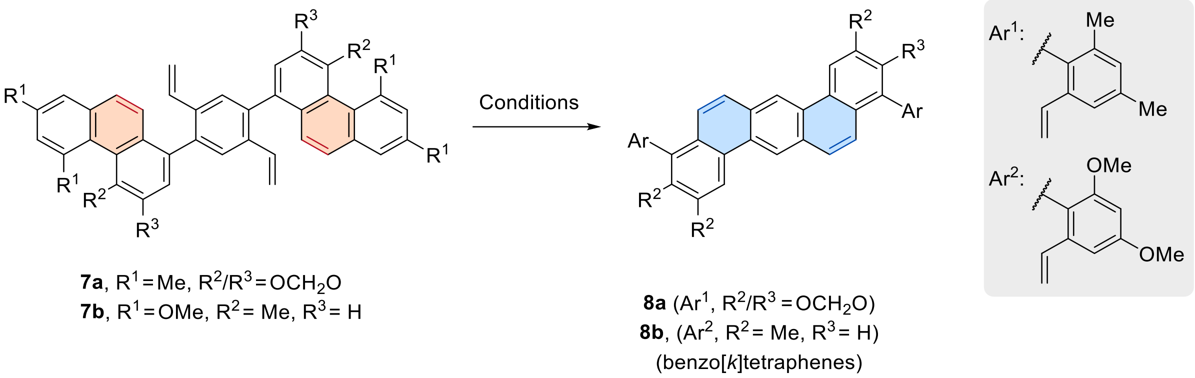

Entry[a]	Substrate	Catalyst (mol%)	Temp. [°C]	Conversion[b] [%]	Yield[b] [%]
1	**7a**	**C1** (20)	65	NR	–
2	**7a**	**C1** (20)	85	>95	–
3	**7b**	**C1** (20)	85	>95	65
4	**7b**	**C1** (10)	85	9	5

[a]Reactions were performed on 3.00 µmol scale of **7a**/**7b** for 18 h. [b]Conversion and yield were determined by ^{1}H-NMR with durene as an internal standard.

Benzo[*k*]tetraphene (8a):

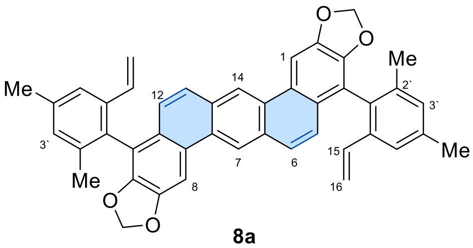

8a

Prepared according to the general procedure **G** using 5,5'-(2,5-divinyl-1,4-phenylene)bis(9,11-dimethylphenanthro[3,4-d][1,3]dioxole) **7a** (13.8 mg, 22.0 µmol) and **C1** (3.66 mg, 4.40 µmol, 20 mol%) in toluene (1.2 mL) to give the title compound as a white solid (9.20 mg, 14.7 µmol, 67%, decomposition without melting at 293 – 295°C): R$_f$ 0.54 (cyclohexane/EtOAc 5:1); ν_{max}(neat): 3003w, 2949w, 2916w, 2851w, 1728w, 1604w, 1453s, 1411m, 1244s, 1164m, 1101w, 1045s, 945w, 851w; ^{1}H NMR (500 MHz, $CDCl_3$) δ = 8.96 (2H, s, C7*H*, C14*H*), 8.28 (2H, s, C1*H*, C8*H*), 7.75 (2H, d, ^{3}J 9.2 Hz, C6*H*, C13*H*), 7.47 (2H, s, C5`*H*), 7.20 (2H, d, $^{3}J$ 9.2 Hz, C5*H*, C12*H*), 7.17 (2H, s, C3`*H*), 6.47 – 6.24 (2H, m, C15*H*), 6.13 – 5.97 (4H, m, OC*H*$_2$O), 5.67 (2H, d, ^{3}J 17.4 Hz, C16*H*), 5.09 – 4.93 (2H, m, C16*H*), 2.46 (6H, s, C4`–C*H*$_3$), 2.03 (6H, s, C2`–C*H*$_3$); ^{13}C NMR (126

MHz, $CDCl_3$) δ = 147.6 (*C2*, *C9*), 145.4 (*C3*, *C10*), 138.2 (*C4`*), 137.9 (*C2`*), 135.3 (*C1`*), 130.7 (*C3`*), 130.3 (*C6a*, *C13a*), 129.5 (*C6`*), 128.9 (*C7a*, *C14a*), 127.2 (*C4a*, *C11a*), 126.7 (*C4b*, *C7b*), 126.1 (*C6*, *C13*), 124.0 (*C5*, *C12*), 123.5 (*C5`*), 121.9 (*C7*, *C14*), 117.3 (*C4*, *C11*), 114.9 (*C16*), 101.6 (OCH_2O), 101.1 (*C1*, *C8*), 21.6 (C4`–$CH_3$), 20.3 (C2`–CH_3); ESI-MS: m/z calcd. for $C_{44}H_{34}O_4$ 626.2452 found 626.2457 [$M^{+\bullet}$].

4,11-Bis(2,4-dimethoxy-6-vinylphenyl)-3,10-dimethylbenzo[*k*]tetraphene (8b):

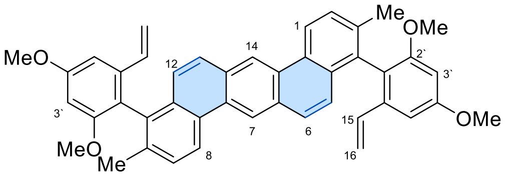

8b

Prepared according to the general procedure **G** using 8,8'-(2,5-divinyl-1,4-phenylene)bis(2,4-dimethoxy-5-methylphenanthrene) **7b** (18.9 mg, 30.0 µmol) and **C1** (5.00 mg, 6.00 µmol, 20 mol%) in toluene (1.5 mL) to give the title compound as a beige solid (10.6 mg, 16.8 µmol, 56%, decomposition without melting at 290 – 293°C): R_f 0.36 (cyclohexane/EtOAc 5:1); ν_{max}(neat): 3055w, 3003w, 2935m, 2849w, 1600s, 1570s, 1457m, 1421m, 1277w, 1201s, 1155s, 1083w, 1034w, 913w, 828w; ^{1}H NMR (500 MHz, $CDCl_3$) δ = 9.10 (2H, s, C14*H*, C7*H*), 8.80 (2H, d, ^3J 8.5 Hz, C1*H*, C8*H*), 7.79 (2H, d, ^3J 9.3 Hz, C6*H*, C13*H*), 7.63 (2H, d, ^3J 8.5 Hz, C8*H*, C9*H*), 7.26 (2H, d, ^3J 9.3 Hz, C5*H*, C12*H*), 6.93 (2H, d, ^4J 2.3 Hz, C5`*H*), 6.61 (2H, d, $^4J$ 2.3 Hz, C3`*H*), 6.23 – 6.00 (2H, m, C15*H*), 5.64 (2H, d, ^3J 17.4 Hz, C16*H*), 4.97 (2H, d, ^3J 10.9 Hz, C16*H*), 3.97 (6H, s, C4`–O$CH_3$), 3.65 (3H, s, C2`–OCH_3), 3.65 (3H, s, C3`–C$H_3$, C10`–CH_3), 2.19 (3H, s, C3`–C$H_3$, C10`–CH_3); ESI-MS: m/z calcd. for $C_{44}H_{39}O_4$ 631.2843 found 631.2841 [$M+H^+$].

4,10-Bis(2,4-dimethoxy-6-vinylphenyl)-3,11-dimethylbenzo[*m*]tetraphene (8c):

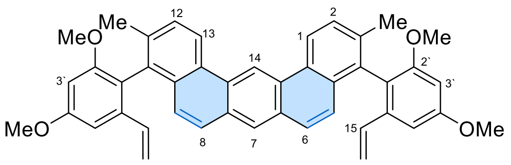

8c

Prepared according to the general procedure **G** 8,8'-(4,6-divinyl-1,3-phenylene)bis(2,4-dimethoxy-5-methylphenanthrene) **7c** (17.7 mg, 28.0 µmol) and **C1** (4.66 mg, 5.60 µmol, 20 mol%) in toluene (1.4 mL) to give the title compound as a white solid (11.1 mg, 17.6 µmol, 63%, m.p. 168 – 169°C): R_f 0.32 (cyclohexane/EtOAc 5:1); ν_{max}(neat): 2992w, 2930w, 2850w, 1735w, 1599m, 1570m, 1455m, 1420m, 1315m, 1276w, 1201s, 1154s, 1080m, 1033w, 917w; ^{1}H NMR (500 MHz, $CDCl_3$) δ = 10.09 (1H, s, C14*H*), 8.99 (2H, d, ^3J 8.4 Hz, C1*H*, C13*H*), 8.23 (1H, s, C7*H*), 7.86 – 7.62 (4H, m, C2*H*, C13*H*), 7.24 (2H, d, ^3J 9.2 Hz, C5*H*, C9*H*), 6.93 (2H, d, ^4J 2.2 Hz, C5`*H*), 6.61 (2H, d, $^4J$ 2.0 Hz, C3`*H*), 6.40 – 6.03 (2H, m, C15*H*), 5.64 (2H, d, ^3J 17.4, C16*H*), 4.97 (2H, d, ^3J 11.1 Hz, C16*H*), 3.97 (6H, s, C4`–OC*$H_3$*), 3.66 (3H, s, C2`–OC*H_3*), 3.65 (3H, s, C2`–OC*$H_3$*), 2.21 (6H, s, C3–C*$H_3$*, C11–C*$H_3$*); $^{13}$C NMR (126 MHz, $CDCl_3$) δ = 160.4 (*C4`*), 158.6 (*C2`*), 138.6 (*C6`*), 136.5 (*C3*, *C11*), 135.0 (*C15*), 134.2 (*C4*, *C10*), 131.7 (*C4a*, *C9a*), 130.2 (*C6a*, *C7a*), 129.4 (*C13b*, *C14a*), 129.0 (*C13a*, *C14b*), 128.8 (*C2*, *C12*), 127.0 (*C7*), 126.9 (*C6*, *C8*), 125.1 (*C5*, *C9*), 122.2 (*C1*, *C13*), 120.6 (*C1`*), 116.2 (*C14*), 115.1 (*C16*), 100.9 (*C5`*), 98.7 (*C3`*), 56.0 (C2`–OCH_3), 55.6 (C4`–OCH_3), 20.6 (C3–*C*H_3, C11–*C*H_3); ESI-MS: m/z calcd. for $C_{88}H_{77}O_8$ 1261.5613 found 1261.5632 [$2M+H^+$].

Dibenzo[*a,j*]tetracene (8d):

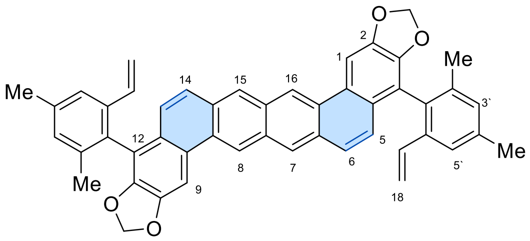

8d

Prepared according to the general procedure **G** using 5,5'-(3,7-divinylnaphthalene-2,6-diyl)bis(9,11-dimethylphenanthro[3,4-d][1,3]dioxole) **7d** (6.77 mg, 10.0 µmol) and **C1** (1.67 mg, 2.00 µmol, 20 mol%) in toluene (0.5 mL) to give the title compound as a yellow solid (2.10 mg, 3.1 µmol, 31%): R_f 0.57 (cyclohexane/EtOAc 5:1); ^{1}H NMR (400 MHz, $CDCl_3$) δ = 9.22 (2H, s, C8*H*, C16*H*), 8.62 (2H, s, C1*H*, C9*H*), 8.33 (2H, s, C7*H*, C15*H*), 7.65 (2H, d, ^3J 9.3 Hz, C6*H*, C14*H*), 7.46 (2H, s, C5`*H*), 7.16 (2H, s, C3`*H*), 7.09 (2H, d, ^3J 9.3 Hz, C5*H*, C13*H*), 6.36 (2H, dd, ^3J 17.5, ^3J 11.1 Hz, C17*H*), 6.15 – 5.98 (4H, m, OCH_2O), 5.76 – 5.47 (2H, m, C18*H*), 5.01 (2H, d, ^3J 11.1 Hz, C18*H*), 2.45 (6H, s, C2`–C$H_3$), 2.04 (6H, s, C4`–CH_3); ESI-MS: m/z calcd. for $C_{48}H_{36}O_4$ 676.2608 found 676.2596 [$M^{+\bullet}$]. Rapid decomposition of **8d** at room temperature in $CDCl_3$ solution was observed (see the ^{1}H-NMR spectra below):

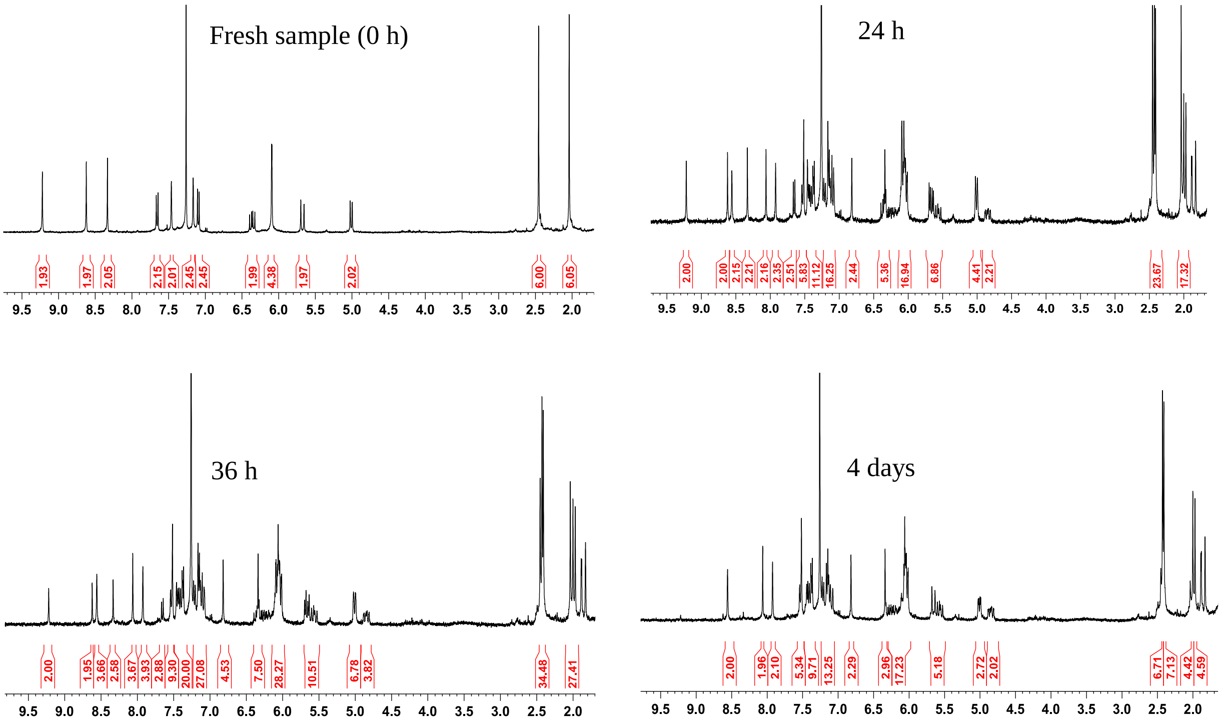

Equilibration of Indoles by ArROM – RCM

Optimization Studies: Performed accroding to the general procedure **F** utilizing 5-fluoro-1-(2-vinylphenyl)-1*H*-indole **9d** (1.78 mg, 7.50 µmol) and toluene (0.25 mL, 0.03 mol·L^{-1}) with the reaction time of 18 h.

Optimization Studies (70.0 µmol scale): Performed accroding to the general procedure **G** utilizing 5-fluoro-1-(2-vinylphenyl)-1*H*-indole **9d** (16.6 mg, 70.0 µmol) and toluene (2.3 mL, 0.03 mol·L^{-1}) with 18h reaction time.

Supplementary Table 5. Reaction conditions optimization equilibration of indoles by ArROM – RCM

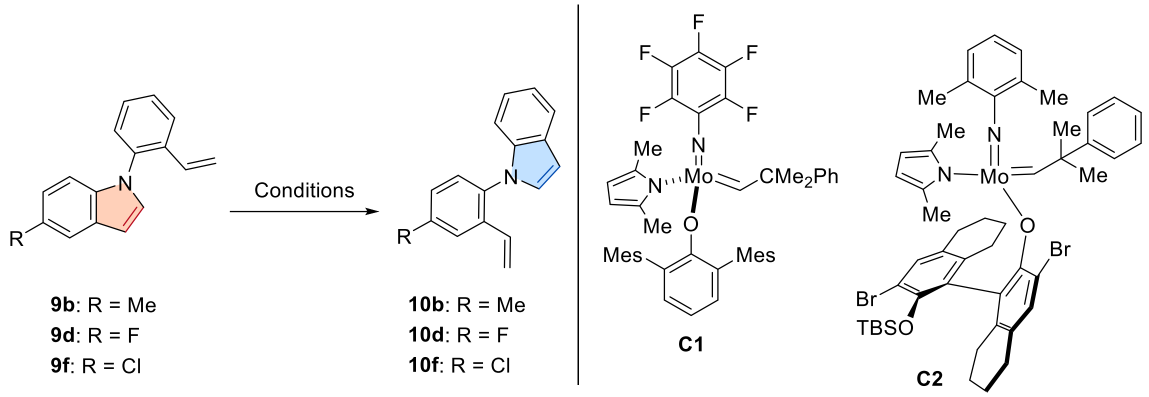

Entry	Substrate	Catalyst (mol%)	Solvent	Temp. [°C]	**(9 : 10)**[b]	Yield[b] [%]
1[a]	**9b**	**C1** (20)	Toluene	65	1 : 2.4	–[d]
2[a]	**9b**	**C2** (5)	Toluene	65	1 : 2.4	–[d]
3[a]	**9b**	**C2** (20)	Toluene	65	1 : 2.3	–[d]
4[a]	**9f**	**C1** (20)	Toluene	65	1 : 0.4	–[d]
5[a]	**9f**	**C2** (5)	Toluene	65	1 : 0.4	–[d]
6[a]	**9f**	**C2** (20)	Toluene	65	1 : 0.4	–[d]
7[a]	**9d**	**C1** (5)	Toluene	65	1 : 0.9	44 (**10d**)
8[a]	**9d**	**C1** (10)	Toluene	65	1 : 1	50 (**10d**)
9[a]	**9d**	**C1** (20)	Toluene	65	1 : 1	45 (**10d**)
10[a]	**9d**	**C1** (20)	Toluene	85	1 : 1	43 (**10d**)
11[a]	**9d**	**C2** (20)	Toluene	65	1 : 0.9	41 **(10d)**
12[c]	**9d**	**C1** (5)	Toluene	65	1 : 1	87[e]
13[c]	**9d**	**C2** (5)	Toluene	65	1 : 1	90[e]

[a]Reactions were performed on 7.50 µmol scale of **9b**, **9d**, **9f** for 18 h. [b]Conversion and yield were determined by ^{1}H-NMR with durene as an internal standard. [c]Reactions were performed on 70.0 µmol scale of **9d.** [d]Only a mixture of **9b/10b** or **9f/10f** was observed by ^{1}H-NMR, no internal standard was added [e]Isolated yield of the mixture.

General procedure J for the equilibration of indoles by ArROM – RCM:

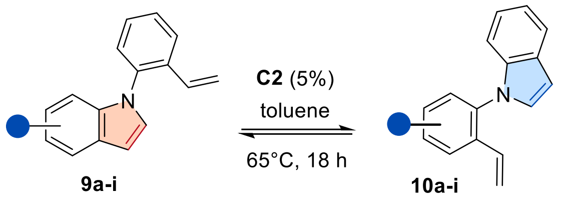

Catalyst **C2** (3.52 mg, 3.50 µmol, 5 mol%) was weighed into a 10 mL crimp cap vial (dried at 130°C overnight) in an argon-filled glovebox. A solution of the corresponding indole **9a-i** (70.0 µmol, 1.00 eq.) in dry and degassed toluene (2.3 mL, 0.03 mol·L^{-1}) was added. The vial was capped in the glovebox, subsequently taken out and stirred at 65°C for 18 h. The reaction mixture was then filtered through a thin layer of silica gel using EtOAc (10-15 mL). The solvents were removed under reduced pressure and the residue was purified by the silica gel chromatography (cyclohexane/EtOAc 100:0→80:20) to give the inseparable mixture of the starting material **9a-i** and the corresponding product **10a-i**. The yield was reported for the obtained mixture, while the ratio of **9a-i** and **10a-i** was analyzed by ^{1}H-NMR of the crude and after the purification of silica gel chromatography.

1-(4-Methoxy-2-vinylphenyl)-1*H*-indole (10a):

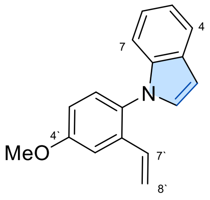

10a

Prepared accroding to the general procedure **J** using 5-methoxy-1-(2-vinylphenyl)-*1H*-indole **9a** (17.5 mg, 70.0 µmol) to obtain the mixture of **9a** and **10a** (1:5.3) as yellow oil (17.1 mg, 68.6 µmol, 98%). ^{1}H-NMR of both crude and purified mixture show the ratio **9a** : **10a** = 1 : 5.3. R_f (**mixture, 1 spot**) 0.31 (cyclohexane/EtOAc 9:1); ν_{max}(neat) (mixture): 3052w, 2992w, 2934w, 2835w, 1610w, 1571wm 1496s, 1458m, 1291m, 1223s, 1161w, 1098w, 1029w, 918w, 853w, 818w, 743m; ^{1}H NMR (**10a**) (500 MHz, $CDCl_3$) δ = 7.71 – 7.65 (1H, m, C4*H*), 7.27 – 7.22 (2H, m, C3\`*H*, C6\`*H*), 7.17 – 7.11 (3H, m, C2*H*, C5*H*, C6*H*), 7.07 – 7.02 (1H, m, C7*H*), 6.93 (1H, dd, ^{3}J 8.6, ^{4}J 2.9 Hz, C5\`*H*), 6.66 (1H, d, $^{3}J$ 2.1 Hz, C3*H*), 6.22 (1H, dd, $^{3}J$ 17.5, $^{3}J$ 11.0 Hz, C7\`*H*), 5.72 – 5.67 (1H, m, C8\`*H*), 5.16 (1H, dd, $^{3}J$ 11.0, $^{2}J$ 0.7 Hz, C8\`*H*), 3.91 (3H, s, OC*H*3); ^{13}C NMR (**10a**) (126 MHz, $CDCl_3$) δ = 159.5 (*C4\`*), 137.9 (*C7a*), 136.6 (*C2\`*), 132.2 (*C7\`*), 130.4 (*C1\`*), 129.8 (*C2*), 129.6 (*C6\`*), 128.4 (*C3a*), 122.2 (*C6*), 120.9 (*C4*), 120.1 (*C5*), 116.5 (*C8\`*), 114.4 (*C5\`*), 110.9 (*C7*), 110.8 (*C3\`*), 102.6 (*C3*), 55.7 (OCH_3); ESI-MS (mixture): m/z calcd. for $C_{17}H_{15}O$ 249.1148 found 249.1138 [$M^{+\cdot}$].

1-(4-Methyl-2-vinylphenyl)-1*H*-indole (10b):

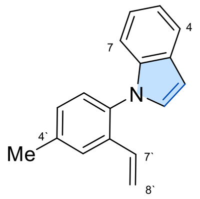

10b

Prepared accroding to the general procedure **J** using 5-methyl-1-(2-vinylphenyl)-*1H*-indole **7c** (16.3 mg, 70.0 µmol) to obtain the mixture of **9b** and **10b** (1:2.4) as yellow oil (15.9 mg, 68.1 µmol, 97%). ^{1}H-NMR of both crude and purified mixture show the ratio **9b** : **10b** = 1 : 2.4. R_f (**mixture, 1 spot**) 0.31 (cyclohexane/EtOAc 9:1); ν_{max}(neat) (mixture): 3078w, 3027w, 2923w, 2857w, 1611w, 1494s, 1457s, 1336m, 1310m, 1222m, 1158w, 1058w, 991w, 915m, 822m, 741s; ^{1}H NMR (**10b**) (500 MHz, $CDCl_3$) δ = 7.72 – 7.67 (1H, m, C4*H*), 7.56 (1H, s, C3\`*H*), 7.22 – 7.20 (2H, m, C5\`*H*, C6\`*H*), 7.18 – 7.14 (3H, m, C5*H*, C6*H*, C2*H*), 6.67 (1H, dd, ^{3}J 3.2, ^{4}J 0.8 Hz, C3*H*), 6.27 (1H, dd,

^3J 17.5, ^3J 11.0 Hz, C7\`*H*), 5.71 (1H, dd, $^3J$ 17.5, $^2J$ 1.1 Hz, C8\`*H*), 5.15 (1H, dd, ^3J 11.0, ^2J 1.1 Hz, C8\`*H*), 2.47 (3H, s, C4\`–C*H*$_3$); ^{13}C NMR (**10b**) (126 MHz, $CDCl_3$) δ = 138.2 (*C4\`*), 137.7 (*C7a*), 134.9 (*C2\`*), 134.8 (*C1\`*), 132.3 (*C7\`*), 129.6 (*C2*), 129.5 (*C5\`*), 128.5 (*C3a*), 128.2 (*C6\`*), 126.8 (*C3\`*), 122.2 (*C6*), 120.9 (*C4*), 120.1 (*C5*), 116.0 (*C8\`*), 111.0 (*C7*), 102.7 (*C3*), 21.4 (C4\`–*C*H$_3$); ESI-MS (of the mixture): m/z calcd. for $C_{17}H_{15}AgN$ 340.0250 found 340.0245 [M+Ag$^+$].

1-(2-Fluoro-6-vinylphenyl)-1*H*-indole (10c):

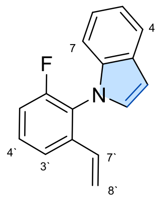

10c

Prepared accroding to the general procedure **J** using 7-fluoro-1-(2-vinylphenyl)-*1H*-indole **9c** (16.6 mg, 70.0 µmol) to obtain the mixture of **9c** and **10c** (1:2.2) as yellow oil (14.5 mg, 61.1 µmol, 87%). ^{1}H-NMR of both crude and purified mixture show the ratio **9c** : **10c** = 1 : 2.2. R$_f$ (**10c**) 0.64 (cyclohexane/EtOAc 9:1); ν_{max}(neat) (mixture): 3070w, 2962w, 2906w, 1701w, 1576m, 1487s, 1452m, 1328m, 1261s, 1135m, 1105w, 1013w, 949m, 921m, 802s, 741s; ^{1}H NMR (**10c**) (500 MHz, $CDCl_3$) δ 7.74 – 7.68 (1H, m, C4*H*), 7.54 (1H, d, ^3J 8.0 Hz, C3\`*H*), 7.38 – 7.33 (1H, m, C4\`*H*), 7.22 – 7.16 (3H, m, C4*H*, C5*H*, C5\`*H*), 7.14 (1H, dd, $^3J$ 3.2, $^5J$ 0.7 Hz, C2*H*), 7.01 – 6.96 (1H, m, C7*H*), 6.73 (1H, dd, $^3J$ 3.2, $^4J$ 0.9 Hz, C3*H*), 6.21 (1H, dd, $^3J$ 17.5, $^3J$ 11.1 Hz, C7\`*H*), 5.74 (1H, dd, ^3J 17.5, ^2J 0.9 Hz, C8\`*H*), 5.22 (1H, d, $^3J$ 11.1 Hz, C8\`*H*); ^{13}C NMR (**10c**) (126 MHz, $CDCl_3$) δ = 159.0 (d, $^1J_{CF}$ 251.0 Hz, *C6\`*), 138.4 (*C2\`*), 137.5 (*C7a*), 131.9 (*C7\`*), 131.3 (d, $^2J_{CF}$ 3.3 Hz, *C1\`*), 129.4 (*C2*), 128.29 (d, $^3J_{CF}$ 2.0 Hz, *C4\`*), 128.4 (*C3a*), 122.5 (*C6*), 121.4 (d, $^3J_{CF}$ 3.6 Hz, C3\`*H*), 121.0 (*C4*), 120.4 (*C5*), 117.8 (*C8\`*), 115.5 (d, $^2J_{CF}$ 20.5 Hz, C5\`*H*), 110.7 (*C7*), 103.6 (*C3*); ^{19}F NMR (**10c**) (376 MHz, $CDCl_3$) δ = –121.39; ESI-MS (of the mixture): m/z calcd. for $C_{16}H_{13}FN$ 238.1027 found 238.1022 [M+H$^+$].

1-(4-Fluoro-2-vinylphenyl)-1*H*-indole (10d):

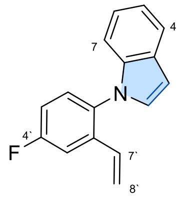

10d

Prepared accroding to the general procedure **J** using 5-fluoro-1-(2-vinylphenyl)-*1H*-indole **9d** (16.6 mg, 70.0 µmol) to obtain the mixture of **9d** and **10d** (1:1) as a transparent oil (14.9 mg, 62.8 µmol, 90%). ^{1}H-NMR of both crude and purified mixture show the ratio **9d** : **10d** = 1 : 1. R$_f$ (**10d**) 0.66 (cyclohexane/EtOAc 9:1); ν_{max}(neat) (mixture): 3062w, 2962w, 2905w, 1511m, 1493s, 1474m, 1334w, 1268w, 1214w, 1145w, 1066w, 920w, 860w, 804w, 745w; ^{1}H NMR (**10d**) (500 MHz, $CDCl_3$) δ = 7.71 – 7.68 (1H, m, C4*H*), 7.44 – 7.41 (1H, m, C3\`*H*), 7.32 – 7.29 (1H, m, C6\`*H*), 7.21 – 7.16 (2H, m, C5*H*, C6*H*), 7.15 (1H, d, ^3J 3.1 Hz, C2*H*), 7.10 (1H, ddd, ^3J 8.6, ^3J 7.8, ^4J 2.9 Hz, C5\`*H*), 7.07 – 7.03 (1H, m, C7*H*), 6.69 (1H, dd, $^3J$ 3.2, $^4J$ 0.8 Hz, C3*H*), 6.22 (1H, dd, $^3J$ 17.5, $^3J$ 11.1,C7\`*H*), 5.73 (1H, dd, ^3J 17.5, ^2J 1.1 Hz, C8\`*H*), 5.23 (1H, d, $^3J$ 11.1 Hz, C8\`*H*); ^{13}C NMR (**10d**) (126 MHz, $CDCl_3$) δ = 162.41 (d, $^1J_{CF}$ 247.3 Hz, *C4\`*), 137.7 (*C7a*), 137.5 (d, $^3J_{CF}$ 8.2 Hz, *C2\`*), 133.3 (d, $^4J_{CF}$ 2.8 Hz, *C1\`*), 131.4 (d, $^4J_{CF}$ 2.1 Hz, *C7\`*), 130.2 (d, $^3J_{CF}$ 8.9 Hz, *C6\`*), 129.5 (*C2*), 128.5 (*C3a*), 122.5 (*C6*), 121.0 (*C4*), 120.3 (*C5*), 117.6 (*C8\`*), 115.7 (d, $^2J_{CF}$ 23.0 Hz, *C5\`*), 112.6 (d, $^2J_{CF}$ 23.3 Hz,

C3`), 110.8 (*C7*), 103.2 (*C3*); ^{19}F NMR (**10d**) (376 MHz, $CDCl_3$) δ = –113.20; ESI-MS (of the mixture): m/z calcd. for $C_{16}H_{12}FN$ 237.0948 found 237.0944 [$M^{+\cdot}$].

1-(5-Fluoro-2-vinylphenyl)-1*H*-indole (10e):

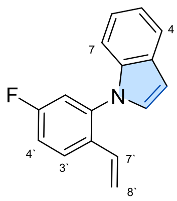

10e

Prepared accroding to the general procedure **J** using 6-fluoro-1-(2-vinylphenyl)-*1H*-indole **9e** (16.6 mg, 70.0 µmol) to obtain the mixture of **9e** and **10e** (1:0.5) as yellow oil (16.0 mg, 67.4 µmol, 96%). ^{1}H-NMR of both crude and purified mixture show the ratio **9e** : **10e** = 1 : 0.5. R_f (**mixture, 1 spot**) 0.70 (cyclohexane/EtOAc 9:1); ν_{max}(neat) (mixture): 3065w, 3013w, 1619m, 1606w, 1486s, 1462s, 1342w, 1214s, 1177w, 1118w, 992w, 933w, 827w, 766w, 716w, 676w; ^{1}H NMR (**10e**) (500 MHz, $CDCl_3$) δ = 7.74 – 7.67 (2H, m, C4*H*, C3`*H*), 7.22 – 7.16 (4H, m, C2*H*, C5*H*, C6*H*, C4`*H*), 7.15 – 7.11 (1H, m, C7*H*), 7.09 (1H, dd, $^3J_{HF}$ 9.0, ^4J 2.7 Hz, C6`*H*), 6.69 (1H, dd, $^3J$ 3.2, $^4J$ 0.8 Hz, C3*H*), 6.28 (1H, dd, $^3J$ 17.5, $^3J$ 11.1 Hz, C7`*H*), 5.67 (1H, d, ^3J 17.5 Hz, C8`*H*), 5.17 (1H, d, $^3J$ 11.1 Hz, C8`*H*); ^{13}C NMR (**10e**) (126 MHz, $CDCl_3$) δ = 162.4 (d, $^1J_{CF}$ 249.8 Hz, *C5*`), 138.4 (d, $^3J_{CF}$ 9.5 Hz, *C1*`), 137.3 (*C7a*), 136.8 (*C2*`), 131.6 (*C7*`), 129.2 (*C2*), 128.7 (*C3a*), 128.0 (d, $^3J_{CF}$ 9.1 Hz, *C3*`), 122.6 (*C6*), 121.1 (*C4*), 120.5(*C5*), 116.1 (*C8*`), 115.6 (d, $^2J_{CF}$ 21.3 Hz, *C4*`), 115.2 (d, $^2J_{CF}$ 22.5 Hz, *C6*`), 110.9 (*C7*), 103.6 (*C3*); ^{19}F NMR (**10e**) (376 MHz, $CDCl_3$) δ = –112.6; ESI-MS (of the mixture): m/z calcd. for $C_{16}H_{13}FN$ 238.1027 found 238.1017 [$M+H^+$].

1-(4-Chloro-2-vinylphenyl)-1*H*-indole (10f):

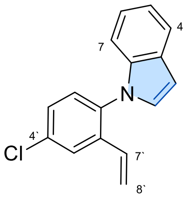

10f

Prepared accroding to the general procedure **J** using 5-chloro-1-(2-vinylphenyl)-*1H*-indole **9f** (17.8 mg, 70.0 µmol) to obtain the mixture of **9f** and **10f** (1:0.4) as a transparent oil (17.0 mg, 67.0 µmol, 96%). ^{1}H-NMR of both crude and purified mixture show the ratio **9f** : **10f** = 1 : 0.4. R_f **(mixture, 1 spot)** 0.74 (cyclohexane/EtOAc 5:1); ν_{max}(neat) (mixture): 3060w, 2926w, 2851w, 1850w, 1600w, 1569w, 1487s, 1461s, 1329m, 1286w, 1230w, 1203w, 1149w, 1121w, 1064w, 990w, 955w, 917w, 754s; ^{1}H NMR (500 MHz, $CDCl_3$) δ = 7.72 (d, ^4J 2.4 Hz, C3`*H*), 7.71 – 7.68 (1H, m, C4*H*), 7.37 (1H, dd, $^4J$ 8.4, $^3J$ 2.4 Hz, C5`*H*), 7.28 (1H, d, ^3J 8.4 Hz, C6`*H*), 7.20 – 7.16 (2H, m, C5*H*, C6*H*), 7.15 (1H, d, $^3J$ 3.2 Hz, C2*H*), 7.09 – 7.05 (1H, m, C7*H*), 6.69 (1H, dd, $^3J$ 3.2, $^5J$ 0.8 Hz, C3*H*), 6.25 (1H, dd, $^3J$ 17.5, $^3J$ 11.0 Hz, C7`*H*), 5.75 (1H, dd, ^3J 17.5, ^2J 0.9 Hz, C8`*H*), 5.24 (1H, dd, $^3J$ 11.0, $^2J$ 0.9 Hz, C8`*H*); ^{13}C NMR (126 MHz, $CDCl_3$) δ = 137.5 (*C7a*), 136.8 (*C2*`), 135.8 (*C1*`), 134.2 (*C4*`), 131.3 (*C7*`), 129.6 (*C6*`), 129.3 (*C2*), 128.7 (*C5*`), 128.6 (*C3a*), 126.4 (*C3*`), 122.5 (*C6*), 121.1 (*C4*), 120.4 (*C5*), 117.7 (*C8*`), 110.8 (*C7*), 103.5 (*C3*); ESI-MS (of the mixture): m/z calcd. for $C_{16}H_{12}ClN$ 253.0653 found 253.0650 [$M^{+\cdot}$].

1-(5-Chloro-2-vinylphenyl)-1*H*-indole (10g):

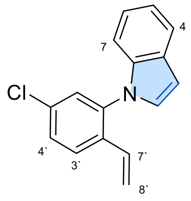

10g

Prepared accroding to the general procedure **J** using 6-chloro-1-(2-vinylphenyl)-1*H*-indole **9g** (17.8 mg, 70.0 µmol) to obtain the mixture of **9g** and **10g** (1:0.3) as yellow oil (16.0 mg, 67.4 µmol, 96%). ^{1}H-NMR of both crude and purified mixture show the ratio **9g** : **10g** = 1 : 0.3. R_f (**mixture, 1 spot**) 0.36 (cyclohexane/EtOAc 9:1); ν_{max}(neat) (mixture): 3063w, 3021w, 2926w, 2841w, 1713w, 1597m, 1507m, 1487s, 1335m, 1141m, 1108w, 1080w, 991w, 846w, 906w, 767w; ^{1}H NMR (500 MHz, $CDCl_3$) δ = 7.72 – 7.66 (2H, m, C4*H*, C3`*H*), 7.43 – 7.40 (1H, m, C4`*H*), 7.36 (1H, d, ^{4}J 2.2 Hz, C6`*H*), 7.23 – 7.17 (2H, m, C5*H*, C6*H*), 7.16 (1H, d, $^{3}J$ 3.2 Hz, C2*H*), 7.12 – 7.09 (1H, m, C7*H*), 6.70 (1H, dd, $^{3}J$ 3.2, $^{5}J$ 0.8 Hz, C3*H*), 6.28 (1H, dd, $^{3}J$ 17.4, $^{3}J$ 11.0 Hz, C7`*H*), 5.72 (1H, dd, ^{3}J 17.4, ^{2}J 0.9 Hz, C8`*H*), 5.23 – 5.19 (1H, m, C8`*H*); ^{13}C NMR (126 MHz, $CDCl_3$) δ = 138.1 (*C1`*), 137.3 (*C7a*), 133.9 (*C5`*), 133.8 (*C2`*), 131.4 (*C7`*), 129.2 (*C2*), 128.63 (*C3a*), 128.55 (*C4`*), 128.3 (*C6`*), 127.5 (*C3`*), 122.6 (*C6*), 121.1 (*C4*), 120.5 (*C5*), 116.9 (*C8`*), 110.9 (*C7*), 103.7 (*C3*); ESI-MS (of the mixture): m/z calcd. for $C_{16}H_{13}ClN$ 254.0731 found 254.0720 [M+H^{+}].

Supplementary Table 6. Gibbs free energies for selected pairs of idoles **9** and **10** obtained by density functional theory (DFT) calculations

SMD(toluene)/B3LYP-D3(BJ)/def2-TZVP//B3LYP-D3(BJ)/def2-TZVP

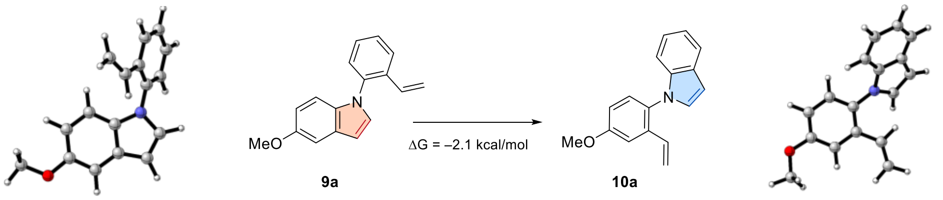

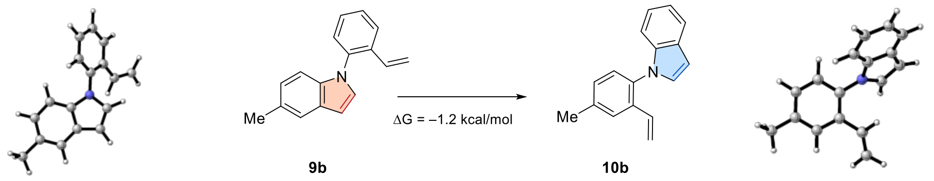

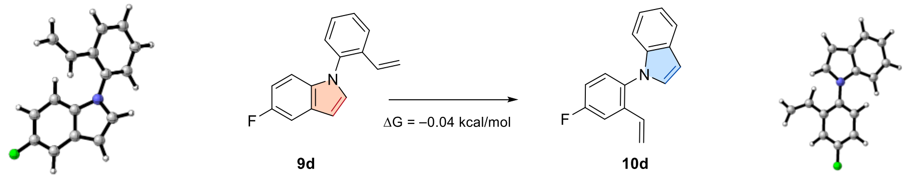

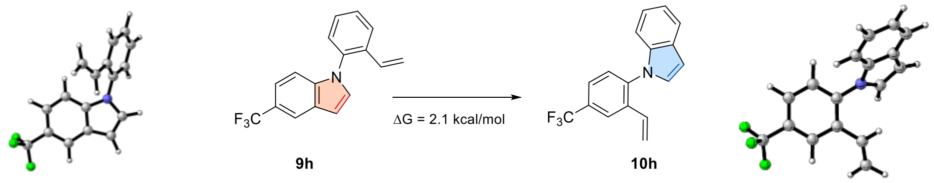

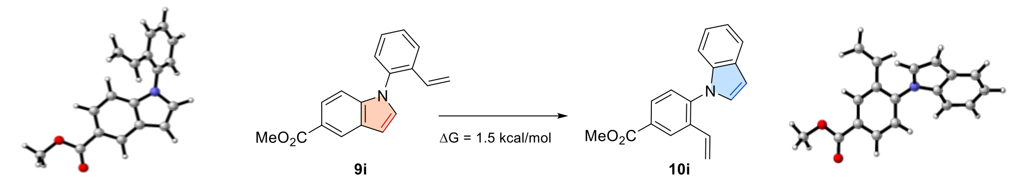

Density functional theory (DFT) calculations were performed using the Gaussian 16 (revision C.01) program. Geometry optimizations were performed at the B3LYP-D3(BJ) level of theory with the def2-TZVP basis set in the gas phase.[12] Harmonic vibrational frequencies were evaluated for the optimized geometries, with minima characterized by the absence of imaginary frequencies. Quasiharmonic corrections to the entropy for frequencies below 100 cm^{-1} were calculated with the Goodvibes program by employing the method of Grimme. Single point energies were calculated at B3LYP-D3(BJ) level of theory with the def2-TZVP basis set with implicit solvation model SMD for toluene. The optimized structures were visualized in CYLView[13].

Supplementary Table 7. Hammett plots for experimental and DFT calculated data of indoles equilibration

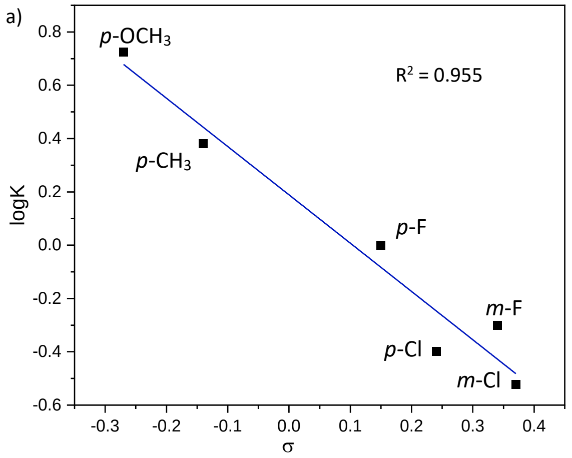

Hammett plot for experimental equilibrium constants

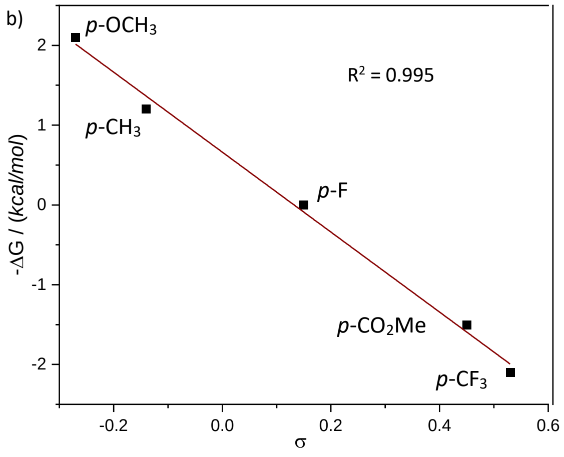

Hammett plot for DFT calculated Gibbs free energies

Cartesian Coordinates for Optimized Geometries

9a:

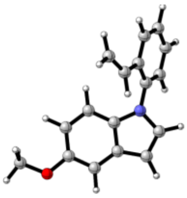

G_sol = –786.925232

C -2.28315700 0.95764900 -0.68909600
C -3.24804100 0.05734700 -0.20184800
C -2.87111700 -1.13770800 0.39408700
C -1.51335000 -1.43685600 0.51166300
C -0.55329600 -0.51081700 0.03061300
C -0.92832400 0.67764900 -0.57750000
C -0.78435800 -2.55725200 1.03248400
C 0.54252600 -2.29150500 0.85692200
N 0.70714200 -1.04998000 0.26613900
O -4.59618600 0.28392700 -0.28097200
C -5.04457600 1.47987800 -0.88615900
C 1.94763500 -0.49549000 -0.13266600
C 2.37588900 0.74106000 0.38031300
C 3.60491400 1.23901600 -0.06936200
C 4.38272900 0.54050300 -0.97710500
C 3.95023000 -0.68994600 -1.46199200
C 2.73157900 -1.19965900 -1.04310300
C 1.57942600 1.44645800 1.39049800
C 1.63849200 2.74858800 1.66153200
H -2.58530900 1.88185800 -1.15701200
H -3.63617200 -1.81520300 0.74885000
H -0.19318100 1.37586800 -0.95151000
H -1.19551200 -3.44228400 1.48803900
H 1.40986600 -2.87142700 1.12403100
H -6.13105700 1.45048400 -0.84177400
H -4.72897400 1.54571800 -1.93229200
H -4.68639600 2.36337100 -0.34808700
H 3.96911500 2.17359500 0.33526300
H 5.33345800 0.94706400 -1.29621100
H 4.55416600 -1.24397000 -2.16831200
H 2.36239300 -2.14083800 -1.42819800
H 0.87823000 0.83172200 1.94190200
H 2.28244800 3.42740100 1.11622500
H 1.02321000 3.18160300 2.43820000

10a:

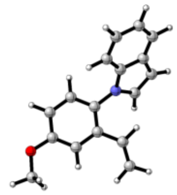

G_sol = –786.92865

C -2.11098600 -1.65828800 0.88839900
C -3.04784200 -0.84175000 0.24625500
C -2.64932300 0.37042600 -0.29976000
C -1.31542900 0.79578900 -0.21682500
C -0.39314900 -0.02529400 0.44173800
C -0.79943500 -1.24345000 0.98562200
C -0.87917000 2.05508900 -0.83198100
C 1.42271300 1.45413400 1.30593400
N 0.96284100 0.37010900 0.57491500
O -4.31671400 -1.32549000 0.19486600
C -5.31057600 -0.54372700 -0.44596500
C 2.05272800 -0.27603700 0.01699900
C 3.21808400 0.42948700 0.41245700
C 4.46516500 -0.02259000 -0.03468200
C 4.52230200 -1.13926000 -0.84914400
C 3.35387300 -1.81755600 -1.23414700
C 2.10515000 -1.39615100 -0.80896600
C 2.78319600 1.52335800 1.23098200
C -1.65608100 3.10040600 -1.10969600
H -2.43609600 -2.60212900 1.30366000
H -3.35438500 0.98836800 -0.83302600
H -0.06795100 -1.85732900 1.49364400
H 0.17793000 2.11640000 -1.06196800
H 0.71853600 2.08219000 1.82437000
H -6.23607000 -1.10756900 -0.36103800
H -5.07555000 -0.38889000 -1.50292200
H -5.43004400 0.42701300 0.04391200
H 5.37082900 0.49703100 0.25208800
H 5.48110100 -1.49810000 -1.20024700
H 3.43074500 -2.68494000 -1.87673700
H 1.20417600 -1.91423500 -1.10820500
H 3.40442100 2.26238200 1.70850300
H -2.71094100 3.12180400 -0.86503600
H -1.25019900 3.98364600 -1.58364600

9b:

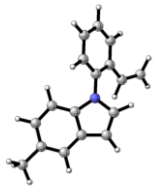

G_sol = –711.691284
C -2.70285900 -2.33639100 0.70125700
C -3.77699800 -1.75916400 0.03047400
C -3.66241600 -0.48047900 -0.48761100
C -2.48226200 0.26128200 -0.35339500
C -1.41432300 -0.33184500 0.34001400
C -1.52776800 -1.61935200 0.85820600
C -2.33861000 1.59316400 -0.95380600
C -0.07719800 1.58642000 1.20544800
N -0.20482200 0.37859500 0.53578500
C 1.05974100 -0.04137400 0.14921600
C 1.98788300 0.93386000 0.58532100
C 3.34732600 0.75385900 0.30065600
C 3.76649800 -0.36455900 -0.40090100
C 2.81146800 -1.31031600 -0.82886900
C 1.46124100 -1.16638500 -0.56675100
C 1.23534900 1.95402300 1.25523700
C -3.33222600 2.44213300 -1.20885100
C 5.22273700 -0.58386700 -0.71532000
H -2.78336400 -3.33640400 1.10637200
H -4.69754200 -2.31274400 -0.10031700
H -4.48586000 -0.05375300 -1.04414300
H -0.68920300 -2.04033200 1.39592000
H -1.32442800 1.88944700 -1.19547500
H -0.95339600 2.07220400 1.59888200
H 4.07124200 1.49140600 0.62657400
H 3.14886300 -2.17747100 -1.38397100
H 0.74506100 -1.89986500 -0.91103400
H 1.62258400 2.84440300 1.72154700
H -4.36088800 2.22436200 -0.94925400
H -3.14078900 3.39684800 -1.67946900
H 5.83429800 0.23975300 -0.34669200
H 5.38848200 -0.66855300 -1.79224500
H 5.59318700 -1.50553000 -0.25913200

10b:

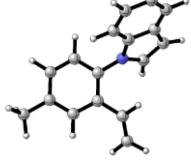

G_sol = –711.693176

C -2.38698100 -1.77833400 0.72597000
C -3.36581300 -0.99193100 0.11116200
C -2.99509500 0.25058100 -0.38220300
C -1.68611900 0.74004100 -0.28409500
C -0.73259800 -0.06769300 0.35096400
C -1.08842100 -1.31859200 0.84863800
C -1.30428300 2.03449900 -0.86146700
C 1.00038900 1.50148400 1.22088100
N 0.60327400 0.37988700 0.50833200
C 1.73566000 -0.25266400 0.02091700
C 2.86116400 0.50161500 0.44044200
C 4.13963900 0.07643600 0.06137500
C 4.26664600 -1.06308300 -0.71244900
C 3.13720900 -1.78947500 -1.12416900
C 1.85886600 -1.39515400 -0.76606200
C 2.35966300 1.60824400 1.20193100
C -2.12096300 3.06484900 -1.07299200
C -4.77851500 -1.49211900 -0.01807600
H -2.64823300 -2.75447600 1.11630000
H -3.73378700 0.85137000 -0.89695300
H -0.33489000 -1.91763000 1.34192200
H -0.25753700 2.13741800 -1.12317500
H 0.25760600 2.12411400 1.68920100
H 5.01523400 0.63465700 0.36818500
H 5.25026400 -1.40180300 -1.01085900
H 3.26778700 -2.67322300 -1.73499200
H 0.98902900 -1.95076200 -1.08859000
H 2.93811400 2.38062700 1.68036100
H -3.16687700 3.04217700 -0.79293200
H -1.75866200 3.97704500 -1.52715400
H -4.81099200 -2.45375500 -0.53481600
H -5.39865700 -0.78940300 -0.57386800
H -5.23401100 -1.63778700 0.96443300

9d:

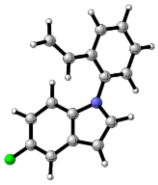

G_sol = –771.670593
C -2.42486200 1.24003600 -1.05366400
C -3.48776100 0.51039600 -0.51484200
C -3.32342900 -0.62559700 0.24223900
C -2.01203500 -1.05802700 0.48073700
C -0.92981300 -0.31235700 -0.05196700
C -1.12302000 0.82832400 -0.82508900
C -1.44647700 -2.17186400 1.18067400
C -0.08990400 -2.07729300 1.05648200
N 0.24321000 -0.95090300 0.32501300
F -4.74537800 0.95389800 -0.76249500
C 1.55994500 -0.58133800 -0.05088500
C 2.10056800 0.64740600 0.36330700
C 3.40005100 0.95689900 -0.05659400

C 4.13497900 0.08568100 -0.84265400
C 3.58755100 -1.13310600 -1.23103700
C 2.29899400 -1.45929200 -0.83858100
C 1.33886200 1.54080000 1.24333400
C 1.52810400 2.85194200 1.37479700
H -2.63872300 2.12035100 -1.64307700
H -4.18426600 -1.15551300 0.62589400
H -0.28844000 1.38153500 -1.23099100
H -1.97946400 -2.94000200 1.71500700
H 0.69088400 -2.70828100 1.44620100
H 3.84947000 1.88294700 0.27527100
H 5.14098300 0.34938200 -1.14158100
H 4.15737800 -1.82058600 -1.84181300
H 1.84270200 -2.38939400 -1.15024200
H 0.54859800 1.06947800 1.81529600
H 2.26860000 3.39275000 0.79866000
H 0.92769700 3.43292800 2.06120300

10d:

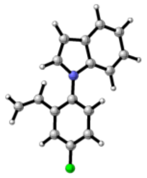

G_sol = –771.670654
C -2.40367000 -1.82174500 0.73617800
C -3.33531100 -1.01426900 0.10490400
C -3.01554700 0.22568300 -0.40326100
C -1.70831100 0.71247900 -0.28809900
C -0.76000800 -0.09151600 0.36616200
C -1.11070300 -1.34202200 0.86756200
C -1.32014600 2.00548200 -0.86375300
C 0.95916300 1.47154000 1.27477300
N 0.57110000 0.36375000 0.53505500
F -4.59935900 -1.46584000 -0.03242700
C 1.70758800 -0.24226200 0.02359000
C 2.82634400 0.51386400 0.45643800
C 4.10725700 0.11298400 0.05957300
C 4.24213300 -1.00494600 -0.74382900
C 3.11872100 -1.73374300 -1.16745900
C 1.83812200 -1.36322400 -0.79237000
C 2.31674100 1.59440000 1.24977100
C -2.14182900 3.02267200 -1.11373900
H -2.69390200 -2.79073600 1.11659900
H -3.77395700 0.79340800 -0.92280700
H -0.35771800 -1.93180100 1.37185800
H -0.26573200 2.11692800 -1.08641000
H 0.21207200 2.07312700 1.76353400
H 4.97842200 0.67272000 0.37585800
H 5.22762500 -1.32478300 -1.05634000
H 3.25575600 -2.60022300 -1.80106300
H 0.97279400 -1.92069500 -1.12407500
H 2.88940400 2.36056400 1.74482200
H -3.19732000 2.98941700 -0.87343700
H -1.77418500 3.93612100 -1.56072600

9h:

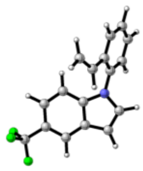

G_sol = –1009.585083
C -1.68414100 0.94270200 -0.73562500
C -2.65149800 0.08747700 -0.18354800
C -2.29212800 -1.07470900 0.48151800
C -0.93899100 -1.38799100 0.60502800
C 0.02497800 -0.50350400 0.05318200
C -0.33552600 0.65670900 -0.62443100
C -0.21750700 -2.47909100 1.19183600
C 1.10807100 -2.23719500 0.98606100
N 1.27380300 -1.03752800 0.30842900
C 2.51979700 -0.50815500 -0.12045900
C 2.96475100 0.73658300 0.35427300
C 4.19508900 1.20722500 -0.12118800
C 4.95311000 0.47488200 -1.01871300
C 4.50100000 -0.76245700 -1.46712100
C 3.28175800 -1.24719600 -1.01996200
C 2.18305900 1.48231100 1.34455000
C 2.24387000 2.78060000 1.55057500
C -4.11110800 0.40252800 -0.32540400
F -4.75009300 -0.49199000 -1.11911500
F -4.75614000 0.37121500 0.86486600
F -4.33389900 1.62015600 -0.85882200
H -2.00042000 1.83746100 -1.25061400
H -3.05027600 -1.72639100 0.89536000
H 0.40894300 1.31752200 -1.04410800
H -0.63331300 -3.32869800 1.70636400
H 1.97503100 -2.80460400 1.27925800
H 4.57421100 2.14825500 0.25342100
H 5.90475700 0.86127700 -1.35919100
H 5.09025200 -1.34166100 -2.16534800
H 2.89766400 -2.19463500 -1.37391100
H 1.49171100 0.89456500 1.93586200
H 2.87870200 3.43178200 0.96333100
H 1.63981000 3.24907400 2.31477800

10h:

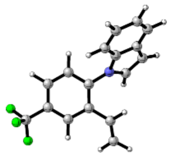

G_sol = –1009.581787
C -1.70341200 -1.29020700 0.71509600

C -2.56677100 -0.35178900 0.15327700
C -2.07342200 0.84637300 -0.33320100
C -0.70996600 1.15106800 -0.26858800
C 0.14649700 0.20342600 0.31621100
C -0.35372600 -1.00516600 0.79838800
C -0.18187500 2.39664000 -0.83890600
C -0.85824600 3.53569800 -0.96902400
N 1.52764700 0.46855800 0.44353200
C 2.08104600 1.55787700 1.10708600
C 3.43891600 1.45982600 1.10673200
C 3.77967800 0.25264500 0.41010000
C 2.56178000 -0.35156900 0.01038300
C 4.98720000 -0.36879100 0.07395400
C 4.95644700 -1.55253700 -0.64112500
C 3.73866200 -2.12771000 -1.03715700
C 2.52677000 -1.53622300 -0.72062400
C -4.02659900 -0.68121500 0.01986100
F -4.26349400 -1.47848400 -1.04807100
F -4.49401900 -1.33973900 1.10105600
F -4.78938400 0.41765600 -0.13899000
H -2.08813000 -2.22508000 1.09768000
H -2.74707400 1.54764200 -0.80311900
H 0.32776300 -1.70702300 1.25799500
H 0.84958500 2.35826600 -1.16904100
H -1.87597900 3.64907400 -0.61640500
H -0.40292800 4.40283900 -1.42737200
H 1.43543600 2.30420700 1.53638900
H 4.12070800 2.16119300 1.55755000
H 5.93048100 0.07422000 0.36723500
H 5.88365200 -2.04354600 -0.90601900
H 3.74563500 -3.04963900 -1.60358200
H 1.59150700 -1.97703900 -1.03675500

9i:

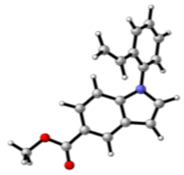

G_sol = –900.331177
C 1.73366700 -0.81491100 -0.54835800
C 2.63730500 0.16598300 -0.08733700
C 2.17493600 1.37444100 0.41980400
C 0.80270200 1.60792500 0.47439500
C -0.08594400 0.59963400 0.01742700
C 0.36923800 -0.61052000 -0.50318000
C -0.00555700 2.71300700 0.90413500
C -1.30561100 2.35968400 0.70250700
N -1.37227100 1.07498000 0.17627100
C 4.10357100 -0.04362900 -0.12740800
O 4.92746700 0.75708000 0.24995300
O 4.44333300 -1.24800300 -0.63961400
C 5.84837900 -1.51525900 -0.70774000
C -2.56839500 0.41315100 -0.20632200
C -2.94493000 -0.79005400 0.41273200
C -4.12851700 -1.39805300 -0.02367900
C -4.90752200 -0.83594600 -1.02068600
C -4.52442200 0.36387900 -1.61205800
C -3.35216100 0.98160700 -1.20592000
C -2.14365000 -1.35250600 1.50558400
C -2.13441100 -2.62756500 1.88781700
H 2.12440100 -1.74104200 -0.94165100
H 2.88998000 2.11149600 0.75846100
H -0.32009800 -1.36521900 -0.85363700
H 0.34043900 3.64547600 1.31689300
H -2.21638100 2.89761900 0.90388500
H 5.93689500 -2.51014000 -1.13590000
H 6.29287500 -1.48517300 0.28664300
H 6.34796700 -0.78086800 -1.33891700
H -4.45636800 -2.30890400 0.45849000
H -5.82235600 -1.32524400 -1.32824600
H -5.13051300 0.81080200 -2.38871900
H -3.02071100 1.90188800 -1.66817500
H -1.49994600 -0.65068800 2.02223600
H -2.71769700 -3.38910600 1.38530900
H -1.52105100 -2.95357900 2.71645500

10i:

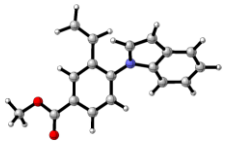

G_sol = –900.328796
C -1.58533000 -1.42982900 0.72649700
C -2.49751700 -0.50559600 0.21329100
C -2.04460700 0.72647700 -0.24217000
C -0.69189000 1.07201100 -0.19595700
C 0.20702200 0.13296000 0.33988600
C -0.24432300 -1.10709800 0.79138500
C -0.21439100 2.34957900 -0.74098500
C 2.11272600 1.53376500 1.11790200
N 1.58158200 0.43844500 0.44663000
C -3.93114300 -0.89332000 0.15980300
O -4.36225100 -1.96029700 0.52598400
O -4.71239100 0.08217500 -0.34418900
C -6.11084100 -0.22545600 -0.42885800
C 2.63327000 -0.34052900 -0.01946600
C 3.83882600 0.29605100 0.36790600
C 5.05917400 -0.27966300 -0.00163100
C 5.05318600 -1.45113500 -0.73725700
C 3.84716900 -2.05848200 -1.12104200
C 2.62309500 -1.51255900 -0.77137700
C 3.47287500 1.47971000 1.09145400
C -0.92548900 3.47223400 -0.81875300

H -1.94786100 -2.38547200 1.07751900
H -2.75014000 1.41677400 -0.67870100
H 0.46938400 -1.80146100 1.21208400
H 0.80802900 2.35196300 -1.10068800
H 1.45197200 2.25136800 1.57219800
H -6.57918400 0.65883200 -0.85158200
H -6.51421500 -0.43862100 0.56028700
H -6.27219100 -1.09015000 -1.07138900
H 5.99304500 0.18890100 0.28202800
H 5.99058700 -1.90703300 -1.02793500
H 3.87291200 -2.96984400 -1.70385000
H 1.69672600 -1.97830800 -1.07740100
H 4.14018700 2.19471500 1.54255000
H -1.93616300 3.54429200 -0.43622000
H -0.50788300 4.36518700 -1.26342600

Supplementary Table 8. Reaction conditions optimization for metathesis involving twofold ArROM and threefold RCM

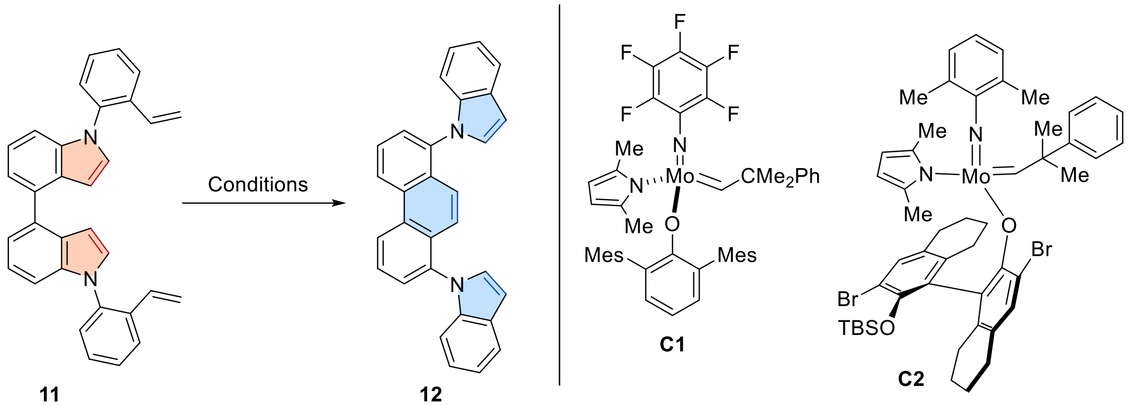

Entry	Catalyst (mol%)	Solvent	Temp. [°C]	Conversion[b] [%]	Yield[b] [%]
1[a]	**C2** (20)	Toluene	65	NR	–
2[a]	**C2** (20)	Toluene	85	9	6
3[a]	**C2** (20)	Toluene	120	32	27
4[a]	**C2** (30)	Toluene	120	34	16
5[a]	**C1** (20)	Toluene	120	50	49
6[c]	**C1** (10)	Toluene	120	77	41[d]

[a]Reactions were performed on 7.50 µmol scale of **11** for 18 h. [b]Conversion and yield were determined by ^{1}H-NMR with durene as an internal standard. [c] on 70.0 µmol scale. [d] Isolated yield.

1,8-Di(1-*H*-indol-1-yl)phenanthrene (12):

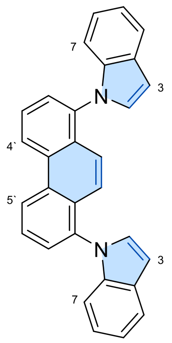

12

The catalyst **C1** (5.83 mg, 7.00 µmol, 10 mol%) was weighed into a 10 mL crimp cap vial. A solution of the **11** (30.6 mg, 70.0 µmol, 1.00 eq.) in toluene (2.3 mL, 0.03 mol·L^{-1}) was then added. The reaction was stirred for 18 h at the indicated temperature, cooled down to room temperature, diluted with EtOAc and filtered through a thin layer of silica gel. Solvents were then removed under reduced pressure. The obtained crude product was purified by silica gel column chromatography (cyclohexane/EtOAc 100:0→80:20) to give the desired product **12** as a beige solid (11.8 mg, 28.9 µmol, 41%): R_f 0.67 (cyclohexane/EtOAc 3:1); ν_{max} (neat): 3054w, 2926w, 2854w, 1674w, 1599m, 1512m, 1464s, 1329m, 1260m, 1082m, 1013w, 883w, 788w, 741s; ^{1}H NMR (500 MHz, $CDCl_3$) δ = 8.84 (2H, d, ^{3}J 8.4 Hz, C4`*H*, C5`*H*), 7.76 (2H, dd, ^{3}J 8.4, ^{3}J 7.5 Hz, C3`*H*, C6`*H*), 7.66 – 7.60 (4H, m, C2`*H*, C7`*H*, C4*H*), 7.28 – 7.22 (2H, m, C2*H*), 7.17 (s, 1H, C9`*H*/C10`*H*), 7.14 (1H, s, C9`*H*/C10`*H*), 7.11 – 6.99 (4H, m, C5*H*, C6*H*), 7.04 – 6.95 (2H, m, C7*H*), 6.70 – 6.64 (2H, m, C3*H*); ^{13}C NMR (126 MHz, $CDCl_3$) δ = 138.24, 138.21 *(C7a)*, 137.0 (*C1*`), 131.63 (*C10a*`/*C8a*`), 131.61 (*C10a*`/*C8a*`), 129.87, 129.84 (*C2*), 129.4 (*C4a*`/*C4b*`), 128.59, 128.56 (*C3a*), 126.98 (*C3*`/*C6*`), 126.97(*C3*`/*C6*`), 126.7 (*C2*`), 123.47, 123.45 (*C4*`/*C5*`), 122.98, 122.96 (*C9*`/*C10*`), 122.5, 122.4 (*C6*), 121.14, 121.10 (*C4*), 120.37, 120.34 (*C5*), 110.8 (*C7*), 103.4 (*C3*); ESI-MS: m/z calcd. for $C_{30}H_{21}N_2$ 409.1699 found 409.1691 [M+H^{+}].

ArROM – Twofold RCM for *N*-Aryl Indoles

Optimization Studies (7.50 µmol scale): Performed accroding to the general procedure **F** utilizing 1,4-bis(2-vinylphenyl)-*1H*-indole **13a** (2.41 mg, 7.50 µmol) and toluene (0.25 mL, 0.03 mol·L^{-1}) with the reaction time of 18 h.

Optimization Studies (70.0 µmol scale): Performed accroding to the general procedure **G** utilizing 1,4-bis(2-vinylphenyl)-*1H*-indole **13a** (22.5 mg, 70.0 µmol) and toluene (2.3 mL, 0.03 mol·L^{-1}) with the reaction time of 18 h.

Supplementary Table 9. Reaction conditions optimization for ArROM – twofold RCM for *N*-aryl indoles

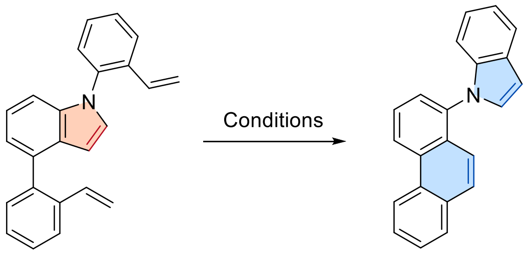

Entry	Catalyst (mol%)	Ligand (mol%)	Solvent	Temp. [°C]	Conversion[b] [%]	Yield[b] [%]
1[a]	**C1** (10)	–	Toluene	65	>95	76
2[a]	**C1** (20)	–	Toluene	65	>95	93
3[a]	**C4** (10)	–	Toluene	65	63	47
4[a]	**C7** (10)	–	Toluene	65	15	9
5[a]	**C3** (10)	–	Toluene	65	53	45
6[a]	**C2** (10)	–	Toluene	**rt**	67	47
7[a]	**C2** (10)	–	Toluene	**65**	>95	95
8[a]	**C2** (10)	–	Toluene	**85**	>95	83
9[a]	**C2** (10)	–	Toluene	**110**	>95	48
10[a]	**Precat. 1** (10)	(R_a)-**L1**	Toluene	65	67	67
11[a]	**Precat. 1** (10)	(S_a)-**L2**	Toluene	65	>95	96
12[a]	**Precat. 1** (10)	(R_a)-**L13**	Toluene	65	NR	–
13[c]	**C1** (10)	–	Toluene	65	42	42[d]
14[c]	**C1** (15)	–	Toluene	65	>95	90[d]
15[c]	**C2** (10)	–	Toluene	65	>95	96[d]

[a]Reactions were performed on 7.50 µmol scale of **13a** for 18 h. [b]Conversion and yield were determined by ^{1}H-NMR with durene as an internal standard. [c]Reactions were performed on 70.0 µmol scale. [d]Isolated yield.

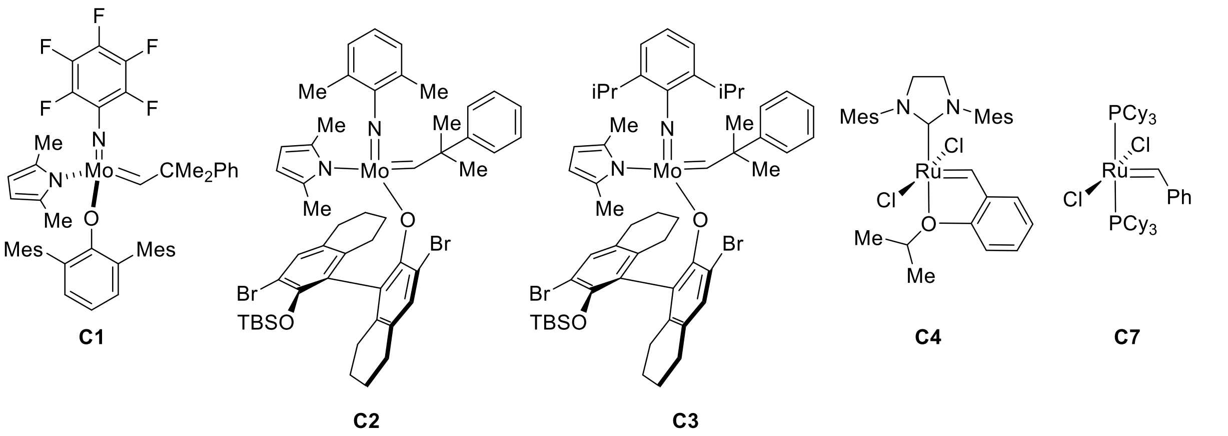

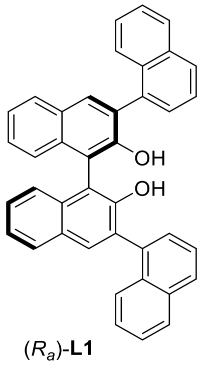

(R_a)-**L1**

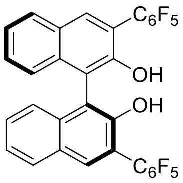

(S_a)-**L2**

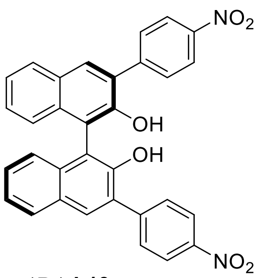

(R_a)-**L13**

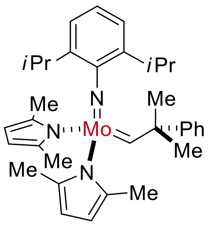

Mo-Precatalyst

General Procedure K for indole ring-opening metathesis:

The catalyst **C2** (7.05 mg, 7.00 µmol, 10 mol%) was weighed into a 10 mL crimp cap vial. A solution of the indole derivative **13a-i** (70.0 µmol, 1.00 eq.) in toluene (2.3 mL, 0.03 mol·L^{-1}) was then added. The reaction was stirred for 18 h at the indicated temperature, cooled down to room temperature, diluted with EtOAc and filtered through a thin layer of silica gel. Solvents were then removed under reduced pressure. The obtained crude product was purified by silica gel column chromatography (cyclohexane/EtOAc 100:0→80:20) to give the desired product.

1-(Phenanthren-1-yl)-1*H*-indole (14a):

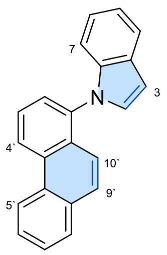

14a

Prepared according to the general procedure **K** using 1,4-bis(2-vinylphenyl)-1*H*-indole **13a** (22.5 mg, 70.0 µmol) to give the title compound **14a** as a white solid (19.8 mg, 67.5 µmol, 96%, m.p.150.5 – 151°C): R$_f$ 0.74 (cyclohexane/EtOAc 8:1); ν$_{max}$ (neat): 3052w, 2923w, 2844w, 1730w, 1599m, 1511m, 1465s, 1328s, 1215m, 1167w, 1078w, 1011w, 907w, 807m, 741s; ^{1}H NMR (500 MHz, CDCl$_3$) δ = 8.83 (1H, d, ^3J 8.5 Hz, C4`*H*), 8.78 (1H, d, $^3J$ 8.3 Hz, C5`*H*), 7.88 (1H, dd, ^3J 7.9, ^4J 1.3 Hz, C8`*H*), 7.80 – 7.74 (2H, m, C3`*H*, C4*H*), 7.72 (1H, ddd, ^3J 8.3, ^3J 7.0, ^4J 1.4 Hz, C6`*H*), 7.68 – 7.61 (3H, m, C2`*H*, C7`*H*, C9`*H*), 7.39 (1H, d, ^3J 3.2 Hz, C2*H*), 7.31 (1H, d, ^3J 9.1 Hz, C10*H*), 7.19 (1H, ddd, ^3J 8.0, ^3J 7.0, ^4J 1.1 Hz, C5*H*), 7.13 (1H, ddd, ^3J 8.2, ^3J 7.0, ^4J 1.3 Hz, C6*H*), 7.02 (1H, dt, ^3J 8.2, ^4J 1.1 Hz, C7*H*), 6.80 (1H, dd, ^3J 3.2, ^4J 0.9 Hz, C3*H*); ^{13}C NMR (126 MHz, CDCl$_3$) δ = 138.2 (*C7a*), 136.6 (*C1`*), 132.0 (*C8a`*), 131.7 (*C4a`*), 130.1 (*C4b`*), 129.9 (*C2*), 129.3 (*C10a`*), 128.7 (*C8`*), 128.5 (*C3a*), 128.2 (*C9`*), 127.2 (*C7`*), 127.1 (*C6`*), 126.3 (*C3`*), 126.0 (*C2`*), 123.04 (*C4`*), 122.98 (*C5`*), 122.2 (*C6*), 121.6 (*C10`*), 120.9 (*C4*), 120.2 (*C5*), 110.9 (*C7*), 103.0 (*C3*); ESI-MS: m/z calcd. for $C_{22}H_{15}NAg$ 400.0250 found 400.0248 [M+Ag$^+$].

Scale-up (C2 (5 mol%)): Performed according to the modified general procedure **K** using 1,4-bis(2-vinylphenyl)-1*H*-indole **13a** (112 mg, 350 µmol) and **C2** (17.6 mg, 17.5 µmol, 5 mol%) with the reaction time of 18 h to give the title compound **14a** as a white solid (101 mg, 344 µmol, 98%).

Scale-up (C2 (2 mol%)): Performed according to the modified general procedure **K** using 1,4-bis(2-vinylphenyl)-1*H*-indole **13a** (112 mg, 350 µmol) and **C2** (7.05 mg, 7.00 µmol, 2 mol%) with the reaction time of 18 h to give the title compound **14a** as a white solid (99.5 mg, 339 µmol, 97%).

Reaction monitoring versus time:

The reaction of **13a** on 350 µmol scale with 2 mol% loading of **C2** performed according to the procedure described above was monitored versus time over 18 hours. The reaction was placed in the glovebox and aliquots were taken after the indicated time. Conversion was determined via ^{1}H-NMR analysis.

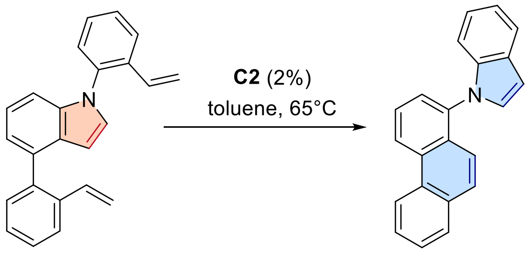

13a (112 mg)
(0.35 mmol)

14a

Supplementary Table 10. Reaction progress

Reaction time min	Conversion %
0	0
10	2
70	16
100	24
120	30
165	40
250	63
295	69
320	74
340	76
380	80
435	85
500	89
540	92
1080	100

Reaction conditions: **13a** (112 mg, 350 µmol), **C2** (7.05 mg, 7.00 µmol, 2 mol%), toluene, Ar.

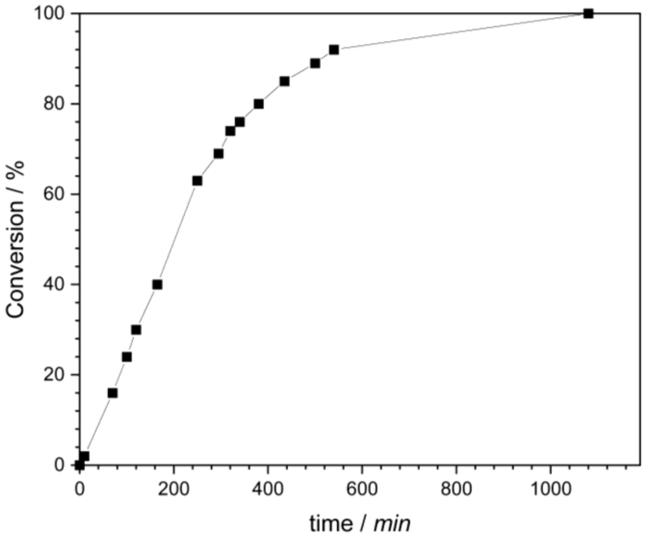

1-(6-Chlorophenanthren-1-yl)-1*H*-indole (14b):

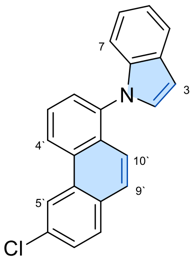

14b

Prepared according to the general procedure **K** using 4-(5-chloro-2-vinylphenyl)-1-(2-vinylphenyl)-1*H*-indole **13b** (24.9 mg, 70.0 µmol) to give the title compound as a beige solid (22.0 mg, 67.1 µmol, 96%): R_f 0.74 (cyclohexane/EtOAc 6:1); ν_{max} (neat): 3060w, 2930w, 2855w, 1793w, 1593w, 1513w, 1463m, 1401w, 1327w, 1212w, 1149w, 1094w, 1012w, 978w, 840m, 747m; ^{1}H NMR (500 MHz, $CDCl_3$) δ = 8.80 – 8.66 (2H, m, C4`*H*, C5`*H*), 7.82 (1H, d, ^{3}J 8.5 Hz, C8`*H*), 7.80 – 7.74 (2H, m, C4*H*, C3`*H*), 7.71 – 7.66 (1H, m, C2`*H*), 7.64 – 7.57 (2H, m, C7`*H*, C9`*H*), 7.37 (1H, d, $^{3}J$ 3.2 Hz, C2*H*), 7.31 (1H, d, $^{3}J$ 9.1 Hz, C10`*H*), 7.24 – 7.17 (1H, m, C5*H*), 7.14 (1H, td, ^{3}J 7.6, ^{4}J 1.1 Hz, C6*H*), 7.01 (1H, d, ^{3}J 8.2 Hz, C7*H*), 6.80 (1H, dd, ^{3}J 3.2, ^{4}J 0.7 Hz, C3*H*); ^{13}C NMR (126 MHz, $CDCl_3$) δ = 138.1 (*C7a*), 136.7 (*C1*`), 133.1 (*C8a*`), 131.2 (*C4b*`), 130.7 (*C4a*`), 130.3 (*C6*`), 130.1 (*C8*`),

129.8 (*C2*), 129.6 (*C10a`*), 128.5 (*C3a*), 127.7 (*C7`*), 127.5 (*C9`*), 126.65 (*C2`*), 126.63 (*C3`*), 123.1 (*C4`*), 122.8 (*C5`*), 122.3 (*C6*), 122.0 (*C10`*), 121.0 (*C4*), 120.3 (*C5*), 110.8 (*C7*), 103.2 (*C3*); ESI-MS: m/z calcd. for $C_{22}H_{14}ClNNa$ 350.0707 found 350.0701 [M+Na$^+$].

1-(6-Methoxyphenanthren-1-yl)-1*H*-indole (14c):

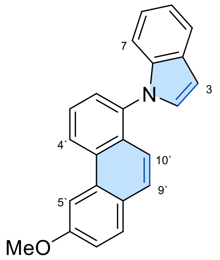

14c

Prepared according to the modified general procedure **K** using 4-(5-methoxy-2-vinylphenyl)-1-(2-vinylphenyl)-1*H*-indole **13c** (24.6 mg, 70.0 µmol) and the catalyst **C2** (14.1 mg, 14.0 µmol, 20 mol%) to give the title compound **14c** as a beige solid (22.0 mg, 68.0 µmol, 97%, m.p.165.0 – 166.7°C): R$_f$ 0.50 (cyclohexane/EtOAc 6:1); ν_{max} (neat): 3054w, 2934w, 2835w, 1609m, 1602m, 1512m, 1469s, 1399w, 1329w, 1291w, 1234s, 1145w, 1036w, 908w, 837w, 736m; ^{1}H NMR (500 MHz, $CDCl_3$) δ = 8.74 (1H, d, ^3J 8.5 Hz, C4`*H*), 8.12 (1H, d, $^4J$ 2.4 Hz, C5`*H*), 7.79 (1H, d, ^3J 8.7 Hz, C8`*H*), 7.78 – 7.71 (2H, m, C4*H*, C3`*H*), 7.64 (1H, dd, ^3J 7.4, ^4J 1.0 Hz, C2`*H*), 7.59 (1H, d, $^3J$ 9.1 Hz, C9`*H*), 7.38 (1H, d, ^3J 3.2 Hz, C2*H*), 7.29 (1H, dd, ^3J 8.7, ^4J 2.4 Hz, C7`*H*), 7.21 – 7.15 (2H, m, C5*H*, C10`*H*), 7.13 (1H, td, ^3J 7.6, ^4J 1.2 Hz, C6*H*), 7.04 – 6.99 (1H, m, C7*H*), 6.79 (1H, dd, *J* ^3J 3.2, ^4J 0.8 Hz, C3*H*), 4.05 (3H, OC*H*$_3$); ^{13}C NMR (126 MHz, $CDCl_3$) δ = 159.0 (*C6`*), 138.3 (*C7a*), 136.7 (*C1`*), 131.6 (*C4b`*), 131.2 (*C4a`*), 130.3 (*C8`*), 130.0 (*C2*), 129.9 (*C10a`*), 128.6 (*C3a*), 127.9 (*C9`*), 126.8 (*C8a`*), 126.2 (*C2`*), 125.9 (*C3`*), 123.2 (*C4`*), 122.3 (*C6*), 121.1 (*C4*), 120.3 (*C5*), 119.3 (*C10`*), 117.4 (*C7`*), 111.0 (*C7*), 104.5 (*C5`*), 103.1 (*C3*), 55.7 (OCH_3); ESI-MS: m/z calcd. for $C_{23}H_{17}NOAg$ 430.0356 found 430.0349 [M+Ag$^+$].

1-(3-Chlorophenanthren-1-yl)-1*H*-indole (14d):

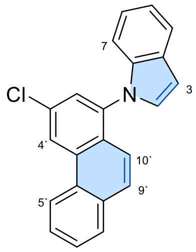

14d

Prepared according to the general procedure **K** using 6-chloro-1,4-bis(2-vinylphenyl)-1*H*-indole **13d** (24.9 mg, 70.0 µmol) to give the title compound **14d** as a beige oil (22.5 mg, 68.6 µmol, 98%): R$_f$ 0.76 (cyclohexane/EtOAc 5:1); ν_{max} (neat): 3055w, 2926w, 2855w, 1719w, 1684w, 1593m, 1515m, 1459s, 1326m, 1214m, 1134w, 1083w, 1034w, 966w, 906m, 823m, 639w; ^{1}H NMR (500 MHz, $CDCl_3$) δ = 8.78 (1H, d, ^4J 1.9 Hz, C4`*H*), 8.68 (1H, d, $^3J$ 8.3 Hz, C5`*H*), 7.89 (1H, dd, ^3J 7.8, ^4J 1.4 Hz, C8*H*), 7.81 – 7.70 (2H, m, C4*H*, C6`*H*), 7.70 – 7.60 (3H, m, C7`*H*, C9`*H*, C2`*H*), 7.36 (1H, d, ^3J 3.2 Hz, C2*H*), 7.31 – 7.28 (1H, m, C10`*H*), 7.20 (1H, ddd, $^3J$ 7.9, $^3J$ 7.0, $^4J$ 1.2 Hz, C5*H*), 7.16 (ddd, $^3J$ 8.2, $^3J$ 7.0, $^4J$ 1.3 Hz, C6*H*), 7.07 – 7.01 (1H, m, C7*H*), 6.81 (1H, dd, $^3J$ 3.2, $^4J$ 0.8 Hz, C3*H*); $^{13}$C NMR (126 MHz, $CDCl_3$) δ = 138.1 (*C7a*), 137.9 (*C1`*), 132.7 (*C4a`*), 132.5 (*C8a`*), 132.2 (*C3*), 129.7 (*C2*), 129.3 (*C4b`*), 128.9 (*C8*), 128.7 (*C3a*), 128.5 (*C9`*), 128.0 (*C7`*), 127.8 (*C10a`*), 127.6 (*C6`*), 126.6 (*C2`*), 123.2 (*C5`*), 122.8 (*C4`*), 122.7 (*C6*), 121.4 (*C10`*) 121.2 (*C4*), 120.6 (*C5*), 110.9 (*C7*), 103.8 (*C3*); ESI-MS: m/z calcd. for $C_{22}H_{15}ClN$ 328.0888 found 328.0883 [M+H$^+$].

1-(3-(Trifluoromethyl)phenanthren-1-yl)-1*H*-indole (14e):

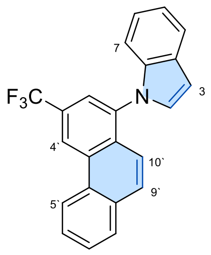

14e

Prepared according to the general procedure **K** using 6-(trifluoromethyl)-1,4-bis(2-vinylphenyl)-1*H*-indole **13e** (27.3 mg, 70.0 µmol) to give the title compound **14e** as a beige oil (25.0 mg, 69.2 µmol, 99%): R_f 0.73 (cyclohexane/EtOAc 6:1); ν_{max} (neat): 3059w, 2932w, 862w, 1607w, 1516m, 1461s, 1383w, 1334m, 1287s, 1235m, 1164s, 1125s, 990w, 888w, 827m, 744s, 668m, 618m; ^{1}H NMR (500 MHz, $CDCl_3$) δ = 9.09 (1H, s, C4`*H*), 8.80 (1H, d, $^3J$ 8.1 Hz, C5`*H*), 7.93 (1H, dd, ^3J 7.9, ^4J 1.2 Hz, C8`*H*), 7.87 (1H, d, $^3J$ 1.4 Hz, C2`*H*), 7.84 – 7.76 (3H, m, C4*H*, C6`*H*, C9`*H*), 7.72 (1H, ddd, ^3J 7.9, ^3J 7.1, ^4J 1.1 Hz, C7`*H*), 7.44 – 7.35 (2H, m, C2*H*, C10`*H*), 7.22 (1H, ddd, ^3J 8.0, ^3J 7.0, ^4J 1.1 Hz, C5*H*), 7.16 (1H, ddd, ^3J 8.2, ^3J 7.0, ^4J 1.2 Hz, C6*H*), 7.00 (1H, dd, ^3J 8.2, ^4J 0.9 Hz, C7*H*), 6.83 (1H, dd, ^3J 3.2, ^4J 0.8 Hz, C3*H*); ^{13}C NMR (126 MHz, $CDCl_3$) δ= 138.2 (*C7a*), 137.7 (*C1`*), 132.3 (*C8a`*), 131.6 (*C4a`*), 131.3 (*C10a`*), 130.6 (*C6`*), 130.1 (*C4b`*), 129.7 (*C2*), 129.1 (*C8`*), 128.8 (*C3a*), 128.2 (*C7`*), 128.0 (*C9`*), 123.2 (*C5`*), 122.8 (*C6*), 122.1 (q, $^3J_{CF}$ 3.1 Hz, *C2`*), 121.31 (*C4*), 121.27 (*C10`*), 120.7 (*C5*), 120.41 (q, J_{CF} 3.7 Hz, *C4`*), 110.8 (*C7*), 104.0 (*C3*); ^{19}F NMR (376 MHz, $CDCl_3$) δ= –61.8; ESI-MS: m/z calcd. for $C_{23}H_{14}F_3NAg$ 468.0124 found 468.0116 [M+Ag$^+$].

1-(2-Fluorophenanthren-1-yl)-1*H*-indole (14f):

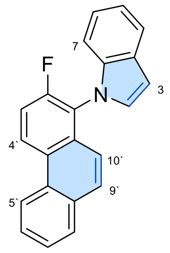

14f

Prepared according to the general procedure **K** using 7-fluoro-1,4-bis(2-vinylphenyl)-1*H*-indole **13f** (23.8 mg, 70.0 µmol) to give the title compound **14f** as a white solid (21.6 mg, 69.4 µmol, 99%, m.p. 119.5 – 120.1°C): R_f 0.55 (cyclohexane/EtOAc 5:1); ν_{max} (neat): 3054w, 2927w, 2854w, 1731w, 1615m, 1515m, 1480s, 1299m, 1259m, 1139m, 1090w, 1012w, 908w, 816s, 741s; ^{1}H NMR (500 MHz, $CDCl_3$) δ = 8.83 (1H, dd, ^3J 9.2, $^4J_{HF}$ 4.9 Hz, C4`*H*), 8.70 (1H, d, $^3J$ 8.4 Hz, C5`*H*), 7.88 (1H, d, ^3J 7.7 Hz, C8`*H*), 7.77 (1H, d, $^3J$ 7.8 Hz, C4*H*), 7.75 – 7.71 (1H, m, C6`*H*), 7.69 (1H, d, ^3J 9.2 Hz, C9`*H*), 7.67 – 7.57 (2H, m, C7`*H*, C3`*H*), 7.32 (1H, d, $^3J$ 3.2 Hz, C2*H*), 7.23 – 7.19 (2H, m, C5*H*, C10`*H*), 7.18 – 7.11 (1H, m, C6*H*), 6.93 (1H, d, ^3J 8.1 Hz, C7*H*), 6.85 (1H, d, ^3J 3.2 Hz, C3*H*); ^{13}C NMR (126 MHz, $CDCl_3$) δ = 157.3 (d, $^1J_{CF}$ 251.8 Hz, *C2`*), 138.0 (*C7a*), 131.6 (*C8a`*), 131.5 (*C10a`*), 130.0 (*C4b`*), 129.8 (*C2*), 129.6 (*C9`*), 129.0 (*C8`*), 128.6 (*C3a*), 127.8 (d, $^4J_{CF}$ 2.2 Hz, *C4a`*), 127.6 (*C6`*), 127.2 (*C7`*), 125.0 (d, $^3J_{CF}$ 8.8 Hz, *C4`*), 122.8 (*C5`*), 122.6 (*C6*), 122.2 (d, $^2J_{CF}$ 12.7 Hz, *C1`*), 121.3 (d, $^4J_{CF}$ 4.8 Hz, *C10`*), 121.2 (*C4*), 120.5 (*C5*), 115.8 (d, $^2J_{CF}$ 22.5 Hz, *C3`*), 110.7 (*C7*), 103.9 (*C3*); ^{19}F NMR (376 MHz, $CDCl_3$) δ = –121.43; ESI-MS: m/z calcd. for $C_{22}H_{14}AgFN$ 418.0156 found 418.0151 [M+Ag$^+$].

1-(10-Methylphenanthren-1-yl)-1*H*-indole (14g):

Prepared according to the modified general procedure **K** using 3-methyl-1,4-bis(2-vinylphenyl)-1*H*-indole **13g** (23.5 mg, 70.0 µmol) and **C2** (14.1 mg, 14.0 µmol, 20 mol%) give the title compound **14g** as a white solid (11.2 mg, 36.4 µmol, 52%, m.p. 119.1 – 120.0°C) after preparative thin layer chromatography (pTLC, cyclohexane/EtOAc

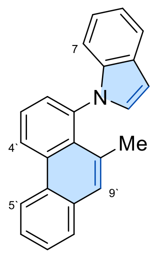

14g

8:1): R_f 0.69 (cyclohexane/EtOAc 8:1); ν_{max} (neat): 3057w, 2967w, 2932w, 1597w, 1509m, 1464s, 1369w, 1329m, 1214m, 1147w, 1094w, 1049w, 1031w, 887w, 821w, 743s; ^{1}H NMR (500 MHz, $CDCl_3$) δ = 8.92 (1H, dd, ^3J 8.5, ^4J 1.0 Hz, C4`*H*), 8.72 (1H, d, $^3J$ 8.2 Hz, C5`*H*), 7.83 – 7.77 (1H, m, C8`*H*), 7.76 – 7.68 (2H, m, C4*H*, C3`*H*), 7.68 – 7.58 (2H, m, C6`*H*, C7`*H*), 7.53 – 7.47 (2H, m, C2`*H*, C9`*H*), 7.26 (1H, d, ^3J 3.1 Hz, C2*H*), 7.19 – 7.08 (2H, m, C5*H*, C6*H*), 6.88 (1H, d, ^3J 8.2 Hz, C7*H*), 6.74 (1H, dd, ^3J 3.1, ^4J 0.8 Hz, C3*H*), 1.84 (3H, d, ^4J 0.9 Hz, CH_3); ^{13}C NMR (126 MHz, $CDCl_3$) δ = 139.6 (*C7a*), 136.5 (*C1`*), 133.0 (*C4a`*), 132.0 (*C8a`*), 131.7 (*C10`*), 130.8 (*C9`*), 130.5 (*C2*), 130.2 (*C10a`*), 129.6 (*C4b`*), 129.3 (*C2`*), 128.3 (*C3a*), 127.8 (*C8`*), 127.4 (*C7`*), 126.6 (*C6`*), 126.0 (*C3`*), 124.3 (*C4`*), 123.0 (*C5`*), 122.6 (*C6*), 121.0 (*C4*), 120.1 (*C5*), 110.8 (*C7*), 102.9 (*C3*), 20.7 (*C*H_3); ESI-MS: m/z calcd. for $C_{23}H_{17}AgN$ 414.0406 found 414.0412 [M+Ag$^+$].

4-(1*H*-Indol-1-yl)benzo[h]isoquinoline (14h):

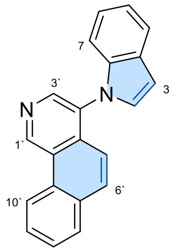

14h

Prepared according to the modified general procedure **K** 1,4-bis(2-vinylphenyl)-1*H*-pyrrolo[2,3-c]pyridine **13h** (22.6 mg, 70.0 µmol) and **C2** (14.1 mg, 14.0 µmol, 20 mol%) give the title compound **14h** as a white solid (9.50 mg, 32.3 µmol, 46%, m.p. 119.1 – 120.0°C): R_f 0.64 (cyclohexane/EtOAc 3:2); ν_{max} (neat): 3123w, 3051w, 2930w, 1706w, 1595w, 1512m, 1334m, 1081w, 1005w, 968w, 906w, ^{1}H NMR (500 MHz, $CDCl_3$) δ = 10.17 (1H, s, C3`*H*), 8.91 (1H, d, $^3J$ 8.1 Hz, C10`*H*), 8.81 (1H, s, C1`*H*), 7.95 (1H, d, $^3J$ 7.9 Hz, C7`*H*), 7.90 (1H, d, ^3J 9.1 Hz, C6`*H*), 7.83 (1H, ddd, $^3J$ 8.1, $^3J$ 7.1, $^4J$ 1.4 Hz, C9`*H*), 7.78 (1H, dt, ^3J 7.9, ^4J 1.2 Hz, C4*H*), 7.73 (1H, ddd, ^3J 7.9, ^3J 7.1, ^4J 1.1 Hz, C8`*H*), 7.38 (1H, d, $^3J$ 3.2 Hz, C2*H*), 7.36 (1H, d, $^3J$ 9.1 Hz, C5`*H*), 7.22 (1H, ddd, ^3J 7.9, ^3J 7.1, ^4J 1.1 Hz, C5*H*), 7.20 – 7.14 (1H, m, C6*H*), 7.04 (1H, dd, ^3J 8.1, ^5J 0.8 Hz, C7*H*), 6.84 (1H, dd, ^3J 3.2, ^5J 0.8 Hz, C3*H*); ^{13}C NMR (126 MHz, $CDCl_3$) δ = 146.8 (*C3`*), 144.0 (*C1`*), 138.3 (*C7a*), 133.4 (*C4a`*), 133.0 (*C6`*), 132.3 (*C6a`*), 129.7 (*C2*), 129.3 (*C7`/C10a`*), 128.9 (*C3a*), 128.6 (*C9`*), 128.2 (*C8`*), 126.0 (*C10b`*), 122.8 (*C6*), 122.6 (*C10`*), 121.3 (*C4*), 120.7 (*C5*), 120.1 (*C5`*), 110.7 (*C7*), 104.1 (*C3*); ESI-MS: m/z calcd. for $C_{21}H_{15}N_2$ 295.1230 found 295.1235 [M+H$^+$].

1-(Benzo[c]phenanthren-4-yl)-1*H*-indole (14i):

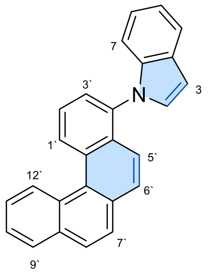

14i

Prepared according to the general procedure **K** using 4-(2-vinylnaphthalen-1-yl)-1-(2-vinylphenyl)-1*H*-indole **13i** (26.0 mg, 70.0 µmol) to give the title compound as a white solid (23.5 mg, 68.4 µmol, 98%, m.p. 166.2 – 167.0°C): R_f 0.68 (cyclohexane/EtOAc 5:1); ν_{max} (neat): 3053w, 2927w, 2854w, 1735w, 1602m, 1566w, 1463s, 1328m, 1237m, 1142w, 1036w, 837s; ^{1}H NMR (500 MHz, $CDCl_3$) δ = 9.33 – 9.21 (1H, m, C1`*H*), 9.14 (1H, d, $^3J$ 8.4 Hz, C12`*H*), 8.06 (1H, dd, ^3J 8.0, ^4J 1.5 Hz, C9`*H*), 7.95 (1H, d, $^3J$ 8.5 Hz, C8`*H*), 7.84 – 7.62 (7H, m, C2`*H*, C3`*H*, C7`*H*, C10`*H*, C11`*H*, C4*H*, C6`*H*), 7.46 (1H, d, ^3J 8.8, C5`*H*), 7.44 (1H, d, ^3J 3.2 Hz, C2*H*), 7.20 (1H, ddd, ^3J 8.0, ^3J 6.9, ^4J 1.2 Hz, C5*H*), 7.15 (1H, ddd, ^3J 8.0, ^3J 6.9, ^4J 1.2 Hz,

C6*H*), 7.12 – 7.06 (1H, m, C7*H*), 6.83 (1H, dd, ^3J 3.2, ^5J 0.8 Hz, C3*H*); ^{13}C NMR (126 MHz, $CDCl_3$) δ = 138.2 (*C7a*), 136.5 (*C4`*), 133.8 (*C12`b*), 131.8 (*C12c`*), 131.3 (*C6a`*), 130.8 (*C4a`*), 130.3 (*C12a`*), 130.2 (*C2*), 128.8 (*C9`*), 128.7 (*C3a*), 128.5 (*C1`*), 128.3 (*C8`*), 128.2 (*C12`*/*C6`*), 127.6 (*C8a`*), 126.8 (*C4*), 126.6 (*C11`*), 126.3 (*C10`*), 125.8 (*C2`*), 125.0 (*C3`*), 122.5 (*C6*), 122.4 (*C5`*), 121.1 (*C7`*), 120.4 (*C5*), 111.1 (*C7*), 103.3 (*C3*); ESI-MS: m/z calcd. for $C_{26}H_{18}N$ 344.1434 found 344.1427 [M+H$^+$].

Atroposelective Aromatic Ring-Opening Metathesis – RCM Cascade

Optimization Studies (15.0 µmol scale) of 15a: Performed according to the general procedure **H** utilizing 5,7-dimethoxy-4-methyl-1-(2-vinylphenyl)phenanthrene **15a** (5.32 mg, 15.0 µmol) and the corresponding solvent (0.50 mL, 0.03 mol·L^{-1}) with the reaction time of 18 h.

Supplementary Table 11. Reaction conditions optimization for atroposelective aromatic ring-opening metathesis – RCM cascade

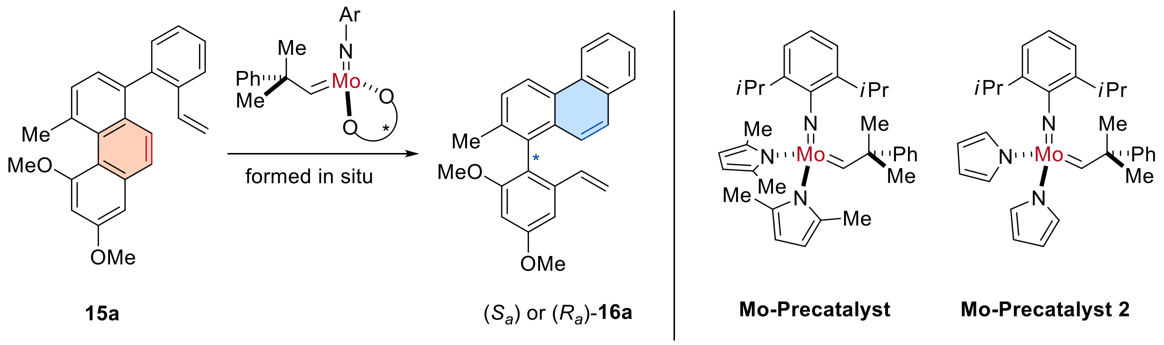

Entry[a]	Catalyst (mol%)	Ligand (mol%)	Solvent	Temp. [°C]	Conversion[b] [%]	Yield[b] [%]	e. r.[c]
1	**C1** (10)	–	Toluene	65	>95	–	50 : 50
2	**Mo-Precat.** (20)	(R_a)-**L4** (60)	Toluene	65	>95	48	14 : 86
3	**Mo-Precat.** (10)	(R_a)-**L4** (30)	Toluene	50	NR	0	–
4	**Mo-Precat.** (20)	(R_a)-**L4** (60)	Toluene	50	>95	75	13 : 87
5	**Mo-Precat.** (30)	(R_a)-**L4** (90)	Toluene	50	>95	60	15 : 85
6	**Mo-Precat.** (10)	(S_a)-**L3** (30)	Toluene	65	NR	–	–
7	**Mo-Precat. 2** (10)	(S_a)-**L3** (30)	Toluene	65	NR	–	–
8	**Mo-Precat.** (20)	(S_a)-**L3** (60)	Toluene	65	75	57	97 : 3
9	**Mo-Precat. 2** (20)	(S_a)-**L3** (60)	Toluene	65	NR	–	–
10	**Mo-Precat.** (20)	(S_a)-**L3** (60)	Toluene	50	NR	–	–
11	**Mo-Precat.** (30)	(S_a)-**L3** (90)	Toluene	65	>95	54	95 : 5
12	**Mo-Precat.** (10)	(S_a)-**L5** (30)	Toluene	65	NR	–	–
13	**Mo-Precat.** (10)	(R_a)-**L6** (30)	Toluene	65	>95	51	4 : 96
14	**Mo-Precat.** (10)	(R_a)-**L7** (30)	Toluene	65	>95	67	19 : 81
15	**Mo-Precat.** (10)	(R_a)-**L8** (30)	Toluene	65	>95	61	10 : 90
16	**Mo-Precat.** (10)	(R_a)-**L2** (30)	Toluene	65	>95	66	33 : 67
17	**Mo-Precat.** (10)	(R_a)-**L9** (30)	Toluene	65	>95	68	15 : 85
18	**Mo-Precat.** (10)	(R_a)-**L1** (30)	Toluene	65	>95	80	6 : 94
19	**Mo-Precat.** (10)	(S_a)-**L1** (30)	Toluene	65	>95	76	95 : 5
20	**Mo-Precat.** (10)	(R_a)-**L1** (30)	Toluene	50	16	14	–
21	**Mo-Precat.** (20)	(R_a)-**L1** (60)	Toluene	50	>95	88	6 : 94
22	**Mo-Precat.** (10)	(R_a)-**L1** (30)	C_6F_6	65	13	4	–
23	**Mo-Precat.** (10)	(R_a)-**L1** (30)	CF_3-C_6H_5	65	NR	–	–
24	**Mo-Precat.** (10)	(R_a)-**L1** (30)	DCE	65	>95	70	2 : 98
25	**C5** (20)	–	Toluene	65	4	3	–

[a]Reactions were performed on 15.0 µmol scale of **15a** for 18 h. [b]Conversion and yield were determined by ^{1}H-NMR with durene as an internal standard. [c]Determined by HPLC on a chiral stationary phase of the crude product (Chiralpak IC-N3 column (3 µm, 250x4.6 mm, heptane/*i*PrOH 97.5:2.5, 1.0 mL/min, 20°C).

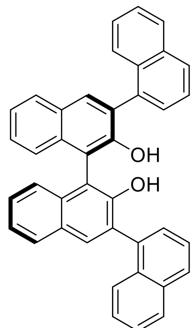

(R_a)-**L1**

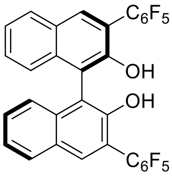

(R_a)-**L2**

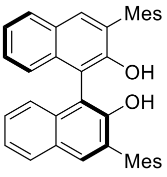

(S_a)-**L3**

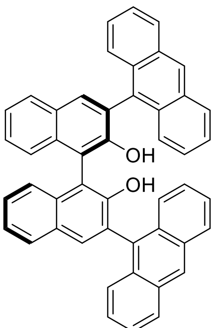

(R_a)-**L4**

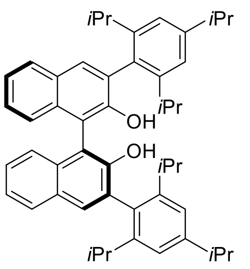

(S_a)-**L5**

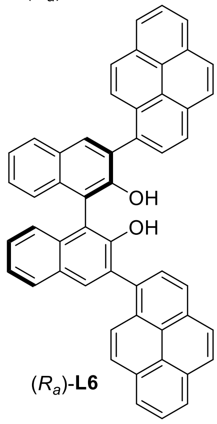

(R_a)-**L6**

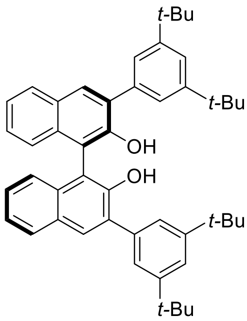

(R_a)-**L7**

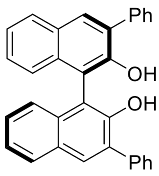

(R_a)-**L8**

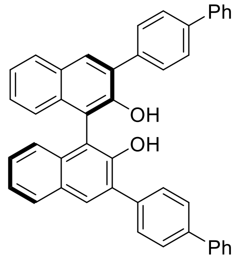

(R_a)-**L9**

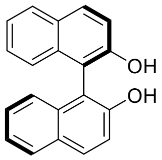

(R_a)-**L10**

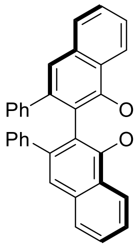

(S_a)-**L11**

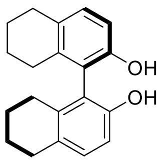

(R_a)-**L12**

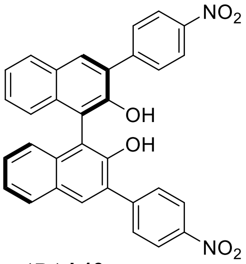

(R_a)-**L13**

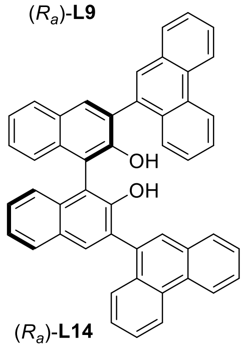

(R_a)-**L14**

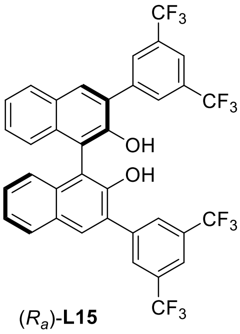

(R_a)-**L15**

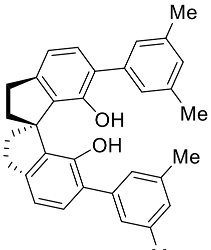

(R)-**L16**

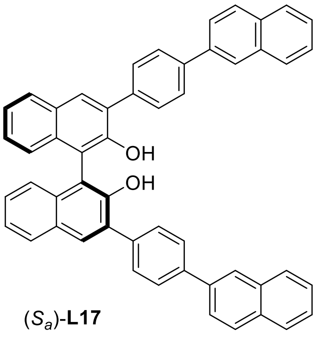

(S_a)-**L17**

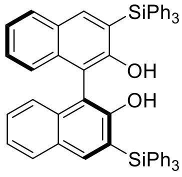

(S_a)-**L18**

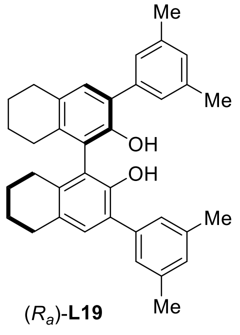

(R_a)-**L19**

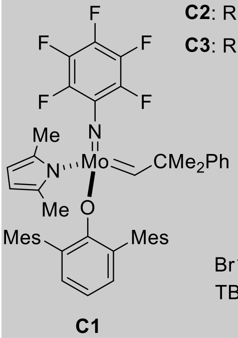

C1

C2: R = Me
C3: R = *i*Pr

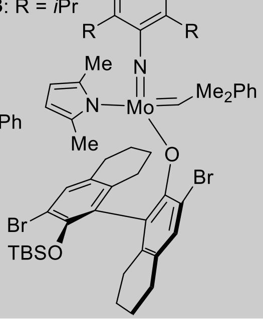

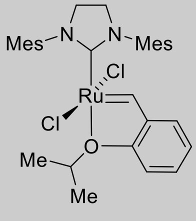

C4

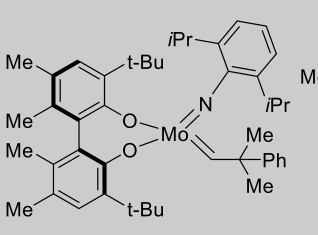

C5

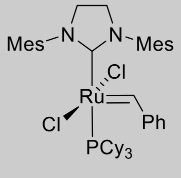

C6

Optimization Studies (15.0 µmol scale) of 15b: Performed according to the general procedure **H** utilizing 9,11-dimethyl-5-(2-vinylphenyl)phenanthro[3,4-d][1,3]dioxole **15b** (5.29 mg, 15.0 µmol) with the reaction time of 18 h.

Supplementary Table 12. Reaction conditions optimization for atroposelective aromatic ring-opening metathesis – RCM cascade

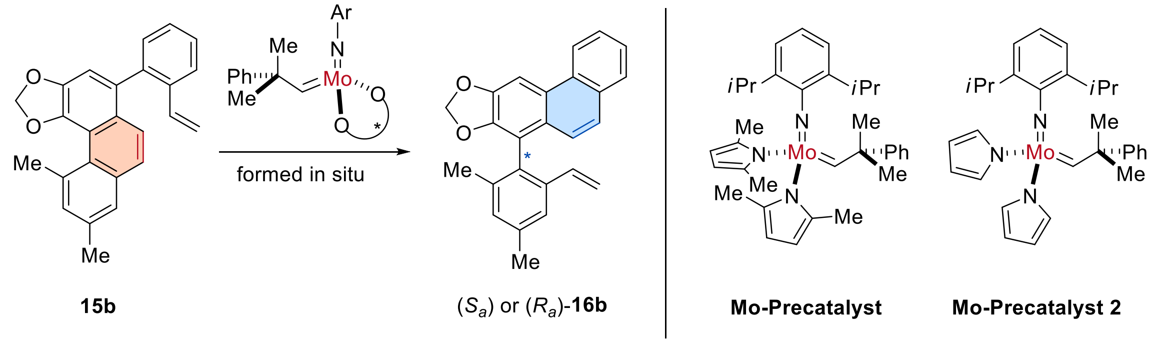

15b (*S*$_a$) or (*R*$_a$)-**16b** **Mo-Precatalyst** **Mo-Precatalyst 2**

Entry[a]	Catalyst (mol%)	Ligand (mol%)	Conc. mol·L^{-1}	Solvent	Temp. [°C]	Conversion[b] [%]	Yield[b] [%]	e. r.[c]
1	**C6** (10)	–	0.03	Toluene	Rt	9	–	50 :50
2	**C6** (10)	–	0.03	Toluene	65	35	–	50 :50
3	**C4** (10)	–	0.03	Toluene	Rt	5	–	50 :50
4	**C4** (10)	–	0.03	Toluene	65	56	–	50 :50
5	**C4** (10)	–	0.03	Toluene	80	92	50	50 :50
6	**C1** (10)	–	0.03	Toluene	40	>95	84	50 :50
7	**C1** (10)	–	0.03	Toluene	65	>95	84	50 :50
8	**C5** (10)	–	0.03	Toluene	40	NR	–	–
9	**C5** (10)	–	0.03	Toluene	65	5	4	51 : 49
10	**C5** (30)	–	0.03	Toluene	65	31	17	51 : 49
11	**C5** (30)	–	0.03	Toluene	80	47	22	52 : 48
12	**C2** (10)	–	0.03	Toluene	65	>95	67	41 : 59
13	**Mo-Precat.** (10)	(*R*$_a$)-**L10** (10)	0.03	Toluene	65	NR	–	–
14	**Mo-Precat.** (10)	(*S*$_a$)-**L11** (10)	0.03	Toluene	65	NR	–	—
15	**Mo-Precat.** (10)	(*S*$_a$)-**L12** (10)	0.03	Toluene	65	NR	–	–
16	**Mo-Precat.** (10)	(*R*$_a$)-**L10** (10)	0.03	Toluene	65	>95	86	41 : 59
17	**Mo-Precat.** (10)	(*R*$_a$)-**L10** (30)	0.03	Toluene	65	>95	89	44 : 56
18	**Mo-Precat.** (10)	(*R*$_a$)-**L10** (30)	0.03	Toluene	40	>95	88	40 : 60
19	**Mo-Precat.** (10)	(*R*$_a$)-**L4** (30)	0.03	Toluene	65	>95	89	22 : 78
20	**Mo-Precat.** (10)	(*R*$_a$)-**L4** (30)	0.03	Toluene	40	91	88	30 : 70
21	**Mo-Precat.** (10)	(*R*$_a$)-**L4** (30)	0.03	Toluene	Rt	47	45	20 : 80
22	**Mo-Precat.** (10)	(*R*$_a$)-**L13** (30)	0.03	Toluene	65	0	–	–
23	**Mo-Precat.** (10)	(*S*$_a$)-**L3** (30)	0.03	Toluene	65	25	24	87 : 13
24	**Mo-Precat.** (10)	(*S*$_a$)-**L3** (30)	0.03	Toluene	80	18	18	87 : 13
25	**Mo-Precat.** (20)	(*S*$_a$)-**L3** (60)	0.03	Toluene	65	>95	74	87 : 13
26	**Mo-Precat.** (20)	(*S*$_a$)-**L3** (60)	**0.06**	Toluene	65	>95	67	87 : 13
27	**Mo-Precat.** (20)	(*S*$_a$)-**L3** (60)	**0.015**	Toluene	65	>95	64	84 :16
28	**Mo-Precat.** (20)	(*S*$_a$)-**L3** (60)	0.03	Toluene:CH_2Cl_2 (3 : 2)	50	>95	85	84 : 16
29[d]	**Mo-Precat.** (20)	(*S*$_a$)-**L3** (60)	0.03	Toluene	50	>95	77	86 : 14
30	**Mo-Precat.** (10)	(*S*$_a$)-**L3** (30)	0.03	Toluene	50	8	7	90 : 10
31	**Mo-Precat.** (30)	(*S*$_a$)-**L3** (90)	0.03	Toluene	50	>95	76	87 : 13
32	**Mo-Precat.** (20)	(*S*$_a$)-**L3** (60)	0.03	Toluene	40	5	5	84 : 16

Entry	Catalyst (mol%)	Ligand (mol%)	Concen. mol·L^{-1}	Solvent	Temp. [°C]	Conversion [%]	Yield [%]	e. r.
33	**Mo-Precat.** (20)	(S_a)-**L3** (60)	0.03	CH_2Cl_2	40	58	57	80 : 20
34	**Mo-Precat.** (10)	(R_a)-**L1** (30)	0.03	Toluene	65	83	77	29 : 71
35	**Mo-Precat.** (10)	(R_a)-**L6** (30)	0.03	Toluene	65	>95	98	40 : 60
36	**Mo-Precat.** (10)	(R_a)-**L7** (30)	0.03	Toluene	65	>95	83	35 : 65
37	**Mo-Precat.** (10)	(R_a)-**L14** (30)	0.03	Toluene	65	0	–	–
38	**Mo-Precat.** (10)	(R_a)-**L15** (30)	0.03	Toluene	65	>95	95	44 : 56
39	**Mo-Precat.** (10)	(R_a)-**L9** (30)	0.03	Toluene	65	>95	90	41 : 59
40	**Mo-Precat.** (10)	(S_a)-**L5** (30)	0.03	Toluene	65	NR	–	–
41	**Mo-Precat.** (10)	(R)-**L16** (30)	0.03	Toluene	65	5	5	58 : 42
42	**Mo-Precat.** (10)	(S_a)-**L17** (30)	0.03	Toluene	65	>95	84	63 : 37
43	**Mo-Precat.** (10)	(S_a)-**L18** (30)	0.03	Toluene	65	<5%	–	56 : 44
44	**Mo-Precat.** (10)	(R_a)-**L19** (30)	0.03	Toluene	65	NR	–	–
45	**Mo-Precat.** (20)	(S_a)-**L3** (60)	0.03	Benzene	50	>95	74	87 : 13
46	**Mo-Precat.** (20)	(S_a)-**L3** (60)	0.03	Benzene	40	>95	81	86 : 14
47	**Mo-Precat.** (20)	(S_a)-**L3** (60)	0.03	m-Xylene	50	>95	79	87 : 13
48	**Mo-Precat.** (20)	(S_a)-**L3** (60)	0.03	DCE	50	25	24	69 : 31
49	**Mo-Precat.** (20)	(S_a)-**L3** (60)	0.03	CF_3-toluene	50	>95	81	86 : 14
50	**Mo-Precat.** (20)	(S_a)-**L3** (60)	0.03	C_6F_6	50	31	25	72 : 28
51	**Mo-Precat.** (20)	(S_a)-**L3** (60)	0.03	Mesitylene	50	>95	74	86 : 14
53	**Mo-Precat.** (20)	(S_a)-**L3** (60)	0.03	1,2-Dichloro-benzene	50	>95	–	85 : 15

[a]Reactions were performed on 15.0 µmol scale of **15b** for 18 h. [b]Conversion and yield were determined by ^{1}H-NMR with durene as an internal standard. [c]Determined by HPLC on a chiral stationary phase of the crude product (Chiralpak IG-N3 column (3 µm, 250x4.6 mm, heptane/*i*PrOH 96:4, 1.0 mL/min, 20°C). [d] Mo-Precatalyst and the ligand were stirred at 55°C for 1 h before the substrate was added

Competition experiment: Investigation of the effect of substituent`s size on the reaction rate

Control experiment was performed according to the general procedure **H** using **15c** (2.55 mg, 7.50 µmol), **15d** (2.78 mg, 7.50 µmol) in toluene (0.5 mL) at 65°C for 15 minutes. After the indicated time ,^{1}H-NMR analysis of the crude reaction mixture revealed that full conversion was achieved for the transformation of **15d** to **16d**, while 80% conversion was observed for the substrate **15c**.

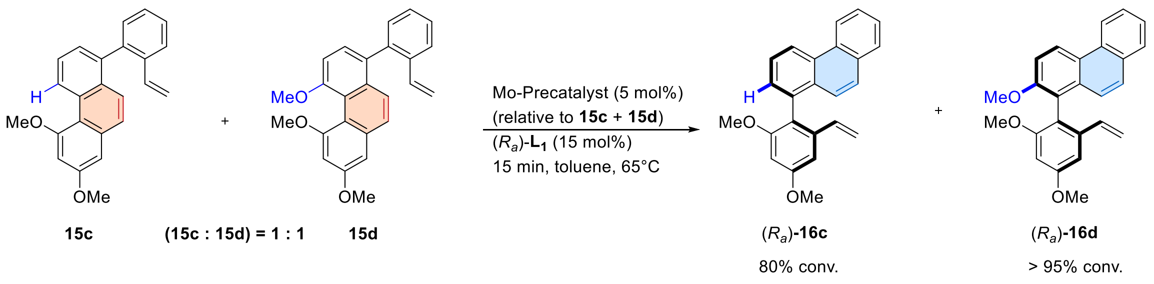

General procedure L for atroposelective aromatic ring-opening metathesis – RCM cascade:

Mo-precursor (2.07 mg, 3.50 µmol, 5 mol%), (R_a)-[1,2':4',1":3",1'"-quaternaphthalene]-2",3'-diol (R_a)-**L1** (5.66 mg, 10.5 µmol, 15 mol%) were weighed in 10mL crimp cap vial and toluene (1.5 mL) was added. The obtained solution was stirred for 1 h at room temperature in an argon-filled glovebox before a solution of the corresponding substrate (70.0 µmol, 1.00 eq.) in toluene (0.83 mL) was added. The capped vial was then taken out of the glovebox and stirred at 65°C for 18 h. The reaction mixture was diluted with EtOAc and filtered through a thin layer of silica gel. The solvents were removed under reduced pressure and the residue was purified by silica gel column chromatography (cyclohexane/EtOAc 100:0→80:20) to give the desired product. The e.r. of the isolated products were determined by HPLC on a chiral stationary phase.

(R_a)-1-(2,4-Dimethoxy-6-vinylphenyl)-2-methylphenanthrene ((R_a)-16a):

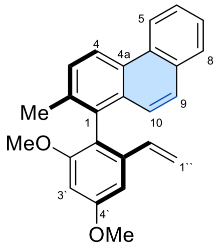

(R_a)-**16a**

Prepared according to the general procedure **L** using 5,7-dimethoxy-4-methyl-1-(2-vinylphenyl)phenanthrene **15a** (24.8 mg, 70.0 µmol) to give the title compound (R_a)-**16a** as a beige solid (20.6 mg, 58.1 µmol, 83%, 2:98 e.r., m.p. 136.1 – 137.0°C): R_f 0.36 (cyclohexane/EtOAc 5:1); $[\alpha]_D^{20}$ = +29. (c 0.18, $CHCl_3$); ν_{max} (neat): 3062w, 2921m, 2850w, 1729w, 1600s, 1594s, 1458s, 1422m, 1316s, 1276w, 1201s, 1156s, 1083m, 1033w, 915w, 816s, 754s; ^{1}H NMR (500 MHz, $CDCl_3$) δ = 8.71 (1H, d, ^3J 8.2 Hz, C5*H*), 8.65 (1H, d, ^3J 8.5 Hz, C4*H*), 7.82 (1H, d, ^3J 6.7 Hz, C8*H*), 7.63 (1H, ddd, ^3J 8.3, ^3J 7.0, ^4J 1.4 Hz, C6*H*), 7.59 (1H, d, ^3J 8.5 Hz, C3*H*), 7.57 – 7.52 (2H, m, C7*H*, C9*H*), 7.24 (1H, d, ^3J 8.8 Hz, C10*H*), 6.91 (1H, d, ^4J 2.3 Hz, C5`*H*), 6.59 (1H, d, $^4J$ 2.3 Hz, C3`*H*), 6.06 (1H, dd, ^3J 17.5, ^3J 11.0 Hz, C2``*H*), 5.61 (1H, dd, $^3J$ 17.5, $^2J$ 1.2 Hz, C1``*H*), 4.93 (1H, dd, ^3J 11.0, ^2J 1.1 Hz, C1``*H*), 3.95 (3H, s, C4`–OC$H_3$), 3.61 (3H, s, C2`–OC$H_3$), 2.17 (3H, s, C2–C$H_3$); $^{13}$C NMR (126 MHz, $CDCl_3$) δ = 160.4 (*C4`*), 158.6 (*C2`*), 138.6 (*C6`*), 136.1 (*C2*), 135.0 (*C2``*), 133.9 (*C1*), 131.7 (*C10a*), 131.6 (*C8a*), 130.7 (*C4b*), 128.8 (*C3*), 128.6 (*C4a*), 128.5 (*C8*), 126.9 (*C9*), 126.5 (*C6*), 126.2 (*C7*), 125.0 (*C10*), 122.7 (*C5*), 122.0 (*C4*), 120.5 (*C1`*), 115.1 (*C1``*), 100.8 (*C5`*), 98.7 (*C3`*), 56.0 (*C2`*–O$CH_3$), 55.6 (*C4`*–OCH_3), 20.5 (C2–CH_3); ESI-MS: m/z calcd. for $C_{25}H_{22}AgO_2$ 461.0665 found 461.0658 [M+Ag$^+$]. The e.r. of the isolated product was determined by HPLC on a chiral stationary phase (Chiralcel IC 3 µm, 250x 4.6 mm, 1.0 mL/min, heptane/*i*PrOH 97.5 : 2.5, 20°C): t_{major} = 5.7 min and t_{minor} = 5.1 min.

(S_a)-7-(2,4-Dimethyl-6-vinylphenyl)phenanthro[2,3-d][1,3]dioxole ((S_a)-16b):

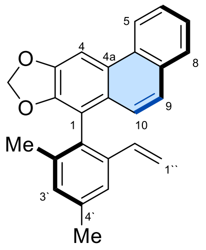

(S_a)-**16b**

Prepared according to the modified general procedure **L** using 9,11-dimethyl-5-(2-vinylphenyl)phenanthro[3,4-d][1,3]dioxole **15b** (24.7 mg, 70.0 µmol), Mo-precursor (4.14 mg, 7.00 µmol, 10 mol%), (S_a)-[3-(2,4-dimethylphenyl)-3'-mesityl-[1,1'-binaphthalene]-2,2'-diol ((S_a)-**L3**) (11.0 mg, 21.0 µmol, 30 mol%) to give the title compound (S_a)-**16b** as a white solid (20.4 mg, 57.9 µmol, 83%, 86:14 e.r., m.p. 146.6 – 147.0°C): R_f 0.78 (cyclohexane/EtOAc 8:1); $[\alpha]_D^{20}$ = –10 (c 0.56, $CHCl_3$); ν_{max} (neat): 3043w, 2985w, 2956w, 2917w, 2855w, 1607w, 1563w, 1452s, 1391m, 1265m, 1233s, 1161w, 1104m, 1042s,

941m, 908m, 857m, 816s, 748s, 667w; ^{1}H NMR (500 MHz, $CDCl_3$) δ = 8.54 (1H, d, ^{3}J 8.3 Hz, C5*H*), 8.11 (1H, s, C4*H*), 7.87 – 7.78 (1H, m, C8*H*), 7.62 (1H, ddd, ^{3}J 8.3, ^{3}J 7.0, ^{4}J 1.4 Hz, C6*H*), 7.56 – 7.50 (2H, m, C7*H*, C9*H*), 7.44 (1H, s, C5`*H*), 7.19 (1H, d, $^{3}J$ 9.0 Hz, C10*H*), 7.14 (1H, s, C3`*H*), 6.27 (1H, dd, ^{3}J 17.5, ^{3}J 11.0 Hz, C2``*H*), 6.11 – 5.95 (2H, m, $OCH_2O$), 5.64 (1H, dd, *J* $^{3}J$ 17.5, $^{2}J$ 1.2 Hz, C1``*H*), 4.96 (1H, dd, ^{3}J 11.0, ^{2}J 1.2 Hz, C1``*H*), 2.44 (3H, s, C4`–$CH_3$), 1.98 (3H, s, C2`–$CH_3$); $^{13}$C NMR (126 MHz, $CDCl_3$) δ = 147.6 (*C2*/*C3*), 145.2 (*C2*/*C3*), 138.2 (*C4`*), 137.8 (*C2`*), 137.3 (*C6`*), 135.3 (*C2``*), 131.5 (*C8a*), 130.7 (*C3`*), 130.4 (*C4b*), 129.5 (*C1`*), 128.7 (*C8*), 127.6 (*C10a*), 126.9 (*C4a*), 126.4 (*C6*), 125.9 (*C7*), 125.5 (*C9*), 123.8 (*C10*), 123.5 (*C5`*), 122.7 (*C5*), 116.7 (*C1*), 114.9 (*C1``*), 101.5 (OCH_2O), 100.4 (*C4*), 21.5 (C2`–$CH_3$), 20.2 (C4`–CH_3); ESI-MS: m/z calcd. for $C_{25}H_{20}AgO_2$ 459.0509 found 459.0503 [M+Ag^{+}]. The e.r. of the isolated product was determined by HPLC on a chiral stationary phase (Chiralcel IG 3 µm, 250x 4.6 mm, 1.0 mL/min, heptane/*i*PrOH 96 : 4, 20°C): t_{major} = 8.1 min and t_{minor} = 9.1 min.

(*R*$_a$)-1-(2,4-Dimethoxy-6-vinylphenyl)phenanthrene ((*R*$_a$)-16c):

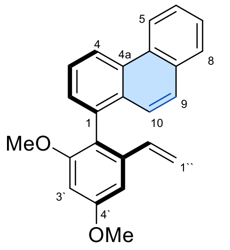

(*R*$_a$)-**16c**

Prepared according to the general procedure **L** using 5,7-dimethoxy-1-(2-vinylphenyl)phenanthrene **15c** (23.8 mg, 70.0 µmol) to give the title compound (*R*$_a$)-**16c** as a beige oil (19.0 mg, 55.8 µmol, 80%, 30:70 e.r.): R$_f$ 0.41 (cyclohexane/EtOAc 5:1); $[\alpha]_D^{20}$ = +7.0 (c 0.18, $CHCl_3$); ν_{max} (neat): 3054w, 2999w, 2959w, 2874w, 2834w, 1600s, 1454m, 1420m, 1318s, 1277w, 1201s, 1156s, 1088w, 1033w, 990w, 914w, 832w; ^{1}H NMR (500 MHz, $CDCl_3$) δ = 8.81 – 8.63 (2H, m, C4*H*, C5*H*), 7.88 – 7.82 (1H, m, C8*H*), 7.70 (1H, dd, ^{3}J 8.3, ^{3}J 7.2 Hz, C3*H*), 7.66 (1H, ddd, ^{3}J 8.4, ^{3}J 7.0, ^{4}J 1.4 Hz, C6*H*), 7.62 – 7.55 (2H, m, C7*H*, C9*H*), 7.41 (1H, dd, ^{3}J 7.2, ^{4}J 1.0 Hz, C2*H*), 7.36 (1H, d, ^{3}J 9.1 Hz, C10*H*), 6.90 (1H, d, ^{4}J 2.3 Hz, C5`*H*), 6.58 (1H, d, $^{4}J$ 2.3 Hz, C3`*H*), 6.20 (1H, dd, ^{3}J 17.5, ^{3}J 11.0 Hz, C2``*H*), 5.63 (1H, dd, $^{3}J$ 17.5, $^{2}J$ 1.1 Hz, C1``*H*), 4.97 (1H, dd, ^{3}J 11.0, ^{2}J 1.1 Hz, C1``*H*), 3.95 (3H, s, C4`–O$CH_3$), 3.62 (3H, s, C2`–O$CH_3$); $^{13}$C NMR (126 MHz, $CDCl_3$) δ = 160.4 (*C4`*), 158.9 (*C2`*), 138.9 (*C6`*), 135.4 (*C1*), 135.4 (*C2``*), 132.0 (*C8a*), 131.6 (*C10a*), 130.7 (*C4b*), 130.5 (*C4a*), 129.6 (*C2*), 128.6 (*C8*), 126.9 (*C9*), 126.62 (*C6*/*C7*), 126.61 (*C6*/*C7*), 126.1 (*C3*), 125.0 (*C10*), 123.0 (*C5*), 122.2 (*C4*), 121.7 (*C1`*), 115.0 (*C1``*), 100.8 (*C5`*), 98.5 (*C3`*), 56.0 (C2`–O$CH_3$), 55.6 (C4`–OCH_3); ESI-MS: m/z calcd. for $C_{24}H_{20}AgO$ 447.0509 found 447.0506 [M+Ag^{+}]. The e.r. of the isolated product was determined by HPLC on a chiral stationary phase (Chiralcel IB 3 µm, 250x 4.6 mm, 1.0 mL/min, heptane/*i*PrOH 97.5 : 2.5, 20°C): t_{major} = 7.1 min and t_{minor} = 6.6 min.

(S_a)-1-(2,4-Dimethoxy-6-vinylphenyl)-2-methoxyphenanthrene ((S_a)-16d):

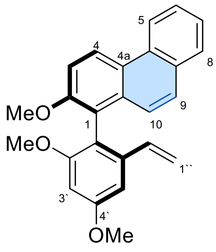

(S_a)-**16d**

Prepared according to the modified general procedure **L** using 4,5,7-trimethoxy-1-(2-vinylphenyl)phenanthrene **15d** (25.9 mg, 70.0 µmol), Mo-precursor (4.14 mg, 7.00 µmol, 10 mol%), and (R_a)-[1,2':4',1":3",1'''-quaternaphthalene]-2",3'-diol (11.3 mg, 21.0 µmol, 30 mol%) to give the title compound (R_a)-**16d** as a white solid (25.4 mg, 68.6 µmol, 98%, 94:6 e.r., m.p. 157.0 – 158.0°C): R_f 0.33 (cyclohexane/EtOAc 5:1); $[\alpha]_D^{20}$ = –5.1 (c 0.44, $CHCl_3$); ν_{max} (neat): 3058w, 3005w, 2934w, 2836w, 1732w, 1599s, 1571s, 1528w, 1460s, 1418m, 1316s, 1274s, 1200s, 1155s, 1068s, 1032m, 911w, 815m, 752m, 632s; ^{1}H NMR (500 MHz, $CDCl_3$) δ = 8.74 (d, ^3J 9.1 Hz, C4*H*), 8.66 (1H, d, ^3J 8.3 Hz, C5*H*), 7.80 (1H, d, ^3J 7.9 Hz, C8*H*), 7.62 (1H, ddd, ^3J 8.3, ^3J 7.1, ^4J 1.3 Hz, C6*H*), 7.56 (1H, d, ^3J 9.1 Hz, C9*H*), 7.52 (1H, td, ^3J 7.5, ^4J 1.0 Hz, C7*H*), 7.45 (1H, d, ^3J 9.1 Hz, C3*H*), 7.21 (1H, d, ^3J 9.1 Hz, C10*H*), 6.92 (1H, d, ^4J 2.3 Hz, C5`*H*), 6.60 (1H, d, $^4J$ 2.3 Hz, C3`*H*), 6.18 (1H, dd, ^3J 17.5, ^3J 11.0 Hz, C2``*H*), 5.63 (1H, dd, $^3J$ 17.5, $^2J$ 1.1 Hz, C1``*H*), 4.95 (1H, dd, ^3J 11.0, ^2J 1.1 Hz, C1``*H*), 3.94 (3H, s, C4`–OC$H_3$), 3.86 (3H, s, C2–OC$H_3$), 3.62 (3H, s, 3H, s, C2`–OC$H_3$); $^{13}$C NMR (126 MHz, $CDCl_3$) δ = 160.4 (*C4`*), 159.0 (*C2`*), 156.0 (*C2*), 139.0 (*C6`*), 135.3 (*C2``*), 133.0 (*C4b*), 131.0 (*C8a*), 130.8 (*C10a*), 128.6 (*C8*), 127.5 (*C9*), 126.7 (*C6*), 125.8 (*C7*), 124.9 (*C4a*), 124.6 (*C10*), 123.8 (*C4*), 122.4 (*C5*), 121.4 (*C1*), 117.6 (*C1`*), 114.8 (*C1``*), 112.6 (*C3*), 100.9 (*C5`*), 98.8 (*C3`*), 56.8 (C2–OCH_3), 56.1 (C2`–O$CH_3$), 55.5 (C4`–OCH_3); ESI-MS: m/z calcd. for $C_{25}H_{22}AgO_3$ 477.0614 found 447.0620 [M+Ag$^+$]. The e.r. of the isolateGd product was determined by HPLC on a chiral stationary phase (Chiralcel I 3 µm, 250x 4.6 mm, 1.0 mL/min, heptane/*i*PrOH 90 : 10, 20°C): t_{major} = 7.0 min and t_{minor} = 7.7 min.

(R_a)-1-(6-Methoxy-3-methyl-2-vinylphenyl)-2-methylphenanthrene ((R_a)-16e):

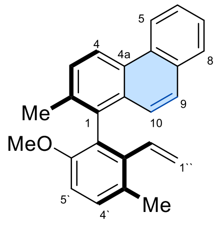

(R_a)-**16e**

Prepared according to the general procedure **L** using 4-methoxy-1,5-dimethyl-8-(2-vinylphenyl)phenanthrene **15e** (23.7 mg, 70.0 µmol) to give the title compound (R_a)-**16e** as a yellow oil (20.6 mg, 60.9 µmol, 87%, >99:1 e.r.): R_f 0.85 (cyclohexane/EtOAc 5:1); $[\alpha]_D^{20}$ = +55 (c 0.15, $CHCl_3$); ν_{max} (neat): 3008w, 2925w, 2855w, 1579m, 1463s, 1378w, 1257s, 1088m, 1056m, 922w, 867w, 813s, 750s, 683w, 637w; ^{1}H NMR (500 MHz, $CDCl_3$) δ = 8.70 (1H, d, ^3J 8.3 Hz, C5*H*), 8.62 (1H, d, ^3J 8.5 Hz, C4*H*), 7.82 (1H, dd, ^3J 7.9, ^4J 1.3 Hz, C8*H*), 7.63 (1H, ddd, ^3J 8.5, ^3J 7.0, ^4J 1.4 Hz, C6*H*), 7.59 – 7.50 (3H, m, C3*H*, C7*H*, C9*H*), 7.29 (1H, d, ^3J 8.5 Hz, C4`*H*), 7.26 – 7.22 (1H, m, C10*H*), 6.90 (1H, d, $^3J$ 8.5 Hz, C5`*H*), 6.18 (1H, dd, ^3J 17.9, ^3J 11.7 Hz, C2``*H*), 5.01 – 4.82 (2H, m, C1``*H*), 3.60 (3H, s, OCH_3), 2.40 (3H, s, C3`–C$H_3$), 2.16 (3H, s, C2–C$H_3$); $^{13}$C NMR (126 MHz, $CDCl_3$) δ = 155.5 (*C6`*), 138.5 (*C2`*), 135.3 (*C3*), 135.1 (*C2*), 134.3 (*C2``*), 131.6 (*C8a*), 131.2 (*C10a*), 130.8 (*C4b*), 130.6 (*C4`*), 128.8 (*C3*), 128.5 (*C8*), 128.4 (*C3`*), 128.3 (*C4a*), 127.5 (*C1`*), 126.7 (*C9*), 126.5 (*C6*), 126.1 (*C7*), 125.0 (*C10*), 122.8 (*C5*), 121.8 (*C4*), 119.0 (*C1``*), 110 (*C5`*), 56.1 (O$CH_3$), 20.8 (C3`–CH_3), 20.5 (C2–CH_3); ESI-MS: m/z calcd. for $C_{25}H_{23}O$ 339.1743 found 339.1742 [M+H$^+$]. The e.r. of the isolated

product was determined by HPLC on a chiral stationary phase (Chiralcel IG 3 µm, 250x 4.6 mm, 1.0 mL/min, heptane/*i*PrOH 97.5 : 2.5, 20°C): t_{major} = 4.4 min and t_{minor} = 4.7 min.

(*Ra*)-1-(3,6-Dimethyl-2-vinylphenyl)-2-methoxyphenanthrene ((*Ra*)-16f):

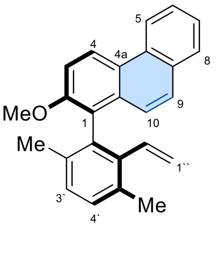

(R_a)-**16f**

Prepared according to the general procedure **L** using 4-methoxy-5,8-dimethyl-1-(2-vinylphenyl)phenanthrene **15f** (23.7 mg, 70.0 µmol) to give the title compound (R_a)-**16f** as a a beige solid (5.30 mg, 15.7 µmol, 22%, >99:1 e.r., m.p. 127.5 – 129.0°C): R_f 0.44 (cyclohexane/EtOAc 5:1); $[\alpha]_D^{20}$ = +25 (c 0.55, $CHCl_3$); ν_{max} (neat): 3060w, 3003w, 2959w, 2916w, 2837w, 1731w, 1592w, 1527w, 1461m, 1257m, 1169w, 1082m, 921w, 795s; ^{1}H NMR (500 MHz, $CDCl_3$) δ = 8.72 (1H, d, ^3J 9.0 Hz, C4*H*), 8.66 (1H, d, ^3J 7.9 Hz, C5*H*), 7.81 (1H, dd, ^3J 7.9, ^4J 1.3 Hz, C8*H*), 7.63 (1H, ddd, ^3J 7.9, ^3J 7.0, ^4J 1.4 Hz, C6*H*), 7.58 – 7.49 (2H, m, C7*H*, C10*H*), 7.42 (1H, d, ^3J 9.0 Hz, C3*H*), 7.22 (1H, d, ^3J 7.7 Hz, C4`*H*), 7.18 (1H, d, $^3J$ 7.8 Hz, C3`*H*), 7.10 (1H, d, ^3J 9.1 Hz, C9*H*), 6.32 – 6.23 (1H, m, C2``*H*), 4.95 – 4.87 (2H, m, C1``*H*), 3.88 (3H, s, OCH_3), 2.41 (3H, s, C5`–C$H_3$), 1.85 (3H, s, C2`–CH_3); ^{13}C NMR (126 MHz, $CDCl_3$) δ = 154.9 (*C2*), 138.2 (*C6`*), 135.2 (*C1`*/*C2``*), 135.1 (*C2`*), 133.2 (*C5`*), 131.8 (*C10a*), 131.0 (*C8a*), 130.8 (*C4b*), 129.8 (*C4`*), 128.6 (*C8*), 128.5 (*C3`*), 127.7 (*C10*), 126.8 (*C6*), 125.8 (*C7*), 125.6 (*C1*), 124.8 (*C4a*), 124.2 (*C9*), 123.4 (*C4*), 122.4 (*C5*), 118.4 (*C1``*), 112.0 (*C3*), 56.3 (OCH_3), 21.1 (C5`–$CH_3$), 20.1 (C2`–CH_3); ESI-MS: m/z calcd. for $C_{25}H_{22}AgO$ 445.0716 found 445.0713 [M+Ag^+]. The e.r. of the isolated product was determined by HPLC on a chiral stationary phase (Chiralcel IG 3 µm, 250x 4.6 mm, 1.0 mL/min, heptane/*i*PrOH 99 : 1, 20°C): t_{major} = 5.1 min and t_{minor} = 5.4 min.

(*Ra*)-6-Chloro-1-(2,4-dimethoxy-6-vinylphenyl)-2-methylphenanthrene ((*Ra*)-16g):

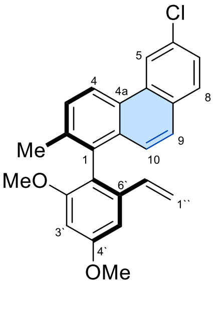

(R_a)-**16g**

Prepared according to the general procedure **L** using 1-(5-chloro-2-vinylphenyl)-5,7-dimethoxy-4-methylphenanthrene **15g** (27.2 mg, 70.0 µmol) to give the title compound (R_a)-**16g** as a transparent oil (17.6 mg, 45.3 µmol, 65%, 95:5 e.r.,): R_f 0.40 (cyclohexane/EtOAc 5:1); $[\alpha]_D^{20}$ = +10 (c 0.14, $CHCl_3$); ν_{max} (neat): 2995w, 2936w, 2834w, 1600m, 1570m, 1454m, 1421w, 1316w, 1278w, 1201m, 1156s, 1082w, 1011w, 910w, 836w, 732w; ^{1}H NMR (500 MHz, $CDCl_3$) δ = 8.66 (1H, d, ^3J 1.9 Hz, C5*H*), 8.55 (1H, d, ^3J 8.5 Hz, C4*H*), 7.75 (1H, d, ^3J 8.5 Hz, C8*H*), 7.60 (1H, d, ^3J 8.5 Hz, C3*H*), 7.54 – 7.46 (2H, m, C7*H*, C9*H*), 7.25 (1H, d, ^3J 9.2 Hz, C10*H*), 6.90 (1H, d, ^3J 2.3 Hz, C5`*H*), 6.59 (1H, d, $^3J$ 2.3 Hz, C3`*H*), 6.04 (1H, dd, ^3J 17.5, ^3J 11.0 Hz, C2``*H*), 5.62 (1H, dd, $^3J$ 17.5, $^2J$ 1.1 Hz, C1``*H*), 4.94 (1H, dd, ^3J 11.0, ^2J 1.1 Hz, C1``*H*), 3.95 (3H, s, C4`–OC$H_3$), 3.62 (3H, s, C2`–OC$H_3$), 2.17 (3H, s, C2–C$H_3$); $^{13}$C NMR (126 MHz, $CDCl_3$) δ = 160.5 (*C4`*), 158.5 (*C2`*), 138.6 (*C6`*), 136.9 (*C2*), 134.8 (*C2``*), 134.0 (*C1*), 132.5 (*C8a*), 132.0 (*C10a*/*C6*), 131.9 (*C4b*), 129.9 (*C8*), 129.1 (*C3*), 127.7 (*C4a*), 126.7 (*C7*), 126.2 (*C9*), 125.3 (*C10*), 122.5 (*C5*), 122.0 (*C4*), 120.2 (*C1`*), 115.2 (*C1``*), 100.9 (*C5`*), 98.6 (*C3`*), 56.0 (C2`–OCH_3),

55.6 (C4`–OCH3), 20.5 (C2–CH3); ESI-MS: m/z calcd. for $C_{25}H_{21}AgClO_2$ 495.0276 found 495.0273 [M+Ag$^+$]. The e.r. of the isolated product was determined by HPLC on a chiral stationary phase (Chiralcel IG 3 µm, 250x 4.6 mm, 1.0 mL/min, heptane/*i*PrOH 97.5 : 2.5, 20°C): t_{major} = 5.7 min and t_{minor} = 6.0 min.

(*S*$_a$)-6-Chloro-2-methoxy-1-(2-methoxy-6-vinylphenyl)phenanthrene ((*S*$_a$)-16h):

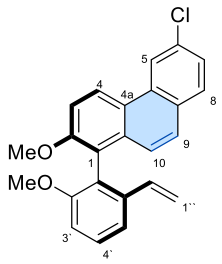

(*S*$_a$)-**16h**

Prepared according to the general procedure **L** using 1-(5-chloro-2-vinylphenyl)-4,5-dimethoxyphenanthrene **15h** (26.2 mg, 70.0 µmol) to give the title compound (*S*$_a$)-**16h** as a beige solid (18.0 mg, 48.0 µmol, 69%, 89:11 e.r., m.p. 182.3 – 183.3°C): R_f 0.28 (cyclohexane/EtOAc 5:1); ν_{max} (neat): 3062w, 2934w, 2837w, 1833w, 1736w, 1570m, 1515m, 1468s, 1400w, 1254s, 1198w, 1062s, 1010s, 911m, 871m, 838s, 775w, 743m; $[\alpha]_D^{21}$ = –27 (c 0.16, $CHCl_3$); ^{1}H NMR (500 MHz, $CDCl_3$) δ = 8.65 (1H, d, ^3J 9.1 Hz, C4*H*), 8.61 (1H, d, ^4J 1.8 Hz, C5*H*), 7.72 (1H, d, ^3J 8.4 Hz, C8*H*), 7.50 (1H, d, ^3J 9.1 Hz, C9*H*), 7.48 – 7.45 (2H, m, C3*H*, C7*H*), 7.44 – 7.38 (2H, m, C4`*H*, C5`*H*), 7.15 (1H, d, ^3J 9.1 Hz, C10*H*), 6.99 (1H, dd, ^3J 7.9, ^4J 1.3 Hz, C3`*H*), 6.15 (1H, dd, ^3J 17.5, ^3J 11.0 Hz, C2``*H*), 5.63 (1H, dd, $^3J$ 17.5, $^2J$ 1.2 Hz, C1``*H*), 4.95 (1H, dd, ^3J 11.0, ^2J 1.2 Hz, C1``*H*), 3.86 (3H, s, C2–OC*H*3), 3.64 (3H, s, C2`–OC*H*3); $^{13}$C NMR (126 MHz, $CDCl_3$) δ = 158.0 (*C2`*), 156.2 (*C2*), 138.8 (*C6`*), 135.0 (*C2``*), 132.8 (*C4b*), 132.7 (*C6*), 132.0 (*C10a*), 129.9 (*C8*), 129.3 (*C8a*), 128.9 (*C4`*), 126.9 (*C9*), 126.3 (*C7*), 124.8 (*C10*), 124.4 (*C1`*), 123.98 (*C4*), 123.91 (*C4a*), 122.1 (*C5*), 121.7 (*C1*), 117.5 (*C5`*), 115.0 (*C1``*), 112.8 (*C3*), 110.4 (*C3`*), 56.7 (C2–O*C*H3), 56.1 (C2`–O*C*H3); ESI-MS: m/z calcd. for $C_{24}H_{20}ClO_2$ 375.1146 found 375.1151 [M+H$^+$]. The e.r. of the isolated product was determined by HPLC on a chiral stationary phase (Chiralcel IG 3 µm, 250x 4.6 mm, 1.0 mL/min, heptane/*i*PrOH 90 : 10, 20°C): t_{major} = 5.4 min and t_{minor} = 6.5 min.

(*S*$_a$)-1-(2,4-Dimethyl-6-vinylphenyl)-2-methylphenanthrene ((*S*$_a$)-16i):

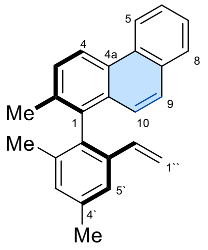

(*S*$_a$)-**16i**

Prepared according to the general procedure **L** using 4,5,7-trimethyl-1-(2-vinylphenyl)phenanthrene **15i** (22.6 mg, 70.0 µmol) to give the title compound (*S*$_a$)-**16i** as a transparent oil (21.0 mg, 65.1 µmol, 93%, 11:89 e.r.): R_f 0.67 (cyclohexane/EtOAc 5:1); $[\alpha]_D^{20}$ = +45 (c 0.10, $CHCl_3$): ν_{max} (neat): 3042w, 2949w, 2917m, 2857w, 1606w, 1559w, 1459m, 1378w, 1304w, 1261w, 1220w, 1035w, 990w, 910w, 856w, 816s; ^{1}H NMR (500 MHz, $CDCl_3$) δ = 8.73 (1H, d, ^3J 8.2 Hz, C5*H*), 8.66 (1H, d, ^3J 8.5 Hz, C4*H*), 7.83 (1H, dd, ^3J 7.9, ^4J 1.2 Hz, C8*H*), 7.65 (1H, ddd, ^3J 8.2, ^3J 7.0, ^4J 1.2 Hz, C6*H*), 7.60 (1H, d, ^3J 8.5 Hz, C3*H*), 7.58 – 7.53 (2H, m, C7*H*, C9*H*), 7.43 (1H, s, C5`*H*), 7.17 (1H, d, $^3J$ 9.1 Hz, C10*H*), 7.13 (1H, s, C3`*H*), 6.08 (1H, dd, ^3J 17.5, ^3J 11.0 Hz, C2``*H*), 5.58 (1H, dd, $^3J$ 17.5, $^2J$ 1.3 Hz, C1``*H*), 4.88 (1H, dd, ^3J 11.0, ^2J 1.3 Hz, C1``*H*), 2.46 (3H, s, C4`–C*H*3), 2.12 (3H, s, C2–C*H*3), 1.80 (3H, s, C2`–C*H*3); ^{13}C NMR (126 MHz, $CDCl_3$) δ = 137.1 (*C4`*), 137.0 (*C2`*), 136.9 (*C1*), 136.7 (*C6`*), 135.3 (*C1`*), 135.0 (*C2*), 131.6 (*C8a*), 130.9 (*C10a*), 130.7 (*C4b*), 130.6 (*C3`*), 129.0 (*C3*),

128.7 (*C4a*), 128.6 (*C8*), 127.1 (*C9*), 126.6 (*C6*), 126.4 (*C7*), 124.6 (*C10*), 123.3 (*C5`*), 122.7 (*C5*), 121.9 (*C4*), 114.3 (*C1``*), 21.5 (C4`–$CH_3$), 20.3 (C2–$CH_3$), 20.0 (C2`–CH_3); ESI-MS: m/z calcd. for $C_{25}H_{22}Ag$ 429.0767 found 429.0763 [M+Ag^+]. The e.r. of the isolated product was determined by HPLC on a chiral stationary phase (Chiralcel IG 3 µm, 250x 4.6 mm, 1.0 mL/min, heptane/*i*PrOH 99.5 : 0.5, 40°C): t_{major} = 9.8 min and t_{minor} = 9.4 min.

(*S_a*)-1-(3-Bromo-6-methoxy-2-vinylphenyl)-2-methoxyphenanthrene ((*S_a*)-16j):

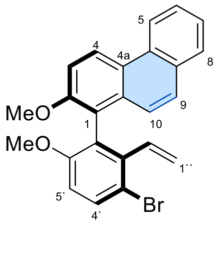

(S_a)-**16j**

Prepared according to the modified general procedure **L** using 1-bromo-4,5-dimethoxy-8-(2-vinylphenyl)phenanthrene **15j** (29.4 mg, 70.0 µmol), Mo-precursor (4.14 mg, 7.00 µmol, 10 mol%), and (*R_a*)-[1,2':4',1":3",1'"-quaternaphthalene]-2",3'-diol (11.3 mg, 21.0 µmol, 30 mol%) to give the title compound (S_a)-**16j** as a white solid (28.0 mg, 66.8 µmol, 95%, >99:1 e.r., m.p. 80.0 – 82.0°C): R_f 0.37 (cyclohexane/EtOAc 5:1); $[\alpha]_D^{20}$ = –2.4 (c 0.20, $CHCl_3$): ν_{max} (neat): 3049w, 3003w, 2934w, 2836w, 170w, 1578w, 1528w, 1454s, 1429m, 1254s, 1065s, 906m, 866w, 807s, 838s, 648w; ^1H NMR (500 MHz, $CDCl_3$) δ = 8.72 (1H, d, ^3J 9.1 Hz, C4*H*), 8.64 (1H, d, ^3J 8.3 Hz, C5*H*), 7.81 (1H, dd, ^3J 7.9, ^4J 1.1 Hz, C8*H*), 7.66 (1H, d, ^3J 8.8 Hz, C4`*H*), 7.63 (1H, ddd, $^3J$ 8.3, $^3J$ 7.0, $^4J$ 1.4 Hz, C6*H*), 7.58 (1H, d, $^3J$ 9.1 Hz, C9*H*), 7.52 (1H, ddd, $^3J$ 7.9, $^3J$ 7.0, $^4J$ 1.1 Hz, C7*H*), 7.40 (1H, d, $^3J$ 9.1 Hz, C3*H*), 7.17 (1H, d, $^3J$ 9.1 Hz, C10*H*), 6.87 (1H, d, $^3J$ 8.8 Hz, C5`*H*), 6.38 (1H, dd, ^3J 17.7, ^3J 11.6 Hz, C2``*H*), 5.01 – 4.75 (2H, m, C1``*H*), 3.87 (3H, s, C2–OCH_3), 3.61 (3H, s, C6`–OC$H_3$); $^{13}C$ NMR (126 MHz, $CDCl_3$) δ = 157.3 (*C6`*), 155.2 (*C2*), 139.8 (*C2`*), 134.8 (*C2``*), 132.8 (*C4`*), 132.2 (*C10a*), 131.0 (*C8a*), 130.8 (*C4b*), 128.6 (*C8*), 127.7 (*C9*), 126.8 (*C6*), 126.4 (*C1`*), 125.8 (*C7*), 124.7 (*C4a*), 124.1 (*C10*), 123.9 (*C4*), 122.4 (*C5*), 122.0 (*C1*), 120.1 (*C1``*), 114.7 (*C3`*), 112.2 (*C3*), 111.7 (*C5`*), 56.5 (C2–OCH_3), 56.3 (C6`–OCH_3); ESI-MS: m/z calcd. for $C_{24}H_{30}BrO_2$ 419.0641 found 419.0641 [M+H^+]. The e.r. of the isolated product was determined by HPLC on a chiral stationary phase (Chiralcel IG 3 µm, 250x 4.6 mm, 1.0 mL/min, heptane/*i*PrOH 97.5 : 2.5, 20°C): t_{major} = 7.3 min and t_{minor} = 8.2 min.

(*S_a*)-1-(3-Bromo-6-methoxy-2-vinylphenyl)-6-chloro-2-methoxyphenanthrene ((*S_a*)-16k):

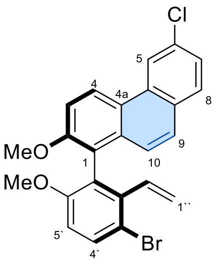

(S_a)-**16k**

Prepared according to the general procedure **L** using 1-bromo-8-(5-chloro-2-vinylphenyl)-4,5-dimethoxyphenanthrene **15k** (31.8 mg, 70.0 µmol) to give the title compound (S_a)-**16k** as a beige solid (28.5 mg, 62.8 µmol, 90%, >99:1 e.r., m.p. 90.9 – 92.0°C): R_f 0.36 (cyclohexane/EtOAc 5:1); $[\alpha]_D^{20}$ = –11 (c 0.10, $CHCl_3$); ν_{max} (neat): 3005w, 2933w, 2837w, 1578w, 1254m, 1447m, 1274s, 1254s, 1203w, 1093m, 1065m, 1034m, 930m, 867w, 808m, 694w; ^1H NMR (500 MHz, $CDCl_3$) δ = 8.62 (1H, d, ^3J 9.0 Hz, C4*H*), 8.58 (1H, d, ^4J 1.9 Hz, C5*H*), 7.73 (1H, d, ^3J 8.5 Hz, C8*H*), 7.66 (1H, d, ^3J 8.8 Hz, C4`*H*), 7.53 (1H, d, ^3J 9.1 Hz, C9*H*), 7.47 (1H, dd, ^3J 8.5, 1.9 Hz, C7*H*), 7.40 (1H, d, ^3J 9.0 Hz, C3*H*), 7.17 (1H, dd, ^3J 9.1, ^5J 0.6 Hz, C10*H*), 6.87 (1H, d,

^{3}J 8.8 Hz, C5`*H*), 6.37 (1H, dd, ^{3}J 17.7, ^{3}J 11.6 Hz, C2``*H*), 4.91 (1H, dd, $^{3}J$ 11.6, $^{2}J$ 1.6 Hz, C1``*H*), 4.85 (1H, dd, ^{3}J 17.7, ^{2}J 1.6 Hz, C1``*H*), 3.88 (3H, s, C2–OC$H_3$), 3.62 (3H, s, C6`–OC$H_3$); $^{13}$C NMR (126 MHz, $CDCl_3$) δ = 157.3 (*C6`*), 155.7 (*C2*), 139.8 (*C2`*), 134.8 (*C2``*), 132.9 (*C4`*), 132.8 (*C6*), 132.4 (*C10a*), 132.0 (*C4b*), 130.0 (*C8*), 129.2 (*C8a*), 127.0 (*C9*), 126.3 (*C7*), 126.1 (*C1`*), 124.5 (*C10*), 124.0 (*C4*), 123.7 (*C4a*), 122.14 (*C1*), 122.11 (*C5*), 120.1 (*C1``*), 114.7 (*C3`*), 112.4 (*C3*), 111.7 (*C5`*), 56.4 (C2–O*C*H_3), 56.3 (C6`–O*C*H_3); ESI-MS: m/z calcd. for $C_{24}H_{19}BrClO_2$ 453.0251 found 453.0232 [M+H$^+$]. The e.r. of the isolated product was determined by HPLC on a chiral stationary phase (Chiralcel IG 3 µm, 250x 4.6 mm, 1.0 mL/min, heptane/*i*PrOH 90 : 10, 20°C): t_{major} = 5.8 min and t_{minor} = 6.0 min.

Atroposelective Aromatic Ring-Opening Metathesis – Twofold RCM

Optimization Studies (15.0 µmol scale) of 17c: Performed according to the general procedure **H** utilizing 4-methoxy-5-methyl-1,8-bis(2-vinylphenyl)phenanthrene **17c** (3.20 mg, 7.50 µmol) and the corresponding solvent (0.25 mL, 0.03 mol·L^{-1}) with the reaction time of 18 h.

Supplementary Table 13. Reaction conditions optimization for atroposelective phenanthrene ring-opening metathesis and RCM

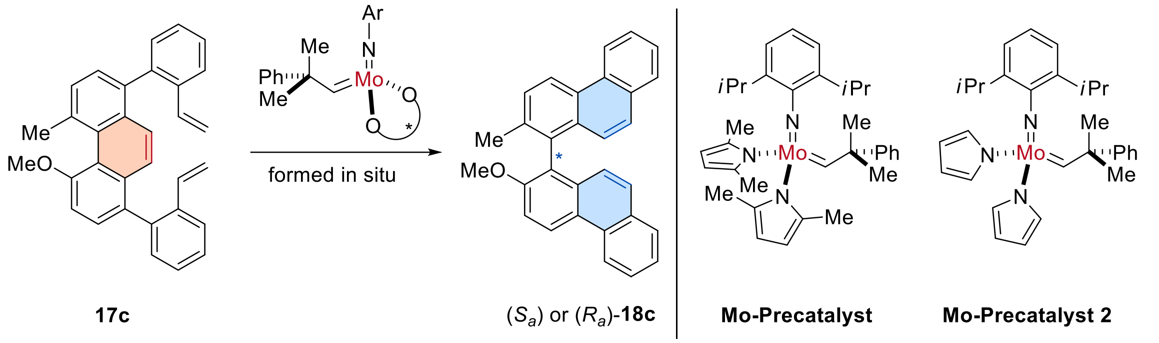

17c (*S*a) or (*R*a)-**18c** **Mo-Precatalyst** **Mo-Precatalyst 2**

Entry[a]	Catalyst (mol%)	Ligand (mol%)	Solvent	Temp. [°C]	Conversion[b] [%]	Yield[b] [%]	e. r.[c]
1	**C1** (20)	–	Toluene	85	44	37	50 : 50
2	**Mo-Precat.** (20)	(*S*a)-**L3** (60)	Toluene	85	54	42	> 1 : 99
3	**Mo-Precat.** (30)	(*R*a)-**L4** (90)	Toluene	85	76	45	> 99 : 1
4	**Mo-Precat.** (20)	(*R*a)-**L4** (60)	Toluene	85	78	68	> 99 : 1
5	**Mo-Precat.** (10)	(*R*a)-**L4** (30)	Toluene	85	NR	–	–
6[d]	**Mo-Precat.** (20)	(*R*a)-**L4** (60)	Toluene	85	52	47	> 99 : 1
7	**Mo-Precat. 2** (20)	(*R*a)-**L4** (60)	Toluene	85	69	59	> 99 : 1
8[e]	**Mo-Precat.** (20)	(*R*a)-**L4** (60)	Toluene	85	30	24	> 99 : 1
9	**Mo-Precat.** (20)	(*R*a)-**L4** (60)	Toluene	100	56	53	> 99 : 1
10	**Mo-Precat.** (20)	(*R*a)-**L1** (60)	Toluene	85	75	66	> 99 : 1

[a]Reactions were performed on 7.50 µmol scale of **17c** for 18 h with the solvent concentration of 0.03 mol·L^{-1}. [b]Conversion and yield were determined by ^{1}H-NMR with durene as an internal standard. [c]Determined by HPLC on a chiral stationary phase of the crude product (Chiralpak IG-N3 column (3 µm, 250x4.6 mm, heptane/*i*PrOH 85:15, 1.0 mL/min, 20°C). [d]48 h reaction time. [e]Concentration = 0.015 mol·L^{-1}.

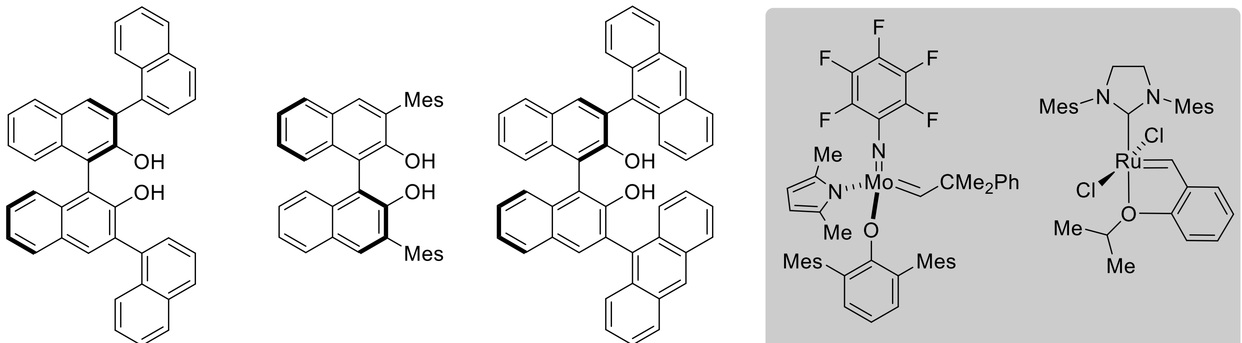

(*R*a)-**L1** (*S*a)-**L3** (*R*a)-**L4** **C1** **C4**

Supplementary Table 14. Reaction conditions optimization for atroposelective phenanthrene ring-opening metathesis and RCM

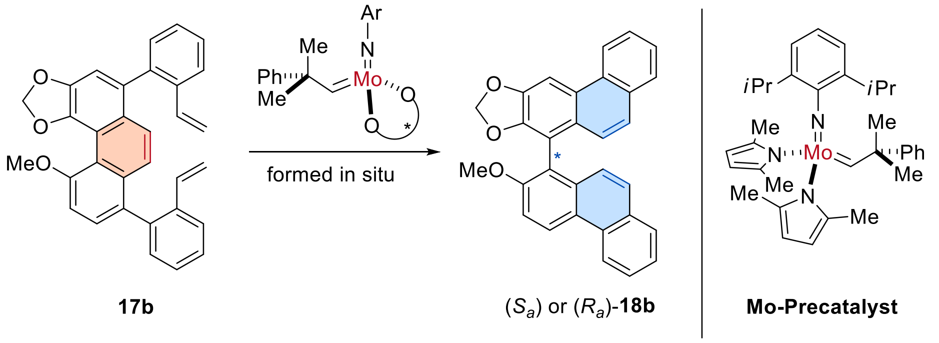

Entry[a]	Catalyst (mol%)	Ligand (mol%)	Solvent	Temp. [°C]	Conversion[b] [%]	Yield[b] [%]	e. r.[c]
1	**C4** (10)	–	Toluene	65	NR	–	–
2	**C4** (20)	–	Toluene	85	NR	–	–
3	**C1** (10)	–	Toluene	65	NR	–	–
4	**C1** (20)	–	Toluene	85	53		50 : 50
5	**Mo-Precat.** (20)	(S_a)-**L3** (60)	Toluene	85	13	11	> 99 : 1
6	**Mo-Precat.** (20)	(R_a)-**L4** (60)	Toluene	85	54	45	> 1 : 99
7[d]	**Mo-Precat.** (20)	(R_a)-**L4** (60)	Toluene	85	52	48	> 1 : 99
8	**Mo-Precat.** (30)	(R_a)-**L4** (90)	Toluene	85	88	79	2 : 98
9	**Mo-Precat.** (20)	(R_a)-**L1** (60)	Toluene	85	>95	80	> 1 : 99

[a]Reactions were performed on 7.50 µmol scale of **17b** for 18 h with the solvent concentration of 0.03 mol·L^{-1}. [b]Conversion and yield were determined by ^{1}H-NMR with durene as an internal standard. [c]Determined by HPLC on a chiral stationary phase of the crude product (Chiralpak IC-N3 column (3 µm, 250x4.6 mm, heptane/*i*PrOH 80:20, 1.0 mL/min, 20°C). [d]48 h reaction time.

General procedure M for atroposelective aromatic ring-opening metathesis – twofold RCM cascade:

Mo-precursor (4.14 mg, 7.00 µmol, 10 mol%), (*R*$_a$)-[1,2':4',1":3",1"'-quaternaphthalene]-2",3'-diol (11.3 mg, 21.0 µmol, 30 mol%) were weighed in 10mL crimp cap vial and toluene (1.5 mL) was added. The obtained solution was stirred for 1 h at room temperature in an argon-filled glovebox before a solution of the corresponding substrate (70.0 µmol, 1.00 eq.) in toluene (0.83 mL) was added. The capped vial was then taken out of the glovebox and stirred at 85°C for 18 h. The reaction mixture was diluted with EtOAc and filtered through a thin layer of silica gel. The solvents were removed under reduced pressure and the residue was purified by silica gel column chromatography (cyclohexane/EtOAc 100:0→80:20) to give the desired product. The e.r. of the isolated products were determined by HPLC on a chiral stationary phase.

(*S*$_a$)-2,2'-Dimethoxy-1,1'-biphenanthrene ((*S*$_a$)-18a):

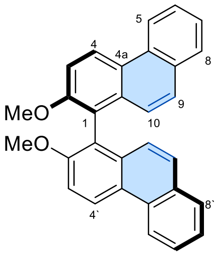

(*S*$_a$)-**18a**

Prepared according to the general procedure **M** using 4,5-dimethoxy-1,8-bis(2-vinylphenyl)phenanthrene **17a** (31.0 mg, 70.0 µmol) to give the title compound (*S*$_a$)**-18a** as a white solid (26.0 mg, 62.7 µmol, 90%, >99:1 e.r., m.p. 210.0 – 212.0°C (decomposition)): R$_f$ 0.36 (cyclohexane/EtOAc 5:1); $[\alpha]_D^{20}$ = –77 (c 0.19, CHCl$_3$); ν_{max} (neat): 3058w, 2995w, 2934w, 2837w, 1590w, 1527w, 1461m, 1377w, 1274s, 1256s, 1201w, 1086w,, 945w, 865w, 787m, 752m; ^{1}H NMR (500 MHz, CDCl$_3$) δ = 8.89 – 8.82 (2H, m, C4*H*, C4`*H*), 8.71 (2H, d, $^3J$ 8.4 Hz, C5*H*, C5`*H*), 7.78 (2H, ^3J 7.9, ^4J 1.4 Hz, C8*H*, C8`*H*), 7.65 (2H, ddd, $^3J$ 8.4, $^3J$ 7.0, $^4J$ 1.4 Hz, C6*H*, C6`*H*), 7.55 (2H, d, ^3J 9.2 Hz, C3*H*, C3`*H*), 7.52 (2H, ddd, $^3J$ 8.0, $^3J$ 7.0, $^4J$ 1.1 Hz, C7*H*, C7`*H*), 7.46 (2H, d, ^3J 9.2 Hz, C9*H*, C9`*H*), 7.07 (2H, dd, $^3J$ 9.2, $^5J$ 0.7 Hz, C10*H*, C10`*H*), 3.82 (6H, s, 2 × OC*H*$_3$); ^{13}C NMR (126 MHz, CDCl$_3$) δ = 156.2 (*C2*, *C2*`), 133.0 (*C10a*, *C10a*`), 131.0 (*C8a*, *C8a*`), 130.8 (*C4b*, *C4b*`), 128.6 (*C8*, *C8*`), 127.7 (*C9*, *C9*`), 126.8 (*C6*, *C6*`), 125.9 (*C7*, *C7*`) 125.1 (*C1*, *C1*`), 124.6 (*C10*, *C10*`), 124.1 (*C4*, *C4*`), 122.4 (*C5*, *C5*`), 121.5 (*C4a*, *C4a*`), 112.8 (*C3*, *C3*`), 56.8 (O*C*H$_3$); ESI-MS: m/z calcd. for $C_{30}H_{22}AgO_2$ 521.0665 found 521.0659 [M+Ag$^+$]. The e.r. of the isolated product was determined by HPLC on a chiral stationary phase (Chiralcel IG 3 µm, 250x 4.6 mm, 1.0 mL/min, heptane/*i*PrOH 90 : 10, 20°C): t_{major} = 12.6 min and t_{minor} = 14.8 min.

Scale-up (5 mol% catalyst loading)): Performed according to the modified general procedure **M** using 4,5-dimethoxy-1,8-bis(2-vinylphenyl)phenanthrene **17a** (111 mg, 250 µmol), Mo-precursor (7.40 mg, 12.5 µmol, 5.0 mol%), and (*R*$_a$)-[1,2':4',1":3",1"'-quaternaphthalene]-2",3'-diol (13.5 mg, 25.0 µmol, 10 mol%) with the reaction time of 18 h to give the title compound **18a** as a beige solid (100 mg, 241 µmol, 97%).

(*S*$_a$)-7-(2-Methoxyphenanthren-1-yl)phenanthro[2,3-d][1,3]dioxole ((*S*$_a$)-18b):

Prepared according to the general procedure **M** using 11-methoxy-5,8-bis(2-vinylphenyl)phenanthro[3,4-d][1,3]dioxole **17b** (32.0 mg, 70.0 µmol) to give the title compound (*S*$_a$)**-18b** as a beige solid (24.2 mg, 56.5 µmol, 81%, >99:1 e.r., m.p. 266.0 – 267.0°C (decomposition)): R$_f$ 0.36 (cyclohexane/EtOAc 6:1); $[\alpha]_D^{20}$ = –82 (c

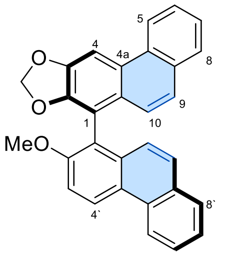

(S_a)-**18b**

0.18, $CHCl_3$); ν_{max}(neat): 3063w, 3010w, 2928w, 2852w, 1592w, 1454s, 1393m, 1258s, 1233s, 1059m, 952m, 815m, 750s; ^{1}H NMR (500 MHz, $CDCl_3$) δ = 8.88 (1H, d, ^3J 9.1 Hz, C4`*H*), 8.70 (1H, d, $^3J$ 8.1 Hz, C5`*H*), 8.59 (1H, d, ^3J 8.2 Hz, C5*H*), 8.21 (1H, s, C4*H*), 7.84 – 7.77 (2H, m, C8*H*, C8`*H*), 7.72 – 7.60 (2H, m, C6*H*, C6`*H*), 7.58 – 7.50 (4H, m, C7*H*, C3`*H*, C7`*H*, C9`*H*), 7.45 (1H, d, $^3J$ 9.0 Hz, C9*H*), 7.21 (1H, d, $^3J$ 9.2 Hz, C10`*H*), 7.16 (1H, d, ^3J 9.0 Hz, C10*H*), 6.07 (2H, dd, ^2J 17.7, ^5J 1.3 Hz, OC*H*$_2$O), 3.87 (3H, s, OC*H*$_3$); ^{13}C NMR (126 MHz, $CDCl_3$) δ = 156.3 (*C2`*), 147.8 (*C3*), 146.2 (*C2*), 132.7 (*C10a`*), 131.5 (*C8a*), 131.0 (*C8a`*), 130.8 (*C4b`*), 130.5 (*C4b*), 128.7 (*C8`*), 128.6 (*C8*), 128.29 (*C10a*), 128.25 (*C10`*), 126.98 (*C6`*), 126.96 (*C4a*), 126.4 (*C6*), 126.1 (*C7`*), 125.9 (*C7*), 125.4 (*C9*), 125.2 (*C4a`*), 125.0 (*C4`*), 124.3 (*C10*), 124.2 (*C10`*), 122.8 (*C5*), 122.4 (*C5`*), 118.4 (*C1`*), 113.7 (*C1*), 112.5 (*C3`*), 101.6 (O*C*H$_2$O), 100.7 (*C4*), 56.7 (O*C*H$_3$); ESI-MS: m/z calcd. for $C_{30}H_{20}AgO_3$ 535.0458 found 535.0453 [M+Ag$^+$]. The e.r. of the isolated product was determined by HPLC on a chiral stationary phase (Chiralcel IC 3 µm, 250x 4.6 mm, 1.0 mL/min, heptane/*i*PrOH 80 : 20, 20°C): t_{major} = 26.2 min and t_{minor} = 12.9 min.

(*R*$_a$)-2-Methoxy-2'-methyl-1,1'-biphenanthrene ((*R*$_a$)-18c):

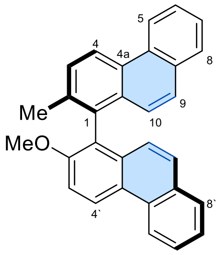

(R_a)-**18c**

Prepared according to the general procedure **M** using 4-methoxy-5-methyl-1,8-bis(2-vinylphenyl)phenanthrene **17c** (29.9 mg, 70.0 µmol) to give (S_a)-**18c** the title compound as a white solid (27.3 mg, 68.5 µmol, 98%, >99:1 e.r., m.p. 209.0 – 210.5°C): R$_f$ 0.57 (cyclohexane/EtOAc 13:2); $[\alpha]_D^{20}$ = –25 (c 0.09, $CHCl_3$); ν_{max}(neat): 3039w, 2924w, 2848w, 1589w, 1527w, 1459m, 1377w, 1273m, 1260m, 1200w, 1081m, 947w, 865w, 814s, 749s; ^{1}H NMR (500 MHz, $CDCl_3$) δ = 8.86 (1H, d, ^3J 9.2 Hz, C4`*H*), 8.82 – 8.68 (3H, m, C4*H*, C5*H*, C5`*H*), 7.82 – 7.76 (2H, m, C8*H*, C8`*H*), 7.70 (1H, d, $^3J$ 8.5 Hz, C3*H*), 7.69 – 7.64 (2H, m, C6*H*, C6`*H*), 7.59 – 7.50 (3H, m, C7*H*, C3`*H*, C7`*H*), 7.48 – 7.43 (2H, m, C9*H*, C9`*H*), 7.10 (1H, d, $^3J$ 9.2 Hz, C10*H*), 6.96 (1H, d, $^3J$ 9.2 Hz, C10`*H*), 3.82 (3H, s, OC*H*$_3$), 2.14 (3H, s, C2–C*H*$_3$); ^{13}C NMR (126 MHz, $CDCl_3$) δ = 155.6 (*C2`*), 136.2 (*C2*), 133.9 (*C1*), 132.5 (*C10a`*), 131.8 (*C10a*), 131.6 (*C8a*), 131.0 (*C8a`*), 130.8 (*C4b`*), 129.0 (*C3*), 128.8 (*C4a*), 128.7 (*C8*), 128.5 (*C8`*), 128.0 (*C9*), 127.0 (*C9`*), 126.9 (*C6`*), 126.6 (*C6*/*C7*), 126.3 (*C6*/*C7*), 126.0 (*C7`*), 125.1 (*C4a`*), 125.0 (*C10*), 124.3 (*C10`*), 124.1 (*C4`*), 124.0 (*C1`*), 122.8 (*C5*), 122.4 (*C4*), 122.3 (*C5`*), 112.4 (*C3`*), 56.5 (O*C*H$_3$), 20.5 (C2–*C*H$_3$); ESI-MS: m/z calcd. for $C_{30}H_{22}AgO$ 505.0716 found 505.0709 [M+Ag$^+$]. The e.r. of the isolated product was determined by HPLC on a chiral stationary phase (Chiralcel IG 3 µm, 250x 4.6 mm, 1.0 mL/min, heptane/*i*PrOH 85 : 15, 20°C): t_{major} = 6.0 min and t_{minor} = 7.3 min.

(*S*$_a$)-2,2'-Dimethyl-1,1'-biphenanthrene ((*S*$_a$)-18d):

Prepared according to the general procedure **M** using 4,5-dimethyl-1,8-bis(2-vinylphenyl)phenanthrene **17d** (28.7 mg, 70.0 µmol) to give the title compound (S_a)-**18d** as a white solid (22.2 mg, 58.0 µmol, 83%,

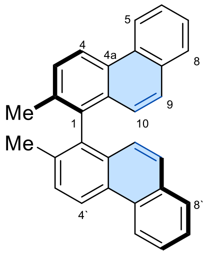

(S_a)-**18d**

>99:1 e.r., m.p. 89.0 – 89.8°C): R_f 0.79 (cyclohexane/EtOAc 5:1); $[\alpha]_D^{20}$ = –26 (c 0.11, $CHCl_3$); ν_{max}(neat): 3054w, 3003w, 2941w, 2915w, 2859w, 1618w, 1523w, 1458w, 1303w, 1260w, 1229w, 1101w, 1038w, 968w, 864w, 816m, 752s; ^{1}H NMR (500 MHz, $CDCl_3$) δ = 8.79 (2H, d, ^3J 8.3 Hz, C5*H*, C5`*H*), 8.77 (2H, d, $^3J$ 8.5 Hz, C4*H*, C4`*H*), 7.81 (2H, dd, ^3J 7.9, ^4J 1.2 Hz, C8*H*, C8`*H*), 7.73 – 7.65 (4H, m, C3*H*, C3`*H*, C6*H*, C6`*H*), 7.57 (2H, ddd, $^3J$ 7.9, $^3J$ 7.1, $^4J$ 1.1 Hz, C7*H*, C7`*H*), 7.47 (2H, d, ^3J 9.1 Hz, C9*H*, C9`*H*), 7.03 (2H, d, $^3J$ 9.1 Hz, C10*H*,C10`*H*), 2.09 (6H, s, C2–CH_3, C2`–C$H_3$); $^{13}$C NMR (126 MHz, $CDCl_3$) δ = 136.7 (*C1*, *C1*`), 135.4 (*C2*, *C2*`), 131.6 (*C8a*, *C8a*`), 131.3 (*C10a*, *C10a*`), 130.7 (*C4b*, *C4b*`), 129.1 (*C3*, *C3*`), 128.9 (*C4a*, *C4a*`), 128.6 (*C8*, *C8*`), 127.3 (*C9*, *C9*`), 126.7 (*C6*, *C6*`), 126.5 (*C7*, *C7*`), 124.7 (*C10*, *C10*`), 122.8 (*C5*, *C5*`), 122.3 (*C4*, *C4*`), 20.3 (C2–$CH_3$, C2`–CH_3); ESI-MS: m/z calcd. for $C_{30}H_{22}Ag$ 489.0772 found 489.0767 [M+Ag$^+$]. The e.r. of the isolated product was determined by HPLC on a chiral stationary phase (Chiralcel IG 3 µm, 250x 4.6 mm, 1.0 mL/min, heptane/*i*PrOH 99 : 1, 20°C): t_{major} = 7.3 min and t_{minor} = 8.7 min.

(*S*$_a$)-6-Chloro-2,2'-dimethoxy-1,1'-biphenanthrene ((*S*$_a$)-18e):

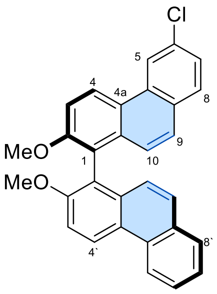

(S_a)-**18e**

Prepared according to the general procedure **M** using 1-(5-chloro-2-vinylphenyl)-4,5-dimethoxy-8-(2-vinylphenyl)phenanthrene **17e** (33.4 mg, 70.0 µmol) to give the title (*S*$_a$)-**18e** compound as a beige solid (20.0 mg, 44.5 µmol, 64%, >99:1 e.r., m.p. 227.0 – 228.4°C): R_f 0.41 (cyclohexane/EtOAc 5:1); $[\alpha]_D^{19}$ = –93 (c 0.417, $CHCl_3$); ν_{max}(neat): 3061w, 3004w, 2952w, 2838w, 1616w, 1589m, 1517m, 1459m, 1271s, 1254s, 1199w, 1086s, 1011m, 906m, 868m, 815m, 729s; ^{1}H NMR (500 MHz, $CDCl_3$) δ = 8.85 (1H, d, ^3J 9.0 Hz, C4`*H*), 8.75 (1H, d, $^3J$ 9.0 Hz, C4*H*), 8.71 (1H, d, $^3J$ 8.0 Hz, C5`*H*), 8.66 (1H, d, ^4J 2.0 Hz, C5*H*), 7.78 (1H, dd, ^3J 7.9, ^4J 1.2 Hz, C8`*H*), 7.70 (1H, d, $^3J$ 8.4 Hz, C8*H*), 7.66 (1H, ddd, $^3J$ 8.0, $^3J$ 7.0, $^4J$ 1.4 Hz, C6`*H*), 7.57 – 7.50 (3H, m, C3*H*, C3`*H*, C7`*H*), 7.49 – 7.45 (2H, m, C7*H*, C9`*H*), 7.41 (1H, d, $^3J$ 9.1 Hz, C9*H*), 7.10 – 7.02 (2H, m, C10*H*, C10`*H*), 3.82 (6H, s, C2–OCH_3, C2`–OC$H_3$); $^{13}$C NMR (126 MHz, $CDCl_3$) δ = 156.6 (*C2*), 156.1 (*C2*`), 133.2 (*C10a*), 132.9 (*C10a*`), 132.7 (*C6*), 132.0 (*C4b*), 131.0 (*C8a*`), 130.8 (*C4b*`), 130.0 (*C8*), 129.3 (*C8a*), 128.6 (*C8*`), 127.8 (*C9*`), 127.0 (*C9*), 126.8 (*C6*`), 126.3 (*C7*), 125.9 (*C7*`), 125.1 (*C4a*`), 124.9 (*C10*), 124.4 (*C10*`), 124.2 (*C4*/*C4*`), 124.1 (*C4a*), 122.4 (*C5*`), 122.1 (*C5*), 121.5 (*C1*), 121.1 (*C1*`), 112.9 (*C3*/*C3*`), 112.7 (*C3*/*C3*`), 56.72 (C2–OCH_3/C2`–OC$H_3$), 56.69 (C2–OC$H_3$/C2`–OCH_3); ESI-MS: m/z calcd. for $C_{30}H_{22}ClO_2$ 449.1303 found 449.1306 [M+H$^+$]. The e.r. of the isolated product was determined by HPLC on a chiral stationary phase (Chiralcel IG 3 µm, 250x 4.6 mm, 1.0 mL/min, heptane/*i*PrOH 90 : 10, 20°C): t_{major} = 11.4 min and t_{minor} = 14.1 min.

(S_a)-6,6'-Difluoro-2,2'-dimethoxy-1,1'-biphenanthrene ((S_a)-18f):

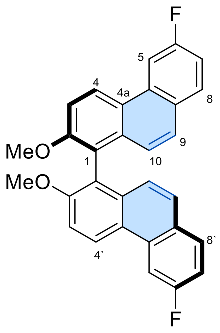

(S_a)-**18f**

Prepared according to the general procedure **M** using 1,8-bis(5-fluoro-2-vinylphenyl)-4,5-dimethoxyphenanthrene **17f** (33.5 mg, 70.0 µmol) to give the title compound (S_a)-**18f** as a beige solid (28.6 mg, 63.5 µmol, 91%, >99:1 e.r., m.p. 235.0 – 237.0°C): R_f 0.45 (cyclohexane/EtOAc 3:1); $[\alpha]_D^{19}$ = –73 (c 0.10, $CHCl_3$);; ν_{max} (neat): 2968w, 2964w, 2838w, 1627m, 1591m, 1529m, 1497s, 1459m, 1379w, 1268s, 1199s, 1082s, 905m, 835s, 730m; ^{1}H NMR (500 MHz, $CDCl_3$) δ = 8.70 (2H, d, ^3J 9.2 Hz, C4*H*, C4`*H*), 8.30 (2H, dd, $^3J_{HF}$ 11.4, $^4J_{HH}$ 2.4 Hz, C5*H*, C5`*H*), 7.75 (2H, dd, $^3J_{HH}$ 8.7, $^3J_{HF}$ 6.0 Hz, C8*H*, C8`*H*), 7.54 (2H, d, $^3J$ 9.2 Hz, C3*H*, C3`*H*), 7.44 (d, ^3J 9.2 Hz, C9*H*, C9`*H*), 7.31 – 7.25 (2H, m, C7*H*, C7`*H*), 7.01 (2H, d, ^3J 9.2 Hz, C10*H*, C10`*H*), 3.82 (6H, s, 2 × OC$H_3$); $^{13}$C NMR (126 MHz, $CDCl_3$) δ = 161.9 (d, $^1J_{CF}$ 245.0 Hz, *C6*, *C6`*), 156.6 (*C2*, *C2`*), 133.2 (*C10a*, *C10a`*), 132.4 (d, $^3J_{CF}$ 8.6 Hz, *C4b*, *C4`b*), 130.6 (d, $^3J_{CF}$ 9.1 Hz, *C8*, *C8`*), 127.7 (d, $^4J_{CF}$ 1.4 Hz, *C8a*, *C8a`*), 127.1 (*C9*, *C9`*), 124.5 (*C4a*, *C4a`*), 124.31 (*C4*, *C4`*), 123.7 (*C10*, *C10`*), 121.3 (*C1*, *C1`*), 114.8 (d, $^2J_{CF}$ 23.9 Hz, *C7*, *C7`*), 112.7 (*C3*, *C3`*), 107.5 (d, J = 22.3 Hz, *C5*, *C5`*), 56.7 (O*C*H_3); ^{19}F NMR (376 MHz, $CDCl_3$) δ = –113.59; ESI-MS: m/z calcd. for $C_{30}H_{20}AgF_2O_2$ 557.0477 found 557.0469 [M+Ag^+]. The e.r. of the isolated product was determined by HPLC on a chiral stationary phase (Chiralcel IG 3 µm, 250x 4.6 mm, 1.0 mL/min, heptane/*i*PrOH 90 : 10, 20°C): t_{major} = 9.6 min and t_{minor} = 13.7 min.

(R_a)-2,6,6'-Trimethoxy-2'-methyl-1,1'-biphenanthrene ((S_a)-18g):

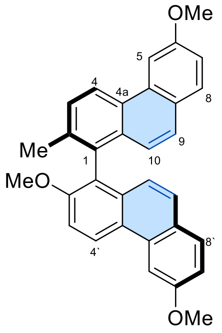

(R_a)-**18g**

Prepared according to the general procedure **M** using 4-methoxy-1,8-bis(5-methoxy-2-vinylphenyl)-5-methylphenanthrene **17g** (34.1 mg, 70.0 µmol) to give the title compound (S_a)-**18g** as a beige solid (30.5 mg, 66.5 µmol, 95%, >99:1 e.r., 99.5 – 101.0°C): R_f 0.40 (cyclohexane/EtOAc 3:1); $[\alpha]_D^{19}$ = –149 (c 0.17, $CHCl_3$); ν_{max}(neat): 2992w, 2931w, 2837w, 1617s, 1500s, 1458m, 1374w, 1260s, 1230s, 1175w, 1079m, 1036m, 959w, 907m, 834s, 730s, 651w; ^{1}H NMR (500 MHz, $CDCl_3$) δ = 8.76 (1H, d, ^3J 9.2 Hz, C4`*H*), 8.66 (1H, d, $^3J$ 8.6 Hz, C4*H*), 8.14 (1H, d, $^4J$ 2.4 Hz, C5*H*), 8.09 (1H, d, $^4J$ 2.3 Hz, C5`*H*), 7.73 – 7.68 (2H, m, C8*H*, C8`*H*), 7.67 (1H, d, $^3J$ 8.5 Hz, C3*H*), 7.52 (1H, d, $^3J$ 9.2 Hz, C3`*H*), 7.43 – 7.36 (2H, m, C9*H*, C9`*H*), 7.22 – 7.15 (2H, m, C7*H*, C7`*H*), 6.97 (1H, d, ^3J 9.1 Hz, C10*H*), 6.82 (1H, d, ^3J 9.1 Hz, C10`*H*), 4.06 (3H, s, C6`–OCH_3), 4.05 (3H, s, C6–OCH_3), 3.81 (3H, s, C2`–OC$H_3$), 2.14 (3H, s, C2–C$H_3$); $^{13}$C NMR (126 MHz, $CDCl_3$) δ = 158.8 (*C6*), 158.6 (*C6`*), 155.7 (*C2`*), 136.2 (*C2*), 134.0 (*C1*), 132.9 (*C10a`*), 132.12 (*C4b*/*C10a*/*C4b`*), 132.08 (*C4b*/*C10a*/*C4b`*), 132.05 (*C4b*/*C10a*/*C4b`*), 130.1 (*C8`*), 129.9 (*C8*), 128.6 (*C3*), 128.2 (*C8a*), 127.6 (*C9`*), 126.6 (*C9*), 126.4 (*C4a*), 125.8 (*C8a`*), 124.6 (*C4a`*), 124.10 (*C1`*), 124.06 (*C4`*), 122.7 (*C10*), 122.3 (*C4*), 122.0 (*C10`*), 116.4 (*C7*), 115.9 (*C7`*), 112.1 (*C3`*), 104.14 (*C5*), 104.06 (*C5`*), 56.5 (C2`–O*C*H_3), 55.72 (C6–O*C*H_3/(C6`–O*C*$H_3$), 55.70 (C6–O*C*$H_3$/(C6`–O*C*H_3), 20.5 (C2–*C*H_3); ESI-MS: m/z calcd. for $C_{32}H_{26}AgO_3$ 565.0927 found 565.0924 [M+Ag^+]. The e.r. of the isolated product was determined by HPLC on a chiral stationary phase

(Chiralcel IG 3 µm, 250x 4.6 mm, 1.0 mL/min, heptane/*i*PrOH 90 : 10, 20°C): t_{major} = 13.5 min and t_{minor} = 16.2 min.

(S_a)-2,2'-Dimethoxy-6,6'-bis(trifluoromethyl)-1,1'-biphenanthrene ((S_a)-18h):

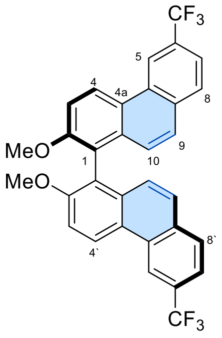

(S_a)-**18h**

Prepared according to the general procedure **M** using 4,5-dimethoxy-1,8-bis(5-(trifluoromethyl)-2-vinylphenyl)phenanthrene **17h** (31.8 mg, 55.0 µmol), Mo-precursor (3.25 mg, 5.50 µmol, 10 mol%), (R_a)-[1,2':4',1":3",1"'-quaternaphthalene]-2",3'-diol (8.90 mg, 16.5 µmol, 30 mol%) to give the title compound (S_a)-**18h** as a white solid (14.5 mg, 26.3 µmol, 48%, >99:1 e.r., m.p. 250 – 253°C (decomposition)): R_f 0.43 (cyclohexane/EtOAc 5:1); $[\alpha]_D^{20}$ = –82 (c 0.47, $CHCl_3$); ν_{max}(neat): 3002w, 2962w, 2918w, 2848w, 1733w, 1620w, 1598w, 1527w, 1462w, 1413m, 1376w, 1320s, 1284s, 1262s, 1162s, 1115s, 1071s, 1020m, 907m, 844s, 731s, 647w, 615w; ^{1}H NMR (500 MHz, $CDCl_3$) δ = 8.97 (2H, s, C5*H*, C5`*H*), 8.87 (2H, d, $^3J$ 9.0 Hz, C4*H*, C4`*H*), 7.89 (2H, d, ^3J 8.3 Hz, C8*H*, C8`*H*), 7.73 (2H, dd, $^3J$ 8.3, $^4J$ 1.4 Hz, C7*H*, C7`*H*), 7.61 (2H, d, ^3J 9.0 Hz, C3*H*, C3`*H*), 7.51 (2H, d, $^3J$ 9.2 Hz, C9*H*, C9`*H*), 7.17 (2H, d, ^3J 9.2 Hz, C10*H*, C10`*H*), 3.84 (6H, s, OC$H_3$); $^{13}$C NMR (126 MHz, $CDCl_3$) δ = 156.7 (*C2*, *C2`*), 133.2 (*C10a*, *C10a`*), 132.8 (*C8a*, *C8a`*), 130.3 (*C4b*, *C4b`*), 129.3 (*C8*, *C8`*), 128.6 (q, $^2J_{CF}$ 32.0 Hz, *C6*, *C6`*), 127.1 (*C9*, *C9`*), 126.7 (*C10*, *C10`*), 124.9 (q, $^1J_{CF}$ 271.2 Hz, *C*$F_3$), 124.8 (*C4a*, *C4a`*), 124.4 (*C4*, *C4`*), 121.8 (q, $^3J_{CF}$ 3.4 Hz, *C7*, *C7`*), 121.2 (*C1*, *C1`*), 120.0 (q, $^3J_{CF}$ 4.5 Hz, *C5*, *C5`*), 113.2 (*C3*, *C3`*), 56.7 (O*C*H_3); ^{19}F NMR (376 MHz, $CDCl_3$) δ –61.69; ESI-MS: m/z calcd. for $C_{32}H_{20}AgF_3O_2$ 657.0413 found 657.0408 [M+Ag$^+$]. The e.r. of the isolated product was determined by HPLC on a chiral stationary phase (Chiralcel IG 3 µm, 250x 4.6 mm, 1.0 mL/min, heptane/*i*PrOH 90 : 10, 20°C): t_{major} = 6.0 min and t_{minor} = 6.9 min.

(S_a)-6,6'-Dichloro-2,2'-dimethoxy-1,1'-biphenanthrene ((S_a)-18i):

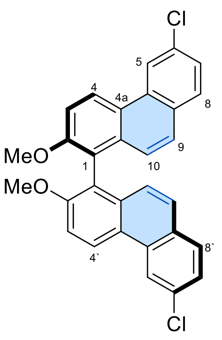

(S_a)-**18i**

Prepared according to the general procedure **M** using 1,8-bis(5-chloro-2-vinylphenyl)-4,5-dimethoxyphenanthrene **17i** (35.8 mg, 70.0 µmol) to give the title compound (S_a)-**18i** as a beige solid (31.0 mg, 64.1 µmol, 92%, >99:1 e.r., m.p. 280.0 – 282.0°C (decomposition)): R_f 0.39 (cyclohexane/EtOAc 5:1); $[\alpha]_D^{19}$ = –182 (c 0.20, $CHCl_3$); ν_{max}(neat): 3081w, 2958w, 2837w, 1730w, 1617w, 1589w, 1490m, 1468m, 1399w, 1285s, 1253s, 1181m, 1086s, 1019m, 907s, 870s, 837s, 729s, 649m; ^{1}H NMR (500 MHz, $CDCl_3$) δ = 8.75 (2H, d, ^3J 9.1 Hz, C4*H*, C4`*H*), 8.66 (2H, d, $^4J$ 1.7 Hz, C5*H*, C5`*H*), 7.70 (2H, d, ^3J 8.4 Hz, C8*H*, C8`*H*), 7.55 (2H, d, $^3J$ 9.1 Hz, C3*H*, C3`*H*), 7.47 (2H, dd, ^3J 8.4, ^4J 1.7 Hz, C7*H*, C7`*H*), 7.42 (2H, d, $^3J$ 9.2 Hz, C9*H*, C9`*H*), 7.05 (2H, d, ^3J 9.2 Hz, C10*H*, C10`*H*), 3.82 (6H, s, OC$H_3$); $^{13}$C NMR (126 MHz, $CDCl_3$) δ = 156.6 (*C2*, *C2`*), 133.2 (*C10a*, *C10a`*), 132.8 (*C6*, *C6`*), 132.0 (*C4b*, *C4b*), 130.0 (*C8*, *C8`*), 129.3 (*C8a*, *C8a`*), 127.1 (*C9*, *C9`*), 126.4 (*C7*, *C7`*), 124.8 (*C10*, *C10`*), 124.3 (*C4*, *C4`*), 124.1 (*C4*, *C4a`*), 122.1 (*C5*, *C5`*), 121.2 (*C1*, *C1`*), 112.9 (*C3*, *C3`*), 56.7 (O*C*H_3); ESI-MS: m/z calcd. for $C_{30}H_{20}AgCl_2O_2$ 588.9886 found 588.9897

[$M+Ag^+$]. The e.r. of the isolated product was determined by HPLC on a chiral stationary phase (Chiralcel IG 3 µm, 250x 4.6 mm, 1.0 mL/min, heptane/*i*PrOH 90 : 10, 20°C): t_{major} = 10.6 min and t_{minor} = 14.7 min.

Atroposelective Indole Ring-Opening Metathesis

Optimization Studies (7.50 µmol scale): Performed according to the general procedure **H** utilizing 7-methyl-1,4-bis(2-vinylphenyl)-1*H*-indole **19c** (2.52 mg, 7.50 µmol) and toluene (0.25 mL, 0.03 mol·L^{-1}) with the reaction time of 18 h.

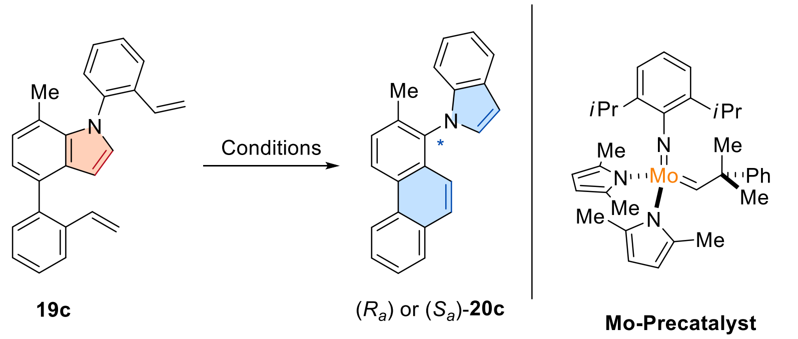

Supplementary Table 15. Reaction conditions optimization for atroposelective indole ring-opening metathesis and twofold RCM

Entry[a]	Catalyst (mol%)	Ligand (mol%)	Solvent	Temp. [°C]	Conversion[b] [%]	Yield[b] [%]	e.r.[c]
1	**C1** (20)	–	Toluene	65	56	46	50:50
2	**C2** (10)	–	Toluene	65	>95	68	50:50
3	**C3** (10)	–	Toluene	65	65	64	50:50
4	**C5** (10)	–	Toluene	65	13	11	93:7
5	**C5** (20)	–	Toluene	65	21	20	93:7
6	**Mo-Precat.** (10)	(S_a)-**L1** (30)	Toluene	65	>95	84	75:25
7	**Mo-Precat.** (20)	(S_a)-**L1** (60)	Toluene	65	>95	87	74:26
8	**Mo-Precat.** (20)	(S_a)-**L1** (60)	Toluene	50	>95	61	70:30
9	**Mo-Precat.** (20)	(R_a)-**L2** (60)	Toluene	65	>95	83	6:94
10	**Mo-Precat.** (20)	(S_a)-**L2** (60)	Toluene	rt	75	–	95:5
11	**Mo-Precat.** (20)	(S_a)-**L3** (60)	Toluene	65	>95	41	91:9

[a]Reactions were performed on 7.50 µmol scale of **19c** for 18 h. [b]Conversion and yield were determined by ^{1}H-NMR with durene as an internal standard. [c]Determined by HPLC on a chiral stationary phase of the crude product (Chiralpak IG-N3 column (3 µm, 250x4.6 mm, heptane/i-PrOH 96:4, 1.0 mL/min, 20°C).

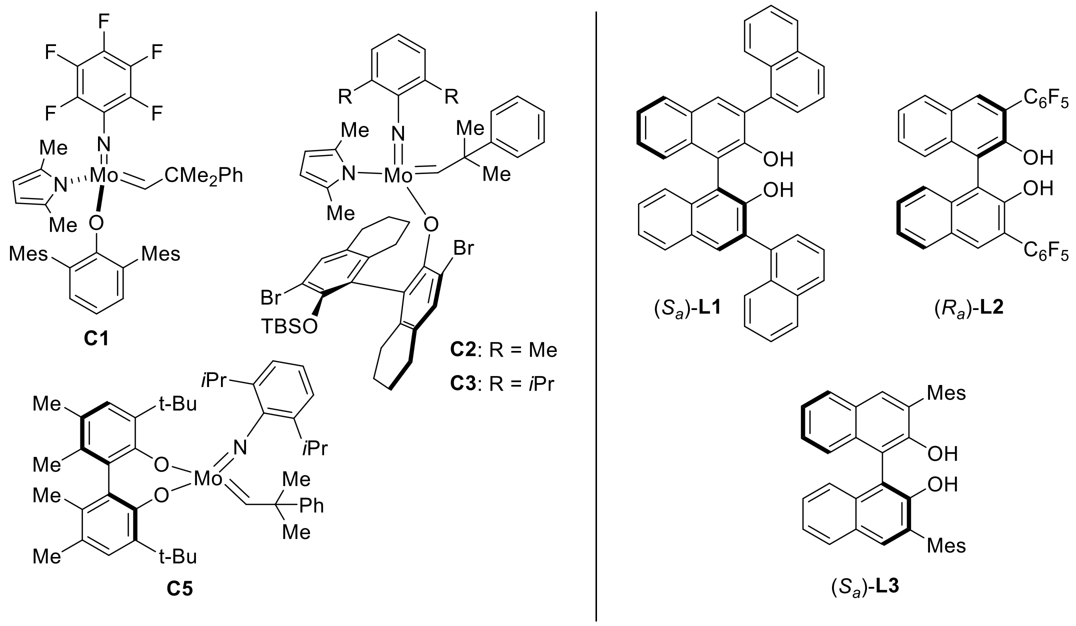

General Procedure N for atroposelective indole ring-opening metathesis:

Mo-precursor (4.14 mg, 7.00 µmol, 10%), (S_a)-3,3'-bis(perfluorophenyl)-[1,1'-binaphthalene]-2,2'-diol (S_a)-**L2** (13.0 mg, 21.0 µmol, 30 mol%) were weighed in 10mL crimp cap vial and toluene (1.5 mL) was added. The obtained solution was stirred for 1 h at room temperature in an argon-filled glovebox before a solution of the corresponding substrate (70.0 µmol, 1.00 eq.) in toluene (0.83 mL) was added. The capped vial was then taken out of the glovebox and stirred at 65°C for 18 h. The reaction mixture was diluted with EtOAc and filtered through a thin layer of silica gel. The solvents were removed under reduced pressure and the residue was purified by silica gel column chromatography (cyclohexane/EtOAc 100:0→80:20) to give the desired product. The e.r. of the isolated products were determined by HPLC on a chiral stationary phase.

(*R*$_a$)-2-Methyl-1-(phenanthren-1-yl)-1*H*-indole ((*R*$_a$)-20a):

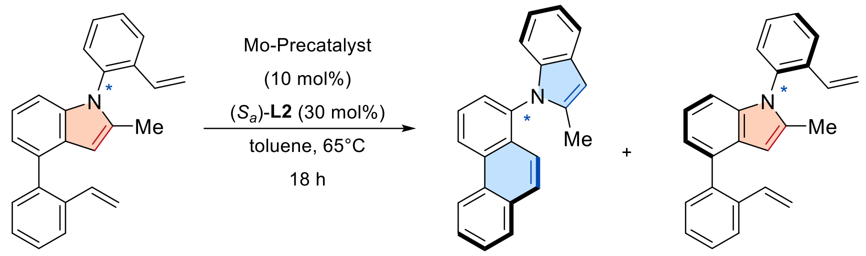

(±)-**19a**

(R_a)-**20a**
41%
87 : 13 e.r.

(S_a)-**19a**
37%
18 : 82 e.r.

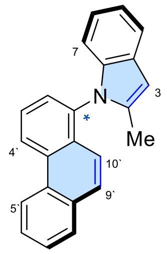

(R_a)-**20a**

Prepared according to the general procedure **N** using 2-methyl-1,4-bis(2-vinylphenyl)-1*H*-indole (±)-**19a** (23.5 mg, 70.0 µmol) to give the title compound (R_a)**-20a** as yellow oil (8.90 mg, 29.0 µmol, 41%, 87 : 13 e.r.) after preparative thin layer chromatography (pTLC, cyclohexane/EtOAc 5:1): R_f 0.55 (cyclohexane/EtOAc 5:1); $[\alpha]_D^{22}$ = – 86 (c 0.47, $CHCl_3$); ν_{max} (neat): 3052w, 2981w, 2917w, 2844w, 1599w, 1554w, 1467s, 1385m, 1321w, 1218w, 1159w; ^{1}H NMR (500 MHz, $CDCl_3$) δ = 8.87 (1H, d, ^3J 8.6 Hz, C4`*H*), 8.78 (1H, d, $^3J$ 8.0 Hz, C5`*H*), 7.92 – 7.84 (1H, m, C8`*H*), 7.80 (1H, dd, $^3J$ 8.6, $^3J$ 7.4 Hz, C3`*H*), 7.72 (1H, ddd, ^3J 8.0, ^3J 7.0, ^4J 1.4 Hz, C6`*H*), 7.67 – 7.57 (4H, m, C4*H*, C2`*H*, C7`*H*, C9`*H*), 7.13 (1H, ddd, ^3J 8.0, ^3J 7.1, ^4J 1.0 Hz, C5*H*), 7.05 – 6.94 (2H, m, C6*H*, C10`*H*), 6.78 (1H, dd, $^3J$ 8.2, $^4J$ 1.0 Hz, C7*H*), 6.56 – 6.46 (1H, m, C3*H*), 2.17 (3H, d, $^4J$ 0.9 Hz, C2–C$H_3$); $^{13}$C NMR (126 MHz, $CDCl_3$) δ = 139.4 (*C7a*), 138.5 (*C2*), 135.2 (*C1*`), 132.1 (*C8a*`), 131.9 (*C4a*`), 130.6 (*C10a*`), 130.3 (*C4b*`), 128.9 (*C8*`), 128.6 (*C9*`), 128.4 (*C3a*), 127.6 (*C2*`), 127.31 (*C6*`/*C7*`), 127.26 (*C6*`/*C7*`), 126.5 (*C3*`), 123.6 (*C4*`), 123.1 (*C5*`), 121.5 (*C10*`), 121.2 (*C6*), 120.1 (*C5*), 119.7 (*C4*), 110.4 (*C7*), 101.1 (*C3*), 13.1 (C2–CH_3); ESI-MS: m/z calcd. for $C_{23}H_{18}N$ 308.1434 found 308.1436 [M+H$^+$]. The e.r. of the isolated product was determined by HPLC on a chiral stationary phase (Chiralcel IG 3 µm, 250x 4.6 mm, 1.0 mL/min, heptane/*i*PrOH 97.5 : 2.5, 20°C): t_{major} = 5.8 min and t_{minor} = 6.1 min.

(S_a)-2-Methyl-1,4-bis(2-vinylphenyl)-1*H*-indole ((S_a)-19a):

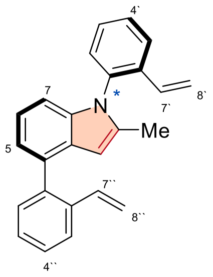

(S_a)-**19a**

Prepared according to the general procedure **N** using 2-methyl-1,4-bis(2-vinylphenyl)-1*H*-indole (±)-**19a** (23.5 mg, 70.0 µmol) to give (R_a)-2-methyl-1,4-bis(2-vinylphenyl)-1*H*-indole (S_a)-**19a** as yellow solid (8.70 mg, 26.0 µmol, 37% recovered, 18 : 82 e.r., m.p. 150.4 – 151.5°C) after preparative thin layer chromatography (pTLC, cyclohexane/EtOAc 5:1): R_f 0.71 (cyclohexane/EtOAc 5:1); $[\alpha]_D^{22}$ = – 8.0 (c 1.1, $CHCl_3$). The e.r. of the isolated product was determined by HPLC on a chiral stationary phase (Chiralcel IG 3 µm, 250x 4.6 mm, 1.0 mL/min, heptane/*i*PrOH 97.5 : 2.5, 20°C): t_{major} = 4.1 min and t_{minor} = 3.9 min.

(S_a)-1-(2-Methoxyphenanthren-1-yl)-1*H*-indole ((S_a)-20b):

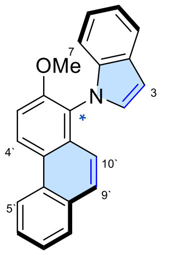

(S_a)-**20b**

Prepared according to the general procedure **N** using 7-methoxy-1,4-bis(2-vinylphenyl)-1*H*-indole **19b** (24.6 mg, 70.0 µmol) to give the title compound (S_a)-**20b** as a beige oil (9.0 mg, 27.8 µmol, 40%, 75 : 25 e.r.) after preparative thin layer chromatography (pTLC, cyclohexane/EtOAc 4:1): R_f 0.29 (cyclohexane/EtOAc 5:1); $[\alpha]_D^{22}$ = -61 (c 0.11, $CHCl_3$); ν_{max} (neat): 3051w, 2962w, 2842w, 1692w, 1611m, 1528w, 1480s, 1454s, 1356w, 1274m, 1068m, 969w, 907m, 816w, 734s; ^{1}H NMR (500 MHz, $CDCl_3$) δ = 8.81 (1H, d, ^3J 9.1 Hz, C4`*H*), 8.67 (1H, d, $^3J$ 8.3 Hz, C5`*H*), 7.85 – 7.80 (1H, m, C8`*H*), 7.76 (1H, dt, $^3J$ 7.9, $^4J$ 0.9 Hz, C4*H*), 7.68 (1H, ddd, $^3J$ 8.3, $^3J$ 7.0, $^4J$ 1.4 Hz, C6`*H*), 7.61 – 7.54 (2H, m, C7`*H*, C9`*H*), 7.51 (1H, d, ^3J 9.1 Hz, C3`*H*), 7.27 (1H, d, $^3J$ 3.2 Hz, C2*H*), 7.19 – 7.14 (1H, m, C5*H*), 7.10 (1H, ddd, $^3J$ 8.1, $^3J$ 7.1, $^4J$ 1.2 Hz, C6*H*), 7.04 (1H, d, $^3J$ 9.2 Hz, C10`*H*), 6.87 – 6.83 (1H, m, C7*H*), 6.81 (1H, dd, ^3J 3.1, ^4J 0.9 Hz, C3*H*), 3.85 (3H, s, OCH_3); ^{13}C NMR (126 MHz, $CDCl_3$) δ = 154.7 (*C2`*), 138.0 (*C7a*), 131.7 (*C10a`*), 131.0 (*C8a`*), 130.29 (*C4b`*), 130.23 (*C2*), 129.0 (*C9`*), 128.9 (*C8`*), 128.4 (*C3a*), 127.3 (*C6`*), 126.4 (*C7`*), 125.2 (*C4a`*), 124.6 (*C4`*), 122.6 (*C1`*), 122.4 (*C5`*), 122.1 (*C6*), 121.4 (*C10`*), 121.0 (*C4*), 120.0 (*C5*), 112.7 (*C3`*), 110.9 (*C7*), 102.8 (*C3*), 56.6 (O*C*H_3); ESI-MS: m/z calcd. for $C_{23}H_{18}O$ 324.1383 found 324.1381 [M+H$^+$]. The e.r. of the isolated product was determined by HPLC on a chiral stationary phase (Chiralcel IB 3 µm, 250x 4.6 mm, 1.0 mL/min, heptane/*i*PrOH 97.5 : 2.5, 20°C): t_{major} = 9.1 min and t_{minor} = 9.5 min.

(R_a)-1-(2-Methylphenanthren-1-yl)-1*H*-indole ((R_a)-20c):

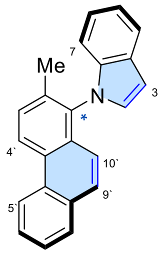

(R_a)-**20c**

Prepared according to the general procedure **N** using 7-methyl-1,4-bis(2-vinylphenyl)-1*H*-indole **19c** (23.5 mg, 70.0 µmol) to give the title compound (R_a)-**20c** as a beige oil (20.1 mg, 65.4 µmol, 93%, 97 : 3 e.r.): R_f 0.72 (cyclohexane/EtOAc 5:1); $[\alpha]_D^{22}$ = –121 (c 0.09, $CHCl_3$); ν_{max} (neat): 3053w, 3017w, 2922w, 2848w, 1600w, 1474s, 1391w, 1320m, 1218m, 1010w, 968w, 816m, 741s; ^{1}H NMR (500 MHz, $CDCl_3$) δ = 8.87 – 8.69 (2H, m, C4`*H*, C5`*H*), 7.85 (1H, d, ^3J 7.8 Hz, C8`*H*), 7.78 (1H, d, $^3J$ 8.0 Hz, C4*H*), 7.73 – 7.64 (2H, m, C3`*H*,

C6`$H$), 7.63 – 7.54 (2H, m, C7`H, C9`$H$), 7.24 (1H, d, $^3J$ 3.0 Hz, C2$H$), 7.21 – 7.16 (1H, m, C5$H$), 7.11 (1H, t, $^3J$ 7.5 Hz, C6$H$), 6.97 (1H, d, $^3J$ 9.1 Hz, C10`H), 6.86 – 6.76 (2H, m, C3H, C7H), 2.17 (3H, s, C2`–C$H_3$); $^{13}$C NMR (126 MHz, $CDCl_3$) δ = 137.7 (*C7a*), 135.6 (*C2`*), 134.4 (*C1`*), 131.7 (*C8a`*), 130.8 (*C10a`*), 130.2 (*C4b`*), 129.7 (*C4a`*), 129.4 (*C2*), 129.0 (*C3`*), 128.8 (*C8`*), 128.5 (*C9`*), 128.3 (*C3a*), 127.1 (*C6`*), 126.9 (*C7`*), 123.3 (*C4`*), 122.8 (*C5`*), 122.4 (*C6*), 121.7 (*C10`*), 121.1 (*C4*), 120.1 (*C5*), 110.7 (*C7*), 103.0 (*C3*), 17.8 (CH_3); ESI-MS: m/z calcd. for $C_{23}H_{18}N$ 308.1434 found 308.1431 [M+H$^+$]. The e.r. of the isolated product was determined by HPLC on a chiral stationary phase (Chiralcel IG 3 µm, 250x 4.6 mm, 1.0 mL/min, heptane/*i*PrOH 96 : 4, 20°C): t_{major} = 5.3 min and t_{minor}= 5.5 min.

(*S*$_a$)-1-(2-Chlorophenanthren-1-yl)-1*H*-indole ((*S*$_a$)-20d):

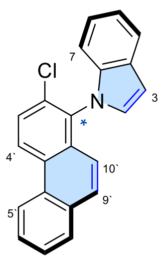

(*S*$_a$)-**20d**

Prepared according to the general procedure **N** using 7-chloro-1,4-bis(2-vinylphenyl)-1*H*-indole **19d** (24.9 mg, 70.0 µmol) to give the title compound (*S*$_a$)-**20d** as a beige solid (20.9 mg, 63.8 µmol, 91%, 96:4 e.r., m.p. 115.7 – 117.0°C): R_f 0.42 (cyclohexane/EtOAc 5:1); $[\alpha]_D^{22}$ = –82 (c 0.11, $CHCl_3$); ν_{max} (neat): 3058w, 2922w, 2855w, 1588w, 1512m, 1469s, 1391w, 1317w, 1227w, 1144w, 1078w, 1034w, 897w, 859w, 817m, 745s; ^{1}H NMR (500 MHz, $CDCl_3$) δ = 8.79 (1H, d, ^3J 9.0 Hz, C4`$H$), 8.72 (1H, d, $^3J$ 8.3 Hz, C5`H), 7.90 – 7.85 (1H, m, C8`$H$), 7.83 (1H, d, $^3J$ 9.0 Hz, C3`H), 7.78 (1H, dt, ^3J 7.9, ^4J 0.9 Hz, C4H), 7.73 (1H, ddd, ^3J 8.3, ^3J 7.0, ^4J 1.4 Hz, C6`$H$), 7.68 – 7.62 (2H, m, C7`H, C9`$H$), 7.27 (1H, d, $^3J$ 3.2 Hz, C2$H$), 7.20 (1H, ddd, $^3J$ 7.9, $^3J$ 7.1, $^4J$ 1.0 Hz, C5$H$), 7.14 (1H, td, $^3J$ 7.9, $^4J$ 1.2 Hz, C6$H$), 7.02 (1H, d, $^3J$ 9.1 Hz, C10`H), 6.85 (1H, dd, ^3J 3.2, ^4J 0.9 Hz, C3H), 6.85 – 6.81 (1H, m, C7H); ^{13}C NMR (126 MHz, $CDCl_3$) δ = 137.6 (*C7a*), 133.4 (*C1`*), 132.4 (*C2`*), 131.92 (*C10a`*), 131.90 (*C8a`*), 130.1 (*C4a`*), 129.8 (*C4b`*), 129.7 (*C9`*), 129.4 (*C2*), 129.0 (*C8`*), 128.4 (*C3a*), 127.64 (*C3`/C6`*), 127.63(*C3`/C6`*), 127.59 (*C7`*), 124.7 (*C4`*), 122.9 (*C5`*), 122.6 (*C6*), 121.4 (*C10`*), 121.2 (*C4*), 120.5 (*C5*), 110.7 (*C7*), 103.7 (*C3*); ESI-MS: m/z calcd. for $C_{22}H_{14}AgClN$ 433.9860 found 433.9860 [M+Ag$^+$]. The e.r. of the isolated product was determined by HPLC on a chiral stationary phase (Chiralcel IG 3 µm, 250x 4.6 mm, 1.0 mL/min, heptane/*i*PrOH 99 : 1, 20°C): t_{major} = 7.8 min and t_{minor} = 8.4 min.

(*S*$_a$)-1-(2,6-Dichlorophenanthren-1-yl)-1*H*-indole ((*S*$_a$)-20e):

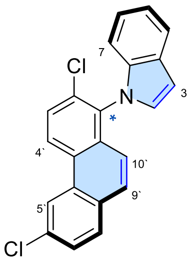

(*S*$_a$)-**20e**

Prepared according to the general procedure **N** using 7-chloro-4-(5-chloro-2-vinylphenyl)-1-(2-vinylphenyl)-1*H*-indole **19e** (27.3 mg, 70.0 µmol) to give the title compound (*S*$_a$)-**20e** as a white solid (17.0 mg, 46.9 µmol, 67%, 96:4 e.r., m.p. 67.0 – 68.0°C) after preparative thin layer chromatography (pTLC, cyclohexane/EtOAc 5:1): R_f 0.61 (cyclohexane/EtOAc 5:1); $[\alpha]_D^{22}$ = –56 (c 0.11, $CHCl_3$); ν_{max} (neat): 2973w, 2902w, 1697w, 1587w, 1511w, 1467s, 1383w, 1317w, 1225w, 1153w, 1076m, 904w, 868w, 839w, 790m; ^{1}H NMR (500 MHz, $CDCl_3$) δ = 8.70 (d, ^3J 8.8 Hz, C4`$H$), 8.68 (1H, d, $^4J$ 1.9 Hz, C5`H), 7.85 (1H, d, ^3J 8.8 Hz, C3`$H$), 7.81 (1H, d, $^3J$ 8.5 Hz, C8`H),

7.78 (1H, dt, ^{3}J 7.9, ^{4}J 1.1 Hz, C4*H*), 7.65 – 7.58 (2H, m, C7`*H*, C9`*H*), 7.26 (1H, d, ^{3}J 2.9 Hz, C2*H*), 7.24 – 7.18 (1H, m, C5*H*), 7.14 (1H, ddd, ^{3}J 8.2, ^{3}J 7.1, ^{4}J 1.1 Hz, C6*H*), 7.03 (1H, dd, ^{3}J 9.2, ^{5}J 0.6 Hz, C10`*H*), 6.86 (1H, dd, $^{3}J$ 3.2, $^{5}J$ 0.9 Hz, C3*H*), 6.82 (1H, dd, $^{3}J$ 8.2, $^{6}J$ 0.9 Hz, C7*H*); $^{13}$C NMR (126 MHz, $CDCl_3$) δ = 137.6 (*C7a*), 133.7 (*C6`*), 133.5 (*C1`*), 133.2 (*C2`*), 132.2 (*C10a`*), 130.9 (*C4b`*), 130.3 (*C8`*), 130.2 (*C8a`*), 129.3 (*C2*), 129.0 (*C4a`*), 128.4 (*C3a*), 128.2 (*C7`*), 128.0 (*C3`*), 124.7 (*C4`*), 122.70 (*C5`*), 122.68 (*C6*), 121.8 (*C10`*), 121.2 (*C4*), 120.6 (*C5*), 110.7 (*C7*), 103.8 (*C3*); ESI-MS: m/z calcd. for $C_{22}H_{13}Cl_2NNa$ 384.0317 found 384.0311 [M+Na^{+}]. The e.r. of the isolated product was determined by HPLC on a chiral stationary phase (Chiralcel IB 3 μm, 250x 4.6 mm, 1.0 mL/min, heptane/*i*PrOH 97.5 : 2.5, 20°C): t_{major} = 6.7 min and t_{minor} = 7.3 min.

Determination of the Rotational Barriers

Separation of atropisomers **(R_a)-19a** and **(S_a)-19a** was performed on a chiral stationary phase (Chiralpak IG (3 µm, 250×4.6 mm), heptane/*i*PrOH 97.5 : 2.5, 1.0 mL/min, 20°C), Separation of atropisomers **(R_a)-20a** and **(S_a)-20a** was performed on a chiral stationary phase (Chiralpak IG (3 µm, 250×4.6 mm), heptane/*i*PrOH 97.5 : 2.5, 1.0 mL/min, 20°C).

Determination of the Rotational Barrier of 19a:

A stirred solution of **(R_a)-19a** (t = 3.9 min) in heptane was heated to 115°C for 5.5 h. Aliquots were withdrawn in regular time intervals and analyzed by NP-HPLC (Chiralpak IG (3 µm, 250×4.6 mm), heptane/*i*PrOH 97.5 : 2.5, 1.0 mL/min, 20°C) (Figure S1). The rate constant was obtained from the slope of the plot ln([ee$_t$]/[ee$_0$]) against t (Figure S2) to give k_r = 4.10•10^{-5} s^{-1}. With k_r and Eyring equation, the Gibbs free energy energy ΔG‡ of the rotational barrier was calculated:

$$\Delta G^{\ddagger} = -RTln\left(\frac{k_r h}{2k_B T}\right) = 130\ kJ \cdot mol^{-1}$$

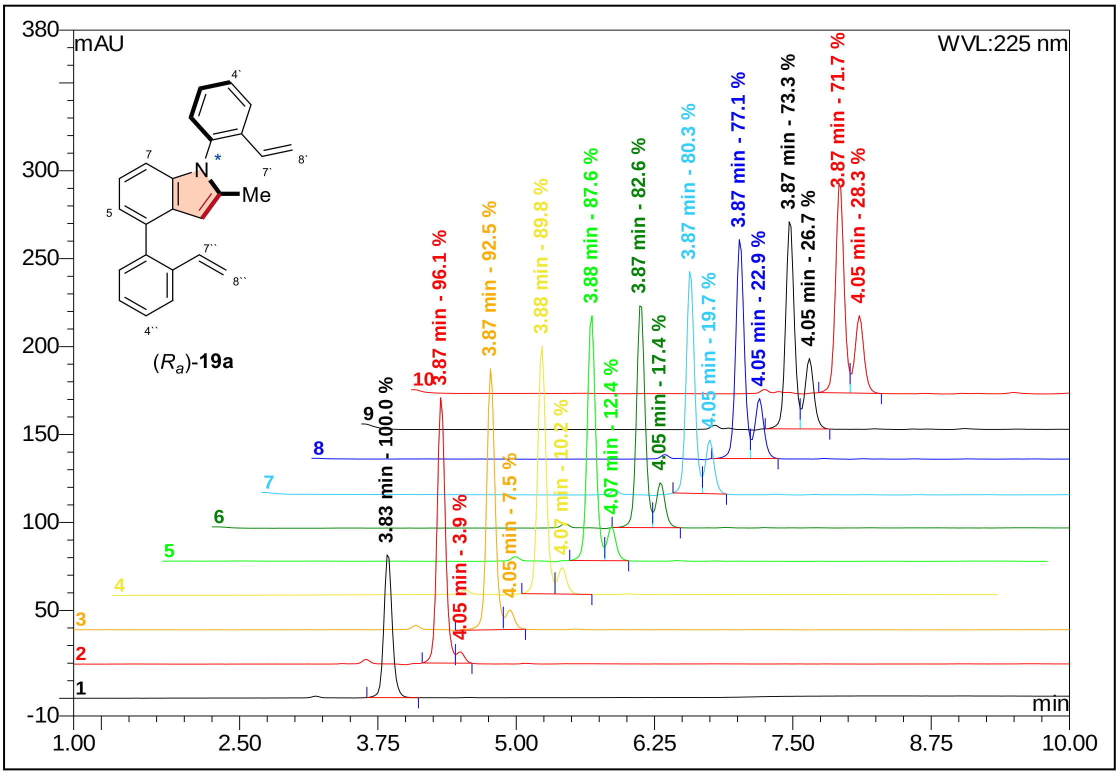

Figure S1. Overlay of the HPLC traces of the thermal isomerization of **(R_a)-19a** after 0, 0.5, 1, 1.5, 2, 2.8, 3.5, 4, 5, 5.5 h

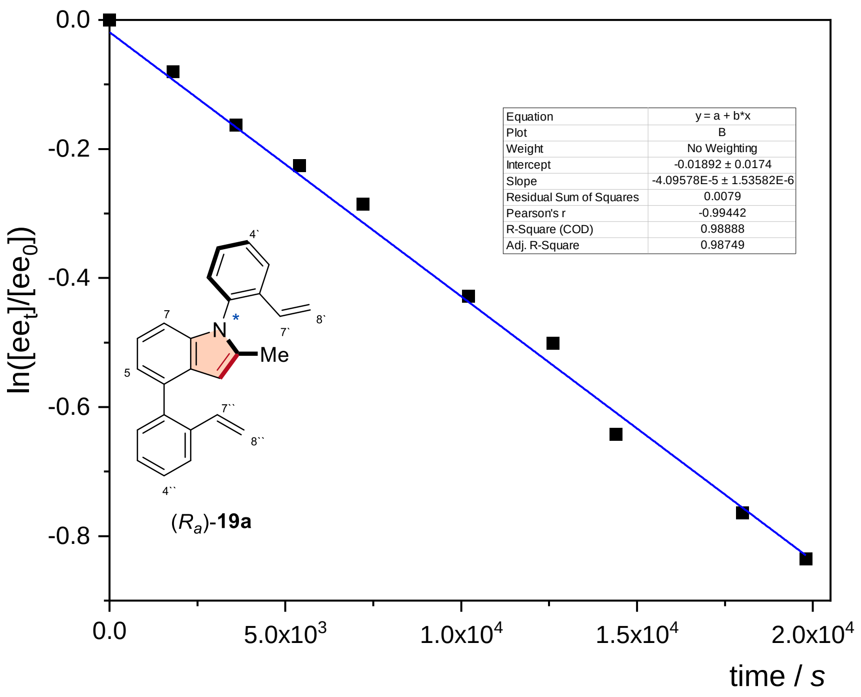

Figure S2. Plot for the racemization of **(R_a)-19a** in heptane at 115°C

Determination of the Rotational Barrier of 20a:

A stirred solution of **(R_a)-20a** (t = 5.8 min) in heptane was heated to 140°C for 3.5 h. Aliquots were withdrawn in regular time intervals and analyzed by NP-HPLC (Chiralpak IG (3 µm, 250×4.6 mm), heptane/*i*PrOH 97.5 : 2.5, 1.0 mL/min, 20°C) (Figure S3). The rate constant was obtained from the slope of the plot ln([ee$_t$]/[ee$_0$]) against *t* (Figure S4) to give k_r = 1.06•10^{-5} s^{-1}. With k_r and Eyring equation, the Gibbs free energy energy ΔG‡ of the rotational barrier was calculated:

$$\Delta G^{\ddagger} = -RT ln\left(\frac{k_r h}{2k_B T}\right) = 144\ kJ \cdot mol^{-1}$$

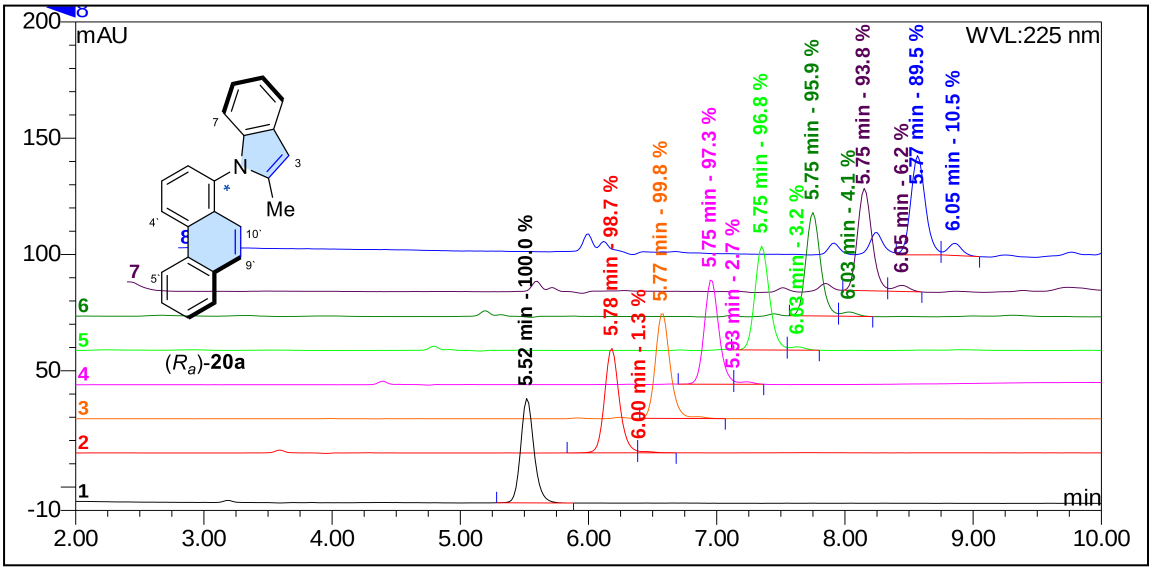

Figure S3. Overlay of the HPLC traces of the thermal isomerization of **(R_a)-20a** after 0, 0.5, 1, 1.5, 2, 2.5, 3, 3.5 h

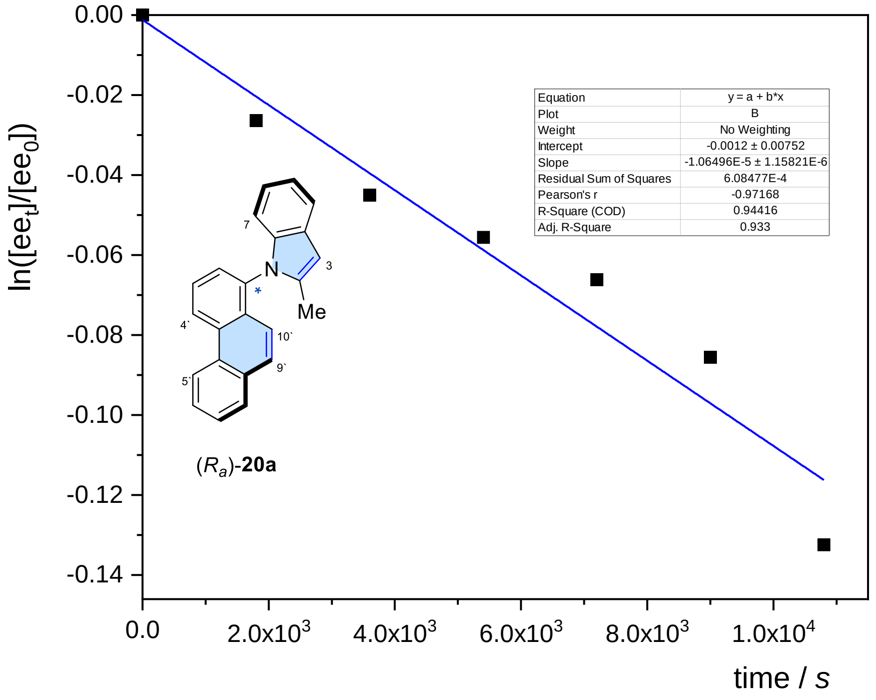

Figure S4. Plot for the racemization of **(*Ra*)-20a** in heptane at **140°C**

Note: Deviation from linearity may arise from the particularly slow racemization of **(*Ra*)-20a** along with the decomposition of the product as indicated by the appearance of new signals in addition to ones for **20a.**

Computational Studies

Gibbs Free Energy Differences of the Selected Pairs of Starting Materials and Products

Density functional theory (DFT) calculations were performed using the Gaussian 16 (revision C.01) program[14]. Geometry optimizations were performed at the B3LYP-D3(BJ) level of theory with the def2-TZVP basis set in the gas phase[12]. Harmonic vibrational frequencies were evaluated for the optimized geometries, with minima characterized by the absence of imaginary frequencies. Quasiharmonic corrections to the entropy for frequencies below 100 cm^{-1} were calculated with the Goodvibes program by employing the method of Grimme. Single point energies were calculated at B3LYP-D3(BJ) level of theory with the def2-TZVP basis set with implicit solvation model SMD for toluene. The optimized structures were visualized in CYLView[13].

Supplementary Table 16. DFT computed Gibbs free energies for selected substrates and the corresponding products (SMD(toluene)/B3LYP-D3(BJ)/def2-TZVP//B3LYP-D3(BJ)/def2-TZVP)

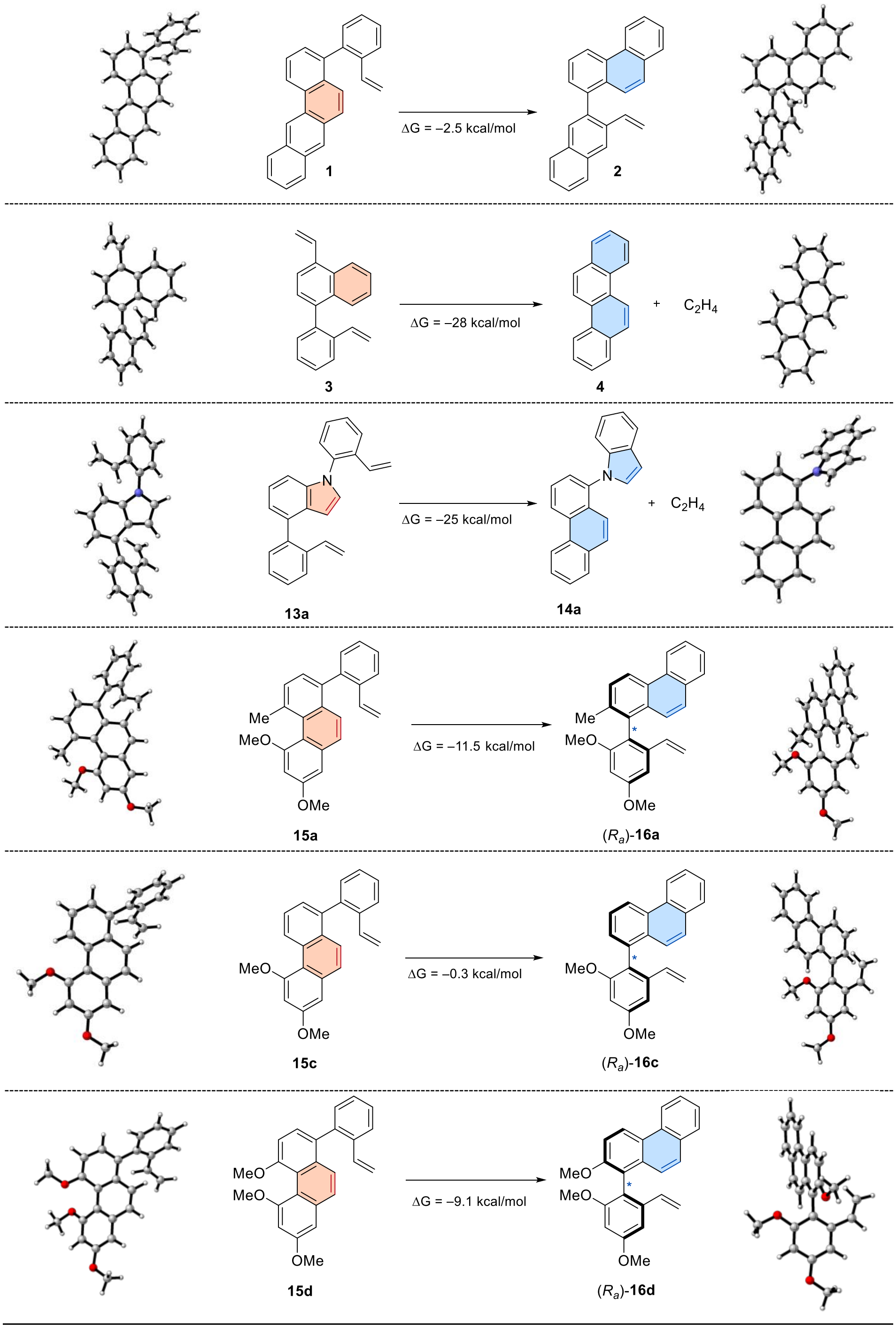

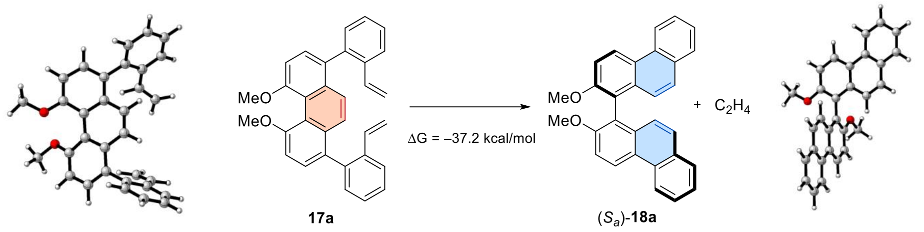
MeO
MeO
17a
ΔG = –37.2 kcal/mol
MeO
MeO
(Sa)-18a
+ C2H4

Comparison of Metathesis Catalysts

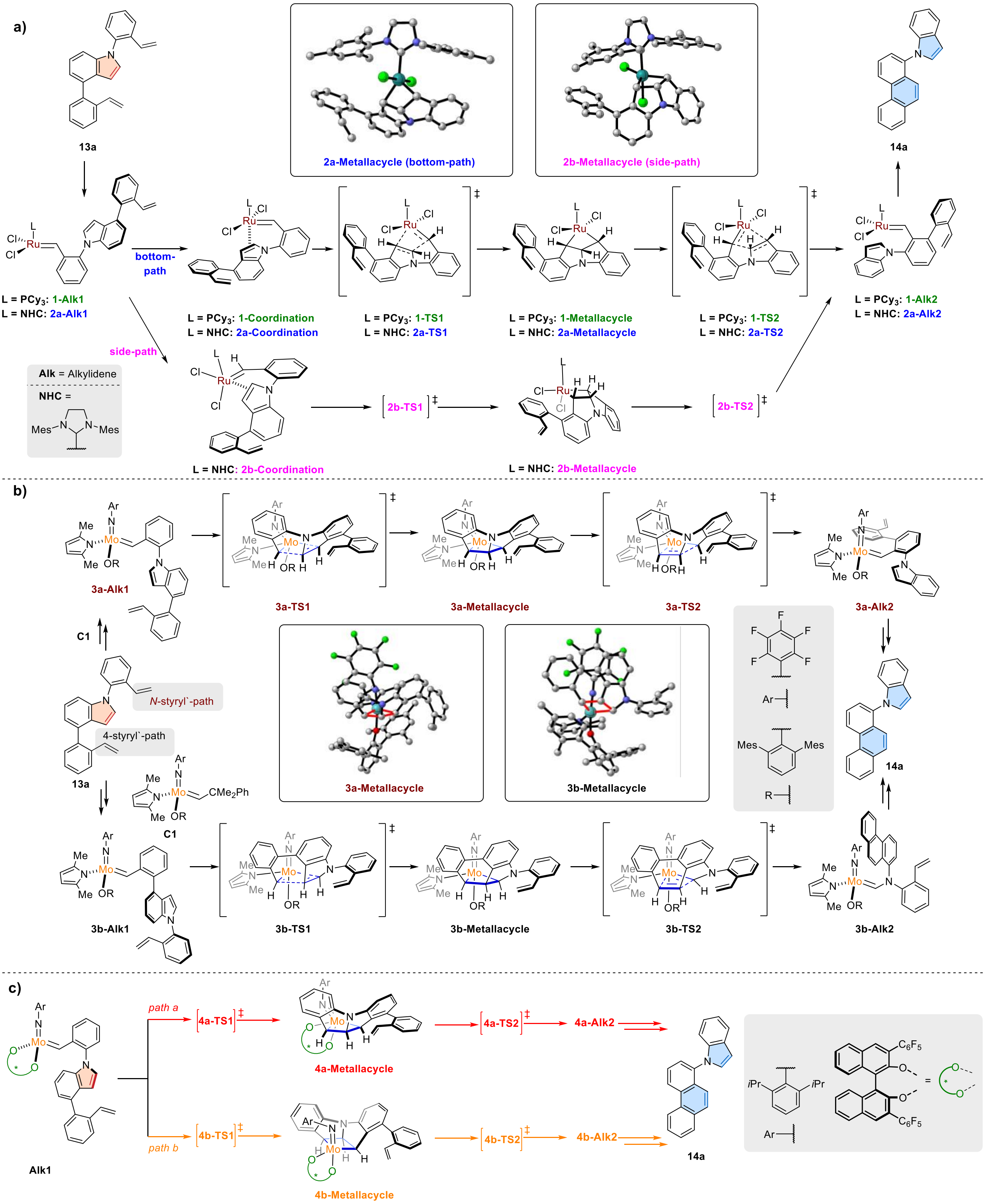

Figure S5. ArROM-twofold RCM for **13a** with different catalytic system: a) Ru-catalyzed metathesis (Grubbs I and Hoveyda-Grubbs II catalysts): bottom- and side-path mechanisms; b) Mo-catalyzed metathesis (**C1** catalyst): *N*-styryl`- and 4-styryl`-paths. c) Catalytic system obtained from Mo-Precatalyst and (S_a)-**L2**: paths a and b.

Density functional theory (DFT) calculations were performed using the Gaussian 16 (revision C.01) program.[14] Geometries of intermediates and transition states were optimized using the dispersion-corrected B3LYP-D3 functional. The SDD basis set was used for Mo/Ru atoms, while the 6-31G(d) basis set was applied for other atoms. Vibrational frequency calculations were performed at the same level of theory of the optimization to confirm if each structure is a local minimum (zero imaginary frequencies) or a transition state (one imaginary frequency). Single-point energy calculations were carried out using the M06 functional with the SDD basis set for Mo/Ru atoms and 6-311+G(d,p) basis set for other atoms[15]. Solvent effects were accounted for with the SMD model of toluene, which was the solvent of choice in experiments. Quasiharmonic corrections to the entropy for frequencies below 100 cm^{-1} were calculated with the Goodvibes program by employing the method of Grimme[16].

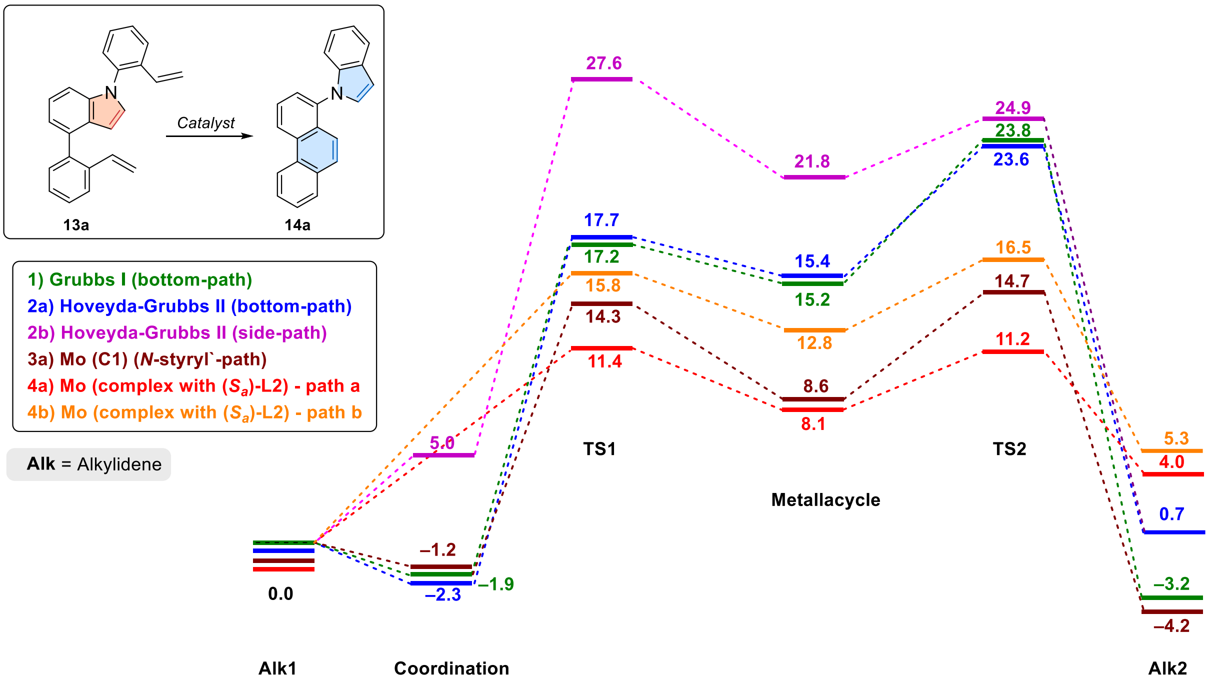

Figure S6. DFT-calculated Gibbs free energies (kcal/mol) of the reaction profiles of **13a** with different Mo- and Ru-based catalysts. SMD(toluene)/MO6/SDD(Mo/Ru)-6-311+G(d,p)//B3LYP-D3(BJ)/SDD(Mo/Ru)-6-31G(d)

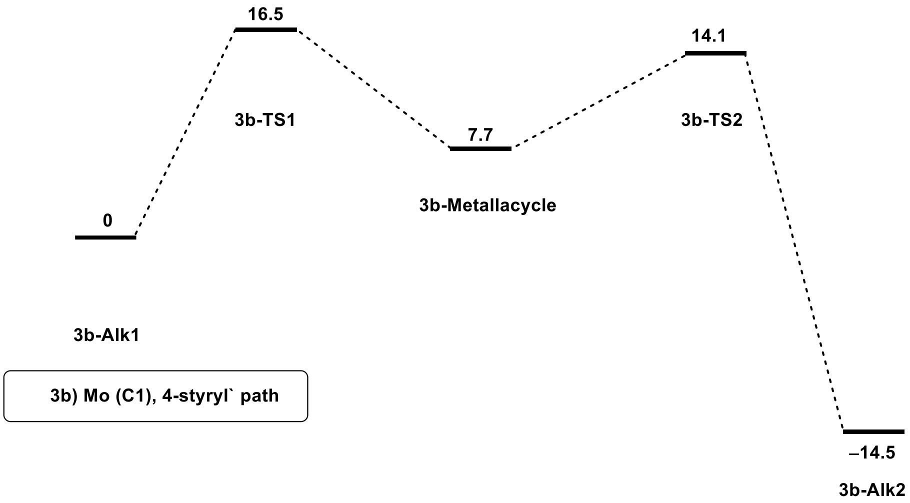

Figure S7. DFT-calculated Gibbs free energies (kcal/mol) of the reaction profile of **13a** with **C1** as a catalyst, 4-styryl` path.

Visualization of Noncovalent interactions (NCI) in DFT Computed Metallacycles

The Multiwgn software[17], version 3.8, and .fchk files from the single-point DFT-calculations were used for the noncovalent interactions (NCI) analysis[18], and VMD[19], version 1.9.4, for the visualization. An isovalue of 0.5 and grid spacing of 0.116 Bohr was used for the isosurfaces in the 3D representations.

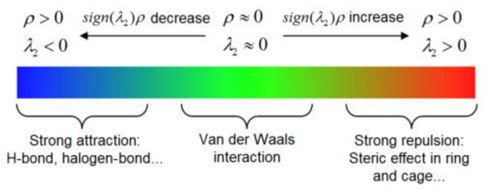

The colour code used for NCI plots

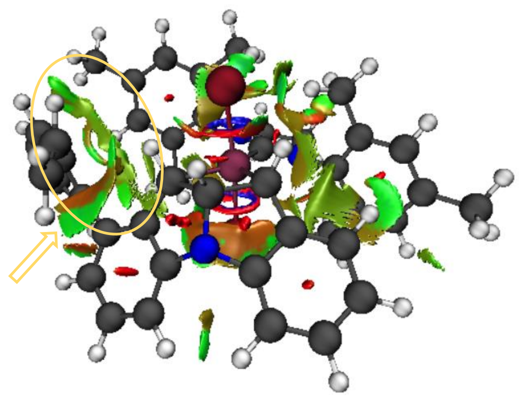

Figure S8. NCI plot for **2a**-Metallacycle

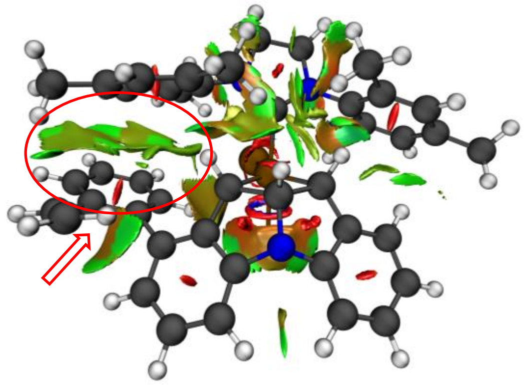

Figure S9. NCI plot for **2b**-Metallacycle

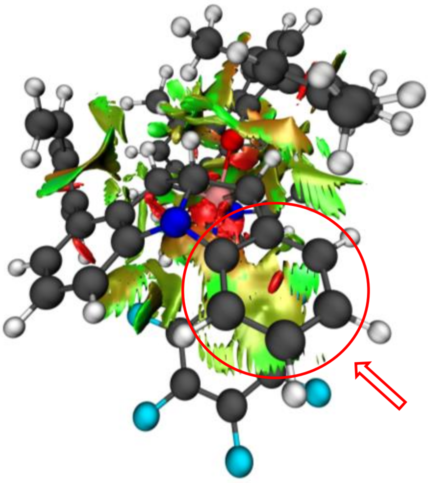

Figure S10. NCI plot for **3a**-Metallacycle

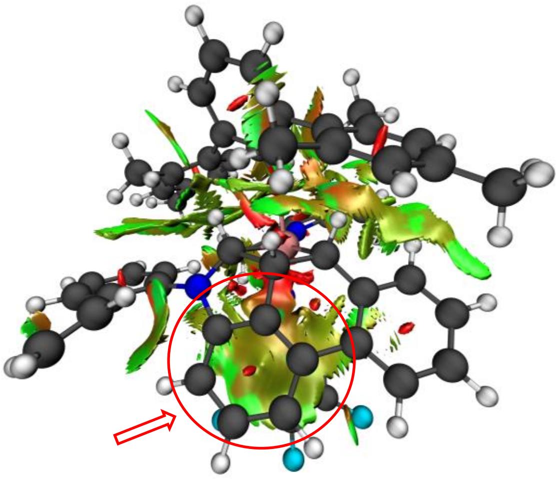

Figure S11. NCI plot for **3b**-Metallacycle

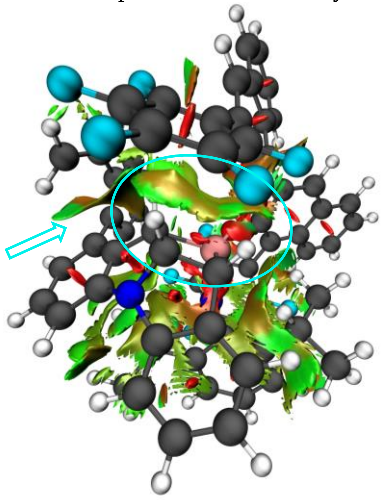

Figure S11. NCI plot for **4a**-Metallacycle

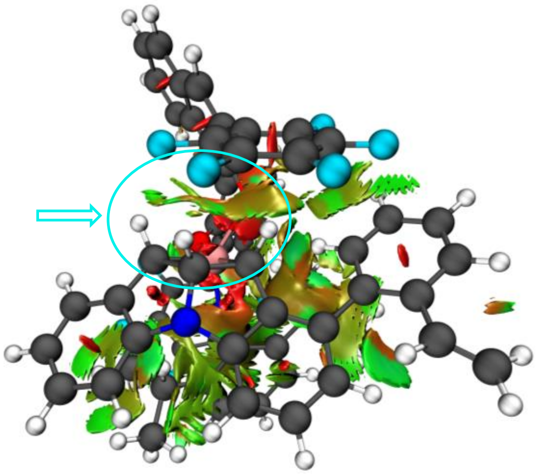

Figure S12. NCI plot for **4b**-Metallacycle

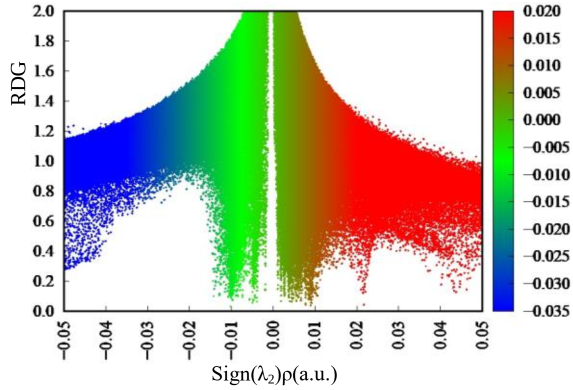

Figure S13. RDG scatter plot for **2a**-Metallacycle

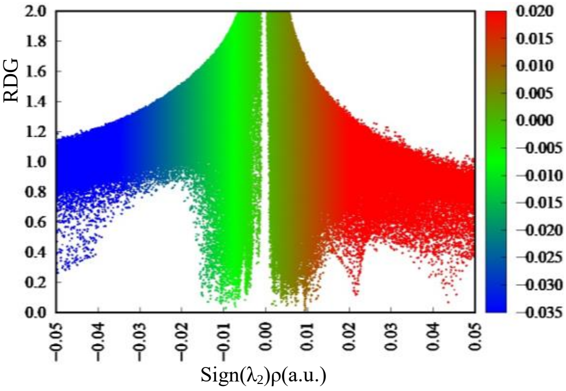

Figure S14. RDG scatter plot for **2b**-Metallacycle

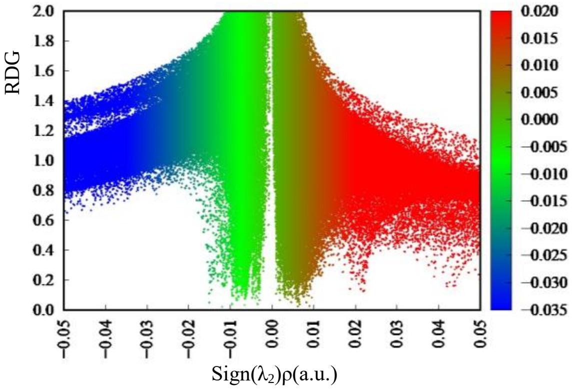

Figure S15. RDG scatter plot for **3a**-Metallacycle

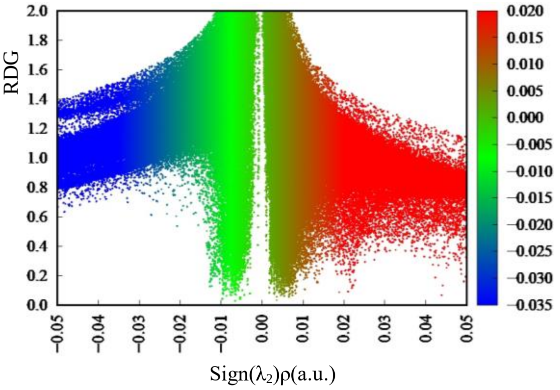

Figure S16. RDG scatter plot for **3b**-Metallacycle

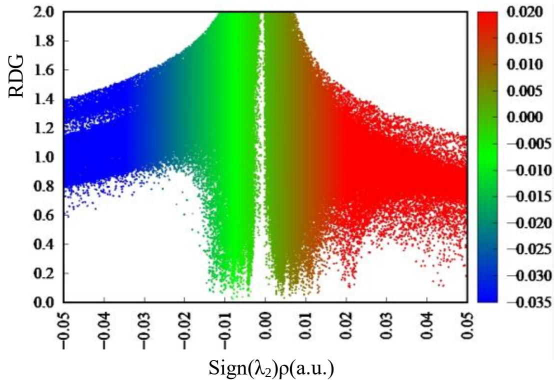

Figure S17. RDG scatter plot for **4a**-Metallacycle

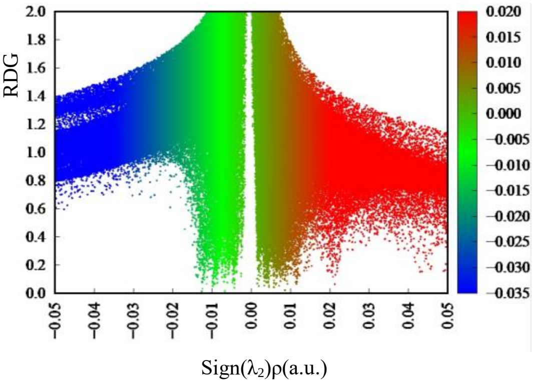

Figure S18. RDG scatter plot for **4b**-Metallacycle

Note: NCI analysis of a selection of metallacycles (**2a**/**b**, **3a**/**b**, **4a**/**b**) was performed to the determine the major NCIs that could contribute to the energy differences between these reaction intermediates. The commonly observed NCIs included C–H···π and π-π interactions. For example, stronger π-π interactions observed for the metallacycle **3b** (Figure S11) in comparison to the metallacycle **3a** (Figure S10) could partially explain its higher stability. On the other hand, metallacycle-**4a** possesses more C–H···π interactions (Figure S11) than metallacycle **4b** (Figure S12), which might be among the factors causing its lower Gibbs free energy.

Cartesian Coordinates for All Optimized Geometries

1-Alk1:

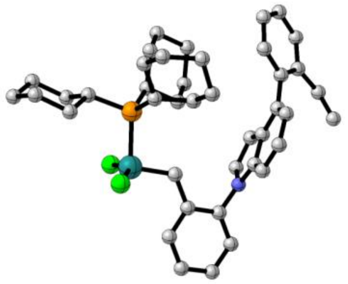

G_sol = –3003.519161

P 1.90460800 -0.82185100 -0.01971800
Ru 2.23636600 1.38719800 -0.28482900
Cl 3.23068400 1.81524000 1.78574400
Cl 2.46978800 1.31570900 -2.60268900
C 0.51856700 1.95169600 -0.02219900
H -0.32800500 1.27605300 0.06995700
H -0.37486100 0.08145600 1.68214500
C 0.81504000 -1.60907800 -1.31606300
C 1.33639700 -1.27197000 1.70912500
C 3.52473000 -1.74251400 -0.21365800
C 5.37384200 -2.58139300 -1.72027700
C 5.85088600 -2.11199600 0.71555300
C 6.42821700 -2.12675200 -0.70450100
C 4.58542100 -1.24812400 0.78857200
C 4.09935600 -1.72687200 -1.64233900
H 5.11521500 -3.63326500 -1.52717300
H 5.60894000 -3.14053100 1.02289000
H 6.76555200 -1.11313900 -0.96451400
H 4.84260600 -0.20833300 0.55681000
H 4.32189400 -0.69644300 -1.93991800
H 3.28339300 -2.78580300 0.03054000
H 5.77905200 -2.53940200 -2.73846700
H 6.59400800 -1.73626100 1.42906500
H 7.31069400 -2.77627100 -0.75380800
H 4.19180800 -1.23410900 1.80939600
H 3.36842200 -2.10363700 -2.36421300
C -0.19827400 -3.72156500 -2.28055500
C -1.17789000 -1.46863700 -2.85045600
C -1.47901800 -2.95370800 -2.62637000
C -0.47465900 -0.83812300 -1.64084700
C 0.51519600 -3.10060400 -1.06972000
H 0.48290500 -3.70746000 -3.14362500
H -0.53327200 -1.35283600 -3.73388700
H -2.19911000 -3.05673400 -1.80409600
H -1.15641700 -0.84216200 -0.78319900
H -0.13370100 -3.19949500 -0.19108800
H 1.44651600 -1.52673300 -2.20921000
H -0.42300600 -4.77527100 -2.07257500
H -2.10124400 -0.91904500 -3.05895800
H -1.95185500 -3.38747200 -3.51617900
H -0.23606800 0.20257900 -1.86384200
H 1.43388200 -3.65780800 -0.85442600
C -0.53272200 -1.17907200 3.42005200
C 1.28009400 -2.91291400 3.64213500
C -0.19961400 -2.60028400 3.88191200
C 1.66639500 -2.70876000 2.16972300
C -0.15169300 -0.95275500 1.95072100
H 0.01176600 -0.45533400 4.04304300
H 1.89660100 -2.25338100 4.26940500
H -0.81687700 -3.31913200 3.32162000
H 1.12486900 -3.43614600 1.55530900
H -0.77718800 -1.59038800 1.31421200
H 1.92207800 -0.57799800 2.32498300
H -1.59818800 -0.96813700 3.55010400
H 1.51322500 -3.94234600 3.94138400
H -0.45177600 -2.72858800 4.94161000
H 2.73185500 -2.92165500 2.04980000
C 0.19056000 3.37476900 -0.04295200
C -1.14100200 3.80102200 -0.28320800
C 1.17184400 4.37249500 0.13764000
N -2.16810700 2.82369800 -0.44083800
C -1.46112400 5.15176000 -0.36754200
C 0.84972800 5.72338400 0.04830800
H 2.18847500 4.07699800 0.38068800
C -2.65959900 2.04029500 0.59698800
C -2.51937200 2.22211800 -1.64675100
C -0.46460900 6.11775000 -0.21120000
H -2.49318500 5.43355100 -0.55052700
H 1.62474600 6.47019900 0.19100800
C -3.33644300 0.93299100 0.01968300
C -2.53502200 2.22067000 1.97752500
C -3.24755700 1.08830800 -1.40584000
H -2.20396400 2.67388300 -2.57619500
H -0.71753600 7.17147800 -0.27794600
C -3.87500300 -0.06201600 0.86808100
C -3.11579400 1.25742900 2.79297300
H -2.00845900 3.07639000 2.38743500
H -3.68683400 0.44309600 -2.15010600
C -3.76094400 0.13041900 2.24571800
C -4.39169000 -1.34321000 0.31788500
H -3.05466500 1.36152700 3.87200100
H -4.17701000 -0.61955600 2.91217800
C -3.72932100 -2.51261200 0.72251500
C -5.44265700 -1.44555300 -0.62796200
C -4.04999800 -3.76223500 0.19533600
H -2.92720900 -2.42218600 1.44885700
C -5.72420900 -2.71081400 -1.17474400
C -6.30896100 -0.33352700 -1.05780800
C -5.04575900 -3.85849300 -0.77611800

H -3.51137700 -4.64603500 0.52526400
H -6.51758500 -2.78894200 -1.91393900
C -6.57607200 0.80615700 -0.40925900
H -6.82841800 -0.51779500 -1.99841500
H -5.29648000 -4.81903900 -1.21671900
H -6.12802200 1.05707400 0.54509700
H -7.27027400 1.52754500 -0.83007400

1-Coordination:

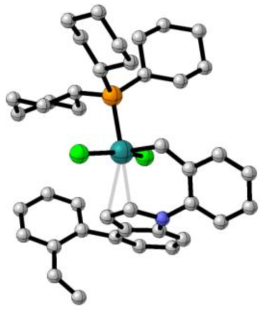

G_sol = –3003.522163

P 2.00792900 -0.63408800 0.07670900
Ru 0.05783700 0.41718000 -0.62788700
Cl 0.33806100 -0.38071000 -2.85432400
Cl -0.71530800 0.43387100 1.61423000
C 0.76560000 2.12154700 -0.65024900
H 1.85113700 2.23144600 -0.60356200
H 3.31048500 1.46452000 -1.54799700
C 2.66680500 -0.07624400 1.73553700
C 3.38044700 -0.67989000 -1.19810200
C 1.63991100 -2.45359200 0.36046300
C 0.45469400 -4.23482200 1.70953100
C 1.04676400 -4.67134500 -0.70721800
C 0.05576500 -4.96850600 0.42375800
C 1.19410100 -3.16112500 -0.93457000
C 0.61046300 -2.72549400 1.47364900
H 1.40336000 -4.64598200 2.08593200
H 2.02930400 -5.09904000 -0.45645900
H -0.94549600 -4.63532800 0.11920400
H 0.23334800 -2.74529400 -1.25815000
H -0.35765000 -2.29959300 1.19129800
H 2.59477600 -2.89431800 0.67889100
H -0.29326000 -4.40490700 2.49426100
H 0.72192700 -5.15287200 -1.63766500
H -0.00839500 -6.04906400 0.60408300
H 1.89016100 -2.96995800 -1.75606200
H 0.89739000 -2.23314800 2.40770400
C 4.30962100 -0.36833300 3.63764100
C 3.07708000 1.81916000 3.35825800
C 4.37524900 1.15179000 3.82701000
C 2.73168900 1.45266200 1.90795600
C 3.98575200 -0.73208500 2.18086300
H 3.53160900 -0.78452200 4.29355500
H 2.24812700 1.49926700 4.00515400
H 5.21927300 1.54976400 3.24407800
H 3.48755600 1.87855100 1.23430700
H 4.80457400 -0.38047200 1.54068500
H 1.87093800 -0.41564700 2.40912300
H 5.25711600 -0.83469800 3.93525900
H 3.14917900 2.90949200 3.45621500
H 4.57487800 1.39726100 4.87731200
H 1.76605100 1.88934600 1.64705000
H 3.94078000 -1.82120500 2.07012500
C 5.01722200 0.66558800 -2.59303800
C 5.37717000 -1.81000600 -2.26565700
C 6.05815400 -0.45202700 -2.46866800
C 4.43878400 -1.79226800 -1.04909100
C 4.06258300 0.68771800 -1.39012600
H 4.42708000 0.51386800 -3.50747100
H 4.79557200 -2.06299300 -3.16358400
H 6.71305900 -0.24445300 -1.60930300
H 5.03383000 -1.63235900 -0.14379700
H 4.62547500 0.95550000 -0.48584600
H 2.81200000 -0.87883500 -2.11498200
H 5.51012700 1.64083100 -2.69202300
H 6.12701600 -2.60118600 -2.14126500
H 6.70162300 -0.47531800 -3.35664000
H 3.96545200 -2.77254900 -0.94681200
C 0.13504000 3.44148300 -0.62375400
C -1.26294400 3.69347200 -0.59501700
C 0.99040200 4.55904600 -0.49765300
N -2.15626700 2.61984700 -0.77839400
C -1.75673200 4.98291400 -0.40122600
C 0.50017200 5.84542300 -0.30889600
H 2.06364600 4.38857700 -0.52398200
C -3.07166600 2.13791500 0.16075200
C -1.82497200 1.57496200 -1.65093900
C -0.88004900 6.05583500 -0.24714900
H -2.83044800 5.13862200 -0.40208600
H 1.18694300 6.67986000 -0.20564500
C -3.32992200 0.77936800 -0.15003400
C -3.61180700 2.75210300 1.29336000
C -2.53966000 0.44286800 -1.29954300
H -1.30991400 1.79768800 -2.57261700
H -1.27573000 7.05627700 -0.10005000
C -4.11547800 -0.00799800 0.71194100
C -4.43366800 1.97544400 2.10149100
H -3.38295900 3.78100100 1.54633700
H -2.56435800 -0.47986100 -1.85978900
C -4.67270500 0.61478500 1.82618500
C -4.16603900 -1.48127700 0.51622300
H -4.87988900 2.42024100 2.98621400
H -5.28302400 0.02962400 2.50751400

C -3.49465200 -2.25771700 1.47130800
C -4.74647000 -2.11469000 -0.60961200
C -3.37642700 -3.64004000 1.33464300
H -3.02591200 -1.74784200 2.30681400
C -4.59015700 -3.50685000 -0.74041300
C -5.52322100 -1.43115900 -1.65892700
C -3.92089100 -4.26740100 0.21397300
H -2.84616100 -4.21658300 2.08692700
H -5.02268300 -3.99732100 -1.60892000
C -6.15222400 -0.25058500 -1.59823700
H -5.61824900 -2.00843200 -2.57882900
H -3.82436500 -5.34137700 0.08261500
H -6.13984300 0.37844600 -0.71612400
H -6.71484600 0.11393600 -2.45275000

1-TS1:

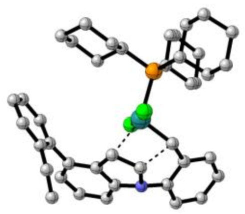

G_sol = –3003.491686

P 1.91001600 -0.79854500 0.17280300
Ru -0.03160000 0.30509000 -0.63335100
Cl 0.22923600 -0.77914500 -2.79514400
Cl -0.46375000 1.13874000 1.59318400
C 0.37824300 1.95833500 -1.44149700
H 0.85992300 1.89771700 -2.42536600
H 2.81304800 0.88386800 -2.06785900
C 2.62649500 -0.01125700 1.69829900
C 3.19183600 -1.04570400 -1.15102300
C 1.43240900 -2.52020500 0.70790900
C -0.13701000 -3.86913300 2.16544700
C 0.78603700 -4.86910400 0.03415800
C -0.40120900 -4.83273000 1.00294200
C 1.14475900 -3.46528700 -0.47398300
C 0.21740000 -2.46698000 1.65644200
H 0.69191400 -4.25161000 2.77953400
H 1.66002100 -5.30323300 0.54263800
H -1.29621400 -4.49917200 0.46261300
H 0.32257800 -3.05944500 -1.07466500
H -0.64677400 -2.05024700 1.11969900
H 2.28771500 -2.92532900 1.26615300
H -1.01352000 -3.80948300 2.82077900
H 0.56304100 -5.52102100 -0.81930400
H -0.61550800 -5.83991500 1.38255100
H 2.00924400 -3.52926100 -1.14196000
H 0.40136400 -1.78871100 2.49526600
C 4.02806300 -0.07302500 3.78605300
C 3.30699200 2.14932900 2.80152700
C 4.38896900 1.40668900 3.59660200
C 3.02187100 1.45792600 1.46253300
C 3.74531900 -0.76153000 2.44039000
H 3.13381500 -0.15078900 4.42060500
H 2.37429600 2.18166400 3.38038800
H 5.34481600 1.47468700 3.05573400
H 3.92017900 1.50484500 0.83300200
H 4.66329600 -0.76089900 1.84105300
H 1.74626400 0.01646800 2.35123500
H 4.83542200 -0.59944100 4.31049600
H 3.60748000 3.19006500 2.62699000
H 4.54531000 1.88528300 4.57124900
H 2.21735200 1.97986400 0.94042000
H 3.47744200 -1.81015800 2.61131500
C 4.49998200 -0.04942700 -3.05806900
C 5.22077700 -2.25179300 -2.02849200
C 5.68390700 -0.97089700 -2.73543200
C 4.39460200 -1.92899200 -0.77347100
C 3.67185200 0.26212200 -1.80366800
H 3.84675000 -0.53214600 -3.79763500
H 4.60569400 -2.84740000 -2.71794900
H 6.38845100 -0.43638800 -2.08094800
H 5.03185500 -1.40090400 -0.05473300
H 4.28636200 0.83183400 -1.09540900
H 2.60478300 -1.56706100 -1.91623000
H 4.85735300 0.88382700 -3.51099200
H 6.08401200 -2.87223000 -1.75720200
H 6.23370900 -1.22054200 -3.65111600
H 4.07096600 -2.85608300 -0.28766300
C 0.29706900 3.31951000 -0.86787100
C -1.00484500 3.78414200 -0.61004500
C 1.38848800 4.15330500 -0.62402700
N -2.01238400 2.83862500 -0.94096200
C -1.22391400 5.05945700 -0.10216800
C 1.17849700 5.42269900 -0.07998500
H 2.39446300 3.80218700 -0.83486800
C -2.93979300 2.33310200 -0.00229500
C -1.52003900 1.73953400 -1.71256400
C -0.11900700 5.86979000 0.17708600
H -2.23115000 5.43049300 0.04687300
H 2.02677700 6.06653100 0.13119300
C -3.08536700 0.94436100 -0.19305200
C -3.63608400 3.01289400 0.99414200
C -2.15131900 0.52328000 -1.22202700
H -1.40613400 1.88150500 -2.78484000
H -0.27887000 6.86614300 0.57803500
C -3.96459500 0.19997100 0.60824400
C -4.50903500 2.26807200 1.78391200
H -3.50429600 4.07401900 1.16268400
H -2.33223000 -0.31320800 -1.88368000
C -4.67415800 0.88780800 1.59650800

C -4.05165000 -1.27903100 0.49489600
H -5.07627800 2.76862600 2.56315700
H -5.36500900 0.33746300 2.22784000
C -3.69581900 -2.02566100 1.62769500
C -4.43421100 -1.95910400 -0.68785700
C -3.70447800 -3.41910400 1.61570300
H -3.38581400 -1.48885900 2.51905600
C -4.38880000 -3.36462800 -0.69134400
C -4.92311700 -1.31044900 -1.91921900
C -4.04156600 -4.09332000 0.44227000
H -3.43076500 -3.97194000 2.50915500
H -4.66457400 -3.88947600 -1.60242400
C -5.55685800 -0.13708900 -2.03801600
H -4.79171500 -1.91240100 -2.81848900
H -4.03412900 -5.17890100 0.40934600
H -5.76190400 0.50732500 -1.19077600
H -5.90135300 0.20529500 -3.00941200

1-Metallacycle:

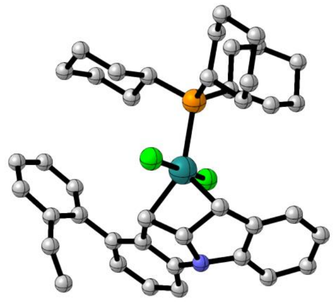

G_sol = –3003.494994

P 1.87200700 -0.84623200 0.17057300
Ru 0.00714800 0.38485500 -0.66633100
Cl 0.23061800 -0.70711300 -2.82318600
Cl -0.35483200 1.26018200 1.56433300
C 0.15521300 2.11001200 -1.60918800
H 0.63908100 2.02254200 -2.58331500
H 2.64796600 0.87007500 -2.11144000
C 2.57549500 -0.15470800 1.74702900
C 3.17068700 -1.00206400 -1.15040000
C 1.36191100 -2.58393500 0.59185000
C -0.24838200 -3.97448600 1.95793400
C 0.70746000 -4.89036800 -0.19758900
C -0.50006700 -4.87912700 0.74626200
C 1.09477700 -3.47097900 -0.63704600
C 0.13129100 -2.55465800 1.52158200
H 0.56457900 -4.39494700 2.56864900
H 1.56416400 -5.35836100 0.31023300
H -1.37817500 -4.50719300 0.20356600
H 0.29587900 -3.02913200 -1.24271200
H -0.71534900 -2.09879100 0.98941300
H 2.20209000 -3.02369000 1.14621600
H -1.13720800 -3.93530900 2.59685000
H 0.49503200 -5.50149600 -1.08327800
H -0.73761500 -5.89905800 1.07448100
H 1.98012400 -3.51758400 -1.27962500
H 0.31489000 -1.92010200 2.39504900
C 3.83927500 -0.37530800 3.91101300
C 3.46421800 1.91095100 2.88503800
C 4.40067700 1.04171300 3.73424200
C 3.16940900 1.25022900 1.53304600
C 3.53528000 -1.04217700 2.55866600
H 2.91114500 -0.32578500 4.49783400
H 2.51366300 2.06380700 3.41306100
H 5.38222600 0.98005200 3.24069700
H 4.10334800 1.17300700 0.96097500
H 4.47333700 -1.19635100 2.01298400
H 1.66642400 -0.01175300 2.34158900
H 4.54117600 -0.99733100 4.48052600
H 3.90318900 2.90380500 2.72669500
H 4.57068500 1.50479300 4.71402600
H 2.47287200 1.86885400 0.95883900
H 3.10229800 -2.03300600 2.73383200
C 4.43379800 0.07308800 -3.04467700
C 5.30788100 -2.05187100 -1.97372900
C 5.68182200 -0.74291500 -2.68180100
C 4.42806500 -1.78784200 -0.74189800
C 3.55277700 0.33401700 -1.81501600
H 3.84062100 -0.47173200 -3.79176200
H 4.75989100 -2.70068300 -2.67168100
H 6.32201900 -0.14597300 -2.01534700
H 5.00321000 -1.20683000 -0.01062600
H 4.09633200 0.96881400 -1.10577400
H 2.63195400 -1.57300700 -1.91540600
H 4.72315900 1.02651800 -3.50380200
H 6.21136500 -2.59834100 -1.67591700
H 6.27437200 -0.95416700 -3.58026600
H 4.16222500 -2.73490800 -0.25786600
C 0.25403300 3.41230600 -0.92013500
C -1.00868500 3.83653400 -0.47029400
C 1.38240100 4.19247600 -0.68831900
N -2.01218200 2.91213900 -0.85514500
C -1.15645600 5.02428000 0.23629500
C 1.24667400 5.38230200 0.03260500
H 2.35555300 3.86283000 -1.04186500
C -2.96740400 2.36452600 0.02272300
C -1.42429900 1.84238700 -1.65752600
C -0.01053000 5.78645700 0.49028500
H -2.12681600 5.36905600 0.57124900
H 2.11936600 5.99526300 0.23631400
C -3.02390400 0.96470200 -0.12431000
C -3.79415100 3.03696700 0.91952000
C -2.00843300 0.52101800 -1.07422800
H -1.68998400 1.87024700 -2.71892700

H -0.10839900 6.71733200 1.04112500
C -3.94930200 0.20624700 0.60809900
C -4.69106800 2.27430800 1.66638600
H -3.75929900 4.11311900 1.03011600
H -2.26222300 -0.25345600 -1.79129000
C -4.77185600 0.88470400 1.51476500
C -4.00726800 -1.27614000 0.51102200
H -5.35305800 2.77169600 2.36925600
H -5.49227200 0.31840500 2.09678200
C -3.74887900 -2.00563100 1.68044000
C -4.30501800 -1.97356200 -0.68570700
C -3.77556200 -3.39880100 1.69270900
H -3.50819500 -1.45647700 2.58563000
C -4.27621200 -3.37917000 -0.66176800
C -4.69344600 -1.34129500 -1.96106400
C -4.03022200 -4.09061700 0.50883100
H -3.58432700 -3.93783500 2.61565400
H -4.48722100 -3.91735300 -1.58228100
C -5.33102900 -0.17775000 -2.14170700
H -4.47692100 -1.94718900 -2.84092200
H -4.03695900 -5.17654300 0.49616900
H -5.61810700 0.46787000 -1.31944200
H -5.59563200 0.15408700 -3.14142600

1-TS2:

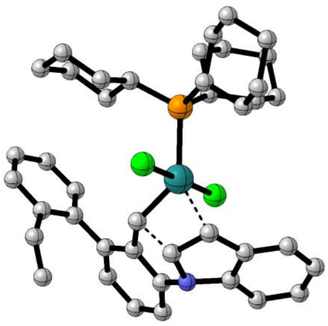

G_sol = –3003.481276

P -1.87991600 0.52230700 0.07874300
Ru 0.01339900 -0.70128800 -0.68420800
Cl -0.42819500 0.00817400 -3.03162700
Cl 0.46595400 -1.50254000 1.58974900
C 0.75872400 -2.63756400 -1.72942000
H 0.19065400 -2.65157200 -2.65130700
H -2.25651100 -1.95288700 -1.48114000
C -2.33284900 0.28783200 1.87675800
C -3.25708800 -0.07326700 -1.02592300
C -1.85373300 2.36698700 -0.14557600
C -0.91370800 4.54807100 0.67032400
C -1.88816100 4.38030600 -1.65876000
C -0.85044400 5.07415000 -0.76797400
C -1.75772400 2.85069900 -1.60142700
C -0.75068100 3.02269500 0.70529200
H -1.88320300 4.81629800 1.11687600
H -2.89809800 4.66998100 -1.33161700
H 0.15380500 4.88151300 -1.16791300
H -0.80371700 2.52999400 -2.03018400
H 0.22958400 2.74101500 0.30263300
H -2.82597700 2.69550400 0.24781200
H -0.14109200 5.01852100 1.28528800
H -1.78648000 4.71692000 -2.69769600
H -0.99847600 6.16140500 -0.78539300
H -2.53656900 2.39373700 -2.21928500
H -0.77481400 2.66307500 1.73956300
C -3.40707200 1.10027700 4.01235800
C -2.97612900 -1.36794800 3.67119100
C -3.88270100 -0.32209300 4.33086500
C -2.86333400 -1.13162300 2.16032100
C -3.27360100 1.33861500 2.49902500
H -2.42722200 1.26735000 4.48224700
H -1.96985700 -1.32236100 4.10867500
H -4.91043800 -0.44927200 3.95903700
H -3.85641100 -1.24837900 1.70736100
H -4.26465300 1.28062600 2.03148700
H -1.35578300 0.35944500 2.37094200
H -4.09341800 1.84065400 4.44230400
H -3.35805500 -2.37879100 3.86021400
H -3.92053300 -0.47399500 5.41667900
H -2.19700700 -1.87590400 1.71899000
H -2.90420400 2.35454700 2.33268900
C -4.25946800 -2.00874200 -2.29760600
C -5.65903900 0.04075400 -1.78460900
C -5.66682400 -1.48007500 -1.98896300
C -4.66111800 0.45560700 -0.69201900
C -3.26073600 -1.60599100 -1.20445500
H -3.91527200 -1.60209300 -3.25836000
H -5.38006800 0.53214700 -2.72747400
H -6.04165300 -1.96388500 -1.07486300
H -4.99325600 0.05053700 0.27186700
H -3.52460300 -2.10205900 -0.26525200
H -2.94899300 0.33874400 -1.99433000
H -4.27701800 -3.10071000 -2.40152800
H -6.66361000 0.39802300 -1.52632600
H -6.36025600 -1.74996900 -2.79472100
H -4.65037600 1.54724800 -0.59148800
C 0.93734700 -3.78509000 -0.86867900
C 2.16678000 -3.64084300 -0.18451100
C 0.10782300 -4.88015200 -0.60514400
N 2.83831200 -2.50201800 -0.66315600
C 2.55667900 -4.54547400 0.79999700
C 0.50249600 -5.79860300 0.36118100
H -0.83483400 -4.99097200 -1.13344600
C 3.53599200 -1.57932100 0.15454900
C 1.99904600 -1.83353300 -1.61754600

C 1.70923600 -5.62110400 1.06028800
H 3.47739700 -4.41905800 1.35572700
H -0.12600300 -6.65470900 0.58598400
C 3.02718800 -0.27423700 0.03257000
C 4.62355900 -1.88342900 0.96363600
C 1.87442700 -0.16951100 -0.90131500
H 2.46386400 -1.52264300 -2.55307700
H 1.99409100 -6.34291900 1.82021300
C 3.66297300 0.77319900 0.71426400
C 5.20877700 -0.84560200 1.69097200
H 5.02356900 -2.88978400 1.00482100
H 2.00705600 0.55247400 -1.70965100
C 4.73686000 0.46032800 1.56383200
C 3.23937700 2.19455400 0.60205000
H 6.05757300 -1.05223300 2.33582200
H 5.22371000 1.26681500 2.10348700
C 2.81714400 2.84032500 1.77268700
C 3.30418700 2.92921500 -0.60675400
C 2.46570500 4.18818100 1.77631400
H 2.76301800 2.25995900 2.68881700
C 2.90046600 4.27605700 -0.58980900
C 3.81545800 2.40839200 -1.89002800
C 2.50571900 4.91027300 0.58386400
H 2.14944900 4.66471000 2.69962500
H 2.93864000 4.84065000 -1.51792600
C 4.77824300 1.49766900 -2.08200800
H 3.38306200 2.88770100 -2.76845400
H 2.22279000 5.95854800 0.56403200
H 5.28979800 1.00257500 -1.26434100
H 5.09073400 1.23400000 -3.08826100

1-Alk2:

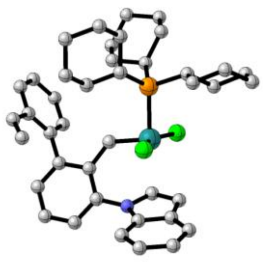

G_sol = –3003.524311

P 1.41036300 -1.17673800 0.13432600
Ru -0.70328800 -0.78969300 -0.71682800
Cl -0.00877600 -1.20882200 -2.95833200
Cl -1.65510400 -1.32466300 1.37391000
C -0.62429300 1.04560900 -0.56714800
H 2.18679600 1.47677100 -0.94755500
C 1.71573600 -0.58087400 1.87688100
C 2.80121700 -0.61603300 -0.99686000
C 1.68429400 -3.02935900 0.28565400
C 1.28615100 -5.19779400 1.52598000
C 1.68319800 -5.23536700 -0.96621100
C 0.96935300 -5.91390100 0.20824400
C 1.33867300 -3.74274200 -1.03515700
C 0.94745100 -3.70060000 1.45923300
H 2.35625900 -5.31745500 1.75294000
H 2.77107600 -5.35212900 -0.84856800
H -0.11606200 -5.88374800 0.03400900
H 0.26458300 -3.62984300 -1.23179800
H -0.13268300 -3.56447500 1.34466000
H 2.75927000 -3.15189000 0.46768400
H 0.73594300 -5.65951200 2.35502900
H 1.41512100 -5.71994300 -1.91296600
H 1.25075800 -6.97243400 0.27046400
H 1.84204600 -3.27056000 -1.88378000
H 1.21900600 -3.23106500 2.40978200
C 3.24799500 -0.53023300 3.89521400
C 1.40921400 1.18292800 3.65630700
C 2.82446000 0.91518300 4.17962100
C 1.28173800 0.86822100 2.15936400
C 3.13866500 -0.85601500 2.39856400
H 2.60030300 -1.21602600 4.46016000
H 0.69337900 0.55871300 4.21043300
H 3.52952200 1.60093500 3.68607300
H 1.88722900 1.58052400 1.58667100
H 3.85888300 -0.23814200 1.84840300
H 1.01578700 -1.20679500 2.44260700
H 4.27439000 -0.70858400 4.23985300
H 1.12357600 2.22531700 3.83138100
H 2.88213600 1.12457200 5.25501600
H 0.24317400 0.99938900 1.84981500
H 3.42193500 -1.90027500 2.22522400
C 4.10170400 1.35523800 -1.92642600
C 5.09735400 -0.95780100 -2.01574000
C 5.39638300 0.53826800 -1.88155100
C 4.10569800 -1.43787200 -0.94490700
C 3.10279800 0.88897100 -0.85875100
H 3.63789200 1.25270500 -2.91783600
H 4.66973100 -1.15340100 -3.00941500
H 5.90679200 0.72145200 -0.92416800
H 4.57211900 -1.35209100 0.04401300
H 3.52228700 1.09854500 0.13281400
H 2.35570500 -0.76662500 -1.98739000
H 4.31321700 2.42067800 -1.78428600
H 6.02234100 -1.54415700 -1.94890600
H 6.08357500 0.86359700 -2.67241700
H 3.90047300 -2.49911900 -1.10954400
H 0.33651600 1.50543600 -0.34609400
C -1.67557400 2.06242100 -0.62580100
C -1.30279100 3.43080000 -0.49694100
C -3.06753600 1.77129600 -0.65711800
C -2.27498600 4.42025400 -0.33743300

C 0.12728500 3.84637900 -0.56119100
C -4.02631900 2.76792700 -0.48269600
N -3.48457300 0.45237000 -0.90278300
C -3.62863200 4.09161500 -0.30923900
H -1.95853800 5.45226100 -0.22699400
C 0.76760300 3.77075000 -1.80567300
C 0.82623200 4.37105500 0.55235300
H -5.07674100 2.50159300 -0.52586700
C -2.84341700 -0.35564900 -1.84795000
C -4.34368300 -0.33334000 -0.15050600
H -4.37642200 4.86911200 -0.18601900
C 2.07480100 4.21873200 -1.98009900
H 0.21680400 3.35934400 -2.64650300
C 2.14437200 4.82159200 0.35054100
C 0.28853200 4.48133300 1.92025700
C -3.31601800 -1.65074000 -1.72560000
H -2.32484700 0.10239600 -2.67574300
C -4.26613600 -1.66232500 -0.65735800
C -5.12638300 -0.01029300 0.96320900
C 2.76686300 4.74985100 -0.89139300
H 2.54593900 4.15170700 -2.95571200
H 2.68747000 5.22961200 1.19919700
C -0.72701000 3.81892000 2.49006800
H 0.84358200 5.17995000 2.54644100
H -3.03096700 -2.47401400 -2.36563800
C -5.02471500 -2.67579800 -0.04744300
C -5.87106800 -1.03176200 1.53596300
H -5.14099300 0.99415500 1.37084700
H 3.78600400 5.10667000 -1.00862600
H -1.32187300 3.07518700 1.97513600
H -0.97820900 4.00243200 3.53059600
C -5.82808700 -2.35085700 1.03406700
H -4.97341000 -3.69541700 -0.41837000
H -6.49563500 -0.81238700 2.39706000
H -6.42475200 -3.12068800 1.51412100

2a-Alk1:

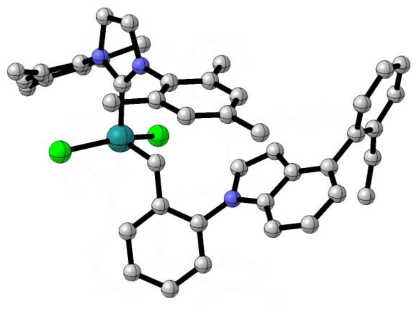

G_sol = –2881.811972

C 2.33838500 -0.82351000 0.92006500
Ru 2.22727400 0.76156200 -0.19963000
Cl 3.59835200 2.05018900 1.20169500
Cl 1.75330300 -0.16334200 -2.30354200
C 0.56185800 1.40939100 0.17783100
H -0.21130800 0.83410600 0.68165300
N 1.52104200 -1.32192600 1.87402500
N 3.44688000 -1.61090800 0.87249100
H 8.42762900 -0.76929100 -2.78841100
C 0.21538400 -0.86884900 2.23232500
C 2.10150400 -2.47706900 2.58085300
C 3.37676500 -2.76711600 1.77401300
C 4.47247900 -1.50255000 -0.12136600
H 1.39506100 -3.31198900 2.58220600
H 2.31427800 -2.20768900 3.62142200
H 4.27412800 -2.81745000 2.39770500
H 3.30582300 -3.69496400 1.19483300
C -2.35357400 -0.04170000 2.93773900
C -0.88807000 -1.42105800 1.56107200
C 0.06814800 0.08737100 3.24592000
C -1.22957000 0.48813500 3.57837400
C -2.16486000 -0.99663800 1.93157200
C -0.68696900 -2.39943200 0.43164800
C 1.27068600 0.69444600 3.92189200
H -1.36414500 1.23338400 4.35865300
H -3.02786600 -1.40670100 1.41589700
C -3.74290900 0.41226900 3.30739400
C 6.42994800 -1.23993000 -2.10103300
C 4.29828300 -2.16398200 -1.35377100
C 5.65025000 -0.79440700 0.17105300
C 6.60578000 -0.66285900 -0.84274400
C 5.28209000 -2.00757100 -2.32851000
C 3.12188900 -3.07378100 -1.60488700
C 5.94457300 -0.25539100 1.54770100
H 7.51260400 -0.09933800 -0.63521500
H 5.14671400 -2.49303100 -3.29202600
C 7.44836200 -1.03952700 -3.19609900
H -0.17468500 -1.91964600 -0.41026700
H -1.64772100 -2.77526900 0.07195300
H -0.07702500 -3.25856300 0.73576900
H 1.89749200 -0.06790900 4.40087000
H 0.96128200 1.40356300 4.69503300
H 1.90383700 1.22402600 3.20206500
H -3.73856200 0.99405700 4.23451900
H -4.41401100 -0.44325500 3.44689400
H -4.17432900 1.03573100 2.51774700
H 5.05319400 -0.22366100 2.17340400
H 6.34134300 0.76177400 1.49450300
H 6.69878200 -0.88477000 2.03962000
H 2.90004200 -3.13136600 -2.67300800
H 2.21842900 -2.72787600 -1.10183300
H 3.34404600 -4.09161400 -1.25400200
H 7.13860200 -0.23245200 -3.87239300
H 7.56691700 -1.94356000 -3.80321700
C 0.14255400 2.70109200 -0.36627600

C -1.17396900 2.86409700 -0.86814100
C 1.00606800 3.81638000 -0.39579200
N -2.05017300 1.75585700 -0.91454400
C -1.60335100 4.10245900 -1.34773300
C 0.56784500 5.05085300 -0.86552400
H 2.00716100 3.70912500 0.00749800
C -3.36332200 1.72331100 -0.45143700
C -1.74831100 0.51495000 -1.48104500
C -0.73847100 5.19589400 -1.33731500
H -2.60613800 4.19185500 -1.75170600
H 1.24454300 5.89981900 -0.85818200
C -3.87550900 0.42561400 -0.72314100
C -4.11514200 2.70042300 0.20855500
C -2.83364100 -0.30996100 -1.38199500
H -0.77599100 0.34978500 -1.92605800
H -1.08172500 6.15453100 -1.71499100
C -5.18315200 0.09393200 -0.30936100
C -5.41049900 2.36097200 0.58639100
H -3.70388200 3.68058000 0.42346400
H -2.89614700 -1.32250900 -1.75021400
C -5.93230000 1.07620100 0.33928300
C -5.69481300 -1.29403100 -0.46282400
H -6.02752000 3.09590000 1.09491100
H -6.94223400 0.83802100 0.66007200
C -5.96074300 -2.01024200 0.71307700
C -5.86161100 -1.93964200 -1.71394000
C -6.35563500 -3.34696200 0.68710600
H -5.82889800 -1.50109400 1.66346700
C -6.23278900 -3.29642200 -1.71543600
C -5.71168900 -1.29449900 -3.03081500
C -6.48054900 -3.99803500 -0.53925900
H -6.54723700 -3.87587600 1.61633600
H -6.34863300 -3.79938600 -2.67237400
C -5.80327500 0.00476500 -3.33851100
H -5.55952900 -1.99919600 -3.84857800
H -6.77200200 -5.04362700 -0.58172300
H -5.98144000 0.77425400 -2.59693800
H -5.70205800 0.33093700 -4.36948000

2a-Coordination:

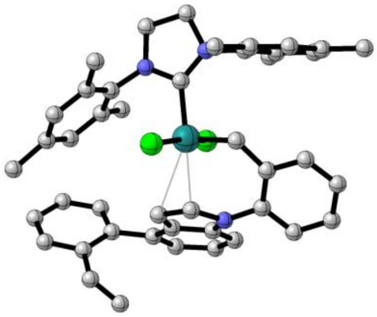

G_sol = –2881.815616

C 1.37523800 -1.77164800 0.48207300
Ru 0.49668600 -0.21300300 -0.39284900
Cl 0.29588700 -1.24880500 -2.55384700
Cl 0.14433100 0.76363600 1.75495400
C 1.99907800 0.77862200 -0.76902800
H 2.93916900 0.25153600 -0.92534500
N 2.59808200 -2.00360700 1.01278400
N 0.58063300 -2.82033400 0.81636700
H -4.73275100 -4.04486400 -2.20968000
C 3.79498100 -1.27647700 0.73114300
C 2.69377100 -3.31893500 1.67453000
C 1.22759300 -3.77589700 1.72326000
C -0.74903700 -2.98791000 0.30501000
H 3.14182300 -3.21434200 2.66597900
H 3.32511700 -3.99160200 1.08096400
H 1.08675800 -4.80205100 1.37396800
H 0.79683300 -3.68955700 2.72828200
C 6.13721100 0.14124800 0.17253100
C 4.27256300 -0.35303800 1.67311200
C 4.46368900 -1.51284200 -0.48146000
C 5.63304000 -0.79144000 -0.73995400
C 5.44310400 0.34763300 1.36912000
C 3.52112700 -0.09702600 2.95275600
C 3.88699000 -2.44557200 -1.51687500
H 6.15334900 -0.95367100 -1.68097900
H 5.81555000 1.07928300 2.08218600
C 7.41065700 0.89814200 -0.11842300
C -3.27086700 -3.14386000 -0.89191500
C -1.85006600 -2.40463400 0.95324300
C -0.90774800 -3.74418500 -0.87360000
C -2.17293100 -3.81003000 -1.44988200
C -3.09717500 -2.46865800 0.31522700
C -1.76410300 -1.81280800 2.33685600
C 0.26249000 -4.45526300 -1.50214800
H -2.30040300 -4.36937800 -2.37336900
H -3.94879600 -1.99282200 0.79013000
C -4.61063400 -3.15314300 -1.58595800
H 2.50262300 0.24762100 2.74390800
H 4.03180100 0.65940100 3.55580600
H 3.43689700 -1.00766100 3.55915700
H 3.64018000 -3.42681900 -1.09642200
H 4.59561100 -2.59946300 -2.33565500
H 2.95972200 -2.03914100 -1.94063800
H 7.59957800 0.96475600 -1.19464900
H 8.27840500 0.40271500 0.33651500
H 7.37057700 1.91551100 0.28542900
H 1.06516000 -3.75183100 -1.73333000
H -0.03901500 -4.93657000 -2.43642600
H 0.66619900 -5.23367600 -0.84131800
H -2.19462800 -0.80990300 2.36504000
H -0.73670000 -1.72833100 2.68838900
H -2.32890400 -2.44361700 3.03618200

H -4.71381900 -2.27727200 -2.23950600
H -5.43407500 -3.11418000 -0.86663400
C 2.19132300 2.22226200 -0.88008000
C 1.16328700 3.20284400 -0.87248600
C 3.52625300 2.68171700 -0.91782200
N -0.18683200 2.79863200 -0.89961000
C 1.47653000 4.56204000 -0.87096700
C 3.83750800 4.03618200 -0.89694100
H 4.31944300 1.94041700 -0.92811000
C -1.16579800 3.10620900 0.03953400
C -0.58951700 1.65281000 -1.59799500
C 2.80738700 4.97907500 -0.86658300
H 0.67078100 5.28740900 -0.90246600
H 4.87484600 4.35691200 -0.90926400
C -2.21244700 2.15248400 -0.09265900
C -1.18405000 4.10462700 1.01811000
C -1.82044900 1.23858900 -1.12568700
H -0.08492100 1.37659500 -2.51079800
H 3.03567500 6.04088600 -0.86362100
C -3.33523400 2.22299700 0.76168200
C -2.30044400 4.16501800 1.83883800
H -0.35550500 4.79156400 1.14237900
H -2.39665800 0.41904600 -1.52796800
C -3.35826200 3.24509800 1.71054200
C -4.42631900 1.21308700 0.73974600
H -2.35950100 4.93444400 2.60311000
H -4.21910000 3.32989600 2.36688500
C -4.70357400 0.55426300 1.94810800
C -5.18913700 0.88029800 -0.40847000
C -5.69113200 -0.42383200 2.04643000
H -4.10730900 0.81077900 2.81823000
C -6.15248200 -0.13843500 -0.29669300
C -5.10505400 1.55885500 -1.71570400
C -6.41560700 -0.78093400 0.90947500
H -5.87596100 -0.91501800 2.99737900
H -6.72776600 -0.40368100 -1.18000400
C -4.75164400 2.82502700 -1.96452300
H -5.44166900 0.94849900 -2.55406600
H -7.17899200 -1.55217400 0.95970600
H -4.43599600 3.50816000 -1.18432200
H -4.77536000 3.21399500 -2.97815400

2a-TS1:

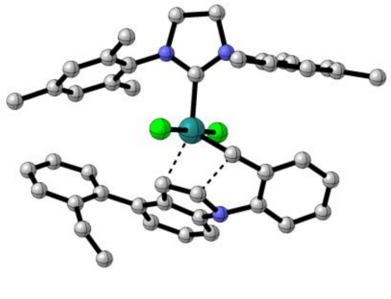

G_sol = –2881.783817

Ru 0.33098800 -0.12584900 -0.42132500
Cl -0.13800200 -1.22226700 -2.54110000
Cl 0.63113300 0.96081100 1.71827400
C 1.66136600 0.87402500 -1.30645000
H 1.98218500 0.44938800 -2.26472500
C 2.44839900 2.03967400 -0.85134000
C 1.71755700 3.22664400 -0.67988000
C 3.82876300 2.04749700 -0.66571100
N 0.33250600 3.06870400 -0.94825800
C 2.34505900 4.41198900 -0.31423200
C 4.46205200 3.22382200 -0.25825300
H 4.39394100 1.13720900 -0.81503100
C -0.68018400 3.32724300 -0.00715500
C 0.01657500 1.84780400 -1.62173400
C 3.72507000 4.39665300 -0.08558000
H 1.78386000 5.33615200 -0.23966900
H 5.53473600 3.22497800 -0.08772200
C -1.68693400 2.34122900 -0.11223400
C -0.75793000 4.38099000 0.89976200
C -1.22490300 1.33298200 -1.05934700
H 0.16499900 1.80703300 -2.69820100
H 4.22564900 5.31537000 0.20574100
C -2.87459100 2.47375900 0.63277500
C -1.91368500 4.47539700 1.66772700
H 0.04306800 5.10101500 1.00559500
H -1.89622900 0.73634600 -1.66143700
C -2.95852000 3.55532200 1.52057900
C -4.03978200 1.55258200 0.54822800
H -2.02010200 5.29292800 2.37476200
H -3.87210900 3.69103200 2.09043300
C -4.55399100 1.05794800 1.75814000
C -4.70735400 1.22292100 -0.65778600
C -5.69763800 0.26487200 1.80479000
H -4.03068700 1.30602300 2.67610600
C -5.82492200 0.37190500 -0.59386100
C -4.38029500 1.76839000 -1.99139300
C -6.33500100 -0.08850200 0.61558600
H -6.07393500 -0.08932200 2.76039400
H -6.32875000 0.11174100 -1.52129100
C -3.94812200 3.00141700 -2.27836200
H -4.59886000 1.09327400 -2.81900000
H -7.21887700 -0.71959000 0.62776800
H -3.74386700 3.74064500 -1.51150300
H -3.79046300 3.30583400 -3.30889100
C 0.97722600 -1.81039700 0.51165500
N 2.21616800 -2.13590300 0.93399000
N 0.11867600 -2.75751600 0.93280700
C 3.46178600 -1.54140300 0.55182700
C 2.24421300 -3.46212800 1.58806800
C 0.75581200 -3.74502800 1.82033800
C -1.24417800 -2.87644700 0.50306200

C 4.20332400 -0.83975600 1.51660500
C 3.97960400 -1.80403700 -0.72942200
H 2.82144000 -3.41848500 2.51365600
H 2.71406100 -4.19375500 0.91888800
H 0.45850900 -4.75903600 1.54467900
H 0.45304200 -3.56175500 2.85802500
C -2.26586100 -2.21670100 1.20207100
C -1.51350900 -3.70648200 -0.60023300
C 5.47977700 -0.38921100 1.16624800
C 3.64858900 -0.58101000 2.89222400
C 5.25747200 -1.32435800 -1.03419500
C 3.20136300 -2.60325400 -1.74308200
C -3.57450600 -2.35980400 0.72953400
C -1.98514600 -1.40316200 2.44096800
C -2.83489100 -3.81989500 -1.02913900
C -0.40989400 -4.44596700 -1.31122100
C 6.02313000 -0.61895400 -0.10015300
H 6.05935800 0.16067300 1.90369600
H 2.66476200 -0.10697200 2.82478200
H 4.31782500 0.07287000 3.45877400
H 3.53371800 -1.51075700 3.46491000
H 5.66859800 -1.52216100 -2.02143400
H 2.87840400 -3.56762200 -1.33291800
H 3.81576600 -2.80603100 -2.62506200
H 2.29471500 -2.08370000 -2.07017000
C -3.87507900 -3.14067600 -0.38794700
H -4.37430900 -1.85090800 1.25342000
H -2.91913300 -1.17272100 2.95986300
H -1.47952700 -0.45940800 2.21312700
H -1.33182400 -1.94285500 3.13593400
H -3.05520000 -4.44708500 -1.88955600
H 0.29995800 -3.73949900 -1.74912300
H -0.81930600 -5.05613700 -2.12114300
H 0.14095000 -5.11467100 -0.63781700
C 7.38289200 -0.07746800 -0.46817600
C -5.29059300 -3.24503700 -0.89983900
H 7.29919800 0.92129500 -0.91768500
H 7.88653600 -0.71951000 -1.19835900
H 8.02886500 0.01399600 0.41094500
H -5.50092800 -2.44830200 -1.62415500
H -6.01829200 -3.14231900 -0.08876700
H -5.46505000 -4.20240900 -1.40204200

2a-Metallacycle:

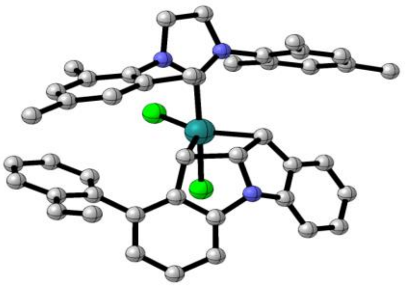

G_sol = –2881.787365

Ru 0.40479700 -0.09919400 -0.45479500
Cl -0.14739100 -1.18449500 -2.55111600
Cl 0.78563300 0.95193100 1.69343300
C 1.60961400 1.07198300 -1.49625500
H 1.90171000 0.60513000 -2.43708900
C 2.54474400 2.05316500 -0.91385200
C 1.86430200 3.21779300 -0.52088400
C 3.91998400 1.95027200 -0.74617800
N 0.49191400 3.11808200 -0.84977200
C 2.53997600 4.27772500 0.07308900
C 4.60732600 3.00258000 -0.13582000
H 4.43535000 1.04984700 -1.05556000
C -0.57516200 3.38809600 0.01798100
C 0.23255900 1.87598800 -1.57318500
C 3.91997100 4.14898200 0.27137100
H 2.03120400 5.18990000 0.35870200
H 5.67911800 2.92761500 0.02324500
C -1.55236900 2.37117100 -0.06448900
C -0.73702100 4.51162600 0.82416100
C -1.05989300 1.28567000 -0.92288300
H 0.02127500 2.00920700 -2.63893500
H 4.46345000 4.96732600 0.73502200
C -2.79011100 2.54395700 0.58290800
C -1.93303600 4.63343200 1.52633900
H 0.02247400 5.27979700 0.88603100
H -1.75737000 0.80644800 -1.60129700
C -2.94649500 3.68156300 1.39098600
C -3.95762200 1.62770500 0.46852900
H -2.09776300 5.50328500 2.15555000
H -3.89662700 3.83739100 1.89133500
C -4.56378000 1.20509100 1.66284500
C -4.55646100 1.26340100 -0.76260600
C -5.73457000 0.45168200 1.66869300
H -4.09558100 1.48392900 2.60165700
C -5.70271600 0.44972200 -0.73565900
C -4.12645500 1.74153200 -2.09344500
C -6.30588000 0.06428500 0.45680300
H -6.18490500 0.15561100 2.61201700
H -6.15356000 0.16238700 -1.68215600

C -3.67125100 2.96093200 -2.40297700
H -4.28032200 1.02640400 -2.90166100
H -7.21032400 -0.53652700 0.43884800
H -3.52821800 3.73601400 -1.65771800
H -3.43290700 3.21745800 -3.43112600
C 0.86702100 -1.82229300 0.51806500
N 2.09535200 -2.20203000 0.92305000
N -0.03209300 -2.71901600 0.95102300
C 3.35971500 -1.65293300 0.52635300
C 2.06820500 -3.52410000 1.58473900
C 0.57175600 -3.72618900 1.84375900
C -1.40465500 -2.80613400 0.54685600
C 4.14097300 -0.98359200 1.48413000
C 3.85291100 -1.93376700 -0.76103900
H 2.66285700 -3.50288300 2.49969900
H 2.48814100 -4.28232000 0.91203500
H 0.21635800 -4.72594900 1.58664600
H 0.29439200 -3.51158600 2.88233800
C -2.39302200 -2.12573800 1.27085600
C -1.71769600 -3.65244100 -0.53123100
C 5.41945800 -0.56066600 1.11070900
C 3.63546700 -0.74205500 2.88156300
C 5.13865200 -1.48850500 -1.08497700
C 3.04790800 -2.71892100 -1.76472400
C -3.72145400 -2.28917500 0.86802500
C -2.04661100 -1.26155600 2.45700600
C -3.05736900 -3.77660400 -0.89817400
C -0.64466900 -4.40244800 -1.27746500
C 5.93413500 -0.79813800 -0.16597600
H 6.02623300 -0.02997600 1.84014500
H 2.64456500 -0.28117000 2.85718300
H 4.31624600 -0.08112300 3.42553000
H 3.56290100 -1.67851500 3.45101800
H 5.53055800 -1.70138200 -2.07688900
H 2.62987200 -3.63133600 -1.32424100
H 3.67586800 -3.01350400 -2.61059900
H 2.20055600 -2.14492900 -2.15385800
C -4.07075700 -3.09751200 -0.21578400
H -4.49819600 -1.77154100 1.41707700
H -2.95598200 -0.93894800 2.97013200
H -1.48292300 -0.36920900 2.16554700
H -1.42060900 -1.79965300 3.17863400
H -3.31432400 -4.42057600 -1.73584000
H 0.03260500 -3.70165800 -1.77340200
H -1.08916400 -5.04282600 -2.04454600
H -0.04914300 -5.04317600 -0.61507900
C 7.29778000 -0.28413400 -0.55703200
C -5.50951200 -3.23217500 -0.64995700
H 7.76409400 -0.91957300 -1.31707700
H 7.96933600 -0.23218300 0.30607900
H 7.22835300 0.72822600 -0.97779300
H -5.70416500 -4.21659800 -1.08863600
H -5.76001900 -2.47823500 -1.40649000
H -6.19613500 -3.08740100 0.18994900

2a-TS2:

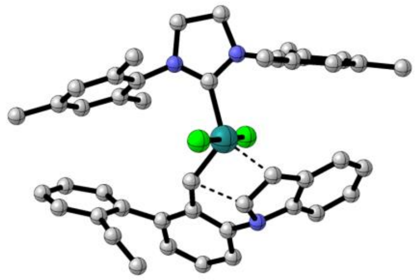

G_sol = –2881.774347

Ru -0.59242700 -0.05432400 0.56144600
Cl -0.03591000 -1.06326000 2.71161400
Cl -1.07092000 0.91917600 -1.60323900
C -1.92453400 1.26132100 1.75619500
H -2.22952500 0.62583400 2.57885600
C -2.84247100 2.05067200 0.94460900
C -2.11043200 3.12239000 0.39267300
C -4.18799200 1.87766600 0.62709000
N -0.79939300 3.09716700 0.92067300
C -2.68639000 3.99812600 -0.52151000
C -4.78121200 2.76555700 -0.26715400
H -4.74232000 1.04949500 1.05070100
C 0.36468700 3.38412300 0.16782800
C -0.68438700 1.96897100 1.76582000
C -4.03140500 3.80334500 -0.84077100
H -2.11671600 4.79361500 -0.98618000
H -5.82800900 2.64854900 -0.53214400
C 1.30178800 2.32688100 0.06297900
C 0.59057300 4.64432500 -0.36851900
C 0.93891600 1.03431800 0.70857900
H -0.01719800 2.01397800 2.61853700
H -4.50662800 4.47805700 -1.54722800
C 2.55095800 2.62023300 -0.52311900
C 1.78022800 4.87980800 -1.05527600
H -0.13454700 5.43487700 -0.21423500
H 1.67200900 0.66159700 1.42897400
C 2.74708400 3.88369800 -1.11326100
C 3.75029200 1.73322400 -0.52475700
H 1.97498200 5.85514400 -1.49078400
H 3.70875300 4.09300900 -1.57006900
C 4.36672500 1.47512300 -1.75910800
C 4.40006900 1.31804600 0.66227900
C 5.60665600 0.84764400 -1.84334100
H 3.85994400 1.79795200 -2.66351800
C 5.62373200 0.63564900 0.55237700
C 3.93303300 1.61491000 2.03383200
C 6.24202300 0.42646200 -0.67540900

H 6.06661100 0.68185600 -2.81349400
H 6.11815100 0.31304600 1.46496300
C 3.39868900 2.76043900 2.47333600
H 4.12178800 0.82157900 2.75714100
H 7.20652000 -0.07029300 -0.71992100
H 3.21715800 3.60858500 1.82185800
H 3.13534300 2.87942900 3.52042000
C -0.63803900 -1.86679000 -0.41251900
N -1.81409100 -2.39011000 -0.82818200
N 0.34116000 -2.71689200 -0.74785500
C -3.12671100 -1.89019600 -0.54134700
C -1.66138200 -3.73362300 -1.41743900
C -0.14238100 -3.84190100 -1.57459000
C 1.73313600 -2.64770900 -0.42857500
C -3.87676100 -1.30977600 -1.58053800
C -3.68949500 -2.12716000 0.72743400
H -2.19683900 -3.79403700 -2.36749300
H -2.07242900 -4.48960200 -0.73721100
H 0.26645900 -4.78559400 -1.20751200
H 0.18500000 -3.70164900 -2.61126000
C 2.59998900 -1.96179400 -1.28862200
C 2.20130900 -3.41008800 0.65485600
C -5.17835800 -0.89198900 -1.29819000
C -3.33204700 -1.19535300 -2.97992700
C -5.00011900 -1.69376700 0.95703900
C -2.94591800 -2.88035900 1.80226100
C 3.97229100 -2.06709000 -1.04867200
C 2.06695200 -1.15998800 -2.44914200
C 3.57970700 -3.46450700 0.86665600
C 1.25250200 -4.16161800 1.55253900
C -5.75424300 -1.06585900 -0.03705000
H -5.75810600 -0.42221100 -2.08888100
H -2.27320400 -0.93552500 -2.97161200
H -3.86506000 -0.41924300 -3.53608200
H -3.46396900 -2.14035800 -3.52631500
H -5.44608400 -1.87364000 1.93259600
H -2.46006000 -3.77749500 1.40254700
H -3.63744000 -3.19622600 2.58900500
H -2.15592300 -2.28116500 2.26567000
C 4.47915500 -2.81011600 0.01991100
H 4.65768900 -1.55975200 -1.71616900
H 2.88809300 -0.71077500 -3.01295800
H 1.39767000 -0.35949300 -2.11619000
H 1.48584800 -1.78470800 -3.13924100
H 3.95927800 -4.05138400 1.70006800
H 0.57274900 -3.46770000 2.05600300
H 1.80605400 -4.71646700 2.31564000
H 0.64346200 -4.88435200 0.99450900
C -7.14792600 -0.56358700 0.24862100
C 5.96513500 -2.91365200 0.26246500
H -7.78674300 -0.62801700 -0.63835600
H -7.12921000 0.49019600 0.55863000
H -7.62237300 -1.13136500 1.05554100
H 6.24687500 -3.92416200 0.57866000
H 6.28561200 -2.22354400 1.05276600
H 6.53490000 -2.66555700 -0.63792900

2a-Alk2:

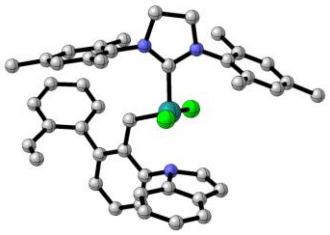

G_sol = –2881.810857

C -0.94439900 -1.74072700 0.72454400
Ru -0.84380300 0.12165700 0.04257900
Cl -1.10890300 -0.40983600 -2.28714900
Cl -1.35384800 0.77732100 2.25455100
C 0.95254400 0.38982900 -0.17574900
H 1.73373900 -0.29336700 0.15333800
N -0.08802300 -2.45938600 1.48718400
N -2.12189400 -2.41749100 0.68829200
H -7.00900400 -1.82092300 -3.14206900
C 1.32229900 -2.25466300 1.59334600
C -0.65571600 -3.74501200 1.93189700
C -2.13716900 -3.59819800 1.56302400
C -3.26493800 -2.06160800 -0.09963900
H -0.49351000 -3.88263100 3.00364700
H -0.16846700 -4.57216500 1.40077000
H -2.53848800 -4.46648900 1.03360600
H -2.76888100 -3.40517700 2.43846500
C 4.08701100 -1.89579300 1.78327000
C 1.86027000 -1.72959200 2.77861700
C 2.13825600 -2.63710600 0.51700600
C 3.51811500 -2.45057000 0.63396300
C 3.24433300 -1.54842300 2.84383400
C 0.97889500 -1.37318900 3.94683500
C 1.53468400 -3.18207700 -0.75328100
H 4.15746200 -2.72538500 -0.19751800
H 3.67428600 -1.12965200 3.75095200
C 5.57389000 -1.65913500 1.87766300
C -5.42832800 -1.36847000 -1.73345200
C -4.26511200 -1.22397200 0.41311200
C -3.36780000 -2.62527700 -1.38857300
C -4.45044900 -2.26598200 -2.18375300
C -5.32713400 -0.87586000 -0.43524600
C -4.28207100 -0.73923400 1.84089500
C -2.32334400 -3.58483200 -1.89708200
H -4.52777300 -2.68092300 -3.18591400
H -6.10044200 -0.21281400 -0.05306700

C -6.56160000 -0.95522600 -2.64005800
H 0.18166800 -0.68925800 3.64301700
H 1.56500400 -0.90097500 4.74082400
H 0.50232300 -2.26500400 4.37520600
H 0.83996400 -4.00646900 -0.55724300
H 2.31565400 -3.54774400 -1.42374400
H 0.96809400 -2.40731500 -1.28270300
H 6.11837700 -2.24323800 1.12997200
H 5.95836500 -1.92423700 2.86898800
H 5.80513700 -0.60107100 1.70536300
H -1.35126300 -3.09034300 -1.96644800
H -2.58981100 -3.94861700 -2.89352800
H -2.21429100 -4.45690100 -1.24025800
H -4.24497100 0.35320600 1.88933800
H -3.42872700 -1.10160500 2.41228800
H -5.20649800 -1.06724400 2.33261700
H -6.20646900 -0.27536000 -3.42491500
H -7.35065600 -0.43835100 -2.08500200
C 1.39457000 1.54647900 -0.94708100
C 2.67476700 1.62898800 -1.54566100
C 0.49985000 2.61619500 -1.14136500
C 3.03765000 2.79751900 -2.22679200
C 3.57758800 0.44821800 -1.65226400
C 0.86088800 3.76604700 -1.82732600
N -0.80917300 2.51393100 -0.52996200
C 2.14778900 3.86088300 -2.36164400
H 4.02201400 2.85040000 -2.68112500
C 3.13556500 -0.60503600 -2.46557800
C 4.89556200 0.42573800 -1.13996200
H 0.14442000 4.57502900 -1.92925700
C -1.98987000 2.58006800 -1.34053800
C -1.05111100 3.38923200 0.57419000
H 2.44695900 4.75441300 -2.90126900
C 3.99168400 -1.64284800 -2.83000500
H 2.11649500 -0.57685400 -2.84208500
C 5.74872100 -0.61667100 -1.54014300
C 5.42704900 1.41872300 -0.19064700
C -2.94588000 3.31297900 -0.71923500
H -2.00644100 2.01749400 -2.26036600
C -2.38016700 3.84950400 0.50339900
C -0.17531800 3.76913200 1.58070700
C 5.31273800 -1.63731500 -2.38070200
H 3.63284000 -2.43621000 -3.47899300
H 6.76943800 -0.62586800 -1.16660500
C 4.73322600 2.12630000 0.70988300
H 6.51205600 1.52374100 -0.20462800
H -3.95871100 3.45276700 -1.07295600
C -2.86596900 4.68646200 1.51536000
C -0.66933900 4.62040100 2.56793600
H 0.84265300 3.39584500 1.60813400
H 5.99803700 -2.42672200 -2.67632400
H 3.65638300 2.04608400 0.81004800
H 5.23782800 2.80932900 1.38668300
C -2.00180600 5.06501200 2.54029900
H -3.89182700 5.04344300 1.49336900
H -0.01701900 4.93283000 3.37792400
H -2.36076200 5.71946700 3.32954200

2b-Coordination:

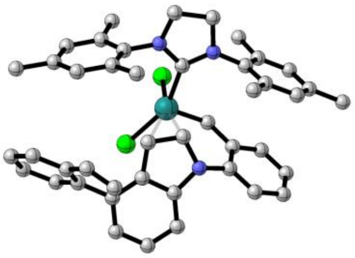

G_sol = –2881.804022

C -0.94139700 -1.82441000 0.33502300
Ru -0.30683500 -0.38020900 -0.95958200
Cl -0.84663400 -1.87227400 -2.77364200
Cl 1.25194900 0.62921300 -2.44817000
C -1.94337500 0.43667000 -1.22618800
H -2.50682800 -0.01530900 -2.04888100
N -2.15497900 -2.08954400 0.88080600
N -0.08772400 -2.78387500 0.76693000
H 6.13045800 -2.41623800 1.00952700
C -3.38086800 -1.36470100 0.74296900
C -2.17868100 -3.34152800 1.66266000
C -0.70489100 -3.76864700 1.66785300
C 1.33442600 -2.77235800 0.58793500
H -2.82786800 -4.07440300 1.17291800
H -2.57512200 -3.15105500 2.66478900
H -0.24969900 -3.70741900 2.66254200
H -0.55037600 -4.78111900 1.28296200
C -5.84636200 -0.04295400 0.55380100
C -4.26125300 -1.69489700 -0.30438200
C -3.74506800 -0.43137500 1.73013700
C -4.97168400 0.22409800 1.60789900
C -5.47746100 -1.01158600 -0.38320800
C -3.92466900 -2.77172300 -1.30056200
C -2.88201100 -0.12889900 2.93168000
H -5.24714500 0.96419600 2.35605200
H -6.15740900 -1.25085400 -1.19723400
C -7.15447000 0.69813300 0.43970200
C 4.12099300 -2.79144100 0.30274800
C 1.90894100 -3.47518900 -0.48628200
C 2.13556900 -2.14371800 1.56256400
C 3.52087700 -2.14718600 1.38859300
C 3.30079200 -3.45594300 -0.61011500
C 1.09054800 -4.27020000 -1.46786200
C 1.54432500 -1.53131200 2.81161500

H 4.14527400 -1.64496700 2.12241500
H 3.75457100 -3.99113700 -1.44110300
C 5.61767100 -2.80127100 0.12358700
H -2.96323900 -2.58961500 -1.79067000
H -4.69817100 -2.83582200 -2.07128500
H -3.86393300 -3.75475000 -0.81504300
H -1.99546200 -0.76504300 2.97784900
H -3.44940600 -0.27751500 3.85764400
H -2.55053600 0.91587500 2.92456900
H -6.98014400 1.77455100 0.32539400
H -7.77015900 0.56323200 1.33702900
H -7.73516400 0.35611300 -0.42222000
H 0.52263900 -1.17797100 2.65707000
H 2.14317300 -0.68200200 3.15364300
H 1.51959600 -2.26504300 3.62848700
H 0.93352300 -3.70330800 -2.38962000
H 0.09583400 -4.50118600 -1.08114200
H 1.60363200 -5.20657400 -1.71246800
H 5.90870600 -2.17245700 -0.72433200
H 5.98417600 -3.81548100 -0.07302000
C -2.62645200 1.61584300 -0.70644100
C -2.07033200 2.51914800 0.22874900
C -3.90992700 1.91180100 -1.20673400
N -0.73934900 2.31753500 0.66534900
C -2.77969000 3.63559700 0.66414100
C -4.6166300 3.03520700 -0.78779400
H -4.34295900 1.23920500 -1.93972900
C 0.33294600 3.07611000 0.17576200
C -0.26593700 1.01826500 0.93947500
C -4.05475000 3.89542200 0.15718300
H -2.32119000 4.29742600 1.39202500
H -5.59945900 3.24204800 -1.20027400
C 1.49812500 2.27991100 0.22775400
C 0.34527500 4.37739100 -0.32669400
C 1.10670100 0.97896100 0.70053600
H -0.83775700 0.42238300 1.62650500
H -4.60049100 4.77157900 0.49483200
C 2.73878700 2.81051300 -0.16190600
C 1.57820100 4.88750900 -0.72334600
H -0.56297800 4.96455200 -0.40412400
H 1.79071000 0.19733800 0.98205300
C 2.75552300 4.12505000 -0.63169000
C 3.98530100 2.00186100 -0.13821700
H 1.63386600 5.90127800 -1.10930400
H 3.70183600 4.56058400 -0.93721800
C 4.58973500 1.73594000 -1.37385900
C 4.58091200 1.50180100 1.04666100
C 5.77486600 1.00935200 -1.46641700
H 4.09358300 2.08978100 -2.27096300
C 5.76601800 0.75290700 0.92552600
C 4.09115200 1.71794000 2.42055100
C 6.37131200 0.52063600 -0.30497400
H 6.22027100 0.82071100 -2.43876100
H 6.23150800 0.37051800 1.83100800
C 3.27469700 2.66562300 2.90129900
H 4.51619000 1.01725900 3.14087300
H 7.29776500 -0.04399800 -0.35539900
H 2.82916900 3.43374800 2.28193700
H 3.04707400 2.69920700 3.96308500

2b-TS1:

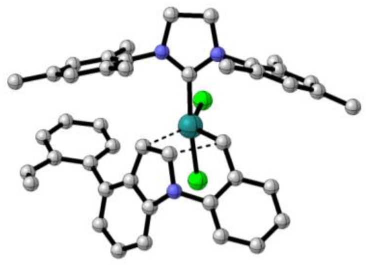

G_sol = –2881.767913

C 2.08032700 0.84834500 -0.12070000
H 2.82965000 0.17737500 -0.53575300
C 2.50430900 2.25075200 -0.23748100
C 1.66668700 3.15523700 -0.91842500
C 3.76605000 2.67932200 0.18887600
N 0.40531500 2.63234500 -1.32679200
C 2.07527300 4.46056800 -1.16435300
C 4.16013100 4.00256500 -0.01092200
H 4.41918100 1.97541200 0.69721000
C -0.80516800 3.11251600 -0.75016100
C 0.32126700 1.22325000 -1.38595100
C 3.31929600 4.88673200 -0.68920200
H 1.44275200 5.12960000 -1.73692500
H 5.12993700 4.33789200 0.34413500
C -1.62700900 2.01711700 -0.42608400
C -1.19108900 4.42124900 -0.50333200
C -0.90905300 0.79349100 -0.79487600
H 0.83087200 0.72988500 -2.20282200
H 3.63879800 5.90816700 -0.87309900
C -2.86809400 2.21479700 0.18485300
C -2.45359500 4.61538000 0.06486500
H -0.54381500 5.26133000 -0.72239400
H -1.43922300 -0.09062400 -1.11722600
C -3.27685400 3.53875400 0.40056300
C -3.65447000 1.09061500 0.76225600
H -2.79366200 5.62730700 0.26516100
H -4.23943700 3.71851800 0.86796200
C -3.06608600 0.40901300 1.83705700
C -4.98651800 0.79070200 0.39010200
C -3.78520700 -0.52984300 2.57526000
H -2.05245900 0.66885900 2.12810000
C -5.69894200 -0.14093700 1.16561000

C -5.68587700 1.37567000 -0.76687500
C -5.11501500 -0.79371200 2.24820100
H -3.30756600 -1.02753900 3.41354800
H -6.73031600 -0.35889800 0.89830900
C -5.15298800 1.90076400 -1.87838900
H -6.77287900 1.31093900 -0.70662900
H -5.69600500 -1.50445900 2.83008800
H -4.08317100 1.98275900 -2.03195100
H -5.79299000 2.27262200 -2.67345300
Ru 0.57488700 0.03139600 0.64610000
C 0.91357400 -1.86694800 -0.15307500
N 2.10581700 -2.52712100 -0.15240400
N -0.03777900 -2.79689100 -0.42268600
C 1.98444400 -3.98387900 -0.33333000
C 3.41882500 -1.95817000 -0.13643000
C -1.41014800 -2.57064400 -0.76365900
C 0.48727700 -4.16317300 -0.60040700
H 2.31674000 -4.49849400 0.57463200
H 2.61432300 -4.31976400 -1.16330000
C 3.97559000 -1.56277700 -1.37247600
C 4.13647800 -1.81804900 1.05770200
C -2.42124300 -2.85703300 0.16410000
C -1.71806200 -2.18535500 -2.08364500
H 0.00977500 -4.84598800 0.10720900
H 0.27119600 -4.51713800 -1.61488300
C 5.22846800 -0.95426100 -1.38070200
C 3.19704400 -1.71524700 -2.65524100
C 3.61783600 -2.30016700 2.38635600
C 5.39014700 -1.18948600 1.00075400
C -3.75051500 -2.68437200 -0.23825900
C -2.10340600 -3.37708900 1.54083700
C -3.05675400 -2.00991400 -2.43279300
C -0.62807300 -1.96185600 -3.10193600
C 5.94296000 -0.73549900 -0.19491100
H 5.65049700 -0.62608200 -2.32774900
H 2.82068700 -2.73405600 -2.79658400
H 3.81902300 -1.46145100 -3.51801000
H 2.32227100 -1.05514400 -2.66662500
H 2.73544400 -2.93056300 2.28182100
H 4.39988200 -2.85660800 2.91575600
H 3.31541600 -1.45701100 3.01490900
H 5.94339600 -1.05518200 1.92749700
C -4.08985000 -2.24846400 -1.51801400
H -4.53896800 -2.88391200 0.47902200
H -2.98942000 -3.33391800 2.17860700
H -1.77773600 -4.42560200 1.49947800
H -1.30449800 -2.80062500 2.01343700
H -3.30088600 -1.69139400 -3.44369400
H -0.00161500 -2.85244100 -3.23087600
H 0.04166900 -1.15001200 -2.80650000
H -1.05516900 -1.70805900 -4.07603600
C 7.26773100 -0.01385700 -0.22183300
C -5.52854900 -2.04608500 -1.91962000
H 7.91819900 -0.39923200 -1.01511800
H 7.79651700 -0.11216900 0.73085800
H 7.12373400 1.05720900 -0.41379500
H -5.76771800 -2.60081800 -2.83496200
H -5.72823300 -0.98612500 -2.11191500
H -6.21011800 -2.37790900 -1.13134100
Cl 0.49578700 -1.13643300 2.68504300
Cl 0.28659700 2.03310500 2.03159300

2b-Metallacycle:

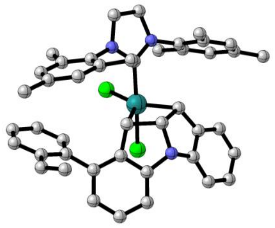

G_sol = –2881.777161

C 1.95493600 0.82904600 -0.64974500
H 2.51571600 0.07419500 -1.18380000
C 2.64370300 2.11506100 -0.48279100
C 1.78774400 3.17776800 -0.82092800
C 3.94851900 2.36173400 -0.06217900
N 0.55036200 2.68107200 -1.28940100
C 2.20206100 4.50042700 -0.70036400
C 4.37274100 3.68483800 0.06275400
H 4.59787200 1.53248100 0.19996100
C -0.71025100 3.14063600 -0.84113100
C 0.57228100 1.22397700 -1.29290500
C 3.50265900 4.73761000 -0.24784400
H 1.54790300 5.32653700 -0.94958700
H 5.37891800 3.90182000 0.40870900
C -1.50049600 2.06292000 -0.40233500
C -1.17843800 4.44667200 -0.81917100
C -0.78528400 0.78027300 -0.58438600
H 0.54837100 0.80203000 -2.30020200
H 3.84587500 5.76330300 -0.14771700
C -2.78817400 2.28207200 0.09401400
C -2.47059500 4.66224800 -0.32674600
H -0.57831500 5.27253900 -1.18054900
H -1.35339700 0.00972200 -1.09605200
C -3.25865100 3.60559700 0.12431400
C -3.57404900 1.19418800 0.73500700
H -2.86367800 5.67419900 -0.29364500
H -4.24952700 3.79838400 0.52137700
C -2.98791000 0.55999000 1.83920700
C -4.90702800 0.87864500 0.37636100
C -3.70659400 -0.34819600 2.61481200

H -1.97291400 0.82453800 2.11801800
C -5.62319900 -0.01069000 1.19546100
C -5.59816900 1.39874400 -0.81652800
C -5.03832700 -0.61982300 2.30286300
H -3.22648900 -0.81572500 3.46882100
H -6.65504400 -0.23798500 0.93817400
C -5.05236200 1.80191100 -1.97143900
H -6.68621300 1.38573200 -0.74382300
H -5.61968300 -1.30465300 2.91461800
H -3.98064700 1.81908000 -2.13466500
H -5.67942200 2.13538500 -2.79334200
Ru 0.60495700 0.04728000 0.65315700
C 0.80433700 -1.87085400 -0.10182800
N 1.98840500 -2.54775600 -0.09549900
N -0.16475900 -2.78726600 -0.33925700
C 1.82421500 -4.01027500 -0.16799800
C 3.31123400 -1.99577100 -0.12021400
C -1.53415400 -2.53540900 -0.67599500
C 0.35069400 -4.15281500 -0.55458500
H 2.04545100 -4.45368300 0.80999700
H 2.50969000 -4.44168700 -0.90212400
C 3.89608100 -1.77164900 -1.38652100
C 4.02465500 -1.74061800 1.05782300
C -2.54422500 -2.83344900 0.24850900
C -1.84303700 -2.11386500 -1.98393500
H -0.18793500 -4.86644300 0.07293300
H 0.21100800 -4.44288800 -1.60342300
C 5.16998700 -1.21239600 -1.44919900
C 3.14598500 -2.08919500 -2.65759800
C 3.48849000 -2.06088500 2.42777800
C 5.30224800 -1.17051900 0.94386500
C -3.87369000 -2.65963000 -0.15037100
C -2.22449500 -3.36470800 1.62043900
C -3.18270000 -1.93288900 -2.32846900
C -0.75673500 -1.88415500 -3.00595400
C 5.88340700 -0.88309000 -0.28901200
H 5.61455000 -1.01890700 -2.42258200
H 2.83663800 -3.13956700 -2.70451300
H 3.76887900 -1.88535700 -3.53281900
H 2.23299700 -1.49089500 -2.74945300
H 2.63037500 -2.73162900 2.38898800
H 4.27395700 -2.51848500 3.03946800
H 3.14750600 -1.15513300 2.93907100
H 5.85288600 -0.95170100 1.85586300
C -4.21509200 -2.20442700 -1.42277700
H -4.66148400 -2.87571100 0.56350900
H -3.10407500 -3.30634400 2.26651000
H -1.91806700 -4.41908500 1.57545600
H -1.41228600 -2.80117800 2.08452000
H -3.42804700 -1.58981400 -3.33102700
H 0.01225900 -1.20016200 -2.63799000
H -1.17269000 -1.46584100 -3.92684000
H -0.24310300 -2.81791700 -3.26741400
C 7.23427700 -0.21790600 -0.38207600
C -5.65587400 -2.02314500 -1.82693100
H 7.85230500 -0.67181300 -1.16458900
H 7.77862300 -0.28258500 0.56481300
H 7.12684800 0.84604900 -0.63061000
H -5.90145500 -2.63344700 -2.70478200
H -5.85569300 -0.97740800 -2.08292200
H -6.33281300 -2.30788000 -1.01639500
Cl 0.32653900 -1.04849600 2.68117400
Cl 0.55898200 2.09010100 1.98781200

2b-TS2:

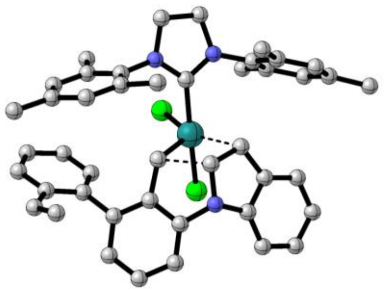

G_sol = –2881.77235

C 2.04436700 0.85519100 -0.89163900
H 2.60622900 0.03473800 -1.30747000
C 2.71296600 2.11732300 -0.57209500
C 1.80628800 3.16773200 -0.80889400
C 3.98589200 2.38263300 -0.07249600
N 0.61072800 2.63639900 -1.36181600
C 2.13118200 4.48712000 -0.51830600
C 4.32961400 3.70549100 0.20428900
H 4.67366300 1.56631200 0.12352200
C -0.68283000 3.05825800 -0.95662300
C 0.75075900 1.22416100 -1.43489400
C 3.40724700 4.73928800 -0.00940000
H 1.42321300 5.29399900 -0.66295200
H 5.31433800 3.93798200 0.59877200
C -1.45944700 2.03374800 -0.37952700
C -1.17712600 4.34562200 -1.10362000
C -0.85782100 0.68129100 -0.36669900
H 0.34216400 0.74216200 -2.31708800
H 3.68846000 5.76258400 0.22216500
C -2.75608200 2.31229600 0.07390400
C -2.46247600 4.62436100 -0.62443300
H -0.58664100 5.11099500 -1.59374800
H -1.45921100 -0.06312100 -0.89224700
C -3.23674600 3.62688300 -0.04175000
C -3.54481900 1.27058800 0.78283300
H -2.86063600 5.63034300 -0.71773200
H -4.23065800 3.85389700 0.32829200

C -2.95389600 0.67661400 1.90723300
C -4.87435700 0.92957000 0.43075600
C -3.66144800 -0.22415400 2.70227400
H -1.94129000 0.95495500 2.18069100
C -5.58027200 0.05397300 1.27195200
C -5.56860000 1.39998600 -0.78162700
C -4.98770300 -0.52228300 2.39356100
H -3.17376900 -0.66719300 3.56470100
H -6.60789700 -0.19511700 1.01805100
C -5.02713800 1.73276100 -1.96059100
H -6.65601900 1.40688800 -0.69998100
H -5.56019600 -1.20216400 3.01898000
H -3.95704000 1.72085500 -2.13485600
H -5.65606700 2.03272300 -2.79391900
Ru 0.65135100 0.06246700 0.59595800
C 0.77977600 -1.87409600 -0.09768800
N 1.95557700 -2.56669100 -0.04317300
N -0.19720800 -2.79053500 -0.30680100
C 1.76957500 -4.02733400 -0.03063700
C 3.28041200 -2.02298600 -0.08906600
C -1.56534200 -2.54287700 -0.64746400
C 0.30845000 -4.16773100 -0.46066500
H 1.94569700 -4.41248800 0.98091500
H 2.47188200 -4.51516800 -0.71132300
C 3.88224000 -1.89371200 -1.36069600
C 3.97679600 -1.67645500 1.07661500
C -2.57739400 -2.82410900 0.28096400
C -1.87373400 -2.13033800 -1.95881200
H -0.25803100 -4.85459500 0.17185000
H 0.20059700 -4.49020000 -1.50435500
C 5.15857100 -1.34311100 -1.44766600
C 3.14401800 -2.29860400 -2.61370100
C 3.41904600 -1.88748700 2.45950000
C 5.25685200 -1.11777300 0.93709800
C -3.90597500 -2.63976800 -0.11747200
C -2.26712600 -3.35565000 1.65498700
C -3.21239600 -1.94286400 -2.30414400
C -0.78591100 -1.91998300 -2.98324700
C 5.85664000 -0.92789000 -0.30574600
H 5.61686400 -1.22373900 -2.42658600
H 2.85420700 -3.35563300 -2.59988800
H 3.76736900 -2.13579900 -3.49719600
H 2.22108800 -1.72283500 -2.74216100
H 2.57957600 -2.58252500 2.46471200
H 4.20274900 -2.26645700 3.12461200
H 3.04341600 -0.95036700 2.88329000
H 5.79453700 -0.82924600 1.83731500
C -4.24607000 -2.19703900 -1.39434500
H -4.69441600 -2.84071200 0.60068700
H -3.11625200 -3.19687400 2.32482300
H -2.07230000 -4.43707500 1.62265400
H -1.39203000 -2.86316200 2.08307800
H -3.45615700 -1.60948200 -3.31039200
H 0.00201400 -1.26054400 -2.61149600
H -1.19394600 -1.48626900 -3.90069800
H -0.29817000 -2.86577900 -3.25166400
C 7.21071000 -0.27409300 -0.42871600
C -5.68677200 -2.01447100 -1.79853700
H 7.82593200 -0.76493500 -1.19068100
H 7.75512300 -0.29953600 0.52007000
H 7.10839200 0.77869000 -0.72293600
H -6.36302100 -2.27558200 -0.97931400
H -5.93956800 -2.64451600 -2.66027800
H -5.88095400 -0.97412800 -2.07853100
Cl 0.19767100 -0.99964900 2.64301900
Cl 0.69139400 2.10876000 1.92816100

3a-Alk1:

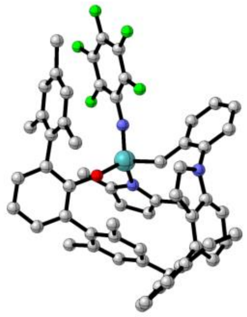

G_sol = –3084.794048

Mo 0.90198000 0.37512600 -0.88166600
N 2.49798200 -0.34534400 -1.02177500
C 3.68628200 -0.91386900 -0.68181000
C 3.71715500 -2.12542500 0.03293300
C 4.91563700 -2.72607600 0.38675500
C 6.12300900 -2.10833900 0.06962700
C 6.12210200 -0.89580400 -0.61532300
C 4.91826800 -0.31754400 -0.99527400
F 2.56507500 -2.70002600 0.39994500
F 4.91725000 -3.87127500 1.08226800
F 7.27875800 -2.65470500 0.46364400
F 7.28067600 -0.28484600 -0.88963200
F 4.94030800 0.85978200 -1.64012200
N 0.52273700 1.45099100 -2.53912400
C 1.24557700 2.64537000 -2.74391600
C 0.80015300 3.22619400 -3.90160300
C -0.21597100 2.37435500 -4.45027200
C -0.37266500 1.29993300 -3.60947500
C 2.29170500 3.10155400 -1.77681300
H 2.68547300 4.07283600 -2.09036700
H 3.13623600 2.40628000 -1.71369800
H 1.89470700 3.23663700 -0.76087300
C -1.30514300 0.14321900 -3.74685000
H -1.87614000 0.25333400 -4.67401200

H -2.02319300 0.08764100 -2.92177400
H -0.77714800 -0.81634200 -3.78781800
H -0.77303300 2.53788900 -5.36421000
H 1.15667900 4.16213500 -4.31292200
O 0.37267200 1.17336000 0.75925200
C 0.50559000 2.31021100 1.48624500
C -0.45991500 3.32186700 1.30630600
C -0.34173200 4.47641500 2.08506900
C 0.70765400 4.62427500 2.99438600
C 1.66289300 3.61739900 3.12967500
C 1.58203200 2.43800700 2.37695800
C 2.25691300 0.13782100 3.14001900
C 2.60374600 1.35915100 2.51581400
C 3.92450600 1.56480300 2.07423500
C 4.89088900 0.57989000 2.32387900
C 4.58205500 -0.60250700 2.99281100
C -3.21842000 1.77714700 -0.77693400
C -2.39376200 2.05339900 0.31978000
C -1.48737400 3.13162600 0.24433600
C -1.46142500 3.94871600 -0.90857400
C -2.31171900 3.64562700 -1.97287700
C -3.18642500 2.55794200 -1.93373800
C 3.24872800 -0.81660100 3.36506900
H 2.48620700 3.73214000 3.82887300
H 0.78223300 5.52631000 3.59438600
H -1.07967600 5.26439800 1.96855100
C -0.22890800 -1.11675000 -1.11862800
H -1.21824400 -0.78645600 -0.76720700
H 5.91385000 0.75607700 1.99709500
H 2.98100600 -1.74293900 3.86901100
H -3.88970500 0.92502600 -0.72850100
H -2.26217200 4.25488600 -2.87139200
C 4.34690600 2.81536900 1.33564900
H 3.49094900 3.38698800 0.97377700
H 4.93718100 3.48062200 1.97870900
H 4.97181300 2.55471100 0.47448800
C 5.65078300 -1.60601000 3.35354600
H 5.32168800 -2.63309500 3.16906000
H 6.57084200 -1.44073800 2.78501300
H 5.90586600 -1.53374200 4.41874000
C 0.84066200 -0.13520200 3.58626800
H 0.80013100 -1.01502600 4.23550100
H 0.42071100 0.71603300 4.13291600
H 0.18572800 -0.31543300 2.72674100
C -2.50050900 1.18178700 1.54918200
H -3.54652900 0.93929400 1.75774300
H -1.97329300 0.23093400 1.41718100
H -2.07850700 1.67182300 2.43036300
C -4.07479400 2.24649100 -3.11225400
H -4.90152000 2.96485000 -3.18976000
H -3.50871800 2.29985600 -4.04897100
H -4.50732600 1.24568700 -3.02786100
C -0.54060200 5.14104000 -1.03199000
H -0.97016100 6.02878400 -0.54939400
H 0.42945800 4.96526500 -0.56168400
H -0.37112900 5.38092200 -2.08474700
C -0.11576200 -2.50675900 -1.50706000
C -1.10880900 -3.43823400 -1.12210900
C 0.96206000 -2.97795400 -2.28132200
N -2.20942400 -2.99642200 -0.33225300
C -1.01844500 -4.77872900 -1.48733300
C 1.05303800 -4.31809200 -2.64132600
H 1.71967800 -2.26829800 -2.59534500
C -3.34613900 -2.36590200 -0.82041000
C -2.22289400 -2.91525500 1.05722100
C 0.06486100 -5.22416700 -2.24610300
H -1.80039000 -5.46087800 -1.16917300
H 1.89396900 -4.65796100 -3.23870900
C -4.09710800 -1.90923600 0.29702700
C -3.75124200 -2.15759800 -2.14195000
C -3.35923400 -2.27362800 1.47399300
H -1.40260300 -3.33735100 1.61990500
H 0.13344500 -6.26950000 -2.53115200
C -5.31693100 -1.22985500 0.07760300
C -4.94944200 -1.47970500 -2.33490800
H -3.15397000 -2.51273000 -2.97536400
H -3.64884700 -2.08565100 2.49686200
C -5.71595000 -1.02214500 -1.24354200
C -6.10178800 -0.67595200 1.21302100
H -5.30545700 -1.30026500 -3.34500800
H -6.64879500 -0.49847300 -1.43032700
C -6.26285400 0.71632800 1.26888800
C -6.64237300 -1.47322200 2.25352400
C -6.90863700 1.33795600 2.33653500
H -5.83952700 1.31869300 0.47099800
C -7.26396000 -0.82207600 3.33422400
C -6.64721100 -2.94729300 2.28222600
C -7.40217700 0.56176600 3.38397500
H -7.00651900 2.41948000 2.35508300
H -7.66713100 -1.42931500 4.14099700
C -6.57239000 -3.80163300 1.25475400
H -6.78960000 -3.36535200 3.27905400
H -7.89386000 1.02849700 4.23271400
H -6.46210600 -3.47771000 0.22664100
H -6.62772400 -4.87244500 1.42810000

3a-Coordination:

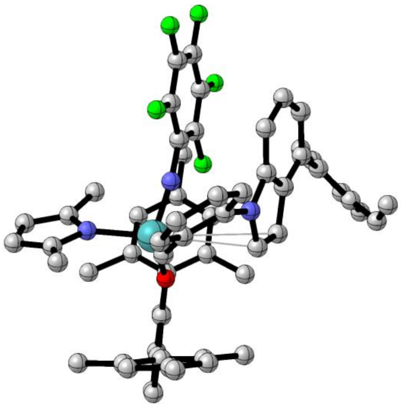

G_sol = –3084.796028

C 2.22575000 -1.66150600 -0.07448600
H 3.20563100 -1.25235700 0.19916900
C 2.05353100 -3.08593800 0.20560200
C 0.92308200 -3.59955000 0.88162600
C 3.04336800 -4.00013800 -0.20207100
N -0.03013200 -2.71282600 1.43770800
C 0.76989300 -4.97538900 1.06945500
C 2.88019900 -5.37188500 -0.02999600
H 3.93442400 -3.61657700 -0.68782600
C -1.40260100 -2.70515600 1.20846300
C 0.30170600 -1.56727800 2.15559500
C 1.73177300 -5.86390900 0.59444400
H -0.09526800 -5.33481300 1.61685900
H 3.64946800 -6.05446100 -0.37863300
C -1.93064200 -1.52986600 1.81211000
C -2.20314800 -3.60604500 0.50097700
C -0.82613700 -0.83098700 2.40520600
H 1.32412600 -1.41046600 2.45868100
H 1.59998800 -6.93152500 0.74119500
C -3.31573600 -1.26309000 1.73445400
C -3.55949100 -3.31376500 0.41297500
H -1.78001200 -4.47808700 0.01523100
H -0.87849100 0.07720000 2.98300300
C -4.10514500 -2.16477400 1.01792200
C -3.90474100 -0.03477500 2.33075700
H -4.21430300 -3.98551200 -0.13414400
H -5.17274100 -1.98024600 0.94280700
C -4.62144800 0.82251700 1.48400400
C -3.74672300 0.32755500 3.69389600
C -5.14332300 2.03373300 1.93430200
H -4.74485500 0.54699600 0.44436000
C -4.23758400 1.57706300 4.11375800
C -3.15968000 -0.52871000 4.74150700
C -4.92996000 2.42587400 3.25506500
H -5.68957200 2.67452600 1.24797500
H -4.09703900 1.86491200 5.15269600
C -3.11815000 -1.86530700 4.78583800
H -2.78416300 0.02196900 5.60433000
H -5.30565800 3.37855300 3.61688700
H -3.49894900 -2.48967900 3.98557100
H -2.69498700 -2.37457800 5.64667200
Mo 1.19464100 -0.22748900 -0.75000500
N 2.05540500 0.12280200 -2.54658400
C 1.48682000 1.11343900 -3.37454400
C 3.29129000 -0.24018300 -3.10285800
C 2.37315400 1.39644700 -4.38249000
C 0.09064100 1.61050700 -3.17549200
C 3.50959800 0.54163300 -4.21234300
C 4.13892300 -1.35546700 -2.58856100
H 2.21008600 2.11175900 -5.17861000
H -0.11300000 1.96041200 -2.15897100
H -0.64686600 0.83300100 -3.41400200
H -0.09561600 2.45845200 -3.83967500
H 4.38225700 0.48795800 -4.85105800
H 4.54577200 -1.17105600 -1.58931800
H 3.57533600 -2.29459100 -2.54520300
H 4.98468600 -1.50003300 -3.26833000
N -0.33511200 -0.97213400 -1.17509800
C -1.58314700 -1.17921300 -1.66820900
C -2.60712600 -0.23065900 -1.50623400
C -1.88549200 -2.34083100 -2.40049700
C -3.86042300 -0.41658200 -2.06933800
F -2.35919400 0.88065700 -0.80665700
C -3.14406100 -2.54344600 -2.94992300
F -0.94908100 -3.28412300 -2.54736700
C -4.13635000 -1.57744700 -2.78809700
F -4.82209200 0.50925100 -1.90483400
F -3.41727800 -3.66939200 -3.62058200
F -5.35155100 -1.76985200 -3.31040400
O 1.39671900 1.44662800 0.17680400
C 2.04195100 2.53899200 0.63970200
C 1.41369100 3.79574700 0.52043700
C 3.30742000 2.41508500 1.24505200
C 0.04844700 3.91839100 -0.06381700
C 2.09261200 4.92571700 0.98759800
C 3.95157500 3.57062600 1.69957700
C 3.92957000 1.07780400 1.45586200
C -1.07026100 3.51723800 0.69465400
C -0.12683200 4.43782400 -1.35828600
H 1.60631500 5.89309300 0.89881800
C 3.35632200 4.82392200 1.56758200
H 4.92594600 3.47081500 2.17016500
C 3.52435400 0.30253700 2.56330000
C 4.93803400 0.60820500 0.59592400
C -2.34297900 3.62823700 0.13890800
C -0.90308700 2.94682200 2.08113900
C -1.42189700 4.52399500 -1.88473200
C 1.06068700 4.82858000 -2.20706400

H 3.86665800 5.71225800 1.92751100
C 4.11946200 -0.94160900 2.77948900
C 2.46530500 0.82122200 3.50546500
C 5.52302100 -0.63956600 0.85718000
C 5.36607900 1.41166600 -0.60899800
C -2.53830700 4.10630600 -1.16004200
H -3.19712600 3.29810400 0.72179000
H -1.87346800 2.77557000 2.55600300
H -0.37967100 1.98675000 2.03275100
H -0.30939500 3.60736700 2.72368500
H -1.55338600 4.90232700 -2.89642100
H 0.73315300 5.22579700 -3.17280800
H 1.68312500 5.58774300 -1.72200200
H 1.70757000 3.96401000 -2.40237900
C 5.11982800 -1.43455900 1.93265900
H 3.80330800 -1.53851600 3.63266900
H 1.52179300 1.00107800 2.98008900
H 2.27606100 0.11157600 4.31650600
H 2.76395600 1.77819500 3.94777100
H 6.30824100 -0.99924400 0.19598600
H 6.24987900 0.96967100 -1.07835600
H 4.57276600 1.44458300 -1.36587700
H 5.59696600 2.44733000 -0.34214100
C -3.92165000 4.10971700 -1.76333000
C 5.71440700 -2.80240300 2.16017000
H -4.64517400 4.61361900 -1.11092300
H -3.93725800 4.61141100 -2.73613800
H -4.27808700 3.08186300 -1.90758100
H 5.02536500 -3.58646200 1.82072000
H 6.65407000 -2.92591100 1.61249400
H 5.91287400 -2.98126600 3.22269700

3a-TS1:

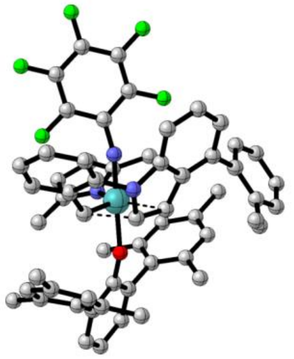

G_sol = –3084.771209

C 0.43957200 2.08369700 0.21466000
H -0.40940900 2.76107700 0.25630000
C 1.74905600 2.68954200 0.49259500
C 2.43126500 2.19667900 1.62527700
C 2.30847300 3.74408300 -0.23118100
N 1.75811500 1.14379800 2.30340900
C 3.64676400 2.74151700 2.02620800
C 3.54323100 4.27511000 0.14946200
H 1.78342000 4.13414200 -1.09682700
C 2.28023600 -0.16679600 2.42476300
C 0.37507200 1.03801600 1.99317700
C 4.20789500 3.77662700 1.27136900
H 4.13020100 2.38982400 2.93083300
H 3.97873300 5.08764100 -0.42383800
C 1.23390600 -1.09974200 2.27660100
C 3.59158400 -0.56275700 2.66742600
C 0.01792500 -0.36362000 1.90378000
H -0.28958700 1.79146100 2.39197300
H 5.15647500 4.20707200 1.57751800
C 1.47246900 -2.46477100 2.46522900
C 3.82753300 -1.92992800 2.83401000
H 4.39867300 0.15616200 2.74176400
H -0.96762200 -0.70559900 2.19185200
C 2.78687200 -2.85985400 2.75574600
C 0.40992500 -3.49412000 2.30819400
H 4.83633200 -2.27362900 3.04259800
H 2.99244300 -3.91464700 2.90846400
C 0.57099100 -4.42882500 1.27554700
C -0.72079900 -3.58869300 3.15660400
C -0.34780000 -5.45597600 1.06967700
H 1.43123800 -4.33014300 0.62234600
C -1.64260100 -4.62419400 2.91972100
C -1.00532800 -2.70628900 4.30447000
C -1.45920700 -5.55799400 1.90435300
H -0.19754600 -6.16492100 0.26122500
H -2.51105400 -4.70294500 3.56907400
C -0.14683900 -2.01968400 5.06961900
H -2.06062200 -2.67320000 4.57672700
H -2.18649700 -6.35140300 1.75850200
H 0.92392200 -2.02307400 4.90475700
H -0.51095100 -1.43886800 5.91222000
Mo -0.10094700 0.26351300 -0.30401300
N -0.62694400 -0.21532300 -2.24814500
C -0.42751700 -1.47125000 -2.81232000
C -0.83554100 0.69831200 -3.27837000
C -0.53159000 -1.35578900 -4.18316400
C -0.15663800 -2.67786100 -1.96916500
C -0.78039900 0.02118000 -4.47911600
C -1.08479800 2.14712700 -3.01401600
H -0.45917500 -2.17254400 -4.89096300
H -0.86013300 -2.75830000 -1.13210600
H 0.85598000 -2.68528100 -1.54799800
H -0.27248800 -3.58531300 -2.56999600
H -0.94006300 0.46000900 -5.45631700
H -1.89860400 2.28766200 -2.29600100
H -0.20237900 2.66220400 -2.60825700
H -1.36572600 2.65354700 -3.94282500

N 1.56483100 -0.23917800 -0.62525500
C 2.79606200 -0.43375200 -1.14930900
C 3.53311100 -1.61167300 -0.92148000
C 3.38560900 0.54071500 -1.98284300
C 4.80712400 -1.78708800 -1.44254000
F 2.98940800 -2.60011800 -0.20162500
C 4.66062100 0.37598100 -2.50514400
F 2.69938500 1.64532200 -2.29367100
C 5.37811900 -0.78805600 -2.23007600
F 5.48863600 -2.91235400 -1.19739900
F 5.20266700 1.32173200 -3.28029100
F 6.60678800 -0.95001700 -2.72855700
O -1.99406700 0.77805400 0.05899600
C -3.25157000 1.17027600 -0.18595100
C -4.19674200 0.25165000 -0.69659200
C -3.64091000 2.50540600 0.09577200
C -3.84795000 -1.17452800 -0.95119200
C -5.50287300 0.69306400 -0.93685600
C -4.95536600 2.90265400 -0.16600400
C -2.66558800 3.46276000 0.69283900
C -3.65557800 -2.05238400 0.13480800
C -3.78905000 -1.66549600 -2.26848500
H -6.22398500 -0.02237900 -1.32200300
C -5.88881200 2.00764700 -0.68490700
H -5.24102300 3.92787100 0.05389700
C -2.27560400 3.29236100 2.04107600
C -2.11702500 4.52284900 -0.05629500
C -3.39895900 -3.40131100 -0.11421800
C -3.77305500 -1.56354600 1.55811200
C -3.52025200 -3.02210700 -2.47639600
C -4.01357300 -0.76008800 -3.45403600
H -6.90846000 2.32746800 -0.87757100
C -1.28177300 4.11242300 2.57874300
C -2.91609500 2.22476900 2.89493700
C -1.10786000 5.31173000 0.51544500
C -2.58176700 4.85331100 -1.45564300
C -3.31846600 -3.90460100 -1.41570100
H -3.25487000 -4.07140200 0.72603300
H -3.62003300 -2.38365600 2.26528700
H -3.03255700 -0.78750000 1.77260200
H -4.75667200 -1.11819500 1.74993400
H -3.45237700 -3.38964300 -3.49765800
H -3.69219900 -1.24946600 -4.37652700
H -5.07089500 -0.48713100 -3.55950500
H -3.44098600 0.16488700 -3.35787200
C -0.65276700 5.10485500 1.81704000
H -0.98118000 3.96841100 3.61498400
H -2.63877600 1.22249400 2.55255500
H -2.61024000 2.32429300 3.94114300
H -4.00854000 2.27959500 2.84577800
H -0.66118300 6.10311900 -0.08323700
H -1.73193600 5.06962800 -2.11181000
H -3.16305000 4.04591900 -1.90056800
H -3.21789800 5.74799800 -1.44333900
C -2.97203200 -5.35206900 -1.66192800
C 0.50391200 5.89485500 2.37625300
H -3.38603500 -6.00285500 -0.88388500
H -3.34785000 -5.69621400 -2.63116500
H -1.88366100 -5.49810800 -1.65992500
H 1.45294600 5.37823500 2.18202100
H 0.57209900 6.88635500 1.91673200
H 0.41456000 6.02506800 3.45996500

3a-Metallacycle

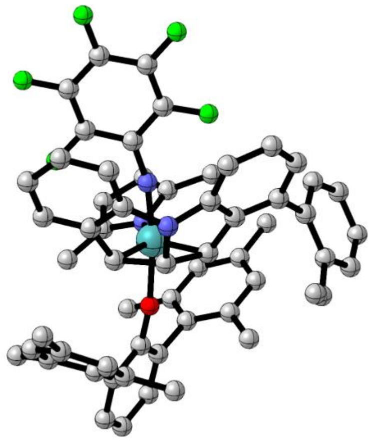

G_sol = –3084.780385

C 0.84711900 1.80704900 0.52871500
H 0.17137000 2.64873200 0.56030300
C 2.28468600 2.10497500 0.42287100
C 3.02164100 1.27067400 1.28686300
C 2.93995200 3.02821500 -0.38601400
N 2.14719500 0.49906300 2.07771400
C 4.41427200 1.28917200 1.28988100
C 4.33805300 3.06342800 -0.38214900
H 2.36726800 3.67995700 -1.03800600
C 2.25843300 -0.88333500 2.32470900
C 0.75451700 0.84368200 1.77415600
C 5.06161300 2.18994200 0.43591900
H 4.99016700 0.64727200 1.94462300
H 4.86351500 3.76225700 -1.02523200
C 1.02570400 -1.52775600 2.10355500
C 3.38291300 -1.57751100 2.76107900
C 0.01851700 -0.55718200 1.61702900
H 0.26404000 1.39534100 2.57746600
H 6.14717200 2.22077900 0.42747100
C 0.89468900 -2.89513000 2.36359600
C 3.25634800 -2.95182500 2.97806900
H 4.31909600 -1.07113400 2.95991800
H -0.95441900 -0.59728400 2.10057900
C 2.03519900 -3.59794600 2.78817700

C -0.37825300 -3.64110900 2.17070400
H 4.11677800 -3.51925200 3.31981900
H 1.94695000 -4.66147900 2.98635800
C -0.39699300 -4.63415200 1.18241100
C -1.53361700 -3.43095900 2.96245900
C -1.52488600 -5.42071200 0.95602200
H 0.49351800 -4.77203100 0.57693900
C -2.66478500 -4.22728200 2.70806500
C -1.64813000 -2.45727500 4.06487900
C -2.66650700 -5.21772600 1.73019700
H -1.51135800 -6.17865000 0.17857100
H -3.55456100 -4.07170400 3.31287100
C -0.68358100 -1.95088800 4.84486400
H -2.67604900 -2.16964200 4.28586800
H -3.55615100 -5.81974500 1.56945100
H 0.36315500 -2.20718300 4.73436900
H -0.93747400 -1.26457400 5.64771500
Mo -0.14543200 0.15069200 -0.37026800
N -0.93158200 -0.43511200 -2.17583000
C -0.85552500 -1.73111500 -2.68968600
C -1.17642500 0.44080300 -3.23472400
C -1.08809300 -1.67282300 -4.04617700
C -0.57268000 -2.91407600 -1.82163700
C -1.28371000 -0.29854800 -4.39243600
C -1.23903600 1.92423500 -3.05078100
H -1.13520400 -2.52603900 -4.71161500
H -1.27397100 -2.97951600 -0.98301600
H 0.43979700 -2.89285000 -1.39889500
H -0.67205800 -3.83421600 -2.40616300
H -1.49326200 0.10427800 -5.37568500
H -2.02915000 2.22163100 -2.35366200
H -0.29406700 2.34075800 -2.67262200
H -1.44214100 2.40495500 -4.01269800
N 1.43358600 -0.49724400 -0.83103700
C 2.68786200 -0.69536400 -1.29271400
C 3.45925800 -1.80515600 -0.89783200
C 3.28543000 0.23708200 -2.16559000
C 4.79210300 -1.92169900 -1.26603800
F 2.90135500 -2.76018400 -0.14661600
C 4.61857400 0.13284000 -2.53002300
F 2.55667100 1.25442100 -2.63698800
C 5.37661200 -0.94590900 -2.07389600
F 5.51958700 -2.96809300 -0.85746000
F 5.18170700 1.05466300 -3.31808200
F 6.66215800 -1.05184600 -2.42190200
O -1.81326300 1.14450900 0.08126100
C -2.96030500 1.84112500 0.03717900
C -4.13449400 1.23712000 -0.47654000
C -3.00683900 3.17351800 0.51530800
C -4.12089600 -0.17376800 -0.94760900
C -5.31694400 1.98277200 -0.51706800
C -4.21064000 3.88212400 0.45325000
C -1.79821700 3.81896200 1.10089000
C -3.96736700 -1.21073000 -0.00875700
C -4.27426200 -0.47720600 -2.31405900
H -6.21110700 1.50687600 -0.90946200
C -5.36558100 3.29906200 -0.06293200
H -4.23059700 4.90216900 0.82775200
C -1.39720900 3.48828800 2.40956800
C -1.04876900 4.74866200 0.35099800
C -3.90892900 -2.53277700 -0.45441600
C -3.90641000 -0.91287800 1.46989000
C -4.20031500 -1.81120200 -2.72075300
C -4.53971000 0.59253700 -3.34720300
H -6.29472800 3.85982700 -0.10087600
C -0.21895200 4.03966800 2.92358500
C -2.20909900 2.51998200 3.23605400
C 0.12827200 5.27052400 0.89621300
C -1.48481000 5.16053500 -1.03448100
C -4.00488700 -2.85041200 -1.81052000
H -3.77586200 -3.32793100 0.27199600
H -3.95077500 -1.83622300 2.05171300
H -2.98188000 -0.39174200 1.73662300
H -4.73351900 -0.26463900 1.78130500
H -4.27190700 -2.03882200 -3.78129800
H -4.19661700 0.26538800 -4.33170000
H -5.61333900 0.81396300 -3.41958800
H -4.03758600 1.53133400 -3.10620100
C 0.57584900 4.90772500 2.17014900
H 0.09617200 3.76929400 3.92953300
H -2.21387000 1.52067600 2.78595600
H -1.80728600 2.43530100 4.25067600
H -3.25644200 2.83326500 3.30398600
H 0.72115500 5.96342500 0.30292800
H -0.71337400 5.76342400 -1.52316200
H -1.69415000 4.29137700 -1.66402600
H -2.40659900 5.75313400 -1.00431300
C -3.89072200 -4.27779000 -2.28573500
C 1.90366200 5.40062900 2.68875900
H -3.17279900 -4.35557000 -3.11064100
H -3.55247800 -4.93708600 -1.48008500
H -4.85094600 -4.66194600 -2.65392300
H 2.72437700 4.81343600 2.25600300
H 2.07689400 6.44880500 2.42129400
H 1.96863600 5.31034000 3.77771600

3a-TS2:

G_sol = –3084.77064

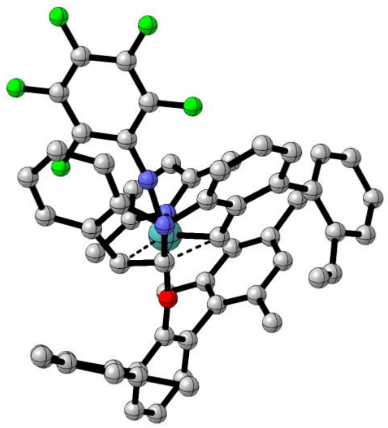

C 0.76156100 2.05091300 0.87088400
H 0.03717200 2.82885100 0.69818800
C 2.20057900 2.24383800 0.67846800
C 2.87500800 1.33861500 1.52512100
C 2.92064300 3.06559700 -0.18470100
N 1.92352100 0.68061600 2.34381300
C 4.25540300 1.17677800 1.46247400
C 4.31192300 2.94021200 -0.22542500
H 2.40248100 3.76489700 -0.83331400
C 2.00498800 -0.69646800 2.67883600
C 0.63199100 1.12720600 1.96252800
C 4.96428000 1.99386400 0.57484600
H 4.77189900 0.44695600 2.07414500
H 4.89044500 3.56624400 -0.89770800
C 0.93306200 -1.49416700 2.21878900
C 3.05397600 -1.23442100 3.41570300
C -0.13371600 -0.80275700 1.46894900
H -0.12907900 1.21243900 2.72655900
H 6.04407500 1.89470900 0.51542700
C 0.91755800 -2.85841400 2.55146200
C 3.06570500 -2.60645400 3.67229400
H 3.83105700 -0.58730000 3.80603900
H -1.12680800 -0.83968000 1.92205200
C 2.00772000 -3.40489800 3.24644600
C -0.22310200 -3.74947700 2.20257900
H 3.88420300 -3.04334600 4.23594800
H 1.99117700 -4.46335300 3.48666000
C -0.00109000 -4.76632700 1.26752500
C -1.49396500 -3.63588700 2.81907100
C -1.00822200 -5.66075300 0.90887800
H 0.97733100 -4.83318000 0.80187500
C -2.49944500 -4.53889700 2.42987300
C -1.85424400 -2.66299500 3.86765900
C -2.26738500 -5.54402400 1.49520700
H -0.81151500 -6.43273700 0.17124200
H -3.47957600 -4.45476400 2.89234900
C -1.06632500 -2.01303500 4.73610900
H -2.92877300 -2.50903700 3.96196000
H -3.06679200 -6.22765900 1.22483300
H 0.01045400 -2.12485400 4.76569400
H -1.50874000 -1.35394900 5.47773200
Mo -0.09611600 0.19024500 -0.22800600
N -0.73059900 -0.58597100 -2.04535300
C -0.50200200 -1.88310700 -2.48855600
C -0.89609800 0.23022800 -3.16242800
C -0.56304500 -1.89456100 -3.86766800
C -0.22096500 -2.99929000 -1.53886200
C -0.80760500 -0.55302600 -4.29524500
C -1.05732500 1.71465700 -3.05518400
H -0.46165200 -2.77172500 -4.49514800
H -0.99190200 -3.07446200 -0.76631100
H 0.74176400 -2.87649200 -1.02522500
H -0.19354800 -3.95267500 -2.07623600
H -0.91609500 -0.19956300 -5.31346700
H -1.94519400 2.00277300 -2.48338900
H -0.18912700 2.19929900 -2.58266200
H -1.15086900 2.14615700 -4.05667300
N 1.55755100 -0.24100200 -0.65604900
C 2.77873000 -0.39409700 -1.20903200
C 3.65707800 -1.40622300 -0.77887100
C 3.22900400 0.47433400 -2.22151300
C 4.93901400 -1.51616900 -1.29647100
F 3.25358700 -2.26656300 0.16508200
C 4.51062800 0.37435100 -2.74104500
F 2.41192700 1.43268900 -2.67374400
C 5.36945200 -0.62137600 -2.27662900
F 5.76805300 -2.47275100 -0.86034200
F 4.93272400 1.23187500 -3.67698700
F 6.60741400 -0.71922100 -2.77080200
O -1.84295200 1.10762000 0.02763200
C -3.02498100 1.71136800 -0.18203700
C -4.09615800 1.01043100 -0.79013600
C -3.21005900 3.05131700 0.23460900
C -3.95751800 -0.41275800 -1.20102600
C -5.31180000 1.67507900 -0.98573600
C -4.44133100 3.67676300 0.01553300
C -2.12445400 3.79545100 0.93296400
C -3.83790600 -1.40735900 -0.21308700
C -3.97378400 -0.77224500 -2.56267700
H -6.12647100 1.12611800 -1.44922000
C -5.49333500 3.00025000 -0.59686400
H -4.56629700 4.70457800 0.34592500
C -1.86577900 3.53644200 2.29298300
C -1.36177100 4.76033600 0.24450000
C -3.66875400 -2.73837600 -0.59911700
C -3.94137200 -1.05906700 1.25182100
C -3.79193600 -2.11292300 -2.90793300
C -4.20607900 0.23936600 -3.66052500
H -6.44661800 3.49495200 -0.75740300
C -0.82458400 4.21547900 2.93231300
C -2.68123400 2.51280200 3.04533300
C -0.32281100 5.41253800 0.91662800

C -1.63373700 5.07064600 -1.20809400
C -3.62130500 -3.10706100 -1.94452900
H -3.55943200 -3.50011000 0.16643500
H -3.92902800 -1.96379700 1.86438200
H -3.11615200 -0.41790100 1.57633500
H -4.86544400 -0.50962500 1.46766200
H -3.75372100 -2.38043500 -3.96080500
H -3.73074000 -0.09047200 -4.58747000
H -5.27928500 0.36784800 -3.85798000
H -3.81005900 1.22402900 -3.40639500
C -0.02605200 5.14128200 2.25551900
H -0.62074400 4.00381200 3.98003800
H -2.52407500 1.50669400 2.63904600
H -2.41409200 2.49952700 4.10679100
H -3.75454900 2.71422700 2.96145300
H 0.27856100 6.14043700 0.37602400
H -0.87979400 5.75520800 -1.60849700
H -1.63181500 4.16169200 -1.81726100
H -2.61756900 5.53401400 -1.34338000
C -3.37569600 -4.53815900 -2.35322600
C 1.15499600 5.79297400 2.93141300
H -2.56065600 -4.59448600 -3.08440800
H -3.09925600 -5.15418800 -1.49188300
H -4.26252300 -4.98687100 -2.81940900
H 2.08092400 5.25092000 2.69759900
H 1.29011700 6.82714900 2.59674800
H 1.04219500 5.79807400 4.02033800

3a-Alk2:

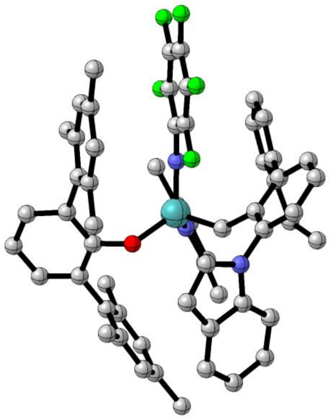

G_sol = –3084.800721

Mo 0.25594200 0.11784400 0.59708500
N -1.48357100 0.10906900 0.78445800
C -2.79216300 0.05186300 0.42301400
C -3.16330700 -0.42837500 -0.84635700
C -4.49374400 -0.57155000 -1.20731900
C -5.49935800 -0.19729500 -0.32002900
C -5.16102500 0.30220000 0.93596900
C -3.82641100 0.42077700 1.29696800
F -2.21915500 -0.76024000 -1.73480800
F -4.81637600 -1.01323700 -2.43048600
F -6.78213400 -0.28048300 -0.68683500
F -6.12537700 0.66985300 1.78884100
F -3.53010800 0.89714400 2.51359800
N 1.25938000 0.02203100 2.35219500
C 0.80331300 0.52169400 3.58644700
C 1.73711400 0.21833600 4.54412000
C 2.81398400 -0.48049900 3.90210300
C 2.50784000 -0.59546700 2.56948400
C -0.48955900 1.25379500 3.73005900
H -0.60500600 1.58384700 4.76706400
H -1.34921400 0.62981900 3.47151600
H -0.53490300 2.14841100 3.09444800
C 3.30681200 -1.22146700 1.47432100
H 4.31297000 -1.43911600 1.84185500
H 3.42562700 -0.56015900 0.60908300
H 2.86758400 -2.16084200 1.12392400
H 3.72741500 -0.83191800 4.36540000
H 1.67380400 0.49426900 5.58919700
O 1.02202600 1.38779600 -0.64350100
C 1.11176000 2.74235600 -0.47605900
C 2.36157300 3.27814900 -0.10290900
C 2.47491500 4.66322000 0.04934800
C 1.38197500 5.50275500 -0.16090000
C 0.15959500 4.95398300 -0.53498200
C -0.00465500 3.57011900 -0.70335300
C -1.50295200 2.49390500 -2.42580800
C -1.33849000 3.06755100 -1.14435300
C -2.46927900 3.29485100 -0.33328100
C -3.74391600 3.01202800 -0.83716000
C -3.92998900 2.49241700 -2.11763100
C 5.31248000 1.01987400 -0.81586200
C 4.16588700 1.78999900 -1.01459800
C 3.55089100 2.40229300 0.09822000
C 4.09031200 2.23060800 1.38699900
C 5.24213400 1.45154900 1.54121800
C 5.86294500 0.83389900 0.45559600
C -2.79243600 2.21475200 -2.88372000
H -0.69400300 5.59872100 -0.72208000
H 1.48686200 6.57767500 -0.04798800
H 3.44105400 5.07656100 0.32381900
C 0.50700500 -1.56191800 -0.19934900
H 1.56272200 -1.50356300 -0.50438400
H -4.61097200 3.20363800 -0.20861400
H -2.91356200 1.78842800 -3.87737500
H 5.77801100 0.54220000 -1.67329600
H 5.64416300 1.30488700 2.54097100
C -2.34563800 3.82998500 1.07574100
H -1.39263100 3.54909900 1.53077600
H -2.40381000 4.92514400 1.10621200
H -3.15076800 3.43875600 1.70513500

C -5.31267300 2.24822500 -2.67289200
H -5.37790500 1.27845000 -3.17613500
H -6.07159800 2.26928600 -1.88443400
H -5.58144600 3.01575500 -3.40994100
C -0.31919500 2.20546700 -3.31534800
H -0.64312400 2.01630800 -4.34359000
H 0.38742900 3.04224900 -3.32549000
H 0.23327100 1.32780000 -2.96472500
C 3.60459400 1.95873100 -2.40509600
H 4.33812600 1.65956100 -3.15915000
H 2.71209700 1.33921400 -2.54194000
H 3.31450900 2.99716600 -2.59775100
C 7.06420100 -0.05957100 0.64310300
H 7.85227000 0.16627700 -0.08479800
H 7.48952700 0.04371400 1.64646200
H 6.79031500 -1.11395800 0.50306100
C 3.43546100 2.84591300 2.60072200
H 3.53915400 3.93717600 2.61491100
H 2.36467800 2.62217300 2.62870100
H 3.87541500 2.44793100 3.51823900
C -0.23859000 -2.71826400 -0.66687900
C -1.20539600 -3.39984100 0.11538500
C -0.01949700 -3.15427800 -1.99257000
C -1.95246300 -4.42783600 -0.47309200
C -1.47190300 -3.03418400 1.53233400
C -0.76781800 -4.18325700 -2.55774400
N 0.96407100 -2.49199200 -2.78158800
C -1.75122500 -4.81346800 -1.79801700
H -2.68780800 -4.94540500 0.13561900
C -2.77939200 -2.67446200 1.88224900
C -0.46265800 -3.00317300 2.52591100
H -0.56849300 -4.47666000 -3.58333000
C 0.70813200 -1.50896200 -3.73173100
C 2.33506500 -2.54650800 -2.56340100
H -2.34213700 -5.61645800 -2.22766200
C -3.09130500 -2.20506000 3.15700200
H -3.55075600 -2.71477800 1.11875400
C -0.78410600 -2.47918200 3.78841600
C 0.90138200 -3.53306200 2.36067800
C 1.88633500 -0.95095100 -4.15386100
H -0.31417400 -1.29401800 -4.00731400
C 2.94694200 -1.59301700 -3.42384000
C 3.06619700 -3.33603800 -1.67080500
C -2.07868100 -2.07741000 4.10631100
H -4.10939000 -1.90963000 3.39288300
H 0.00459000 -2.39447800 4.52951600
C 1.30008400 -4.53307000 1.56373300
H 1.63145100 -3.07983000 3.02761400
H 1.98908300 -0.17195400 -4.89606700
C 4.34514800 -1.45888700 -3.39083600
C 4.44630000 -3.16373300 -1.64934100
H 2.57013400 -4.04431000 -1.01475100
H -2.29554600 -1.67731100 5.09232300
H 0.62802400 -5.05620800 0.89184600
H 2.33315000 -4.86849300 1.58044700
C 5.07890400 -2.23898700 -2.50508200
H 4.84279300 -0.74765300 -4.04383800
H 5.04626700 -3.75505500 -0.96379700
H 6.15946200 -2.13478400 -2.46660100

3b-Alk1:

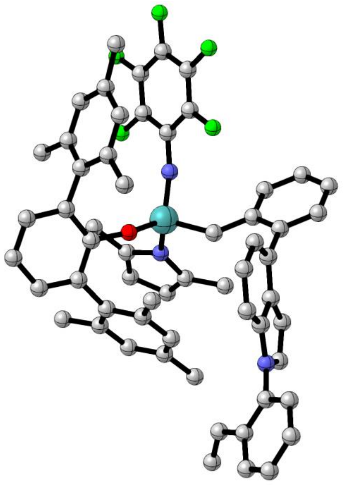

G_sol = –3084.795006

Mo 0.98206000 0.34699000 -0.81836200
N 2.55473200 -0.36541100 -1.14333000
C 3.79684200 -0.87836600 -0.93080700
C 3.95764500 -2.12189800 -0.29310700
C 5.21598700 -2.65457400 -0.05586700
C 6.35431700 -1.94095800 -0.42285200
C 6.22470900 -0.69635200 -1.03326900
C 4.96149500 -0.18181500 -1.29165500
F 2.87495500 -2.79131600 0.12041100
F 5.34408500 -3.82768700 0.57938900
F 7.56941400 -2.42576500 -0.14060500
F 7.31844400 0.00944000 -1.34625300
F 4.85973800 1.03111000 -1.85885300
N 0.43358200 1.44253300 -2.42125200
C 1.06018300 2.68806700 -2.62319700
C 0.50788400 3.27612800 -3.73151900
C -0.47662100 2.37279000 -4.25362500
C -0.50772500 1.26270100 -3.44376300
C 2.13351500 3.17964400 -1.70368200
H 2.46729400 4.17170100 -2.02193600
H 3.01130500 2.52299300 -1.69840300
H 1.78663700 3.28166300 -0.66614700
C -1.34548400 0.03526300 -3.58132200
H -1.97976200 0.13208900 -4.46809300
H -2.00075900 -0.11807200 -2.71661700
H -0.74107600 -0.87139600 -3.69577700
H -1.08955900 2.52164600 -5.13383300

H 0.77728800 4.24658500 -4.12911300
O 0.61477800 1.11576400 0.87897300
C 0.71954500 2.21234800 1.66018300
C -0.32065000 3.16260000 1.60334500
C -0.21989800 4.28261500 2.43309800
C 0.88142100 4.45317900 3.27531600
C 1.90551100 3.50559800 3.29234500
C 1.84511000 2.36357800 2.48347300
C 2.69200700 0.06984800 3.06989000
C 2.92748500 1.33570900 2.48446400
C 4.18834400 1.62207600 1.92875400
C 5.21276500 0.66960100 2.02857300
C 5.01867700 -0.55750000 2.65992600
C -3.06663300 1.48640800 -0.38724600
C -2.21271200 1.79237100 0.67816000
C -1.40149400 2.94336000 0.60113000
C -1.50808400 3.80158200 -0.51714900
C -2.39071400 3.46996100 -1.54580600
C -3.16527000 2.30860600 -1.51071100
C 3.73850300 -0.84893400 3.14761800
H 2.76651700 3.63998700 3.94090300
H 0.94226800 5.32752400 3.91646200
H -1.01151600 5.02569600 2.41097600
C -0.18620400 -1.12563900 -0.95713800
H -1.11914500 -0.77601100 -0.48794700
H 6.18976000 0.90624000 1.61169800
H 3.55814600 -1.81103800 3.62265200
H -3.64616900 0.56908800 -0.34315100
H -2.43984900 4.11329100 -2.42009700
C 4.48116500 2.92815700 1.22480500
H 3.56754100 3.44444400 0.92479800
H 5.04706100 3.61386300 1.86858700
H 5.08316400 2.74902800 0.32747300
C 6.15623800 -1.52925000 2.86269500
H 5.85472500 -2.55909400 2.64769300
H 7.01284500 -1.29038000 2.22568100
H 6.50437700 -1.50488600 3.90351300
C 1.33448800 -0.29467200 3.62115900
H 1.39438600 -1.19590300 4.23879000
H 0.91610200 0.51404300 4.22977400
H 0.62221400 -0.48647200 2.81085300
C -2.18325300 0.86356300 1.86911200
H -3.19411900 0.52684700 2.11596500
H -1.59154800 -0.03667200 1.66905900
H -1.75438400 1.34659900 2.75057000
C -4.07493400 1.96390900 -2.66319400
H -4.92126900 2.65939200 -2.72773600
H -3.53127000 2.02668700 -3.61199000
H -4.48013100 0.95320400 -2.56331700
C -0.68900800 5.06537100 -0.64326200
H -1.15862500 5.90085200 -0.10768700
H 0.31481400 4.94947300 -0.22855000
H -0.59414100 5.35128400 -1.69405600
C -0.16290600 -2.51587900 -1.35653500
C -1.11430000 -3.42054600 -0.82264300
C 0.78853400 -2.98895600 -2.28127400
C -1.08737600 -4.75801100 -1.22730500
C 0.80164900 -4.32242200 -2.67185100
H 1.50876000 -2.28559900 -2.68627700
C -0.14020100 -5.21125400 -2.14639600
H -1.81581500 -5.44609200 -0.80832500
H 1.54040500 -4.66934600 -3.38859400
H -0.13751000 -6.25394500 -2.45084400
C -2.10246700 -2.98090400 0.20432600
C -3.34709600 -2.42535500 -0.16087500
C -1.82868300 -3.13785300 1.56243200
C -4.28103000 -2.07862300 0.85475000
C -2.76037500 -2.76792500 2.55192000
H -0.87276700 -3.56092400 1.85693300
C -4.00098600 -2.23967300 2.21504400
H -2.50350600 -2.90122400 3.59848600
H -4.72048200 -1.95546200 2.97524600
N -5.41377100 -1.56318400 0.22858700
C -3.95863900 -2.10121400 -1.42199900
C -5.20099600 -1.59328600 -1.14994500
C -6.66491600 -1.31353400 0.86072500
C -7.21279100 -2.31285100 1.67227400
C -7.35320500 -0.10250600 0.64312800
C -8.44832500 -2.12385300 2.28409100
H -6.66693200 -3.24146200 1.80078700
C -8.60478500 0.05627500 1.26310500
C -9.15040500 -0.93504500 2.07168300
H -8.86394000 -2.90421100 2.91442700
H -9.13077500 0.99642700 1.13154500
H -10.11367900 -0.77580300 2.54709600
C -6.76653500 0.97700800 -0.16760100
C -7.46448200 1.85647500 -0.89538500
H -5.68353300 1.05250100 -0.15055500
H -6.96491300 2.65255100 -1.43772900
H -8.54695400 1.80845400 -0.98338400
H -5.98200800 -1.25151600 -1.81265000
H -3.52476300 -2.23879400 -2.40182600

3b-TS1:

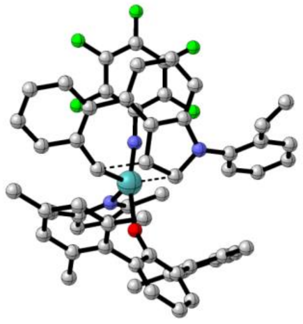

G_sol = –3084.768776

C 0.60452200 2.02752300 -0.06590800
H -0.23573700 2.70986000 0.08985300
C 1.88932300 2.73882300 0.08038900
C 2.99987600 2.32991300 0.86749100
C 1.96457200 3.96043500 -0.61178300
C 4.11257700 3.18064400 0.94744200
C 3.09907900 4.76272700 -0.55675100
H 1.10849500 4.27693500 -1.19741300
C 1.81112300 -1.04450000 2.04310000
C 0.45977100 0.79957200 1.60021100
C 4.17615500 4.37799200 0.24233500
H 4.94228200 2.89408300 1.58570300
H 3.12793400 5.69544500 -1.11190500
C -0.31993500 -0.41581800 1.51003800
H 0.05047000 1.68433900 2.06795800
H 5.05636500 5.00870500 0.32305700
H -1.37278900 -0.47718600 1.75481000
Mo -0.20751500 0.37330200 -0.72680500
N -0.82864200 0.31037900 -2.69484600
C -1.15292200 -0.85435100 -3.38565000
C -1.06357100 1.39433900 -3.53740000
C -1.61403700 -0.50783200 -4.63846700
C -0.96016100 -2.20916700 -2.78423100
C -1.55572200 0.91634500 -4.73523200
C -0.68228500 2.79436100 -3.17324800
H -1.94635300 -1.20179200 -5.40067100
H -1.56716300 -2.35512900 -1.88288100
H 0.08834200 -2.40010900 -2.52085500
H -1.27227200 -2.97815500 -3.49726000
H -1.81850500 1.52297900 -5.59311400
H -1.10724700 3.12112500 -2.21914100
H 0.40821300 2.90705700 -3.09699800
H -1.03566000 3.48507200 -3.94577000
N 1.32713200 -0.44550100 -1.03912000
C 2.57804800 -0.91823700 -1.23400100
C 2.95337900 -2.24116200 -0.93719700
C 3.58368500 -0.06557700 -1.74047600
C 4.26228800 -2.67781900 -1.07746900
F 2.03450400 -3.09840100 -0.47576600
C 4.89641000 -0.49116300 -1.87753500
F 3.27353500 1.18965100 -2.07748500
C 5.24010400 -1.80067500 -1.54192100
F 4.59440300 -3.93198300 -0.74712300
F 5.83690900 0.34556000 -2.33198100
F 6.50520800 -2.21313700 -1.66652100
O -2.06545800 0.91564300 -0.18719800
C -3.13534200 0.99124300 0.61098700
C -4.09310700 -0.05081800 0.62673300
C -3.32579300 2.13098600 1.43309600
C -3.99885500 -1.24036000 -0.26525900
C -5.20151400 0.06438900 1.47614500
C -4.44614000 2.20127800 2.26479100
C -2.36244100 3.26466700 1.39007600
C -3.86805500 -2.52932100 0.29351700
C -4.17434100 -1.09199300 -1.65895600
H -5.93978500 -0.73217700 1.46562200
C -5.38562800 1.17297700 2.29704100
H -4.58035800 3.08679200 2.88024700
C -1.50738000 3.52328100 2.48108700
C -2.32470400 4.10005100 0.25494800
C -3.90895500 -3.64412900 -0.55010800
C -3.71176700 -2.74002100 1.78310000
C -4.21773700 -2.23371100 -2.46197200
C -4.33141700 0.26562500 -2.29395800
H -6.25824800 1.24353200 2.93929200
C -0.58554300 4.57153000 2.39194000
C -1.57954800 2.69919500 3.74837600
C -1.39610400 5.14582800 0.21017000
C -3.29421000 3.88887600 -0.88225800
C -4.08112900 -3.51750300 -1.92995900
H -3.81083600 -4.63512900 -0.11330700
H -3.30247900 -3.73158700 1.99245800
H -3.04432500 -1.99898700 2.23303700
H -4.67128700 -2.65469000 2.30723200
H -4.34377900 -2.10984900 -3.53464500
H -4.75999700 0.17776400 -3.29583200
H -4.97400100 0.91782100 -1.69311200
H -3.35973400 0.75707800 -2.39765300
C -0.51151700 5.39345300 1.26393800
H 0.08907200 4.75255300 3.22632900
H -1.76577100 1.64136900 3.54123700
H -0.65288800 2.78607300 4.32506900
H -2.40021100 3.03785600 4.39255800
H -1.36919500 5.78701000 -0.66829000
H -3.17500000 4.66133800 -1.64758500
H -3.15097900 2.91388000 -1.35625700
H -4.32858700 3.91806400 -0.52100300
C -4.07337400 -4.72927700 -2.82886500
C 0.48311400 6.52642400 1.20385100
H -4.34481700 -5.63813300 -2.28139300

H -4.77163900 -4.61316200 -3.66479200
H -3.07675700 -4.89179600 -3.26185200
H 1.49644400 6.17598000 1.42640500
H 0.49971400 6.98863300 0.21192600
H 0.23576600 7.30901700 1.93217500
C 1.83685600 0.34329400 1.81212500
N 0.49334600 -1.48728500 1.94105800
C 3.03234500 1.00630900 1.52367300
C 2.99169700 -1.77539000 2.20469000
C 4.22005000 0.27553400 1.69124500
C 4.18974800 -1.07603500 2.06264000
H 5.17371500 0.73277400 1.45152300
H 5.12764100 -1.61290200 2.17085200
H 2.97576100 -2.84356400 2.38005600
C 0.07883600 -2.84439500 1.93985400
C 0.30206900 -3.64938000 3.07780700
C -0.53843700 -3.36485500 0.80229300
C -0.08628800 -4.99793500 3.00669400
C -0.90906700 -4.70393800 0.75658500
H -0.69694700 -2.72247900 -0.05236700
C -0.67442200 -5.52606000 1.86152700
H 0.04690800 -5.62532100 3.88229600
H -1.38102700 -5.09564300 -0.13788700
H -0.96613600 -6.57186700 1.83740600
C 0.87726100 -3.07445400 4.30330100
C 1.54971400 -3.74992900 5.24273100
H 0.73742200 -2.00281200 4.42712000
H 1.92067100 -3.25190300 6.13294000
H 1.76380300 -4.81183300 5.15426900

3b-Metallacycle:

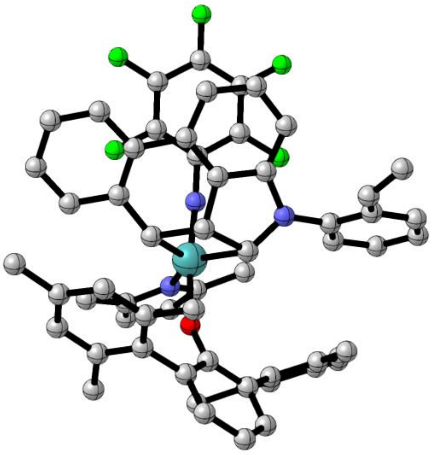

G_sol = –3084.78279

C 0.74112100 1.83627800 0.37173500
H -0.00730200 2.61658500 0.47711900
C 2.09835400 2.42530900 0.27114200
C 3.23859800 1.87970300 0.91416400
C 2.23554000 3.60920300 -0.46711600
C 4.46624600 2.54927200 0.80004600
C 3.46597600 4.24514200 -0.59114300
H 1.35474700 4.02831100 -0.94229700
C 1.64071300 -1.31230700 2.01277100
C 0.56026400 0.73775300 1.47294500
C 4.58754300 3.71617800 0.05372700
H 5.33903400 2.15082400 1.30719900
H 3.54860700 5.15484400 -1.17879300
C -0.38211100 -0.52146700 1.17977000
H 0.09520900 1.24764900 2.32146300
H 5.55030900 4.21248200 -0.02285500
H -1.41320300 -0.44324500 1.51639100
Mo -0.20684500 0.36488400 -0.78591800
N -0.83367000 0.29514200 -2.73080700
C -1.16638600 -0.82893900 -3.47937900
C -0.96920000 1.42393400 -3.54133500
C -1.54813100 -0.41183900 -4.73706300
C -1.04857800 -2.21014900 -2.92175100
C -1.41968700 1.01185800 -4.77735900
C -0.53243600 2.78179200 -3.08480500
H -1.87461600 -1.06006400 -5.54072400
H -1.69321500 -2.35938100 -2.04879800
H -0.01764500 -2.44581800 -2.62598800
H -1.35686000 -2.94114400 -3.67551300
H -1.61600900 1.66061900 -5.62198000
H -0.92132100 3.04226400 -2.09513500
H 0.56307300 2.85985900 -3.03385700
H -0.88615100 3.54386400 -3.78673700
N 1.33065400 -0.44738200 -1.11997100
C 2.57579900 -0.95744000 -1.25485400
C 2.88857600 -2.28715700 -0.91817600
C 3.63041000 -0.14459400 -1.72420600
C 4.19075400 -2.76098700 -0.96375600
F 1.91280700 -3.10935100 -0.51196100
C 4.93609200 -0.61017600 -1.77032500
F 3.37336200 1.10488200 -2.11948000
C 5.21959500 -1.92029000 -1.38432000
F 4.46704500 -4.01528800 -0.58776500
F 5.92618400 0.18760000 -2.18671900
F 6.47789500 -2.36924400 -1.41791200
O -1.96755300 1.14833500 -0.22860000
C -3.03971900 1.28476500 0.56151500
C -4.06294500 0.30593400 0.55410200
C -3.16197800 2.41939500 1.40392000
C -4.03355700 -0.88812400 -0.33633300
C -5.17490400 0.48546800 1.38778400
C -4.28864300 2.55472700 2.21853000
C -2.10932100 3.47072800 1.41915300
C -3.98299100 -2.18073000 0.22653700
C -4.19876000 -0.73291800 -1.73013500
H -5.96191200 -0.26247900 1.35890600
C -5.29824200 1.59471700 2.21856400
H -4.36831300 3.43550300 2.84995500
C -1.25936200 3.61325100 2.53382900

C -1.97559700 4.33930400 0.31669200
C -4.09309400 -3.29313600 -0.61402100
C -3.84618900 -2.39862200 1.71693000
C -4.31216800 -1.87144000 -2.53039500
C -4.28154000 0.63201700 -2.36366300
H -6.17455800 1.71626700 2.84808400
C -0.25215700 4.58341000 2.50349600
C -1.42136500 2.74648500 3.76314300
C -0.95811700 5.29765400 0.32623400
C -2.94979100 4.26670800 -0.83429100
C -4.25772500 -3.15994500 -1.99388100
H -4.05294700 -4.28655600 -0.17432400
H -3.46845400 -3.40225300 1.92807300
H -3.16503800 -1.67667500 2.17633500
H -4.80917100 -2.28794000 2.23052100
H -4.43042600 -1.74334700 -3.60343000
H -4.68130900 0.56506000 -3.37897700
H -4.91727000 1.30661000 -1.77996300
H -3.28914900 1.08605300 -2.43366800
C -0.07785700 5.42927100 1.40556300
H 0.41457600 4.67837800 3.35807100
H -1.72117300 1.72494400 3.50932900
H -0.49223400 2.70998300 4.34151000
H -2.20322100 3.13824700 4.42579700
H -0.85722000 5.96629600 -0.52612800
H -2.70385700 5.00459600 -1.60357500
H -2.95482000 3.27715500 -1.29890300
H -3.97218900 4.46014100 -0.48830100
C -4.32777000 -4.37282500 -2.88859900
C 1.04076200 6.44091900 1.38198200
H -3.34307900 -4.60227800 -3.31834000
H -4.66006800 -5.25955400 -2.33840100
H -5.01496800 -4.21415600 -3.72671000
H 1.99373000 5.95976000 1.12981800
H 0.85635800 7.22204000 0.63731900
H 1.16421200 6.92328000 2.35784500
C 1.85021700 0.04905700 1.77955700
N 0.29452000 -1.61314800 1.78716100
C 3.12474800 0.57176400 1.58679500
C 2.70791000 -2.16376100 2.29780400
C 4.21099800 -0.27868000 1.87673000
C 3.99056700 -1.60407000 2.25952300
H 5.22927700 0.06786900 1.74085900
H 4.84528800 -2.24246500 2.46393700
H 2.55659000 -3.22006400 2.48108500
C -0.25602500 -2.91917100 1.82852700
C -0.25462400 -3.64920900 3.03700300
C -0.77741000 -3.46980400 0.65668000
C -0.77208200 -4.95507100 3.00914000
C -1.27893600 -4.76679800 0.65551900
H -0.76148900 -2.88170200 -0.25005300
C -1.27069400 -5.51435000 1.83602200
H -0.81166000 -5.52073600 3.93467900
H -1.67687800 -5.18593100 -0.26276400
H -1.66775500 -6.52514600 1.84641100
C 0.23412100 -3.03467600 4.28008500
C 0.71419200 -3.69271900 5.34182100
H 0.20081900 -1.94772400 4.30735000
H 1.03407600 -3.16134800 6.23256700
H 0.81733800 -4.77457600 5.35735900

3b-TS2:

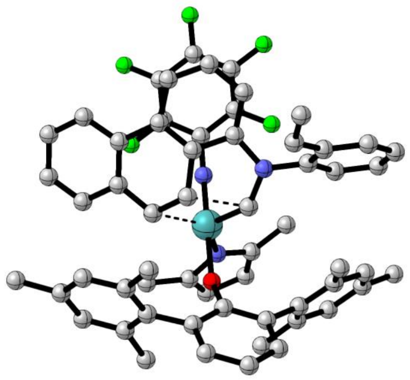

G_sol = –3084.772545

C -0.63617700 1.89751300 -0.69316400
H 0.16833800 2.62121000 -0.71522700
C -1.97815700 2.50190400 -0.59599700
C -3.09960000 1.91371900 -1.23542100
C -2.12204600 3.73167200 0.05946600
C -4.32211400 2.60329800 -1.21734500
C -3.34799900 4.38648600 0.08790800
H -1.25140400 4.17001000 0.53664900
C -1.56009200 -1.43052900 -1.84035700
C -0.46727700 0.73509900 -1.52515400
C -4.45029800 3.82384900 -0.56388300
H -5.18607800 2.17981200 -1.71900600
H -3.44451700 5.33522500 0.60776300
C 0.48588200 -0.94587900 -0.71986900
H 0.31067700 0.75866400 -2.28599900
H -5.40818700 4.33534000 -0.55957300
H 1.54941600 -1.04567700 -0.93938800
Mo 0.13867400 0.38680900 0.77097800
N 0.72723500 0.35725200 2.75852500
C 0.98588400 -0.75622700 3.54717200
C 0.94535600 1.50004900 3.52499900
C 1.40035300 -0.32175500 4.79051600
C 0.76193700 -2.14424900 3.03775700
C 1.36923300 1.10788100 4.77839300
C 0.58036600 2.85913600 3.01027300

H 1.68345500 -0.95999300 5.61853200
H 1.33528700 -2.34527800 2.12559400
H -0.29612300 -2.33023300 2.81105900
H 1.07891400 -2.87404800 3.78928400
H 1.60767100 1.77187900 5.60023300
H 0.91387200 3.02336100 1.97948700
H -0.50756400 3.02240900 3.02795700
H 1.04033000 3.63885500 3.62601800
N -1.48433400 -0.22514200 1.11887200
C -2.74022400 -0.70881800 1.23724900
C -3.02576800 -2.07568700 1.05765300
C -3.82578100 0.14603200 1.52131800
C -4.32637500 -2.55446100 1.07262600
F -2.02069800 -2.93352600 0.82890300
C -5.13065300 -0.32554400 1.53902000
F -3.59879800 1.43787200 1.76967400
C -5.38428700 -1.67735800 1.30764700
F -4.57295700 -3.85051700 0.84669300
F -6.14871000 0.50869400 1.77960000
F -6.64082700 -2.13263000 1.31382400
O 1.97665100 1.07250100 0.28490000
C 3.06712700 1.14538700 -0.48472000
C 4.01326500 0.08738200 -0.49609900
C 3.30063300 2.29510200 -1.28555700
C 3.89442800 -1.11390100 0.37880800
C 5.13955600 0.19520100 -1.32339400
C 4.43848500 2.35474700 -2.09379300
C 2.36827200 3.45492200 -1.25107300
C 3.78349700 -2.39920200 -0.19468500
C 4.01671300 -0.98022400 1.78004400
H 5.86405900 -0.61377700 -1.30829000
C 5.35949700 1.30998000 -2.12552300
H 4.60018900 3.24694200 -2.69280600
C 1.55935400 3.76146200 -2.36425400
C 2.31147000 4.26483200 -0.09828900
C 3.76730300 -3.52025400 0.64149100
C 3.71102200 -2.61199300 -1.69136200
C 4.00908200 -2.12809100 2.57486100
C 4.18172900 0.36783600 2.43225900
H 6.24543600 1.37369400 -2.75006100
C 0.67085500 4.83854000 -2.28706100
C 1.61713500 2.94202000 -3.63336900
C 1.40404600 5.32795800 -0.05772400
C 3.24824200 4.01650200 1.05895200
C 3.87471300 -3.40600700 2.02812700
H 3.67116700 -4.50555500 0.19257600
H 3.24231300 -3.57194900 -1.92285200
H 3.14675000 -1.82346300 -2.19853800
H 4.71008600 -2.61366000 -2.14465800
H 4.09105800 -2.01311600 3.65277300
H 4.57533400 0.26129400 3.44662400
H 4.85832500 1.00998700 1.85858600
H 3.21925400 0.88060400 2.50947200
C 0.56837900 5.62676100 -1.13838900
H 0.03120900 5.05614900 -3.13997700
H 2.38563500 3.32127300 -4.31908300
H 1.87254600 1.89789500 -3.43250700
H 0.65977200 2.97640800 -4.16355300
H 1.35828200 5.94573400 0.83655400
H 3.10559100 4.76412000 1.84467100
H 3.09531700 3.02773900 1.49879500
H 4.29234600 4.06019900 0.72765700
C 3.80454400 -4.62369200 2.91615800
C -0.43075100 6.75539500 -1.06835000
H 2.78626500 -4.77121700 3.30118100
H 4.08594100 -5.53314900 2.37454200
H 4.46508900 -4.52605600 3.78451400
H -1.45713500 6.36799700 -1.07097700
H -0.29867100 7.34955700 -0.15859200
H -0.33557600 7.42842500 -1.92845800
C -1.70144800 -0.03696000 -1.80648600
N -0.26727400 -1.84809700 -1.45977900
C -2.97319200 0.54442700 -1.76853700
C -2.63934100 -2.25693300 -2.12186000
C -4.07603800 -0.29252900 -2.05275800
C -3.89667900 -1.65358200 -2.27468800
H -5.08239000 0.10997800 -2.03713300
H -4.75970000 -2.27857200 -2.48381700
H -2.52287800 -3.33384000 -2.15246100
C 0.22647100 -3.16094100 -1.71149900
C 0.32925000 -3.64492700 -3.03329700
C 0.59599100 -3.94856500 -0.61977700
C 0.80439700 -4.95420200 -3.20814500
C 1.06937300 -5.24224300 -0.82300500
H 0.49723700 -3.54056300 0.37874800
C 1.16642200 -5.74720400 -2.12134900
H 0.92686300 -5.33498000 -4.21710400
H 1.36045000 -5.84870900 0.02878300
H 1.53922500 -6.75317500 -2.28971100
C -0.00835200 -2.78211300 -4.17512900
C -0.41233200 -3.20556000 -5.37810000
H 0.07981400 -1.71250800 -3.99866700
H -0.62102600 -2.50286200 -6.17848300
H -0.56295800 -4.25823700 -5.60240200

3b-Alk2:

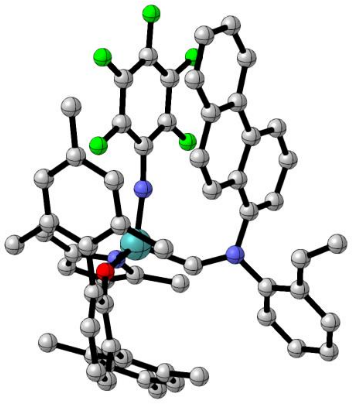

G_sol = –3084.818185

C -2.61099300 -0.00919700 -1.77189200
H -2.54033200 0.99486100 -2.17001500
C -3.90677200 -0.52398600 -1.44690200
C -4.02774200 -1.83453300 -0.90408500
C -5.05903300 0.27527200 -1.62085900
C -5.32071100 -2.29143100 -0.56146100
C -6.30797000 -0.19459200 -1.26577100
H -4.93817800 1.27869200 -2.01958900
C -0.37861300 -2.77518200 -0.63680200
C -1.48352600 -0.72530000 -1.52765900
C -6.43595500 -1.49093800 -0.73401600
H -5.45130400 -3.27440100 -0.12418400
H -7.18492100 0.43426100 -1.38571300
C 1.38609300 -1.14755900 -0.15228500
H -0.51500000 -0.29235200 -1.74399500
H -7.41340600 -1.86228600 -0.44167700
H 2.42752800 -0.91876500 -0.41724200
Mo 0.87256400 0.30039200 1.00798100
N 1.88724300 -0.09112900 2.73141100
C 2.65698800 -1.14888500 3.20897400
C 1.87226500 0.90710000 3.72248100
C 3.14749600 -0.81149700 4.45105100
C 2.84377800 -2.41617300 2.44866100
C 2.65705800 0.49497600 4.77244100
C 1.03093500 2.13921300 3.59217400
H 3.76891100 -1.44312800 5.07385300
H 3.49242200 -2.26334000 1.57999300
H 1.89037300 -2.82193900 2.09160800
H 3.31872900 -3.16561200 3.08977500
H 2.83121000 1.05049400 5.68558500
H 1.29292900 2.75455100 2.72249400
H -0.03987200 1.90025400 3.51441800
H 1.16038100 2.76820800 4.47810900
N -0.79493800 -0.07048000 1.38758000
C -2.11933000 -0.15456600 1.66138100
C -2.67399700 -1.32341900 2.21645900
C -2.99752200 0.90273000 1.36042700
C -4.04206300 -1.44400800 2.41604600
F -1.87865300 -2.35540500 2.50830600
C -4.36241200 0.79887300 1.58315800
F -2.50344800 2.02493400 0.82830700
C -4.88793200 -0.38103600 2.10494100
F -4.56165700 -2.59178600 2.87091900
F -5.18447100 1.81101100 1.26859600
F -6.20585800 -0.50349400 2.28251000
O 1.35151500 1.94029900 0.08643400
C 2.03403000 2.53770800 -0.91836000
C 3.41163100 2.29102400 -1.09942400
C 1.34771400 3.44229900 -1.75415600
C 4.13711600 1.28985600 -0.26823500
C 4.09176800 2.99102100 -2.10212600
C 2.06647600 4.11454100 -2.74921400
C -0.11726700 3.67695100 -1.61021600
C 4.56440000 0.08053400 -0.85592900
C 4.42235600 1.55391900 1.08775100
H 5.15564900 2.80997800 -2.22758900
C 3.43254500 3.90336600 -2.92280800
H 1.53536600 4.81869400 -3.38366700
C -0.99923800 3.17668900 -2.58567400
C -0.61769800 4.43454500 -0.53287900
C 5.25932500 -0.84670600 -0.07107100
C 4.26633600 -0.24010900 -2.30420200
C 5.10255900 0.59464700 1.83985400
C 4.05640500 2.87494100 1.71901300
H 3.97801600 4.44362300 -3.69077700
C -2.36898600 3.43704600 -2.46247300
C -0.49499400 2.34300700 -3.74211000
C -1.99159300 4.66147000 -0.43955700
C 0.31427500 4.99269500 0.51353400
C 5.53502900 -0.60860400 1.27769500
H 5.57736100 -1.78255600 -0.52461000
H 4.45276900 -1.29652400 -2.51460800
H 3.22334800 -0.02067100 -2.55879400
H 4.88294000 0.35599300 -2.98679800
H 5.27798900 0.78330200 2.89503400
H 4.11028000 2.80799700 2.80866900
H 4.74096200 3.66607700 1.38599600
H 3.04864700 3.19256100 1.44554600
C -2.88793000 4.15526400 -1.38443200
H -3.04811200 3.05340800 -3.22210500
H 0.18924800 1.55873900 -3.39869700
H -1.32616600 1.86461300 -4.26944100
H 0.06198700 2.94316000 -4.47074400
H -2.37438800 5.23366900 0.40289900
H -0.20864100 5.69005200 1.17494800
H 0.72863000 4.18664500 1.12859400
H 1.16243200 5.51650200 0.05895900
C 6.26847600 -1.62074800 2.12167300
C -4.37610900 4.31905000 -1.20203100

H 5.75576700 -1.76542200 3.07880900
H 6.33804200 -2.59001400 1.61732900
H 7.29028900 -1.28824500 2.34486200
H -4.75713700 3.56154200 -0.50547000
H -4.62812800 5.30049700 -0.78549600
H -4.91395700 4.20357100 -2.14936500
C -1.54847100 -2.03507100 -0.94729900
N 0.92616300 -2.21667100 -0.86395300
C -2.82178200 -2.61750300 -0.66839300
C -0.45818800 -4.05758200 -0.12540400
C -2.86818300 -3.92733600 -0.14289600
C -1.71154100 -4.63803000 0.11377800
H -3.82368500 -4.39233400 0.06732500
H -1.77017700 -5.64315900 0.51969700
H 0.45530300 -4.60189900 0.08980100
C 1.84249100 -2.98443800 -1.66516300
C 1.54532800 -3.26630800 -3.01592400
C 3.02348400 -3.45514500 -1.08678400
C 2.47882700 -4.01762500 -3.74817800
C 3.93633200 -4.19387000 -1.83634800
H 3.21486200 -3.24427700 -0.04383200
C 3.66061900 -4.47887200 -3.17423500
H 2.27985800 -4.20353500 -4.79885200
H 4.85203800 -4.54856400 -1.37245800
H 4.36663200 -5.04789200 -3.77170700
C 0.32159500 -2.75898800 -3.65576100
C -0.36129700 -3.39262100 -4.61518100
H -0.04148800 -1.79866100 -3.30473600
H -1.24717600 -2.94758100 -5.05742200
H -0.07643300 -4.37828200 -4.97419300

4a-Alk1:

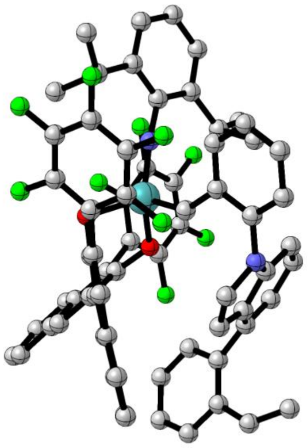

G_sol = –3905.714963

Mo -0.81540100 0.50912200 -0.22498400
O 1.14733000 0.59266300 -0.10302200
O -0.92050600 -0.36803500 -1.97533400
N -2.07064500 1.69612900 0.04213400
C -1.30480300 -0.83930100 1.00053500
C -2.83371400 2.79208600 0.37407400
C -2.40991700 3.66906800 1.40710300
C -3.20977900 4.77781700 1.68379500
C -4.38822200 5.00996400 0.97508600
C -4.80224800 4.12429400 -0.01923300
C -4.04362100 2.99772100 -0.33800400
C 1.95393500 0.49909200 -1.18357400
C 1.94426000 -0.68415200 -1.99714000
C 2.87686100 -0.76964300 -3.01243300
C 3.82112200 0.25070700 -3.27768800
C 3.77989300 1.45465600 -2.50514400
C 2.81173900 1.55482100 -1.46526300
C -0.38625200 -1.62329600 -1.88761700
C 0.99230800 -1.81303900 -1.80279800
C 1.49438100 -3.14340900 -1.58775200
C 0.57680700 -4.24219400 -1.53201500
C -0.81246800 -3.99676400 -1.66145600
C -1.30089300 -2.72285700 -1.81915800
C -1.17015600 3.34609000 2.21984000
C -1.49148700 2.25980300 3.26476500
C -0.53722500 4.55999200 2.91086900
C -4.45265000 2.01309200 -1.42006700
C -3.67170700 2.27588100 -2.72319500
C -5.96385300 1.98890700 -1.68186800
H -0.48538000 -1.56328800 0.90659000
H -2.91187100 5.47116000 2.46262700
H -4.99256100 5.88270500 1.20595300
H -5.72984400 4.31314300 -0.54832100
H -0.42156100 2.92838200 1.53034200
H -0.58105700 1.96162000 3.79318700
H -1.91936700 1.36746900 2.80446300
H -2.21143300 2.64523800 3.99653700
H 0.42327800 4.27324700 3.35277100
H -1.17005900 4.92747000 3.72695600
H -0.36353000 5.38520600 2.21748800
H -4.15769000 1.01606600 -1.06875200
H -3.91065900 1.51306700 -3.47070900
H -2.59221400 2.25539000 -2.54921600
H -3.93430500 3.25807600 -3.13378400
H -6.52994100 1.83955700 -0.75568700
H -6.21209600 1.17590500 -2.36763900
H -6.30838600 2.92114500 -2.14419500
C -2.75225300 -2.43749100 -1.80859200
C -3.36847700 -1.67704300 -2.80813100
C -3.55899100 -2.85824000 -0.74617500
C -4.71286100 -1.33075500 -2.73977700
C -4.90114500 -2.50506200 -0.64683200
C -5.47884200 -1.73014500 -1.64825900
C 2.62783200 2.79964400 -0.68531100
C 3.59035000 3.30279300 0.18951000
C 1.42958400 3.51241400 -0.78059700
C 3.38515200 4.46998000 0.92274000

C 1.19623400 4.67841600 -0.06568400
C 2.18368600 5.16156300 0.79044800
F -3.03779300 -3.59963000 0.24551300
F -5.63320200 -2.89558900 0.40253500
F -6.76458100 -1.37430100 -1.56725300
F -5.27467900 -0.60296600 -3.71652200
F -2.67356100 -1.26958600 -3.87457200
F 0.44035500 3.05814100 -1.57897600
F 0.03100000 5.32776800 -0.18152000
F 1.97113400 6.27883100 1.49189000
F 4.75135800 2.64786100 0.35056000
F 4.32786400 4.92585700 1.75446400
C 2.86985100 -3.41285600 -1.35963700
C 1.06411800 -5.55346700 -1.30125500
C 3.31144200 -4.69757400 -1.12598000
C 2.40768500 -5.78317700 -1.10634800
H 2.77082600 -6.78983300 -0.92160700
H 4.36593000 -4.86701800 -0.93536700
H 0.35086800 -6.37305200 -1.26700200
H 3.57591500 -2.59345400 -1.32861500
H -1.50080000 -4.83447400 -1.60179300
C 4.70447300 2.48746000 -2.81422600
C 4.79039200 0.11515300 -4.30500800
C 5.68635600 1.12581700 -4.56772400
C 5.63516600 2.32389900 -3.81615100
H 4.80944400 -0.80635900 -4.88116700
H 2.89001100 -1.65866700 -3.63626400
H 6.42732700 1.01174200 -5.35321900
H 6.33691500 3.12402400 -4.03350600
H 4.67181200 3.41553900 -2.25426700
C -2.02631800 -2.19685100 2.92691800
C -3.62758900 -0.67422300 1.92857400
N -0.68375300 -2.70035100 2.97756900
C -2.98844300 -2.62832900 3.82978600
C -4.59368300 -1.11631400 2.82937900
H -3.86364800 0.07984900 1.18512900
C 0.37284900 -1.84420400 3.30856800
C -0.19123200 -3.62657200 2.04655000
C -4.28009900 -2.09444700 3.77580500
H -2.71913700 -3.37579400 4.56919800
H -5.59663700 -0.70245400 2.78801400
C 1.52590100 -2.25789300 2.59359700
C 0.35518500 -0.72777100 4.14530000
C 1.13966500 -3.40984500 1.81841900
H -0.86595500 -4.35099400 1.61582100
H -5.03614800 -2.43958500 4.47439600
C 2.70595700 -1.48432700 2.68667600
C 1.53580500 -0.00316900 4.25656200
H 1.77955300 -4.00073500 1.18287800
C 2.68240100 -0.36849000 3.52731700
C 3.88208100 -1.71456200 1.80461300
H 1.57374300 0.86756700 4.90503200
H 3.58046300 0.23639900 3.61130900
C 4.23946900 -0.64837500 0.96411300
C 4.62432700 -2.91860200 1.73404200
C 5.27912500 -0.74745700 0.04258200
H 3.66111800 0.26539700 1.02538600
C 5.66039400 -3.00492600 0.78547500
C 4.42633400 -4.09180700 2.60276700
C 5.99018800 -1.94407900 -0.05366300
H 5.51440400 0.09737000 -0.59611700
H 6.23078200 -3.92925900 0.72834200
C 3.88932600 -4.13618000 3.82766500
H 4.82440200 -5.01839400 2.18810700
H 6.79816300 -2.04933200 -0.77222300
H 3.49602800 -3.25862700 4.32713900
H 3.83518500 -5.07529700 4.37055000
C -2.33078900 -1.21331100 1.95060200
H -0.54740900 -0.43776500 4.67251600

4a-TS1:

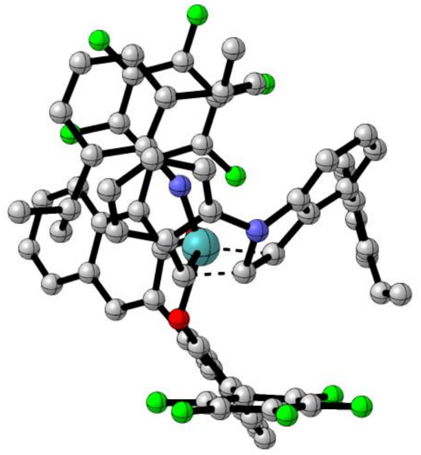

G_sol = –3905.696718
C 1.50452900 -1.51911000 -1.86298400
H 2.02591200 -0.88975000 -2.57450600
C 1.05157000 -2.85119000 -2.29877700
C 1.25904300 -3.79790700 -1.27283800
C 0.45356500 -3.24967800 -3.49161100
N 2.00479500 -3.22008100 -0.22624700
C 0.77502000 -5.09874300 -1.37667300
C -0.02020300 -4.55858700 -3.61506000
H 0.33618900 -2.54171300 -4.30451100
C 1.74686800 -3.39440700 1.15432300
C 2.24058800 -1.80414400 -0.52198300
C 0.11910400 -5.46266500 -2.55754600
H 0.91100000 -5.81844400 -0.57885500
H -0.50356300 -4.87264800 -4.53504700
C 1.59669300 -2.15223200 1.79081100

C 1.64781200 -4.58599700 1.86714900
C 1.73357500 -1.05428200 0.81626400
H 3.29884200 -1.55412000 -0.61368900
H -0.26233400 -6.47431100 -2.65947900
C 1.34073800 -2.06542600 3.16181000
C 1.37460500 -4.50322800 3.23786100
H 2.39378800 -0.23306000 1.10432700
C 1.22080900 -3.26961000 3.87491700
C 1.09660100 -0.74435700 3.80175800
H 1.29557600 -5.41780300 3.81798900
H 1.01544100 -3.22948300 4.94012600
C -0.18156600 -0.50679800 4.32330600
C 2.05735600 0.29754400 3.82534500
C -0.54159000 0.73791700 4.83622000
H -0.91707800 -1.30215000 4.28637000
C 1.66312700 1.55227400 4.32271000
C 3.45154000 0.18265100 3.36515500
C 0.38542000 1.77768700 4.82576400
H -1.55086700 0.89700300 5.19920600
H 2.38988100 2.36057000 4.32144300
C 4.21566400 -0.91165200 3.23961200
H 3.90783600 1.14044000 3.12557200
H 0.11521500 2.76111500 5.19891200
H 3.86730600 -1.90800600 3.48675900
H 5.23399600 -0.82260100 2.87393900
Mo 0.32964200 -0.30834600 -0.45603600
N -0.94375000 -1.51240400 -0.51906500
O 1.28729000 1.15888600 -1.49019500
C 1.73322700 2.31284400 -0.96326100
C 0.88690100 3.37001200 -0.62869200
C 3.15418900 2.44132400 -0.78303400
C -0.58047700 3.28839600 -0.84757500
C 1.43343600 4.55409300 -0.02340600
C 3.68061600 3.60175500 -0.26128600
C 4.04867600 1.30478200 -1.10692200
C -1.35899500 2.24444900 -0.25253100
C -1.24922900 4.28177500 -1.53106800
C 2.85285000 4.67416500 0.13683400
C 0.62980400 5.61815500 0.47795900
H 4.75483700 3.69611500 -0.13396800
C 4.05487700 0.66829500 -2.35338300
C 4.95013600 0.80719200 -0.15628000
O -0.74763900 1.17268700 0.26391200
C -2.75155100 2.32548500 -0.18715800
C -2.66407800 4.36781700 -1.55315900
H -0.67548300 5.06063400 -2.02570600
C 3.40760100 5.84631000 0.71475500
C 1.19596000 6.73594300 1.04966700
H -0.44838900 5.53793100 0.42448800
C 4.85217400 -0.44257600 -2.61616600
F 3.26774000 1.10103000 -3.34472900
C 5.76104800 -0.29809100 -0.39533900
F 5.04447000 1.38450600 1.05293000
C -3.43440700 3.41266700 -0.81380600
C -3.47963200 1.25640600 0.53943400
C -3.32626300 5.42221900 -2.23297900
C 2.60012600 6.86634700 1.15828200
H 4.48866300 5.91264400 0.80963700
H 0.55252000 7.52481000 1.42928300
C 5.71018900 -0.93221800 -1.63449900
F 4.78502800 -1.05075300 -3.80600300
F 6.58339300 -0.75370000 0.55883600
C -4.84127500 3.59477700 -0.74823600
C -3.16027000 0.96445100 1.87333500
C -4.49393200 0.48370300 -0.03740900
C -4.69501900 5.55275500 -2.17352300
H -2.72558200 6.13356500 -2.79411400
H 3.03178900 7.75788500 1.60342100
F 6.47792300 -1.99619500 -1.87947200
C -5.45432700 4.63443900 -1.41168100
H -5.43835700 2.91085800 -0.15636400
C -3.78606500 -0.05680700 2.57715000
F -2.23494400 1.68553100 2.51602300
C -5.14873800 -0.53052400 0.65503100
F -4.87926400 0.69869700 -1.30683600
H -5.19216500 6.36440800 -2.69641800
H -6.53216600 4.75244500 -1.34585600
C -4.78449200 -0.80820200 1.96590000
F -3.43262600 -0.33606100 3.84224000
F -6.12101000 -1.23682100 0.06677600
F -5.37502700 -1.80722600 2.63229800
C -2.07119300 -2.13954400 -0.99080000
C -2.63970300 -3.15785400 -0.17972800
C -2.67613800 -1.73331100 -2.21402800
C -3.90348100 -3.63500700 -0.51995700
C -1.84979500 -3.70425400 0.99628700
C -3.94800600 -2.23565600 -2.49334900
C -1.92967100 -0.86567600 -3.22078100
C -4.57026400 -3.15077000 -1.64569900
H -4.37305900 -4.39548500 0.09527100
C -2.05586200 -5.20995100 1.20212700
C -2.12894400 -2.92448600 2.28835800
H -0.79424200 -3.55481000 0.75946000
H -4.45638300 -1.91971700 -3.39808000
C -2.22625200 0.65697000 -3.13647000
C -2.15655900 -1.36712100 -4.65783300
H -0.86862400 -1.00378700 -3.00331100
H -5.56695700 -3.51187200 -1.88053800
H -1.34800800 -5.57389700 1.95461200
H -1.88537000 -5.76254100 0.27202300
H -3.06540600 -5.44319100 1.55993000
H -1.53452000 -3.33674600 3.10988500
H -3.18666200 -2.97956300 2.56495800
H -1.85216600 -1.87506400 2.16500300

H -2.95402900 0.89302600 -2.36630900
H -2.63299100 1.02533800 -4.08468300
H -1.31771700 1.22957200 -2.92790800
H -1.99711600 -2.44611900 -4.74148500
H -1.46624600 -0.85650500 -5.33939500
H -3.17261300 -1.14597500 -5.00277700
H 1.80033500 -5.54596500 1.38908600

4a-Metallacycle:

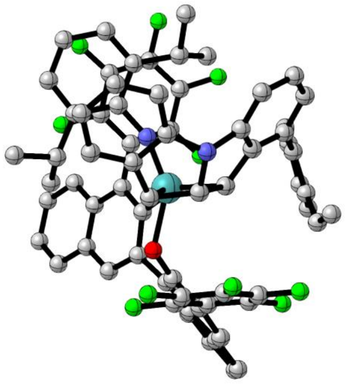

G_sol = –3905.702039

C 1.44210700 -1.55626800 -1.85170200
H 1.96234800 -0.94495200 -2.57960400
C 0.97427000 -2.89285300 -2.25658700
C 1.18976200 -3.82285800 -1.21697700
C 0.35910500 -3.30899800 -3.43442600
N 1.95348500 -3.23177500 -0.19088300
C 0.69719500 -5.12246300 -1.29149600
C -0.12339500 -4.61710400 -3.52875500
H 0.23530200 -2.61451200 -4.25780300
C 1.71438200 -3.38214700 1.19618800
C 2.19051900 -1.82219700 -0.51330100
C 0.02422600 -5.50328700 -2.45727400
H 0.83960500 -5.82858300 -0.48268500
H -0.62019700 -4.94458900 -4.43683900
C 1.57233900 -2.12895400 1.81244200
C 1.62388600 -4.56064700 1.93144100
C 1.70070200 -1.04975700 0.81750900
H 3.24830200 -1.57728700 -0.61990400
H -0.36430900 -6.51425900 -2.53649500
C 1.32941900 -2.01656200 3.18413700
C 1.36516400 -4.45268900 3.30327700
H 2.36480700 -0.22432800 1.08311100
C 1.21646300 -3.20784700 3.91965600
C 1.08962900 -0.68288700 3.79957500
H 1.29256400 -5.35659800 3.90072400
H 1.02126300 -3.14902100 4.98592600
C -0.18148600 -0.43677200 4.33460900
C 2.04555700 0.36340600 3.78145200
C -0.53993200 0.82030200 4.81737900
H -0.91390600 -1.23565600 4.33078800
C 1.65287900 1.63017200 4.24816400
C 3.43346000 0.23848700 3.30492500
C 0.38130100 1.86395900 4.76287600
H -1.54484600 0.98519500 5.19012300
H 2.37558700 2.44131500 4.21275600
C 4.20119600 -0.85718500 3.22138500
H 3.87938700 1.18656200 3.01255000
H 0.11118000 2.85659900 5.11081500
H 3.85998500 -1.84130300 3.52231000
H 5.21371900 -0.78320700 2.83677100
Mo 0.29472800 -0.31122200 -0.45329400
N -0.99464100 -1.50181600 -0.48759900
O 1.30435900 1.12339200 -1.48678700
C 1.78162900 2.26993400 -0.97377200
C 0.96552700 3.35088100 -0.63788700
C 3.20783400 2.36687100 -0.81054200
C -0.50380400 3.31135600 -0.85433800
C 1.54613600 4.52182100 -0.03916000
C 3.76684700 3.51540400 -0.29606600
C 4.07249500 1.21066500 -1.14500000
C -1.31213800 2.29179200 -0.25888900
C -1.14449300 4.32045700 -1.54214900
C 2.96910800 4.60728300 0.10992300
C 0.77266100 5.60586900 0.46696900
H 4.84429400 3.58510400 -0.18136800
C 4.04015700 0.56576600 -2.38700400
C 4.98209300 0.69903400 -0.20955500
O -0.73427500 1.20370500 0.26334400
C -2.70304500 2.40506500 -0.20584900
C -2.55684300 4.44037100 -1.57497000
H -0.54878200 5.08330300 -2.03574600
C 3.55692400 5.76561200 0.68303900
C 1.37037900 6.70949200 1.03403200
H -0.30758400 5.55213700 0.42131600
C 4.80387500 -0.56702100 -2.65605600
F 3.24708700 1.01145800 -3.36776900
C 5.75872800 -0.42918900 -0.45457400
F 5.12000300 1.28514000 0.99148900
C -3.35608500 3.50271300 -0.84414000
C -3.45665700 1.34874200 0.51248600
C -3.18930000 5.50808200 -2.26236800
C 2.77813900 6.80523900 1.13236400
H 4.64000000 5.80543100 0.76950200
H 0.74940900 7.51413700 1.41794600
C 5.66708700 -1.07200400 -1.68685800
F 4.69868900 -1.18158600 -3.83987500
F 6.58648700 -0.89791700 0.48836700
C -4.76001700 3.71022000 -0.79833500
C -3.15879500 1.05533500 1.85057000
C -4.46273200 0.58060400 -0.08342200
C -4.55589400 5.66557200 -2.21997700

H -2.56790200 6.20700000 -2.81649500
H 3.23491100 7.68595600 1.57396500
F 6.40155300 -2.15789300 -1.93747100
C -5.34368000 4.76154100 -1.46980600
H -5.37738800 3.03580200 -0.21627900
C -3.79998300 0.03732000 2.54479600
F -2.23779700 1.77103600 2.50576300
C -5.13349400 -0.42989400 0.59982700
F -4.82504400 0.79892200 -1.35710900
H -5.03004300 6.48709400 -2.74880100
H -6.41993400 4.89998100 -1.41988700
C -4.79071600 -0.71045600 1.91615200
F -3.46699200 -0.24183200 3.81546400
F -6.10103400 -1.13019800 -0.00331200
F -5.39838200 -1.70542500 2.57316000
C -2.12019800 -2.15656500 -0.92758400
C -2.66827300 -3.15719500 -0.08053200
C -2.74445500 -1.80123800 -2.15713100
C -3.92722300 -3.66481000 -0.39446900
C -1.86667400 -3.65693000 1.10768400
C -4.00914600 -2.33407400 -2.41097200
C -2.03708800 -0.94352400 -3.19733300
C -4.61044500 -3.23106300 -1.53015600
H -4.37896700 -4.41172600 0.24988400
C -2.05327400 -5.15811800 1.36068500
C -2.15381700 -2.84141200 2.37536400
H -0.81410200 -3.50138000 0.86292200
H -4.52804300 -2.05616000 -3.32226500
C -2.41043200 0.54469100 -3.14805100
C -2.26055000 -1.48169200 -4.62136400
H -0.96641000 -1.01347400 -2.98466900
H -5.60239300 -3.61659900 -1.74553400
H -1.33586100 -5.49034300 2.11852400
H -1.88271800 -5.73682000 0.44661800
H -3.05738500 -5.39224700 1.73283200
H -1.56279900 -3.22897400 3.21120500
H -3.21287500 -2.89056600 2.64779900
H -1.87888900 -1.79542700 2.22328800
H -1.80254400 1.10500900 -3.86762800
H -2.23811400 0.97378000 -2.16277100
H -3.46514100 0.69558000 -3.39344900
H -2.09460200 -2.56109700 -4.67779700
H -1.57548300 -0.98474500 -5.31793400
H -3.27883700 -1.27697300 -4.97029900
H 1.77043500 -5.52920300 1.46904600

4a-TS2:

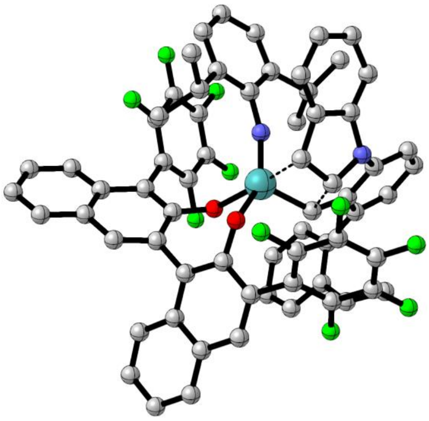

G_sol = –3905.697097

C 1.50397500 -1.57883500 -1.98375600
H 1.93034000 -0.89828500 -2.70757900
C 0.88358700 -2.85161300 -2.37403800
C 1.06059900 -3.77863300 -1.32556600
C 0.18061000 -3.22940100 -3.51533100
N 1.90734700 -3.21062900 -0.34297300
C 0.46060500 -5.03274200 -1.34878100
C -0.40633400 -4.49573800 -3.56207300
H 0.07851200 -2.54151900 -4.34685300
C 1.73008700 -3.38630700 1.05543800
C 2.18572300 -1.86203800 -0.72586100
C -0.28601900 -5.37533500 -2.48036300
H 0.56577400 -5.72766200 -0.52466200
H -0.96710700 -4.79689100 -4.44155000
C 1.51279200 -2.18138900 1.74535800
C 1.75375400 -4.60345900 1.73045700
C 1.49903600 -0.98186400 0.90567500
H 3.18914000 -1.49809700 -0.53881200
H -0.76101500 -6.35079400 -2.52430300
C 1.33305400 -2.17445300 3.13597800
C 1.52662700 -4.60123100 3.11064300
H 2.23803600 -0.20982700 1.15012200
C 1.31975300 -3.40653400 3.80419300
C 1.09532700 -0.88394100 3.84117500
H 1.53705600 -5.54193700 3.65269900
H 1.16866100 -3.41875800 4.87915100
C -0.16565800 -0.66356700 4.40768100
C 2.06060900 0.15410600 3.87217000
C -0.50500500 0.56399300 4.97423600
H -0.90599200 -1.45389500 4.36349400
C 1.68811300 1.38991000 4.42854100
C 3.43383400 0.05374800 3.34780400
C 0.42644600 1.59966700 4.97660200
H -1.50286000 0.71495400 5.37033400
H 2.41727500 2.19595500 4.43377100

C 4.19220200 -1.03660800 3.16360000
H 3.87860200 1.01743900 3.10820500
H 0.17053100 2.56948200 5.39278900
H 3.85758800 -2.03842300 3.40766800
H 5.19373300 -0.93860100 2.75704800
Mo 0.30852100 -0.27849900 -0.47187600
N -1.01100000 -1.42518900 -0.59270300
O 1.29692100 1.15804800 -1.50225500
C 1.79392400 2.29655900 -0.98313000
C 0.98910800 3.37953300 -0.63131000
C 3.21975600 2.37520800 -0.82326600
C -0.48590100 3.33331300 -0.80292400
C 1.58651600 4.54895000 -0.04792000
C 3.79525900 3.51987400 -0.31754300
C 4.06917900 1.20428500 -1.14441700
C -1.26318700 2.31324800 -0.16351000
C -1.15190500 4.32789600 -1.48609200
C 3.01212500 4.62387000 0.08320000
C 0.82735700 5.64359900 0.45717500
H 4.87402300 3.57626100 -0.20649100
C 4.04977100 0.56328300 -2.38799500
C 4.95134700 0.67629200 -0.19170700
O -0.64995500 1.25079100 0.36730900
C -2.65357900 2.41527100 -0.08151300
C -2.56535000 4.43834100 -1.47807700
H -0.57596900 5.08630800 -2.00953900
C 3.61591300 5.78319900 0.63785600
C 1.44067700 6.74811900 1.00507200
H -0.25364300 5.59741300 0.42395500
C 4.80517200 -0.57677400 -2.64857300
F 3.27192100 1.01801000 -3.37755200
C 5.72256500 -0.45757800 -0.42986200
F 5.06469100 1.25183700 1.01695800
C -3.33333800 3.50200600 -0.71379300
C -3.39046000 1.34907600 0.63896500
C -3.22568100 5.49306400 -2.15909200
C 2.85049400 6.83330400 1.08537300
H 4.70021600 5.81545800 0.71123300
H 0.83111500 7.56201700 1.38773000
C 5.64601200 -1.09366600 -1.66674000
F 4.70901500 -1.18937100 -3.83382300
F 6.53226200 -0.93932600 0.52283900
C -4.73701500 3.69969200 -0.63113000
C -3.08705300 1.04179200 1.97293300
C -4.39939500 0.58644900 0.03921000
C -4.59176200 5.64076200 -2.07998500
H -2.62554200 6.19024600 -2.73850800
H 3.31942800 7.71493700 1.51223500
F 6.37010100 -2.18889200 -1.90681400
C -5.34892300 4.73868200 -1.29712100
H -5.33232200 3.02825000 -0.02294200
C -3.73413300 0.02145000 2.65928200
F -2.15858000 1.74358500 2.63243100
C -5.07412300 -0.42612100 0.71474900
F -4.76049100 0.81291000 -1.23347800
H -5.08780500 6.45235300 -2.60408600
H -6.42450300 4.86859600 -1.21676200
C -4.73315400 -0.71331400 2.02952300
F -3.39699000 -0.28024000 3.92431200
F -6.04129100 -1.12292800 0.10643700
F -5.33998900 -1.71356900 2.68097200
C -2.14407400 -2.05608600 -1.04544100
C -2.69141600 -3.08501700 -0.23137000
C -2.76703100 -1.65793300 -2.26090200
C -3.94721700 -3.58553800 -0.56746200
C -1.88752200 -3.62838000 0.93730600
C -4.02875700 -2.18683200 -2.53776000
C -2.06064600 -0.75239700 -3.26050600
C -4.62839100 -3.11600900 -1.69048400
H -4.39895800 -4.35578200 0.04872600
C -2.12692500 -5.12322000 1.18390500
C -2.10835500 -2.82402100 2.22508500
H -0.83567300 -3.51025900 0.66969700
H -4.54697600 -1.87830800 -3.43951100
C -2.40791400 0.73609700 -3.11673200
C -2.31044500 -1.19827200 -4.71153100
H -0.98763700 -0.85171300 -3.06711100
H -5.61807800 -3.49827600 -1.92194000
H -1.39427700 -5.49376000 1.90938600
H -2.02146400 -5.70112500 0.26003200
H -3.12384700 -5.31674500 1.59670300
H -1.49853600 -3.24366700 3.03159800
H -3.15699200 -2.85520400 2.53628800
H -1.81223200 -1.78248500 2.08294600
H -1.79981000 1.32940900 -3.80900600
H -2.21722500 1.10109200 -2.10961600
H -3.46313100 0.91830000 -3.33795700
H -2.17124800 -2.27574400 -4.83741000
H -1.62198800 -0.67479400 -5.38478600
H -3.32753800 -0.95009200 -5.03488400
H 1.96702000 -5.52573800 1.20288300

4a-Alk2:

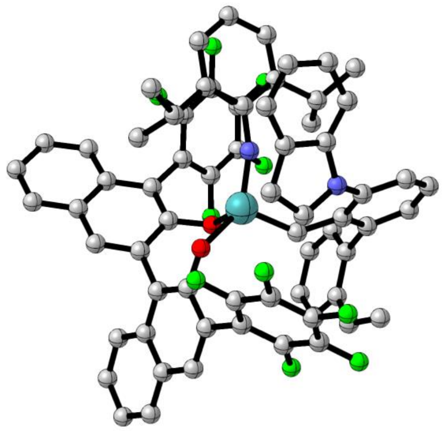

G_sol = –3905.708656

C 1.76854900 -2.01582100 -2.24719400
H 2.09240400 -1.29662400 -2.98437800
C 0.90252300 -3.14913800 -2.46576900
C 0.86181700 -3.88475600 -1.25326500
C 0.15611300 -3.58692600 -3.56672500
N 1.71007600 -3.24883300 -0.33054000
C 0.06432600 -5.01973100 -1.11202900
C -0.64338400 -4.71473100 -3.42893900
H 0.19767000 -3.04395300 -4.50490600
C 1.56822600 -3.31778700 1.08621300
C 2.24964900 -2.12251500 -0.95562300
C -0.69345700 -5.41562300 -2.21058200
H 0.01979400 -5.56724300 -0.17911900
H -1.24043300 -5.05863900 -4.26771500
C 1.32499200 -2.12737900 1.80720900
C 1.65993200 -4.54078500 1.75219600
C 1.20748900 -0.84980900 1.12001200
H 3.04596100 -1.57863000 -0.47587300
H -1.33220900 -6.28932600 -2.12383100
C 1.21364600 -2.18088300 3.21351600
C 1.46784000 -4.59160500 3.13302600
H 1.90038200 -0.06021900 1.44660300
C 1.24928200 -3.41838000 3.85973200
C 1.02150800 -0.89914200 3.95404500
H 1.52046300 -5.54641000 3.64706300
H 1.13568400 -3.45436600 4.93890900
C -0.24910200 -0.59402400 4.45370800
C 2.05909300 0.06216400 4.05877700
C -0.52744200 0.64329300 5.03365000
H -1.04254300 -1.32566100 4.34989500
C 1.75290700 1.30499500 4.64010500
C 3.43235200 -0.10831300 3.55128700
C 0.48183400 1.59935700 5.12342500
H -1.53203700 0.86395900 5.37522300
H 2.53890900 2.05289800 4.70627500
C 4.11116600 -1.22975900 3.26833300
H 3.95516100 0.83509800 3.40369700
H 0.27850100 2.57463700 5.55562100
H 3.70649800 -2.22463600 3.40980900
H 5.12317900 -1.16363300 2.88228500
Mo 0.27826500 -0.15781000 -0.37162700
N -0.99160400 -1.31758200 -0.69558500
O 1.30241900 1.11928300 -1.50003400
C 1.84587600 2.26763300 -1.03670500
C 1.04784100 3.36656500 -0.73037400
C 3.26432000 2.31882000 -0.85823800
C -0.42925400 3.29687400 -0.88727400
C 1.65531000 4.55872200 -0.21603600
C 3.85135000 3.47754600 -0.39763000
C 4.08640200 1.11006600 -1.09785400
C -1.19744400 2.34464400 -0.13778900
C -1.09963300 4.19028100 -1.69362400
C 3.08114100 4.61646500 -0.07321300
C 0.90459700 5.69480700 0.19955700
H 4.92838700 3.51805200 -0.26418400
C 4.09729800 0.42844600 -2.31874100
C 4.90750200 0.59196900 -0.08792600
O -0.55961700 1.38708900 0.56692200
C -2.58767900 2.40056400 -0.12289600
C -2.51516300 4.24427000 -1.75063600
H -0.52765200 4.89797500 -2.28787000
C 3.69444000 5.79917500 0.41825200
C 1.52837800 6.82290600 0.68329000
H -0.17638500 5.66158500 0.14330200
C 4.85482400 -0.72143200 -2.52006200
F 3.34965400 0.85755600 -3.34385100
C 5.67596800 -0.55512200 -0.26599100
F 4.96085200 1.19331300 1.11140900
C -3.28006100 3.36522100 -0.91869700
C -3.32520900 1.42425100 0.71461100
C -3.18532600 5.17714800 -2.58301200
C 2.93837900 6.88708800 0.78461100
H 4.77774600 5.82100100 0.50737000
H 0.92876500 7.67268800 0.99731300
C 5.64857100 -1.21816200 -1.49030500
F 4.79988500 -1.36533800 -3.69134400
F 6.43865200 -1.02472100 0.73074300
C -4.69448500 3.48802900 -0.93745300
C -3.15651000 1.39107000 2.10453700
C -4.23238900 0.51186100 0.16773200
C -4.55920400 5.25635600 -2.59343300
H -2.58761200 5.83338300 -3.21066200
H 3.41546200 7.78654000 1.16242100
F 6.36806200 -2.32783700 -1.67018100
C -5.31640900 4.40677000 -1.75375400
H -5.29059200 2.85455900 -0.29034500
C -3.86818200 0.50741900 2.90818100
F -2.30294900 2.23035500 2.70134500
C -4.95704800 -0.37876200 0.95281800

F -4.43081100 0.47209400 -1.15792500
H -5.06297400 5.97322400 -3.23505500
H -6.40016400 4.48137600 -1.75048000
C -4.78079200 -0.36894900 2.33077400
F -3.67744600 0.47796400 4.23731300
F -5.79709500 -1.25719600 0.39358700
F -5.43294000 -1.25458300 3.09416600
C -2.07399700 -1.99016300 -1.21838300
C -2.70582000 -2.96976400 -0.40472200
C -2.55445300 -1.68199800 -2.51884200
C -3.89530400 -3.53155400 -0.86504400
C -2.05378200 -3.41193100 0.89427500
C -3.74746400 -2.28025800 -2.92648500
C -1.78166000 -0.74887900 -3.43794900
C -4.42804100 -3.17351100 -2.10264600
H -4.40928200 -4.26718900 -0.25578800
C -2.40646300 -4.85128400 1.29185600
C -2.34078300 -2.46872400 2.06926500
H -0.97665600 -3.37058900 0.72285500
H -4.15068300 -2.04914800 -3.90657700
C -2.12600900 0.73068400 -3.20199900
C -1.96197800 -1.08804200 -4.92509900
H -0.71976800 -0.88084000 -3.19861300
H -5.36315700 -3.61359600 -2.43714200
H -1.75077000 -5.17600000 2.10765000
H -2.28671800 -5.54552700 0.45477100
H -3.43926400 -4.93063900 1.65112500
H -1.75524000 -2.78813600 2.93739100
H -3.39806700 -2.50295500 2.34716100
H -2.06246900 -1.43981100 1.83222100
H -1.48466700 1.37372800 -3.81468300
H -1.98939400 1.01716200 -2.16081500
H -3.17082400 0.93290600 -3.45836000
H -1.82031300 -2.15499900 -5.12082700
H -1.23705600 -0.52517300 -5.52368800
H -2.96011400 -0.80930400 -5.28185100
H 1.89138800 -5.43892000 1.19024500

4b-TS1:

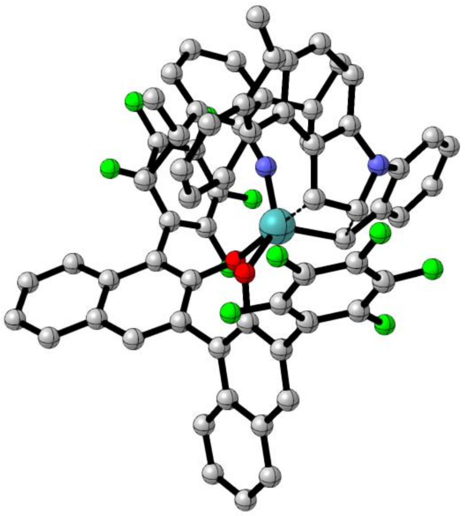

G_sol = –3905.689731

C 2.31617100 -0.65253900 0.48283000
H 2.87555200 0.16967400 0.07098600
C 2.42152100 -2.01412400 -0.03439900
C 2.11700300 -2.90360100 1.02214600
C 2.71824800 -2.49316800 -1.32107500
N 2.06453300 -2.17027000 2.23229900
C 1.91626400 -4.25925800 0.80302200
C 2.50453000 -3.87019600 -1.53437300
C 1.21272200 -2.42426500 3.33424500
C 2.15933900 -0.78747300 1.92009800
C 2.08798400 -4.72047900 -0.50720200
H 2.66371400 -4.27912900 -2.52489700
C 0.34446200 -1.34714600 3.62083000
C 1.21562700 -3.57823300 4.10769600
C 0.45652500 -0.19813900 2.70290800
H 2.72118400 -0.16971300 2.61110300
H 1.91931700 -5.77053900 -0.72561600
C -0.51932900 -1.44174600 4.71124100
C 0.32466300 -3.66650500 5.18305500
H 0.65738200 0.77622800 3.16929700
C -0.53164400 -2.60639000 5.48540400
H 0.31789800 -4.56144500 5.79765100
H -1.21143200 -2.68225500 6.32833700
Mo 0.10973400 0.05232300 0.77240000
N -0.53637900 -1.49098300 0.26786100
O 0.90033700 1.67141500 -0.20562600
C 0.82929700 2.90831500 0.32733900
C -0.33126700 3.67643300 0.29517500
C 2.02676400 3.44645100 0.90212600
C -1.51080600 3.21235900 -0.47259200
C -0.36381300 4.93892200 0.97785100
C 2.01687800 4.67590300 1.51634300
C 3.28092800 2.66735000 0.76484800

C -2.13133700 1.95171400 -0.19879300
C -2.05641200 4.02060700 -1.44753600
C 0.82835000 5.44030000 1.59772400
C -1.55071100 5.71406000 1.10991000
H 2.93425400 5.07619100 1.93973600
C 3.89923900 2.51231400 -0.47994000
C 3.86707300 2.01463800 1.84512200
O -1.57483600 1.10967400 0.67854600
C -3.33820800 1.60819500 -0.81331000
C -3.25219900 3.68726800 -2.12732200
H -1.57031500 4.96340000 -1.68304800
C 0.80261200 6.68930900 2.27071200
C -1.54690800 6.91634300 1.78162600
H -2.47440000 5.33759800 0.68759300
C 5.03654900 1.72794300 -0.64664700
F 3.37908700 3.11318700 -1.55754000
C 4.99500200 1.21158800 1.70713900
F 3.27392800 2.05682700 3.05765500
C -3.93651700 2.48047500 -1.77473300
C -3.99874800 0.33895400 -0.42807300
C -3.80729000 4.54792900 -3.10927400
C -0.35853600 7.42084700 2.35962000
H 1.72252900 7.05259600 2.72221400
H -2.47068900 7.48050800 1.87437300
C 5.58280600 1.06859400 0.45432900
F 5.61006400 1.60098900 -1.84483300
F 5.47440700 0.53575200 2.75946000
C -5.18630100 2.21821000 -2.39808900
C -4.36772900 0.10144600 0.90116300
C -4.32406700 -0.65984900 -1.35243000
C -5.00658800 4.24970700 -3.71386100
H -3.26656700 5.45521100 -3.36680400
H -0.36947900 8.37396800 2.88006800
F 6.65652600 0.29072800 0.30269900
C -5.70451100 3.07702000 -3.34167500
H -5.74748600 1.33548000 -2.11503300
C -5.02128600 -1.06093100 1.29100300
F -4.10931200 1.01218400 1.84896000
C -4.98661200 -1.82711300 -0.98663100
F -4.01999200 -0.50930700 -2.65125000
H -5.42471500 4.91481300 -4.46366500
H -6.66245600 2.85283000 -3.80238600
C -5.31652500 -2.03744200 0.34628400
F -5.34041100 -1.26200800 2.57694200
F -5.30208100 -2.74675500 -1.90708500
F -5.89616100 -3.18273500 0.72630300
C -1.18767100 -2.46458800 -0.45973100
C -1.85850700 -3.49614400 0.25134300
C -1.22980400 -2.40226600 -1.88080000
C -2.67718900 -4.36486700 -0.46976900
C -1.66366700 -3.66331500 1.74612800
C -2.07067100 -3.29365800 -2.54626000
C -0.33505000 -1.44438100 -2.64277000
C -2.80600000 -4.25201800 -1.85216900
H -3.22007200 -5.14336300 0.05597000
C -1.53609800 -5.13756000 2.15696600
C -2.77066000 -2.96615400 2.54802200
H -0.72342600 -3.17381300 1.99913700
H -2.14186600 -3.24731200 -3.62727600
C -0.98937000 -0.08843400 -2.94524100
C 0.22224500 -2.06417200 -3.93215600
H 0.51362800 -1.25535900 -1.98592300
H -3.46557300 -4.92424200 -2.39197900
H -1.23180300 -5.19984700 3.20667700
H -0.78829800 -5.65665600 1.54799200
H -2.48583800 -5.67503900 2.05696000
H -2.62531300 -3.13060100 3.61962600
H -3.75705900 -3.35240500 2.27314500
H -2.75386300 -1.88861800 2.36729100
H -0.24586100 0.59287500 -3.37392800
H -1.39393600 0.38022500 -2.04794700
H -1.81171700 -0.19828000 -3.65726600
H 0.68349900 -3.03750000 -3.73909800
H 0.98236100 -1.40392600 -4.35925700
H -0.56125100 -2.19763800 -4.68661100
H 1.91473800 -4.37917500 3.89413400
H -1.19538800 -0.62274900 4.93698400
C 3.25069400 -1.59754600 -2.38250200
C 4.25966000 -2.04079900 -3.27918400
C 2.76870700 -0.28187100 -2.52196600
C 4.69491500 -1.17352800 -4.29571300
C 3.21152600 0.55864700 -3.53803600
H 2.00714100 0.09600500 -1.85431500
C 4.17269000 0.10612400 -4.44139400
H 5.49240600 -1.50771700 -4.95179100
H 2.81589800 1.56644700 -3.60481200
H 4.53625100 0.75574500 -5.23222000
C 4.92630700 -3.34943000 -3.13195700
C 5.39393300 -4.09717200 -4.13890000
H 5.04656700 -3.71166600 -2.11327600
H 5.91629100 -5.03026700 -3.95060400
H 5.26072700 -3.81332300 -5.17964800
H 1.63784100 -4.93325100 1.60457400

4b-Metallacycle:

G_sol = –3905.694549

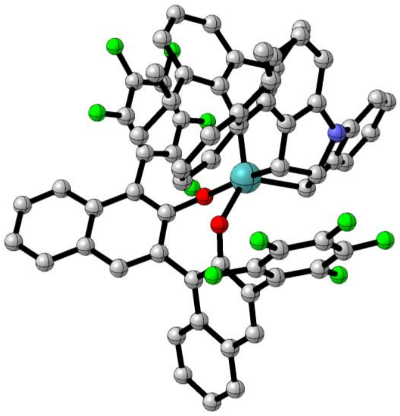

C 2.18773600 -0.61939700 0.41676100
H 2.79506300 0.19894900 0.05993300
C 2.47243400 -1.97504900 -0.05300000
C 2.22897200 -2.89443200 0.99624000
C 2.88035600 -2.41776300 -1.32221900
N 2.07578000 -2.19397900 2.20564900
C 2.18404900 -4.26364900 0.76569300
C 2.82180000 -3.80938700 -1.54659900
C 1.22233100 -2.48214500 3.28787300
C 2.07320200 -0.75917500 1.94582000
C 2.45340600 -4.70012200 -0.53727200
H 3.06228900 -4.19412500 -2.53034600
C 0.44281800 -1.35157400 3.61522900
C 1.13424500 -3.65975000 4.02068000
C 0.73779000 -0.23334100 2.68663800
H 2.89561700 -0.24336700 2.44445300
H 2.40593800 -5.76184700 -0.75999600
C -0.43526400 -1.40126200 4.69072400
C 0.23645100 -3.70008100 5.09500400
H 0.88411500 0.75120900 3.14352300
C -0.53897600 -2.58748800 5.42847300
H 0.15610400 -4.60889100 5.68386300
H -1.22606100 -2.63961100 6.26726400
Mo 0.10524200 0.02672300 0.74281900
N -0.55366100 -1.54100200 0.32670900
O 0.86981400 1.68557000 -0.21474700
C 0.77653100 2.89877700 0.36144700
C -0.39676400 3.64885700 0.35711800
C 1.96665800 3.44124500 0.95085000
C -1.56098100 3.19640000 -0.43897000
C -0.45307700 4.88460700 1.08571100
C 1.93377300 4.64655200 1.60988700
C 3.23295700 2.68848100 0.78410900
C -2.15710900 1.91148300 -0.23713100
C -2.11863200 4.03902000 -1.37784200
C 0.73058100 5.38418400 1.72292900
C -1.65370000 5.63210800 1.24785900
H 2.84423000 5.04926000 2.04571100
C 3.83272600 2.55356000 -0.47239600
C 3.84390400 2.03229700 1.84863400
O -1.59064600 1.02778400 0.59412500
C -3.35491400 1.57350100 -0.87161900
C -3.29985100 3.71201100 -2.08540500
H -1.65268300 5.00288700 -1.56242900
C 0.68264200 6.60567600 2.44313300
C -1.67120400 6.80797100 1.96495000
H -2.57137300 5.25512600 0.81284500
C 4.97410300 1.78155300 -0.66563100
F 3.29028900 3.16075200 -1.53466400
C 4.97459300 1.23826900 1.68194200
F 3.27503200 2.06275900 3.07170100
C -3.96241500 2.47589200 -1.79776000
C -4.01153300 0.29062000 -0.52493400
C -3.86404900 4.60577800 -3.03197800
C -0.49172600 7.31189900 2.56093200
H 1.59633500 6.96836300 2.90753400
H -2.60519700 7.35085100 2.07987300
C 5.54074000 1.11066800 0.41780000
F 5.53329600 1.67522700 -1.87235800
F 5.47387500 0.55127000 2.71853300
C -5.20006800 2.21576500 -2.44598900
C -4.40945700 0.03291300 0.79184400
C -4.31976400 -0.69005200 -1.47301000
C -5.05061700 4.31021100 -3.66239500
H -3.34104200 5.53543100 -3.24134800
H -0.51946800 8.24399400 3.11752600
F 6.61636300 0.34105400 0.23933100
C -5.72708300 3.10664900 -3.35399400
H -5.74374900 1.30829200 -2.21173300
C -5.08186000 -1.12960000 1.14753000
F -4.16429400 0.92677300 1.75927100
C -4.99033600 -1.86238600 -1.13985800
F -3.99318500 -0.51338700 -2.76292700
H -5.47560600 5.00079300 -4.38477900
H -6.67472600 2.88420700 -3.83613200
C -5.35080800 -2.09347500 0.18199500
F -5.44862000 -1.33973100 2.41861200
F -5.28495900 -2.76685800 -2.08174600
F -5.94284200 -3.24176900 0.52981500
C -1.20667200 -2.52176200 -0.38939600
C -1.91661700 -3.51755200 0.33465300
C -1.21850200 -2.49885900 -1.81317300
C -2.74417200 -4.38394200 -0.38011000
C -1.75024500 -3.65841300 1.83582400
C -2.06546400 -3.38863000 -2.47202400
C -0.29096500 -1.57902200 -2.58224700
C -2.83972100 -4.30855300 -1.76734400
H -3.31946700 -5.13206800 0.15507800

C -1.59738500 -5.12743500 2.25800400
C -2.88842300 -2.97682000 2.60615000
H -0.82726700 -3.14561000 2.10534800
H -2.11203300 -3.37213300 -3.55525700
C -0.92669600 -0.22698900 -2.94012800
C 0.28590900 -2.24619900 -3.83891900
H 0.54656300 -1.38249600 -1.91317800
H -3.50416900 -4.97951400 -2.30254700
H -1.35475800 -5.17966500 3.32333200
H -0.79415000 -5.61774300 1.69690100
H -2.51903900 -5.69857100 2.10001100
H -2.76118400 -3.12816600 3.68232900
H -3.86286600 -3.38163800 2.31448600
H -2.88781500 -1.90034200 2.41741700
H -0.17276100 0.43161100 -3.38521900
H -1.33759300 0.27821600 -2.06467000
H -1.74168600 -0.35574300 -3.65722500
H 0.73406800 -3.21609700 -3.60206400
H 1.06097500 -1.60697100 -4.27105700
H -0.48219300 -2.39815400 -4.60553400
H 1.75972200 -4.51336900 3.78601200
H -1.04378100 -0.53754100 4.94181000
C 3.35524800 -1.47184600 -2.36741200
C 4.44116100 -1.80761000 -3.22066200
C 2.73918700 -0.21760900 -2.53901500
C 4.81386700 -0.90706700 -4.23286400
C 3.12068200 0.65603800 -3.55205500
H 1.92471600 0.08287300 -1.89600900
C 4.15579000 0.30319000 -4.41696500
H 5.66839600 -1.15518600 -4.85440800
H 2.61897300 1.61346200 -3.64532300
H 4.47270100 0.98075300 -5.20442100
C 5.24515700 -3.03048800 -3.02902800
C 5.84065800 -3.72686700 -4.00486700
H 5.35953400 -3.36960200 -2.00178400
H 6.45688700 -4.59255600 -3.78171300
H 5.72401500 -3.46786800 -5.05404500
H 1.95707800 -4.96882900 1.55660600

4b-TS2:

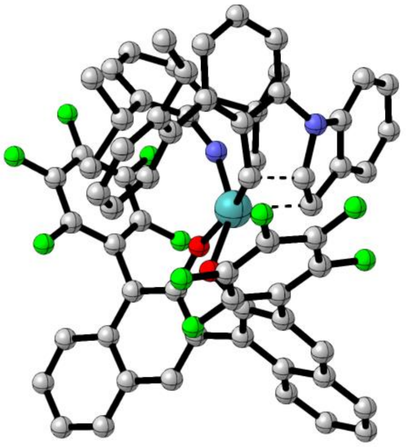

G_sol = –3905.688699

C 2.12932500 -0.36052900 0.43873100
H 2.61894600 0.38843100 -0.16785000
C 2.66814500 -1.72384100 0.40275000
C 2.57342600 -2.31446400 1.68477500
C 3.23116400 -2.42767800 -0.67311600
N 2.23024800 -1.33158800 2.62745300
C 2.85289200 -3.66138100 1.88426500
C 3.51629600 -3.79150400 -0.45986600
C 1.39733500 -1.41430800 3.75398900
C 2.04920000 -0.04307700 1.96195800
C 3.30078100 -4.39537100 0.77882900
H 3.90081400 -4.38281700 -1.28254300
C 0.53630900 -0.29611500 3.80731200
C 1.37908400 -2.38426700 4.75193700
C 0.70503200 0.54018700 2.59721200
H 2.82781600 0.67694900 2.21889900
H 3.51854800 -5.45236600 0.89925700
C -0.31284800 -0.11875000 4.89297000
C 0.49994700 -2.20593500 5.82661300
H 0.73607400 1.62295000 2.74699800
C -0.32916500 -1.08417500 5.90649100
H 0.47713500 -2.94875000 6.61823100
H -0.99498500 -0.96311600 6.75518100
Mo 0.01651800 0.12124400 0.68725800
N -0.50641600 -1.53984100 0.87129300
O 0.55122800 1.64290200 -0.65286800
C 0.49045800 2.79498100 0.03468700
C -0.73305000 3.41756600 0.30786500
C 1.71510900 3.40180100 0.49276700
C -1.92929100 3.00872600 -0.46568000
C -0.78431200 4.51150200 1.23196200
C 1.66995800 4.47930800 1.34870400
C 3.00271100 2.87275600 -0.01153700
C -2.39587700 1.65535000 -0.50900300

C -2.59576200 3.96011900 -1.21319100
C 0.43657700 5.03052400 1.77671400
C -2.00804800 5.08490600 1.67585700
H 2.59467500 4.93059900 1.69658000
C 3.23451300 2.71995900 -1.38499200
C 4.03416400 2.47388300 0.84100800
O -1.82293400 0.70201300 0.23434200
C -3.51865200 1.31930000 -1.26680800
C -3.72447900 3.65119300 -2.00771200
H -2.23575500 4.98506800 -1.20019200
C 0.39080700 6.10289500 2.70425500
C -2.01949200 6.11754400 2.58799500
H -2.94146700 4.68401500 1.29809600
C 4.40851200 2.15886300 -1.87686000
F 2.31878900 3.12148600 -2.27114600
C 5.19865400 1.87070600 0.37783400
F 3.87897900 2.57927900 2.17966700
C -4.20573400 2.30360400 -2.03224800
C -3.99313700 -0.08638300 -1.24104800
C -4.39082100 4.64347800 -2.77272900
C -0.81102100 6.64151900 3.10358500
H 1.32822700 6.48815000 3.09761800
H -2.96824900 6.53017000 2.91918400
C 5.38553500 1.70858800 -0.99225500
F 4.61543900 2.07149900 -3.19282400
F 6.12781600 1.43943900 1.23914900
C -5.35450200 2.01204600 -2.81638500
C -4.56836700 -0.63693900 -0.09351600
C -3.87577100 -0.91934100 -2.35318100
C -5.49558800 4.32729900 -3.52938300
H -4.00957000 5.66118900 -2.74657300
H -0.83539400 7.45956400 3.81737300
F 6.50034000 1.14127200 -1.45586700
C -5.98115300 2.99758700 -3.54511700
H -5.74189700 0.99906800 -2.83034800
C -4.97130600 -1.96716400 -0.03783900
F -4.75003800 0.12936300 0.99056600
C -4.26262300 -2.25494600 -2.31963900
F -3.34038800 -0.44450900 -3.48796400
H -5.99892000 5.09344900 -4.11168000
H -6.85776300 2.75341000 -4.13841500
C -4.81857500 -2.77851400 -1.15821000
F -5.51861600 -2.47581000 1.07587300
F -4.07253100 -3.04491200 -3.38654700
F -5.20852900 -4.05649200 -1.11851700
C -1.15180400 -2.73035500 0.62801800
C -1.99088000 -3.25337200 1.65492700
C -1.03335300 -3.37855200 -0.63262700
C -2.75619100 -4.38048100 1.35866000
C -2.04374000 -2.59018000 3.01924000
C -1.82628400 -4.50131800 -0.86871500
C -0.07740800 -2.85936800 -1.68697300
C -2.68676300 -4.99466400 0.10917400
H -3.41642400 -4.78907800 2.11478000
C -2.40721800 -3.56230900 4.14892100
C -3.00115100 -1.38608300 3.01960900
H -1.04133400 -2.21045100 3.22501400
H -1.76821500 -4.99971200 -1.83025500
C -0.77459200 -1.86500400 -2.62861800
C 0.60138100 -3.97729800 -2.48920500
H 0.70926400 -2.32631000 -1.15348400
H -3.30322100 -5.86291100 -0.10113000
H -2.28811200 -3.05824000 5.11219000
H -1.75969800 -4.44607700 4.14594400
H -3.44831200 -3.89865600 4.08034400
H -2.98279900 -0.89708000 3.99988600
H -4.02590500 -1.70816500 2.81630600
H -2.72005900 -0.64995100 2.26453900
H -0.06838900 -1.48329000 -3.37290700
H -1.18300200 -1.01135000 -2.07970700
H -1.59602400 -2.35071000 -3.16292000
H 1.05513200 -4.72110400 -1.82731500
H 1.39156800 -3.54797200 -3.11315100
H -0.10332700 -4.48807400 -3.15521900
H 2.04992600 -3.23449900 4.72394100
H -0.97231300 0.74288600 4.93572200
C 3.50522700 -1.76416400 -1.97373600
C 4.70428500 -2.01261500 -2.69193600
C 2.56923000 -0.86851600 -2.52053400
C 4.88384700 -1.39487700 -3.94155800
C 2.76189000 -0.27549300 -3.76359800
H 1.66420300 -0.64121000 -1.97534400
C 3.92218000 -0.55260200 -4.48689100
H 5.82022100 -1.55352000 -4.46696900
H 2.01721200 0.41431800 -4.14881300
H 4.09596000 -0.08128800 -5.44959000
C 5.79427500 -2.83340300 -2.13107100
C 6.65145800 -3.57490400 -2.84319300
H 5.89564400 -2.81441600 -1.04826400
H 7.45712500 -4.12021200 -2.36111400
H 6.57274200 -3.67399500 -3.92273000
H 2.75710900 -4.12768600 2.85721000

4b-Alk2:

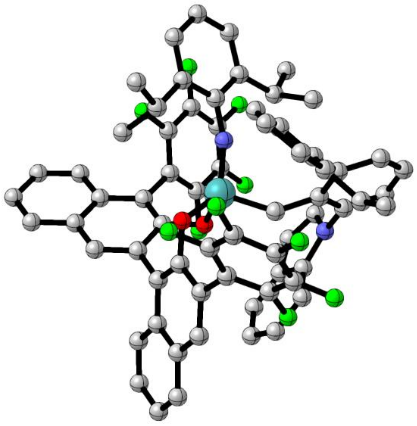

G_sol = –3905.706586

C 1.22913100 -0.56885200 1.06249300
H 1.75852500 0.38891500 1.20225100
C 1.65015700 -1.66374300 1.93793000
C 1.19579200 -1.67501400 3.27734400
C 2.49145000 -2.70731900 1.49317300
N 0.30296300 -0.66619900 3.72322500
C 1.55104200 -2.70658400 4.14475200
C 2.84737100 -3.72754000 2.38625600
C 0.48719200 0.70825200 3.59470500
C -1.05074500 -0.89979500 3.98234500
C 2.37590400 -3.73675800 3.69661000
H 3.49455900 -4.52402500 2.03287600
C -0.77507600 1.33333400 3.77829900
C 1.65193600 1.43918200 3.33743300
C -1.72655000 0.28624200 4.04191300
H -1.39954300 -1.91737900 4.08545200
H 2.65620400 -4.54067200 4.37035000
C -0.86618700 2.72816500 3.64519200
C 1.52706100 2.81728700 3.19985300
H -2.78008300 0.40532100 4.23972800
C 0.27846700 3.45546100 3.34256200
H 2.40705000 3.40806700 2.96854900
H 0.21625700 4.53069200 3.20754300
Mo -0.00331900 -0.14859600 -0.28781500
N -0.87266400 -1.63017300 -0.61189600
O 0.88316500 1.08062100 -1.54685800
C 1.16613800 2.29889300 -0.99282600
C 0.12534400 3.18727900 -0.72741800
C 2.51392600 2.62663900 -0.65346100
C -1.27429700 2.82129700 -1.06793900
C 0.39702800 4.43492900 -0.08751900
C 2.77567100 3.84832100 -0.06570300
C 3.62997800 1.68035000 -0.87602000
C -1.94727100 1.77911300 -0.34676400
C -1.95380400 3.48472000 -2.06494600
C 1.74922700 4.76852300 0.23998800
C -0.62849200 5.34722300 0.28304500
H 3.79740400 4.10519900 0.19556700
C 3.89767700 1.09858200 -2.12032200
C 4.54346900 1.41056500 0.15187700
O -1.25878600 1.09398800 0.60639200
C -3.27094600 1.45991600 -0.62992000
C -3.29877200 3.17988100 -2.39047200
H -1.44454400 4.26702500 -2.62138200
C 2.02895700 5.99512700 0.89762800
C -0.32673500 6.52531300 0.92854300
H -1.65985100 5.09582200 0.06598000
C 5.02564500 0.31266600 -2.33494800
F 3.08745500 1.32343600 -3.16139000
C 5.69467400 0.65850200 -0.04930300
F 4.32754300 1.88525500 1.39404100
C -3.98235500 2.16306200 -1.64920800
C -3.92017000 0.37879300 0.14589400
C -3.98376500 3.86831600 -3.42482800
C 1.01407800 6.85914700 1.23757600
H 3.06242400 6.23185000 1.13745200
H -1.12681700 7.20374200 1.21054700
C 5.94202600 0.11890000 -1.30662500
F 5.24768400 -0.24181600 -3.53262000
F 6.55388300 0.45697000 0.95565800
C -5.34077700 1.89694700 -1.96836200
C -4.12639100 0.50959100 1.52142200
C -4.37534000 -0.79470000 -0.45933200
C -5.29558400 3.57612700 -3.71892700
H -3.44860800 4.63448600 -3.97984000
H 1.23596300 7.79204300 1.74719000
F 7.03996200 -0.61382200 -1.52058300
C -5.97865800 2.58402400 -2.97644000
H -5.88245000 1.14962800 -1.39950000
C -4.75980400 -0.48101500 2.26409600
F -3.74362900 1.62597300 2.15139800
C -5.03670000 -1.78544500 0.25749100
F -4.19190200 -0.98551100 -1.77414600
H -5.81091100 4.10772300 -4.51329900
H -7.01800300 2.36431800 -3.20286800
C -5.22410900 -1.62849300 1.62760100
F -4.95252900 -0.33087000 3.58287500
F -5.47481600 -2.89378800 -0.35440200
F -5.84630000 -2.57888500 2.33266300
C -1.50906500 -2.64824800 -1.28677300
C -2.19440600 -3.64733500 -0.54524200
C -1.46578400 -2.66378600 -2.70812900
C -2.85477600 -4.64590900 -1.26238900
C -2.16144600 -3.63613500 0.97574500
C -2.14542900 -3.68708700 -3.36817700
C -0.72813500 -1.56966100 -3.46187500
C -2.83732100 -4.66584200 -2.65592400

H -3.39679000 -5.41745600 -0.72748500
C -0.80478300 -4.15472200 1.48490500
C -3.28638900 -4.45152700 1.62829600
H -2.25375700 -2.59011400 1.29502300
H -2.13817400 -3.72265300 -4.45225100
C -1.58459400 -0.29253500 -3.57556900
C -0.23628900 -1.99677200 -4.85041700
H 0.16737800 -1.31928600 -2.87653200
H -3.36430000 -5.45098700 -3.19061100
H -0.76785800 -4.13439500 2.57979600
H 0.01949000 -3.54663100 1.11870300
H -0.64478400 -5.18893400 1.15783600
H -3.31132600 -4.24982000 2.70460700
H -3.11511900 -5.52760000 1.50516600
H -4.27047600 -4.22120600 1.21848600
H -0.99467700 0.52582100 -4.00061100
H -1.96698500 0.03533400 -2.60745000
H -2.45222100 -0.47479700 -4.21958300
H 0.33205700 -2.93247600 -4.81351300
H 0.41281300 -1.21883100 -5.26575100
H -1.06901100 -2.13590800 -5.54935700
H 2.61616000 0.95146800 3.23321500
H -1.82578200 3.22465400 3.75675000
C 2.97404100 -2.77962000 0.08291400
C 4.34985800 -2.68762900 -0.22405500
C 2.05640800 -3.02346800 -0.94929200
C 4.74976300 -2.81435700 -1.56705600
C 2.47081400 -3.14559000 -2.27311700
H 1.01120600 -3.14842400 -0.70607100
C 3.82699700 -3.03236000 -2.58360800
H 5.79775900 -2.69432300 -1.81651700
H 1.73777300 -3.33849900 -3.04948500
H 4.16488400 -3.10412400 -3.61295200
C 5.33666100 -2.42139900 0.83606700
C 6.63724700 -2.73304200 0.78925900
H 4.95330800 -1.92652700 1.72515100
H 7.30511700 -2.46543000 1.60155300
H 7.07906900 -3.26212100 -0.05038700
H 1.18165600 -2.68353500 5.16508900

1:

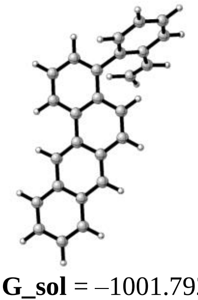

G_sol = –1001.792236

C -1.05764200 3.07482100 -0.05322000
C -2.22509900 2.31436800 -0.14710800
C -2.16157300 0.93530400 -0.22045100
C -0.90078400 0.28921500 -0.23432300
C 0.28629900 1.05918500 -0.13478100
C 0.17303200 2.45629300 -0.03903800
C -0.80732100 -1.13991400 -0.34587200
C 0.38496300 -1.77107100 -0.34951800
C 1.61710500 -1.04741100 -0.24094300
C 1.58062000 0.38367800 -0.13474500
C 2.83581400 -1.71115900 -0.24056100
C 4.05086300 -1.02669200 -0.13773800
C 4.02177800 0.40089700 -0.03165200
C 2.78621600 1.06212100 -0.03427300
C 5.30753200 -1.68907900 -0.13454300
C 6.47115300 -0.98090300 -0.03206000
C 6.44351300 0.43221100 0.07308600
C 5.25279400 1.10207000 0.07315600
C -3.42601000 0.15612800 -0.31213100
C -4.11384000 0.18715000 -1.52447400
C -5.29091800 -0.52386400 -1.71616500
C -5.79642200 -1.29441300 -0.67839200
C -5.12497600 -1.33139500 0.53271200
C -3.94564500 -0.60649400 0.75551600
C -3.36166900 -0.70823900 2.10058100
C -2.46377500 0.06891000 2.70592700
H -1.12112500 4.15308700 0.01458900
H -3.19240300 2.79956700 -0.13986300
H 1.06023900 3.06659200 0.04160600
H -1.72196600 -1.70834100 -0.43041700
H 0.43310200 -2.84970400 -0.43603200
H 2.84477100 -2.79207500 -0.32209100
H 2.80142900 2.13990200 0.04712100
H 5.32542700 -2.76917900 -0.21501100
H 7.42285800 -1.49638900 -0.03062100
H 7.37435000 0.97855000 0.15348600
H 5.22989000 2.18220100 0.15339600
H -3.69980100 0.77313700 -2.33510100
H -5.79965500 -0.48274800 -2.67041100
H -6.70734400 -1.86376500 -0.81002600
H -5.52363400 -1.92735100 1.34468500
H -3.76889900 -1.53333600 2.67825500
H -2.00888000 0.92859600 2.23974600
H -2.16441300 -0.14904400 3.72274300

2:

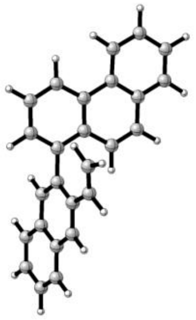

G_sol = –1001.796143

C 1.40491700 3.04743600 -0.51710100
C 2.51530000 2.23689200 -0.52160600
C 2.40272100 0.84564800 -0.33255700
C 1.11042000 0.29407900 -0.12170700
C -0.02357600 1.14826300 -0.09151200
C 0.13344400 2.50207000 -0.30485500
C 0.97222000 -1.11793600 0.05683900
C -1.05801500 0.20223000 2.66595500
C -1.83295900 0.13294300 1.42074500
C -1.38717000 0.59923300 0.13884100
C -3.09896700 -0.41338700 1.51100000
C -3.97967700 -0.50005200 0.41437100
C -3.54129400 -0.00669200 -0.84623000
C -2.23643100 0.52798500 -0.93760600
C -5.27861800 -1.05200500 0.52056200
C -6.10344800 -1.11193100 -0.57234400
C -5.66711600 -0.62327500 -1.82238400
C -4.41363200 -0.08341000 -1.95560200
C 3.55721200 -0.03454500 -0.34888000
C 4.87262400 0.43073200 -0.54498900
C 5.94581600 -0.43146000 -0.55218100
C 5.75183800 -1.80714900 -0.36402600
C 4.48051800 -2.29195000 -0.17166200
C 3.36758900 -1.42826900 -0.15923000
C 2.04848900 -1.93765600 0.03836400
C 0.03308800 0.90893500 2.96225400
H 1.51314600 4.11316300 -0.67159000
H 3.48544500 2.68369500 -0.67988700
H -0.73639100 3.14543100 -0.28223500
H -0.01709700 -1.52553000 0.20572800
H -1.48580900 -0.40012500 3.46240000
H -3.44227800 -0.77717500 2.47272600
H -1.88569400 0.87589200 -1.90182700
H -5.61106400 -1.42557800 1.48148400
H -7.09532700 -1.53535300 -0.48108700
H -6.32831900 -0.67750700 -2.67752500
H -4.07510200 0.29090100 -2.91406100
H 5.05592900 1.48477300 -0.69290800
H 6.94469000 -0.04340500 -0.70424400
H 6.59894400 -2.48062300 -0.37095000
H 4.31449300 -3.35239800 -0.02554300
H 1.92521800 -3.00521700 0.17414700
H 0.53307100 1.55807200 2.26133000
H 0.45438500 0.85060600 3.95729400

3:

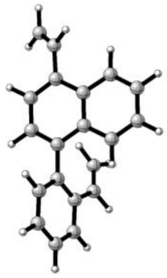

G_sol = –771.85614

C 1.73945700 -1.95109800 -0.16506000
C 0.34195100 -1.82588300 -0.18498200
C -0.26488900 -0.59490200 -0.25599900
C 0.54652500 0.57417000 -0.33819100
C 1.97024100 0.45036000 -0.30026500
C 2.56507700 -0.84819700 -0.20675800
C -0.02476300 1.86436600 -0.44866400
C 0.75932600 2.98565200 -0.50130700
C 2.16054800 2.86915900 -0.43775100
C 2.74688400 1.63448400 -0.33813800
C 4.02518600 -1.01262800 -0.18678800
C 4.68465700 -1.94087900 0.50352100
C -1.74849100 -0.50487200 -0.28765100
C -2.38976000 -0.89466400 -1.46303500
C -3.76942600 -0.82503400 -1.60206200
C -4.53557200 -0.34689500 -0.54825900
C -3.91214100 0.04007000 0.62669100
C -2.52271100 -0.03985700 0.79720300
C -2.00397300 0.36196800 2.11226300
C -0.82142800 0.11922200 2.67694000
H 2.17917500 -2.93924100 -0.13929600
H -0.27236100 -2.71582400 -0.13602600
H -1.10093000 1.95337200 -0.48768800
H 0.30275800 3.96331200 -0.58568500
H 2.77683400 3.75858700 -0.46588000
H 3.82305000 1.56436300 -0.27520700
H 4.59946700 -0.32019600 -0.79260100
H 4.17497700 -2.63767600 1.15763900
H 5.76164000 -2.02353300 0.44341100
H -1.78177800 -1.24442700 -2.28756500
H -4.23686000 -1.13151900 -2.52871000
H -5.61161000 -0.27526600 -0.63928800
H -4.51125900 0.40521900 1.45198700

H -2.73048900 0.91057300 2.70515700
H -0.03280700 -0.43438400 2.19220500
H -0.61998800 0.47939100 3.67729200

4:

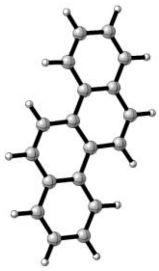

G_sol = – –693.301514

C -3.66822200 -1.47954300 0.00000000
C -2.45599100 -0.75875500 0.00000000
C -1.21802500 -1.45676300 0.00000000
C -1.26162300 -2.86820600 -0.00000100
C -2.45599100 -3.54926300 -0.00000100
C -3.67453900 -2.85183600 -0.00000100
C -2.44777600 0.66249800 0.00000100
C -1.28340900 1.35999800 0.00000100
C -0.01740400 0.70512700 0.00000000
C 0.01740400 -0.70512700 0.00000000
C 1.21802500 1.45676300 0.00000000
C 2.45599100 0.75875500 0.00000000
C 2.44777600 -0.66249800 0.00000100
C 1.28340900 -1.35999800 0.00000100
C 1.26162300 2.86820600 -0.00000100
C 2.45599100 3.54926300 -0.00000100
C 3.67453900 2.85183600 -0.00000100
C 3.66822200 1.47954300 0.00000000
H -4.59930800 -0.92581600 0.00000000
H -0.34448800 -3.43776200 -0.00000200
H -2.45551000 -4.63159300 -0.00000200
H -4.61038700 -3.39526800 -0.00000100
H -3.39639900 1.18533900 0.00000200
H -1.32535400 2.43848400 0.00000200
H 3.39639900 -1.18533900 0.00000200
H 1.32535400 -2.43848400 0.00000200
H 0.34448800 3.43776200 -0.00000200
H 2.45551000 4.63159300 -0.00000200
H 4.61038700 3.39526800 -0.00000100
H 4.59930800 0.92581600 0.00000000

13a:

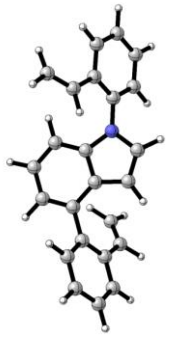

G_sol = – –980.876099

C 0.16582600 1.58281300 2.09579400
C 1.47570000 1.39899300 1.62620600
C 1.75212700 0.59648800 0.52788700
C 0.66839400 -0.00925800 -0.13133500
C -0.64674100 0.20425600 0.34920600
C -0.91454100 0.98547100 1.47040100
C 0.56508100 -0.88035300 -1.26359000
C -0.75775700 -1.16466300 -1.43220800
N -1.51042200 -0.50472900 -0.47097200
C -2.91401000 -0.62403000 -0.31235000
C 3.14128700 0.41336600 0.03934300
C 3.79288300 1.52879600 -0.48824900
C 5.07095700 1.44550900 -1.02333000
C 5.71972700 0.21854000 -1.04902700
C 5.08881700 -0.89624600 -0.52150300
C 3.81028700 -0.82975300 0.04945300
C 3.29736500 -2.07106400 0.64772000
C 2.36413700 -2.23110500 1.58461000
C -3.74348000 0.50457200 -0.42317600
C -5.11909800 0.31759500 -0.23975200
C -5.65161100 -0.93143800 0.03075400
C -4.81575200 -2.04016900 0.12195700
C -3.44914300 -1.88087700 -0.04405400
C -3.17994100 1.81449200 -0.76799400
C -3.74328600 2.99515900 -0.52119300
H -0.00025600 2.20261900 2.96711400
H 2.29740300 1.87290100 2.14751600
H -1.92337200 1.12726500 1.83062700
H 1.37506000 -1.24588600 -1.87044900
H -1.25414700 -1.76929100 -2.17218000
H 3.26645200 2.47463000 -0.49337800
H 5.54694100 2.32854300 -1.42959700
H 6.71080900 0.12954100 -1.47478000
H 5.60092300 -1.85094000 -0.52718900
H 3.80583500 -2.96294900 0.29181800
H 1.81974900 -1.40742500 2.02111400
H 2.12677700 -3.22275600 1.94715000
H -5.78092600 1.16579400 -0.35063200
H -6.72042700 -1.04478500 0.15645000

H -5.22510000 -3.02003700 0.32878200
H -2.77673200 -2.72342900 0.04778500
H -2.21148100 1.79158500 -1.25306200
H -4.68668200 3.09116700 0.00181800
H -3.26124700 3.91398700 -0.82552200

14a:

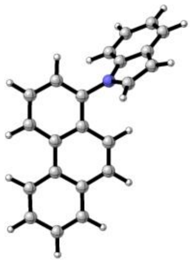

G_sol = –902.316956

C 0.53911300 2.93017700 -0.48960000
C 1.73681700 2.29449900 -0.25534800
C 1.81752900 0.89200900 -0.16585500
C 0.62401700 0.13384900 -0.31591000
C -0.58803300 0.81308900 -0.58944700
C -0.63177300 2.18670000 -0.66722800
C 3.06672900 0.19832900 0.08964200
C 3.05919700 -1.21532900 0.21558700
C 1.82728900 -1.92744700 0.09283100
C 0.66283700 -1.28768100 -0.16484600
C 4.30030200 0.86606400 0.21921300
C 5.46669800 0.17659600 0.46244400
C 5.45258900 -1.21950400 0.58910300
C 4.26477200 -1.89945300 0.46646900
N -1.78503100 0.07207000 -0.77085000
C -2.06850900 -0.75782200 -1.84389600
C -3.30576600 -1.31177900 -1.68888100
C -3.84124300 -0.81390700 -0.45548500
C -2.86351000 0.05243200 0.09678800
C -5.03813900 -1.01285600 0.24290800
C -5.22779400 -0.36271500 1.44893900
C -4.24132500 0.48522500 1.97969300
C -3.04723800 0.70386900 1.31364500
H 0.50289400 4.00976200 -0.55057800
H 2.62642300 2.89366700 -0.13165700
H -1.57508400 2.67414600 -0.87246100
H 1.84245900 -3.00354500 0.21479700
H -0.25915100 -1.84427900 -0.24813700
H 4.34555200 1.94085700 0.12299900
H 6.39967500 0.71708500 0.55573600
H 6.37246100 -1.75605000 0.78135800
H 4.23821600 -2.97812400 0.56124900
H -1.34720600 -0.86922400 -2.63562800
H -3.78472000 -1.98727400 -2.37766100
H -5.80212300 -1.66841000 -0.15584800
H -6.14934300 -0.50852900 1.99735900

H -4.41737500 0.97599500 2.92800700
H -2.28481000 1.35336600 1.72181700

15a:

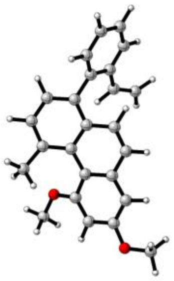

G_sol = –1116.516113

C -0.97095200 3.00629700 -0.41378300
C 0.27663600 2.40652900 -0.45650600
C 0.35744200 0.99400400 -0.30604300
C -0.85700900 0.24455000 -0.37678900
C -2.11340600 0.89451200 -0.28840500
C -2.14741500 2.27472300 -0.27119400
C 1.59950600 0.24361800 -0.19812900
C 1.62698500 -1.11631300 -0.59603400
C 0.39026800 -1.78114500 -0.85924400
C -0.79153000 -1.15654700 -0.66025300
C 2.79704900 0.73934700 0.39616600
C 3.97761700 0.02707500 0.33386600
C 4.00459700 -1.25519200 -0.23616600
C 2.83308000 -1.84438400 -0.65383300
C -3.38904600 0.13407200 -0.26286000
C -3.71230400 -0.74411300 0.79187200
C -4.94072000 -1.41423000 0.75297300
C -5.82634900 -1.24188600 -0.29750000
C -5.50543200 -0.37459700 -1.33598000
C -4.29785700 0.30667900 -1.30879000
C -2.79255300 -0.92195100 1.92340800
C -2.72767300 -1.98850500 2.71807000
C 1.44190600 3.28774300 -0.83180700
O 2.68884200 1.88951800 1.10624700
C 3.84591500 2.43701400 1.70919500
O 5.22966500 -1.84505400 -0.26633900
C 5.31970400 -3.16062400 -0.78577000
H -1.03261900 4.08218000 -0.52874800
H -3.09927800 2.78517500 -0.20098900
H 0.41874400 -2.82325600 -1.15215500
H -1.71780500 -1.69939800 -0.77873900
H 4.89189000 0.40287600 0.76456300
H 2.80091100 -2.86677800 -0.99825200
H -5.21326000 -2.05722500 1.57926400
H -6.77116400 -1.77009300 -0.29939900
H -6.19248400 -0.22759800 -2.15925400
H -4.03543900 0.97644800 -2.11757000
H -2.09995400 -0.10644700 2.09530700

H -3.35523000 -2.85920100 2.57276300
H -2.02282600 -2.02902000 3.53741100
H 1.10775100 4.01382300 -1.57492500
H 2.26143200 2.71843000 -1.26564000
H 1.83627900 3.84024900 0.01994800
H 3.52020300 3.34812700 2.20549100
H 4.60788000 2.68052300 0.96320900
H 4.27061700 1.75315100 2.44921800
H 6.37202900 -3.43042400 -0.74140000
H 4.97559300 -3.20159500 -1.82309800
H 4.73793300 -3.86570500 -0.18504200

15c:

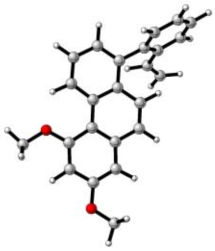

G_sol = –1077.219238

C -0.94075700 3.11719800 0.08260000
C 0.28933100 2.50107800 0.08122400
C 0.41668500 1.10172900 -0.08006700
C -0.79186200 0.35500300 -0.24537200
C -2.05345400 1.00679300 -0.21365100
C -2.11517700 2.37623400 -0.06144900
C 1.69104900 0.39465200 -0.09879000
C 1.69476000 -1.00963400 -0.31777700
C 0.45955500 -1.69759600 -0.50666500
C -0.72550400 -1.05227600 -0.46715400
C 2.98039400 0.99137000 0.09130800
C 4.13633900 0.24130700 0.05005900
C 4.09530700 -1.14153100 -0.17754500
C 2.88579600 -1.76462500 -0.35817300
C -3.32390700 0.24461500 -0.35819000
C -3.79822100 -0.60727600 0.65882700
C -5.01279600 -1.27442500 0.46057300
C -5.73740700 -1.12381900 -0.70984300
C -5.26500900 -0.28354400 -1.71198800
C -4.06917800 0.39510500 -1.52793000
C -3.03901600 -0.76501200 1.90622300
C -3.11230400 -1.79775100 2.74386200
O 3.03388100 2.32985700 0.32293400
C 4.28958900 2.95221900 0.52933200
O 5.30699700 -1.75785900 -0.19540300
C 5.33468500 -3.15721500 -0.41970300
H -0.99592800 4.19156700 0.20488800
H 1.16929900 3.10238100 0.19999500
H -3.07969000 2.86686000 -0.04026000
H 0.49439000 -2.76563800 -0.68211800
H -1.64499400 -1.59953400 -0.61241400
H 5.10529600 0.69106400 0.19309300
H 2.81181900 -2.82731800 -0.53117800
H -5.40461800 -1.89889600 1.25241300
H -6.67580000 -1.64861300 -0.83504500
H -5.82653100 -0.15385200 -2.62809500
H -3.69059800 1.04792000 -2.30395500
H -2.34609900 0.03649600 2.13424900
H -3.74831600 -2.65402000 2.55620200
H -2.51753200 -1.82370500 3.64681200
H 4.07294700 4.00471600 0.69633400
H 4.93506600 2.84837800 -0.34683700
H 4.79813700 2.54198300 1.40578000
H 6.38283800 -3.44513100 -0.39661600
H 4.90825400 -3.41110400 -1.39433700
H 4.79163000 -3.69513800 0.36257500

15d:

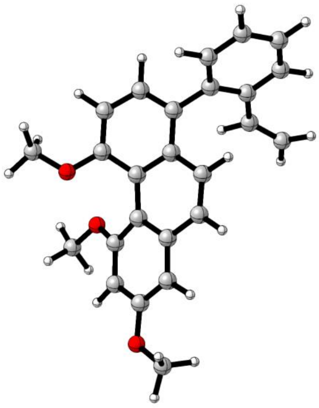

G_sol = –1191.753662

C -0.91402400 2.87459800 -0.22785000
C 0.29162500 2.20235500 -0.31974200
C 0.33201000 0.78182900 -0.22466700
C -0.90741600 0.09193500 -0.33762400
C -2.14036700 0.79082600 -0.23381700
C -2.11506700 2.16594700 -0.14353800
C 1.55405300 0.00651500 -0.14274500
C 1.53885800 -1.33997400 -0.58401300
C 0.28156300 -1.95583100 -0.86641300
C -0.88390900 -1.30111800 -0.66015300
C 2.76903800 0.45614300 0.45793800
C 3.92848500 -0.28439700 0.34988100
C 3.91484600 -1.54928900 -0.25951500
C 2.72433400 -2.09848200 -0.67677300
C -3.44577500 0.08296000 -0.25433000
C -3.81720700 -0.82735000 0.75678600
C -5.07033400 -1.44587900 0.67540400
C -5.93662000 -1.19095200 -0.37446900
C -5.56941400 -0.29148500 -1.36928000
C -4.33524800 0.33738400 -1.30037800
C -2.91950100 -1.08960500 1.88948500

C -2.90773300 -2.18887800 2.64101400
O 2.69742100 1.56940000 1.22414900
C 3.88586000 2.10381000 1.77119400
O 5.12527100 -2.16761000 -0.32466300
C 5.17491700 -3.46932300 -0.88169000
H -0.94346400 3.95285900 -0.26002300
H -3.04578900 2.71020700 -0.04856400
H 0.27839100 -2.98997700 -1.18803000
H -1.82532400 -1.81163900 -0.80039900
H 4.85846200 0.05214900 0.77975600
H 2.66202500 -3.10841600 -1.05232000
H -5.37775100 -2.11379900 1.46908100
H -6.90159900 -1.68015100 -0.40942000
H -6.24060700 -0.08004600 -2.19166800
H -4.03630100 1.03013700 -2.07650400
H -2.19615200 -0.31088200 2.10103000
H -3.56868900 -3.02637100 2.45436000
H -2.21468100 -2.29134400 3.46500500
H 3.59100500 3.00764800 2.29932400
H 4.60591100 2.35696300 0.98760900
H 4.35178800 1.40976600 2.47709600
H 6.22051800 -3.76690000 -0.85971800
H 4.81606000 -3.47259400 -1.91491600
H 4.58332900 -4.17660400 -0.29306800
O 1.45616200 2.83053300 -0.61188000
C 1.49565900 4.24223800 -0.59427200
H 2.52843700 4.51517400 -0.79880800
H 0.85104800 4.67429500 -1.36587800
H 1.20074400 4.63706900 0.38260000

16c:

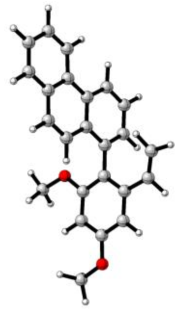

G_sol = –1077.219727

C 1.29373900 2.25637400 2.04113000
C 0.02015200 1.89886200 1.58718400
C -0.13608300 0.91307000 0.63664000
C 1.00583100 0.24676200 0.12240800
C 2.30164600 0.60150100 0.58563400
C 2.41057900 1.62125000 1.55010500
C 0.87320200 -0.76765000 -0.87681100
C 1.95457500 -1.40575200 -1.38106300
C 3.27519400 -1.09464300 -0.93463900
C 3.46116500 -0.08963100 0.05039800
C 4.39348000 -1.77004600 -1.46182400
C 5.66653700 -1.47367700 -1.03721700
C 5.85679000 -0.48330400 -0.06339800
C 4.77840200 0.19018400 0.46488000
C -1.50413400 0.55557400 0.17221200
C -2.10883800 -0.58376300 0.71862300
C -3.40555900 -0.96968700 0.37830900
C -4.12210900 -0.18836500 -0.52435300
C -3.54361500 0.94585400 -1.07481200
C -2.23723500 1.32353100 -0.75138500
O -1.34774700 -1.28156600 1.60456300
O -5.39496000 -0.46632500 -0.91977600
C -6.03262300 -1.61253500 -0.38692300
C -1.86381000 -2.47082400 2.16997700
C -1.73401400 2.52265500 -1.44190000
C -0.48266400 2.94086900 -1.62823000
H 1.39976600 3.03672000 2.78373900
H -0.85543700 2.40420700 1.97279900
H 3.38215700 1.91696200 1.91720200
H -0.11894100 -1.01191400 -1.22950300
H 1.83586200 -2.16938100 -2.14027600
H 4.23033200 -2.53276400 -2.21371100
H 6.51746500 -1.99983200 -1.45001300
H 6.85667400 -0.24555800 0.27616500
H 4.95816500 0.94656300 1.21467600
H -3.84453200 -1.84901200 0.81695000
H -4.12126300 1.53045200 -1.77753800
H -7.02339000 -1.64017000 -0.83391700
H -6.12899600 -1.54746900 0.70110100
H -5.49319200 -2.52814600 -0.64768200
H -2.09574800 -3.21229900 1.39957600
H -2.76010900 -2.27579900 2.76654500
H -1.07949700 -2.85728100 2.81605600
H -2.52566300 3.11370600 -1.89296700
H 0.38309300 2.42258800 -1.24809800
H -0.29874200 3.84191400 -2.19907200

16a:

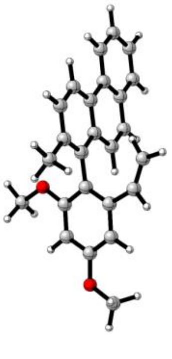

G_sol = –1116.534424

C -1.28175600 2.79952800 0.81624000
C -0.00177300 2.24737300 0.65123200
C 0.10403800 0.92829200 0.23806400
C -1.05736200 0.14854000 0.00911800
C -2.34616700 0.71834700 0.18528000
C -2.41953700 2.06284400 0.58879700
C 1.45177500 0.32426700 0.04849800
C 2.21463800 0.47542700 -1.11494600
C 1.78990300 1.21350600 -2.31503000
C -0.95154900 -1.21266000 -0.42006700
C 1.99883800 -0.41155700 1.11694800
C 3.25677600 -0.98876400 1.03793100
C 4.00843300 -0.82655300 -0.12588000
C 3.49809700 -0.09820000 -1.18676400
C -3.52577400 -0.09191000 -0.05548200
C -3.36635400 -1.43857100 -0.47428600
C -4.50395800 -2.23444400 -0.71238900
C -5.77113400 -1.72902900 -0.54475100
C -5.93517800 -0.39996200 -0.12986700
C -4.83725600 0.39622700 0.10788300
C -2.05109700 -1.96712700 -0.65028400
C 0.58422500 1.64900800 -2.68205100
C 1.22037600 3.08552100 0.90137500
O 1.20925100 -0.50555600 2.21933100
C 1.66870100 -1.26215300 3.32435400
O 5.23303600 -1.42108500 -0.11653600
C 6.04895900 -1.29197600 -1.26786500
H -1.37013200 3.83180800 1.13260200
H -3.38040200 2.53566400 0.72962900
H 2.60731100 1.39723000 -3.00667700
H 0.03483100 -1.63061100 -0.56325100
H 3.68398900 -1.55599500 1.84977500
H 4.06963200 0.03570800 -2.09224500
H -4.36089300 -3.25961600 -1.03225300
H -6.63732900 -2.35069600 -0.73065900
H -6.93028800 0.00407800 0.00499400
H -4.99662400 1.41555600 0.42797400
H -1.95059500 -2.99535100 -0.97641500
H -0.30824400 1.50791100 -2.09457500
H 0.46994300 2.16660400 -3.62583100
H 0.94766600 4.05662300 1.31388200
H 1.77656900 3.25293600 -0.02387600
H 1.90144000 2.59425500 1.59849000
H 2.58799200 -0.84156200 3.74215800
H 1.84256300 -2.30578800 3.04722400
H 0.87743800 -1.21247300 4.06813800
H 6.96511500 -1.83487600 -1.04850800
H 6.28772800 -0.24416300 -1.47164400
H 5.56880900 -1.72995100 -2.14763600

16d:

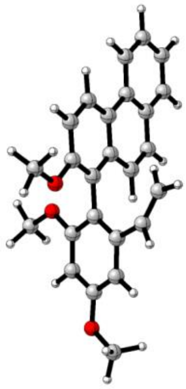

G_sol = –1191.768188

C -1.23423700 2.71687400 0.55565900
C 0.01368800 2.08895500 0.43199300
C 0.08892800 0.73651500 0.12723100
C -1.10060000 -0.00663000 -0.03735000
C -2.37019900 0.62031800 0.09514200
C -2.39243200 1.99192300 0.38897300
C 1.42308800 0.09528000 -0.00830000
C 2.23523100 0.24168300 -1.13686000
C 1.85983300 0.99600000 -2.34380400
C -1.04658400 -1.39969800 -0.36620600
C 1.91294900 -0.65556800 1.07685000
C 3.16182400 -1.25643100 1.04124400
C 3.96364400 -1.09598300 -0.08868600
C 3.51137900 -0.34811100 -1.16258200
C -3.57943700 -0.16248100 -0.08015800
C -3.47013000 -1.54134000 -0.39740200
C -4.63517100 -2.31255200 -0.57220900
C -5.88394500 -1.75272800 -0.43946200
C -5.99875800 -0.39184200 -0.12452000
C -4.87241500 0.38135100 0.05003700
C -2.17400800 -2.12694400 -0.53808400
C 0.64974700 1.28339100 -2.82110100
O 1.08055000 -0.74184700 2.14789100
C 1.48877200 -1.50485700 3.26766800
O 5.17721400 -1.71223300 -0.03454200
C 6.04273000 -1.58349100 -1.14847800
H -1.29923800 3.76974900 0.78491400
H -3.33505900 2.50911200 0.49147700
H 2.71522900 1.31677000 -2.93191800
H -0.07646800 -1.86188200 -0.48128800
H 3.54720800 -1.83589100 1.86526900
H 4.12175200 -0.21410800 -2.04247000
H -4.52886000 -3.36308200 -0.81502000
H -6.77205000 -2.35591900 -0.57602900
H -6.97835300 0.05609100 -0.01699400
H -4.99569900 1.42650600 0.29431900
H -2.11172900 -3.17908600 -0.78909600
H -0.26580400 0.98461400 -2.33508100

H 0.55351500 1.83146600 -3.74956500
H 2.39852200 -1.09769100 3.71839900
H 1.65752200 -2.55120600 2.99699100
H 0.67209500 -1.44414200 3.98277000
H 6.93984600 -2.14462800 -0.89801800
H 6.30739400 -0.53756800 -1.32914600
H 5.59307500 -2.00233200 -2.05342900
O 1.19616000 2.74175300 0.58947800
C 1.17874500 4.13609300 0.82862900
H 2.22085000 4.44001300 0.89222700
H 0.69385500 4.67615400 0.01017600
H 0.67516000 4.37730800 1.76955400

17a:

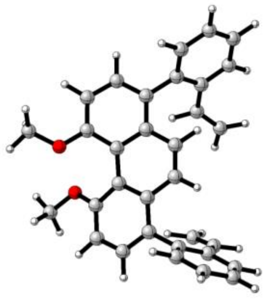

G_sol = –1385.699341

C -2.86061900 2.60544000 -0.62237000
C -1.47757400 2.63816100 -0.57993300
C -0.74155400 1.45412100 -0.29317000
C -1.44562000 0.22141700 -0.35962900
C -2.86581600 0.19770400 -0.38588500
C -3.54306600 1.39521000 -0.48096900
C 0.68955000 1.42466200 -0.05064500
C 1.40277800 0.22499800 -0.31801400
C 0.65931500 -0.96546600 -0.58575900
C -0.69006200 -0.98353700 -0.50819000
C 1.41516800 2.49216000 0.55048500
C 2.79829800 2.46209300 0.58362500
C 3.49079300 1.32941100 0.14720900
C 2.81837700 0.19121000 -0.23799300
C -3.62777300 -1.07551700 -0.34313600
C 3.58354700 -1.04372700 -0.55675300
C -3.54949600 -1.95367500 0.75799800
C -4.30516400 -3.13146100 0.73182000
C -5.11091100 -3.45074900 -0.34841500
C -5.18901800 -2.58242000 -1.43155600
C -4.45344400 -1.40677800 -1.41955100
C -2.71887800 -1.61925900 1.92270100
C -2.17525800 -2.49053900 2.77002700
C 4.22300000 -1.10418800 -1.79412800
C 4.95868000 -2.21463000 -2.18658200
C 5.05621100 -3.30228900 -1.33016200
C 4.42749700 -3.25604500 -0.09677300
C 3.69332400 -2.13887800 0.32811900
C 3.10945200 -2.22157600 1.67397700
C 2.53875400 -1.28454000 2.43195200
O -0.76335100 3.74067900 -0.90992200
C -1.43948400 4.97039900 -1.07483000
O 0.69125900 3.45762400 1.16687900
C 1.35859800 4.59707000 1.66946500
H -3.42515600 3.50782900 -0.79957200
H -4.62545200 1.39438100 -0.48763100
H 1.20270500 -1.87694500 -0.78675600
H -1.22590500 -1.91360400 -0.62713800
H 3.35505600 3.28829800 0.99796400
H 4.57187100 1.31789300 0.19697400
H -4.27989300 -3.78875200 1.59082300
H -5.68799100 -4.36649100 -0.33970100
H -5.81917900 -2.81906500 -2.27912400
H -4.49768900 -0.73203200 -2.26479700
H -2.52626700 -0.56327000 2.07199900
H -2.28316600 -3.56133300 2.64834300
H -1.58056000 -2.15402900 3.60832100
H 4.12461800 -0.25841500 -2.46267200
H 5.44089600 -2.23100500 -3.15534900
H 5.61710300 -4.18152700 -1.61954300
H 4.50871500 -4.10313700 0.57358300
H 3.18825000 -3.21616300 2.10421100
H 2.41425800 -0.25897000 2.12420600
H 2.17625400 -1.54491200 3.41791800
H -0.66844600 5.71107800 -1.27422600
H -1.98689700 5.24798300 -0.16922400
H -2.13384000 4.93920900 -1.91988000
H 0.58220000 5.24818400 2.06459700
H 1.90534100 5.11943900 0.87884600
H 2.05227700 4.33624000 2.47455000

18a:

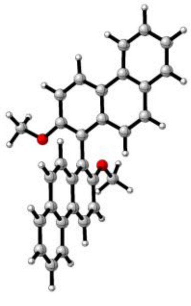

G_sol = –1307.158936
C -2.18562200 1.87843500 -1.96919000
C -0.93065400 1.86269500 -1.34248800
C -0.66914000 0.95565200 -0.32465100
C -1.67599300 0.05269200 0.08135400
C -2.95060500 0.06070700 -0.55054300
C -3.16447500 0.99416600 -1.57553700
C 0.66914000 0.95565200 0.32465000
C 1.67599300 0.05269200 -0.08135400

C 1.42860600 -0.88720500 -1.13410000
C -1.42860600 -0.88720500 1.13410000
C 0.93065400 1.86269500 1.34248800
C 2.18562200 1.87843500 1.96919000
C 3.16447500 0.99416600 1.57553700
C 2.95060500 0.06070700 0.55054300
C -3.96946200 -0.87955200 -0.12254700
C 3.96946200 -0.87955200 0.12254700
C -3.67208000 -1.79203200 0.92286200
C -4.65064600 -2.71123200 1.34744900
C -5.89603100 -2.74157600 0.76581900
C -6.19626100 -1.84252100 -0.26669400
C -5.25415000 -0.93518300 -0.69785800
C -2.37943100 -1.76341900 1.53154500
C 5.25415000 -0.93518300 0.69785800
C 6.19626100 -1.84252000 0.26669400
C 5.89603100 -2.74157600 -0.76581900
C 4.65064700 -2.71123100 -1.34744900
C 3.67208000 -1.79203100 -0.92286200
C 2.37943200 -1.76341900 -1.53154500
O 0.08549400 2.70093100 -1.68046900
C -0.12465300 3.64786000 -2.71120800
O -0.08549400 2.70093100 1.68046800
C 0.12465300 3.64785900 2.71120900
H -2.39507700 2.57959800 -2.76280100
H -4.11798500 1.03414900 -2.08126900
H 0.45782300 -0.88398800 -1.60867200
H -0.45782300 -0.88398800 1.60867100
H 2.39507700 2.57959800 2.76280100
H 4.11798500 1.03414900 2.08126900
H -4.40239500 -3.39919200 2.14672900
H -6.63985400 -3.45271800 1.10072700
H -7.17464200 -1.86009000 -0.72954100
H -5.51779200 -0.25632600 -1.49587200
H -2.17296300 -2.46724000 2.32888100
H 5.51779200 -0.25632600 1.49587200
H 7.17464200 -1.86009000 0.72954100
H 6.63985400 -3.45271700 -1.10072700
H 4.40239500 -3.39919200 -2.14673000
H 2.17296300 -2.46724000 -2.32888100
H 0.80889700 4.19731800 -2.80500400
H -0.93022300 4.34345600 -2.45828200
H -0.35339700 3.16065000 -3.66359500
H -0.80889800 4.19731600 2.80500600
H 0.93022200 4.34345500 2.45828400
H 0.35339600 3.16064800 3.66359500

NMR Spectra

4-(2-Vinylphenyl)tetraphene (1):

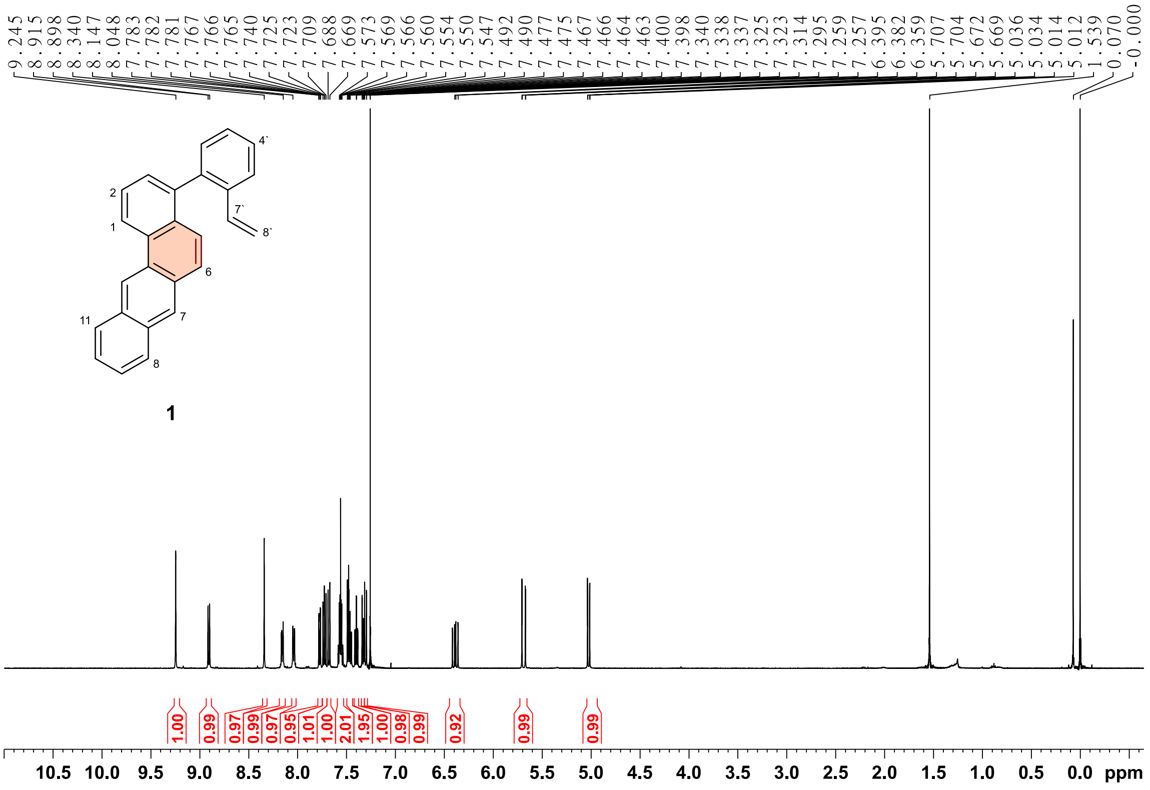

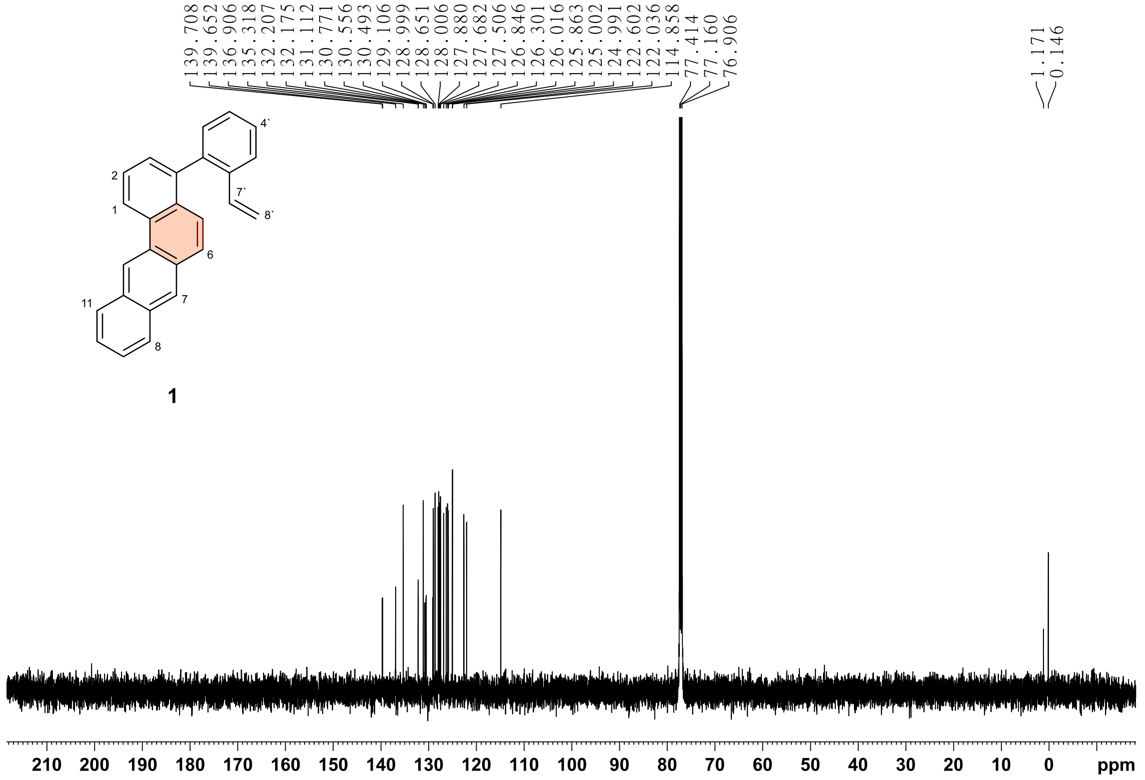

1-Vinyl-4-(2-vinylphenyl)naphthalene (3):

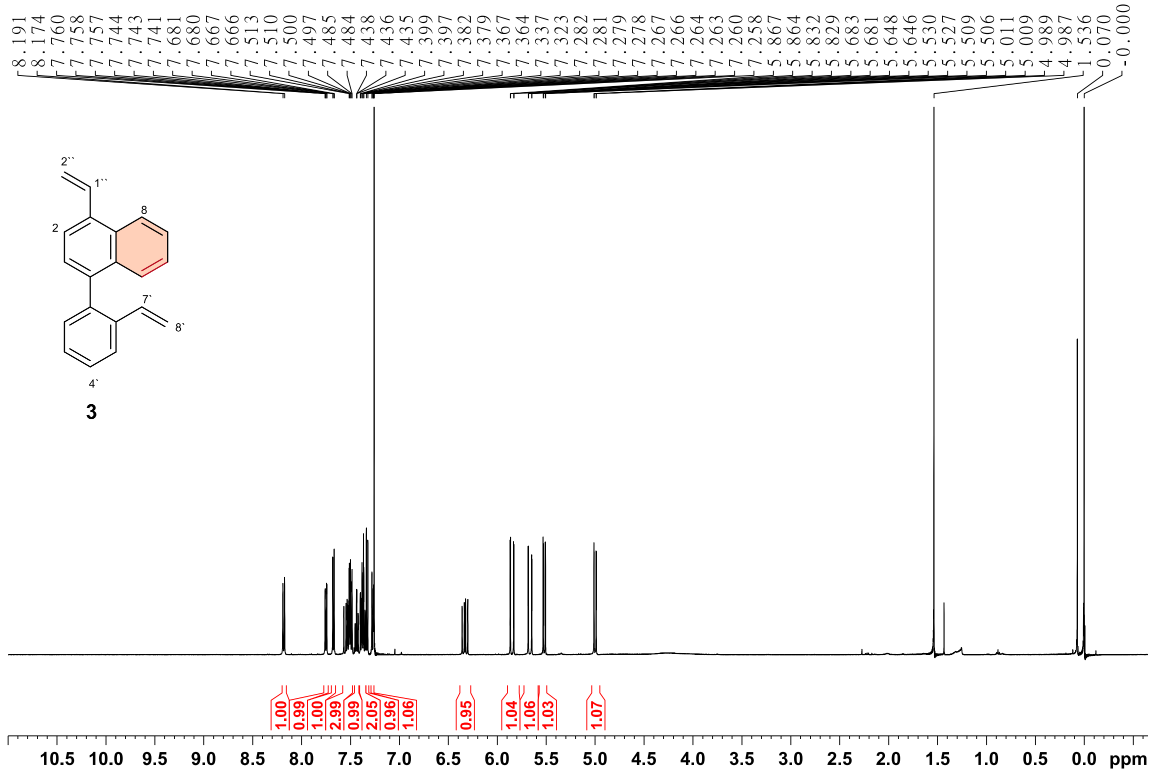

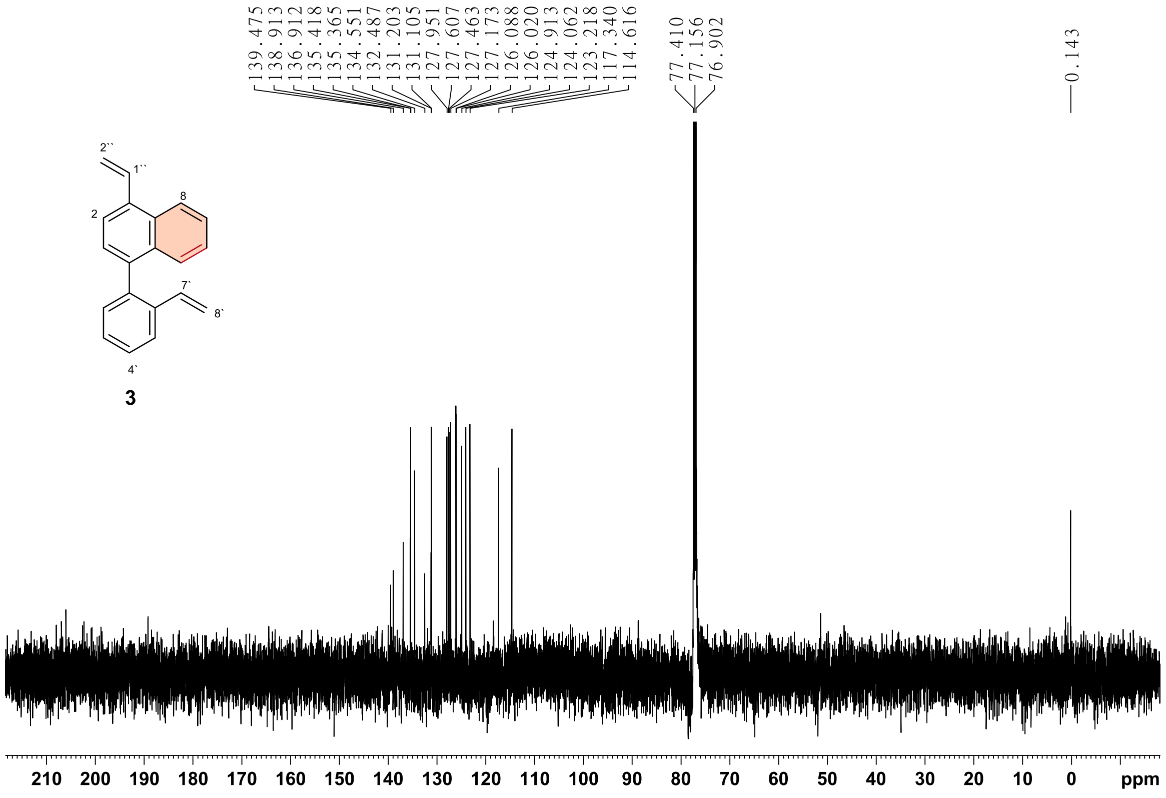

1-Methyl-4-(2-vinylphenyl)-1*H*-indole-7-carbaldehyde

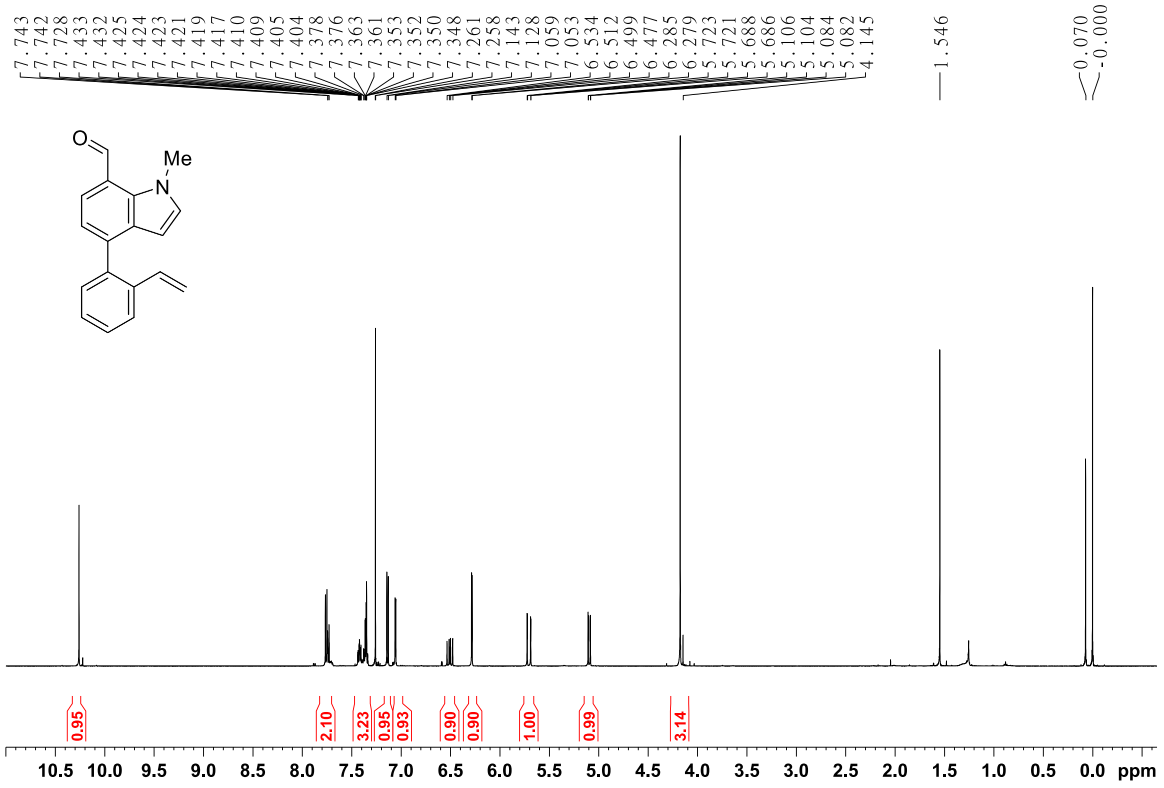

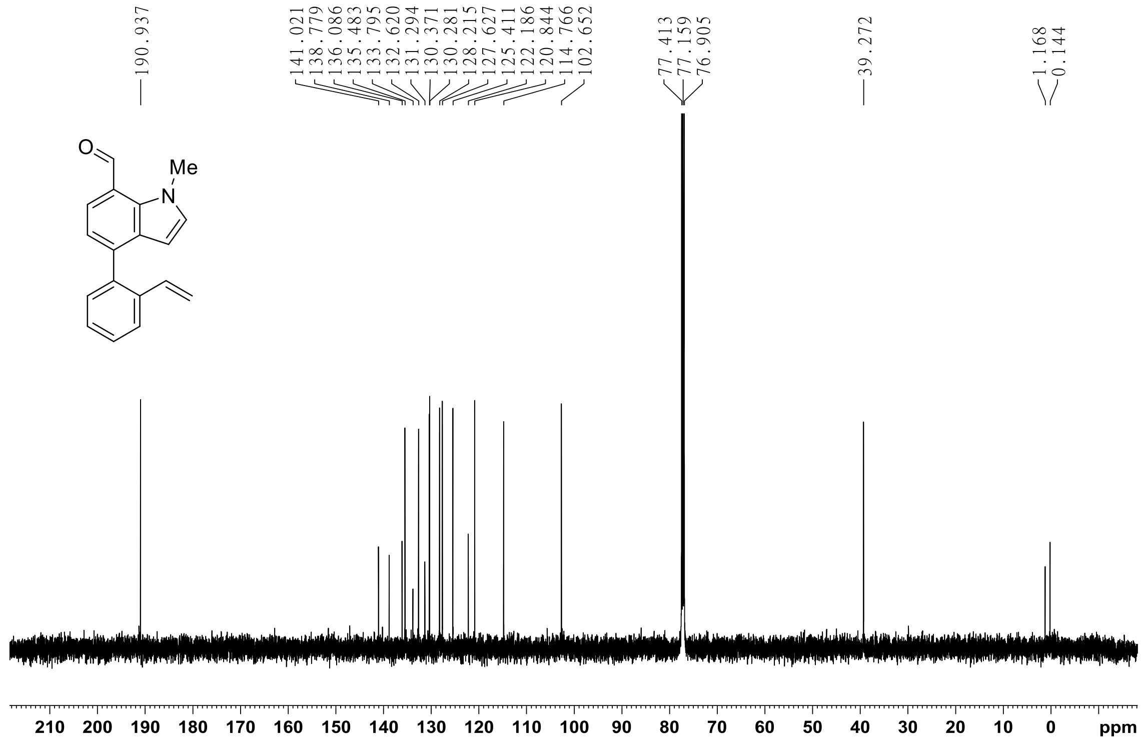

1-Methyl-7-vinyl-4-(2-vinylphenyl)-1*H*-indole (5a):

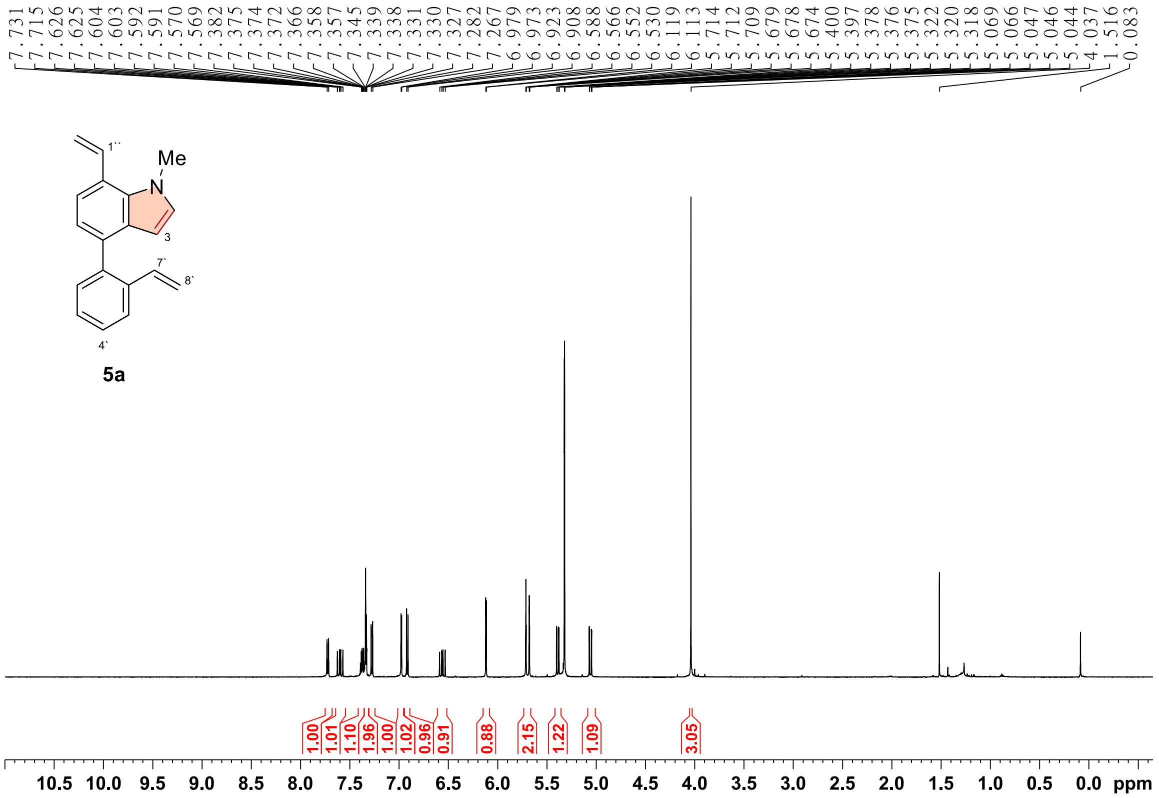

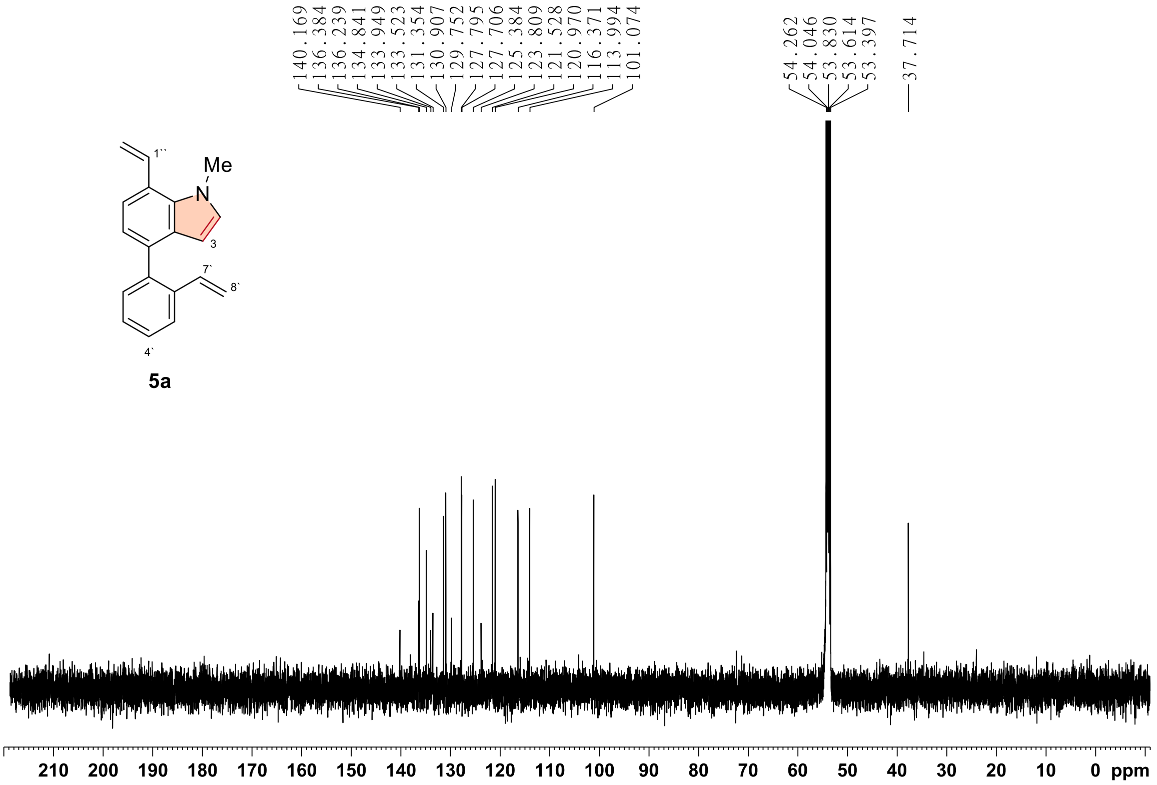

4-(2-Vinylphenyl)benzofuran-7-carbaldehyde (S1):

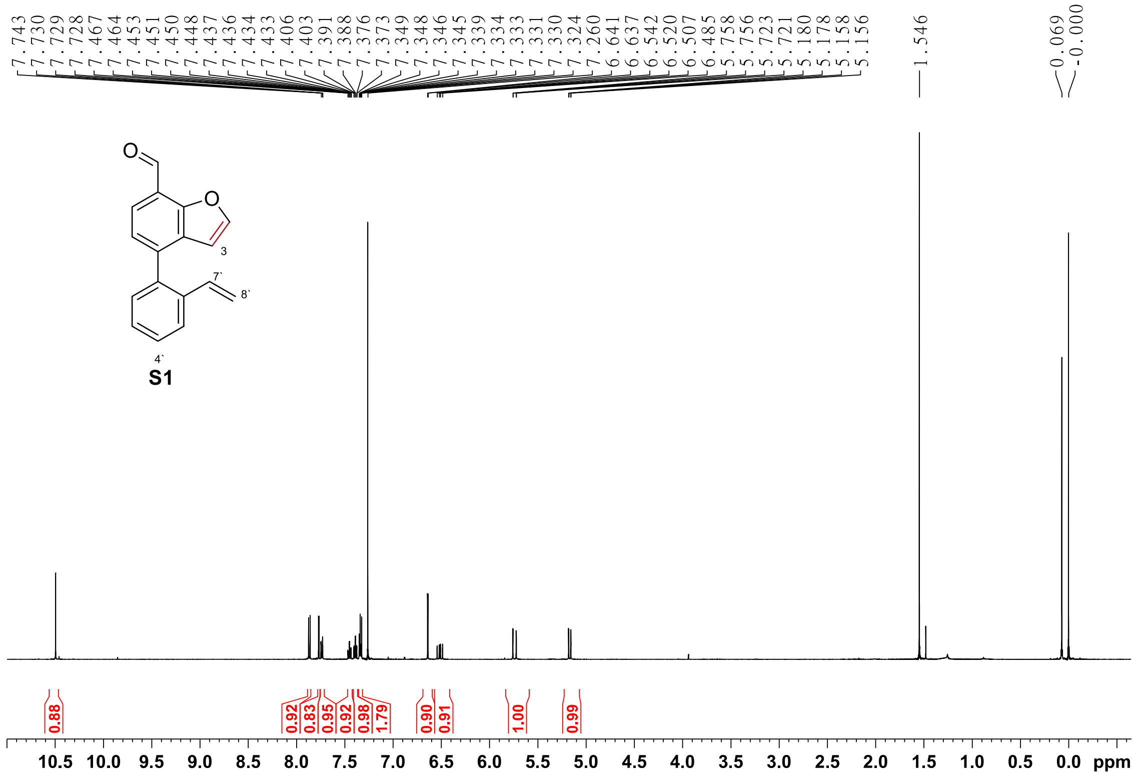

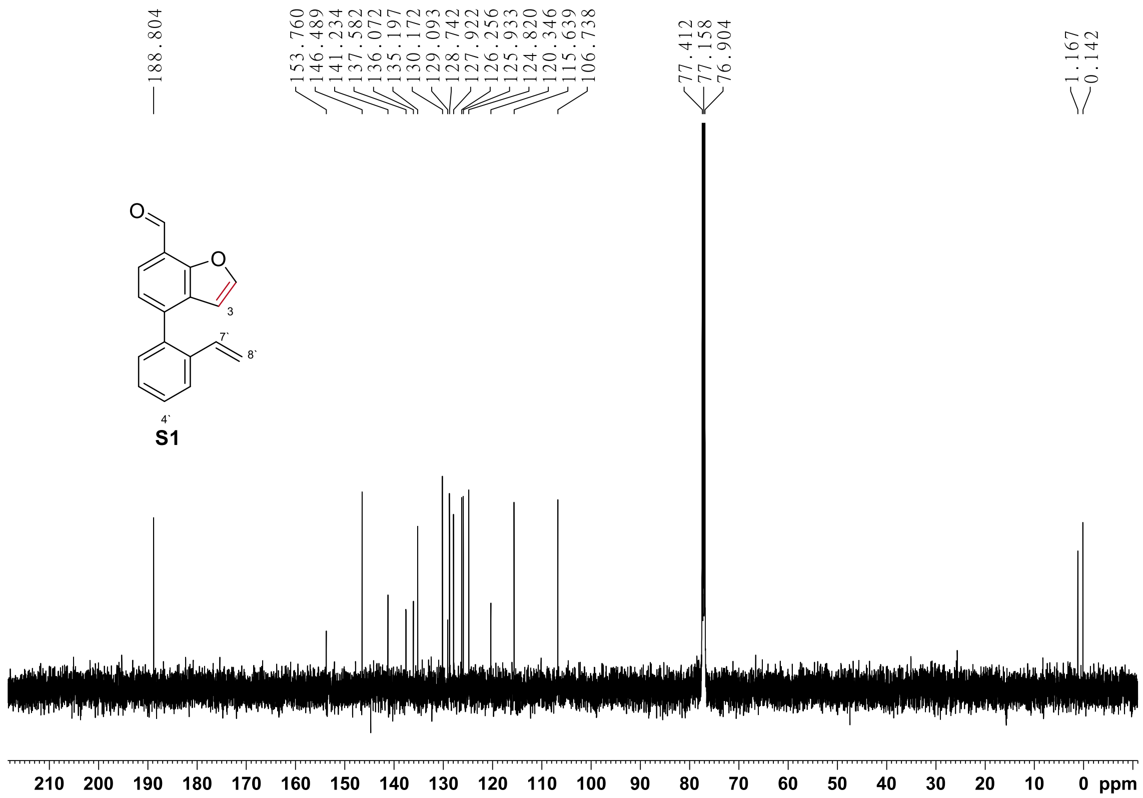

7-Vinyl-4-(2-vinylphenyl)benzofuran (5b):

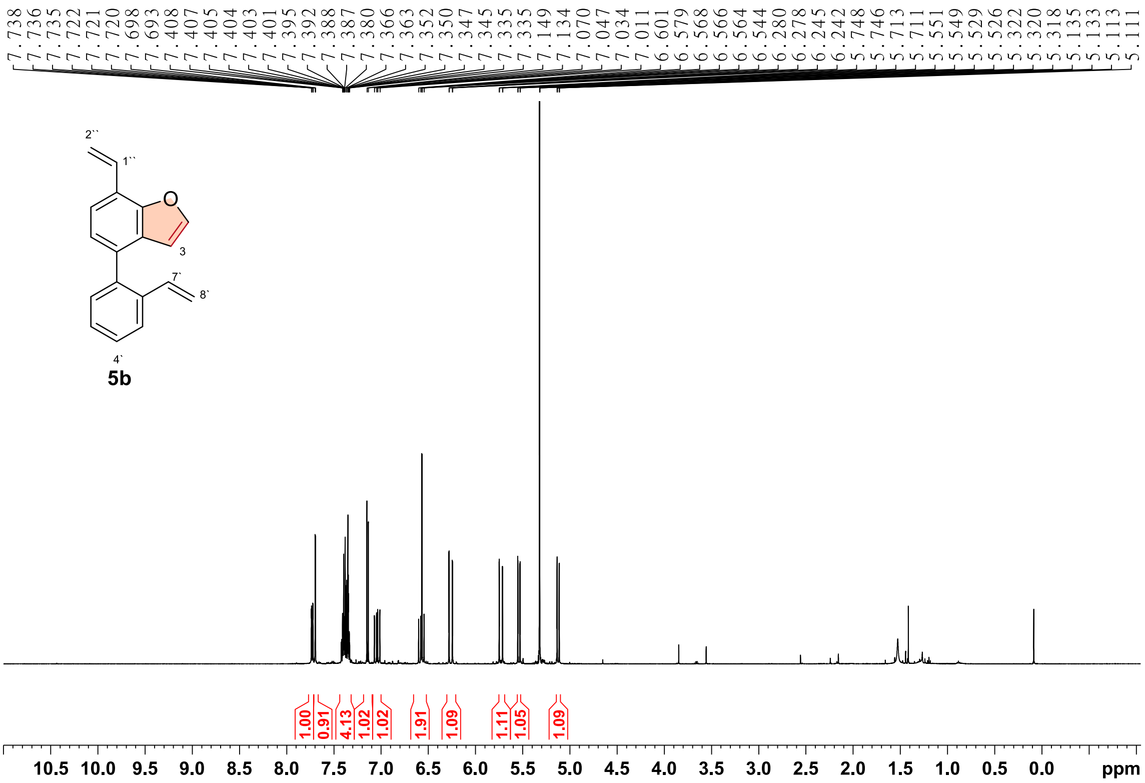

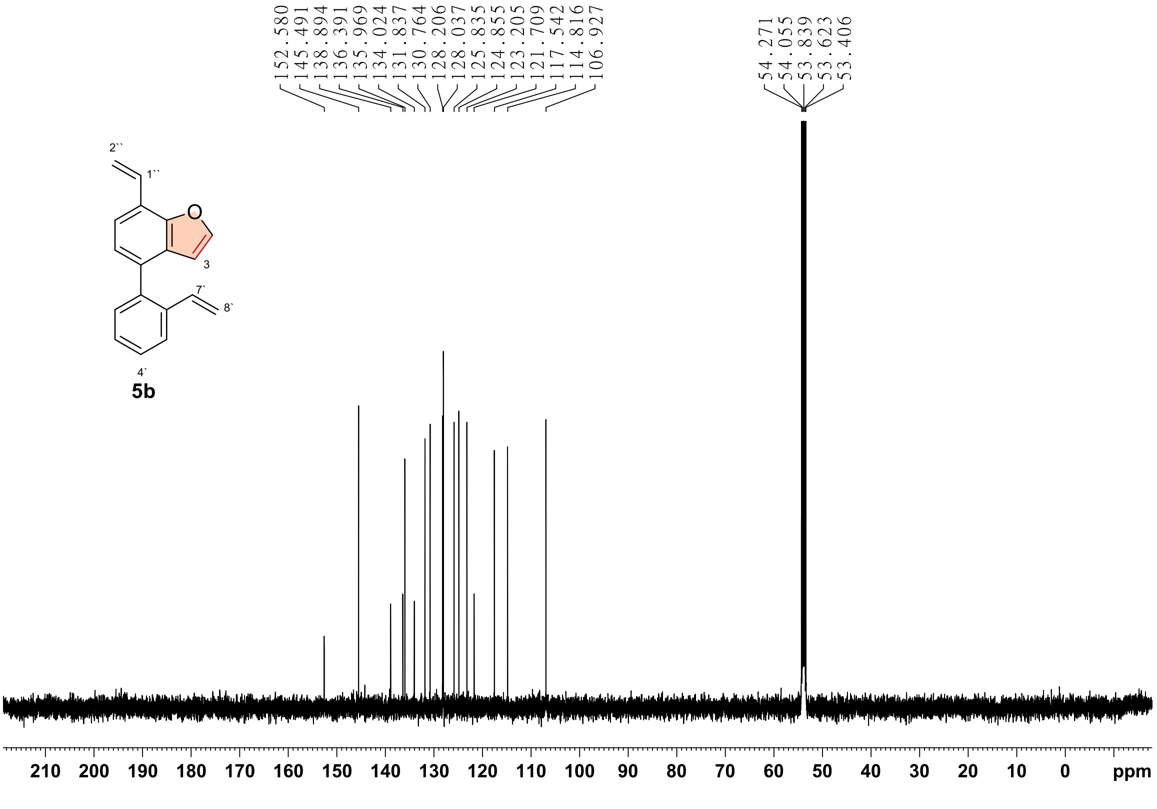

2-(9,11-Dimethylphenanthro[3,4-d][1,3]dioxol-5-yl)-4,4,5,5-tetramethyl-1,3,2-dioxaborolane (S2a):

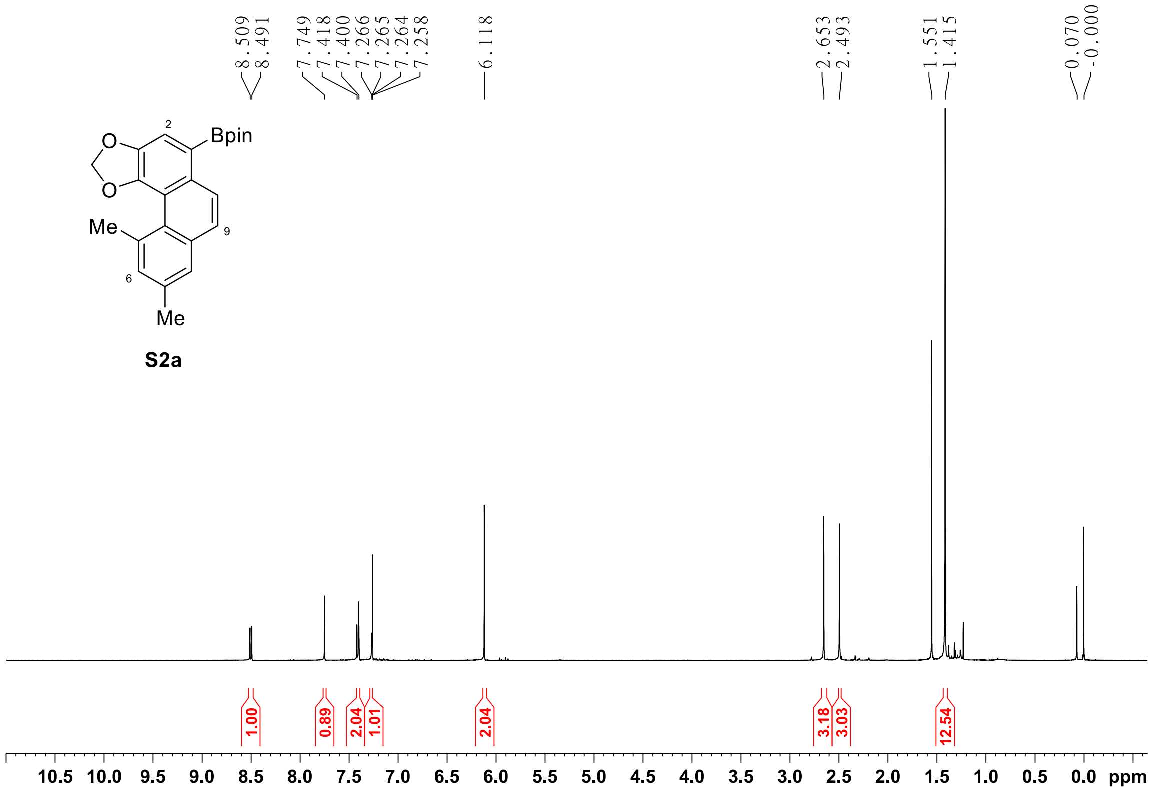

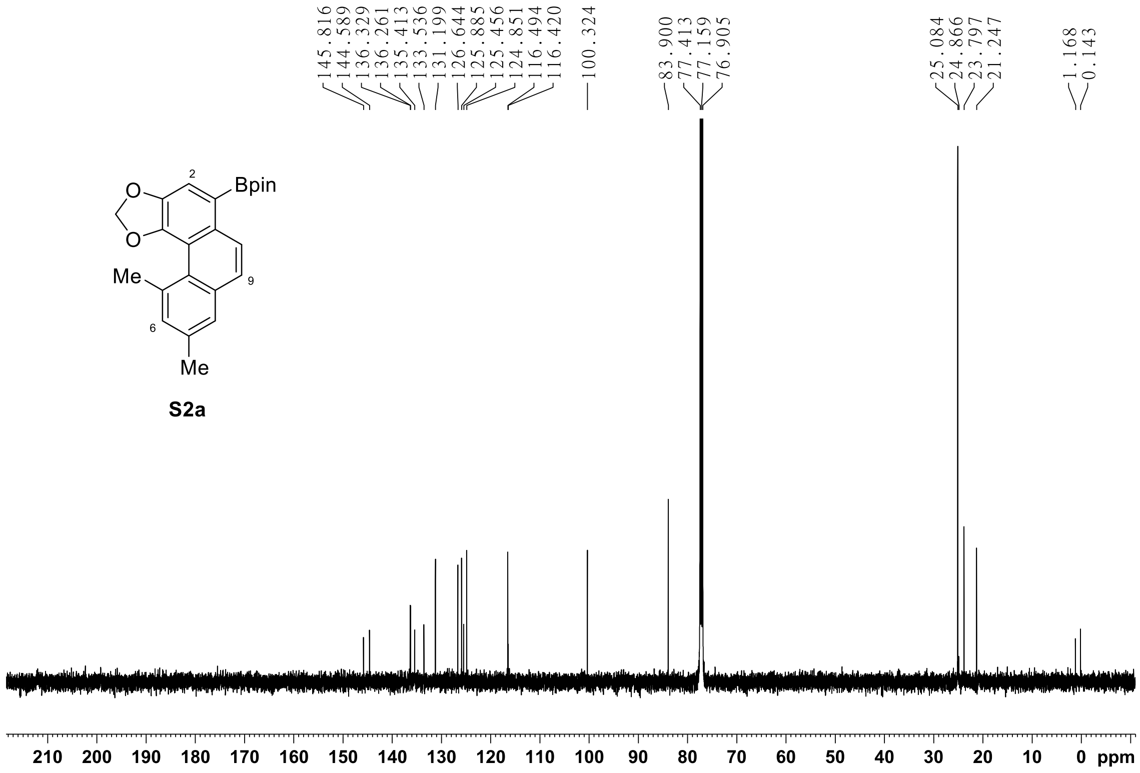

2,5-Bis(9,11-dimethylphenanthro[3,4-d][1,3]dioxol-5-yl)terephthalaldehyde (S3a):

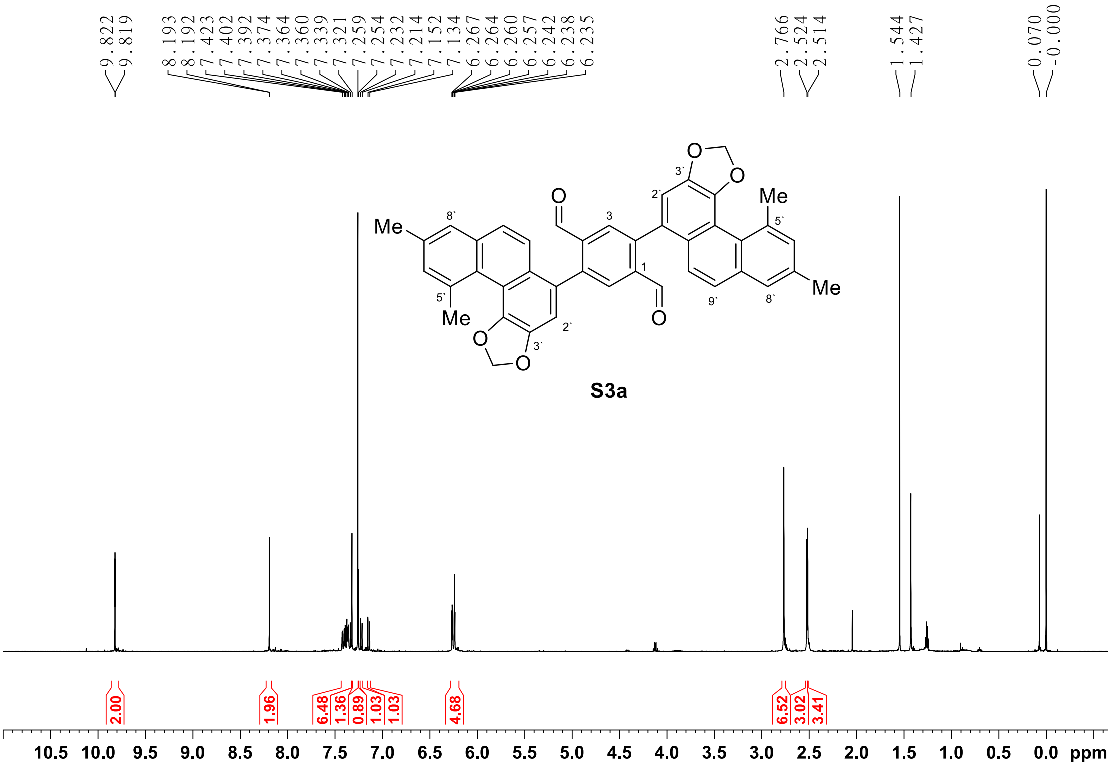

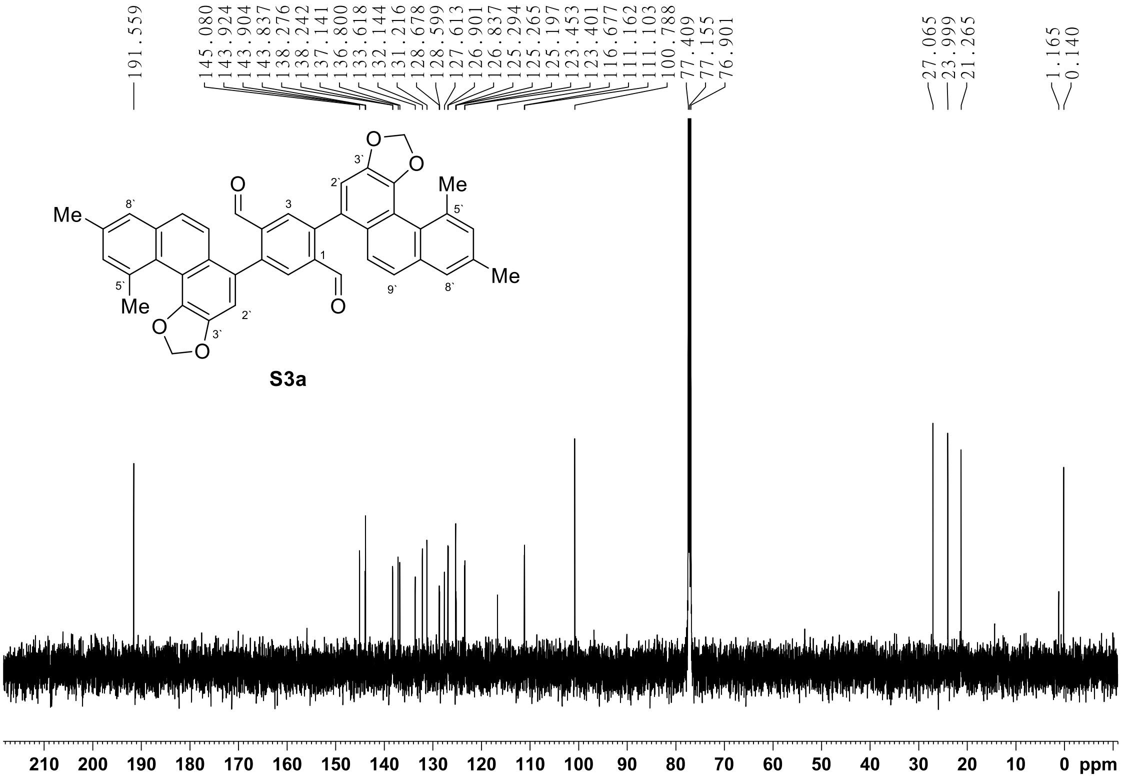

5,5'-(2,5-Divinyl-1,4-phenylene)bis(9,11-dimethylphenanthro[3,4-*d*][1,3]dioxole) (7a):

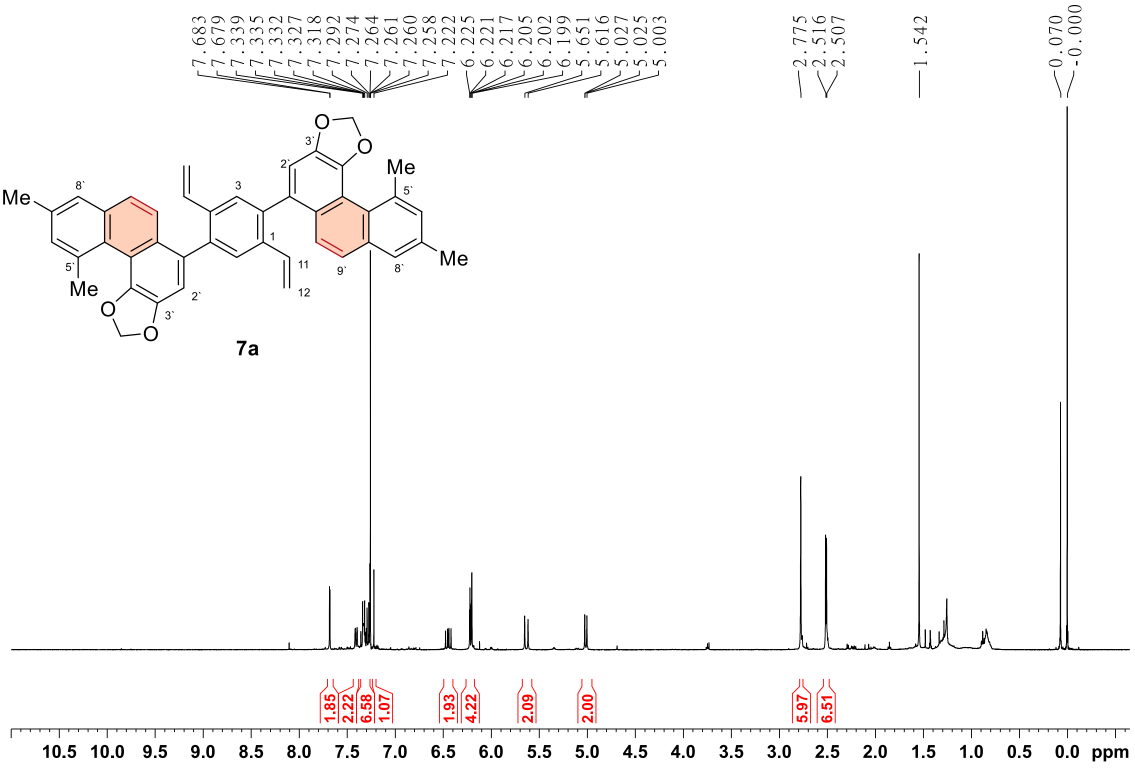

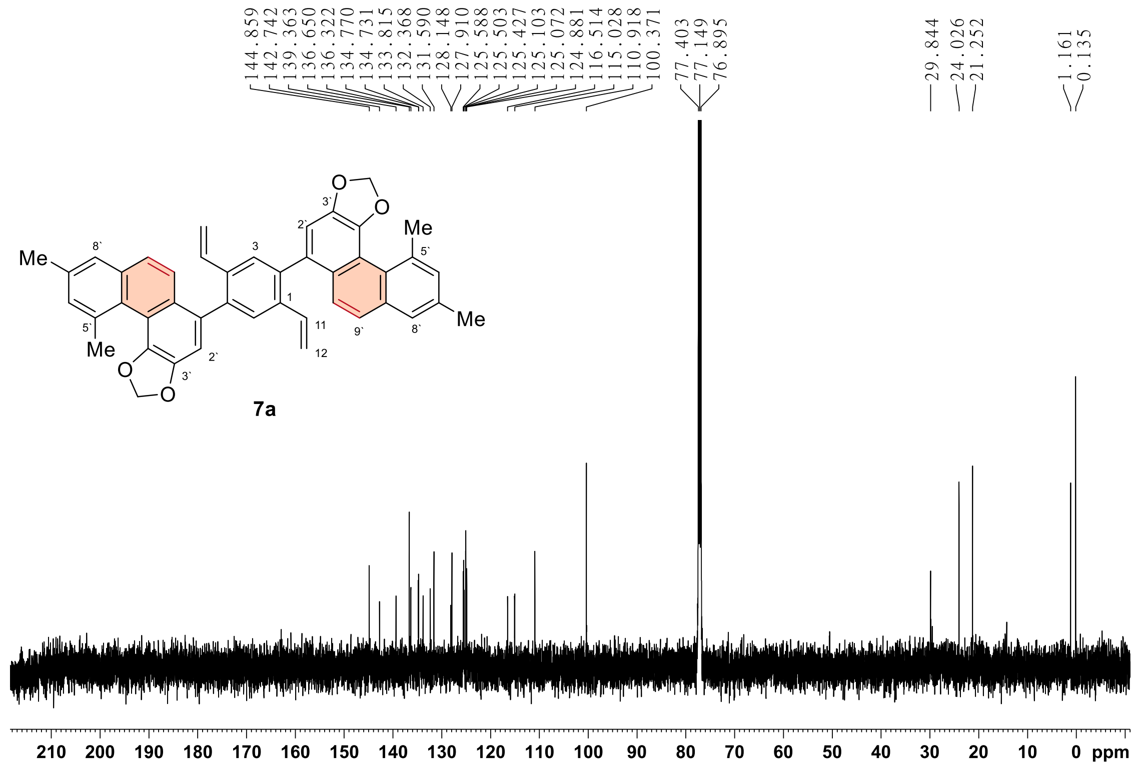

2-(5,7-Dimethoxy-4-methylphenanthren-1-yl)-4,4,5,5-tetramethyl-1,3,2-dioxaborolane (S2b):

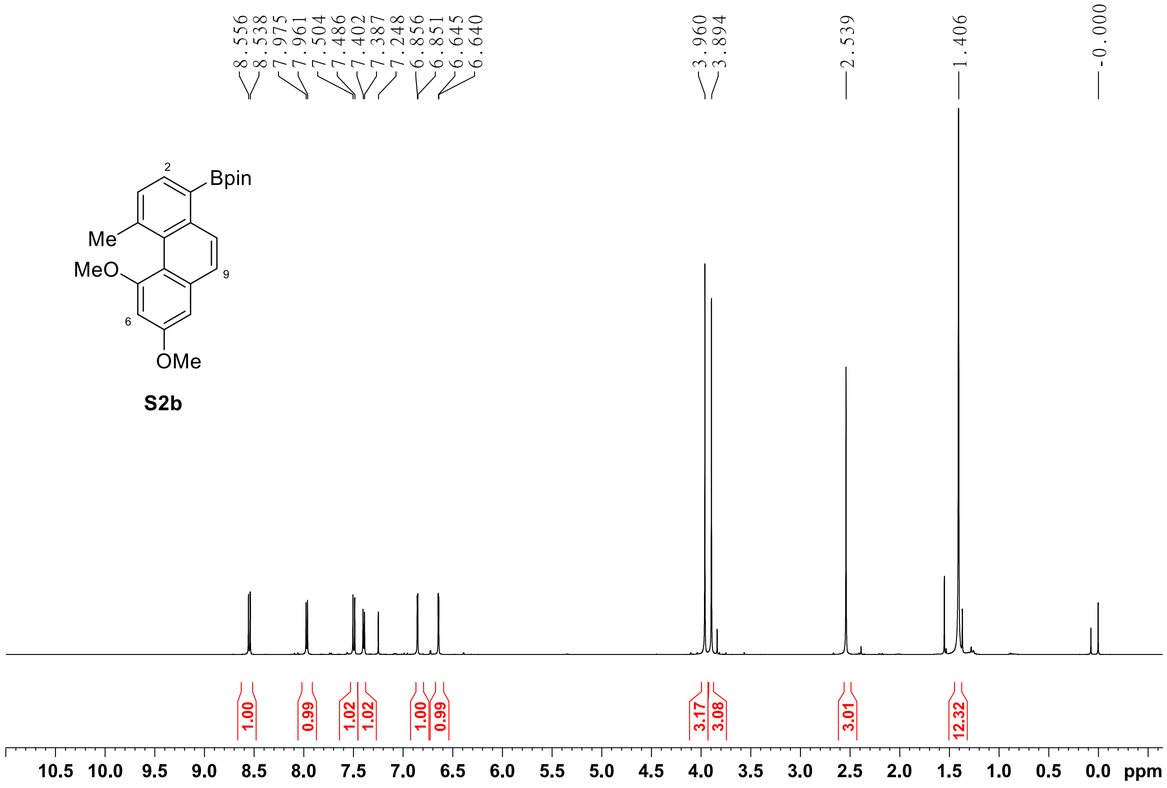

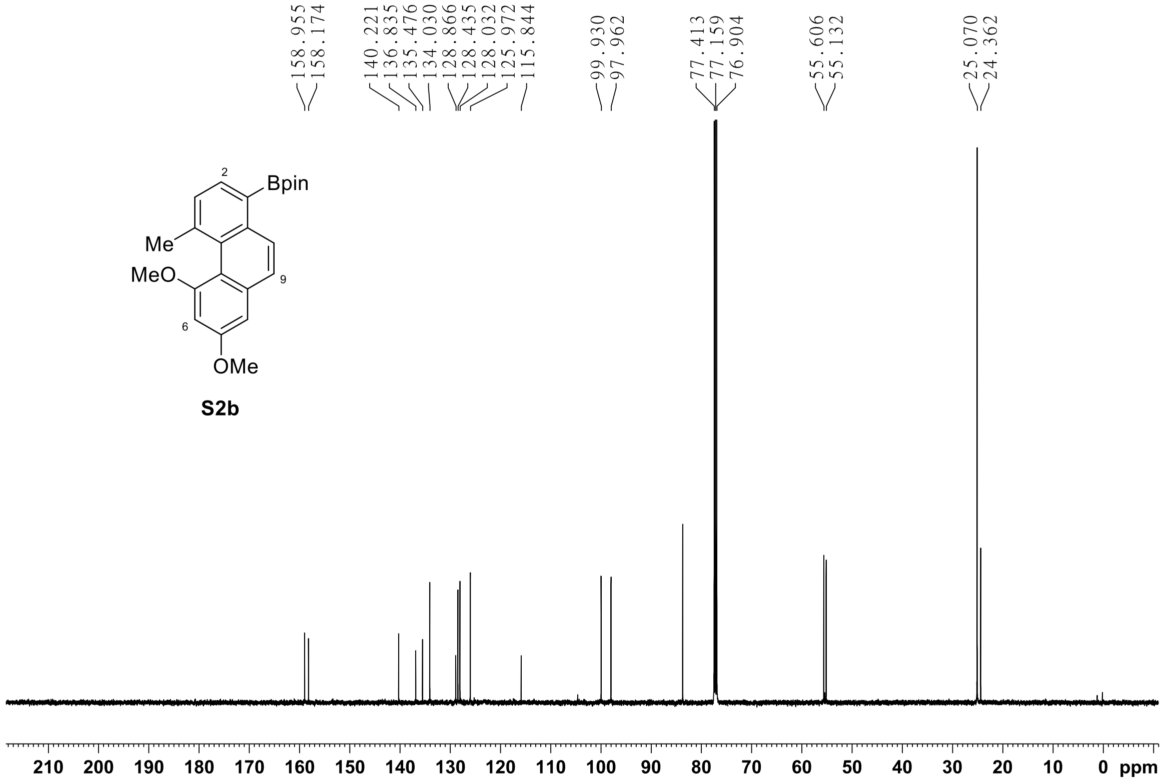

2,5-Bis(5,7-dimethoxy-4-methylphenanthren-1-yl)terephthalaldehyde (S3b):

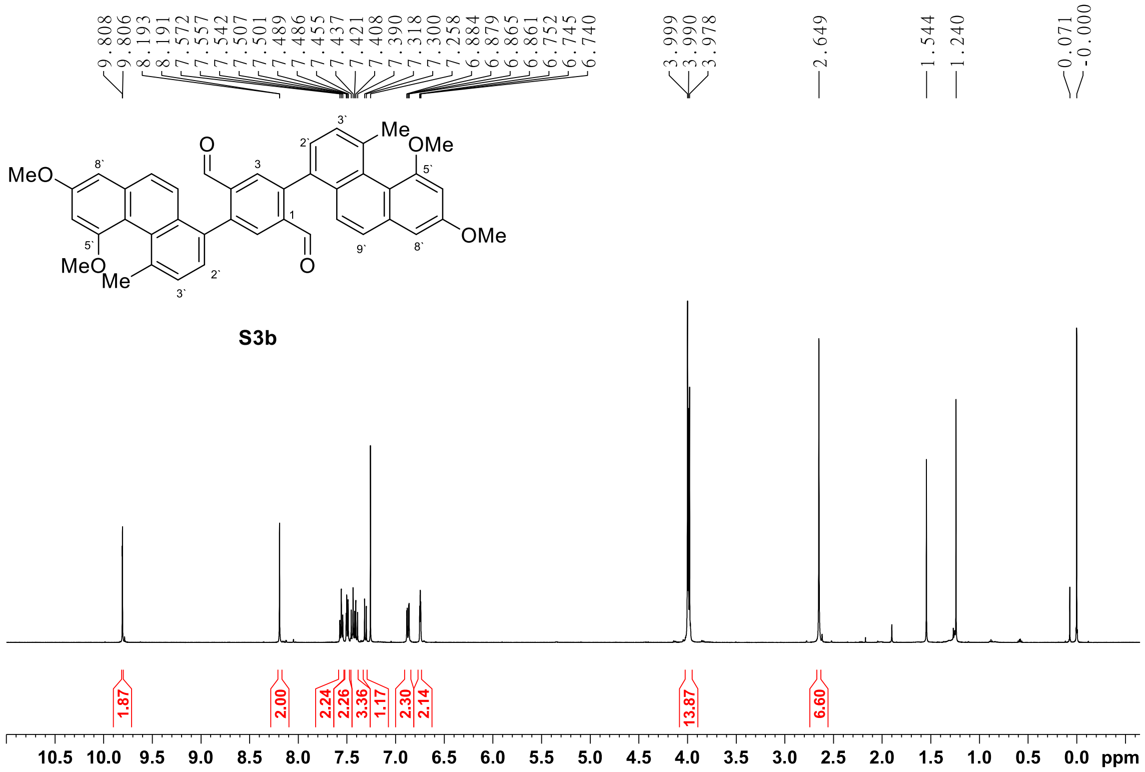

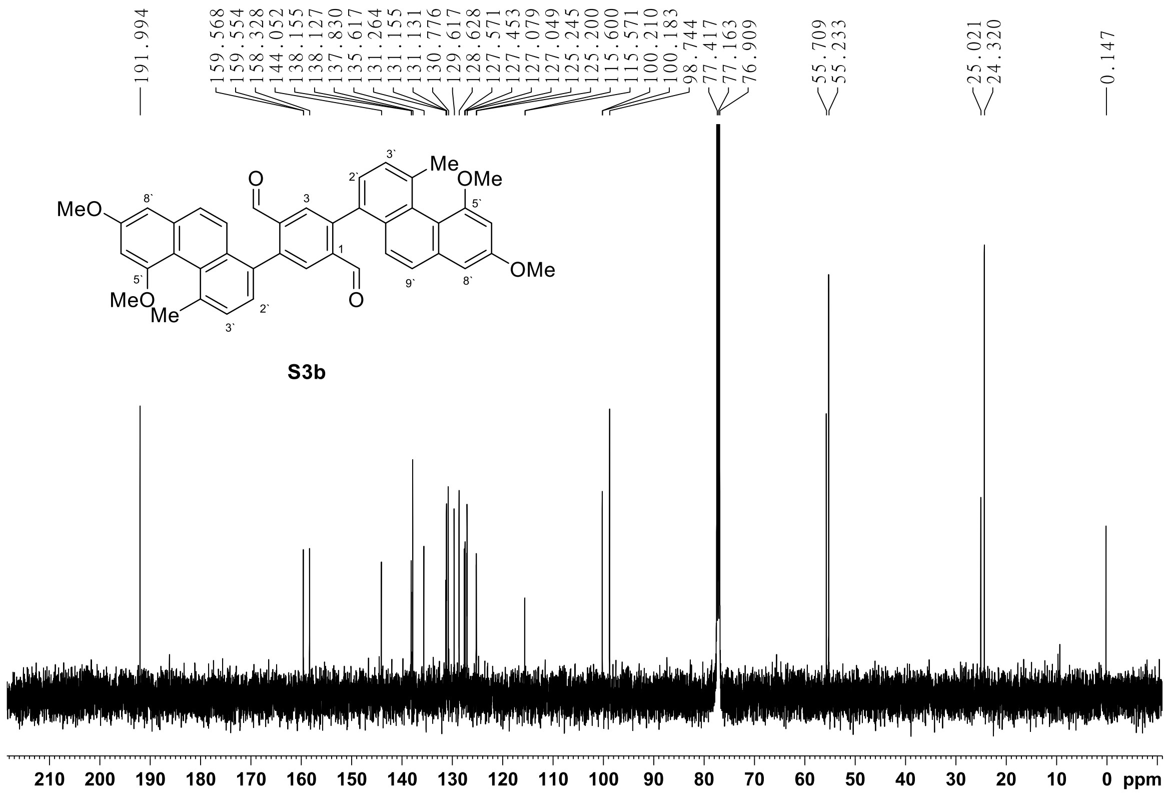

8,8'-(2,5-Divinyl-1,4-phenylene)bis(2,4-dimethoxy-5-methylphenanthrene) (7b):

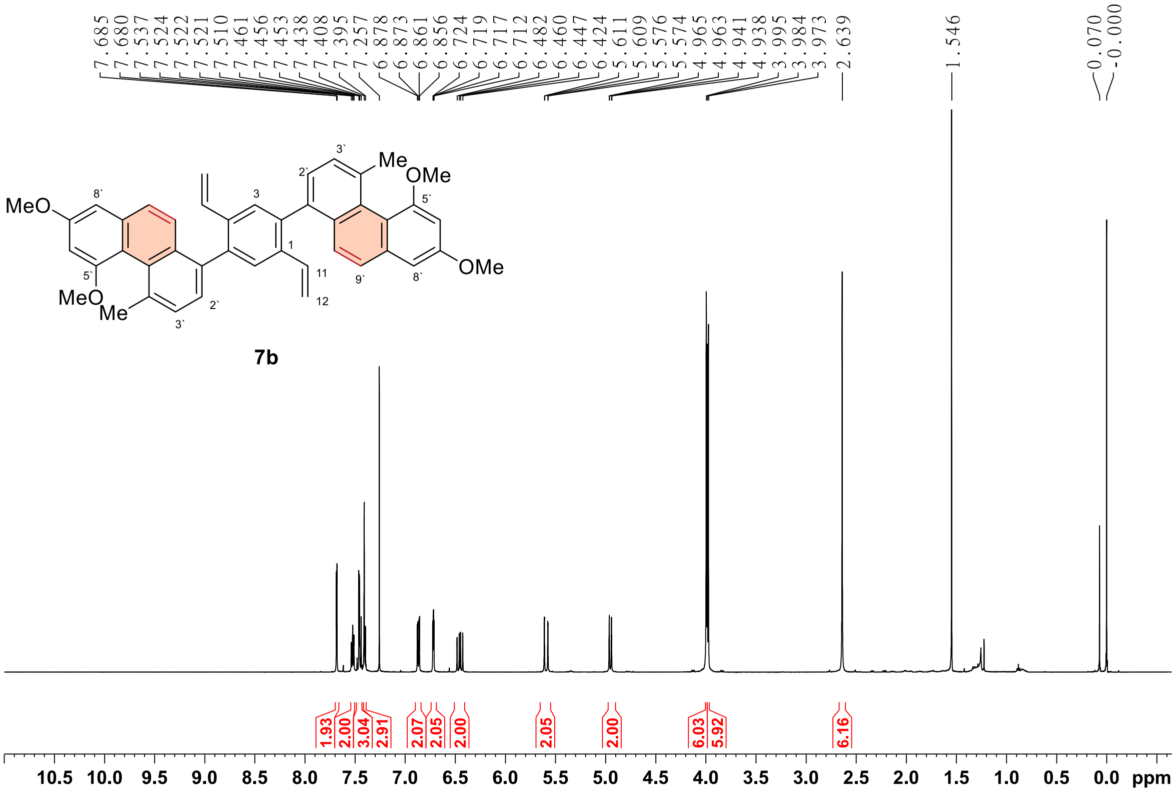

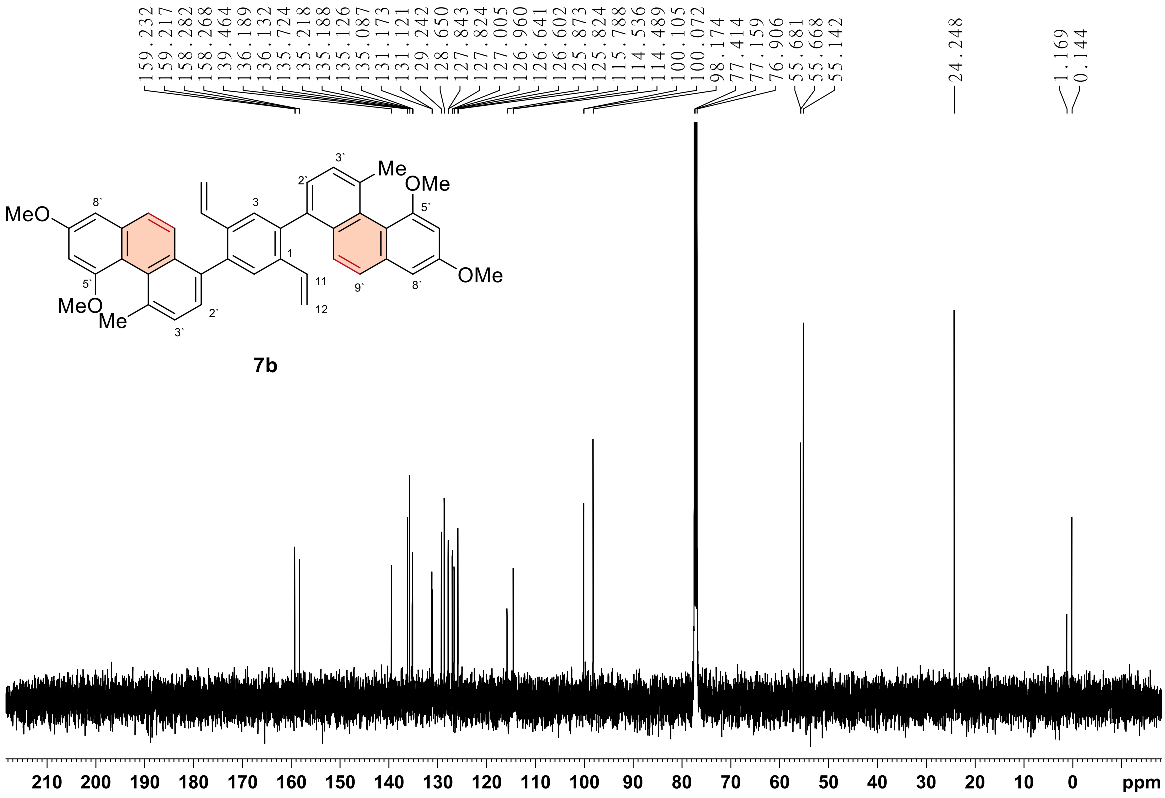

4,6-Bis(5,7-dimethoxy-4-methylphenanthren-1-yl)isophthalaldehyde (S3c):

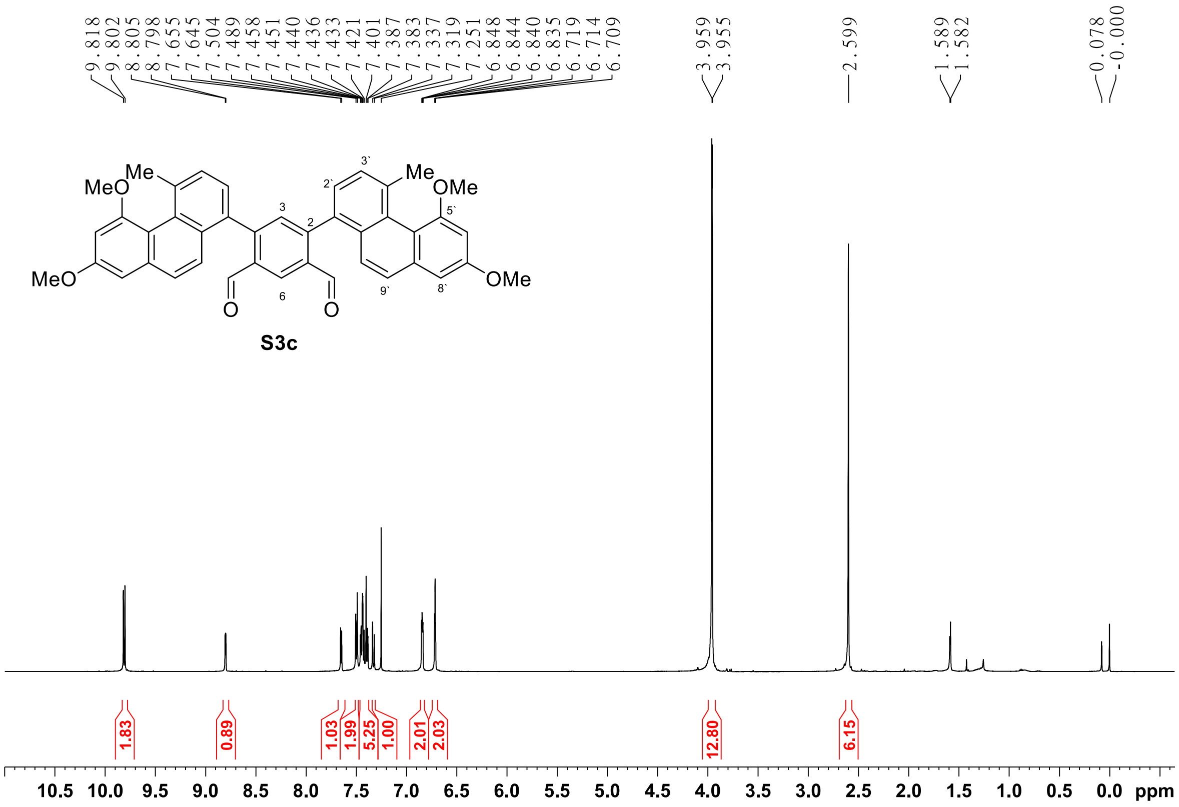

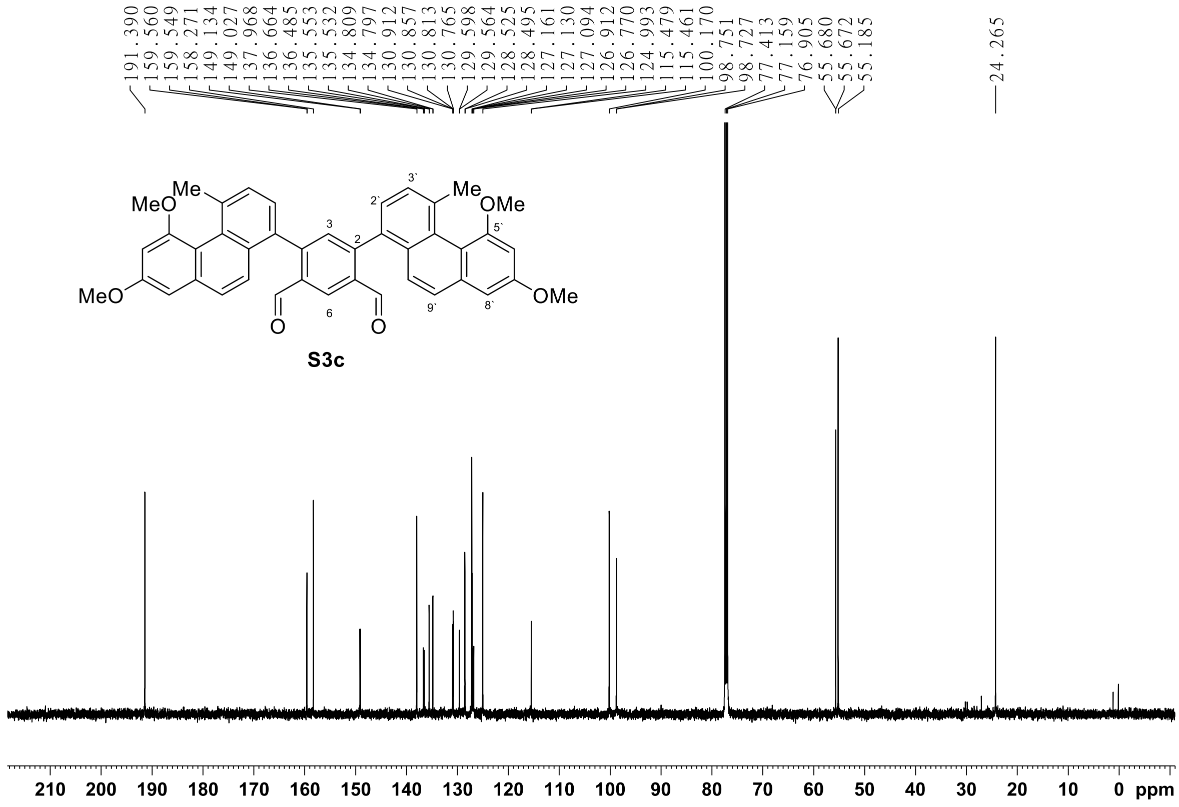

8,8'-(4,6-Divinyl-1,3-phenylene)bis(2,4-dimethoxy-5-methylphenanthrene) (7c):

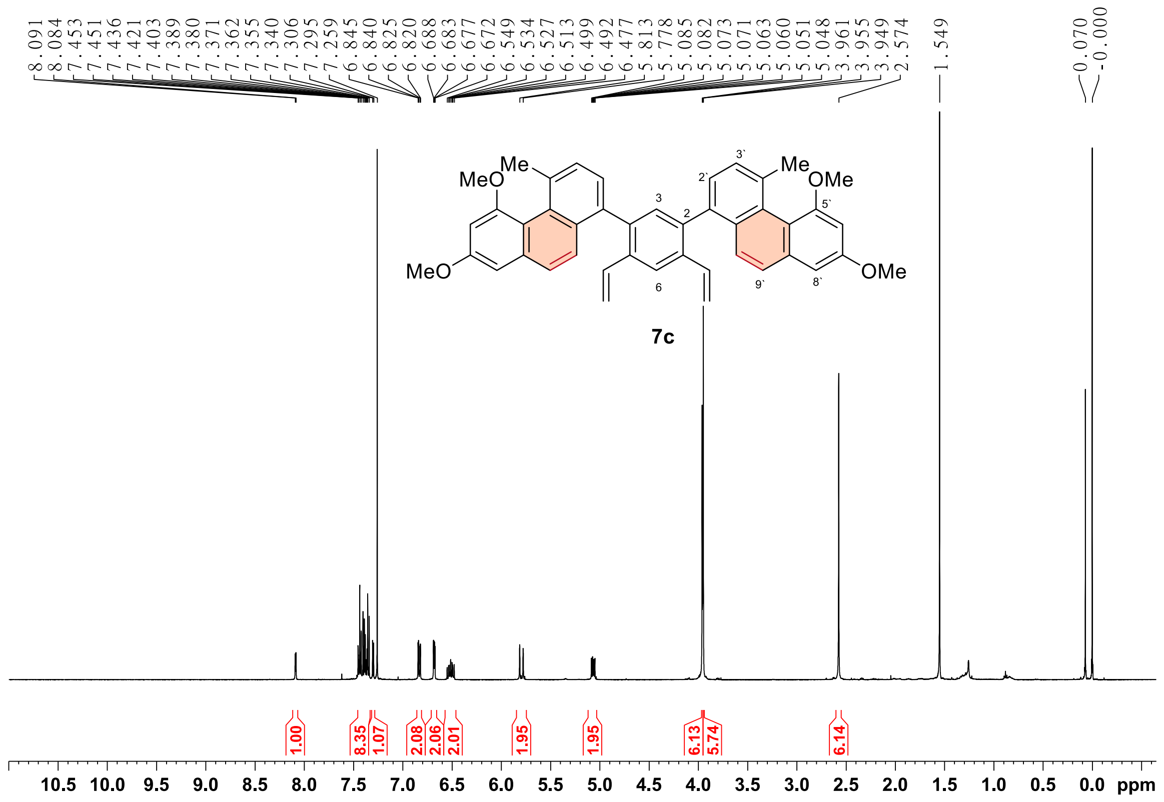

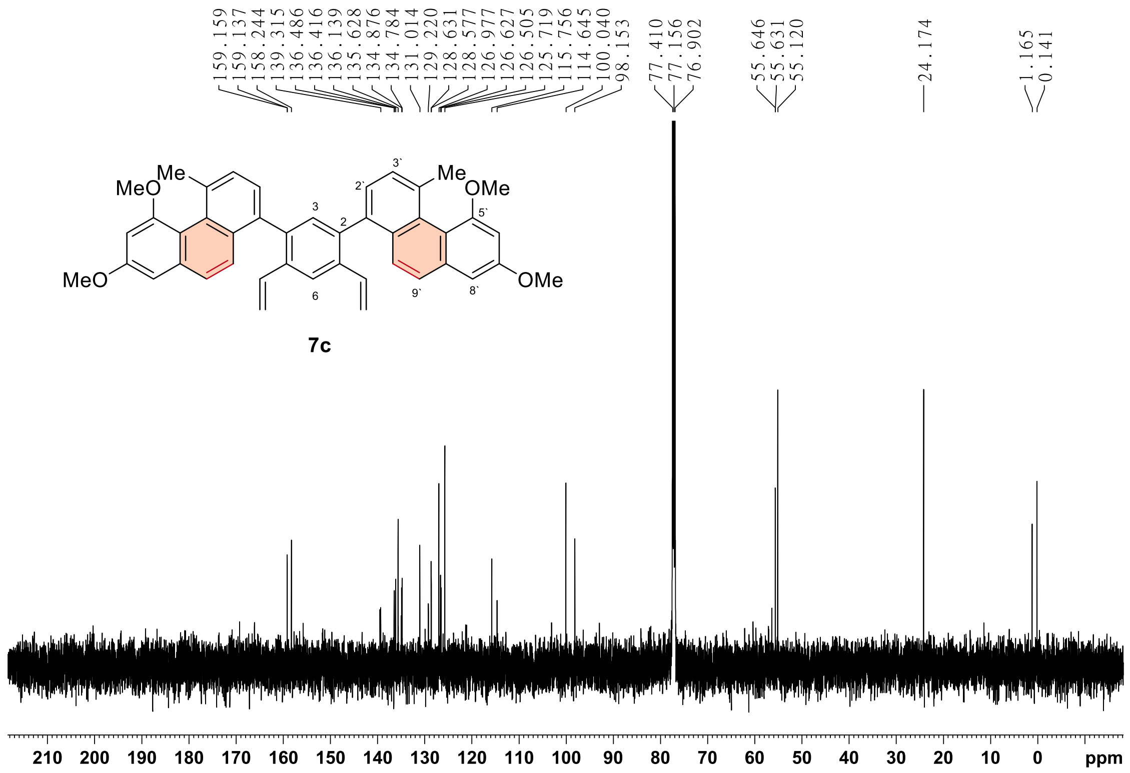

3,7-Bis(9,11-dimethylphenanthro[3,4-d][1,3]dioxol-5-yl)naphthalene-2,6-dicarbaldehyde (S3d):

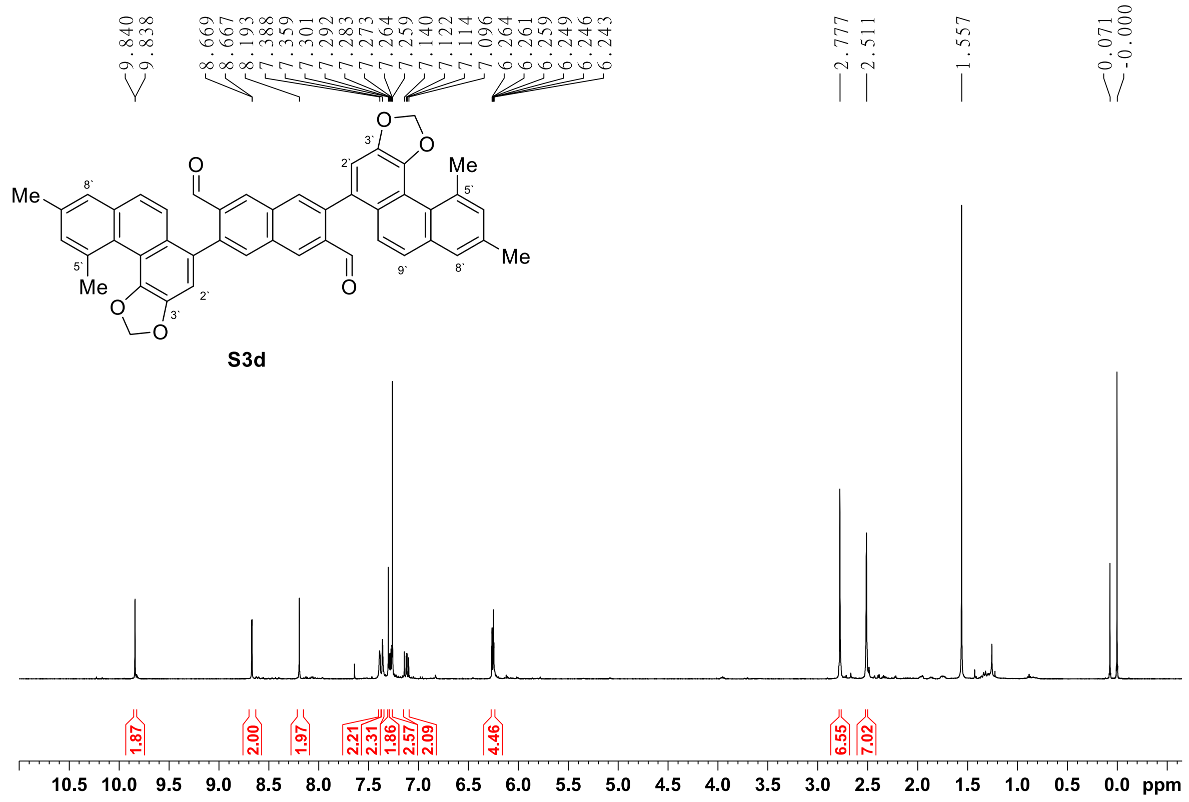

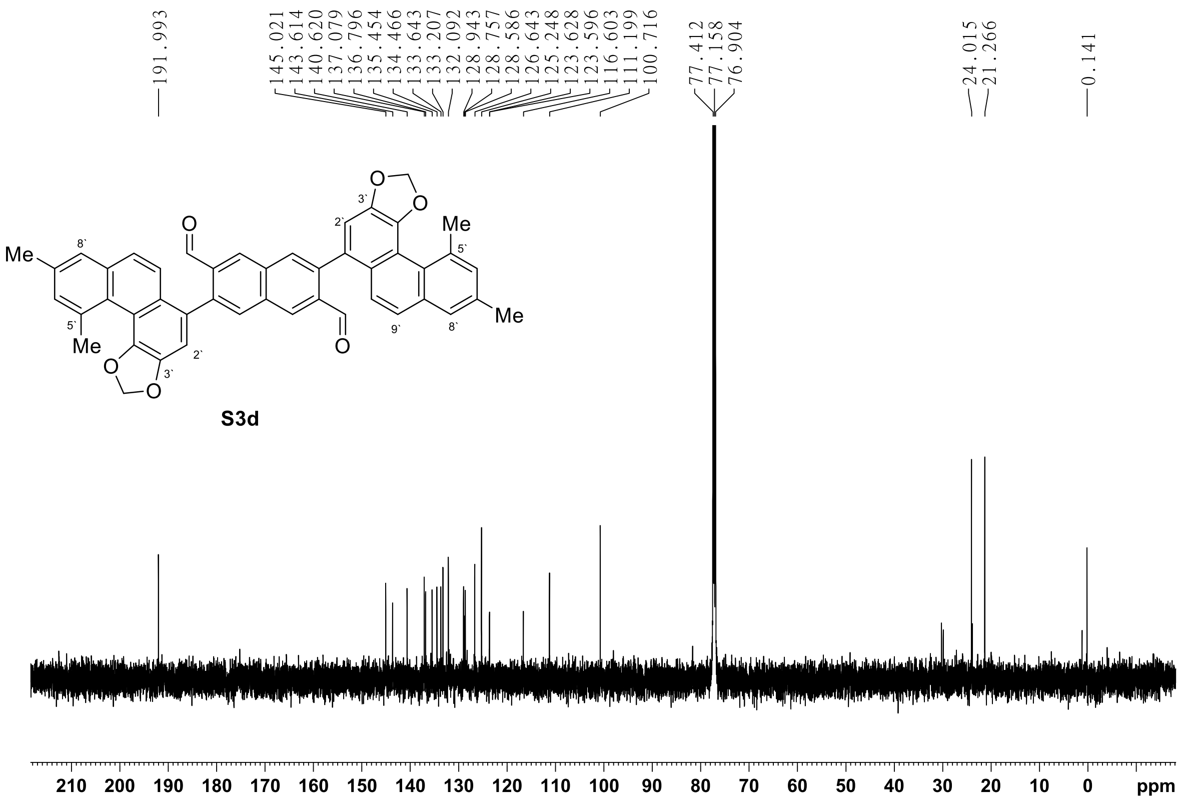

5,5'-(3,7-Divinylnaphthalene-2,6-diyl)bis(9,11-dimethylphenanthro[3,4-*d*][1,3]dioxole) (7d):

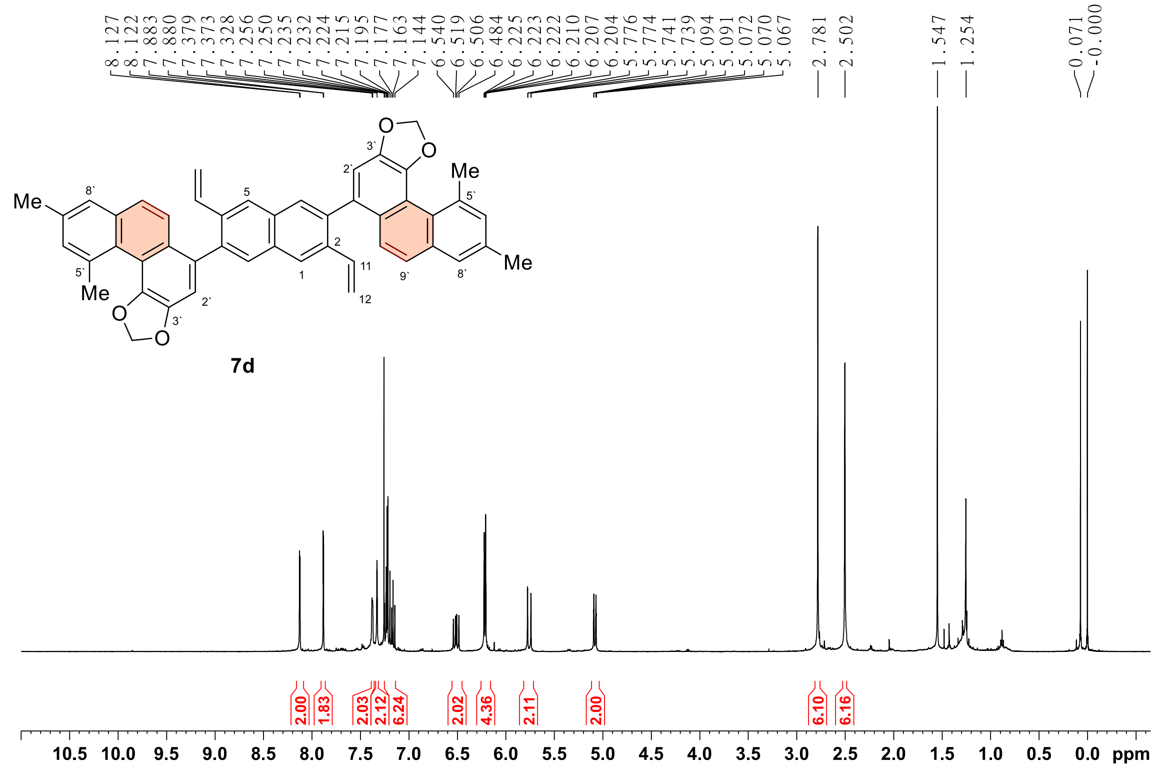

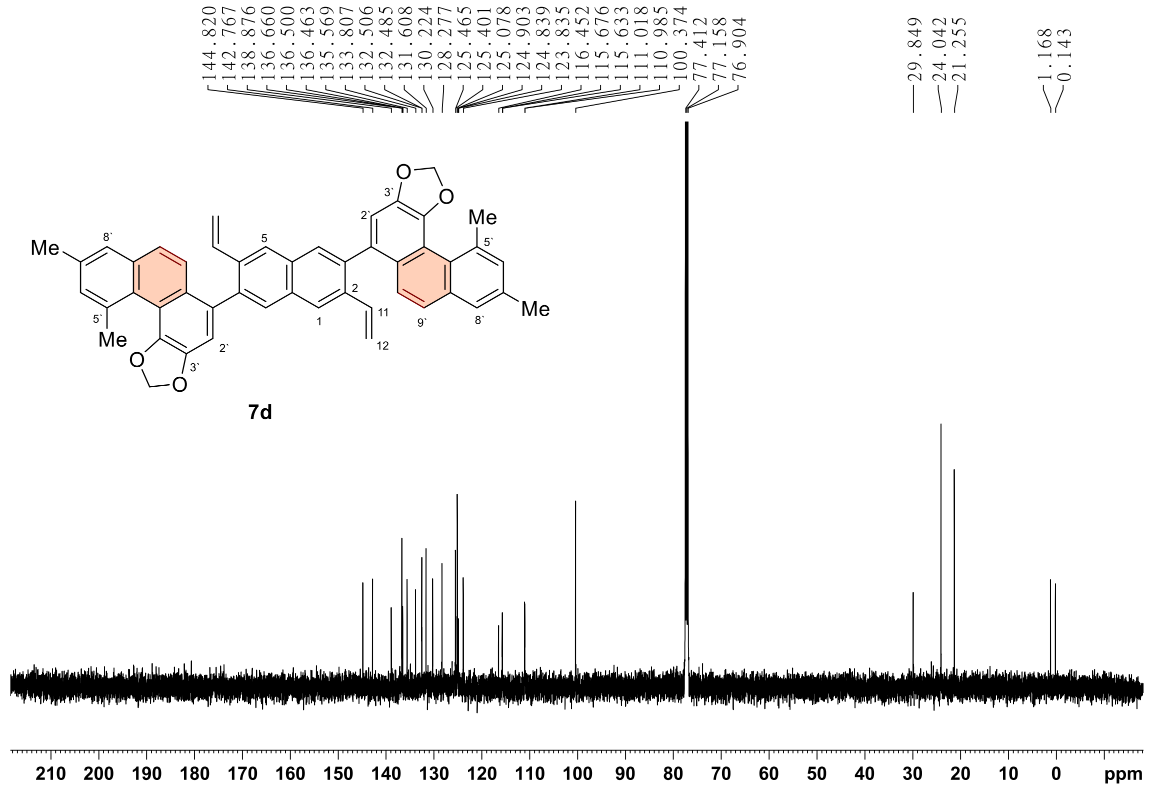

5-Methoxy-1-(2-vinylphenyl)-1*H*-indole (9a):

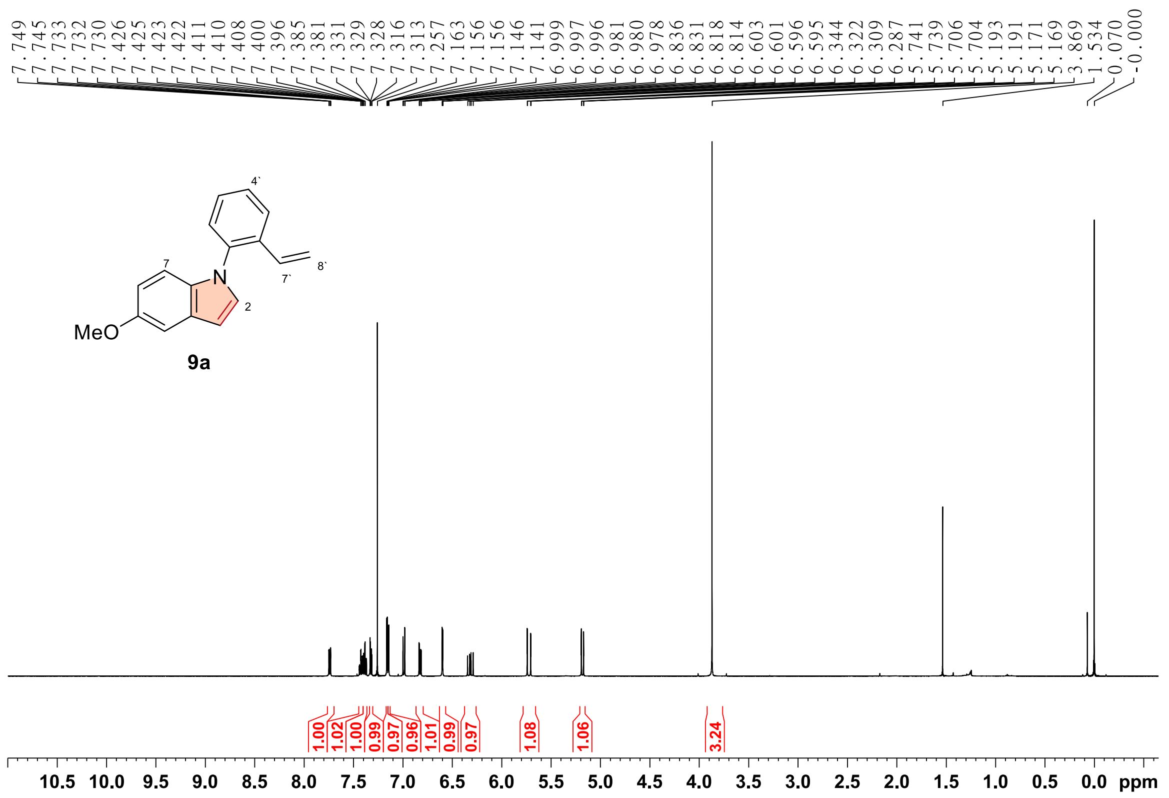

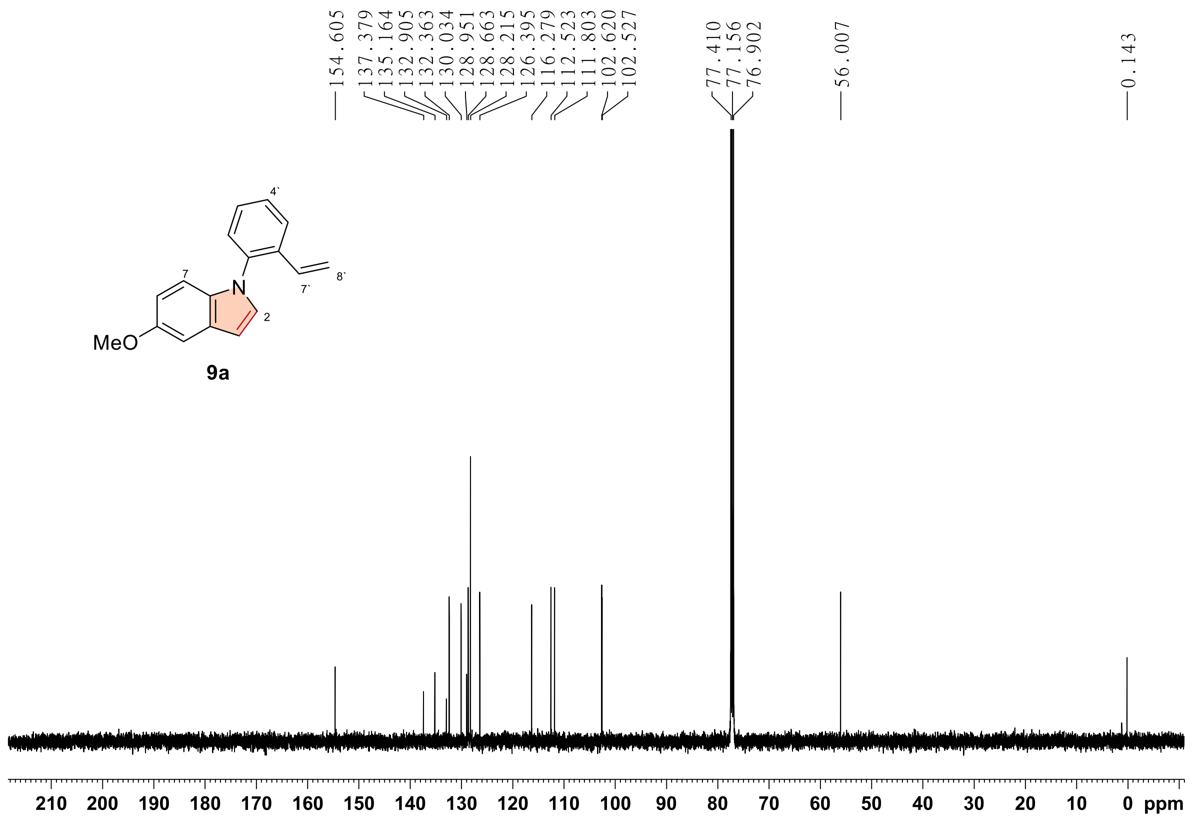

5-Methyl-1-(2-vinylphenyl)-1*H*-indole (9b):

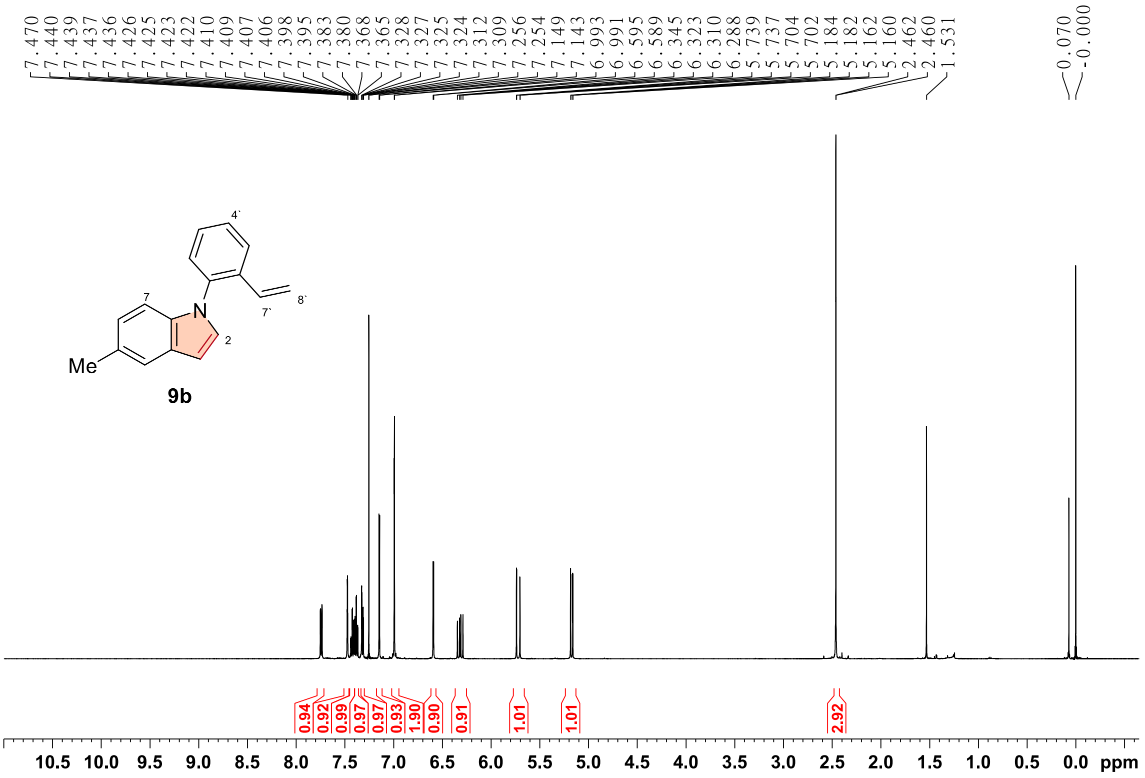

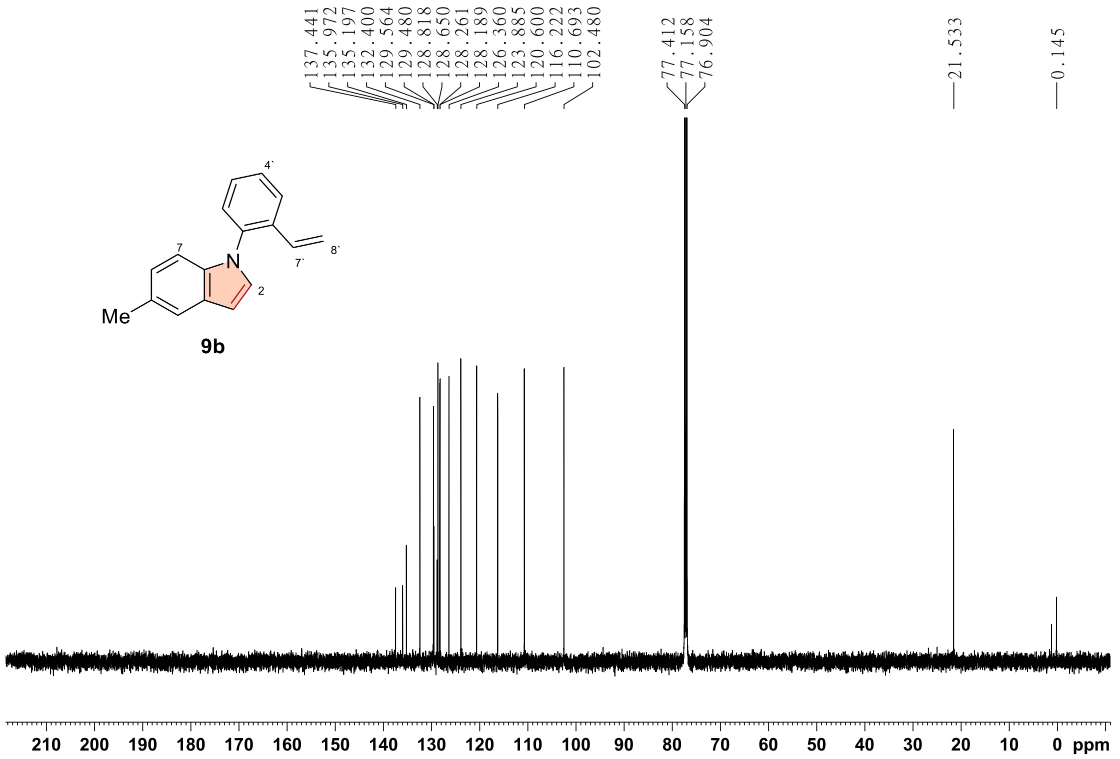

7-Fluoro-1-(2-vinylphenyl)-1*H*-indole (9c):

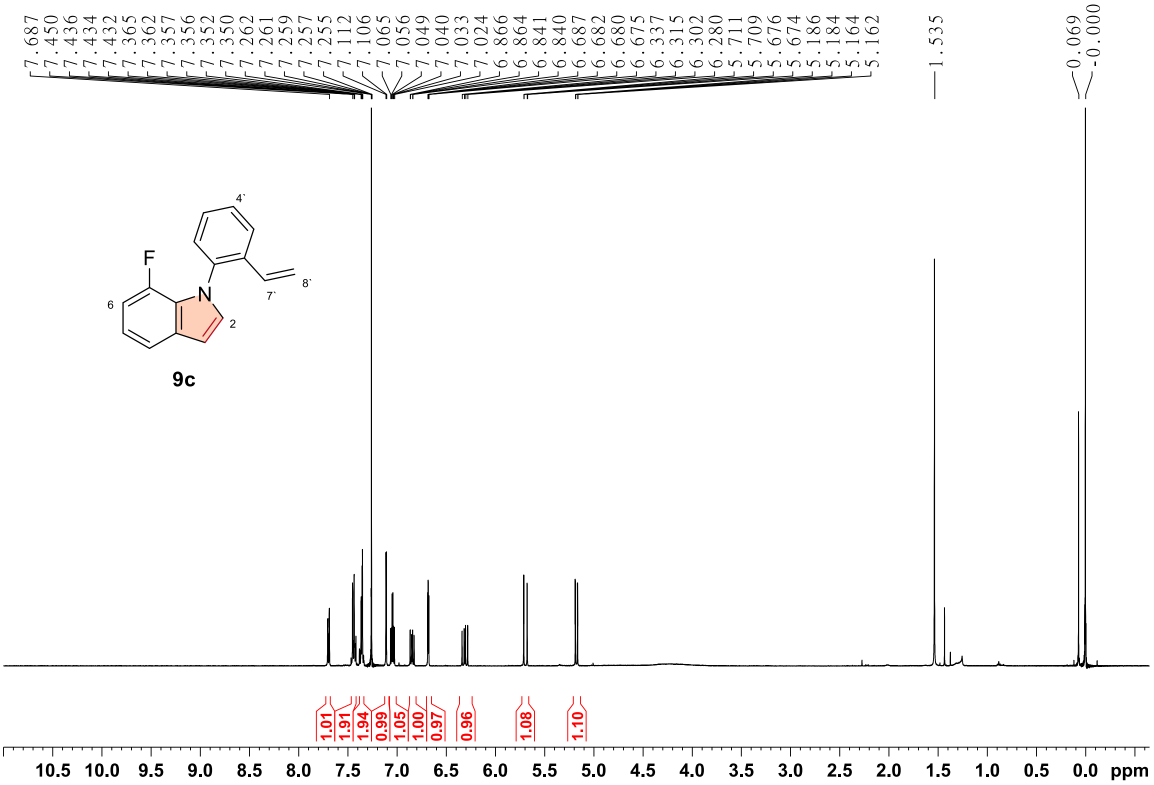

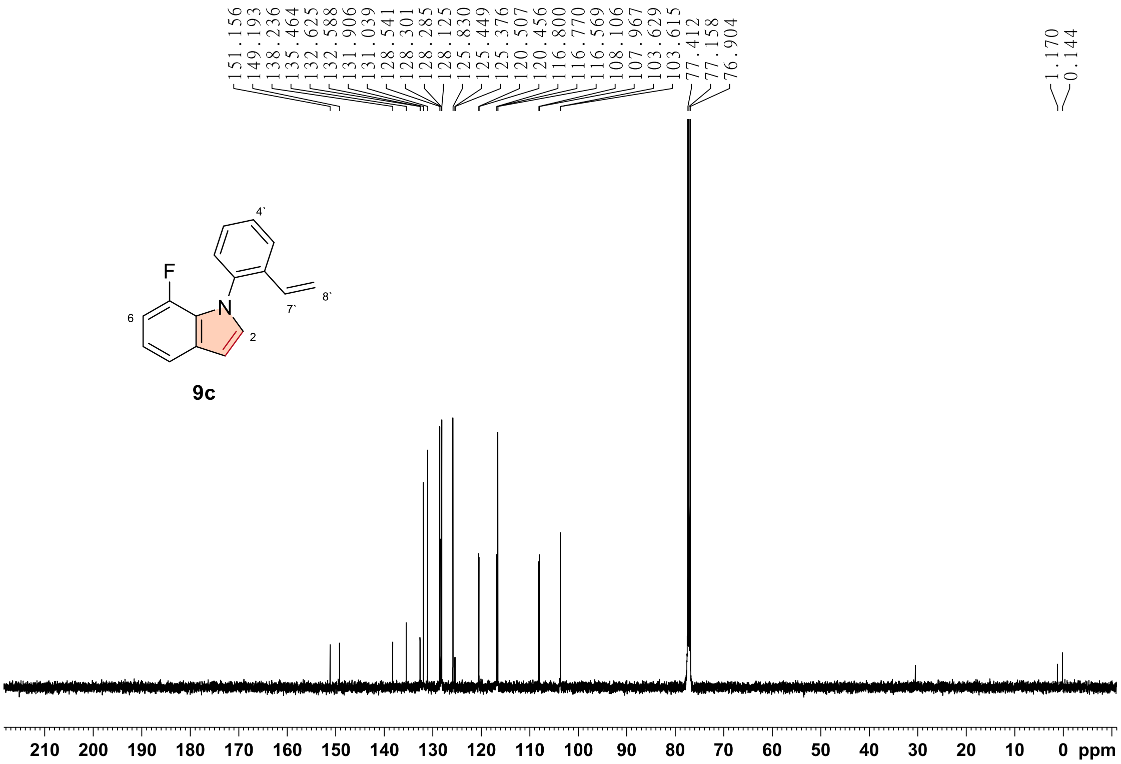

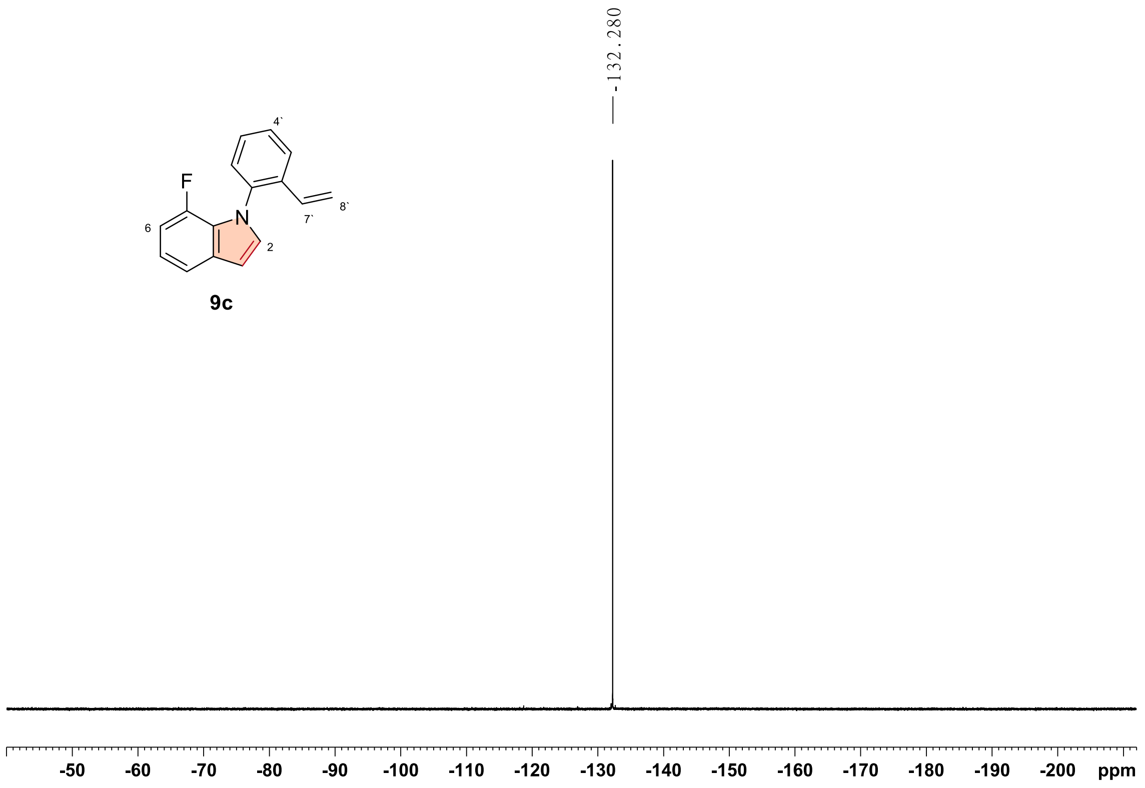
F
N
4`
6
2
7`
8`
-132.280
-50
-60
-70
-80
-90
-100
-110
-120
-130
-140
-150
-160
-170
-180
-190
-200
ppm

9c

5-Fluoro-1-(2-vinylphenyl)-*1H*-indole (9d):

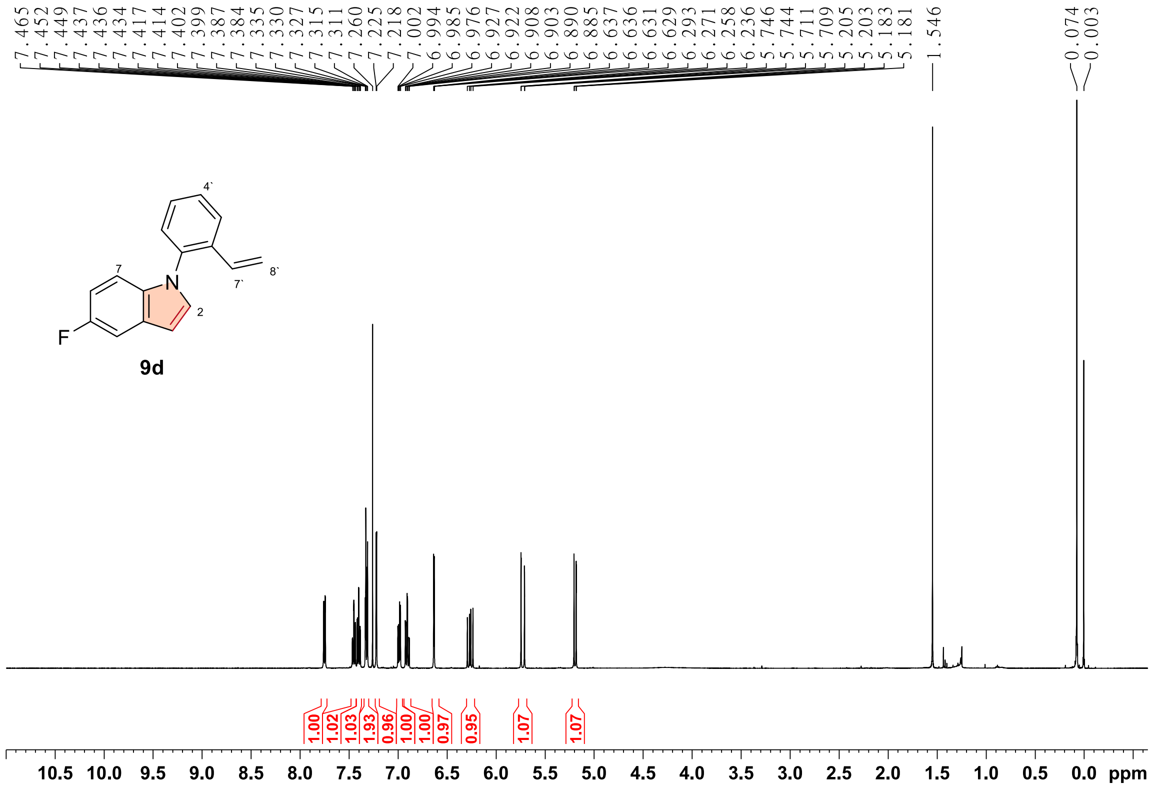

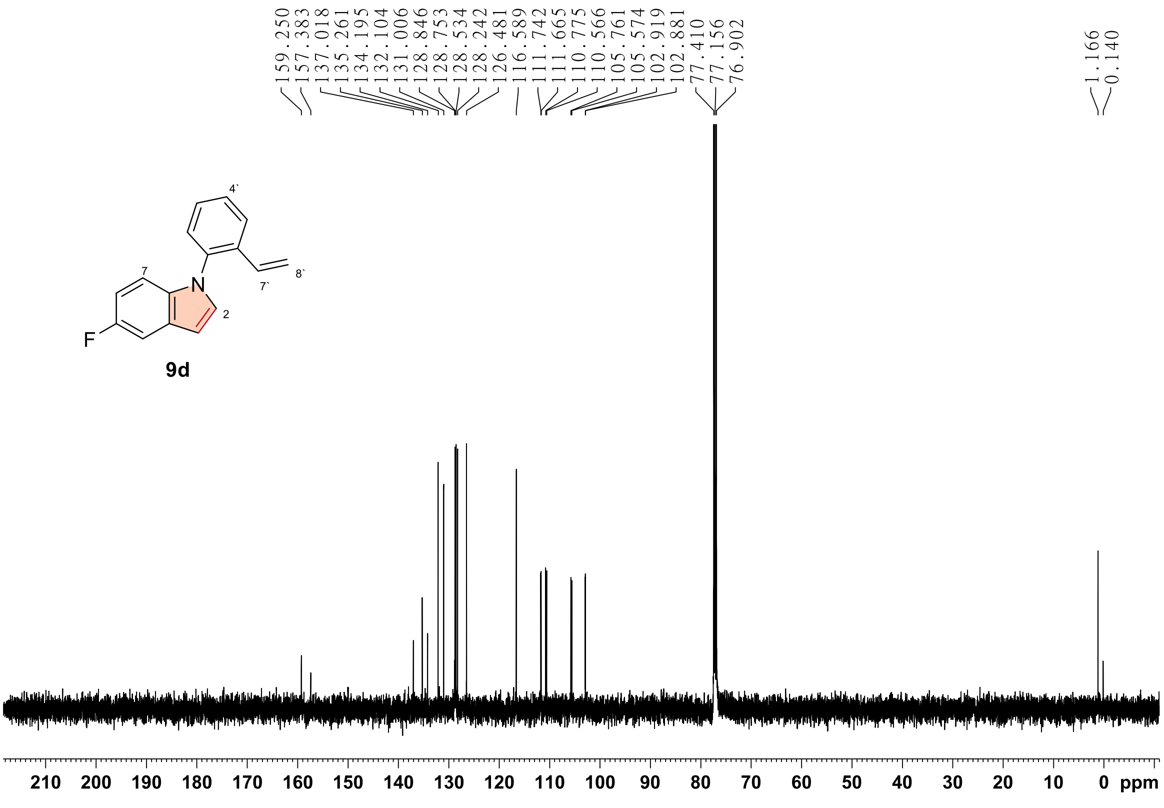

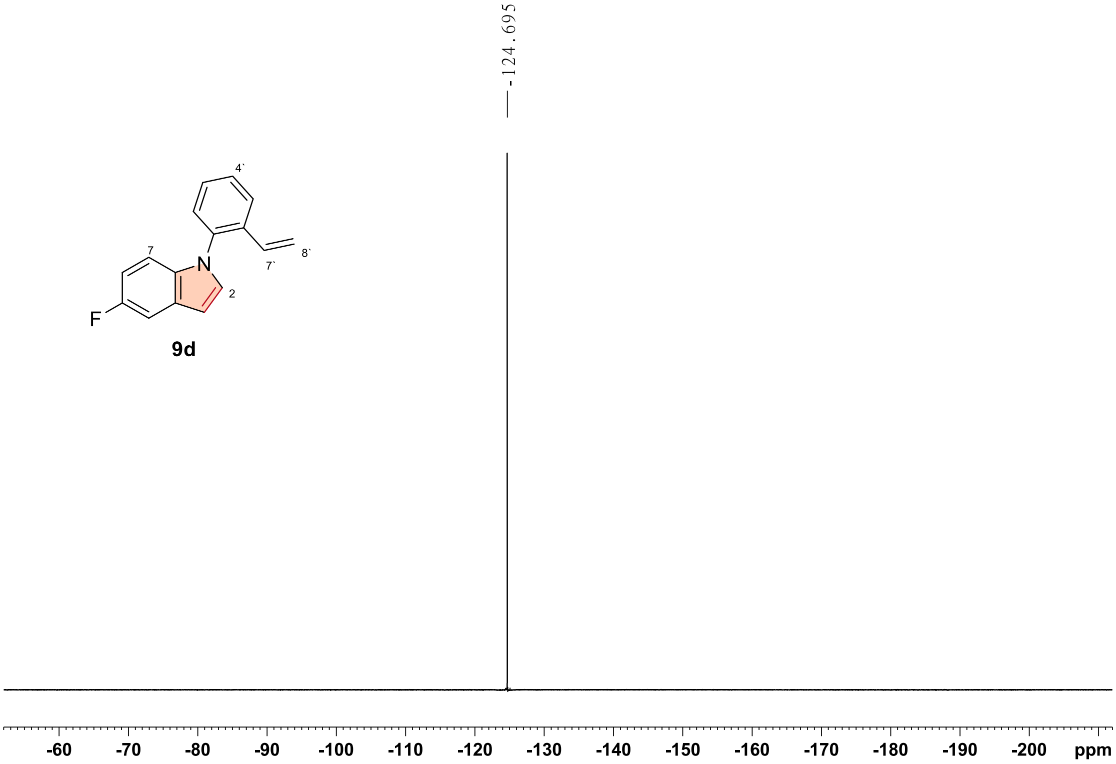
-124.695
4`
7
N
7`
8`
2
F
9d
-60
-70
-80
-90
-100
-110
-120
-130
-140
-150
-160
-170
-180
-190
-200
ppm

6-Fluoro-1-(2-vinylphenyl)-1*H*-indole (9e):

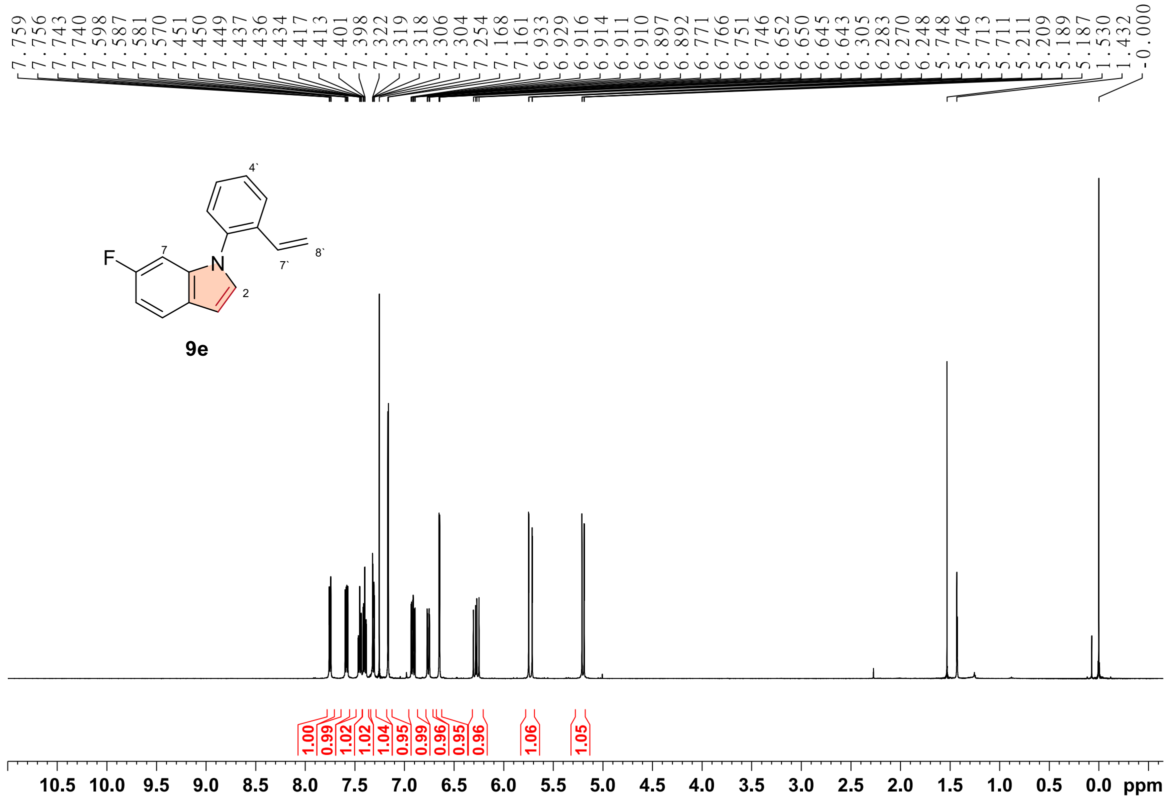

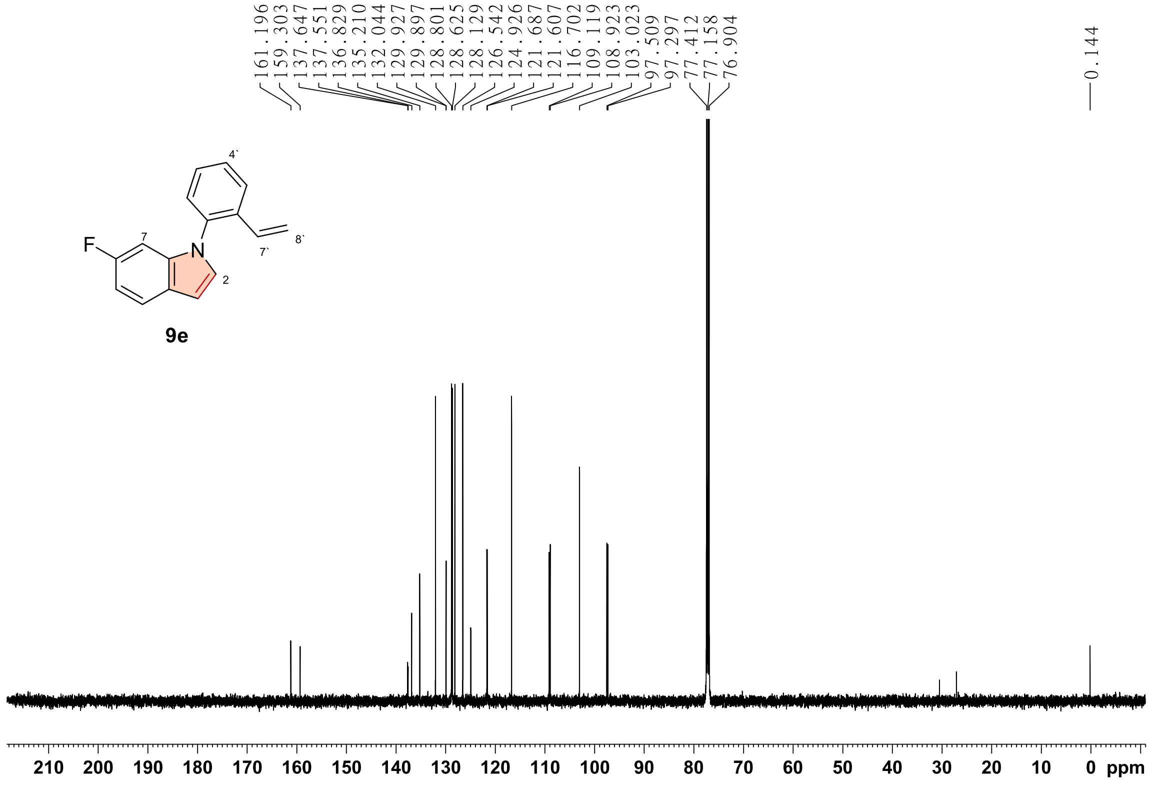

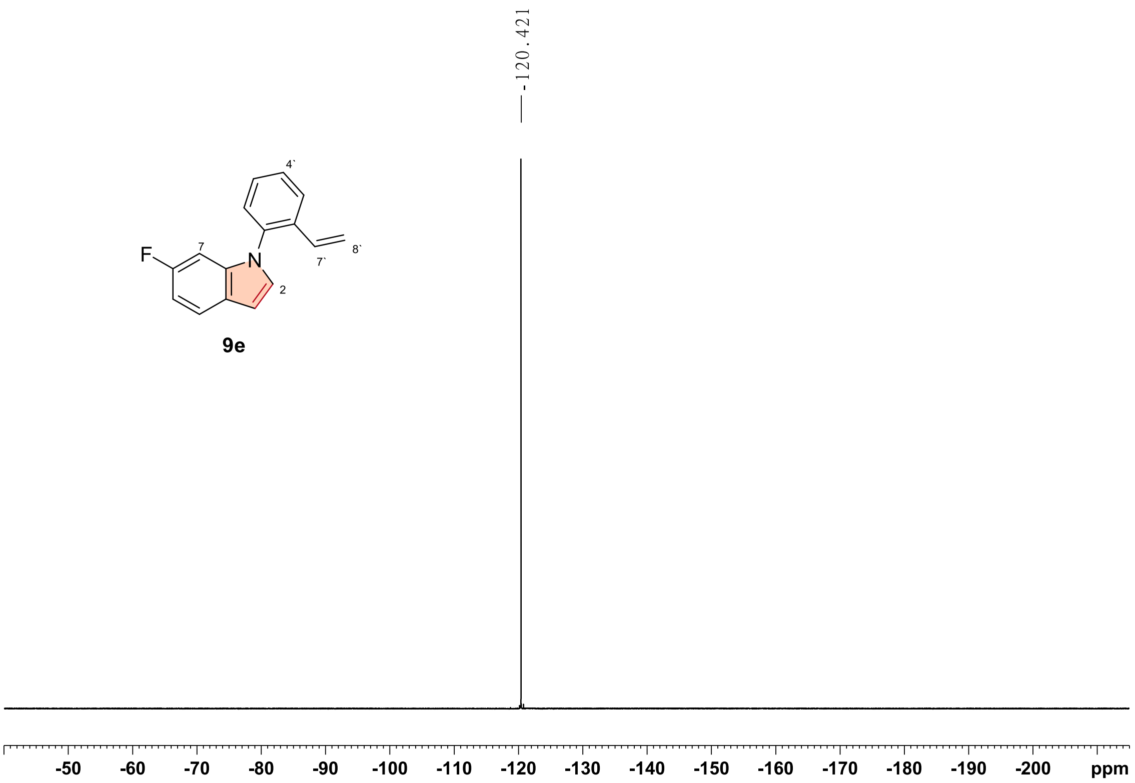
-120.421
F
N
7
2
4`
7`
8`
9e
-50 -60 -70 -80 -90 -100 -110 -120 -130 -140 -150 -160 -170 -180 -190 -200 ppm

5-Chloro-1-(2-vinylphenyl)-1*H*-indole (9f):

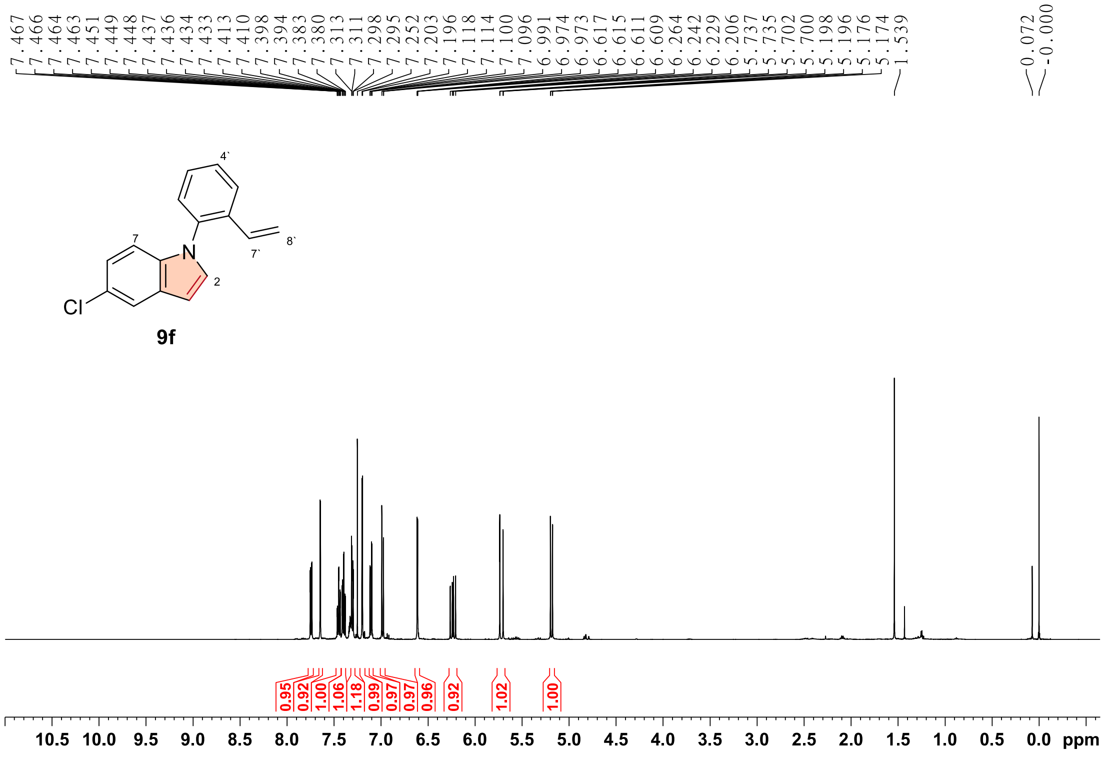

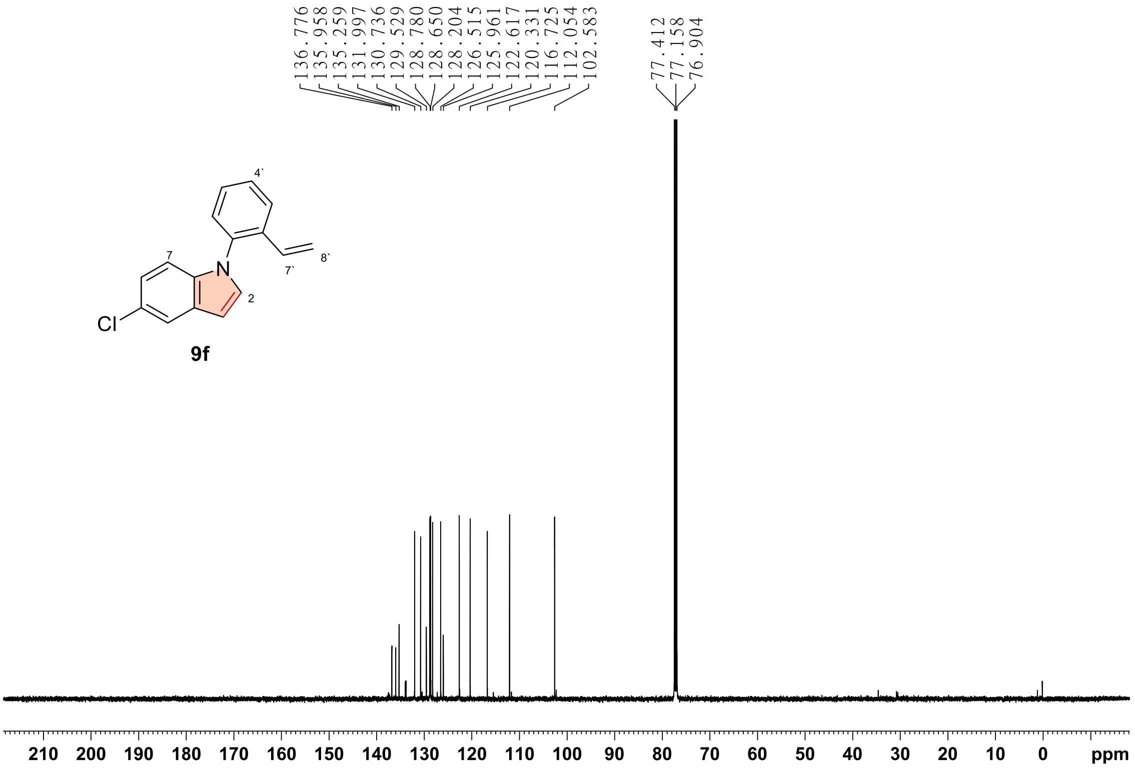

6-Chloro-1-(2-vinylphenyl)-1*H*-indole (9g):

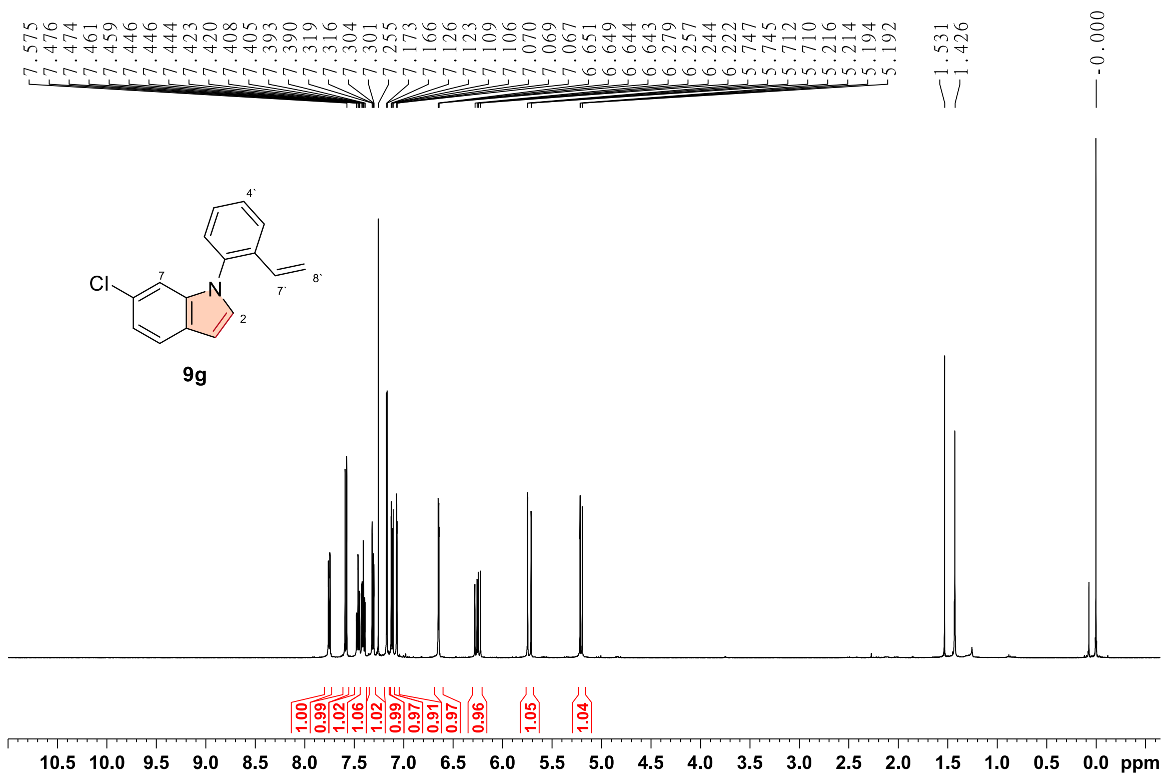

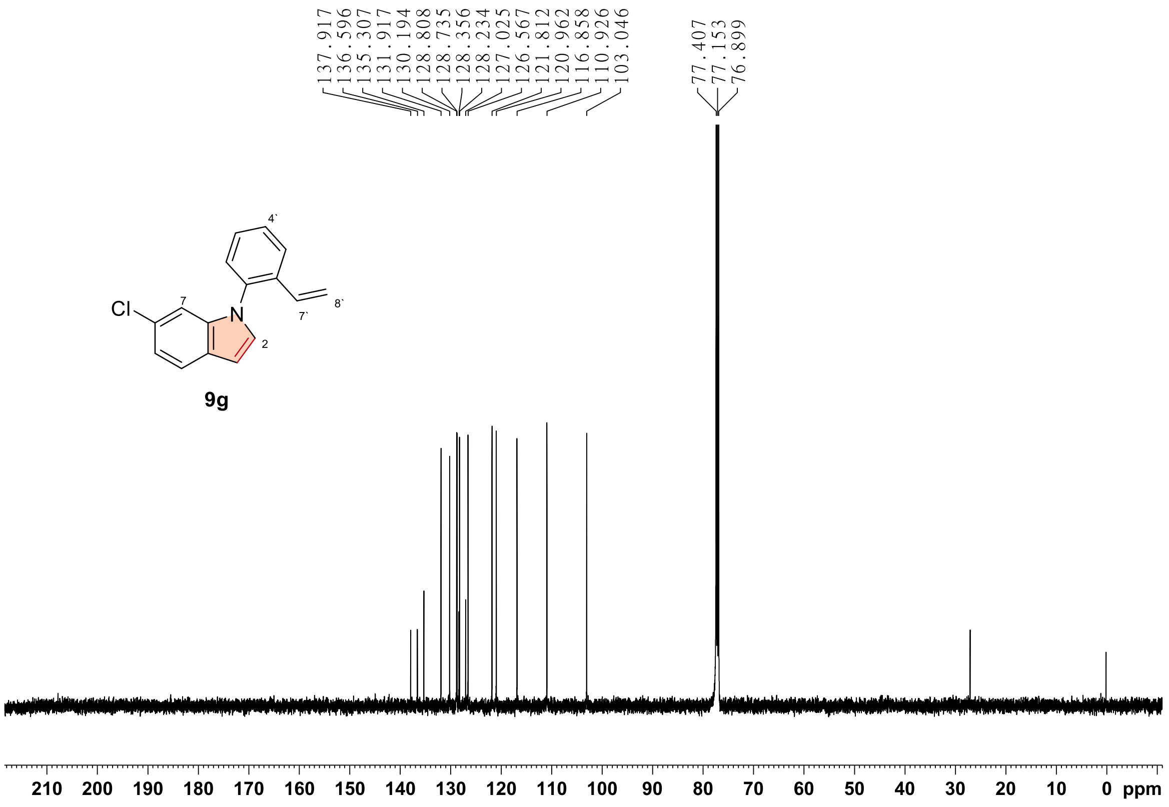

5-(Trifluoromethyl)-1-(2-vinylphenyl)-1*H*-indole (9h):

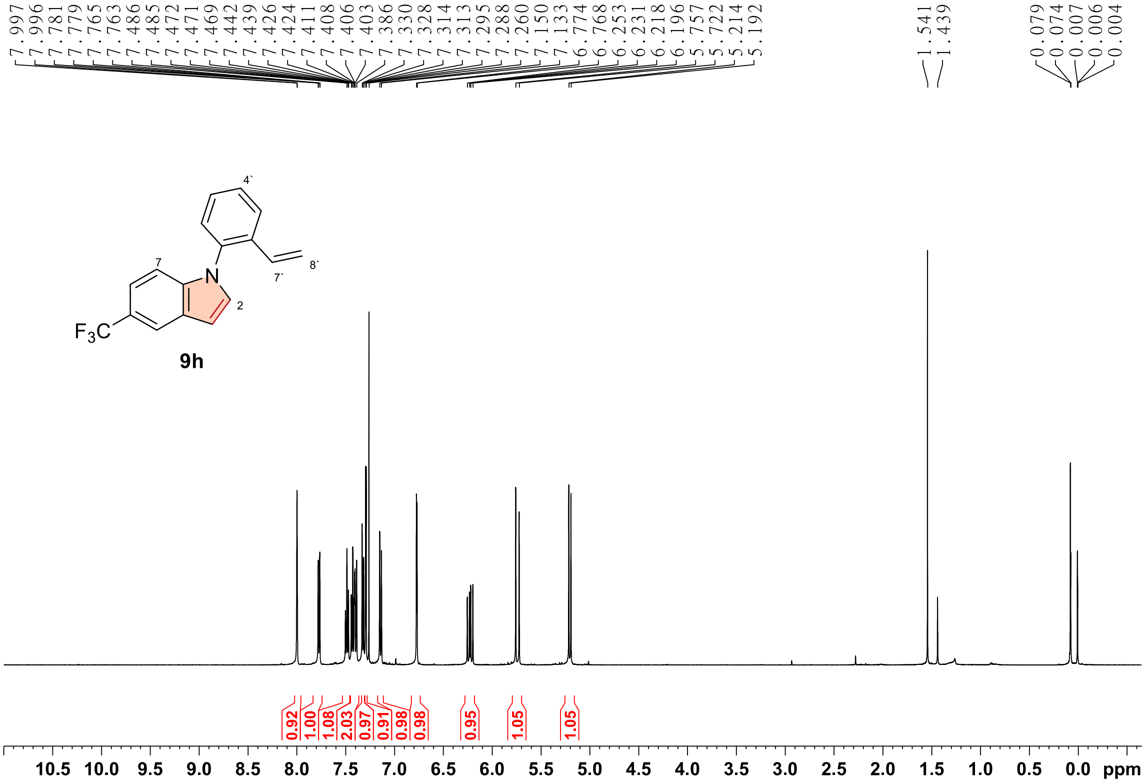

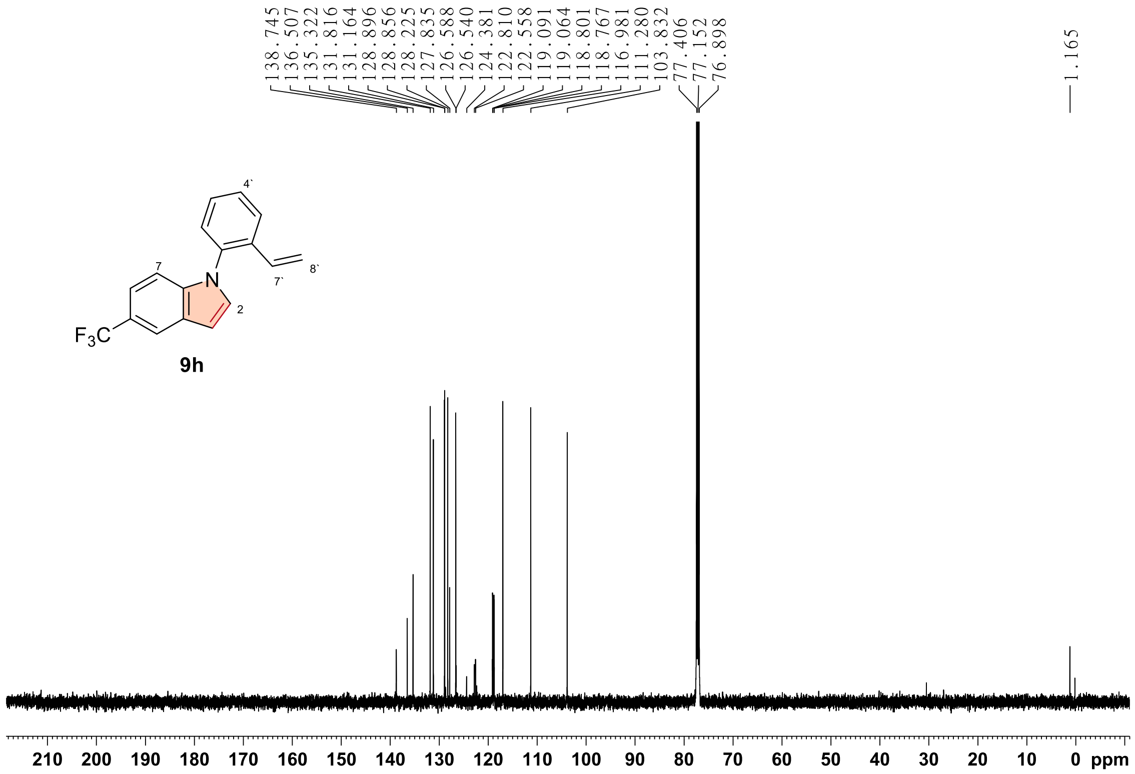

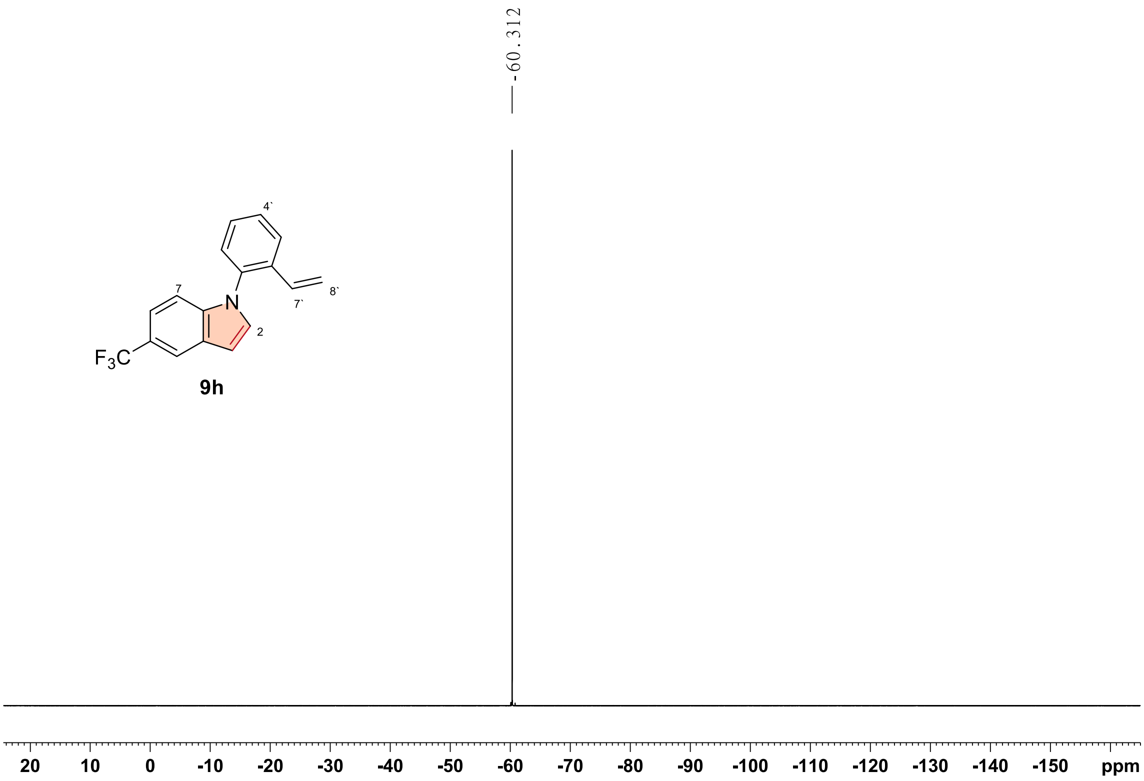
-60.312
F3C
N
7
2
4`
7`
8`
9h
20
10
0
-10
-20
-30
-40
-50
-60
-70
-80
-90
-100
-110
-120
-130
-140
-150
ppm

Methyl 1-(2-vinylphenyl)-1*H*-indole-5-carboxylate (9i):

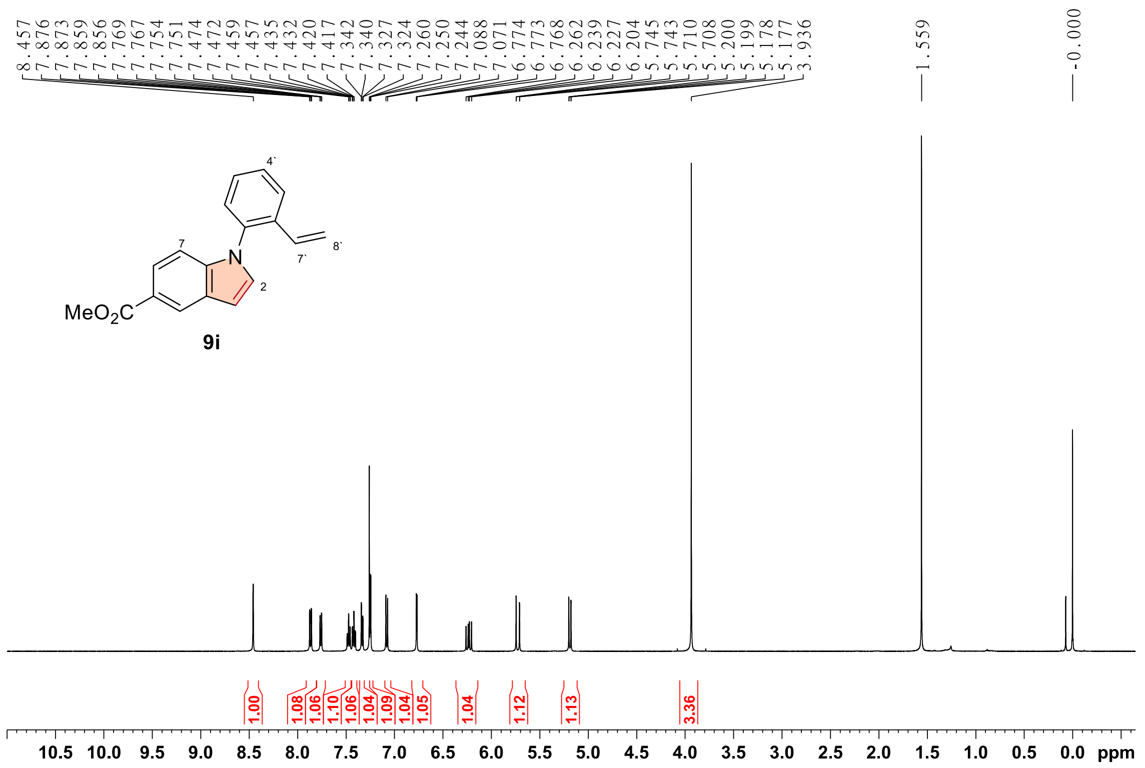

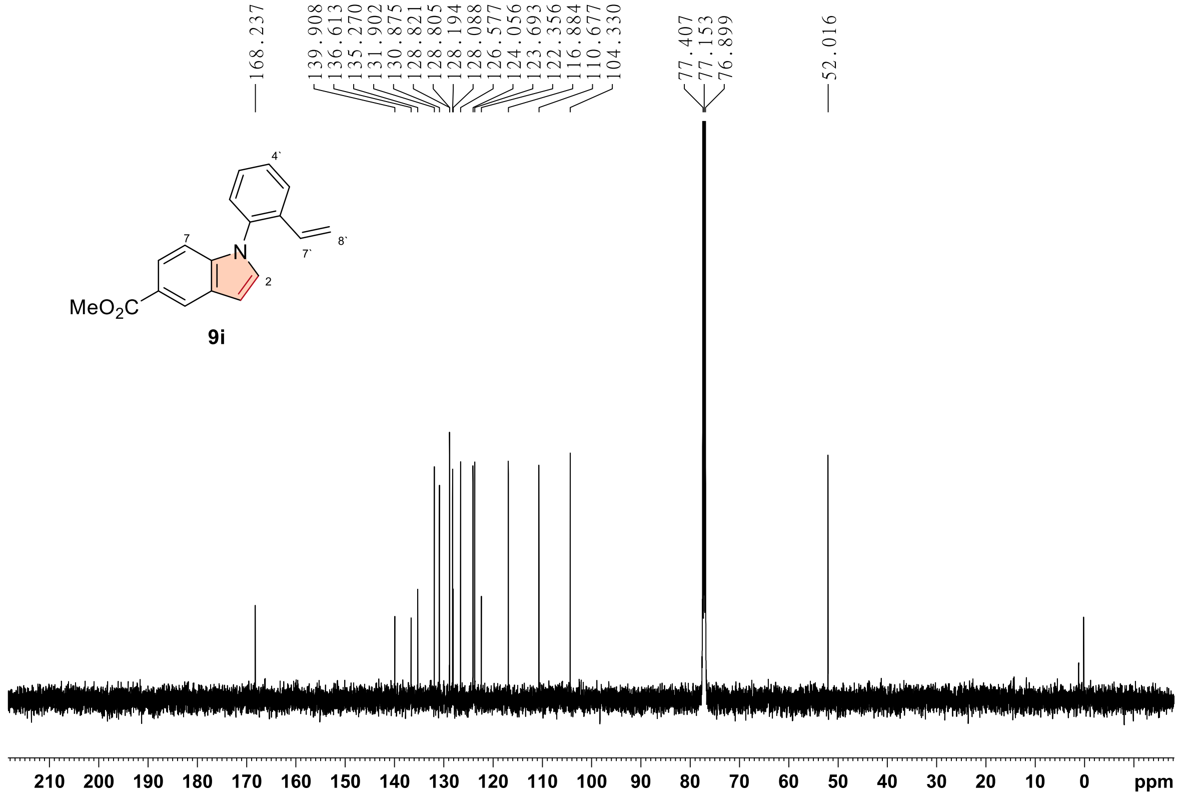

2-(4-(4,4,5,5-tetramethyl-1,3,2-dioxaborolan-2-yl)-1*H*-indol-1-yl)benzaldehyde (S5):

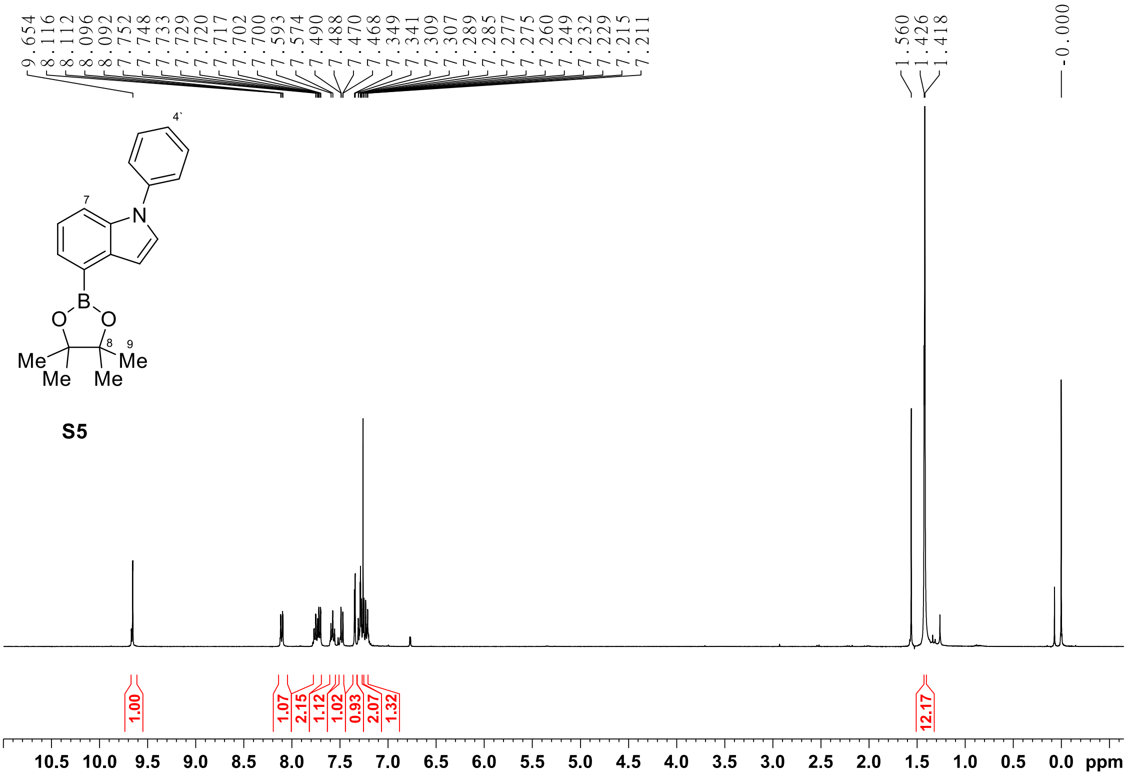

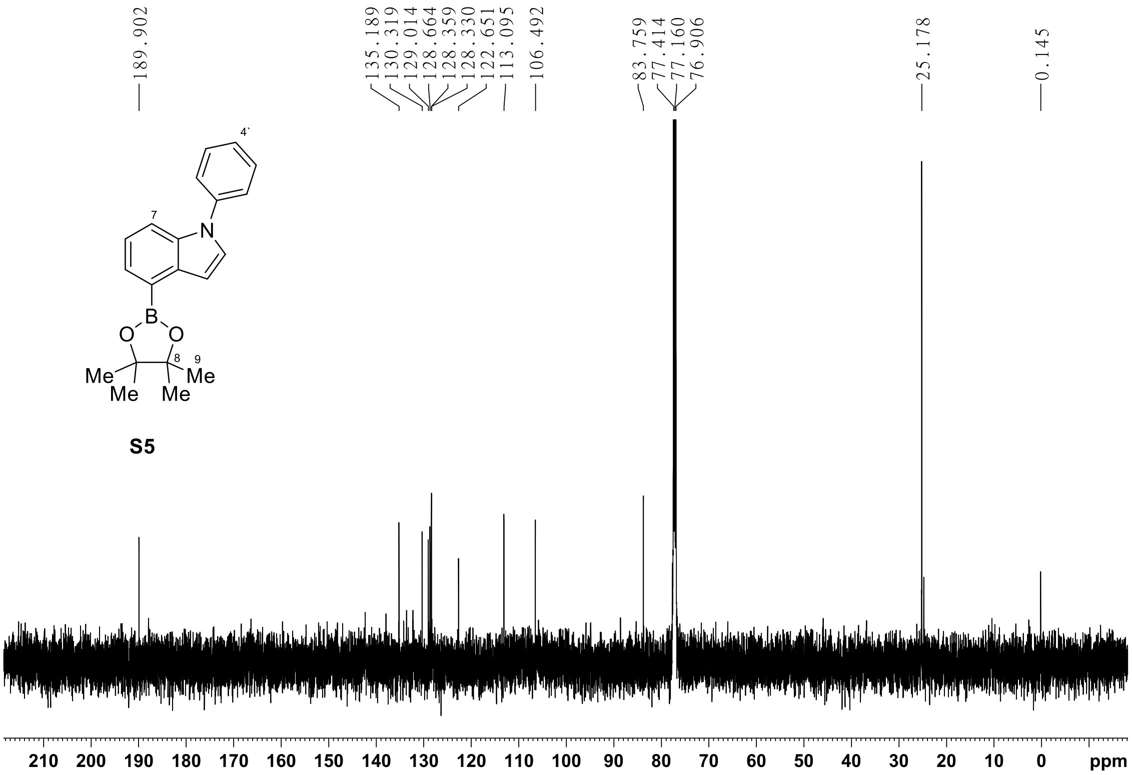

2,2'-(1*H*,1'*H*-[4,4'-biindole]-1,1'-diyl)dibenzaldehyde (S6):

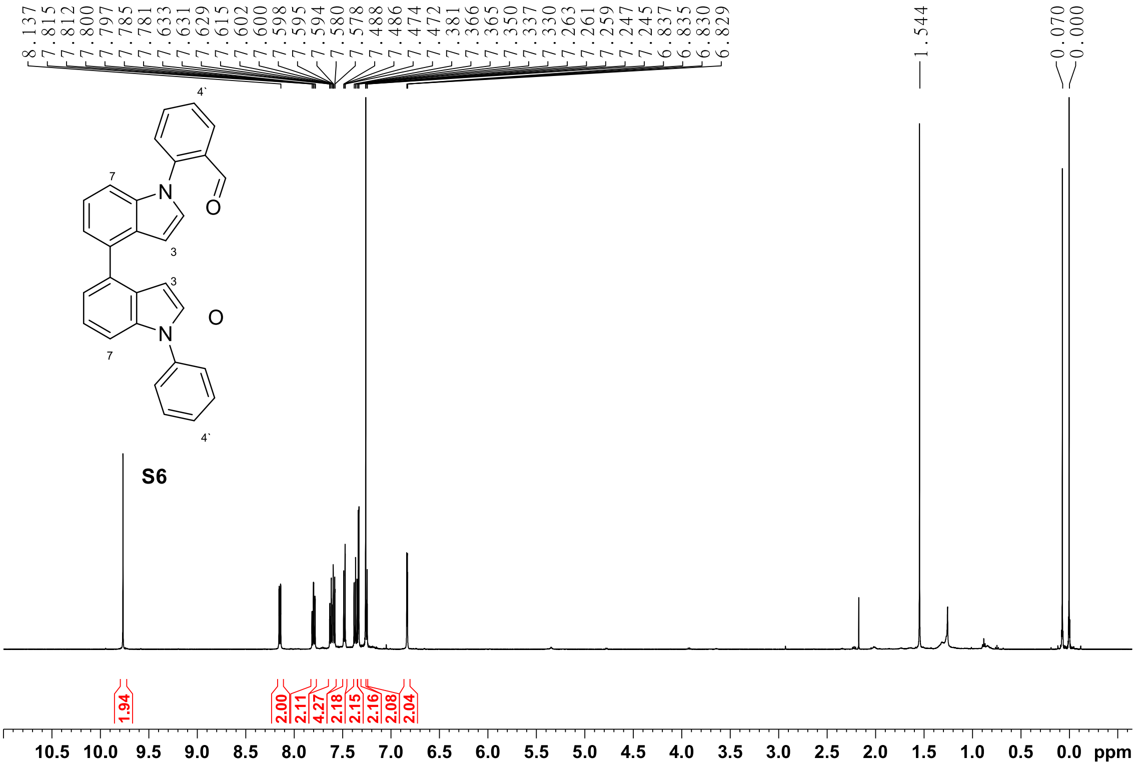

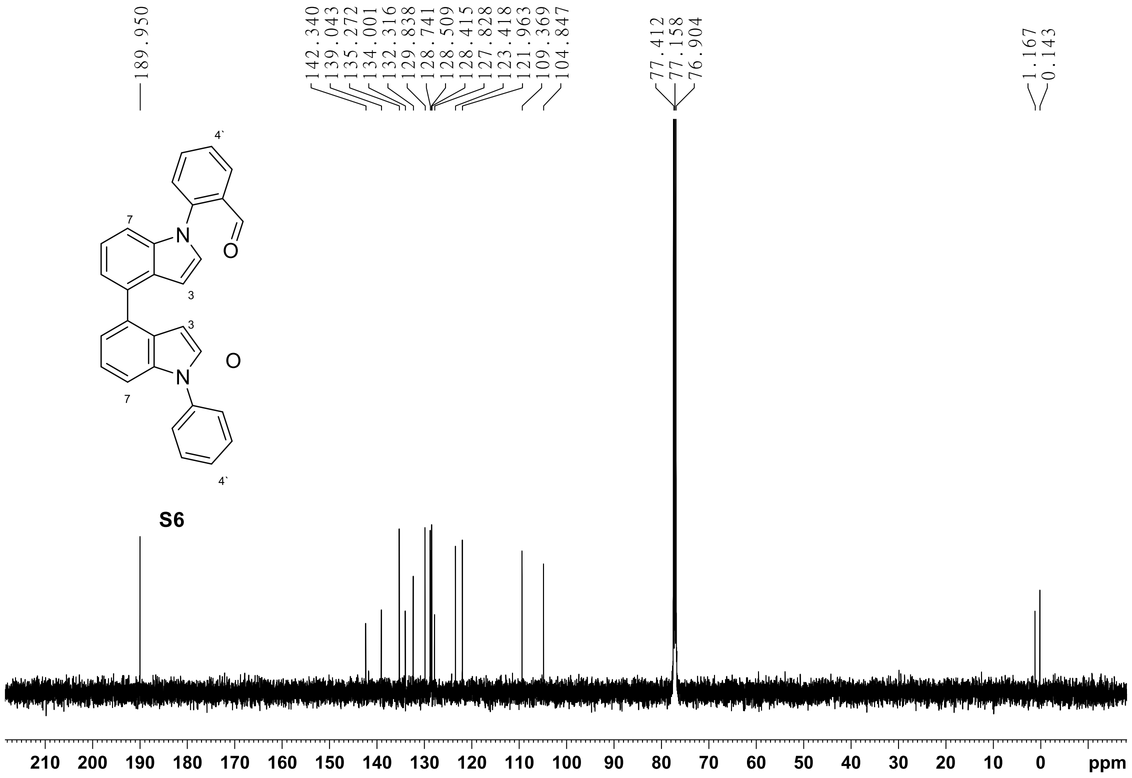

1,1'-Bis(2-vinylphenyl)-1*H*,1'*H*-4,4'-biindole (11):

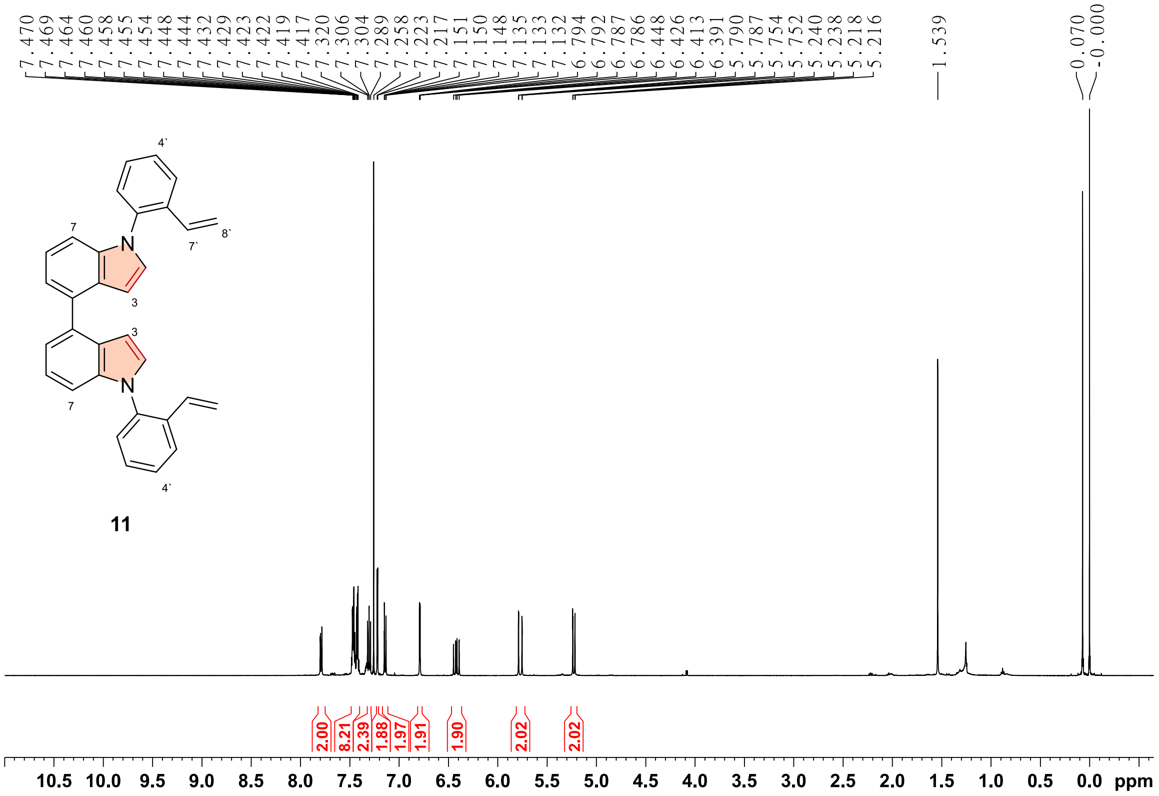

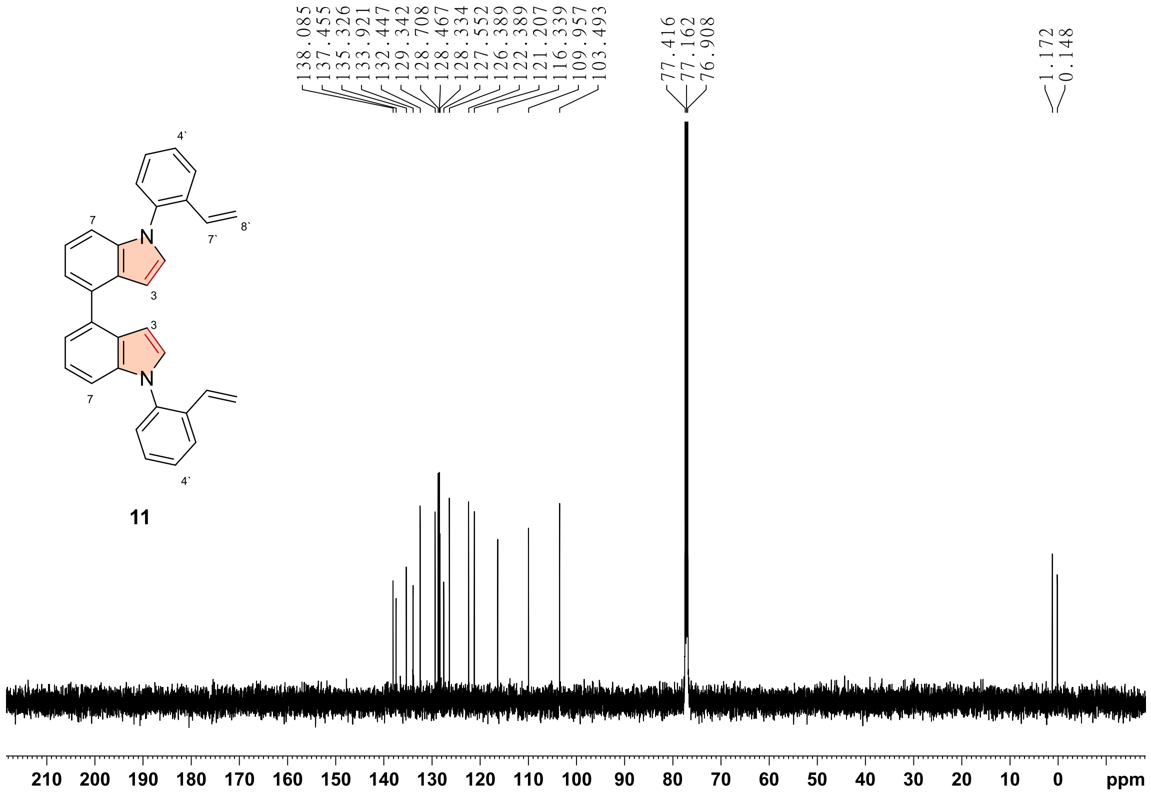

2-(4-(2-Vinylphenyl)-1*H*-indol-1-yl)benzaldehyde (S8a):

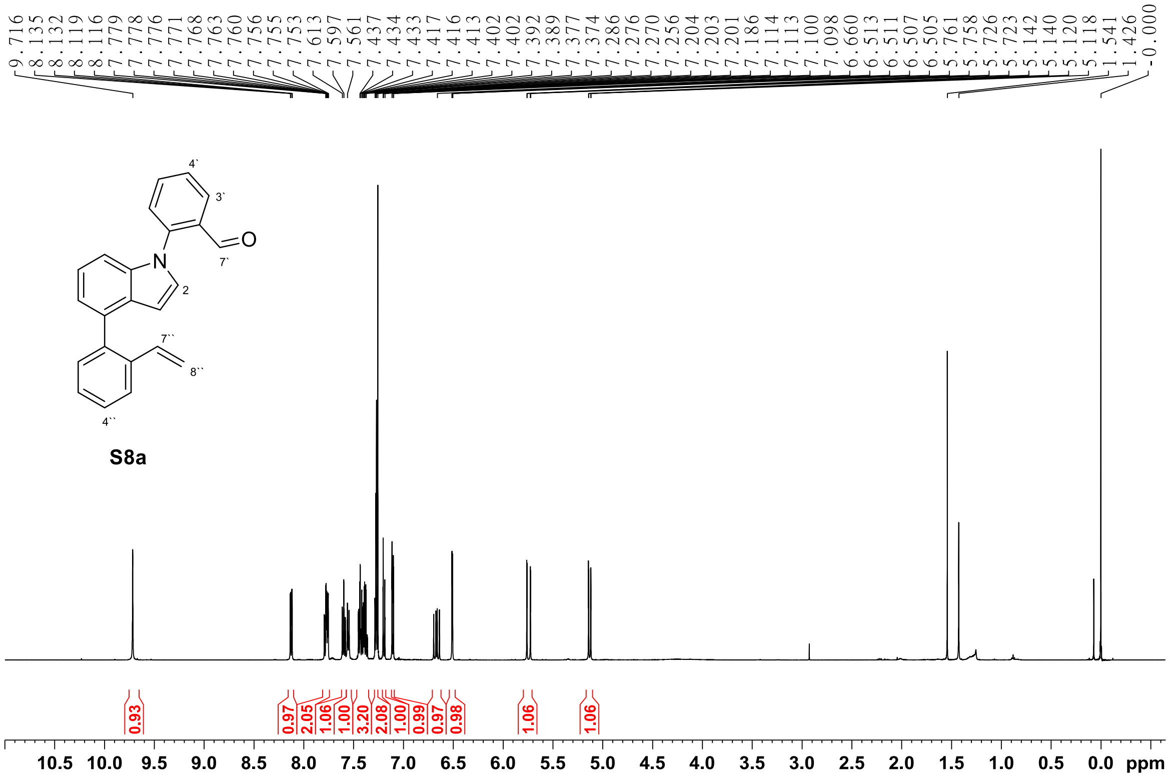

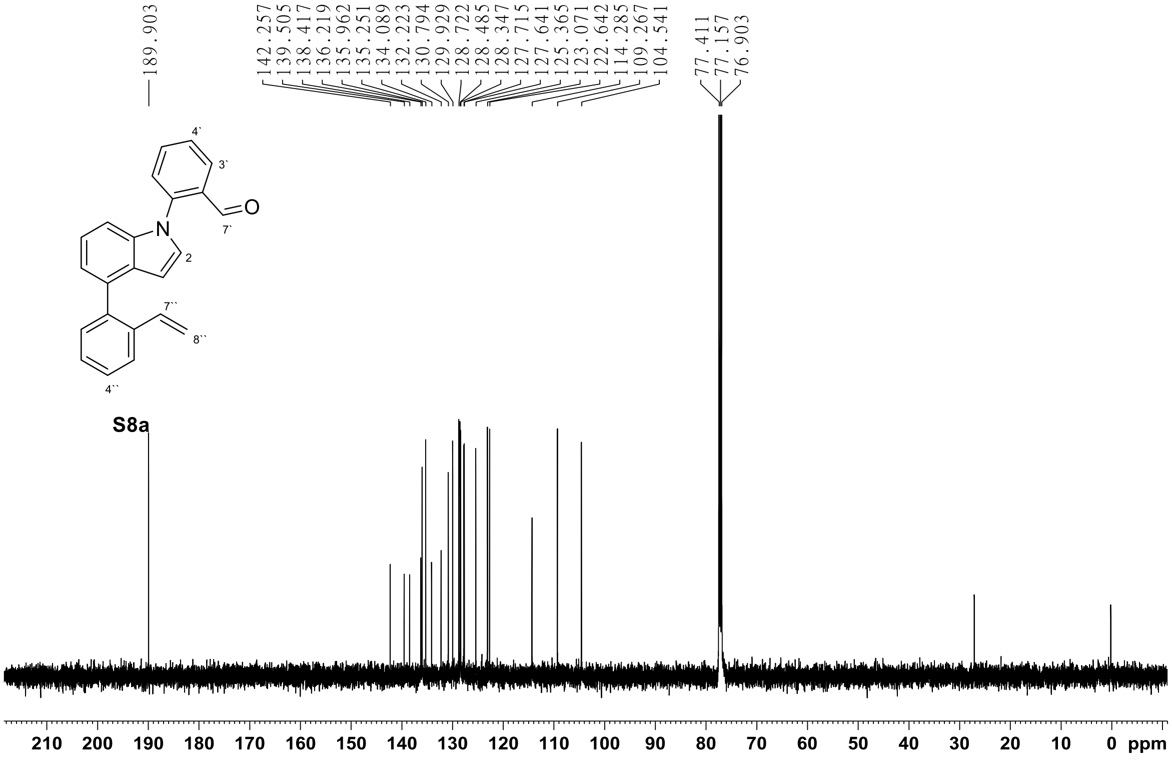

2-(4-(5-Chloro-2-vinylphenyl)-1*H*-indol-1-yl)benzaldehyde (S8b):

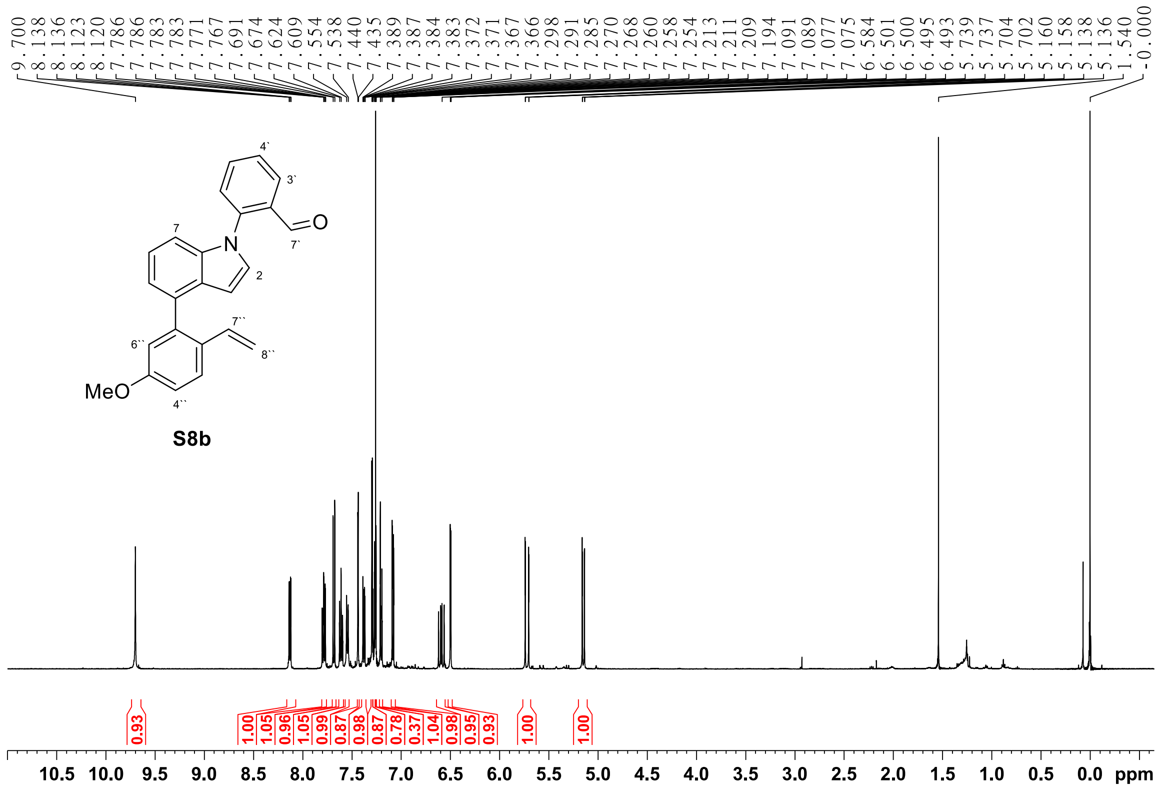

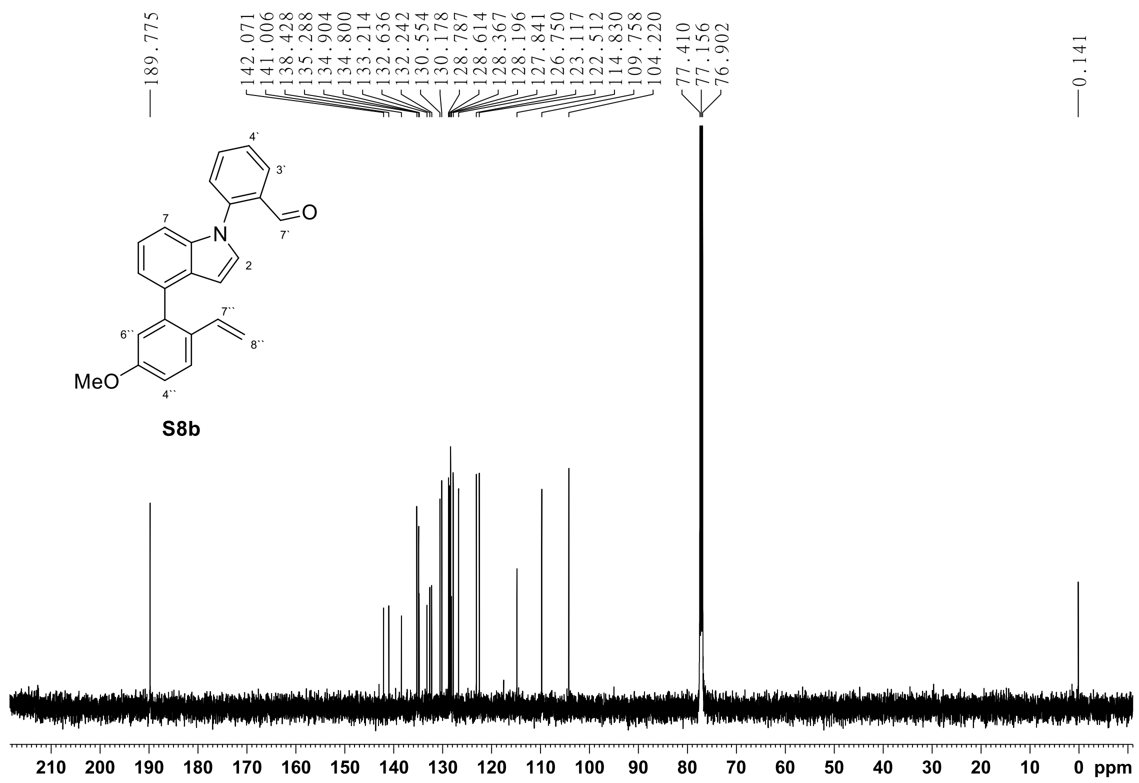

2-(4-(5-Methoxy-2-vinylphenyl)-1*H*-indol-1-yl)benzaldehyde (S8c):

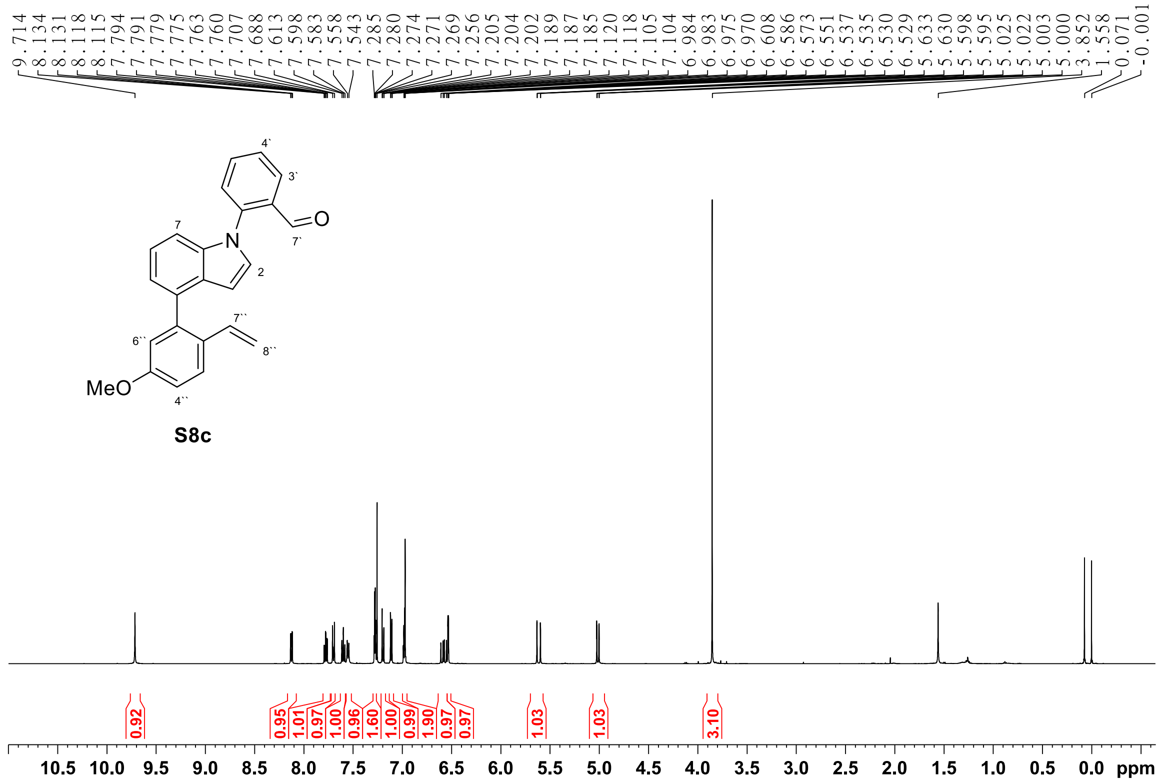

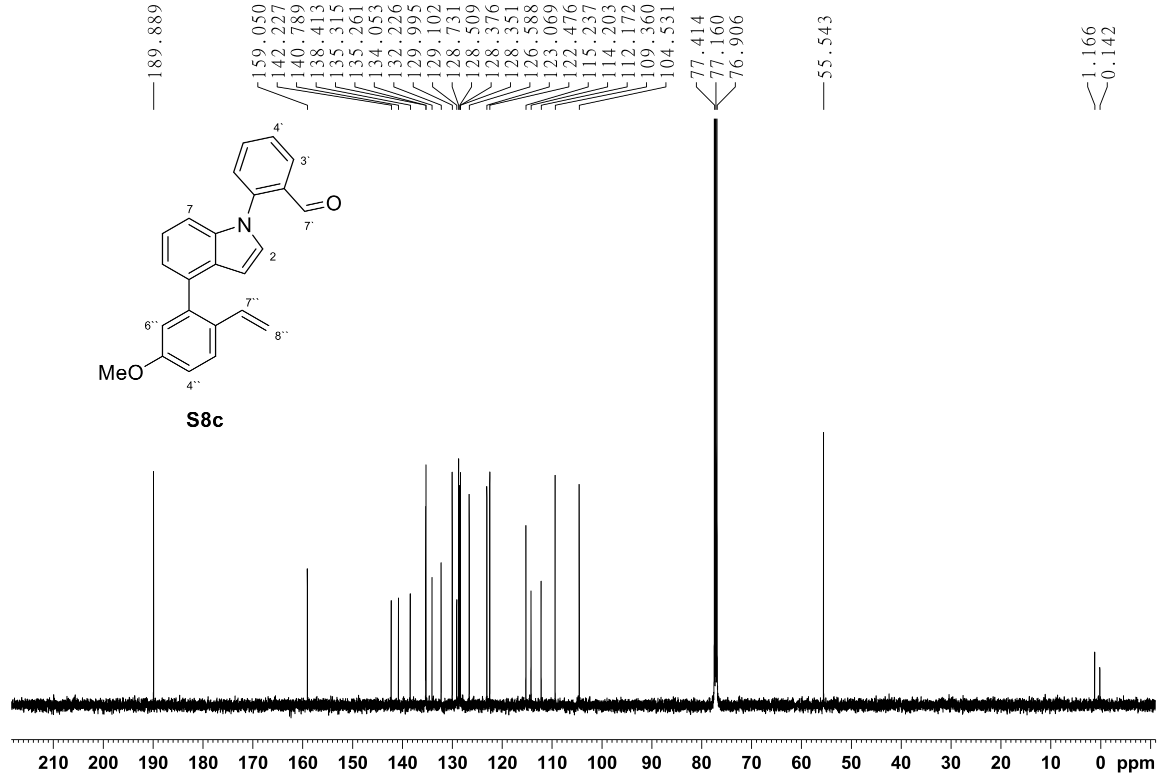

2-(6-Chloro-4-(2-vinylphenyl)-1*H*-indol-1-yl)benzaldehyde (S8d):

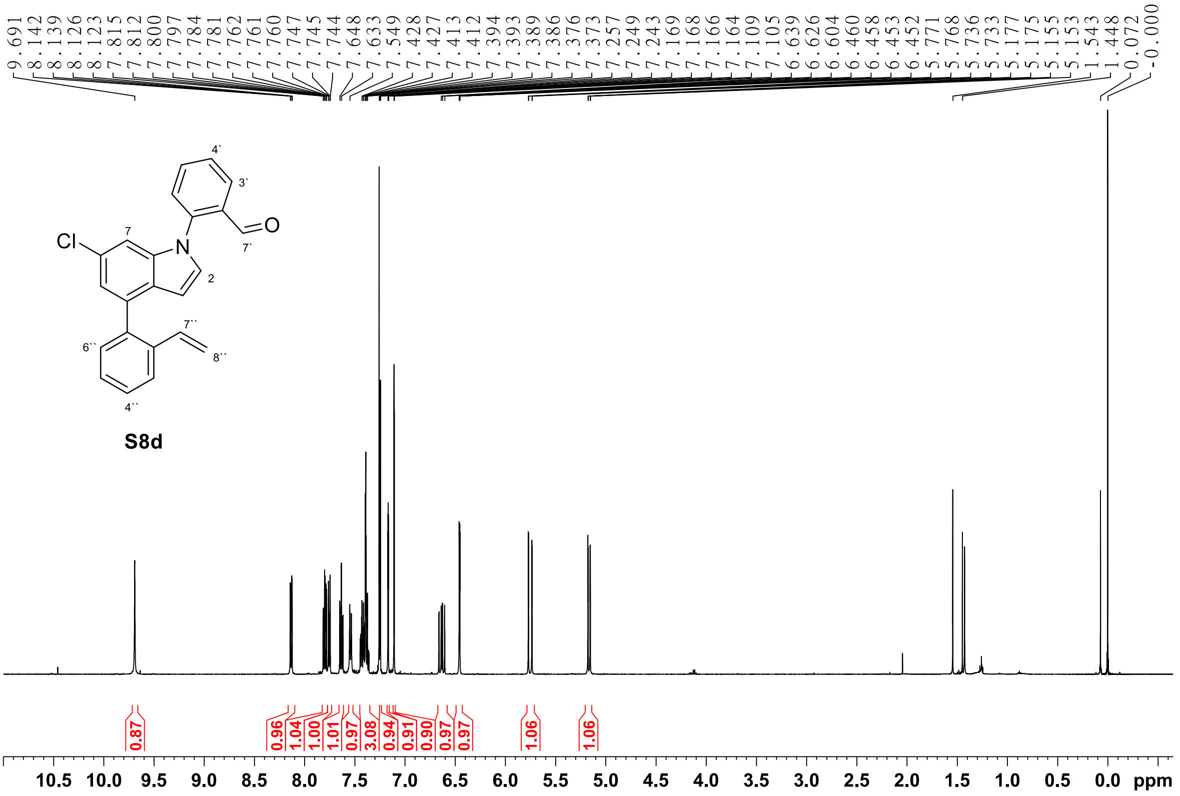

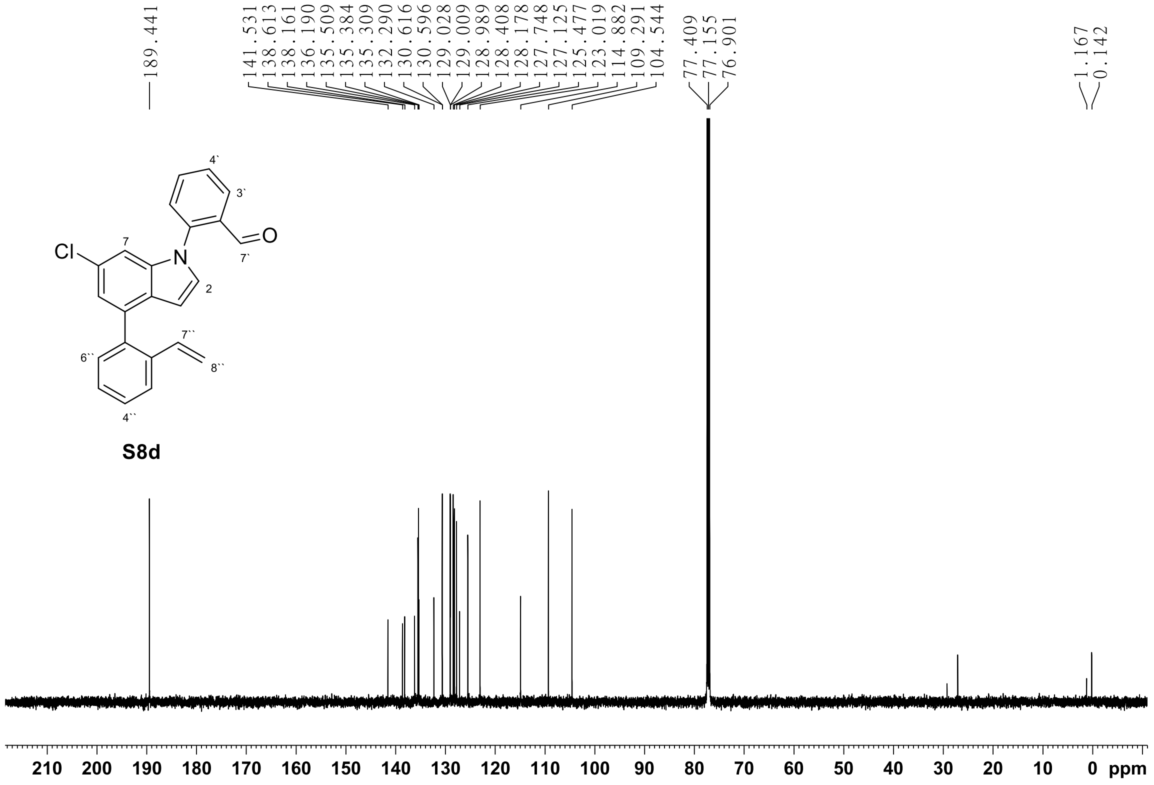

2-(6-(Trifluoromethyl)-4-(2-vinylphenyl)-1*H*-indol-1-yl)benzaldehyde (S8e):

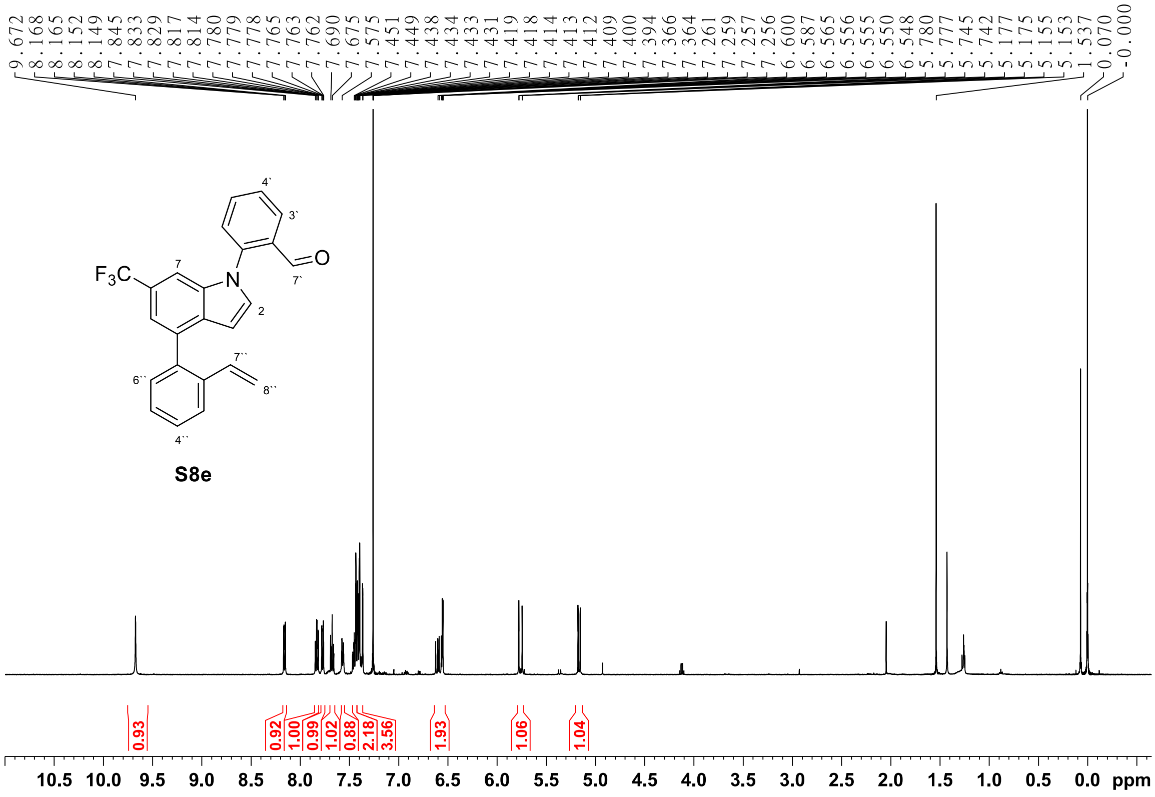

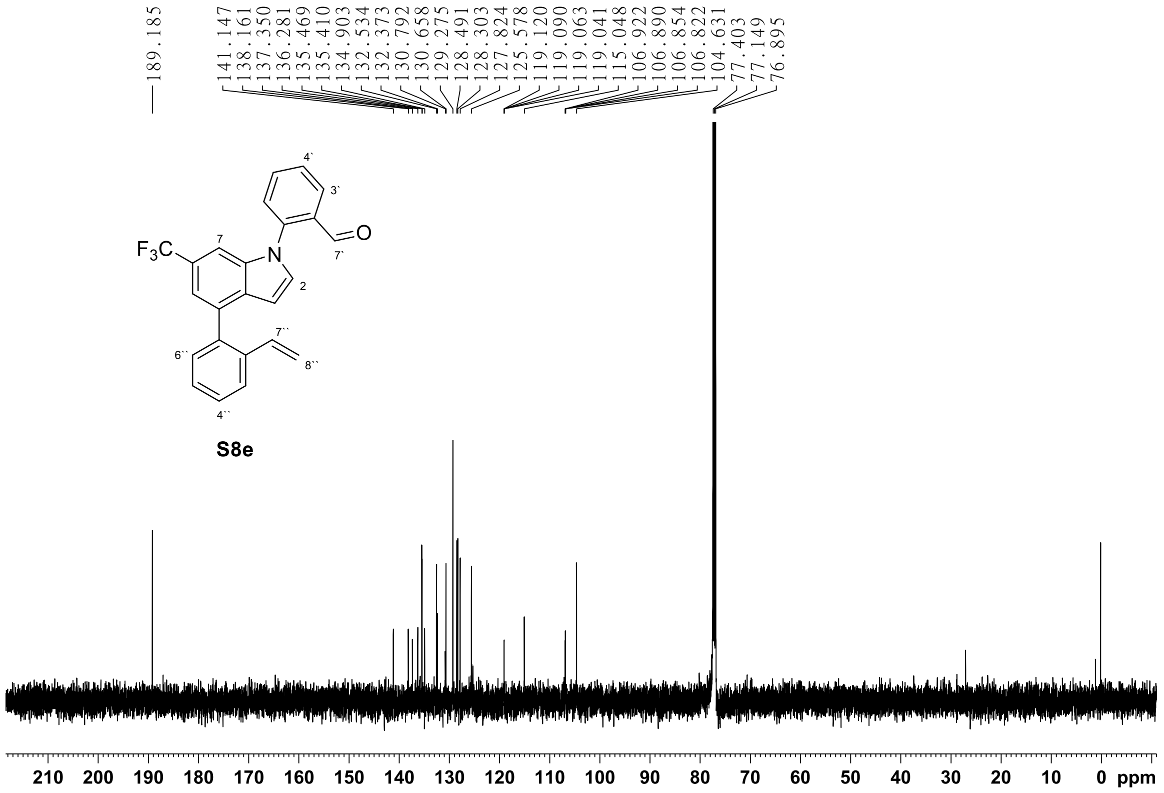

2-(7-Fluoro-4-(2-vinylphenyl)-1*H*-indol-1-yl)benzaldehyde (S8f):

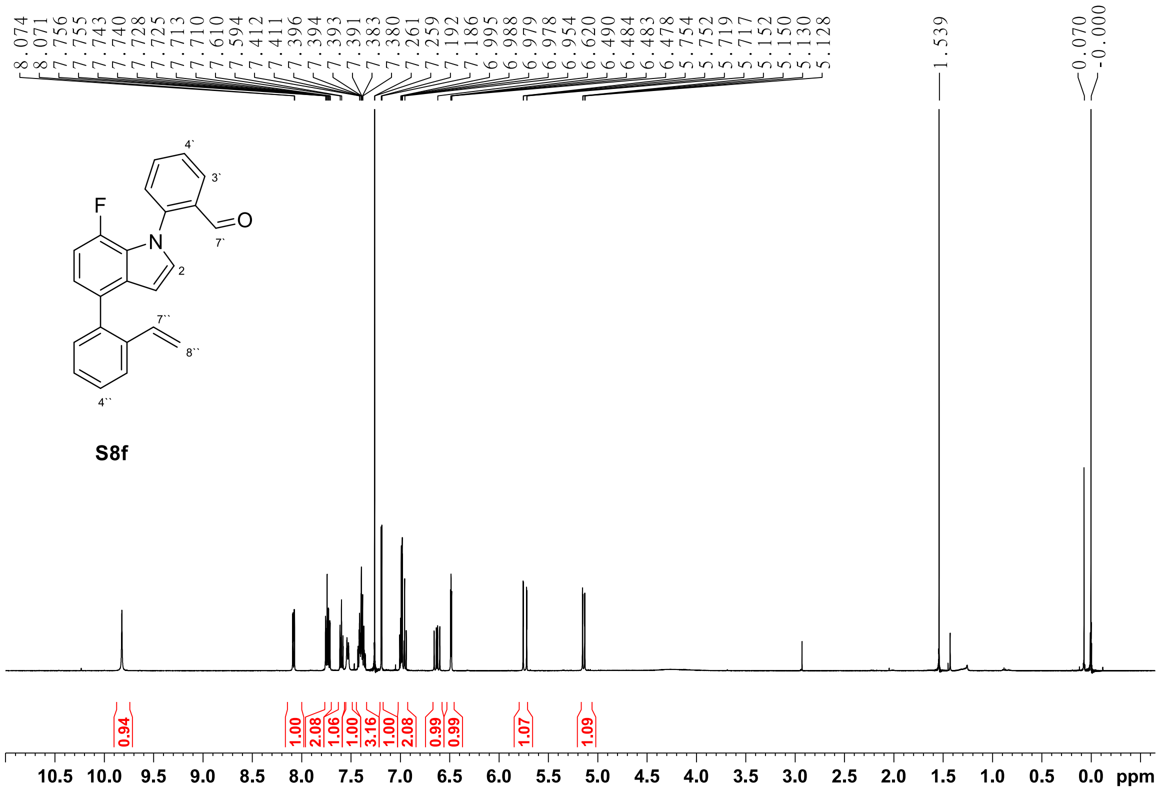

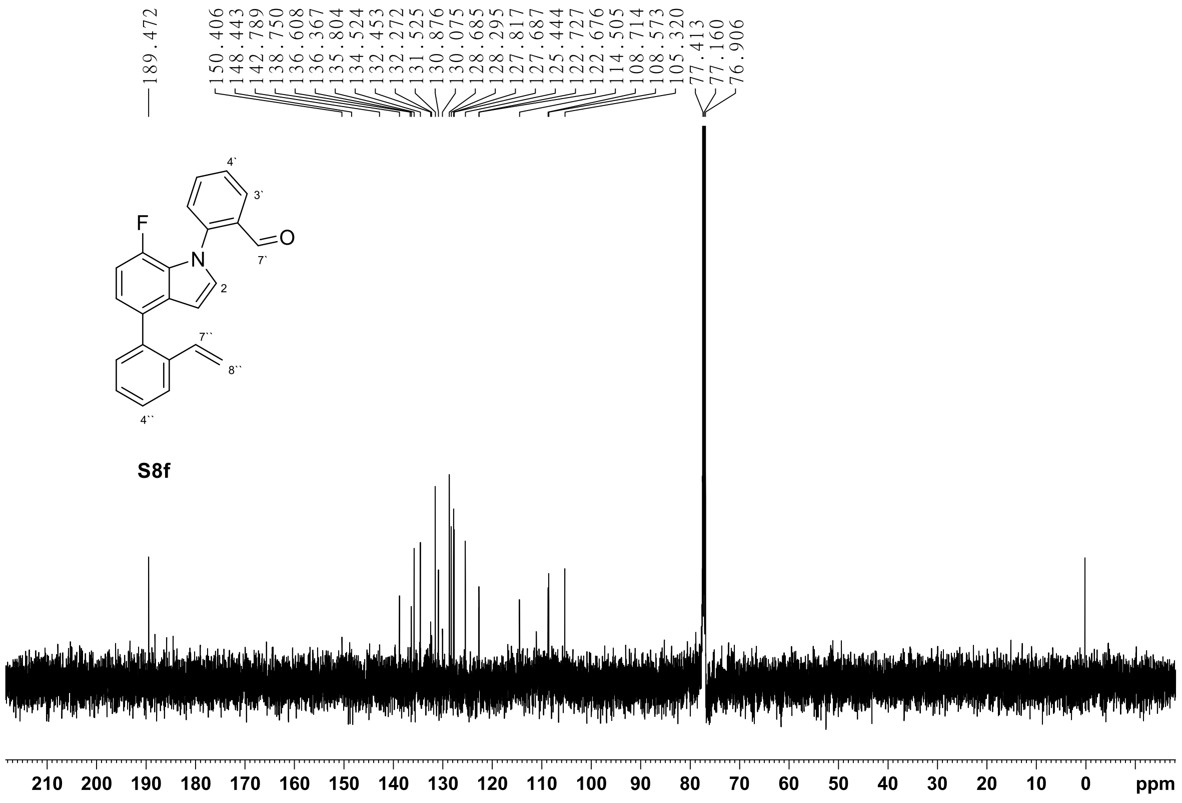

2-(3-Methyl-4-(2-vinylphenyl)-1*H*-indol-1-yl)benzaldehyde (S8g):

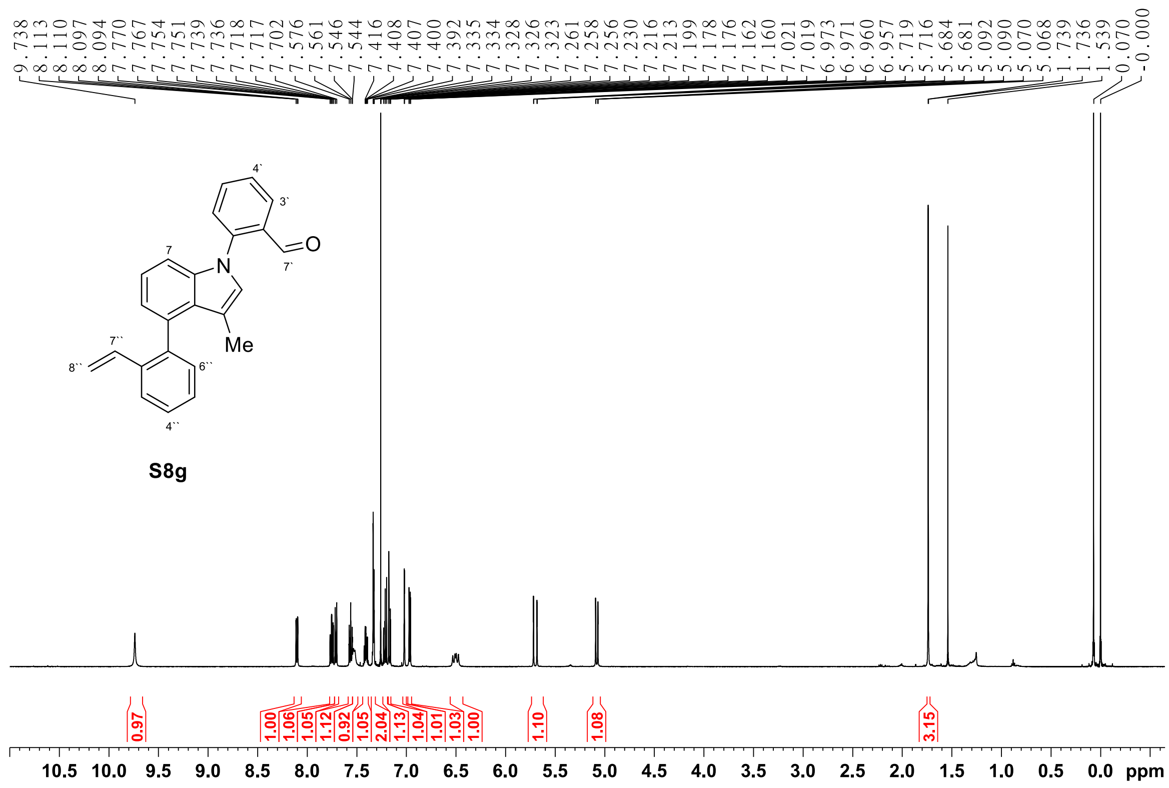

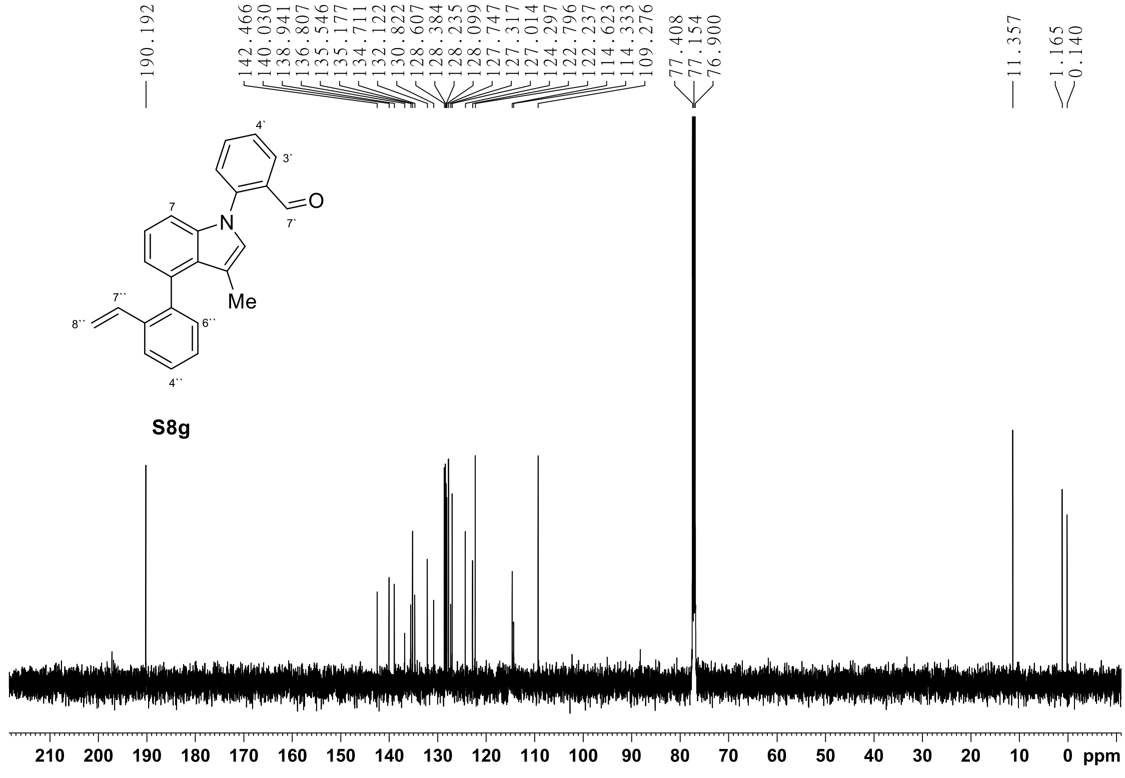

2-(4-(2-Vinylphenyl)-1*H*-pyrrolo[2,3-c]pyridin-1-yl)benzaldehyde (S8h):

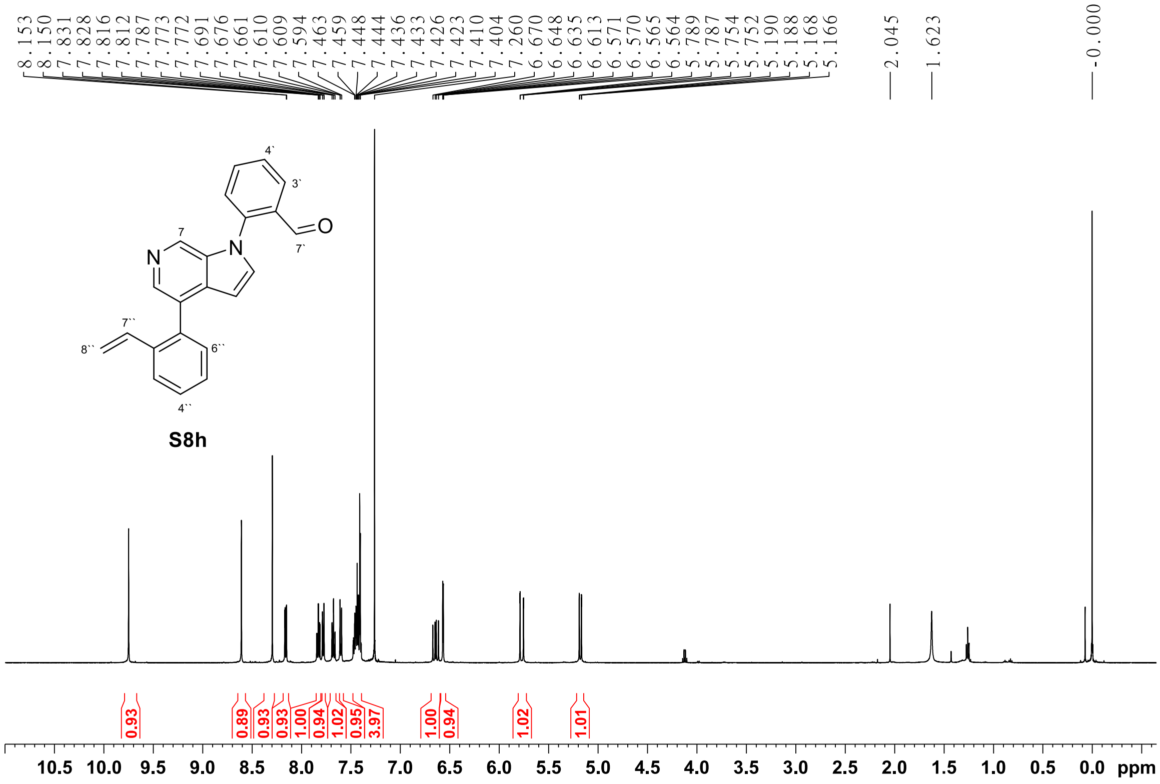

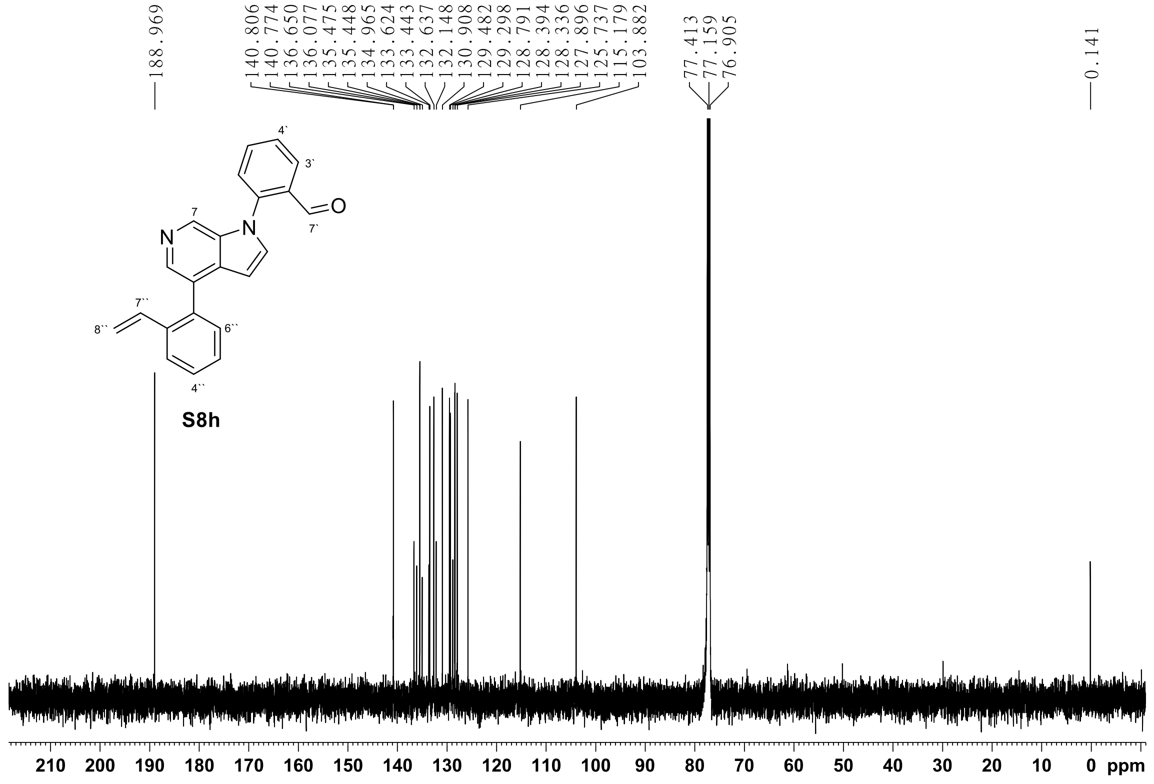

2-(2-Methyl-4-(2-vinylphenyl)-1*H*-indol-1-yl)benzaldehyde (S8i):

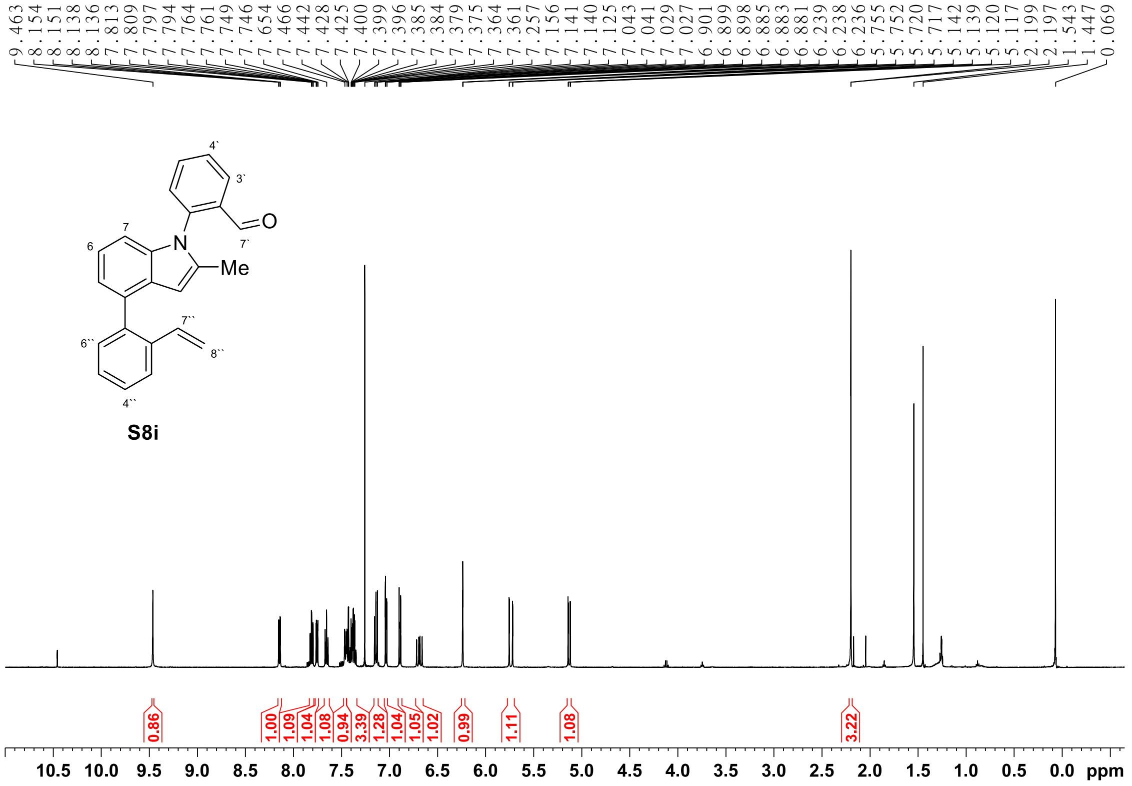

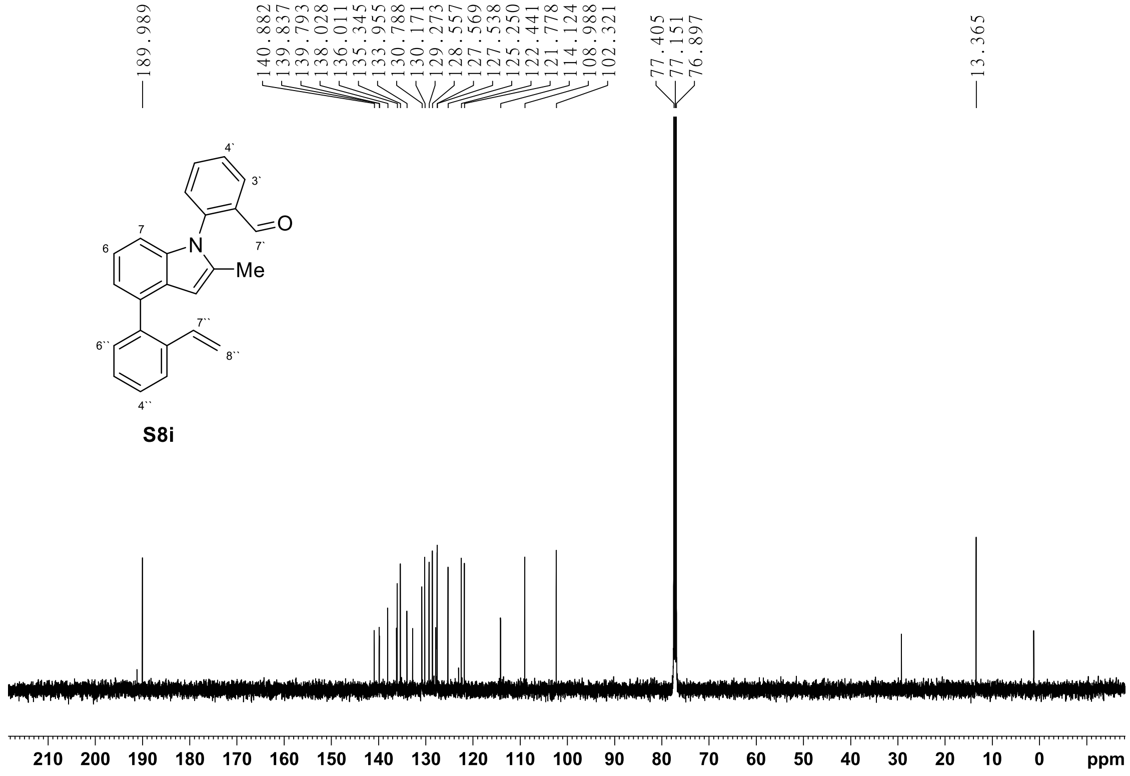

2-(7-Methyl-4-(2-vinylphenyl)-1*H*-indol-1-yl)benzaldehyde (S8j):

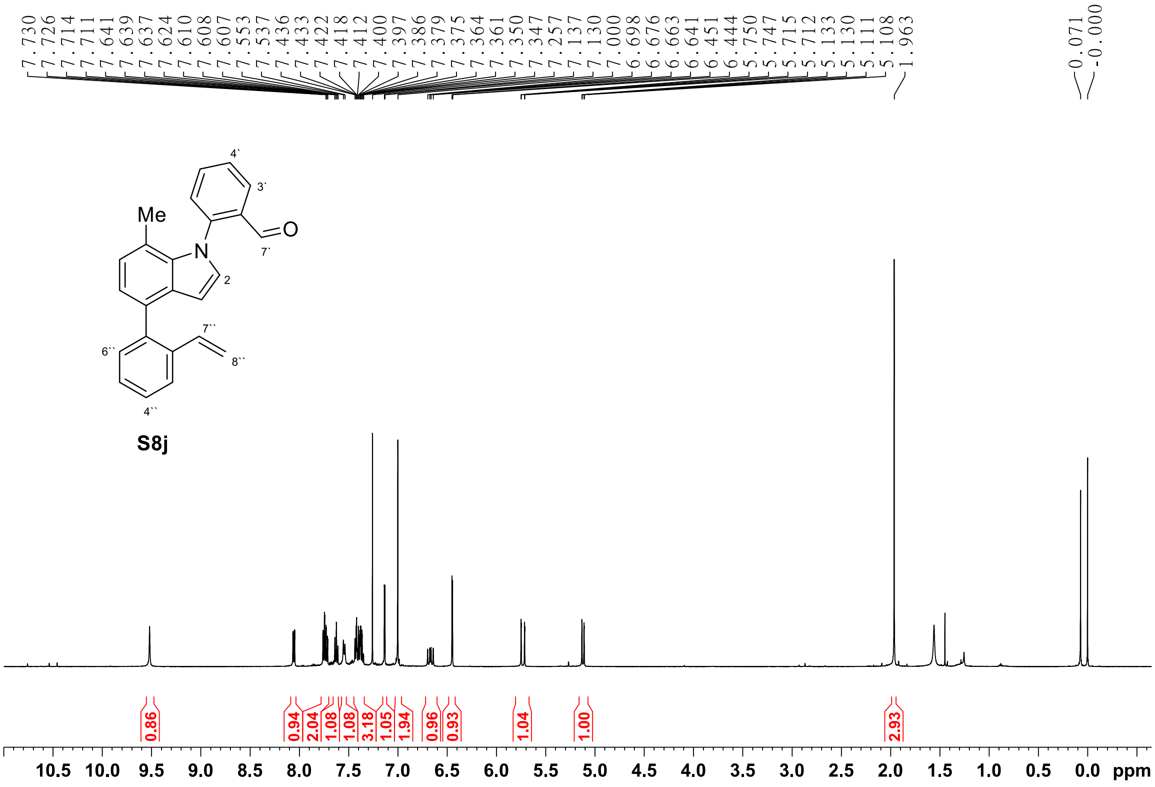

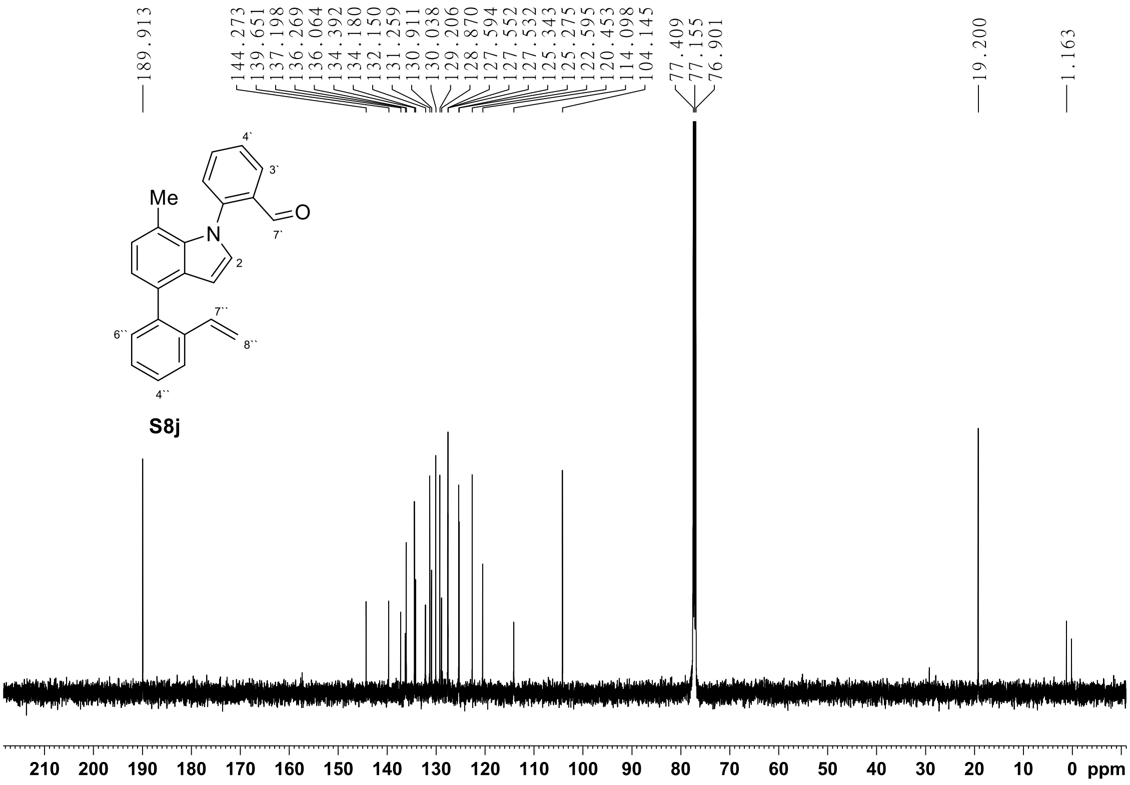

2-(7-Chloro-4-(2-vinylphenyl)-1*H*-indol-1-yl)benzaldehyde (S8k):

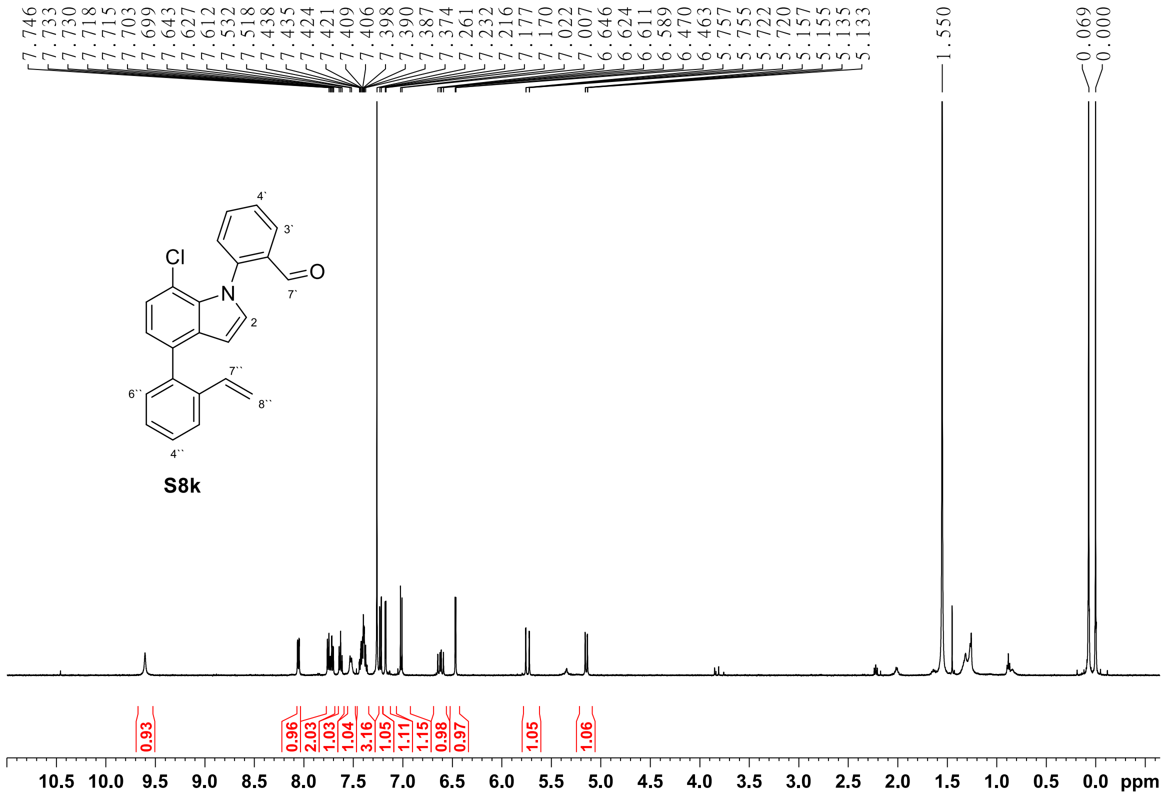

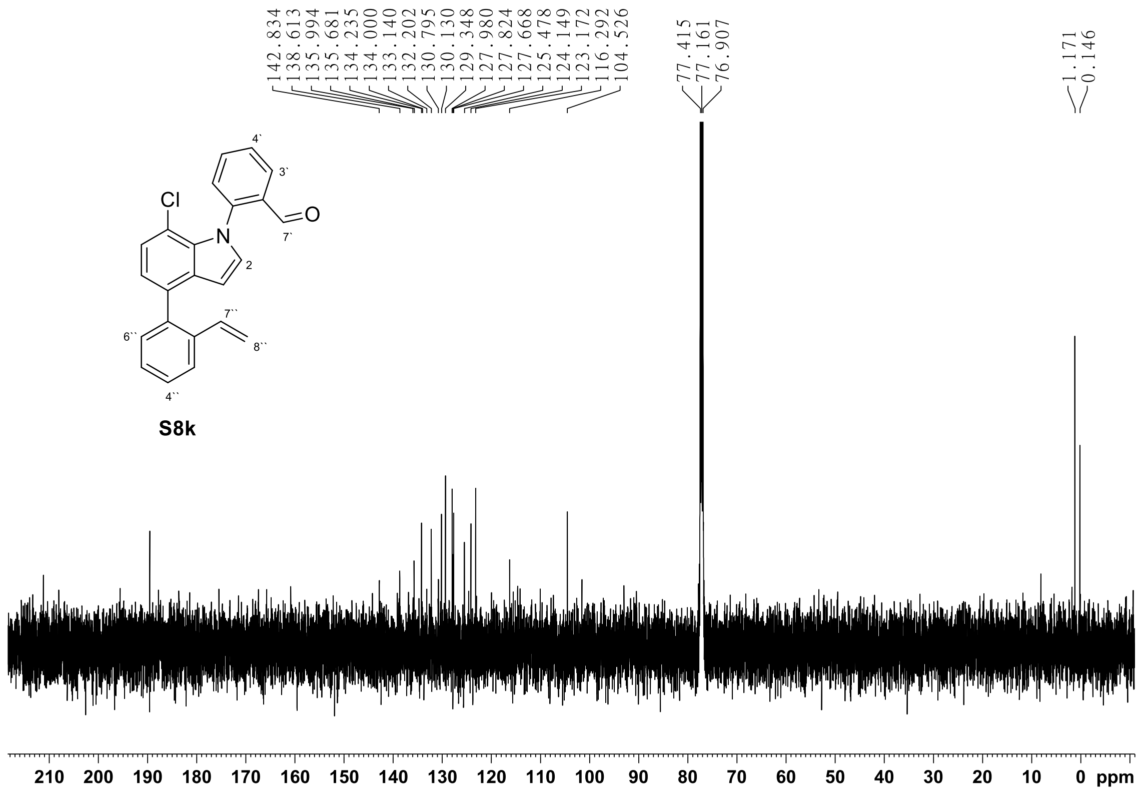

2-(7-Chloro-4-(5-chloro-2-vinylphenyl)-1*H*-indol-1-yl)benzaldehyde (S8l):

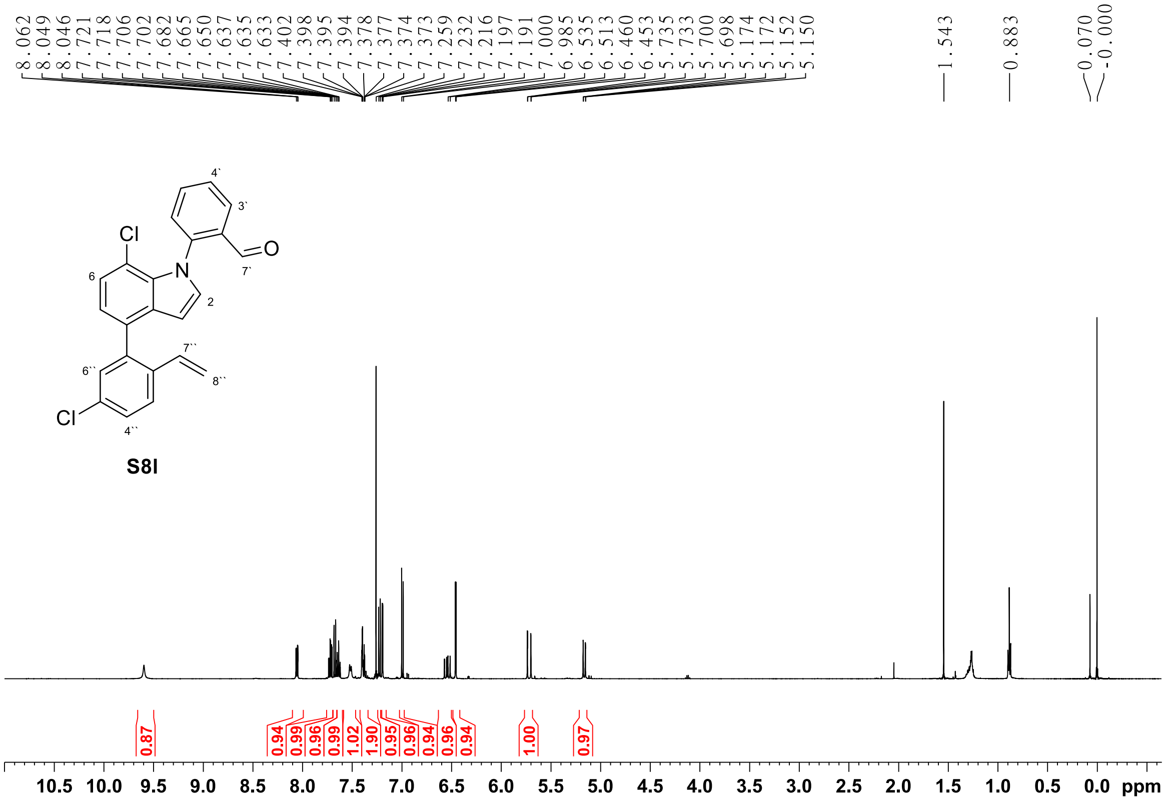

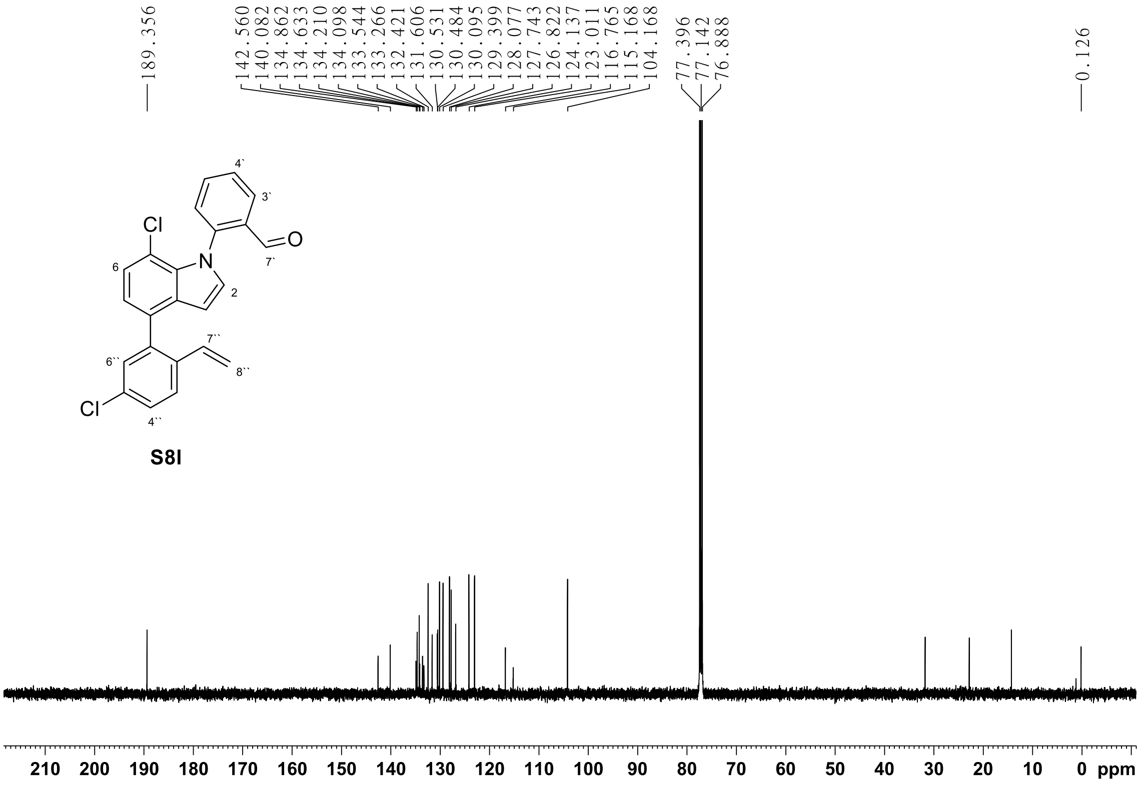

1,4-Bis(2-vinylphenyl)-1*H*-indole (13a):

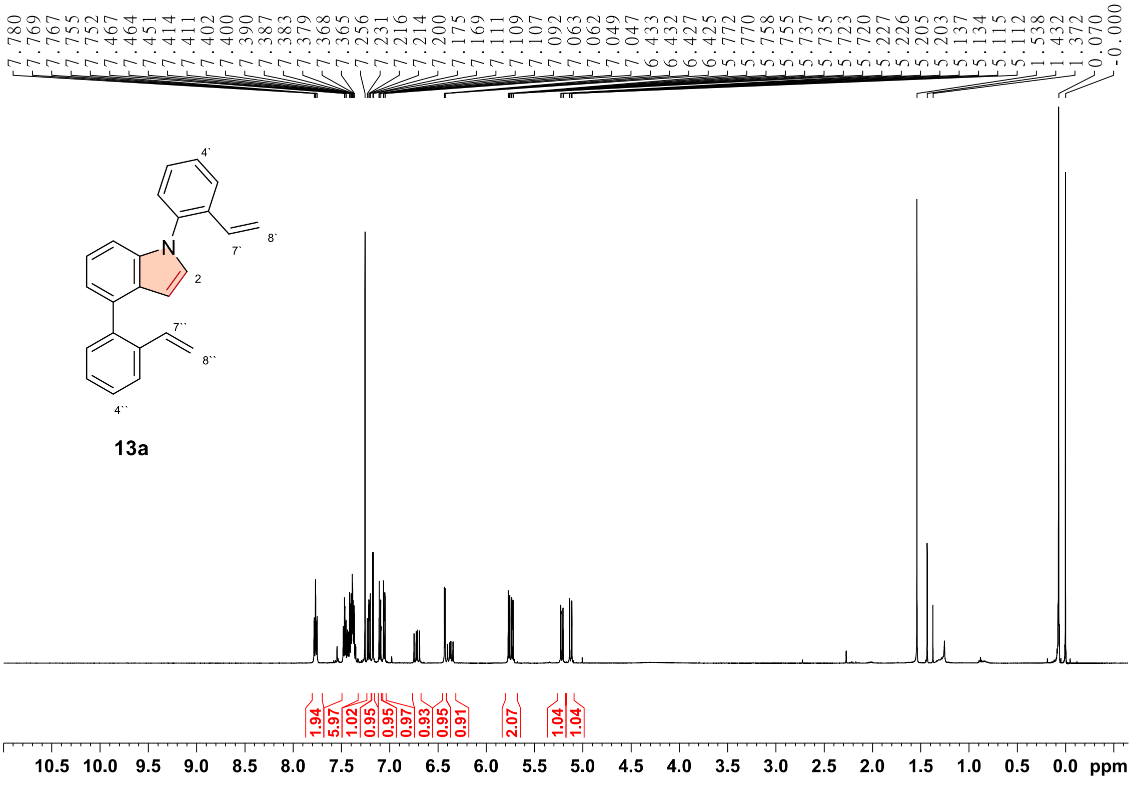

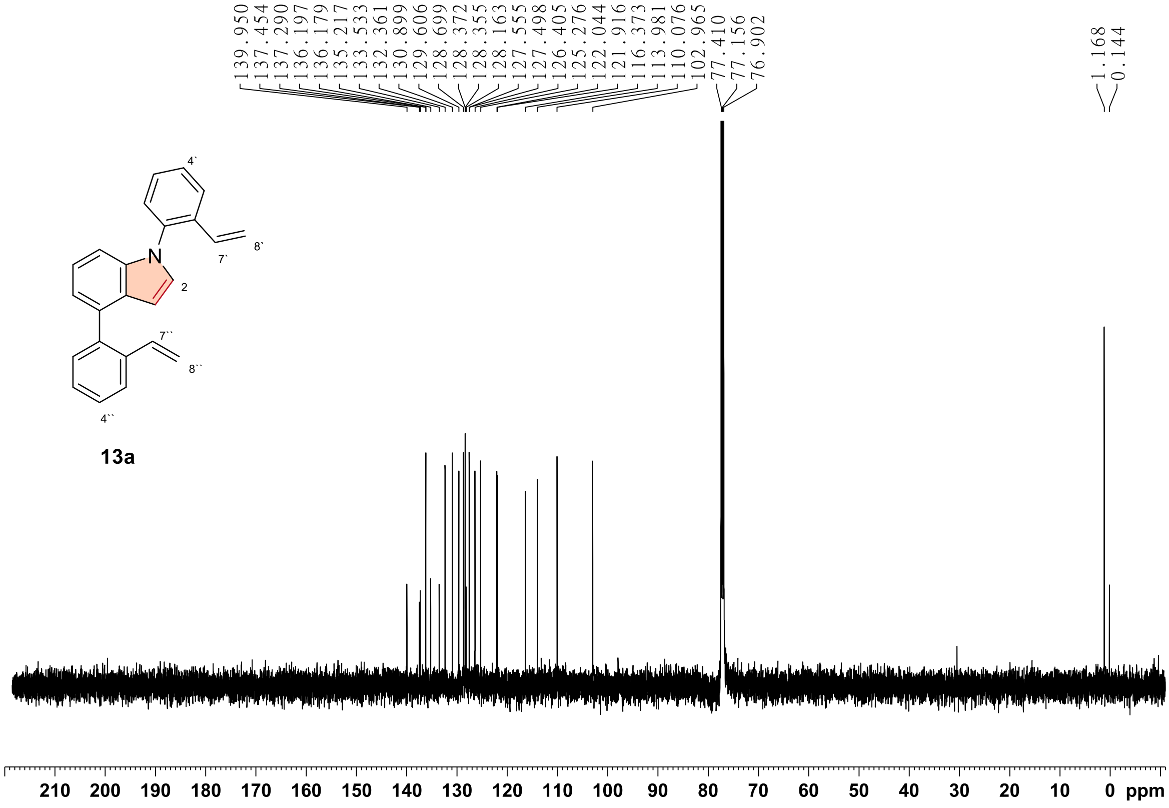

4-(5-Chloro-2-vinylphenyl)-1-(2-vinylphenyl)-1*H*-indole (13b):

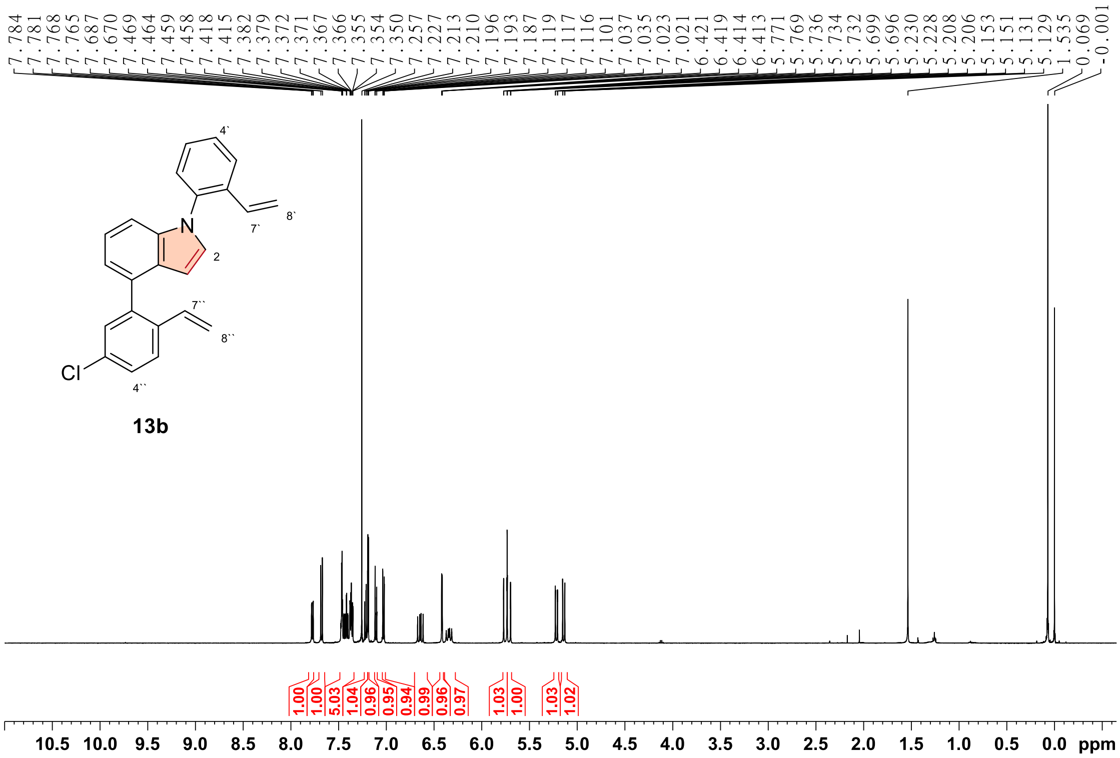

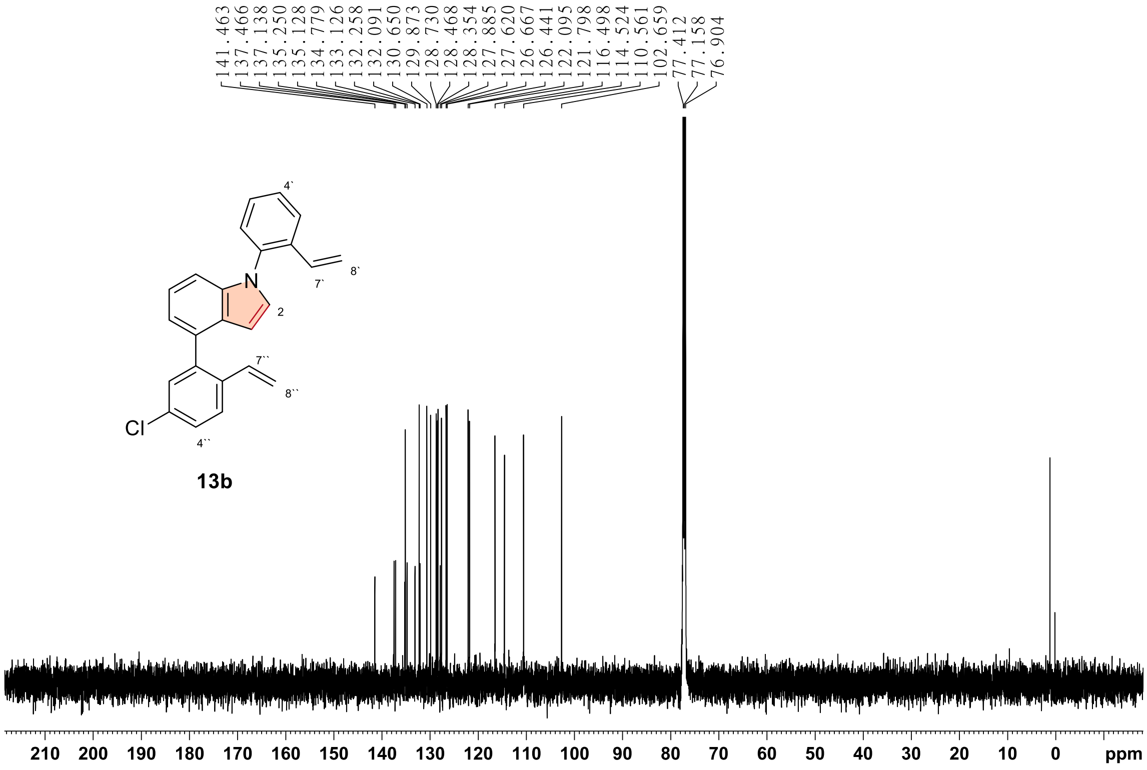

4-(5-Methoxy-2-vinylphenyl)-1-(2-vinylphenyl)-1*H*-indole (13c):

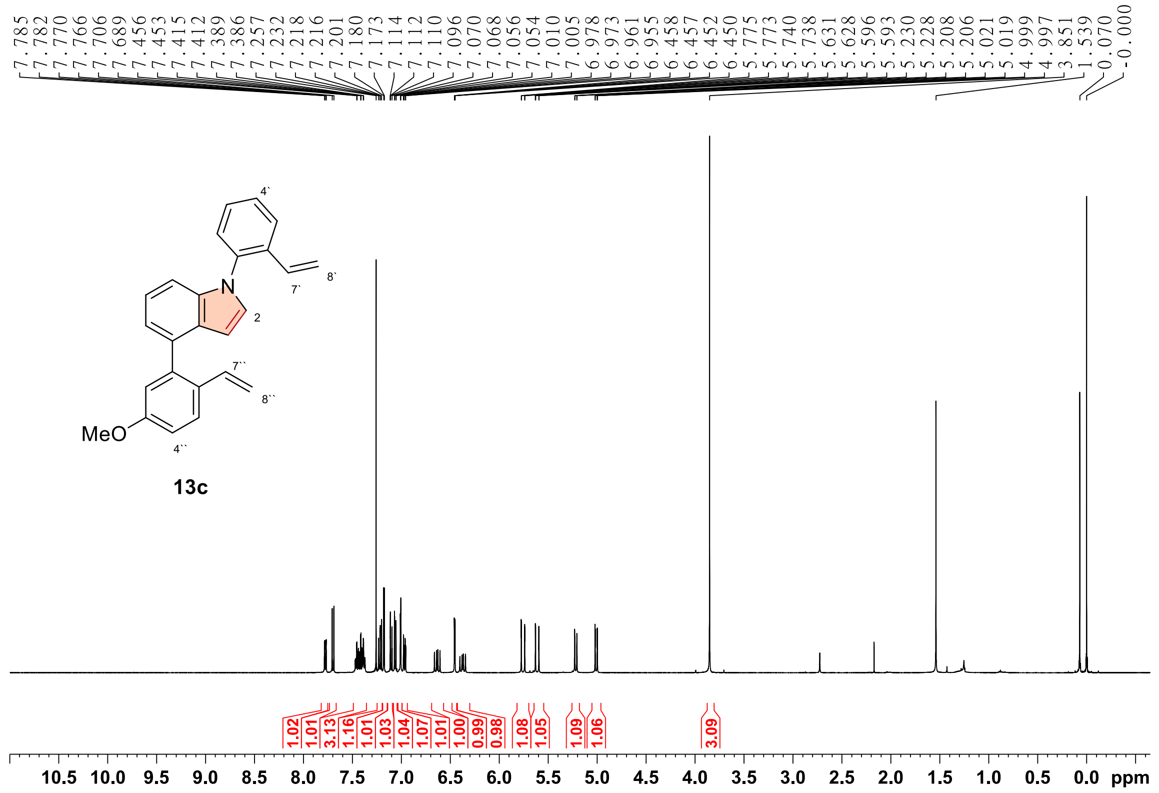

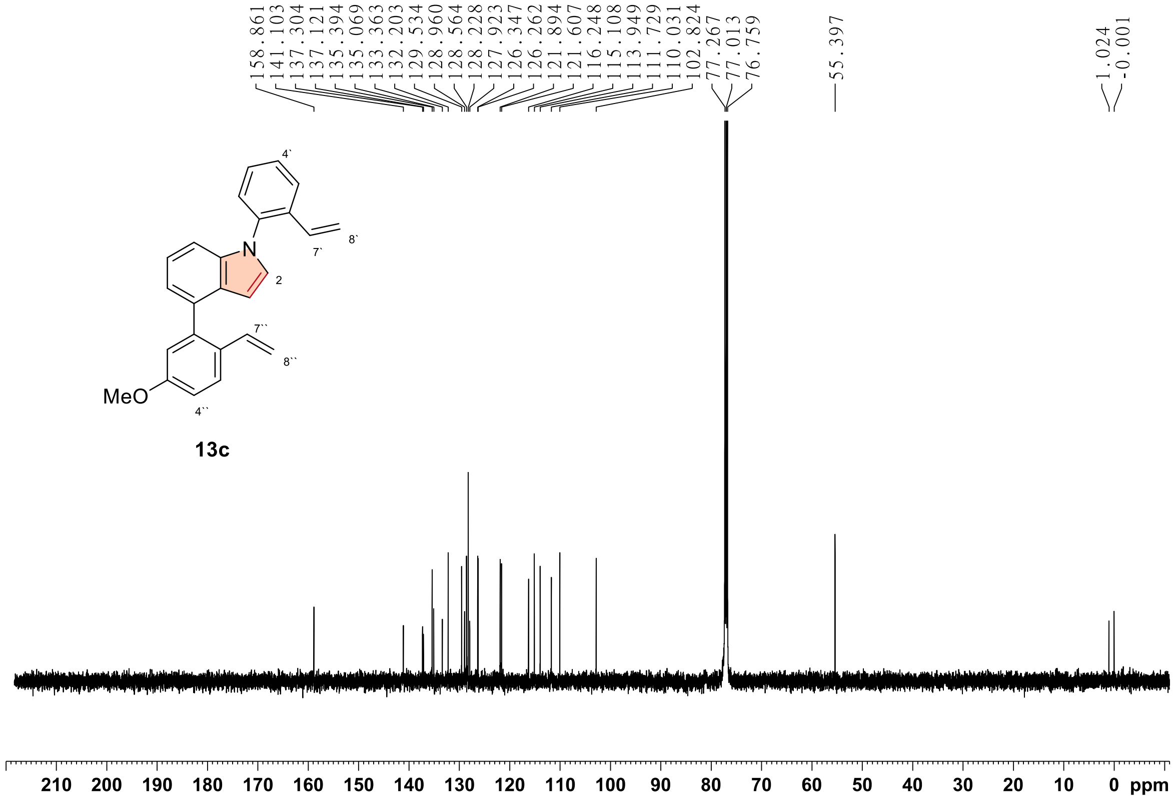

6-Chloro-1,4-bis(2-vinylphenyl)-1*H*-indole (13d):

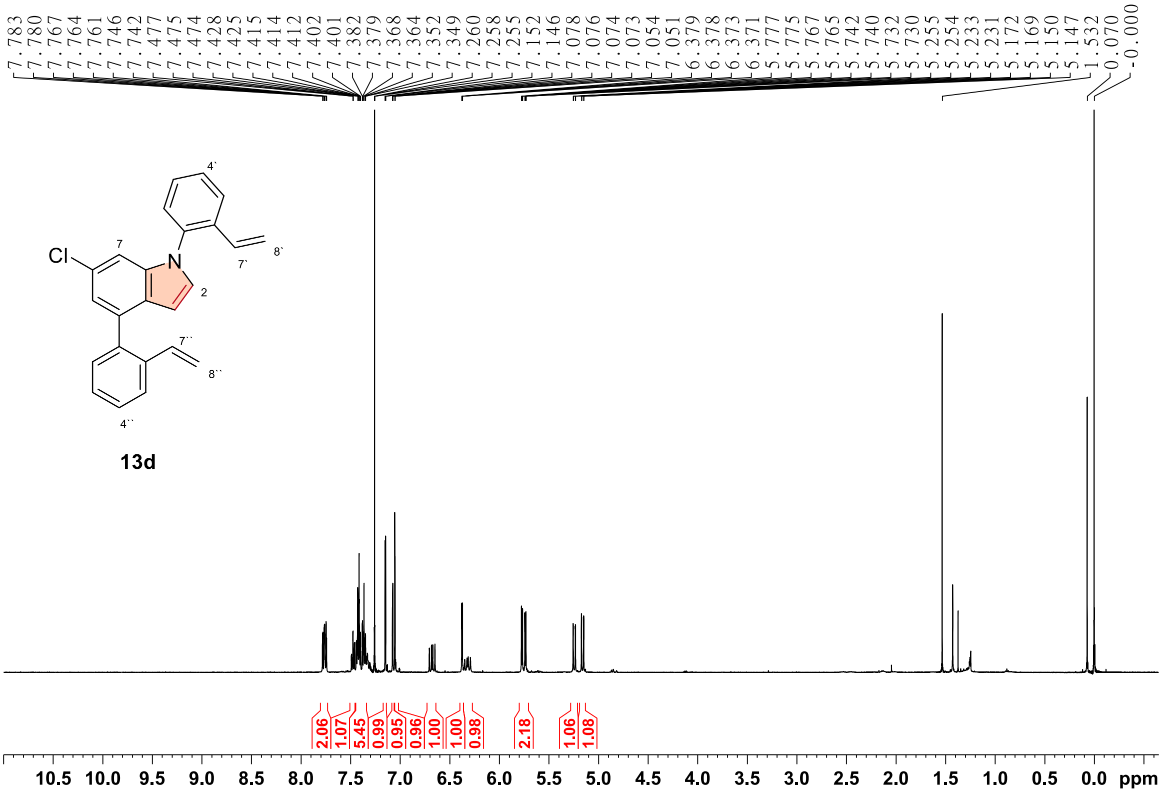

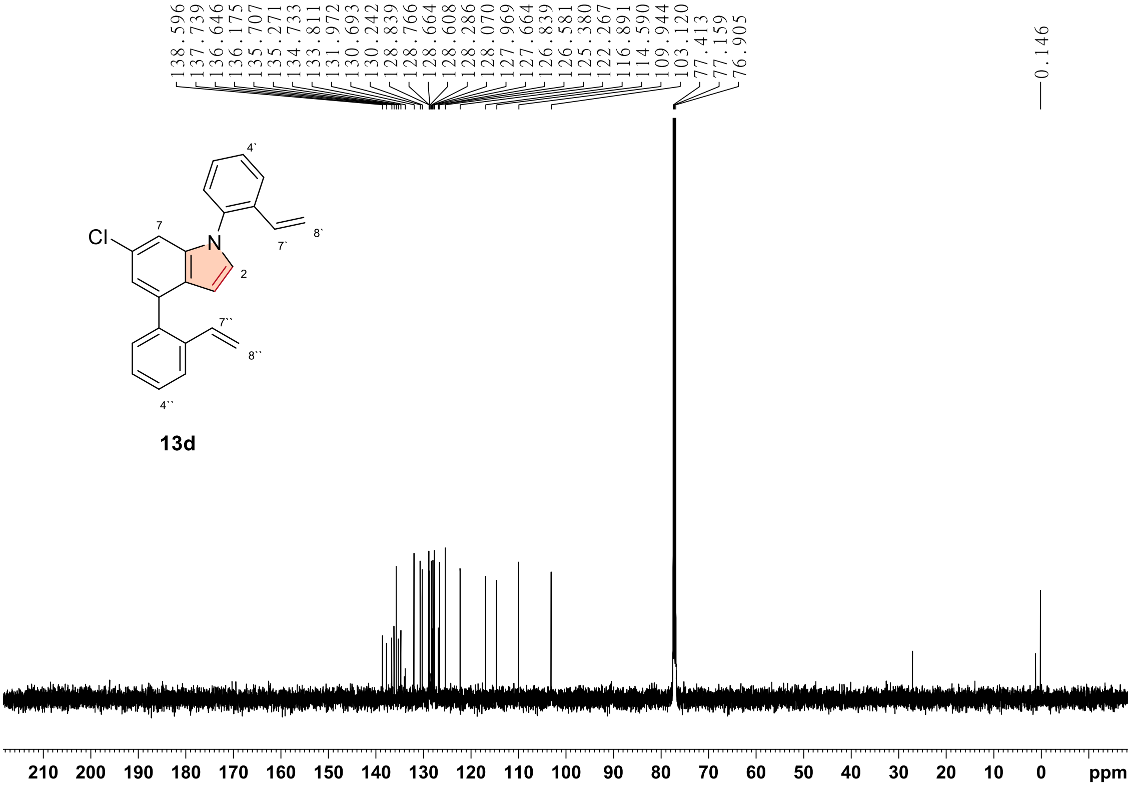

6-(Trifluoromethyl)-1,4-bis(2-vinylphenyl)-1*H*-indole (13e):

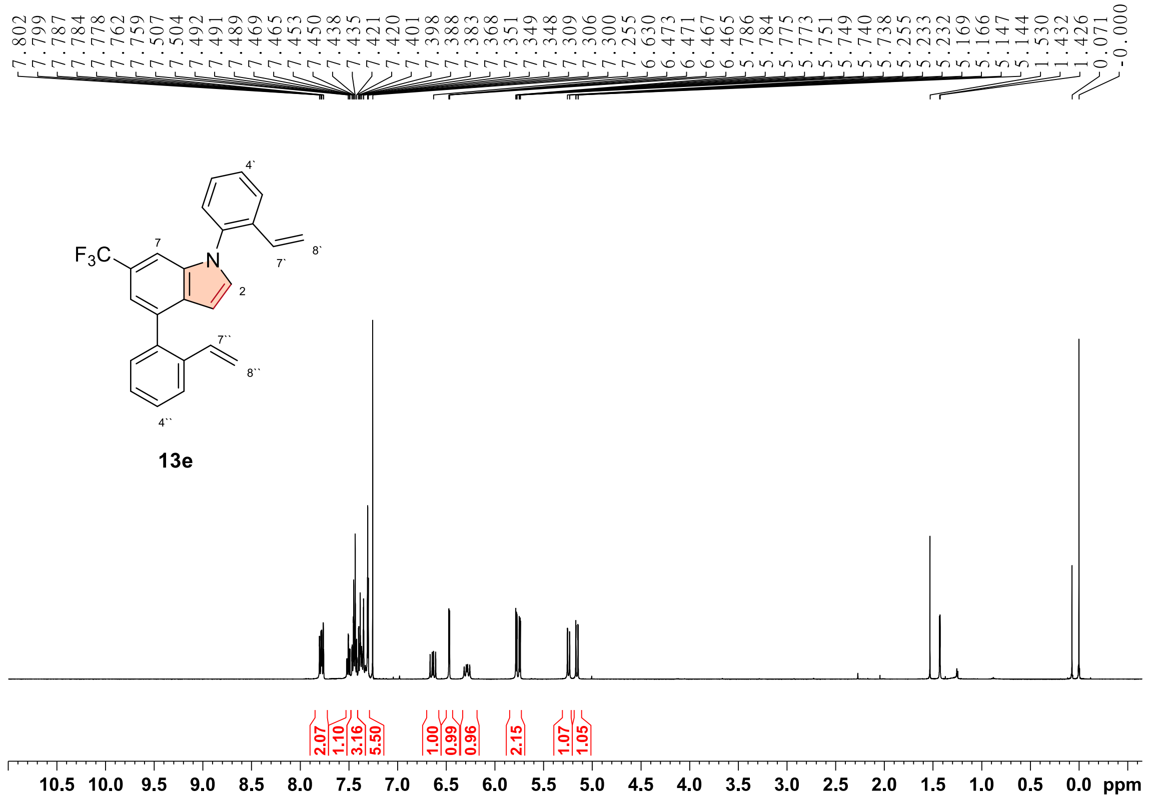

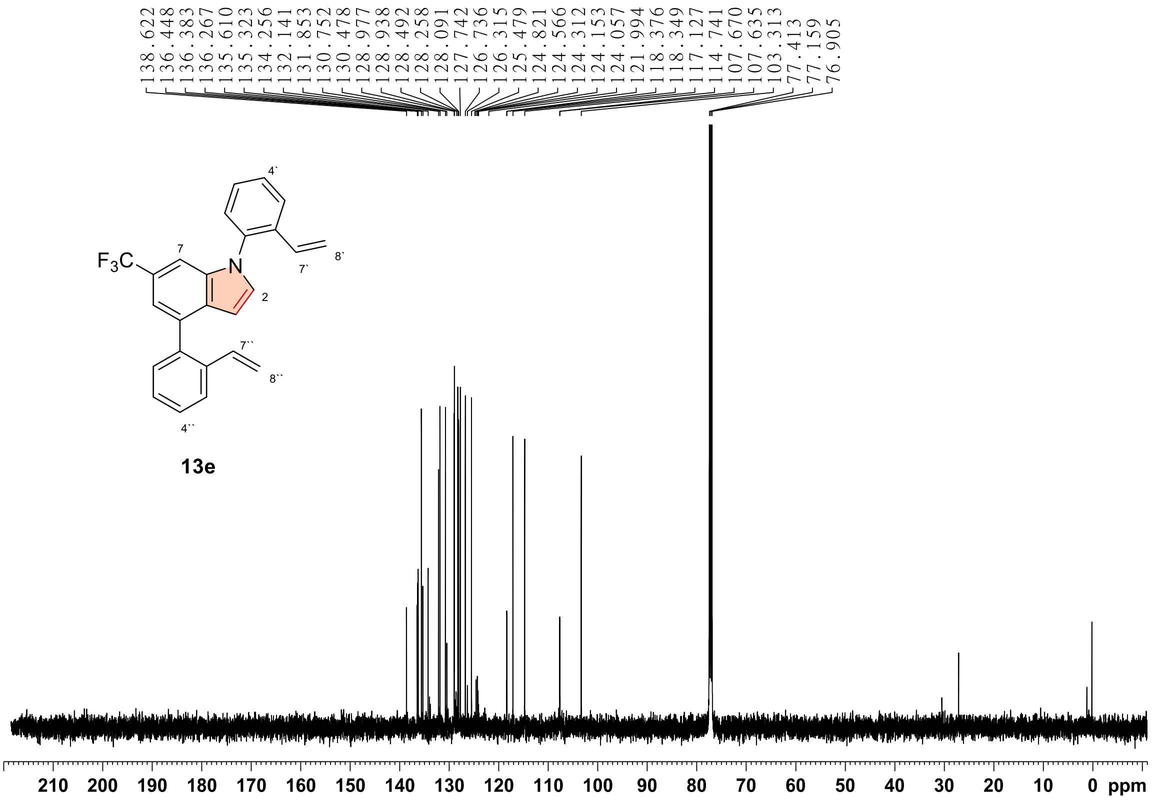

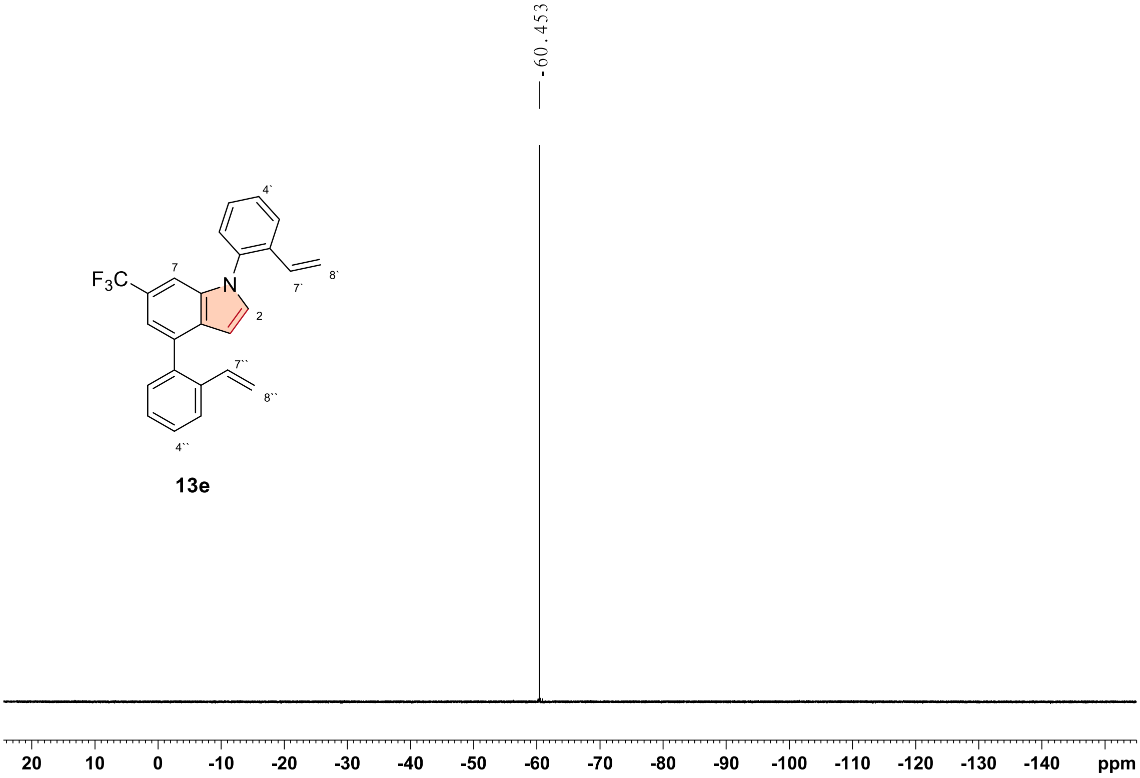

-60.453
F3C
N
13e
20
10
0
-10
-20
-30
-40
-50
-60
-70
-80
-90
-100
-110
-120
-130
-140
ppm

7-Fluoro-1,4-bis(2-vinylphenyl)-1*H*-indole (13f):

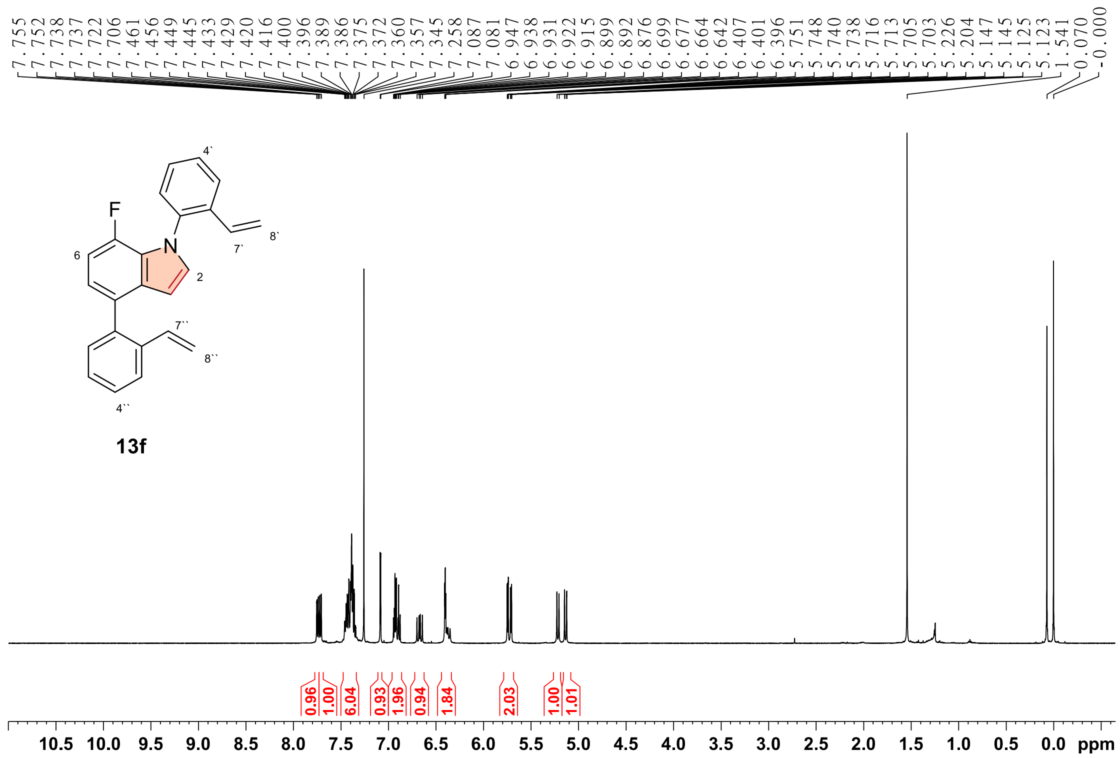

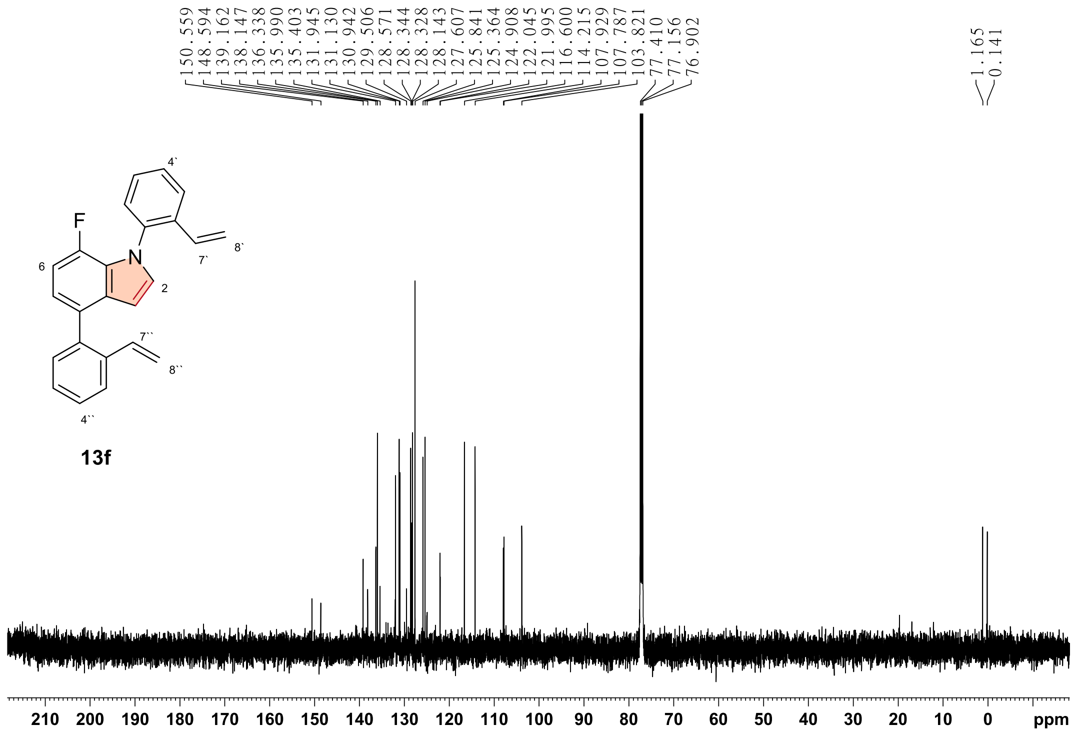

3-Methyl-1,4-bis(2-vinylphenyl)-1*H*-indole (13g):

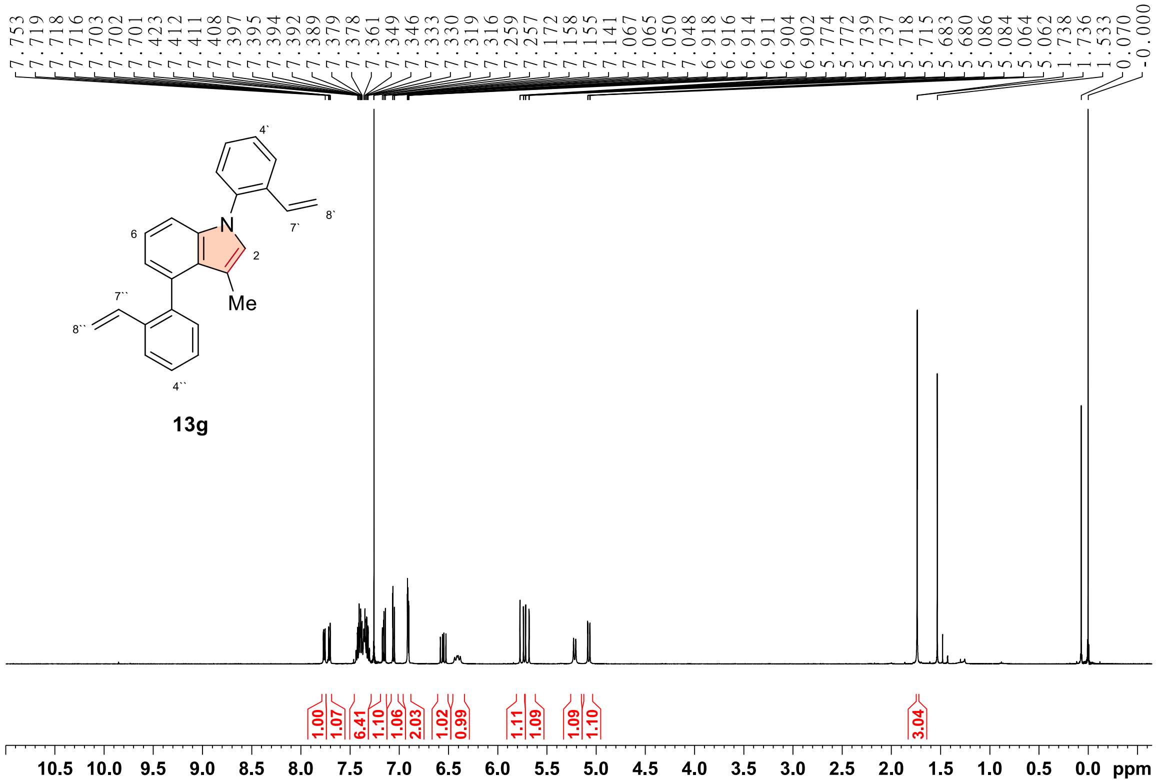

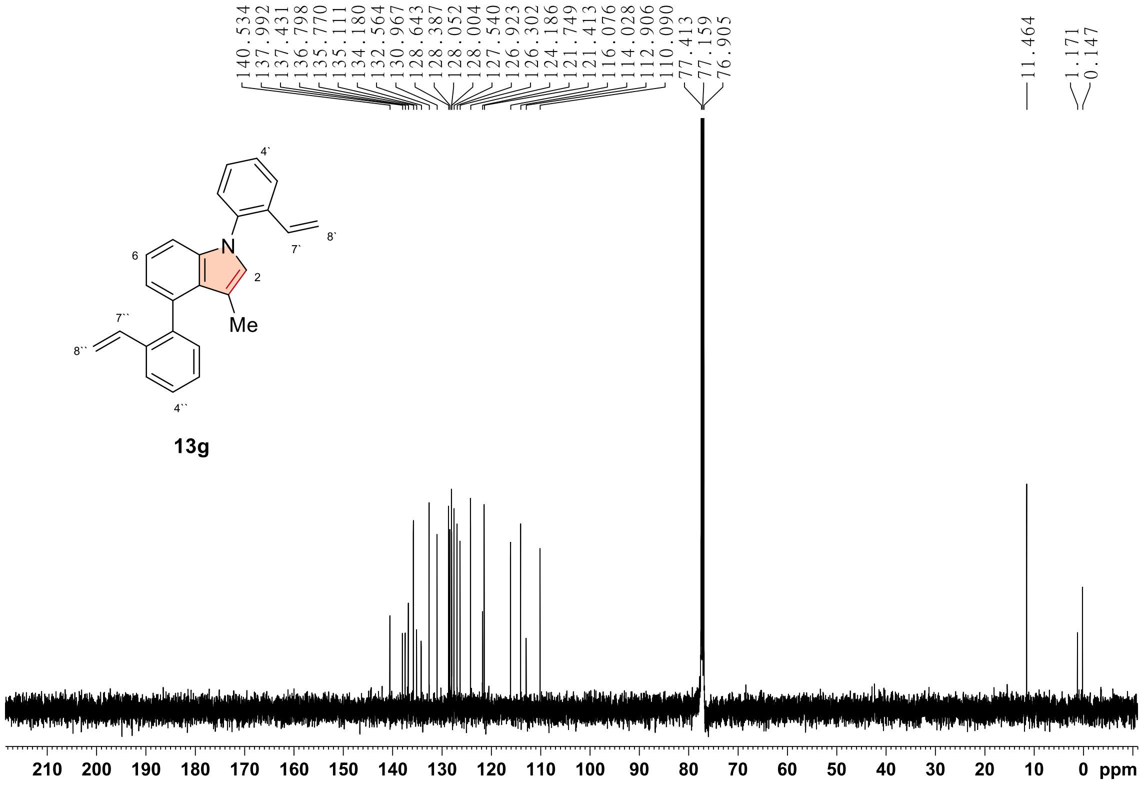

1,4-Bis(2-vinylphenyl)-1*H*-pyrrolo[2,3-c]pyridine (13h):

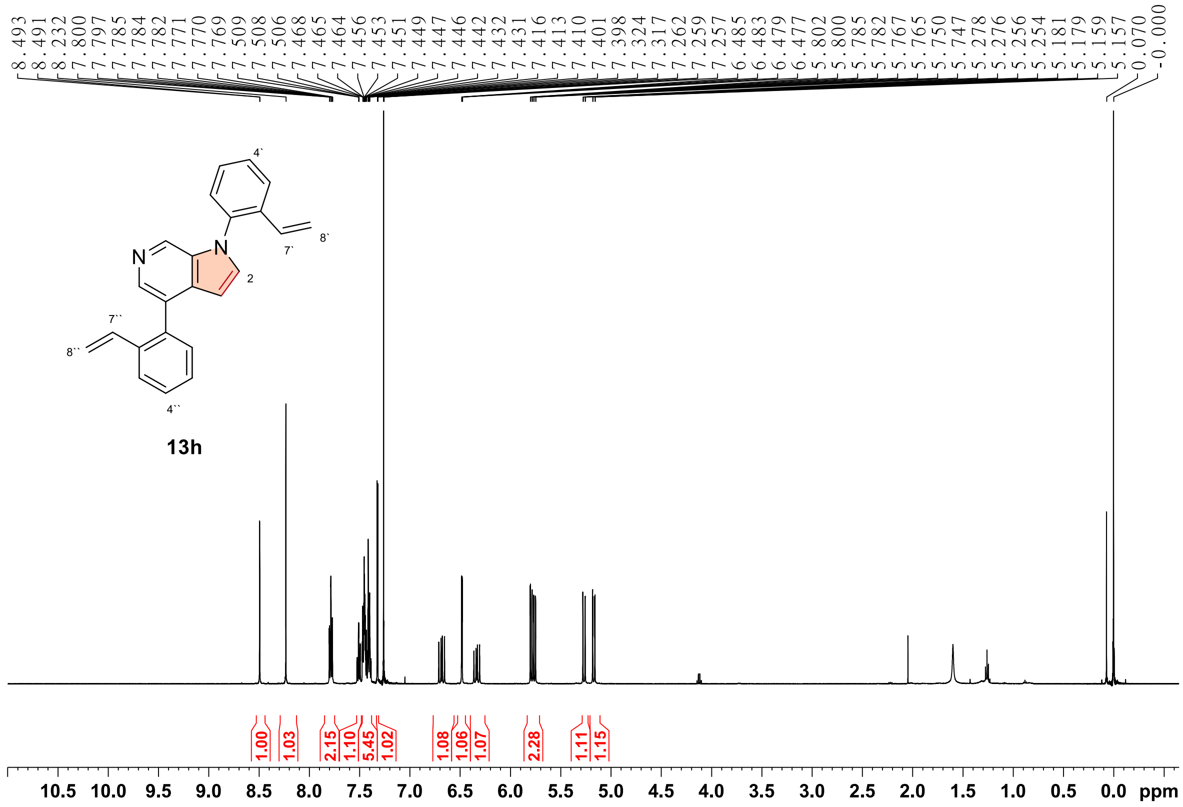

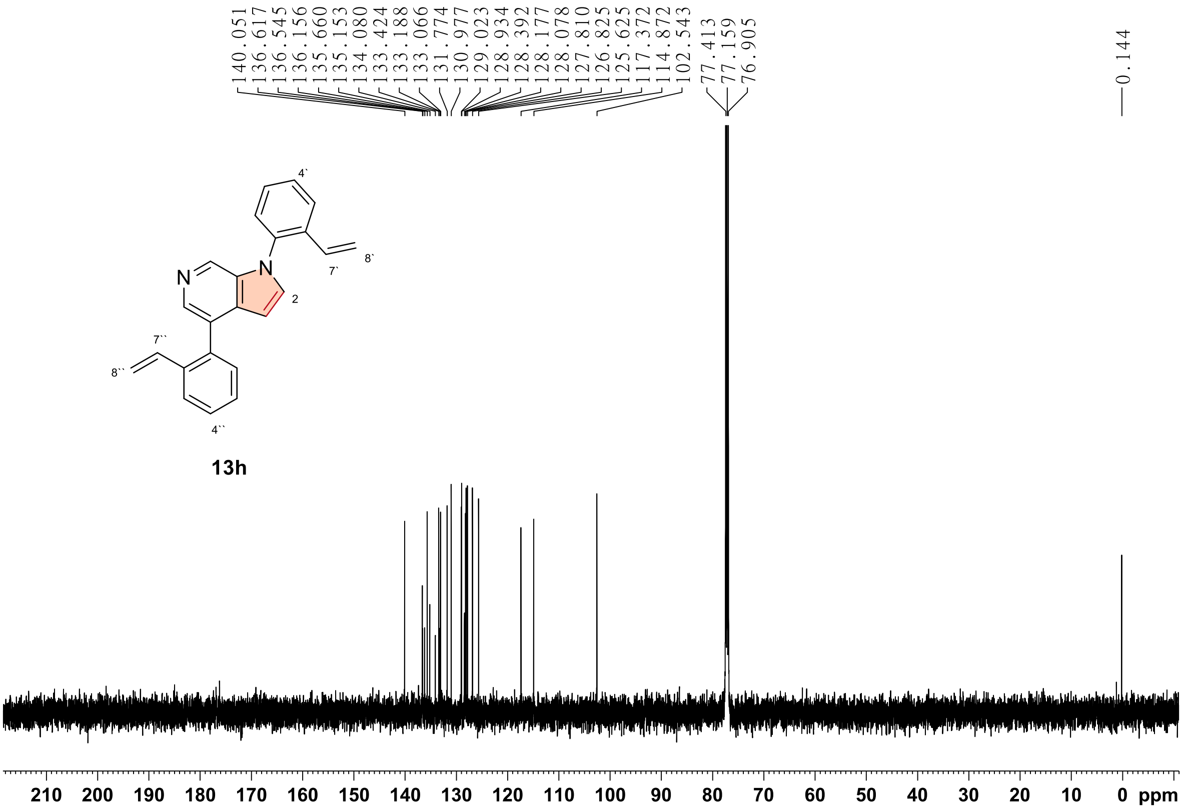

1-(1-(2-Formylphenyl)-1*H*-indol-4-yl)-2-naphthaldehyde (S8m):

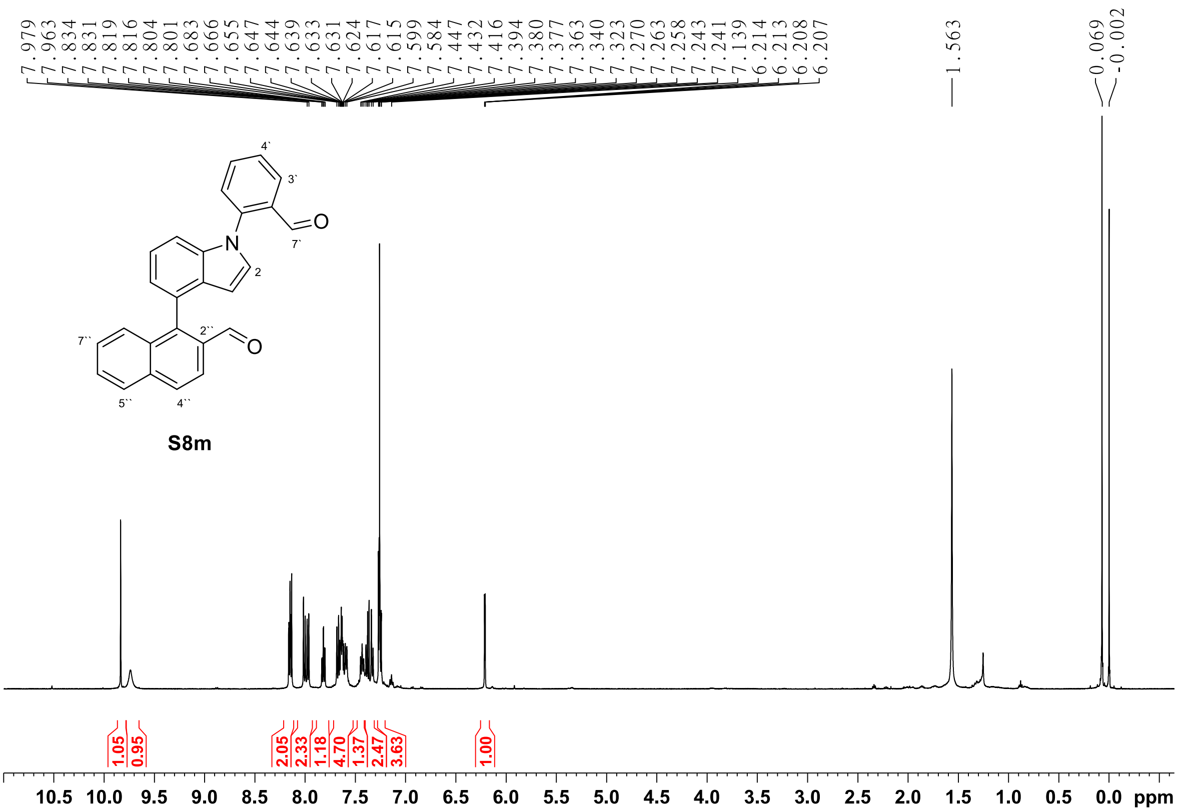

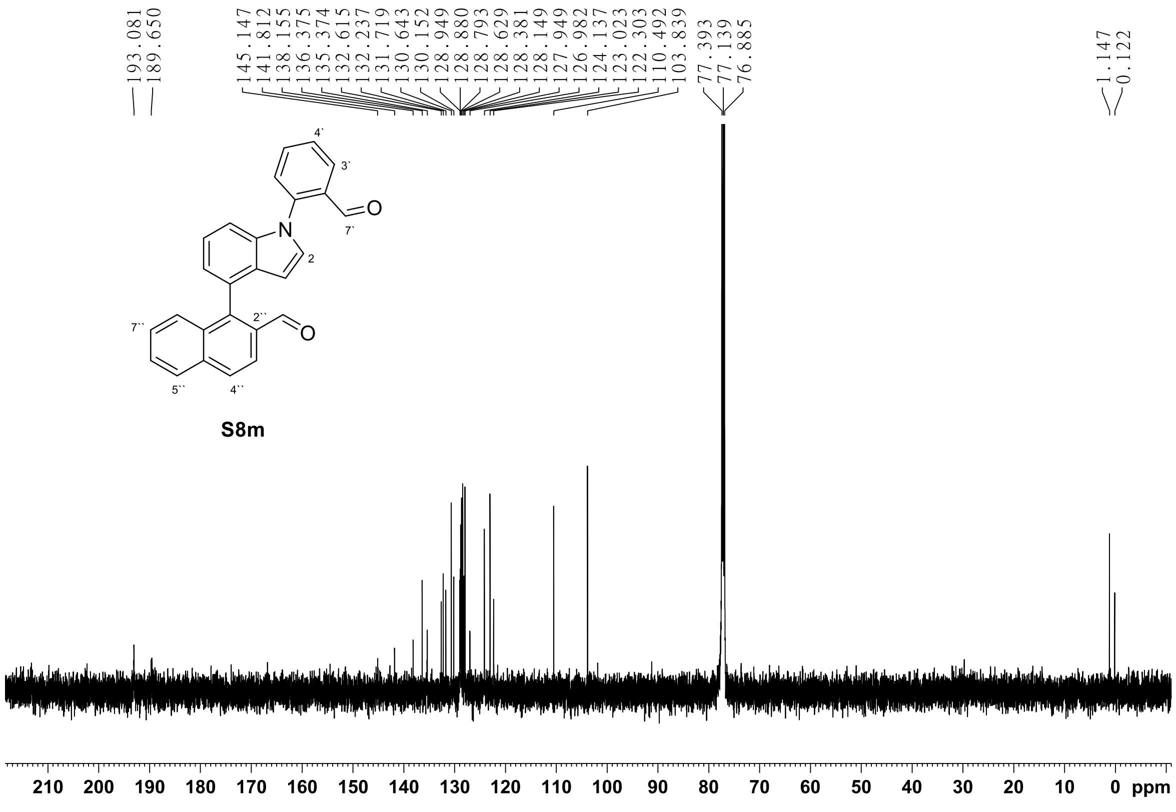

4-(2-Vinylnaphthalen-1-yl)-1-(2-vinylphenyl)-1H-indole (13i):

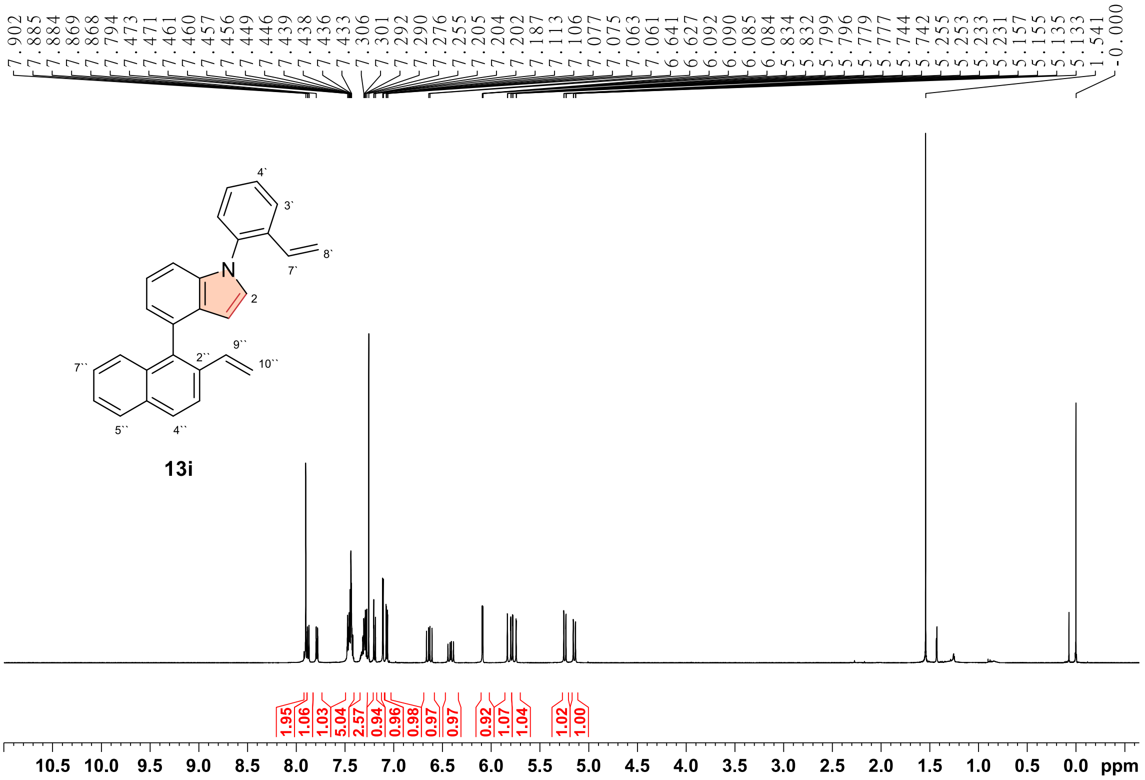

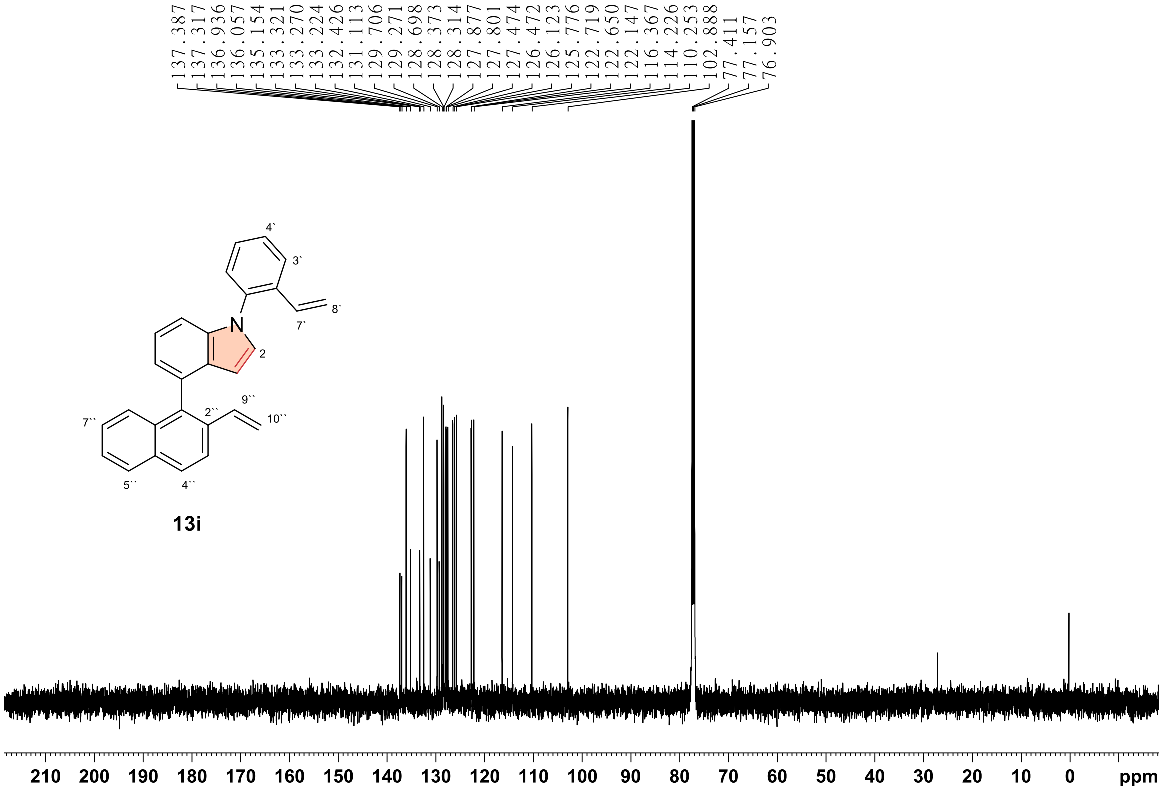

2-Methyl-1,4-bis(2-vinylphenyl)-1*H*-indole (19a):

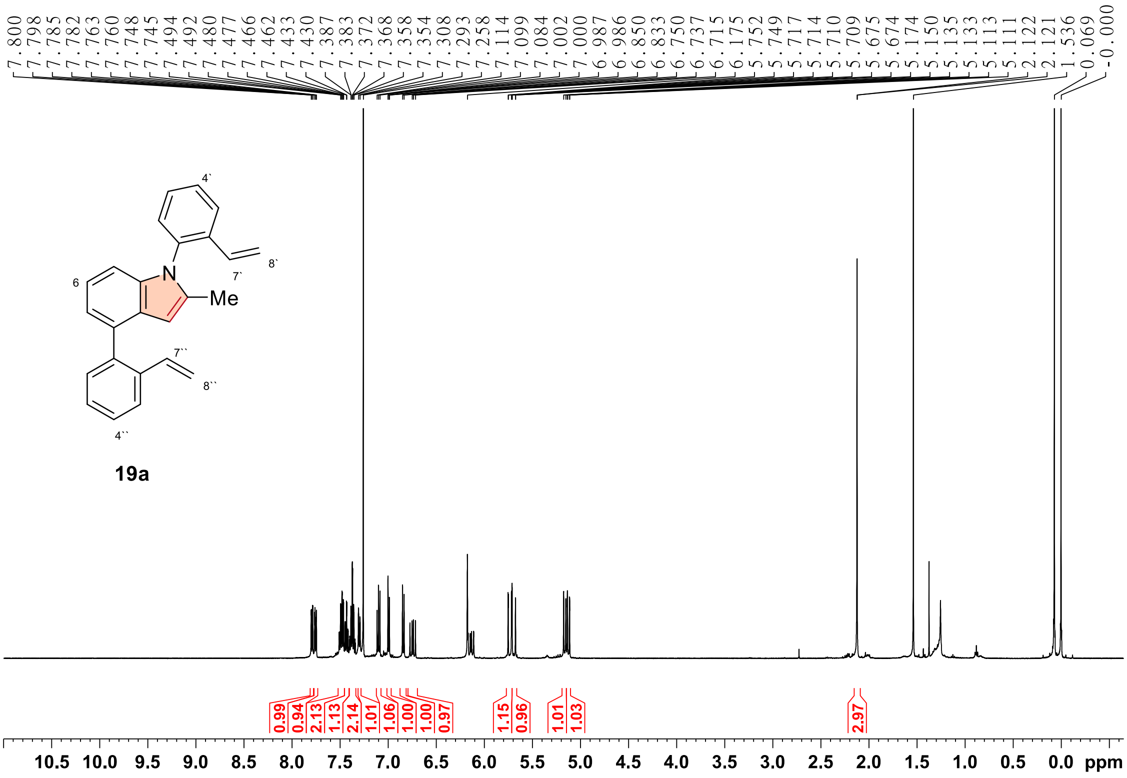

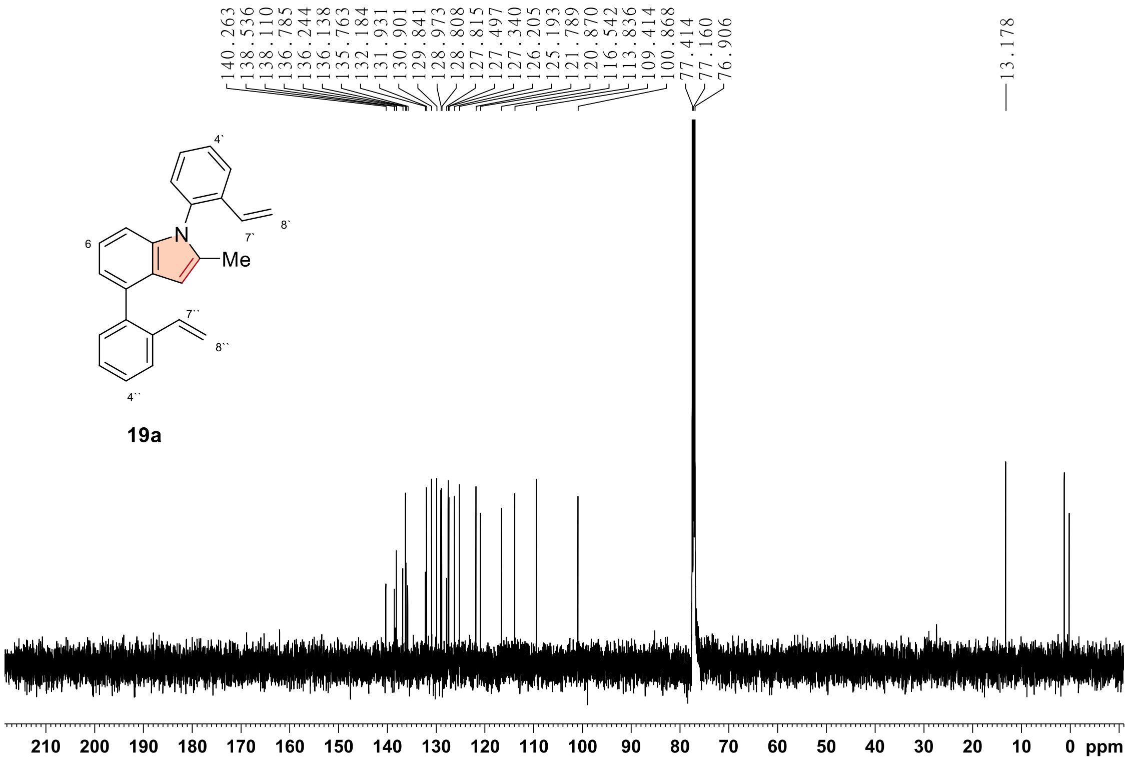

7-Methoxy-1,4-bis(2-vinylphenyl)-1*H*-indole (19b):

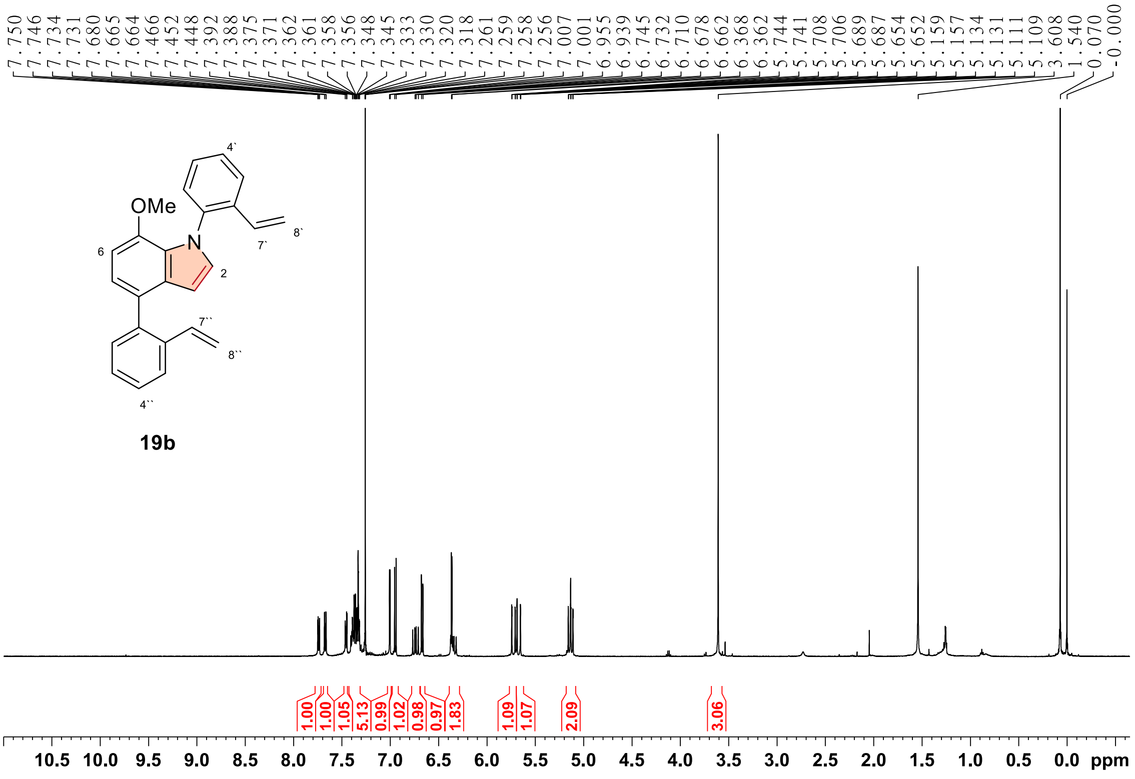

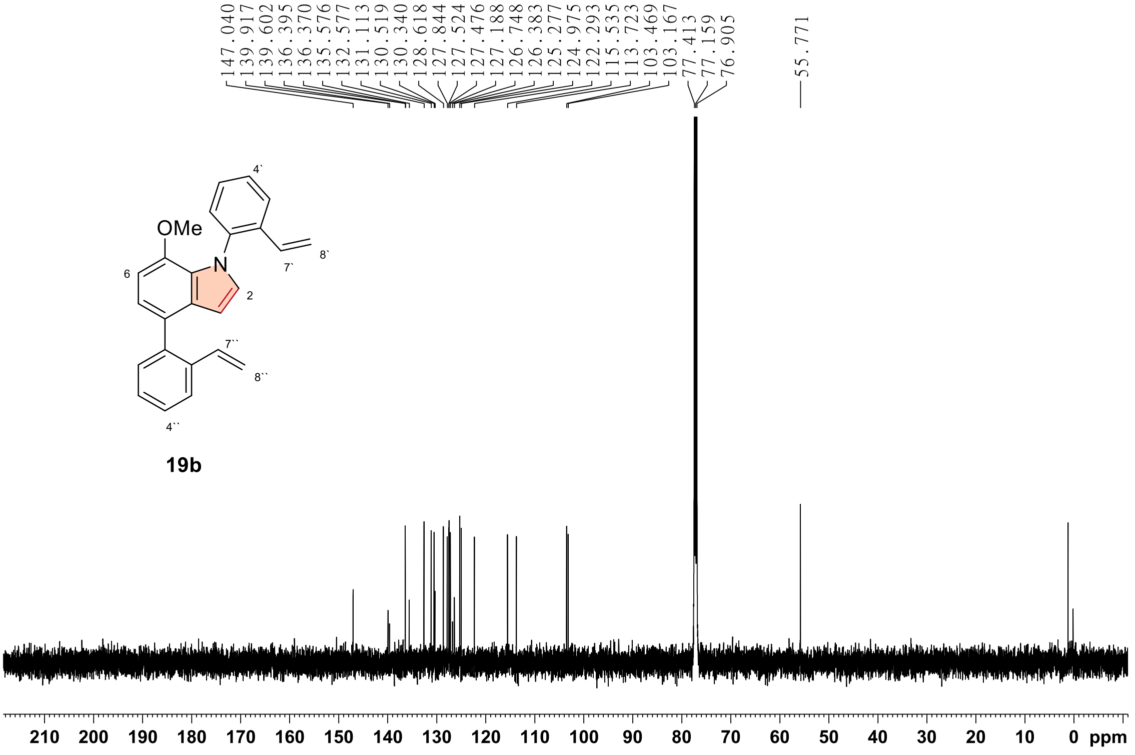

7-Methyl-1,4-bis(2-vinylphenyl)-1*H*-indole (19c):

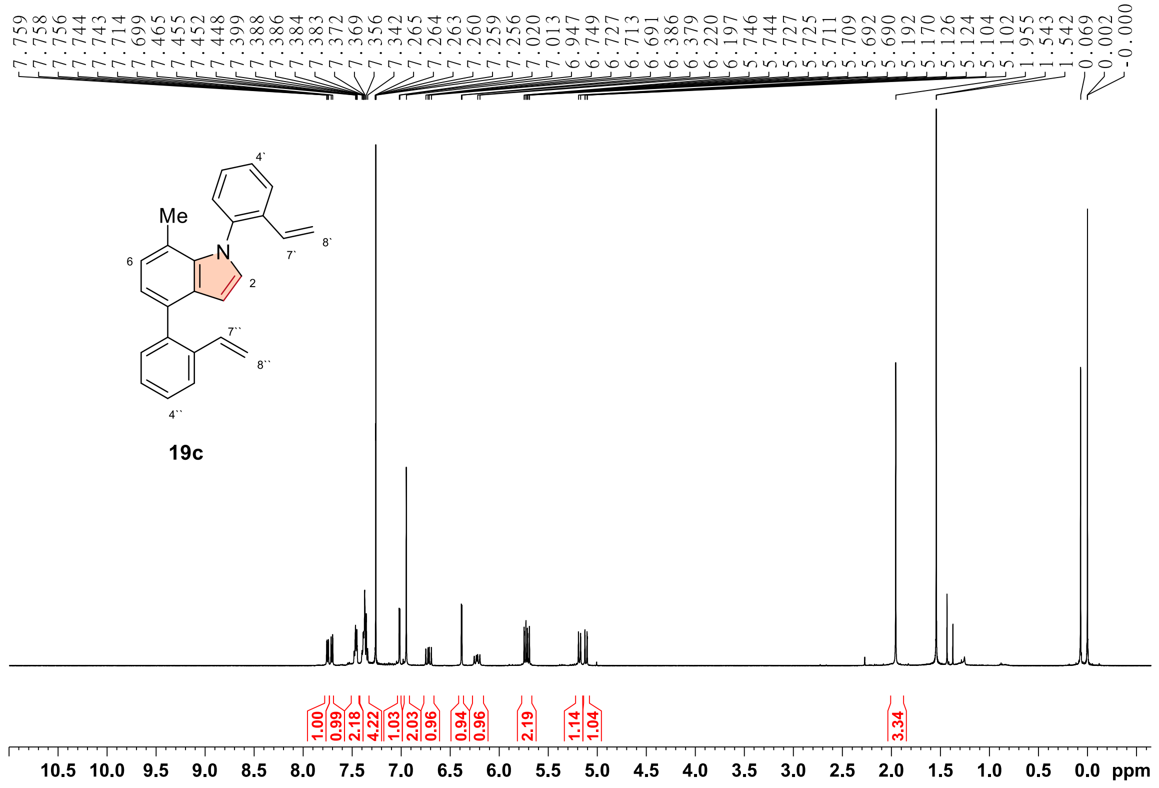

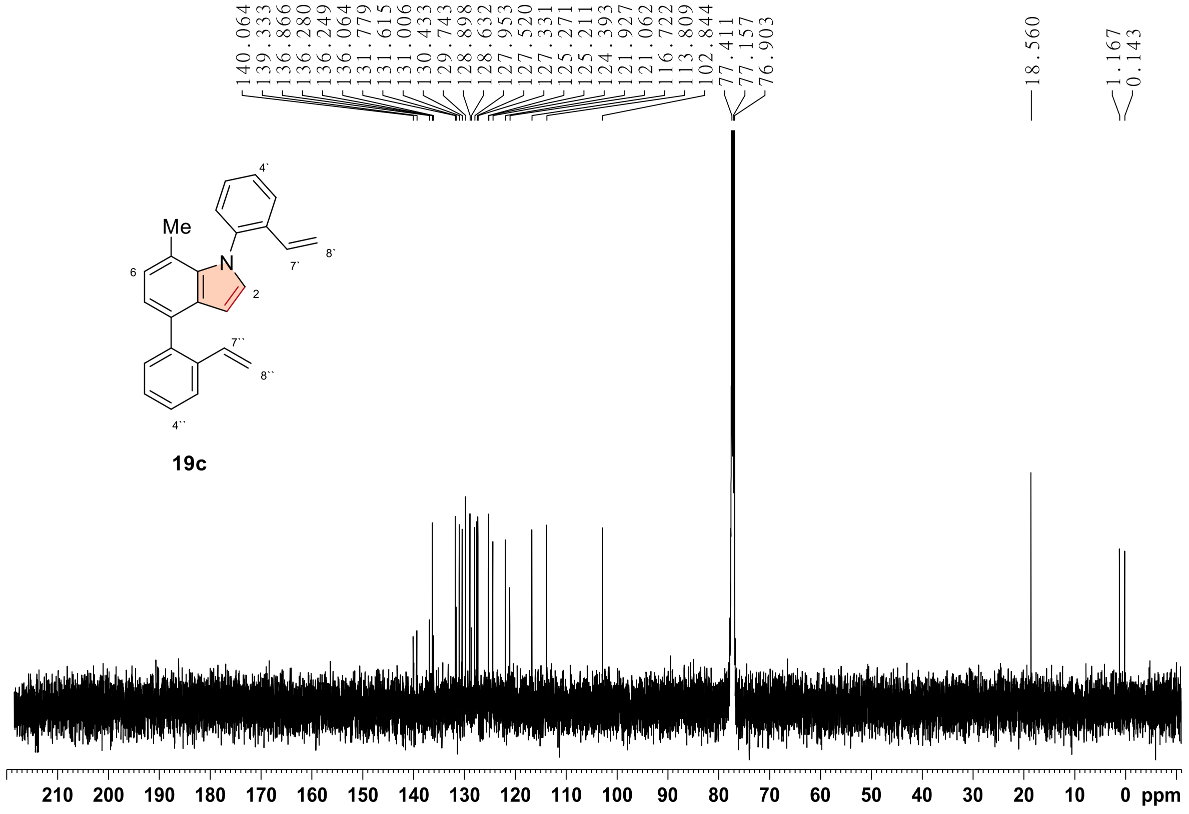

7-Chloro-1,4-bis(2-vinylphenyl)-1*H*-indole (19d):

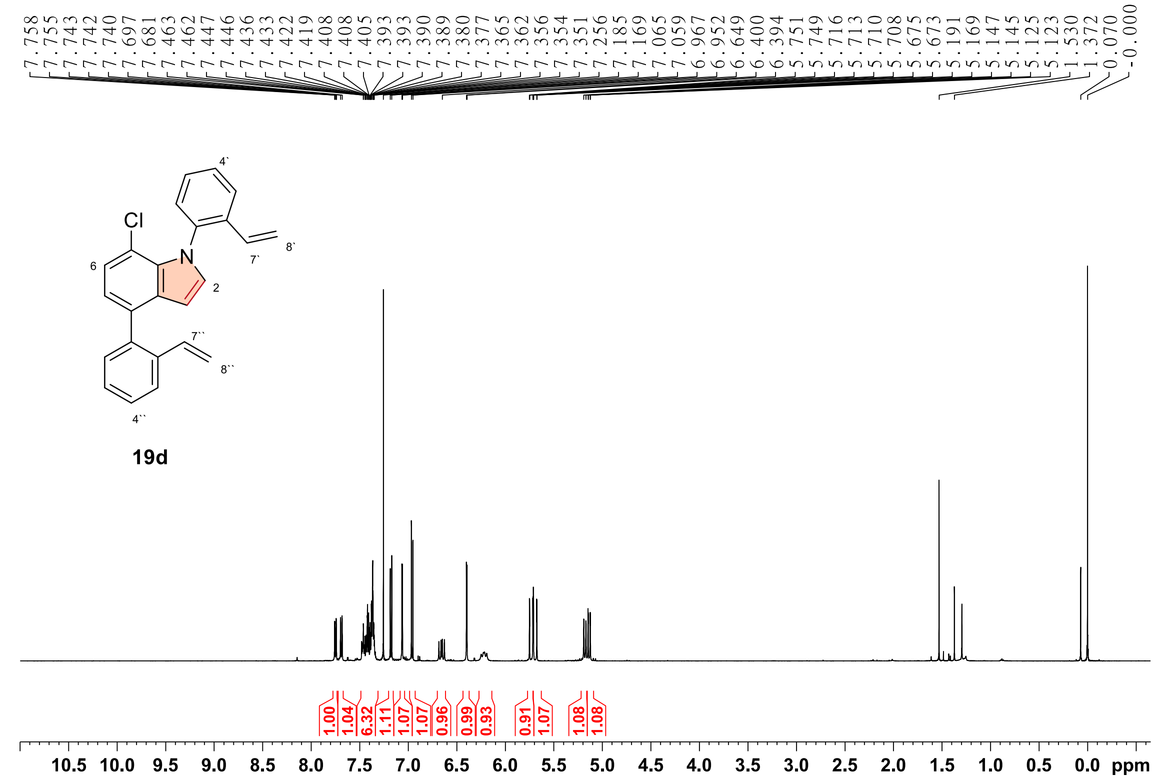

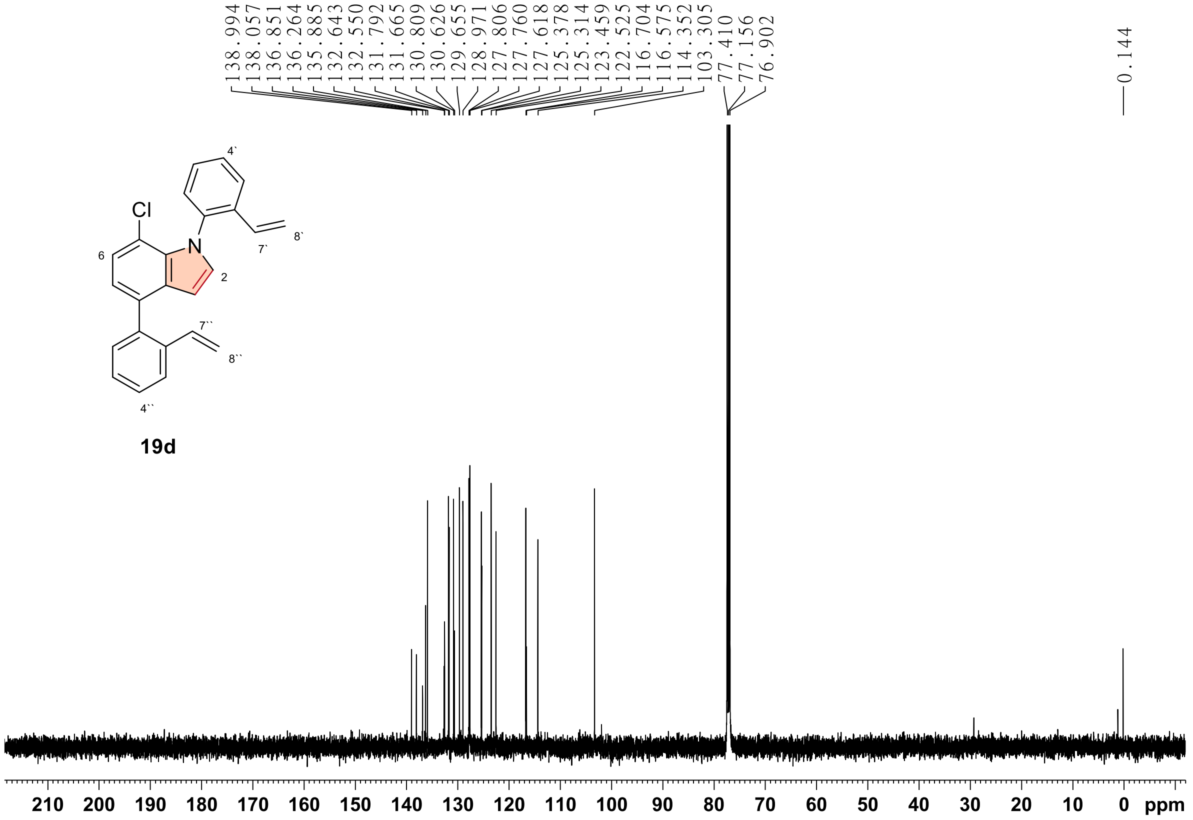

7-Chloro-4-(5-chloro-2-vinylphenyl)-1-(2-vinylphenyl)-1*H*-indole (19e):

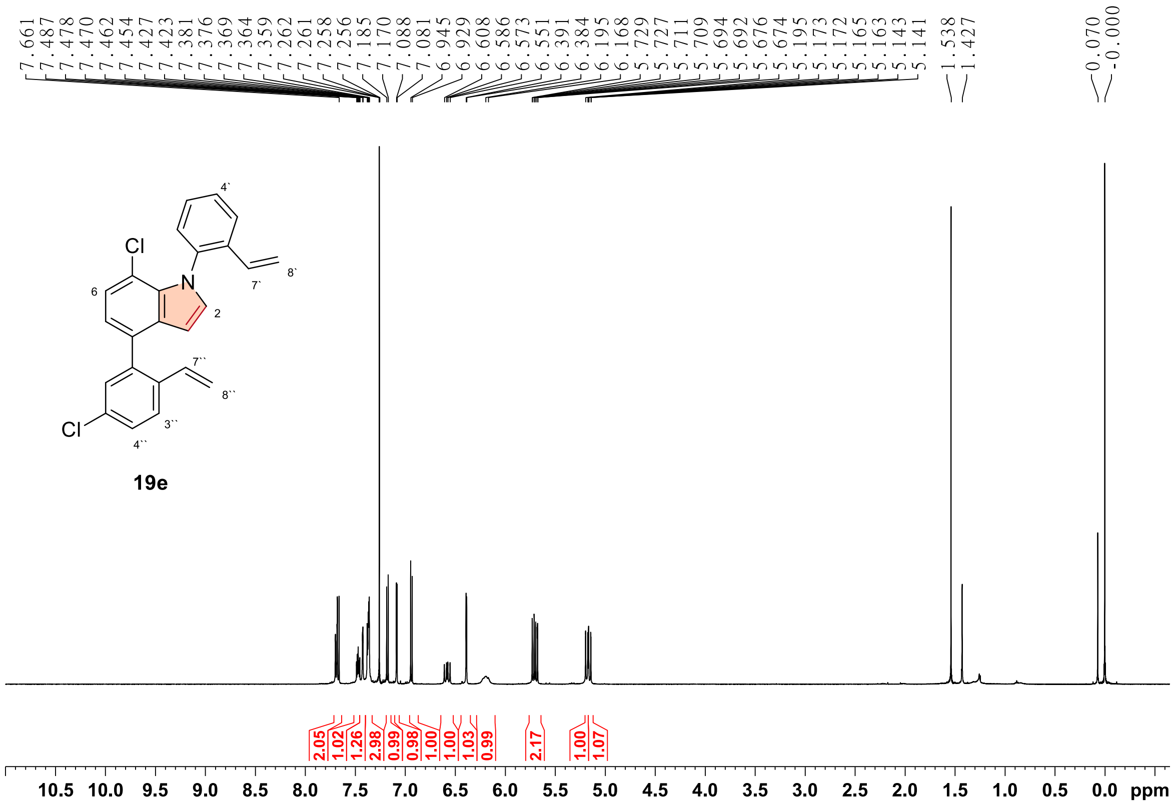

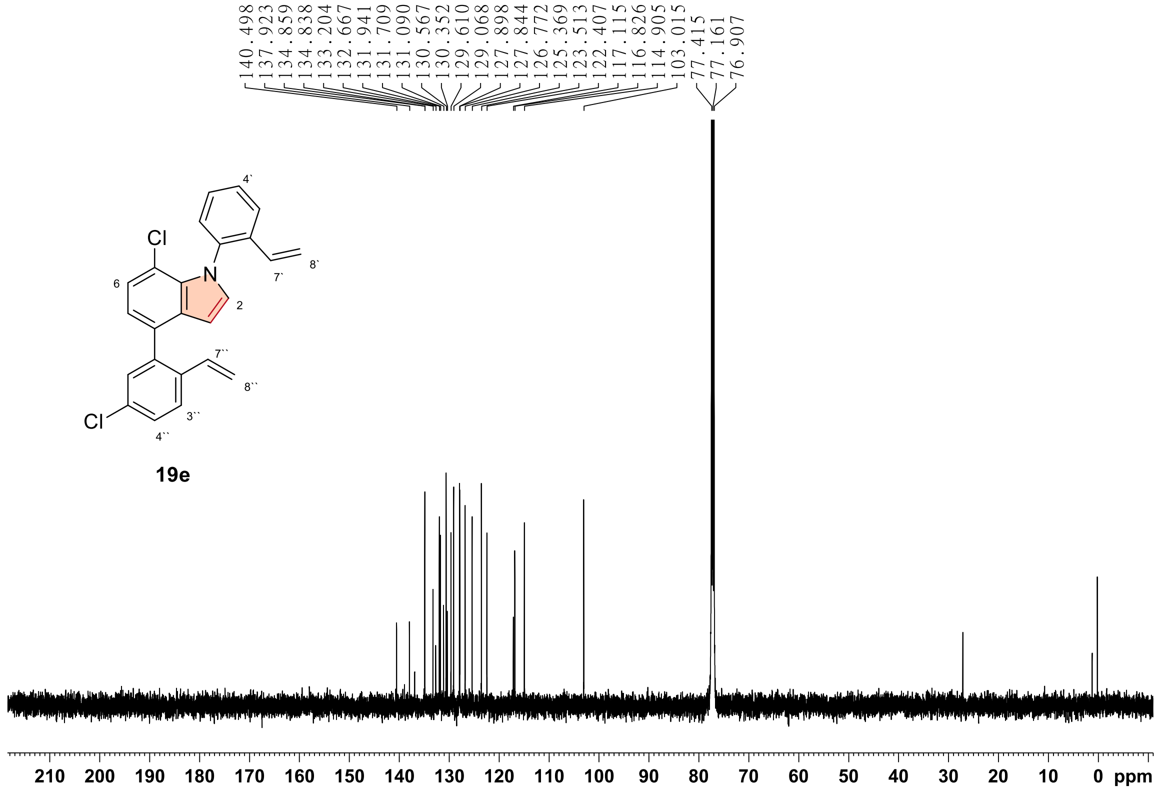

1-Bromo-5,7-dimethoxy-4-methylphenanthrene (S9a):

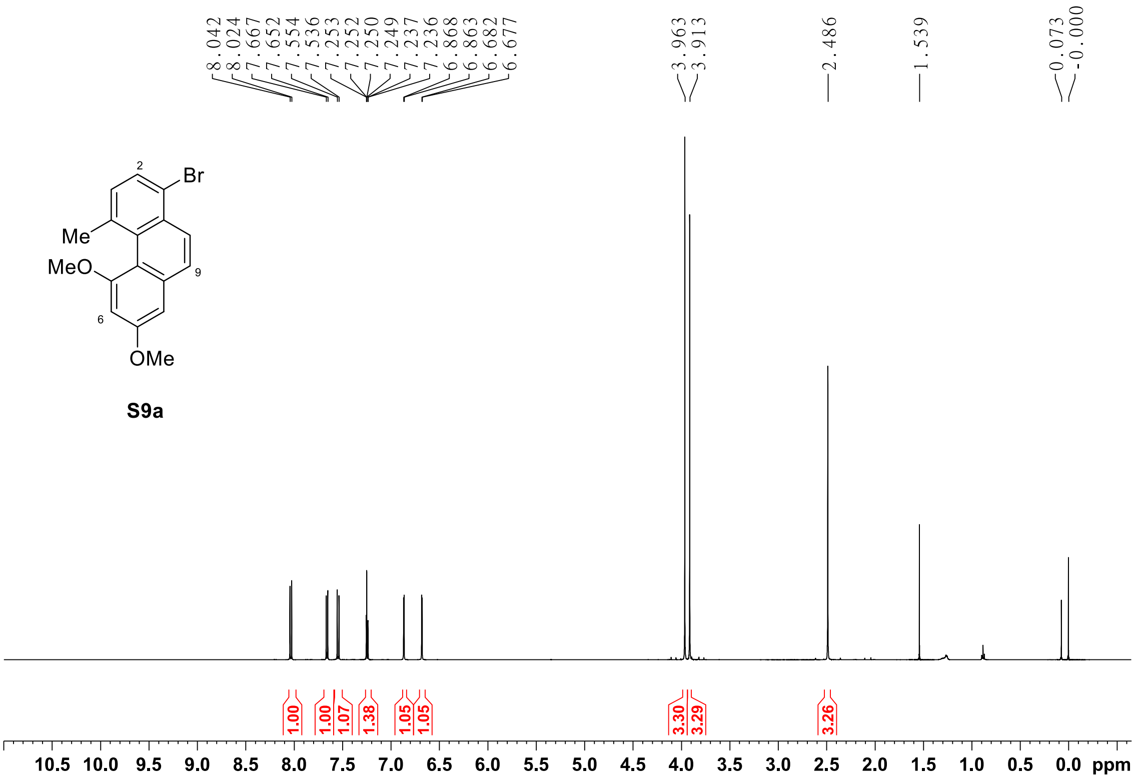

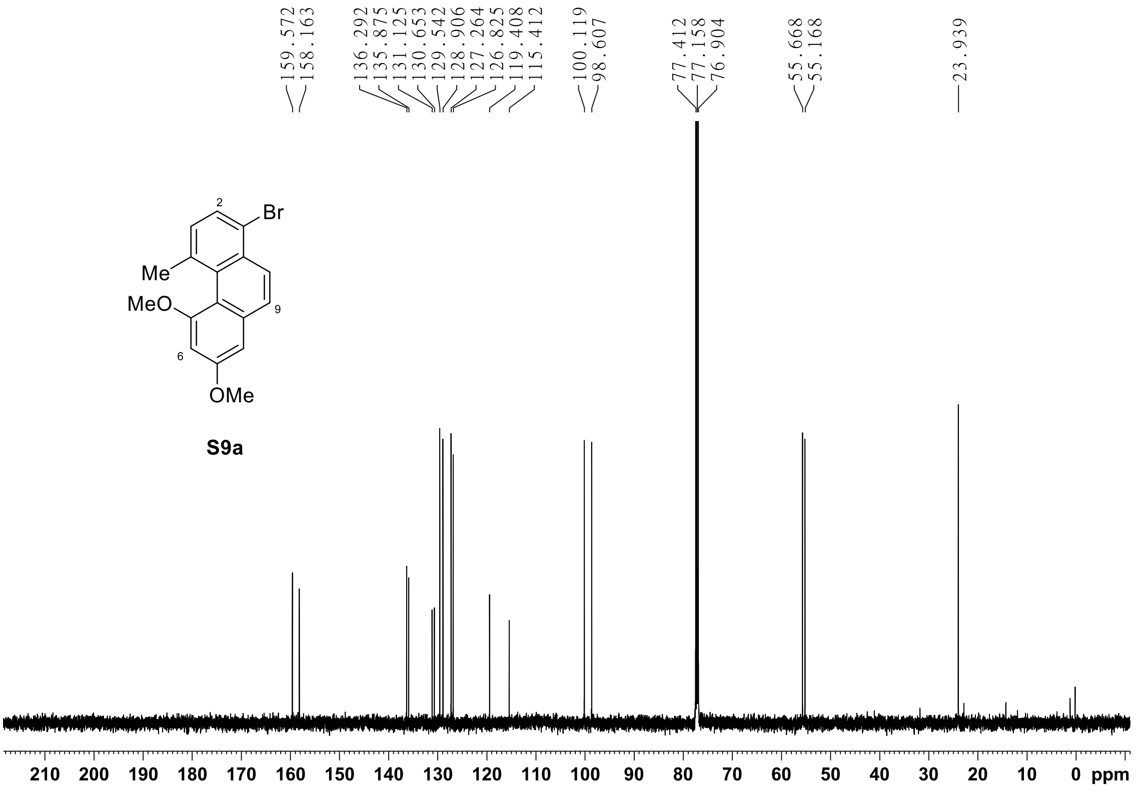

5-Bromo-9,11-dimethylphenanthro[3,4-d][1,3]dioxole (S9b):

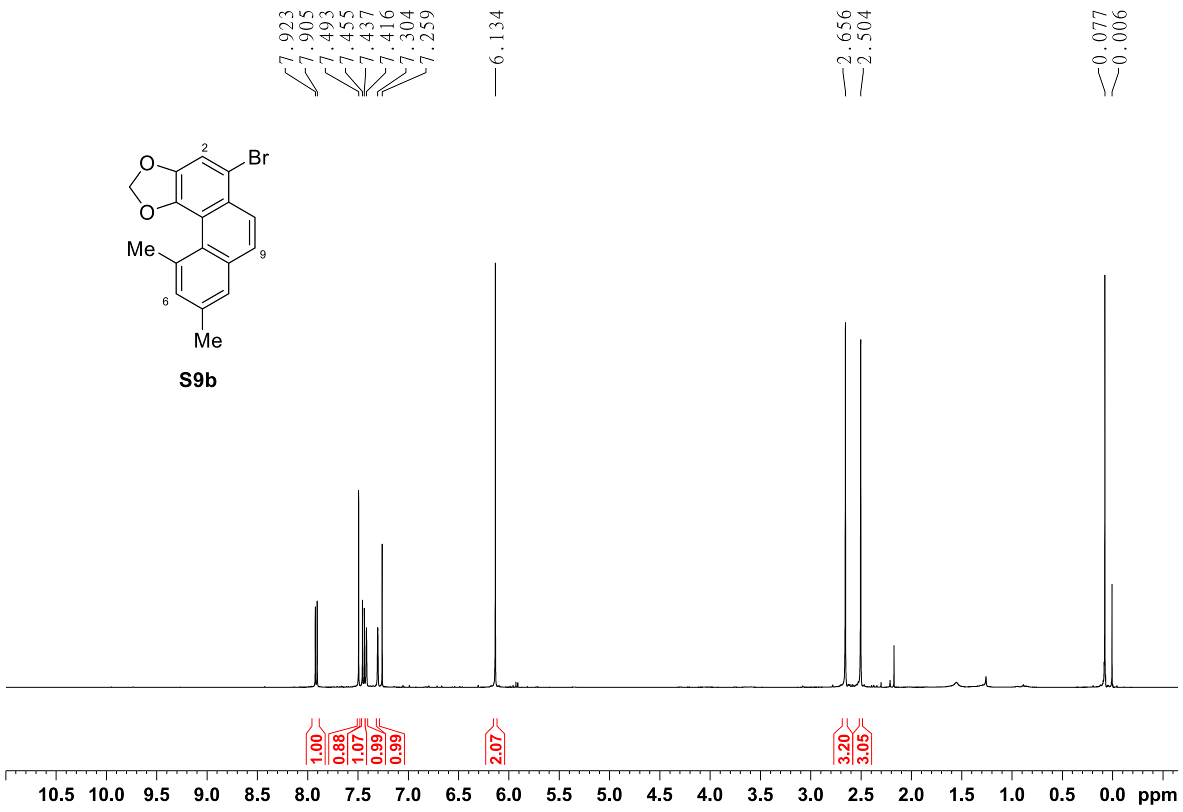

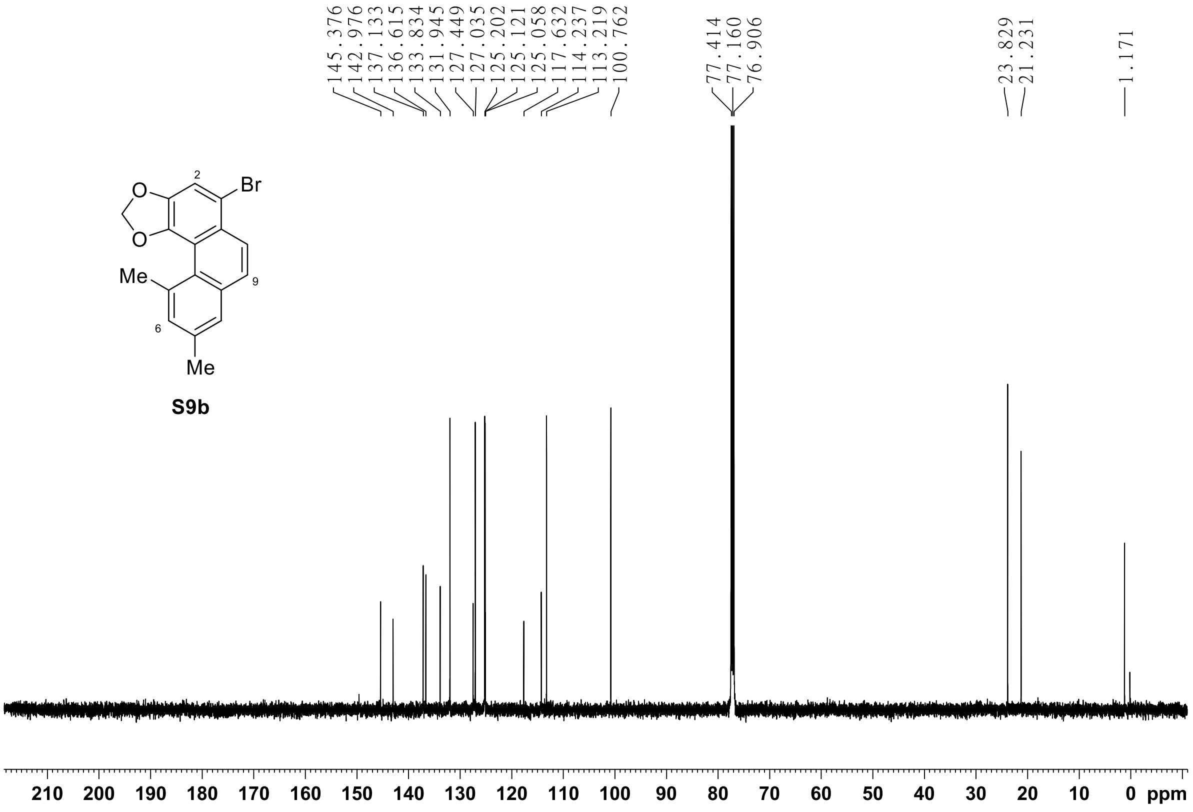

1-Bromo-5,7-dimethoxyphenanthrene (S9c):

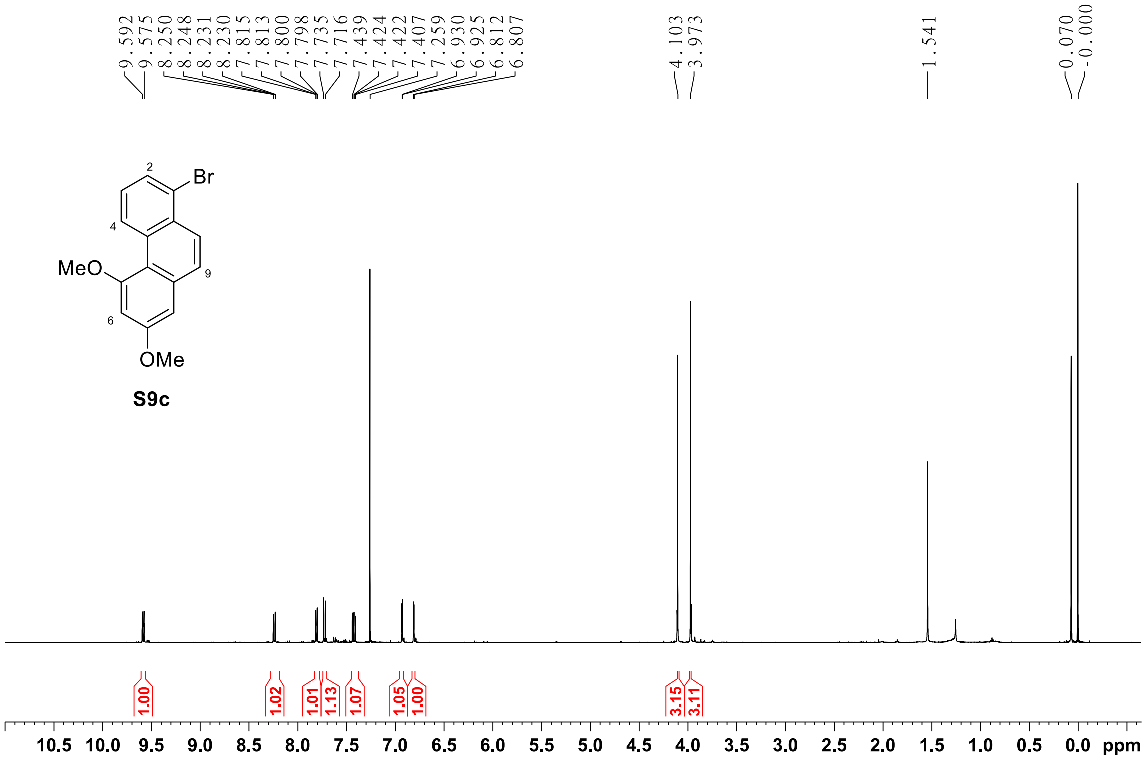

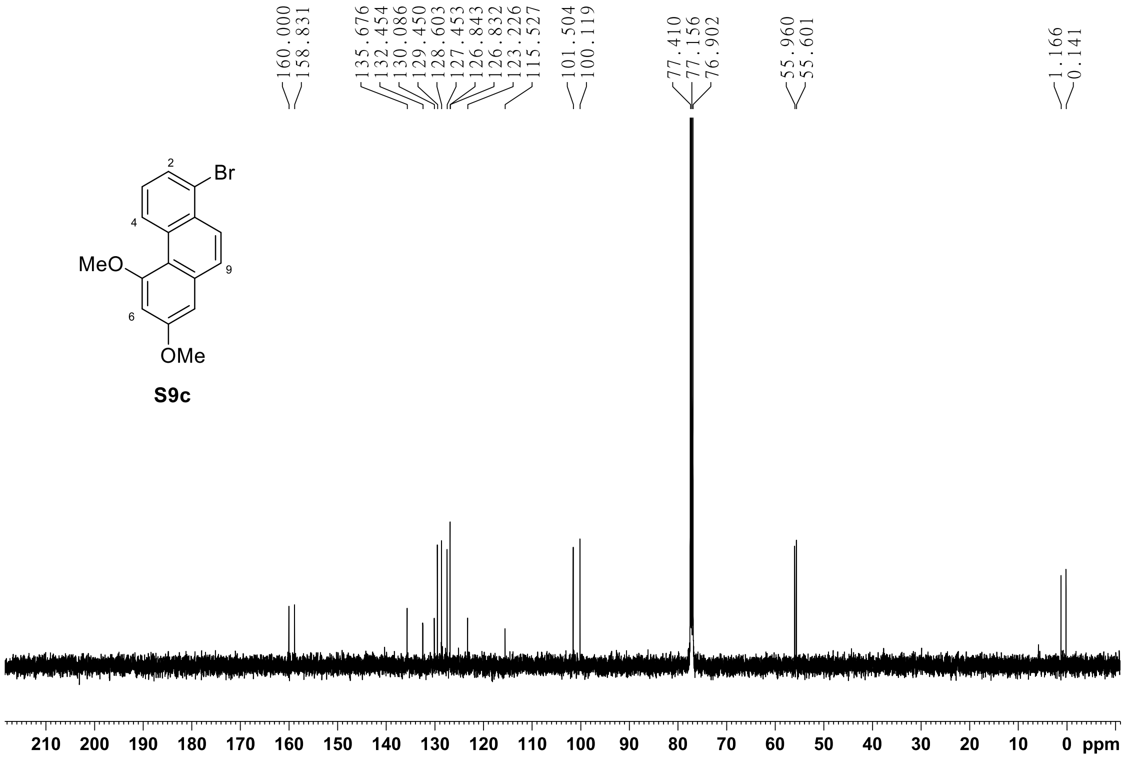

1-Bromo-4,5,7-trimethoxyphenanthrene (S9d):

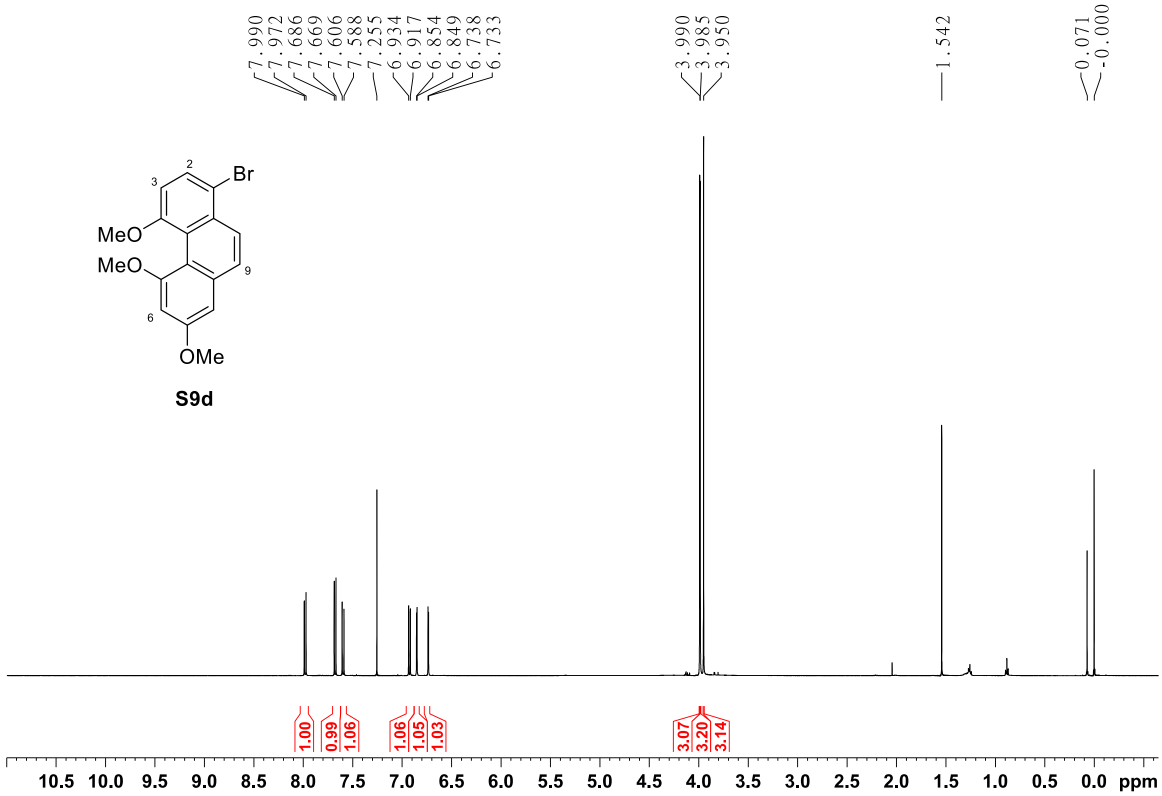

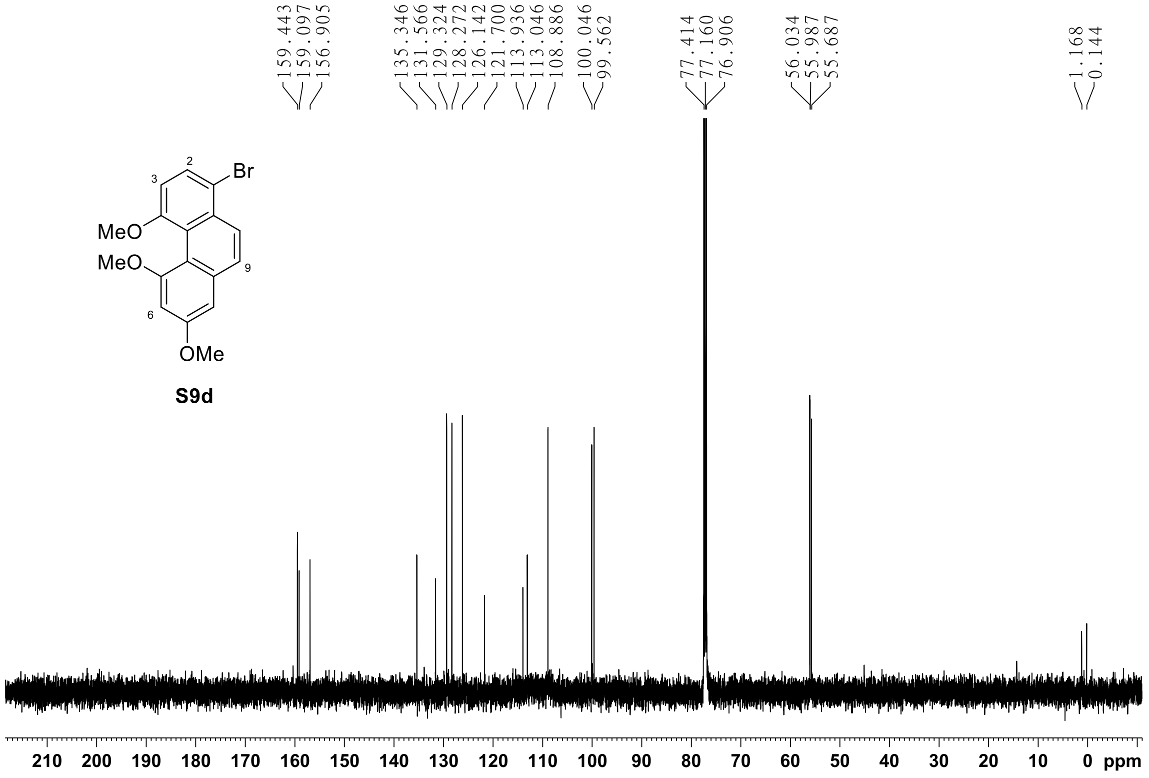

1-Bromo-5-methoxy-4,8-dimethylphenanthrene (S9e):

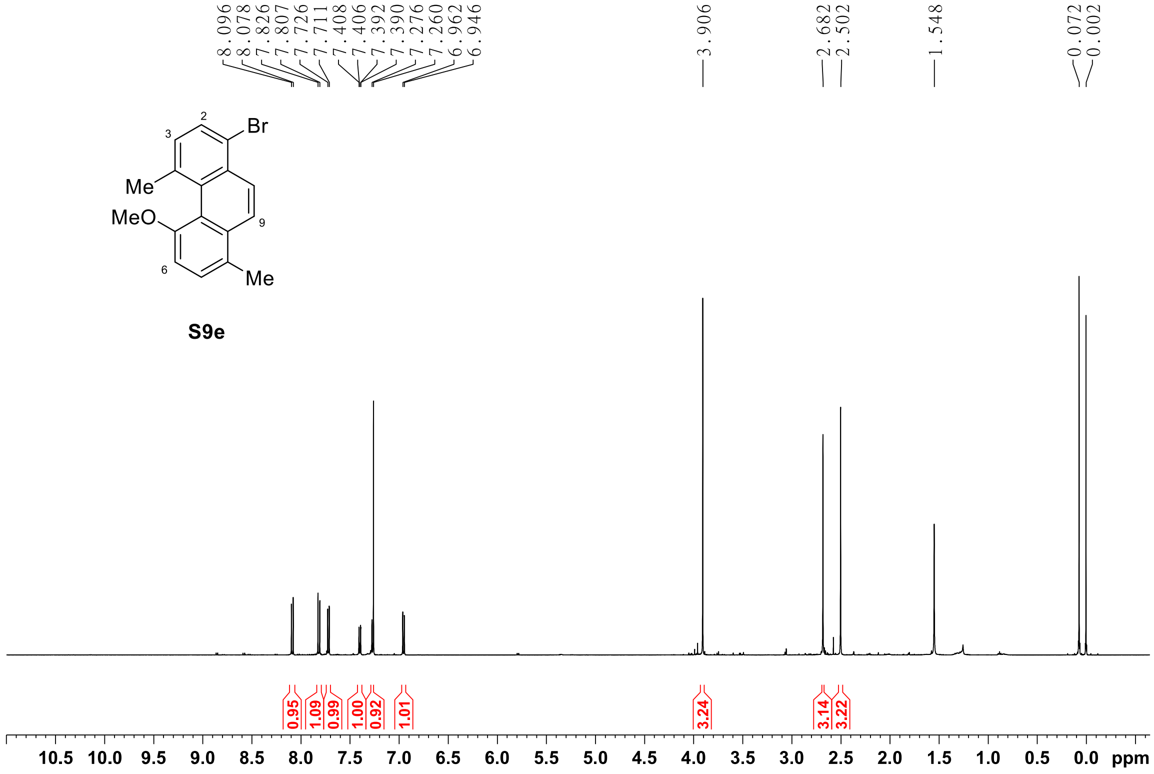

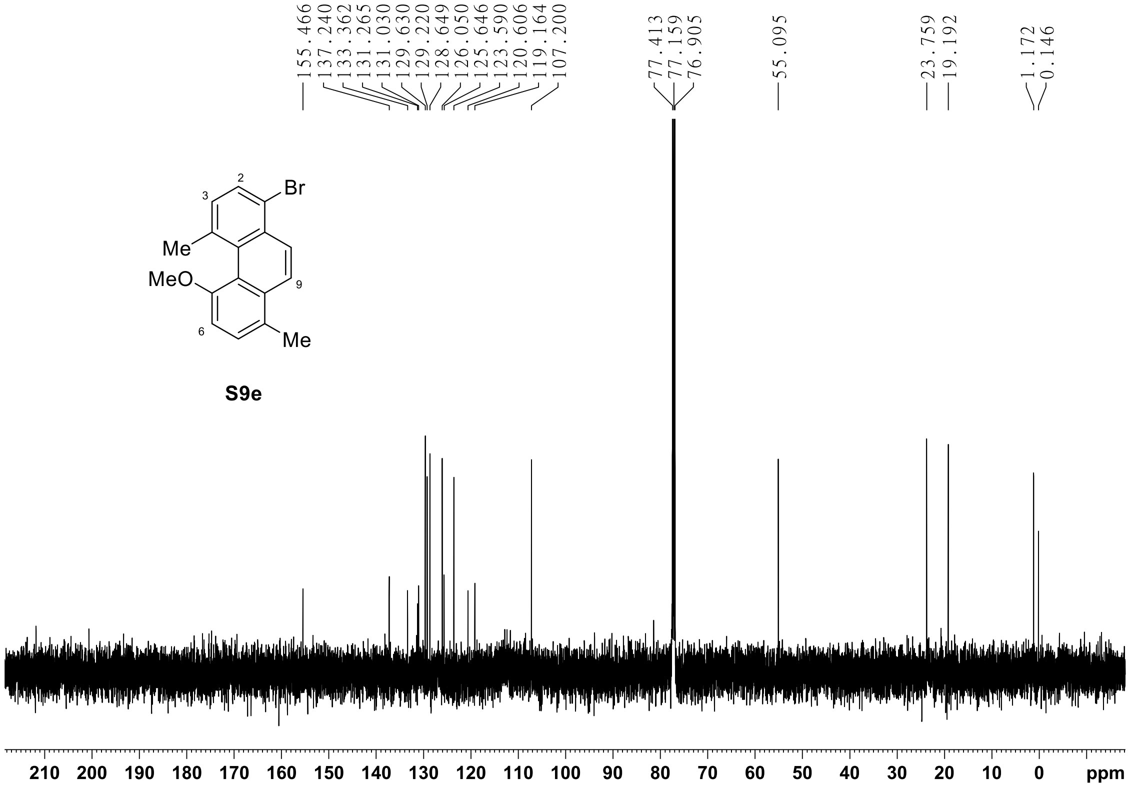

1-Bromo-4-methoxy-5,8-dimethylphenanthrene (S9f):

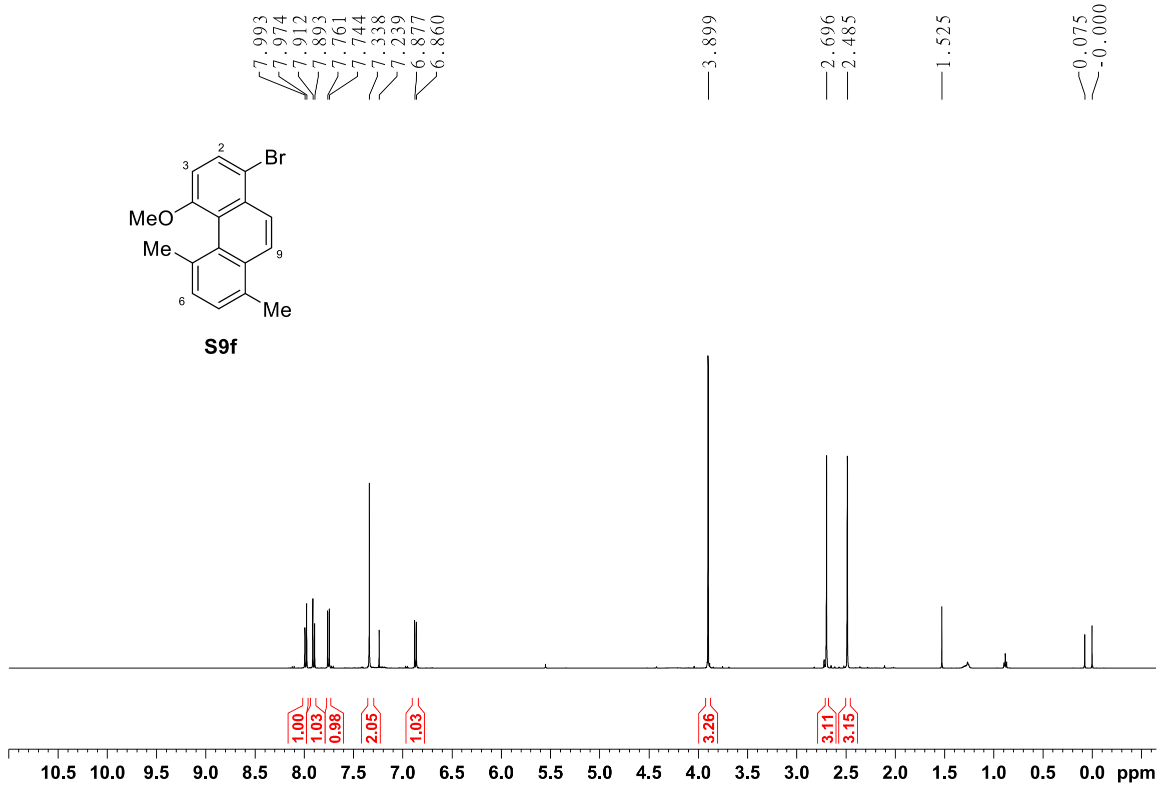

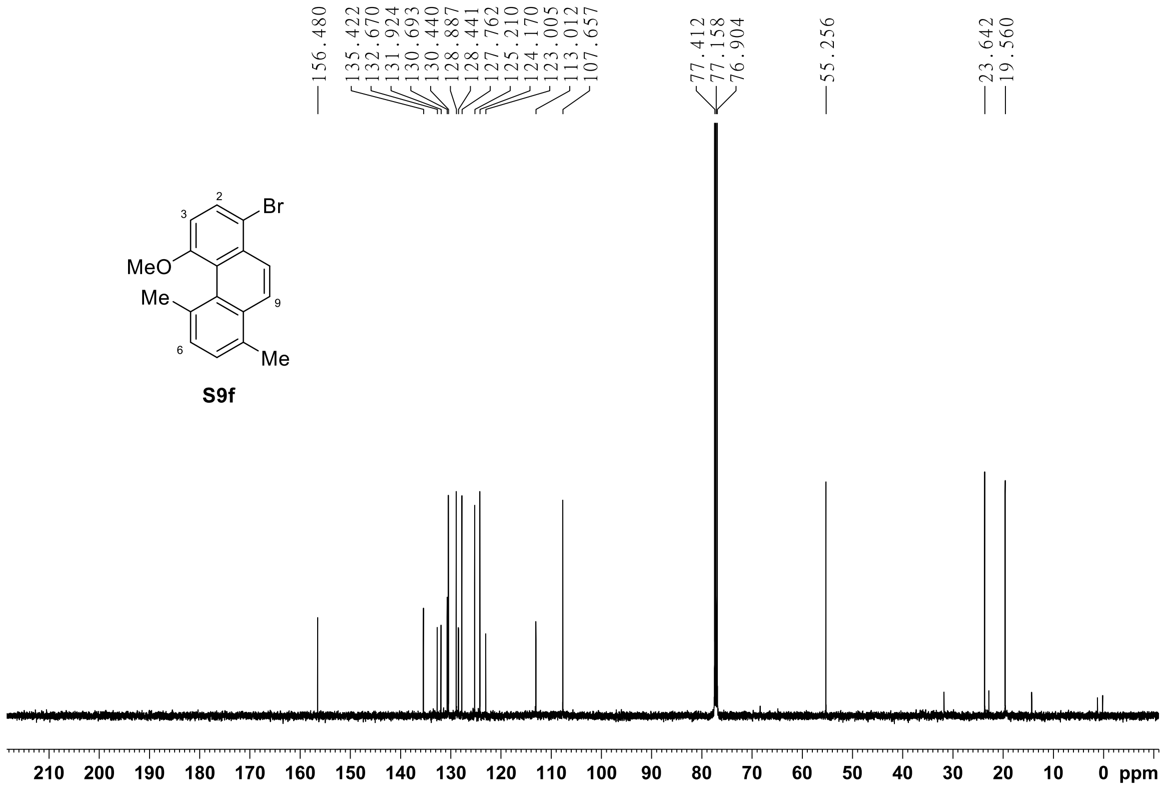

1,8-Dibromo-4,5-dimethoxyphenanthrene (S9g):

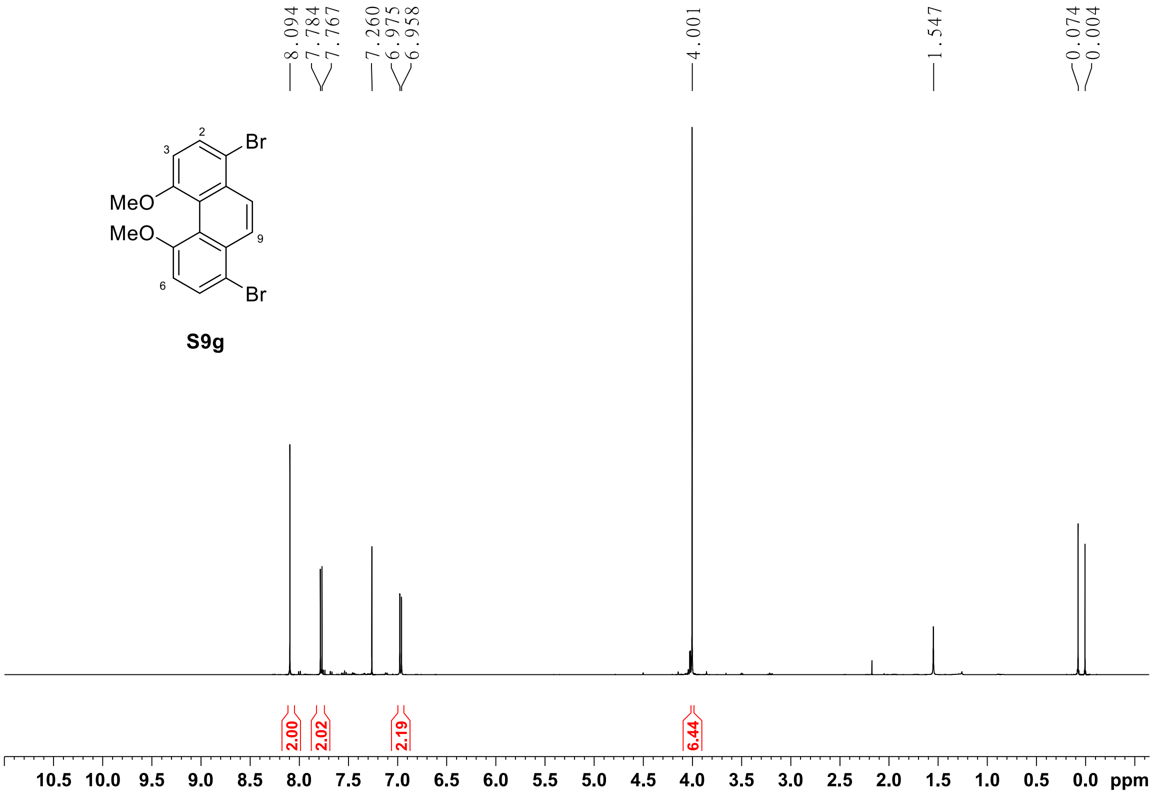

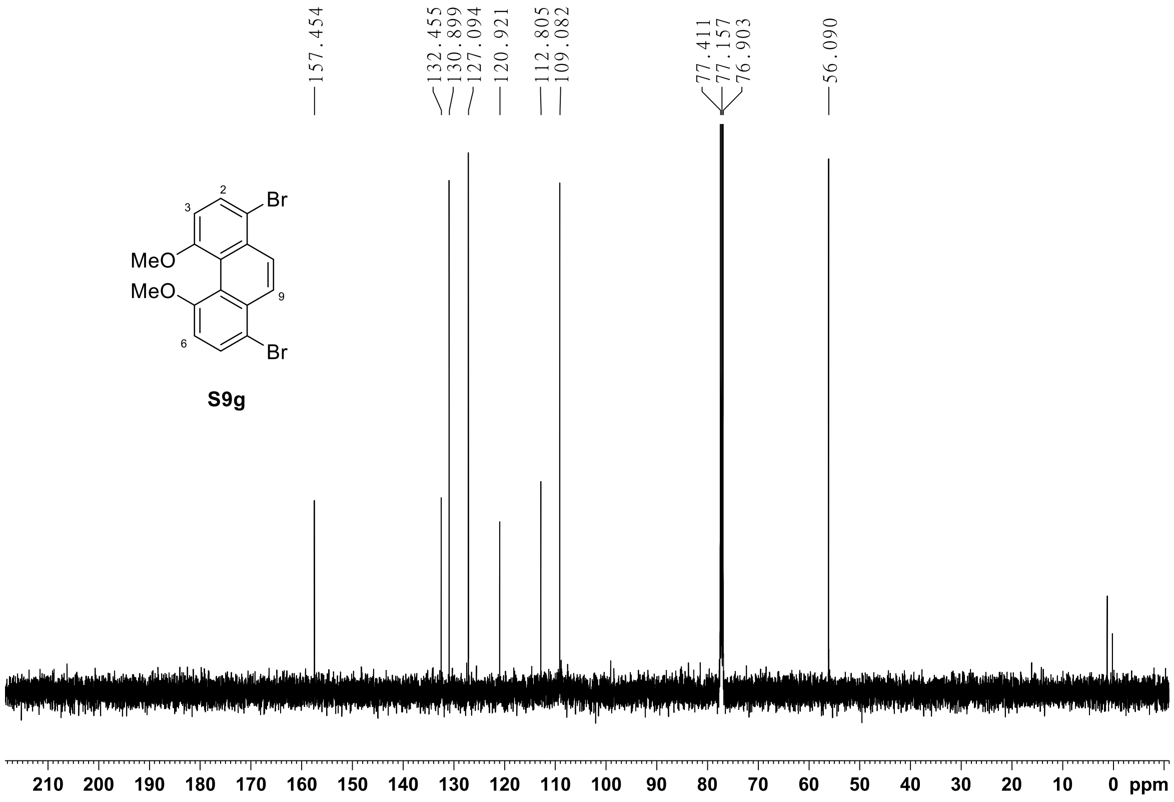

1-Methoxy-4,5,7-trimethylphenanthrene (S9h):

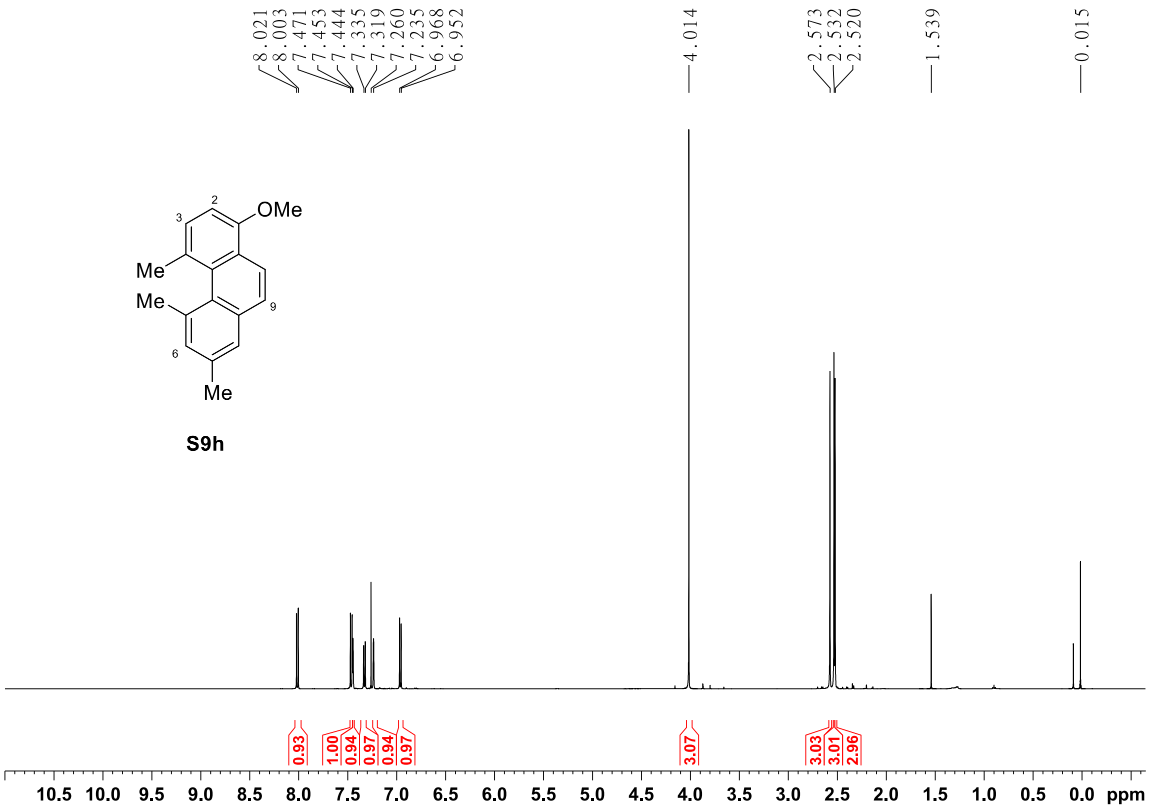

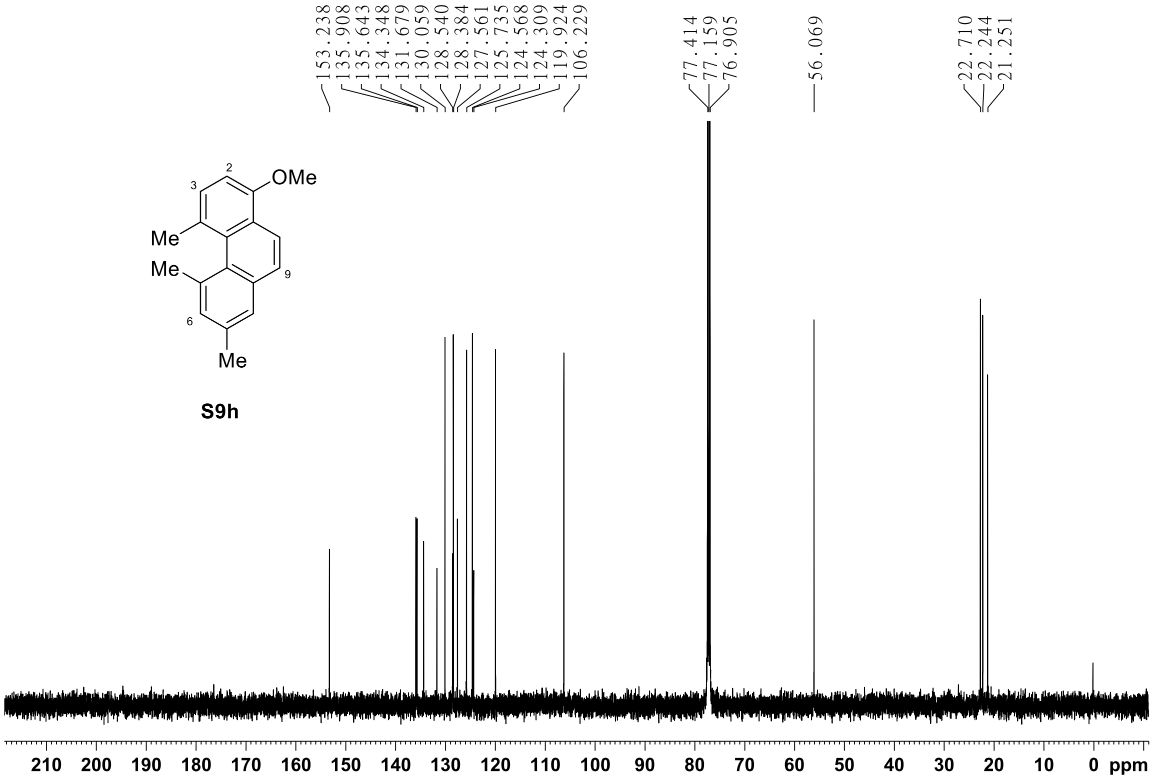

4,5,7-Trimethylphenanthren-1-ol (S9i):

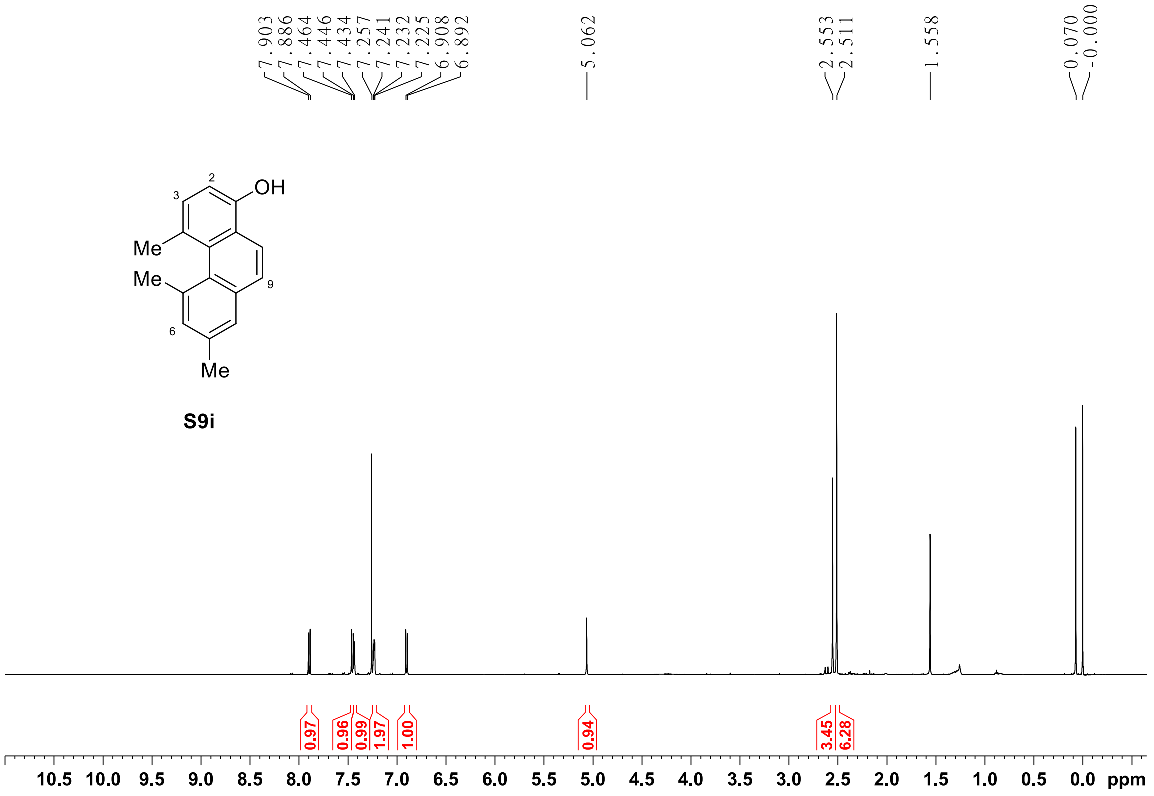

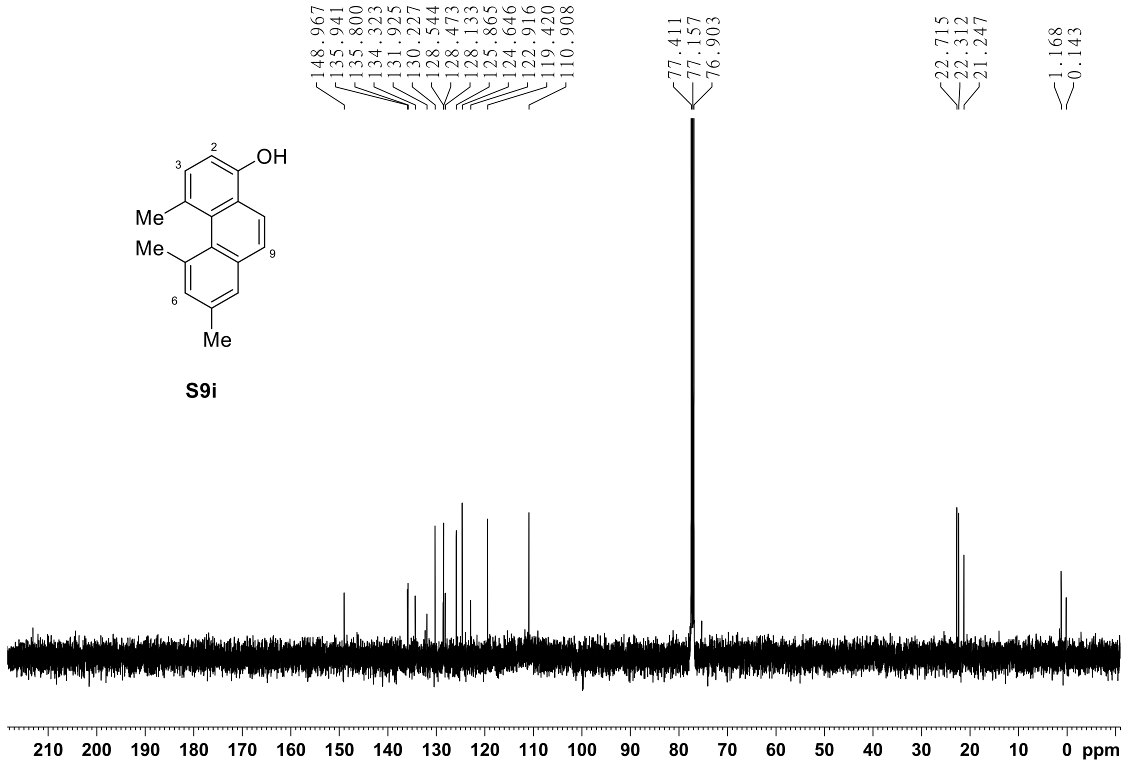

4,5,7-Trimethylphenanthren-1-yl trifluoromethanesulfonate (S9j):

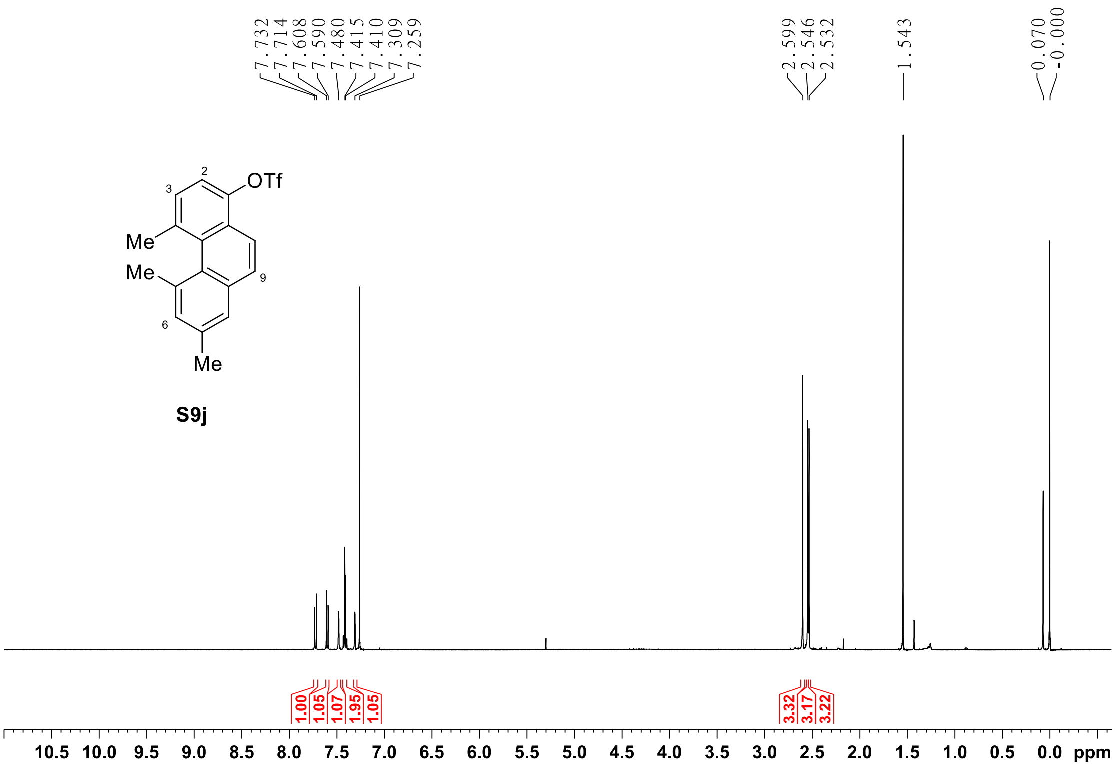

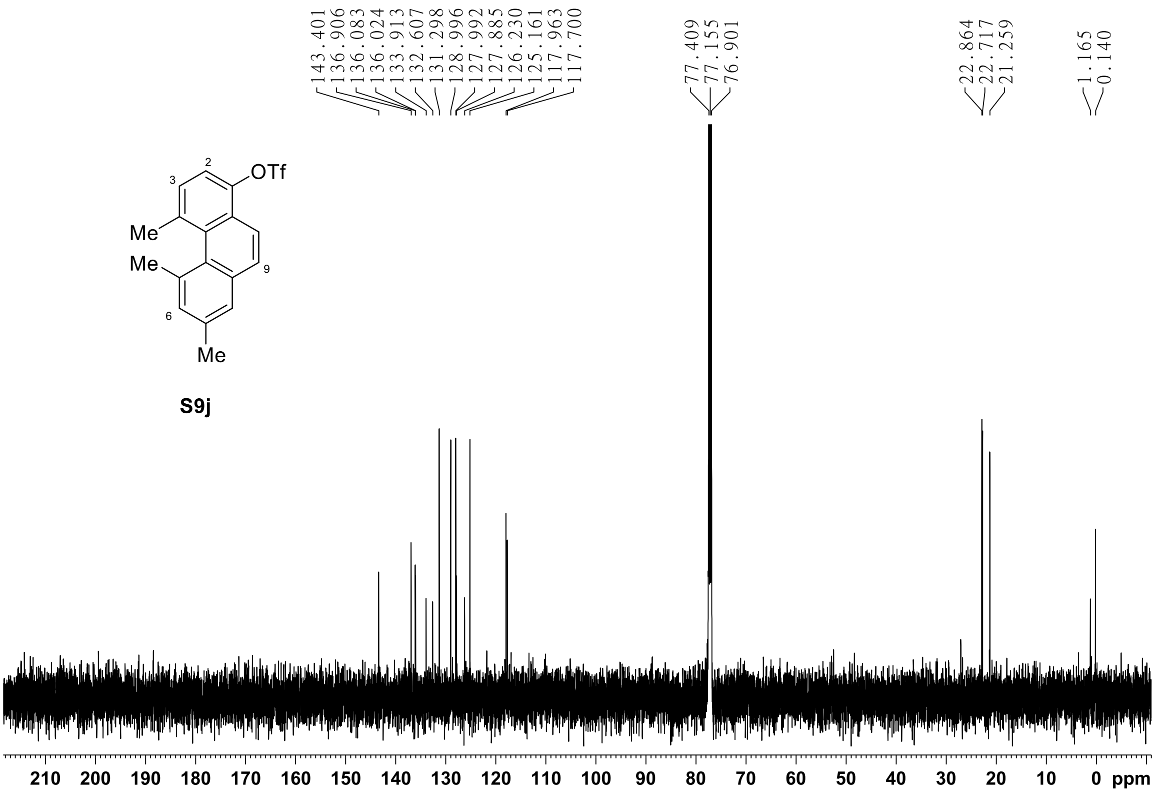

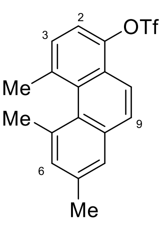
2
3
OTf
Me
Me
9
6
Me

S9j

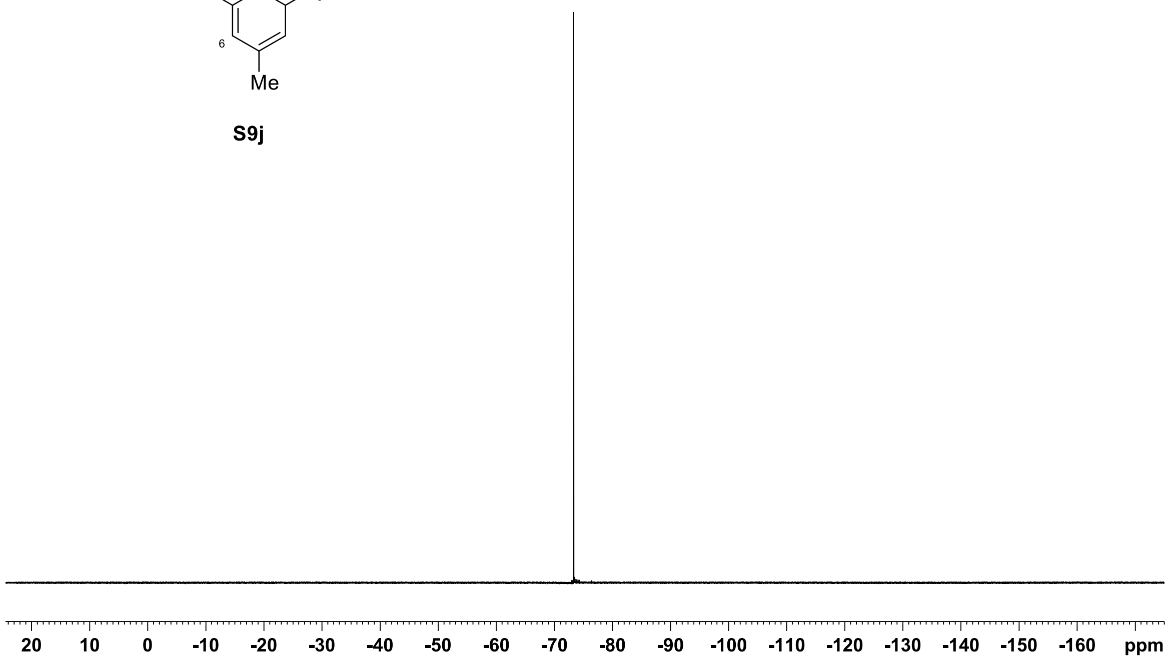
-73.345
20 10 0 -10 -20 -30 -40 -50 -60 -70 -80 -90 -100 -110 -120 -130 -140 -150 -160 ppm

5,8-Dibromo-11-methoxyphenanthro[3,4-d][1,3]dioxole (S9k):

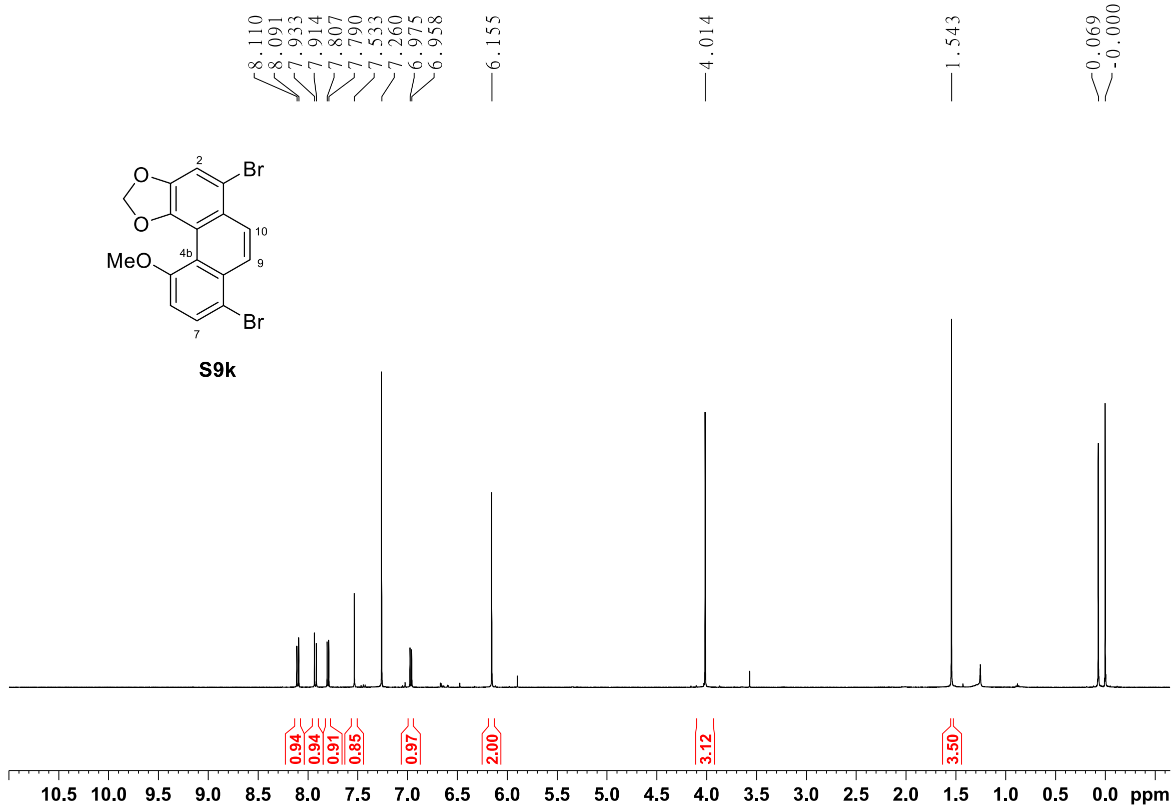

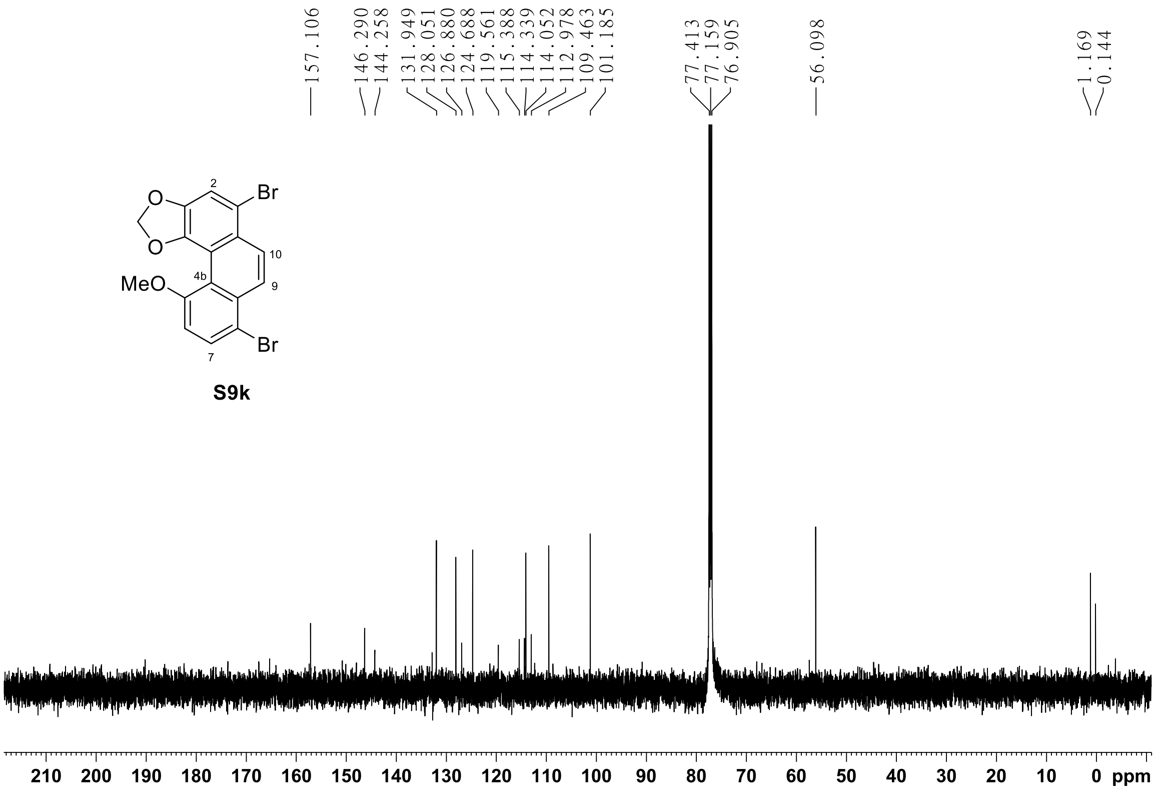

1,8-Dibromo-4-methoxy-5-methylphenanthrene (S9l):

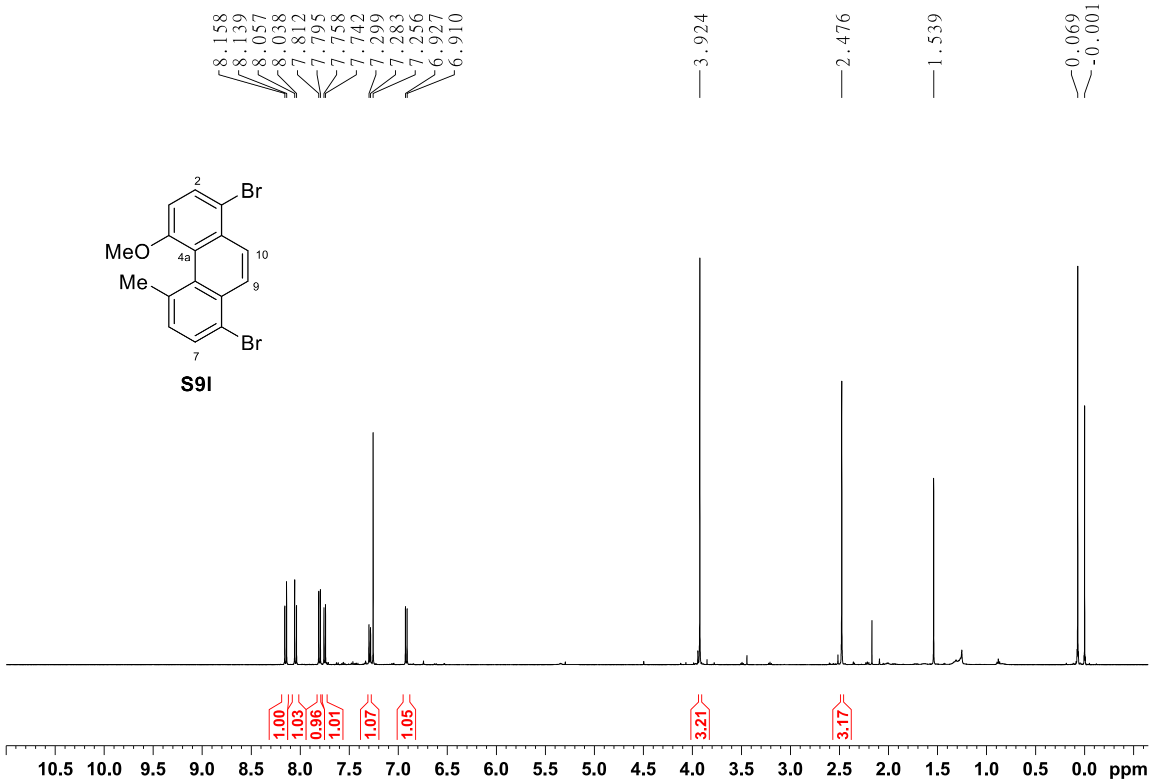

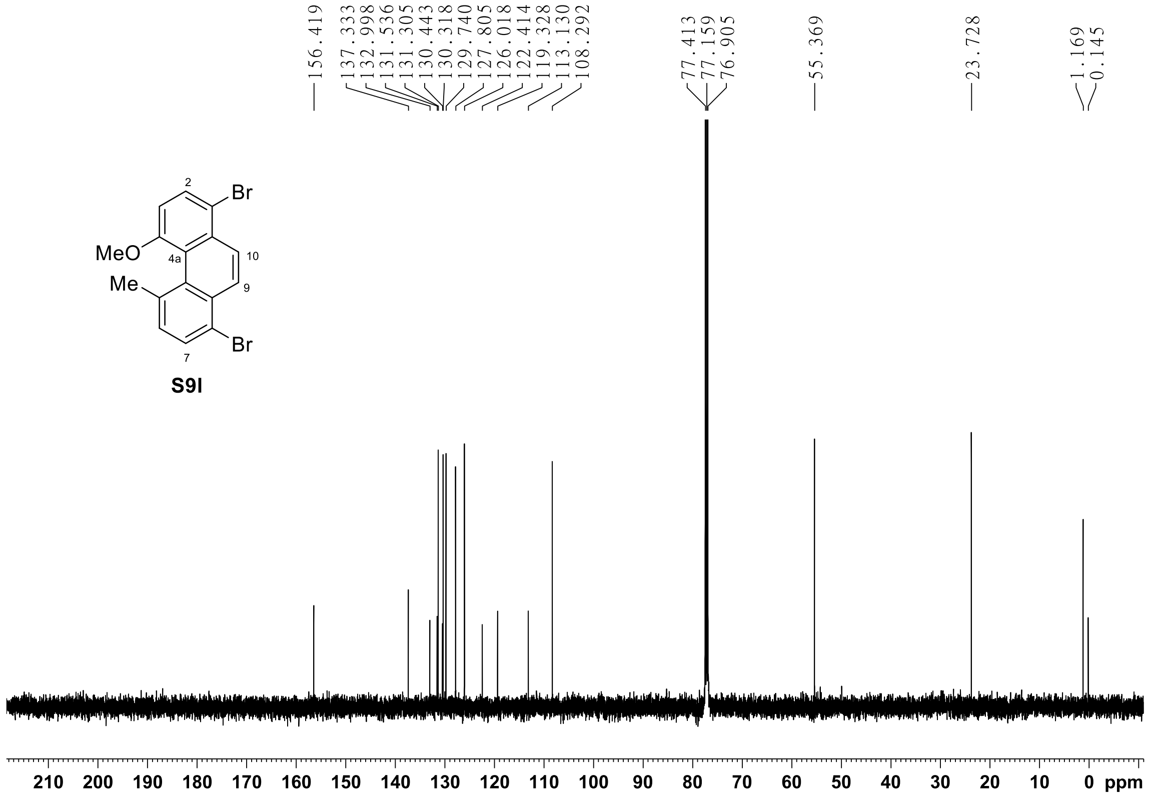

1,8-Dimethoxy-4,5-dimethylphenanthrene (S9m):

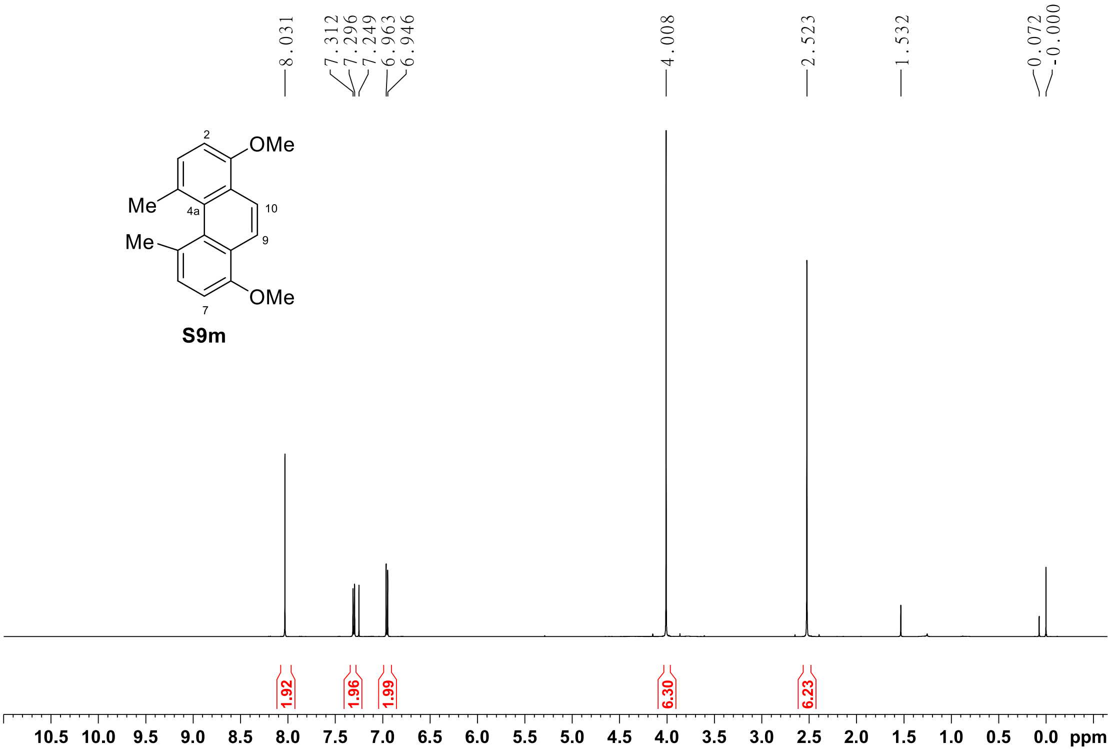

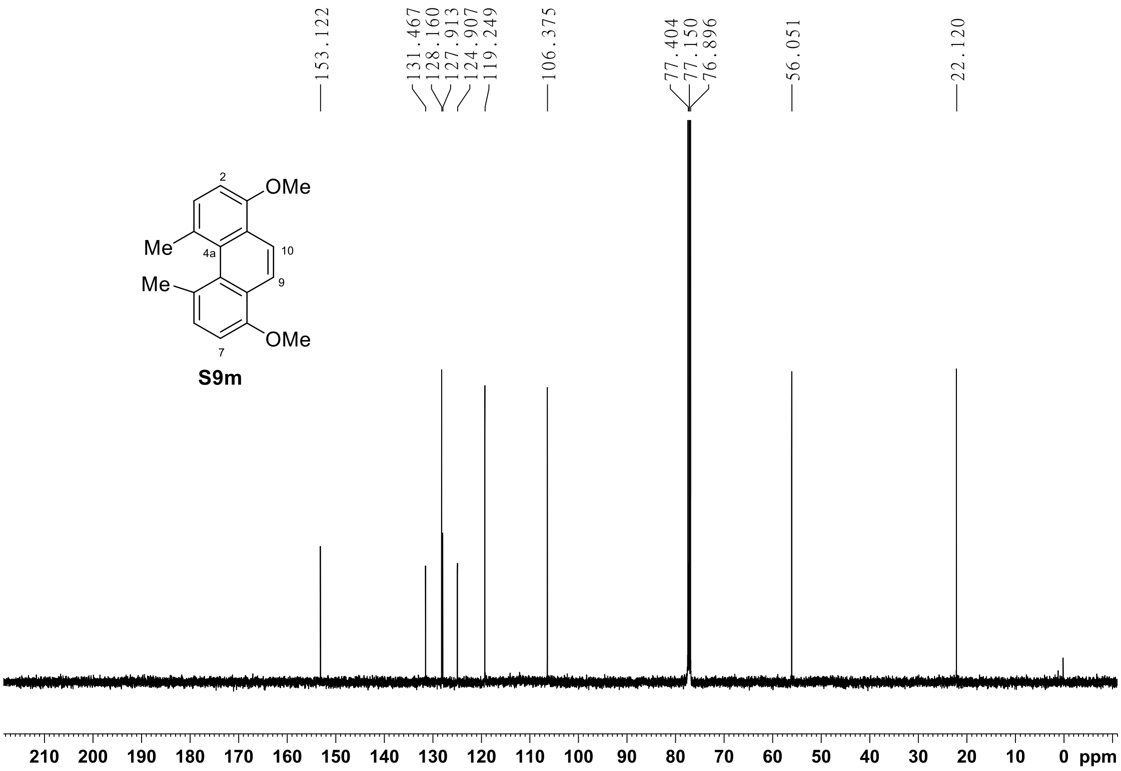

4,5-Dimethylphenanthrene-1,8-diol (S9n):

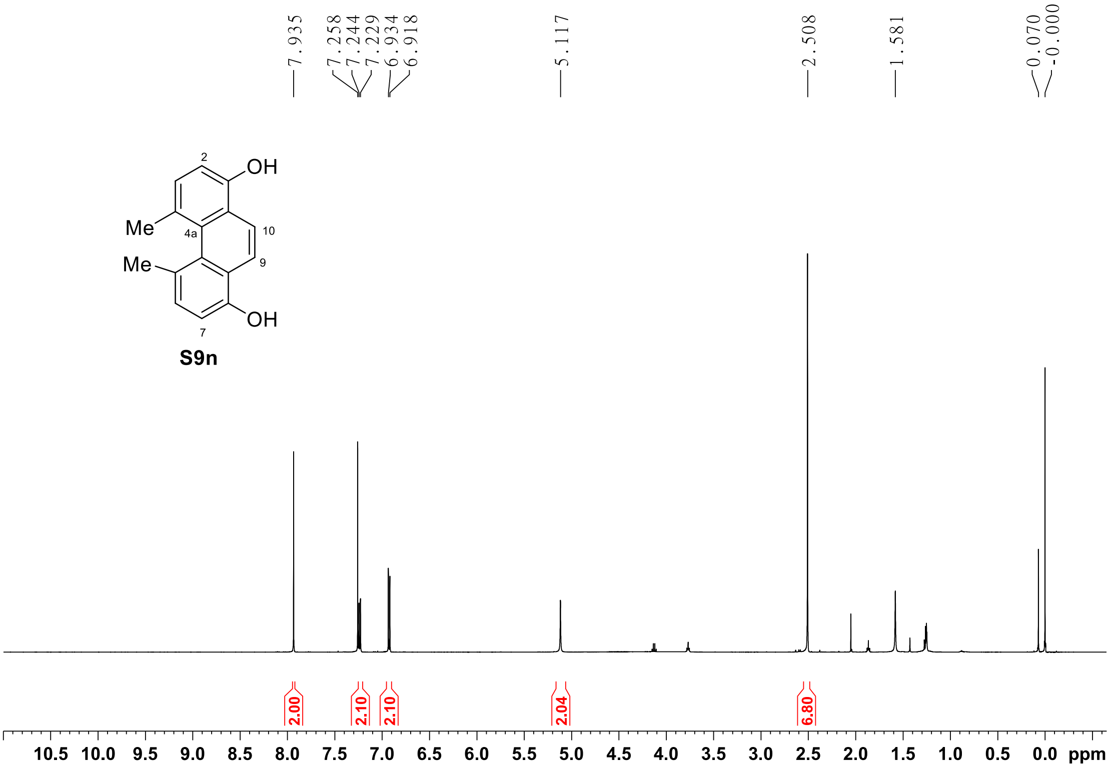

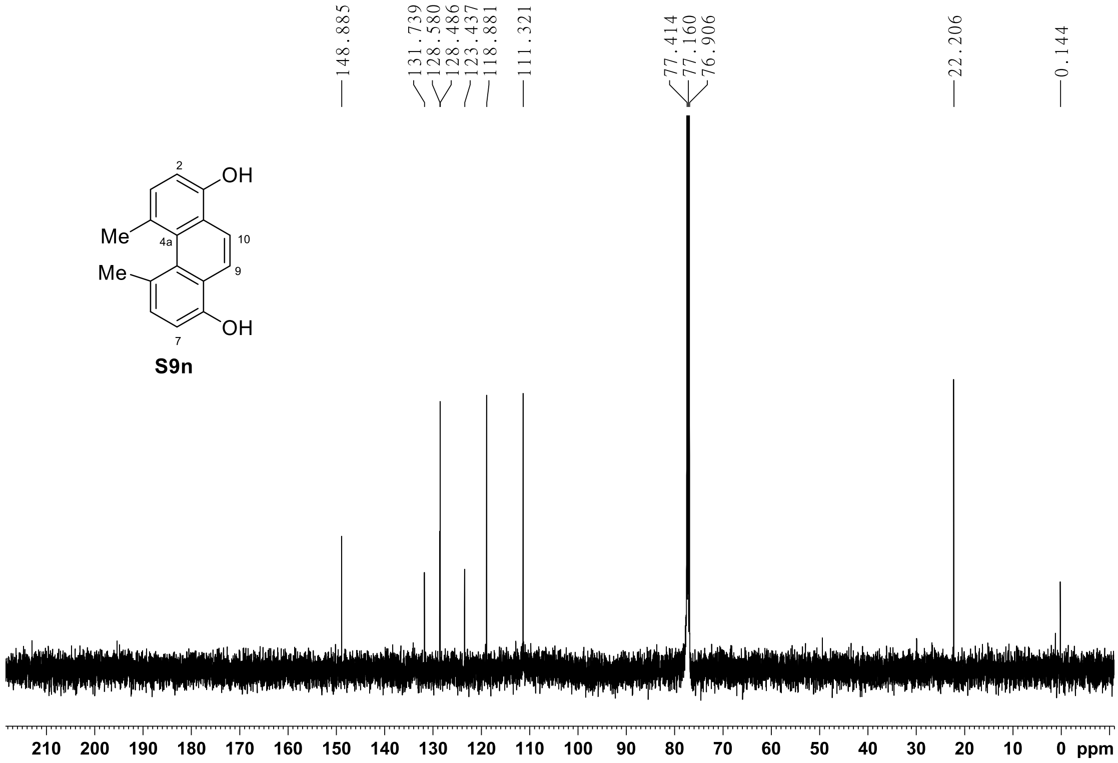

4,5-Dimethylphenanthrene-1,8-diyl bis(trifluoromethanesulfonate) (S9o):

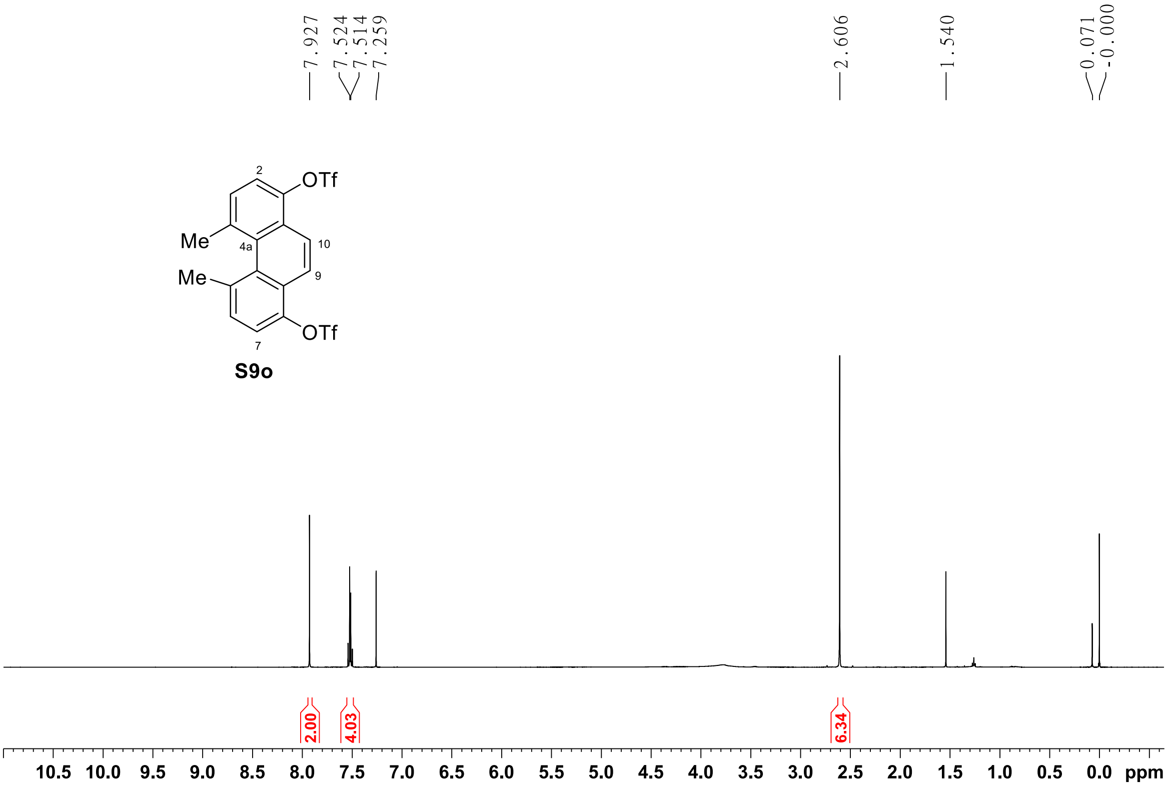

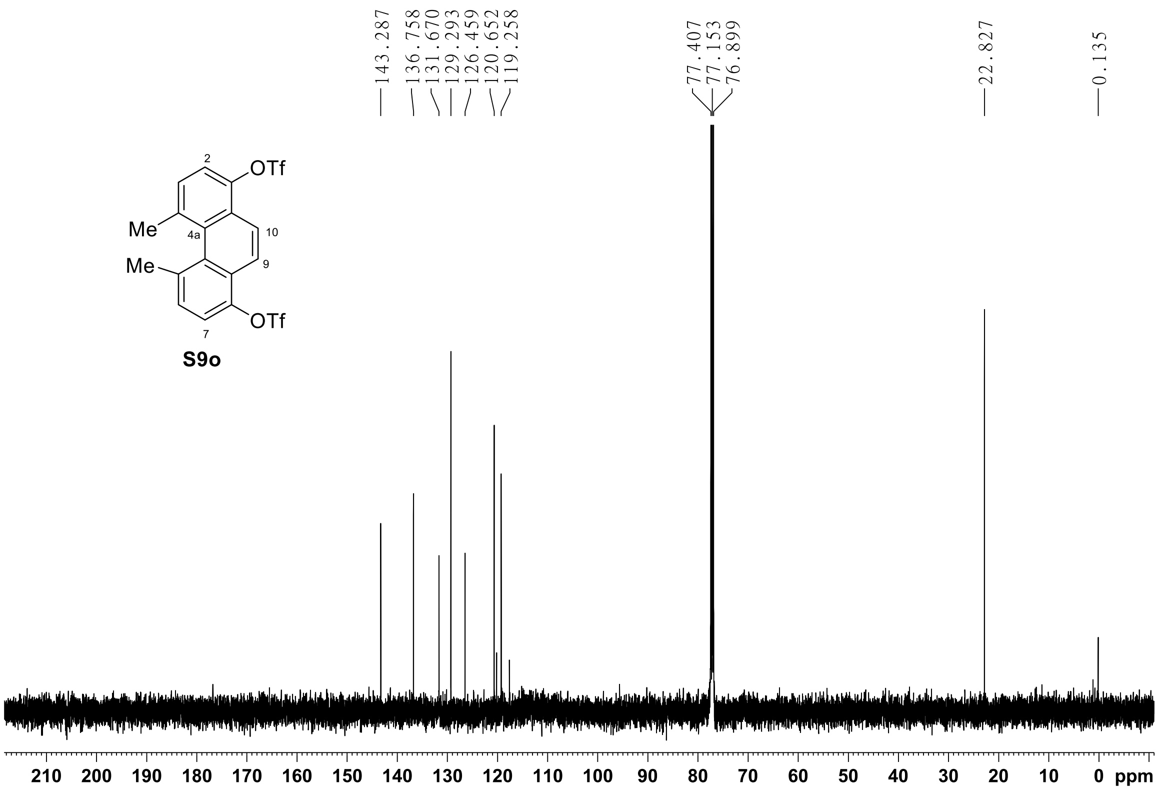

5,7-Dimethoxy-4-methyl-1-(2-vinylphenyl)phenanthrene (15a):

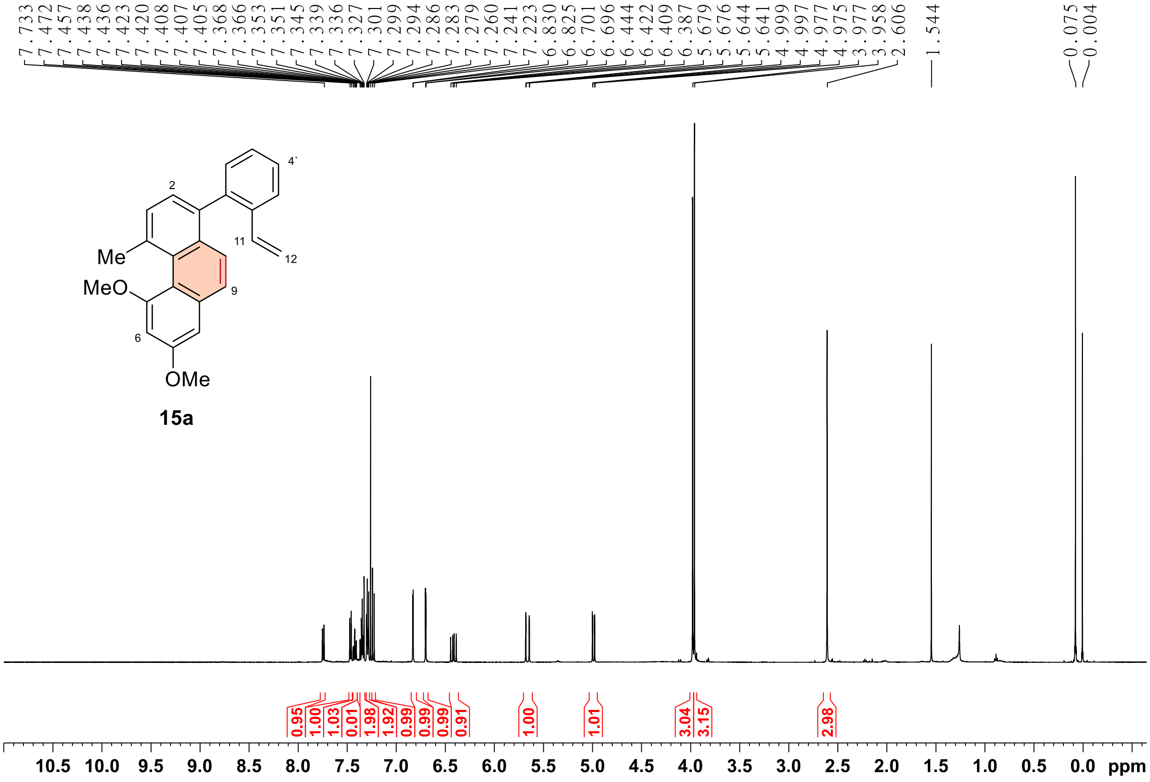

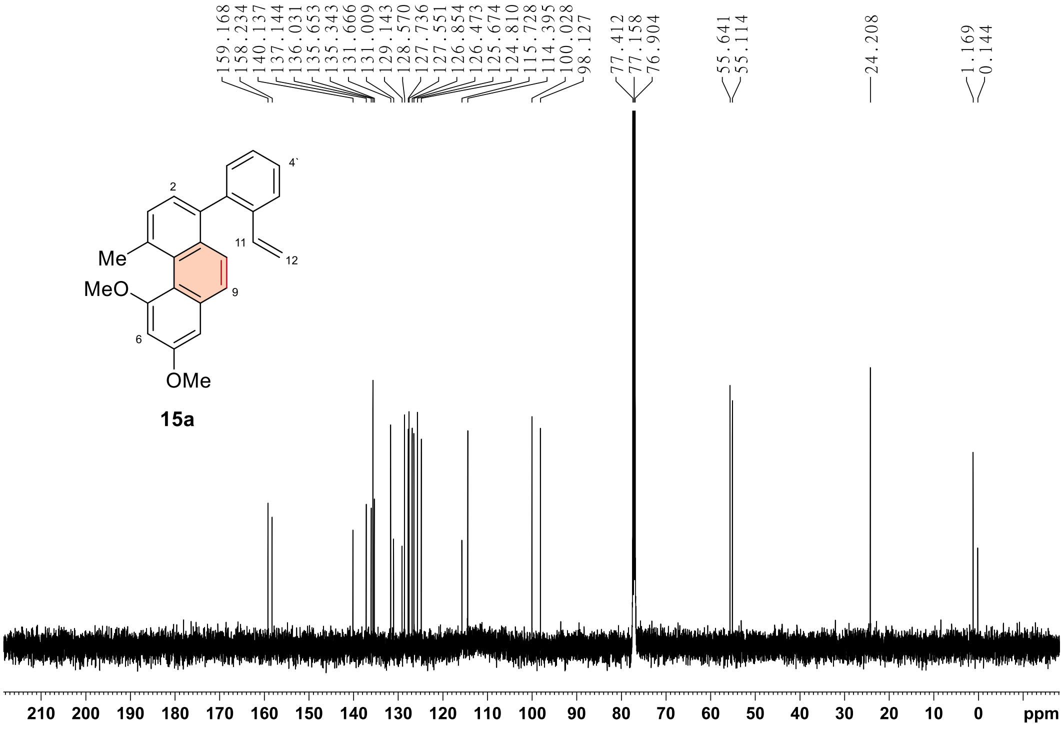

9,11-Dimethyl-5-(2-vinylphenyl)phenanthro[3,4-d][1,3]dioxole (15b):

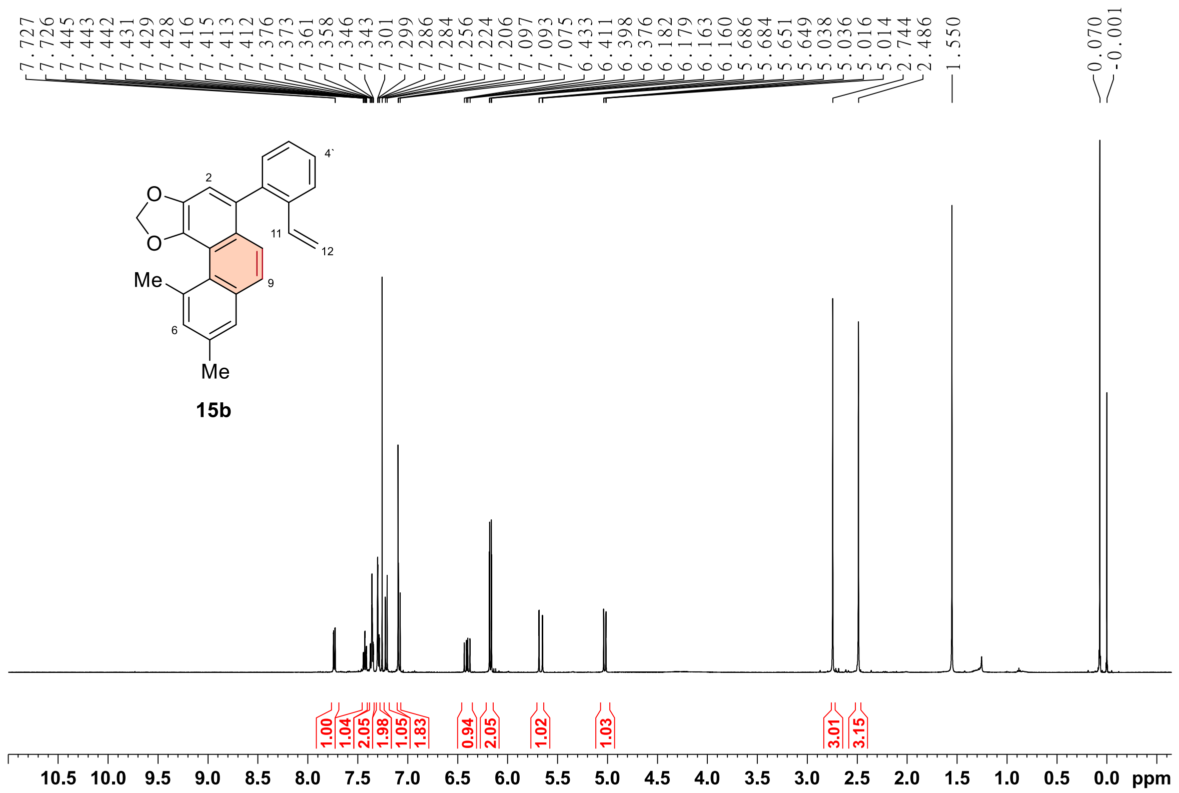

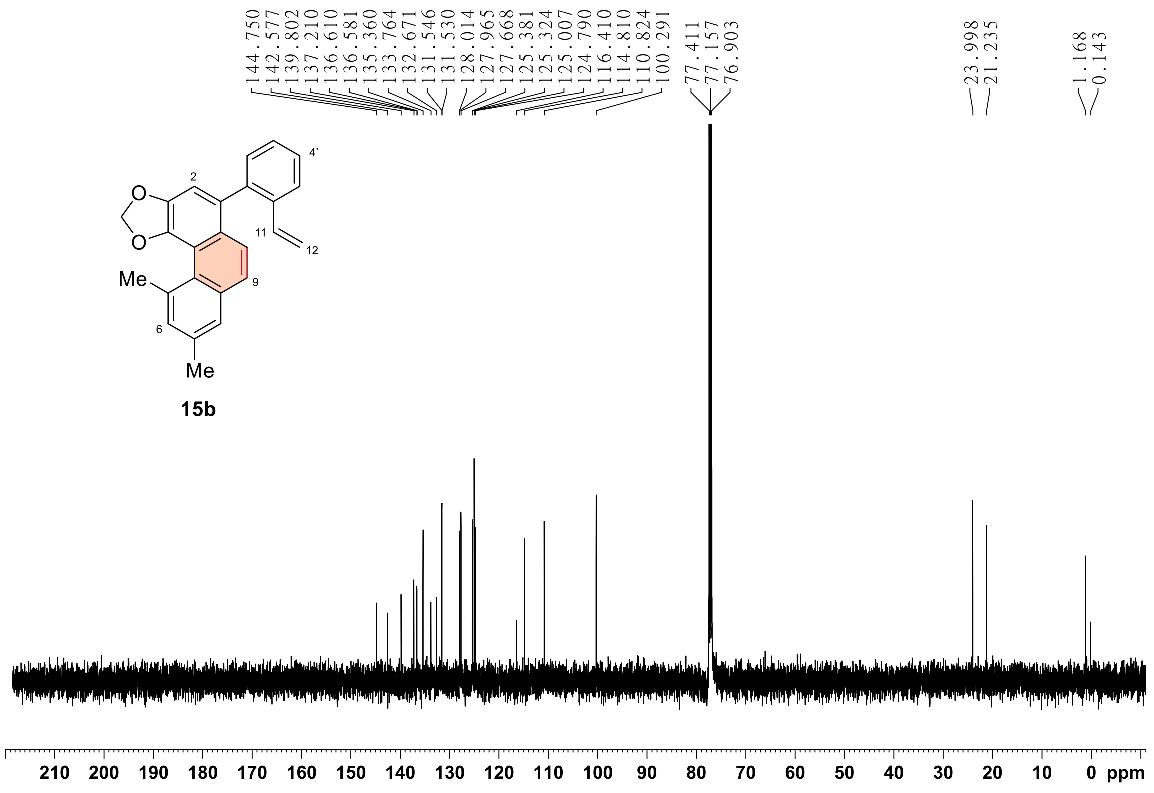

5,7-Dimethoxy-1-(2-vinylphenyl)phenanthrene (15c):

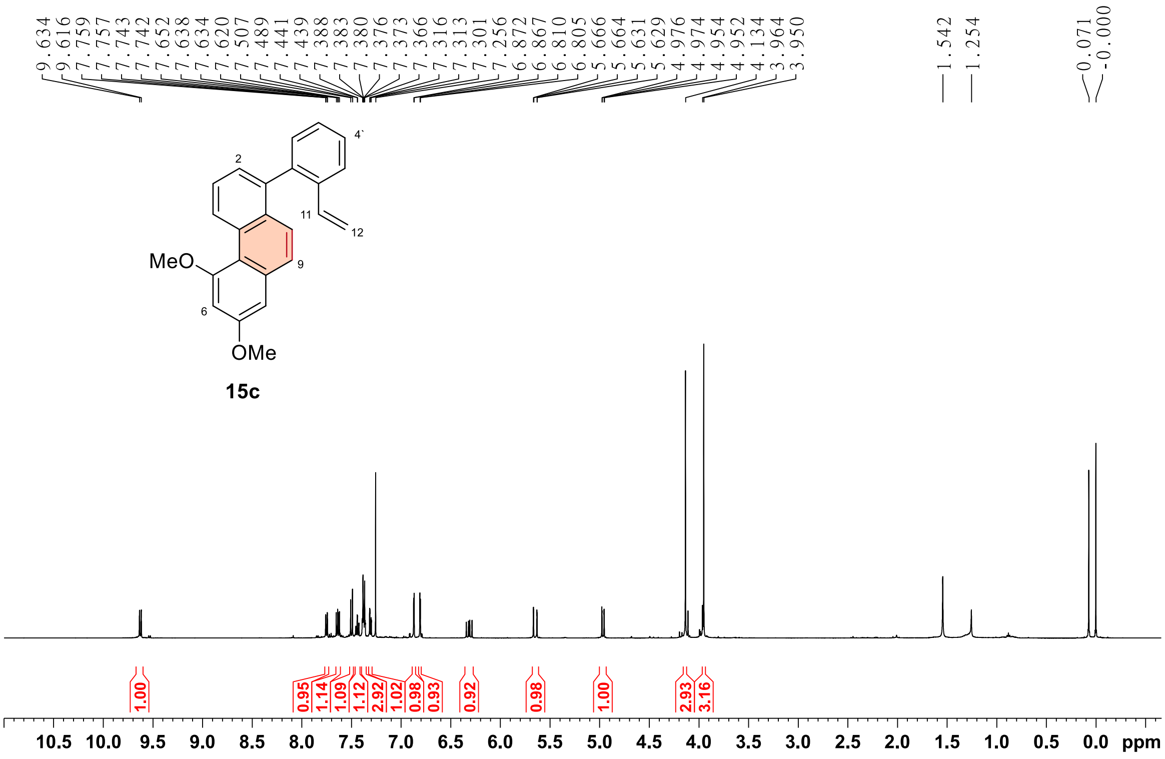

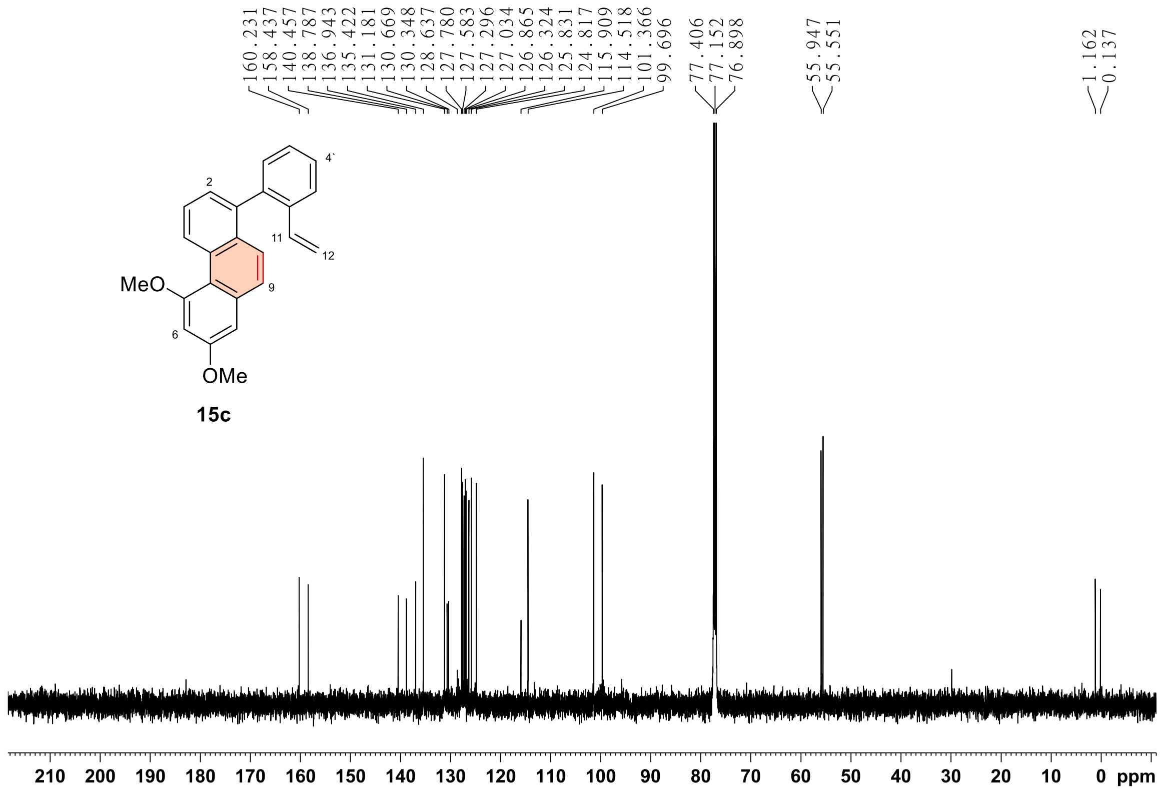

4,5,7-Trimethoxy-1-(2-vinylphenyl)phenanthrene (15d):

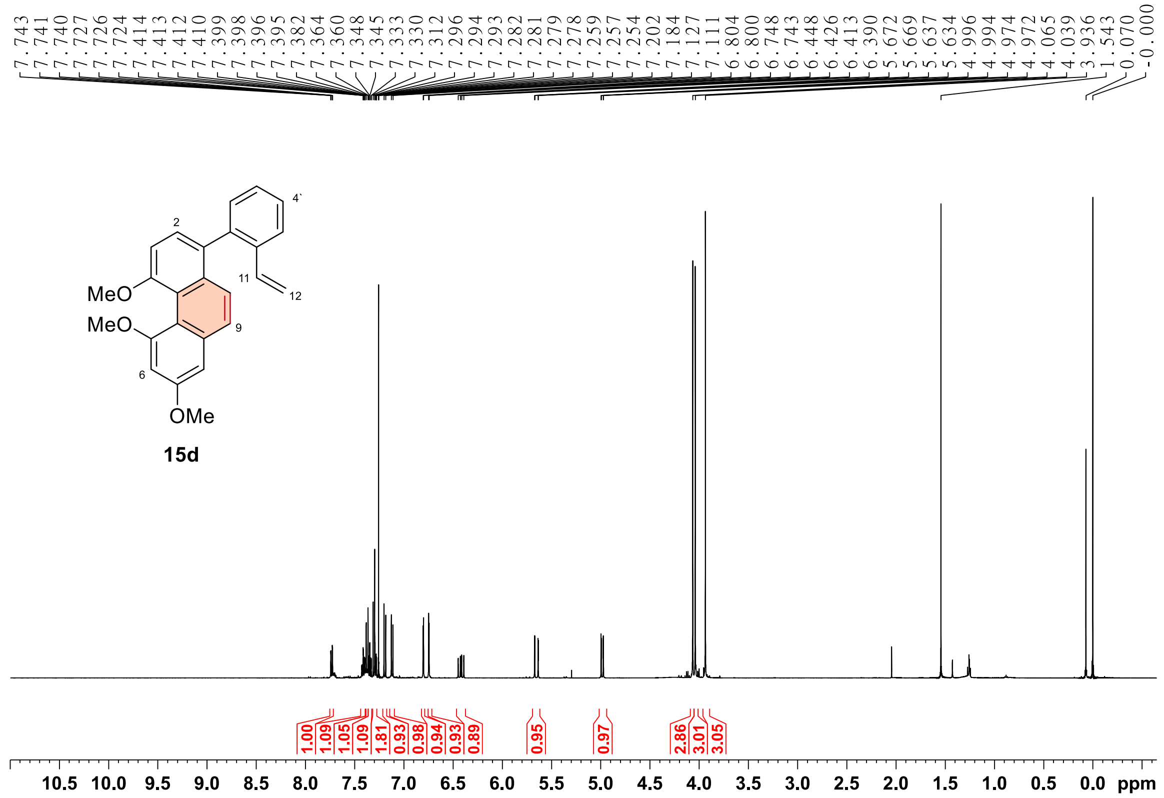

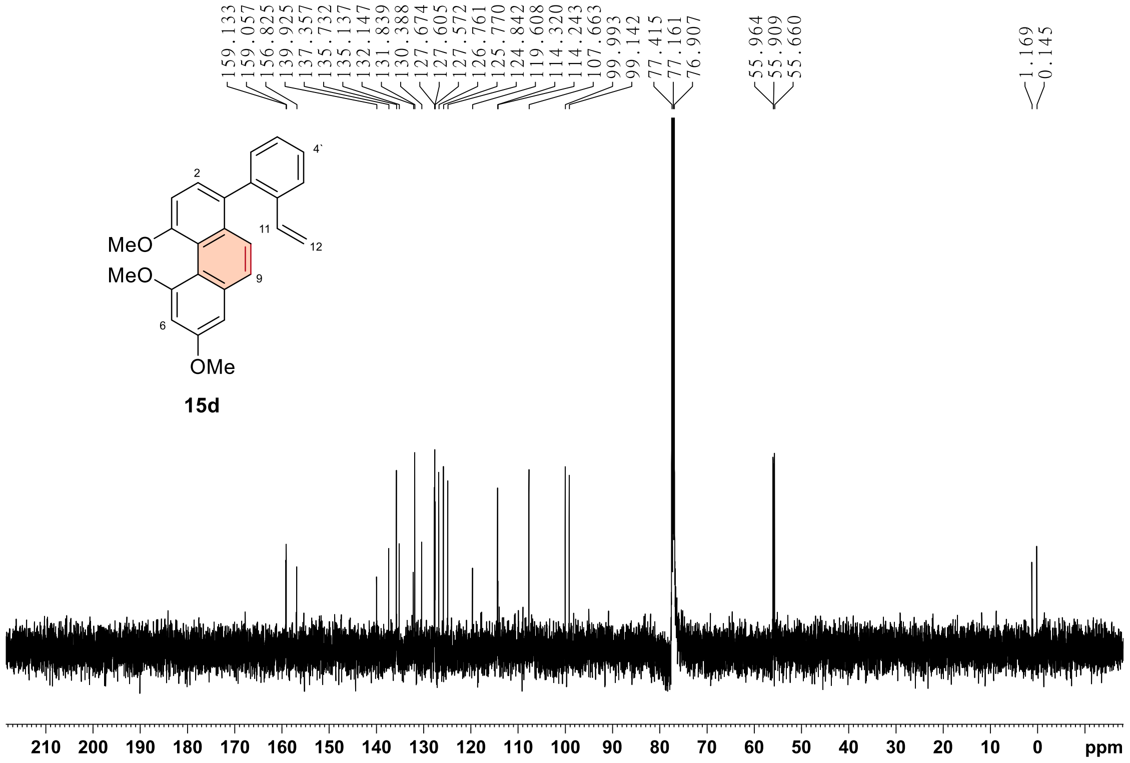

4-Methoxy-1,5-dimethyl-8-(2-vinylphenyl)phenanthrene (15e):

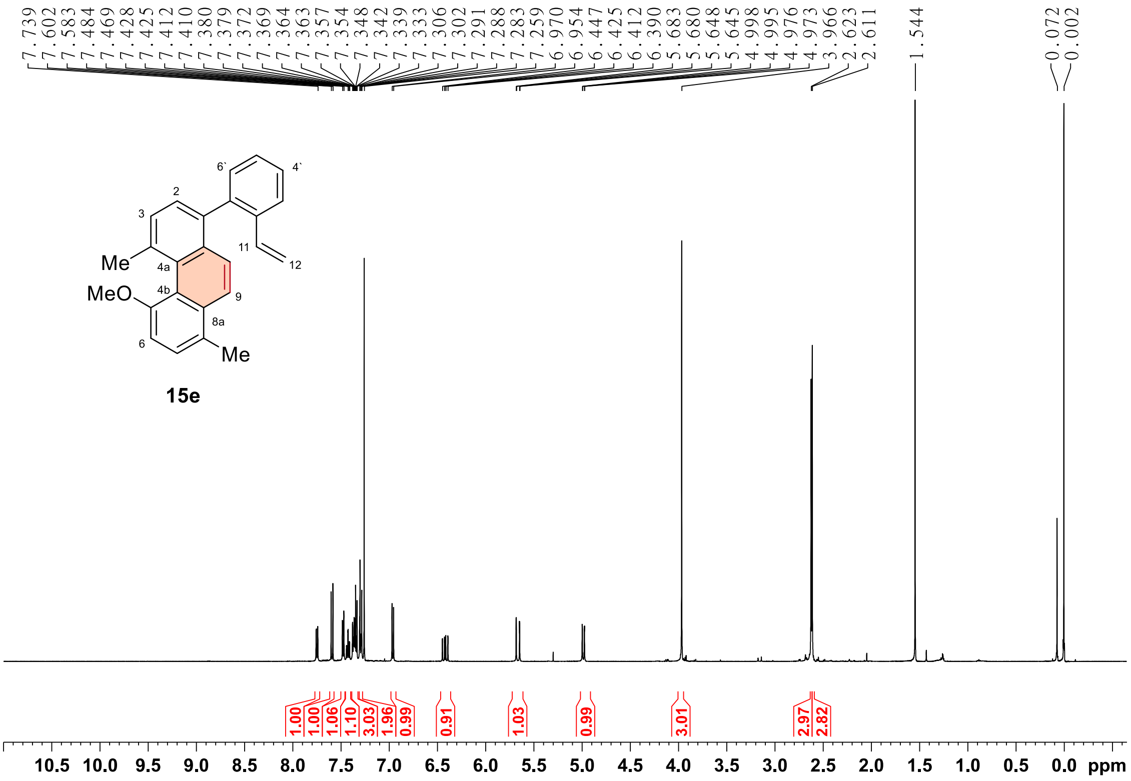

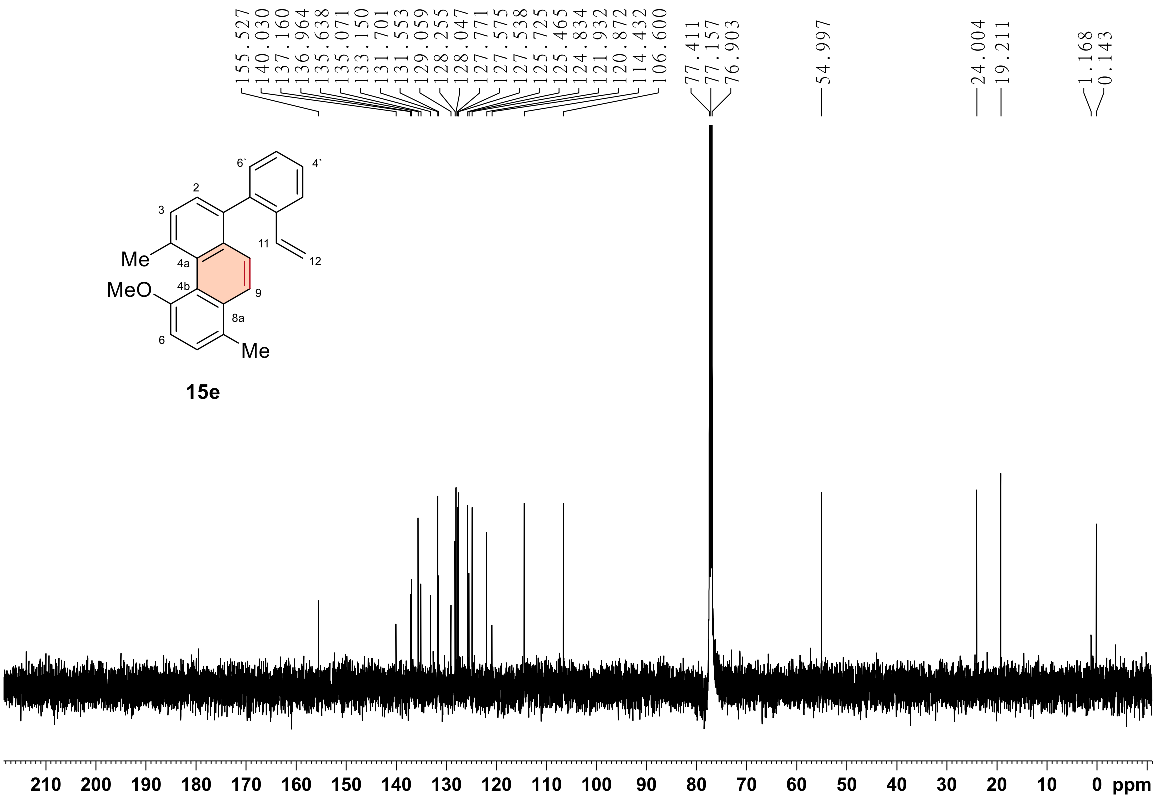

4-Methoxy-5,8-dimethyl-1-(2-vinylphenyl)phenanthrene (15f):

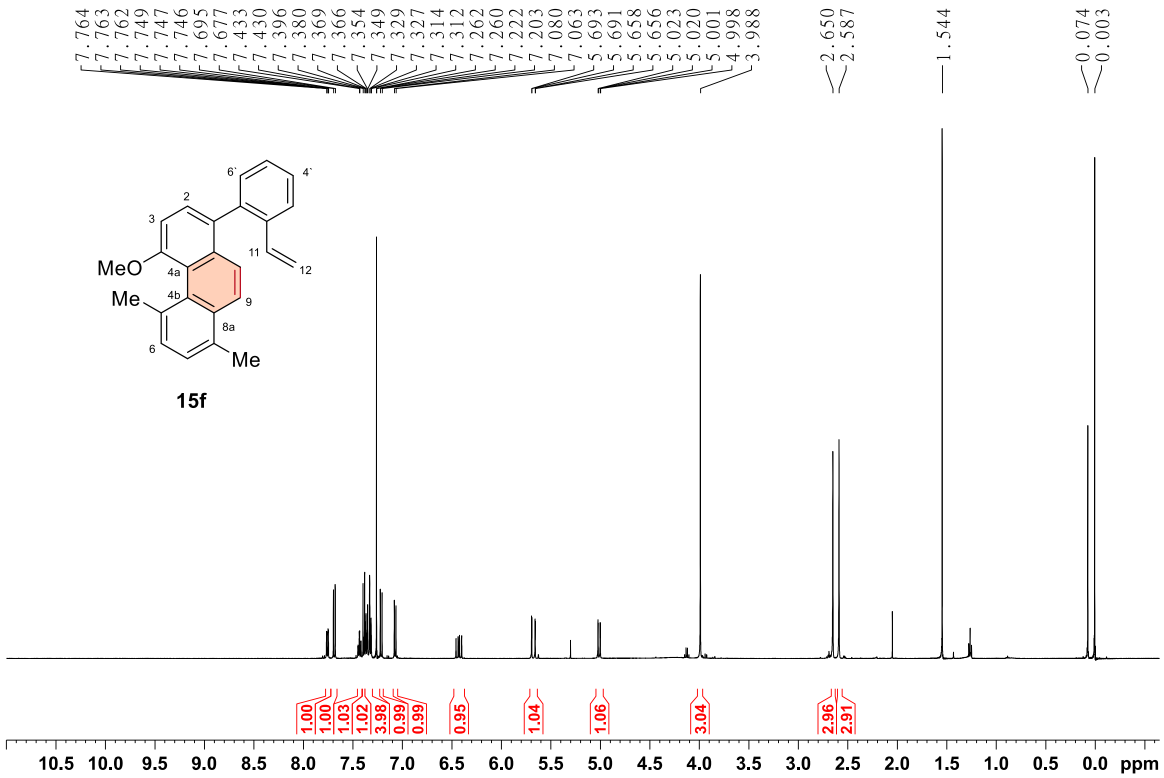

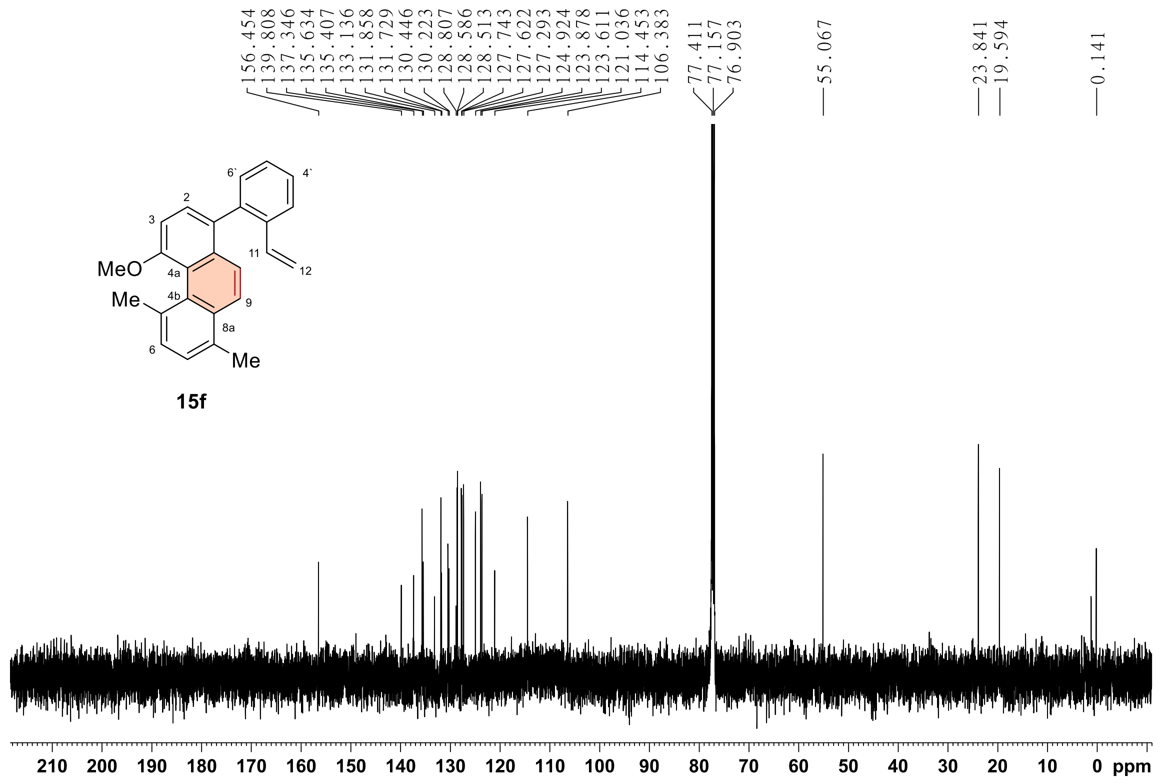

1-(5-Chloro-2-vinylphenyl)-5,7-dimethoxy-4-methylphenanthrene (15g):

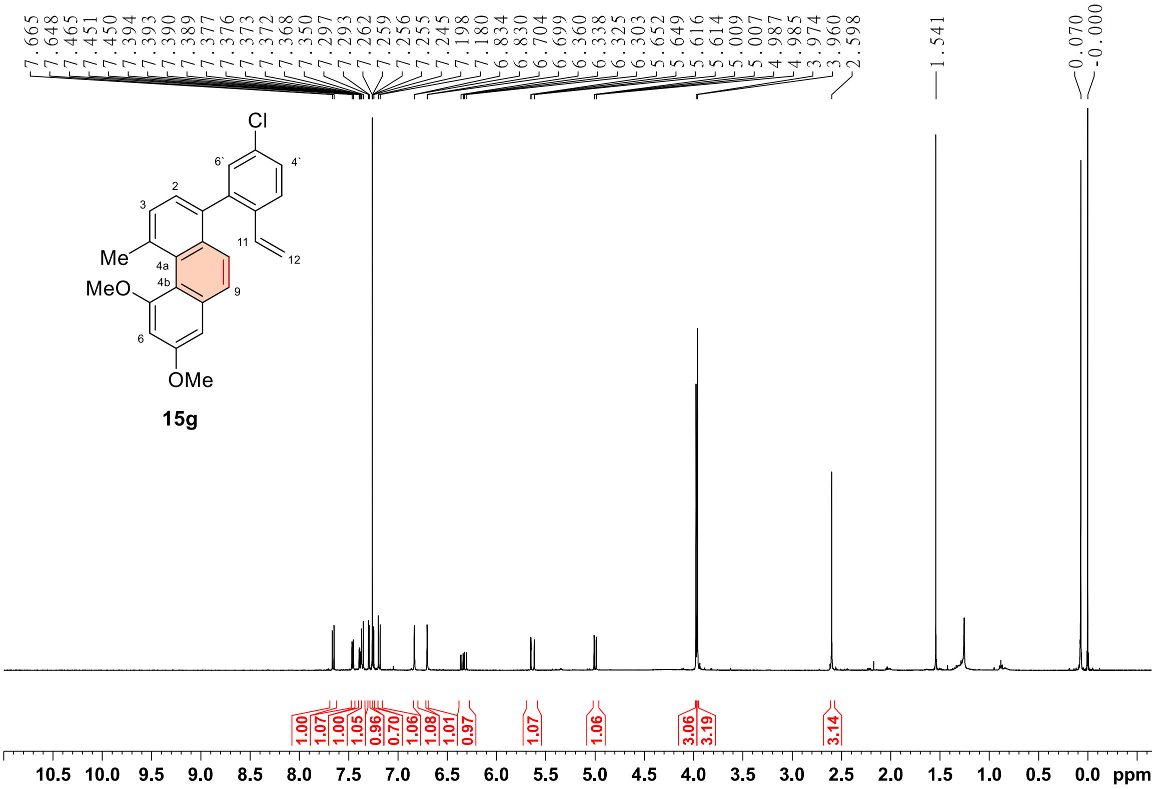

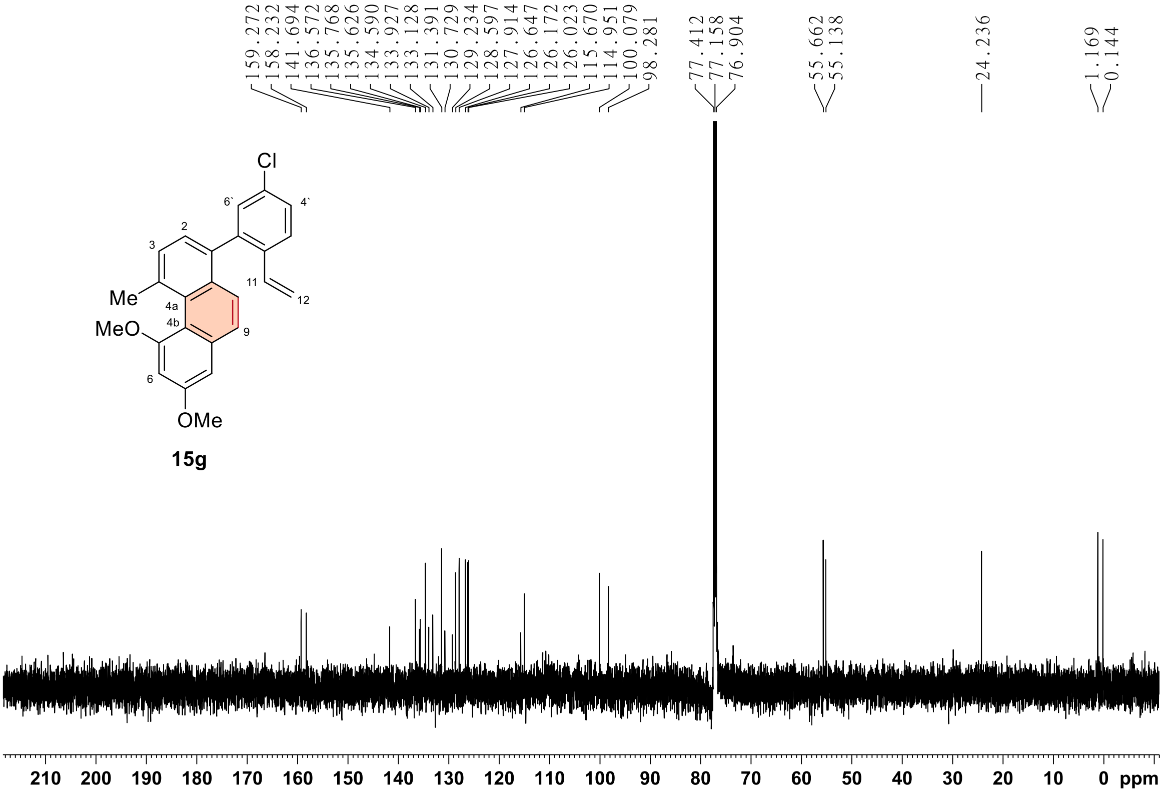

1-(5-Chloro-2-vinylphenyl)-4,5-dimethoxyphenanthrene (15h):

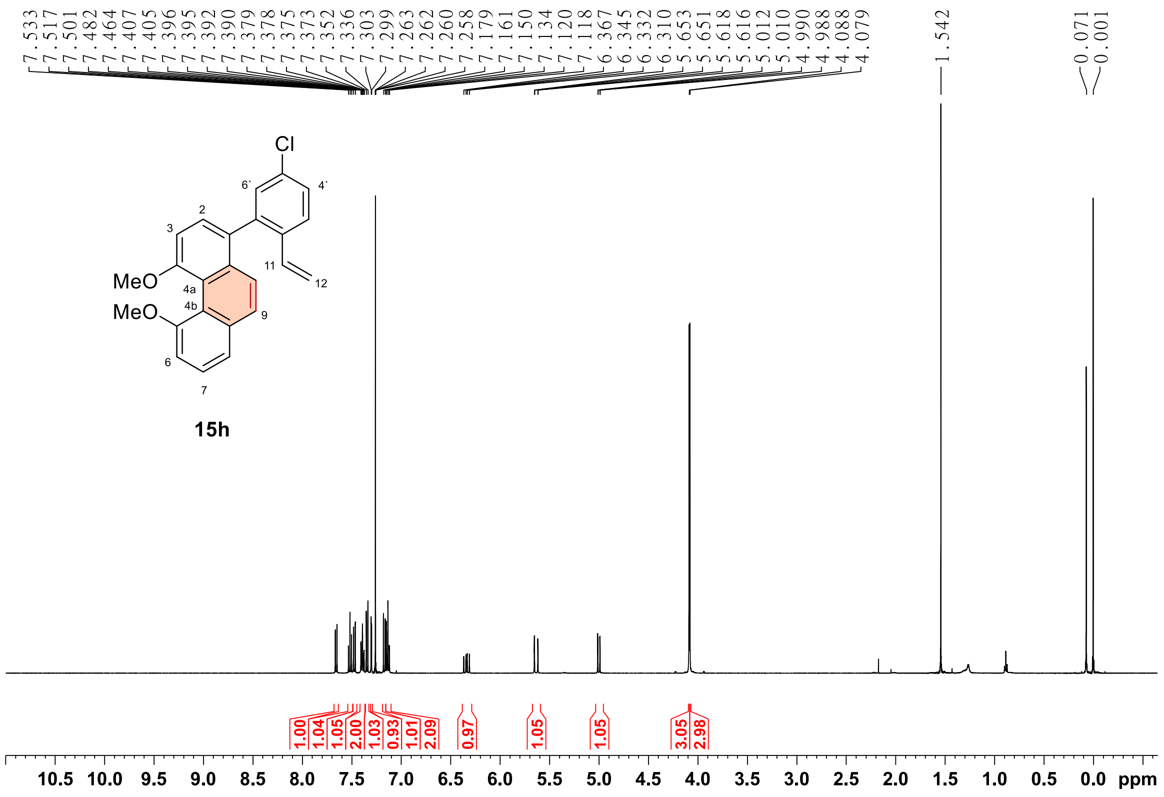

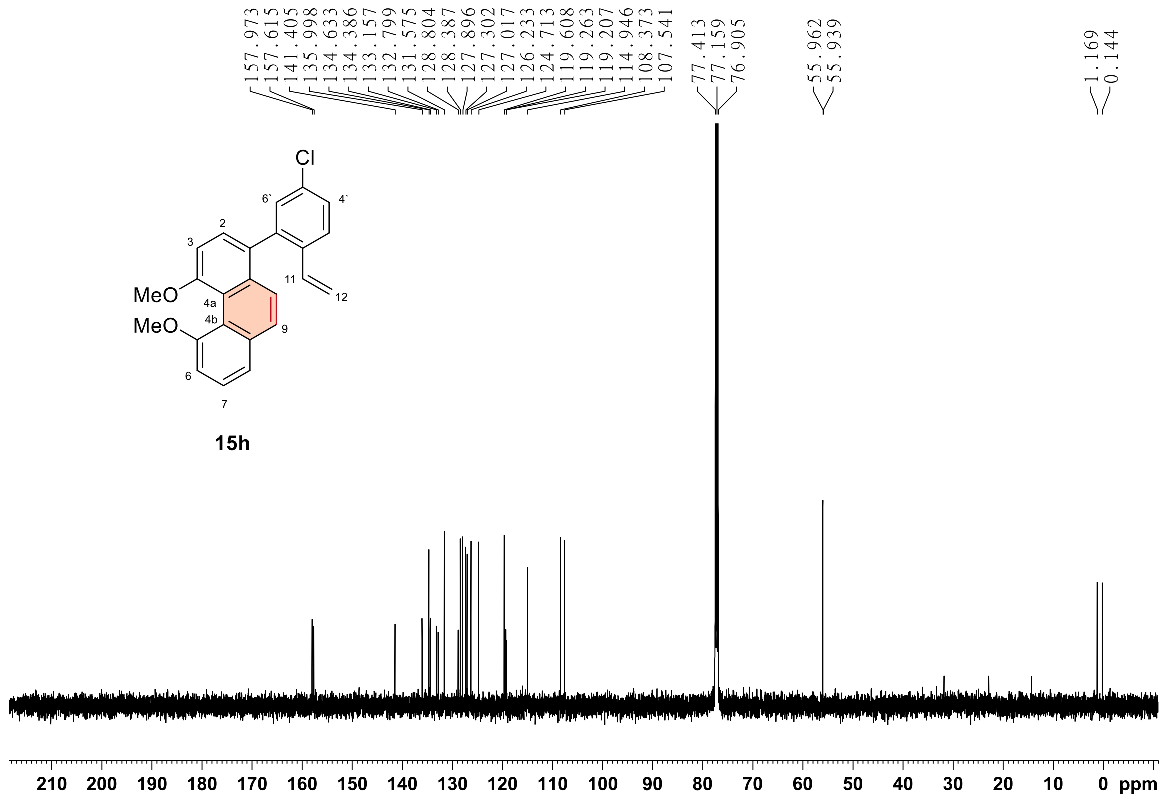

4,5,7-Trimethyl-1-(2-vinylphenyl)phenanthrene (15i):

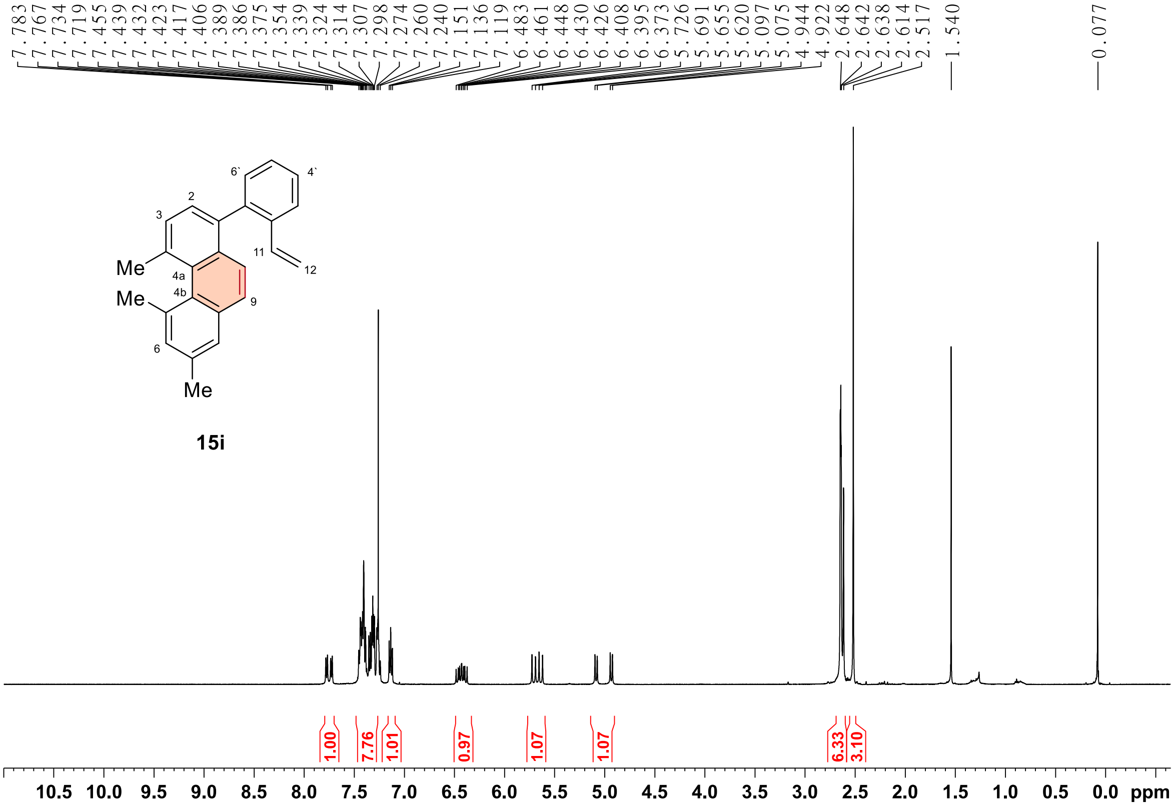

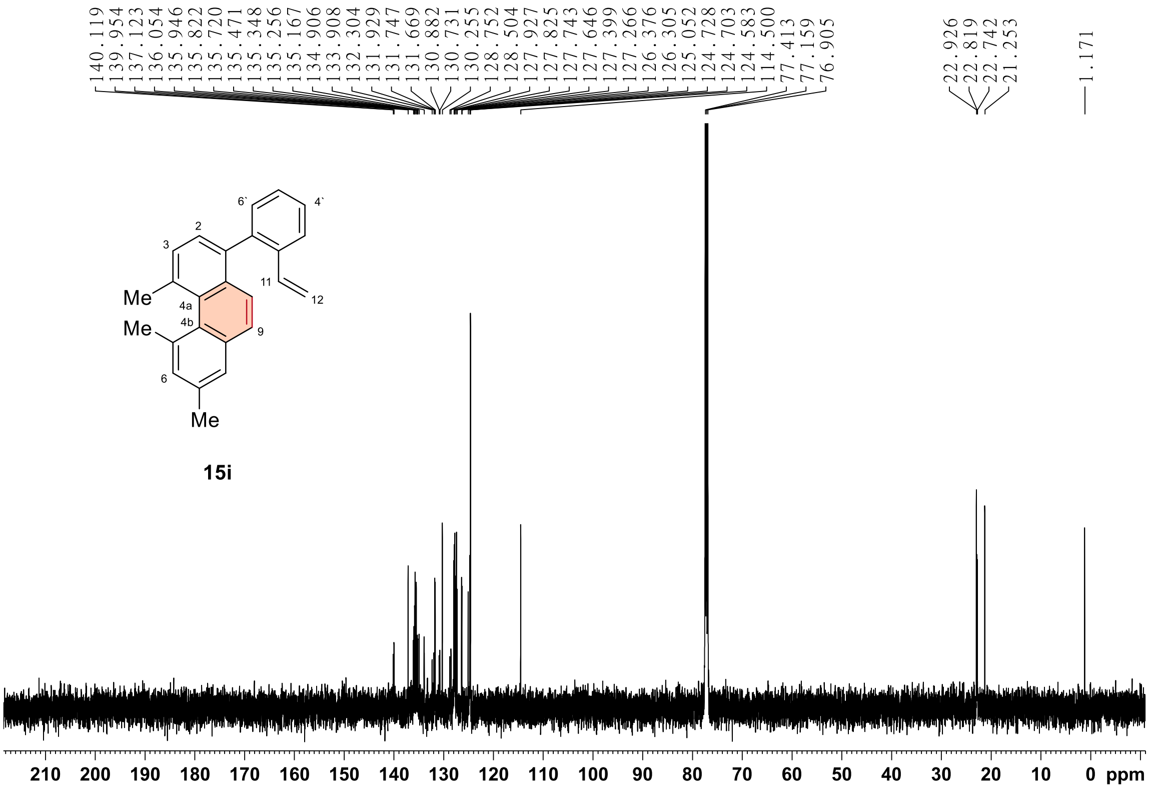

1-Bromo-4,5-dimethoxy-8-(2-vinylphenyl)phenanthrene (15j):

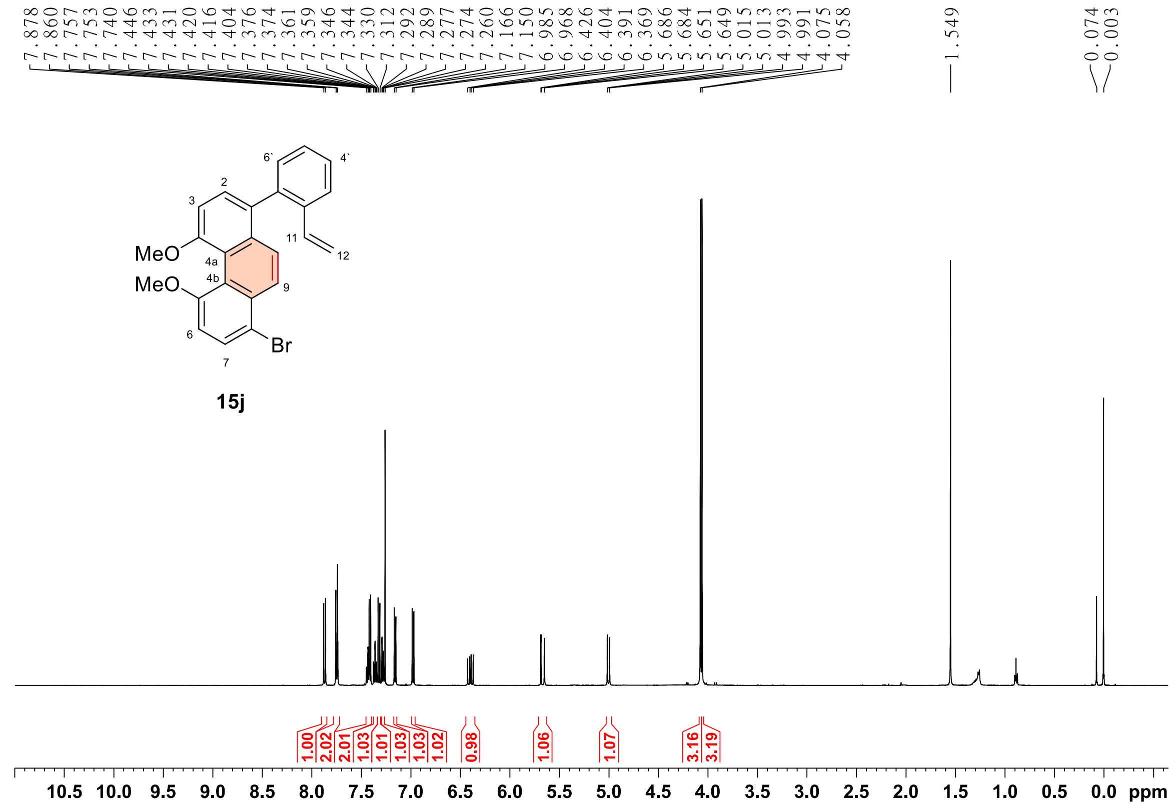

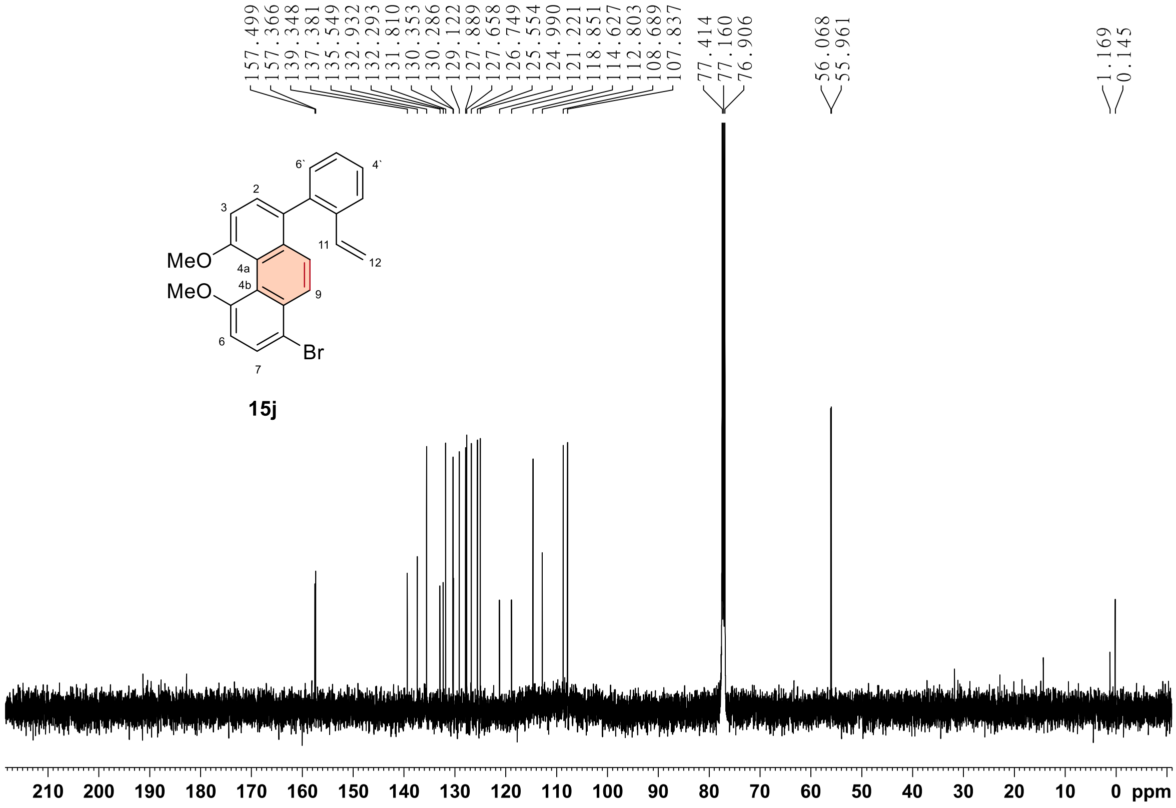

1-Bromo-8-(5-chloro-2-vinylphenyl)-4,5-dimethoxyphenanthrene (15k):

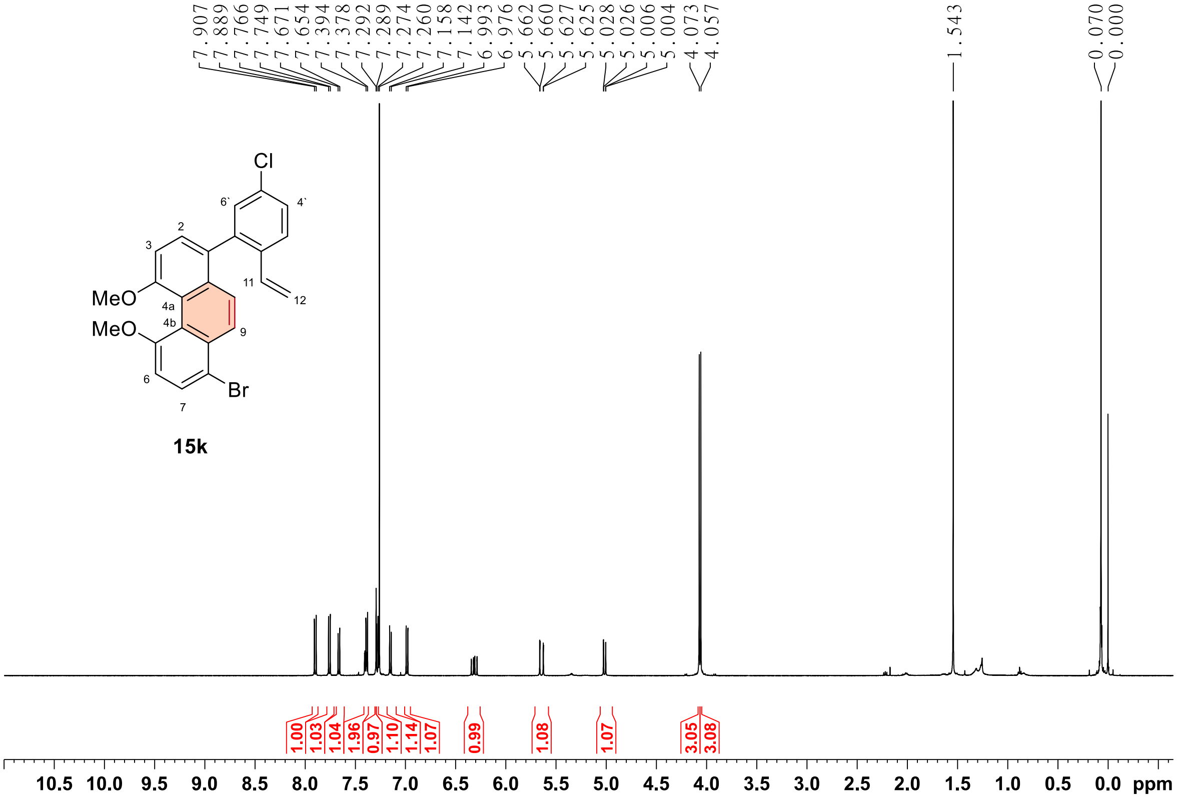

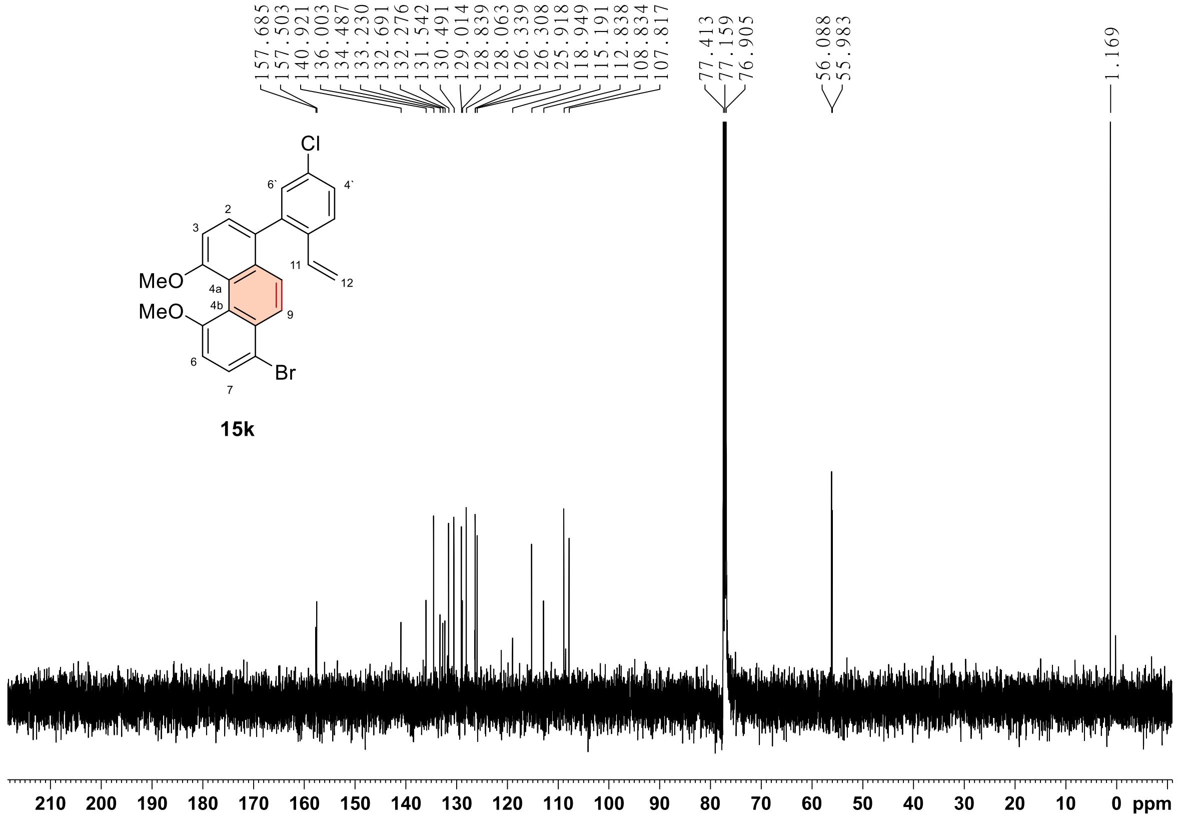

4,5-Dimethoxy-1,8-bis(2-vinylphenyl)phenanthrene (17a):

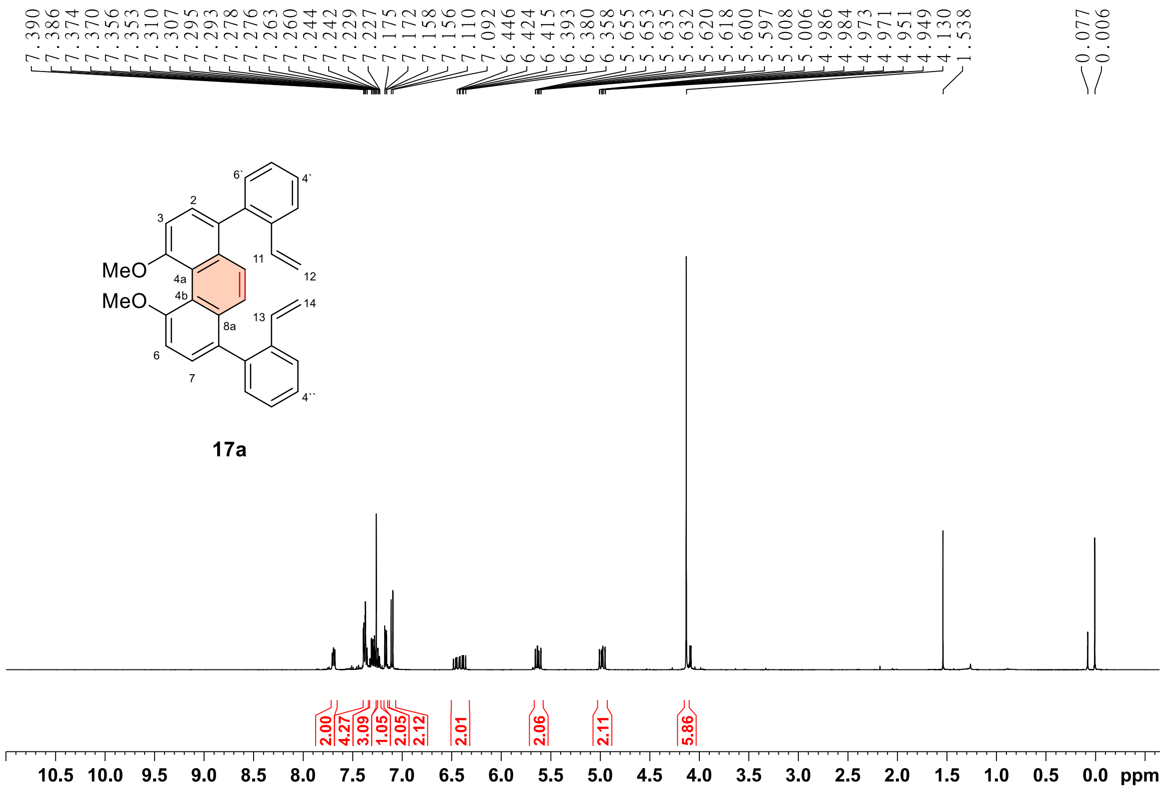

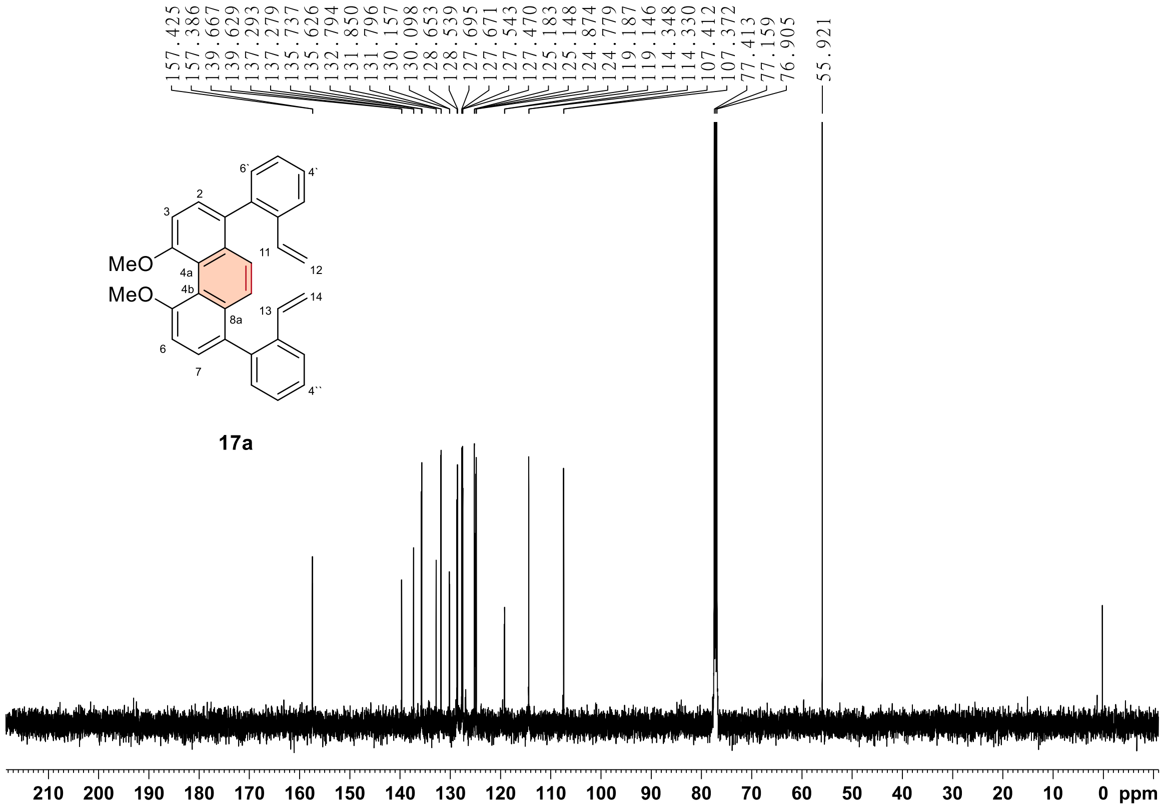

11-Methoxy-5,8-bis(2-vinylphenyl)phenanthro[3,4-d][1,3]dioxole (17b):

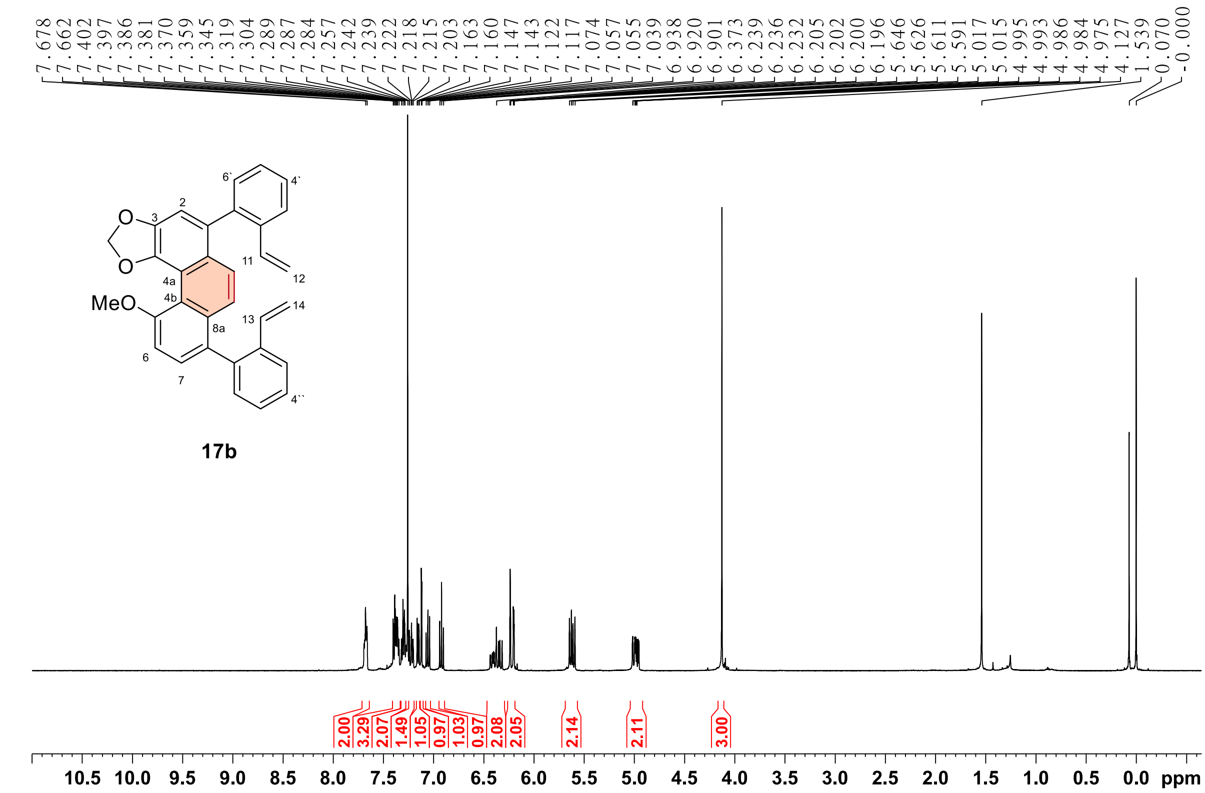

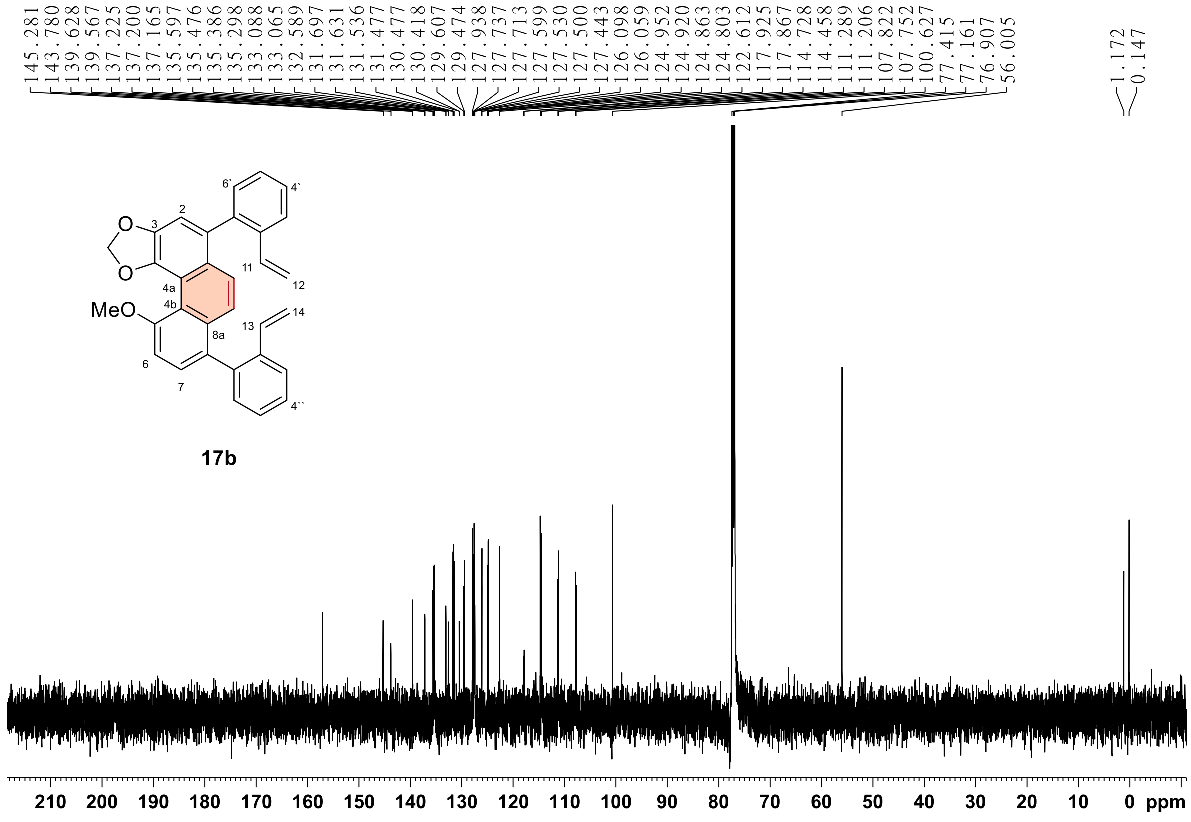

4-Methoxy-5-methyl-1,8-bis(2-vinylphenyl)phenanthrene (17c):

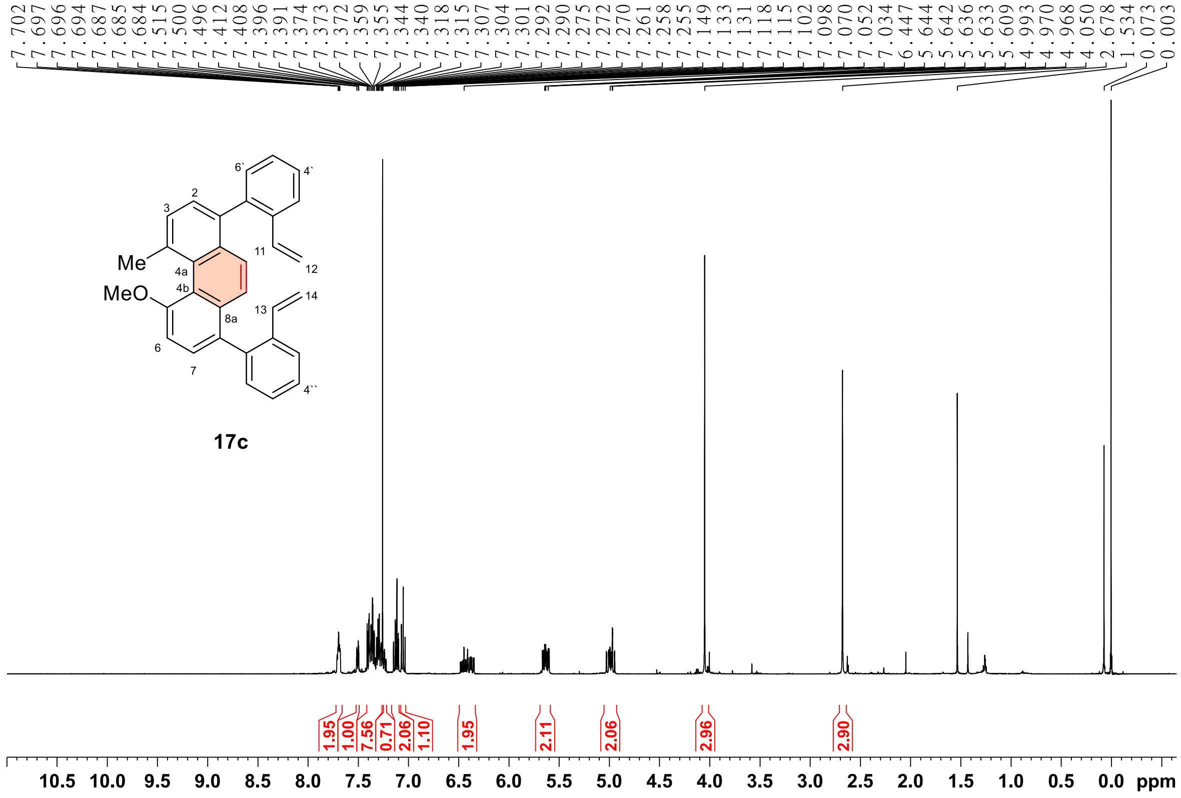

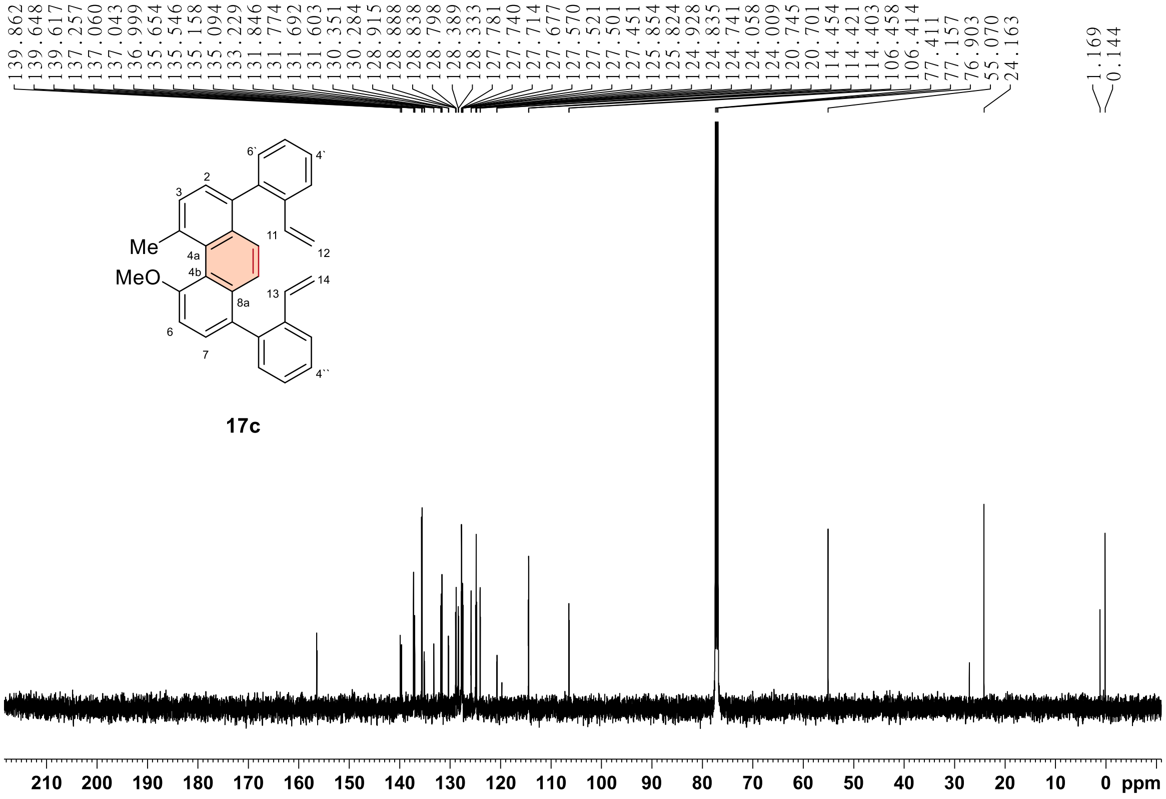

4,5-Dimethyl-1,8-bis(2-vinylphenyl)phenanthrene (17d):

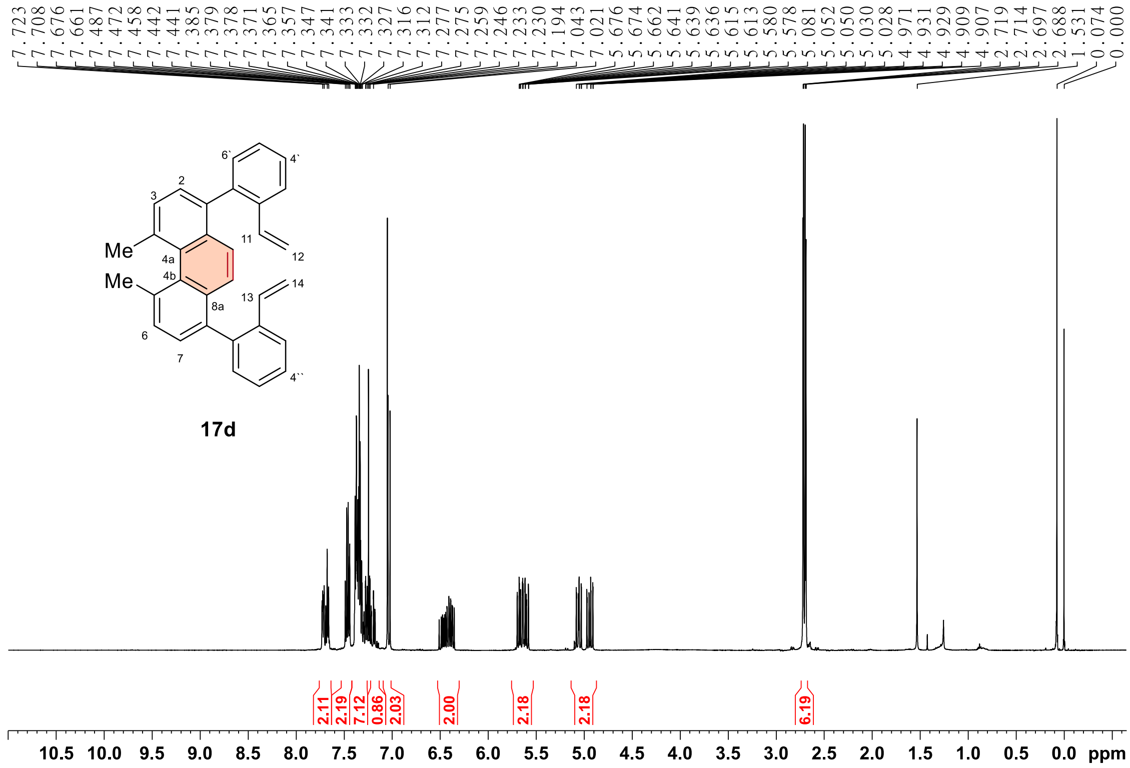

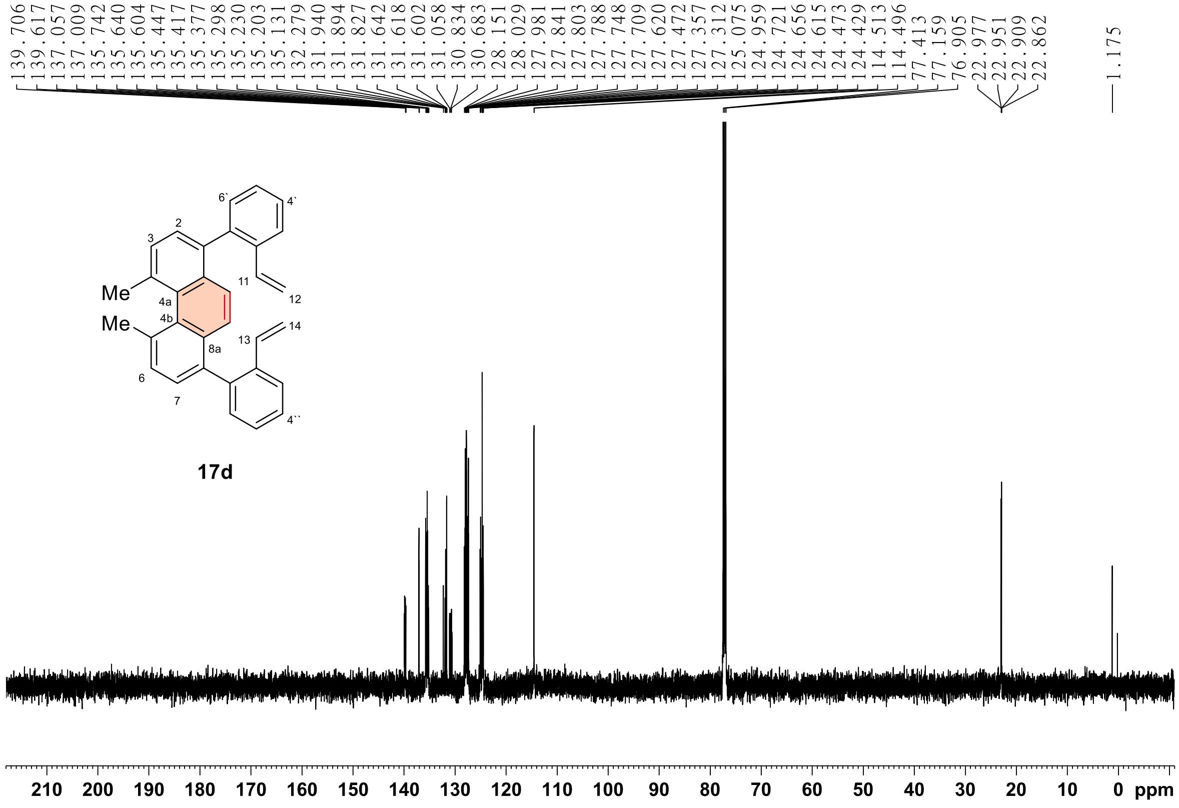

1-(5-Chloro-2-vinylphenyl)-4,5-dimethoxy-8-(2-vinylphenyl)phenanthrene (17e):

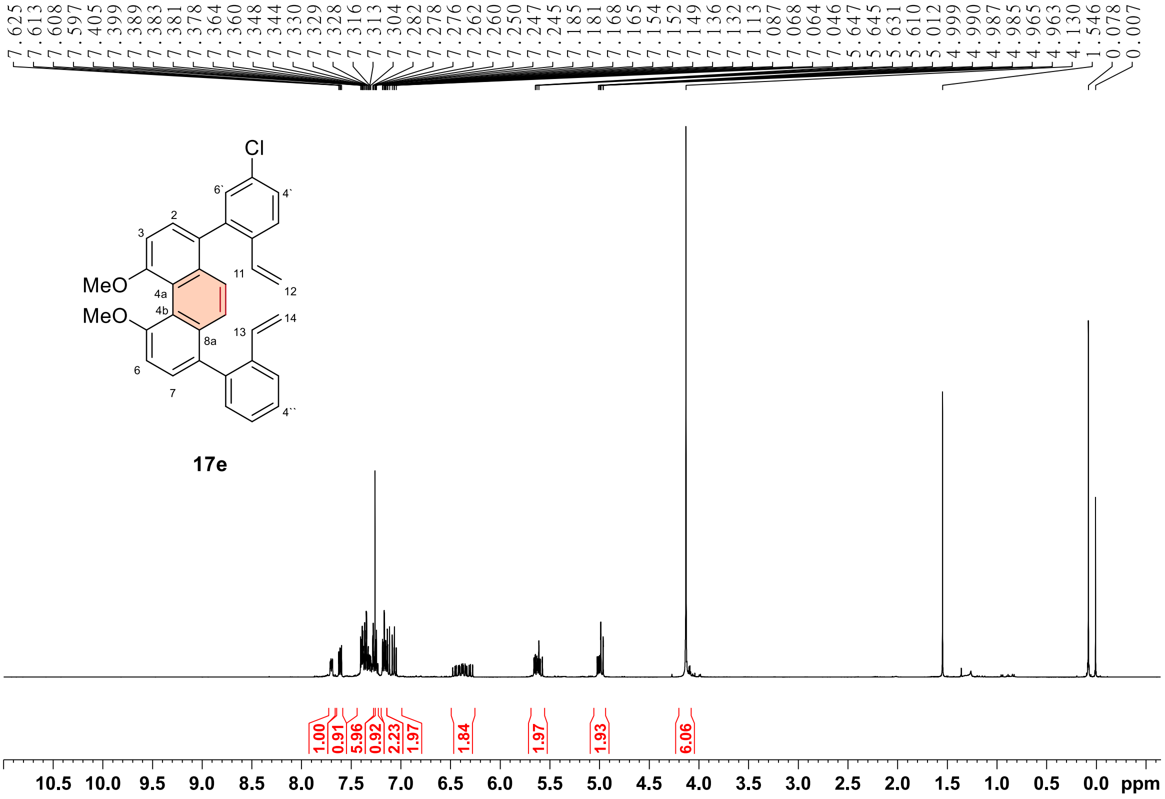

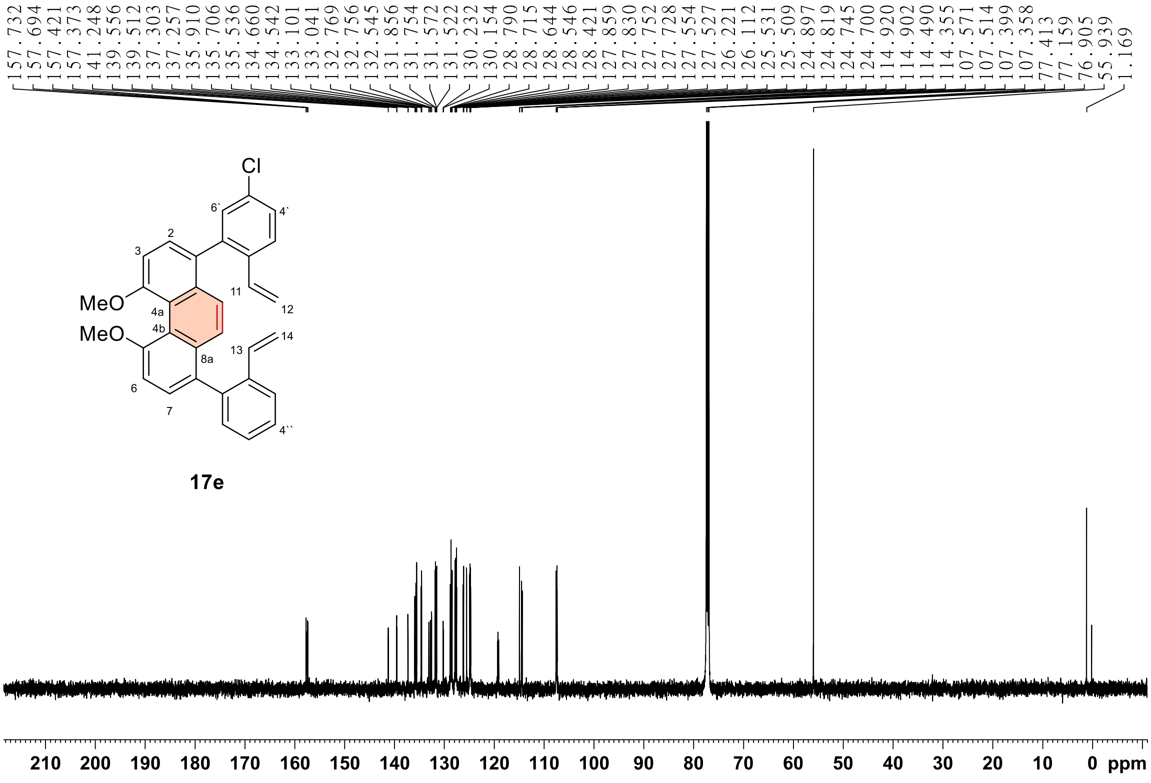

1,8-Bis(5-fluoro-2-vinylphenyl)-4,5-dimethoxyphenanthrene (17f):

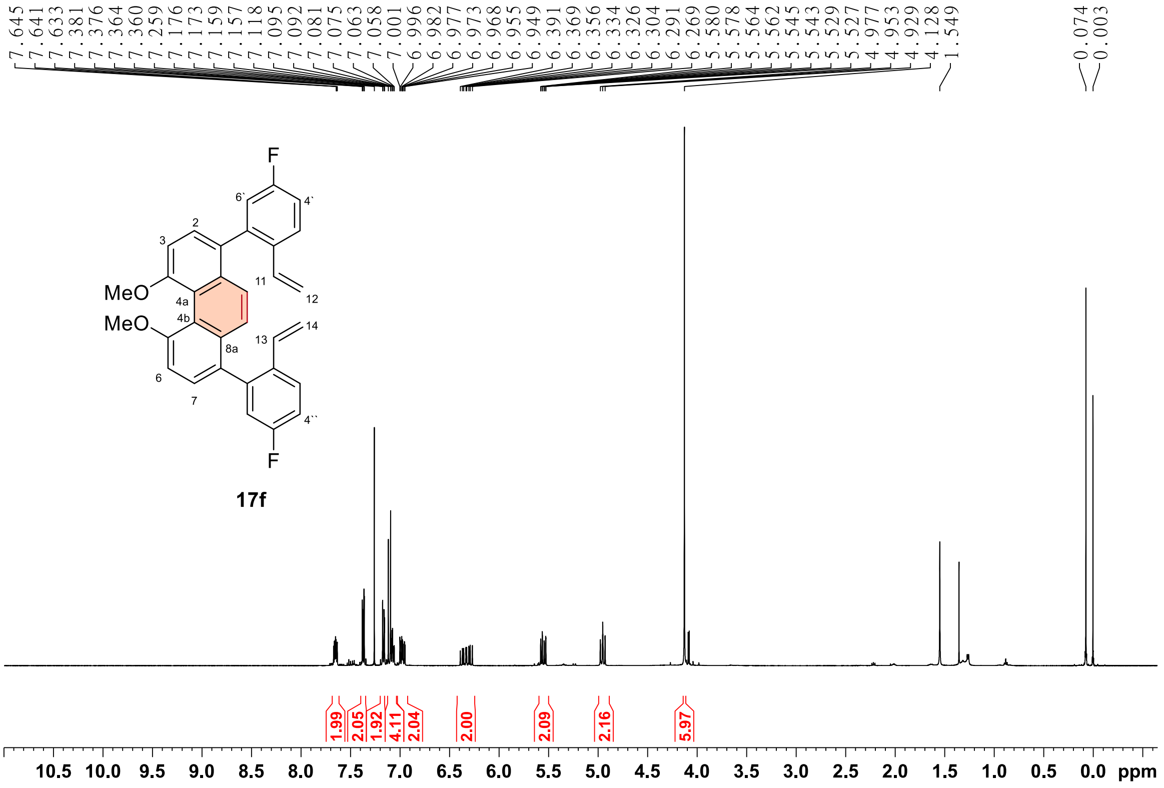

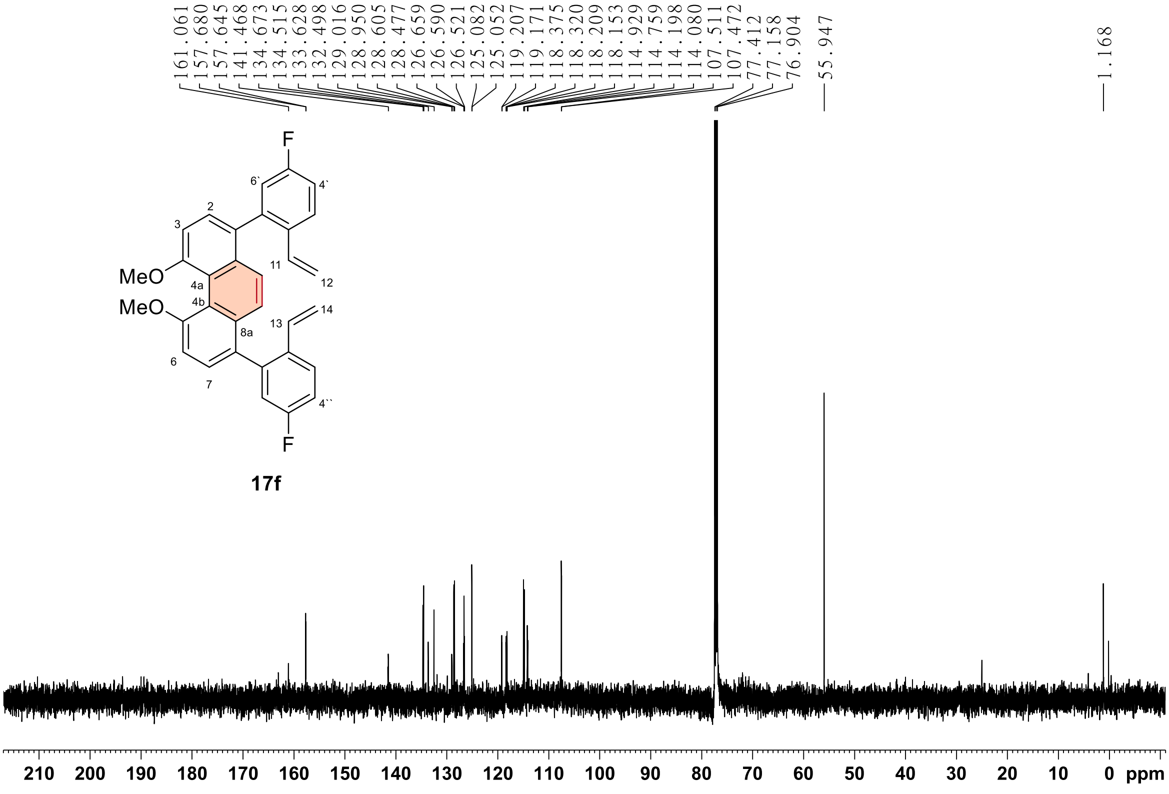

4-Methoxy-1,8-bis(5-methoxy-2-vinylphenyl)-5-methylphenanthrene (17g):

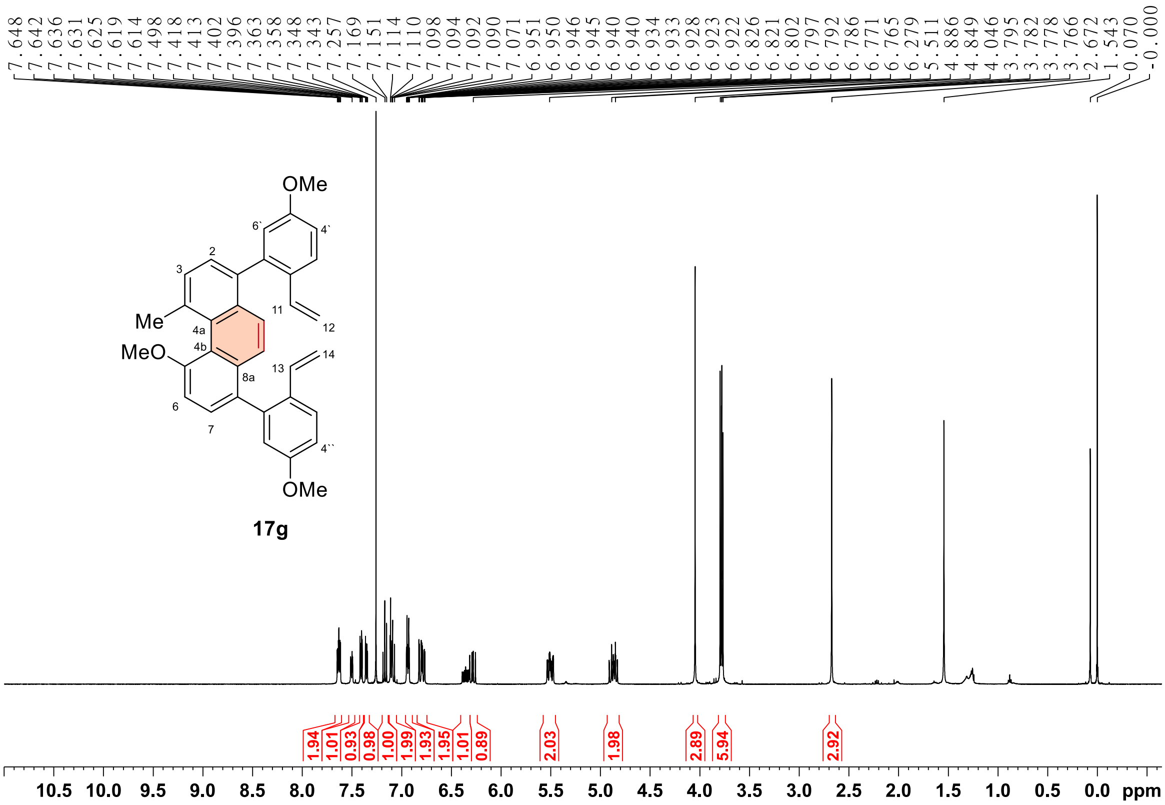

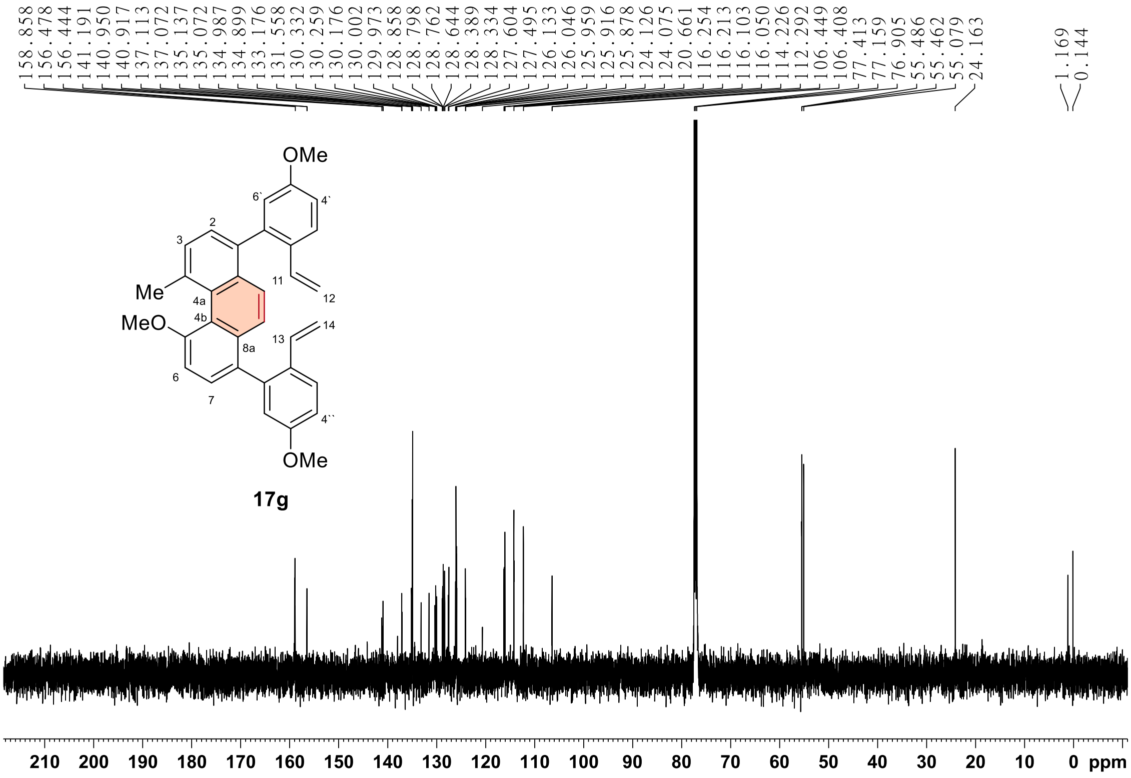

4,5-Dimethoxy-1,8-bis(5-(trifluoromethyl)-2-vinylphenyl)phenanthrene (17h):

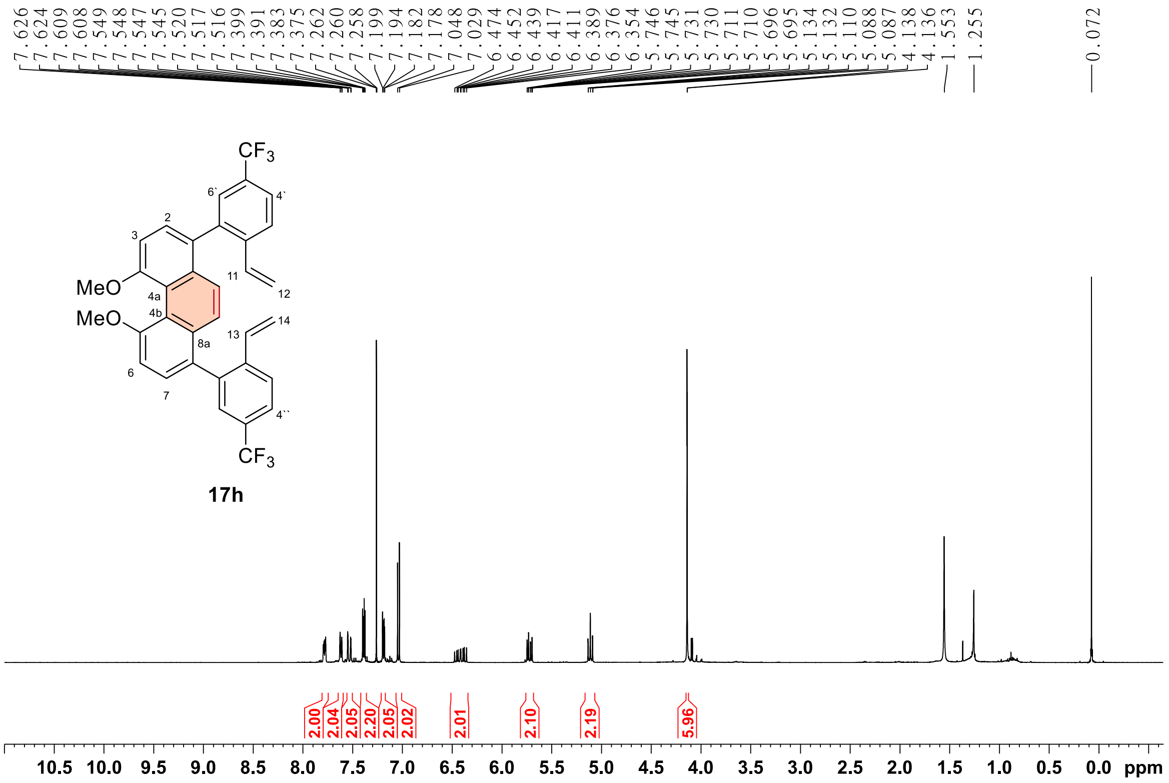

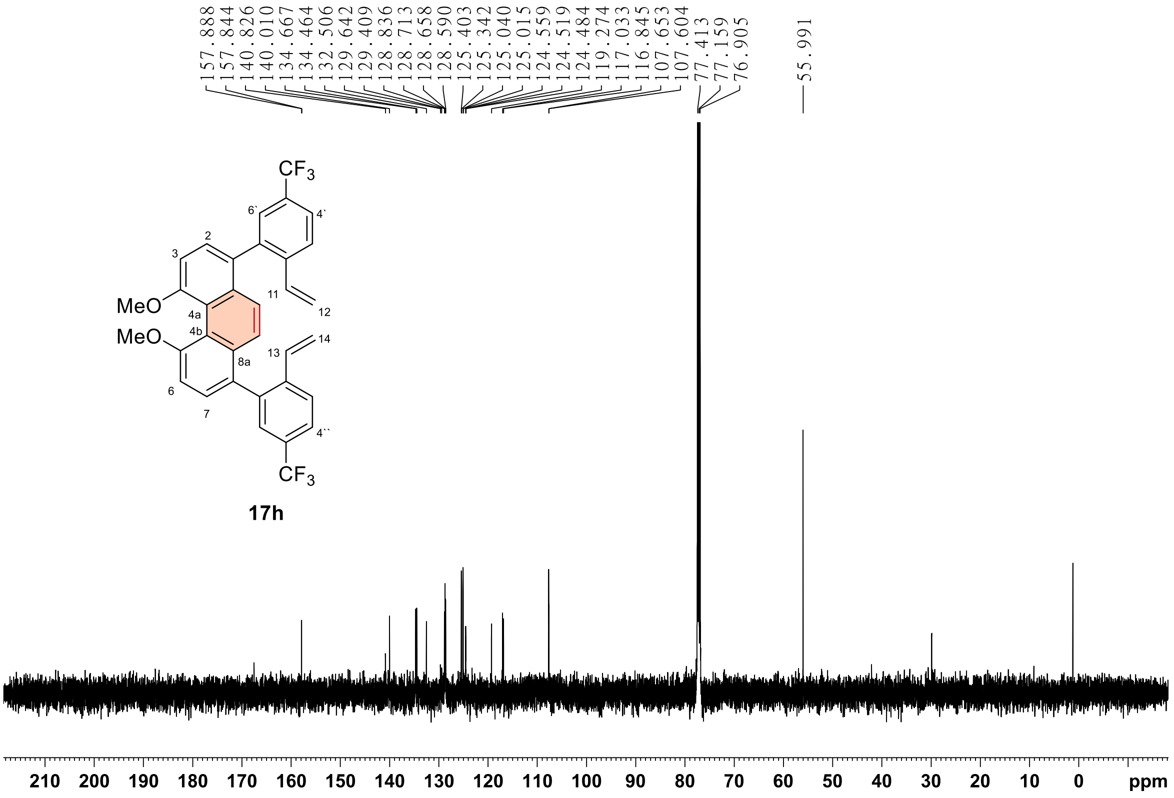

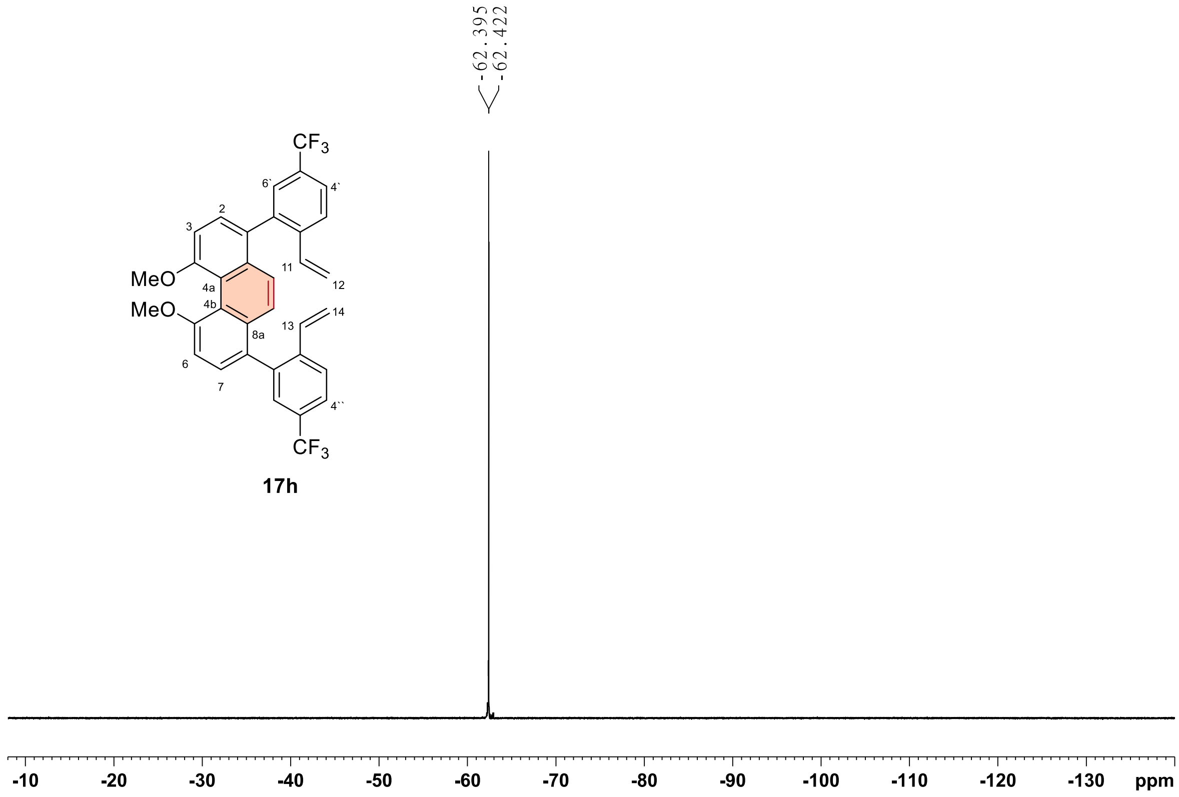
-62.395
-62.422
CF3
6`
4`
2
3
11
12
MeO
4a
4b
MeO
13
14
8a
6
7
4``
CF3
17h
-10
-20
-30
-40
-50
-60
-70
-80
-90
-100
-110
-120
-130
ppm

1,8-Bis(5-chloro-2-vinylphenyl)-4,5-dimethoxyphenanthrene (17i):

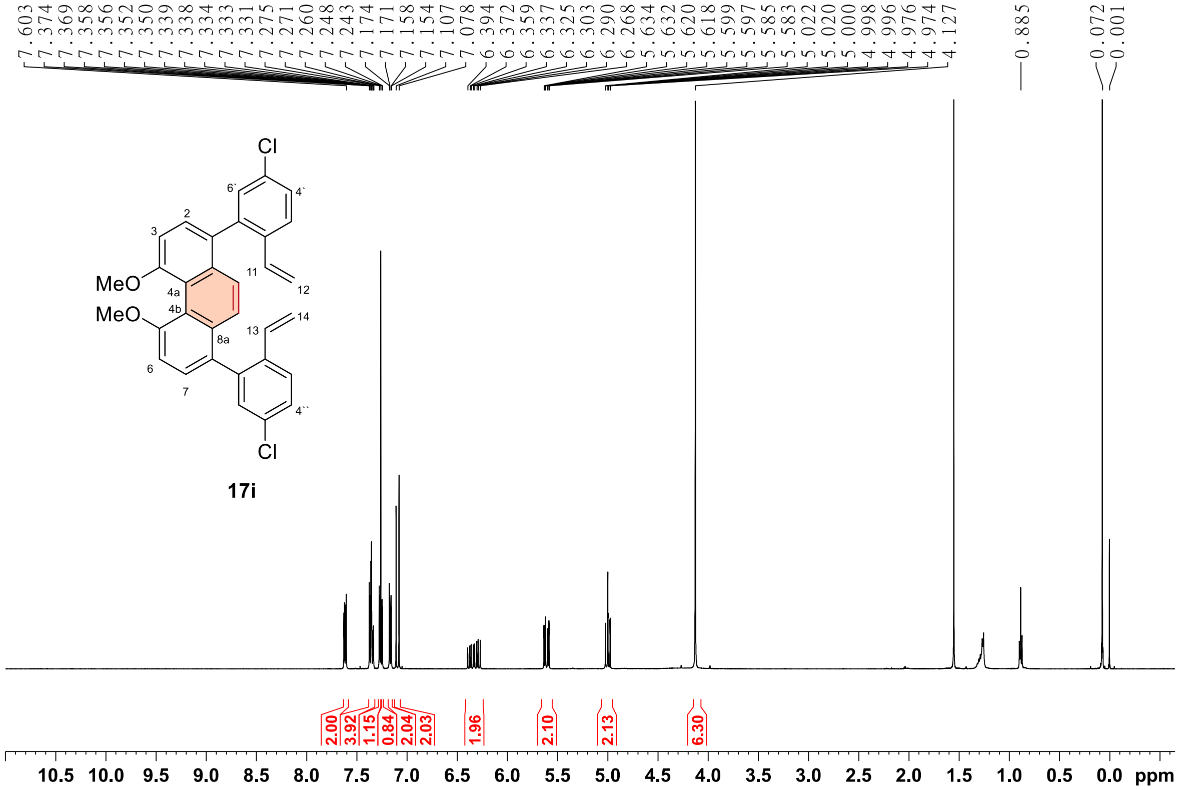

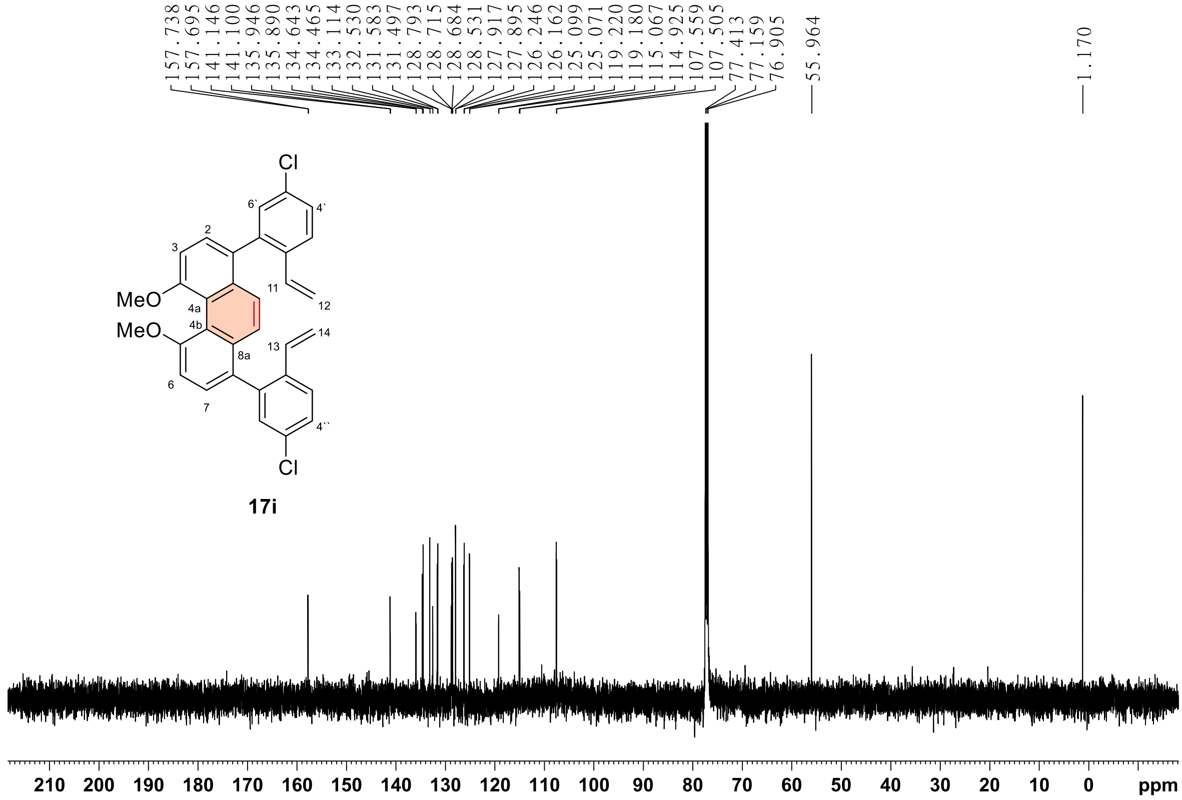

1-(3-Vinylnaphthalen-2-yl)phenanthrene (2):

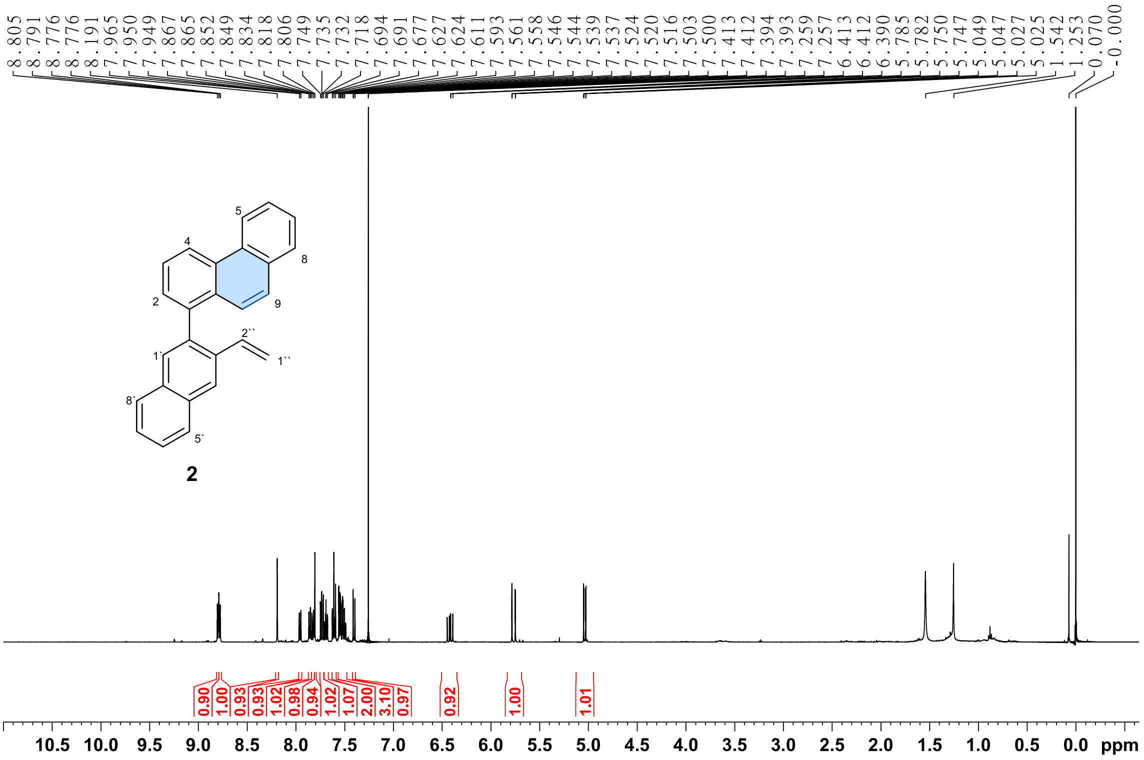

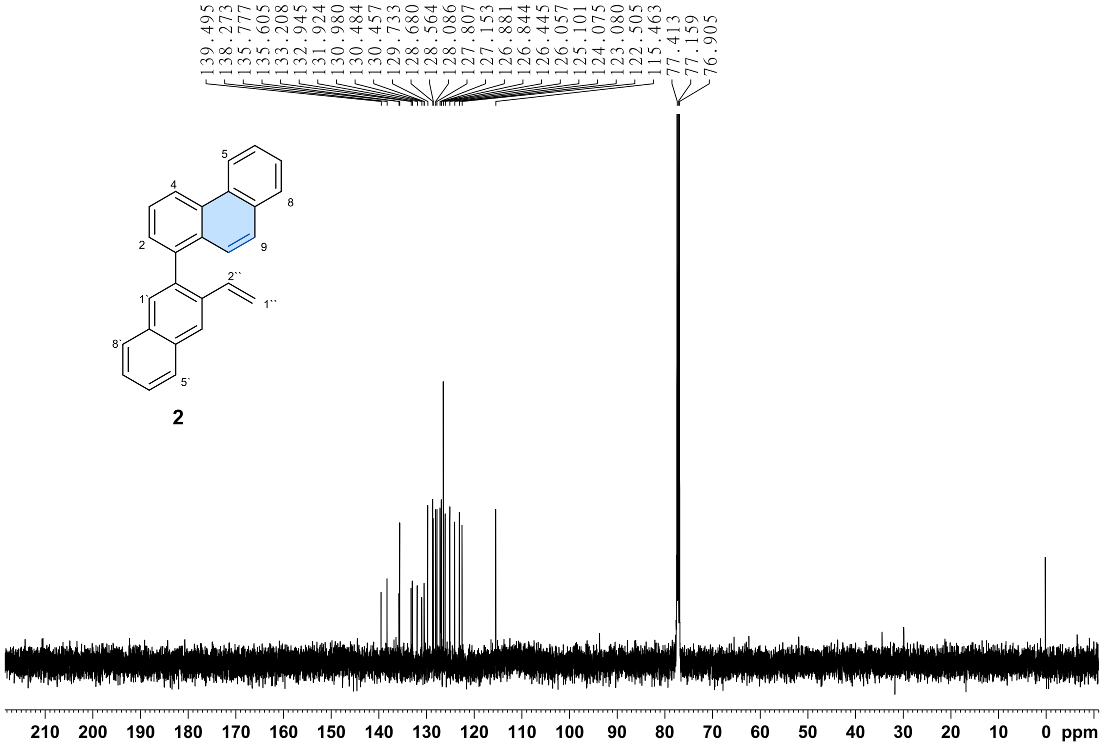

3-Methyl-3*H*-naphtho[1,2-g]indole (6a):

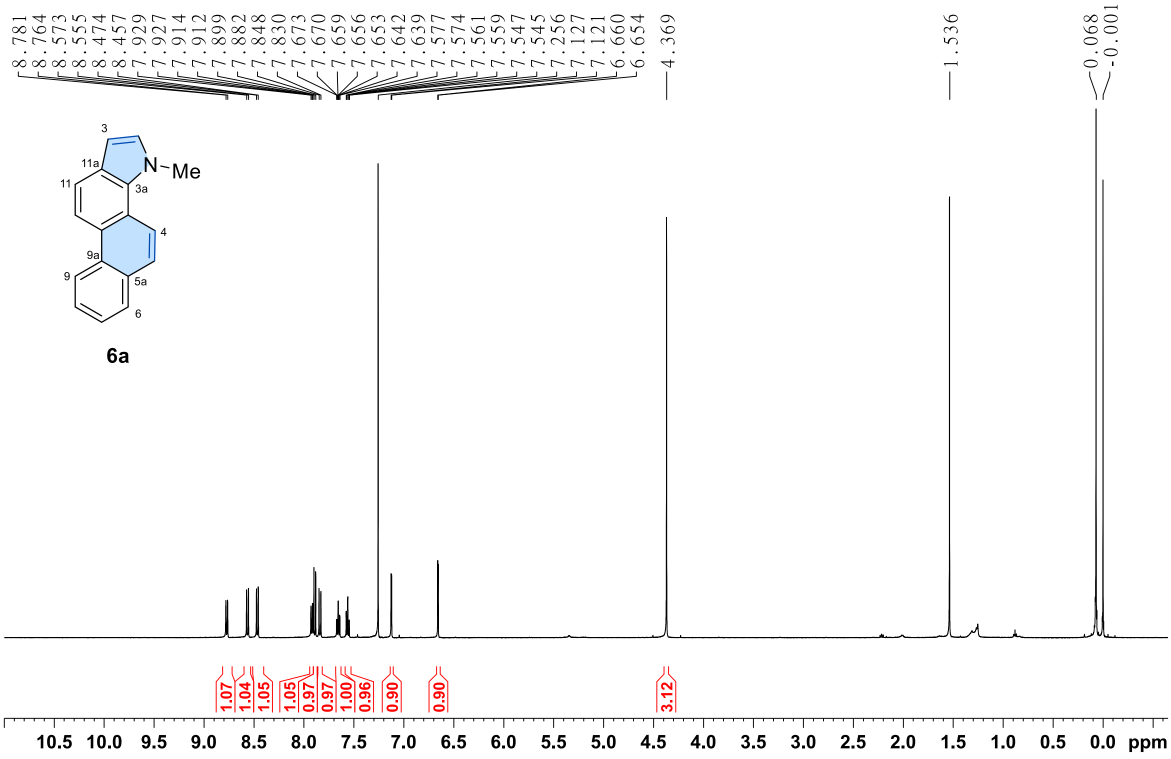

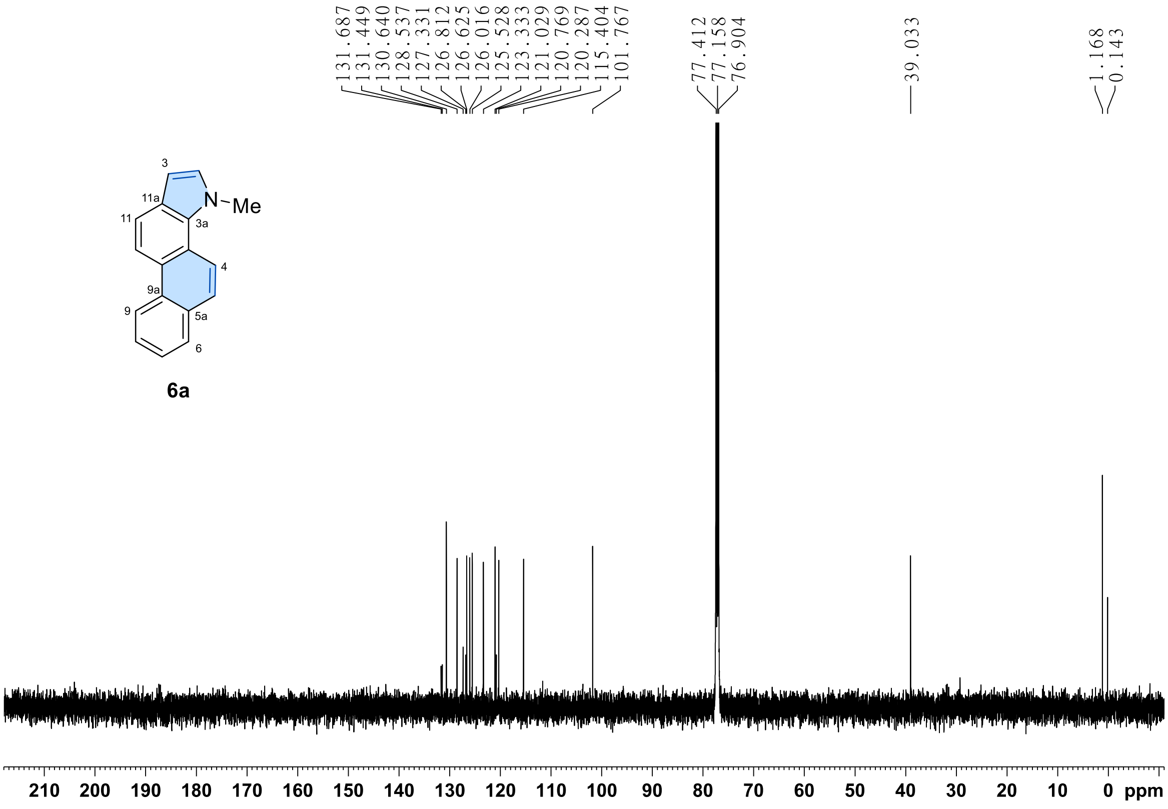

Phenanthro[1,2-b]furan (6b):

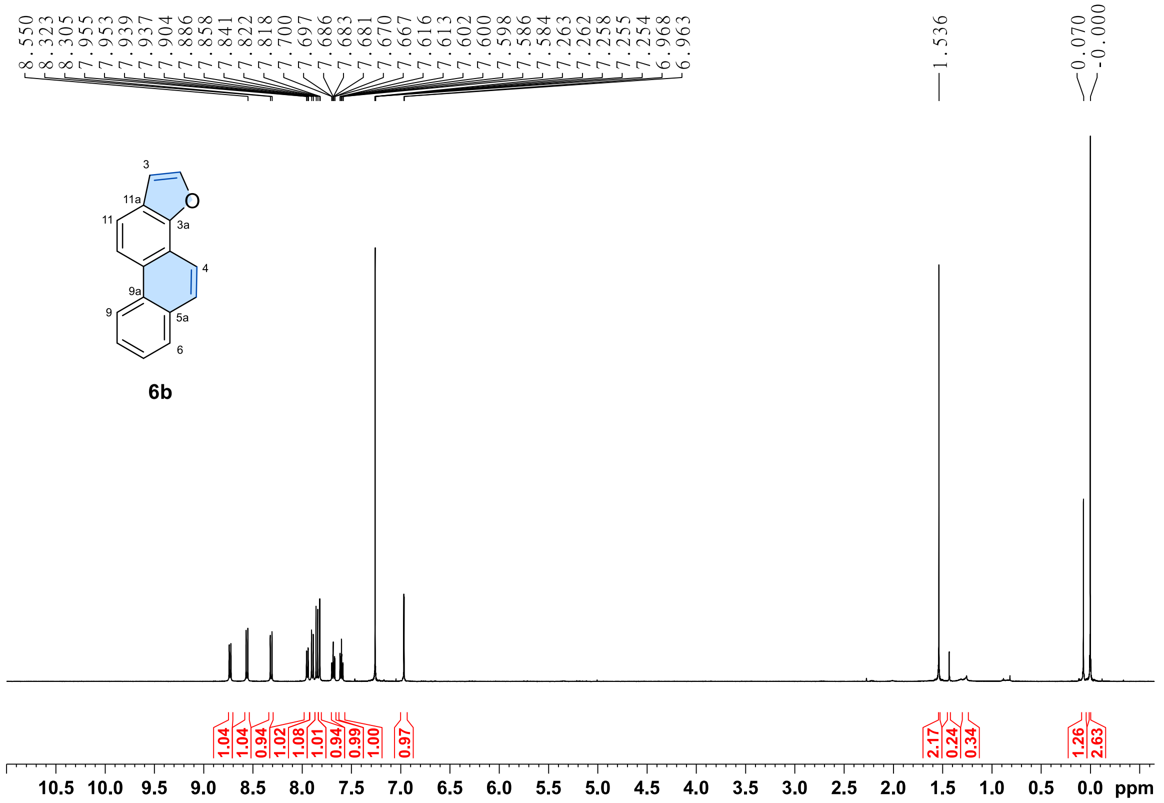

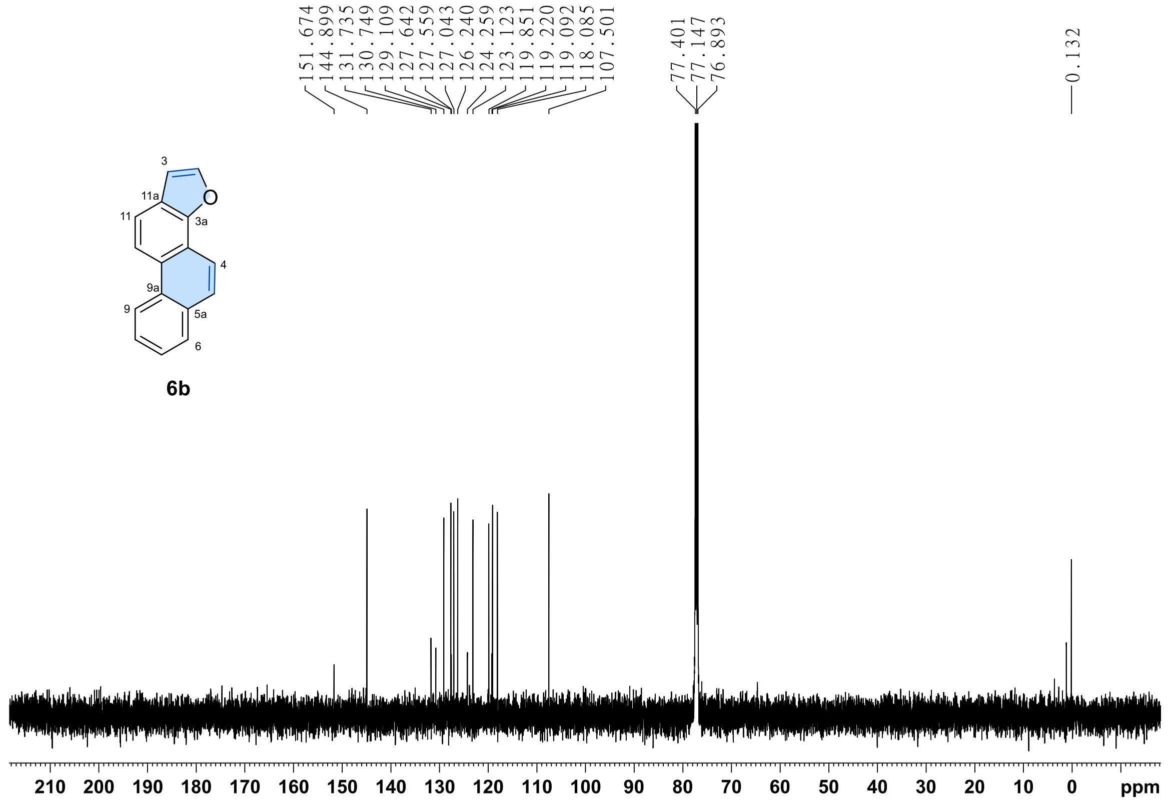

Benzo[*k*]tetraphene (8a):

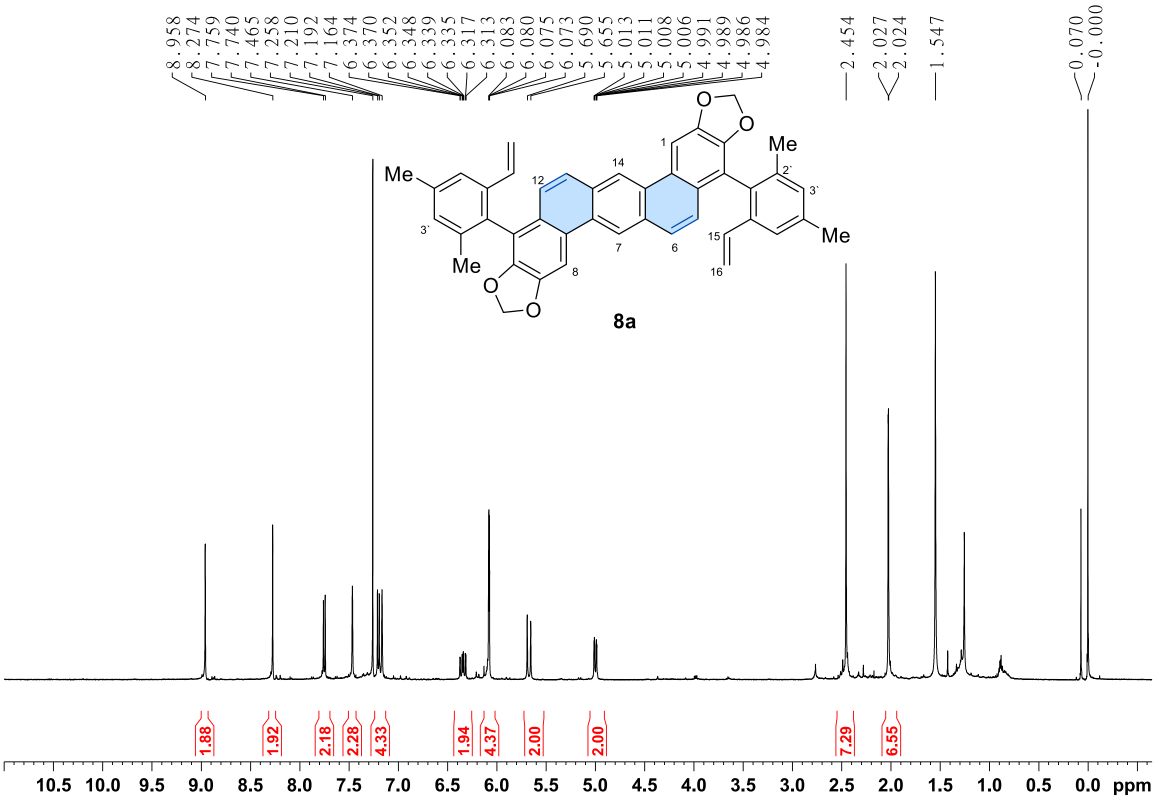

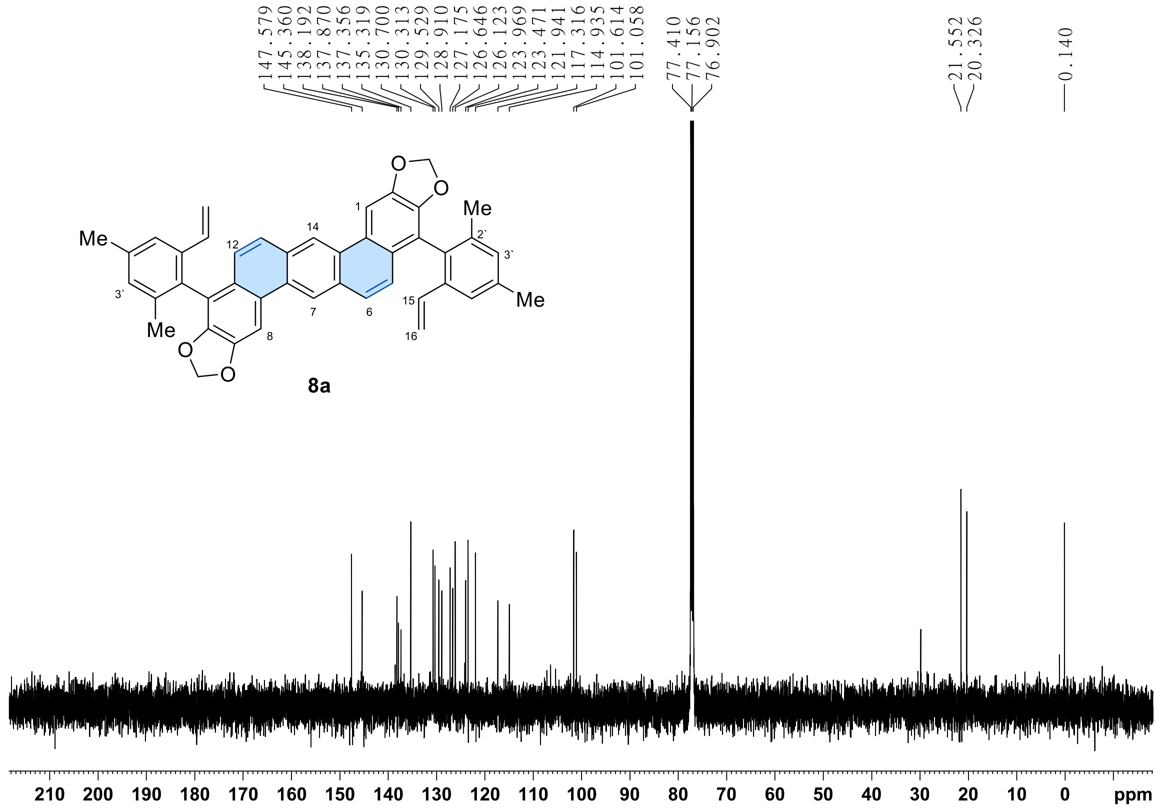

4,11-Bis(2,4-dimethoxy-6-vinylphenyl)-3,10-dimethylbenzo[*k*]tetraphene (8b):

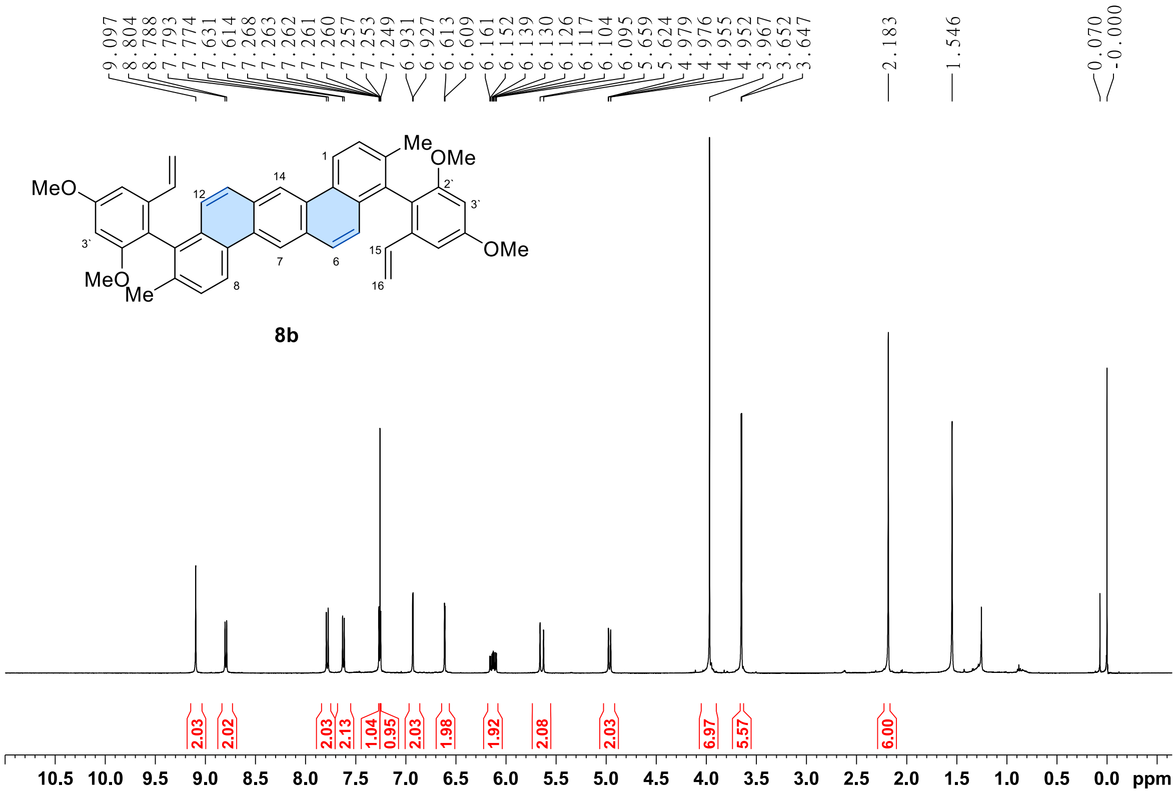

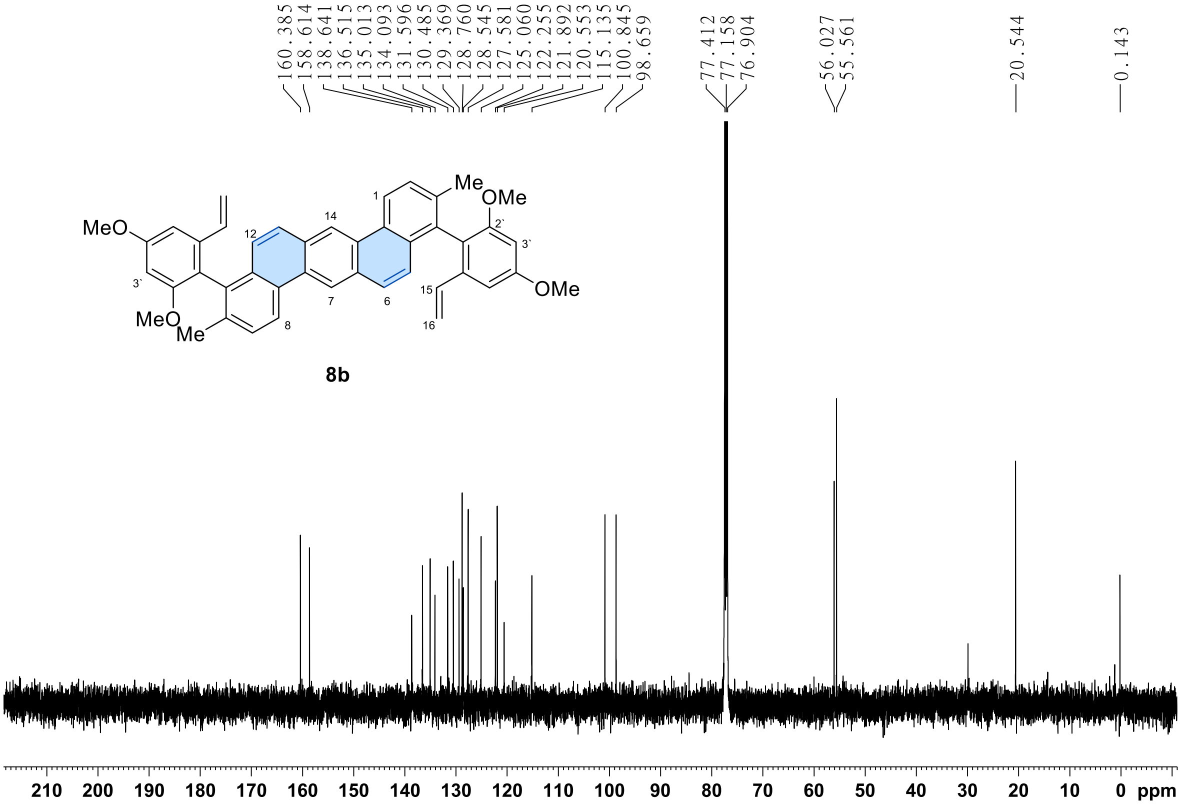

4,10-Bis(2,4-dimethoxy-6-vinylphenyl)-3,11-dimethylbenzo[*m*]tetraphene (8c):

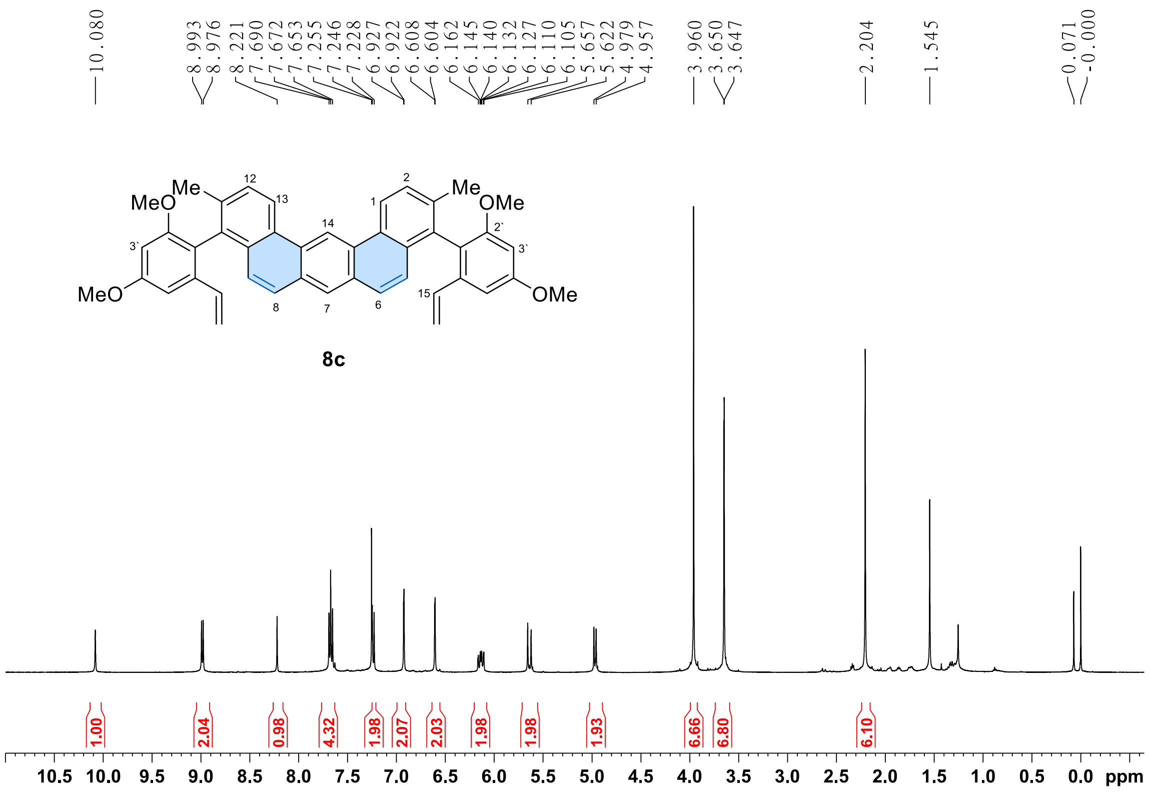

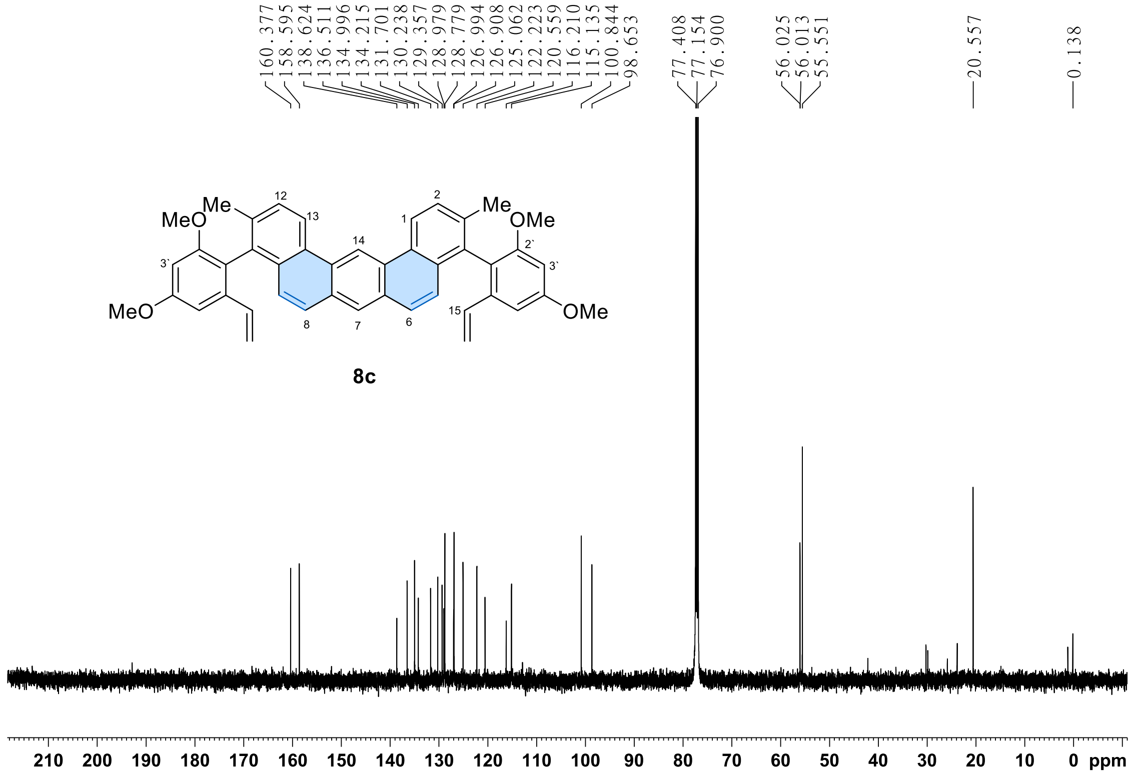

1-(4-Methoxy-2-vinylphenyl)-1*H*-indole (10a):

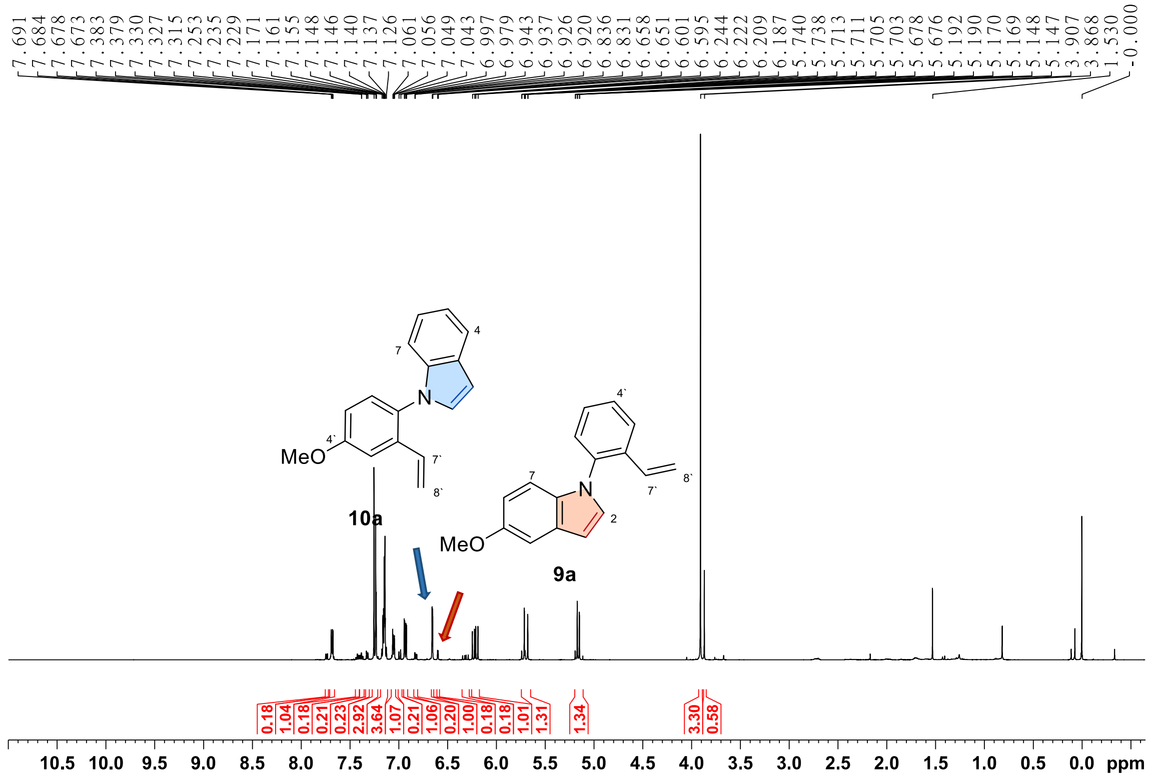

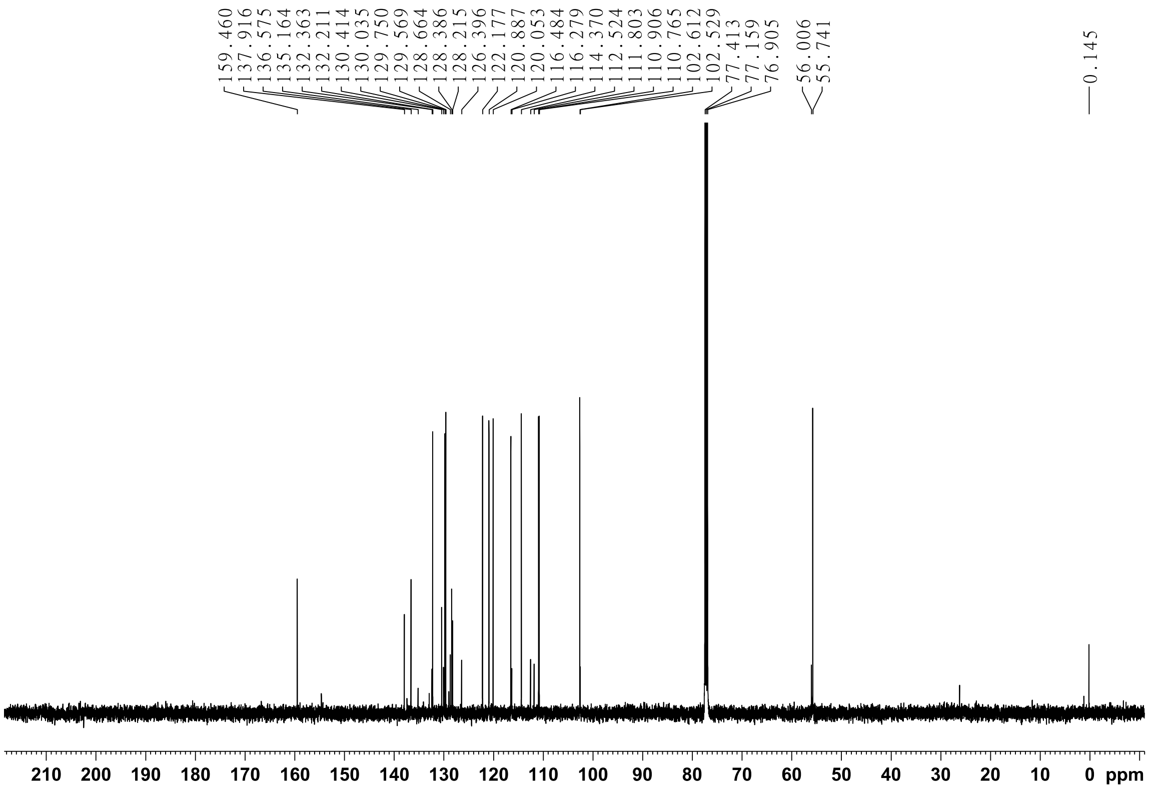

1-(4-Methyl-2-vinylphenyl)-1*H*-indole (10b):

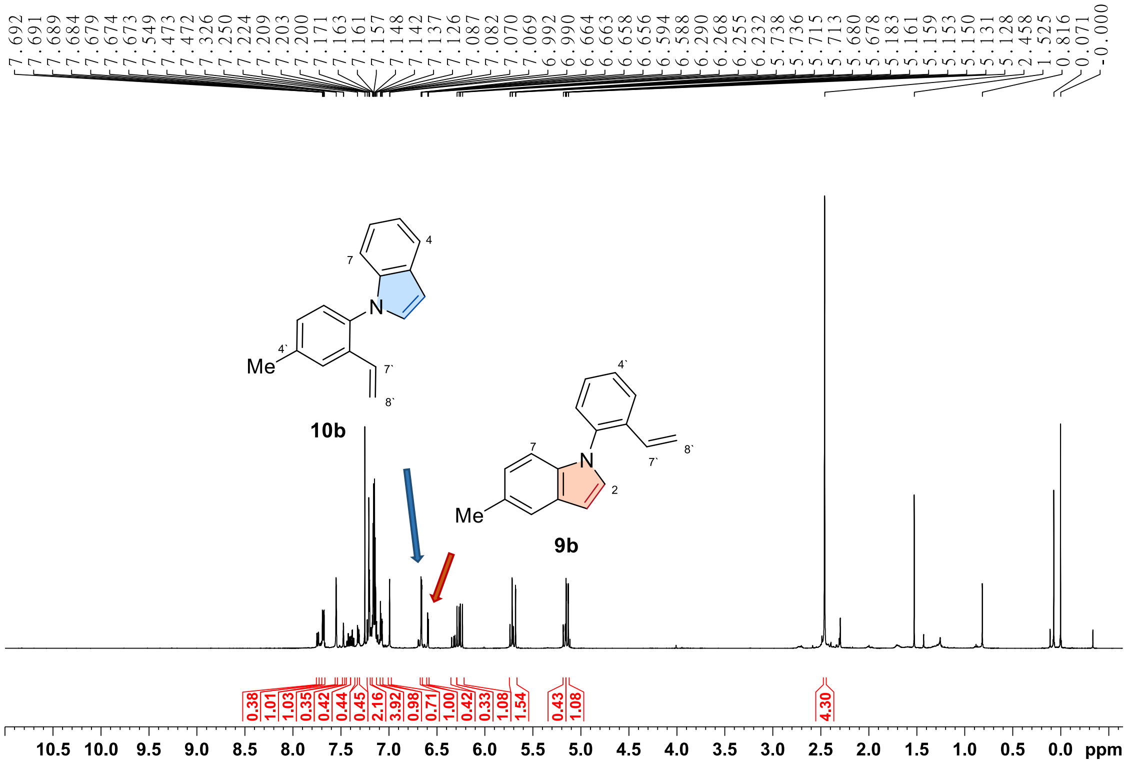

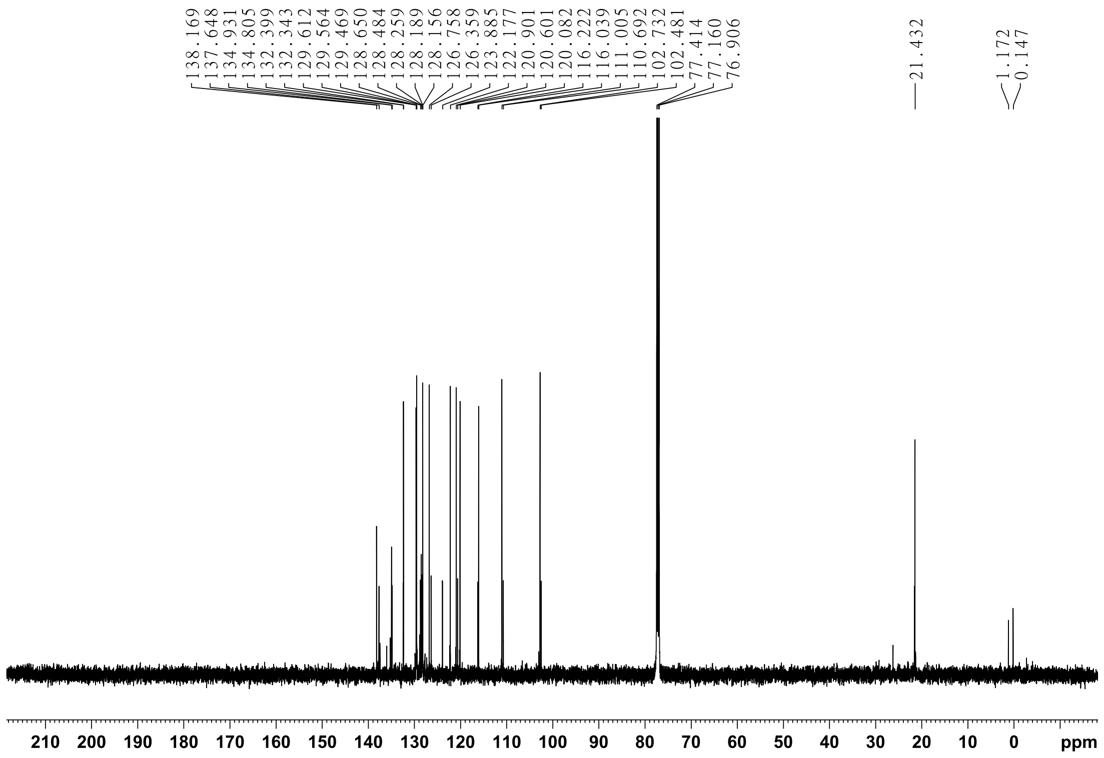

1-(2-Fluoro-6-vinylphenyl)-1*H*-indole (10c):

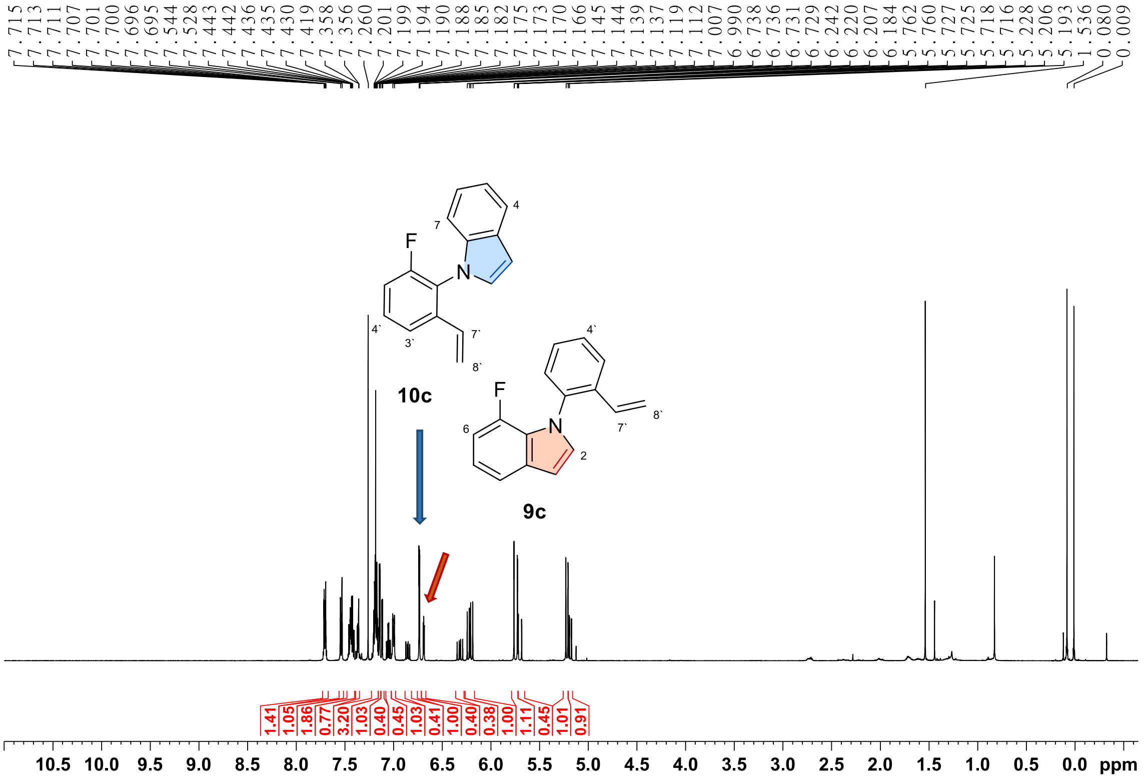

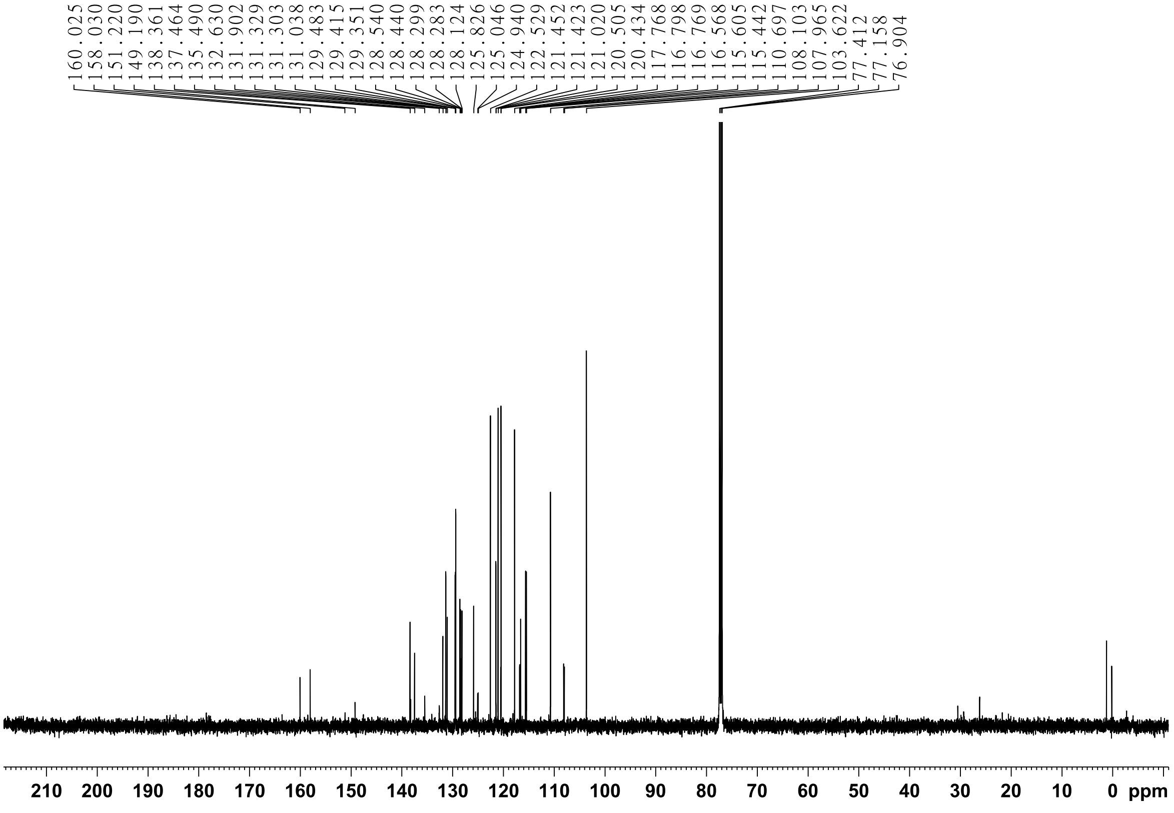

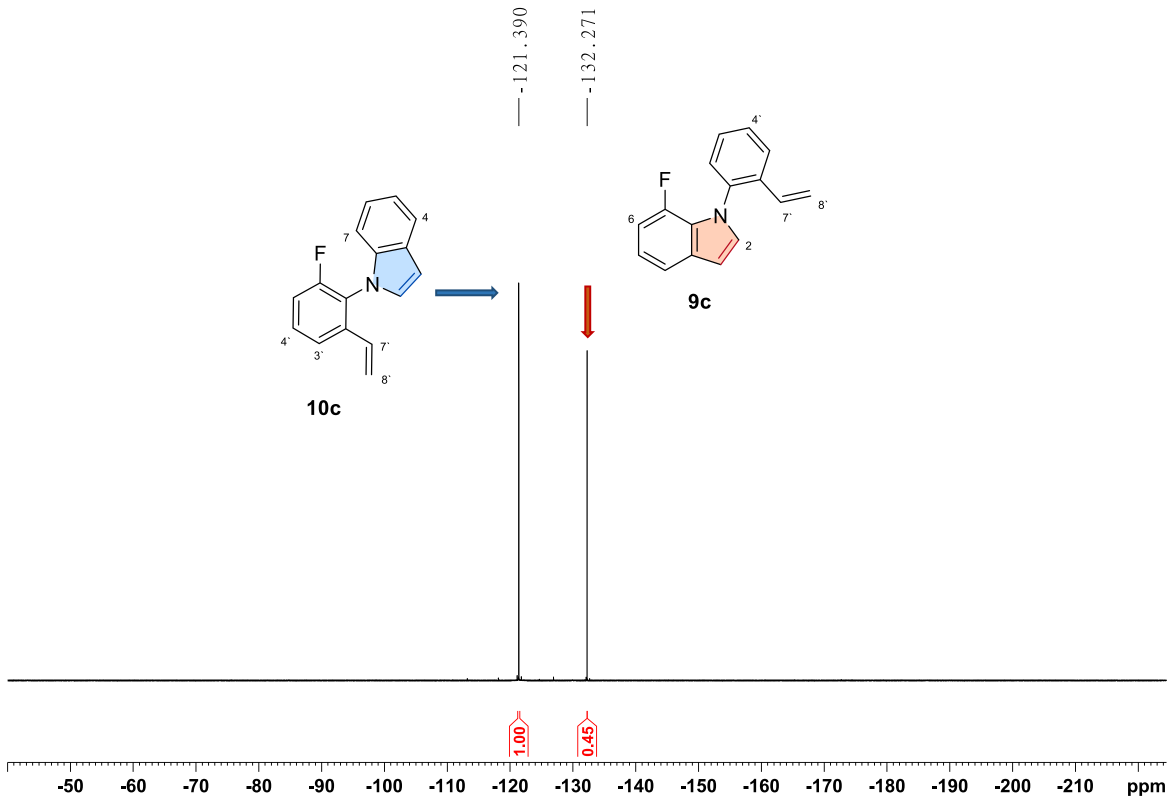

-121.390
-132.271
10c
9c
1.00
0.45
-50 -60 -70 -80 -90 -100 -110 -120 -130 -140 -150 -160 -170 -180 -190 -200 -210 ppm

1-(4-Fluoro-2-vinylphenyl)-1*H*-indole (10d):

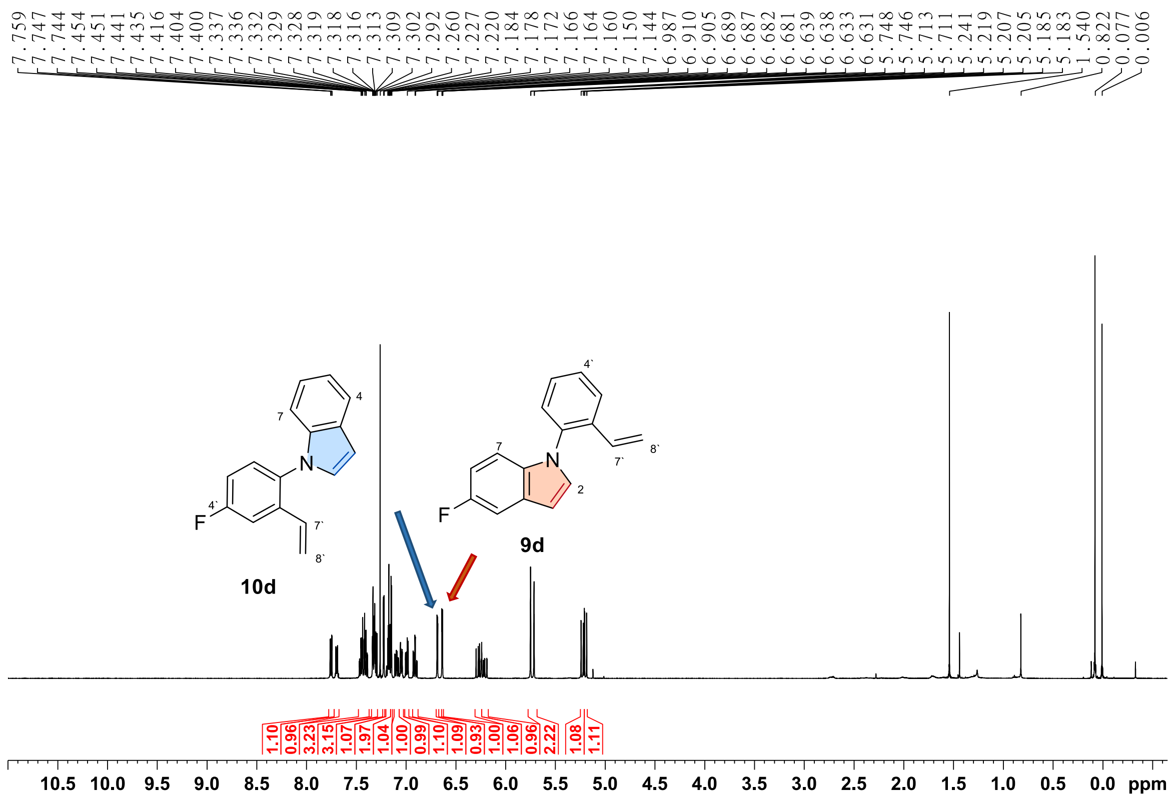

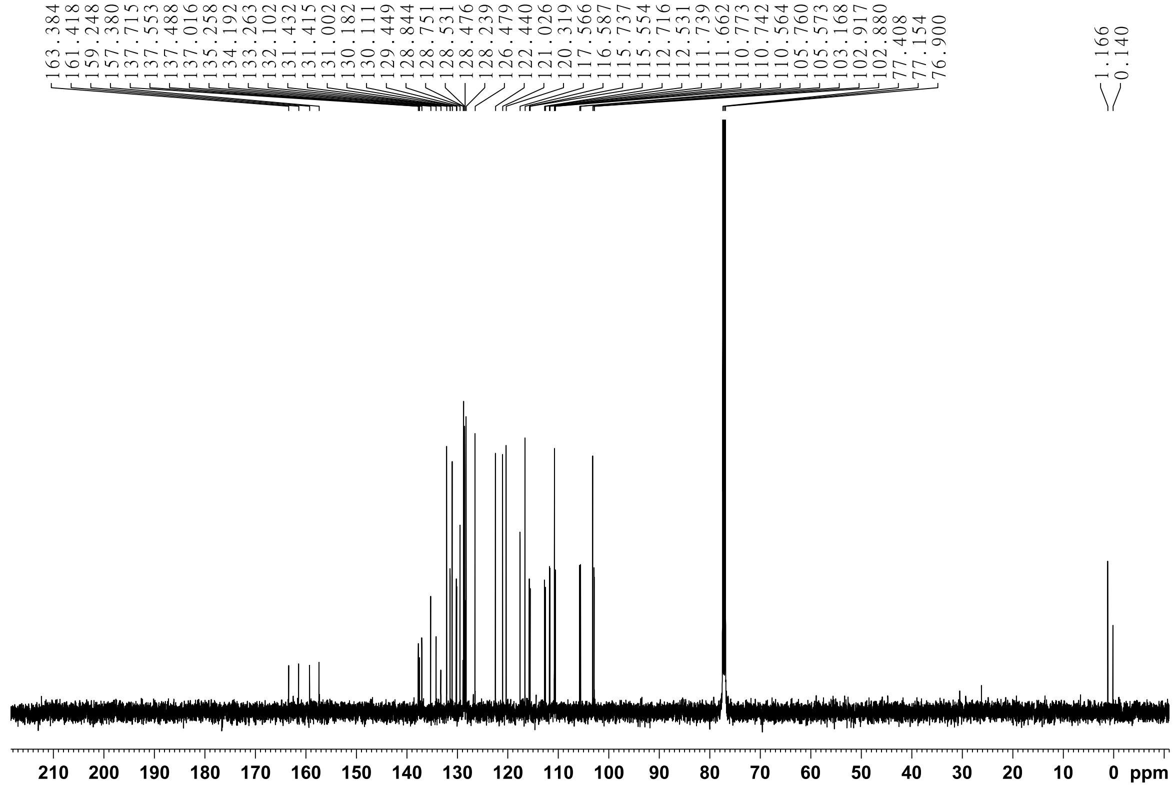

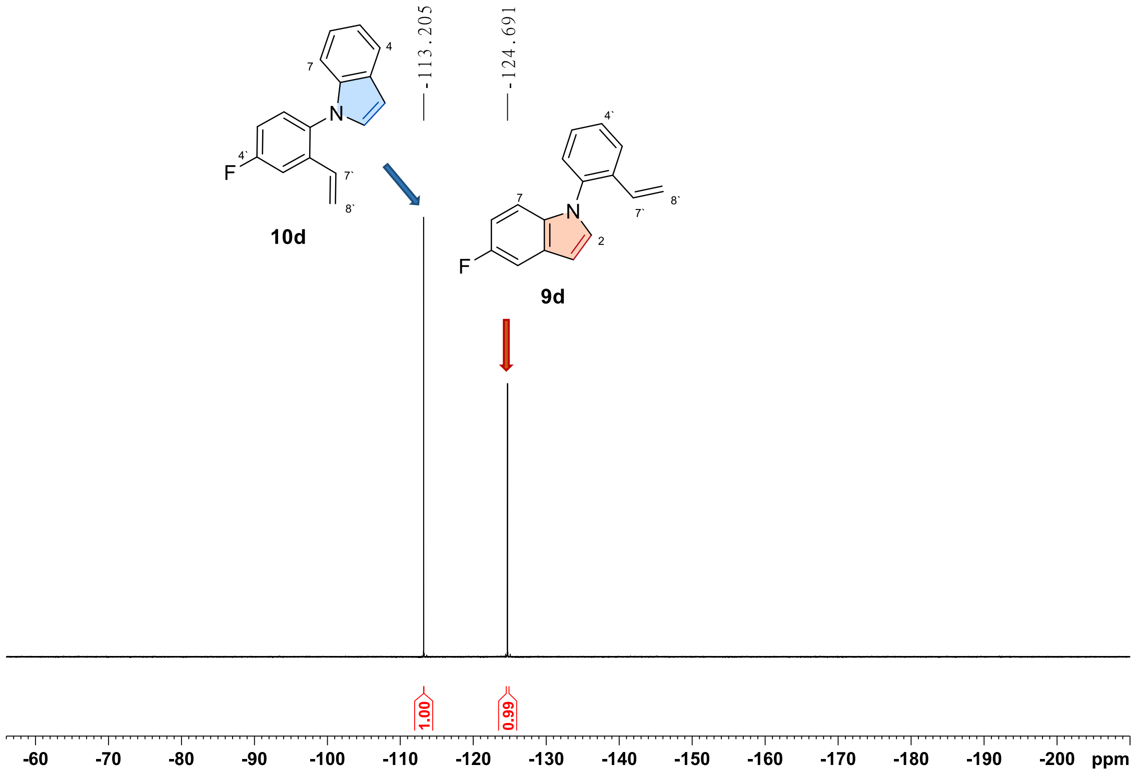

-113.205
-124.691
10d
9d
F
N
1.00
0.99
-60
-70
-80
-90
-100
-110
-120
-130
-140
-150
-160
-170
-180
-190
-200
ppm

1-(5-Fluoro-2-vinylphenyl)-1*H*-indole (10e):

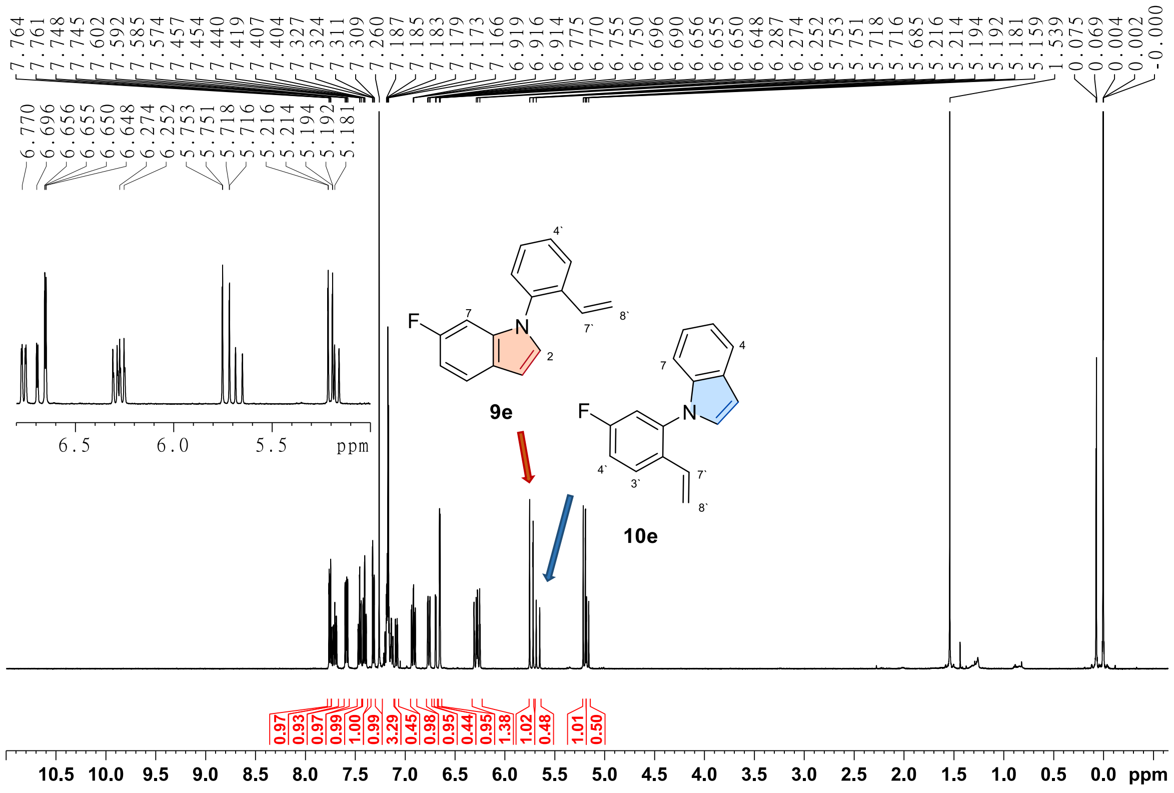

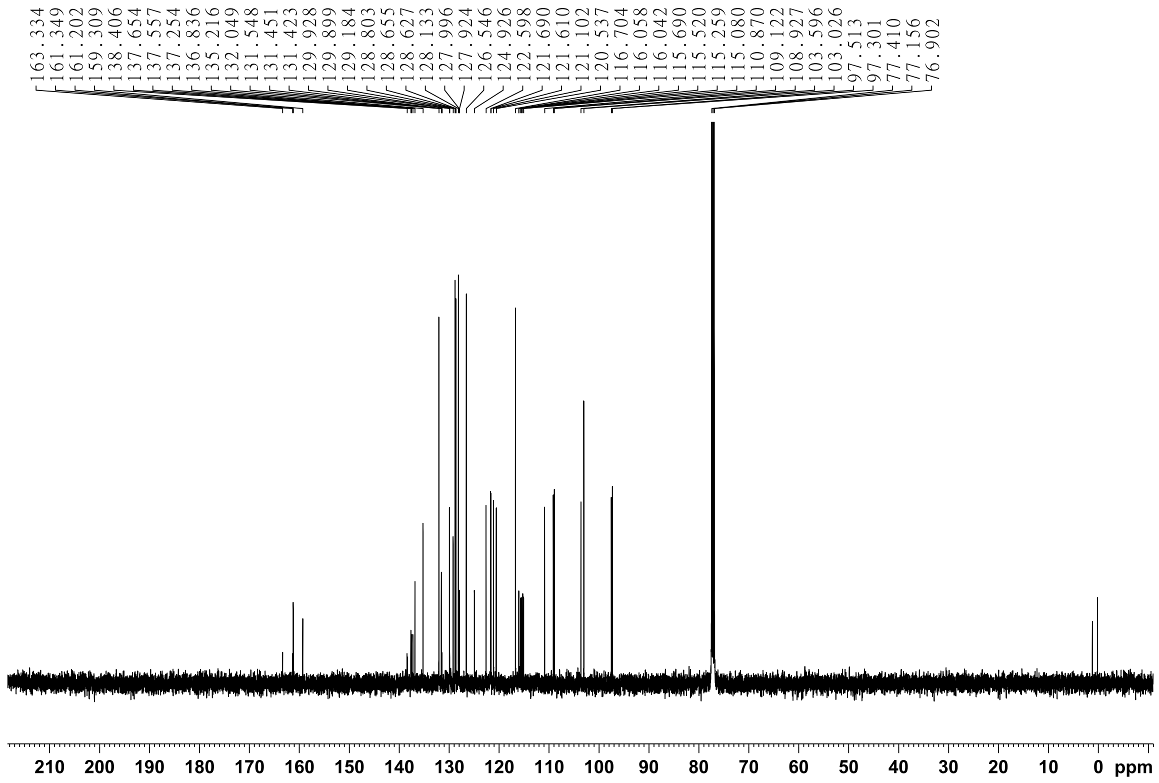

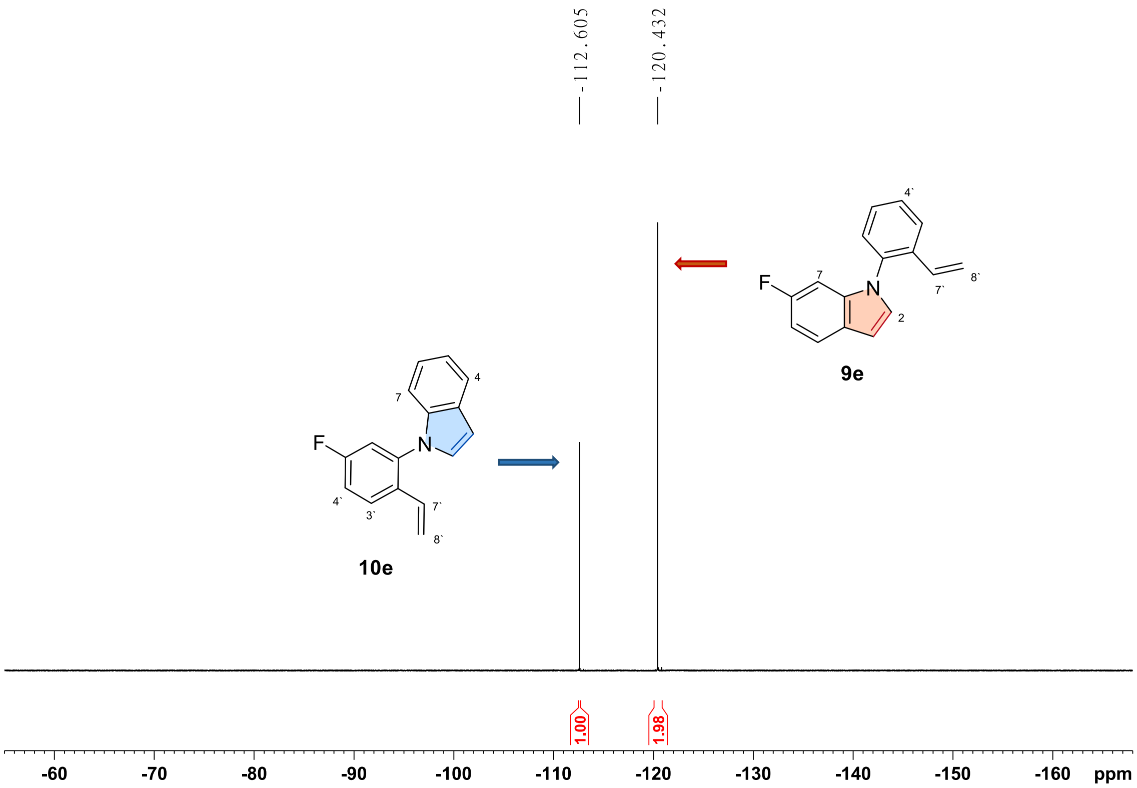

-112.605
-120.432
F
N
7
4
4`
3`
7`
8`
10e
F
7
N
2
4`
7`
8`
9e
1.00
1.98
-60
-70
-80
-90
-100
-110
-120
-130
-140
-150
-160
ppm

1-(4-Chloro-2-vinylphenyl)-1*H*-indole (10f):

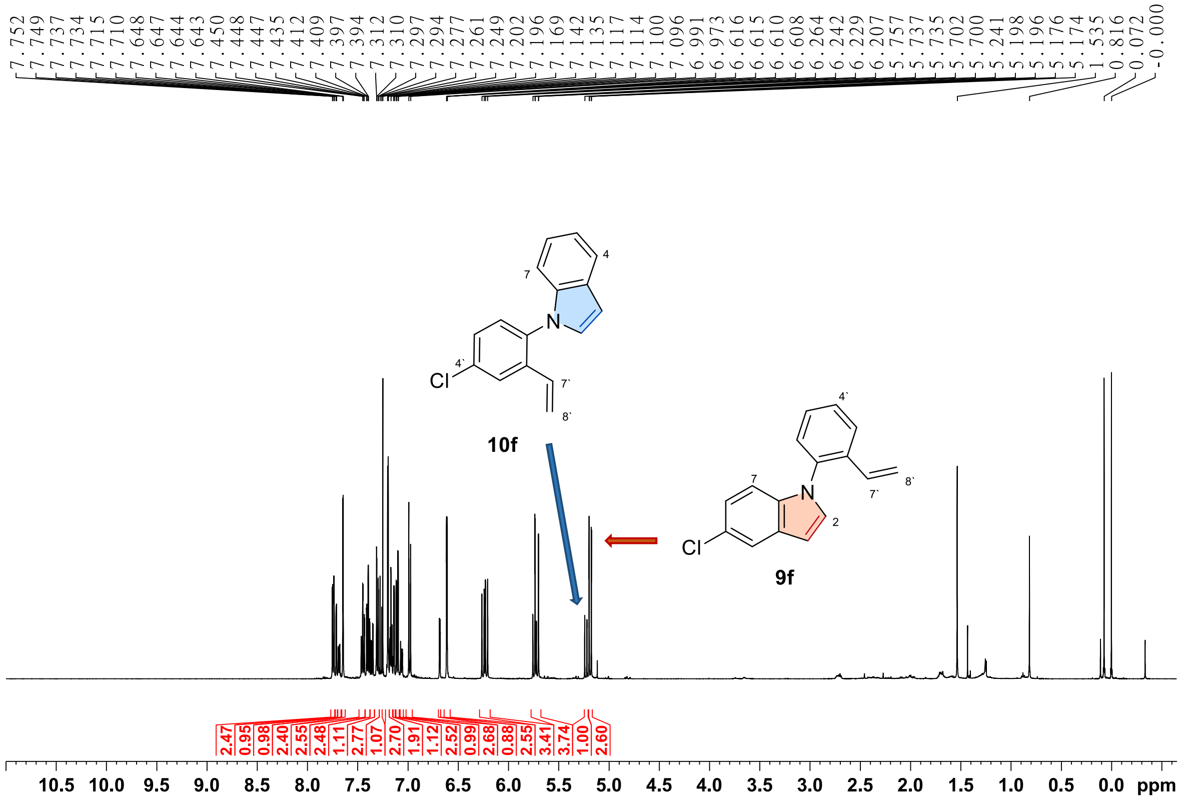

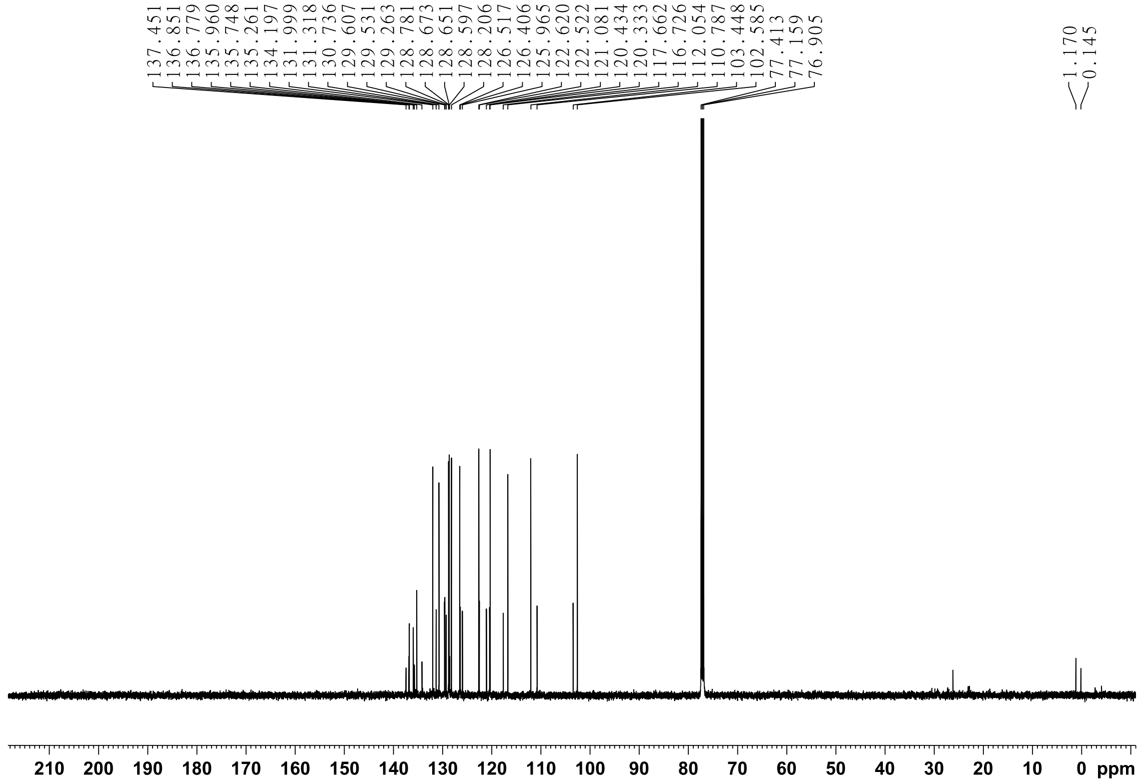

1-(5-Chloro-2-vinylphenyl)-1*H*-indole (10g):

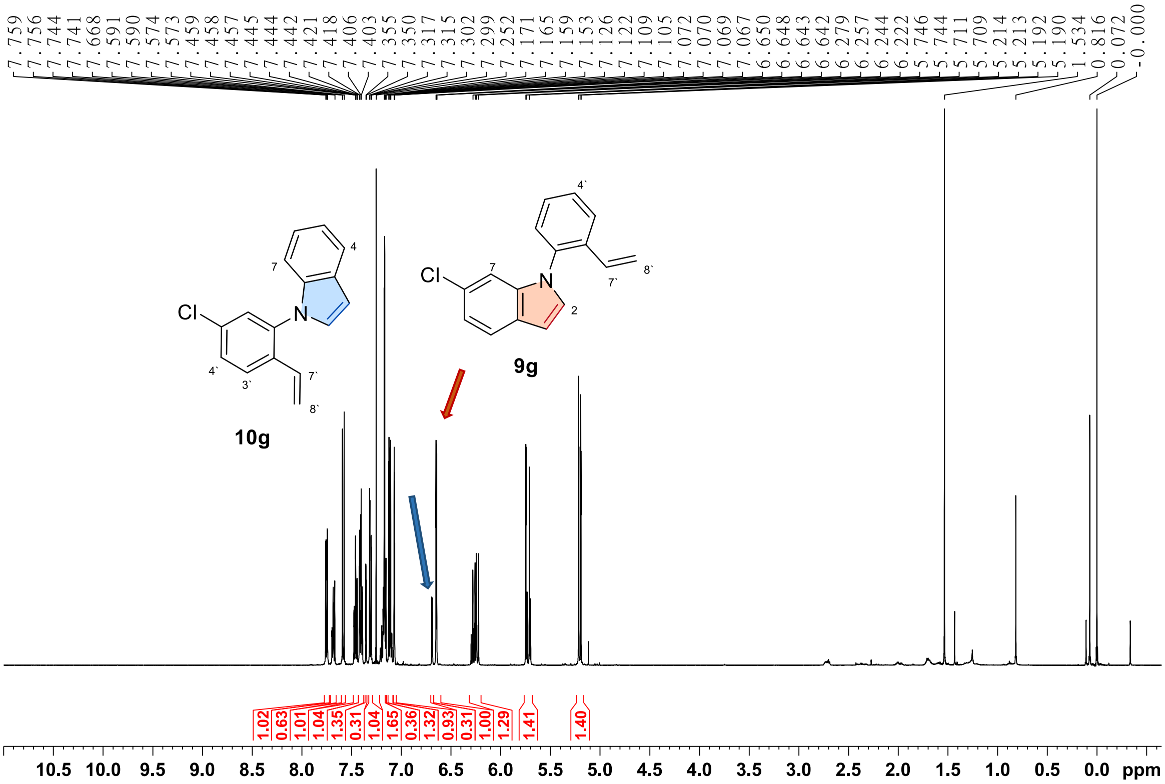

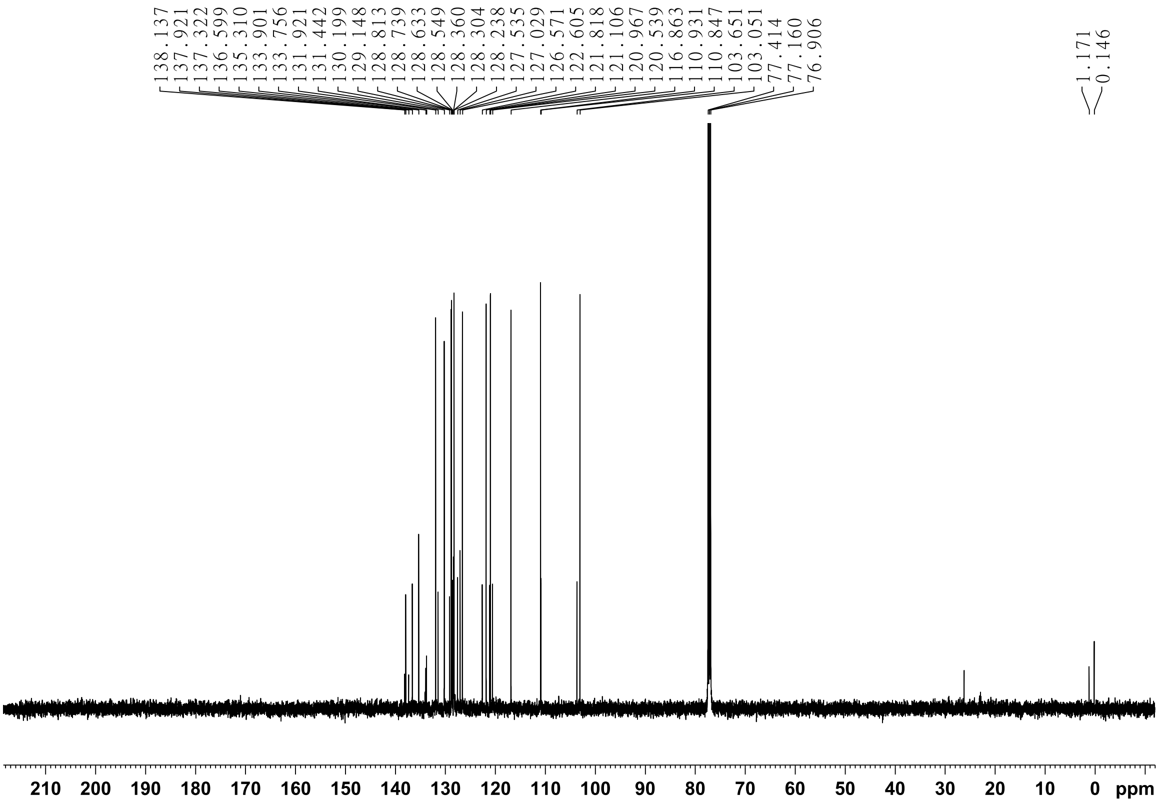

1,8-Di(1-*H*-indol-1-yl)phenanthrene (12):

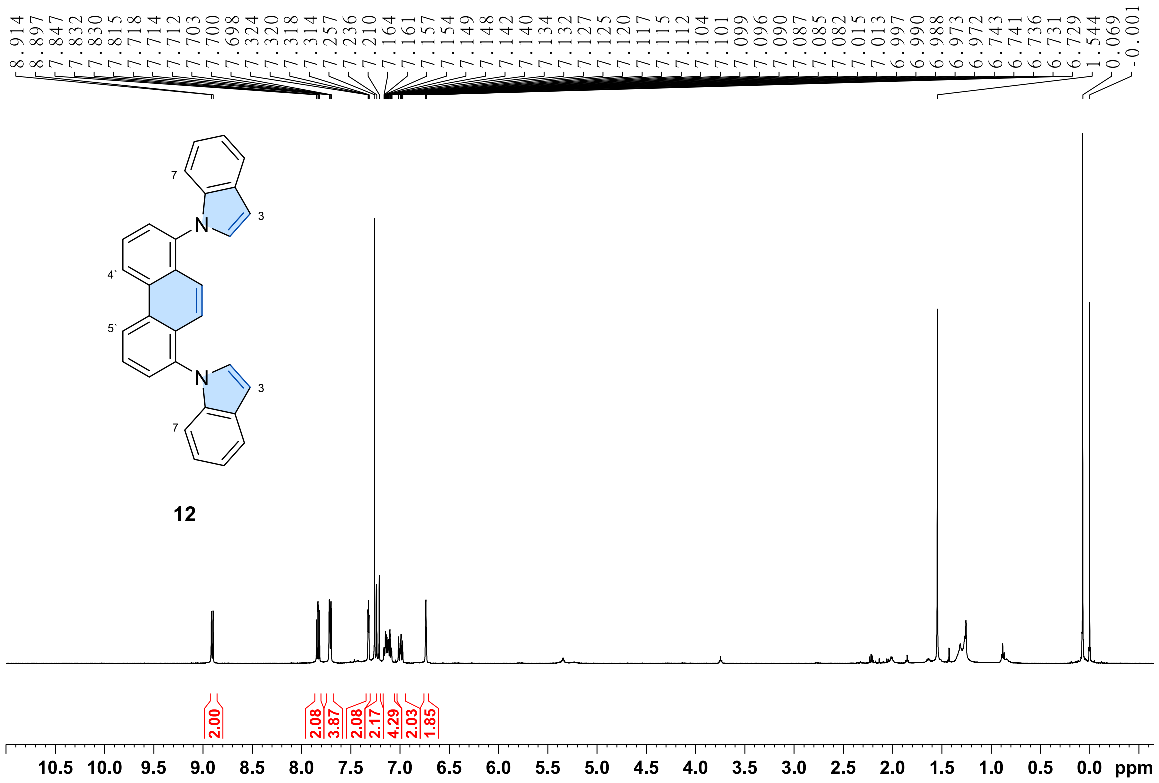

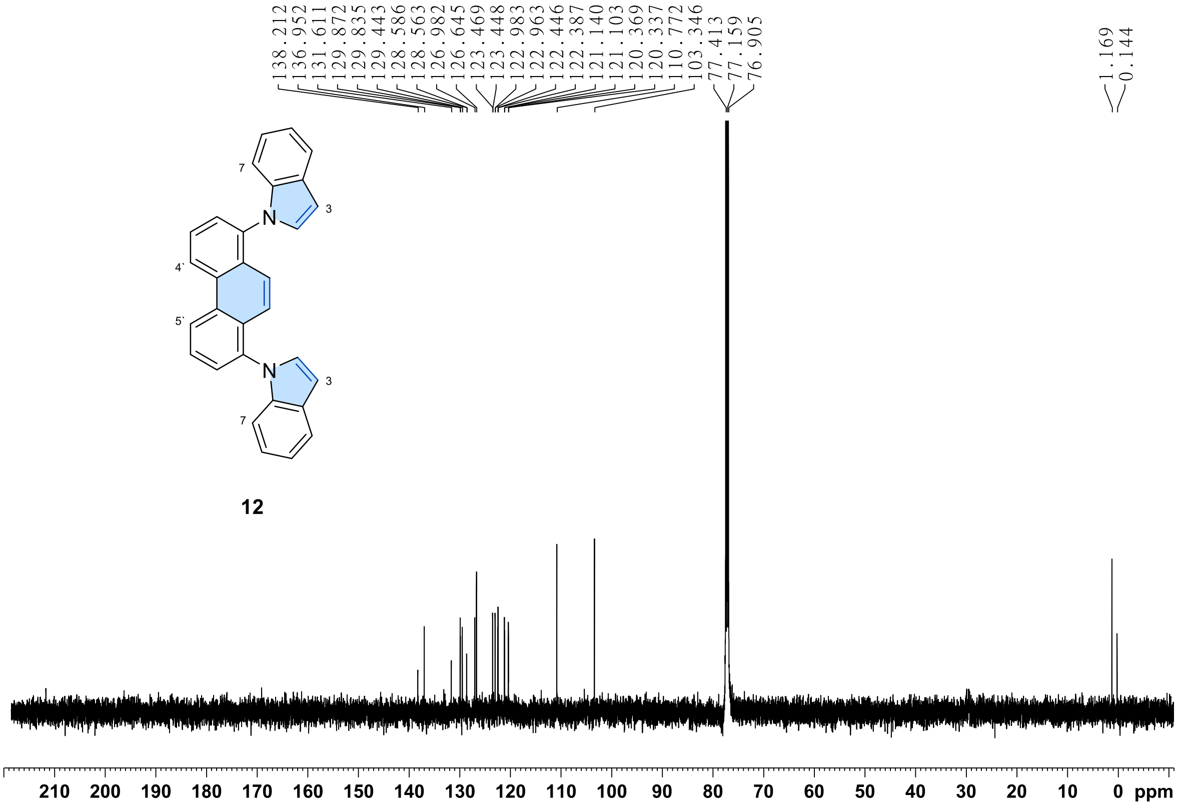

1-(Phenanthren-1-yl)-1*H*-indole (14a):

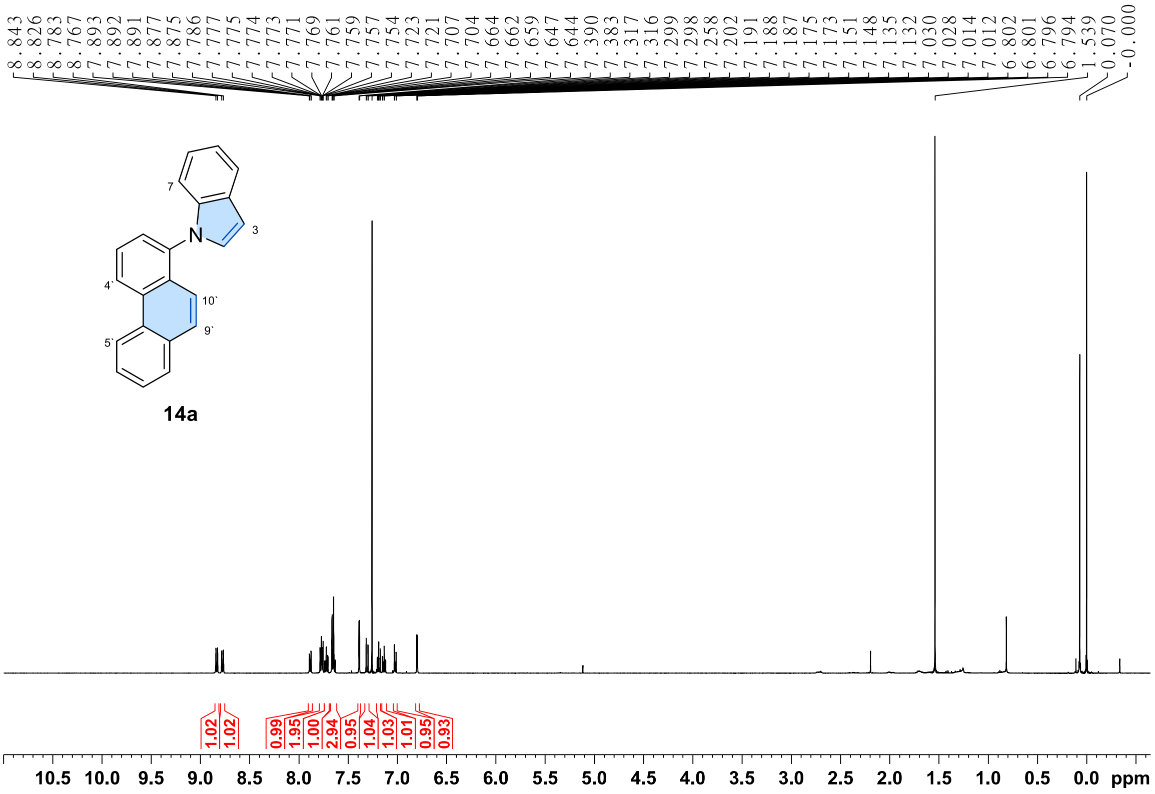

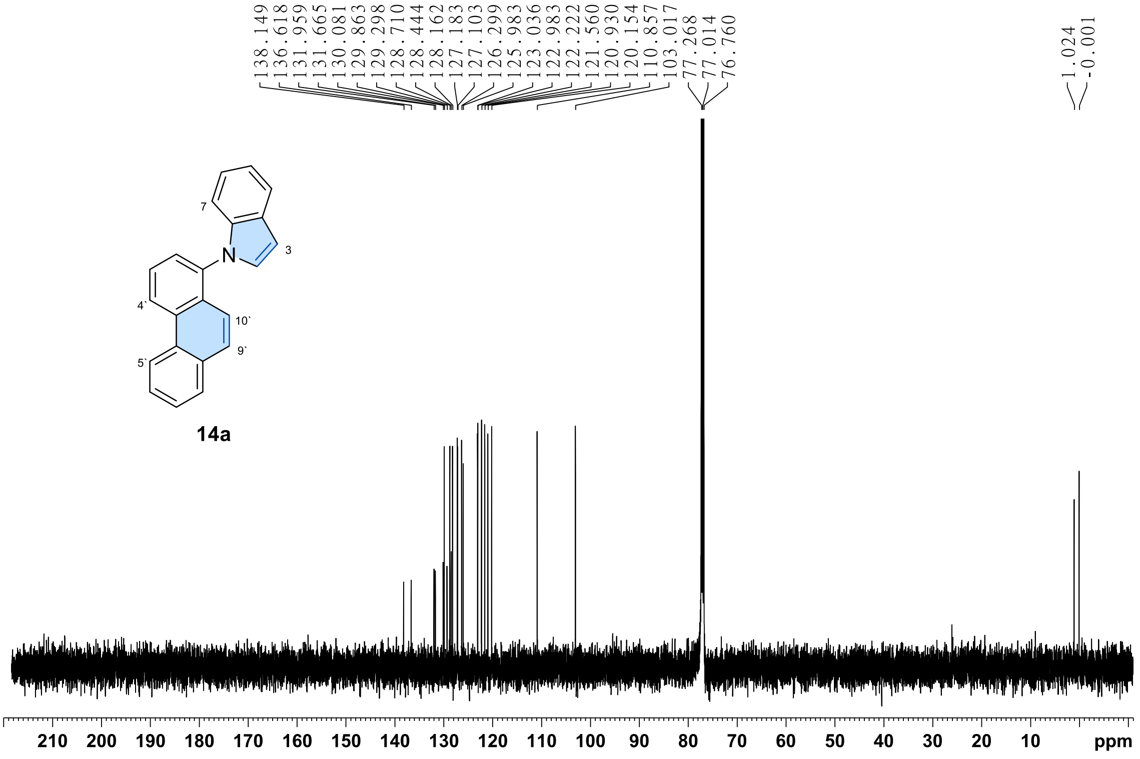

1-(6-Chlorophenanthren-1-yl)-1*H*-indole (14b):

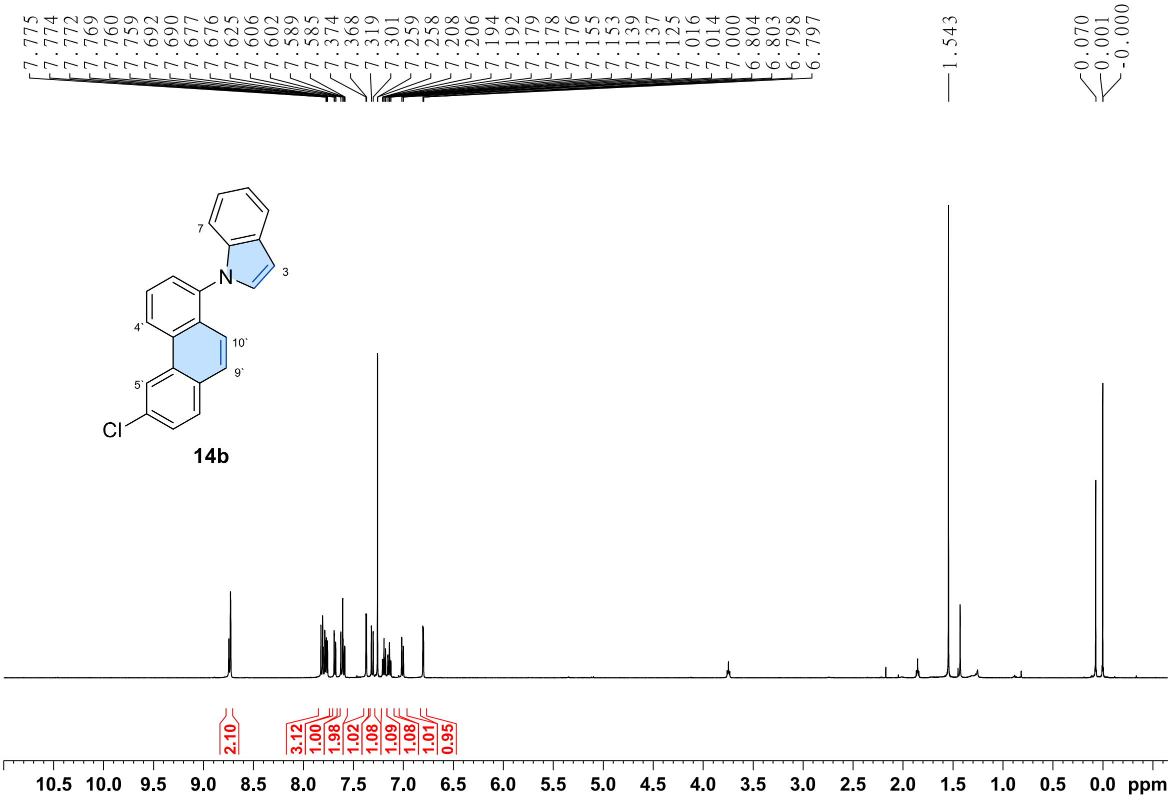

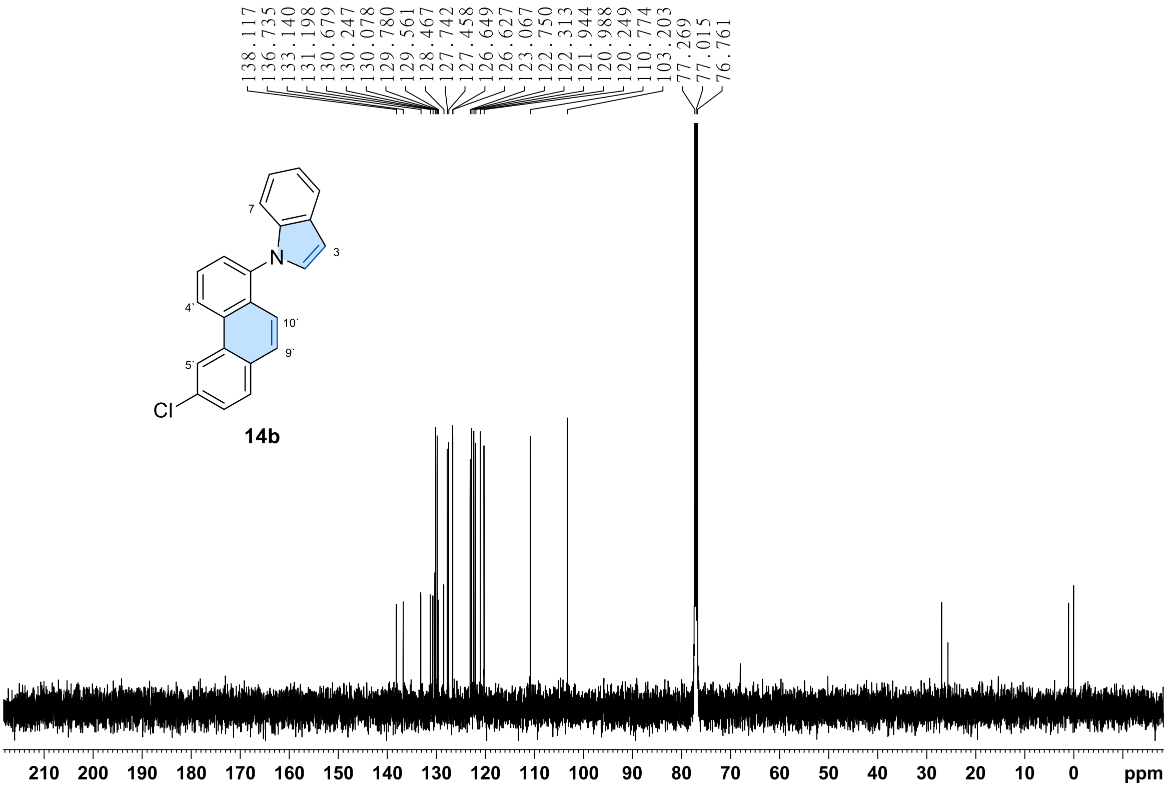

1-(6-Methoxyphenanthren-1-yl)-1*H*-indole (14c):

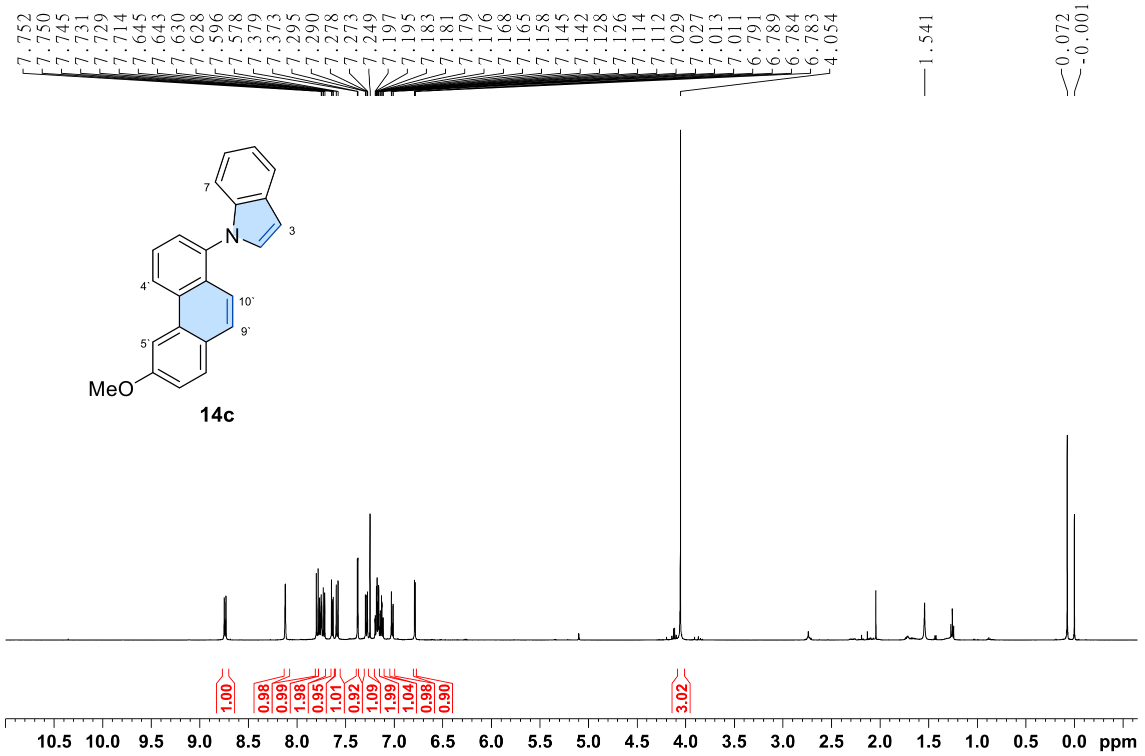

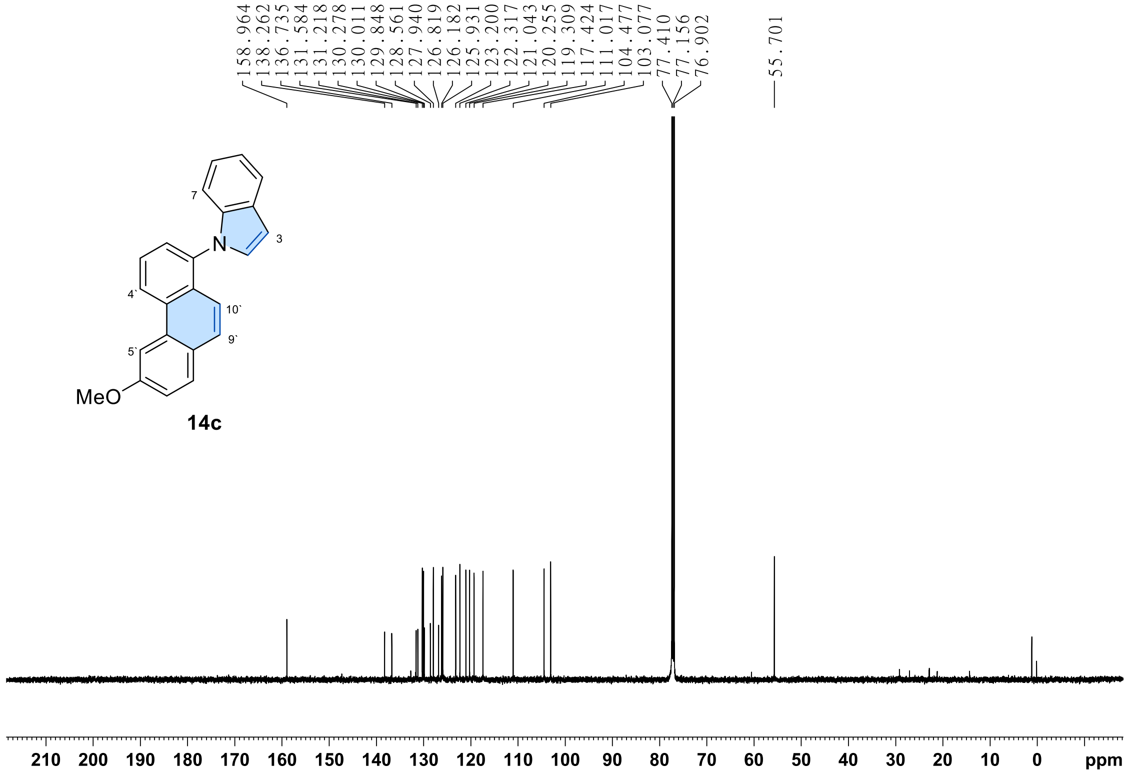

1-(3-Chlorophenanthren-1-yl)-1*H*-indole (14d):

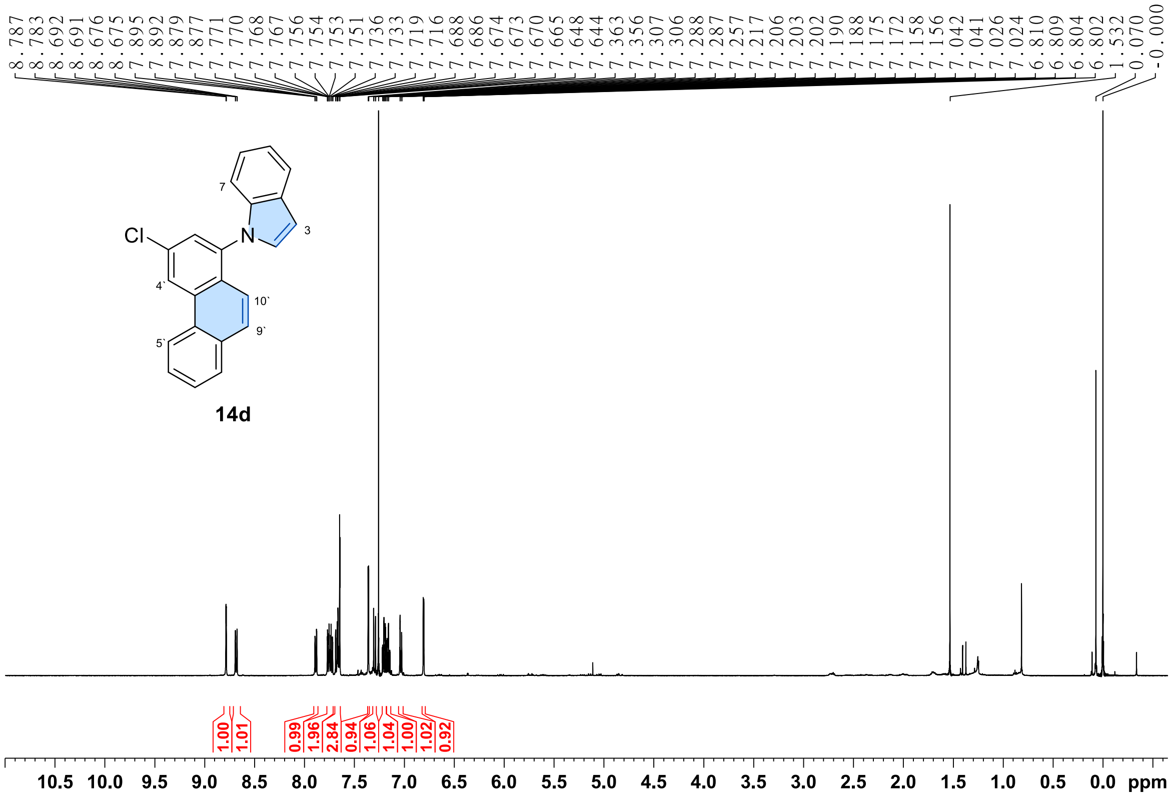

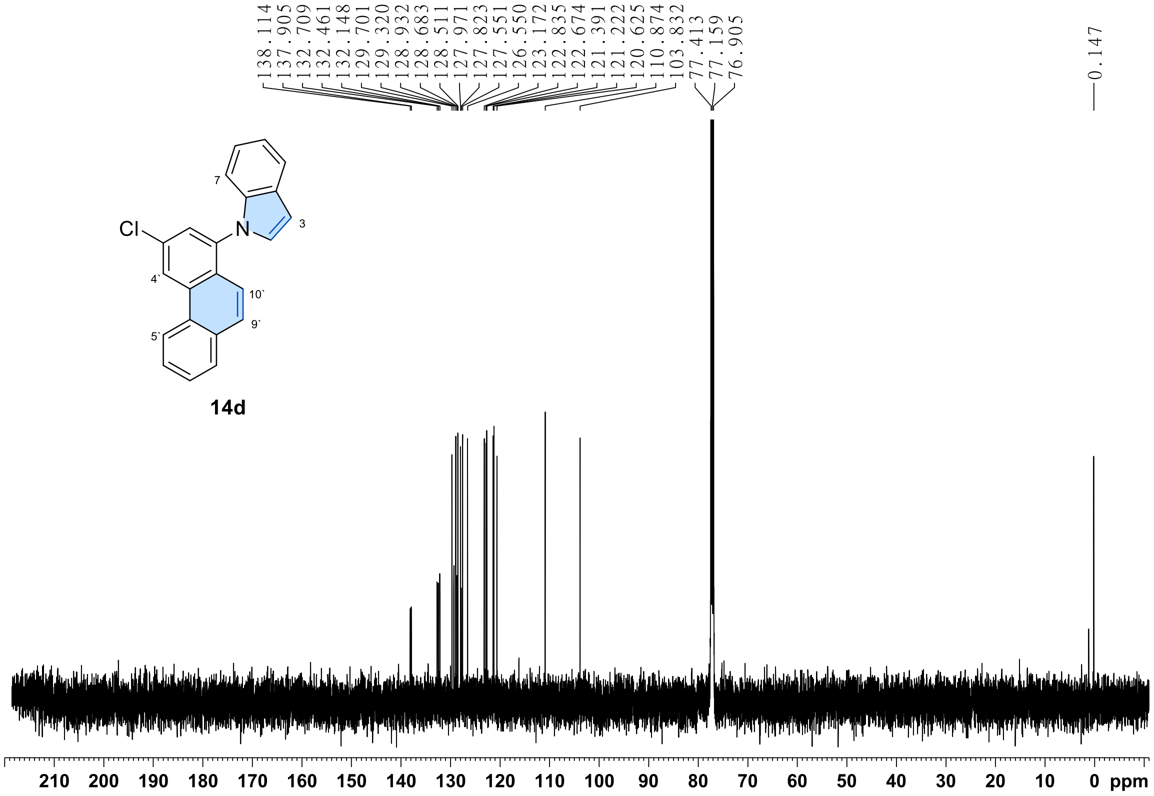

1-(3-(Trifluoromethyl)phenanthren-1-yl)-1*H*-indole (14e):

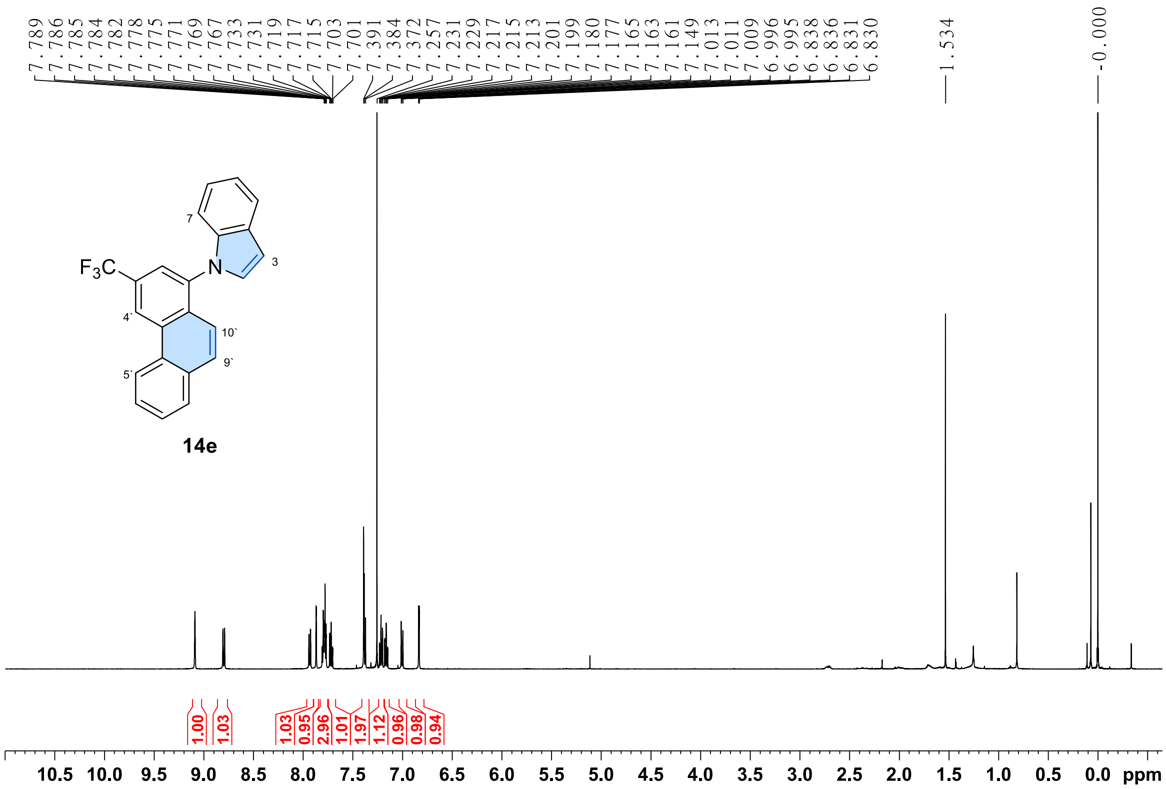

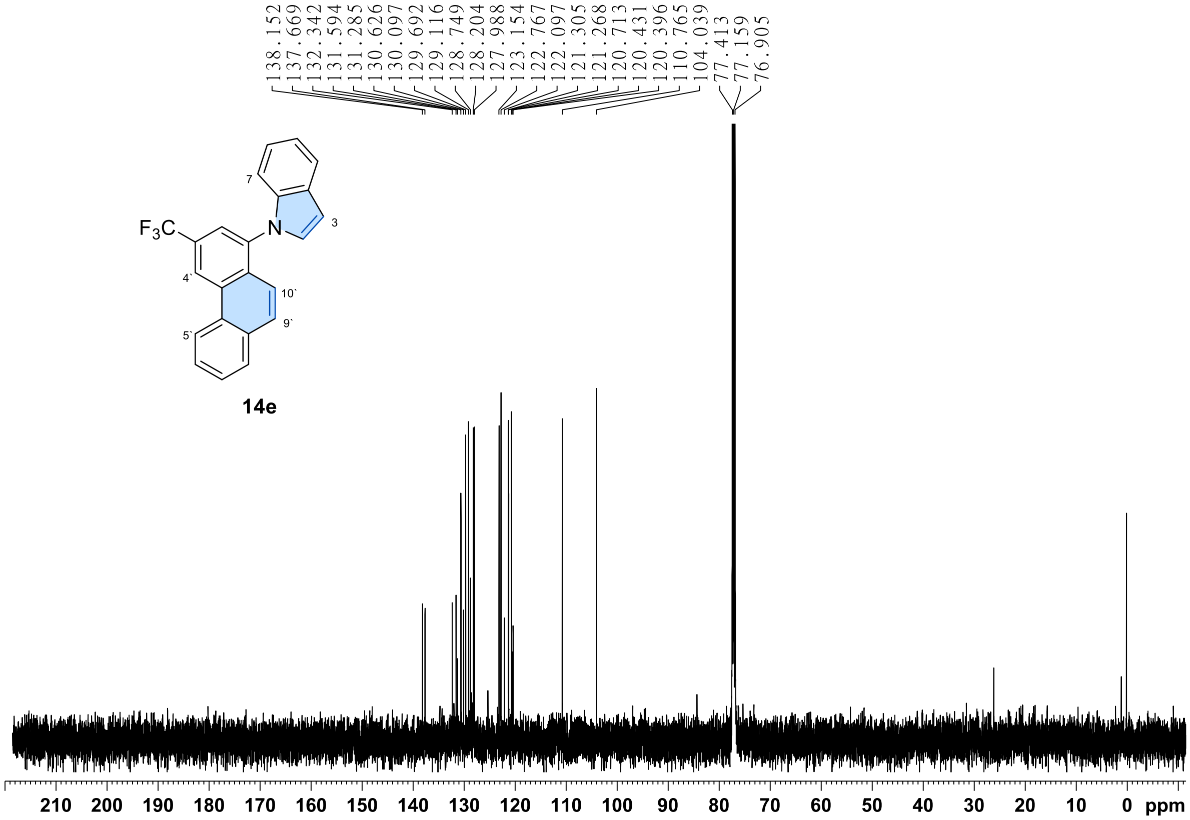

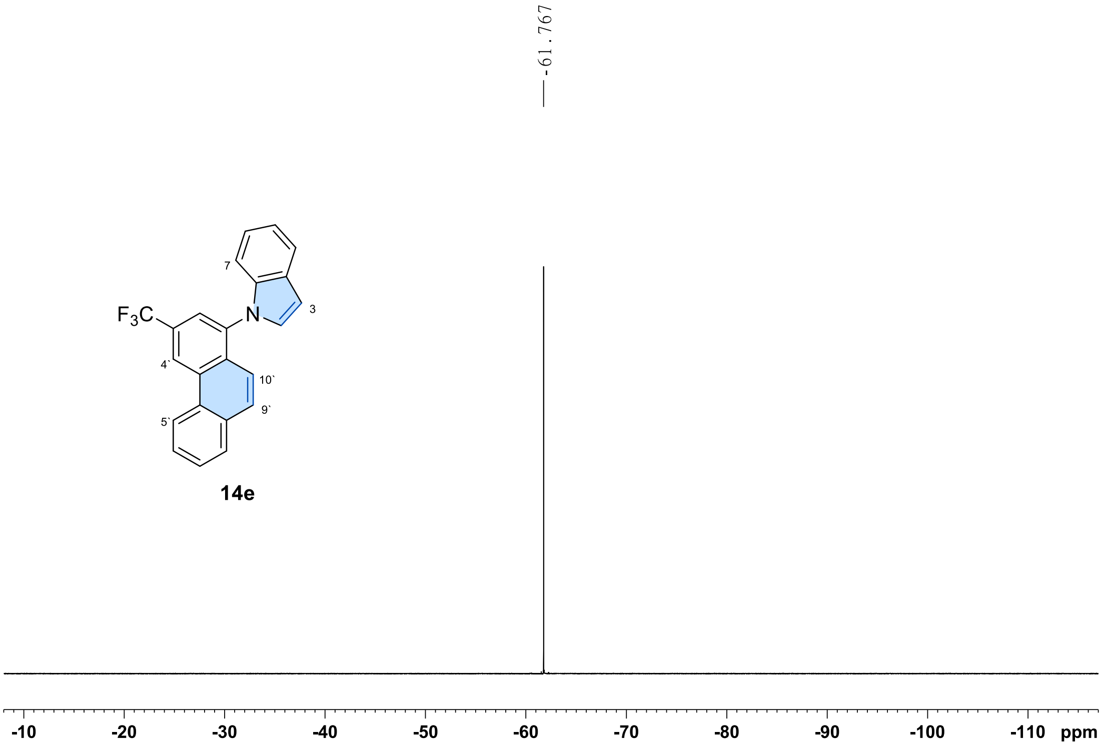

14e

1-(2-Fluorophenanthren-1-yl)-1*H*-indole (14f):

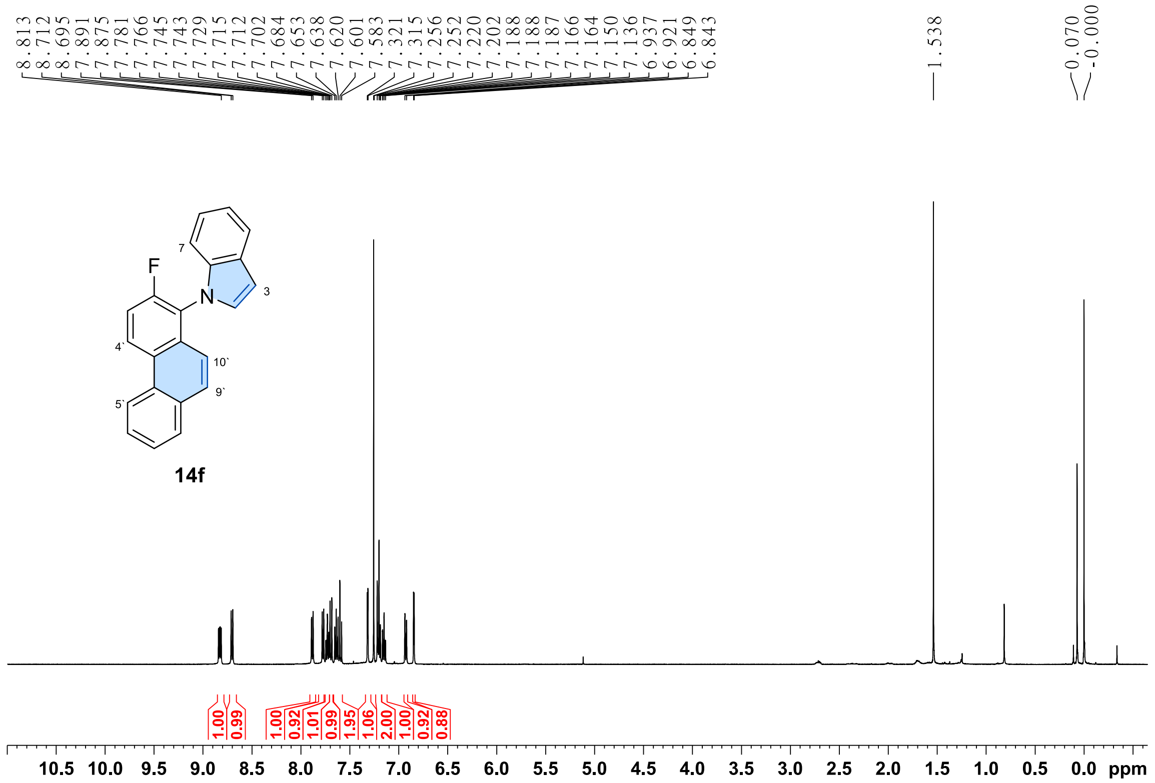

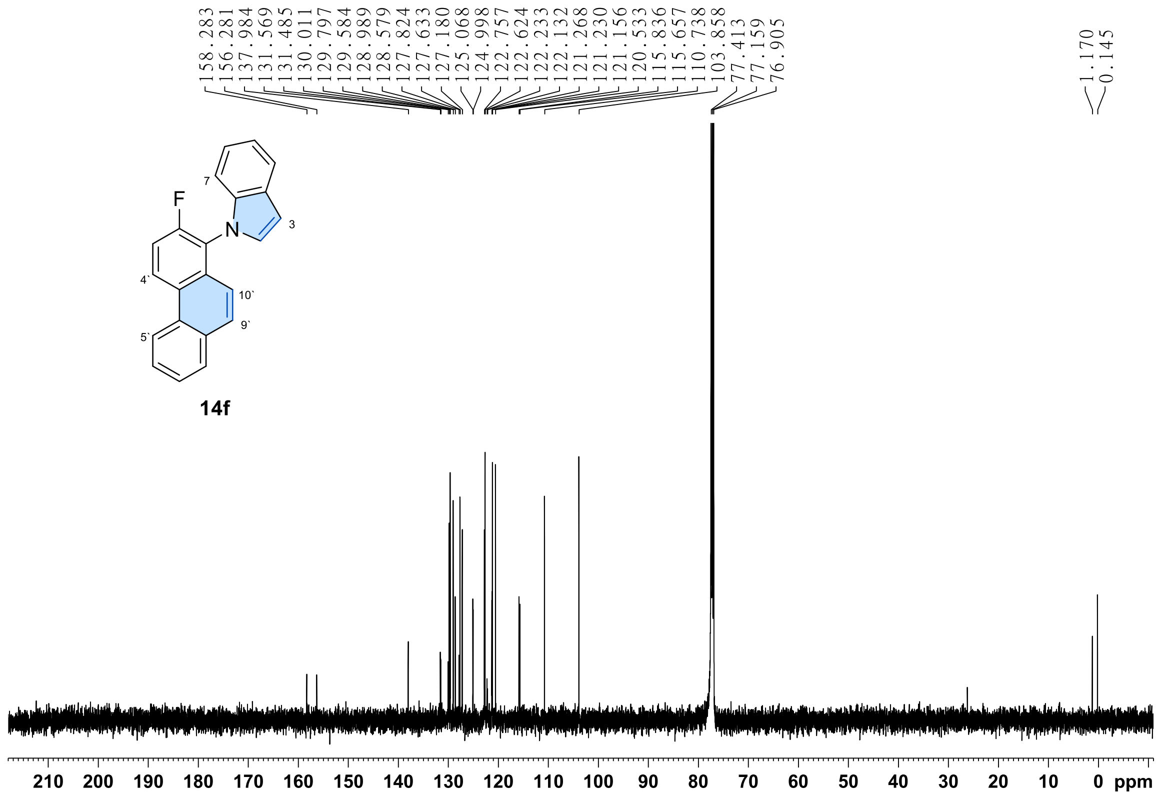

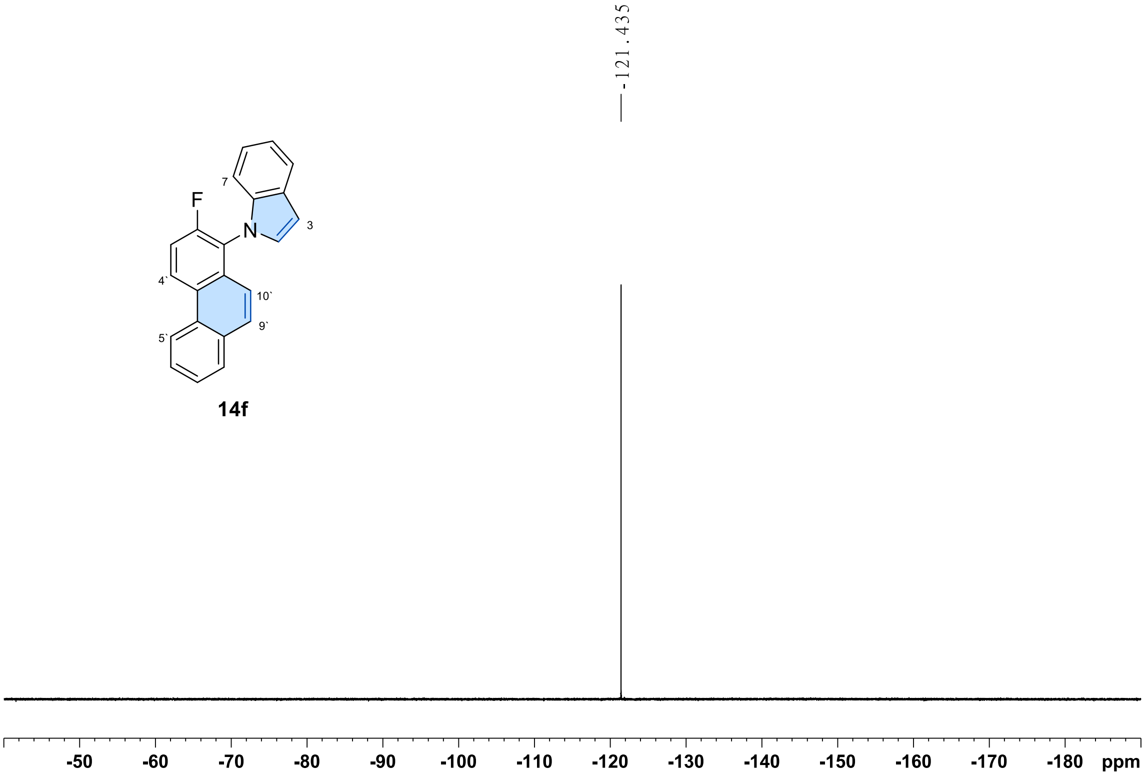
-121.435
F
N
7
3
4`
10`
9`
5`
14f
-50
-60
-70
-80
-90
-100
-110
-120
-130
-140
-150
-160
-170
-180
ppm

1-(10-Methylphenanthren-1-yl)-1*H*-indole (14g):

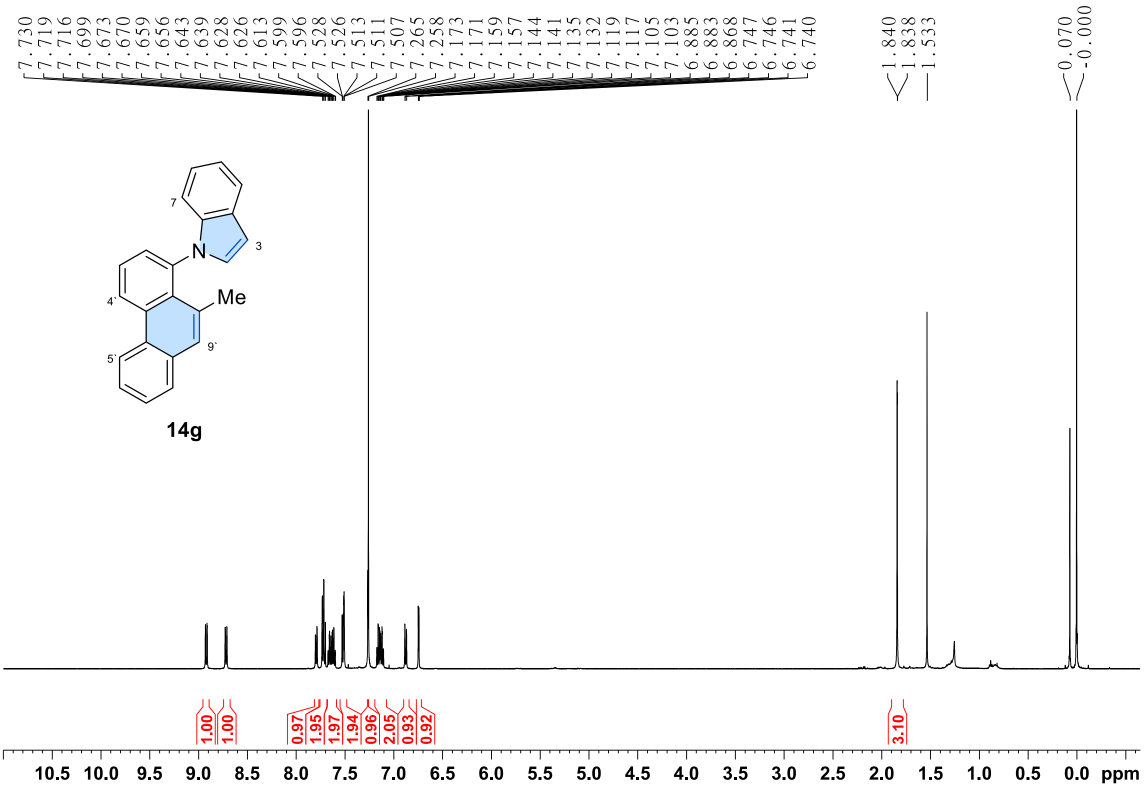

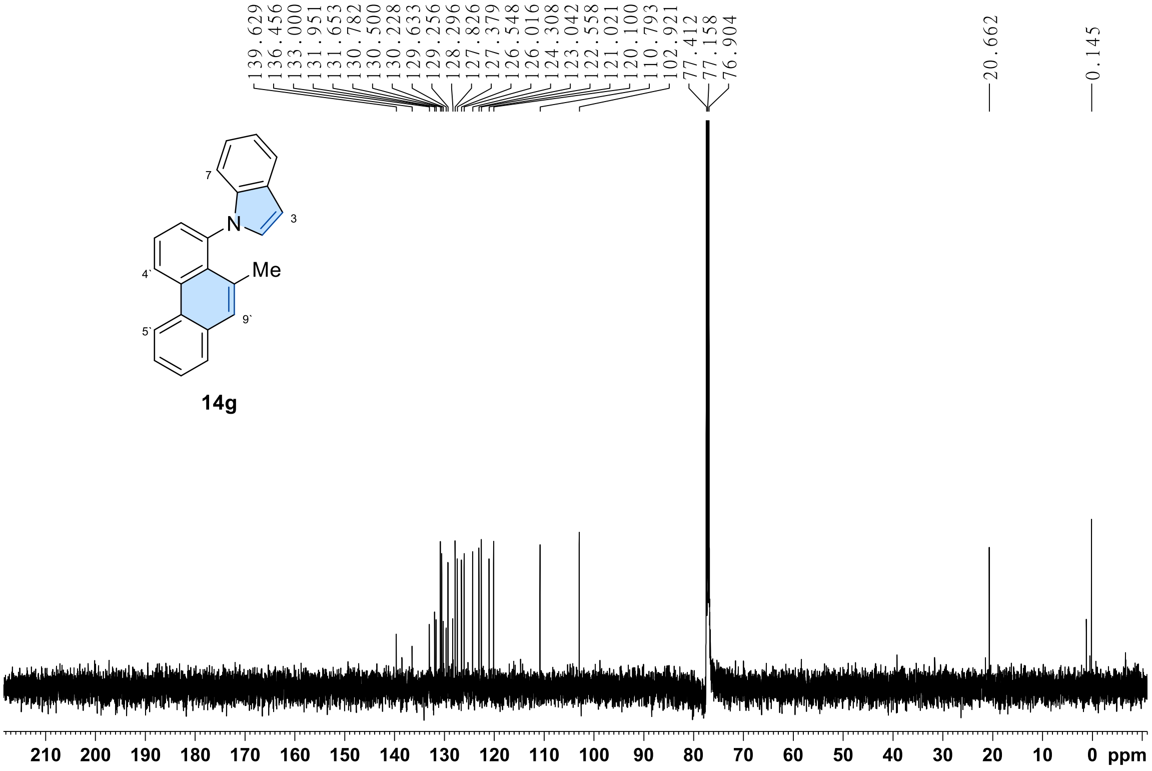

4-(1*H*-Indol-1-yl)benzo[h]isoquinoline (14h):

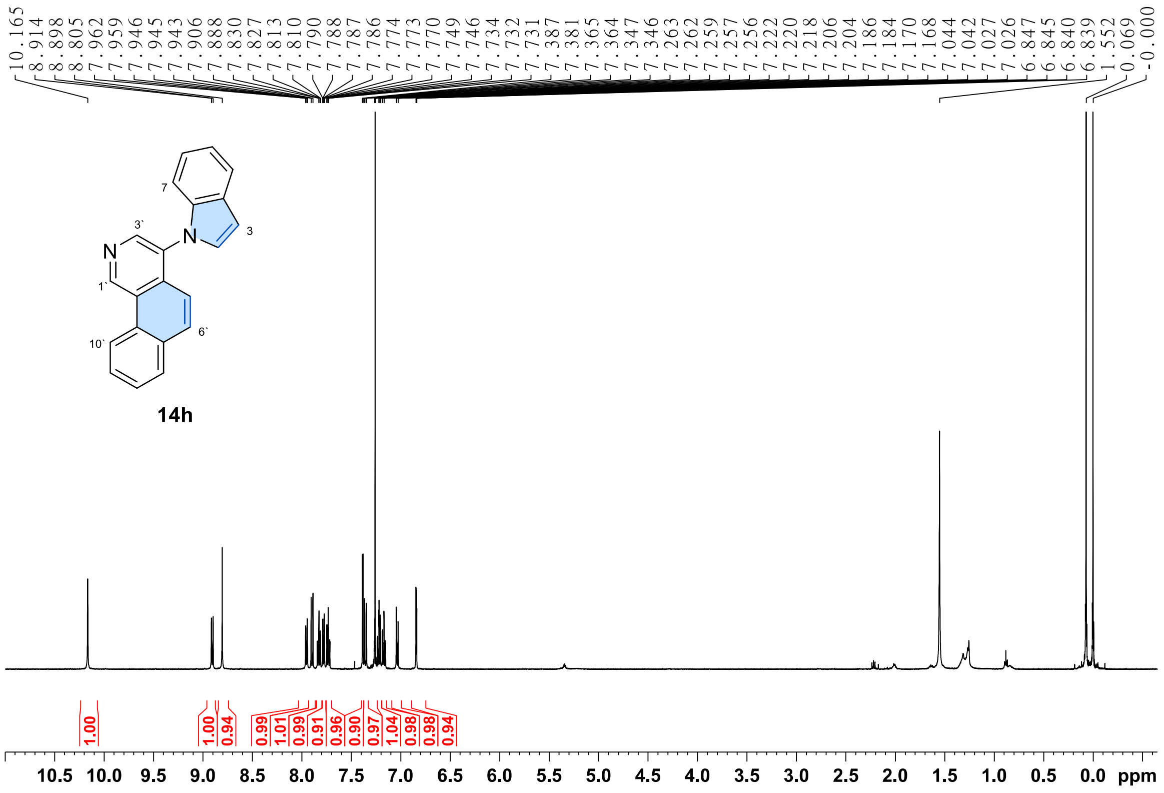

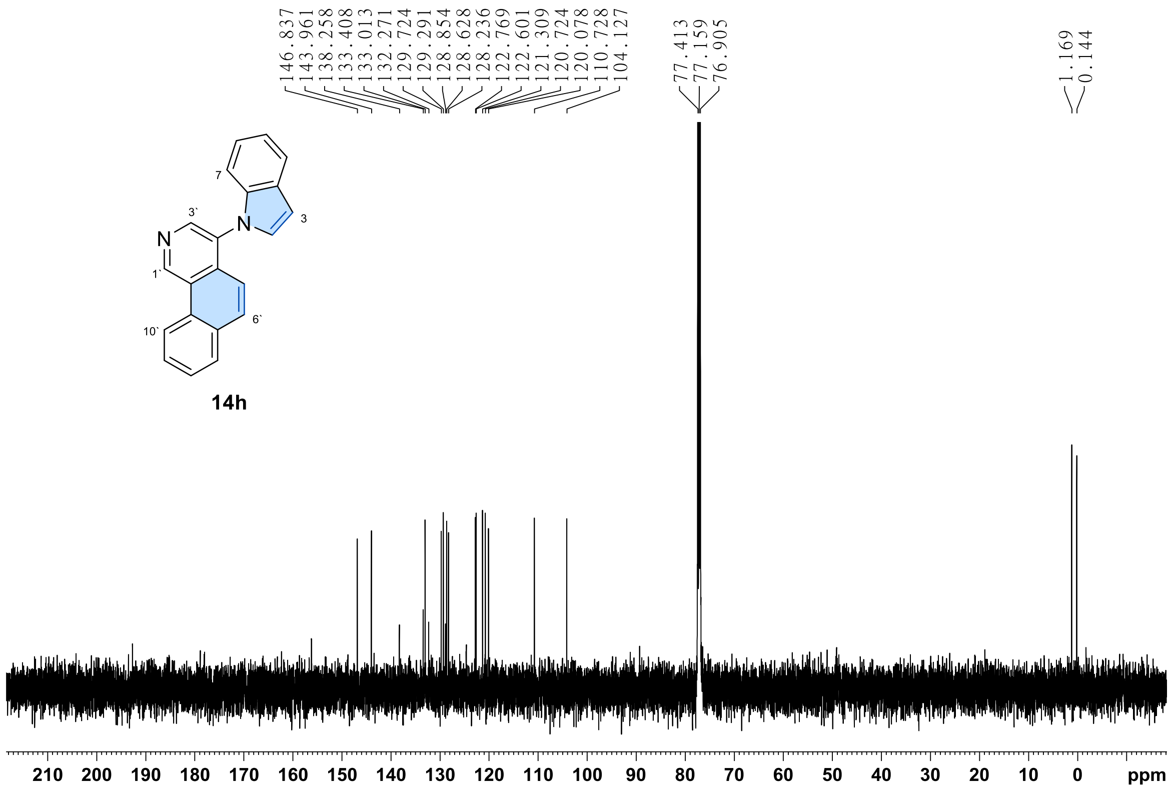

1-(Benzo[c]phenanthren-4-yl)-1*H*-indole (14i):

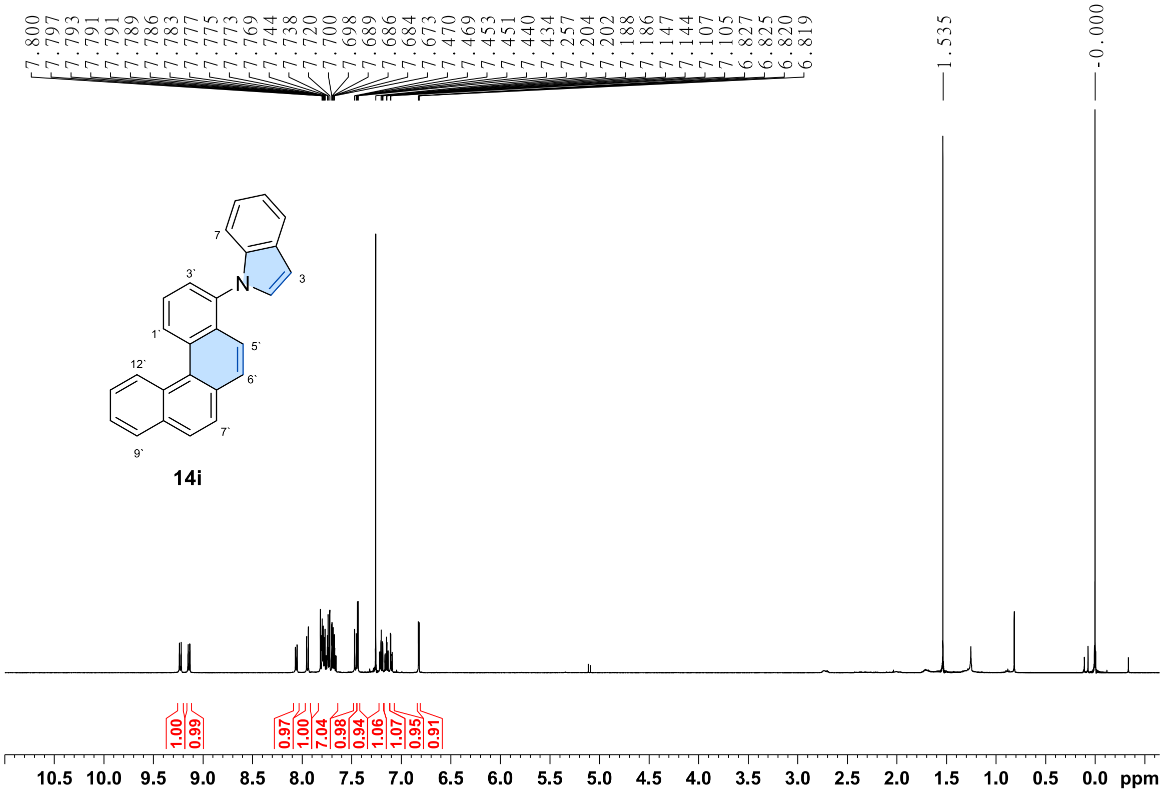

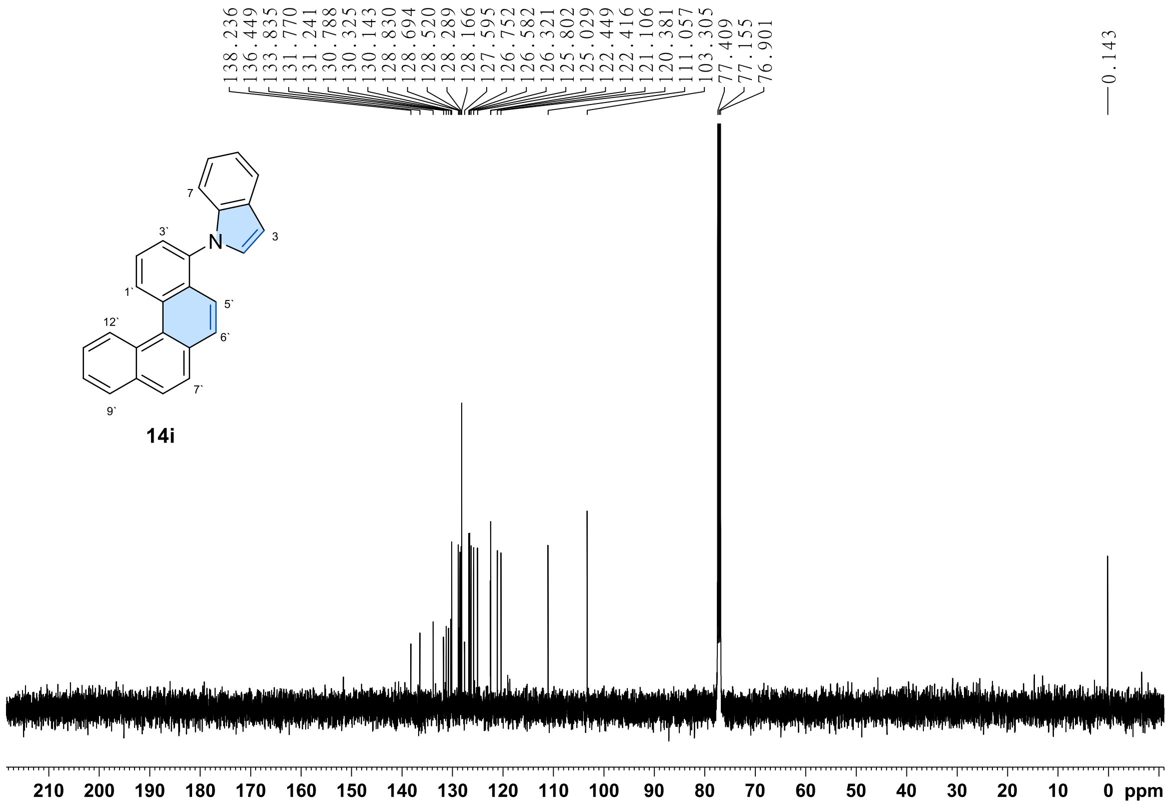

(R_a)-1-(2,4-Dimethoxy-6-vinylphenyl)-2-methylphenanthrene ((R_a)-16a):

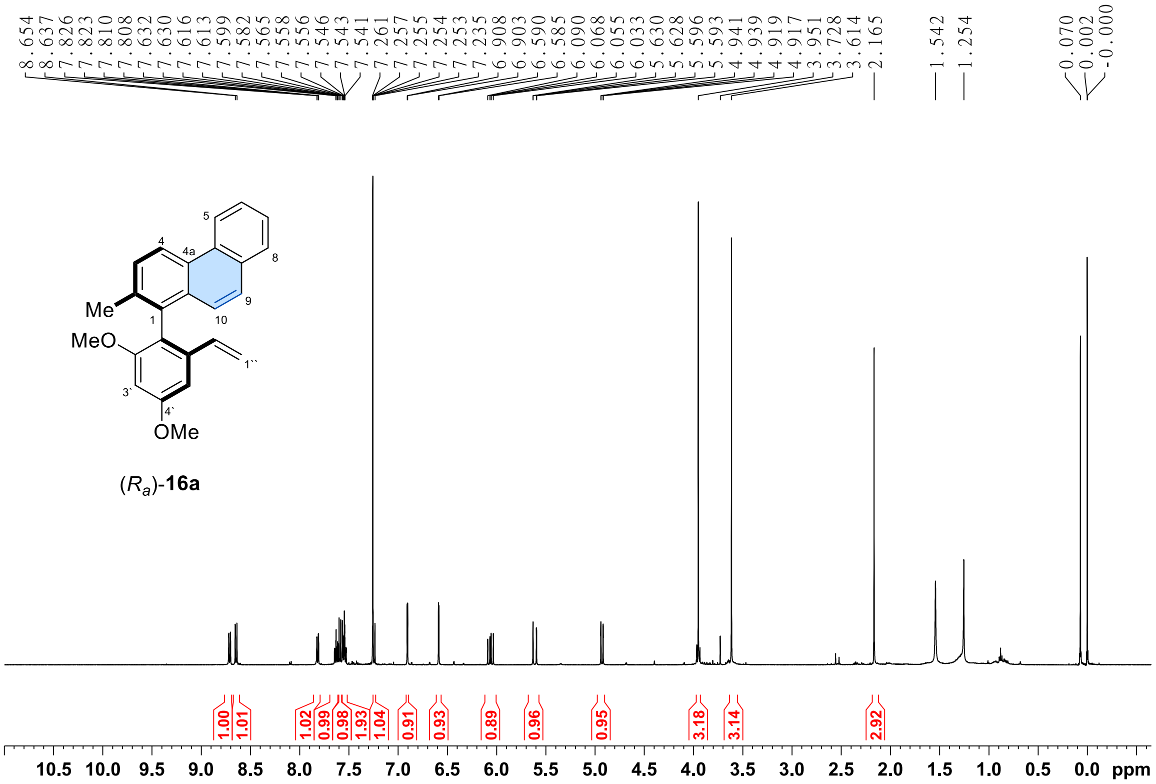

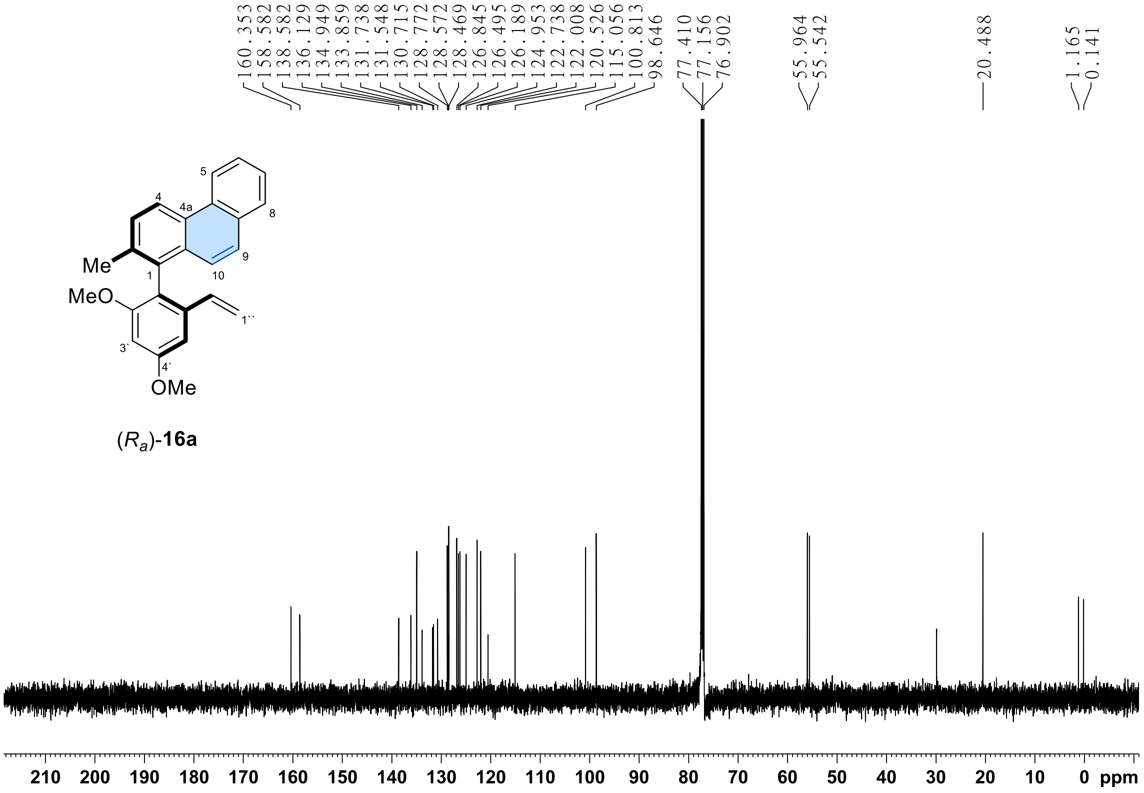

(*S*$_a$)-7-(2,4-Dimethyl-6-vinylphenyl)phenanthro[2,3-d][1,3]dioxole ((*S*$_a$)-16b):

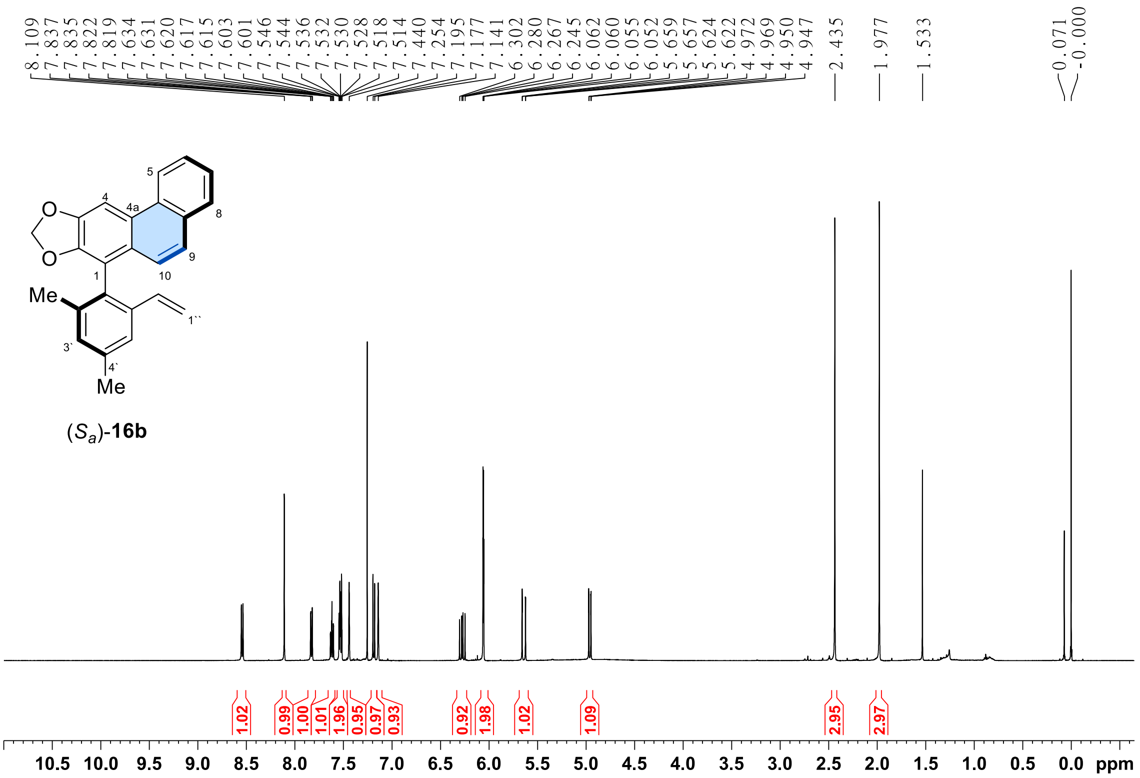

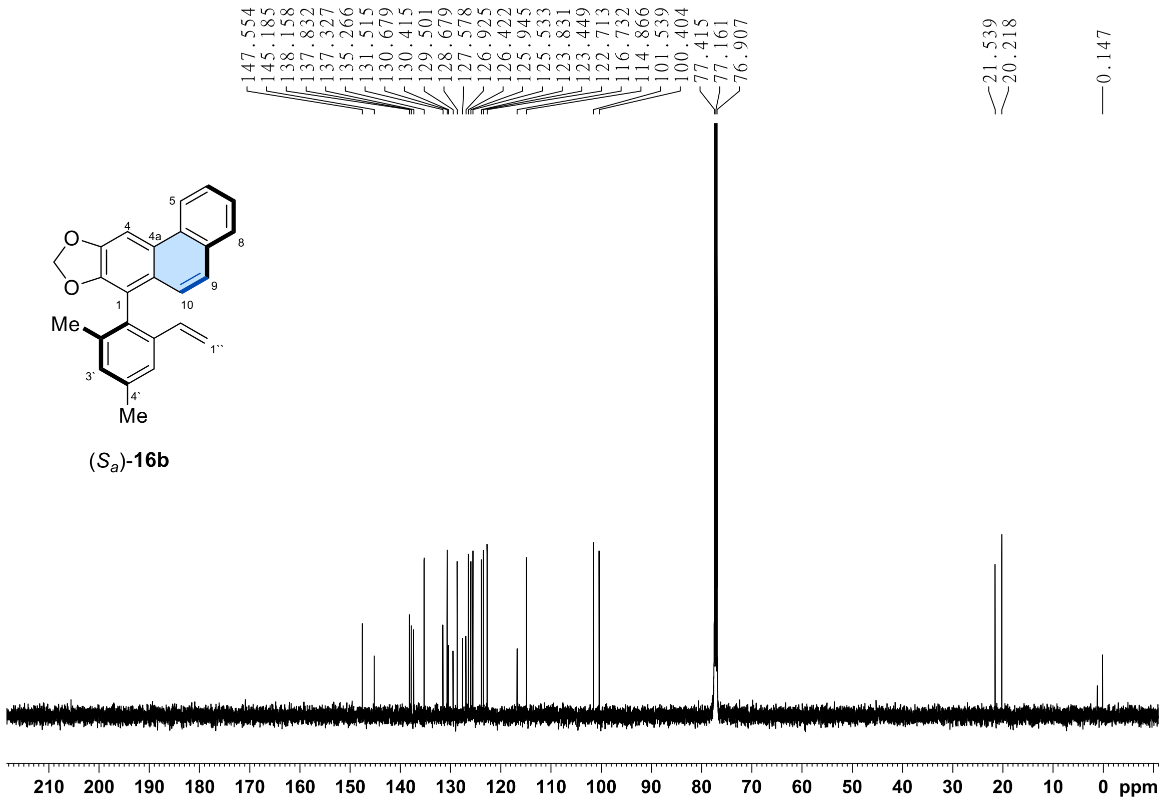

(R_a)-1-(2,4-Dimethoxy-6-vinylphenyl)phenanthrene ((R_a)-16c):

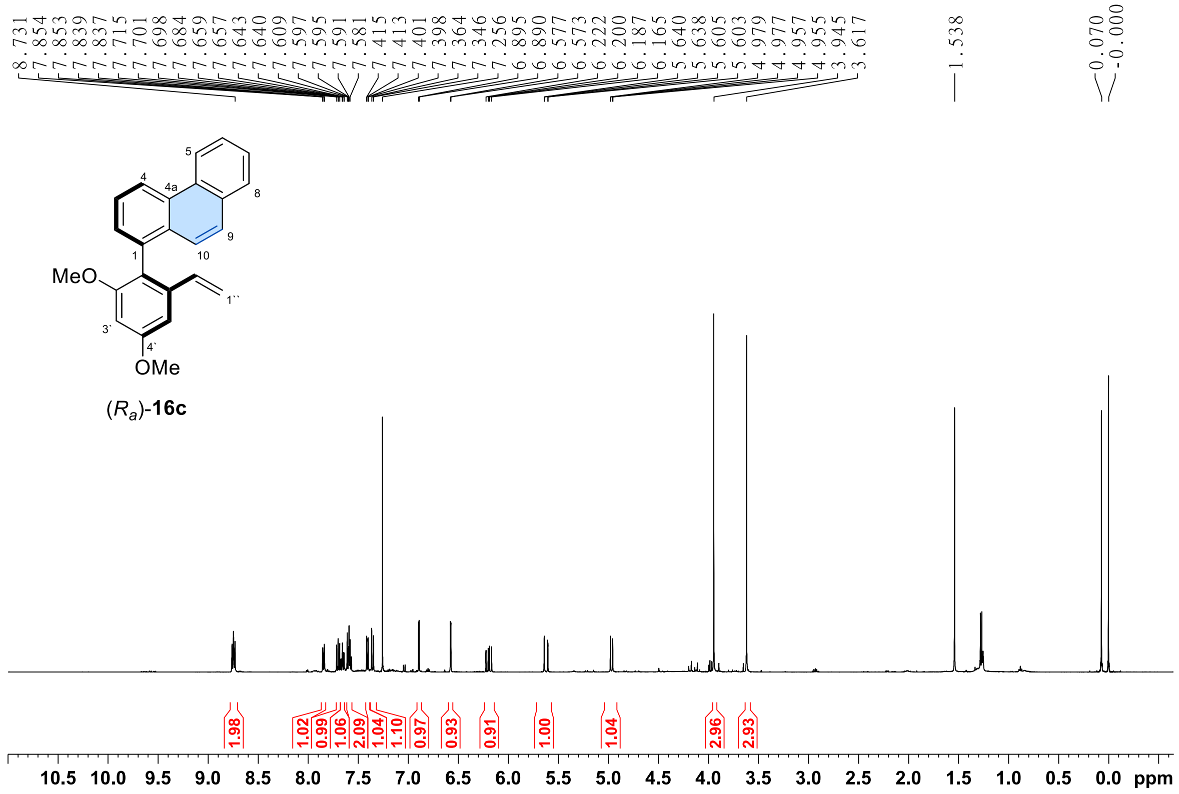

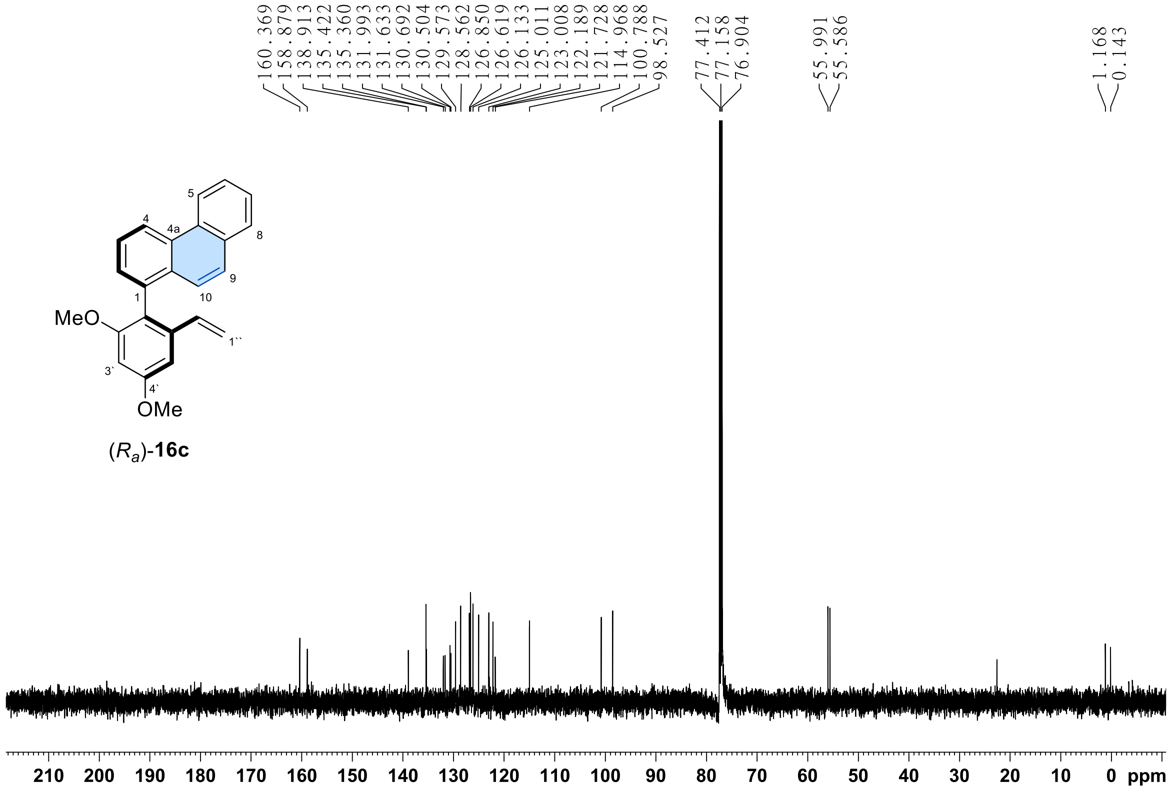

(S_a)-1-(2,4-Dimethoxy-6-vinylphenyl)-2-methoxyphenanthrene ((S_a)-16d):

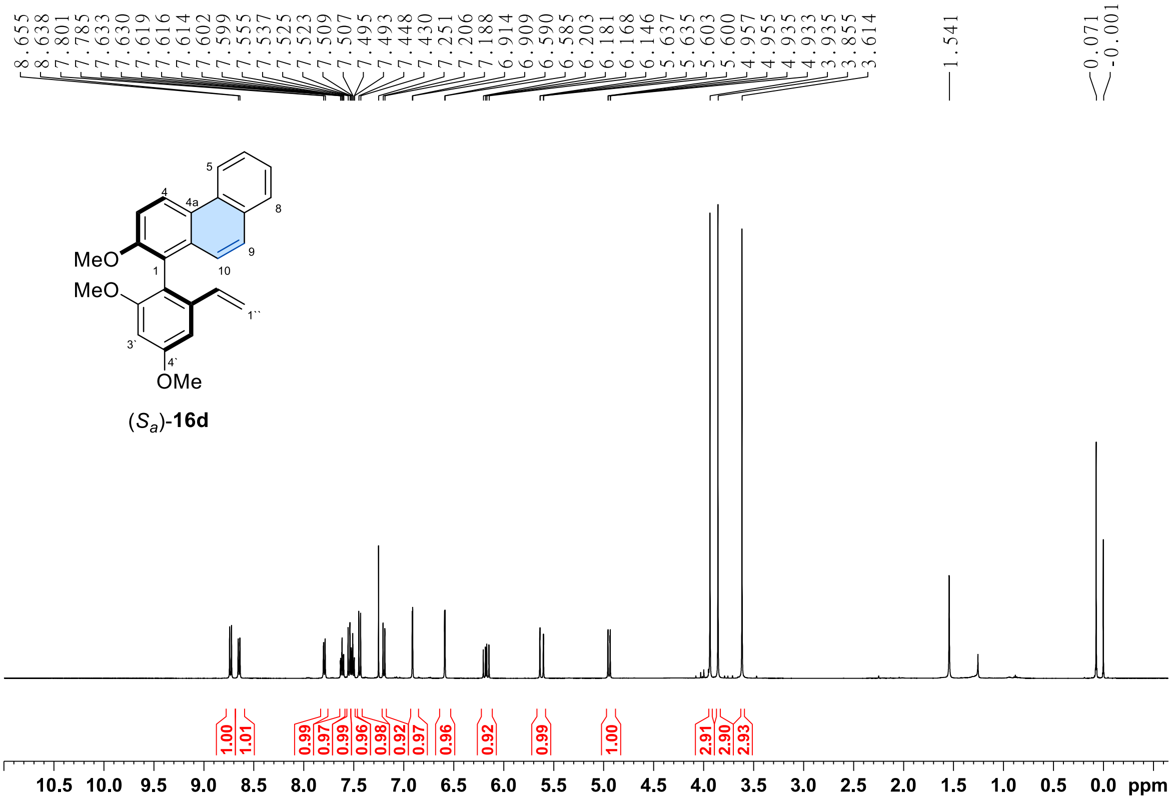

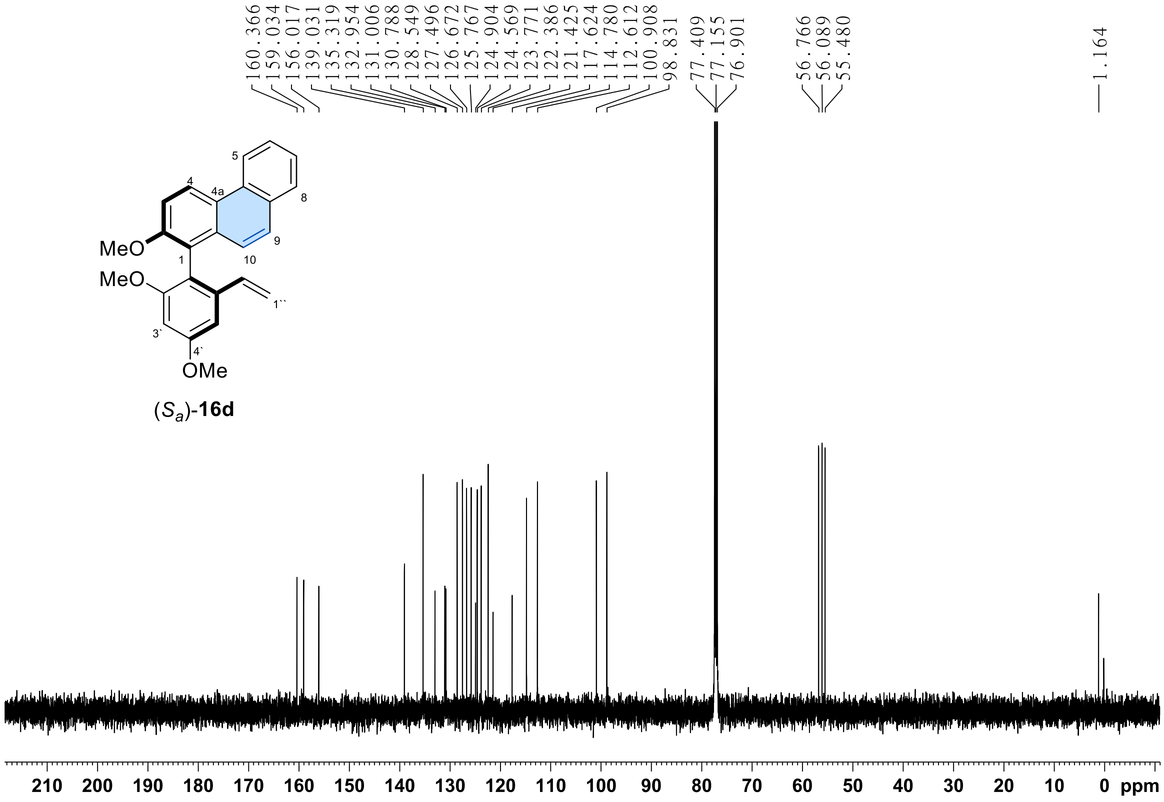

(R_a)-1-(6-Methoxy-3-methyl-2-vinylphenyl)-2-methylphenanthrene ((R_a)-16e):

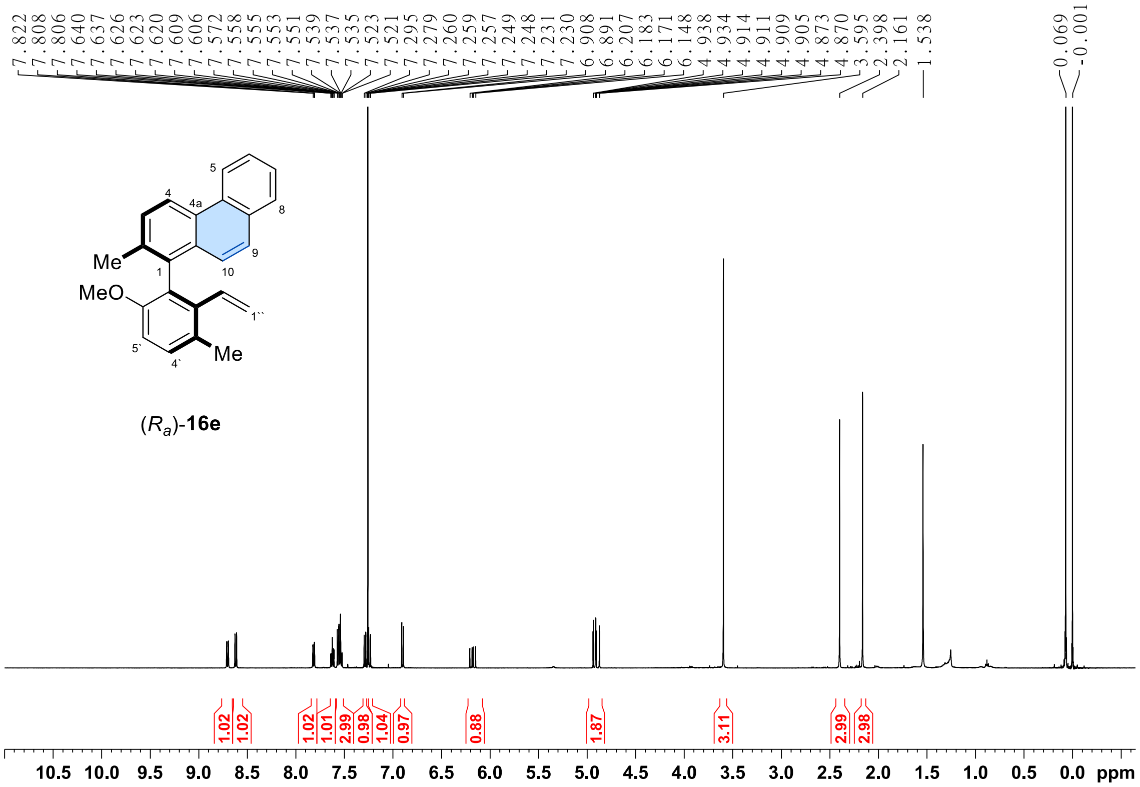

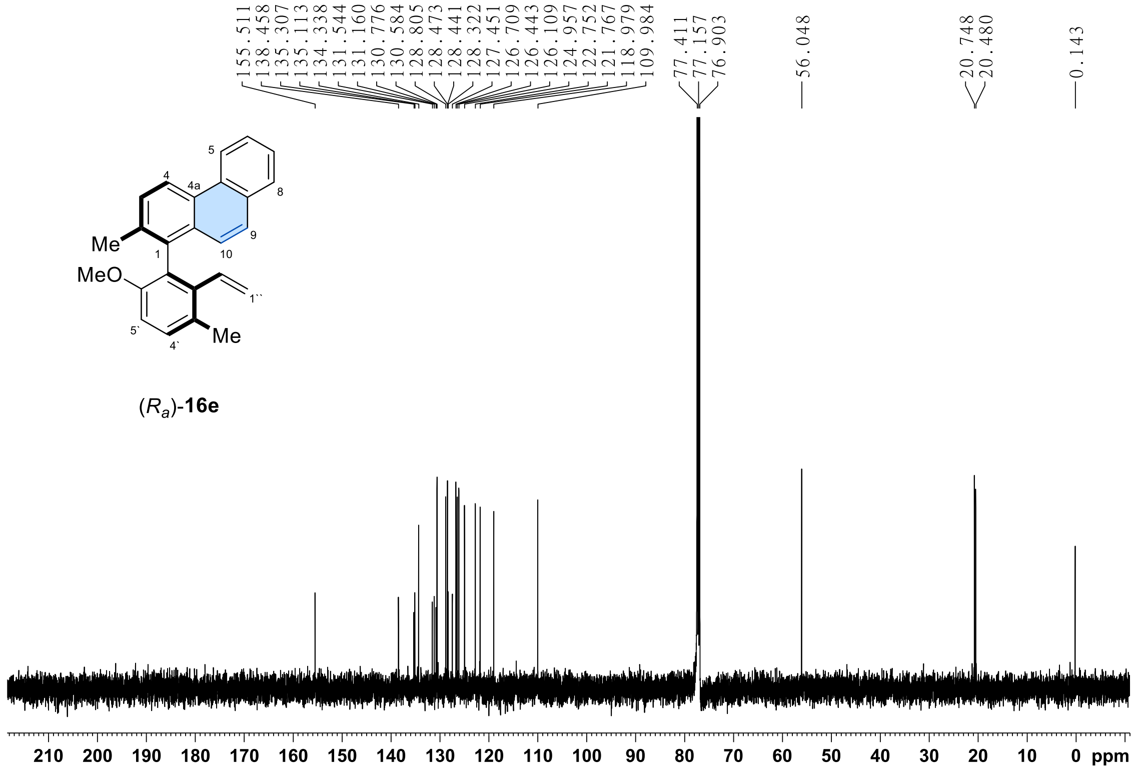

(R_a)-1-(3,6-Dimethyl-2-vinylphenyl)-2-methoxyphenanthrene ((R_a)-16f):

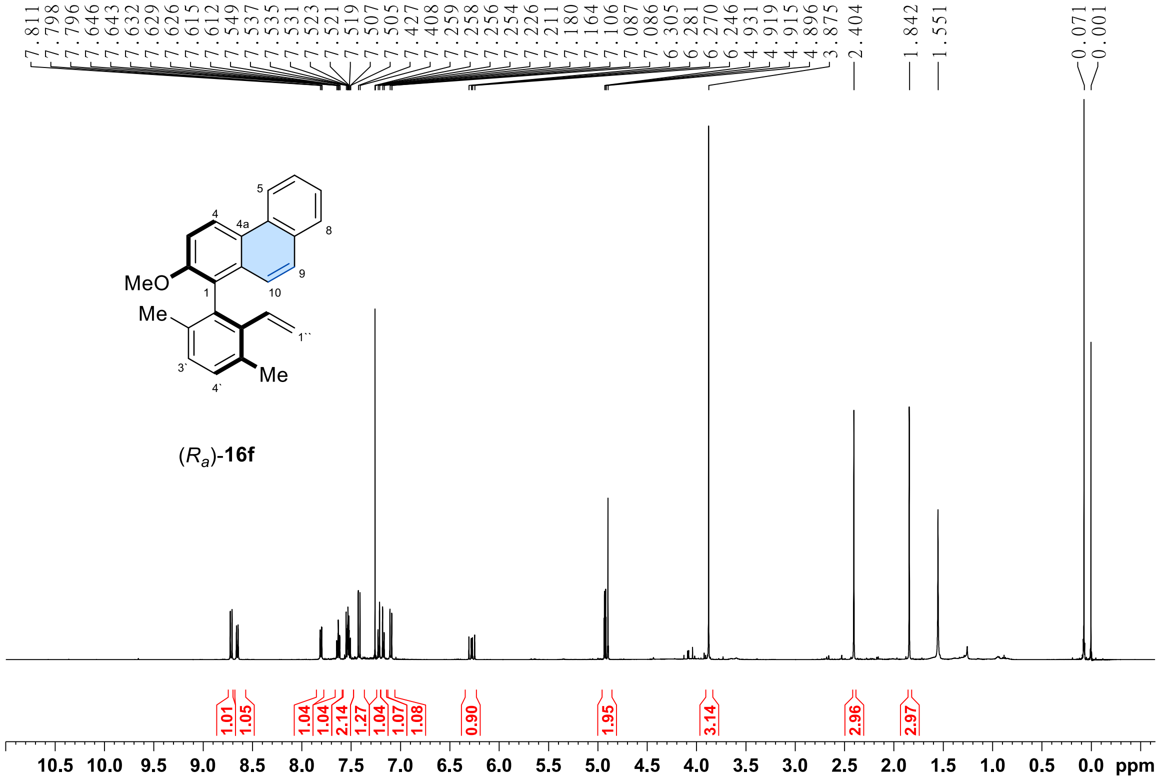

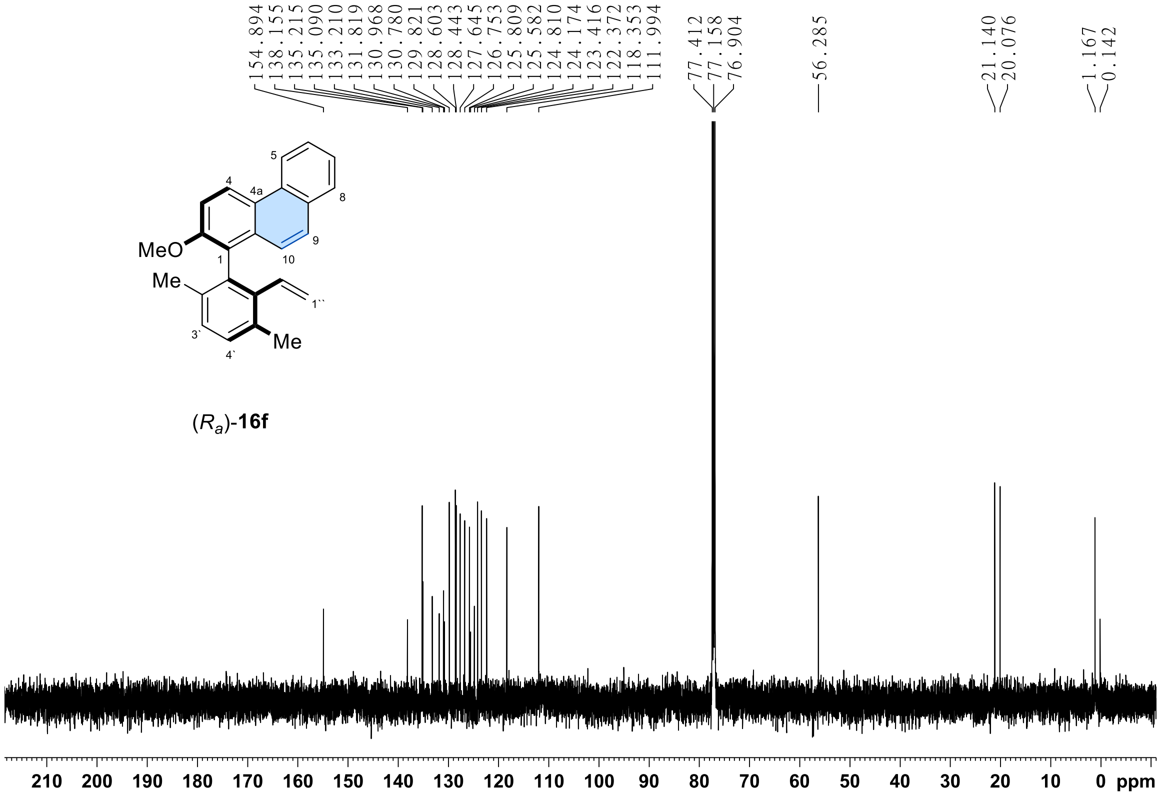

(R_a)-6-Chloro-1-(2,4-dimethoxy-6-vinylphenyl)-2-methylphenanthrene ((R_a)-16g):

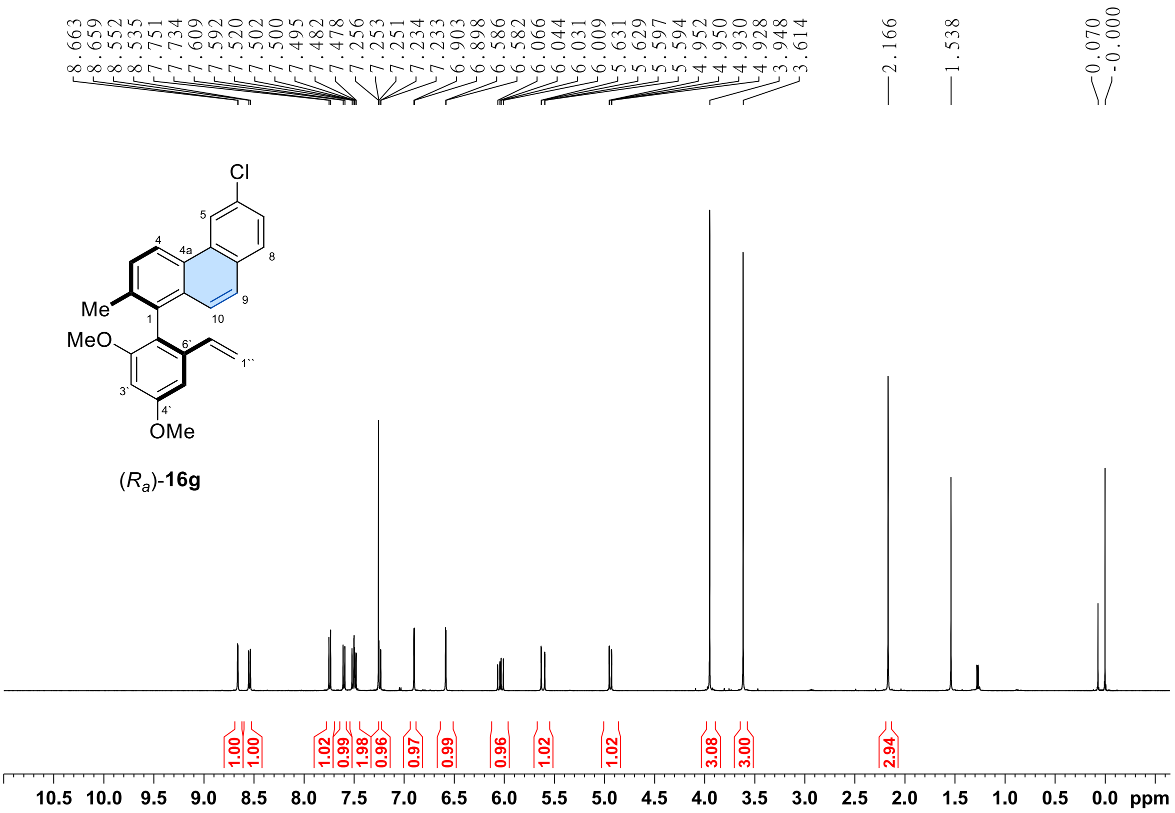

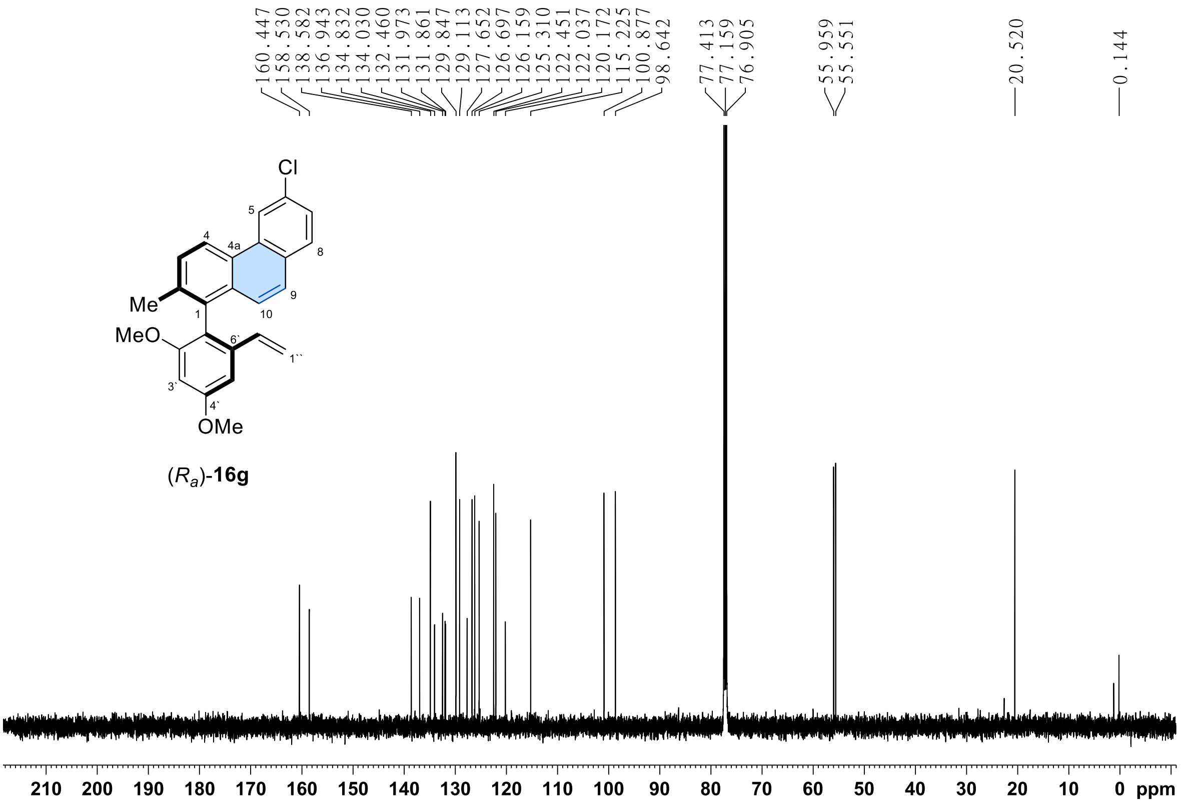

(S_a)-6-Chloro-2-methoxy-1-(2-methoxy-6-vinylphenyl)phenanthrene ((S_a)-16h):

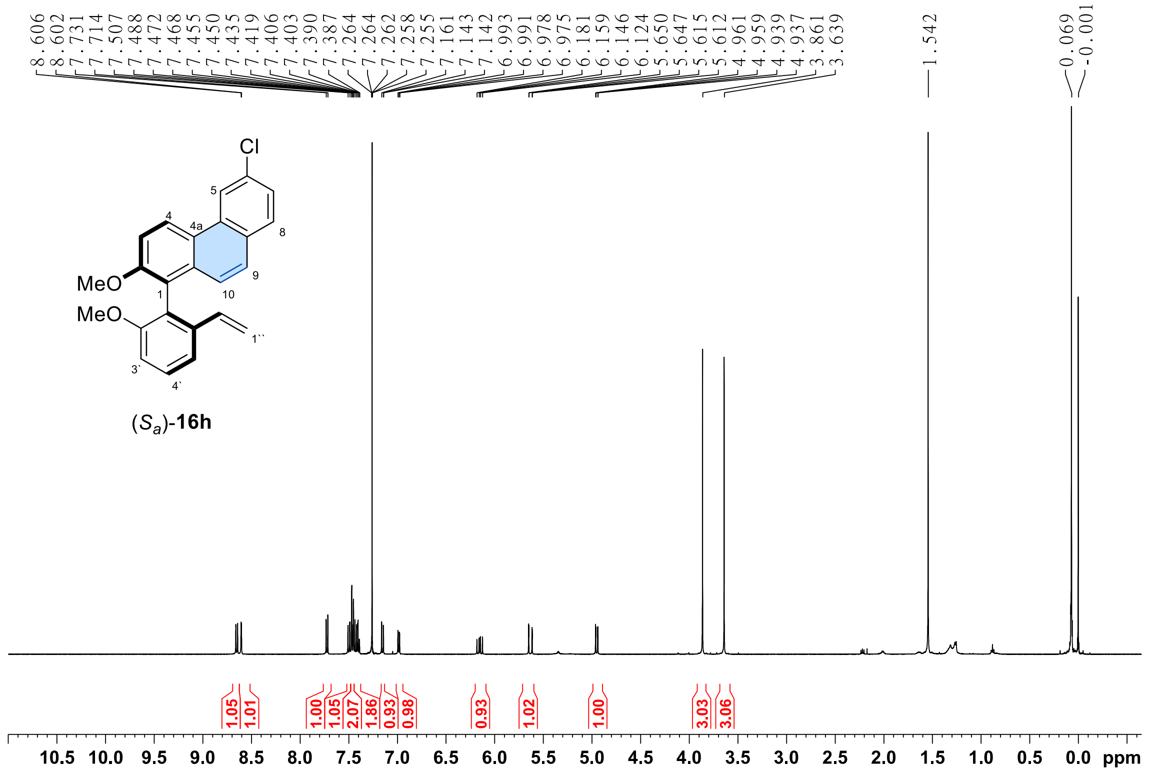

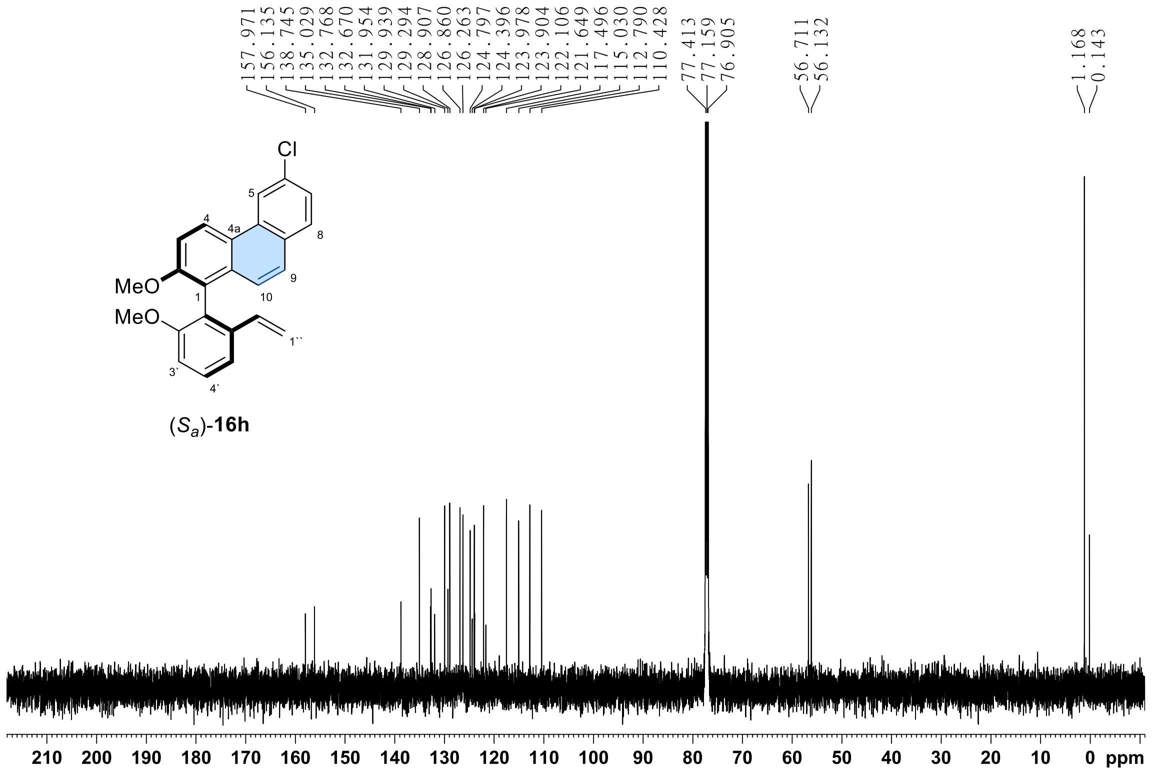

(S_a)-1-(2,4-Dimethyl-6-vinylphenyl)-2-methylphenanthrene ((S_a)-16i):

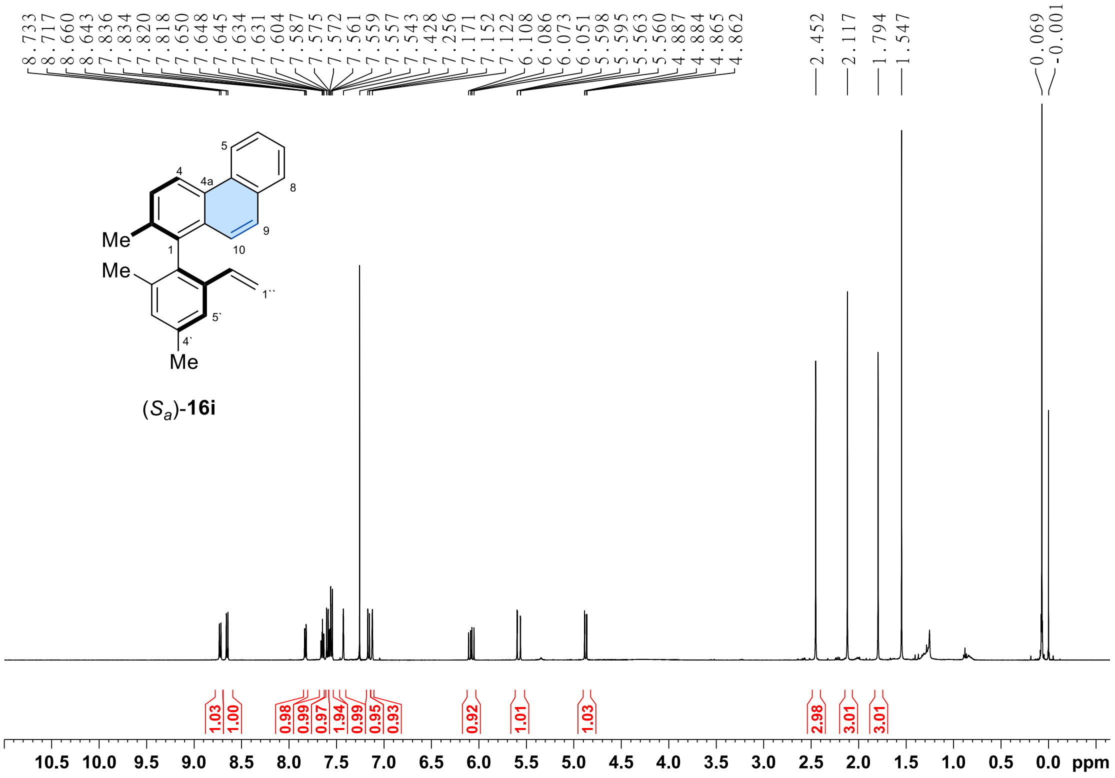

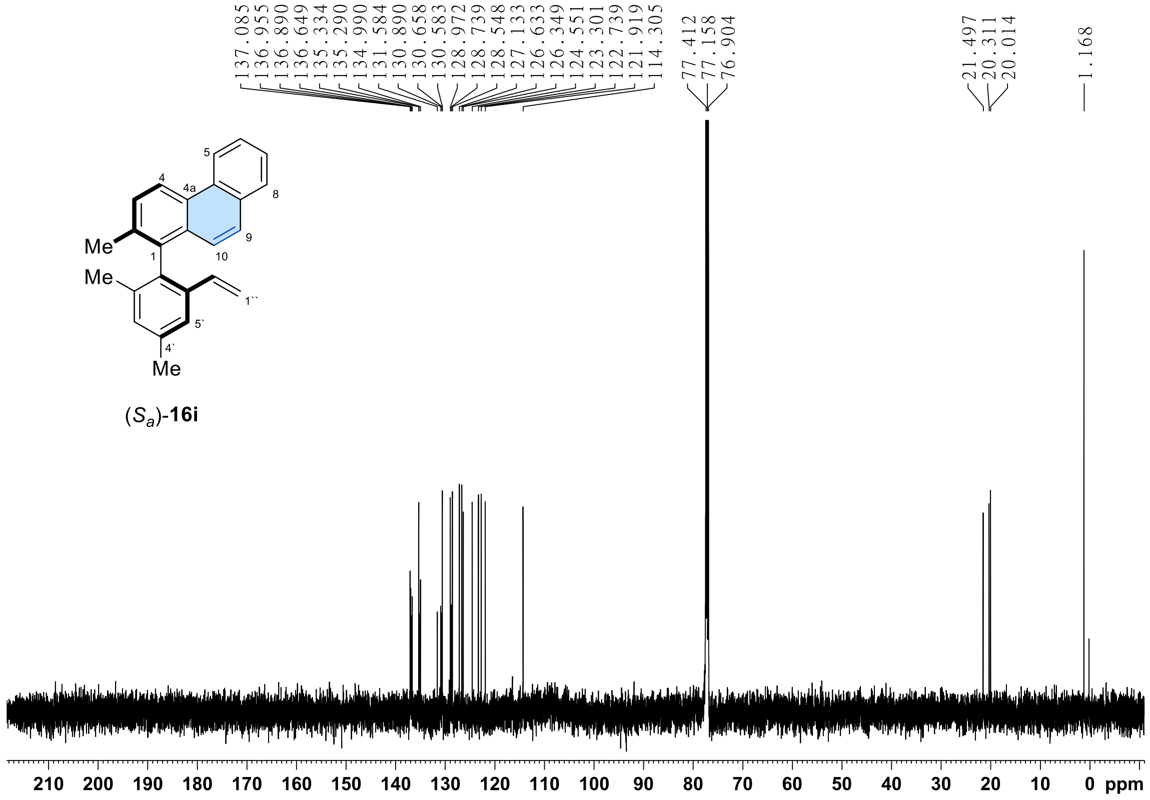

(S_a)-1-(3-Bromo-6-methoxy-2-vinylphenyl)-2-methoxyphenanthrene ((S_a)-16j):

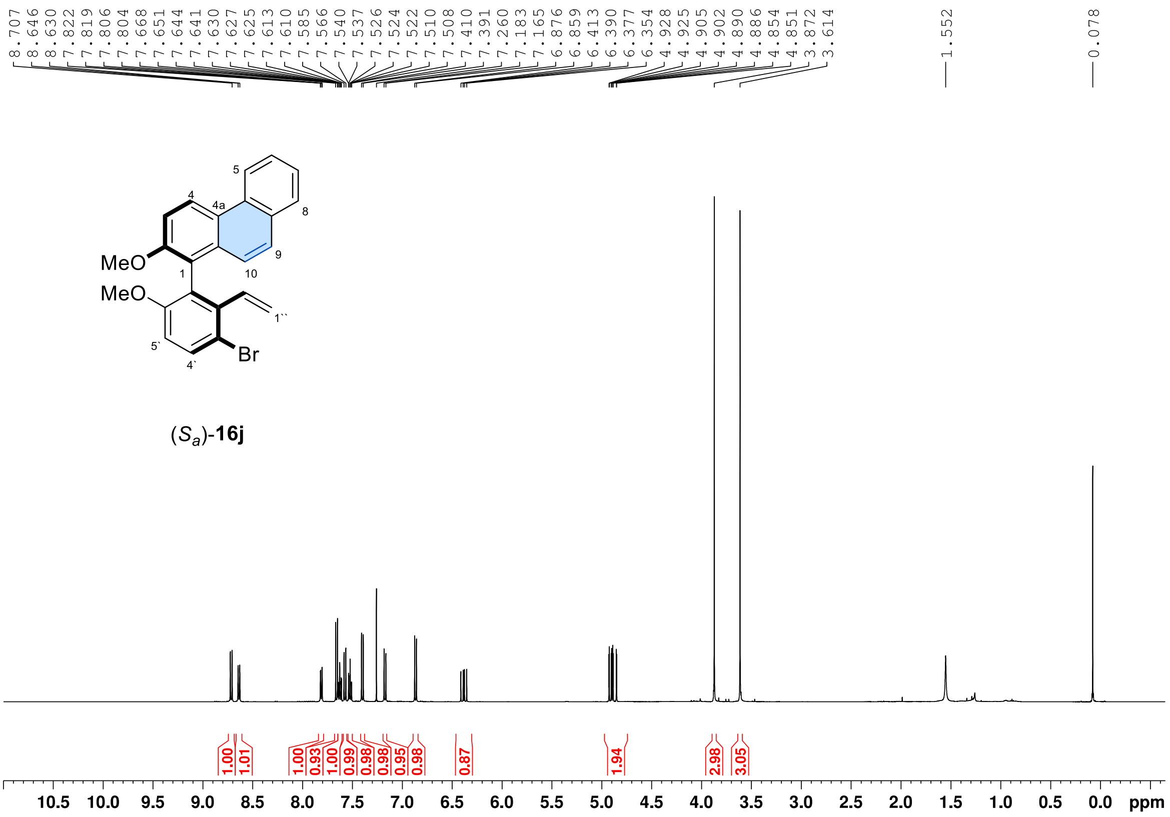

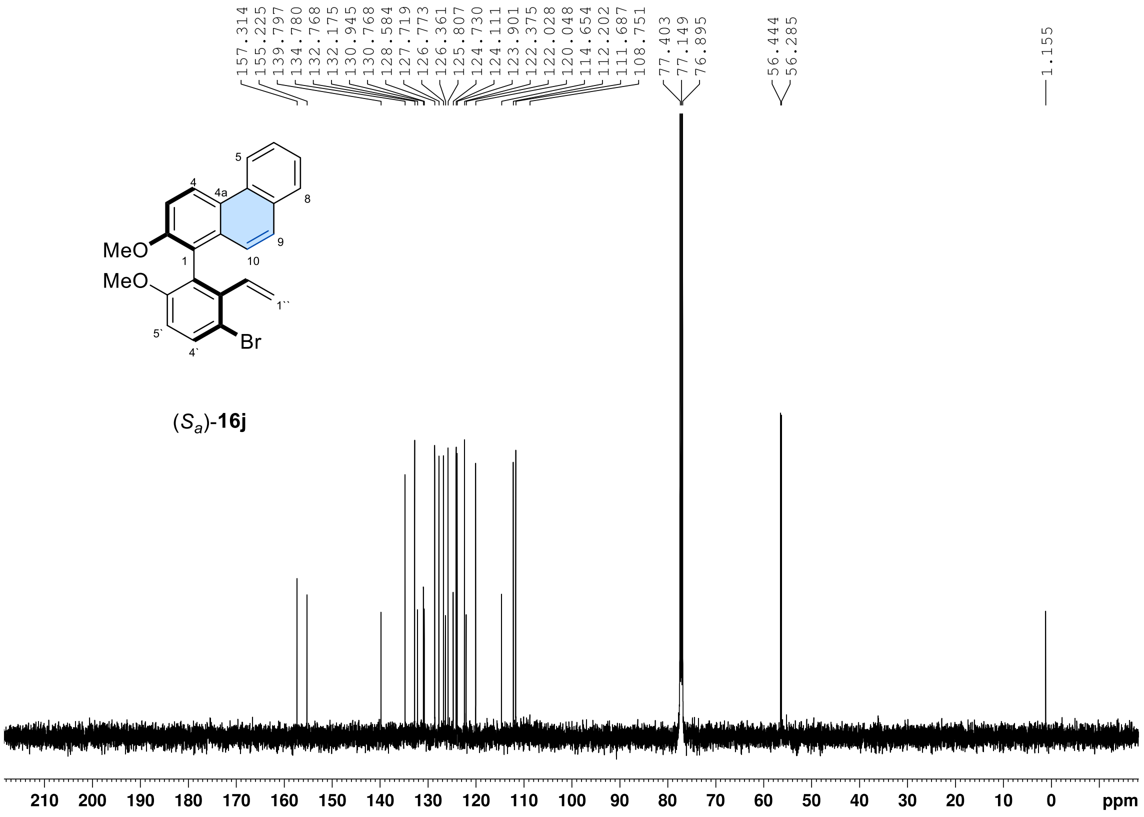

(S_a)-1-(3-Bromo-6-methoxy-2-vinylphenyl)-6-chloro-2-methoxyphenanthrene ((S_a)-16k):

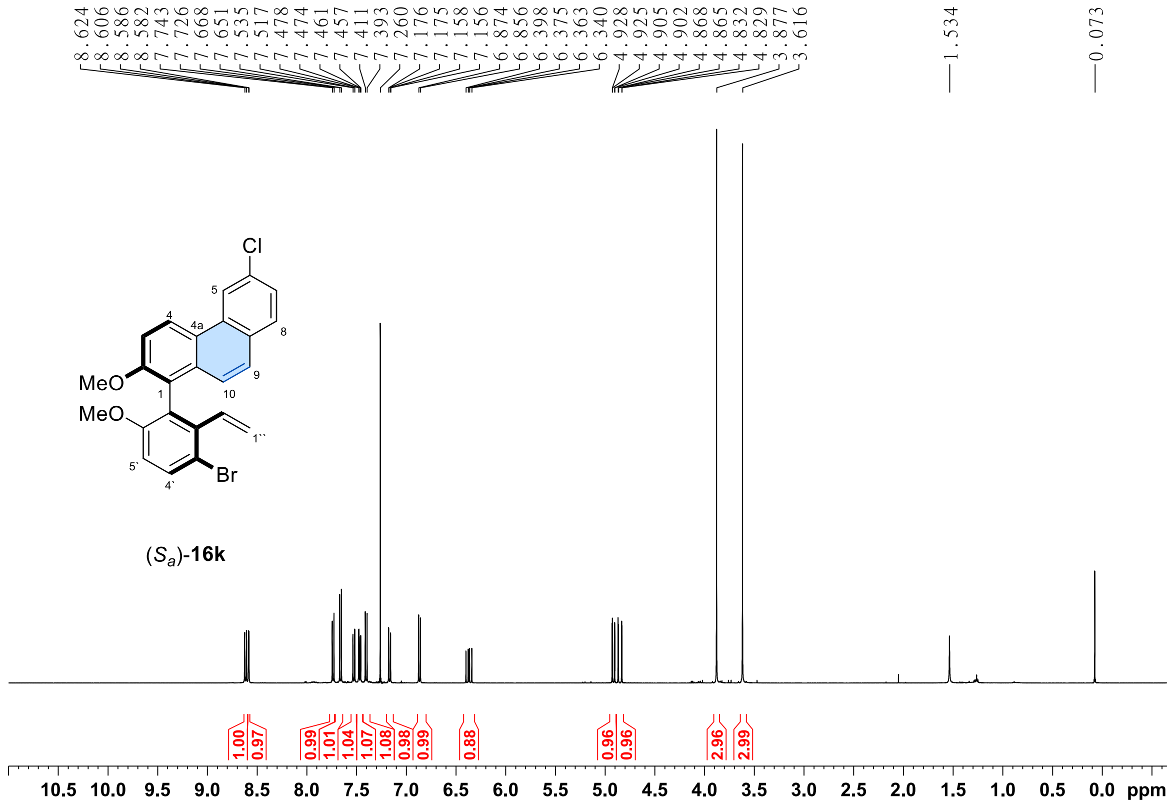

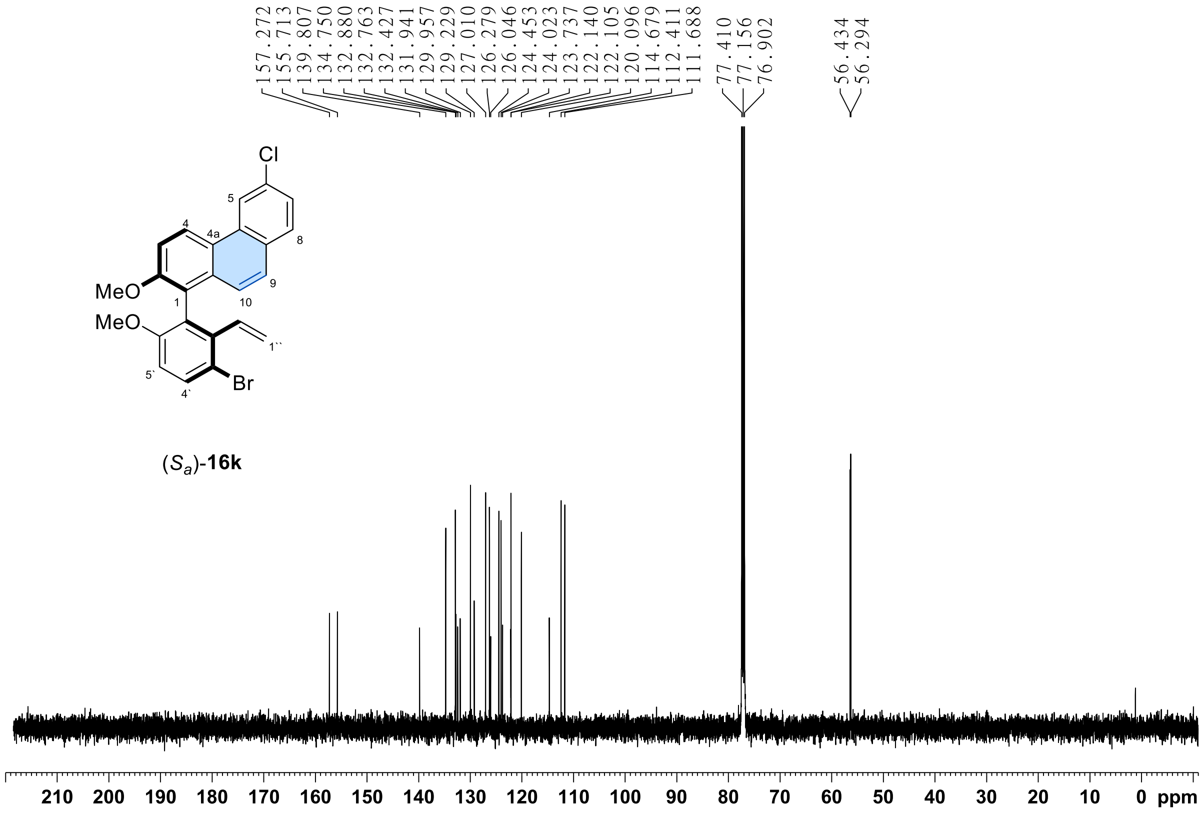

(S_a)-2,2'-Dimethoxy-1,1'-biphenanthrene ((S_a)-18a):

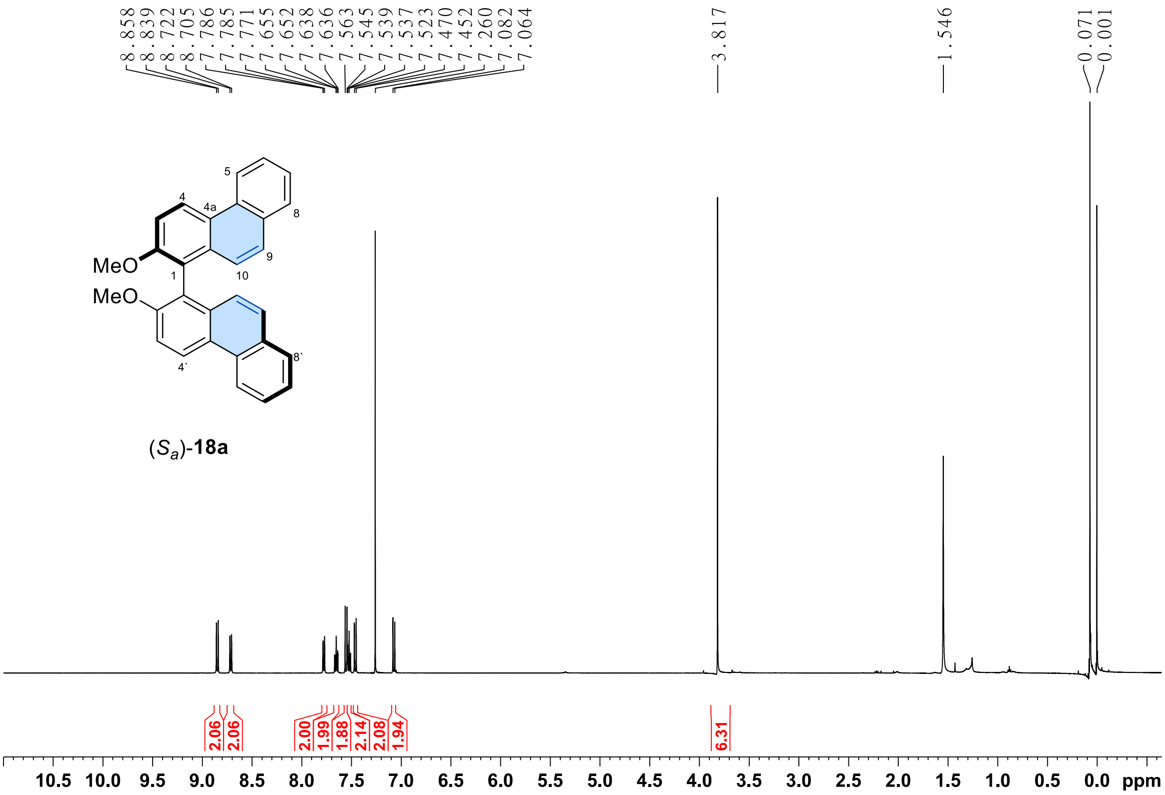

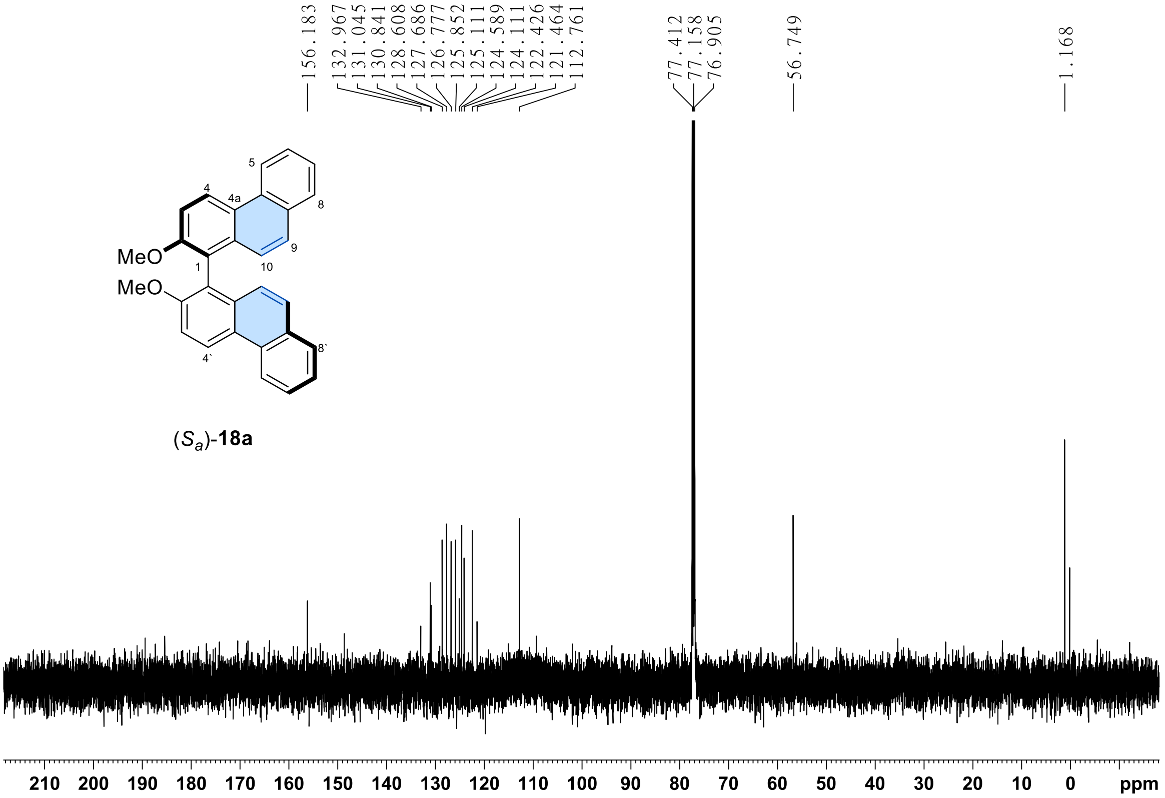

(S_a)-7-(2-Methoxyphenanthren-1-yl)phenanthro[2,3-d][1,3]dioxole ((S_a)-18b):

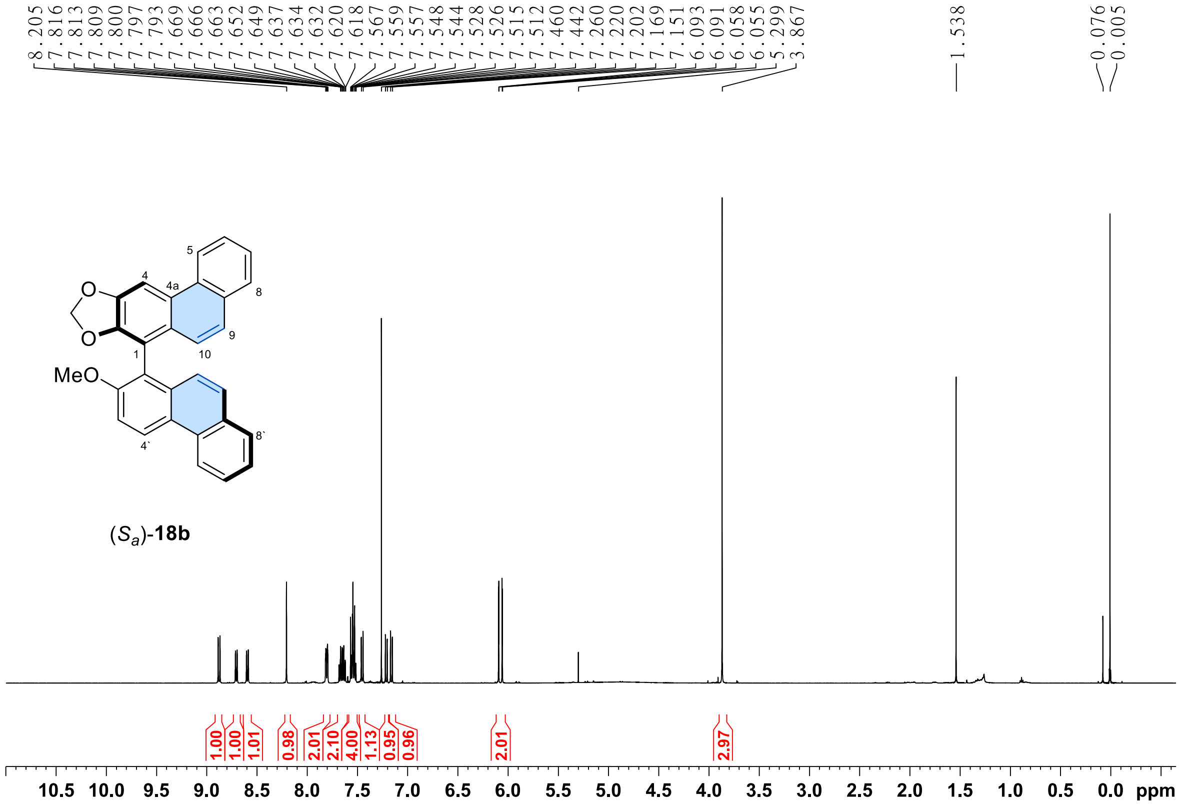

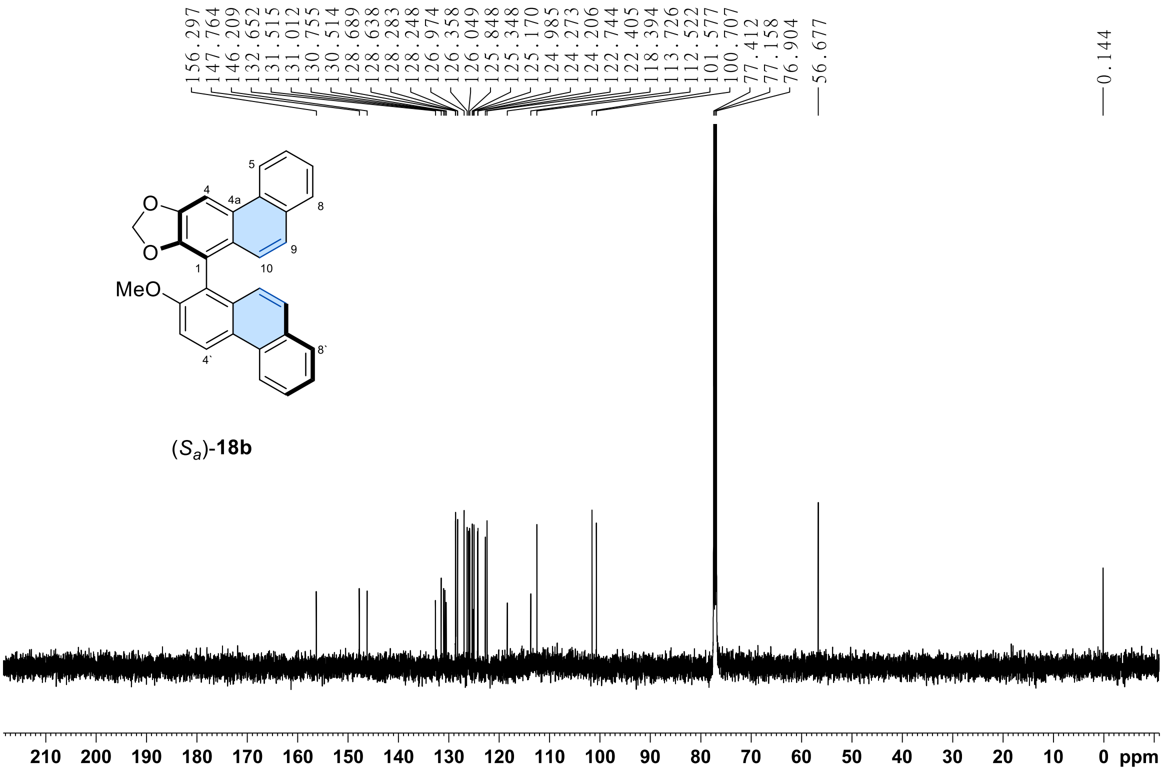

(R_a)-2-Methoxy-2'-methyl-1,1'-biphenanthrene ((R_a)-18c):

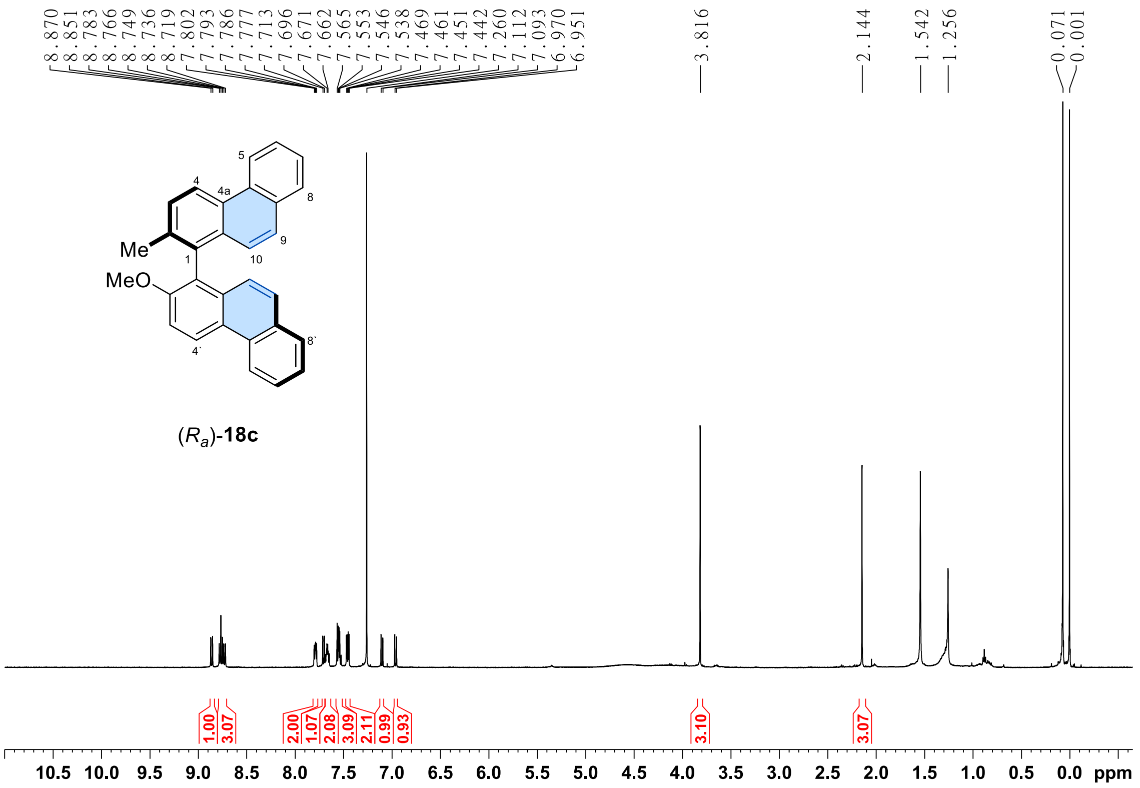

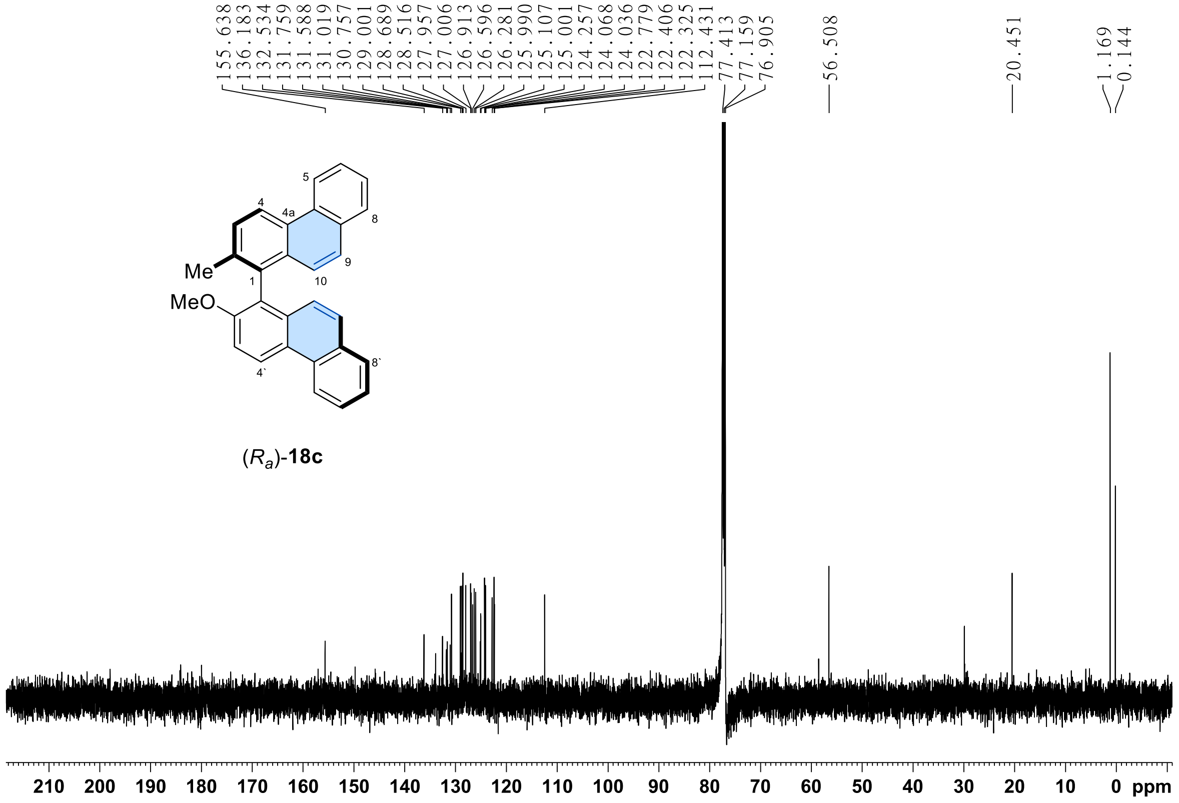

(S_a)-2,2'-Dimethyl-1,1'-biphenanthrene ((S_a)-18d):

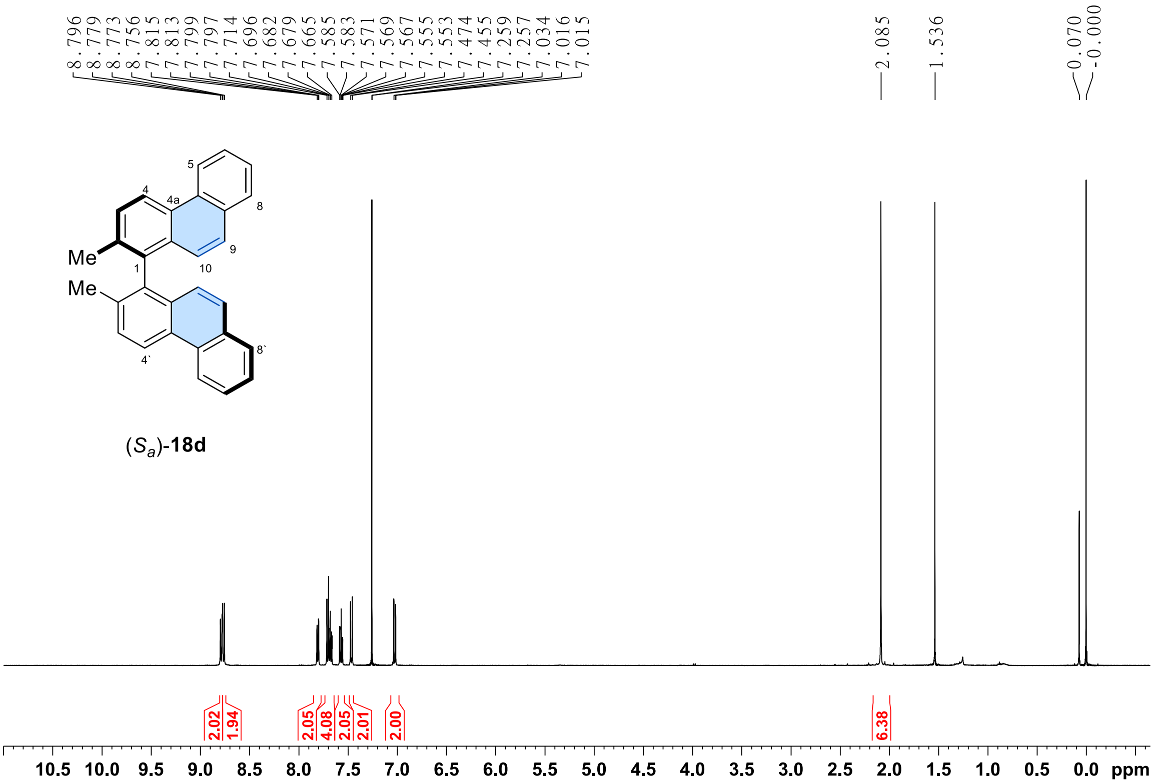

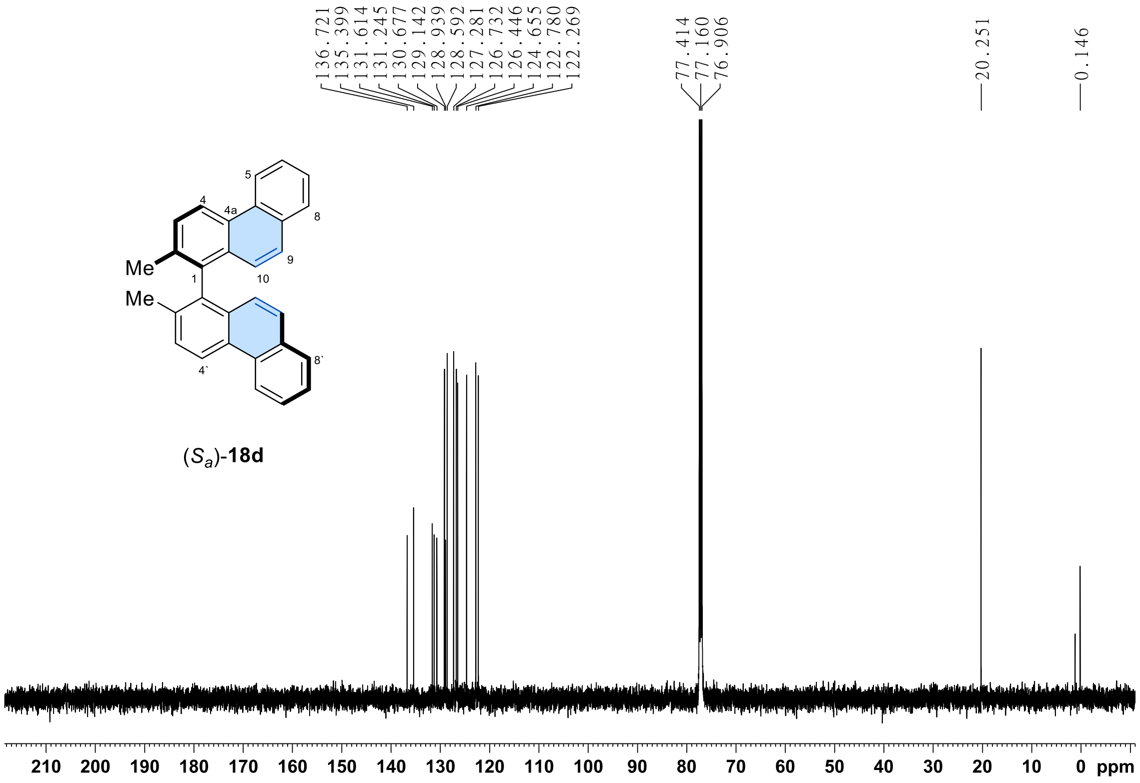

(S_a)-6-Chloro-2,2'-dimethoxy-1,1'-biphenanthrene ((S_a)-18e):

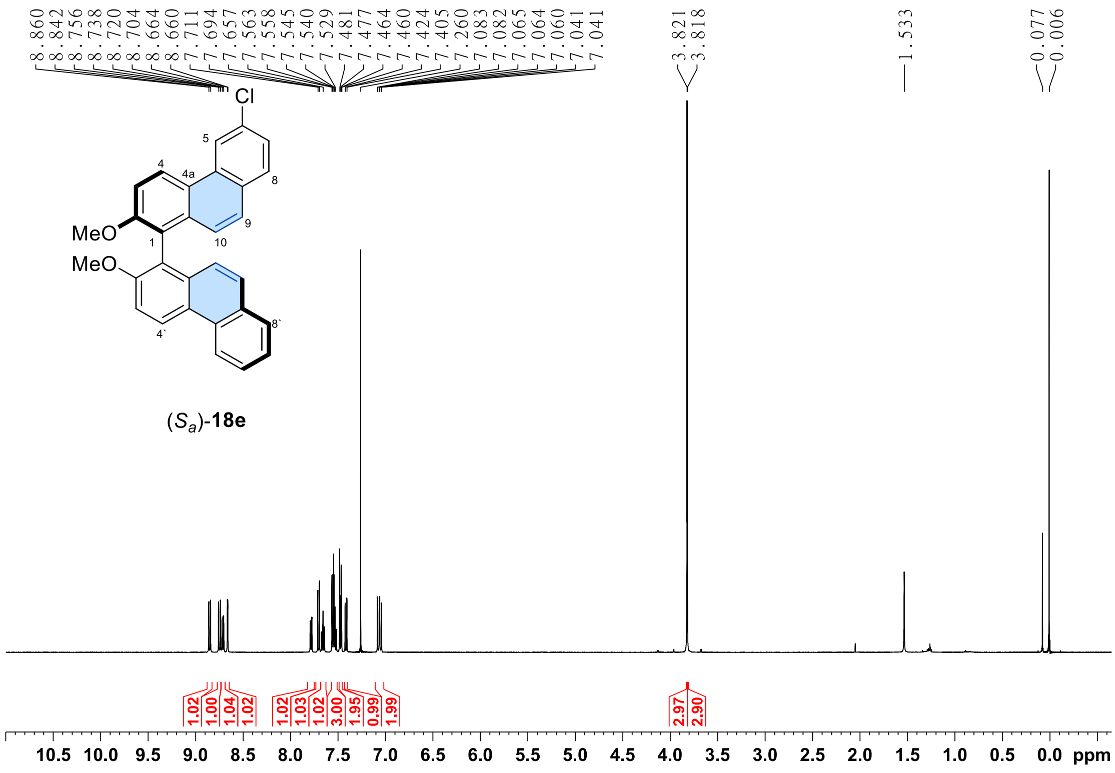

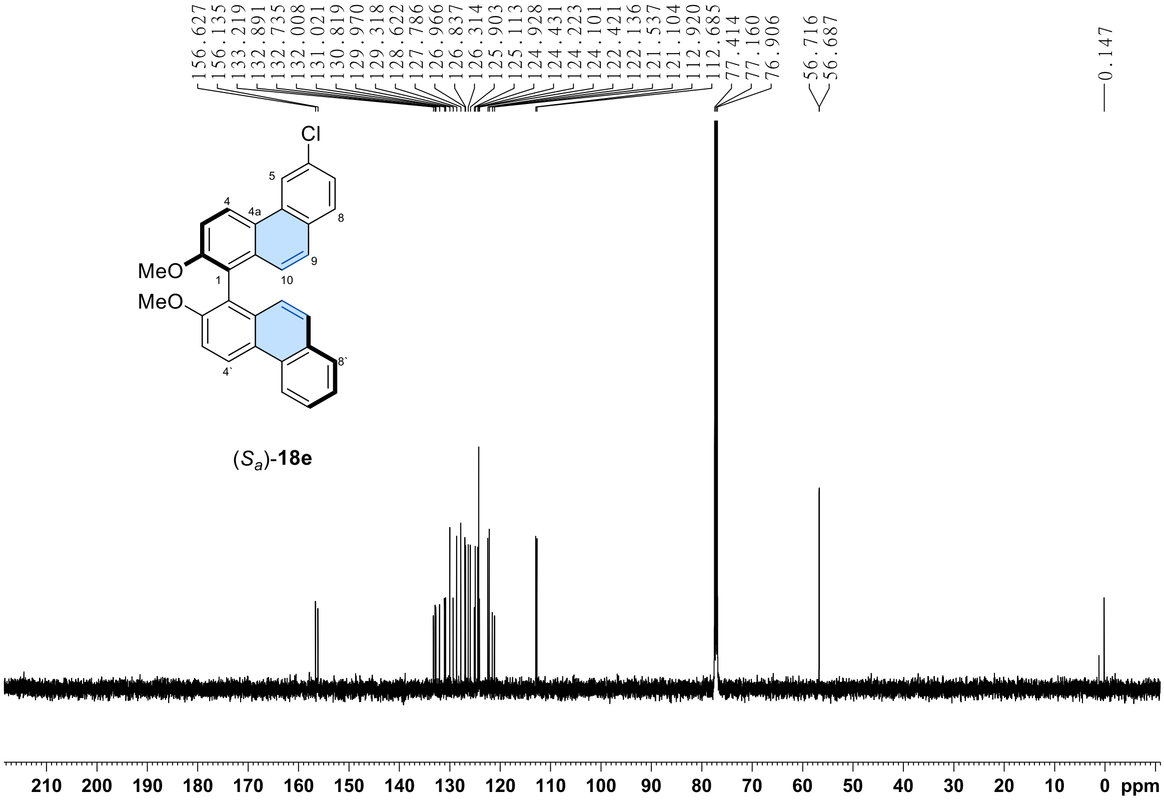

(S_a)-6,6'-Difluoro-2,2'-dimethoxy-1,1'-biphenanthrene ((S_a)-18f):

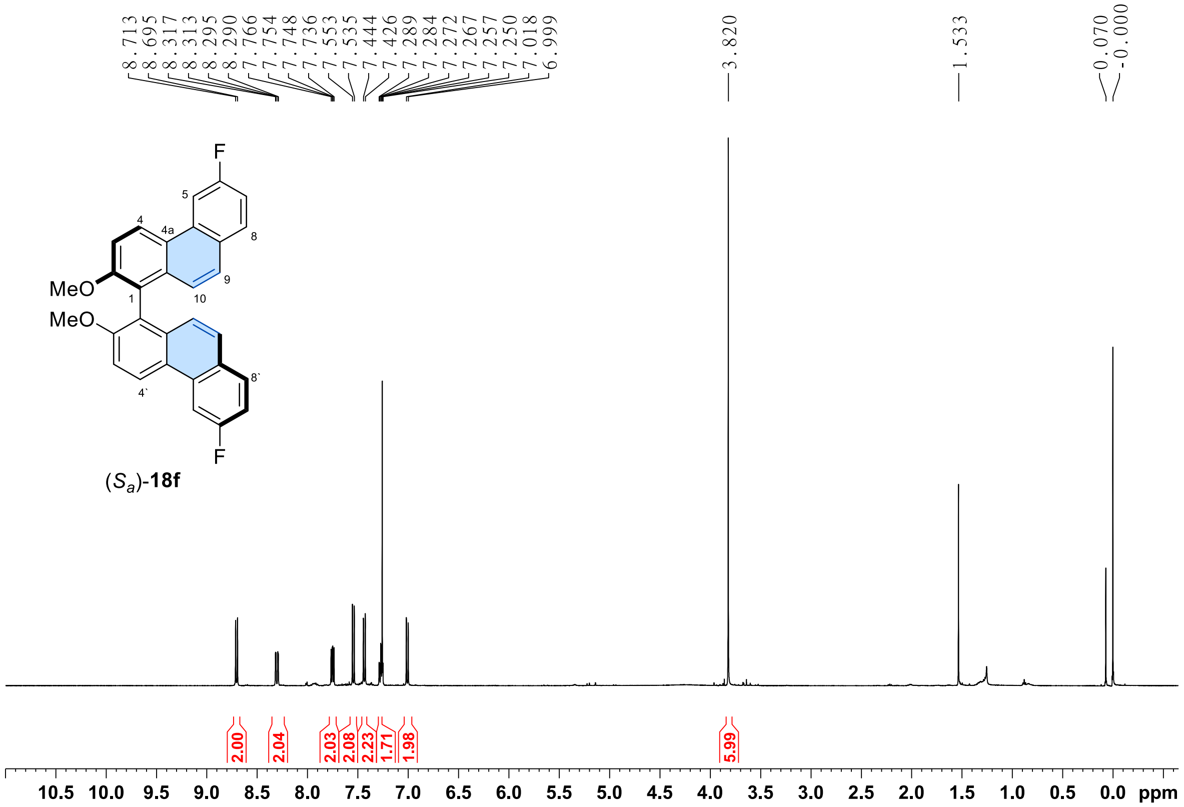

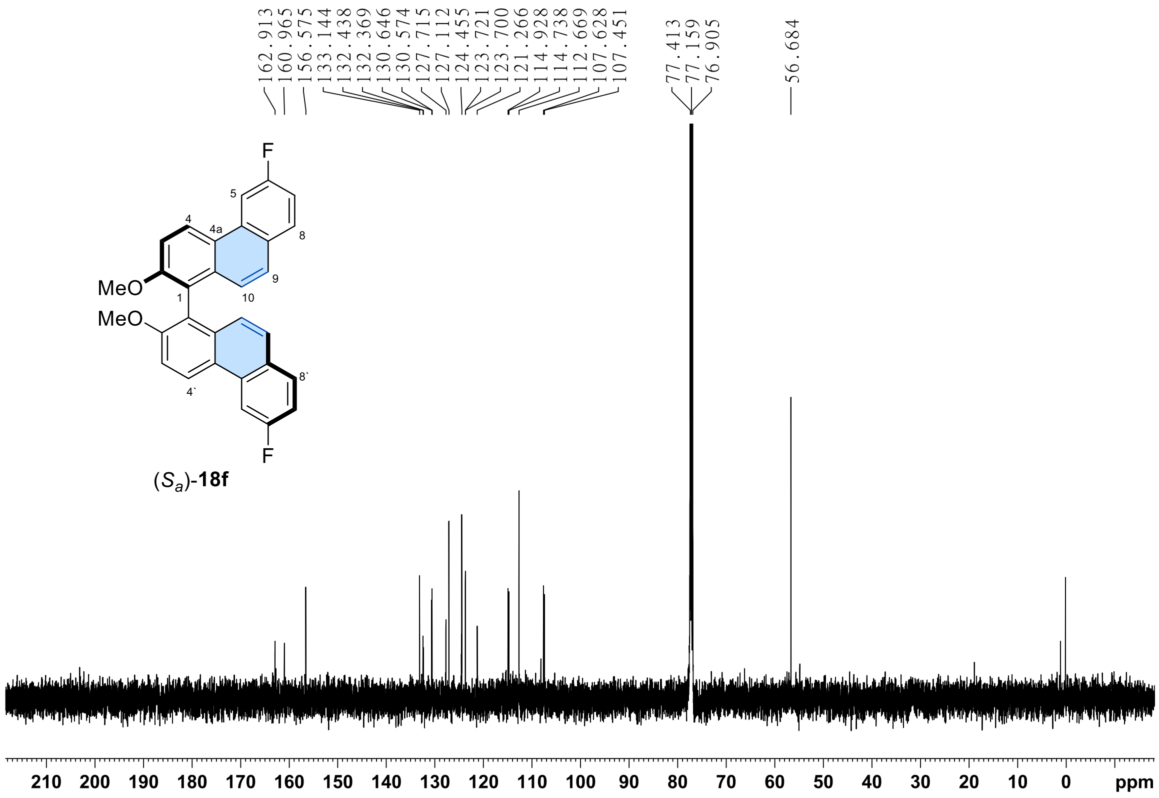

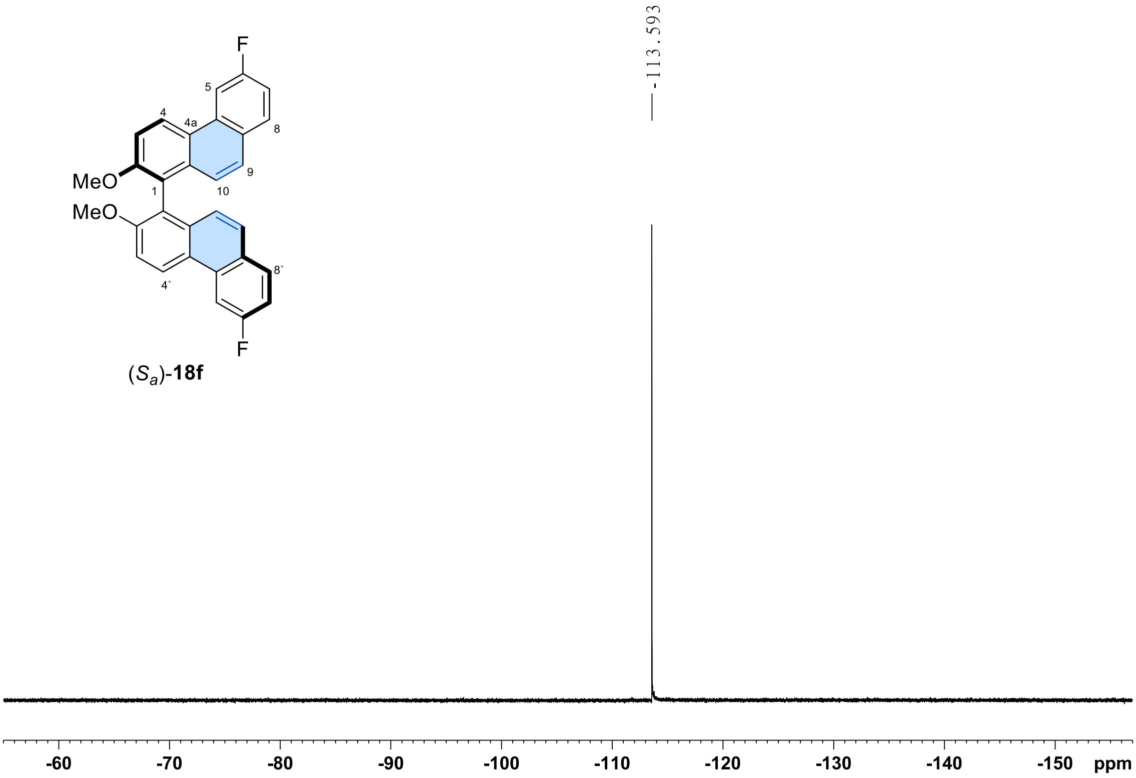
F
5
4
4a
8
MeO
1
9
10
MeO
8`
4`
F
-113.593
-60
-70
-80
-90
-100
-110
-120
-130
-140
-150
ppm

(S_a)-**18f**

(R_a)-2,6,6'-Trimethoxy-2'-methyl-1,1'-biphenanthrene ((S_a)-18g):

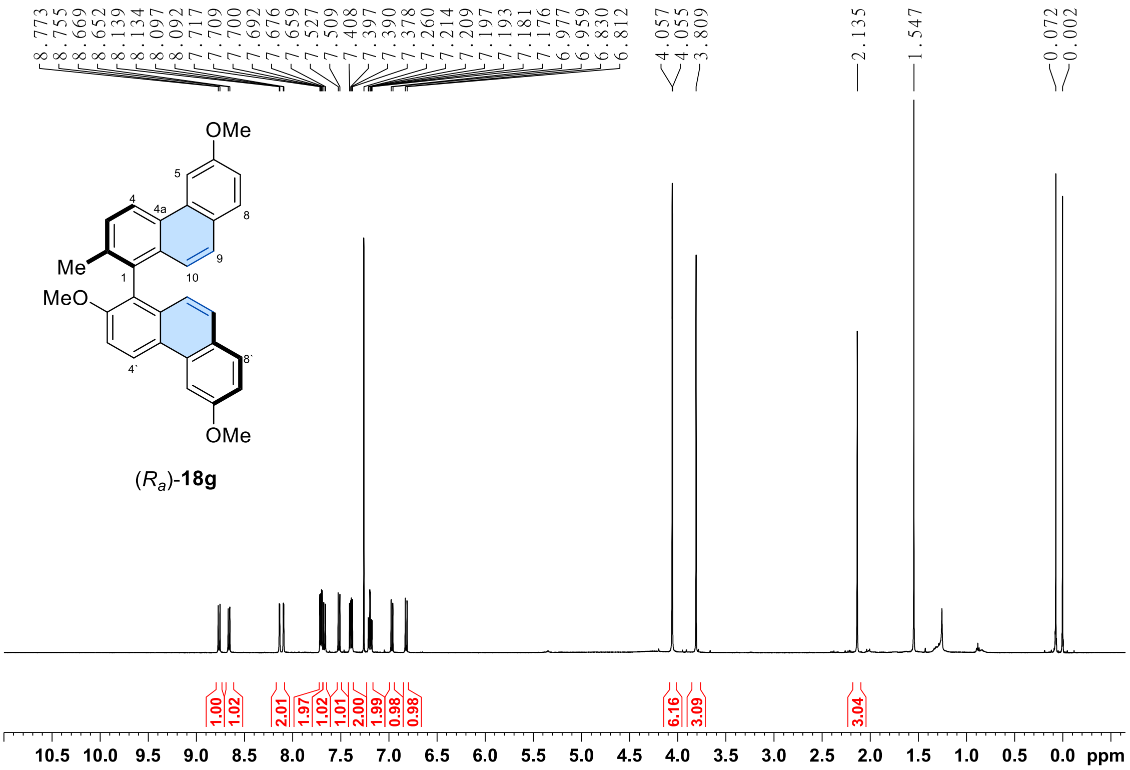

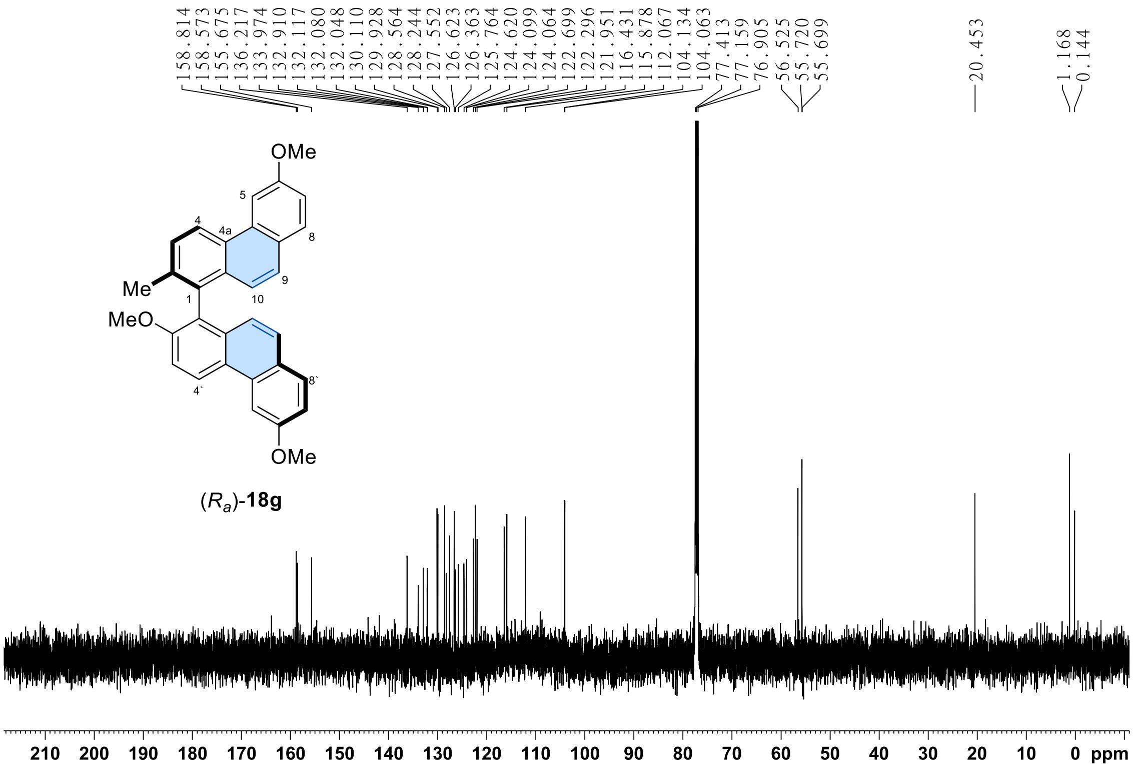

(S_a)-2,2'-Dimethoxy-6,6'-bis(trifluoromethyl)-1,1'-biphenanthrene ((S_a)-18h):

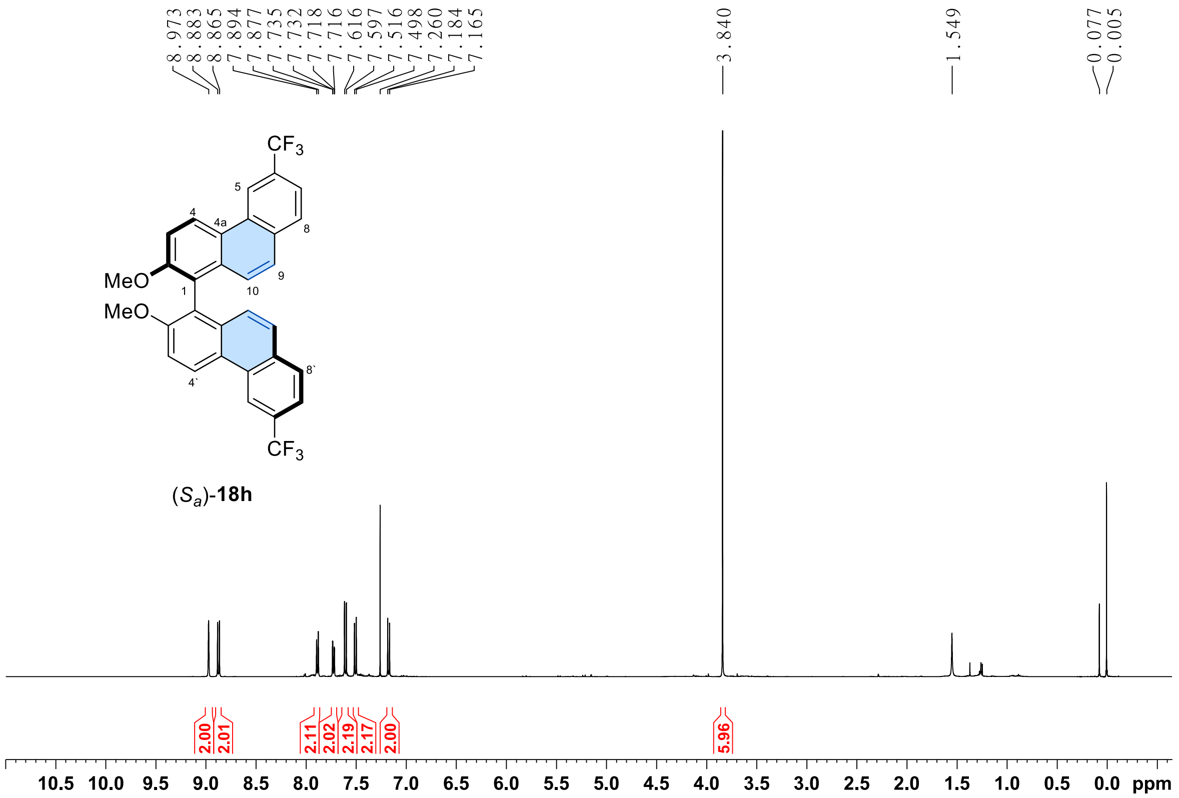

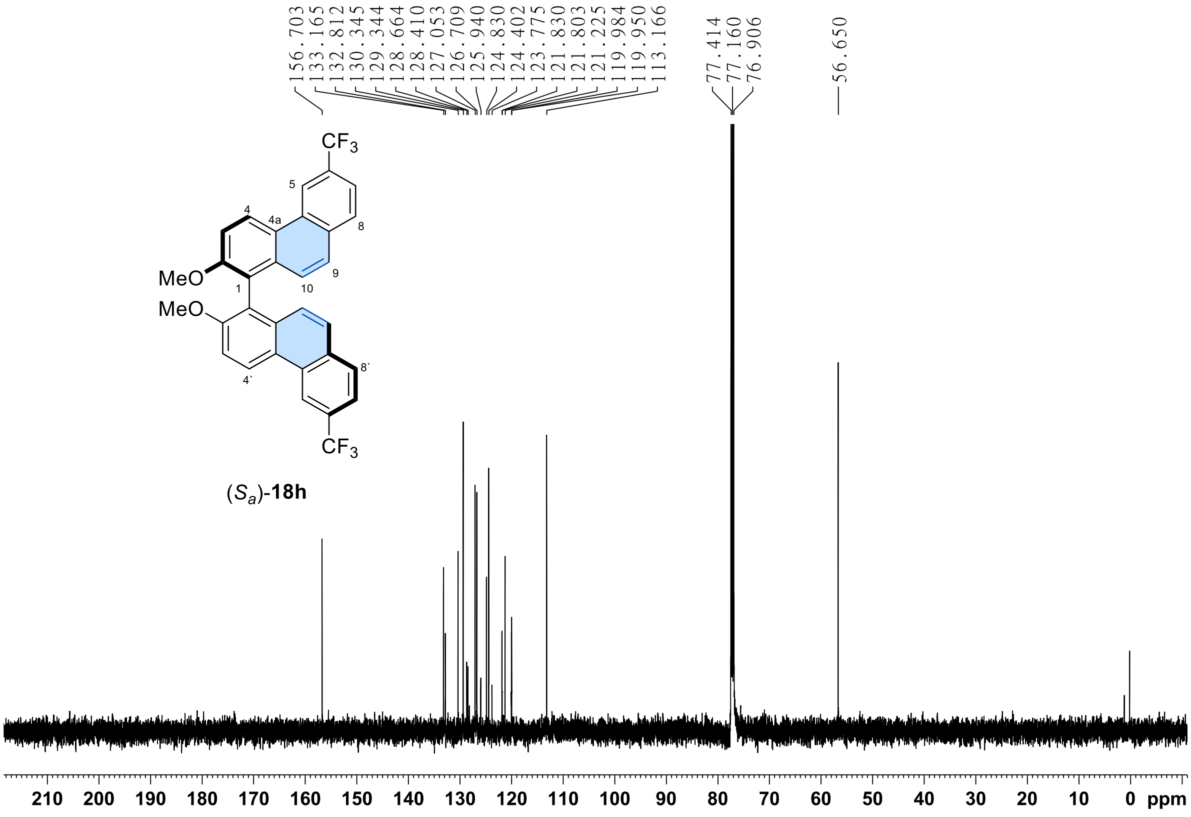

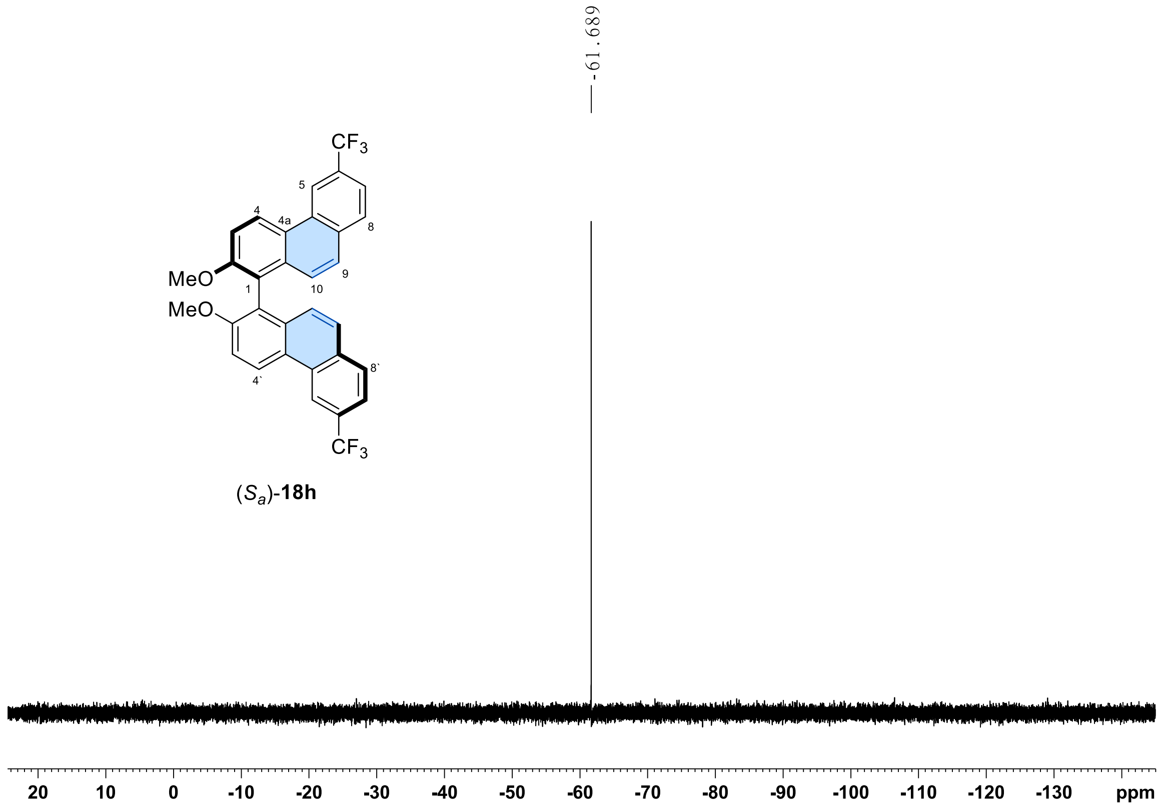
-61.689
CF3
5
4
4a
8
MeO
9
1
10
MeO
8`
4`
CF3
(Sa)-18h
20
10
0
-10
-20
-30
-40
-50
-60
-70
-80
-90
-100
-110
-120
-130
ppm

(S_a)-6,6'-Dichloro-2,2'-dimethoxy-1,1'-biphenanthrene ((S_a)-18i):

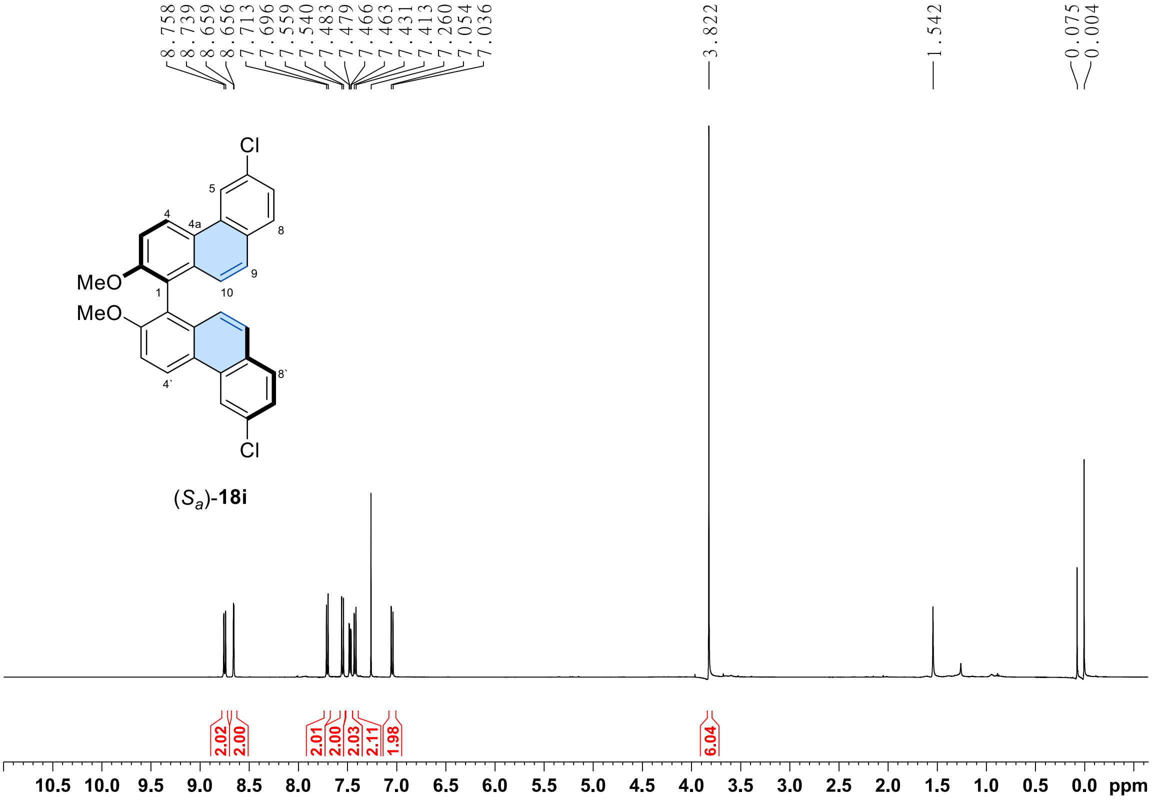

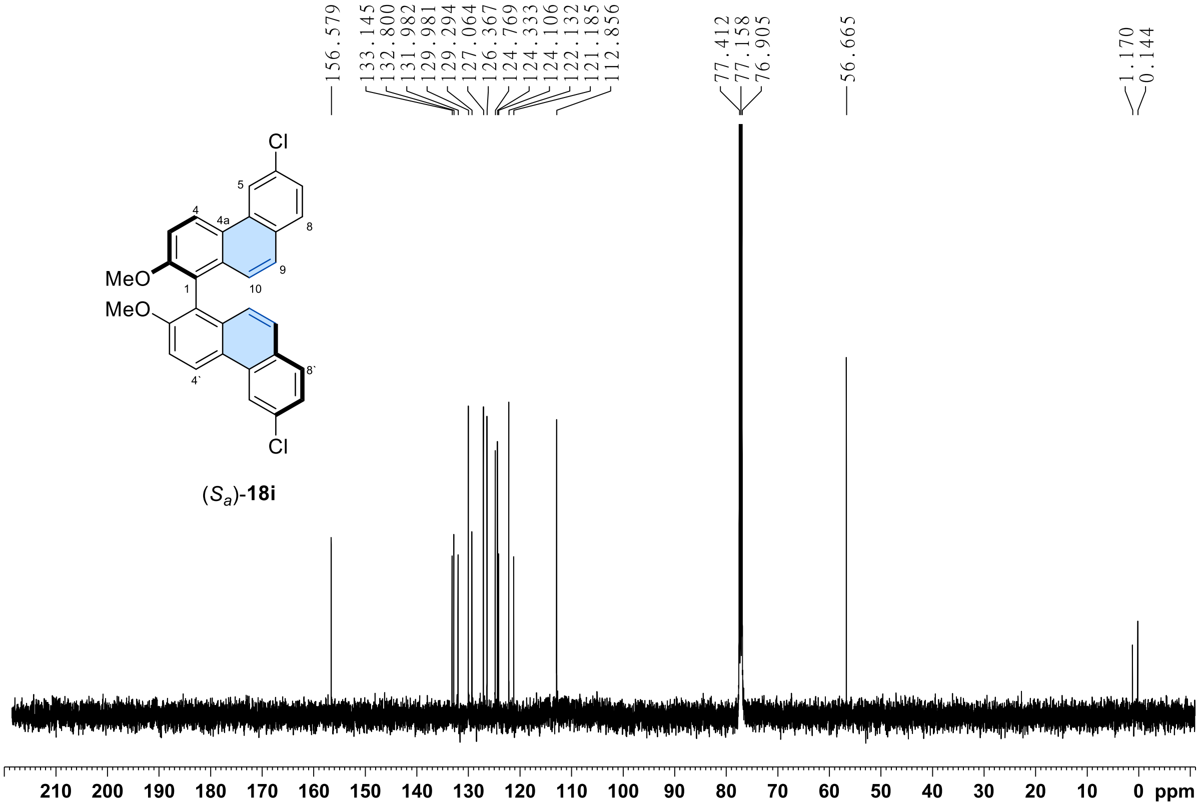

(R_a)-2-Methyl-1-(phenanthren-1-yl)-1H-indole ((R_a)-20a):

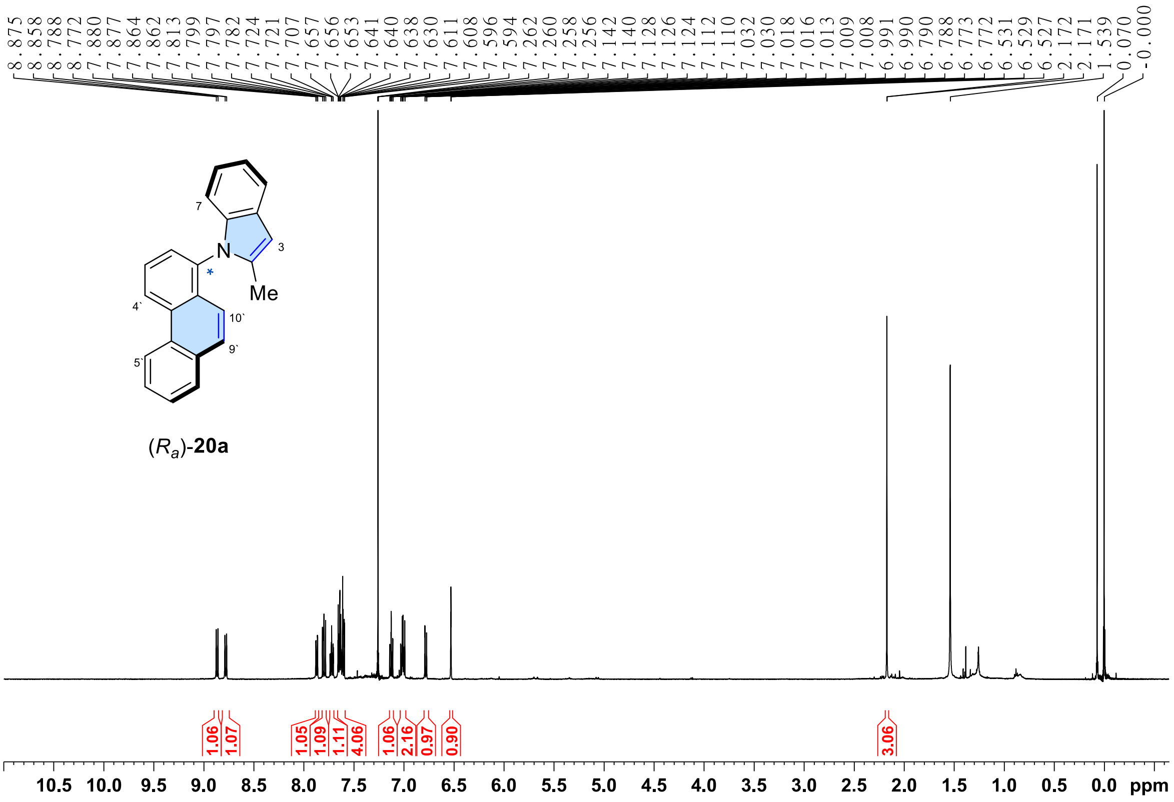

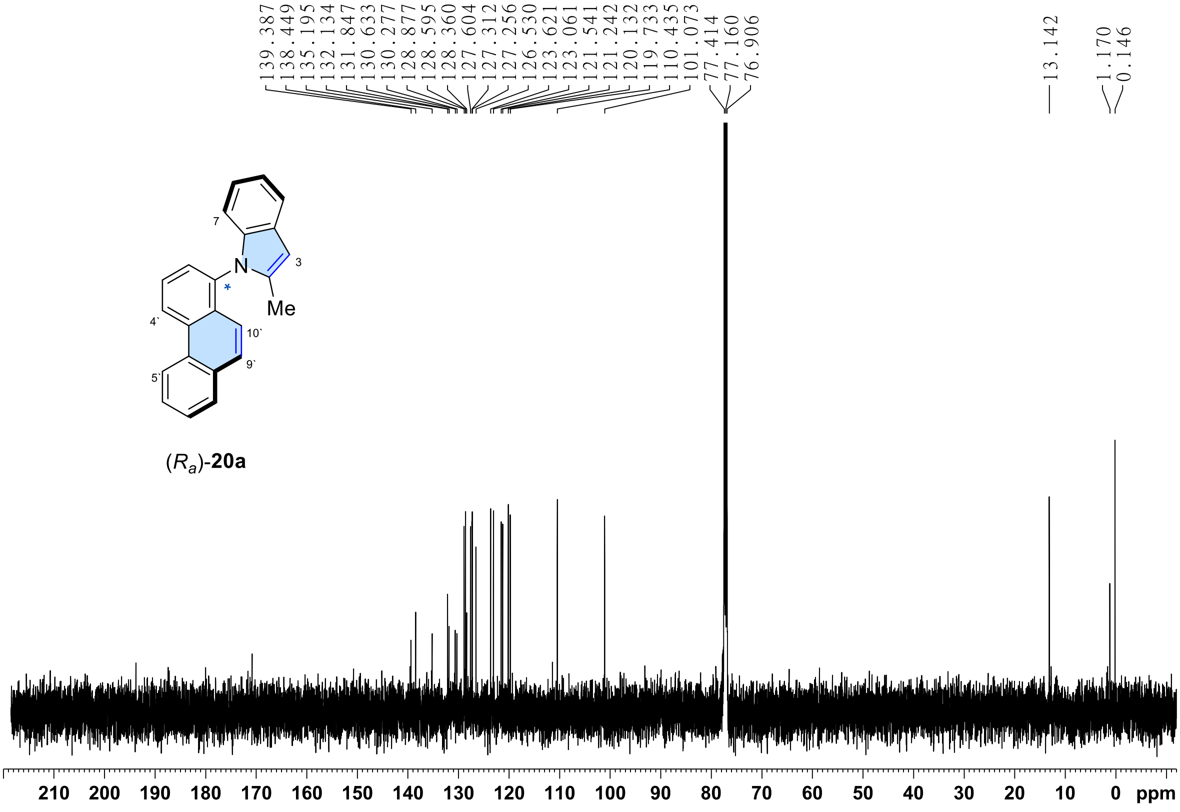

(S_a)-1-(2-Methoxyphenanthren-1-yl)-1*H*-indole ((S_a)-20b):

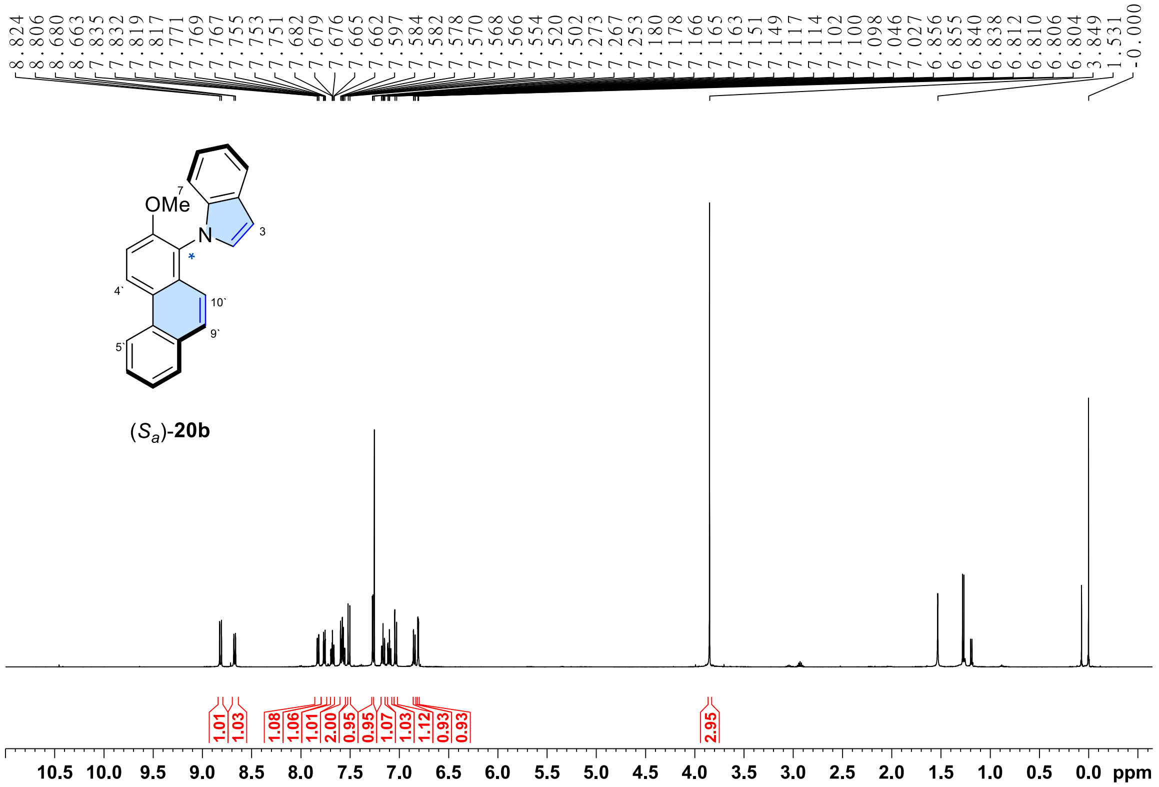

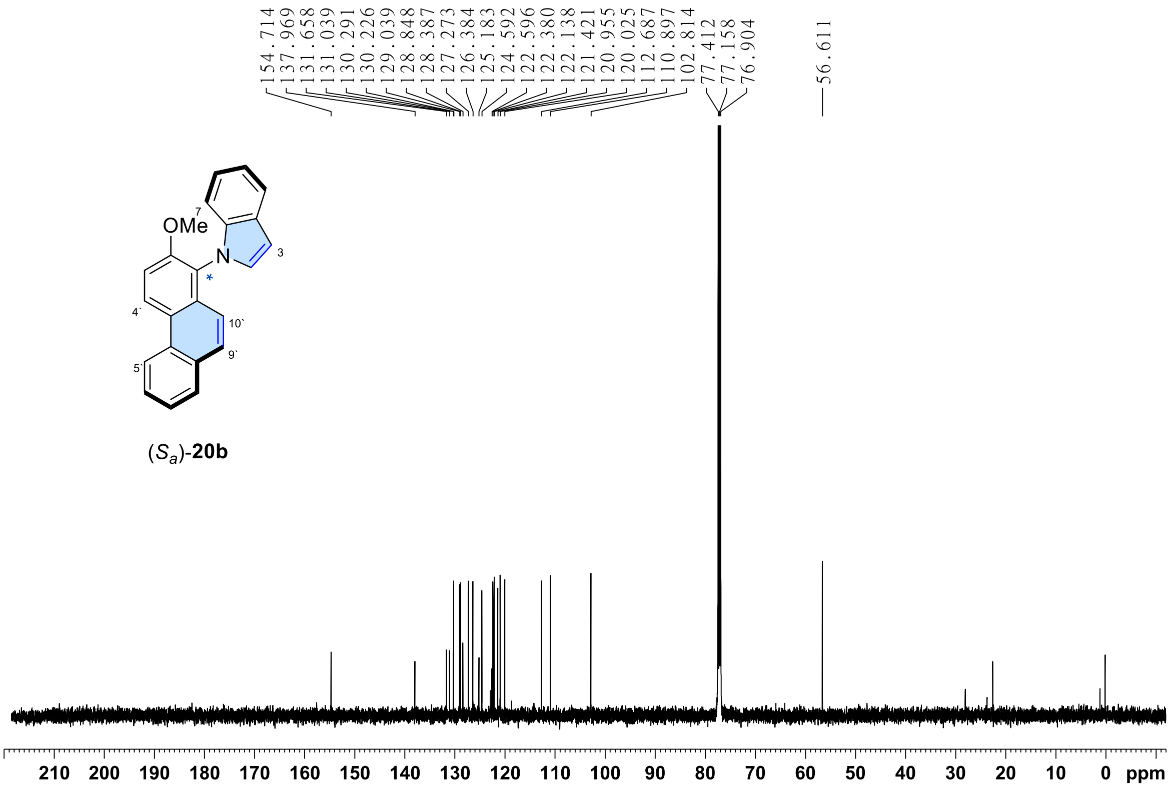

(R_a)-1-(2-Methylphenanthren-1-yl)-1*H*-indole ((R_a)-20c):

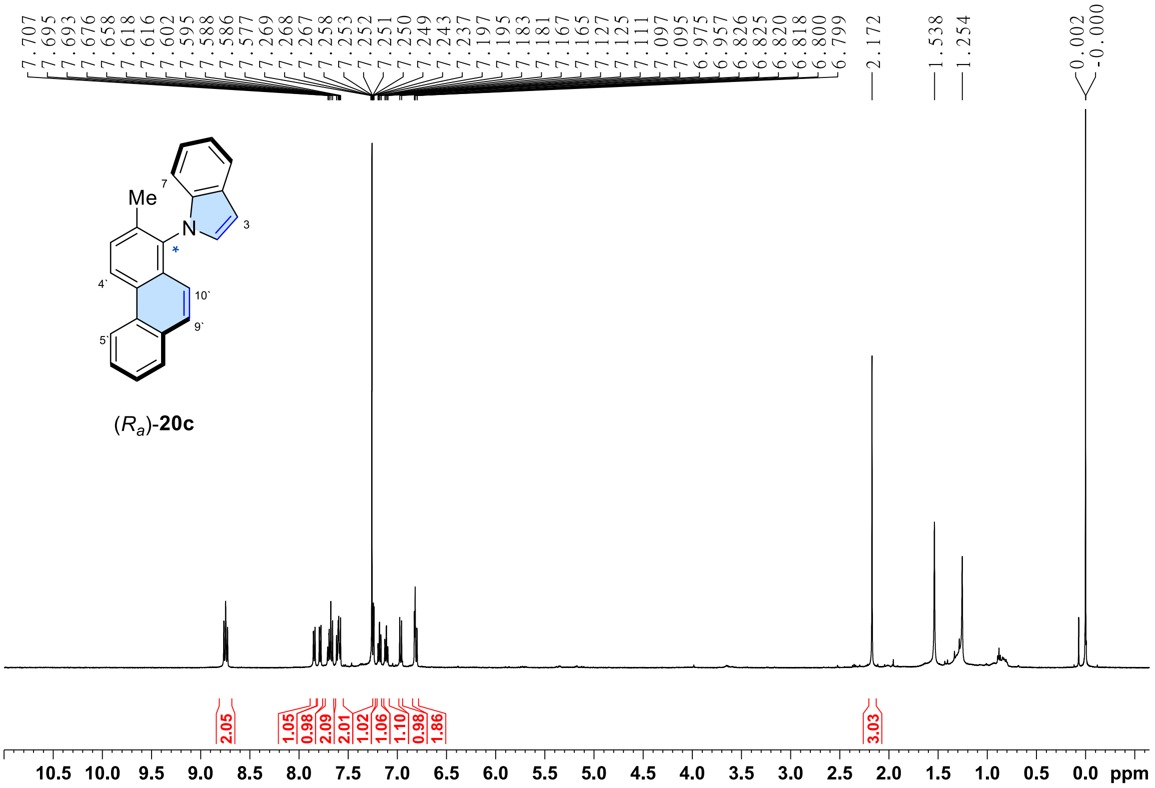

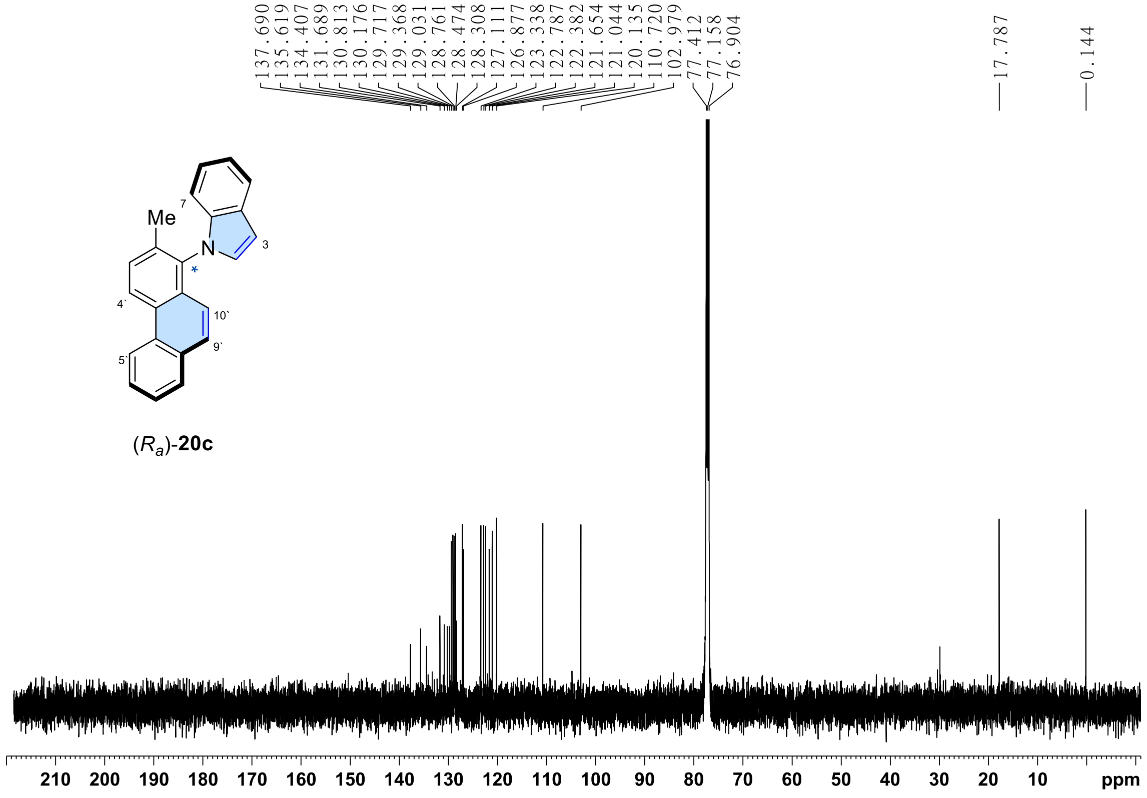

(*S*$_a$)-1-(2-Chlorophenanthren-1-yl)-1*H*-indole ((*S*$_a$)-20d):

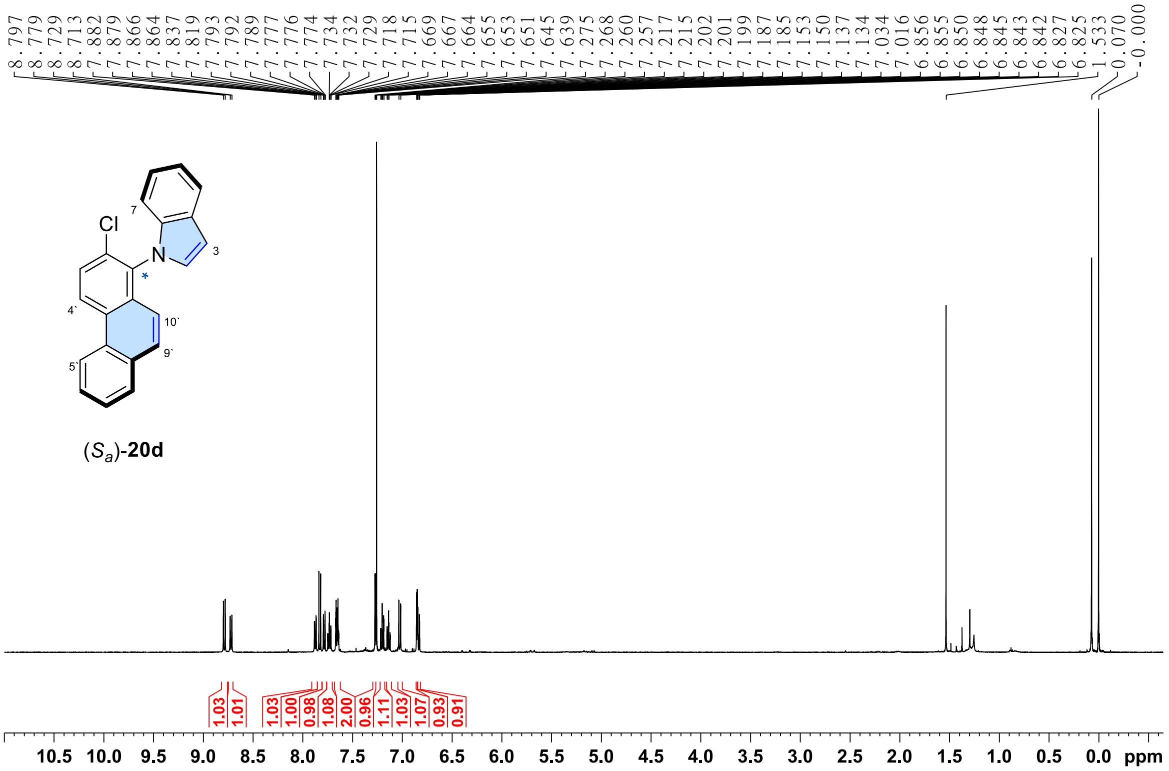

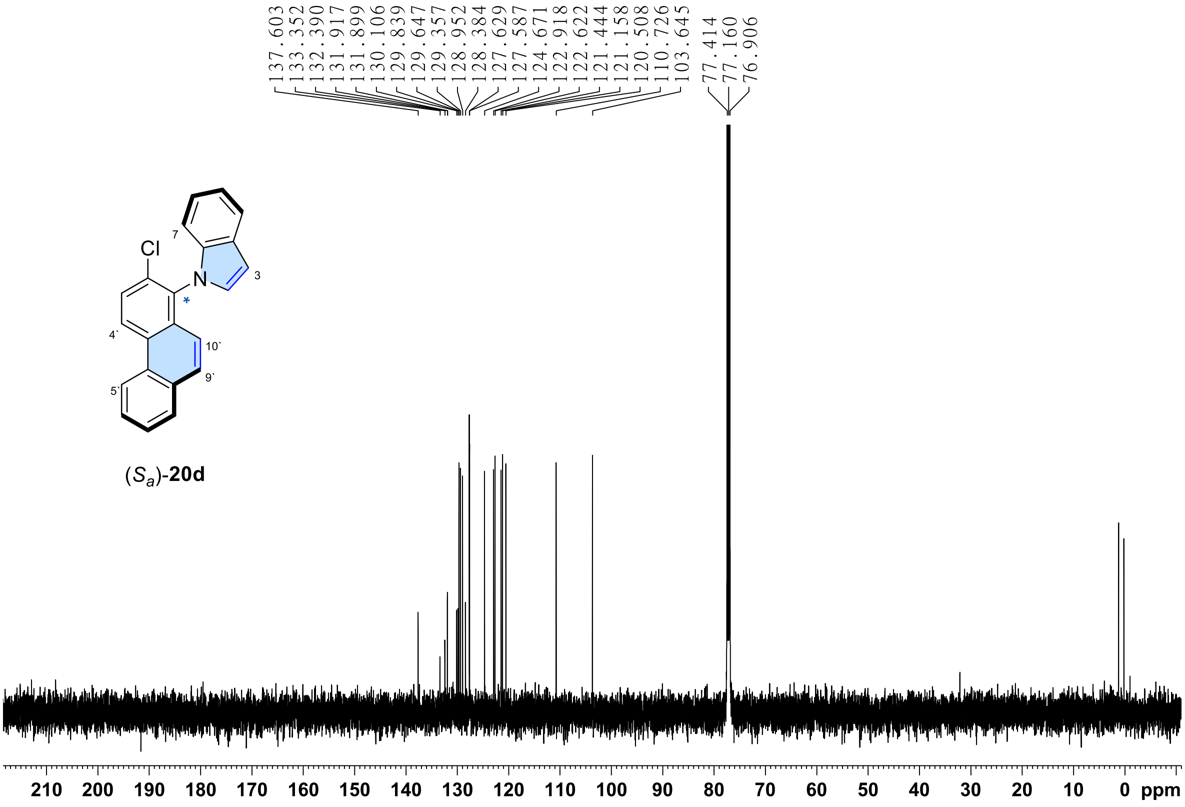

(S_a)-1-(2,6-Dichlorophenanthren-1-yl)-1H-indole ((S_a)-20e):

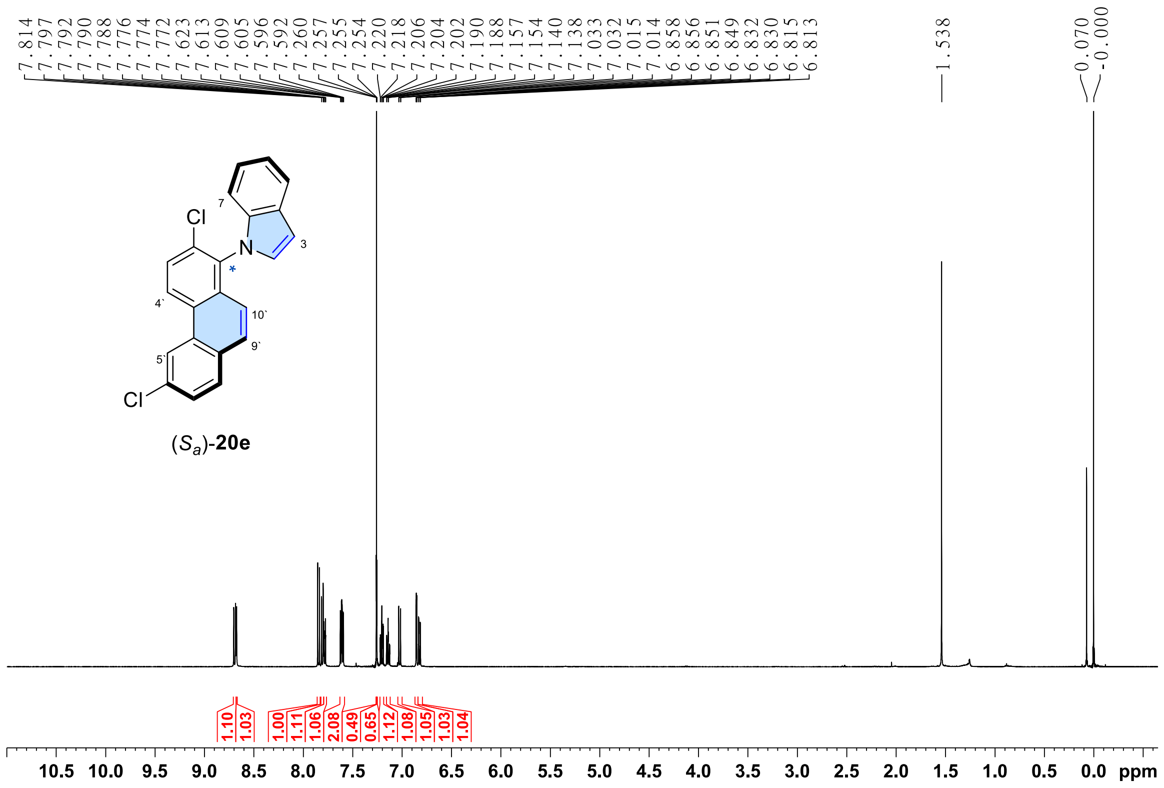

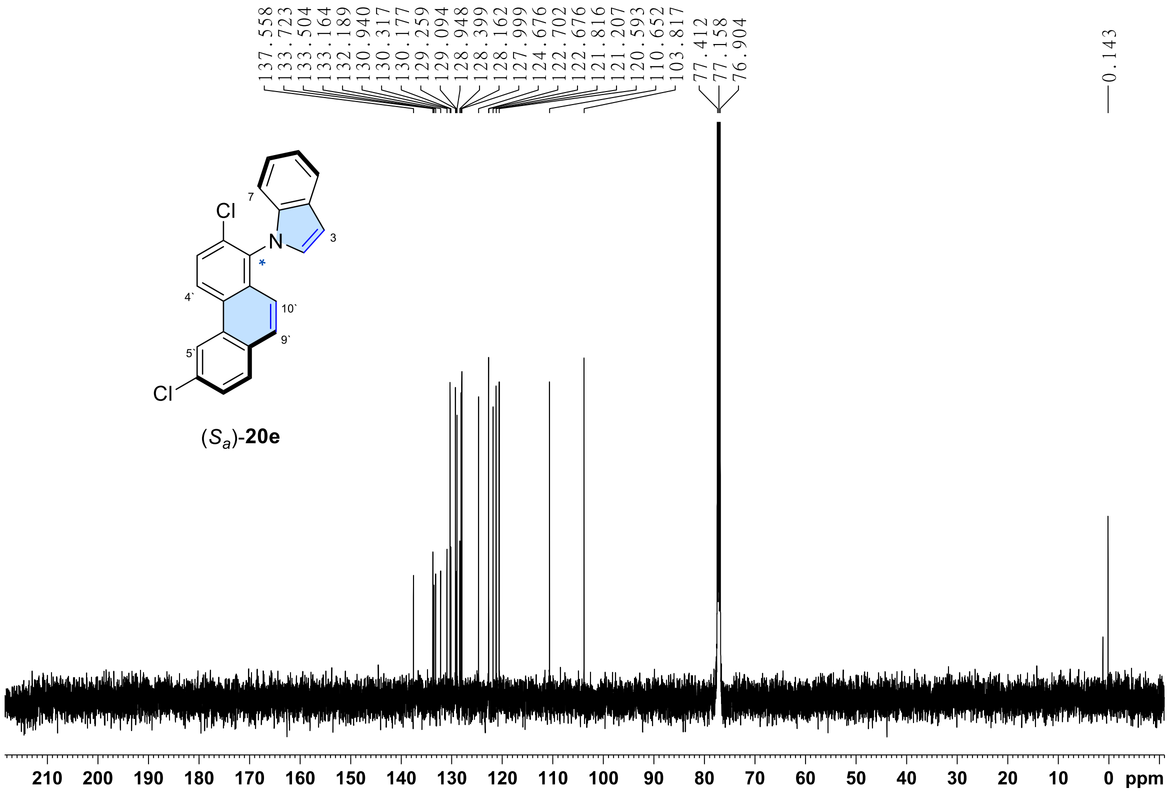

HPLC Data

(R_a)-1-(2,4-Dimethoxy-6-vinylphenyl)-2-methylphenanthrene ((R_a)-16a):

HPLC conditions: Chiralcel IC (3 µm, 250×4.6 mm), heptane/*i*-PrOH 97.5 : 2.5, 1.0 mL/min, 20ºC

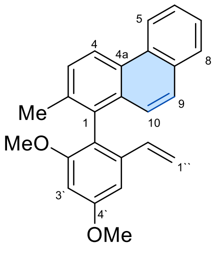

(±)-**16a**

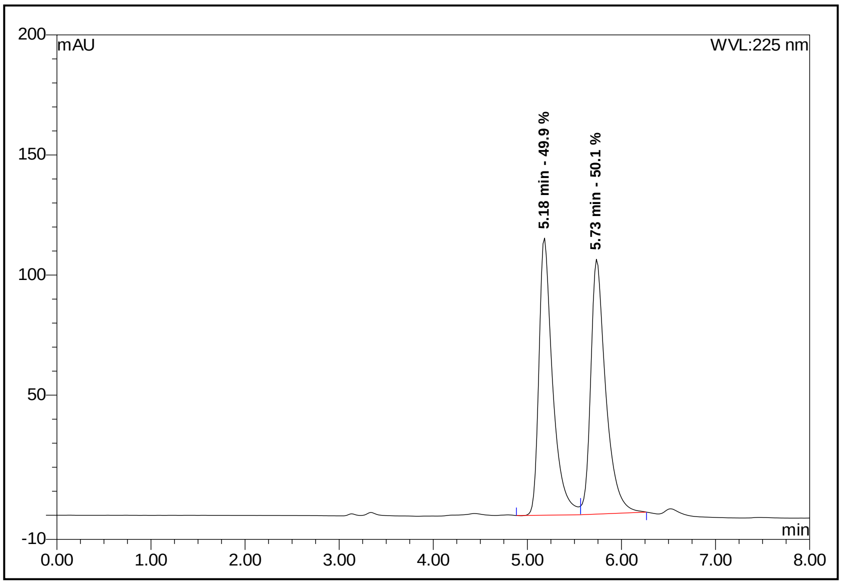

No.	Ret.Time min	Height mAU	Area mAU*min	Rel.Area %
1	5.18	115.398	19.595	49.93
2	5.73	106.133	19.653	50.07
**Total:**		221.532	39.248	100.00

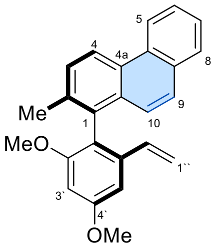

(R_a)-**16a**

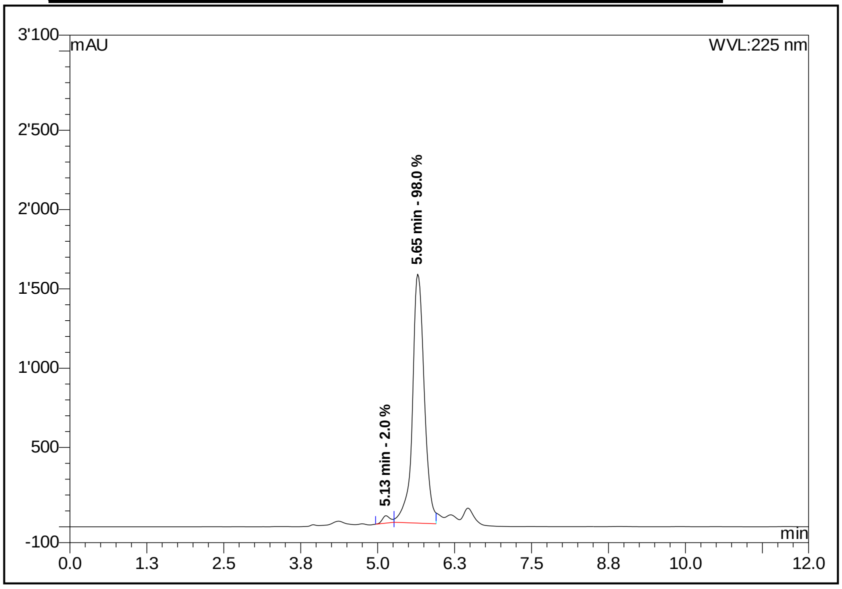

No.	Ret.Time min	Height mAU	Area mAU*min	Rel.Area %
1	5.13	46.571	7.018	1.96
2	5.65	1570.519	351.729	98.04
**Total:**		1617.089	358.746	100.00

(S_a)-7-(2,4-Dimethyl-6-vinylphenyl)phenanthro[2,3-d][1,3]dioxole ((S_a)-16b) :

HPLC conditions: Chiralcel IG (3 µm, 250×4.6 mm), heptane/*i*-PrOH 96 : 4, 1.0 mL/min, 20ºC

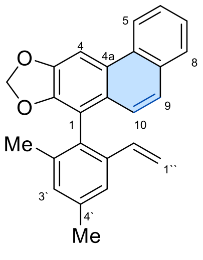

(±)-**16b**

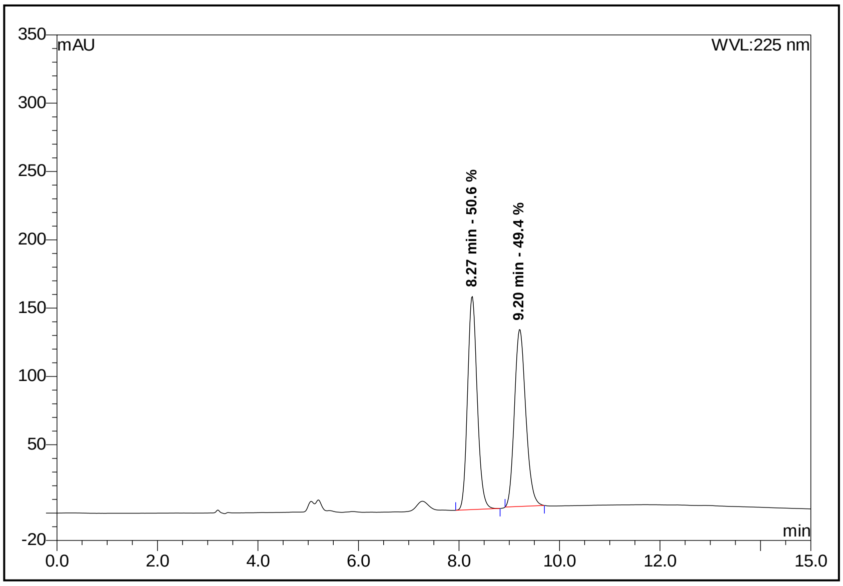

No.	Ret.Time min	Height mAU	Area mAU*min	Rel.Area %
1	8.27	155.997	34.686	50.57
2	9.20	129.497	33.906	49.43
**Total:**		285.494	68.592	100.00

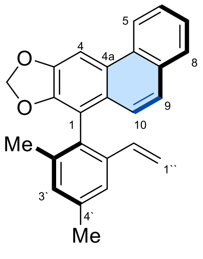

(S_a)-**16b**

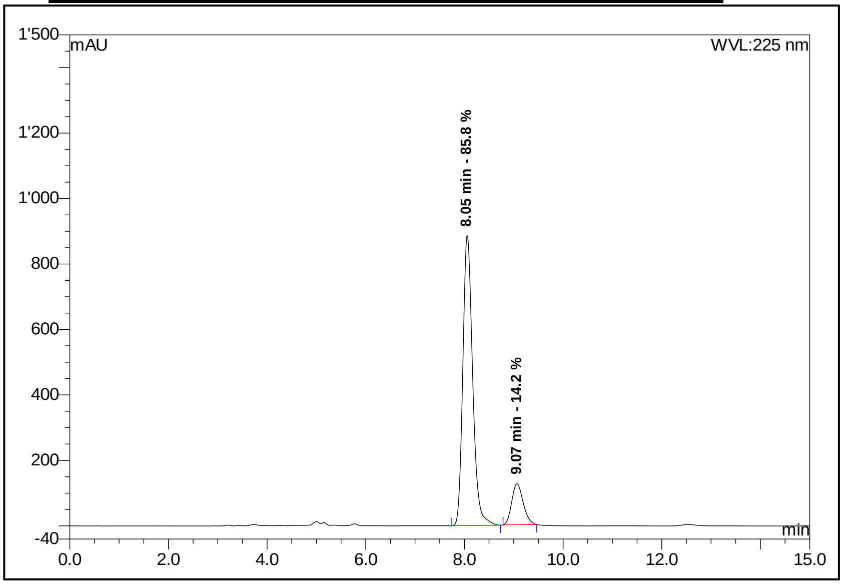

No.	Ret.Time min	Height mAU	Area mAU*min	Rel.Area %
1	8.05	885.442	203.904	85.80
2	9.07	125.794	33.754	14.20
**Total:**		1011.236	237.658	100.00

(R_a)-1-(2,4-Dimethoxy-6-vinylphenyl)phenanthrene ((R_a)-16c):

HPLC conditions: Chiralcel IB (3 µm, 250×4.6 mm), heptane/*i*-PrOH 97.5 : 2.5, 1.0 mL/min, 20ºC

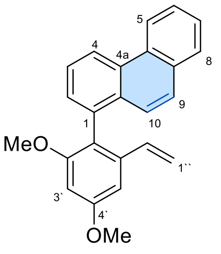

(±)-**16c**

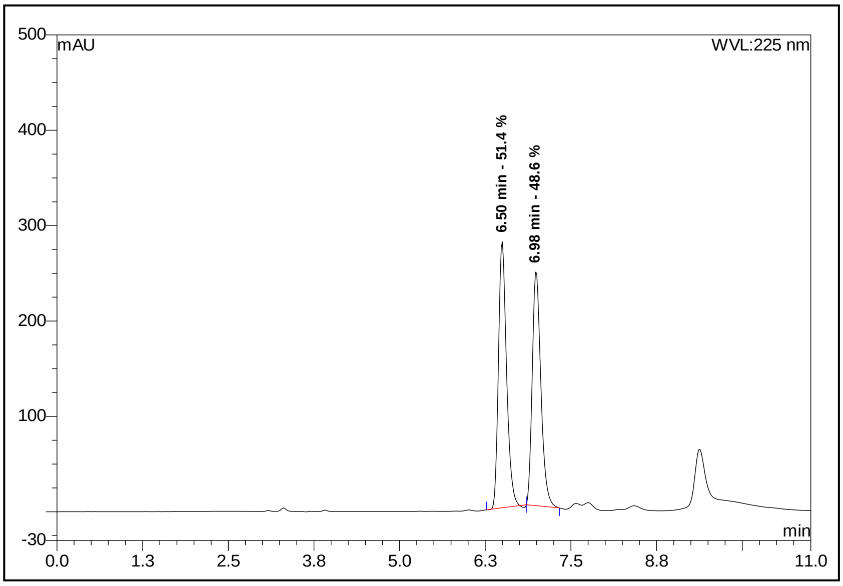

No.	Ret.Time min	Height mAU	Area mAU*min	Rel.Area %
1	6.50	278.786	37.982	51.41
2	6.98	244.889	35.904	48.59
**Total:**		523.674	73.886	100.00

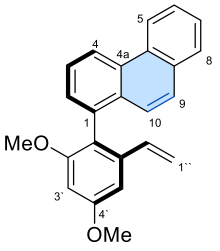

(R_a)-**16c**

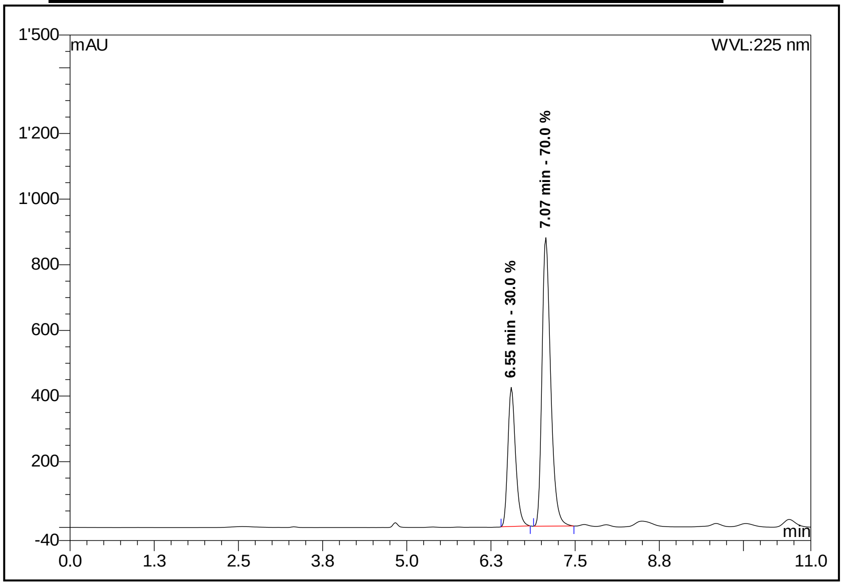

No.	Ret.Time min	Height mAU	Area mAU*min	Rel.Area %
1	6.55	423.759	56.370	30.02
2	7.07	879.139	131.389	69.98
**Total:**		1302.898	187.759	100.00

(S_a)-1-(2,4-Dimethoxy-6-vinylphenyl)-2-methoxyphenanthrene ((S_a)-16d):

HPLC conditions: Chiralcel IG (3 µm, 250×4.6 mm), heptane/*i*-PrOH 90 : 10, 1.0 mL/min, 20ºC

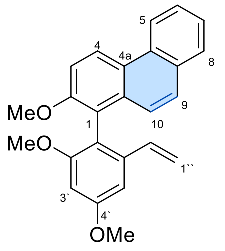

(±)-**16d**

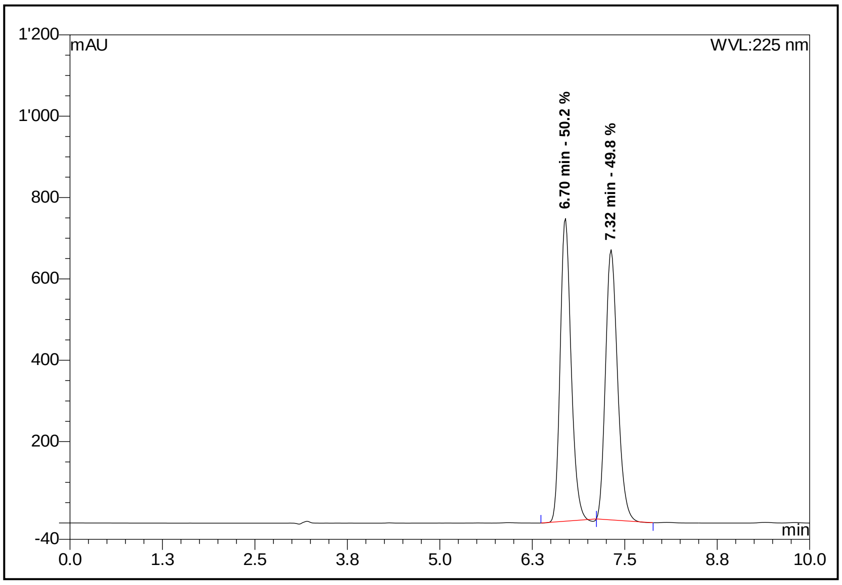

No.	Ret.Time min	Height mAU	Area mAU*min	Rel.Area %
1	6.70	744.348	126.821	50.19
2	7.32	664.322	125.858	49.81
**Total:**		1408.670	252.678	100.00

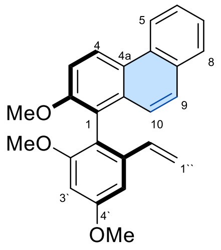

(S_a)-**16d**

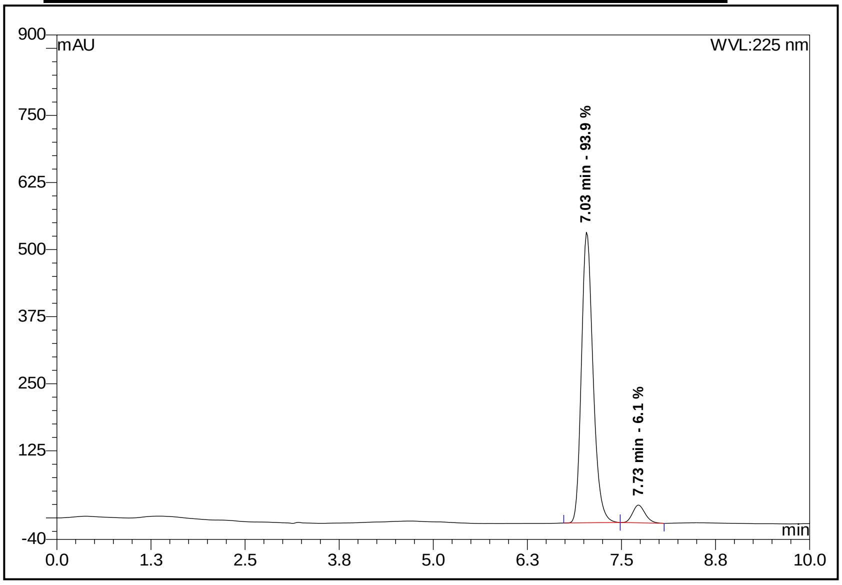

No.	Ret.Time min	Height mAU	Area mAU*min	Rel.Area %
1	7.03	541.560	96.897	93.86
2	7.73	32.901	6.336	6.14
**Total:**		574.461	103.232	100.00

(R_a)-1-(6-Methoxy-3-methyl-2-vinylphenyl)-2-methylphenanthrene ((R_a)-16e):

HPLC conditions: Chiralcel IG (3 µm, 250×4.6 mm), heptane/*i*-PrOH 97.5 : 2.5, 1.0 mL/min, 20ºC

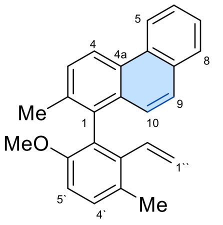

(±)-**16e**

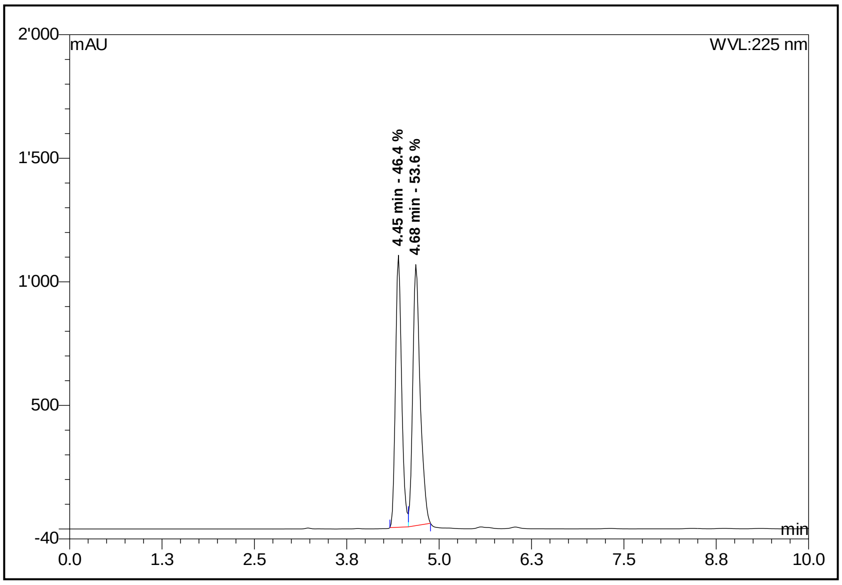

No.	Ret.Time min	Height mAU	Area mAU*min	Rel.Area %
1	4.45	1101.108	106.326	46.38
2	5.68	1056.595	122.908	53.62
**Total:**		1828.666	337.446	100.00

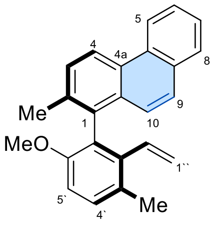

(R_a)-**16e**

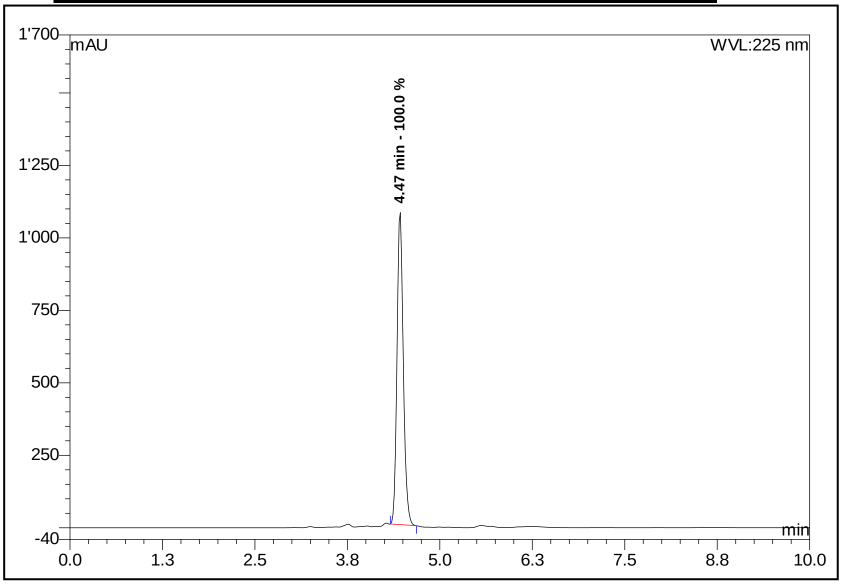

No.	Ret.Time min	Height mAU	Area mAU*min	Rel.Area %
1	4.47	1076.485	106.301	100.00
**Total:**		1076.485	106.301	100.00

(R_a)-1-(3,6-Dimethyl-2-vinylphenyl)-2-methoxyphenanthrene ((R_a)-16f):

HPLC conditions: Chiralcel IG (3 µm, 250×4.6 mm), heptane/*i*-PrOH 99 : 1, 1.0 mL/min, 20ºC

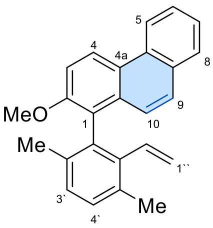

(±)-**16f**

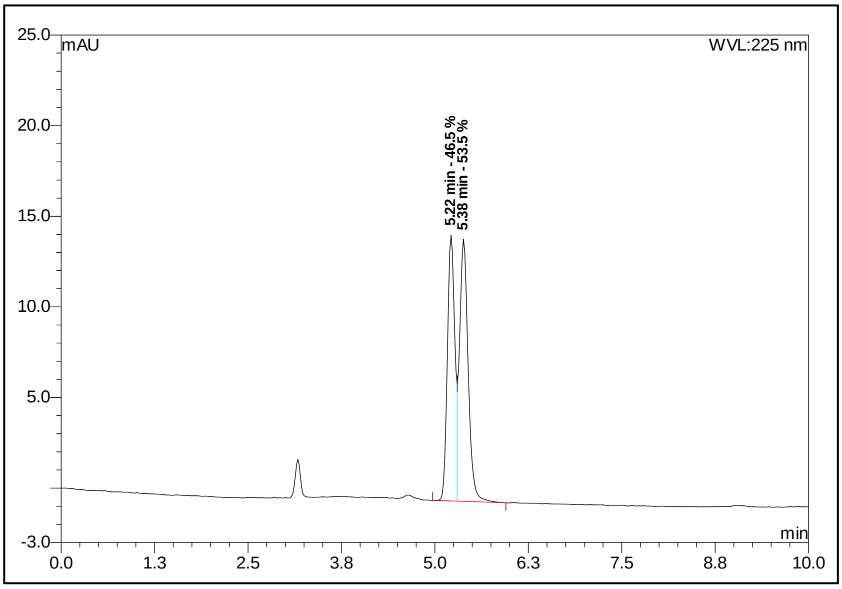

No.	Ret.Time min	Height mAU	Area mAU*min	Rel.Area %
1	5.22	14.668	1.688	46.50
2	5.38	14.463	1.942	53.50
**Total:**		29.131	3.630	100.00

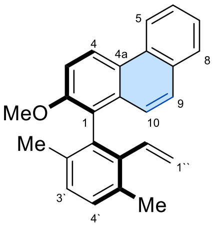

(R_a)-**16f**

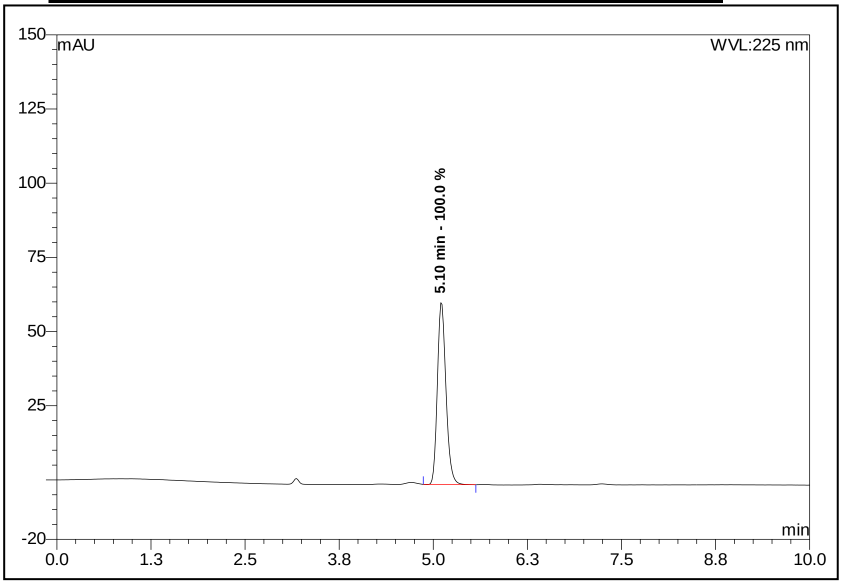

No.	Ret.Time min	Height mAU	Area mAU*min	Rel.Area %
1	5.10	61.268	8.126	100.00
**Total:**		61.268	8.126	100.00

(R_a)-6-Chloro-1-(2,4-dimethoxy-6-vinylphenyl)-2-methylphenanthrene ((R_a)-16g):

HPLC conditions: Chiralcel IG (3 µm, 250×4.6 mm), heptane/*i*-PrOH 97.5 : 2.5, 1.0 mL/min, 20ºC

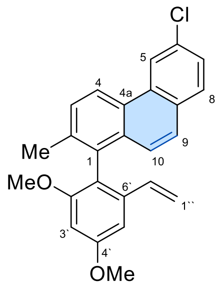

(±)-**16g**

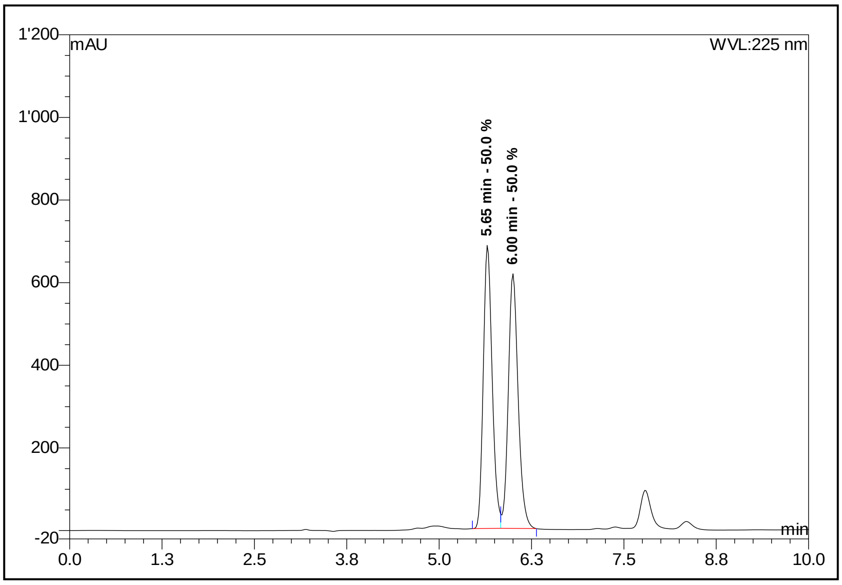

No.	Ret.Time min	Height mAU	Area mAU*min	Rel.Area %
1	5.65	685.091	95.527	50.05
2	6.00	616.242	95.349	49.95
**Total:**		1301.333	190.876	100.00

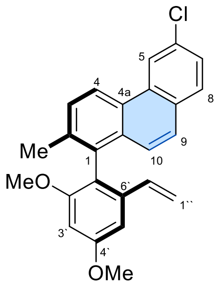

(R_a)-**16g**

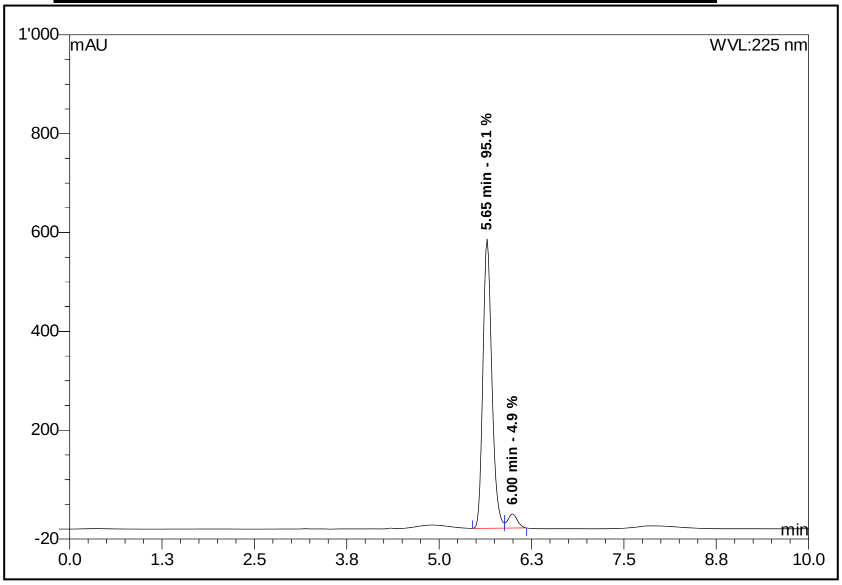

No.	Ret.Time min	Height mAU	Area mAU*min	Rel.Area %
1	5.65	584.750	82.191	95.06
2	6.00	28.279	4.275	4.94
**Total:**		613.029	86.465	100.00

(S_a)-6-Chloro-2-methoxy-1-(2-methoxy-6-vinylphenyl)phenanthrene ((S_a)-16h):

HPLC conditions: Chiralcel IG (3 µm, 250×4.6 mm), heptane/*i*-PrOH 90 : 10, 1.0 mL/min, 20ºC

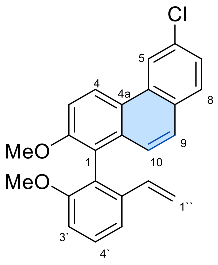

(±)-**16h**

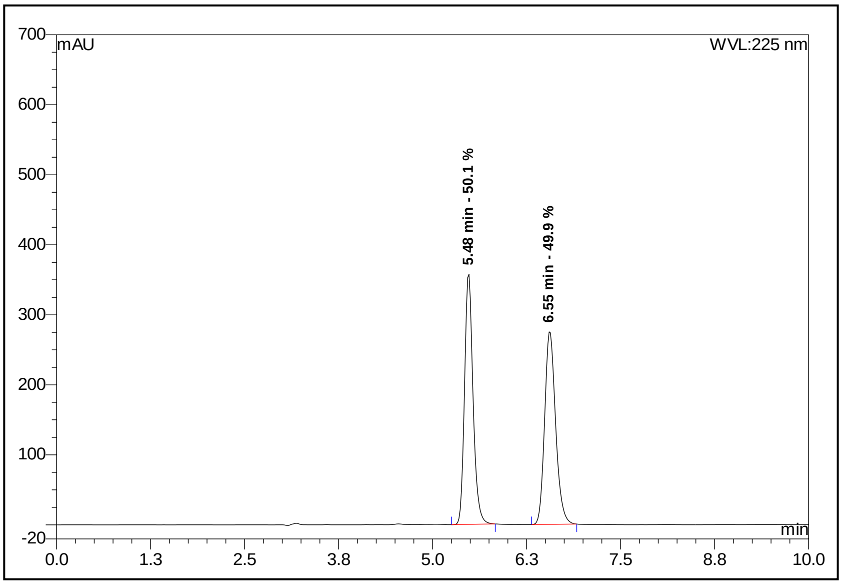

No.	Ret.Time min	Height mAU	Area mAU*min	Rel.Area %
1	5.48	357.042	46.935	50.15
2	6.55	275.174	46.657	49.85
**Total:**		632.216	93.593	100.00

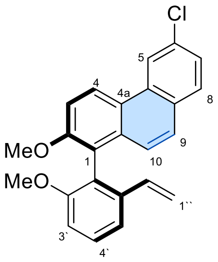

(S_a)-**16h**

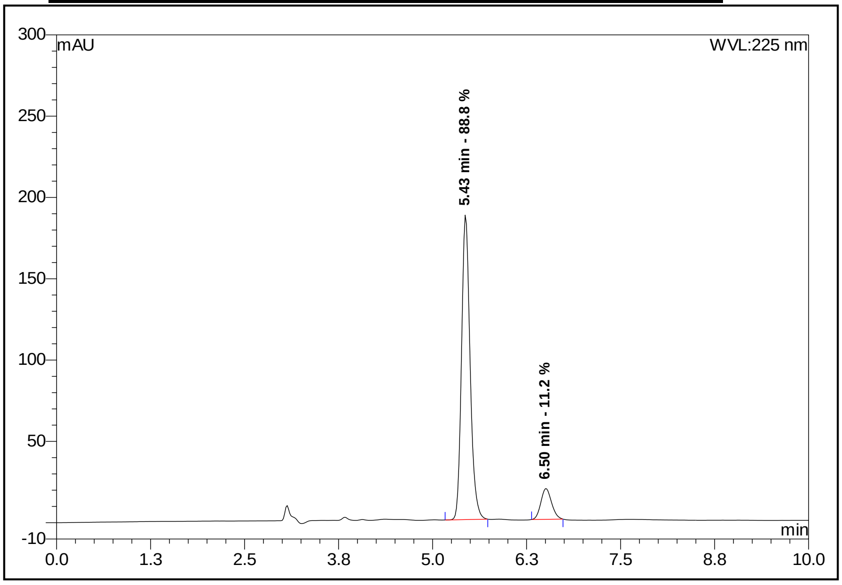

No.	Ret.Time min	Height mAU	Area mAU*min	Rel.Area %
1	5.43	187.261	24.695	88.83
2	6.50	18.878	3.106	11.17
**Total:**		206.139	27.801	100.00

(S_a)-1-(2,4-Dimethyl-6-vinylphenyl)-2-methylphenanthrene ((S_a)-16i):

HPLC conditions: Chiralcel IG (3 µm, 250×4.6 mm), heptane/*i*-PrOH 99.5 : 0.5, 1.0 mL/min, 40ºC

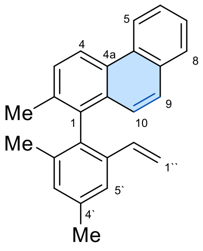

(±)-**16i**

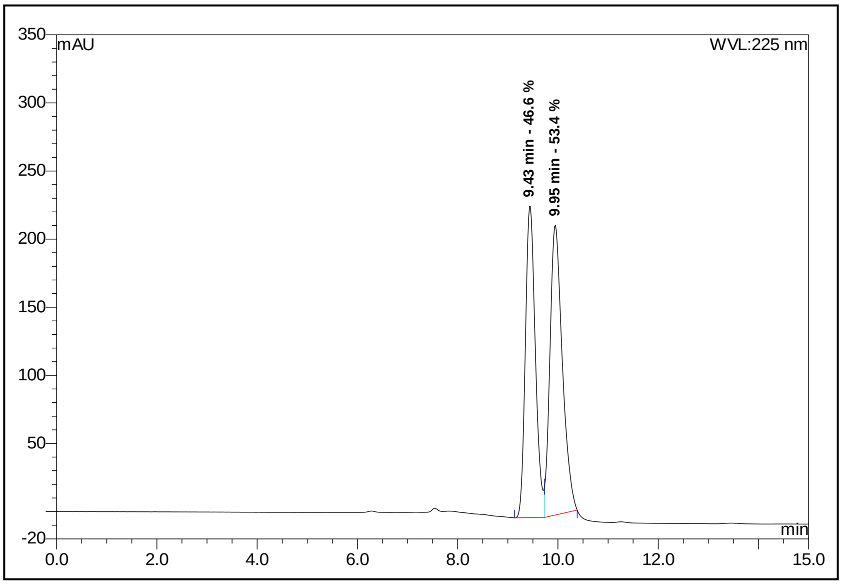

No.	Ret.Time min	Height mAU	Area mAU*min	Rel.Area %
1	9.43	228.526	52.939	46.57
2	9.95	212.699	60.735	53.43
**Total:**		441.225	113.673	100.00

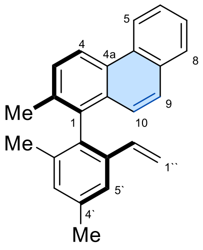

(S_a)-**16i**

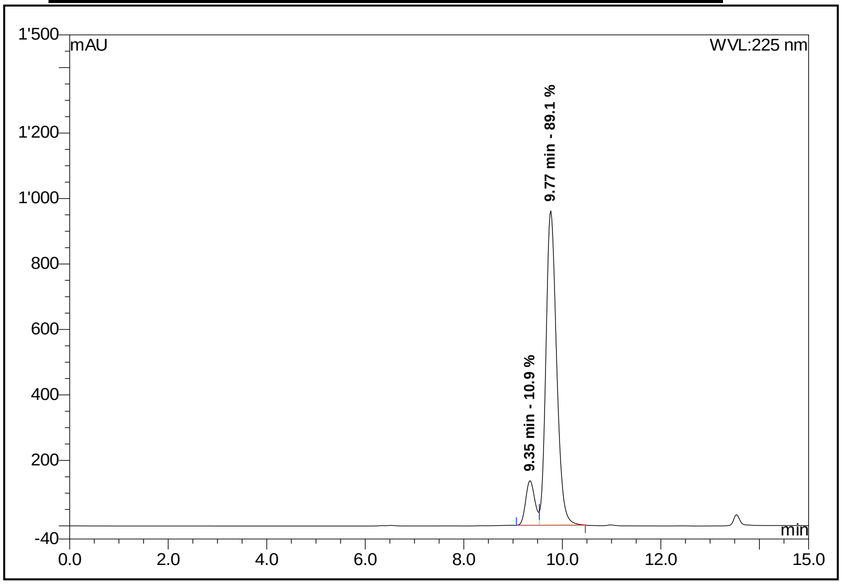

No.	Ret.Time min	Height mAU	Area mAU*min	Rel.Area %
1	9.35	135.957	29.409	10.90
2	9.77	960.388	240.383	89.10
**Total:**		1096.345	269.792	100.00

(S_a)-1-(3-Bromo-6-methoxy-2-vinylphenyl)-2-methoxyphenanthrene ((S_a)-16j):

HPLC conditions: Chiralcel IG (3 µm, 250×4.6 mm), heptane/*i*-PrOH 97.5 : 2.5, 1.0 mL/min, 20ºC

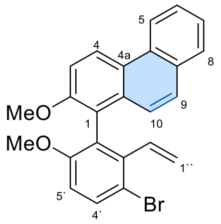

(±)-**16j**

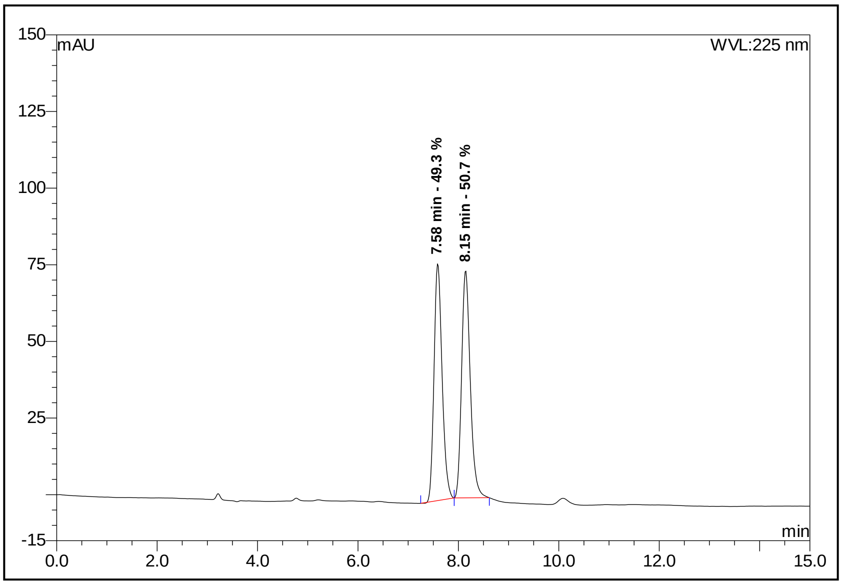

No.	Ret.Time<br>min	Height<br>mAU	Area<br>mAU*min	Rel.Area<br>%
1	7.58	77.230	14.428	49.27
2	8.15	73.981	14.856	50.73
**Total:**		151.211	29.283	100.00

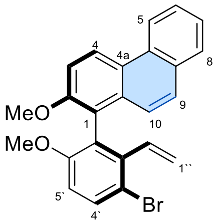

(S_a)-**16j**

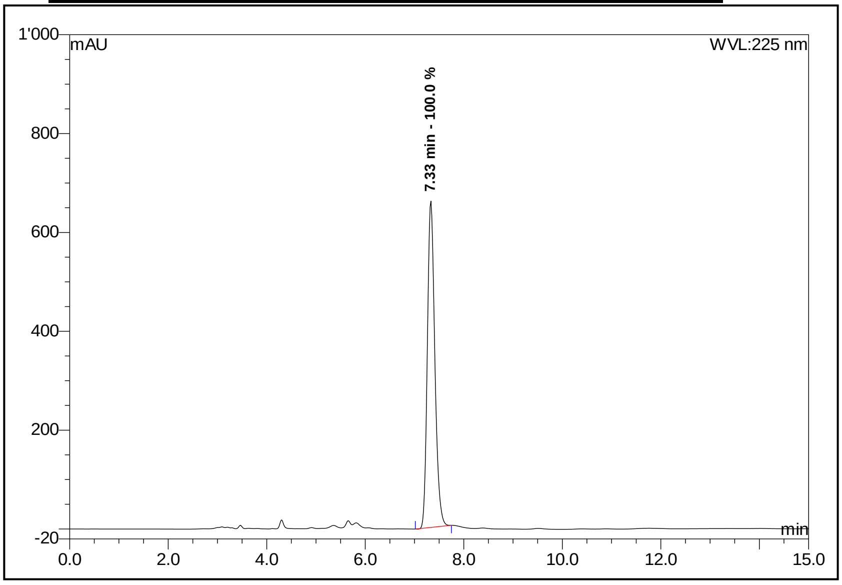

No.	Ret.Time<br>min	Height<br>mAU	Area<br>mAU*min	Rel.Area<br>%
1	7.33	660.290	115.707	100.00
**Total:**		660.290	115.707	100.00

(S_a)-1-(3-Bromo-6-methoxy-2-vinylphenyl)-6-chloro-2-methoxyphenanthrene ((S_a)-16k):

HPLC conditions: Chiralcel IG (3 µm, 250×4.6 mm), heptane/*i*-PrOH 90 : 10, 1.0 mL/min, 20ºC

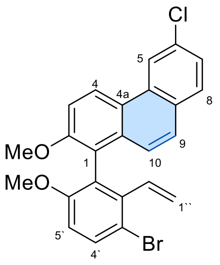

(±)-**16k**

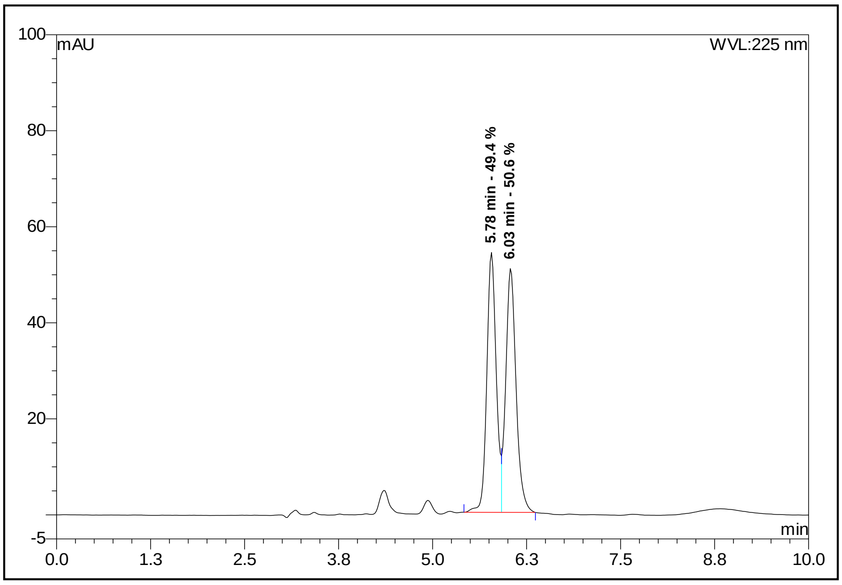

No.	Ret.Time min	Height mAU	Area mAU*min	Rel.Area %
1	5.78	54.104	7.903	49.45
2	6.03	50.771	8.080	50.55
**Total:**		104.875	15.983	100.00

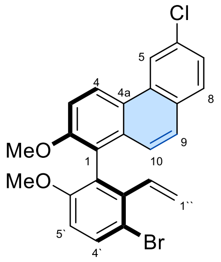

(S_a)-**16k**

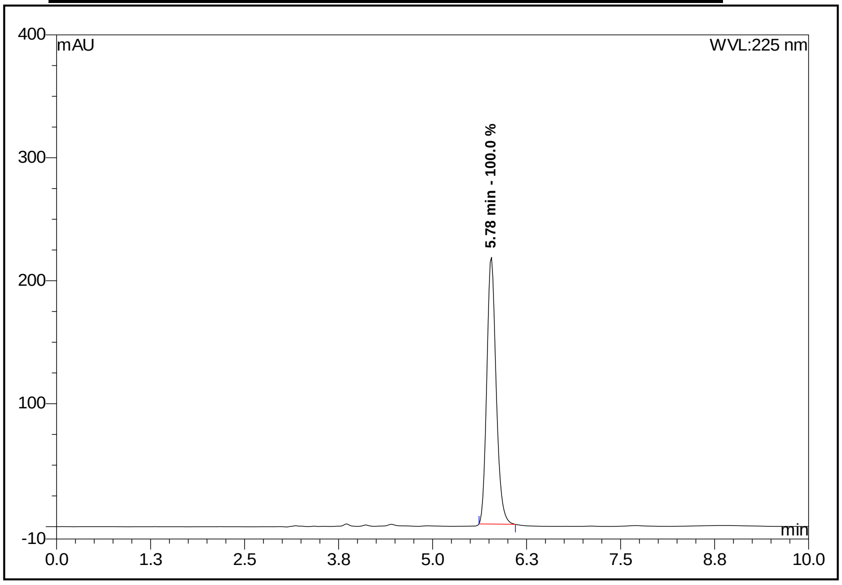

No.	Ret.Time min	Height mAU	Area mAU*min	Rel.Area %
1	5.78	216.893	31.186	100.00
**Total:**		216.893	31.186	100.00

(S_a)-2,2'-Dimethoxy-1,1'-biphenanthrene ((S_a)-18a):

HPLC conditions: Chiralcel IG (3 µm, 250×4.6 mm), heptane/*i*-PrOH 90.0 : 10.0, 1.0 mL/min, 20ºC

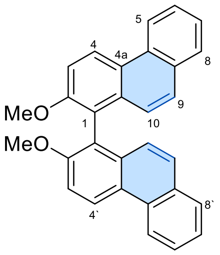

(±)-**18a**

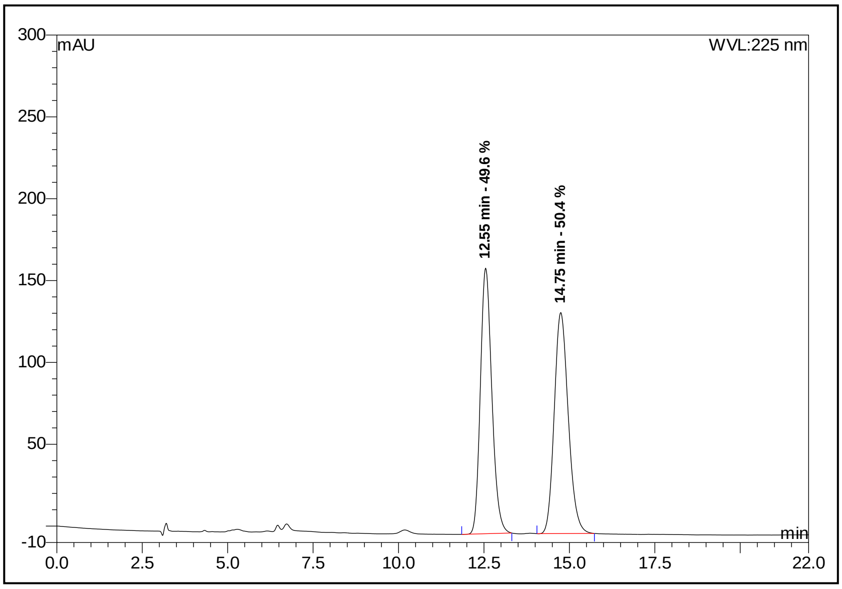

No.	Ret.Time min	Height mAU	Area mAU*min	Rel.Area %
1	12.55	162.269	63.215	49.57
2	14.75	135.020	64.323	50.43
**Total:**		297.288	127.537	100.00

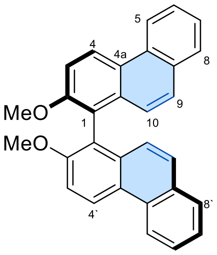

(S_a)-**18a**

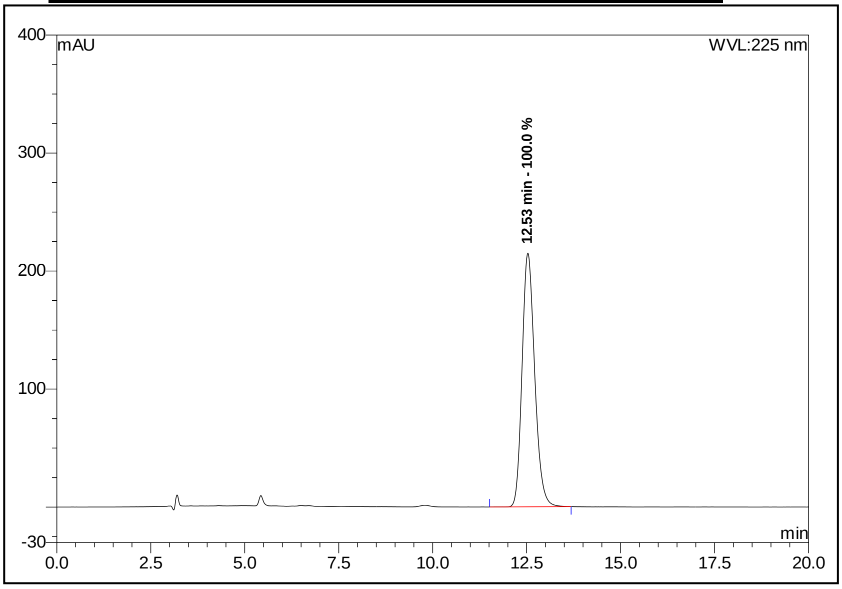

No.	Ret.Time min	Height mAU	Area mAU*min	Rel.Area %
1	12.53	215.002	83.727	100.00
**Total:**		215.002	83.727	100.00

(S_a)-7-(2-Methoxyphenanthren-1-yl)phenanthro[2,3-d][1,3]dioxole ((S_a)-18b):

HPLC conditions: Chiralcel IC(3 µm, 250×4.6 mm), heptane/*i*-PrOH 80.0 : 20.0, 1.0 mL/min, 20ºC

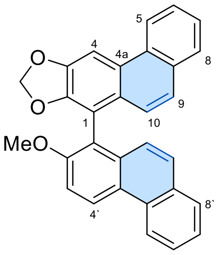

(±)-**18b**

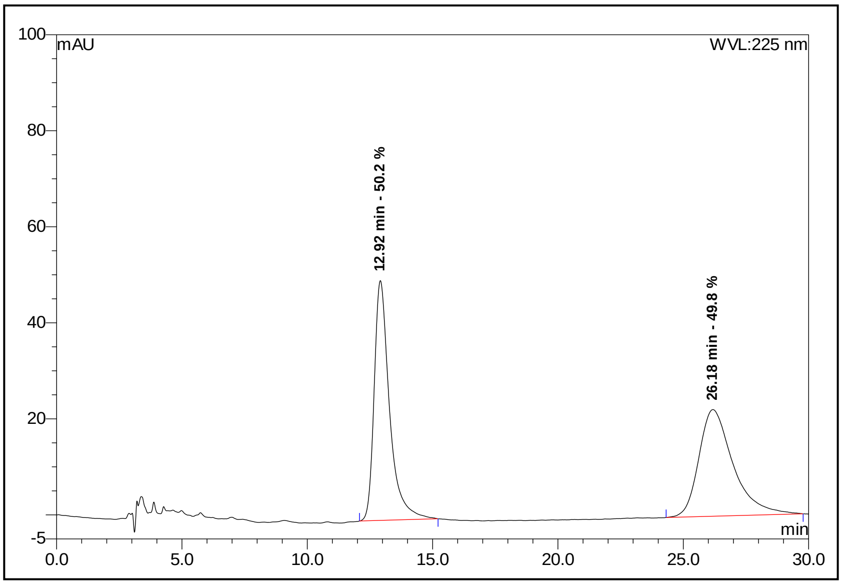

No.	Ret.Time min	Height mAU	Area mAU*min	Rel.Area %
1	12.92	49.950	35.429	50.16
2	26.18	22.209	35.199	49.84
**Total:**		72.159	70.629	100.00

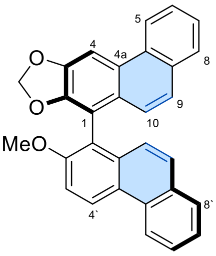

(S_a)-**18b**

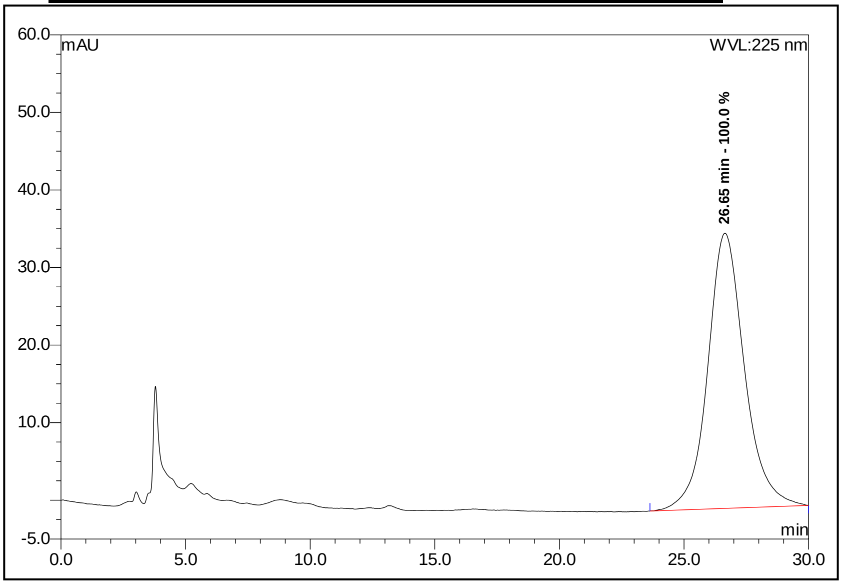

No.	Ret.Time min	Height mAU	Area mAU*min	Rel.Area %
1	26.65	35.473	60.640	100.00
**Total:**		35.473	60.640	100.00

(R_a)-2-Methoxy-2'-methyl-1,1'-biphenanthrene ((R_a)-18c):

HPLC conditions: Chiralcel IG (3 µm, 250×4.6 mm), heptane/*i*-PrOH 85.0 : 15.0, 1.0 mL/min, 20ºC

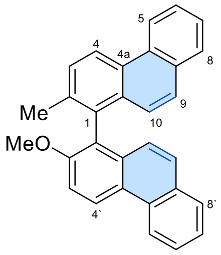

(±)-**18c**

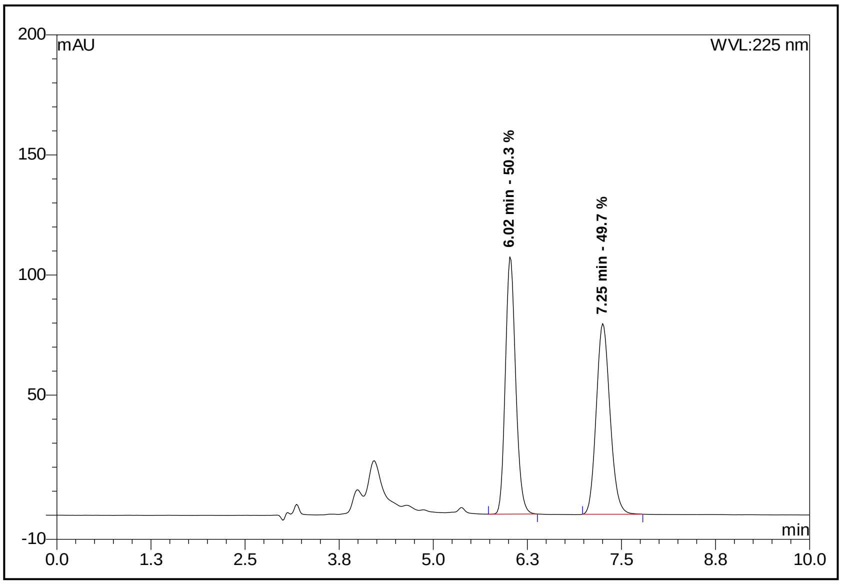

No.	Ret.Time min	Height mAU	Area mAU*min	Rel.Area %
1	6.02	107.163	17.032	50.26
2	7.25	79.422	16.855	49.74
**Total:**		186.585	33.887	100.00

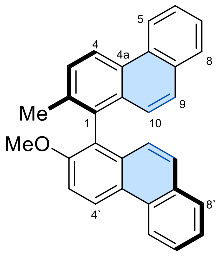

(R_a)-**18c**

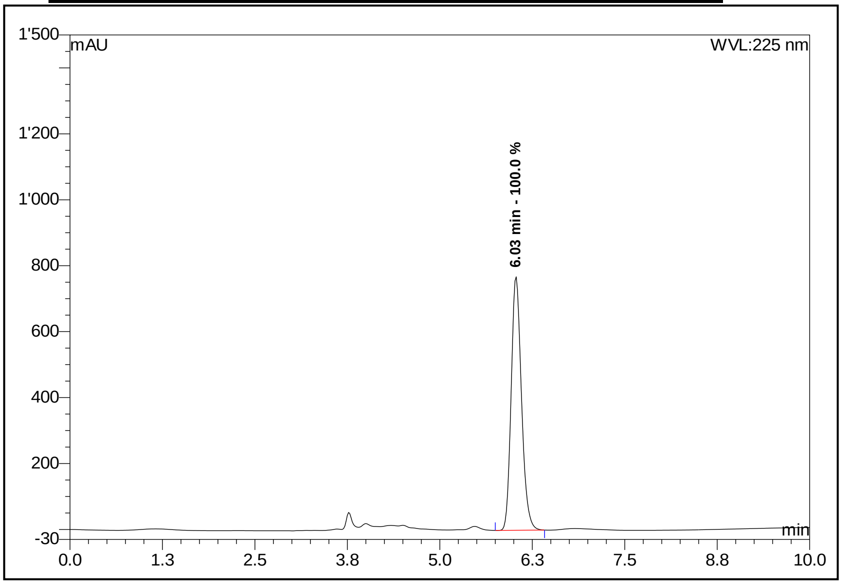

No.	Ret.Time min	Height mAU	Area mAU*min	Rel.Area %
1	6.03	768.645	124.112	100.00
**Total:**		768.645	124.112	100.00

(S_a)-2,2'-Dimethyl-1,1'-biphenanthrene ((S_a)-18d):

HPLC conditions: Chiralcel IG (3 μm, 250×4.6 mm), heptane/*i*-PrOH 99.0 : 1.0, 1.0 mL/min, 20ºC

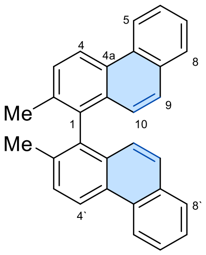

(±)-**18d**

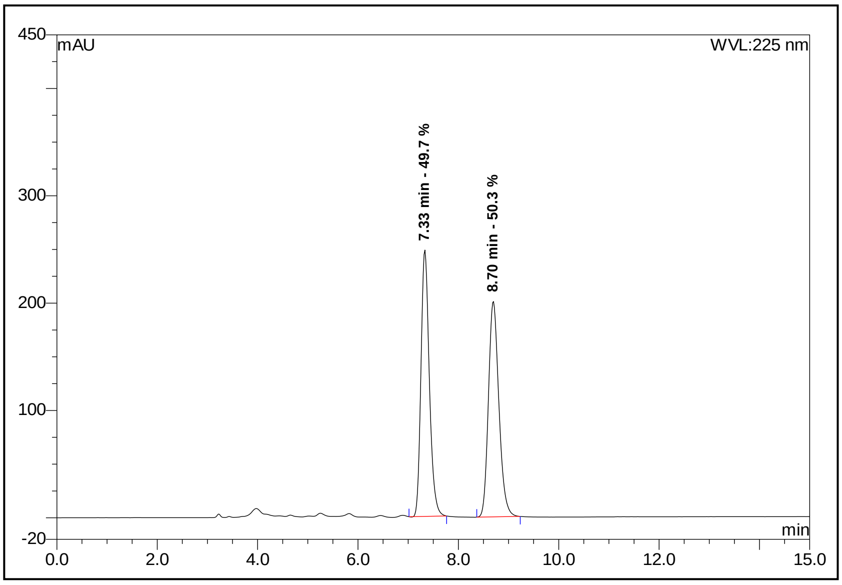

No.	Ret.Time min	Height mAU	Area mAU*min	Rel.Area %
1	7.33	248.202	47.773	49.70
2	8.70	200.761	48.355	50.30
**Total:**		448.963	96.128	100.00

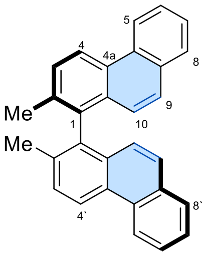

(S_a)-**18d**

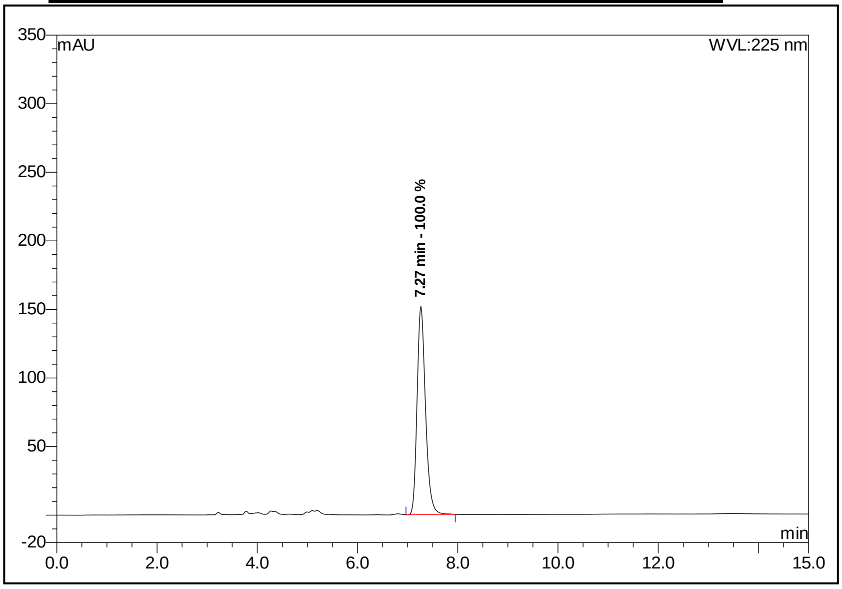

No.	Ret.Time min	Height mAU	Area mAU*min	Rel.Area %
1	7.27	151.703	29.581	100.00
**Total:**		151.703	29.581	100.00

(S_a)-6-Chloro-2,2'-dimethoxy-1,1'-biphenanthrene ((S_a)-18e):

HPLC conditions: Chiralcel IG (3 µm, 250×4.6 mm), heptane/*i*-PrOH 90.0 : 10.0, 1.0 mL/min, 20ºC

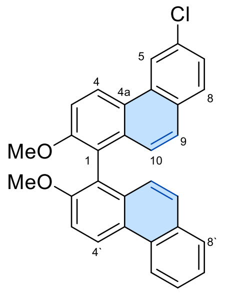

(±)-**18e**

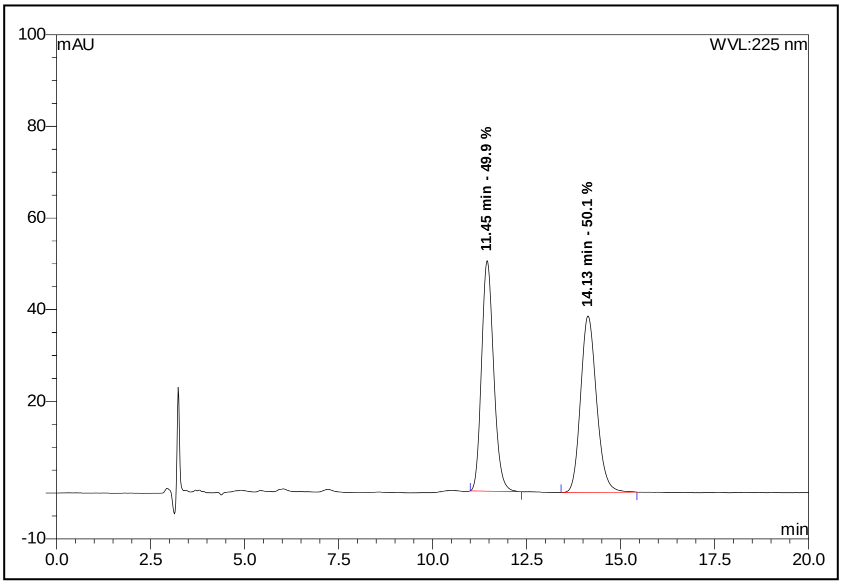

No.	Ret.Time min	Height mAU	Area mAU*min	Rel.Area %
1	11.45	50.311	18.846	49.87
2	14.13	38.520	18.943	50.13
**Total:**		88.831	37.788	100.00

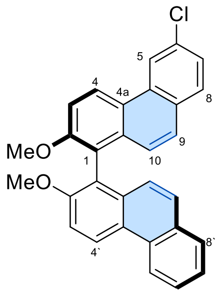

(S_a)-**18e**

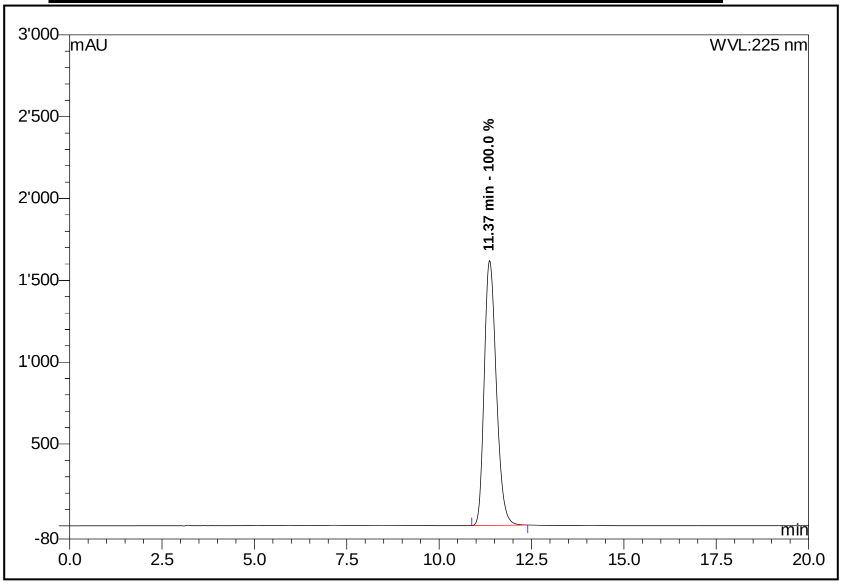

No.	Ret.Time min	Height mAU	Area mAU*min	Rel.Area %
1	11.37	1618.303	614.977	100.00
**Total:**		1618.303	614.977	100.00

(S_a)-6,6'-Difluoro-2,2'-dimethoxy-1,1'-biphenanthrene ((S_a)-18f):

HPLC conditions: Chiralcel IG (3 µm, 250×4.6 mm), heptane/*i*-PrOH 90.0 : 10.0, 1.0 mL/min, 20ºC

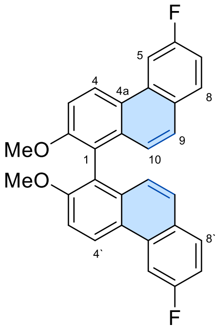

(±)-**18f**

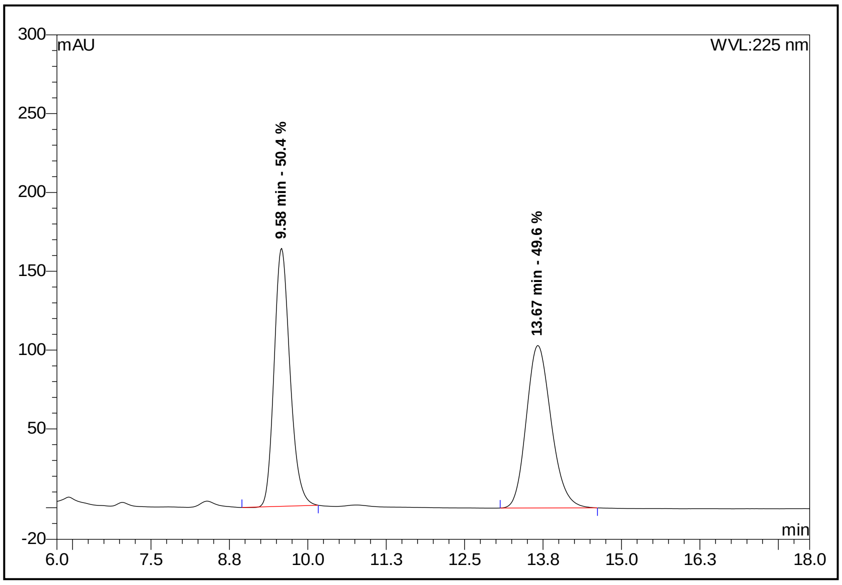

No.	Ret.Time min	Height mAU	Area mAU*min	Rel.Area %
1	9.58	163.713	48.161	50.40
2	13.67	103.096	47.398	49.60
**Total:**		266.808	95.559	100.00

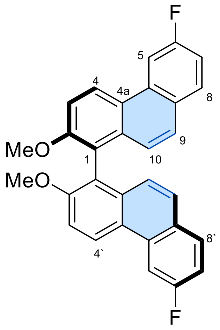

(S_a)-**18f**

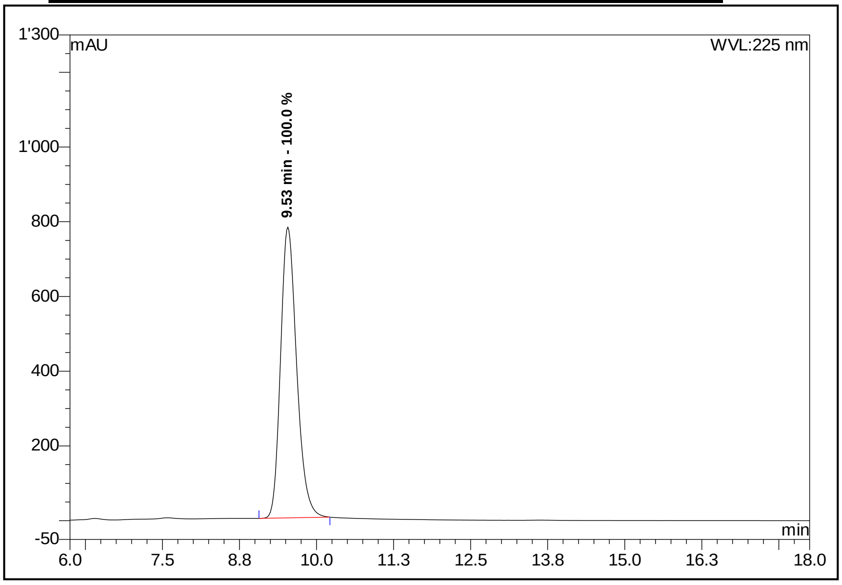

No.	Ret.Time min	Height mAU	Area mAU*min	Rel.Area %
1	9.53	778.435	235.581	100.00
**Total:**		778.435	235.581	100.00

(R_a)-2,6,6'-Trimethoxy-2'-methyl-1,1'-biphenanthrene ((S_a)-18g):

HPLC conditions: Chiralcel IG (3 µm, 250×4.6 mm), heptane/*i*-PrOH 90.0 : 10.0, 1.0 mL/min, 20ºC

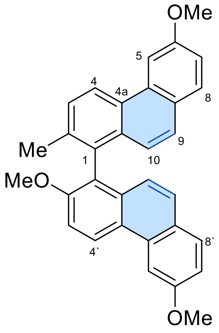

(±)-**18g**

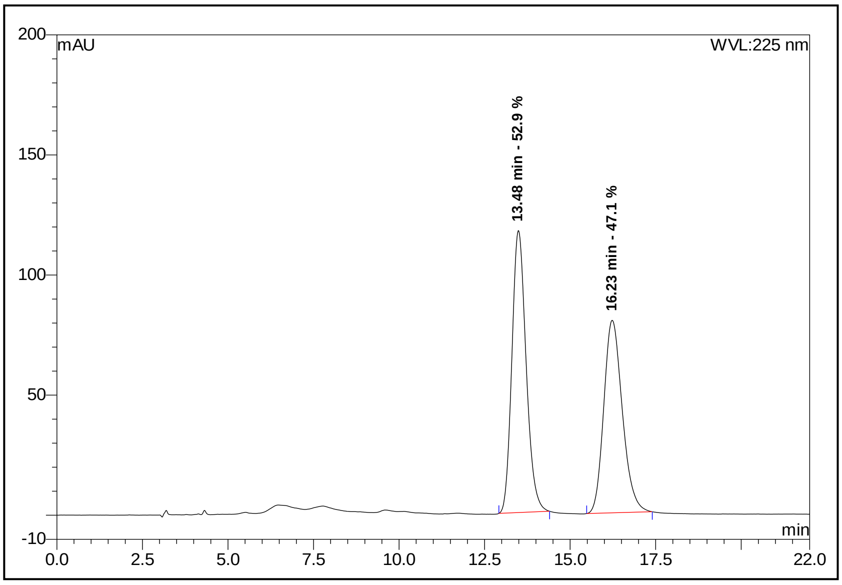

No.	Ret.Time min	Height mAU	Area mAU*min	Rel.Area %
1	13.48	117.392	56.535	52.88
2	16.23	80.175	50.377	47.12
**Total:**		197.567	106.911	100.00

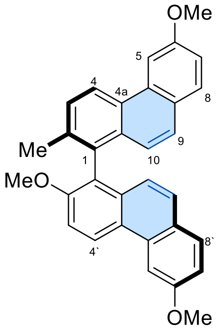

(R_a)-**18g**

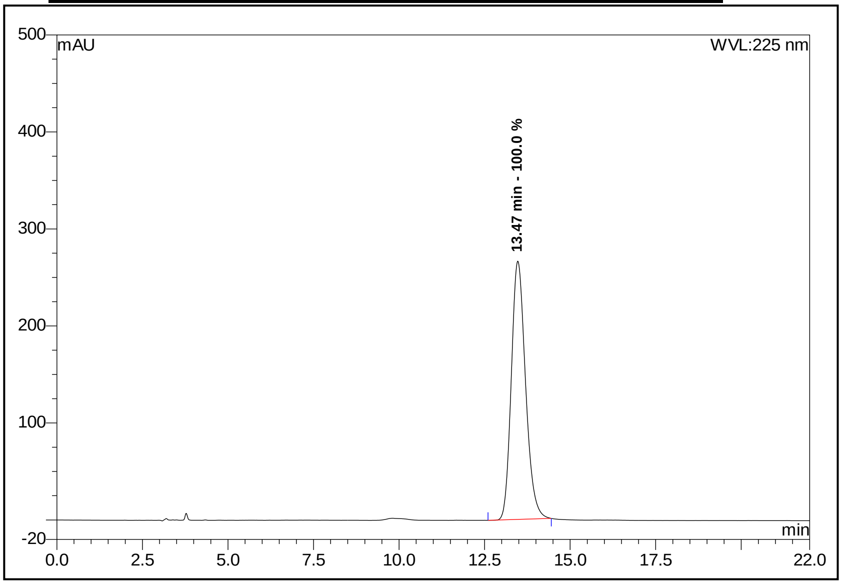

No.	Ret.Time min	Height mAU	Area mAU*min	Rel.Area %
1	13.47	266.271	128.323	100.00
**Total:**		266.271	128.323	100.00

(S_a)-2,2'-Dimethoxy-6,6'-bis(trifluoromethyl)-1,1'-biphenanthrene ((S_a)-18h):

HPLC conditions: Chiralcel IG (3 μm, 250×4.6 mm), heptane/*i*-PrOH 90.0 : 10.0, 1.0 mL/min, 20ºC

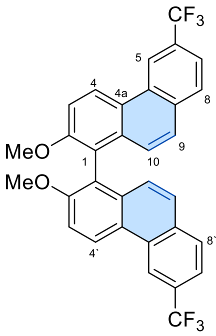

(±)-**18h**

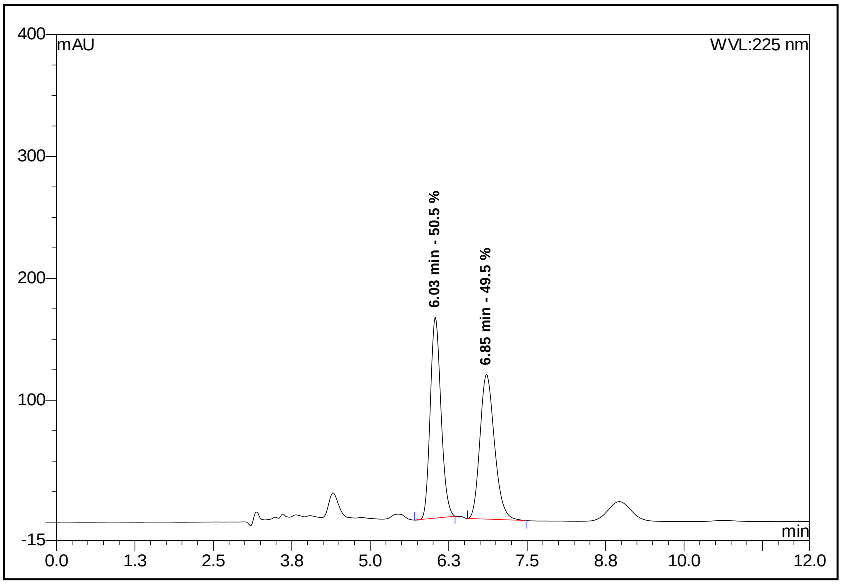

No.	Ret.Time min	Height mAU	Area mAU*min	Rel.Area %
1	6.03	164.964	32.500	50.48
2	6.85	118.816	31.879	49.52
**Total:**		283.780	64.379	100.00

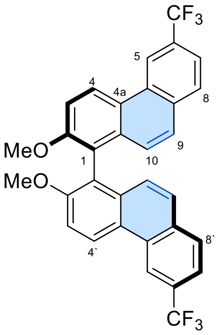

(S_a)-**18h**

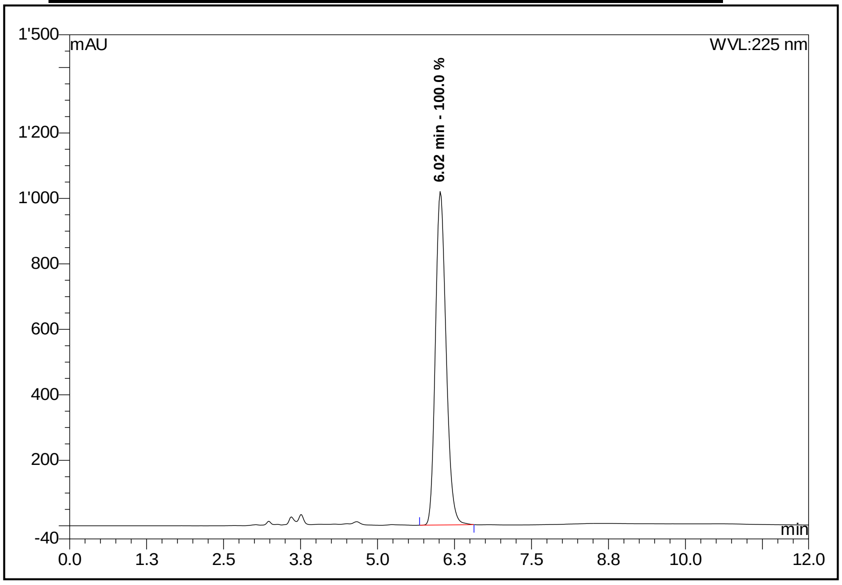

No.	Ret.Time min	Height mAU	Area mAU*min	Rel.Area %
1	6.02	1018.776	205.499	100.00
**Total:**		1018.776	205.499	100.00

(S_a)-6,6'-Dichloro-2,2'-dimethoxy-1,1'-biphenanthrene ((S_a)-18i):

HPLC conditions: Chiralcel IG (3 µm, 250×4.6 mm), heptane/*i*-PrOH 90.0 : 10.0, 1.0 mL/min, 20ºC

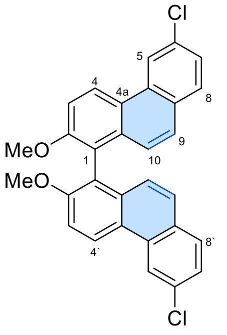

(±)-**18i**

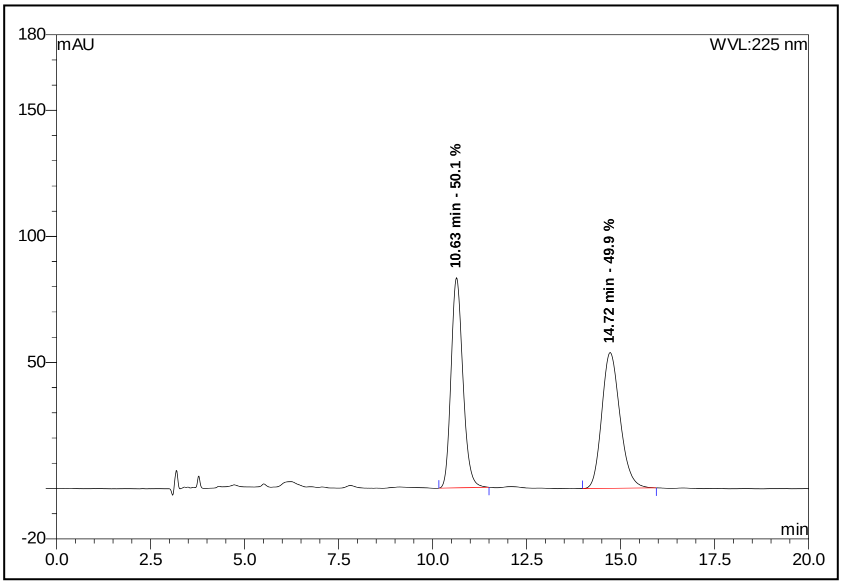

No.	Ret.Time min	Height mAU	Area mAU*min	Rel.Area %
1	10.63	83.409	30.460	50.06
2	14.72	53.848	30.392	49.94
**Total:**		137.256	60.852	100.00

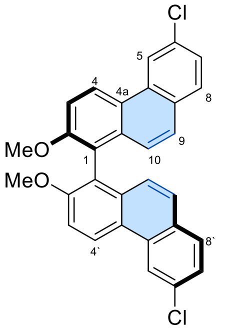

(S_a)-**18i**

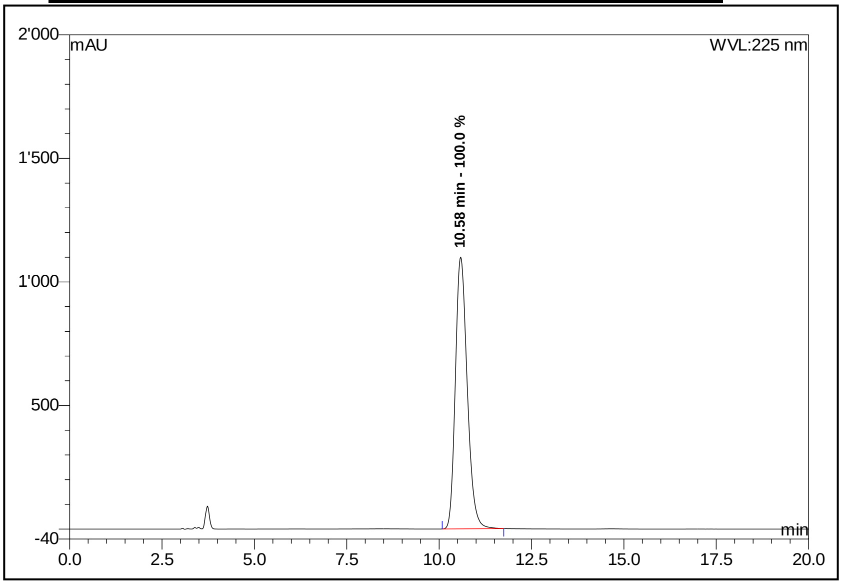

No.	Ret.Time min	Height mAU	Area mAU*min	Rel.Area %
1	10.58	1099.953	409.374	100.00
**Total:**		1099.953	409.374	100.00

(R_a)-2-Methyl-1-(phenanthren-1-yl)-1*H*-indole ((R_a)-20a):

HPLC conditions: Chiralcel IG (3 μm, 250×4.6 mm), heptane/*i*-PrOH 97.5 : 2.5, 1.0 mL/min, 20ºC

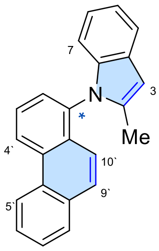

(±)-**20a**

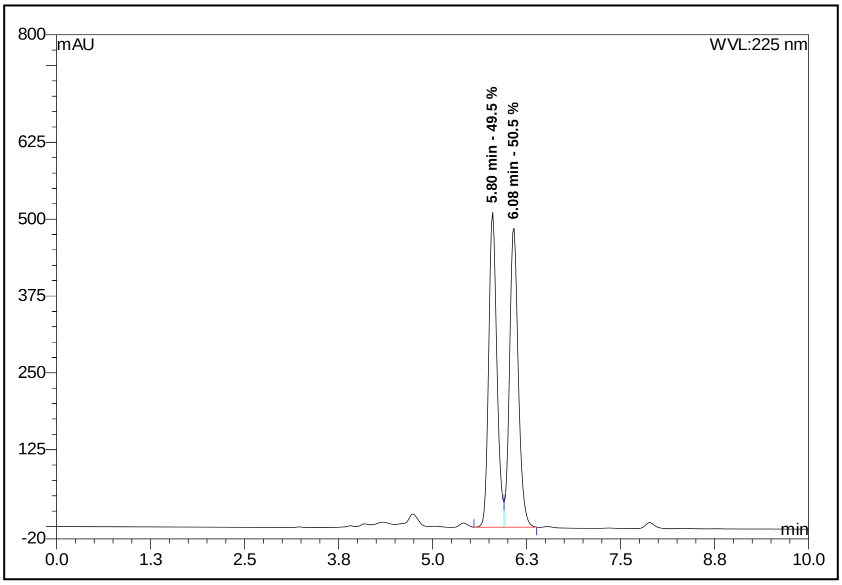

No.	Ret.Time min	Height mAU	Area mAU*min	Rel.Area %
1	5.80	512.000	64.653	49.47
2	6.08	486.341	66.038	50.53
**Total:**		998.341	130.692	100.00

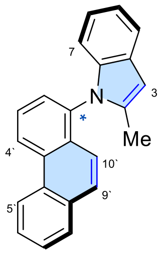

(R_a)-**20a**

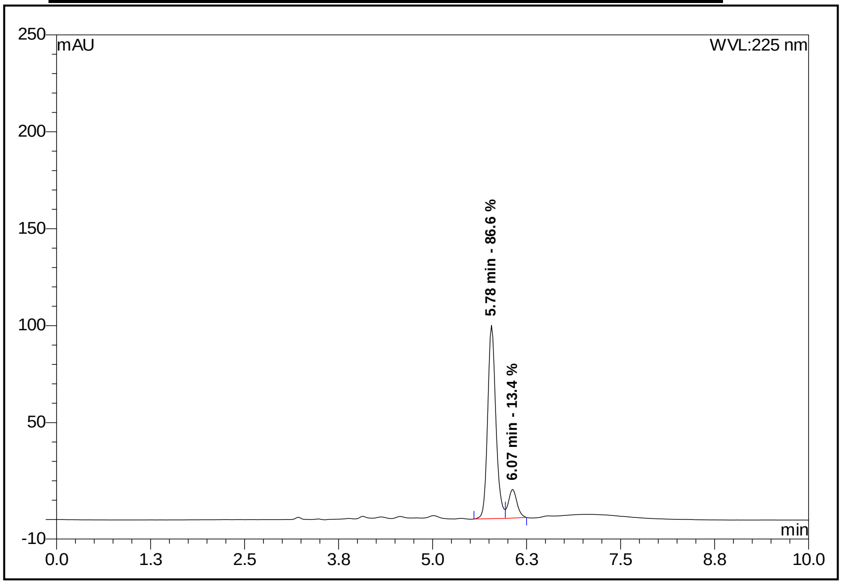

No.	Ret.Time min	Height mAU	Area mAU*min	Rel.Area %
1	5.78	99.721	12.627	86.61
2	6.07	14.810	1.952	13.39
**Total:**		114.532	14.579	100.00

(S_a)-2-Methyl-1,4-bis(2-vinylphenyl)-1*H*-indole ((S_a)-19a):

HPLC conditions: Chiralcel IG (3 µm, 250×4.6 mm), heptane/*i*-PrOH 97.5 : 2.5, 1.0 mL/min, 20ºC

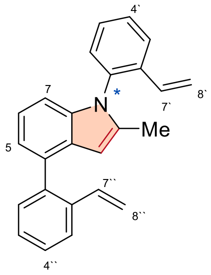

(±)-**19a**

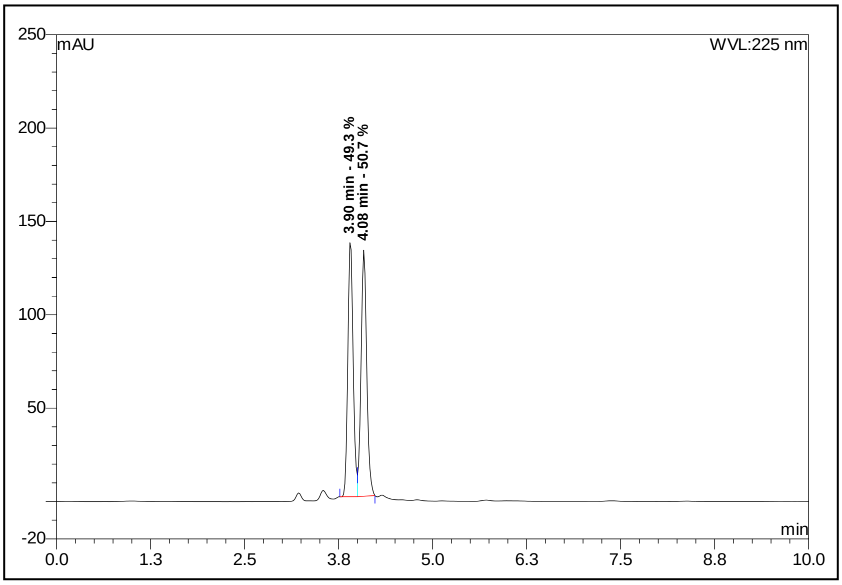

No.	Ret.Time min	Height mAU	Area mAU*min	Rel.Area %
1	3.90	136.041	11.228	49.29
2	4.08	131.821	11.551	50.71
**Total:**		267.862	22.778	100.00

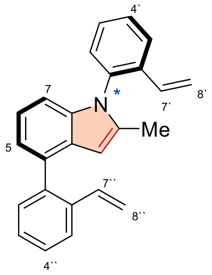

(S_a)-**19a**

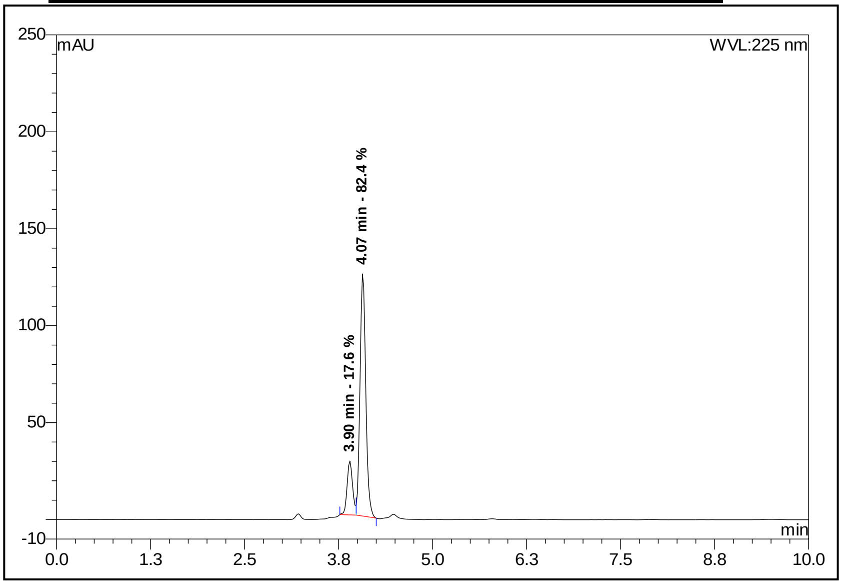

No.	Ret.Time min	Height mAU	Area mAU*min	Rel.Area %
1	3.90	27.817	2.361	17.57
2	4.07	124.892	11.078	82.43
**Total:**		152.709	13.439	100.00

(S_a)-1-(2-Methoxyphenanthren-1-yl)-1H-indole ((S_a)-20b):

HPLC conditions: Chiralcel IB (3 µm, 250×4.6 mm), heptane/i-PrOH 97.5 : 2.5, 1.0 mL/min, 20ºC

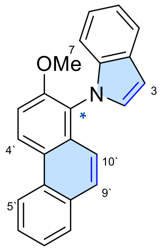

(±)-**20b**

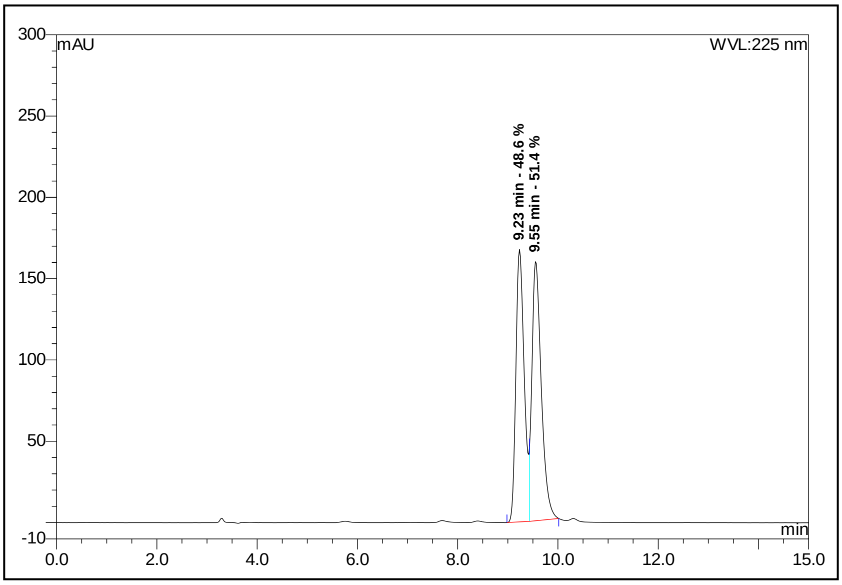

No.	Ret.Time min	Height mAU	Area mAU*min	Rel.Area %
1	9.23	167.459	32.482	48.59
2	9.55	159.365	34.369	51.41
**Total:**		326.824	66.851	100.00

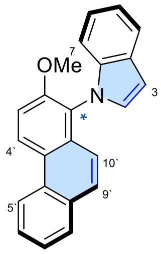

(S_a)-**20b**

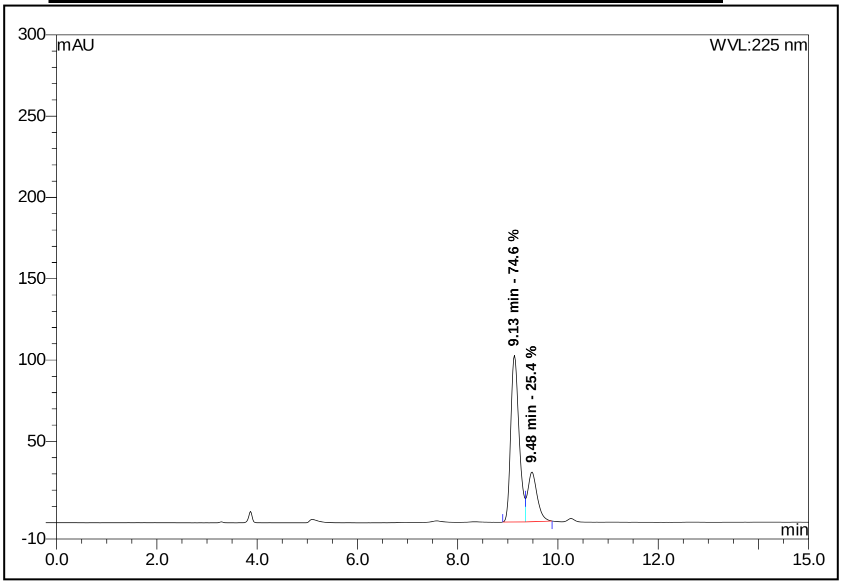

No.	Ret.Time min	Height mAU	Area mAU*min	Rel.Area %
1	9.13	102.544	19.732	74.61
2	9.48	30.523	6.716	25.39
**Total:**		133.067	26.448	100.00

(R_a)-1-(2-Methylphenanthren-1-yl)-1H-indole ((R_a)-20c):

HPLC conditions: Chiralcel IG (3 µm, 250×4.6 mm), heptane/i-PrOH 96.0 : 4.0, 1.0 mL/min, 20ºC

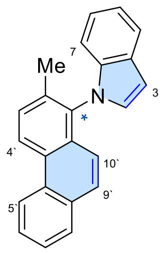

(±)-**20c**

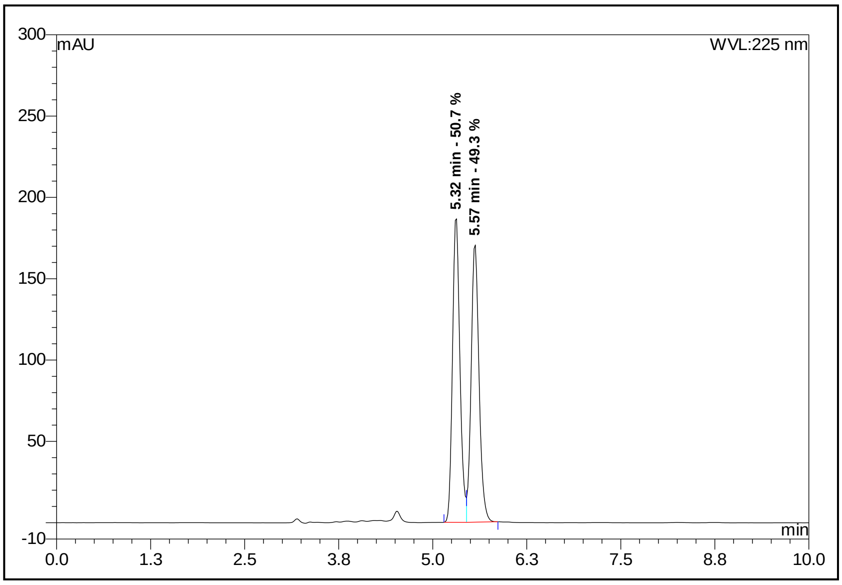

No.	Ret.Time min	Height mAU	Area mAU*min	Rel.Area %
1	5.32	186.610	21.401	50.73
2	5.57	170.463	20.786	49.27
**Total:**		357.073	42.186	100.00

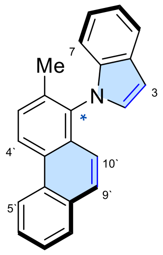

(R_a)-**20c**

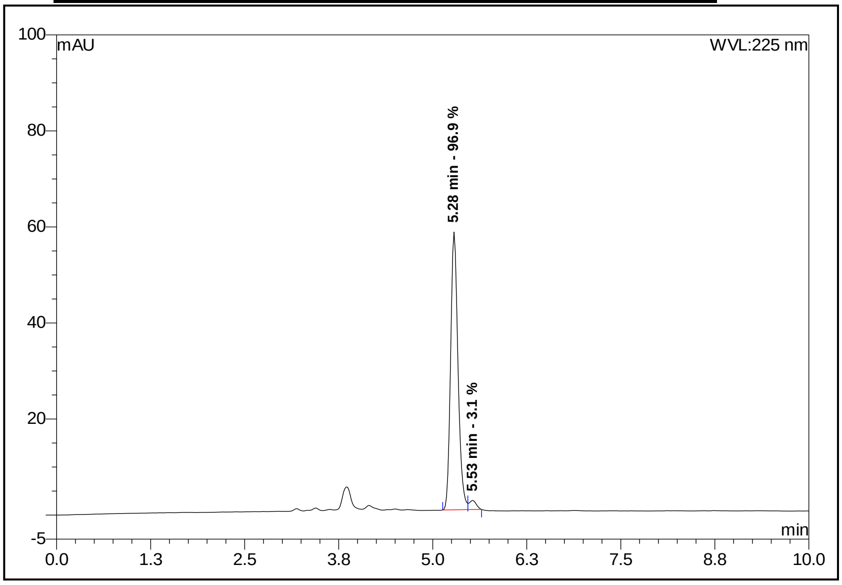

No.	Ret.Time min	Height mAU	Area mAU*min	Rel.Area %
1	5.28	57.851	6.599	96.92
2	5.53	1.911	0.210	3.08
**Total:**		59.762	6.809	100.00

(S_a)-1-(2-Chlorophenanthren-1-yl)-1*H*-indole ((S_a)-20d):

HPLC conditions: Chiralcel IG (3 µm, 250×4.6 mm), heptane/*i*-PrOH 99.0 : 1.0, 1.0 mL/min, 20°C

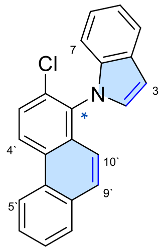

(±)-**20d**

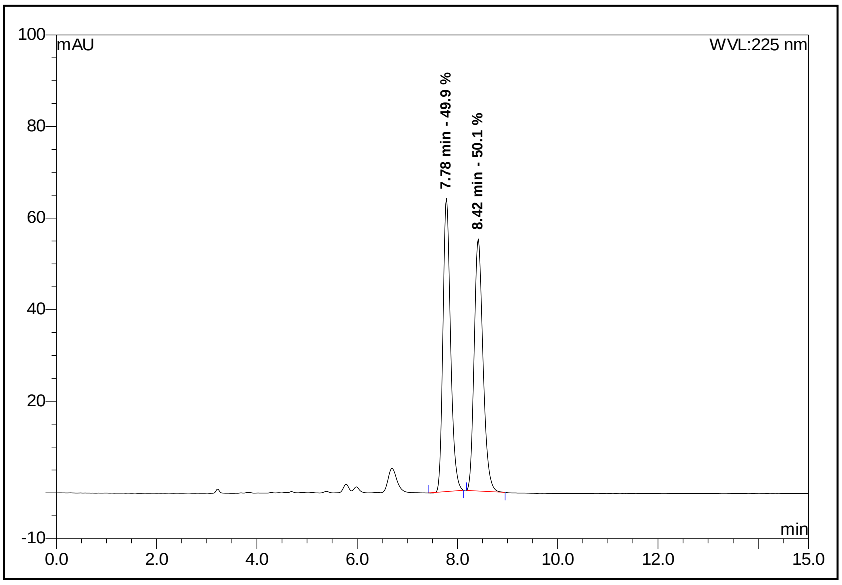

No.	Ret.Time<br>min	Height<br>mAU	Area<br>mAU*min	Rel.Area<br>%
1	7.78	64.011	11.224	49.94
2	8.42	55.070	11.249	50.06
**Total:**		119.081	22.473	100.00

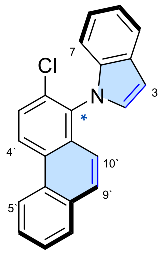

(S_a)-**20d**

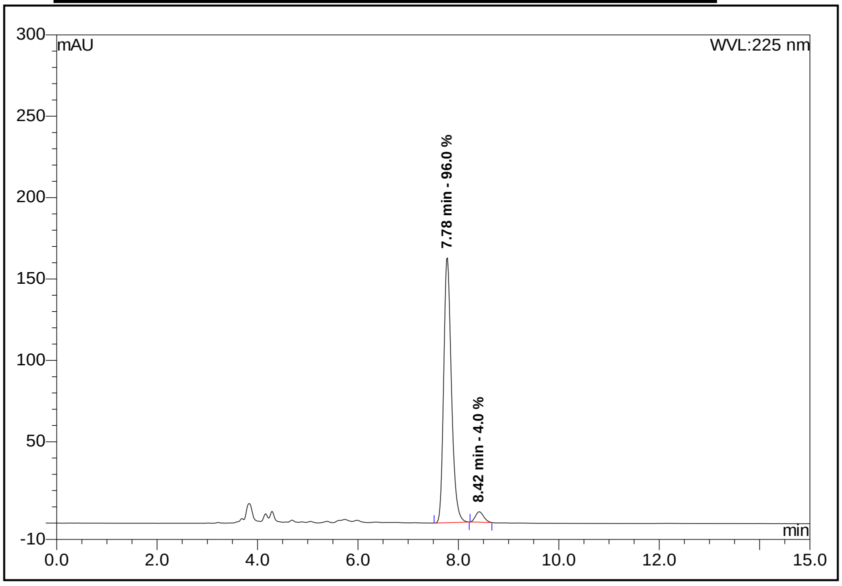

No.	Ret.Time<br>min	Height<br>mAU	Area<br>mAU*min	Rel.Area<br>%
1	7.78	162.725	29.061	96.00
2	8.42	6.398	1.210	4.00
**Total:**		169.123	30.271	100.00

(S_a)-1-(2,6-Dichlorophenanthren-1-yl)-1*H*-indole ((S_a)-20e):

HPLC conditions: Chiralcel IB (3 µm, 250×4.6 mm), heptane/*i*-PrOH 97.5 : 2.5, 1.0 mL/min, 20ºC

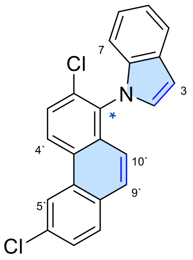

(±)-**20e**

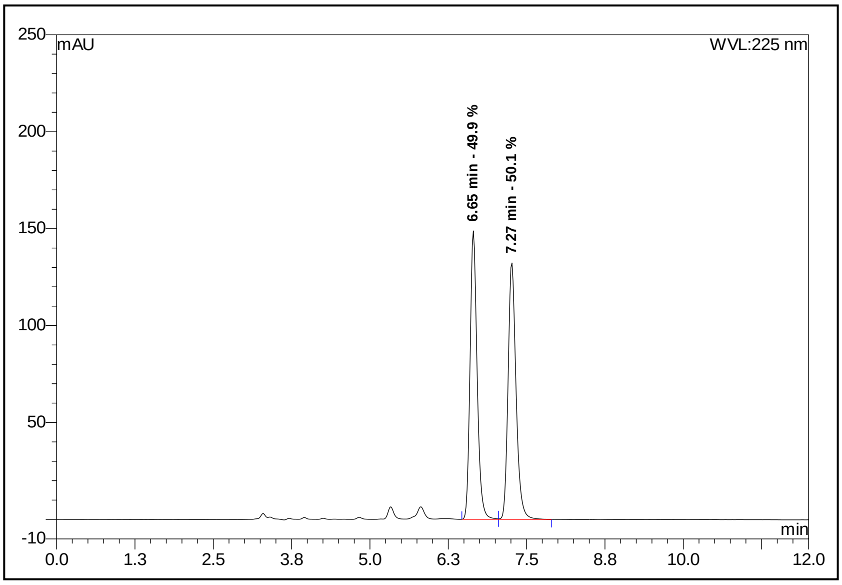

No.	Ret.Time min	Height mAU	Area mAU*min	Rel.Area %
1	6.65	148.800	19.449	49.89
2	7.27	132.282	19.535	50.11
**Total:**		281.081	38.983	100.00

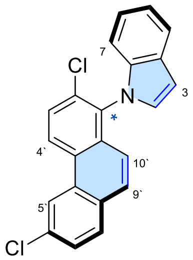

(S_a)-**20e**

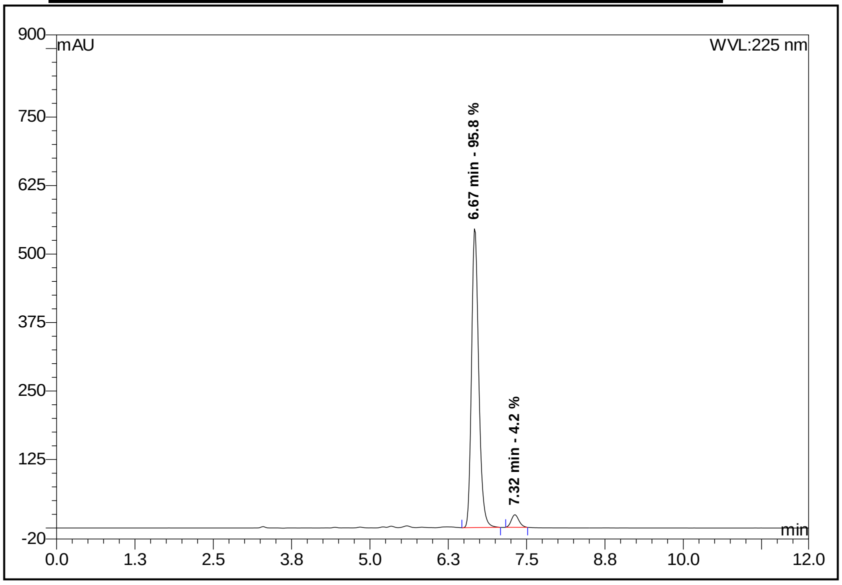

No.	Ret.Time min	Height mAU	Area mAU*min	Rel.Area %
1	6.67	545.279	72.910	95.76
2	7.32	22.928	3.228	4.24
**Total:**		568.207	76.138	100.00

X-Ray Data (Dr. Alessandro Prescimone)

(*S*$_a$)-6,6'-Difluoro-2,2'-dimethoxy-1,1'-biphenanthrene ((*S*$_a$)-18f):

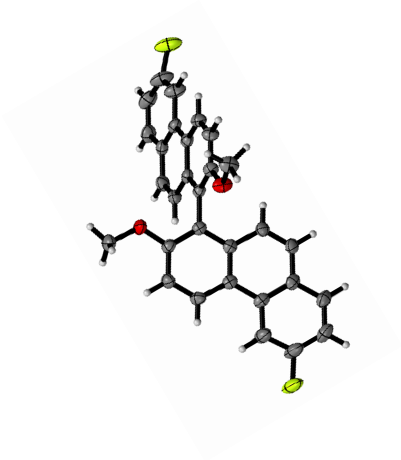

CCDC-2358712

Experimental: Single colourless block-shaped crystals of (*S*$_a$)-**18f** crystallised from a mixture of dichloromethane and methanol by solvent layering. A suitable crystal with dimensions 0.2 × 0.167 × 0.15 mm^3 was selected and mounted on a mylar loop in perfluoroether oil on a Basel-Cu-Stoe diffractometer. The crystal was kept at a steady T = 150 K during data collection. The structure was solved with the **ShelXT** (Sheldrick, 2015) solution program using dual methods and by using **Olex2** (Dolomanov et al., 2009) as the graphical interface. The model was refined with **ShelXL** (Sheldrick, 2015) using full matrix least squares minimisation on $\boldsymbol{F}^2$.

Crystal Data. $C_{30}H_{20}F_2O_2$ (M =450.46 g/mol): tetragonal, space group P4$_1$2$_1$2 (no. 92), a = 7.99310(10) Å, c = 34.2446(7) Å, V = 2187.88(7) Å^3, Z = 4, T = 150 K, μ(Cu Kα) = 0.789 mm^{-1}, *Dcalc* = 1.368 g/cm^3, 39624 reflections measured (10.332° ≤ 2Θ ≤ 139.652°), 2058 unique (R_{int} = 0.0461, R_{sigma} = 0.0188) which were used in all calculations. The final R_1 was 0.0349 (I > 2σ(I)) and wR_2 was 0.0856 (all data).

Empirical formula	$C_{30}H_{20}F_2O_2$
Formula weight	450.46
Temperature/K	150
Crystal system	tetragonal
Space group	P4$_1$2$_1$2
a/Å	7.99310(10)
b/Å	7.99310(10)
c/Å	34.2446(7)
α/°	90
β/°	90
γ/°	90
Volume/Å^3	2187.88(7)
Z	4
ρ_{calc}g/cm^3	1.368
μ/mm^{-1}	0.789
F(000)	936.0
Crystal size/mm^3	0.2 × 0.167 × 0.15
Radiation	Cu Kα (λ = 1.54186)
2Θ range for data collection/°	10.332 to 139.652
Index ranges	-9 ≤ h ≤ 9, -8 ≤ k ≤ 9, -40 ≤ l ≤ 27
Reflections collected	39624
Independent reflections	2058 [R_{int} = 0.0461, R_{sigma} = 0.0188]
Data/restraints/parameters	2058/19/165
Goodness-of-fit on F^2	1.046
Final R indexes [I>=2σ (I)]	R_1 = 0.0349, wR_2 = 0.0780
Final R indexes [all data]	R_1 = 0.0499, wR_2 = 0.0856
Largest diff. peak/hole / e Å^{-3}	0.13/-0.11
Flack parameter	0.04(7)

Microcrystal Electron Diffraction (Dr. Alessandro Prescimone, Eldico)

(*S*$_a$)-1-(2-Chlorophenanthren-1-yl)-1*H*-indole ((*S*$_a$)-20d):

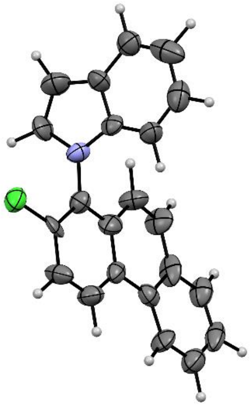

This structure collected with Electron Diffraction was obtained by kinematical refinement by the merging of 4 datasets collected on 4 different crystals. The absolute configuration has been successfully determined by the means of dynamical refinements[20] according to the following procedure: The structure of both enantiomers obtained by the kinetical refinement of the 4 separate datasets were used for the dynamical refinement keeping the structural parameters fixed, only parameters specific to dynamical refinement were refined. In all four cases the R factors for this enantiomer were consistently 3-4 percent better than those of the other enantiomer.

Experimental: (*S*$_a$)**-16d** was crystallised from a mixture of acteone and water by solvent layering. A suitable crystal was selected and **measured** on a **Eldico_ED** diffractometer. The crystal was kept at 293.15 K during data collection. Using Olex2 (Dolomanov et al., 2009), the structure was solved with the SHELXT (Sheldrick, 2015) structure solution program using Intrinsic Phasing and refined with the SHELXL (Sheldrick, 2015) refinement package using Least Squares minimisation.

Crystal Data for $C_{22}H_{14}ClN$ (*M* =327.79 g/mol): orthorhombic, space group $P2_12_12_1$ (no. 19), *a* = 5.97(16) Å, *b* = 22.5(6) Å, *c* = 24.2(7) Å, *V* = 3241(153) Å^3, *Z* = 8, *T* = 293.15 K, μ(electron) = 0.000 mm^{-1}, *Dcalc* = 1.344 g/cm^3, 42897 reflections measured (0.136° ≤ 2Θ ≤ 2.048°), 6616 unique (R_{int} = 0.2168, R_{sigma} = 0.2264) which were used in all calculations. The final R_1 was 0.1660 (I > 2σ(I)) and wR_2 was 0.3877 (all data).

Empirical formula	$C_{22}H_{14}ClN$
Formula weight	327.79
Temperature/K	293.15
Crystal system	orthorhombic
Space group	$P2_12_12_1$
a/Å	5.97(16)
b/Å	22.5(6)
c/Å	24.2(7)
α/°	90
β/°	90
γ/°	90
Volume/Å^3	3241(153)
Z	8
ρ_{calc}g/cm^3	1.344
μ/mm^{-1}	0.000
F(000)	557.0
Crystal size/mm^3	0.02 × 0.002 × 0.001
Radiation	electron (λ = 0.02851)
2Θ range for data collection/°	0.136 to 2.048
Index ranges	-7 ≤ h ≤ 7, -27 ≤ k ≤ 27, -30 ≤ l ≤ 29
Reflections collected	42897
Independent reflections	6616 [R_{int} = 0.2168, R_{sigma} = 0.2264]
Data/restraints/parameters	6616/0/434
Goodness-of-fit on F^2	1.044
Final R indexes [I>=2σ (I)]	R_1 = 0.1660, wR_2 = 0.3630
Final R indexes [all data]	R_1 = 0.1893, wR_2 = 0.3877
Largest diff. peak/hole / e Å^{-3}	0.23/-0.1

Supplementary References

1. Williams, D. B. & Lawton, M. Drying of organic solvents: quantitative evaluation of the efficiency of several desiccants. *J. Org. Chem.* **75**, 8351-8354 (2010).
2. Luo, H. X. *et al.* Indolo-quinoline boron difluoride dyes: synthesis and spectroscopic properties. *Org. Biomol. Chem.* **14**, 4185-4188 (2016).
3. Nishikawa, K. *et al.* Substituent effects of *cis*-cinnamic acid analogues as plant growh inhibitors. *Phytochemistry* **96**, 132-147 (2013).
4. Bałczewski, P. *et al.* Efficient Synthesis of Bis(dibromomethyl)arenes as Important Precursors of Synthetically Useful Dialdehydes. *Synthesis* **48**, 3509-3514 (2016).
5. Bonifacio, M. C., Robertson, C. R., Jung, J. Y. & T., K. B. Polycyclic Aromatic Hydrocarbons by Ring-Closing Metathesis. *J. Org. Chem.* **70**, 8522-8526 (2005).
6. De Lucca, G. V. *et al.* Small Molecule Reversible Inhibitors of Bruton's Tyrosine Kinase (BTK): Structure-Activity Relationships Leading to the Identification of 7-(2-Hydroxypropan-2-yl)-4-[2-methyl-3-(4-oxo-3,4-dihydroquinazolin-3-yl)phenyl]-9H-carbazole-1-carboxamide (BMS-935177). *J. Med. Chem.* **59**, 7915-7935 (2016).
7. Chen, P. C. *et al.* Neuroprotective and Antineuroinflammatory Effects of Hydroxyl-Functionalized Stilbenes and 2-Arylbenzo[b]furans. *J. Med. Chem.* **60**, 4062-4073 (2017).
8. Ravat, P. *et al.* Configurational Stability of [5]Helicenes. *Org. Lett.* **19**, 3707-3710 (2017).
9. Hosokawa, T. *et al.* Synthesis, Structures, and Properties of Hexapole Helicenes: Assembling Six [5]Helicene Substructures into Highly Twisted Aromatic Systems. *J. Am. Chem. Soc.* **139**, 18512-18521 (2017).
10. Si, T., Li, B., Xiong, W., Xu, B. & Tang, W. Efficient cross-coupling of aryl/alkenyl triflates with acyclic secondary alkylboronic acids. *Org. Biomol. Chem.* **15**, 9903-9909 (2017).
11. Hao, T. *et al.* One-Pot Three-Component Synthesis of Phenanthrenes via Palladium-Catalyzed Catellani and Retro-Diels-Alder Reactions. *J. Org. Chem.* **88**, 10426-10433 (2023).
12. Nathusius, M. *et al.* Chrysene-Based Blue Emitters. *Chem. Eur. J.* **26**, 15089-15093 (2020).
13. Legault, C. Y.M CTLview20. Université de Scherbrooke (2020). http://www.cylview.org.
14. Frisch, M. J., Trucks, G. W., Schlegel, H. B., Scuseria, G. E., Robb, M. A., Cheeseman, J. R., Scalmani, G., Barone, V., Mennucci, B., Petersson, G. A., Nakatsuji, H., Caricato, M., Li, X., Hratchian, H. P., Izmaylov, A. F., Bloino, J., Zheng, G., Sonnenberg, J. L., Hada, M., Ehara, M., Toyota, K., Fukuda, R., Hasegawa, J., Ishida, M., Nakajima, T., Honda, Y., Kitao, O., Nakai, H., Vreven, T., Montgomery, Jr., J. A., Peralta, J. E., Ogliaro, F., Bearpark, M., Heyd, J. J., Brothers, E., Kudin, K. N., Staroverov, V. N., Kobayashi, R., Normand, J., Raghavachari, K., Rendell, A., Burant, J. C., Iyengar, S. S., Tomasi, J., Cossi, M., Rega, N., Millam, J. M., Klene, M., Knox, J. E., Cross, J. B., Bakken, V., Adamo, C., Jaramillo, J., Gomperts, R., Stratmann, R. E., Yazyev, O., Austin, A. J., Cammi, R., Pomelli, C., Ochterski, J. W., Martin, R. L., Morokuma, K., Zakrzewski, V. G., Voth, G. A., Salvador, P., Dannenberg, J. J., Dapprich, S., Daniels, A. D., Farkas, Ö., Foresman, J. B., Ortiz, J. V., Cioslowski, J., Fox, D. J. Gaussian 09, Revision D.01, Gaussian, Inc., Wallingford CT (2009).
15. Koengeter, T. *et al.* Catalytic Cross-Metathesis Reactions That Afford *E*- and *Z*-Trisubstituted Alkenyl Bromides: Scope, Applications, and Mechanistic Insights. *J. Am. Chem. Soc.* **145**, 3774-3785 (2023).
16. Luchini, G., Alegre-Requena, J. V., Funes-Ardoiz, I., Paton, R. S. GoodVibes: Automated thermochemistry for heterogeneous computational chemistry data. *F1000 Research* 9, **219** (2020).
17. Lu, T. & Chen, F. Multiwfn: a multifunctional wavefunction analyzer. *J. Comput. Chem.* **33**, 580-592 (2012).
18. Contreras-Garcia, J. *et al.* NCIPLOT: a program for plotting non-covalent interaction regions. *J. Chem. Theory Comput.* **7**, 625-632 (2011).
19. William Humphrey, Andrew Dalke & Schulten, K. VMD: Visual molecular dynamics. *J. Mol. Graph.* **14**, 33-38 (1996).
20. Klar, P. B. *et al.* Accurate structure models and absolute configuration determination using dynamical effects in continuous-rotation 3D electron diffraction data. *Nat. Chem.* **15**, 848-855 (2023).
